# Birth of prominent scientists

**DOI:** 10.1371/journal.pone.0193374

**Published:** 2018-03-15

**Authors:** Leonardo Reyes Gonzalez, Claudia N. González Brambila, Francisco Veloso

**Affiliations:** 1 Medallia, San Mateo, California, United States of America; 2 Business School, Instituto Tecnologico Autonomo de Mexico, Tizapan San Angel, Mexico D.F., Mexico; 3 Imperial College Business School, South Kensington Campus, London, United Kingdom; Northwestern University, UNITED STATES

## Abstract

This paper analyzes the influence key scientists have in the development of a science and technology system. In particular, this work appraises the influence that star scientists have on the productivity and impact of young faculty, as well as on the likelihood that these young researchers become a leading personality in science. Our analysis confirms previous results that eminent scientist have a prime role in the development of a scientific system, especially within the context of an emerging economy like Mexico. In particular, in terms of productivity and visibility, this work shows that between 1984 and 2001 the elite group of physicists in Mexico (approximate 10% of all scientists working in physics and its related fields) published 42% of all publications, received 50% of all citations and bred 18% to 26% of new entrants. In addition our work shows that scientists that enter the system by the hand of a highly productive researcher increased their productivity on average by 28% and the ones that did it by the hand of a highly visible scientist received on average 141% more citations, vis-à-vis scholars that did not published their first manuscripts with an eminent scientist. Furthermore, scholars that enter the system by the hand of a highly productive researcher were on average 2.5 more likely to also become a star.

## 1. Introduction

Today’s emphasis on economic activity based on knowledge and innovation is leading industrialized as well as developing nations to place an important emphasis on policies to advance their science, technology and innovation (ST&I) systems and reap their benefits [1–4]. At the core of these efforts are policies to expand the scientific base and to generate, attract and retain highly talented scholars [5–6].

The most common approach is an effort to grow the size of their research system, aiming to build a critical mass of researchers across a variety of areas.

The underlying assumption is that key scientists can play a vital role in the development of a research system not only because they will make groundbreaking scientific discoveries, but also because they will create and develop internationally renowned research centers, improve universities’ capacity for generating and applying new knowledge, train the next generation of highly qualified personnel and also enable the establishment of successful high-technology startups. Consequently, there is growing interest within research administrators, policy makers and scholars on the role scientific stars have on the development of a ST&I system.

This has motivated an important stream of research focused on quantifying the impact leading researchers have on an established system, their peers and the institutions they work for. According to Zuker and Darby [7] 0.8% of the scientist in the GenBank in the 1990s were 22 times more productive than the average scientist, publishing 17.3% more papers. In addition, Azoulay, Graff Zivin and Wang [8] as well as Oettl [9] have shown the impact that “superstars” have on their peers by calculating the drop (8 to 18%) in productivity when a leading scientist dies; [10] documents a higher loss in productivity, (19 to 35%). In a related study, [11] shows that accomplished scholars appointed as presidents (vice chancellors) of a university have a positive impact on the research quality of their institutions. Furthermore, Zucker and Darby [12] find that stars themselves, rather than the disembodied knowledge associated to them, are crucial for the entry of a broad range of high-tech startups.

Another dimension that has been studied is research collaboration [13–14]. The results suggest that teamwork research model rather than individual-based approach is now the norm in most scientific endeavors [15–16]. The idea is that a collaborative approach in the production of knowledge enables scientists to access complementary expertise [13, 17–18], valuable equipment and resources [13 and 18], while exposing them to new ideas and encouraging cross-fertilization across fields [17–21]. This change also appears to have had a positive effect on publishing productivity [18, 22], quality [16 and 23–25], as well as visibility and prestige [13, 17, 19–21 and 26].

Other authors have also considered the extent to which ensembles of scientists provide a nurturing environment where researchers can flourish, in particular younger ones. For example, Bozeman and Corley [27] emphasize the importance of collaboration on the development of the scientific and technical human capital of researchers, especially when a senior scientist works with a junior one and the former acts as her mentor. Oettl [9] complements this perspective, noting that helpful eminent scientist have a higher impact (between 58% to 84% more) on the productivity of their co-authors than just highly productive researchers.

Research focused on the effects of sponsorships in academia has assessed the extent to which science follows Merton’s norms [28] by trying to disentangle the impact achievement and ascription (like race, gender, institutional affiliation or recognition of close collaborators) have on scientific performance, allocation of resources and academic career success. Crane [29] showed that the caliber of the institution has a positive impact on the productivity and prestige of its scientist’s, as well as the rate of the number of grants [30–32] suggest that changes in productivity can be ascribed to changes in departments and the prestige of these entities, while Reskin [33], as well as Long and McGinnis [34], establish that organizational context (like industry or academia) can influence a scientist’s level of performance. In addition, work focused on individuals has shown that the sponsor’s talent also matters. For example, Long’s [31] study of academic biochemists found that sponsors’ citations affected their students’ number of publications and citations, as well as their prestige; and Reskin [35] work on doctoral chemists showed that the advisor’s productivity influenced the pre-doctoral productivity of its advisees. Furthermore, advisees have a propensity to follow the steps of their advisors by replicating their success and skills. For example, Zuckerman [36] showed that Nobel Prize winners tend to positively influence the chances of their students and collaborators in also becoming Nobel Laureates; and Malmgren et al. [37] found that protégés that were trained by high fecundity mentors also score high on this indicator. Finally, there is a set of studies that have assessed the extent to which science is stratified by race and gender [38–40].

Despite the advances in our understanding of how the research context impacts the development of scientists, old and new, much remains to be explored. First, work focused on quantifying the influence of mentors and eminent scientists on others have not considered how they interact with other scholars in the context of research teams, and their impact in the evolution of the system, especially in terms of others at the beginning of their career. Second, previous research is typically composed of case studies that recount the mentoring experience, cross sectional studies or longitudinal analysis with usually short time frames as well as small and often random data sets [35, 41–45]. Third, with the exception of Malmgren et al. [37], research on mentorship has not looked at the extent to which protégés mimic their mentors’ steps, performance and reputation. Furthermore, because the Science and Technology (S&T) community has different characteristics around the world [46], a better understanding of the factors that condition research output, impact and success in science requires an analysis of a diverse set of countries. S&T systems have particular disparities between developing and developed nations. In developing nations, there are less resources and infrastructure dedicated to research and development (R&D). Moreover, government funds most R&D and, human as well as financial resources are centralized in a few institutions. Thus, studying emerging economies provides a better understanding of the factors that influence the performance, impact and overall contribution of scientists in this environment [46]. In addition, studying this type of countries is relevant because these nations are actively developing and implementing policies to improve their S&T systems. Therefore, a better understanding of the factors that foster success at individual and aggregated levels could help leap forward their system. This is particularly pertinent because, with a few exceptions [47–51], research in this area has mostly focused on the developed world.

This research tries to bridge existing gaps by combining the different research streams described above, with two complementary dimensions. One is to look at the role that scientific stars (i.e. the most accomplished and salient researchers) have in a science system. In particular we assess how relevant these eminent scientists are for the development of a system. This means understanding how much they contribute to the output and impact of the system, as well as how influential are them in breeding the next generation of successful scientists, i.e. how successful theirs protégés mimic their stellar performance. The other dimension is to assess how collaboration conditions the development of incoming scientists. In particular, we will look at the importance of the collaboration network of early co-authors for the productivity of new scientists and the likelihood that they also become leading scientists. Furthermore, this study uses a unique data that spans for almost two decades and is the one to look at the research system of a developing country, Mexico.

This work is divided in six sections. In the following section we lay out the purpose of this study and its research questions. In the third we explain the methods and the data, and define some key concepts. The fourth part shows the regression analysis. The fifth part provides the results. Finally, we provide conclusions and policy implications in the last section.

## 2. Research questions

Previous research on the impact that (lead) scientists and mentors/sponsors have had on other researchers has focused on quantifying the direct influence the former have on the productivity of their collaborators [8–9]. In other venue, other studies have assessed how these lead scientists contribute to the success of postdoctoral careers, including their first job and subsequent awards [35, 42, 44–45]. In this study we also consider the importance of accomplished scholars in a scientific system. Yet, instead of quantifying the impact these scientists have on ongoing relationships, we measure the influence they exert on young faculty when the first enter a particular field. In addition, we measure the influence different nurturing environments (or research collaborations) have on the incoming scientist. Furthermore, we look at the extent to which new scientist follow the steps of their mentors and also become a star.

### 2.1 Overall contribution of eminent scientists to a science system

The notion that a small percentage of researchers contribute to a disproportionally share of output in terms of papers [52–54] and citations [55–58] is well established in the literature. For example, de Solla Price [53] found that a minority of scientists in physics (around six percent) publishes 50 percent of all the publications, while Cole [59] and Reskin [33 and 60] have shown that this percentage of contributing scientist is fifteen percent in several other fields. Allison and Stewart [58] also found that the distribution of citations is more unequal than the one for articles and that for both measures the inequality increases with tenure. In addition, highly accomplished researchers also influence the realm of science by training, coaching and working with the next generation of eminent scientists. For instance, Zuckerman [36] found that 62% of the Noble laureates (in his sample) worked as young researchers under the supervision of previous prize winners; and these eminent scientists were more inclined to collaborate with other distinguished and highly productive researchers than their less renowned counterpart. Furthermore, this skewness in productivity and impact can be more pervasive in emerging economies were limited resources and heterogeneity within the system might favor a few scientists. By quantifying their total output and citations, as well as the number of subsequent stars that they breed, our first research question is: What is the direct impact that eminent scientists have had in the system? We also assess the indirect contribution of these researchers by looking at the performance of the scientists they breed.

### 2.2 Mentorship, research environments, apprenticeship and performance

As previously stated, mentors can play an important role in the development of their protégés, having a positive impact in their careers [35, 45 and 61–69]. Within academia, doctoral students are more than protégés. They are typically apprentices of their advisors and sometimes part of the research group of their mentors[35–44].

Students working under the supervision of an eminent scientist could have additional benefits by being exposed early on their careers to promising research ideas and being able to interact and collaborate with other renown researches, including Nobel Laureates [36]. Ham and Weinberg [70] analysis on Nobel laureate showed that being surrounded by other prize winners had a significant positive effect on starting its own work that would yield this type of recognition. With this in mind, we will study how much the productivity of a new researcher increases if he or she enters into the system by the hand of a star.

While the presence of stars is likely to be important, it is clearly not the only key aspect in the research environment of a nascent scientist. The organizational context and research/collaboration environment where scientists do their work are also likely to play an important role in fostering or hindering the productivity of budding researchers, in particular graduate students and postdoctoral fellows [71–73]. For example, Long and McGinnis [34] found that an appointment in non-intensive research organizations depresses publication output, whereas employment in research universities fosters publication. Work at the department level has also found that scientists publish more when they are surrounded by productive peers [74] and research-oriented coworkers [75].

Furthermore, leadership within research organizations of accomplished and experienced scientists is an additional factor that can affect productivity As Dill [76] noted, a team leader is there “to influence member’s knowledge and values, to facilitate contact and networks, to attract other competent researchers, to help colleagues who are blocked or stopped in their researcher efforts, and so on.” [75–76].

Thus, our second research question is: Which are the different research/collaborative environments that influence the performance of incoming researchers? To this purpose we consider four contexts in which a new researcher becomes active in a scientific system. First, we consider that a new researcher enters the system in the context of an established research group (RG). Second, we separate the top RG from the rest, recognizing that leading groups may have some different characteristics from an average research group. We then consider whether the early collaboration of the new researcher is with the leader of a RG vs. the mentoring of other members of the group. Finally, we consider early mentoring by the leader of top RG.

### 2.3 Following the steps of giants: Mimicking success

Previous studies on eminent scientist have stated the importance of sponsorship of leading researchers in their success. For example, Zuckerman [36] found that young scientist working with Nobel Laureates tend to replicate the success of their senior collaborators; and Crane [29] showed that the best students work under the supervision of top researchers at leading schools and become the next generation’s most productive scientists. In addition, the mentorship literature has noted that “the majority of participating mentors had been involved in a previous mentoring relationship as a protégé” [77], suggesting that some advisees follow the steps of their advisors. Yet, with the exception of Malmgren [37], little has been done to characterize who are the ones that follow the same career path and measure the likelihood of becoming a successful researcher. Thus, our third research question is: What is the likelihood that a new researcher becomes a star if she enters by the hand of one star? It is important to stress that we are assuming that a higher probability of success is correlated with entering to the system by the hand of a star scientists, and hence follow the same career path. This assumption must be taken cautiously, since additional evidence on career dynamics would be necessarily to reinforce the premise.

## 3. Method

### 3.1 Database

To answer to the previous questions we will use a database from Thomson Scientific [78] containing all papers published between 1980 and 2003 with at least one address in Mexico. This database contains the following information: article name, author(s), author(s), address(es), year of publication, journal, volume, pages, backward citations (i.e. references) and total number of citations received.

From this database we selected all papers published in Mexico in the areas of *Physics* and *Applied Physics/Condensed Matter/Materials Science* in the period of 1981–2003, this means that letters, proceedings, notes and reviews were excluded. The classification of the selected areas of knowledge is done by Thompson Reuters. All journals that are indexed in the Science and Social Citation Index are classified in one or more categories depending on the area of knowledge. We chose these areas because in the past the field of Physics and its related areas have been widely studied around the world [79–80] and Mexico has a long tradition of publishing in international peer reviewed journals, indexed by ISI in these areas [78 and 81].

Once all the papers were identified, we created a dataset containing the name of the article, its author or authors, the institutional affiliation of these scientists and the number of citations these articles received within a three-year window (e.g. for the papers published in 1990 we restricted the citation count to the period 1990–1992). The three-year citation window was chosen, in an effort to balance the loss of observation years in our panel due to the citation window against the desire to include to the extent possible this measure of quality and impact. Besides, on average, publications in our sample receive 60% of cites in this window, with the remaining 40% received in the following 10 years. Moreover, there is evidence [82] that shows that the 3-year and 4-year windows were quite consistent with the cited peak age of documents, at least in the ophthalmologic journals. In addition we divided this set into three periods. Period one (which includes all the papers published between 1981 and 1983) is used to identify the scientists that were in the system before the focal period used in the study. Period two (from 1984 to 2001) is the *sample period* and includes only scientists that entered the system, i.e. published an article for the first time, after 1984 up to 2000. Period three (2002–2003) was used to identify the scientists that exited the system before 2001 and entered after 1983. We say a researcher *entered the system* (within the focus period) if her name was not present in the first period but appeared (or published an article) after 1983; in the same respect, a researcher *exited the system* if she was present in period two but not in the third one. In order to avoid sporadic authors, we excluded from this dataset all the researchers that published only one paper and were present only one year within the sample period 1984–1999.

### 3.2 Definitions

For this analysis, we classify all the researchers in our sample along several dimensions.

#### Star and non-star scientists

First, we characterize the researchers on our sample as *star* (or eminent) and *non-star* scientists based on their performance for a certain period of time along two dimensions: *productivity* (measured in terms of the number of publications per year) and *impact* (citations per year). Previous studies have used a 5% cutoff point in output or impact to define an elite group of scientists [8–9]. Although this characterization seems easy enough, it is difficult to decide whether to draw a precise cutoff point at a certain number like 1%, 5% or 10% because of the skewness of these variables. With this in mind, we follow a different approach and define this select group of researchers using the sample’s performance distribution. In this study a *star scientist* is a researcher that is above the average productivity plus one standard deviation (STDEV) of all scientists in the sample and a *non-star scientist* is the one that is below this threshold. We use one STDEV as minimum performance level however more stringent levels (two or three STDEVs) can be used to identify star scientists. Later in the paper, we show the number of stars by different STDEV levels and their average contribution and performance. In addition, we show a complete regression analysis of the one STDEV definition and the most important results for the two STDEV.

#### Research environments

Second, using a novel method for the characterization of research groups [83], we identify all the groups that are present in the system, as well as leaders of these communities; and based on this, single out different research environments to which researchers might be exposed to. The main idea behind this new method is that it defines all the research groups based on self-organizing characteristics of the research endeavor [84]. The proposed method uses the notion that modern science is conducted primarily through a network of collaborators (or groups) who organize themselves around key researchers, often known as the principal investigators (PIs). Specifically, this method uses the patterns of collaboration and the strength of ties in a coauthorship network to, first, identify the PIs, or leaders, of these ensembles and, then, to characterize the boundaries of different research groups (RGs) centered at these PIs. This method defines a *Principal Investigator* (PI) as an author with a high number of repeated connections, i.e. a researcher that has written several papers with a high number of coauthors. The method allows identifying disjoint groups within certain period of time, from two years to the entire period of study, by including all the papers that were published within those years. For this research we used a three-year rolling window. This meant that, first we characterized all the groups between *t*_*0*_ and *t*_*2*_, then we did it for *t*_*1*_*-t*_*3*_, up to *t*_*n-2*_*- t*_*n*_ where *n* is the maximum number of years of our sample. This method provides a list of all the PIs in the system and the composition of these communities.

Based on the output of this method (i.e. the PIs and composition of RGs) and the performance of these groups, we distinguish four types of research environments to which scientists might be exposed (at the beginning of their careers). Following a similar procedure to define star and non-star scientists, we classify these communities into top and non-top research groups, a *top research group* is a group that is above the average productivity plus one standard deviation (STDEV) of all the groups in the sample, and a *non-top research group* is below this threshold. Table 1 defines these environments.

**Table 1:** Definition of research environments.

Research Environment	Definition
Exposure to a research group	A scientist is said to be exposed to a RG if he or she collaborates with a researcher that belongs to a RG
Exposure to a top research group	A scientist is said to be exposed to a top-RG if he or she collaborates with a researcher that belongs to a top-RG
Exposure to the leader of research group	A scientist is said to be exposed to the leader of a RG if he or she collaborates with the PI of a RG
Exposure to the leader of a top research group	A scientist is said to be exposed to the leader of a top-RG if he or she collaborates with the PI of a top-RG

Tables 2 to 4 provide the summary statistics for the total number of papers, citations, authors and different type of stars for the 1981–2001 period. From Table 2 it can be seen that 4,180 unique authors were identified and 2,018 (or 48% of the total) were defined as suitable ones; in order to avoid sporadic authors we restricted our analysis to scientist that published two or more papers between 1984 and 2001 in at least two different years and entered the system before 1999. In addition, we can observe that the latter published 6,550 articles in the sample period. Table 3 shows how the 2,018 authors break into two groups: the ones that entered the system before 1984 (8%) and the ones that did it after 1983 (92%). In addition, this table shows the number of eminent scientist based on articles and citations for these periods of time for the one STDev definition (11% and 9% respectively) and two STDev definition (~4% for both categories).

**Table 2:** Summary statistics, absolute numbers of papers and authors.

	Authors		Papers	
Total number, 1981–2001	4180		7223	
Excluded from the analysis	2162*		673**	
Remaining number 1984–2001	2018	48%***	6550	91%***

*Authors were excluded because they only published one paper, were only present one year, exited the system before 1984 or entered in the system after 1998.

**Papers were not included because they were published before 1984 or by one of the 2,162 excluded authors.

***Percentage from the total number.

**Table 3:** Summary statistics, absolute numbers of stars.

		Stars Based on							
		Articles per year (by star-articles)				Citations per year (by star-citations)			
		One STDev*		Two STDeV**		One STDev*		Two STDev**	
Authors that entered before the studied period (1981–1983)	169	16	9%	9	5%	17	10%	7	4%
Authors that entered in the studied period (1984–1998)	1857	201	11%	81	4%	167	9%	71	4%
Remaining number, 1984–2001	2018	217	11%	90	4%	184	9%	78	4%

*One STDev = Stars are above the average plus one Standard Deviation.

**Two STDev = Stars are above the average plus two Standard Deviation.

**Table 4:** Direct Contribution ^1^ by different type of star (one STDev)^2^.

Papers			Citations		
by star- articles^3^	2740	42%^6^	by star-articles^3^	6368	50%^6^
by non-star-articles^5^	5331		by non-star-articles^5^	9866	
by star-citations^4^	1822	28%^6^	by star-citations^4^	6263	49%^6^
by non-star-citations^5^	5766		by non-star-citations^5^	9435	
Total number of papers 1984–2001	6549		Total number of citations, 1984–2003	12770	

^1^Direct Contribution is the total number of published papers by a star; as well as the received citations of those papers.

^2^Stars are above the average plus one Standard Deviation.

^3^star-articles, stars are defined based on articles per year.

^4^star-citations, stars are defined based on citations per year.

^5^non-star papers or citations could be double counted with star ones since in a paper are published by stars and non-stars.

^6^Percentage from the total number.

Furthermore, Table 4 provides how much stars scientists (directly) contribute to the system. Depending on how you define an eminent scientist (articles or citations) they publish 28% to 42% of all articles and receive roughly 50% of all citations for the one STDev definition.

Table 5 gives a general overview of the evolution of the system in terms of number of researchers, stars and research groups; total and average productivity (articles and citations), and average performance by type of stars and research groups for the three-year periods of 1984–1996 to 1999–2001 (the studied period) and 1981–1984 (as reference). From this table it can be seen that between 1984–1986 and 1999–2001 the system expanded six-times in terms of number of researchers and publications, as well as 4.6 times in terms of citations; growing 13.8%, 14.1% and 12.2% each year, respectively. In addition the number of research groups grew 5.3 times and the average size of increased by 50%. Furthermore, the number of stars grew at faster rates. During this period of time, individual performance steadily increased by any measure, but group efficiency declined on all variables.

**Table 5:** System evolution, 1981–2001.

								Growth^2^		CAGR^3^	
	81–83^1^	84–86	87–89	90–92	93–95	96–98	99–01	81-83/	84-86/	81-83/	84-86/
								99–01	99–01	99–01	99–01
**Number of researchers**											
**Total number**	169	216	334	531	930	1,420	1,500	7.9x	6x	12.9%	13.8%
**Stars, articles**^4^	16	19	36	55	110	178	202	11.6x	9.6x	15.1%	17.1%
**Stars, citations**^5^	17	18	32	43	91	129	142	7.4x	6.9x	12.5%	14.8%
**Number of articles**											
**Total number**	245	303	408	658	1,16	1,820	2,200	8x	6.3x	12.2%	14.1%
**by star, articles**^4^	71	83	129	168	408	825	1,130	14.9x	12.6x	15.7%	19.0%
**by star, citations**^5^	67	76	116	128	296	521	685	9.2x	8x	13.0%	15.8%
**Average number of articles per author**											
**Average total**	1.9	2.0	1.8	2.0	2.1	2.5	3.2	0.7x	0.6x	2.7%	3.3%
**by star, articles**^4^	4.6	4.6	3.9	3.6	4.5	6.2	7.7	0.7x	0.7x	2.8%	3.5%
**by star, citations**^5^	4.2	4.9	3.9	3.5	4.1	5.5	6.7	0.6x	0.4x	2.5%	2.1%
**Number of citations**											
**Total number**	633	736	867	1,260	2,240	3,540	4,140	5.5x	4.6x	10.4%	12.2%
**by star, articles**^4^	212	246	308	424	1,050	1,990	2,360	10.1x	8.6x	13.5%	16.3%
**by star, citations**^5^	209	292	380	573	1,070	1,770	2,180	9.4x	6.5x	13.1%	14.3%
**Average number of citations per author**											
**Average total**	6.3	6.9	5.3	5.8	6.4	7.0	8.2	0.3x	0.2x	1.4%	1.1%
**by star, articles**^4^	16.1	15.3	10.0	11.2	14.4	17.2	19.3	0.2x	0.3x	1.0%	1.5%
**by star, citations**^5^	16.7	22.5	13.3	18.2	18.0	23.6	27.8	0.7x	0.2x	2.7%	1.4%
**Research Groups (RGs)**											
**Total number of RG**	32	36	52	101	173	271	226	6.1x	5.3x	11.5%	13.0%
**Top RGs, articles**^4^	8	5	7	14	24	44	34	3.3x	5.8x	8.4%	13.6%
**Top RGs, citations**^5^	3	7	5	12	18	37	28	8.3x	3x	13.2%	9.7%
**Researchers per researcher group**											
**Average number**	4.1	3.9	4.4	4.2	4.8	5.5	6.2	0.5x	0.6x	2.3%	3.1%
**Average number of articles per researcher per group**											
**Total average**	9.1	10.1	9.5	7.4	6.5	4.5	5.0	-0.5x	-0.5x	-3.3%	-4.6%
**by top RGs, articles**^4^	17.1	20.6	16.2	17.6	14.2	12.5	12.9	-0.2x	-0.4x	-1.6%	-3.1%
**by top RGs, citations**^5^	18.0	20.5	15.0	16.5	13.9	10.1	11.4	-0.4x	-0.4x	-2.5%	-3.8%
**Average number of citations per researcher per group**											
**Total average**	19.1	21.4	18.0	15.0	11.7	7.9	8.7	-0.5x	-0.6x	-4.3%	-5.8%
**by top RGs, articles**^4^	39.9	43.0	29.9	39.2	26.0	22.7	28.1	-0.3x	-0.3x	-1.9%	-2.8%
**by top RGs, citations**^5^	44.7	50.8	36.2	45.8	35.4	32.3	35.4	-0.2x	-0.3x	-1.3%	-2.4%

^1^ Period 1.

^2^ “x” denotes times of expansion or contraction.

^3^ CAGR: Compound Annual Growth Rate.

^4^ Based on articles, one STDev defintion.

^5^ Based on citations, one STDev defintion.

#### Entry into the system

In addition to establishing if a researcher is a star (within a certain period) and the different type of environments this person has been exposed to during the early part of her academic career, we also identify (1) when (*year of entry*), (2) where (institution of entry) and (3) how (*type of entry*) this scientist entered the system.

For each researcher, we first define her *year of entry* as the year (for this analysis we use calendar years to define the year of entry of a scientist) when she published her first paper(s). In addition, we use the address that appears on her first publication(s) to identify her *institution of entry*. Finally, we define the *type of entry* by looking at the research environment(s) a scientist was exposed to within her year of entry.

We say that a researcher *enters with a star* if she publishes a paper with a star scientist in her first year. We use first year, and not first paper, because the time frame of our data is restricted to years; this means that within a particular year we cannot know which paper was published first.

**Fig 1:**
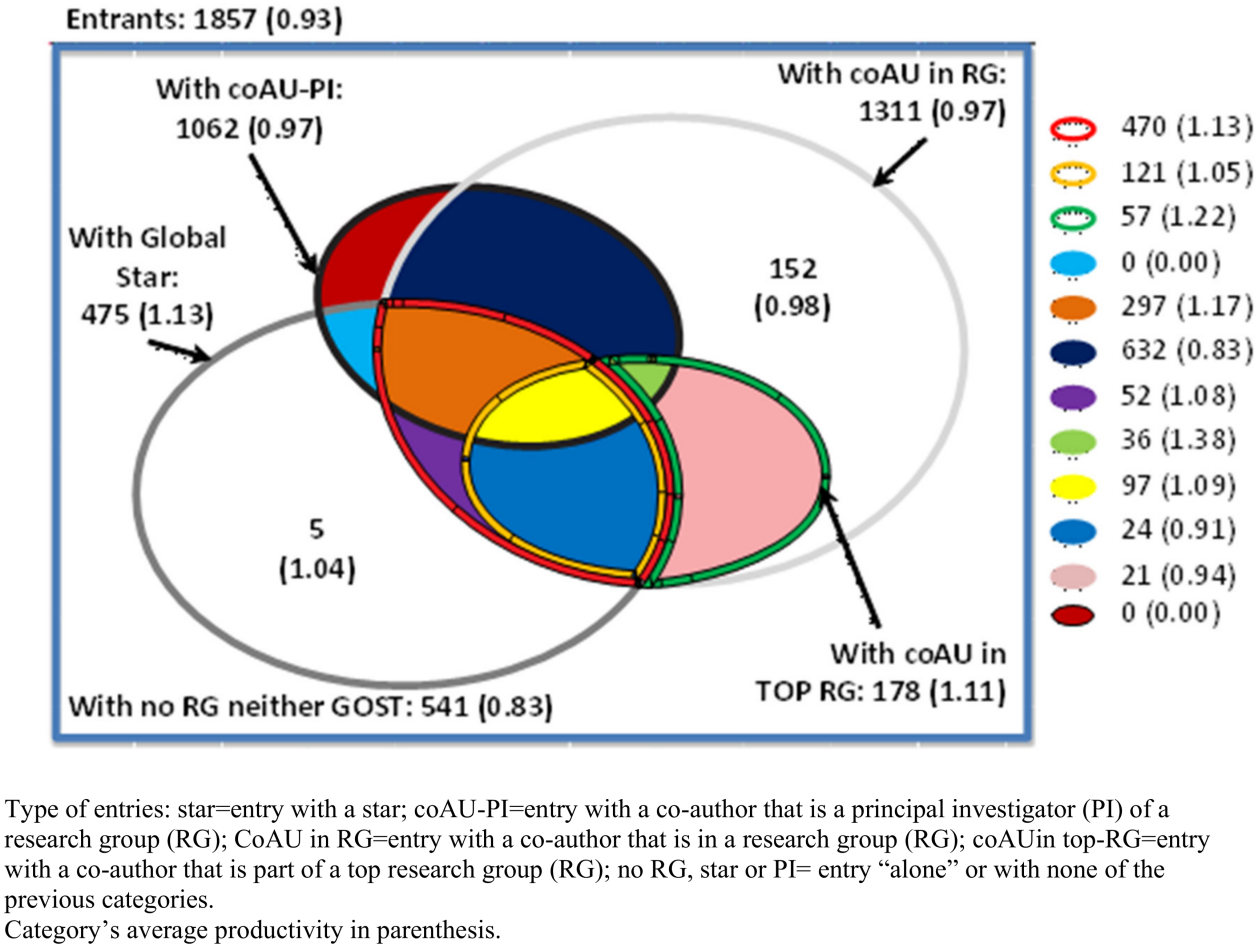
Entry space based on articles. This figure depicts how the entry space is broken down by type of entry for all the scientists that entered the system between 1984 and 1999. In addition this figure shows in parenthesis the average productivity (number of articles per year per researcher) by each type of entry.

**Fig 2:**
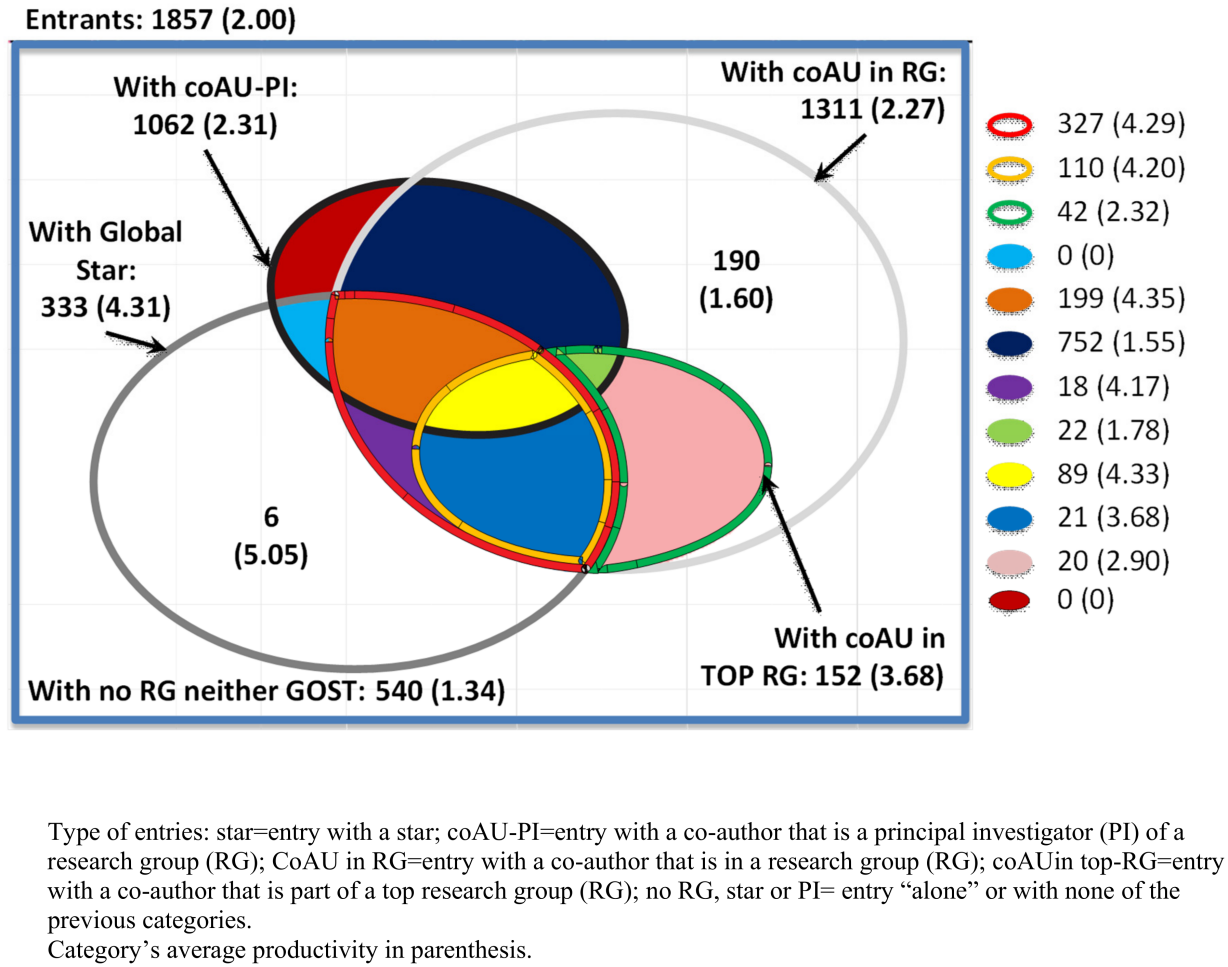
Entry space based on citations. This figure depicts how the entry space is broken down by type of entry for all the scientists that entered the system between 1984 and 1999. In addition this figure shows in parenthesis the average impact (number of citations per year per researcher) by each type of entry.

In addition, we say a researcher enters within a particular research environment if she is exposed to one on her first year of entry; this means that a scientists *enters with* a (1) *RG*, (2) *top-RG*, (3) *the leader of a RG* or (4) *the leader of a top-RG* if she is exposed to one of these environments on her year of entry. Figs 1 and 2 depict how the entry space is broken down by type of entry for all the scientists that entered the system between 1984 and 1999. In addition these figures show in parenthesis the productivity by each type of entry. From these figures it can be seen that 71% of the sample can be characterized by one (or more) of our entry definitions. In addition, these figures show that star scientists (with high citations rate and productivity) collaborate directly with 18% to 26% of new entrants.

Finally, Table 6 quantifies the indirect and total contributions each type of star makes to the system. *Indirect contribution* is defined as the total number of published papers by an author that entered with a star, excluding all the ones that were published together; as well as the received citations of those papers. *Direct Contribution* is the total number of published papers by a star; as well as the received citations of those papers. Total Contribution is the sum of both quantities.

**Table 6:** Indirect and total contribution to the system by type of star (one STDev)_1_.

Paper			Citations		
Indirect Contribution			Indirect Contribution_2_		
by author that enters w/star-articles_4_	1414	22%_6_	by author that enters w/star-articles_4_	3129	25%_6_
by author that enters w/star-citations_5_	1077	16%_6_	by author that enters w/star-citations_5_	2189	17%_6_
Total Contribution_3_			Total Contribution_3_		
by star-articles_3_	4154	64%_6_	by star-articles_3_	9497	75%_6_
by star- citations_4_	2899	44%_6_	by star- citations_4_	8452	66%_6_
Total number of papers, 1984–2001	6549		Total number of papers, 1984–2003	12770	

^1^ Stars are above the average plus one Standard Deviation.

^2^ *Indirect Contribution* is the total number of published papers by an author that entered with a star, excluding all the ones that were published together; as well as the received citations of those papers.

^3^ *Total Contribution* is de sum of direct (see Table 3) and indirect contribution.

^4^ Star-articles, stars are defined based on articles per year.

^5^ Star-citations, stars are defined based on citations per year.

^6^ Percentage from the total number.

From this table it can be seen that authors that entered by the hand of highly productive scientists contribute 22% of all articles and a quarter of all citations, whereas researchers that entered by the hand of one that has high impact supply 16% of all articles and 17% of all citations. Overall, eminent scientists contribute (directly and indirectly) to two thirds of the system.

## 4. Regression analysis

To quantify the effect different types of entry (i.e. collaboration upon entry with a star scientists or a researcher that is part of a RG or top-RG, or with a PI in a RG or top- RG) have on the productivity of the incoming scientists we used an ordinary least square (OLS) regression model. We used an OLS (and not a negative binomial) model because our dependent variables (publications per year and citations per year) are continuous (see below for the description of these variables). In addition, we employed a logistic regression model to measure the extent to which these new researchers mimic the steps of their mentors and the degree to which different research environments are conducive to becoming a leading scientist.

A key characteristic of our analysis is the use of year and institution fixed effects on our regression models [85–87]. The model controls for unobserved heterogeneity between year of entry and institution of entry. These controls are important because different institutions will be associated with important heterogeneity in scientific performance and the ability of incoming researchers can vary over time. For example, it is likely that the Physics Department of CINVESTAV, a very well-known department attracts better people for its ranks, when compared to a smaller regional university. If this were the case, results on the comparison of productivities for scientists across institutions could be entirely driven by unobserved differences between the institutions, rather than the differences between entry with star and non-star scientists. This could generate misleading results. The model assumed to be:

$$Y_{ijt}=\beta_{0}+\sum \beta_{k}x_{kijt}+\varepsilon_{ijt}$$ (1)

Where

$$\varepsilon_{ijt}=\emptyset_{ij}+\upsilon_{it}+\omega_{ijt}$$ (2)

In this equation regression coefficients are denoted as *β*, *k* indexes the measured independent variables (*x*_s_), *i* indexes individuals, *t* indexes time, *j* indexes institutions, and *ε* = error terms; ∅ = cross-sectional (institutional) component of error; *υ* = time-wise component of error; *ω* = purely random error component; and *β*_0_ = intercept. *Y*, the dependent variable, is explained in the next section; as well as the different independent ones.

### 4.1 Dependent variables

In order to quantify the increase (or decrease) on productivity and impact that different types of collaborations at the time of entry have on the incoming researchers, two continuous variables were used on the left hand side of the OLS model. The sum of all papers published by a scientist during her tenure (the *tenure* of a research is defined as the total number of years this person was present in the system, from the year she enters the system up to when she leaves it or the year 2001, even if she didn’t publish any paper in any given year) divided by the number of years in *the researcher’s tenure* was used as a measure for productivity, whereas the sum of all citations received for each paper in a three-year window divided by the number of years in the scientist’s tenure was used to indicate the level of impact a researcher has had. For the logistic model a dummy variable was used on the left hand side of Eq 1, taking the value of “one” if the new scientist became a star within the studied period and “zero” otherwise. This indicates if this researcher followed the steps of their mentors.

For this work we used the one STDev definition to identify the star scientists and top-RG in our sample. This means that this output is a lower bound for the impact early collaboration with elite scientists and groups have on incoming researchers.

### 4.2 Independent variables

Five different dummy variables were used (individually and combined) on the right hand side of Eq 1 to assess the impact different types of entry have on the performance of new researchers and the likelihood of becoming a star scientist, Table 7 shows these variables.

**Table 7:** Independent variables.

Variables	Description
Star (articles or citations)	1 = entry with a star,
	0 = otherwise
coAU in RG (articles or citations)	1 = entry with a coauthor that belongs to a RG;
	0 = otherwise
coAU in top RG (articles or citations)	1 = entry with a coauthor that belongs to a top RG;
	0 = otherwise
coAU-PI in RG (articles or citations)	1 = entry with a coauthor that that is a PI in a RG;
	0 = otherwise
coAU-PI in top RG (articles or citations)	1 = entry with a coauthor that that is a PI in a top RG;
	0 = otherwise

## 5. Results of regression models

In this section we present the results of the regression models discussed previously. We divide the analysis in two parts. Part one shows the estimates for the increase in productivity and impact for different type of entries, while part two shows the extent to which incoming researchers follow the same path of eminent scientists or if other type of entry is associated with their success, e.g. collaboration with a PI or a coauthor that belongs to a highly productive group. The regressions were ran first with single variables (e.g. entry by the hand of a star scientist) to assess the individual impact these variables had on the different dependent variables and then with two or more variables (e.g. early collaboration with a star scientist and a co-author that belongs to a RG) to measure the combined effect of these variables on the left hand side of these equations.

### 5.1 Type of entry and productivity impact

In this part we present the results of the OLS regression for papers and citations per year while controlling for different type of entry (Tables 8 and 9). These models suggest that highly productive scientist have a positive influence in the output of their protégés, boosting their productivity by 27% on average (Table 8 models AII-01 to AII-04). In addition, the regression models show that entry with a co-author that belongs to a top RG increases the productivity of the new researcher by an average 18% (Table 8 models AII- 01 and AII-02). Furthermore, entry with the leader of a top RG raises the output of the incoming collaborators by 38%, or 40% more than entering with a star (Table 8 models AII-03 and AII-04).

**Table 8:** Productivity increase by type of entry, articles per year, 1984–2001.

Type of Entry (Std. Err.) [Total Effect*]	AI-01	AI-02	AI-03	AI-04	AI-05	AII-01	AII-02	AII-03	AII-04
Star**, articles	0.296^c^					0.259^c^	0.252^c^	0.244^c^	0.245^c^
	(0.034)					(0.036)	(0.037)	(0.035)	(0.036)
	[32%]					[28%]	[27%]	[26%]	[26%]
coAU in RG, Articles		0.136^c^					0.028		
		(0.035)					(0.037)		
		[15%]					[NA]		
coAU in top RG**, articles			0.290^c^			0.168^c^	0.161^c^		
			(0.050)			(0.053)	(0.054)		
			[31%]			[18%]	[17%]		
coAU-PI of RG, Articles				0.098^c^					-0.005
				(0.031)					(0.032)
				[11%]					[NA]
coAU-PI of top RG**, articles					0.476^c^			0.348^c^	0.350^c^
					(0.064)			(0.066)	(0.068)
					[51%]			[37%]	[38%]

* Total Effect = coefficient divided by average

** Star and top RG defined based on the average plus one standard deviation

^a^ 10% confidence level,

^b^ 5% confidence level,

^c^ 1% confidence level

NA coefficient is not significant

**Table 9:** Productivity increase by type of entry, citation per year, 1984–2001.

Type of Entry (Std.Err.) [Total Effect*]	CI-01	CI-02	CI-03	CI-04	CI-05	CII-01	CII-02	CII-03	CII-04
Star**,citations	2.811^c^					2.842^c^	2.790^c^	2.866^c^	2.861^c^
	(0.183)					(0.199)	(0.201)	(0.195)	(0.198)
	[140%]					[142%]	[139%]	[143%]	[143%]
coAU in RG, citations		0.814^c^					0.296^a^		
		(0.176)					(0.173)		
		[41%]					[15%]		
coAU in top RG**, citations			1.454^c^			-0.113	-0.172		
			(0.273)			(0.283)	(0.285)		
			[73%]			[NA]	[NA]		
coAU-PI of RG, citations				0.536^c^					0.023
				(0.152)					(0.151)
				[27%]					[NA]
coAU-PI of top RG**, citations					1.515^c^			-0.302	-0.309
					(0.357)			(0.363)	(0.366)
					[76%]			[NA]	[NA]

* Total Effect = coefficient divided by average

** Star and top RG defined based on the average plus one standard deviation

^a^ 10% confidence level,

^b^ 5% confidence level,

^c^ 1% confidence level

NA coefficient is not significant

Then we look at the influence of eminent scientists on impact, or number of citations per year, of new researchers. Models CII-01 to CII-04 in Table 9 show that stars are associated with an increase in the amount of citations received by their advisees by an average of 142%, while other forms of entry have a negligible effect on their citation rate.

We also analyze the number of citations divided by the number of publications as a measure of impact. The results are shown in Table 10. As can be seen the results are similar in sign and significance than those of Table 9, but the total effect is smaller. The results suggest that entry with a star increases the number of citations per publication by around 20%.

**Table 10:** Productivity increase by type of entry, citations per publications (CA), 1984–2001.

Type of Entry (Std.Err.) [Total Effect*]	CA-01	CA-02	CA-03	CA-04	CA-05	CAI-01	CAI-02	CAI-03	CAI-04
Star**,citations	0.432^c^					0.413^c^	0.314^c^	0.425^c^	0.339^c^
	(0.104)					(0.109)	(0.113)	(0.108)	(0.110)
	[21%]					[20%]	[11%]	[20%]	[16%]
coAU in RG, citations		0.497^c^					0.401^c^		
		(0.106)					(0.113)		
		[24%]					[19%]		
coAU in top RG**, citations			0.281^a^			0.086	-0.013		
			(0.152)			(0.160)	(0.162)		
			[14%]			[NA]	[NA]		
coAU-PI of RG, citations				0.413^c^					0.342
				(0.109)					(0.097)
				[20%]					[NA]
coAU-PI of top RG**, citations					0.269			0.044	-0.099
					(0.194)			(0.202)	(0.206)
					[NA]			[NA]	[NA]

* Total Effect = coefficient divided by average

** Star and top RG defined based on the average plus one standard deviation

^a^ 10% confidence level,

^b^ 5% confidence level,

^c^ 1% confidence level

NA coefficient is not significant

In addition to independently assessing the effect that highly productive and visible environments have on the productivity and citations of incoming scientist, we combine both types of environments (publication output and received citations) on a single regression model and measure which milieu has the highest influence on the publication output and received citations of new researchers. Table 11 show that starts with a high citation rate have on average 1.4 more impact on the productivity of new scientists than highly productive ones. In addition, early collaboration with members of a highly productive group (especially the leader of the group) also enhances the productivity of the incoming researcher. In contrast early collaboration with researchers that belong to a group with high citations rates does not have an impact on the productivity of new scientists. Furthermore, Table 11 show that a highly visible star, based on citations, is the only variable that has a significant and positive effect of the level of citations of new researchers.

**Table 11:** Productivity increase by type of entry, articles per year, 1984–2001.

Type of Entry (Std.Err.) [Total Effect*]	Articles per year					Citations per year				
	AIII-01	AIII-02	AIII-03	AIII-04	AIII-05	CIII-01	CIII-02	CIII-03	CIII-04	CIII-05
Star**,articles	0.180^c^		0.153^c^		0.141^c^	-0.040		-0.061		-0.087
	(0.040)		(0.041)		(0.041)	(0.195)		(0.201)		(0.199)
	[19%]		[16%]		[15%]	[NA]		[NA]		[NA]
Star**,citations	0.239^c^		0.243^c^		0.229^c^	2.835^c^		2.871^c^		2.900^c^
	(0.045)		(0.047)		(0.047)	(0.218)		(0.230)		(0.227)
	[26%]		[26%]		[25%]	[141%]		[143%]		[145%]
coAU in top RG**,articles		0.260^c^	0.182^c^				0.376	0.173		
		(0.063)	(0.063)				(0.311)	(0.306)		
		[28%]	[20%]				[NA]	[NA]		
coAU in top RG**, citations		0.055	-0.091				1.212^c^	-0.215		
		(0.068)	(0.070)				(0.338)	(0.340)		
		[NA]	[NA]				[61%]	[NA]		
coAU-PI in top RG**, articles				0.460^c^	0.364^c^				.842^b^	0.485
				(0.078)	(0.078)				(0.388)	(0.380)
				[50%]	[39%]				[42%]	[NA]
coAU-PI in top RG**, citations				0.032	-0.112				.990^b^	-0.589
				(0.086)	(0.088)				(0.431)	(0.428)
				[NA]	[NA]				[49%]	[NA]

* Total Effect = coefficient divided by average

** Star and top RG defined based on the average plus one standard deviation

^a^ 10% confidence level,

^b^ 5% confidence level,

^c^ 1% confidence level

NA coefficient is not significant

### 5.2 Type of entry and likelihood of also becoming an eminent scientist

In this section we measure the extent to which incoming scientists follow the steps of their mentors. According to the output of the logistics model a scientist that enters the system by the hand of a highly productive researcher is on average 2.5 more likely of mimicking the success of their mentor and also be regarded as highly productive one (Table 12 models AV-01 to AV-04). In addition, early collaboration with a researcher that belongs to a highly productive group has almost the same effect (Table 12 models AV-03 and AV-04). These results suggest that a nurturing environment is as important as entry with a highly productive scientist for success, at least in terms of output.

**Table 12:** Likelihood of becoming a star by type of entry, citations per year, 1984–2001.

Type of Entry (Std. Err.)	AIV-01	AIV-02	AIV-03	AIV-04	AIV-05	AV-01	AV-02	AV-03	AV-04
Star*, articles	2.779^c^					2.317^c^	2.384^c^	2.499^c^	2.591^c^
	(0.387)					(0.339)	(0.368)	(0.312)	(0.334)
coAU in RG, articles		1.491^b^					0.893		
		(0.249)					(0.165)		
coAU in top RG*, articles			3.232^c^			2.205^c^	2.257^c^		
			(0.610)			(0.444)	(0.463)		
coAU-PI of RG, articles				1.519^c^					0.854
				(0.203)					(0.112)
coAU-PI of top RG,* articles					4.849^c^			2.150^c^	2.264^c^
					(1.139)			(0.471)	(0.507)

* Star and top RG defined based on the average plus one standard deviation

^a^ 10% confidence level,

^b^ 5% confidence level,

^c^ 1% confidence level

In terms of visibility (i.e. citations), our analysis suggests that a new scientist is on average seven times more likely of becoming a star if he or she enters the system by the hand of star (Table 13). However, early co-authorship with a researcher that belongs to highly cited groups has in the best case a small effect (at least when compared to entry with a star) on the chances of the new scientist (Table 13 model CV-02) or no effect at all (Table 13 model CV-03 and CV-04). These results suggest that the protégées of highly cited scientists will mimic the success of their mentors.

**Table 13:** Likelihood of becoming a star by type of entry, citations per year, 1984–2001.

Type of Entry (Std.Err.)	CIV-01	CIV-02	CIV-03	CIV-04	CIV-05	CV-01	CV-02	CV-03	CV-04
Star*,citations	6.904^c^					8.240^c^	6.011^c^	8.713^c^	7.084^c^
	(1.141)					(1.226)	(1.097)	(1.276)	(1.302)
coAU in RG, citations		1.865^c^					0.974		
		(0.383)					(0.221)		
coAU in top RG*, citations			4.332^c^			1.229	1.785^b^		
			(0.927)			(0.240)	(0.425)		
coAU-PI of RG, citations				1.444^b^					0.759
				(0.226)					(0.138)
coAU-PI of top RG*, citations					4.141^c^			1.004	1.511
					(1.150)			(0.242)	(0.458)

* Star and top RG defined based on the average plus one standard deviation

^a^ 10% confidence level,

^b^ 5% confidence level,

^c^ 1% confidence level

In terms of the impact that combined environments (i.e. different type of eminent scientist—articles vs. citations—or other forms of early collaborations) have on the likelihood of replicating success, Table 14 shows that a new scientist is more likely to become a star (based on articles) if he or she enters the system with a highly cited researcher instead of a highly productive one (models AVI-01, AVI-03 and AVI-05). In addition, early collaboration with researchers belonging to highly productive groups also has a positive and significant effect on the chances of the new scientist (models AVI-02 to AVI-05). Furthermore, models CVI-01, CVI-03 and CVI-05 of Table 12 show that entry with a highly cited scientist has the highest effect, compared to any other form of entry, on the likelihood of new researchers to replicate the same success. Models CVI-02 and CVI-03 show that early collaboration with a co-author that belongs to a top RG based on citations also has a positive effect of the chances of becoming a highly visible star.

**Table 14:** Likelihood of becoming a star by type of entry, 1984–2001.

Type of Entry (Std.Err.)	Based on articles per year					Based on citations per year				
	AVI-01	AVI-02	AVI-03	AVI-04	AVI-05	CVI-01	CVI-02	CVI-03	CVI-04	CVI-05
Star*, articles	0.593^c^		1.607^c^		1.556^b^	0.560^b^		0.600^b^		0.566^b^
	(0.169)		(0.279)		(0.270)	(0.126)		(0.139)		(0.130)
Star*, citations	0.812^c^		2.104^c^		2.159^c^	9.847^c^		8.184^c^		9.218^c^
	(0.176)		(0.390)		(0.397)	(2.161)		(1.842)		(2.077)
coAU in top RG*, articles		2.580^c^	2.005^c^				0.537^b^	0.584		
		(0.638)	(0.513)				(0.170)	(0.192)		
coAU in top RG*, citations		1.472	0.961				6.466^c^	2.571^c^		
		(0.397)	(0.270)				(1.924)	(0.806)		
coAU-PI in top RG*, articles				4.195^c^	3.083^c^				0.944	0.892
				(1.228)	(0.941)				(0.355)	(0.335)
coAU-PI in top RG*, citations				1.327	0.812				4.289^c^	1.521
				(0.450)	(0.286)				(1.542)	(0.556)

* Star and top RG defined based on the average plus one standard deviation

^a^ 10% confidence level,

^b^ 5% confidence level,

^c^ 1% confidence level

Overall, these results suggest that highly productive and cited researchers have a positive and significant influence on the productivity of early career scientist and their ability to replicate the same success. In addition, the outcome of these models suggests that early collaboration with scientists that are embedded in productive environments also enhances the productivity of new researchers.

## 6. Conclusions and policy implications

In the last decades ST&I have been seen as a major source of economic growth.

To be able to leverage ST&I, government officials and policy makers around the world have developed policies to improve their systems. At the core of these initiatives are strategies to expand the scientific base and to generate, attract and retain highly talented scholars. The main rational behind these developments is that key scientists play a vital role in the growth of the system.

This paper contributes to this body of knowledge by assessing the impact key scientists have in the development of the system. In particular, this work quantifies the influence that star scientists have, by themselves and within the context of different research environments, on the productivity and impact of young faculty, as well as on the likelihood of also becoming a leading personality in science.

Our analysis confirms our expectations and previous results that eminent scientist have a prime role in the development of a scientific system, especially within the context of an emerging economy like Mexico. In particular, in terms of productivity and visibility, this work shows that within 1984 and 2001 the elite group of physicists in Mexico (approximate 10% of all scientists working in physics and its related fields) published 42% of all publications, received 50% of all citations and bred 18% to 26% of new entrants.

In addition our work shows that scientists that enter the system by the hand of a highly productive researcher increased their productivity on average by 28% and the ones that did it by the hand of a highly visible scientist received on average 141% more citations, vis-à-vis scholars that did not published their first manuscripts with an eminent scientist. Furthermore, incoming scientist also had an additional boost on their productivity if they were exposed early on in their careers to the appropriate research environment. For example, young faculty increased their publication rate by 18% if they had an early collaboration with a scientist that belong to a highly productive research group, and 38% if this collaborator was the leader of the group. But these environments did not have any effect on the citation rate of new faculty. In summary, key scientists have a positive and significant influence on the productivity and visibility of young faculty, but nurturing environments only impact their productivity.

In terms of mimicking success, this work suggests that scientists working at the beginning of their careers with eminent researchers tend to replicate the success of their mentors. In particular, scholars that enter the system by the hand of a highly productive researcher were on average 2.5 more likely to also become a star, when compared to the ones that did their initial work with non-star scientists; and early collaboration with highly visible researchers increased 7.4 the chances of a new scientists of mimicking the success of their mentors. In addition, early collaboration with scholars belonging to a highly productive group and the leaders of such ensembles also had on average an additional impact of 2.2 on the probability of someone becoming a leading personality in science; whereas early collaboration with a co-author that belongs to a highly visible group was the only environment that had an additional effect on someone’s likelihood of becoming a highly visible scientist, in this case by 1.8 chance.

These results could suggest that eminent scientists have a primary role in the growth of these systems and the development and productivity of young faculty. In addition, they could insinuate that nurturing envelopments play an additional role in the construction of these systems. This means that if a country or region wants to improve or become the leader in a certain area of knowledge it should focused on attracting and retaining the best and the brightest, and creating around these key figures appropriate collaborative environments so research and researchers can flourish.

This paper has important limitations. Probably the most important limitation is the selection problem. It is highly likely that the best and well known scientists select the best students to work with, as the best universities select the best students. Even though, our model controls for unobserved heterogeneity between year of entry and institution of entry, we are unable to control for individual unobserved heterogeneity. Second, we are assuming that the probability of future success of young researchers depends on working previously with star scientists, assuming that young researchers follow similar career path. To this purpose, we narrow the areas of knowledge by selecting only papers in the areas of Physics and Applied Physics, Condensed Matter and Materials Science so, all researchers that are not publishing in these areas of knowledge are excluded from the analysis. This implies that publications in journals such as Nature or Science, that are under the category of multidisciplinary sciences, are not included in the analysis. Notwithstanding, we acknowledge that mimicking success would require additional evidence and a more detailed analysis of career dynamics. Despite these important limitations, we believe that this research advances our knowledge on the development of a science and technology system.

## Supporting information

S1 File This file has the database of that was used in the analysis of the paper. (PDF)
