# Supplementary material for: Birth of prominent scientists
Source: PLoS One. 2018 Mar 15;13(3):e0193374. doi: 10.1371/journal.pone.0193374 (PMC5854281; doi:10.1371/journal.pone.0193374)
Supplement: S1 File — (PDF) [file pone.0193374.s001.pdf]

nnau	nnau_id	douubleautho	entryorg	dorg	entryorgid	entryorgid_in	entryorgii
ABOITES, V	1	FALSE	CTR INVEST O	FALSE	148	1	CTR INVEST O
ABURTO, S	2	#REF!	UNAM	FALSE	698	1	UNAM
ACEVEDO, AM	4	FALSE	CINVESTAV	FALSE	94	1	CINVESTAV
ACEVES, AB	5	FALSE	UNIV NEW M	FALSE	918	1	UNIV NEW M
ACEVES, R	6	TRUE		FALSE		0	DELFT UNIV T
ACEVES, R	7	TRUE	RES INST IND	FALSE	577	1	RES INST IND
ACEVES, R	8	TRUE		TRUE		0	UAM I
ACEVES, R	9	FALSE		FALSE		0	UNIV SONORA
ACEVESMIJAR	11	TRUE		FALSE		0	BUAP
ACEVESMIJAR	12	FALSE	INAOE	FALSE	276	1	INAOE
ACOCAL, JLC	13	FALSE		FALSE		0	BUAP
ACOSTA, DR	14	FALSE	UNAM	FALSE	698	1	UNAM
ACOSTA-ALEJ	18	FALSE		FALSE		0	CINVESTAV
ACOSTA-ORTI	19	FALSE	UASLP	FALSE	689	1	UASLP
ACUNA-CAMP	20	TRUE		FALSE		0	UASLP
ACUNA-CAMP	21	FALSE	UNIV SONORA	FALSE	987	1	UNIV SONORA
AGUERO-GRA	25	FALSE	UAZ	FALSE	690	1	UAZ
AGUIAR, JA	26	FALSE		FALSE		0	UNIV FED PER
AGUILAR, A	27	TRUE	UAM I	FALSE	686	1	UAM I
AGUILAR, A	28	FALSE	UNAM	FALSE	698	1	UNAM
AGUILAR, J	29	FALSE	UANL	FALSE	687	1	UANL
AGUILAR, JF	30	FALSE		FALSE		0	CICESE
AGUILAR, MA	33	TRUE	CINVESTAV	FALSE	94	1	CINVESTAV
AGUILAR, MA	34	TRUE		TRUE		0	UNAM
AGUILAR, MA	35	FALSE		FALSE		0	UNIV AUTONO
AGUILAR, MR	37	FALSE		FALSE		0	UNAM
AGUILAR, PAM	38	TRUE		FALSE		0	HUGHES
AGUILAR, PAM	39	FALSE	INAOE	FALSE	276	1	INAOE
AGUILAR, VV	40	FALSE	VANDERBILT U	FALSE	1048	1	VANDERBILT U
AGUILAR-FRU	41	FALSE	CINVESTAV	FALSE	94	1	CINVESTAV
AGUILAR-HER	42	FALSE		FALSE		0	IPN
AGUILERA, EF	45	FALSE		FALSE		0	ININ
AGUSTIN, FSG	55	FALSE		FALSE		0	INAOE
AKACHI, T	59	FALSE	UNAM	FALSE	698	1	UNAM
ALARCON-WA	67	FALSE		FALSE		0	UDLAP
ALAVI, A	68	TRUE	UAM A	FALSE	685	1	UAM A
ALAVI, A	69	FALSE		FALSE		0	UNAM
ALBA, F	71	FALSE	UNAM	FALSE	698	1	UNAM
ALBARRAN, G	73	FALSE	UNAM	FALSE	698	1	UNAM
ALBARRAN, JL	74	FALSE		FALSE		0	UNAM
ALDANA, M	79	FALSE		FALSE		0	UNAM
ALEJO-ARMEN	82	FALSE	UNIV AUTONO	FALSE	728	1	UNIV AUTONO
ALEJO-ARMEN	83	TRUE	CINVESTAV	FALSE	94	1	CINVESTAV
ALEJO-ARMEN	84	TRUE	UAQ	FALSE	688	1	UAQ
ALEJO-ARMEN	85	FALSE		FALSE		0	UNIV AUTONO
ALEXANDERKA	89	TRUE	UAM I	FALSE	686	1	UAM I

ALEXANDERK/	90	FALSE		FALSE		0	UNAM
ALFARO, J	93	FALSE	PONTIFICIA U	FALSE	562	1	PONTIFICIA U
ALGORA, A	97	FALSE	UNAM	FALSE	698	1	UNAM
ALIEV, R	100	FALSE	UNAM	FALSE	698	1	UNAM
ALLRED, DD	103	FALSE	BRIGHAM YOU	FALSE	46	1	BRIGHAM YOU
ALMAGUERAI	105	FALSE	UNAM	FALSE	698	1	UNAM
ALMANZA, R	106	FALSE	UNAM	FALSE	698	1	UNAM
ALONSO, JC	111	FALSE	UNAM	FALSE	698	1	UNAM
ALONSO, MA	112	FALSE	UNAM	FALSE	698	1	UNAM
ALVARADO, F	118	FALSE	IPN	FALSE	376	1	IPN
ALVARADO, M	119	FALSE	UNAM	FALSE	698	1	UNAM
ALVARADO, R	120	FALSE		FALSE		0	UNIV AUTON(
ALVARADO-G	121	FALSE	UNIV MAINZ	FALSE	880	1	UNIV MAINZ
ALVARADO-LE	122	FALSE	UASLP	FALSE	689	1	UASLP
ALVARADO-R(	123	TRUE	UNIV AUTON(	FALSE	725	1	UNIV AUTON(
ALVARADO-R(	124	FALSE		FALSE		0	UNIV CALIF LC
ALVAREZ, E	126	FALSE	UAM I	FALSE	686	1	UAM I
ALVAREZ, LJ	132	FALSE		FALSE		0	UNIV CAMBRI
ALVAREZ-BOF	134	FALSE		FALSE		0	CICESE
ALVAREZ-CAB	135	FALSE	IPN	FALSE	376	1	IPN
ALVAREZ-FRE(	137	TRUE		FALSE		0	CINVESTAV
ALVAREZ-FRE(	138	FALSE	UNAM	FALSE	698	1	UNAM
ALVAREZGAR(	139	FALSE		FALSE		0	CENIDET
ALVAREZ-RAM	141	FALSE		FALSE		0	UAM I
ALVAREZ-ROM	142	TRUE	ININ	FALSE	289	1	ININ
ALVAREZ-ROM	143	FALSE		FALSE		0	UAM I
AMADOR, C	145	FALSE		FALSE		0	UNAM
AMANO, E	146	FALSE		FALSE		0	UNAM
AMAYA-TAPI/	148	FALSE	UNIV CONNE(	FALSE	776	1	UNIV CONNE(
AMORIN, H	151	TRUE		TRUE		0	CICESE
AMORIN, H	152	TRUE		FALSE		0	UNAM
AMORIN, H	153	TRUE		TRUE		0	UNIV HAVAN/
AMORIN, H	154	TRUE		TRUE		0	UNIV LA HAB/
AMORIN, H	155	FALSE		FALSE		0	UNIV ORIENTI
ANDERSON, JI	159	TRUE	STEVENS INST	FALSE	634	1	STEVENS INST
ANDERSON, JI	160	FALSE		FALSE		0	UNAM
ANDRADE, J	162	FALSE	UNAM	FALSE	698	1	UNAM
ANDRADE, L	163	FALSE	UNAM	FALSE	698	1	UNAM
ANDRADE-IB/	164	FALSE		FALSE		0	UNAM
ANDRADE-LU(	165	TRUE	INAOE	FALSE	276	1	INAOE
ANDRADE-LU(	166	TRUE		TRUE		0	Preparat Fed (
ANDRADE-LU(	167	FALSE		FALSE		0	UNIV GUANA.
ANDRES, P	170	TRUE		FALSE		0	INAOE
ANDRES, P	171	FALSE	UNIV VALENC	FALSE	1010	1	UNIV VALENC
ANGULO-BRC	174	FALSE	UAM I	FALSE	686	1	UAM I
ANJOS, JC	175	FALSE	CTR BRASILEII	FALSE	135	1	CTR BRASILEII
ANTONIOU, I	181	TRUE	CINVESTAV	FALSE	94	1	CINVESTAV

ANTONIOU, I	182	FALSE		FALSE		0 FREE UNIV BR
ANZALDO-ME	183	FALSE	UAM A	FALSE	685	1 UAM A
APATIGA, LM	184	FALSE		FALSE		0 UNAM
APOLINAR-IRI	185	FALSE	INAOE	FALSE	276	1 INAOE
AQUINO, N	190	FALSE	UAM I	FALSE	686	1 UAM I
ARAGON, G	191	FALSE	UAM A	FALSE	685	1 UAM A
ARAGON, JL	192	TRUE		FALSE		0 IPN
ARAGON, JL	193	FALSE	UNAM	FALSE	698	1 UNAM
ARAIZA, JJ	196	FALSE	CINVESTAV	FALSE	94	1 CINVESTAV
ARAIZA, LC	197	FALSE	UNAM	FALSE	698	1 UNAM
ARANCIBIA-BU	200	FALSE	INAOE	FALSE	276	1 INAOE
ARANDA, JI	202	TRUE		FALSE		0 BUAP
ARANDA, JI	203	FALSE	CINVESTAV	FALSE	94	1 CINVESTAV
ARCE, H	205	FALSE	UNAM	FALSE	698	1 UNAM
ARENAS, J	210	FALSE	INAH	FALSE	275	1 INAH
ARENCIBIA, P	212	FALSE	UNIV LA HABA	FALSE	855	1 UNIV LA HABA
ARES, O	213	FALSE	CINVESTAV	FALSE	94	1 CINVESTAV
ARESDEPARGA	214	TRUE	IPN	FALSE	376	1 IPN
ARESDEPARGA	215	FALSE	UAM I	FALSE	686	1 UAM I
AREVALO-AGU	216	FALSE		FALSE		0 INAOE
ARIAS, JM	218	FALSE	UNIV SEVILLA	FALSE	982	1 UNIV SEVILLA
ARIAS-HERNA	219	TRUE	IPN	FALSE	376	1 IPN
ARIAS-HERNA	220	FALSE	UAM A	FALSE	685	1 UAM A
ARITA, IH	222	FALSE	UNAM	FALSE	698	1 UNAM
ARIZA-CALDEI	224	FALSE	UNIV QUINDI	FALSE	955	1 UNIV QUINDI
ARIZPE-CHAV	227	TRUE		FALSE		0 CINVESTAV
ARIZPE-CHAV	228	FALSE	UNIV SONORA	FALSE	987	1 UNIV SONORA
ARRIAGA, J	235	TRUE	BUAP	FALSE	51	1 BUAP
ARRIAGA, J	236	FALSE		FALSE		0 INAOE
ARRIZON, V	239	TRUE		FALSE		0 BUAP
ARRIZON, V	240	FALSE		FALSE		0 INAOE
ARROYO, R	242	FALSE	UAM I	FALSE	686	1 UAM I
ARTACHO, E	243	TRUE		FALSE		0 CSIC
ARTACHO, E	244	FALSE		FALSE		0 UNIV AUTONO
ARZATE, N	245	FALSE		FALSE		0 CTR INVEST O
ASAJI, T	247	TRUE	INST MOLEC S	FALSE	324	1 INST MOLEC S
ASAJI, T	248	FALSE	NAGOYA UNIV	FALSE	491	1 NAGOYA UNIV
ASCENCIO, JA	249	TRUE		FALSE		0 ININ
ASCENCIO, JA	250	FALSE		FALSE		0 UNAM
ASHTEKAR, A	252	FALSE	PENN STATE U	FALSE	553	1 PENN STATE U
ASOMOZA, M	253	TRUE	IMP	FALSE	272	1 IMP
ASOMOZA, M	254	FALSE		FALSE		0 UAM I
ASOMOZA, R	255	FALSE		FALSE		0 CINVESTAV
ASTORGA, F	258	FALSE	UNIV MICHOA	FALSE	888	1 UNIV MICHOA
ATAKISHIYEV,	259	TRUE	ACAD SCI AZS	FALSE	4	1 ACAD SCI AZS
ATAKISHIYEV,	260	FALSE	UNAM	FALSE	698	1 UNAM
AUDIERE, JP	265	FALSE	UAM I	FALSE	686	1 UAM I

AUGER, G	266	TRUE	INST SCI MAT	FALSE	347	1	INST SCI MAT
AUGER, G	267	FALSE		FALSE		0	ISMRA UNIV
AVALOS-BORJ	270	TRUE	UNAM	FALSE	698	1	UNAM
AVALOS-BORJ	271	TRUE	VIRGINIA COM	FALSE	1051	1	VIRGINIA COM
AVALOS-BORJ	272	FALSE	XEROX CORP	FALSE	1063	1	XEROX CORP
AVENDANOLC	274	FALSE	IPN	FALSE	376	1	IPN
AVIGNON, M	275	TRUE		FALSE		0	BUAP
AVIGNON, M	276	FALSE		FALSE		0	CNRS
AVILA, A	277	FALSE	CINVESTAV	FALSE	94	1	CINVESTAV
AVILA, MA	279	TRUE	UNAM	FALSE	698	1	UNAM
AVILA, MA	280	FALSE		FALSE		0	UNIV AUTONC
AVILA, O	281	TRUE	ININ	FALSE	289	1	ININ
AVILA, O	282	FALSE		FALSE		0	UNAM
AYALA, A	287	FALSE	UNAM	FALSE	698	1	UNAM
AYALA-VALEN	289	TRUE		FALSE		0	UNAM
AYALA-VALEN	290	FALSE	UNIV LA HAB/	FALSE	855	1	UNIV LA HAB/
AYON-BEATO,	292	FALSE		FALSE		0	CINVESTAV
AYTA, WEF	293	TRUE	ATOM ENERG	FALSE	28	1	ATOM ENERG
AYTA, WEF	294	TRUE		TRUE		0	UNIV SAO PAU
AYTA, WEF	295	FALSE		FALSE		0	UNIV SONOR/
AZAMAR-BAR	298	FALSE	UNAM	FALSE	698	1	UNAM
AZORIN- NIET	299	FALSE	ININ	FALSE	289	1	ININ
BABIN, V	301	FALSE	UNIV SONOR/	FALSE	987	1	UNIV SONOR/
BAEZ, NB	310	TRUE		FALSE		0	CINVESTAV
BAEZ, NB	311	FALSE	IPN	FALSE	376	1	IPN
BAEZ-ROJAS, .	312	FALSE	INAOE	FALSE	276	1	INAOE
BAGNOLI, F	314	FALSE	UNIV FLOREN	FALSE	810	1	UNIV FLOREN
BAHRI, C	315	FALSE	LOUISIANA ST	FALSE	451	1	LOUISIANA ST
BALANKIN, AS	321	FALSE	ITESM	FALSE	385	1	ITESM
BALBAS, LC	323	FALSE		FALSE		0	UNIV STRASB(
BALCAZAR, M	324	TRUE		FALSE		0	FDN CLIN MEI
BALCAZAR, M	325	FALSE	ININ	FALSE	289	1	ININ
BALDERAS-NA	327	FALSE	UASLP	FALSE	689	1	UASLP
BALMORI-RAI	329	FALSE	IPN	FALSE	376	1	IPN
BALTAGA, IV	330	TRUE	ALL RUSSIAN	FALSE	16	1	ALL RUSSIAN
BALTAGA, IV	331	FALSE		FALSE		0	UKRAINIAN A
BANOS, L	335	FALSE	UNAM	FALSE	698	1	UNAM
BARA, S	338	FALSE	UNAM	FALSE	698	1	UNAM
BARANGER, H	341	FALSE		FALSE		0	AT&T
BARBOSA-GA	346	FALSE	CTR INVEST O	FALSE	148	1	CTR INVEST O
BARBOZA-FLC	347	FALSE		FALSE		0	UNIV SONOR/
BARMENKOV,	350	TRUE		FALSE		0	CTR INVEST O
BARMENKOV,	351	FALSE	POLAROID CC	FALSE	558	1	POLAROID CC
BARRAGAN-V	354	FALSE	UNAM	FALSE	698	1	UNAM
BARRANCO, A	355	TRUE		TRUE		0	UNAM
BARRANCO, A	356	TRUE		FALSE		0	UNIV HAVAN/
BARRANCO, A	357	FALSE	UNIV LA HAB/	FALSE	855	1	UNIV LA HAB/

BARRANON, A	359	FALSE		FALSE		0	UAM A
BARREIRO, JC	362	FALSE	UNIV VALENC	FALSE	1010	1	UNIV VALENC
BARRERA-CAL	366	FALSE		FALSE		0	UAM I
BARRIENTOS,	368	FALSE	CTR INVEST O	FALSE	148	1	CTR INVEST O
BARRIENTOS,	369	TRUE	UAM A	FALSE	685	1	UAM A
BARRIENTOS,	370	FALSE		FALSE		0	UAM I
BARTOLO-PEF	376	TRUE		TRUE		0	CINVESTAV
BARTOLO-PEF	377	TRUE		FALSE		0	IPN
BARTOLO-PEF	378	FALSE		FALSE		0	UNAM
BARTOS, J	379	FALSE	SLOVAK ACAD	FALSE	613	1	SLOVAK ACAD
BASHIR, A	381	TRUE		TRUE		0	UNAM
BASHIR, A	382	FALSE		FALSE		0	UNIV MICHOA
BASURTO-PEN	386	TRUE		FALSE		0	INAOE
BASURTO-PEN	387	FALSE	UNIV GUANA.	FALSE	823	1	UNIV GUANA.
BAUDET, C	390	FALSE	UNAM	FALSE	698	1	UNAM
BAUDIN, T	391	TRUE		FALSE		0	ININ
BAUDIN, T	392	FALSE	IPN	FALSE	376	1	IPN
BAUSA, LE	397	FALSE	UNIV AUTON(	FALSE	727	1	UNIV AUTON(
BAUTISTA, C	399	FALSE	BUAP	FALSE	51	1	BUAP
BAUTISTA, F	401	TRUE	UNAM	FALSE	698	1	UNAM
BAUTISTA, F	402	FALSE		FALSE		0	UNIV GUADAI
BAUTISTA, R	403	FALSE	UNAM	FALSE	698	1	UNAM
BECERRIL, M	406	TRUE	CINVESTAV	FALSE	94	1	CINVESTAV
BECERRIL, M	407	FALSE		FALSE		0	UNIV SONORA
BECERRIL, R	408	TRUE	CINVESTAV	FALSE	94	1	CINVESTAV
BECERRIL, R	409	FALSE		FALSE		0	UAM A
BEITIA, C	415	TRUE	OHIO UNIV	FALSE	539	1	OHIO UNIV
BEITIA, C	416	TRUE		FALSE		0	UNIV PARIS 6
BEITIA, C	417	FALSE	UNIV ROMA T	FALSE	963	1	UNIV ROMA T
BELMONT-MC	420	TRUE	COMIS NACL I	FALSE	116	1	COMIS NACL I
BELMONT-MC	421	FALSE		FALSE		0	UNAM
BELTRAN, FJE	423	TRUE	CINVESTAV	FALSE	94	1	CINVESTAV
BELTRAN, FJE	424	TRUE		FALSE		0	INST TECNOL
BELTRAN, FJE	425	FALSE	UAQ	FALSE	688	1	UAQ
BELTRAN, MR	427	FALSE		FALSE		0	UNAM
BELTRAN-LOP	429	FALSE	UNAM	FALSE	698	1	UNAM
BELTRAN-PER	430	FALSE	INAOE	FALSE	276	1	INAOE
BELYAEVA, TL	431	FALSE	UNIV JENA	FALSE	845	1	UNIV JENA
BENAISSA, M	433	TRUE		TRUE		0	ININ
BENAISSA, M	434	TRUE		TRUE		0	UNAM
BENAISSA, M	435	TRUE		TRUE		0	UNIV CALIF BI
BENAISSA, M	436	FALSE		FALSE		0	UNIV CONNE(
BENAVIDES, A	437	FALSE		FALSE		0	UNIV GUANA.
BENAVRAHAN	438	FALSE	CLARKSON UN	FALSE	99	1	CLARKSON UN
BENET, L	439	TRUE		FALSE		0	UNAM
BENET, L	440	FALSE	UNIV BASEL	FALSE	734	1	UNIV BASEL
BENITEZ, J	443	FALSE	UAM I	FALSE	686	1	UAM I

BERGUES, JM	451	TRUE		FALSE		0	Univ Oriente A
BERGUES, JM	452	FALSE		FALSE		0	UNIV SONORA
BERISHEV, IE	453	FALSE		FALSE		0	UASLP
BERISSO, MC	454	TRUE		FALSE		0	UNIV GUANAJ
BERISSO, MC	455	FALSE	UNIV MASSAC	FALSE	886	1	UNIV MASSAC
BERMAN, GP	456	FALSE		FALSE		0	LOS ALAMOS
BERNAL, R	457	TRUE	CICESE	FALSE	89	1	CICESE
BERNAL, R	458	FALSE		FALSE		0	CINVESTAV
BERNAL-ALVA	460	FALSE	UNIV GUANAJ	FALSE	823	1	UNIV GUANAJ
BERNICHA, A	462	FALSE		FALSE		0	CATHOLIC UN
BERRIEL-VALD	465	FALSE	INAOE	FALSE	276	1	INAOE
BERRONDO, M	466	FALSE	UNAM	FALSE	698	1	UNAM
BESCOS, J	470	TRUE		FALSE		0	Fdn Ramon Ar
BESCOS, J	471	FALSE	INAOE	FALSE	276	1	INAOE
BESPROSVAN	472	FALSE	UNAM	FALSE	698	1	UNAM
BETANCOURT	473	FALSE	UNAM	FALSE	698	1	UNAM
BETANCOURT	474	TRUE	UNIV ORIENTE	FALSE	927	1	UNIV ORIENTE
BETANCOURT	475	TRUE		TRUE		0	Univ Oriente A
BETANCOURT	476	FALSE		FALSE		0	UNIV SONORA
BEUNARD, R	477	TRUE		FALSE		0	IPN
BEUNARD, R	478	FALSE	KERNFYS VERS	FALSE	403	1	KERNFYS VERS
BEUSCHEL, T	480	TRUE	LOUISIANA ST	FALSE	451	1	LOUISIANA ST
BEUSCHEL, T	481	TRUE		FALSE		0	UNAM
BEUSCHEL, T	482	FALSE	UNIV GIESSEN	FALSE	818	1	UNIV GIESSEN
BEUTHE, M	483	FALSE		FALSE		0	CATHOLIC UN
BHASKARA, K	487	FALSE	CINVESTAV	FALSE	94	1	CINVESTAV
BHATTACHAR	488	TRUE		FALSE		0	UNAM
BHATTACHAR	489	FALSE	UNIV COLORA	FALSE	774	1	UNIV COLORA
BIANCHI, L	490	TRUE	INST PHYS NU	FALSE	342	1	INST PHYS NU
BIANCHI, L	491	FALSE		FALSE		0	KERNFYS VERS
BIJKER, R	495	TRUE		TRUE		0	IST NAZL FIS N
BIJKER, R	496	TRUE		TRUE		0	UNAM
BIJKER, R	497	FALSE		FALSE		0	UNIV SEVILLA
BISHOP, AR	503	FALSE	CINVESTAV	FALSE	94	1	CINVESTAV
BLAISTEN-BAR	506	FALSE	UNAM	FALSE	698	1	UNAM
BLANCO, JM	509	FALSE	UNAM	FALSE	698	1	UNAM
BLATTER, G	511	FALSE	ETH ZURICH	FALSE	205	1	ETH ZURICH
BLUM, L	517	FALSE		FALSE		0	UNIV PUERTO
BLUMENFELD	519	TRUE	IPN	FALSE	376	1	IPN
BLUMENFELD	520	FALSE		FALSE		0	UNIV IDAHO
BOGDANCHIK	527	TRUE		TRUE		0	BELARUSSIAN
BOGDANCHIK	528	TRUE	UNIV NACL AU	FALSE	902	1	UNIV NACL AU
BOGDANCHIK	529	FALSE	UNIV SOUTHA	FALSE	988	1	UNIV SOUTHA
BOKHIMI, X	535	FALSE	UNAM	FALSE	698	1	UNAM
BOLDU, JL	536	FALSE	UNAM	FALSE	698	1	UNAM
BOLOTIN, YL	539	FALSE		FALSE		0	KHARKOV PHY
BONET, F	542	FALSE	UNIV PICARDI	FALSE	946	1	UNIV PICARDI

BORENSZTEIN	546	TRUE		FALSE		0	UNIV PARIS 6
BORENSZTEIN	547	FALSE	UNIV PIERRE &	FALSE	947	1	UNIV PIERRE &
BORJA, MA	548	FALSE	NATL INST STA	FALSE	508	1	NATL INST STA
BOSCH, P	555	FALSE	ININ	FALSE	289	1	ININ
BOTELLO, S	556	TRUE	CIMAT	FALSE	93	1	CIMAT
BOTELLO, S	557	FALSE		FALSE		0	CTR INVEST O
BOUARAB, S	559	FALSE	CINVESTAV	FALSE	94	1	CINVESTAV
BOULLOSA, RI	562	FALSE	UNAM	FALSE	698	1	UNAM
BOUZAS, AO	565	FALSE		FALSE		0	CINVESTAV
BOZHEVOLNY	572	TRUE	TECH UNIV DE	FALSE	651	1	TECH UNIV DE
BOZHEVOLNY	573	FALSE		FALSE		0	UNAM
BOZZOLO, G	574	FALSE		FALSE		0	ANALEX CORP
BRAVO, A	579	TRUE	Colegio Bachi	FALSE	107	1	Colegio Bachi
BRAVO, A	580	FALSE		FALSE		0	ITESM
BRAY, AJ	583	FALSE	UNIV MANCH	FALSE	881	1	UNIV MANCH
BRETON, N	585	TRUE		FALSE		0	BUAP
BRETON, N	586	FALSE	CINVESTAV	FALSE	94	1	CINVESTAV
BRIGGS, A	592	TRUE	CTR RECH TRE	FALSE	154	1	CTR RECH TRE
BRIGGS, A	593	FALSE		FALSE		0	UNIV RENNES
BRITO-ORTA,	598	FALSE	BUAP	FALSE	51	1	BUAP
BRODY, TA	602	FALSE	UNAM	FALSE	698	1	UNAM
BROWN, FA	604	FALSE	IPN	FALSE	376	1	IPN
BRUCE, NC	605	FALSE		FALSE		0	UNAM
BRUCE, S	606	TRUE	CONCEPCION	FALSE	118	1	CONCEPCION
BRUCE, S	607	FALSE		FALSE		0	UNIV GUADAI
BRUDNY, VL	609	TRUE	CTR INVEST O	FALSE	148	1	CTR INVEST O
BRUDNY, VL	610	TRUE		TRUE		0	UNAM
BRUDNY, VL	611	TRUE		FALSE		0	UNIV AUTONO
BRUDNY, VL	612	TRUE	UNIV BUENOS	FALSE	745	1	UNIV BUENOS
BRUDNY, VL	613	FALSE	UNIV NIJMEG	FALSE	921	1	UNIV NIJMEG
BUCIO, E	614	FALSE	UNAM	FALSE	698	1	UNAM
BUENFIL, AE	618	FALSE	UNAM	FALSE	698	1	UNAM
BULAEVSKII, L	622	FALSE		FALSE		0	UASLP
BULBULIAN, S	624	FALSE	UAM I	FALSE	686	1	UAM I
BUNGE, AV	625	TRUE		FALSE		0	UAM I
BUNGE, AV	626	FALSE	UNAM	FALSE	698	1	UNAM
BURGESS, CP	628	FALSE		FALSE		0	MCGILL UNIV
BURLAK, GN	630	FALSE	Kiev Natl Univ	FALSE	411	1	Kiev Natl Univ
BURNSTEIN, F	632	TRUE	IIT	FALSE	266	1	IIT
BURNSTEIN, F	633	FALSE		FALSE		0	UNIV LAUSAN
BUSE, K	634	FALSE		FALSE		0	UNIV OSNABF
CABALLERO-B	641	TRUE		TRUE		0	CINVESTAV
CABALLERO-B	642	FALSE		FALSE		0	UNIV LA HABA
CABANAS-MC	643	TRUE		FALSE		0	IPN
CABANAS-MC	644	FALSE	NORTHWESTE	FALSE	530	1	NORTHWESTE
CABO, A	646	TRUE		FALSE		0	INT CTR THEO
CABO, A	647	FALSE		FALSE		0	UNIV GUANA

CABRAL-PRIE	649	FALSE	ININ	FALSE	289	1	ININ
CABRERA, A	650	TRUE	CINVESTAV	FALSE	94	1	CINVESTAV
CABRERA, A	651	FALSE		FALSE		0	UNAM
CABRERA, E	652	FALSE	UNAM	FALSE	698	1	UNAM
CABRERATRU	653	FALSE	UASLP	FALSE	689	1	UASLP
CALDERON, A	658	FALSE		FALSE		0	CINVESTAV
CALDERON, F	659	FALSE	UNIV LA HAB	FALSE	855	1	UNIV LA HAB
CALDERON, H	660	FALSE	Swiss Fed Inst	FALSE	642	1	Swiss Fed Inst
CALDERON, IH	661	FALSE	CINVESTAV	FALSE	94	1	CINVESTAV
CALDINO, U	662	TRUE		FALSE		0	UAM A
CALDINO, U	663	FALSE	UAM I	FALSE	686	1	UAM I
CALDWELL, CI	665	TRUE	NATL INST ST	FALSE	508	1	NATL INST ST
CALDWELL, CI	666	FALSE		FALSE		0	UNIV CENT FL
CALEYO, F	667	FALSE	ININ	FALSE	289	1	ININ
CALIXTO, ME	668	TRUE		FALSE		0	UNAM
CALIXTO, ME	669	FALSE	UNIV STRASB	FALSE	990	1	UNIV STRASB
CALIXTO, S	670	FALSE		FALSE		0	CTR INVEST O
CALLEJA, W	671	FALSE	CINVESTAV	FALSE	94	1	CINVESTAV
CALLES, AG	673	FALSE		FALSE		0	UNAM
CALOGERO, F	674	FALSE		FALSE		0	UNAM
CAMACHO, A	676	FALSE		FALSE		0	CTR INVEST O
CAMACHO, A	677	FALSE	CINVESTAV	FALSE	94	1	CINVESTAV
CAMACHO-BA	683	TRUE		FALSE		0	BUAP
CAMACHO-BA	684	FALSE	INAOE	FALSE	276	1	INAOE
CAMACHO-LO	686	TRUE	UAM I	FALSE	686	1	UAM I
CAMACHO-LO	687	FALSE		FALSE		0	UNIV PARIS 6
CAMARILLO, E	688	FALSE		FALSE		0	UNAM
CAMPA-MOLI	690	FALSE	UNIV GUADAI	FALSE	822	1	UNIV GUADAI
CAMPERO, A	692	FALSE	UNIV PARIS 6	FALSE	940	1	UNIV PARIS 6
CAMPILLO, B	694	FALSE	UNAM	FALSE	698	1	UNAM
CAMPOS, I	695	FALSE	UNAM	FALSE	698	1	UNAM
CAMPOS, J	696	TRUE		FALSE		0	BUAP
CAMPOS, J	697	TRUE	UNAM	FALSE	698	1	UNAM
CAMPOS, J	698	FALSE		FALSE		0	UNIV HAVAN
CAMPOS, RG	700	FALSE	UNIV MICHO	FALSE	888	1	UNIV MICHO
CAMPOY, G	703	FALSE	UNIV SONOR	FALSE	987	1	UNIV SONOR
CAMPS, E	704	FALSE		FALSE		0	ININ
CANALES-PAL	706	FALSE	UNAM	FALSE	698	1	UNAM
CANIZAL, G	710	FALSE	UNIV PETRO 8	FALSE	945	1	UNIV PETRO 8
CANNON, JR	711	FALSE	WASHINGTON	FALSE	1055	1	WASHINGTON
CANTARERO,	713	FALSE	MAX PLANCK	FALSE	470	1	MAX PLANCK
CANTORAL, E	715	FALSE	BUAP	FALSE	51	1	BUAP
CAPITAN, MJ	721	FALSE	UNIV SEVILLA	FALSE	982	1	UNIV SEVILLA
CAPOVILLA, R	722	FALSE		FALSE		0	CINVESTAV
CARBAJAL-TIN	724	FALSE	UASLP	FALSE	689	1	UASLP
CARBALLO-SA	725	TRUE		TRUE		0	BUAP
CARBALLO-SA	726	FALSE		FALSE		0	CINVESTAV

CARDENAS, M	730	FALSE	CINVESTAV	FALSE	94	1	CINVESTAV
CARDENASGA	731	FALSE	UNAM	FALSE	698	1	UNAM
CARDONA, M	733	TRUE	UASLP	FALSE	689	1	UASLP
CARDONA, M	734	FALSE		FALSE		0	UNIV VALENC
CARDONA-NU	735	FALSE	INAOE	FALSE	276	1	INAOE
CARDOSO, JL	736	TRUE		FALSE		0	AUSTRALIAN
CARDOSO, JL	737	FALSE	UNAM	FALSE	698	1	UNAM
CARLSON, G	741	TRUE	UNIV CONNE	FALSE	776	1	UNIV CONNE
CARLSON, G	742	FALSE		FALSE		0	UNIV LOWELL
CARPIO, JJ	748	TRUE	OLD DOMINI	FALSE	542	1	OLD DOMINIC
CARPIO, JJ	749	FALSE		FALSE		0	UNIV AUTON
CARRANZA, J	752	TRUE	INAOE	FALSE	276	1	INAOE
CARRANZA, J	753	FALSE	UNIV VALENC	FALSE	1010	1	UNIV VALENC
CARRASCO, E	754	FALSE		FALSE		0	UNAM
CARRASCO, M	756	FALSE	INAOE	FALSE	276	1	INAOE
CARRASCOSA	757	TRUE		TRUE		0	UNAM
CARRASCOSA	758	FALSE		FALSE		0	UNIV OXFORD
CARREON, MI	760	FALSE	UNAM	FALSE	698	1	UNAM
CARRERA, LM	762	FALSE	ININ	FALSE	289	1	ININ
CARRILLO-LO	771	TRUE		FALSE		0	BUAP
CARRILLO-LO	772	FALSE		FALSE		0	CINVESTAV
CARTAS-FUEN	773	FALSE		FALSE		0	BUAP
CASADEMUN	776	TRUE	UAM I	FALSE	686	1	UAM I
CASADEMUN	777	FALSE		FALSE		0	UNIV BARCEL
CASANDJIAN,	778	TRUE		FALSE		0	GANIL
CASANDJIAN,	779	FALSE	IFUSP	FALSE	265	1	IFUSP
CASAR, I	780	FALSE		FALSE		0	UNAM
CASAS, M	782	FALSE	UNIV ILLES BA	FALSE	839	1	UNIV ILLES BA
CASASVAZQU	783	TRUE	COLEGIO NAC	FALSE	108	1	COLEGIO NAC
CASASVAZQU	784	FALSE		FALSE		0	UAM I
CASCALES, C	785	FALSE	UNAM	FALSE	698	1	UNAM
CASTANEDA,	789	TRUE		FALSE		0	UNIV AUTON
CASTANEDA,	790	FALSE	UNIV SONOR	FALSE	987	1	UNIV SONOR
CASTANEDA-(	791	FALSE	UNAM	FALSE	698	1	UNAM
CASTANEDO,	792	FALSE	CINVESTAV	FALSE	94	1	CINVESTAV
CASTANEDO-I	793	FALSE	CINVESTAV	FALSE	94	1	CINVESTAV
CASTANO, E	794	FALSE	UAM I	FALSE	686	1	UAM I
CASTANO, VM	796	FALSE		FALSE		0	UNAM
CASTELLANOS	800	FALSE	UNAM	FALSE	698	1	UNAM
CASTELLANOS	801	TRUE	CINVESTAV	FALSE	94	1	CINVESTAV
CASTELLANOS	802	FALSE		FALSE		0	UNIV GUADA
CASTILLO, MD	806	FALSE		FALSE		0	INAOE
CASTILLO, RC	808	FALSE		FALSE		0	UNAM
CASTILLO, S	811	FALSE	UAM A	FALSE	685	1	UAM A
CASTILLO-ALV	812	FALSE	IPN	FALSE	376	1	IPN
CASTILLOALV	813	TRUE		TRUE		0	IPN
CASTILLOALV	814	FALSE		FALSE		0	UNAM

CASTILLOORT	815	FALSE	UNIV SONOR/	FALSE	987	1	UNIV SONOR/
CASTRO, A	819	TRUE	INAOE	FALSE	276	1	INAOE
CASTRO, A	820	FALSE		FALSE		0	UDLAP
CASTRO, C	821	TRUE	CINVESTAV	FALSE	94	1	CINVESTAV
CASTRO, C	822	FALSE		FALSE		0	UNIV GUANA.
CASTRO, GL	823	TRUE		TRUE		0	CINVESTAV
CASTRO, GL	824	FALSE		FALSE		0	UNIV AUTON(
CASTRO, JJ	825	FALSE		FALSE		0	CINVESTAV
CASTRO-BELT	828	FALSE		FALSE		0	INAOE
CASTRO-RAM	829	TRUE		FALSE		0	BUAP
CASTRO-RAM	830	FALSE	INAOE	FALSE	276	1	INAOE
CASTRO-RODI	831	TRUE	CINVESTAV	FALSE	94	1	CINVESTAV
CASTRO-RODI	832	FALSE		FALSE		0	CNR INST
CEDILLO, A	840	FALSE	UAM I	FALSE	686	1	UAM I
CERDEIRA, A	850	FALSE		FALSE		0	CINVESTAV
CERDEIRA, F	851	TRUE	CINVESTAV	FALSE	94	1	CINVESTAV
CERDEIRA, F	852	TRUE		TRUE		0	CPQD TELEBR
CERDEIRA, F	853	TRUE		TRUE		0	UNIV AUTON(
CERDEIRA, F	854	FALSE		FALSE		0	UNIV ESTADU
CERDEIRA, HA	855	TRUE		TRUE		0	INT CTR THEO
CERDEIRA, HA	856	FALSE		FALSE		0	UNIV BUENOS
CERECEDO-NU	857	TRUE		TRUE		0	INAOE
CERECEDO-NU	858	FALSE		FALSE		0	UNIV AUTON(
CERON, VE	859	FALSE		FALSE		0	CINVESTAV
CERVANTES, M	860	TRUE	CTR INVEST O	FALSE	148	1	CTR INVEST O
CERVANTES, M	861	FALSE	UNIV SONOR/	FALSE	987	1	UNIV SONOR/
CERVANTES-C	863	TRUE	ININ	FALSE	289	1	ININ
CERVANTES-C	864	FALSE		FALSE		0	UNIV KONSTA
CETTO, AM	868	FALSE		FALSE		0	UNAM
CHABANE, A	869	TRUE	INST NATL PH	FALSE	328	1	INST NATL PH
CHABANE, A	870	FALSE		FALSE		0	UNIV GRENOE
CHACON, M	872	FALSE	UNAM	FALSE	698	1	UNAM
CHADDERTON	873	TRUE		FALSE		0	AUSTRALIAN
CHADDERTON	874	FALSE	CSIRO	FALSE	131	1	CSIRO
CHAICHIAN, M	875	FALSE		FALSE		0	UNIV HELSINK
CHAIKINA, EI	876	FALSE		FALSE		0	CICESE
CHAKRAVORT	877	TRUE		FALSE		0	FERMILAB NA
CHAKRAVORT	878	FALSE	UNIV CALIF BI	FALSE	747	1	UNIV CALIF BI
CHAMPION, Y	881	FALSE		FALSE		0	UASLP
CHAN, A	882	TRUE	FERMILAB NA	FALSE	219	1	FERMILAB NA
CHAN, A	883	FALSE		FALSE		0	UNIV MICHIG
CHAN, Y	884	TRUE		FALSE		0	INST PHYS NU
CHAN, Y	885	FALSE	UNIV WARSA\	FALSE	1023	1	UNIV WARSA\
CHARVET, M	891	FALSE	UNIV LYON 1	FALSE	879	1	UNIV LYON 1
CHAUVET, PA	894	FALSE	UAM I	FALSE	686	1	UAM I
CHAVEZ, A	896	FALSE	ININ	FALSE	289	1	ININ
CHAVEZ, E	898	FALSE	UNIV SALERN	FALSE	972	1	UNIV SALERN

CHAVEZ-CERD	906	TRUE		FALSE		0 INAOE
CHAVEZ-CERD	907	FALSE	UNIV LONDON	FALSE	871	1 UNIV LONDON
CHAVEZ-LOM	908	FALSE	UNAM	FALSE	698	1 UNAM
CHAVEZ-PAEZ	909	TRUE	CTR RIC SVILU	FALSE	155	1 CTR RIC SVILU
CHAVEZ-PAEZ	910	FALSE		FALSE		0 UASLP
CHAVEZ-RIVA	911	FALSE	UAM A	FALSE	685	1 UAM A
CHAVIRA, E	912	FALSE		FALSE		0 UNAM
CHAVOYA-AC	913	FALSE	UAM A	FALSE	685	1 UAM A
CHAZOTTES, J	915	FALSE	CNRS	FALSE	103	1 CNRS
CHEANG-WOI	916	FALSE	UNAM	FALSE	698	1 UNAM
CHEN, X	923	TRUE	STEVENS INST	FALSE	633	1 STEVENS INST
CHEN, X	924	TRUE		FALSE		0 UNAM
CHEN, X	925	FALSE	UNIV CONNE(	FALSE	776	1 UNIV CONNE(
CHEN, YC	926	FALSE	UNIV GUANA.	FALSE	823	1 UNIV GUANA.
CHIBISOV, MI	930	TRUE	KURCHATOV I	FALSE	417	1 KURCHATOV I
CHIBISOV, MI	931	FALSE		FALSE		0 RUSSIAN RES
CHOONG, WS	935	TRUE	UNIV LAUSAN	FALSE	861	1 UNIV LAUSAN
CHOONG, WS	936	FALSE		FALSE		0 UNIV VIRGINI
CHRISTIAN, D	937	FALSE	UNIV GUANA.	FALSE	823	1 UNIV GUANA.
CHRISTIAN, D	938	FALSE		FALSE		0 UNIV MASSA(
CHROMIK, S	939	TRUE	SAS	FALSE	599	1 SAS
CHROMIK, S	940	FALSE		FALSE		0 SLOVAK ACAD
CHUBYKALO, .	943	FALSE		FALSE		0 UAZ
CHUMAKOV, :	944	TRUE	INAOE	FALSE	276	1 INAOE
CHUMAKOV, :	945	TRUE		TRUE		0 TECH UNIV BE
CHUMAKOV, :	946	FALSE		FALSE		0 UNAM
CHURCH, MD	948	TRUE		FALSE		0 TEXAS A&M U
CHURCH, MD	949	FALSE	UNIV GUANA.	FALSE	823	1 UNIV GUANA.
CILIBERTO, S	951	FALSE	UNIV FLOREN	FALSE	810	1 UNIV FLOREN
CINAUSERO, I	953	TRUE		FALSE		0 TEXAS A&M U
CINAUSERO, I	954	FALSE	UNIV PAVODA	FALSE	944	1 UNIV PAVODA
CLARK, A	960	FALSE	UNIV SONORA	FALSE	987	1 UNIV SONORA
CLEM, JR	964	FALSE	IOWA STATE U	FALSE	372	1 IOWA STATE U
COCHO, G	967	FALSE		FALSE		0 UNAM
COCOLETZI, G	968	FALSE	BUAP	FALSE	51	1 BUAP
COLAS, R	971	FALSE		FALSE		0 UNAM
COLE, AJ	972	TRUE	INST NATL PH	FALSE	328	1 INST NATL PH
COLE, AJ	973	FALSE		FALSE		0 UNAM
COLIN, LSG	974	FALSE	COLEGIO NAC	FALSE	108	1 COLEGIO NAC
COLLINS, KE	975	FALSE	UNIV ESTADU	FALSE	792	1 UNIV ESTADU
COMAS, F	978	FALSE	UNIV VALENC	FALSE	1010	1 UNIV VALENC
CONDE-GALLA	981	FALSE		FALSE		0 CINVESTAV
CONRADSON,	982	FALSE	DUPONT CO I	FALSE	178	1 DUPONT CO I
CONSTANTIN(	983	FALSE		FALSE		0 UASLP
CONTRERAS, .	984	FALSE	CINVESTAV	FALSE	94	1 CINVESTAV
CONTRERAS, (	985	TRUE		FALSE		0 CICESE
CONTRERAS, (	986	FALSE	HUNGARIAN /	FALSE	260	1 HUNGARIAN /

CONTRERAS-(	987	FALSE	UNIV MICHOA	FALSE	888	1	UNIV MICHOA
CONTRERASP	988	FALSE	UNAM	FALSE	698	1	UNAM
CONTRERAS-S	989	FALSE	UASLP	FALSE	689	1	UASLP
COOK, DC	990	FALSE		FALSE		0	OLD DOMINIC
COOLEN, ACC	991	FALSE	UNIV OXFORD	FALSE	933	1	UNIV OXFORD
CORDERO, R	994	FALSE	BUAP	FALSE	51	1	BUAP
CORDERO-BO	995	FALSE	UNAM	FALSE	698	1	UNAM
CORDOVA, JA	999	TRUE	IMP	FALSE	272	1	IMP
CORDOVA, JA	1000	FALSE		FALSE		0	UNAM
CORELLA-MAI	1001	FALSE		FALSE		0	UNIV SONORA
CORICHI, A	1003	TRUE		TRUE		0	PENN STATE U
CORICHI, A	1004	TRUE		TRUE		0	UNAM
CORICHI, A	1005	FALSE		FALSE		0	UNIV CALIF RI
CORONA, GC	1010	FALSE		FALSE		0	UAM A
CORONAGALI	1012	FALSE	INAOE	FALSE	276	1	INAOE
CORREA, G	1014	FALSE	UNAM	FALSE	698	1	UNAM
CORTES, E	1017	TRUE	UAM I	FALSE	686	1	UAM I
CORTES, E	1018	FALSE		FALSE		0	UNAM
COTA, E	1027	FALSE	UNAM	FALSE	698	1	UNAM
COTA-ARAIZA	1029	FALSE	UNAM	FALSE	698	1	UNAM
COTTI, U	1031	TRUE	BUAP	FALSE	51	1	BUAP
COTTI, U	1032	TRUE	CINVESTAV	FALSE	94	1	CINVESTAV
COTTI, U	1033	TRUE		TRUE		0	ISAS
COTTI, U	1034	FALSE		FALSE		0	SISSA
COYOTECATL,	1038	TRUE	BUAP	FALSE	51	1	BUAP
COYOTECATL,	1039	FALSE		FALSE		0	UNAM
CRESPO, A	1041	FALSE	UNIV PARIS 7	FALSE	941	1	UNIV PARIS 7
CRISCUOLO, A	1042	FALSE		FALSE		0	UNAM
CRUZ, A	1046	FALSE	UAM I	FALSE	686	1	UAM I
CRUZ, E	1047	FALSE	UNAM	FALSE	698	1	UNAM
CRUZ, F	1048	TRUE		FALSE		0	CINVESTAV
CRUZ, F	1049	TRUE	ININ	FALSE	289	1	ININ
CRUZ, F	1050	TRUE		TRUE		0	UNIV HAVANA
CRUZ, F	1051	FALSE		FALSE		0	UNIV LA HABA
CRUZ, GJ	1052	FALSE	UAM I	FALSE	686	1	UAM I
CRUZ, JLD	1055	FALSE		FALSE		0	CINVESTAV
CRUZ, M	1057	FALSE	UNAM	FALSE	698	1	UNAM
CRUZ, MD	1058	TRUE	CICESE	FALSE	89	1	CICESE
CRUZ, MD	1059	FALSE		FALSE		0	UNIV LA HABA
CRUZ-OREA, A	1062	FALSE	IPN	FALSE	376	1	IPN
CRUZ-PACHE(	1063	TRUE	UNAM	FALSE	698	1	UNAM
CRUZ-PACHE(	1064	FALSE		FALSE		0	UNIV EDINBU
CRUZ-REYES, .	1065	FALSE	UNAM	FALSE	698	1	UNAM
CRUZVAZQUE	1066	FALSE	UNIV ARIZON	FALSE	715	1	UNIV ARIZON
CSEH, J	1067	FALSE	HUNGARIAN A	FALSE	260	1	HUNGARIAN A
CUAUTLE, E	1069	FALSE	CINVESTAV	FALSE	94	1	CINVESTAV
CUAUTLE, MP	1070	FALSE		FALSE		0	INST INVEST E

CUAUTLI, LCC	1071	TRUE	BUAP	FALSE	51	1	BUAP
CUAUTLI, LCC	1072	FALSE		FALSE		0	CINVESTAV
CUDNEY, RS	1075	TRUE		FALSE		0	CICESE
CUDNEY, RS	1076	FALSE	ETH ZURICH	FALSE	205	1	ETH ZURICH
CUEVAS, FJ	1078	TRUE		FALSE		0	CIMAT
CUEVAS, FJ	1079	TRUE	CTR INVEST O	FALSE	148	1	CTR INVEST O
CUEVAS, FJ	1080	FALSE		FALSE		0	UNIV COMPLU
CUEVAS, S	1081	TRUE		TRUE		0	KITAMI INST T
CUEVAS, S	1082	TRUE	UNAM	FALSE	698	1	UNAM
CUEVAS, S	1083	FALSE		FALSE		0	UNIV AUTONO
CUSSO, F	1084	TRUE	CTR INVEST EI	FALSE	146	1	CTR INVEST EI
CUSSO, F	1085	FALSE		FALSE		0	UNIV SONORA
CYWIAK, M	1086	FALSE		FALSE		0	CTR INVEST O
CZANK, M	1087	TRUE		TRUE		0	UNIV COLIMA
CZANK, M	1088	FALSE	UNIV GUADAI	FALSE	822	1	UNIV GUADAI
DAGDUG, L	1093	TRUE		TRUE		0	UAM I
DAGDUG, L	1094	FALSE		FALSE		0	UNAM
DAMZEN, MJ	1102	TRUE		FALSE		0	CTR INVEST O
DAMZEN, MJ	1103	TRUE	INAOE	FALSE	276	1	INAOE
DAMZEN, MJ	1104	FALSE	UNIV LONDOI	FALSE	871	1	UNIV LONDOI
DARDEN, SE	1105	TRUE		FALSE		0	ININ
DARDEN, SE	1106	FALSE	UNIV NOTRE I	FALSE	923	1	UNIV NOTRE I
DASILVA, EG	1108	FALSE		FALSE		0	UNIV FED MIN
DATARS, WR	1109	FALSE		FALSE		0	NATL RES COU
DAVALOS-ORO	1112	FALSE	UNAM	FALSE	698	1	UNAM
DAVIDSON, N	1113	FALSE		FALSE		0	UNAM
DAVILA, A	1114	FALSE	CTR INVEST O	FALSE	148	1	CTR INVEST O
DAVILA, AC	1115	FALSE	BUAP	FALSE	51	1	BUAP
DAVILA-JIMEN	1117	FALSE	FAC CHEM BU	FALSE	208	1	FAC CHEM BU
DEANDA-SALA	1123	FALSE		FALSE		0	UASLP
DECARCER, IA	1126	FALSE	UNIV SONORA	FALSE	987	1	UNIV SONORA
DECOSS, R	1127	FALSE	IPN	FALSE	376	1	IPN
DEDIEGO, DM	1128	TRUE	UNIV FED RIO	FALSE	805	1	UNIV FED RIO
DEDIEGO, DM	1129	FALSE		FALSE		0	UNIV LA LAGU
DEITA, A	1137	TRUE		FALSE		0	IPN
DEITA, A	1138	FALSE	UAM A	FALSE	685	1	UAM A
DELACRUZ, AI	1139	FALSE		FALSE		0	UNIV COMPLU
DELACRUZ, FA	1140	TRUE	BUAP	FALSE	51	1	BUAP
DELACRUZ, FA	1141	FALSE		FALSE		0	UNIV GUADAI
DELACRUZ, GO	1142	FALSE	CINVESTAV	FALSE	94	1	CINVESTAV
DELACRUZ-M	1143	FALSE	UASLP	FALSE	689	1	UASLP
DELAGARZA, I	1146	FALSE	CORNELL UNI	FALSE	124	1	CORNELL UNI
DELAMACORI	1148	TRUE		FALSE		0	MCGILL UNIV
DELAMACORI	1149	FALSE	UNAM	FALSE	698	1	UNAM
DELAMORA, F	1151	FALSE	UNAM	FALSE	698	1	UNAM
DELANGEL, P	1152	FALSE	BUAP	FALSE	51	1	BUAP
DELAROSA, E	1157	FALSE	CTR INVEST O	FALSE	148	1	CTR INVEST O

DELAROSACRU	1159	FALSE		FALSE		0	CTR INVEST O
DELCASTILLO,	1166	FALSE	UNAM	FALSE	698	1	UNAM
DELCASTILLO-	1167	TRUE	CINVESTAV	FALSE	94	1	CINVESTAV
DELCASTILLO-	1168	FALSE		FALSE		0	UNAM
DELEON, GC	1171	FALSE		FALSE		0	UASLP
DELEON, M	1173	FALSE	CSIC	FALSE	130	1	CSIC
DELGADILLO,	1174	TRUE		TRUE		0	CINVESTAV
DELGADILLO,	1175	TRUE		FALSE		0	INST NACL PE
DELGADILLO,	1176	TRUE	IPN	FALSE	376	1	IPN
DELGADILLO,	1177	FALSE	UASLP	FALSE	689	1	UASLP
DELGADO, F	1179	TRUE	CINVESTAV	FALSE	94	1	CINVESTAV
DELGADO, F	1180	FALSE	ITESM	FALSE	385	1	ITESM
DELGADO, J	1181	FALSE	UAM I	FALSE	686	1	UAM I
DELIRA-HUES	1182	FALSE		FALSE		0	UNAM
DELREAL, A	1189	FALSE	UAM I	FALSE	686	1	UAM I
DELRIO, F	1190	TRUE		FALSE		0	UAM I
DELRIO, F	1191	FALSE	UNIV COMPLU	FALSE	775	1	UNIV COMPLU
DELRIO, JA	1192	TRUE		FALSE		0	UNAM
DELRIO, JA	1193	FALSE	UNIV AUTONO	FALSE	722	1	UNIV AUTONO
DELRIO, R	1195	TRUE	CALTECH	FALSE	58	1	CALTECH
DELRIO, R	1196	FALSE		FALSE		0	UNAM
DELRIO-CORR	1197	TRUE	COLEGIO NAC	FALSE	108	1	COLEGIO NAC
DELRIO-CORR	1198	FALSE		FALSE		0	UAM I
DELSOLE, R	1199	FALSE	UNIV ROMA T	FALSE	963	1	UNIV ROMA T
DEMANGEAT,	1203	TRUE		FALSE		0	LAB PHYS SOL
DEMANGEAT,	1204	FALSE	UNIV VALLAD	FALSE	1011	1	UNIV VALLAD
DEMELO, O	1205	TRUE	CINVESTAV	FALSE	94	1	CINVESTAV
DEMELO, O	1206	FALSE		FALSE		0	UNIV LA HABA
DEOLIVEIRA, I	1211	FALSE	UNAM	FALSE	698	1	UNAM
DEPARGA, GA	1213	FALSE	IPN	FALSE	376	1	IPN
DEREVANKO,	1216	FALSE	KHARKOV AM	FALSE	405	1	KHARKOV AM
DESJONQUER	1223	TRUE	CENS	FALSE	73	1	CENS
DESJONQUER	1224	FALSE		FALSE		0	UASLP
DETERESA, C	1225	TRUE	Swiss Fed Inst	FALSE	642	1	Swiss Fed Inst
DETERESA, C	1226	FALSE	UNAM	FALSE	698	1	UNAM
DEVARGAS, L	1229	FALSE	IPN	FALSE	376	1	IPN
DEYOUNG, PA	1232	TRUE	ININ	FALSE	289	1	ININ
DEYOUNG, PA	1233	FALSE		FALSE		0	Univ Fernand
DIAMANT, R	1236	TRUE	CNRS	FALSE	103	1	CNRS
DIAMANT, R	1237	TRUE		TRUE		0	ININ
DIAMANT, R	1238	FALSE		FALSE		0	UAM I
DIAZ, A	1241	FALSE	UNAM	FALSE	698	1	UNAM
DIAZ, C	1243	FALSE	UNIV AUTONO	FALSE	718	1	UNIV AUTONO
DIAZ, P	1247	TRUE	UNIV HAVANA	FALSE	831	1	UNIV HAVANA
DIAZ, P	1248	FALSE		FALSE		0	UNIV LA HABA
DIAZ, S	1250	FALSE	UNAM	FALSE	698	1	UNAM
DIAZ, T	1251	TRUE	BUAP	FALSE	51	1	BUAP

DIAZ, T	1252	FALSE		FALSE		0	CINVESTAV
DIAZ-ARENCIE	1253	FALSE	UAM A	FALSE	685	1	UAM A
DIAZ-CRUZ, JL	1255	TRUE		TRUE		0	CINVESTAV
DIAZ-CRUZ, JL	1256	FALSE		FALSE		0	UNIV AUTONO
DIAZ-HERRER/	1258	FALSE	UAM I	FALSE	686	1	UAM I
DIAZ-ORTIZ, A	1259	FALSE	UASLP	FALSE	689	1	UASLP
DIAZ-REYES, J	1260	FALSE	CINVESTAV	FALSE	94	1	CINVESTAV
DIAZ-RIVERA,	1261	FALSE	UAM I	FALSE	686	1	UAM I
DIAZ-TORRES,	1262	TRUE		FALSE		0	CTR INVEST O
DIAZ-TORRES,	1263	FALSE	OSRAM SYLVA	FALSE	545	1	OSRAM SYLVA
DIAZ-URIBE, F	1264	TRUE	BUAP	FALSE	51	1	BUAP
DIAZ-URIBE, F	1265	FALSE		FALSE		0	INAOE
DIAZ-VALDES,	1266	FALSE	IPN	FALSE	376	1	IPN
DIETZ, B	1268	FALSE		FALSE		0	UNAM
DIRL, R	1277	FALSE	VIENNA TECH	FALSE	1050	1	VIENNA TECH
DITTES, FM	1278	FALSE	MAX PLANCK	FALSE	465	1	MAX PLANCK
DJAFARI-ROU	1279	TRUE	BUAP	FALSE	51	1	BUAP
DJAFARI-ROU	1280	FALSE		FALSE		0	UNIV LILLE 1
DOBRZYNSKI,	1282	FALSE	UNIV LILLE 1	FALSE	865	1	UNIV LILLE 1
DODONOV, V\	1283	FALSE	MOSCOW INS	FALSE	487	1	MOSCOW INS
DOMINGUEZ,	1285	TRUE		TRUE		0	LOS ALAMOS
DOMINGUEZ,	1286	FALSE		FALSE		0	UASLP
DOMINGUEZ,	1287	FALSE		FALSE		0	UNAM
DOMINGUEZ,	1288	FALSE	IMP	FALSE	272	1	IMP
DOMINGUEZ-	1292	FALSE		FALSE		0	UASLP
DOOLEN, GD	1293	TRUE		FALSE		0	LOS ALAMOS
DOOLEN, GD	1294	FALSE	LV KIRENSKII I	FALSE	452	1	LV KIRENSKII I
DORANTES-R(	1297	FALSE		FALSE		0	IPN
DORSO, CO	1298	TRUE		FALSE		0	UAM A
DORSO, CO	1299	FALSE	UNIV BUENOS	FALSE	745	1	UNIV BUENOS
DRAAYER, JP	1301	TRUE	LOUISIANA ST	FALSE	451	1	LOUISIANA ST
DRAAYER, JP	1302	FALSE		FALSE		0	UNAM
DRAGANIC, ZI	1306	FALSE	BORIS KIDRIC	FALSE	42	1	BORIS KIDRIC
DREYSSE, H	1309	TRUE		TRUE		0	IPCMS
DREYSSE, H	1310	TRUE		TRUE		0	LAB PHYS SOL
DREYSSE, H	1311	TRUE		FALSE		0	UASLP
DREYSSE, H	1312	FALSE		FALSE		0	UNIV NANCY
DUDA, Y	1320	TRUE		TRUE		0	ININ
DUDA, Y	1321	FALSE		FALSE		0	UNAM
DUFTY, JW	1325	FALSE	UNIV FLORIDA	FALSE	811	1	UNIV FLORIDA
DURDEVIC, M	1330	FALSE	UNAM	FALSE	698	1	UNAM
DURDEVICH, I	1331	FALSE		FALSE		0	UNAM
DVOEGLAZOV	1337	TRUE		FALSE		0	UAZ
DVOEGLAZOV	1338	FALSE	UNAM	FALSE	698	1	UNAM
EBNER, DW	1340	TRUE		FALSE		0	UNAM
EBNER, DW	1341	FALSE	UNIV KONSTA	FALSE	854	1	UNIV KONSTA
EFREMOV, VN	1346	FALSE		FALSE		0	UNIV GUADAI

ELIZALDE-GOI	1354	FALSE	BUAP	FALSE	51	1	BUAP
ELIZALDE-TOF	1355	FALSE	UNAM	FALSE	698	1	UNAM
ELLIOTT, RJ	1356	TRUE		FALSE		0	UNAM
ELLIOTT, RJ	1357	FALSE	UNIV OXFORI	FALSE	933	1	UNIV OXFORI
ELYUKHIN, VA	1360	FALSE	AF IOFFE PHYS	FALSE	10	1	AF IOFFE PHYS
ENCISO, A	1362	TRUE	UAZ	FALSE	690	1	UAZ
ENCISO, A	1363	FALSE		FALSE		0	UNAM
ERASME, D	1369	FALSE		FALSE		0	CICESE
ERNST, FJ	1370	FALSE		FALSE		0	CLARKSON UI
ESCALANTE, V	1371	FALSE		FALSE		0	UNAM
ESCALANTE-G	1372	TRUE		TRUE		0	CINVESTAV
ESCALANTE-G	1373	TRUE		TRUE		0	CTR INVEST M
ESCALANTE-G	1374	FALSE		FALSE		0	INST TECNOL
ESCALONA, J	1376	TRUE	UNAM	FALSE	698	1	UNAM
ESCALONA, J	1377	FALSE		FALSE		0	UNIV AUTON
ESCAMILLA, B	1378	FALSE	INAOE	FALSE	276	1	INAOE
ESCAMILLA, H	1379	TRUE	CICESE	FALSE	89	1	CICESE
ESCAMILLA, H	1380	FALSE		FALSE		0	UNIV CALIF IR
ESCAMILLA, R	1381	FALSE	UNAM	FALSE	698	1	UNAM
ESCAMILLA-ES	1382	TRUE	CINVESTAV	FALSE	94	1	CINVESTAV
ESCAMILLA-ES	1383	FALSE		FALSE		0	IPN
ESCOBAR-ALA	1387	TRUE	ININ	FALSE	289	1	ININ
ESCOBAR-ALA	1388	FALSE		FALSE		0	UAM I
ESCUDERO, R	1390	FALSE	UNAM	FALSE	698	1	UNAM
ESPARZA, A	1392	FALSE	CINVESTAV	FALSE	94	1	CINVESTAV
ESPINOSA, F	1397	FALSE	ITESM	FALSE	385	1	ITESM
ESPINOSA, FJ	1398	FALSE		FALSE		0	CINVESTAV
ESPINOSA, G	1399	FALSE	UNAM	FALSE	698	1	UNAM
ESPINOZA, PB	1402	FALSE		FALSE		0	UNIV GUANA
ESPINOZA-BEI	1403	TRUE		FALSE		0	CINVESTAV
ESPINOZA-BEI	1404	FALSE	UNIV SONOR	FALSE	987	1	UNIV SONOR
ESPOSITO, G	1405	TRUE	ISAS	FALSE	379	1	ISAS
ESPOSITO, G	1406	FALSE		FALSE		0	IST NAZL FIS N
ESQUIVEL, RO	1408	TRUE	UAM A	FALSE	685	1	UAM A
ESQUIVEL, RO	1409	FALSE		FALSE		0	UAM I
ESQUIVEL-SIR	1410	FALSE	UNAM	FALSE	698	1	UNAM
ESTRADA, CA	1416	FALSE	TEXAS A&M U	FALSE	663	1	TEXAS A&M U
ESTRADA, M	1419	FALSE		FALSE		0	CINVESTAV
ESTRADA, W	1420	TRUE	CTR INVEST M	FALSE	147	1	CTR INVEST M
ESTRADA, W	1421	FALSE		FALSE		0	UNAM
ESTRADAGAS	1422	FALSE		FALSE		0	UNAM
FABENI, P	1427	FALSE	CNR	FALSE	101	1	CNR
FABRIS, D	1428	TRUE		TRUE		0	IST NAZL FIS N
FABRIS, D	1429	FALSE		FALSE		0	UNIV PADUA
FACCIO, RJ	1430	FALSE	UNIV NACL SA	FALSE	911	1	UNIV NACL SA
FAHRNER, WF	1431	FALSE	UAM I	FALSE	686	1	UAM I
FALCON, DAL	1434	FALSE		FALSE		0	BUAP

FALCONY, C	1435	FALSE	CINVESTAV	FALSE	94	1	CINVESTAV
FARIAS, MH	1440	TRUE	CINVESTAV	FALSE	94	1	CINVESTAV
FARIAS, MH	1441	FALSE		FALSE		0	UNAM
FELIPE, RG	1447	FALSE	UNIV MANITC	FALSE	882	1	UNIV MANITC
FELIX, J	1449	TRUE		TRUE		0	COLUMBIA UI
FELIX, J	1450	TRUE		FALSE		0	Fermilab
FELIX, J	1451	FALSE	UNIV GUANA.	FALSE	823	1	UNIV GUANA.
FEMAT, R	1453	TRUE		TRUE		0	UAM I
FEMAT, R	1454	FALSE		FALSE		0	UASLP
FENG, DH	1457	TRUE	DROPSIE UNI\	FALSE	175	1	DROPSIE UNI\
FENG, DH	1458	FALSE		FALSE		0	NATL SCI FDN
FERNANDEZ, /	1461	TRUE		TRUE		0	BUAP
FERNANDEZ, /	1462	FALSE		FALSE		0	CINVESTAV
FERNANDEZ, /	1463	FALSE		FALSE		0	UNAM
FERNANDEZ, I	1464	FALSE	CINVESTAV	FALSE	94	1	CINVESTAV
FERNANDEZ, I	1465	TRUE	IPN	FALSE	376	1	IPN
FERNANDEZ, I	1466	FALSE		FALSE		0	UNIV SALAM/
FERNANDEZ, I	1468	TRUE	ININ	FALSE	289	1	ININ
FERNANDEZ, I	1469	FALSE		FALSE		0	UNAM
FERNANDEZ, I	1470	FALSE	UNIV COMPLU	FALSE	775	1	UNIV COMPLU
FERNANDEZ-(	1475	TRUE		FALSE		0	CINVESTAV
FERNANDEZ-(	1476	FALSE	UAM I	FALSE	686	1	UAM I
FERNANDEZV	1477	FALSE	ININ	FALSE	289	1	ININ
FETTER, G	1482	FALSE	UAM I	FALSE	686	1	UAM I
FINK, D	1485	FALSE	HAHN MEITNI	FALSE	242	1	HAHN MEITNI
FIONOVA, L	1488	TRUE	CHERNOGOL(	FALSE	83	1	CHERNOGOL(
FIONOVA, L	1489	FALSE	UNAM	FALSE	698	1	UNAM
FIORETTO, E	1490	TRUE		FALSE		0	HOPE COLL
FIORETTO, E	1491	FALSE	IST NAZL FIS N	FALSE	383	1	IST NAZL FIS N
FISHER, LM	1497	TRUE		FALSE		0	ALL RUSSIAN
FISHER, LM	1498	FALSE	ALL RUSSIAN	FALSE	17	1	ALL RUSSIAN
FLEMMING, N	1500	TRUE		FALSE		0	MAX PLANCK
FLEMMING, N	1501	FALSE	UNAM	FALSE	698	1	UNAM
FLORES, A	1503	FALSE	CINVESTAV	FALSE	94	1	CINVESTAV
FLORES, C	1505	TRUE	UNAM	FALSE	698	1	UNAM
FLORES, C	1506	TRUE	UNIV AUTON(	FALSE	727	1	UNIV AUTON(
FLORES, C	1507	FALSE		FALSE		0	UNIV GRENOI
FLORES, GA	1508	FALSE	CALIF STATE U	FALSE	56	1	CALIF STATE U
FLORES, M	1514	FALSE	UNAM	FALSE	698	1	UNAM
FLORES, MB	1515	TRUE		FALSE		0	ESTONIAN AC
FLORES, MB	1516	FALSE	UNIV SONOR/	FALSE	987	1	UNIV SONOR/
FLORES, O	1519	FALSE	UNAM	FALSE	698	1	UNAM
FLORESLLAM/	1524	TRUE		FALSE		0	ININ
FLORESLLAM/	1525	FALSE	UNAM	FALSE	698	1	UNAM
FLORES-MEN/	1526	FALSE	BUAP	FALSE	51	1	BUAP
FLORES-MENI	1527	FALSE		FALSE		0	CINVESTAV
FLORESRAMIF	1528	FALSE	UNAM	FALSE	698	1	UNAM

FLORES-RIVEF	1530	FALSE	UAM I	FALSE	686	1	UAM I
FLORESZUNIG	1533	FALSE	UNAM	FALSE	698	1	UNAM
FOLDVARI, I	1535	FALSE	HUNGARIAN /	FALSE	260	1	HUNGARIAN /
FONT, R	1537	FALSE	UNIV LA HAB/	FALSE	855	1	UNIV LA HAB/
FORBUSH, M	1540	FALSE	UNIV GUANA.	FALSE	823	1	UNIV GUANA.
FREEMAN, RM	1551	FALSE		FALSE		0	UNIV STRASB(
FRIPIAT, JJ	1560	TRUE	IPN	FALSE	376	1	IPN
FRIPIAT, JJ	1561	FALSE	UNIV WISCON	FALSE	1027	1	UNIV WISCON
FUCHS, OL	1564	FALSE	BUAP	FALSE	51	1	BUAP
FUENTES, AF	1567	FALSE		FALSE		0	UANL
FUENTES, OU'	1569	TRUE	CICESE	FALSE	89	1	CICESE
FUENTES, OU'	1570	FALSE		FALSE		0	UNIV CALIF S/
FUENTES, R	1571	TRUE		FALSE		0	HYLSA SA CV
FUENTES, R	1572	FALSE	UNAM	FALSE	698	1	UNAM
FUENTES, S	1573	FALSE	UNIV CALIF S/	FALSE	752	1	UNIV CALIF S/
FUENTES, V	1574	FALSE	UNAM	FALSE	698	1	UNAM
FUJIOKA, J	1578	FALSE	UNAM	FALSE	698	1	UNAM
FUJITA, S	1579	TRUE		TRUE		0	SUNY BUFFAL
FUJITA, S	1580	FALSE	UNAM	FALSE	698	1	UNAM
FUNDORA, A	1581	TRUE		FALSE		0	CICESE
FUNDORA, A	1582	TRUE		TRUE		0	UNAM
FUNDORA, A	1583	TRUE		TRUE		0	UNIV HAVAN/
FUNDORA, A	1584	TRUE		TRUE		0	UNIV LA HAB/
FUNDORA, A	1585	FALSE		FALSE		0	UNIV ORIENTI
FUNES-GALLA	1586	FALSE	INAOE	FALSE	276	1	INAOE
GAFTOI, V	1590	FALSE	UAM A	FALSE	685	1	UAM A
GAGGERO-SA	1591	TRUE	CTR INVEST O	FALSE	148	1	CTR INVEST O
GAGGERO-SA	1592	TRUE	UAZ	FALSE	690	1	UAZ
GAGGERO-SA	1593	FALSE		FALSE		0	UNIV AUTON(
GAITAN, R	1597	FALSE	CINVESTAV	FALSE	94	1	CINVESTAV
GALEANA, AH	1600	FALSE	CINVESTAV	FALSE	94	1	CINVESTAV
GALEENER, FL	1601	FALSE	XEROX CORP	FALSE	1063	1	XEROX CORP
GALLIGAN, JM	1605	FALSE	UNIV CONNE(	FALSE	776	1	UNIV CONNE(
GALTSOV, DV	1608	FALSE	MOSCOW MV	FALSE	488	1	MOSCOW MV
GALVAN, DH	1609	FALSE	UNAM	FALSE	698	1	UNAM
GALVAN, M	1610	FALSE	MIT	FALSE	480	1	MIT
GAMALY, EG	1612	TRUE		FALSE		0	AUSTRALIAN I
GAMALY, EG	1613	FALSE	UAM I	FALSE	686	1	UAM I
GAMBAR, K	1614	FALSE	TECH UNIV BU	FALSE	649	1	TECH UNIV BU
GAMBINI, R	1615	TRUE	UNIV GUANA.	FALSE	823	1	UNIV GUANA.
GAMBINI, R	1616	FALSE		FALSE		0	UNIV LA REPU
GAMBOA, SA	1618	TRUE	NATL RENEW/	FALSE	510	1	NATL RENEW/
GAMBOA, SA	1619	FALSE	UNAM	FALSE	698	1	UNAM
GAMEZ-CORR	1621	TRUE	LD LANDAU TI	FALSE	437	1	LD LANDAU TI
GAMEZ-CORR	1622	FALSE		FALSE		0	UASLP
GAMMAGE, R	1624	TRUE	OAK RIDGE N/	FALSE	533	1	OAK RIDGE N/
GAMMAGE, R	1625	FALSE		FALSE		0	UNAM

GANDOLFINI,	1626	FALSE	UNIV OTTAW.	FALSE	930	1	UNIV OTTAW.
GAONA-COUT	1627	FALSE		FALSE		0	UNAM
GARA, A	1628	FALSE	COLUMBIA UI	FALSE	112	1	COLUMBIA UI
GARCIA, AA	1631	FALSE		FALSE		0	CINVESTAV
GARCIA, AE	1634	FALSE		FALSE		0	CINVESTAV
GARCIA, BB	1636	FALSE	CTR INVEST O	FALSE	148	1	CTR INVEST O
GARCIA, CM	1637	FALSE	IPN	FALSE	376	1	IPN
GARCIA, DA	1638	FALSE		FALSE		0	CINVESTAV
GARCIA, I	1639	FALSE	ININ	FALSE	289	1	ININ
GARCIA, JA	1641	FALSE	UNAM	FALSE	698	1	UNAM
GARCIA, KL	1643	FALSE	UNAM	FALSE	698	1	UNAM
GARCIA, M	1644	TRUE	CINVESTAV	FALSE	94	1	CINVESTAV
GARCIA, M	1645	FALSE	CTR BRASILEII	FALSE	135	1	CTR BRASILEII
GARCIA, S	1649	FALSE	UNIV LA HAB/	FALSE	855	1	UNIV LA HAB/
GARCIA, T	1650	FALSE	UNIV LA HAB/	FALSE	855	1	UNIV LA HAB/
GARCIA, VG	1651	FALSE		FALSE		0	IPN
GARCIA, VM	1652	FALSE	UNAM	FALSE	698	1	UNAM
GARCIA-ALVA	1655	TRUE	UANL	FALSE	687	1	UANL
GARCIA-ALVA	1656	FALSE		FALSE		0	UNIV SAN PAI
GARCIA-BORC	1657	FALSE	IPN	FALSE	376	1	IPN
GARCIA-CERD	1661	TRUE	CINVESTAV	FALSE	94	1	CINVESTAV
GARCIA-CERD	1662	TRUE		FALSE		0	INST TECNOL
GARCIA-CERD	1663	TRUE	UAQ	FALSE	688	1	UAQ
GARCIA-CERD	1664	FALSE		FALSE		0	UNIV SONOR/
GARCIA-COM	1669	FALSE		FALSE		0	CINVESTAV
GARCIA-LLAN	1675	TRUE	CICESE	FALSE	89	1	CICESE
GARCIA-LLAN	1676	FALSE	UNIV AUTON(	FALSE	728	1	UNIV AUTON(
GARCIA-MAR(	1677	FALSE	CTR INVEST O	FALSE	148	1	CTR INVEST O
GARCIA-MEN	1678	FALSE		FALSE		0	UNIV NACL AI
GARCIAMOLII	1679	TRUE	UNAM	FALSE	698	1	UNAM
GARCIAMOLII	1680	FALSE		FALSE		0	UNIV JAUME
GARCIAPELAY	1681	FALSE	UNAM	FALSE	698	1	UNAM
GARCIA-ROCH	1684	TRUE	CINVESTAV	FALSE	94	1	CINVESTAV
GARCIA-ROCH	1685	FALSE	UNIV LA HAB/	FALSE	855	1	UNIV LA HAB/
GARCIA-RODI	1686	FALSE	UAQ	FALSE	688	1	UAQ
GARCIA-RUIZ,	1687	FALSE	UNAM	FALSE	698	1	UNAM
GARCIA-SANC	1688	FALSE		FALSE		0	IMP
GARCIA-SANT	1690	FALSE	ININ	FALSE	289	1	ININ
GARCIA-SEGU	1691	FALSE	UNAM	FALSE	698	1	UNAM
GARCIA-SERR.	1692	TRUE	BUAP	FALSE	51	1	BUAP
GARCIA-SERR.	1693	FALSE		FALSE		0	UAEH
GARCIA-TELL(	1697	TRUE		FALSE		0	IEHU
GARCIA-TELL(	1698	FALSE	UPV EHU	FALSE	1040	1	UPV EHU
GARCIA-VALE	1699	TRUE		FALSE		0	INAOE
GARCIA-VALE	1700	FALSE	UNAM	FALSE	698	1	UNAM
GARCIA-WEID	1701	FALSE	CICESE	FALSE	89	1	CICESE
GARCIAZENTE	1703	FALSE	UNAM	FALSE	698	1	UNAM

GARRIDO, AA	1711	FALSE	JOINT INST NU	FALSE	395	1	JOINT INST NU
GARZA, J	1714	FALSE	UAM I	FALSE	686	1	UAM I
GARZA-HUME	1716	FALSE	UNAM	FALSE	698	1	UNAM
GARZO, V	1717	TRUE	UNAM	FALSE	698	1	UNAM
GARZO, V	1718	FALSE		FALSE		0	UNIV EXTREM
GARZON, IL	1719	FALSE	UNAM	FALSE	698	1	UNAM
GASPARIAN, V	1725	FALSE		FALSE		0	UNIV GUANA
GAZQUEZ, JL	1729	FALSE		FALSE		0	UAM I
GEFFROY, E	1730	FALSE		FALSE		0	UNAM
GEORGE, PJ	1734	FALSE		FALSE		0	UNAM
GEORGIEV, M	1735	FALSE	UNAM	FALSE	698	1	UNAM
GERMAN, G	1737	FALSE	UNAM	FALSE	698	1	UNAM
GIAMBIAGI, J.	1740	TRUE		FALSE		0	CTR BRASILEI
GIAMBIAGI, J.	1741	FALSE	NATL UNIV LA	FALSE	519	1	NATL UNIV LA
GILES-GUZMA	1746	FALSE	CICESE	FALSE	89	1	CICESE
GILMORE, R	1747	FALSE		FALSE		0	UNAM
GIL-VILLEGAS,	1748	FALSE	UAM I	FALSE	686	1	UAM I
GIRALDO, J	1750	FALSE	NATL UNIV CO	FALSE	516	1	NATL UNIV CO
GLEASON, RJ	1756	FALSE		FALSE		0	UNAM
GODINA, JJ	1759	FALSE	CINVESTAV	FALSE	94	1	CINVESTAV
GODOY, SV	1762	TRUE	UAM I	FALSE	686	1	UAM I
GODOY, SV	1763	FALSE		FALSE		0	UNAM
GOITIA, ARBY	1765	TRUE		FALSE		0	CTR INVEST O
GOITIA, ARBY	1766	FALSE	POLAROID CO	FALSE	558	1	POLAROID CO
GOLDSTEIN, P	1767	FALSE	UNAM	FALSE	698	1	UNAM
GOLZARRI, JI	1768	TRUE	BRIGHAM YOU	FALSE	46	1	BRIGHAM YOU
GOLZARRI, JI	1769	FALSE		FALSE		0	UNAM
GOMEZ, CM	1773	FALSE	INAOE	FALSE	276	1	INAOE
GOMEZ, E	1774	FALSE	BUAP	FALSE	51	1	BUAP
GOMEZ, JP	1776	TRUE	ININ	FALSE	289	1	ININ
GOMEZ, JP	1777	FALSE	IPN	FALSE	376	1	IPN
GOMEZ, R	1780	TRUE	NIHON UNIV	FALSE	525	1	NIHON UNIV
GOMEZ, R	1781	FALSE	UNAM	FALSE	698	1	UNAM
GOMEZ-DAZA	1786	FALSE		FALSE		0	UNAM
GONCALVES,	1793	FALSE	UNAM	FALSE	698	1	UNAM
GONCHAR, VY	1794	FALSE		FALSE		0	KHARKOV PHY
GONGORA, A	1795	FALSE	UNAM	FALSE	698	1	UNAM
GONIN, M	1796	FALSE	IST NAZL FIS N	FALSE	383	1	IST NAZL FIS N
GONSALVES, I	1797	TRUE		FALSE		0	ININ
GONSALVES, I	1798	FALSE	UNIV CONNEC	FALSE	776	1	UNIV CONNEC
GONZALEZ, A	1799	FALSE		FALSE		0	UNAM
GONZALEZ, E	1802	FALSE	BUAP	FALSE	51	1	BUAP
GONZALEZ, G	1803	FALSE	UAM A	FALSE	685	1	UAM A
GONZALEZ, G	1804	FALSE	UNIV SONORA	FALSE	987	1	UNIV SONORA
GONZALEZ, H	1805	TRUE		TRUE		0	CINVESTAV
GONZALEZ, H	1806	FALSE		FALSE		0	UNIV SURCOL
GONZALEZ, I	1807	TRUE	UAM I	FALSE	686	1	UAM I

GONZALEZ, I	1808	FALSE	UNAM	FALSE	698	1 UNAM
GONZALEZ, J	1809	FALSE	UNIV PAIS VA	FALSE	936	1 UNIV PAIS VA
GONZALEZ, JC	1810	FALSE	CINVESTAV	FALSE	94	1 CINVESTAV
GONZALEZ, LA	1813	TRUE	BUAP	FALSE	51	1 BUAP
GONZALEZ, LA	1814	FALSE		FALSE		0 INAOE
GONZALEZ, M	1816	FALSE	UNAM	FALSE	698	1 UNAM
GONZALEZ, R	1820	FALSE	UNIV PANAM	FALSE	938	1 UNIV PANAM
GONZALEZ, RV	1822	FALSE	UNAM	FALSE	698	1 UNAM
GONZALEZ-HE	1827	TRUE		TRUE		0 BUAP
GONZALEZ-HE	1828	TRUE	CINVESTAV	FALSE	94	1 CINVESTAV
GONZALEZ-HE	1829	FALSE		FALSE		0 UNAM
GONZALEZ-M	1831	TRUE		FALSE		0 ININ
GONZALEZ-M	1832	FALSE	UAQ	FALSE	688	1 UAQ
GONZALEZ-M	1833	TRUE		TRUE		0 CICESE
GONZALEZ-M	1834	FALSE		FALSE		0 UNAM
GONZALEZ-M	1835	FALSE		FALSE		0 CINVESTAV
GONZALEZ-RC	1837	FALSE	UNIV AUTONC	FALSE	725	1 UNIV AUTONC
GONZALEZTO	1838	FALSE		FALSE		0 ININ
GONZALEZ-TC	1839	TRUE		FALSE		0 IMP
GONZALEZ-TC	1840	FALSE	UAM I	FALSE	686	1 UAM I
GONZALO, C	1844	FALSE	CSIC	FALSE	130	1 CSIC
GOODWIN, FE	1845	FALSE		FALSE		0 INT LEAD & ZI
GOPAR, VA	1846	FALSE		FALSE		0 UNAM
GORBATCHEV	1848	TRUE		FALSE		0 AF IOFFE PHYS
GORBATCHEV	1849	FALSE	UASLP	FALSE	689	1 UASLP
GOREV, NB	1850	TRUE		FALSE		0 CINVESTAV
GOREV, NB	1851	TRUE	INST TECH ME	FALSE	354	1 INST TECH ME
GOREV, NB	1852	TRUE		FALSE		0 NATL ACAD SC
GOREV, NB	1853	FALSE	UAQ	FALSE	688	1 UAQ
GORIN, T	1854	TRUE	TECH UNIV DI	FALSE	652	1 TECH UNIV DI
GORIN, T	1855	FALSE		FALSE		0 UNAM
GOTTSCHALK,	1858	FALSE	TEXAS A&M U	FALSE	663	1 TEXAS A&M U
GRACIA, FF	1861	FALSE	BUAP	FALSE	51	1 BUAP
GRACIA-JIMEI	1863	FALSE		FALSE		0 BUAP
GRANADOS, N	1867	FALSE	UAZ	FALSE	690	1 UAZ
GRANADOS, V	1868	TRUE		FALSE		0 IPN
GRANADOS, V	1869	FALSE	UAM I	FALSE	686	1 UAM I
GREBERT, B	1874	FALSE	UNAM	FALSE	698	1 UNAM
GREINER, W	1877	FALSE	UNAM	FALSE	698	1 UNAM
GRETHER, M	1878	FALSE	UNAM	FALSE	698	1 UNAM
GRIJALVA, H	1881	FALSE	NAGOYA UNIV	FALSE	491	1 NAGOYA UNIV
GRIMALSKY, V	1882	TRUE		FALSE		0 Natl Kiev Univ
GRIMALSKY, V	1883	FALSE	UNAM	FALSE	698	1 UNAM
GROBERT, N	1890	TRUE		FALSE		0 UNAM
GROBERT, N	1891	TRUE	UNIV CALIF SA	FALSE	753	1 UNIV CALIF SA
GROBERT, N	1892	FALSE		FALSE		0 UNIV SUSSEX
GROSS, CJ	1894	TRUE		FALSE		0 UNAM

GROSS, CJ	1895	FALSE	UNIV TENNES	FALSE	996	1 UNIV TENNES
GU, ZH	1899	TRUE		TRUE		0 CICESE
GU, ZH	1900	FALSE		FALSE		0 SURFACE OPT
GUARDIOLA, I	1902	FALSE	UNAM	FALSE	698	1 UNAM
GUASTI, MF	1903	TRUE		TRUE		0 CICESE
GUASTI, MF	1904	TRUE		FALSE		0 UAM A
GUASTI, MF	1905	FALSE	UNIV PARIS 6	FALSE	940	1 UNIV PARIS 6
GUDINO-MAF	1906	TRUE	CINVESTAV	FALSE	94	1 CINVESTAV
GUDINO-MAF	1907	FALSE	UASLP	FALSE	689	1 UASLP
GUEKOS, G	1908	FALSE	ECOLE NATL S	FALSE	186	1 ECOLE NATL S
GUEL-SANDO'	1909	FALSE		FALSE		0 UASLP
GUERRERO, F	1913	TRUE		TRUE		0 UNAM
GUERRERO, F	1914	TRUE		TRUE		0 UNIV LA HAB/
GUERRERO, F	1915	FALSE		FALSE		0 UNIV ORIENTI
GUERRERO, L	1917	FALSE	UNAM	FALSE	698	1 UNAM
GUERRERO, R	1918	TRUE		FALSE		0 UAM A
GUERRERO, R	1919	TRUE	UNAM	FALSE	698	1 UNAM
GUERRERO, R	1920	FALSE	UNIV SONOR/	FALSE	987	1 UNIV SONOR/
GUERRERO-P/	1921	FALSE	IPN	FALSE	376	1 IPN
GUEVARA, J	1924	TRUE	BOSTON UNI\	FALSE	44	1 BOSTON UNI\
GUEVARA, J	1925	TRUE		TRUE		0 COMIS NACL I
GUEVARA, J	1926	FALSE		FALSE		0 UNIV LA HAB/
GUILLAUMIN-	1932	TRUE	BUAP	FALSE	51	1 BUAP
GUILLAUMIN-	1933	FALSE	UAM A	FALSE	685	1 UAM A
GUIMARAES,	1935	TRUE		FALSE		0 HOPE COLL
GUIMARAES,	1936	TRUE	INDIANA UNI'	FALSE	282	1 INDIANA UNI'
GUIMARAES,	1937	TRUE		FALSE		0 INST SUPER TI
GUIMARAES,	1938	FALSE	UNIV NOTRE I	FALSE	923	1 UNIV NOTRE I
GUIRADO-LOI	1939	FALSE	UASLP	FALSE	689	1 UASLP
GUMEN, LN	1940	FALSE		FALSE		0 UKRAINIAN A
GUPTA, V	1943	FALSE		FALSE		0 CINVESTAV
GUREVICH, Y(	1945	TRUE		FALSE		0 BAR ILAN UNI
GUREVICH, Y(	1946	TRUE		TRUE		0 CINVESTAV
GUREVICH, Y(	1947	FALSE		FALSE		0 POLITECH UN
GUTIERREZ, A	1948	TRUE	BUAP	FALSE	51	1 BUAP
GUTIERREZ, A	1949	TRUE		TRUE		0 ININ
GUTIERREZ, A	1950	FALSE		FALSE		0 UNAM
GUTIERREZ, E	1951	FALSE	INAOE	FALSE	276	1 INAOE
GUTIERREZ, G	1953	FALSE	UNAM	FALSE	698	1 UNAM
GUTIERREZ, L	1956	FALSE	UNIV AUTON(	FALSE	718	1 UNIV AUTON(
GUTIERREZ-A	1958	FALSE	UNAM	FALSE	698	1 UNAM
GUTIERREZ-R(	1962	TRUE	BUAP	FALSE	51	1 BUAP
GUTIERREZ-R(	1963	TRUE		TRUE		0 ISAS
GUTIERREZ-R(	1964	FALSE		FALSE		0 UAZ
GUTIERREZ-T/	1965	FALSE		FALSE		0 ININ
GUTIERREZ-VI	1966	TRUE		TRUE		0 IMPERIAL COI
GUTIERREZ-VI	1967	FALSE		FALSE		0 INAOE

GUVEN, J	1968	FALSE	UNAM	FALSE	698	1	UNAM
GUZMAN, E	1972	TRUE		FALSE		0	INST INVEST E
GUZMAN, E	1973	FALSE	UAM I	FALSE	686	1	UAM I
GUZMAN, J	1974	FALSE	UNAM	FALSE	698	1	UNAM
HAAS, F	1979	TRUE	Inst Rech Sub	FALSE	344	1	Inst Rech Sub
HAAS, F	1980	FALSE		FALSE		0	UNIV HASSAN
HAGEL, K	1982	FALSE	UNIV BARI	FALSE	733	1	UNIV BARI
HANIC, F	1989	TRUE	SLOVAK ACAD	FALSE	613	1	SLOVAK ACAD
HANIC, F	1990	FALSE		FALSE		0	UNAM
HARO-PONIAT	1999	FALSE	UAM I	FALSE	686	1	UAM I
HART, C	2003	FALSE	UNIV HAVAN	FALSE	831	1	UNIV HAVAN
HARTOUNI, EI	2005	FALSE	UNIV MASSAC	FALSE	886	1	UNIV MASSAC
HAUS, JW	2008	TRUE		FALSE		0	INAOE
HAUS, JW	2009	TRUE	RENSSELAER I	FALSE	576	1	RENSSELAER I
HAUS, JW	2010	FALSE		FALSE		0	UNIV DAYTON
HAYASHI, M	2014	TRUE		FALSE		0	RUSSIAN FRIE
HAYASHI, M	2015	FALSE	SAITAMA UNI	FALSE	596	1	SAITAMA UNI
HE, XF	2020	FALSE	UNIV BRITISH	FALSE	744	1	UNIV BRITISH
HEHL, FW	2022	TRUE	CINVESTAV	FALSE	94	1	CINVESTAV
HEHL, FW	2023	FALSE	UAM I	FALSE	686	1	UAM I
HELLWIG, KE	2029	FALSE	UNAM	FALSE	698	1	UNAM
HENDERSON,	2033	TRUE		TRUE		0	BRIGHAM YOU
HENDERSON,	2034	TRUE		TRUE		0	UAM I
HENDERSON,	2035	FALSE		FALSE		0	UNAM
HERAS, JA	2039	FALSE	UNAM	FALSE	698	1	UNAM
HERNANDEZ,	2042	FALSE	UNAM	FALSE	698	1	UNAM
HERNANDEZ,	2048	FALSE		FALSE		0	CINVESTAV
HERNANDEZ,	2050	TRUE		TRUE		0	CINVESTAV
HERNANDEZ,	2051	TRUE		TRUE		0	UNIV HAVAN
HERNANDEZ,	2052	FALSE		FALSE		0	UNIV SONOR
HERNANDEZ,	2054	FALSE	CINVESTAV	FALSE	94	1	CINVESTAV
HERNANDEZ,	2055	TRUE	BUAP	FALSE	51	1	BUAP
HERNANDEZ,	2056	TRUE		TRUE		0	UAM I
HERNANDEZ,	2057	FALSE		FALSE		0	UAZ
HERNANDEZ,	2059	TRUE	CINVESTAV	FALSE	94	1	CINVESTAV
HERNANDEZ,	2060	FALSE		FALSE		0	UNIV HAVAN
HERNANDEZ,	2062	FALSE	CINVESTAV	FALSE	94	1	CINVESTAV
HERNANDEZ-	2073	TRUE	IPN	FALSE	376	1	IPN
HERNANDEZ-	2074	FALSE		FALSE		0	UNIV WISCON
HERNANDEZ-	2076	FALSE	UNAM	FALSE	698	1	UNAM
HERNANDEZ-	2077	FALSE	CINVESTAV	FALSE	94	1	CINVESTAV
HERNANDEZ-	2078	TRUE	CINVESTAV	FALSE	94	1	CINVESTAV
HERNANDEZ-	2079	FALSE	UNIV SURCOL	FALSE	992	1	UNIV SURCOL
HERNANDEZG	2080	FALSE		FALSE		0	IPN
HERNANDEZJ	2081	FALSE	UASLP	FALSE	689	1	UASLP
HERNANDEZ-	2088	FALSE		FALSE		0	UNAM
HERRERA, A	2092	FALSE	UNIV FED MIN	FALSE	803	1	UNIV FED MIN

HERRERA, G	2093	FALSE	CINVESTAV	FALSE	94	1	CINVESTAV
HERRERA, JJE	2096	FALSE	UNAM	FALSE	698	1	UNAM
HERRERA, JN	2098	FALSE	BUAP	FALSE	51	1	BUAP
HERRERA, MD	2100	FALSE	UNAM	FALSE	698	1	UNAM
HERRERA, R	2101	FALSE		FALSE		0	UNAM
HERRERA-GOI	2102	FALSE	STANFORD UI	FALSE	628	1	STANFORD UI
HERSEE, SD	2106	FALSE		FALSE		0	UNIV NEW MI
HESS, PO	2109	TRUE	LOUISIANA ST	FALSE	451	1	LOUISIANA ST
HESS, PO	2110	FALSE	UNAM	FALSE	698	1	UNAM
HEUER, D	2112	TRUE		TRUE		0	CNRS
HEUER, D	2113	TRUE		FALSE		0	INST NATL PH
HEUER, D	2114	FALSE	UNAM	FALSE	698	1	UNAM
HINOJOSA, G	2121	FALSE	ITESM	FALSE	385	1	ITESM
HIRATA, GA	2125	FALSE	UNAM	FALSE	698	1	UNAM
HIRATA, VML	2126	FALSE	IPN	FALSE	376	1	IPN
HIRSCH, JG	2128	FALSE	UNAM	FALSE	698	1	UNAM
HNATOWICZ,	2131	TRUE	FERNUNIV	FALSE	220	1	FERNUNIV
HNATOWICZ,	2132	FALSE		FALSE		0	FERNUNIVERS
HOCHST, H	2136	TRUE	CINVESTAV	FALSE	94	1	CINVESTAV
HOCHST, H	2137	FALSE		FALSE		0	UNIV WISCON
HOWIE, RA	2149	FALSE	CYDSA	FALSE	161	1	CYDSA
HSU, L	2151	FALSE		FALSE		0	UNIV CALIF BI
HSU, WK	2152	TRUE		TRUE		0	MAX PLANCK
HSU, WK	2153	TRUE		TRUE		0	UNAM
HSU, WK	2154	FALSE		FALSE		0	UNIV SUSSEX
HU, HL	2155	FALSE		FALSE		0	UNAM
HU, WB	2156	FALSE	UNIV SUSSEX	FALSE	994	1	UNIV SUSSEX
HUANG, L	2157	FALSE		FALSE		0	TEXAS A&M U
HUGHEY, B	2167	TRUE		FALSE		0	HOPE COLL
HUGHEY, B	2168	FALSE	UNIV MICHIG	FALSE	887	1	UNIV MICHIG
HUSAIN, V	2171	FALSE		FALSE		0	UNIV ALBERT/
HUSSIN, V	2172	FALSE	UNIV MONTR	FALSE	897	1	UNIV MONTR
IACHELLO, F	2174	TRUE	UNAM	FALSE	698	1	UNAM
IACHELLO, F	2175	FALSE		FALSE		0	YALE UNIV
IBARRA, J	2177	FALSE	INAOE	FALSE	276	1	INAOE
IBARRA, JG	2178	FALSE	INAOE	FALSE	276	1	INAOE
IBARRA-BRAC	2179	FALSE	UNAM	FALSE	698	1	UNAM
IBORT, A	2182	TRUE		FALSE		0	UNIV CARLOS
IBORT, A	2183	FALSE	UNIV COMPLI	FALSE	775	1	UNIV COMPLI
IIDA, M	2184	TRUE	NAGOYA UNI'	FALSE	491	1	NAGOYA UNI'
IIDA, M	2185	FALSE	UNIV SONOR/	FALSE	987	1	UNIV SONOR/
ILENKO, KV	2189	FALSE	NATL ACAD S(	FALSE	500	1	NATL ACAD S(
ILICHEV, NN	2190	FALSE		FALSE		0	RUSSIAN ACA
ILINSKII, AV	2191	FALSE	BUAP	FALSE	51	1	BUAP
INIGUEZ, MP	2196	FALSE	UNIV VALLAD	FALSE	1011	1	UNIV VALLAD
INOUE, MB	2198	FALSE		FALSE		0	UNIV SONOR/
IOFFE, LM	2199	FALSE	UAM I	FALSE	686	1	UAM I

IRIBARREN, A	2200	TRUE		TRUE		0	IPN
IRIBARREN, A	2201	TRUE		FALSE		0	UNIV HAVANA
IRIBARREN, A	2202	FALSE		FALSE		0	UNIV LA HABA
IRIBE, AA	2203	FALSE	CTR INVEST O	FALSE	148	1	CTR INVEST O
IRVINE, JTS	2204	FALSE		FALSE		0	UNIV ABERDE
ITSUKAICHI, T	2208	FALSE	TOYOHASHI U	FALSE	676	1	TOYOHASHI U
ITURBE-CASTI	2209	FALSE	INAOE	FALSE	276	1	INAOE
IVLEV, BI	2212	FALSE	UASLP	FALSE	689	1	UASLP
IZOTOV, AD	2214	FALSE	NS KURNAKO	FALSE	532	1	NS KURNAKO
IZRAILEV, FM	2215	TRUE		TRUE		0	BUAP
IZRAILEV, FM	2216	TRUE		TRUE		0	HARVARD UN
IZRAILEV, FM	2217	FALSE		FALSE		0	UNAM
JACOBO-ESCO	2221	TRUE	BUAP	FALSE	51	1	BUAP
JACOBO-ESCO	2222	FALSE		FALSE		0	UNIV AUTON
JACOBS, JP	2223	TRUE	UNAM	FALSE	698	1	UNAM
JACOBS, JP	2224	FALSE		FALSE		0	UNIV SEVILLA
JAMASBI, N	2228	TRUE	CALIF STATE U	FALSE	56	1	CALIF STATE U
JAMASBI, N	2229	FALSE		FALSE		0	CICESE
JAQUE, D	2232	TRUE	UAM I	FALSE	686	1	UAM I
JAQUE, D	2233	FALSE	UNIV AUTON	FALSE	727	1	UNIV AUTON
JARA, DR	2236	FALSE	INST NATL SCI	FALSE	333	1	INST NATL SCI
JARAMILLO, A	2239	TRUE	INAOE	FALSE	276	1	INAOE
JARAMILLO, A	2240	FALSE		FALSE		0	UNIV AUTON
JARAMILLO, D	2241	TRUE		FALSE		0	CINVESTAV
JARAMILLO, D	2242	FALSE	UNIV ANTIOQ	FALSE	712	1	UNIV ANTIOQ
JARAMILLO, C	2243	FALSE		FALSE		0	UNAM
JARAMILLO-N	2244	FALSE	INAOE	FALSE	276	1	INAOE
JARAMILLO-V	2245	FALSE	ECOLE POLYTE	FALSE	190	1	ECOLE POLYTE
JELLINEK, J	2250	TRUE	ARGONNE NA	FALSE	21	1	ARGONNE NA
JELLINEK, J	2251	FALSE		FALSE		0	UNAM
JENSEN, DA	2252	FALSE	TEXAS A&M U	FALSE	663	1	TEXAS A&M U
JENSEN, P	2253	FALSE		FALSE		0	UNIV LYON 1
JERGEL, M	2255	TRUE		FALSE		0	IPN
JERGEL, M	2256	FALSE	SLOVAK ACAD	FALSE	613	1	SLOVAK ACAD
JIANG, HG	2258	FALSE	UNAM	FALSE	698	1	UNAM
JIANG, Y	2259	TRUE	UNAM	FALSE	698	1	UNAM
JIANG, Y	2260	FALSE	UNIV AUTON	FALSE	725	1	UNIV AUTON
JIMENEZ, M	2265	TRUE	UNAM	FALSE	698	1	UNAM
JIMENEZ, M	2266	FALSE	UNIV PANAM	FALSE	937	1	UNIV PANAM
JIMENEZ-AQU	2269	TRUE		FALSE		0	UAM I
JIMENEZ-AQU	2270	FALSE	UNIV BARCEL	FALSE	732	1	UNIV BARCEL
JIMENEZ-BECI	2271	TRUE		FALSE		0	ININ
JIMENEZ-BECI	2272	FALSE	UAM I	FALSE	686	1	UAM I
JIMENEZ-MIEI	2276	TRUE		FALSE		0	UNAM
JIMENEZ-MIEI	2277	FALSE	UNIV CENT FL	FALSE	766	1	UNIV CENT FL
JIMENEZ-SAN	2278	FALSE		FALSE		0	CINVESTAV
JIMENEZ-SAN	2279	TRUE	CINVESTAV	FALSE	94	1	CINVESTAV

JIMENEZ-SAN	2280	FALSE		FALSE		0	TOYOHASHI U
JITRIK, O	2283	FALSE	UNAM	FALSE	698	1	UNAM
JOLIE, J	2285	TRUE	INST NUCL W	FALSE	336	1	INST NUCL W
JOLIE, J	2286	FALSE	UNAM	FALSE	698	1	UNAM
JORGE, MC	2290	TRUE	UNAM	FALSE	698	1	UNAM
JORGE, MC	2291	FALSE		FALSE		0	UNIV EDINBU
JOU, D	2296	TRUE		TRUE		0	UNAM
JOU, D	2297	FALSE		FALSE		0	UNIV AUTON(
JOUANNE, M	2298	TRUE	UAM I	FALSE	686	1	UAM I
JOUANNE, M	2299	FALSE		FALSE		0	UNIV PARIS 6
JUAREZ, H	2303	FALSE	UNIV GUANA.	FALSE	823	1	UNIV GUANA.
JUAREZ-ISLAS	2306	FALSE	UNAM	FALSE	698	1	UNAM
JUAREZ-PERE:	2307	FALSE	INAOE	FALSE	276	1	INAOE
JULIEN, C	2308	TRUE	UNAM	FALSE	698	1	UNAM
JULIEN, C	2309	FALSE	UNIV PARIS 6	FALSE	940	1	UNIV PARIS 6
JUNG, C	2310	FALSE	UNAM	FALSE	698	1	UNAM
JUNGNICKEL,	2311	TRUE		FALSE		0	UNAM
JUNGNICKEL,	2312	FALSE	UNIV EXETER	FALSE	797	1	UNIV EXETER
KALINOV, AV	2315	FALSE	UKRAINIAN A	FALSE	697	1	UKRAINIAN A
KAMSHILIN, A	2320	FALSE	UNIV JOENSU	FALSE	846	1	UNIV JOENSU
KANAUN, SK	2321	FALSE	ITESM	FALSE	385	1	ITESM
KAPLAN, IG	2323	FALSE	UNAM	FALSE	698	1	UNAM
KASAI, H	2330	TRUE	HERIOT WATT	FALSE	250	1	HERIOT WATT
KASAI, H	2331	FALSE		FALSE		0	NATL INST MA
KAUFMANN, (	2335	TRUE		FALSE		0	CTR INVEST O
KAUFMANN, (	2336	FALSE	INST FIS	FALSE	310	1	INST FIS
KAWECKI, L	2337	FALSE	IPN	FALSE	376	1	IPN
KERNER, R	2343	FALSE	UNIV PARIS 6	FALSE	940	1	UNIV PARIS 6
KERR, D	2344	TRUE		FALSE		0	CTR INVEST O
KERR, D	2345	FALSE	LOUGHBOROU	FALSE	450	1	LOUGHBOROU
KHOMENKO, /	2348	TRUE	CICESE	FALSE	89	1	CICESE
KHOMENKO, /	2349	FALSE		FALSE		0	UNIV JOENSU
KIKUCHI, R	2354	FALSE		FALSE		0	UNIV CALIF L(
KILNER, JA	2355	FALSE		FALSE		0	UNAM
KIRCHBACH, M	2360	FALSE	UAZ	FALSE	690	1	UAZ
KIRYANOV, AV	2363	TRUE		TRUE		0	CTR INVEST O
KIRYANOV, AV	2364	FALSE		FALSE		0	UNIV JOENSU
KLEIN, R	2365	FALSE	CINVESTAV	FALSE	94	1	CINVESTAV
KLEIN-KREISLI	2367	FALSE	UNAM	FALSE	698	1	UNAM
KLETT, R	2368	FALSE		FALSE		0	GESELL SCHW
KLIMOV, AB	2371	TRUE	AN LEBEDEV F	FALSE	18	1	AN LEBEDEV F
KLIMOV, AB	2372	TRUE	PN LEBEDEV F	FALSE	555	1	PN LEBEDEV F
KLIMOV, AB	2373	TRUE	SV LEBEDEV P	FALSE	640	1	SV LEBEDEV P
KLIMOV, AB	2374	FALSE		FALSE		0	UNIV GUADAI
KLIMOVA, T	2375	FALSE	UNAM	FALSE	698	1	UNAM
KOBAYASHI, T	2383	TRUE		FALSE		0	KYOTO UNIV
KOBAYASHI, T	2384	FALSE	UNIV HELSINK	FALSE	833	1	UNIV HELSINK

KODZHESPIRO	2386	FALSE		FALSE		0	NATL ACAD SC
KOLATA, JJ	2388	FALSE	ININ	FALSE	289	1	ININ
KORNEEV, NA	2392	FALSE		FALSE		0	INAOE
KOROLIJA, M	2393	TRUE	OAK RIDGE NA	FALSE	533	1	OAK RIDGE NA
KOROLIJA, M	2394	FALSE		FALSE		0	UNAM
KOSHEVAYA, S	2396	TRUE		FALSE		0	INAOE
KOSHEVAYA, S	2397	FALSE	SIMON FRASE	FALSE	610	1	SIMON FRASE
KOSHIZAKI, N	2398	TRUE	BUAP	FALSE	51	1	BUAP
KOSHIZAKI, N	2399	FALSE		FALSE		0	NATL INST MA
KOTLICKI, A	2404	FALSE	CINVESTAV	FALSE	94	1	CINVESTAV
KOTSARENKO	2406	TRUE	UNAM	FALSE	698	1	UNAM
KOTSARENKO	2407	FALSE		FALSE		0	UNIV AUTONO
KOTTOS, T	2408	FALSE		FALSE		0	BUAP
KOUZNETSOV	2409	TRUE	PN LEBEDEV P	FALSE	555	1	PN LEBEDEV P
KOUZNETSOV	2410	FALSE		FALSE		0	UNAM
KOVALENKO,	2412	TRUE		FALSE		0	IIT
KOVALENKO,	2413	FALSE	UNAM	FALSE	698	1	UNAM
KOZIEROWSK	2417	FALSE	ADAM MICKIE	FALSE	9	1	ADAM MICKIE
KRASHENINN	2425	TRUE	MIT	FALSE	480	1	MIT
KRASHENINN	2426	FALSE	UNAM	FALSE	698	1	UNAM
KRATZIG, E	2430	FALSE		FALSE		0	INAOE
KRAUSE, MO	2431	FALSE	OAK RIDGE NA	FALSE	533	1	OAK RIDGE NA
KRAVCHENKO	2433	FALSE	IPN	FALSE	376	1	IPN
KREISLER, MN	2435	FALSE	FERMILAB NA	FALSE	219	1	FERMILAB NA
KROKHIN, AA	2441	FALSE		FALSE		0	BUAP
KROTZSCH, G	2443	FALSE		FALSE		0	UNAM
KUBO, J	2445	FALSE	KANAZAWA U	FALSE	398	1	KANAZAWA U
KUCHERENKO	2448	TRUE		TRUE		0	CINVESTAV
KUCHERENKO	2449	FALSE		FALSE		0	IPN
KULKARNI, RN	2453	TRUE		TRUE		0	ATOM ENERG
KULKARNI, RN	2454	TRUE		TRUE		0	Univ Sao Marc
KULKARNI, RN	2455	TRUE		FALSE		0	UNIV SAO PAU
KULKARNI, RN	2456	FALSE	UNIV SONORA	FALSE	987	1	UNIV SONORA
KUMAR, J	2457	TRUE		FALSE		0	UNIV LOWELL
KUMAR, J	2458	FALSE	UNIV MASSAC	FALSE	886	1	UNIV MASSAC
KUMAR, N	2459	FALSE	INDIAN INST S	FALSE	280	1	INDIAN INST S
KUNOLD, A	2463	FALSE	UAM A	FALSE	685	1	UAM A
KURMYSHEV,	2464	TRUE	CENT BUR DES	FALSE	74	1	CENT BUR DES
KURMYSHEV,	2465	FALSE		FALSE		0	UNIV SONORA
KUSHWAHA, I	2466	FALSE		FALSE		0	BUAP
KUZIN, E	2467	FALSE	INAOE	FALSE	276	1	INAOE
KUZIN, EA	2468	FALSE		FALSE		0	INAOE
LACH-HAB, M	2472	FALSE		FALSE		0	GEORGE MAS
LACOMBA, EA	2474	TRUE	UAM A	FALSE	685	1	UAM A
LACOMBA, EA	2475	FALSE		FALSE		0	UAM I
LAKAMP, S	2479	TRUE		FALSE		0	INST MAGNET
LAKAMP, S	2480	FALSE	IPCMS	FALSE	373	1	IPCMS

LAMMERZAHI	2482	FALSE	UNIV KONSTA	FALSE	854	1	UNIV KONSTA
LANDGRAVE,	2484	FALSE	CTR INVEST O	FALSE	148	1	CTR INVEST O
LARIOS, F	2488	FALSE	CTR BRASILEII	FALSE	135	1	CTR BRASILEII
LARRALDE, H	2489	FALSE		FALSE		0	UNAM
LASTRAS-MAF	2496	TRUE		FALSE		0	MAX PLANCK
LASTRAS-MAF	2497	FALSE	UASLP	FALSE	689	1	UASLP
LAU, ML	2500	FALSE	UNIV CALIF IR	FALSE	749	1	UNIV CALIF IR
LAVERNIA, EJ	2502	FALSE		FALSE		0	UNIV CALIF IR
LEA, MJ	2504	FALSE	UNAM	FALSE	698	1	UNAM
LEAUSTIC, A	2506	FALSE	UNIV PARIS 1	FALSE	939	1	UNIV PARIS 1
LEE, WB	2511	TRUE		TRUE		0	HONG KONG
LEE, WB	2512	FALSE		FALSE		0	UNAM
LEGARDA-SAE	2513	FALSE		FALSE		0	CTR INVEST O
LEMUS, R	2517	FALSE		FALSE		0	UNAM
LEON, C	2520	FALSE		FALSE		0	UNIV COMPLU
LEON, H	2522	TRUE	CUJAE	FALSE	159	1	CUJAE
LEON, H	2523	FALSE		FALSE		0	IS Politecn JA
LEPINESZILY, /	2528	TRUE		FALSE		0	DIPARTIMENT
LEPINESZILY, /	2529	FALSE	GANIL	FALSE	230	1	GANIL
LESKOVA, TA	2534	TRUE		FALSE		0	RUSSIAN ACA
LESKOVA, TA	2535	FALSE	UNIV CALIF IR	FALSE	749	1	UNIV CALIF IR
LEVAI, G	2539	TRUE		TRUE		0	HUNGARIAN /
LEVAI, G	2540	FALSE		FALSE		0	UNAM
LEVIATAN, A	2543	FALSE		FALSE		0	HEBREW UNIV
LEYVA, A	2546	FALSE	UNAM	FALSE	698	1	UNAM
LEYVA-LUCER	2548	FALSE	CICESE	FALSE	89	1	CICESE
LEYVRAZ, F	2549	FALSE		FALSE		0	UNAM
LEZNOV, AN	2551	TRUE	PROTVINO HI	FALSE	567	1	PROTVINO HI
LEZNOV, AN	2552	FALSE		FALSE		0	UNAM
LI, CL	2553	FALSE		FALSE		0	E CHINA UNIV
LICEA-CLAVEF	2557	TRUE		FALSE		0	INST TECNOL
LICEA-CLAVEF	2558	FALSE	UNAM	FALSE	698	1	UNAM
LICHTENTHAL	2559	TRUE	IST NAZL FIS N	FALSE	383	1	IST NAZL FIS N
LICHTENTHAL	2560	FALSE		FALSE		0	UNIV SAO PAU
LIEBSCH, A	2562	FALSE	KFA JULICH G	FALSE	404	1	KFA JULICH G
LIMA, R	2563	TRUE		TRUE		0	Harbison Wal
LIMA, R	2564	FALSE		FALSE		0	UNAM
LINAN, A	2566	FALSE	UPM	FALSE	1037	1	UPM
LINDENBERG,	2568	FALSE	UNIV CALIF S/	FALSE	752	1	UNIV CALIF S/
LIPP, C	2572	FALSE	UNIV BASEL	FALSE	734	1	UNIV BASEL
LIRA, A	2574	FALSE		FALSE		0	UAM I
LLAMAS, RG	2581	FALSE		FALSE		0	CICESE
LOGVINOV, G	2591	FALSE	KHARKOV AM	FALSE	405	1	KHARKOV AM
LOHMANN, A	2592	FALSE		FALSE		0	UNIV ERLANG
LOMBARDI, M	2597	TRUE	UNIV JOSEPH	FALSE	847	1	UNIV JOSEPH
LOMBARDI, M	2598	FALSE		FALSE		0	UNIV JOSEPH
LOMNITZADLI	2600	FALSE	UNAM	FALSE	698	1	UNAM

LOPEZ, A	2605	FALSE	UNAM	FALSE	698	1	UNAM
LOPEZ, D	2607	FALSE	INST INVEST E	FALSE	318	1	INST INVEST E
LOPEZ, GV	2611	TRUE		FALSE		0	LOS ALAMOS
LOPEZ, GV	2612	FALSE	UNIV ILLINOIS	FALSE	840	1	UNIV ILLINOIS
LOPEZ, HF	2614	FALSE	INST TECNOL	FALSE	361	1	INST TECNOL
LOPEZ, JA	2616	TRUE		TRUE		0	INST MEXICAN
LOPEZ, JA	2617	TRUE		FALSE		0	UNIV BUENOS
LOPEZ, JA	2618	FALSE	UNIV TEXAS	FALSE	997	1	UNIV TEXAS
LOPEZ, JC	2619	TRUE	UNAM	FALSE	698	1	UNAM
LOPEZ, JC	2620	FALSE		FALSE		0	UNIV BONN
LOPEZ, JEH	2621	FALSE	CTR INVEST O	FALSE	148	1	CTR INVEST O
LOPEZ, JG	2622	FALSE	UNIV PARIS 7	FALSE	941	1	UNIV PARIS 7
LOPEZ, M	2626	FALSE	CINVESTAV	FALSE	94	1	CINVESTAV
LOPEZ, R	2630	TRUE	ININ	FALSE	289	1	ININ
LOPEZ, R	2631	TRUE		TRUE		0	UNAM
LOPEZ, R	2632	FALSE		FALSE		0	UNIV TOKYO
LOPEZ, S	2633	FALSE	CINVESTAV	FALSE	94	1	CINVESTAV
LOPEZ, T	2634	FALSE	UAM I	FALSE	686	1	UAM I
LOPEZ, VM	2635	TRUE		FALSE		0	IPN
LOPEZ, VM	2636	FALSE	TOYOHASHI U	FALSE	676	1	TOYOHASHI U
LOPEZ-BASTID	2637	TRUE	UNAM	FALSE	698	1	UNAM
LOPEZ-BASTID	2638	FALSE		FALSE		0	UNIV AUTON(
LOPEZ-BOLAN	2639	FALSE	BUAP	FALSE	51	1	BUAP
LOPEZ-BONILI	2640	TRUE		TRUE		0	UAM A
LOPEZ-BONILI	2641	FALSE		FALSE		0	UDLAP
LOPEZ-CHAVE	2642	FALSE	IPN	FALSE	376	1	IPN
LOPEZ-HIRAT/	2644	FALSE		FALSE		0	IPN
LOPEZ-LEMUS	2645	FALSE		FALSE		0	UAM I
LOPEZ-LOPEZ,	2646	FALSE	UAM A	FALSE	685	1	UAM A
LOPEZ-LOPEZ,	2647	TRUE		FALSE		0	ARGONNE NA
LOPEZ-LOPEZ,	2648	FALSE	CINVESTAV	FALSE	94	1	CINVESTAV
LOPEZMORAL	2651	TRUE		TRUE		0	COLUMBIA UI
LOPEZMORAL	2652	TRUE		FALSE		0	IBM CORP
LOPEZMORAL	2653	FALSE	UNAM	FALSE	698	1	UNAM
LOPEZ-MOREI	2654	FALSE	UNAM	FALSE	698	1	UNAM
LOPEZOLAZA(	2655	FALSE	INAOE	FALSE	276	1	INAOE
LOPEZ-PENA,	2656	FALSE		FALSE		0	UNAM
LOPEZ-RIOS, T	2657	FALSE	UNAM	FALSE	698	1	UNAM
LOPEZTELLEZ,	2661	FALSE		FALSE		0	UNAM
LORO, H	2667	FALSE	INST PHYS & T	FALSE	339	1	INST PHYS & T
LOSEV, A	2669	TRUE	BULGARIAN A	FALSE	53	1	BULGARIAN A
LOSEV, A	2670	FALSE		FALSE		0	INST GEN & IN
LOSKE, AM	2671	FALSE	UNAM	FALSE	698	1	UNAM
LOYOLA, G	2677	TRUE		FALSE		0	MOSCOW MV
LOYOLA, G	2678	FALSE	UNAM	FALSE	698	1	UNAM
LOZADA-CASS	2680	FALSE	UAM I	FALSE	686	1	UAM I
LOZADA-MOR	2681	TRUE		FALSE		0	CINVESTAV

LOZADA-MOR	2682	TRUE	INPE	FALSE	291	1	INPE
LOZADA-MOR	2683	FALSE		FALSE		0	UNIV LA HABA
LU, JQ	2684	TRUE		FALSE		0	CICESE
LU, JQ	2685	TRUE	CSIC	FALSE	130	1	CSIC
LU, JQ	2686	FALSE	UNIV CALIF IR	FALSE	749	1	UNIV CALIF IR
LUGO, JE	2696	TRUE		FALSE		0	UNAM
LUGO, JE	2697	FALSE	UNIV AUTON	FALSE	725	1	UNIV AUTON
LUNA, E	2699	FALSE		FALSE		0	UNAM
LUNA, H	2700	TRUE		FALSE		0	IMP
LUNA, H	2701	FALSE	UAM A	FALSE	685	1	UAM A
LUNA, RE	2702	TRUE	CICESE	FALSE	89	1	CICESE
LUNA, RE	2703	TRUE	SURFACE OPT	FALSE	639	1	SURFACE OPT
LUNA, RE	2704	FALSE		FALSE		0	UNIV AUTON
LUNA-ACOSTA	2705	FALSE		FALSE		0	BUAP
LUNA-AGUILA	2706	FALSE	UNAM	FALSE	698	1	UNAM
LUNA-MOREN	2708	TRUE		FALSE		0	CTR INVEST O
LUNA-MOREN	2709	FALSE	INAOE	FALSE	276	1	INAOE
LUNARDON, M	2710	TRUE		FALSE		0	IST NAZL FIS N
LUNARDON, M	2711	TRUE	UNAM	FALSE	698	1	UNAM
LUNARDON, M	2712	FALSE		FALSE		0	UNIV PADUA
LUSSON, A	2713	FALSE	CNRS	FALSE	103	1	CNRS
LUTHER-DAVI	2714	TRUE	AUSTRALIAN	FALSE	30	1	AUSTRALIAN
LUTHER-DAVI	2715	FALSE		FALSE		0	UAM I
LUTY, F	2716	FALSE	NANJING UNI	FALSE	493	1	NANJING UNI
LUYO-ALVARA	2717	TRUE		TRUE		0	CINVESTAV
LUYO-ALVARA	2718	FALSE		FALSE		0	UASLP
LUZZI, R	2719	TRUE	UAM A	FALSE	685	1	UAM A
LUZZI, R	2720	FALSE	UAM I	FALSE	686	1	UAM I
LYUBIMOV, O	2722	TRUE		FALSE		0	KHARKOV AM
LYUBIMOV, O	2723	FALSE	UKRAINIAN A	FALSE	697	1	UKRAINIAN A
MAAROOS, A	2728	FALSE	KYOTO SANGY	FALSE	420	1	KYOTO SANGY
MACHORRO,	2729	FALSE	CICESE	FALSE	89	1	CICESE
MACIAS, A	2730	FALSE	UAM I	FALSE	686	1	UAM I
MADRIGAL, R	2735	FALSE	UNIV AUTON	FALSE	725	1	UNIV AUTON
MADRIGAL-M	2736	FALSE	BUAP	FALSE	51	1	BUAP
MAGALINSKY,	2737	FALSE		FALSE		0	BUAP
MAGNIN, J	2740	TRUE	CINVESTAV	FALSE	94	1	CINVESTAV
MAGNIN, J	2741	FALSE		FALSE		0	CTR BRASILEI
MAKAROV, NI	2744	TRUE	ALFRED UNIV	FALSE	15	1	ALFRED UNIV
MAKAROV, NI	2745	TRUE		TRUE		0	BUAP
MAKAROV, NI	2746	TRUE		FALSE		0	NASU
MAKAROV, NI	2747	FALSE	UKRAINIAN A	FALSE	697	1	UKRAINIAN A
MAKSYMYK, I	2749	FALSE		FALSE		0	TRIUMF
MALACARA, D	2750	TRUE		FALSE		0	CTR INVEST O
MALACARA, D	2751	FALSE	UNIV ROCHES	FALSE	961	1	UNIV ROCHES
MALACARA, Z	2752	FALSE	CTR INVEST O	FALSE	148	1	CTR INVEST O
MALACARA-D	2753	FALSE		FALSE		0	CTR INVEST O

MALACARA-H	2754	FALSE		FALSE		0 CTR INVEST O
MALDONADO	2757	TRUE		TRUE		0 CINVESTAV
MALDONADO	2758	TRUE		FALSE		0 TOKYO INST T
MALDONADO	2759	FALSE	UNAM	FALSE	698	1 UNAM
MALLICK, S	2762	FALSE	CICESE	FALSE	89	1 CICESE
MALO, BM	2763	FALSE	UNAM	FALSE	698	1 UNAM
MAMEDOV, T	2764	FALSE	HACETTEPE U	FALSE	241	1 HACETTEPE U
MAN, HC	2765	TRUE		TRUE		0 HONG KONG
MAN, HC	2766	FALSE	UNAM	FALSE	698	1 UNAM
MANCILLA, JE	2767	FALSE		FALSE		0 BUAP
MANERO, O	2768	TRUE	CALTECH	FALSE	58	1 CALTECH
MANERO, O	2769	TRUE	UNAM	FALSE	698	1 UNAM
MANERO, O	2770	FALSE		FALSE		0 UNIV ILLES BA
MANKO, OV	2772	TRUE	MOSCOW MV	FALSE	488	1 MOSCOW MV
MANKO, OV	2773	FALSE		FALSE		0 UNIV SALAMA
MANKO, VI	2775	TRUE		FALSE		0 PN LEBEDEV P
MANKO, VI	2776	FALSE	UNAM	FALSE	698	1 UNAM
MANKO, VS	2777	TRUE		FALSE		0 CINVESTAV
MANKO, VS	2778	FALSE	UNIV SALAMA	FALSE	971	1 UNIV SALAMA
MANSUROVA	2780	TRUE		FALSE		0 UNIV AUTON
MANSUROVA	2781	FALSE	UNIV LONDOI	FALSE	872	1 UNIV LONDOI
MANZANARES	2782	TRUE	CICESE	FALSE	89	1 CICESE
MANZANARES	2783	FALSE		FALSE		0 UNIV SONORA
MANZANO-RA	2785	FALSE		FALSE		0 CINVESTAV
MANZUR, A	2786	FALSE	UAM I	FALSE	686	1 UAM I
MAPLE, MB	2787	TRUE	CINVESTAV	FALSE	94	1 CINVESTAV
MAPLE, MB	2788	TRUE		TRUE		0 DEPT PHYS
MAPLE, MB	2789	TRUE		TRUE		0 INST TECNOL
MAPLE, MB	2790	FALSE		FALSE		0 UNIV CALIF SA
MARADUDIN,	2791	TRUE		FALSE		0 CICESE
MARADUDIN,	2792	FALSE	UNIV CALIF IR	FALSE	749	1 UNIV CALIF IR
MARES, R	2795	FALSE	IPN	FALSE	376	1 IPN
MARIN, E	2796	TRUE	BUAP	FALSE	51	1 BUAP
MARIN, E	2797	FALSE		FALSE		0 CINVESTAV
MARKUS, F	2803	FALSE	LORAND EOTV	FALSE	448	1 LORAND EOTV
MARQUEZ, H	2806	TRUE	CICESE	FALSE	89	1 CICESE
MARQUEZ, H	2807	FALSE	UNAM	FALSE	698	1 UNAM
MARQUINA, V	2812	FALSE	UNAM	FALSE	698	1 UNAM
MARRERO, JC	2813	FALSE	UAM I	FALSE	686	1 UAM I
MARROQUIN,	2814	FALSE		FALSE		0 CIMAT
MARTEL, A	2816	TRUE		FALSE		0 CINVESTAV
MARTEL, A	2817	FALSE	LAMEL INST	FALSE	432	1 LAMEL INST
MARTINELL, J.	2822	FALSE		FALSE		0 UNAM
MARTINEZ, A	2823	TRUE	CATHOLIC UN	FALSE	64	1 CATHOLIC UN
MARTINEZ, A	2824	FALSE		FALSE		0 CINVESTAV
MARTINEZ, AI	2825	TRUE		FALSE		0 CINVESTAV
MARTINEZ, AI	2826	FALSE	INT CTR THEO	FALSE	369	1 INT CTR THEO

MARTINEZ, E	2830	FALSE	UNIV CAMBRI	FALSE	755	1	UNIV CAMBRI
MARTINEZ, EA	2831	FALSE		FALSE		0	UNAM
MARTINEZ, H	2834	FALSE		FALSE		0	UNAM
MARTINEZ, I	2835	FALSE	UNAM	FALSE	698	1	UNAM
MARTINEZ, JR	2844	FALSE	UASLP	FALSE	689	1	UASLP
MARTINEZ, L	2846	FALSE		FALSE		0	UNAM
MARTINEZ, M	2848	TRUE		FALSE		0	AT&T
MARTINEZ, M	2849	FALSE	WISSENSCHAI	FALSE	1062	1	WISSENSCHAI
MARTINEZ, O	2850	TRUE	UNIV HABANA	FALSE	825	1	UNIV HABANA
MARTINEZ, O	2851	FALSE		FALSE		0	UNIV LA HABA
MARTINEZ, R	2852	TRUE	CINVESTAV	FALSE	94	1	CINVESTAV
MARTINEZ, R	2853	TRUE		FALSE		0	NATL UNIV CO
MARTINEZ, R	2854	TRUE	UNIV MICHIG	FALSE	887	1	UNIV MICHIG
MARTINEZ, R	2855	FALSE	UNIV NACL CO	FALSE	904	1	UNIV NACL CO
MARTINEZ-DA	2861	FALSE	UNAM	FALSE	698	1	UNAM
MARTINEZ-DE	2862	FALSE	UANL	FALSE	687	1	UANL
MARTINEZ-DU	2864	TRUE	INST SUPER P	FALSE	351	1	INST SUPER P
MARTINEZ-DU	2865	FALSE		FALSE		0	UNIV AUTONO
MARTINEZ-M	2867	FALSE	UNAM	FALSE	698	1	UNAM
MARTINEZ-M	2868	TRUE		FALSE		0	INST SCI INTEI
MARTINEZ-M	2869	TRUE	IST NAZL FIS N	FALSE	383	1	IST NAZL FIS N
MARTINEZ-M	2870	TRUE		TRUE		0	UNAM
MARTINEZ-M	2871	FALSE		FALSE		0	UNIV CALIF LO
MARTINEZ-NI	2872	FALSE	INAOE	FALSE	276	1	INAOE
MARTINEZ-PA	2873	FALSE	ININ	FALSE	289	1	ININ
MARTINEZ-QU	2874	TRUE		FALSE		0	ININ
MARTINEZ-QU	2875	FALSE	UNIV TEXAS	FALSE	997	1	UNIV TEXAS
MARTINEZ-Y-I	2877	FALSE	UNAM	FALSE	698	1	UNAM
MARTINGIL, F	2878	TRUE	ESCUELA TECI	FALSE	202	1	ESCUELA TECI
MARTINGIL, F	2879	FALSE		FALSE		0	UNIV VALLAD
MARTINGIL, J	2880	TRUE	UNAM	FALSE	698	1	UNAM
MARTINGIL, J	2881	FALSE		FALSE		0	UNIV VALLAD
MARTI-PANAI	2882	FALSE		FALSE		0	INAOE
MARUHN, JA	2883	TRUE		FALSE		0	UNAM
MARUHN, JA	2884	FALSE	UNIV FRANKF	FALSE	813	1	UNIV FRANKF
MASHHOON,	2887	FALSE	UNIV MISSOU	FALSE	893	1	UNIV MISSOU
MATA-MENDI	2890	FALSE		FALSE		0	IPN
MATEOS, JL	2892	FALSE	UNAM	FALSE	698	1	UNAM
MATHELITSCH	2893	FALSE	GRAZ UNIV	FALSE	240	1	GRAZ UNIV
MATHER, GC	2894	TRUE	UNAM	FALSE	698	1	UNAM
MATHER, GC	2895	FALSE		FALSE		0	UNIV ABERDE
MATHEW, X	2896	TRUE		FALSE		0	NREL
MATHEW, X	2897	TRUE		FALSE		0	UNAM
MATHEW, X	2898	FALSE	UNIV STRASBO	FALSE	990	1	UNIV STRASBO
MATOS, T	2900	TRUE	CINVESTAV	FALSE	94	1	CINVESTAV
MATOS, T	2901	FALSE		FALSE		0	UAM A
MATSUDA, H	2903	TRUE		FALSE		0	CICESE

MATSUDA, H	2904	FALSE	TOHOKU UNI\	FALSE	669	1	TOHOKU UNI\
MATSUMOTC	2905	FALSE		FALSE		0	CINVESTAV
MATULIC, L	2907	FALSE	ST JOHN FISH	FALSE	621	1	ST JOHN FISH
MATUTES-AQ	2908	TRUE		TRUE		0	CINVESTAV
MATUTES-AQ	2909	FALSE		FALSE		0	CTR INVEST M
MAYAGOITIA,	2917	TRUE		FALSE		0	UAM I
MAYAGOITIA,	2918	FALSE	UNIV NACL SA	FALSE	911	1	UNIV NACL SA
MAYORGA, M	2919	FALSE	UAM I	FALSE	686	1	UAM I
MAYORGA, O	2920	FALSE	UNIV GUANA.	FALSE	823	1	UNIV GUANA.
MAYORGA-CF	2921	FALSE		FALSE		0	INAOE
MAYTORENA,	2922	TRUE		FALSE		0	UNAM
MAYTORENA,	2923	FALSE	UNIV AUTONO	FALSE	725	1	UNIV AUTONO
MCCLUNE, JC	2926	TRUE	UNAM	FALSE	698	1	UNAM
MCCLUNE, JC	2927	FALSE		FALSE		0	UNIV MISSOU
MCFARLANE,	2932	TRUE		FALSE		0	OSRAM SYLVA
MCFARLANE,	2933	FALSE	UNIV SO CALI	FALSE	985	1	UNIV SO CALI
MCGURN, AR	2934	TRUE		TRUE		0	GEORGIA INST
MCGURN, AR	2935	FALSE		FALSE		0	WESTERN MIC
MCINERNEY, .	2936	TRUE		FALSE		0	NATL UNIV IR
MCINERNEY, .	2937	FALSE	UNIV COLL CC	FALSE	771	1	UNIV COLL CC
MCKITTRICK, .	2939	TRUE		FALSE		0	UNAM
MCKITTRICK, .	2940	FALSE	UNIV CALIF SA	FALSE	752	1	UNIV CALIF SA
MCVOY, KW	2944	FALSE	UNIV WISCON	FALSE	1027	1	UNIV WISCON
MEDINA, A	2947	TRUE	UAZ	FALSE	690	1	UAZ
MEDINA, A	2948	TRUE		TRUE		0	UNIV AUTONO
MEDINA, A	2949	FALSE		FALSE		0	UNIV AUTONO
MEDINA, M	2950	FALSE		FALSE		0	UNIV MICHOA
MEDINA, R	2953	TRUE	UAM I	FALSE	686	1	UAM I
MEDINA, R	2954	FALSE		FALSE		0	UNIV ESTADU
MEDINA, SF	2955	TRUE		FALSE		0	CSIC
MEDINA, SF	2956	FALSE	MET RES NAT	FALSE	476	1	MET RES NAT
MEDINA-NOY	2957	TRUE	CINVESTAV	FALSE	94	1	CINVESTAV
MEDINA-NOY	2958	FALSE		FALSE		0	UNIV KONSTA
MEDRANO, F	2962	FALSE	UNIV ARIZON	FALSE	715	1	UNIV ARIZON
MEJIA, CR	2968	TRUE	UAM	FALSE	684	1	UAM
MEJIA, CR	2969	FALSE		FALSE		0	UNAM
MEJIA, JE	2972	FALSE		FALSE		0	CTR INVEST O
MEJIA-GARCI/	2973	FALSE	IPN	FALSE	376	1	IPN
MEJIA-MONA	2976	TRUE		FALSE		0	MAX PLANCK
MEJIA-MONA	2977	FALSE	UNAM	FALSE	698	1	UNAM
MEJIA-ROSAL	2978	FALSE		FALSE		0	UASLP
MELENDEZ, F	2980	FALSE	CINVESTAV	FALSE	94	1	CINVESTAV
MELENDEZ-LI	2981	FALSE	CINVESTAV	FALSE	94	1	CINVESTAV
MELENDREZ,	2982	TRUE	CICESE	FALSE	89	1	CICESE
MELENDREZ,	2983	FALSE	UNIV SONORA	FALSE	987	1	UNIV SONORA
MELNIKOV, A.	2986	FALSE	UNIV MINSK	FALSE	891	1	UNIV MINSK
MENA, B	2988	FALSE	UNAM	FALSE	698	1	UNAM

MENA, M	2989	TRUE		FALSE		0	ININ
MENA, M	2990	FALSE	UNAM	FALSE	698	1	UNAM
MENDEZ, A	2993	FALSE	CINVESTAV	FALSE	94	1	CINVESTAV
MENDEZ, ER	2995	TRUE		TRUE		0	CICESE
MENDEZ, ER	2996	TRUE		TRUE		0	CSIC
MENDEZ, ER	2997	FALSE		FALSE		0	GEORGIA INS
MENDEZ, F	2998	TRUE		TRUE		0	UNAM
MENDEZ, F	2999	FALSE		FALSE		0	UNIV BUENOS
MENDEZ, JM	3001	FALSE		FALSE		0	UNAM
MENDEZ, M	3002	TRUE	IPN	FALSE	376	1	IPN
MENDEZ, M	3003	FALSE		FALSE		0	UNAM
MENDEZ-ALC	3006	TRUE		FALSE		0	INST CHARLES
MENDEZ-ALC	3007	TRUE	UASLP	FALSE	689	1	UASLP
MENDEZ-ALC	3008	FALSE		FALSE		0	UNIV KONSTA
MENDEZ-GAR	3009	FALSE	CINVESTAV	FALSE	94	1	CINVESTAV
MENDEZ-OTE	3011	TRUE		FALSE		0	BUAP
MENDEZ-OTE	3012	FALSE	INAOE	FALSE	276	1	INAOE
MENDEZ-VIV	3014	FALSE	UAM I	FALSE	686	1	UAM I
MENDOZA, A	3019	FALSE	UNAM	FALSE	698	1	UNAM
MENDOZA, B	3020	FALSE		FALSE		0	CTR INVEST O
MENDOZA, C	3021	FALSE		FALSE		0	UNAM
MENDOZA, D	3022	FALSE		FALSE		0	UNAM
MENDOZA, H	3023	TRUE	BUAP	FALSE	51	1	BUAP
MENDOZA, H	3024	FALSE		FALSE		0	SAITAMA UNI
MENDOZA, M	3026	FALSE	BUAP	FALSE	51	1	BUAP
MENDOZA-AL	3027	FALSE	UNAM	FALSE	698	1	UNAM
MENDOZA-AL	3028	TRUE	IPN	FALSE	376	1	IPN
MENDOZA-AL	3029	FALSE		FALSE		0	UNAM
MENDOZA-AL	3030	FALSE	BUAP	FALSE	51	1	BUAP
MENDOZA-AN	3031	TRUE		TRUE		0	CTR INVEST O
MENDOZA-AN	3032	TRUE		FALSE		0	INAH
MENDOZA-AN	3033	TRUE		FALSE		0	UAQ
MENDOZA-AN	3034	FALSE	UNAM	FALSE	698	1	UNAM
MENDOZA-GA	3036	FALSE		FALSE		0	BUAP
MENDOZA-GO	3037	TRUE		FALSE		0	CINVESTAV
MENDOZA-GO	3038	TRUE	INST TECNOL	FALSE	361	1	INST TECNOL
MENDOZA-GO	3039	FALSE		FALSE		0	UNIV N TEXAS
MENDOZA-SE	3040	FALSE		FALSE		0	UAM I
MENDOZA-SU	3041	FALSE	CICESE	FALSE	89	1	CICESE
MENDOZA-SU	3042	FALSE		FALSE		0	CINVESTAV
MENENDEZ-P	3045	TRUE	CINVESTAV	FALSE	94	1	CINVESTAV
MENENDEZ-P	3046	FALSE		FALSE		0	UNIV LA HAB
MENESES-NA	3047	TRUE		FALSE		0	CTR INVEST O
MENESES-NA	3048	TRUE	INAOE	FALSE	276	1	INAOE
MENESES-NA	3049	FALSE	UNIV FED AL	FALSE	799	1	UNIV FED AL
MERCANDALL	3056	FALSE	THOMSON CS	FALSE	668	1	THOMSON CS
MESA, AD	3060	FALSE		FALSE		0	UNAM

MEURICE, Y	3063	FALSE	CINVESTAV	FALSE	94	1	CINVESTAV
MEZA-MONTI	3067	FALSE	BUAP	FALSE	51	1	BUAP
MICENMACHI	3068	FALSE	COLEGIO NAC	FALSE	108	1	COLEGIO NAC
MICHAELIAN,	3069	FALSE	UNAM	FALSE	698	1	UNAM
MICHEL, T	3070	FALSE		FALSE		0	UNIV CALIF IR
MIELKE, EW	3071	TRUE	CSIC	FALSE	130	1	CSIC
MIELKE, EW	3072	FALSE		FALSE		0	UAM I
MIELNIK, B	3073	FALSE		FALSE		0	CINVESTAV
MIHOKOVA, E	3076	TRUE		FALSE		0	ACAD SCI CZE
MIHOKOVA, E	3077	FALSE	UNIV SONORA	FALSE	987	1	UNIV SONORA
MIKI-YOSHIDA	3080	TRUE	MOBIL RES &	FALSE	482	1	MOBIL RES &
MIKI-YOSHIDA	3081	FALSE		FALSE		0	UNAM
MIMILA-ARRO	3087	FALSE	CINVESTAV	FALSE	94	1	CINVESTAV
MINZONI, AA	3089	FALSE	UNAM	FALSE	698	1	UNAM
MIRABAL-GAI	3091	FALSE	UASLP	FALSE	689	1	UASLP
MIRANDA, J	3094	FALSE	UNIV CALIF D.	FALSE	748	1	UNIV CALIF D.
MIRANDA, OC	3096	FALSE		FALSE		0	CINVESTAV
MIRIDONOV,	3099	FALSE	CICESE	FALSE	89	1	CICESE
MISHONOV, T	3104	FALSE		FALSE		0	UAZ
MISHURNYI, V	3105	FALSE		FALSE		0	UASLP
MITSKIEVICH,	3111	FALSE	UNIV GUADAI	FALSE	822	1	UNIV GUADAI
MITTIG, W	3112	FALSE	UNAM	FALSE	698	1	UNAM
MOHEBI, M	3119	TRUE	CICESE	FALSE	89	1	CICESE
MOHEBI, M	3120	FALSE	UNIV NEW M	FALSE	918	1	UNIV NEW M
MOKRANI, A	3121	FALSE	CNRS	FALSE	103	1	CNRS
MONDRAGON	3128	FALSE		FALSE		0	UNAM
MONDRAGON	3129	FALSE	CTR SCI ORSA	FALSE	156	1	CTR SCI ORSA
MONDRAGON	3130	FALSE	UNAM	FALSE	698	1	UNAM
MONDRAGON	3131	FALSE	UNAM	FALSE	698	1	UNAM
MONDRAGON	3133	FALSE	BUAP	FALSE	51	1	BUAP
MONSIVAIS, C	3137	TRUE	UAM I	FALSE	686	1	UAM I
MONSIVAIS, C	3138	FALSE		FALSE		0	UNAM
MONTANERO	3139	FALSE	UNIV EXTREM	FALSE	798	1	UNIV EXTREM
MONTANO, LI	3140	FALSE	CINVESTAV	FALSE	94	1	CINVESTAV
MONTEJANO-	3142	FALSE	UASLP	FALSE	689	1	UASLP
MONTEMAYC	3145	FALSE	UNAM	FALSE	698	1	UNAM
MONTERO, PI	3147	FALSE	INAOE	FALSE	276	1	INAOE
MONTESINOS	3149	TRUE		FALSE		0	CINVESTAV
MONTESINOS	3150	TRUE	CNRS	FALSE	103	1	CNRS
MONTESINOS	3151	FALSE	UNIV PITTSBU	FALSE	949	1	UNIV PITTSBU
MONTIEL, R	3153	TRUE		FALSE		0	AUSTRALIAN I
MONTIEL, R	3154	FALSE	UAM I	FALSE	686	1	UAM I
MONTOYA, A	3158	FALSE	UAM I	FALSE	686	1	UAM I
MONTOYA-HE	3159	FALSE	CTR INVEST O	FALSE	148	1	CTR INVEST O
MONZON-HEI	3161	TRUE		TRUE		0	CTR INVEST O
MONZON-HEI	3162	FALSE		FALSE		0	POLAROID CC
MOORE, AJ	3165	TRUE	CTR INVEST O	FALSE	148	1	CTR INVEST O

MOORE, AJ	3166	FALSE	LOUGHBORO	FALSE	450	1	LOUGHBORO
MORA, C	3167	FALSE	CINVESTAV	FALSE	94	1	CINVESTAV
MORALES, F	3172	FALSE		FALSE		0	UNAM
MORALES, J	3174	FALSE	IMP	FALSE	272	1	IMP
MORALES, M	3176	TRUE		FALSE		0	CINVESTAV
MORALES, M	3177	FALSE	UNAM	FALSE	698	1	UNAM
MORALES, U	3179	FALSE	CIDETEQ	FALSE	92	1	CIDETEQ
MORALES-AC	3180	FALSE		FALSE		0	CINVESTAV
MORALES-SA	3182	TRUE	BUAP	FALSE	51	1	BUAP
MORALES-SA	3183	TRUE		TRUE		0	CINVESTAV
MORALES-SA	3184	TRUE		TRUE		0	ENERGY CON
MORALES-SA	3185	FALSE		FALSE		0	UAQ
MORALES-TE	3186	TRUE		FALSE		0	DIPARTIMEN
MORALES-TE	3187	TRUE	INST ADV STU	FALSE	294	1	INST ADV STU
MORALES-TE	3188	FALSE	UAM I	FALSE	686	1	UAM I
MORAN, E	3189	FALSE	UNIV COMPL	FALSE	775	1	UNIV COMPL
MORA-RAMO	3195	TRUE	CTR INVEST O	FALSE	148	1	CTR INVEST O
MORA-RAMO	3196	TRUE	UAZ	FALSE	690	1	UAZ
MORA-RAMO	3197	FALSE		FALSE		0	UNIV AUTON
MOREAU, M	3198	TRUE		FALSE		0	UNAM
MOREAU, M	3199	FALSE	UNIV PARIS 6	FALSE	940	1	UNIV PARIS 6
MORENO, G	3201	FALSE	CINVESTAV	FALSE	94	1	CINVESTAV
MORENO, M	3203	FALSE	CINVESTAV	FALSE	94	1	CINVESTAV
MORGAN, TJ	3205	TRUE		FALSE		0	LAWRENCE B
MORGAN, TJ	3206	FALSE	WESLEYAN U	FALSE	1059	1	WESLEYAN U
MORHANGE,	3207	TRUE		FALSE		0	CSIC
MORHANGE,	3208	FALSE	UNIV PARIS 6	FALSE	940	1	UNIV PARIS 6
MOROZOV, D	3212	TRUE	CHALMERS U	FALSE	82	1	CHALMERS U
MOROZOV, D	3213	TRUE		TRUE		0	IV KURCHATO
MOROZOV, D	3214	FALSE		FALSE		0	UNAM
MORRISON, N	3215	FALSE	UNIV CAMBR	FALSE	755	1	UNIV CAMBR
MORSAD, A	3216	FALSE	UNIV NOTRE	FALSE	923	1	UNIV NOTRE
MOTA, RD	3220	FALSE		FALSE		0	IPN
MOYA-CESSA,	3225	TRUE	BRITISH TELE	FALSE	47	1	BRITISH TELE
MOYA-CESSA,	3226	TRUE		FALSE		0	INAOE
MOYA-CESSA,	3227	TRUE	SLOVAK ACAD	FALSE	613	1	SLOVAK ACAD
MOYA-CESSA,	3228	FALSE	UNIV LONDO	FALSE	871	1	UNIV LONDO
MUCINO, J	3230	FALSE	UNAM	FALSE	698	1	UNAM
MUHL, S	3234	TRUE	IPN	FALSE	376	1	IPN
MUHL, S	3235	FALSE		FALSE		0	UNAM
MULLER, M	3236	TRUE	MAX PLANCK	FALSE	467	1	MAX PLANCK
MULLER, M	3237	FALSE		FALSE		0	UNAM
MUMFORD, J	3240	FALSE		FALSE		0	UNAM
MUNOZ, A	3243	TRUE	UAM I	FALSE	686	1	UAM I
MUNOZ, A	3244	FALSE		FALSE		0	UNAM
MUNOZ, E	3245	TRUE	UNAM	FALSE	698	1	UNAM
MUNOZ, E	3246	FALSE	UNIV SONOR	FALSE	987	1	UNIV SONOR

MUNOZ, G	3247	TRUE	UAM I	FALSE	686	1	UAM I
MUNOZ, G	3248	FALSE	UNAM	FALSE	698	1	UNAM
MUNOZ, JA	3249	FALSE	UNIV AUTON(	FALSE	727	1	UNIV AUTON(
MUNOZ, JH	3251	TRUE		TRUE		0	CINVESTAV
MUNOZ, JH	3252	TRUE		TRUE		0	UNIV ANTIOQ
MUNOZ, JH	3253	TRUE		TRUE		0	UNIV NACL
MUNOZ, JH	3254	FALSE		FALSE		0	UNIV TOLIMA
MUNOZ, MS	3255	FALSE	UNAM	FALSE	698	1	UNAM
MUNOZ-AND	3257	FALSE	UNAM	FALSE	698	1	UNAM
MUNOZ-LOPE	3261	FALSE	INAOE	FALSE	276	1	INAOE
MUNOZ-SANI	3262	FALSE	UNIV SONOR/	FALSE	987	1	UNIV SONOR/
MURCHADHA	3263	FALSE	UNAM	FALSE	698	1	UNAM
MURGUIA, M	3264	FALSE	UNIV GUANA.	FALSE	823	1	UNIV GUANA.
MURILLO, JG	3266	FALSE	UNAM	FALSE	698	1	UNAM
MURILLO, N	3267	FALSE	UNAM	FALSE	698	1	UNAM
NA, KS	3275	TRUE	BUAP	FALSE	51	1	BUAP
NA, KS	3276	FALSE		FALSE		0	UNAM
NABIEV, RF	3277	TRUE	INAOE	FALSE	276	1	INAOE
NABIEV, RF	3278	TRUE		TRUE		0	PN LEBEDEV F
NABIEV, RF	3279	FALSE		FALSE		0	UNIV CALIF S/
NAGELE, G	3280	TRUE		FALSE		0	UASLP
NAGELE, G	3281	FALSE	UNIV MICHIG	FALSE	887	1	UNIV MICHIG
NAGIYEV, SM	3282	TRUE	AZERBAIJAN /	FALSE	32	1	AZERBAIJAN /
NAGIYEV, SM	3283	FALSE	UNAM	FALSE	698	1	UNAM
NAHMADACH	3284	FALSE		FALSE		0	UNAM
NAHMAD-MC	3285	FALSE	CINVESTAV	FALSE	94	1	CINVESTAV
NAIR, MTS	3286	FALSE		FALSE		0	UNAM
NAIR, PK	3287	FALSE		FALSE		0	UNAM
NAKAMURA,	3288	FALSE	NAGOYA UNI\	FALSE	491	1	NAGOYA UNI\
NAKANISHI, H	3289	TRUE	CICESE	FALSE	89	1	CICESE
NAKANISHI, H	3290	FALSE		FALSE		0	TOHOKU UNI\
NAPOLI, DR	3293	TRUE		FALSE		0	JAGIELLONIAN
NAPOLI, DR	3294	FALSE	TEXAS A&M U	FALSE	663	1	TEXAS A&M U
NAPSUCIALE,	3295	TRUE		FALSE		0	CINVESTAV
NAPSUCIALE,	3296	FALSE	UNIV AUTON(	FALSE	728	1	UNIV AUTON(
NASSER, L	3301	FALSE		FALSE		0	UAM I
NAUMIS, GG	3304	FALSE		FALSE		0	UNAM
NAVA, JJG	3307	TRUE	BUAP	FALSE	51	1	BUAP
NAVA, JJG	3308	FALSE	CINVESTAV	FALSE	94	1	CINVESTAV
NAVA, N	3309	FALSE	IMP	FALSE	272	1	IMP
NAVARRETE, /	3312	FALSE	UNIV AUTON(	FALSE	728	1	UNIV AUTON(
NAVARRETE-(	3318	TRUE	IPN	FALSE	376	1	IPN
NAVARRETE-(	3319	FALSE		FALSE		0	UAM A
NAVARRO, AZ	3320	FALSE	IPN	FALSE	376	1	IPN
NAVARRO, O	3322	FALSE	UNAM	FALSE	698	1	UNAM
NAVARRO-CO	3324	TRUE		FALSE		0	CINVESTAV
NAVARRO-CO	3325	TRUE	UASLP	FALSE	689	1	UASLP

NAVARRO-CO	3326	FALSE	UNIV ILLINOIS	FALSE	840	1	UNIV ILLINOIS
NEBBIA, G	3330	TRUE	HOPE COLL	FALSE	254	1	HOPE COLL
NEBBIA, G	3331	TRUE		TRUE		0	IST NAZL FIS N
NEBBIA, G	3332	FALSE		FALSE		0	Univ Padova
NEBESNY, KW	3333	FALSE		FALSE		0	UNIV SONORA
NEGRETE, J	3335	FALSE	UASLP	FALSE	689	1	UASLP
NEGRETE, MA	3336	FALSE	UNAM	FALSE	698	1	UNAM
NEGRO, J	3338	FALSE	UNIV VALLAD	FALSE	1011	1	UNIV VALLAD
NEGRON-MEN	3339	FALSE	UNAM	FALSE	698	1	UNAM
NELLEN, L	3340	TRUE	UNAM	FALSE	698	1	UNAM
NELLEN, L	3341	FALSE		FALSE		0	UNIV MICHOA
NESTEROV, AI	3343	FALSE		FALSE		0	UNIV GUADAL
NGUYEN, VL	3346	FALSE	UNIV AUTON	FALSE	718	1	UNIV AUTON
NICHO, ME	3348	TRUE	INST INVEST E	FALSE	318	1	INST INVEST E
NICHO, ME	3349	FALSE	UNAM	FALSE	698	1	UNAM
NICOLI, MP	3352	TRUE	Inst Rech Suba	FALSE	344	1	Inst Rech Suba
NICOLI, MP	3353	FALSE		FALSE		0	RUDJER BOSK
NIERMANN, S	3355	TRUE		TRUE		0	CINVESTAV
NIERMANN, S	3356	FALSE		FALSE		0	UNIV IOWA
NIETO, J	3357	TRUE	CICESE	FALSE	89	1	CICESE
NIETO, J	3358	FALSE		FALSE		0	UNIV MICHOA
NIETO, LM	3360	FALSE	UNIV VALLAD	FALSE	1011	1	UNIV VALLAD
NIEVES, JF	3362	FALSE	UNIV PUERTO	FALSE	954	1	UNIV PUERTO
NIEWIEROWI	3363	FALSE	IPN	FALSE	376	1	IPN
NIKL, M	3366	FALSE	INST PHYS	FALSE	337	1	INST PHYS
NIKONOV, DE	3367	TRUE		FALSE		0	PN LEBEDEV P
NIKONOV, DE	3368	FALSE	UNAM	FALSE	698	1	UNAM
NILES, DW	3369	TRUE		FALSE		0	CINVESTAV
NILES, DW	3370	FALSE	UNIV WISCON	FALSE	1027	1	UNIV WISCON
NING, XG	3371	FALSE	UNAM	FALSE	698	1	UNAM
NOGUEZ, C	3380	TRUE		FALSE		0	OHIO UNIV
NOGUEZ, C	3381	FALSE		FALSE		0	UNAM
NOJIRI, S	3382	FALSE		FALSE		0	NATL DEF ACA
NORDMARK, I	3383	FALSE	CINVESTAV	FALSE	94	1	CINVESTAV
NOVARO, O	3387	FALSE		FALSE		0	UNAM
NOVIKOV, IV	3390	TRUE		FALSE		0	UNIV AUTON
NOVIKOV, IV	3391	FALSE	UNIV CALIF IR	FALSE	749	1	UNIV CALIF IR
NOWOTNY, E	3393	FALSE	UAM I	FALSE	686	1	UAM I
NOYOLA, APG	3394	FALSE	UAM I	FALSE	686	1	UAM I
NUCAMENDI,	3395	FALSE		FALSE		0	CINVESTAV
NUNEZ, D	3396	FALSE	UNAM	FALSE	698	1	UNAM
NUNEZ, MA	3398	FALSE		FALSE		0	UAM I
NUNEZ-YEPEZ	3400	TRUE	UAM A	FALSE	685	1	UAM A
NUNEZ-YEPEZ	3401	FALSE		FALSE		0	UAM I
NYIKOS, L	3403	FALSE	LAJOS KOSSUT	FALSE	430	1	LAJOS KOSSUT
OCHOA, NA	3408	FALSE	CTR INVEST O	FALSE	148	1	CTR INVEST O
OCONNOR, D	3409	FALSE		FALSE		0	DUBLIN INST A

ODINTSOV, SI	3412	TRUE	TOMSK STATE	FALSE	672	1	TOMSK STATE
ODINTSOV, SI	3413	FALSE		FALSE		0	UNIV GUANA.
ODRIOZOLA, J	3415	FALSE	UNIV SEVILLA	FALSE	982	1	UNIV SEVILLA
OH, SJ	3422	TRUE		FALSE		0	POHANG UNI'
OH, SJ	3423	FALSE	UNIV AUTON(	FALSE	720	1	UNIV AUTON(
OKTAY, MB	3427	FALSE		FALSE		0	UNIV IOWA
OKUNO, E	3428	FALSE	INST ASTRON	FALSE	297	1	INST ASTRON
OLEA, A	3431	FALSE		FALSE		0	UNAM
OLEA, O	3432	TRUE	ININ	FALSE	289	1	ININ
OLEA, O	3433	FALSE		FALSE		0	UNIV AUTON(
OLGUIN, D	3434	FALSE	CINVESTAV	FALSE	94	1	CINVESTAV
OLGUIN, MT	3436	TRUE	ININ	FALSE	289	1	ININ
OLGUIN, MT	3437	FALSE		FALSE		0	IPN
OLIVA, AI	3438	FALSE	CINVESTAV	FALSE	94	1	CINVESTAV
OLIVARES, JA	3439	FALSE	UNAM	FALSE	698	1	UNAM
OLIVARES, W	3440	FALSE	UNIV LOS ANI	FALSE	875	1	UNIV LOS ANI
OLIVARES-PEI	3441	FALSE	CTR INVEST O	FALSE	148	1	CTR INVEST O
OLIVARES-RO	3442	TRUE		TRUE		0	COLEGIO NAC
OLIVARES-RO	3443	FALSE		FALSE		0	UAM I
OLIVAS, A	3444	TRUE	CICESE	FALSE	89	1	CICESE
OLIVAS, A	3445	FALSE		FALSE		0	UNIV NACL AI
OLIVER, A	3447	FALSE	UNAM	FALSE	698	1	UNAM
OLVERA, MDL	3451	FALSE	CINVESTAV	FALSE	94	1	CINVESTAV
ONDARZA-RO	3457	FALSE		FALSE		0	ININ
ORDEJON, P	3459	TRUE		FALSE		0	UNAM
ORDEJON, P	3460	FALSE		FALSE		0	UNIV OVIEDO
ORDONEZ-RE(	3461	TRUE		FALSE		0	CINVESTAV
ORDONEZ-RE(	3462	TRUE	ININ	FALSE	289	1	ININ
ORDONEZ-RE(	3463	FALSE		FALSE		0	UAM I
OREA, AC	3464	FALSE	CINVESTAV	FALSE	94	1	CINVESTAV
OREA, P	3465	FALSE		FALSE		0	UAM I
ORGAZ, E	3466	TRUE	UNAM	FALSE	698	1	UNAM
ORGAZ, E	3467	FALSE		FALSE		0	UNIV PARIS 1
ORLOV, VG	3469	FALSE	UNAM	FALSE	698	1	UNAM
ORNELAS-ROI	3472	FALSE	CTR INVEST O	FALSE	148	1	CTR INVEST O
OROZCO, S	3474	FALSE		FALSE		0	UNAM
OROZCO-TER/	3475	TRUE		TRUE		0	CINVESTAV
OROZCO-TER/	3476	TRUE		FALSE		0	INST TECNOL
OROZCO-TER/	3477	FALSE	UNAM	FALSE	698	1	UNAM
ORTEGABLAK	3483	FALSE	CSIRO	FALSE	131	1	CSIRO
ORTEGA-LOPI	3484	FALSE	CINVESTAV	FALSE	94	1	CINVESTAV
ORTEGA-MAR	3485	FALSE	PN LEBEDEV F	FALSE	555	1	PN LEBEDEV F
ORTIZ, A	3486	FALSE	UNAM	FALSE	698	1	UNAM
ORTIZ, MA	3488	FALSE	UNAM	FALSE	698	1	UNAM
ORTIZ, ME	3489	TRUE		FALSE		0	LAWRENCE BI
ORTIZ, ME	3490	FALSE	UNAM	FALSE	698	1	UNAM
ORTIZ, U	3493	FALSE	UANL	FALSE	687	1	UANL

ORTIZ-LOPEZ,	3495	FALSE	IPN	FALSE	376	1	IPN
ORTIZSAAVED	3497	TRUE		FALSE		0	FREE UNIV BE
ORTIZSAAVED	3498	TRUE	UASLP	FALSE	689	1	UASLP
ORTIZSAAVED	3499	FALSE		FALSE		0	UAZ
OSEGUERA, J	3502	FALSE	UNAM	FALSE	698	1	UNAM
OVANDO, G	3513	TRUE	IPN	FALSE	376	1	IPN
OVANDO, G	3514	TRUE		TRUE		0	Sri Aurobindo
OVANDO, G	3515	FALSE		FALSE		0	Sri Aurobindo
OVIEDO-ROA,	3516	FALSE	UNAM	FALSE	698	1	UNAM
OZIEWICZ, Z	3518	FALSE		FALSE		0	UNAM
PACHECO, G	3521	TRUE	IPN	FALSE	376	1	IPN
PACHECO, G	3522	FALSE	UNIV WISCON	FALSE	1027	1	UNIV WISCON
PACHECOMAI	3524	TRUE	CINVESTAV	FALSE	94	1	CINVESTAV
PACHECOMAI	3525	FALSE		FALSE		0	UNIV WISCON
PADILLA, A	3527	TRUE	IPN	FALSE	376	1	IPN
PADILLA, A	3528	FALSE		FALSE		0	UNAM
PADILLA, GP	3529	TRUE	CALTECH	FALSE	58	1	CALTECH
PADILLA, GP	3530	FALSE		FALSE		0	CTR INVEST O
PADILLA, P	3531	FALSE		FALSE		0	UNAM
PAEZ, G	3532	TRUE	CSIC	FALSE	130	1	CSIC
PAEZ, G	3533	FALSE		FALSE		0	CTR INVEST O
PAEZ-HERNAI	3534	FALSE	UAM A	FALSE	685	1	UAM A
PAGE, D	3535	FALSE	UNAM	FALSE	698	1	UNAM
PAL, PB	3541	FALSE	UNIV OREGOI	FALSE	925	1	UNIV OREGOI
PAL, U	3542	FALSE		FALSE		0	BUAP
PALACIOS, AF	3543	FALSE	IMP	FALSE	272	1	IMP
PALACIOS, J	3544	TRUE		FALSE		0	IPN
PALACIOS, J	3545	FALSE	UNAM	FALSE	698	1	UNAM
PALMA, A	3551	FALSE	BUAP	FALSE	51	1	BUAP
PALOMARES-!	3555	FALSE	UASLP	FALSE	689	1	UASLP
PALOMINO, R	3556	FALSE	UPAEP	FALSE	1035	1	UPAEP
PALOMINO-O	3557	FALSE	BUAP	FALSE	51	1	BUAP
PALUMMO, M	3558	FALSE	UNIV ROMA T	FALSE	963	1	UNIV ROMA T
PANDO, CL	3563	FALSE		FALSE		0	BUAP
PANKOV, V	3568	FALSE	UNAM	FALSE	698	1	UNAM
PAPENBROCK	3571	TRUE		FALSE		0	MAX PLANCK
PAPENBROCK	3572	FALSE	UNAM	FALSE	698	1	UNAM
PARAGUAY-D	3573	FALSE		FALSE		0	CTR INVEST M
PARDO, F	3574	FALSE	UNAM	FALSE	698	1	UNAM
PAREDES, R	3576	FALSE		FALSE		0	UNAM
PARIS, MA	3579	TRUE		FALSE		0	CSIC
PARIS, MA	3580	FALSE	UANL	FALSE	687	1	UANL
PARMANAND	3583	TRUE		FALSE		0	UAM I
PARMANAND	3584	TRUE	UNAM	FALSE	698	1	UNAM
PARMANAND	3585	FALSE		FALSE		0	UNIV AUTON(
PASTOR, GM	3590	TRUE		FALSE		0	FLORIDA STAT
PASTOR, GM	3591	TRUE	INST PHYS & (	FALSE	338	1	INST PHYS & (

PASTOR, GM	3592	TRUE		TRUE		0	NATL HIGH M
PASTOR, GM	3593	TRUE	UNIV AUTON(	FALSE	727	1	UNIV AUTON(
PASTOR, GM	3594	FALSE		FALSE		0	UNIV TOULOU
PASTRANA-SA	3595	TRUE	BUAP	FALSE	51	1	BUAP
PASTRANA-SA	3596	FALSE		FALSE		0	CTR INVEST O
PATRYKIEJEW	3598	FALSE		FALSE		0	MARIE CURIE
PAXTON, AH	3602	FALSE		FALSE		0	UASLP
PEDRAYES, M	3609	FALSE	UNAM	FALSE	698	1	UNAM
PEDRAZA, F	3610	FALSE	UNAM	FALSE	698	1	UNAM
PEDRERO, E	3614	TRUE		FALSE		0	IPN
PEDRERO, E	3615	FALSE	UNAM	FALSE	698	1	UNAM
PEDRINI, G	3616	FALSE	UNIV STUTTG	FALSE	991	1	UNIV STUTTG
PEDROZA-MC	3617	FALSE	CINVESTAV	FALSE	94	1	CINVESTAV
PELAIZ-BARRA	3619	FALSE	UNAM	FALSE	698	1	UNAM
PENA, I	3623	FALSE	UNAM	FALSE	698	1	UNAM
PENA, JJ	3624	FALSE	UAM A	FALSE	685	1	UAM A
PENA, JL	3625	FALSE	UASLP	FALSE	689	1	UASLP
PENA, MT	3626	FALSE	IPN	FALSE	376	1	IPN
PENA, P	3627	TRUE	UNAM	FALSE	698	1	UNAM
PENA, P	3628	FALSE		FALSE		0	UNIV MONTP
PENELLE, R	3632	TRUE		FALSE		0	CENS
PENELLE, R	3633	FALSE	UNIV PARIS 1	FALSE	939	1	UNIV PARIS 1
PENTON, A	3635	FALSE	CNR INST	FALSE	102	1	CNR INST
PERALTA-FAB	3637	FALSE	UNAM	FALSE	698	1	UNAM
PEREYRA, I	3643	TRUE	CINVESTAV	FALSE	94	1	CINVESTAV
PEREYRA, I	3644	FALSE		FALSE		0	UNIV SAO PAI
PEREZ, G	3649	FALSE		FALSE		0	CINVESTAV
PEREZ, LA	3654	TRUE	CNRS	FALSE	103	1	CNRS
PEREZ, LA	3655	FALSE		FALSE		0	UNAM
PEREZ-ALVAR	3664	FALSE	UAZ	FALSE	690	1	UAZ
PEREZ-BENITE	3666	FALSE	UNAM	FALSE	698	1	UNAM
PEREZ-BERNA	3667	TRUE	UNAM	FALSE	698	1	UNAM
PEREZ-BERNA	3668	FALSE		FALSE		0	UNIV SEVILLA
PEREZ-BUENC	3669	TRUE	CINVESTAV	FALSE	94	1	CINVESTAV
PEREZ-BUENC	3670	FALSE		FALSE		0	UAQ
PEREZ-CASER(	3671	FALSE	UNAM	FALSE	698	1	UNAM
PEREZESTEVA	3673	FALSE	UNAM	FALSE	698	1	UNAM
PEREZGARCIA	3674	TRUE	UNAM	FALSE	698	1	UNAM
PEREZGARCIA	3675	FALSE		FALSE		0	UNIV AUTON(
PEREZ-GONZA	3676	FALSE	BUAP	FALSE	51	1	BUAP
PEREZ-GONZA	3677	TRUE	IPN	FALSE	376	1	IPN
PEREZ-GONZA	3678	FALSE		FALSE		0	UAM A
PEREZ-LOPEZ,	3681	TRUE		FALSE		0	CTR INVEST O
PEREZ-LOPEZ,	3682	FALSE	HERIOT WATT	FALSE	250	1	HERIOT WATT
PEREZ-LOPEZ,	3683	FALSE	UNIV GUADAI	FALSE	822	1	UNIV GUADAI
PEREZ-LOREN	3685	FALSE		FALSE		0	CINVESTAV
PEREZ-ORTA,	3687	TRUE		FALSE		0	UASLP

PEREZ-ORTA,	3688	FALSE	UNIV HAVAN/	FALSE	831	1	UNIV HAVAN/
PEREZRAMIRE	3691	FALSE		FALSE		0	UNAM
PEREZ-ROBLE:	3692	TRUE		TRUE		0	CINVESTAV
PEREZ-ROBLE:	3693	TRUE		TRUE		0	INST TECNOL
PEREZ-ROBLE:	3694	TRUE	UAQ	FALSE	688	1	UAQ
PEREZ-ROBLE:	3695	FALSE		FALSE		0	UASLP
PEREZ-RODRI(	3696	FALSE	BUAP	FALSE	51	1	BUAP
PEREZ-SALAS,	3697	TRUE		TRUE		0	CICESE
PEREZ-SALAS,	3698	TRUE		TRUE		0	UNAM
PEREZ-SALAS,	3699	FALSE		FALSE		0	UNIV SONOR/
PEREZ-TREJO,	3700	FALSE		FALSE		0	IPN
PESTIEAU, J	3704	FALSE	CATHOLIC UN	FALSE	64	1	CATHOLIC UN
PETER, A	3705	FALSE	HUNGARIAN /	FALSE	260	1	HUNGARIAN /
PETERSON, D	3706	TRUE	HOPE COLL	FALSE	254	1	HOPE COLL
PETERSON, D	3707	TRUE	ININ	FALSE	289	1	ININ
PETERSON, D	3708	FALSE		FALSE		0	UNIV MICHIG
PETRANOVSK	3709	TRUE		TRUE		0	CICESE
PETRANOVSK	3710	TRUE		FALSE		0	UNAM
PETRANOVSK	3711	TRUE	UNIV AUTON(	FALSE	718	1	UNIV AUTON(
PETRANOVSK	3712	FALSE		FALSE		0	UNIV NACL AI
PEYKOV, P	3720	TRUE		TRUE		0	BUAP
PEYKOV, P	3721	FALSE		FALSE		0	INAOE
PFEIFFER, H	3722	TRUE	ININ	FALSE	289	1	ININ
PFEIFFER, H	3723	FALSE		FALSE		0	UAM I
PIANETTA, P	3725	TRUE		TRUE		0	CINVESTAV
PIANETTA, P	3726	TRUE		FALSE		0	STANFORD UI
PIANETTA, P	3727	FALSE	UAQ	FALSE	688	1	UAQ
PICCINELLI, G	3728	FALSE	UNIV PADUA	FALSE	935	1	UNIV PADUA
PICHARD, JL	3729	TRUE	CENS	FALSE	73	1	CENS
PICHARD, JL	3730	FALSE	UNAM	FALSE	698	1	UNAM
PICHARDO, JL	3731	FALSE		FALSE		0	CINVESTAV
PICK, S	3732	FALSE	ACAD SCI CZE	FALSE	6	1	ACAD SCI CZE
PICOS-VEGA, .	3734	TRUE		TRUE		0	CINVESTAV
PICOS-VEGA, .	3735	TRUE		FALSE		0	UNAM
PICOS-VEGA, .	3736	FALSE	UNIV SONOR/	FALSE	987	1	UNIV SONOR/
PICQUART, M	3737	FALSE	UAM I	FALSE	686	1	UAM I
PIMENTEL, LC	3742	FALSE	UAM I	FALSE	686	1	UAM I
PINAR, FC	3747	FALSE	CINVESTAV	FALSE	94	1	CINVESTAV
PINA-VILLALP	3748	FALSE	ININ	FALSE	289	1	ININ
PINCIK, E	3750	FALSE	SLOVAK ACAD	FALSE	613	1	SLOVAK ACAD
PINTO-ROBLE	3754	TRUE		FALSE		0	UNAM
PINTO-ROBLE	3755	FALSE	UNIV ESSEX	FALSE	790	1	UNIV ESSEX
PIQUERAS, J	3756	TRUE		FALSE		0	BUAP
PIQUERAS, J	3757	FALSE	UNIV AUTOM	FALSE	716	1	UNIV AUTOM
PITA-LARRAN.	3759	FALSE		FALSE		0	UNAM
PITANGA, P	3760	TRUE	UAM I	FALSE	686	1	UAM I
PITANGA, P	3761	FALSE		FALSE		0	UNED

PITERS, TM	3762	TRUE	UNIV SAO PAU	FALSE	980	1	UNIV SAO PAU
PITERS, TM	3763	FALSE		FALSE		0	UNIV SONORA
PITTEL, S	3764	TRUE	UNAM	FALSE	698	1	UNAM
PITTEL, S	3765	TRUE	UNIV DELAWA	FALSE	781	1	UNIV DELAWA
PITTEL, S	3766	FALSE	YALE UNIV	FALSE	1065	1	YALE UNIV
PIZIO, OA	3767	TRUE		FALSE		0	MARIE CURIE
PIZIO, OA	3768	TRUE	NATL ACAD SC	FALSE	500	1	NATL ACAD SC
PIZIO, OA	3769	FALSE		FALSE		0	UNAM
PLASCHKO, P	3770	FALSE	UAM I	FALSE	686	1	UAM I
PLATA, P	3774	FALSE		FALSE		0	UNAM
PLESCH, G	3777	FALSE	COMENIUS UI	FALSE	114	1	COMENIUS UI
POLLNEY, D	3783	TRUE	QUEENS UNIV	FALSE	572	1	QUEENS UNIV
POLLNEY, D	3784	FALSE		FALSE		0	UNIV MICHOA
POLTEV, VI	3785	FALSE	RUSSIAN ACA	FALSE	587	1	RUSSIAN ACA
POLUPAN, GP	3786	TRUE	IPN	FALSE	376	1	IPN
POLUPAN, GP	3787	FALSE		FALSE		0	NATL ACAD SC
PONCE, L	3790	FALSE	UAM I	FALSE	686	1	UAM I
PONCE, MA	3791	FALSE	SURFACE OPT	FALSE	639	1	SURFACE OPT
PONCE, WA	3792	FALSE	INT CTR THEO	FALSE	369	1	INT CTR THEO
PONCE-CASTA	3793	FALSE	UASLP	FALSE	689	1	UASLP
PONIATOWSK	3794	FALSE	UAM A	FALSE	685	1	UAM A
PONS, J	3796	FALSE		FALSE		0	UASLP
POPOV, N	3799	FALSE		FALSE		0	UNIV MUNICH
PORCAYO-CA	3802	FALSE		FALSE		0	INST INVEST E
PORTE, H	3803	FALSE		FALSE		0	UNIV FRANCH
PORTELLES, JJ	3804	TRUE		TRUE		0	UNAM
PORTELLES, JJ	3805	TRUE		TRUE		0	Univ de Orien
PORTELLES, JJ	3806	TRUE		FALSE		0	UNIV HAVANA
PORTELLES, JJ	3807	TRUE		TRUE		0	UNIV LA HABA
PORTELLES, JJ	3808	FALSE		FALSE		0	UNIV ORIENTE
PORTILLA, M	3809	FALSE	UNAM	FALSE	698	1	UNAM
POSADA-AMA	3810	TRUE	CICESE	FALSE	89	1	CICESE
POSADA-AMA	3811	FALSE		FALSE		0	UNIV SONORA
POULAIN, E	3813	FALSE	UNAM	FALSE	698	1	UNAM
POURROY, G	3815	TRUE		FALSE		0	INST PHYS & C
POURROY, G	3816	FALSE	UNAM	FALSE	698	1	UNAM
PRETE, G	3819	FALSE	UNAM	FALSE	698	1	UNAM
PROKHOROV,	3824	TRUE		FALSE		0	CINVESTAV
PROKHOROV,	3825	FALSE	UAQ	FALSE	688	1	UAQ
PRUTSKIJ, TA	3826	FALSE	BUAP	FALSE	51	1	BUAP
PRZANOWSKI	3827	FALSE	TECH UNIV LC	FALSE	654	1	TECH UNIV LC
PUEBLA, H	3829	TRUE		FALSE		0	IPN
PUEBLA, H	3830	FALSE		FALSE		0	UAM I
PUENTE, A	3831	TRUE	SUNY BUFFAL	FALSE	637	1	SUNY BUFFAL
PUENTE, A	3832	FALSE		FALSE		0	UNIV ILLES BA
PUENTE, GC	3833	FALSE	IPN	FALSE	376	1	IPN
PUIG, JE	3835	TRUE	IMP	FALSE	272	1	IMP

PUIG, JE	3836	FALSE		FALSE		0	UNIV AKRON
PULLIN, J	3837	FALSE	UNIV UTAH	FALSE	1009	1	UNIV UTAH
PURON, E	3838	FALSE	UNIV HAVAN/	FALSE	831	1	UNIV HAVAN/
QUESADA, D	3843	TRUE	CINVESTAV	FALSE	94	1	CINVESTAV
QUESADA, D	3844	FALSE		FALSE		0	UNIV LA HAB/
QUEVEDO, F	3846	TRUE		FALSE		0	SHIRAZ UNIV
QUEVEDO, F	3847	FALSE	UNAM	FALSE	698	1	UNAM
QUEVEDO, H	3848	TRUE		FALSE		0	POLISH ACAD
QUEVEDO, H	3849	FALSE	UNAM	FALSE	698	1	UNAM
QUEVEDO-LO	3850	TRUE		TRUE		0	UNIV N TEXAS
QUEVEDO-LO	3851	FALSE		FALSE		0	UNIV SONOR/
QUEZADA, J	3852	FALSE	UNIV GRENOI	FALSE	820	1	UNIV GRENOI
QUICK, RM	3854	TRUE		FALSE		0	AZERBAIJAN /
QUICK, RM	3855	FALSE	IPN	FALSE	376	1	IPN
QUINTANA, J	3857	FALSE	UNAM	FALSE	698	1	UNAM
QUINTANA, P	3858	FALSE	UNIV ABERDE	FALSE	706	1	UNIV ABERDE
QUIRINO, GS(	3861	FALSE	INAOE	FALSE	276	1	INAOE
QUIROGA, JA	3862	TRUE	CIMAT	FALSE	93	1	CIMAT
QUIROGA, JA	3863	FALSE		FALSE		0	CTR INVEST O
QUIROGA, L	3864	TRUE	CINVESTAV	FALSE	94	1	CINVESTAV
QUIROGA, L	3865	FALSE	UNIV LOS ANI	FALSE	875	1	UNIV LOS ANI
QUIROZ, MA	3866	FALSE		FALSE		0	UDLAP
RAGA, AC	3872	FALSE	UNAM	FALSE	698	1	UNAM
RAMIREZ, C	3879	FALSE		FALSE		0	BUAP
RAMIREZ, E	3880	FALSE		FALSE		0	UNAM
RAMIREZ, F	3882	FALSE	CICESE	FALSE	89	1	CICESE
RAMIREZ, G	3884	FALSE	UDLAP	FALSE	693	1	UDLAP
RAMIREZ, JC	3886	FALSE	UAM I	FALSE	686	1	UAM I
RAMIREZ-BOI	3888	TRUE		TRUE		0	CINVESTAV
RAMIREZ-BOI	3889	FALSE		FALSE		0	UNIV SONOR/
RAMIREZ-FLO	3891	TRUE	UASLP	FALSE	689	1	UASLP
RAMIREZ-FLO	3892	FALSE		FALSE		0	UNIV ILLINOIS
RAMIREZ-NIN	3893	FALSE	INST INVEST E	FALSE	318	1	INST INVEST E
RAMIREZ-RAY	3894	TRUE	CINVESTAV	FALSE	94	1	CINVESTAV
RAMIREZ-RAY	3895	FALSE		FALSE		0	ININ
RAMIREZSAN	3897	TRUE	NORTHEASTEI	FALSE	529	1	NORTHEASTEI
RAMIREZSAN	3898	FALSE	UNAM	FALSE	698	1	UNAM
RAMIREZSOLI	3900	FALSE	UNIV TOULOU	FALSE	1004	1	UNIV TOULOU
RAMOS, E	3903	FALSE	UNAM	FALSE	698	1	UNAM
RAMOS, FE	3904	FALSE	UAM I	FALSE	686	1	UAM I
RAMOS, S	3906	FALSE	UNAM	FALSE	698	1	UNAM
RAMOS-BERN	3907	FALSE	UNAM	FALSE	698	1	UNAM
RAMOS-GARC	3910	FALSE	INAOE	FALSE	276	1	INAOE
RAMOS-MENI	3912	FALSE	UNIV SONOR/	FALSE	987	1	UNIV SONOR/
RANGARAJAN	3914	TRUE		TRUE		0	UNAM
RANGARAJAN	3915	FALSE		FALSE		0	UNIV CONNE(
RANGEL, R	3917	FALSE	CICESE	FALSE	89	1	CICESE

RANGEL-HUEI	3918	FALSE	UAM I	FALSE	686	1	UAM I
RANGEL-ROJC	3919	TRUE	CICESE	FALSE	89	1	CICESE
RANGEL-ROJC	3920	FALSE		FALSE		0	NATL INST MA
RAYCES, J	3927	TRUE	J L RAYCES CC	FALSE	388	1	J L RAYCES CC
RAYCES, J	3928	FALSE		FALSE		0	UNAM
RAYCES, JL	3929	FALSE		FALSE		0	UNAM
REBOIRO, M	3930	TRUE	NATL UNIV LA	FALSE	519	1	NATL UNIV LA
REBOIRO, M	3931	FALSE		FALSE		0	UNAM
RECAMIER, J	3934	FALSE	UNAM	FALSE	698	1	UNAM
RECHTMAN, F	3935	FALSE	UNAM	FALSE	698	1	UNAM
REDNER, S	3936	FALSE		FALSE		0	BOSTON UNIV
REGALADO, LI	3937	FALSE	UNIV SONORA	FALSE	987	1	UNIV SONORA
REGUERA, E	3939	TRUE		TRUE		0	CINVESTAV
REGUERA, E	3940	TRUE		TRUE		0	IPN
REGUERA, E	3941	FALSE		FALSE		0	NATL CTR SCI
REICH, I	3943	TRUE	CINVESTAV	FALSE	94	1	CINVESTAV
REICH, I	3944	FALSE		FALSE		0	UNIV LA HABA
REIGADA, R	3946	TRUE		FALSE		0	UNIV BARCEL
REIGADA, R	3947	FALSE	UNIV CALIF SA	FALSE	752	1	UNIV CALIF SA
RENERO, C	3954	FALSE		FALSE		0	UNAM
RENEROCARR	3955	FALSE		FALSE		0	INAOE
RETAMAL, JC	3958	TRUE		TRUE		0	UNIV GUADAI
RETAMAL, JC	3959	TRUE		TRUE		0	UNIV SANTIAC
RETAMAL, JC	3960	FALSE		FALSE		0	UNIV SANTIAC
REYES, JA	3962	FALSE	UNAM	FALSE	698	1	UNAM
REYES, M	3963	TRUE	CINVESTAV	FALSE	94	1	CINVESTAV
REYES, M	3964	FALSE		FALSE		0	INST GRAVITA
REYES, MA	3965	TRUE		TRUE		0	INST GRAVITA
REYES, MA	3966	TRUE		TRUE		0	ITESM
REYES, MA	3967	TRUE		FALSE		0	UNIV GUANA.
REYES, MA	3968	FALSE	UNIV WARSAV	FALSE	1023	1	UNIV WARSAV
REYES, PG	3970	FALSE		FALSE		0	UNAM
REYES-DE-RUI	3971	TRUE	UAQ	FALSE	688	1	UAQ
REYES-DE-RUI	3972	FALSE		FALSE		0	UNAM
REYES-GASGA	3974	TRUE		FALSE		0	INST ELECTR C
REYES-GASGA	3975	FALSE		FALSE		0	UNAM
REYES-GOMEZ	3976	FALSE	UNIV COLIMA	FALSE	770	1	UNIV COLIMA
REYES-NAVA,	3977	TRUE		FALSE		0	CINVESTAV
REYES-NAVA,	3978	FALSE	UNAM	FALSE	698	1	UNAM
REYES-SERRAT	3979	FALSE	UNAM	FALSE	698	1	UNAM
RIBEIRO, E	3982	TRUE	TELEBRAS CPC	FALSE	659	1	TELEBRAS CPC
RIBEIRO, E	3983	FALSE		FALSE		0	UNIV ESTADU
RICHERT, J	3985	FALSE		FALSE		0	UNAM
RICKARDS, J	3986	FALSE	UNAM	FALSE	698	1	UNAM
RIECH, I	3987	TRUE		TRUE		0	CINVESTAV
RIECH, I	3988	FALSE		FALSE		0	UNIV LA HABA
RIERA, R	3989	FALSE		FALSE		0	UNIV SONORA

RIGO, A	3990	TRUE	UNAM	FALSE	698	1	UNAM
RIGO, A	3991	TRUE	UNIV ILLES BA	FALSE	839	1	UNIV ILLES BA
RIGO, A	3992	FALSE		FALSE		0	UNIV PRETOR
RINCON, JM	3994	TRUE	CICESE	FALSE	89	1	CICESE
RINCON, JM	3995	FALSE		FALSE		0	CSIC
RINCON, ME	3996	FALSE		FALSE		0	UNAM
RIOS, M	3998	TRUE	CINVESTAV	FALSE	94	1	CINVESTAV
RIOS, M	3999	TRUE		TRUE		0	UNAM
RIOS, M	4000	FALSE		FALSE		0	UNIV MICHOA
RIOS, S	4001	FALSE		FALSE		0	UNIV SANTIAG
RIOS-JARA, D	4002	FALSE	UNAM	FALSE	698	1	UNAM
RIQUER, V	4005	FALSE		FALSE		0	UNAM
RIVAS-SILVA, .	4009	TRUE	BRIGHAM YOU	FALSE	46	1	BRIGHAM YOU
RIVAS-SILVA, .	4010	FALSE		FALSE		0	BUAP
RIVERA, AL	4011	FALSE	UNAM	FALSE	698	1	UNAM
RIVERA, E	4012	FALSE		FALSE		0	CICESE
RIVERA, M	4016	FALSE		FALSE		0	CTR INVEST O
RIVERA, T	4018	FALSE		FALSE		0	UAM I
RIVERAGASTE	4020	FALSE		FALSE		0	UNIV GUADAL
RIVEROS, HG	4022	TRUE	UNAM	FALSE	698	1	UNAM
RIVEROS, HG	4023	FALSE	UNIV SONORA	FALSE	987	1	UNIV SONORA
ROA-NERI, JAI	4025	TRUE		FALSE		0	UAM A
ROA-NERI, JAI	4026	FALSE	UAM I	FALSE	686	1	UAM I
ROBLEDO-SAN	4030	TRUE		TRUE		0	BUAP
ROBLEDO-SAN	4031	FALSE		FALSE		0	UNAM
ROBLES, J	4032	FALSE	UAM I	FALSE	686	1	UAM I
ROBLESVAZQU	4039	FALSE		FALSE		0	UNAM
ROCHA-MART	4042	TRUE		FALSE		0	IPN
ROCHA-MART	4043	FALSE	UAM A	FALSE	685	1	UAM A
RODE, AV	4044	TRUE	AUSTRALIAN I	FALSE	30	1	AUSTRALIAN I
RODE, AV	4045	FALSE	UAM I	FALSE	686	1	UAM I
RODRIGUEZ, A	4049	TRUE		FALSE		0	BUAP
RODRIGUEZ, A	4050	FALSE	INAOE	FALSE	276	1	INAOE
RODRIGUEZ, F	4054	TRUE	UNAM	FALSE	698	1	UNAM
RODRIGUEZ, F	4055	FALSE	UNIV AUTON(	FALSE	725	1	UNIV AUTON(
RODRIGUEZ, J	4060	TRUE		TRUE		0	CICESE
RODRIGUEZ, J	4061	TRUE		TRUE		0	CINVESTAV
RODRIGUEZ, J	4062	FALSE		FALSE		0	UNAM
RODRIGUEZ, I	4065	FALSE		FALSE		0	BUAP
RODRIGUEZ, I	4066	FALSE		FALSE		0	CINVESTAV
RODRIGUEZ, F	4067	TRUE		FALSE		0	AF IOFFE PHYS
RODRIGUEZ, F	4068	FALSE	INAOE	FALSE	276	1	INAOE
RODRIGUEZ, F	4069	FALSE	UAM I	FALSE	686	1	UAM I
RODRIGUEZ, Y	4074	TRUE	CINVESTAV	FALSE	94	1	CINVESTAV
RODRIGUEZ, Y	4075	TRUE		FALSE		0	UNIV HAVANA
RODRIGUEZ, Y	4076	FALSE	UNIV LA HABA	FALSE	855	1	UNIV LA HABA
RODRIGUEZ-J.	4083	FALSE		FALSE		0	UNAM

RODRIGUEZ-L	4084	FALSE	UASLP	FALSE	689	1	UASLP
RODRIGUEZ-L	4085	FALSE	UNAM	FALSE	698	1	UNAM
RODRIGUEZ-M	4086	FALSE	UNIV SONORA	FALSE	987	1	UNIV SONORA
RODRIGUEZ-R	4088	TRUE		TRUE		0	UNAM
RODRIGUEZ-R	4089	FALSE		FALSE		0	UNIV KONSTA
RODRIGUEZTA	4090	FALSE	UAM I	FALSE	686	1	UAM I
RODRIGUEZ-V	4091	FALSE	CTR INVEST O	FALSE	148	1	CTR INVEST O
RODRIGUEZ-Z	4092	FALSE	BUAP	FALSE	51	1	BUAP
ROJAS, E	4103	TRUE	CINVESTAV	FALSE	94	1	CINVESTAV
ROJAS, E	4104	FALSE		FALSE		0	UNAM
ROJAS, F	4105	FALSE	UAM A	FALSE	685	1	UAM A
ROJAS, HP	4106	TRUE	CINVESTAV	FALSE	94	1	CINVESTAV
ROJAS, HP	4107	TRUE		FALSE		0	UNIV GUANA.
ROJAS, HP	4108	FALSE		FALSE		0	UNIV HELSINK
ROJAS-LOPEZ,	4112	FALSE		FALSE		0	UASLP
ROJO, O	4114	TRUE		FALSE		0	CINVESTAV
ROJO, O	4115	FALSE	IPN	FALSE	376	1	IPN
ROMAN, L	4118	FALSE	UNAM	FALSE	698	1	UNAM
ROMAN-VELA	4122	FALSE	UNAM	FALSE	698	1	UNAM
ROMERO, D	4126	FALSE	UNAM	FALSE	698	1	UNAM
ROMERO, J	4127	TRUE	UNIV AUTONO	FALSE	727	1	UNIV AUTONO
ROMERO, J	4128	FALSE		FALSE		0	UNIV GUADAL
ROMERO, JJ	4129	FALSE	UAM I	FALSE	686	1	UAM I
ROMERO, JL	4130	FALSE	UNIV GUANA.	FALSE	823	1	UNIV GUANA.
ROMERO, RPM	4133	FALSE		FALSE		0	UNAM
ROMERO-BAS	4136	FALSE	UAM I	FALSE	686	1	UAM I
ROMERO-ROC	4138	FALSE	UNAM	FALSE	698	1	UNAM
ROMERO-SAL	4139	FALSE		FALSE		0	UAM I
ROMEU, D	4141	FALSE	UNAM	FALSE	698	1	UNAM
ROMO, R	4142	FALSE		FALSE		0	CICESE
RONNOW, D	4145	FALSE	PAUL DRUDE	FALSE	551	1	PAUL DRUDE
ROPKE, G	4147	FALSE	UNIV ROSTOC	FALSE	966	1	UNIV ROSTOC
ROSADO, A	4150	FALSE	CINVESTAV	FALSE	94	1	CINVESTAV
ROSALES, JJ	4151	TRUE	UAM I	FALSE	686	1	UAM I
ROSALES, JJ	4152	FALSE		FALSE		0	UNIV GUANA.
ROSALES, MA	4153	TRUE	IPN	FALSE	376	1	IPN
ROSALES, MA	4154	FALSE	UDLAP	FALSE	693	1	UDLAP
ROSALES, MI	4155	TRUE	INST INVEST E	FALSE	318	1	INST INVEST E
ROSALES, MI	4156	FALSE		FALSE		0	UNAM
ROSARIO, G	4157	TRUE		FALSE		0	UNIV LA HABA
ROSARIO, G	4158	FALSE	Univ Moa	FALSE	894	1	Univ Moa
ROSAS, E	4159	TRUE	CTR INVEST O	FALSE	148	1	CTR INVEST O
ROSAS, E	4160	FALSE		FALSE		0	IMPERIAL COL
ROSAS, G	4161	TRUE		FALSE		0	UNAM
ROSAS, G	4162	FALSE	UNIV MICHOA	FALSE	888	1	UNIV MICHOA
ROSAS, R	4163	FALSE	UNIV SONORA	FALSE	987	1	UNIV SONORA
ROSAS, RA	4164	FALSE	UNIV SONORA	FALSE	987	1	UNIV SONORA

ROSAS-ORTIZ,	4165	FALSE		FALSE		0	CINVESTAV
ROSENDO, E	4169	FALSE		FALSE		0	UASLP
ROSETE, C	4171	FALSE	UAM I	FALSE	686	1	UAM I
ROSETE-AGUI	4172	FALSE		FALSE		0	UNAM
ROSU, HC	4173	FALSE		FALSE		0	UNIV GUANA.
ROTTER, I	4177	FALSE	ZENT INST KEI	FALSE	1069	1	ZENT INST KEI
ROUHANI, BD	4178	FALSE	BUAP	FALSE	51	1	BUAP
ROUSSEAU, P	4179	TRUE	CINVESTAV	FALSE	94	1	CINVESTAV
ROUSSEAU, P	4180	FALSE	UAQ	FALSE	688	1	UAQ
ROVELLI, C	4181	TRUE	UAM I	FALSE	686	1	UAM I
ROVELLI, C	4182	FALSE		FALSE		0	UNIV PITTSBU
ROVERSI, JA	4183	TRUE		FALSE		0	INAOE
ROVERSI, JA	4184	FALSE	UNIV ESTADU	FALSE	792	1	UNIV ESTADU
ROVIRA, C	4185	TRUE		FALSE		0	CSIC
ROVIRA, C	4186	TRUE	INST CIENCIA	FALSE	304	1	INST CIENCIA
ROVIRA, C	4187	FALSE		FALSE		0	UNIV BARCEL
RUBIN-FALFA	4195	TRUE		FALSE		0	BUAP
RUBIN-FALFA	4196	FALSE	CINVESTAV	FALSE	94	1	CINVESTAV
RUBIO, A	4197	FALSE	UNIV AUTON	FALSE	718	1	UNIV AUTON
RUBIO-PONCI	4202	TRUE		FALSE		0	IPN
RUBIO-PONCI	4203	FALSE	LOS ALAMOS	FALSE	449	1	LOS ALAMOS
RUBO, YG	4204	TRUE		TRUE		0	IPN
RUBO, YG	4205	FALSE		FALSE		0	UNAM
RUELASMAYC	4206	FALSE	UNAM	FALSE	698	1	UNAM
RUFFO, S	4207	FALSE	IST NAZL FIS N	FALSE	383	1	IST NAZL FIS N
RUIZ, E	4209	TRUE		FALSE		0	CINVESTAV
RUIZ, E	4210	FALSE	UNIV SALAMA	FALSE	971	1	UNIV SALAMA
RUIZ, F	4211	TRUE		TRUE		0	CINVESTAV
RUIZ, F	4212	FALSE		FALSE		0	ENERGY CONV
RUIZ-CHAVAR	4216	TRUE	ECOLE NORM.	FALSE	189	1	ECOLE NORM.
RUIZ-CHAVAR	4217	FALSE		FALSE		0	UNAM
RUIZ-ESTRAD	4218	FALSE	CINVESTAV	FALSE	94	1	CINVESTAV
RUIZ-GARCIA,	4219	TRUE		FALSE		0	UASLP
RUIZ-GARCIA,	4220	FALSE	UNAM	FALSE	698	1	UNAM
RUIZMEJIA, C	4222	FALSE		FALSE		0	UNAM
RUIZ-SUAREZ,	4224	FALSE	CINVESTAV	FALSE	94	1	CINVESTAV
RUIZ-TREJO, E	4225	FALSE	UNAM	FALSE	698	1	UNAM
RUPP, T	4227	TRUE		FALSE		0	MAX PLANCK
RUPP, T	4228	FALSE	UNIV STRASB	FALSE	990	1	UNIV STRASB
RYAN, M	4233	TRUE		TRUE		0	UNIV TEXAS
RYAN, M	4234	FALSE		FALSE		0	UNIV UTAH
SAAVEDRA, C	4237	FALSE		FALSE		0	CONCEPCION
SABINA, FJ	4239	FALSE	UNAM	FALSE	698	1	UNAM
SABININ, LV	4240	FALSE	UNIV MICHOA	FALSE	888	1	UNIV MICHOA
SAENZ, A	4241	TRUE	UAQ	FALSE	688	1	UAQ
SAENZ, A	4242	FALSE		FALSE		0	UNAM
SAGAR, RP	4243	TRUE		TRUE		0	QUEENS UNIV

SAGAR, RP	4244	TRUE		FALSE		0	UAM I
SAGAR, RP	4245	FALSE	UNIV ROORKE	FALSE	965	1	UNIV ROORKE
SAHU, S	4247	FALSE		FALSE		0	UNAM
SALAS, FH	4249	TRUE	ITESM	FALSE	385	1	ITESM
SALAS, FH	4250	FALSE		FALSE		0	UNIV AUTON(
SALAS, RP	4254	FALSE		FALSE		0	UNIV SONOR/
SALAS-BRITO,	4255	TRUE	UAM A	FALSE	685	1	UAM A
SALAS-BRITO,	4256	FALSE		FALSE		0	UNAM
SALAZAR, D	4259	TRUE		FALSE		0	CICESE
SALAZAR, D	4260	FALSE	INST CIENCIAS	FALSE	305	1	INST CIENCIAS
SALAZAR-HER	4269	TRUE		FALSE		0	CINVESTAV
SALAZAR-HER	4270	FALSE		FALSE		0	UASLP
SALGADO, M	4276	FALSE		FALSE		0	UNAM
SALINASRODF	4279	FALSE		FALSE		0	CINVESTAV
SALINAS-ROD	4280	FALSE	UAM I	FALSE	686	1	UAM I
SAMANO, EC	4284	TRUE		FALSE		0	CICESE
SAMANO, EC	4285	FALSE	UNAM	FALSE	698	1	UNAM
SAMPAYO, O/	4288	TRUE		TRUE		0	CINVESTAV
SAMPAYO, O/	4289	FALSE		FALSE		0	UNIV CALIF IR
SANABRIA-GC	4290	TRUE	CINVESTAV	FALSE	94	1	CINVESTAV
SANABRIA-GC	4291	TRUE		TRUE		0	UAM I
SANABRIA-GC	4292	FALSE		FALSE		0	UNIV SALAM/
SANCHEZ, A	4294	FALSE	CINVESTAV	FALSE	94	1	CINVESTAV
SANCHEZ, CIR	4298	FALSE	INAOE	FALSE	276	1	INAOE
SANCHEZ, E	4299	FALSE	IMP	FALSE	272	1	IMP
SANCHEZ, GT	4300	TRUE		FALSE		0	CINVESTAV
SANCHEZ, GT	4301	FALSE	UNIV TOLIMA	FALSE	1001	1	UNIV TOLIMA
SANCHEZ, JA	4303	FALSE		FALSE		0	UNIV AUTON(
SANCHEZ, JL	4305	FALSE	CINVESTAV	FALSE	94	1	CINVESTAV
SANCHEZ, JM	4306	TRUE	CINVESTAV	FALSE	94	1	CINVESTAV
SANCHEZ, JM	4307	FALSE		FALSE		0	COLUMBIA UI
SANCHEZ, M	4308	TRUE	CINVESTAV	FALSE	94	1	CINVESTAV
SANCHEZ, M	4309	FALSE	UNIV HAVAN/	FALSE	831	1	UNIV HAVAN/
SANCHEZ, O	4310	FALSE	UNIV AUTON(	FALSE	727	1	UNIV AUTON(
SANCHEZ, V	4314	TRUE		FALSE		0	ININ
SANCHEZ, V	4315	FALSE	UNIV TOKYO	FALSE	999	1	UNIV TOKYO
SANCHEZ-COI	4318	TRUE		TRUE		0	CINVESTAV
SANCHEZ-COI	4319	TRUE		FALSE		0	IPN
SANCHEZ-COI	4320	FALSE	UNIV CALIF RI	FALSE	751	1	UNIV CALIF RI
SANCHEZ-GIL,	4321	FALSE	UNIV CALIF IR	FALSE	749	1	UNIV CALIF IR
SANCHEZ-JU/	4323	TRUE		FALSE		0	INST TECNOL
SANCHEZ-JU/	4324	TRUE	KURUKSHETR/	FALSE	418	1	KURUKSHETR/
SANCHEZ-JU/	4325	FALSE		FALSE		0	UNAM
SANCHEZ-MC	4326	FALSE	CTR INVEST O	FALSE	148	1	CTR INVEST O
SANCHEZSINE	4331	TRUE		FALSE		0	CINVESTAV
SANCHEZSINE	4332	TRUE	IPN	FALSE	376	1	IPN
SANCHEZSINE	4333	FALSE		FALSE		0	UNAM

SANCHEZ-VILI	4335	TRUE	UNIV MANCH	FALSE	881	1	UNIV MANCH
SANCHEZ-VILI	4336	FALSE		FALSE		0	UNIV MICHOA
SANCHO, JM	4337	FALSE	UNIV BARCEL	FALSE	732	1	UNIV BARCEL
SANDOVAL, J	4340	FALSE		FALSE		0	UNIV SONORA
SANDOVAL-JII	4343	TRUE		FALSE		0	ININ
SANDOVAL-JII	4344	FALSE	UNAM	FALSE	698	1	UNAM
SANDOVAL-VI	4345	FALSE		FALSE		0	UNIV IBEROAI
SANIGER, JM	4347	FALSE		FALSE		0	UNAM
SANIGER-BLES	4348	FALSE	UNIV LA HABA	FALSE	855	1	UNIV LA HABA
SANMIGUEL,	4350	TRUE	UAM I	FALSE	686	1	UAM I
SANMIGUEL,	4351	FALSE		FALSE		0	UNIV SEVILLA
SANTAMARIA	4355	TRUE	CSIC	FALSE	130	1	CSIC
SANTAMARIA	4356	FALSE		FALSE		0	UNIV COMPLU
SANTANA, G	4357	TRUE		TRUE		0	CINVESTAV
SANTANA, G	4358	TRUE		FALSE		0	IPN
SANTANA, G	4359	FALSE	UNIV LA HABA	FALSE	855	1	UNIV LA HABA
SANTI, P	4361	TRUE	HOPE COLL	FALSE	254	1	HOPE COLL
SANTI, P	4362	FALSE	UNIV MICHIG	FALSE	887	1	UNIV MICHIG
SANTIAGO, P	4363	TRUE	UNAM	FALSE	698	1	UNAM
SANTIAGO, P	4364	FALSE	UNIV CONNE(	FALSE	776	1	UNIV CONNE(
SANTILLAN, M	4367	FALSE		FALSE		0	IPN
SANTOPINTO,	4369	TRUE	UNIV GENOA	FALSE	816	1	UNIV GENOA
SANTOPINTO,	4370	FALSE		FALSE		0	YALE UNIV
SANTOS, A	4371	FALSE	UNIV SEVILLA	FALSE	982	1	UNIV SEVILLA
SANTOS, E	4372	FALSE		FALSE		0	UNAM
SANTOS, PV	4373	FALSE	MAX PLANCK	FALSE	470	1	MAX PLANCK
SANTOYO, FM	4375	TRUE	CTR INVEST O	FALSE	148	1	CTR INVEST O
SANTOYO, FM	4376	FALSE		FALSE		0	LOUGHBOROU
SANZ, JF	4378	FALSE	UNIV SEVILLA	FALSE	982	1	UNIV SEVILLA
SARMIENTO, /	4382	FALSE	UNAM	FALSE	698	1	UNAM
SASAKI, T	4383	TRUE	BUAP	FALSE	51	1	BUAP
SASAKI, T	4384	TRUE		TRUE		0	MITI
SASAKI, T	4385	FALSE		FALSE		0	UAEH
SASTRE, F	4386	FALSE	CINVESTAV	FALSE	94	1	CINVESTAV
SATCHLER, GF	4387	FALSE	OAK RIDGE NA	FALSE	533	1	OAK RIDGE NA
SAVELEV, SE	4392	FALSE	BUAP	FALSE	51	1	BUAP
SCHAPHORST	4397	FALSE	UNIV CENT FL	FALSE	766	1	UNIV CENT FL
SCHEID, W	4399	TRUE		FALSE		0	CINVESTAV
SCHEID, W	4400	FALSE	UNAM	FALSE	698	1	UNAM
SCHMIDT, J	4408	TRUE	UNAM	FALSE	698	1	UNAM
SCHMIDT, J	4409	FALSE		FALSE		0	UNIV GIESSEN
SCHNEIBEL, JH	4412	FALSE	OAK RIDGE NA	FALSE	533	1	OAK RIDGE NA
SCHOLL, MS	4414	FALSE		FALSE		0	CTR INVEST O
SCORZELLI, RE	4419	FALSE		FALSE		0	CTR BRASILEII
SEBASTIAN, P.	4420	FALSE		FALSE		0	UNAM
SEGOVIA, N	4422	FALSE	ININ	FALSE	289	1	ININ
SEN, M	4431	TRUE	CORNELL UNI	FALSE	124	1	CORNELL UNI

SEN, M	4432	FALSE		FALSE		0	UNIV NOTRE I
SEN, S	4433	TRUE	ININ	FALSE	289	1	ININ
SEN, S	4434	FALSE		FALSE		0	UNIV NOTRE I
SERKIN, VN	4435	FALSE	RUSSIAN ACA	FALSE	587	1	RUSSIAN ACA
SERRANO-HEI	4436	TRUE	INAOE	FALSE	276	1	INAOE
SERRANO-HEI	4437	FALSE		FALSE		0	PENN STATE L
SERVIN, M	4439	FALSE		FALSE		0	CTR INVEST O
SHAPIRA, D	4444	TRUE		FALSE		0	GRAND ACCEI
SHAPIRA, D	4445	TRUE	RUDJER BOSK	FALSE	585	1	RUDJER BOSK
SHAPIRA, D	4446	FALSE		FALSE		0	UNAM
SHAPIRO, B	4447	FALSE	INST MAX VO	FALSE	322	1	INST MAX VO
SHARMA, A	4449	TRUE	NATL ACAD S(	FALSE	500	1	NATL ACAD S(
SHARMA, A	4450	FALSE	UNAM	FALSE	698	1	UNAM
SHCHEGROV,	4452	FALSE	UNIV CALIF IR	FALSE	749	1	UNIV CALIF IR
SHIROKOV, AI	4460	FALSE	MOSCOW MV	FALSE	488	1	MOSCOW MV
SHKREBTII, AI	4462	TRUE	UNIV PARIS 6	FALSE	940	1	UNIV PARIS 6
SHKREBTII, AI	4463	FALSE		FALSE		0	UNIV ROMA T
SHLYAGIN, M(	4465	FALSE	CICESE	FALSE	89	1	CICESE
SIEJKA, J	4470	FALSE	UNAM	FALSE	698	1	UNAM
SIERRA, O	4471	TRUE		FALSE		0	IMP
SIERRA, O	4472	FALSE	INST CONDEN	FALSE	306	1	INST CONDEN
SIGMAR, DJ	4473	TRUE	CHALMERS UI	FALSE	82	1	CHALMERS UI
SIGMAR, DJ	4474	TRUE		TRUE		0	IV KURCHATC
SIGMAR, DJ	4475	FALSE		FALSE		0	UNAM
SILANTEV, NA	4476	FALSE	INAOE	FALSE	276	1	INAOE
SILVA, CCD	4479	FALSE	UNIV FED PER	FALSE	804	1	UNIV FED PER
SILVA, R	4480	FALSE		FALSE		0	BUAP
SILVA-ANDRA	4481	TRUE		TRUE		0	BUAP
SILVA-ANDRA	4482	TRUE		TRUE		0	CINVESTAV
SILVA-ANDRA	4483	FALSE		FALSE		0	Inst Fis Tecn I(
SILVA-CASTILI	4484	FALSE	BUAP	FALSE	51	1	BUAP
SILVA-GONZA	4485	TRUE	UASLP	FALSE	689	1	UASLP
SILVA-GONZA	4486	FALSE		FALSE		0	UNAM
SILVA-ORTIGC	4487	TRUE	BUAP	FALSE	51	1	BUAP
SILVA-ORTIGC	4488	FALSE		FALSE		0	CINVESTAV
SILVERT, PY	4489	TRUE	UNIV PICARDI	FALSE	946	1	UNIV PICARDI
SILVERT, PY	4490	FALSE		FALSE		0	UNIV SONOR/
SINHA, S	4494	TRUE	INDIAN INST /	FALSE	279	1	INDIAN INST /
SINHA, S	4495	FALSE		FALSE		0	INST MATH S(
SINZINGER, S	4496	FALSE	FERNUNIV	FALSE	220	1	FERNUNIV
SIQUEIROS, JI	4497	FALSE	UNAM	FALSE	698	1	UNAM
SKAKLE, JMS	4498	TRUE	UNAM	FALSE	698	1	UNAM
SKAKLE, JMS	4499	FALSE		FALSE		0	UNIV ABERDE
SLEVIN, K	4503	FALSE	CEA SACLAY	FALSE	66	1	CEA SACLAY
SLOAN, DP	4505	FALSE	CH2M HILL C(	FALSE	81	1	CH2M HILL C(
SMIRNOV, YF	4507	FALSE		FALSE		0	UNAM
SMIRNOVA, N	4508	TRUE	MOSCOW MV	FALSE	488	1	MOSCOW MV

SMIRNOVA, N	4509	FALSE		FALSE		0	UNAM
SMIRNOV-RU	4510	TRUE	CSIC	FALSE	130	1	CSIC
SMIRNOV-RU	4511	FALSE		FALSE		0	CTR INT FIS
SMITH, VH	4516	FALSE		FALSE		0	UAM I
SMYTH, NF	4517	FALSE	UNIV EDINBU	FALSE	786	1	UNIV EDINBU
SOBOLEVA, TI	4519	TRUE	IV KURCHATO	FALSE	387	1	IV KURCHATO
SOBOLEVA, TI	4520	FALSE		FALSE		0	UNAM
SOCORRO, J	4522	TRUE	UAM A	FALSE	685	1	UAM A
SOCORRO, J	4523	TRUE		TRUE		0	UAM I
SOCORRO, J	4524	FALSE		FALSE		0	UNIV GUANA.
SOKOLOWSKI	4527	TRUE		TRUE		0	MARIE CURIE
SOKOLOWSKI	4528	TRUE	NATL ACAD S(	FALSE	500	1	NATL ACAD S(
SOKOLOWSKI	4529	TRUE	UAM I	FALSE	686	1	UAM I
SOKOLOWSKI	4530	FALSE		FALSE		0	UNAM
SOLACHE-RIO	4532	FALSE	UAM I	FALSE	686	1	UAM I
SOLANO, C	4534	FALSE	CTR INVEST O	FALSE	148	1	CTR INVEST O
SOLIS, MA	4540	TRUE	UNAM	FALSE	698	1	UNAM
SOLIS, MA	4541	FALSE		FALSE		0	UNIV VALENC
SOLIS-PERALE	4542	FALSE	UASLP	FALSE	689	1	UASLP
SOLORZA, O	4546	FALSE	CINVESTAV	FALSE	94	1	CINVESTAV
SOLORZA-FER	4547	FALSE	CINVESTAV	FALSE	94	1	CINVESTAV
SOLTERO, JFA	4549	FALSE	UNIV GUADAI	FALSE	822	1	UNIV GUADAI
SONTZ, SB	4551	FALSE		FALSE		0	UAM I
SORIA, JL	4553	FALSE		FALSE		0	INST TECNOL
SORIANO, C	4554	FALSE	QUEENS UNIV	FALSE	572	1	QUEENS UNIV
SORKIN, RD	4557	TRUE		TRUE		0	SYRACUSE UN
SORKIN, RD	4558	FALSE		FALSE		0	UNAM
SOSA, M	4560	FALSE		FALSE		0	UNIV GUANA.
SOSA, V	4563	FALSE	CINVESTAV	FALSE	94	1	CINVESTAV
SOTELO-LERM	4566	TRUE		TRUE		0	BUAP
SOTELO-LERM	4567	TRUE		FALSE		0	CINVESTAV
SOTELO-LERM	4568	FALSE	UNIV SONOR/	FALSE	987	1	UNIV SONOR/
SOTO, G	4571	TRUE	CICESE	FALSE	89	1	CICESE
SOTO, G	4572	FALSE		FALSE		0	UNAM
SOTO, H	4574	FALSE		FALSE		0	CICESE
SOTO, J	4575	FALSE	BUAP	FALSE	51	1	BUAP
SOTO, JR	4576	FALSE	UNAM	FALSE	698	1	UNAM
SPANJAARD, I	4583	TRUE		FALSE		0	CEA SACLAY
SPANJAARD, I	4584	FALSE	Universite Pa	FALSE	1034	1	Universite Pa
SPICER, WE	4585	FALSE	CINVESTAV	FALSE	94	1	CINVESTAV
STADLER, PF	4587	FALSE	UNIV VIENNA	FALSE	1017	1	UNIV VIENNA
STARODUMO	4589	FALSE		FALSE		0	CTR INVEST O
STAVROUDIS,	4591	FALSE		FALSE		0	CTR INVEST O
STEPANOV, S	4596	TRUE	INAOE	FALSE	276	1	INAOE
STEPANOV, S	4597	TRUE	UNIV AUTON(	FALSE	725	1	UNIV AUTON(
STEPANOV, S	4598	FALSE		FALSE		0	UNIV OSNABF
STEPHENS, CF	4599	FALSE		FALSE		0	UNAM

STOKSTAD, R(	4605	TRUE	INST NUCL ST	FALSE	335	1	INST NUCL ST
STOKSTAD, R(	4606	FALSE		FALSE		0	UNAM
STRBIK, V	4611	FALSE	SLOVAK TECH	FALSE	614	1	SLOVAK TECH
STROJNIK, M	4612	FALSE		FALSE		0	CTR INVEST O
STRUCK, CW	4613	FALSE	BOSTON COLL	FALSE	43	1	BOSTON COLL
STRUTT, PR	4614	TRUE		FALSE		0	Inframat Corp
STRUTT, PR	4615	FALSE	UNIV CONNE(	FALSE	776	1	UNIV CONNE(
STUART, RG	4616	TRUE		FALSE		0	CINVESTAV
STUART, RG	4617	FALSE	MAX PLANCK	FALSE	466	1	MAX PLANCK
SU, S	4618	FALSE	UNIV CALIF IR	FALSE	749	1	UNIV CALIF IR
SUAREZ, R	4619	FALSE	UAM A	FALSE	685	1	UAM A
SUCKUT, C	4622	FALSE	UNIV ABERDE	FALSE	706	1	UNIV ABERDE
SUDARSKY, D	4623	FALSE	UNAM	FALSE	698	1	UNAM
SUGI, Y	4625	FALSE	GIFU UNIV	FALSE	235	1	GIFU UNIV
SUN, Y	4635	TRUE		TRUE		0	UNAM
SUN, Y	4636	FALSE	UNIV TENNES	FALSE	996	1	UNIV TENNES
SUNTA, CM	4637	TRUE	ATOM ENERG	FALSE	28	1	ATOM ENERG
SUNTA, CM	4638	FALSE		FALSE		0	UNIV FED SAC
SUSARREY, O	4640	FALSE		FALSE		0	IPN
SUSSMAN, RA	4641	FALSE		FALSE		0	UNAM
SZWAYKOWSI	4644	FALSE		FALSE		0	INAOE
TABARES-MU	4645	FALSE	UNAM	FALSE	698	1	UNAM
TADJEDDINE,	4646	TRUE		FALSE		0	LAB ELECTRO(
TADJEDDINE,	4647	FALSE	LAB ELECTRO(	FALSE	424	1	LAB ELECTRO(
TAKEUCHI, N	4652	FALSE		FALSE		0	UNAM
TALANQUER,	4653	FALSE	UNAM	FALSE	698	1	UNAM
TALAVERA, RF	4656	FALSE	UAM I	FALSE	686	1	UAM I
TAMEZ, E	4659	FALSE	LAB GEOFLUII	FALSE	425	1	LAB GEOFLUII
TAPIA, V	4665	FALSE		FALSE		0	UNIV SONORA
TAVARES-VEL	4667	FALSE		FALSE		0	CINVESTAV
TAVERA, L	4668	FALSE	ININ	FALSE	289	1	ININ
TCHIJOV, V	4670	FALSE		FALSE		0	UNAM
TEHUACANER	4674	FALSE	UNAM	FALSE	698	1	UNAM
TEKAAT, EH	4676	TRUE		FALSE		0	CESKOSLOVEN
TEKAAT, EH	4677	FALSE	CESKOSLOVES	FALSE	79	1	CESKOSLOVES
TELLEZ, J	4683	FALSE	UNIV SONORA	FALSE	987	1	UNIV SONORA
TELLO, PG	4684	TRUE		FALSE		0	EUITI
TELLO, PG	4685	FALSE	UNAM	FALSE	698	1	UNAM
TENTORI, D	4686	FALSE	CICESE	FALSE	89	1	CICESE
TEPICHIN, E	4687	FALSE	INAOE	FALSE	276	1	INAOE
TERA, AH	4690	FALSE	UNAM	FALSE	698	1	UNAM
TERAN, LMY	4691	FALSE	UAM I	FALSE	686	1	UAM I
TERRONES, H	4695	FALSE	UNAM	FALSE	698	1	UNAM
TERRONES, M	4696	TRUE		TRUE		0	TOYOHASHI U
TERRONES, M	4697	TRUE	UNAM	FALSE	698	1	UNAM
TERRONES, M	4698	TRUE		TRUE		0	UNIV CALIF SA
TERRONES, M	4699	FALSE		FALSE		0	UNIV SUSSEX

TESSIERI, L	4700	TRUE		FALSE		0	BUAP
TESSIERI, L	4701	FALSE	BUDKER INST	FALSE	52	1	BUDKER INST
TESTORF, M	4703	FALSE	INAOE	FALSE	276	1	INAOE
TIBURCIO-SIL\	4708	FALSE	CINVESTAV	FALSE	94	1	CINVESTAV
TIRADO-MEJI/	4713	TRUE	CINVESTAV	FALSE	94	1	CINVESTAV
TIRADO-MEJI/	4714	FALSE		FALSE		0	UNIV QUINDI(
TITOV, OY	4715	FALSE	TEACHER TRA	FALSE	647	1	TEACHER TRA
TKACH, VI	4717	FALSE		FALSE		0	UNIV GUANA.
TOCHO, JO	4719	FALSE	CTR INVEST O	FALSE	148	1	CTR INVEST O
TOMAS, SA	4721	TRUE	CINVESTAV	FALSE	94	1	CINVESTAV
TOMAS, SA	4722	FALSE	UNIV ANTIOQ	FALSE	712	1	UNIV ANTIOQ
TORCHYNSKA	4727	FALSE		FALSE		0	IPN
TORRES, A	4731	TRUE	CINVESTAV	FALSE	94	1	CINVESTAV
TORRES, A	4732	FALSE	UNAM	FALSE	698	1	UNAM
TORRES, J	4736	TRUE		FALSE		0	CICESE
TORRES, J	4737	TRUE	JOINT INST NI	FALSE	395	1	JOINT INST NI
TORRES, J	4738	TRUE	UNAM	FALSE	698	1	UNAM
TORRES, J	4739	FALSE		FALSE		0	UNIV GUANA.
TORRES, JL	4740	FALSE	UNIV MICHO/	FALSE	888	1	UNIV MICHO/
TORRES, M	4742	TRUE		FALSE		0	CSIC
TORRES, M	4743	FALSE	UNAM	FALSE	698	1	UNAM
TORRES, MZ	4744	FALSE	IPN	FALSE	376	1	IPN
TORRES-CISNI	4745	TRUE		TRUE		0	IMA
TORRES-CISNI	4746	TRUE		FALSE		0	INAOE
TORRES-CISNI	4747	FALSE	ST JOHN FISH	FALSE	621	1	ST JOHN FISH
TORRES-CISNI	4748	TRUE	MOSCOW MV	FALSE	488	1	MOSCOW MV
TORRES-CISNI	4749	FALSE	UNIV GUANA.	FALSE	823	1	UNIV GUANA.
TORRES-DELG	4750	FALSE	CINVESTAV	FALSE	94	1	CINVESTAV
TORRES-MAR	4752	FALSE	UANL	FALSE	687	1	UANL
TORRES-VEGA	4753	FALSE	UNIV NEVAD/	FALSE	917	1	UNIV NEVAD/
TORRES-VILLA	4754	FALSE	UNAM	FALSE	698	1	UNAM
TOSCANO, JJ	4756	FALSE	BUAP	FALSE	51	1	BUAP
TRALLERO-GII	4760	TRUE	UNIV HAVAN/	FALSE	831	1	UNIV HAVAN/
TRALLERO-GII	4761	FALSE	UNIV LA HAB/	FALSE	855	1	UNIV LA HAB/
TRASOBARES,	4763	TRUE	UNAM	FALSE	698	1	UNAM
TRASOBARES,	4764	FALSE	UNIV SUSSEX	FALSE	994	1	UNIV SUSSEX
TREJOLUNA, I	4768	FALSE	UNAM	FALSE	698	1	UNAM
TREVINO, C	4771	FALSE	UNAM	FALSE	698	1	UNAM
TRIPATHI, AN	4773	FALSE	QUEENS UNIV	FALSE	572	1	QUEENS UNIV
TROKHYMCHU	4774	TRUE	MARIE CURIE	FALSE	456	1	MARIE CURIE
TROKHYMCHU	4775	TRUE		FALSE		0	NATL ACAD S(
TROKHYMCHU	4776	FALSE		FALSE		0	UNAM
TROLTENIER,	4778	TRUE	JOINT INST HI	FALSE	394	1	JOINT INST HI
TROLTENIER,	4779	FALSE		FALSE		0	UNIV FRANKF
TROMBETTA,	4780	FALSE	UNIV GENOA	FALSE	816	1	UNIV GENOA
TSIFRINOVICH	4785	FALSE		FALSE		0	UNIV GUADAI
TUFINO-VELA	4790	TRUE	IPN	FALSE	376	1	IPN

TUFINO-VELA	4791	FALSE		FALSE		0	UNIV HAVAN/
TUN, DM	4793	FALSE	IPN	FALSE	376	1	IPN
TUR, A	4794	TRUE		FALSE		0	KHARKOV SIN
TUR, A	4795	FALSE	OBSERV MIDI	FALSE	535	1	OBSERV MIDI
TURBINER, A\	4796	FALSE	UNAM	FALSE	698	1	UNAM
UDAGAWA, T	4800	FALSE	UNIV TEXAS	FALSE	997	1	UNIV TEXAS
UGALDE, E	4801	TRUE		FALSE		0	CPT Luminy
UGALDE, E	4802	FALSE	UASLP	FALSE	689	1	UASLP
ULLOA, SE	4804	TRUE	BUAP	FALSE	51	1	BUAP
ULLOA, SE	4805	TRUE		TRUE		0	OHIO UNIV
ULLOA, SE	4806	TRUE		TRUE		0	UNAM
ULLOA, SE	4807	TRUE		FALSE		0	UNIV ILLINOIS
ULLOA, SE	4808	FALSE	UNIV MUNICH	FALSE	898	1	UNIV MUNICH
UMEMOTO, M	4810	FALSE	IPN	FALSE	376	1	IPN
URBANIAK-KU	4811	FALSE	UNIV LODZ	FALSE	869	1	UNIV LODZ
URBINA, RH	4812	FALSE	UNIV SONOR/	FALSE	987	1	UNIV SONOR/
URIBE, FJ	4816	FALSE		FALSE		0	UAM I
URIBE, J	4817	TRUE	UNIV GUANA.	FALSE	823	1	UNIV GUANA.
URIBE, J	4818	FALSE	UNIV MASSAC	FALSE	886	1	UNIV MASSAC
URIBE, MM	4819	TRUE		FALSE		0	CINVESTAV
URIBE, MM	4820	FALSE	UNIV ESTADU	FALSE	792	1	UNIV ESTADU
VALCARCE, A	4829	TRUE		FALSE		0	IPN
VALCARCE, A	4830	FALSE	UNIV SALAM/	FALSE	971	1	UNIV SALAM/
VALDES, ED	4833	FALSE	CINVESTAV	FALSE	94	1	CINVESTAV
VALDEZ, MA	4835	FALSE		FALSE		0	UNIV SONOR/
VALENZUELA,	4837	FALSE	UNAM	FALSE	698	1	UNAM
VALENZUELA,	4838	FALSE	IPN	FALSE	376	1	IPN
VALLADARES,	4842	TRUE	INST PHYS NU	FALSE	341	1	INST PHYS NU
VALLADARES,	4843	FALSE	UNAM	FALSE	698	1	UNAM
VALLADARES,	4844	TRUE		FALSE		0	UNAM
VALLADARES,	4845	FALSE	UNIV OXFORD	FALSE	933	1	UNIV OXFORD
VALLES-GONZ	4847	TRUE	INST NACL TE	FALSE	327	1	INST NACL TE
VALLES-GONZ	4848	FALSE		FALSE		0	INTA
VALLIERES, M	4851	TRUE	BEIJING UNIV	FALSE	37	1	BEIJING UNIV
VALLIERES, M	4852	FALSE		FALSE		0	DREXEL UNIV
VANDERDONI	4854	FALSE	UNAM	FALSE	698	1	UNAM
VANTENDELO	4859	TRUE		FALSE		0	ANTWERP ST/
VANTENDELO	4860	FALSE	CEN SCK	FALSE	70	1	CEN SCK
VAREA, C	4862	FALSE	UNAM	FALSE	698	1	UNAM
VARGAS, CE	4865	FALSE	CINVESTAV	FALSE	94	1	CINVESTAV
VARGAS, H	4867	TRUE		FALSE		0	INST NACL PE
VARGAS, H	4868	TRUE	UNIV ESTADU	FALSE	796	1	UNIV ESTADU
VARGAS, H	4869	FALSE		FALSE		0	UNIV LA HAB/
VARGAS, M	4870	FALSE		FALSE		0	CINVESTAV
VARGAS, R	4871	FALSE	UAM I	FALSE	686	1	UAM I
VARGAS, S	4872	FALSE	UAM I	FALSE	686	1	UAM I
VASCO, E	4877	TRUE	CINVESTAV	FALSE	94	1	CINVESTAV

VASCO, E	4878	FALSE		FALSE		0	CNR INST
VASCONCELL(	4879	FALSE	UNIV ESTADU	FALSE	792	1	UNIV ESTADU
VASSEUR, JO	4882	FALSE	UNIV LILLE 1	FALSE	865	1	UNIV LILLE 1
VAZQUEZ, A	4885	FALSE		FALSE		0	UNAM
VAZQUEZ, F	4887	TRUE	INST INVEST E	FALSE	318	1	INST INVEST E
VAZQUEZ, F	4888	FALSE		FALSE		0	UNIV AUTON(
VAZQUEZ, GJ	4889	FALSE	UNAM	FALSE	698	1	UNAM
VAZQUEZ, M	4890	TRUE		FALSE		0	UCM
VAZQUEZ, M	4891	FALSE	UNAM	FALSE	698	1	UNAM
VAZQUEZ-CA!	4894	TRUE	BUAP	FALSE	51	1	BUAP
VAZQUEZ-CA!	4895	FALSE		FALSE		0	CTR INVEST O
VAZQUEZ-MC	4898	TRUE		TRUE		0	INAOE
VAZQUEZ-MC	4899	FALSE		FALSE		0	UAM I
VAZQUEZ-NA'	4900	FALSE		FALSE		0	UNIV AUTON(
VAZQUEZ-POI	4901	TRUE		FALSE		0	UNAM
VAZQUEZ-POI	4902	FALSE	UNIV SONOR/	FALSE	987	1	UNIV SONOR/
VAZQUEZ-ZA\	4903	FALSE	UNAM	FALSE	698	1	UNAM
VECIANA, J	4904	FALSE		FALSE		0	BUAP
VEENHUIS, H	4905	TRUE		FALSE		0	INAOE
VEENHUIS, H	4906	FALSE	UNIV OSNABI	FALSE	929	1	UNIV OSNABI
VEGA, A	4907	FALSE		FALSE		0	UNIV VALLAD
VEGA, JJ	4909	FALSE		FALSE		0	UNIV NOTRE I
VELASCO, VR	4913	FALSE	CSIC	FALSE	130	1	CSIC
VELAZQUEZ, I	4916	FALSE	IPN	FALSE	376	1	IPN
VELAZQUEZ, \	4918	FALSE	JUSTUS LIEBI(	FALSE	397	1	JUSTUS LIEBI(
VENEGAS, V	4921	FALSE		FALSE		0	IPN
VERA-GRAZIA	4923	FALSE	UNAM	FALSE	698	1	UNAM
VERBAARSCH	4924	TRUE	MAX PLANCK	FALSE	465	1	MAX PLANCK
VERBAARSCH	4925	FALSE		FALSE		0	UNIV ILLINOIS
VERDIGUEL, H	4927	FALSE	UNAM	FALSE	698	1	UNAM
VERGARA, JD	4931	FALSE	UNAM	FALSE	698	1	UNAM
VIANA, L	4937	FALSE	UNAM	FALSE	698	1	UNAM
VICTOR, GA	4940	FALSE		FALSE		0	HARVARD SM
VIDAL, J	4941	FALSE	CINVESTAV	FALSE	94	1	CINVESTAV
VIDAL, MA	4942	FALSE	CINVESTAV	FALSE	94	1	CINVESTAV
VIDIELLA-BAR	4945	TRUE	INAOE	FALSE	276	1	INAOE
VIDIELLA-BAR	4946	FALSE	UNIV LONDOI	FALSE	871	1	UNIV LONDOI
VIESTI, G	4948	TRUE		FALSE		0	IST NAZL FIS N
VIESTI, G	4949	TRUE	JAGIELLONIAI	FALSE	389	1	JAGIELLONIAI
VIESTI, G	4950	FALSE		FALSE		0	UNIV PADUA
VIGIL, O	4951	TRUE	CINVESTAV	FALSE	94	1	CINVESTAV
VIGIL, O	4952	TRUE	UNIV HAVAN/	FALSE	831	1	UNIV HAVAN/
VIGIL, O	4953	FALSE		FALSE		0	UNIV LA HAB/
VILLA, F	4956	FALSE	CTR INVEST O	FALSE	148	1	CTR INVEST O
VILLA, J	4957	TRUE		FALSE		0	cidesi
VILLA, J	4958	FALSE	CTR INVEST O	FALSE	148	1	CTR INVEST O
VILLAFUERTE-	4959	TRUE	UNAM	FALSE	698	1	UNAM

VILLAFUERTE-	4960	FALSE	UNIV ABERDE	FALSE	706	1	UNIV ABERDE
VILLAGOMEZ,	4961	TRUE	CISECE	FALSE	97	1	CICESE
VILLAGOMEZ,	4962	TRUE		TRUE		0	RUSSIAN ACA
VILLAGOMEZ,	4963	FALSE		FALSE		0	UNAM
VILLAGRAN-M	4964	TRUE		TRUE		0	CONSEJO NAC
VILLAGRAN-M	4965	FALSE		FALSE		0	UNAM
VILLANUEVA,	4968	FALSE		FALSE		0	UNIV GUANA.
VILLANUEVA,	4970	TRUE		FALSE		0	UAM I
VILLANUEVA,	4971	FALSE	UNIV MICHOA	FALSE	888	1	UNIV MICHOA
VILLARI, ACC	4972	TRUE		FALSE		0	IST NAZL FIS N
VILLARI, ACC	4973	FALSE	UNIV CATANI.	FALSE	763	1	UNIV CATANI.
VILLARREAL, (	4974	FALSE		FALSE		0	UNAM
VILLASENOR,	4976	FALSE		FALSE		0	UNAM
VILLASENOR,	4977	TRUE		FALSE		0	BUAP
VILLASENOR,	4978	TRUE	UNIV GUANA.	FALSE	823	1	UNIV GUANA.
VILLASENOR,	4979	FALSE		FALSE		0	UNIV MICHOA
VILLATORO, J	4983	TRUE		FALSE		0	INAOE
VILLATORO, J	4984	FALSE	UNAM	FALSE	698	1	UNAM
VILLAVICENCI	4985	TRUE		TRUE		0	CICESE
VILLAVICENCI	4986	TRUE		TRUE		0	UNAM
VILLAVICENCI	4987	FALSE		FALSE		0	UNIV AUTON(
VILLAVICENCI	4988	FALSE	UNAM	FALSE	698	1	UNAM
VITELA, JE	4997	FALSE	UNAM	FALSE	698	1	UNAM
VIVEROS, T	4998	TRUE		TRUE		0	IMP
VIVEROS, T	4999	FALSE		FALSE		0	UAM I
VLAEV, SJ	5001	TRUE	BULGARIAN A	FALSE	53	1	BULGARIAN A
VLAEV, SJ	5002	FALSE		FALSE		0	UAZ
VOITSEKHOVI	5004	FALSE		FALSE		0	UNAM
VOLOSHIN, IF	5007	TRUE	BUAP	FALSE	51	1	BUAP
VOLOSHIN, IF	5008	FALSE		FALSE		0	UKRAINIAN A
VOLOVICHEV,	5009	TRUE		FALSE		0	CICATA
VOLOVICHEV,	5010	FALSE	CINVESTAV	FALSE	94	1	CINVESTAV
VOROBIEV, YV	5012	TRUE		TRUE		0	CINVESTAV
VOROBIEV, YV	5013	TRUE		TRUE		0	INST TECNOL
VOROBIEV, YV	5014	FALSE	UAQ	FALSE	688	1	UAQ
VYSLOUKH, V/	5015	TRUE		FALSE		0	INAOE
VYSLOUKH, V/	5016	FALSE	MOSCOW MV	FALSE	488	1	MOSCOW MV
WADA, R	5017	FALSE	INST PHYS NU	FALSE	341	1	INST PHYS NU
WAELBROECK	5019	TRUE	UNAM	FALSE	698	1	UNAM
WAELBROECK	5020	FALSE		FALSE		0	UNIV CALIF SA
WALLACE, JL	5023	FALSE	CINVESTAV	FALSE	94	1	CINVESTAV
WALLER, SM	5025	FALSE	UNAM	FALSE	698	1	UNAM
WANG, CM	5028	FALSE	UNAM	FALSE	698	1	UNAM
WANG, HM	5029	FALSE		FALSE		0	CTR INVEST O
WANG, JA	5030	TRUE		TRUE		0	IPN
WANG, JA	5031	TRUE		FALSE		0	UAM A
WANG, JA	5032	FALSE	UNAM	FALSE	698	1	UNAM

WANG, MHLS	5036	FALSE	UNIV MASSA(	FALSE	886	1	UNIV MASSA(
WANG, S	5038	FALSE	UNAM	FALSE	698	1	UNAM
WEBER, A	5047	FALSE	UNAM	FALSE	698	1	UNAM
WEIDENMULL	5049	FALSE	UNAM	FALSE	698	1	UNAM
WHITE-STEVE	5062	TRUE		FALSE		0	HOPE COLL
WHITE-STEVE	5063	FALSE	UNIV MICHIG	FALSE	887	1	UNIV MICHIG
WHITFIELD, SI	5064	FALSE	UNIV AMSTEF	FALSE	711	1	UNIV AMSTEF
WIEDERHOLD	5066	FALSE	CINVESTAV	FALSE	94	1	CINVESTAV
WILLIS, JR	5071	FALSE	UNIV BATH	FALSE	736	1	UNIV BATH
WOBIG, H	5077	FALSE		FALSE		0	MAX PLANCK
WONG, JCC	5082	FALSE	UNIV PARIS 6	FALSE	940	1	UNIV PARIS 6
WUDKA, J	5084	FALSE	UNIV CALIF RI	FALSE	751	1	UNIV CALIF RI
XIAO, MF	5088	TRUE		TRUE		0	CICESE
XIAO, MF	5089	TRUE		TRUE		0	UNAM
XIAO, MF	5090	FALSE		FALSE		0	UNIV NACL AU
XIAO, TD	5091	TRUE	CTR INVEST M	FALSE	147	1	CTR INVEST M
XIAO, TD	5092	TRUE		FALSE		0	Inframat Corp
XIAO, TD	5093	FALSE	US NANOCOR	FALSE	1043	1	US NANOCOR
YADAVA, YP	5094	FALSE	UNAM	FALSE	698	1	UNAM
YAMALEEV, R	5096	FALSE		FALSE		0	UNAM
YAMPOLSKII, '	5097	TRUE		FALSE		0	BUAP
YAMPOLSKII, '	5098	TRUE	KHARKOV AM	FALSE	405	1	KHARKOV AM
YAMPOLSKII, '	5099	FALSE		FALSE		0	KHARKOV STA
YANEZ, G	5101	FALSE	CINVESTAV	FALSE	94	1	CINVESTAV
YANEZ-LIMON	5103	FALSE		FALSE		0	CINVESTAV
YEE-MADEIRA	5108	TRUE	ININ	FALSE	289	1	ININ
YEE-MADEIRA	5109	FALSE		FALSE		0	IPN
YEPEZ, E	5112	TRUE	CINVESTAV	FALSE	94	1	CINVESTAV
YEPEZ, E	5113	FALSE		FALSE		0	IPN
YOUSIF, FB	5117	TRUE		FALSE		0	UNAM
YOUSIF, FB	5118	TRUE	UNIV WESTEF	FALSE	1026	1	UNIV WESTEF
YOUSIF, FB	5119	FALSE		FALSE		0	WESLEYAN UI
YU, J	5120	TRUE		FALSE		0	UAM A
YU, J	5121	FALSE	UNIV AUTON(	FALSE	725	1	UNIV AUTON(
YUAN, CP	5122	TRUE		TRUE		0	ARGONNE NA
YUAN, CP	5123	TRUE		FALSE		0	CINVESTAV
YUAN, CP	5124	FALSE	JORDAN UNIV	FALSE	396	1	JORDAN UNIV
YUSTE, SB	5125	FALSE	UNIV EXTREM	FALSE	798	1	UNIV EXTREM
ZALDIVAR, MI	5129	FALSE	UNIV COMPLU	FALSE	775	1	UNIV COMPLU
ZALDO, C	5130	TRUE		TRUE		0	UAM I
ZALDO, C	5131	TRUE		FALSE		0	UNAM
ZALDO, C	5132	FALSE	UNIV AUTON(	FALSE	727	1	UNIV AUTON(
ZAMORANO-U	5135	TRUE	CEN	FALSE	68	1	CEN
ZAMORANO-U	5136	FALSE		FALSE		0	IPN
ZANNIAS, T	5138	TRUE		TRUE		0	UNAM
ZANNIAS, T	5139	FALSE		FALSE		0	UNIV MICHOA
ZAPATA, JA	5140	TRUE		FALSE		0	PENN STATE U

ZAPATA, JA	5141	FALSE	UNAM	FALSE	698	1	UNAM
ZAPATA-NAV/	5142	FALSE	CINVESTAV	FALSE	94	1	CINVESTAV
ZAPATA-TORF	5143	TRUE	CINVESTAV	FALSE	94	1	CINVESTAV
ZAPATA-TORF	5144	FALSE	Pacific NW La	FALSE	548	1	Pacific NW La
ZAYAS, ME	5150	TRUE		FALSE		0	CICESE
ZAYAS, ME	5151	TRUE	CSIC	FALSE	130	1	CSIC
ZAYAS, ME	5152	FALSE	INST CERAM &	FALSE	300	1	INST CERAM &
ZELAYA-ANGE	5159	TRUE	CINVESTAV	FALSE	94	1	CINVESTAV
ZELAYA-ANGE	5160	FALSE		FALSE		0	IPN
ZENTENO-SAN	5165	FALSE		FALSE		0	CTR INVEST O
ZERTUCHE, F	5167	FALSE	UNAM	FALSE	698	1	UNAM
ZHAO, E	5170	FALSE	UNIV WISCON	FALSE	1027	1	UNIV WISCON
ZHEVANDROV	5171	FALSE	CINVESTAV	FALSE	94	1	CINVESTAV
ZHOU, Y	5174	TRUE		FALSE		0	CINVESTAV
ZHOU, Y	5175	TRUE	UNAM	FALSE	698	1	UNAM
ZHOU, Y	5176	FALSE	UNIV CALIF IR	FALSE	749	1	UNIV CALIF IR
ZHU, YH	5177	TRUE		TRUE		0	TONGJI UNIV
ZHU, YH	5178	FALSE		FALSE		0	UNAM
ZHU, YQ	5179	TRUE		TRUE		0	UNAM
ZHU, YQ	5180	TRUE		TRUE		0	UNIV BAYREU
ZHU, YQ	5181	TRUE		FALSE		0	UNIV CALIF S/
ZHU, YQ	5182	FALSE		FALSE		0	UNIV SUSSEX
ZINGARO, RA	5185	FALSE	UNAM	FALSE	698	1	UNAM
ZIRONI, EP	5186	TRUE		TRUE		0	UNAM
ZIRONI, EP	5187	FALSE		FALSE		0	UNIV NACL IN
ZOGAIB, L	5188	FALSE	UNAM	FALSE	698	1	UNAM
ZOUPANOS, G	5193	TRUE	MAX PLANCK	FALSE	466	1	MAX PLANCK
ZOUPANOS, G	5194	TRUE	NAT TECH UN	FALSE	498	1	NAT TECH UN
ZOUPANOS, G	5195	FALSE		FALSE		0	NATL TECH UI
ZUNIGA, HF	5198	FALSE	UNAM	FALSE	698	1	UNAM
ZUNIGA-SEGU	5199	FALSE	CINVESTAV	FALSE	94	1	CINVESTAV
ZURITA, GR	5200	FALSE	BUAP	FALSE	51	1	BUAP

dorg_ii	entryorgiiid	entryorgiiid_i	entry	exit	enbp	enbp_b	exap
FALSE	148	1	1988	2003		1	
FALSE	698	1	1987	1994		1	
FALSE	94	1	1994	1996		1	
FALSE	918	1	1993	1997		1	
FALSE	165	1	1993	2002		1	
FALSE	577	1	1993	2002		1	
FALSE	686	1	1993	2002		1	
FALSE	987	1	1993	2002		1	
FALSE	51	1	1992	2003		1	
FALSE	276	1	1992	2003		1	
FALSE	51	1	1997	1998		1	
FALSE	698	1	1993	2000		1	
FALSE	94	1	1998	2003		1	
FALSE	689	1	1987	2001		1	
FALSE	689	1	1997	2003		1	
FALSE	987	1	1997	2003		1	
FALSE	690	1	1995	2003		1	
FALSE	804	1	1996	2001		1	
FALSE	686	1	1991	1998		1	
FALSE	698	1	1991	1998		1	
FALSE	687	1	1995	1999		1	
FALSE	89	1	1994	2000		1	
FALSE	94	1	1996	2002		1	
FALSE	698	1	1996	2002		1	
FALSE	727	1	1996	2002		1	
FALSE	698	1	1997	1999		1	
FALSE	259	1	1995	2003		1	
FALSE	276	1	1995	2003		1	
FALSE	1048	1	1991	1997		1	
FALSE	94	1	1998	2003		1	
FALSE	376	1	1992	2003		1	
FALSE	289	1	1990	2003		1	
FALSE	276	1	1999	2000		1	
FALSE	698	1	1986	2003		1	
FALSE	693	1	1998	2002		1	
FALSE	685	1	1991	1992		1	
FALSE	698	1	1991	1992		1	
FALSE	698	1	1996	1999		1	
FALSE	698	1	1988	2003		1	
FALSE	698	1	1989	2002		1	
FALSE	698	1	1998	2000		1	
FALSE	728	1	1993	1999		1	
FALSE	94	1	1999	2001		1	
FALSE	688	1	1999	2001		1	
FALSE	728	1	1999	2001		1	
FALSE	686	1	1990	1994		1	

FALSE	698	1	1990	1994	1
FALSE	562	1	1988	2000	1
FALSE	698	1	1997	2002	1
FALSE	698	1	1998	2000	1
FALSE	46	1	1990	1999	1
FALSE	698	1	1988	1995	1
FALSE	698	1	1998	2000	1
FALSE	698	1	1992	2003	1
FALSE	698	1	1999	2003	1
FALSE	376	1	1995	2001	1
FALSE	698	1	1998	1999	1
FALSE	724	1	1998	2000	1
FALSE	880	1	1989	2001	1
FALSE	689	1	1994	2003	1
FALSE	725	1	1998	2001	1
FALSE	750	1	1998	2001	1
FALSE	686	1	1998	2003	1
FALSE	755	1	1991	2002	1
FALSE	89	1	1993	2003	1
FALSE	376	1	1994	1998	1
FALSE	94	1	1987	2003	1
FALSE	698	1	1987	2003	1
FALSE	72	1	1992	1993	1
FALSE	686	1	1995	2003	1
FALSE	289	1	1996	1998	1
FALSE	686	1	1996	1998	1
FALSE	698	1	1994	1995	1
FALSE	698	1	1990	1999	1
FALSE	776	1	1986	2003	1
FALSE	89	1	1998	2003	1
FALSE	698	1	1998	2003	1
FALSE	831	1	1998	2003	1
FALSE	855	1	1998	2003	1
FALSE	927	1	1998	2003	1
FALSE	634	1	1990	1992	1
FALSE	698	1	1990	1992	1
FALSE	698	1	1986	2000	1
FALSE	698	1	1989	1993	1
FALSE	698	1	1992	2003	1
FALSE	276	1	1999	2001	1
FALSE	565	1	1999	2001	1
FALSE	823	1	1999	2001	1
FALSE	276	1	1989	1996	1
FALSE	1010	1	1989	1996	1
FALSE	686	1	1985	2003	1
FALSE	135	1	1997	2001	1
FALSE	94	1	1994	1995	1

FALSE	228	1	1994	1995	1
FALSE	685	1	1995	2003	1
FALSE	698	1	1996	2003	1
FALSE	276	1	1998	2002	1
FALSE	686	1	1993	2003	1
FALSE	685	1	1998	2000	1
FALSE	376	1	1990	2003	1
FALSE	698	1	1990	2003	1
FALSE	94	1	1998	2001	1
FALSE	698	1	1997	2000	1
FALSE	276	1	1993	2002	1
FALSE	51	1	1996	2001	1
FALSE	94	1	1996	2001	1
FALSE	698	1	1993	2002	1
FALSE	275	1	1996	2001	1
FALSE	855	1	1998	2001	1
FALSE	94	1	1995	2003	1
FALSE	376	1	1989	1999	1
FALSE	686	1	1989	1999	1
FALSE	276	1	1996	2001	1
FALSE	982	1	1991	2003	1
FALSE	376	1	1994	2002	1
FALSE	685	1	1994	2002	1
FALSE	698	1	1988	1992	1
FALSE	955	1	1993	2000	1
FALSE	94	1	1997	2003	1
FALSE	987	1	1997	2003	1
FALSE	51	1	1995	2003	1
FALSE	276	1	1995	2003	1
FALSE	51	1	1992	2003	1
FALSE	276	1	1992	2003	1
FALSE	686	1	1992	2002	1
FALSE	130	1	1998	2001	1
FALSE	727	1	1998	2001	1
FALSE	148	1	1999	2001	1
FALSE	324	1	1992	1993	1
FALSE	491	1	1992	1993	1
FALSE	289	1	1998	2003	1
FALSE	698	1	1998	2003	1
FALSE	553	1	1998	2001	1
FALSE	272	1	1997	1998	1
FALSE	686	1	1997	1998	1
FALSE	94	1	1986	2003	1
FALSE	888	1	1999	2001	1
FALSE	4	1	1989	2003	1
FALSE	698	1	1989	2003	1
FALSE	686	1	1995	1996	1

FALSE	347	1	1995	1997	1
FALSE	380	1	1995	1997	1
FALSE	698	1	1989	2003	1
FALSE	1051	1	1989	2003	1
FALSE	1063	1	1989	2003	1
FALSE	376	1	1991	1997	1
FALSE	51	1	1999	2001	1
FALSE	103	1	1999	2001	1
FALSE	94	1	1993	2003	1
FALSE	698	1	1994	2003	1
FALSE	725	1	1994	2003	1
FALSE	289	1	1999	2001	1
FALSE	698	1	1999	2001	1
FALSE	698	1	1999	2003	1
FALSE	698	1	1999	2001	1
FALSE	855	1	1999	2001	1
FALSE	94	1	1998	2002	1
FALSE	28	1	1997	1999	1
FALSE	980	1	1997	1999	1
FALSE	987	1	1997	1999	1
FALSE	698	1	1992	2000	1
FALSE	289	1	1985	2002	1
FALSE	987	1	1998	2001	1
FALSE	94	1	1985	1986	1
FALSE	376	1	1985	1986	1
FALSE	276	1	1998	2001	1
FALSE	810	1	1991	1997	1
FALSE	451	1	1990	1995	1
FALSE	385	1	1995	2003	1
FALSE	990	1	1991	1994	1
FALSE	215	1	1997	1999	1
FALSE	289	1	1997	1999	1
FALSE	689	1	1999	2000	1
FALSE	376	1	1995	2003	1
FALSE	16	1	1995	1997	1
FALSE	697	1	1995	1997	1
FALSE	698	1	1994	2003	1
FALSE	698	1	1998	2001	1
FALSE	27	1	1994	2002	1
FALSE	148	1	1988	2002	1
FALSE	987	1	1994	2003	1
FALSE	148	1	1998	2003	1
FALSE	558	1	1998	2003	1
FALSE	698	1	1992	2000	1
FALSE	698	1	1997	2001	1
FALSE	831	1	1997	2001	1
FALSE	855	1	1997	2001	1

FALSE	685	1	1999	2003	1
FALSE	1010	1	1991	1993	1
FALSE	686	1	1998	2003	1
FALSE	148	1	1995	2003	1
FALSE	685	1	1992	1993	1
FALSE	686	1	1992	1993	1
FALSE	94	1	1997	2002	1
FALSE	376	1	1997	2002	1
FALSE	698	1	1997	2002	1
FALSE	613	1	1999	2000	1
FALSE	698	1	1999	2003	1
FALSE	888	1	1999	2003	1
FALSE	276	1	1999	2002	1
FALSE	823	1	1999	2002	1
FALSE	698	1	1996	2000	1
FALSE	289	1	1999	2002	1
FALSE	376	1	1999	2002	1
FALSE	727	1	1987	2002	1
FALSE	51	1	1998	2001	1
FALSE	698	1	1999	2003	1
FALSE	822	1	1999	2003	1
FALSE	698	1	1992	1996	1
FALSE	94	1	1997	2001	1
FALSE	987	1	1997	2001	1
FALSE	94	1	1990	2003	1
FALSE	685	1	1990	2003	1
FALSE	539	1	1996	2000	1
FALSE	940	1	1996	2000	1
FALSE	963	1	1996	2000	1
FALSE	116	1	1990	2003	1
FALSE	698	1	1990	2003	1
FALSE	94	1	1999	2000	1
FALSE	361	1	1999	2000	1
FALSE	688	1	1999	2000	1
FALSE	698	1	1993	2003	1
FALSE	698	1	1989	2003	1
FALSE	276	1	1999	2002	1
FALSE	845	1	1997	2003	1
FALSE	289	1	1996	1998	1
FALSE	698	1	1996	1998	1
FALSE	747	1	1996	1998	1
FALSE	776	1	1996	1998	1
FALSE	823	1	1994	1995	1
FALSE	99	1	1992	1994	1
FALSE	698	1	1993	2003	1
FALSE	734	1	1993	2003	1
FALSE	686	1	1990	1997	1

FALSE	928	1	1998	2000	1
FALSE	987	1	1998	2000	1
FALSE	689	1	1996	1998	1
FALSE	823	1	1997	2003	1
FALSE	886	1	1997	2003	1
FALSE	449	1	1997	2003	1
FALSE	89	1	1999	2002	1
FALSE	94	1	1999	2002	1
FALSE	823	1	1998	2003	1
FALSE	64	1	1994	1996	1
FALSE	276	1	1984	2003	1
FALSE	698	1	1986	2000	1
FALSE	216	1	1989	1990	1
FALSE	276	1	1989	1990	1
FALSE	698	1	1997	2003	1
FALSE	698	1	1995	2003	1
FALSE	927	1	1998	2003	1
FALSE	928	1	1998	2003	1
FALSE	987	1	1998	2003	1
FALSE	376	1	1995	1997	1
FALSE	403	1	1995	1997	1
FALSE	451	1	1998	2000	1
FALSE	698	1	1998	2000	1
FALSE	818	1	1998	2000	1
FALSE	64	1	1997	1998	1
FALSE	94	1	1991	1995	1
FALSE	698	1	1996	2000	1
FALSE	774	1	1996	2000	1
FALSE	342	1	1995	1997	1
FALSE	403	1	1995	1997	1
FALSE	383	1	1995	2002	1
FALSE	698	1	1995	2002	1
FALSE	982	1	1995	2002	1
FALSE	94	1	1995	2001	1
FALSE	698	1	1986	2000	1
FALSE	698	1	1997	2000	1
FALSE	205	1	1995	1996	1
FALSE	954	1	1992	2003	1
FALSE	376	1	1999	2001	1
FALSE	838	1	1999	2001	1
FALSE	38	1	1999	2003	1
FALSE	902	1	1999	2003	1
FALSE	988	1	1999	2003	1
FALSE	698	1	1991	2003	1
FALSE	698	1	1985	2001	1
FALSE	406	1	1999	2001	1
FALSE	946	1	1999	2002	1

FALSE	940	1	1993	2000	1
FALSE	947	1	1993	2000	1
FALSE	508	1	1989	2000	1
FALSE	289	1	1992	2002	1
FALSE	93	1	1998	2001	1
FALSE	148	1	1998	2001	1
FALSE	94	1	1990	1998	1
FALSE	698	1	1992	1998	1
FALSE	94	1	1997	2003	1
FALSE	651	1	1999	2001	1
FALSE	698	1	1999	2001	1
FALSE	19	1	1994	1997	1
FALSE	107	1	1999	2001	1
FALSE	385	1	1999	2001	1
FALSE	881	1	1997	2001	1
FALSE	51	1	1986	2003	1
FALSE	94	1	1986	2003	1
FALSE	154	1	1994	2001	1
FALSE	958	1	1994	2001	1
FALSE	51	1	1991	2001	1
FALSE	698	1	1984	1989	1
FALSE	376	1	1993	1996	1
FALSE	698	1	1995	2003	1
FALSE	118	1	1999	2001	1
FALSE	822	1	1999	2001	1
FALSE	148	1	1998	2003	1
FALSE	698	1	1998	2003	1
FALSE	725	1	1998	2003	1
FALSE	745	1	1998	2003	1
FALSE	921	1	1998	2003	1
FALSE	698	1	1996	1999	1
FALSE	698	1	1989	1997	1
FALSE	689	1	1995	1996	1
FALSE	686	1	1992	2002	1
FALSE	686	1	1984	1992	1
FALSE	698	1	1984	1992	1
FALSE	471	1	1997	1999	1
FALSE	411	1	1997	2003	1
FALSE	266	1	1999	2002	1
FALSE	861	1	1999	2002	1
FALSE	929	1	1998	2001	1
FALSE	94	1	1998	2001	1
FALSE	855	1	1998	2001	1
FALSE	376	1	1990	2000	1
FALSE	530	1	1990	2000	1
FALSE	369	1	1995	1999	1
FALSE	823	1	1995	1999	1

FALSE	289	1	1992	2000	1
FALSE	94	1	1989	1991	1
FALSE	698	1	1989	1991	1
FALSE	698	1	1997	2001	1
FALSE	689	1	1990	1996	1
FALSE	94	1	1997	2000	1
FALSE	855	1	1998	1999	1
FALSE	642	1	1993	2003	1
FALSE	94	1	1998	2001	1
FALSE	685	1	1989	2003	1
FALSE	686	1	1989	2003	1
FALSE	508	1	1991	1999	1
FALSE	766	1	1991	1999	1
FALSE	289	1	1999	2002	1
FALSE	698	1	1998	2000	1
FALSE	990	1	1998	2000	1
FALSE	148	1	1987	2003	1
FALSE	94	1	1995	2001	1
FALSE	698	1	1988	2003	1
FALSE	698	1	1999	2000	1
FALSE	148	1	1999	2002	1
FALSE	94	1	1984	2003	1
FALSE	51	1	1997	2000	1
FALSE	276	1	1997	2000	1
FALSE	686	1	1999	2002	1
FALSE	940	1	1999	2002	1
FALSE	698	1	1989	2003	1
FALSE	822	1	1994	2002	1
FALSE	940	1	1990	2001	1
FALSE	698	1	1989	2002	1
FALSE	698	1	1989	2003	1
FALSE	51	1	1999	2001	1
FALSE	698	1	1999	2001	1
FALSE	831	1	1999	2001	1
FALSE	888	1	1987	2002	1
FALSE	987	1	1987	2002	1
FALSE	289	1	1992	2003	1
FALSE	698	1	1995	2003	1
FALSE	945	1	1989	2003	1
FALSE	1055	1	1986	1994	1
FALSE	470	1	1995	2003	1
FALSE	51	1	1989	1998	1
FALSE	982	1	1994	1995	1
FALSE	94	1	1992	2003	1
FALSE	689	1	1996	2003	1
FALSE	51	1	1999	2002	1
FALSE	94	1	1999	2002	1

FALSE	94	1	1990	1998	1
FALSE	698	1	1987	1993	1
FALSE	689	1	1998	2001	1
FALSE	1010	1	1998	2001	1
FALSE	276	1	1986	1998	1
FALSE	30	1	1995	2002	1
FALSE	698	1	1995	2002	1
FALSE	776	1	1996	1997	1
FALSE	878	1	1996	1997	1
FALSE	542	1	1998	2000	1
FALSE	720	1	1998	2000	1
FALSE	276	1	1993	2000	1
FALSE	1010	1	1993	2000	1
FALSE	698	1	1998	1999	1
FALSE	276	1	1998	2001	1
FALSE	698	1	1995	1998	1
FALSE	933	1	1995	1998	1
FALSE	698	1	1999	2001	1
FALSE	289	1	1995	2001	1
FALSE	51	1	1997	2001	1
FALSE	94	1	1997	2001	1
FALSE	51	1	1996	2003	1
FALSE	686	1	1988	1993	1
FALSE	732	1	1988	1993	1
FALSE	230	1	1995	1997	1
FALSE	265	1	1995	1997	1
FALSE	698	1	1998	2003	1
FALSE	839	1	1995	2003	1
FALSE	108	1	1984	1986	1
FALSE	686	1	1984	1986	1
FALSE	698	1	1996	2002	1
FALSE	727	1	1996	2001	1
FALSE	987	1	1996	2001	1
FALSE	698	1	1998	2003	1
FALSE	94	1	1986	2002	1
FALSE	94	1	1995	2002	1
FALSE	686	1	1998	2003	1
FALSE	698	1	1989	2003	1
FALSE	698	1	1984	1996	1
FALSE	94	1	1992	2003	1
FALSE	822	1	1992	2003	1
FALSE	276	1	1993	2003	1
FALSE	698	1	1988	2002	1
FALSE	685	1	1995	2002	1
FALSE	376	1	1992	2003	1
FALSE	376	1	1989	1996	1
FALSE	698	1	1989	1996	1

FALSE	987	1	1989	1997	1
FALSE	276	1	1997	2003	1
FALSE	693	1	1997	2003	1
FALSE	94	1	1999	2000	1
FALSE	823	1	1999	2000	1
FALSE	94	1	1989	2003	1
FALSE	727	1	1989	2003	1
FALSE	94	1	1989	2001	1
FALSE	276	1	1996	1998	1
FALSE	51	1	1997	2001	1
FALSE	276	1	1997	2001	1
FALSE	94	1	1995	2003	1
FALSE	102	1	1995	2003	1
FALSE	686	1	1988	1993	1
FALSE	94	1	1997	2002	1
FALSE	94	1	1989	1991	1
FALSE	126	1	1989	1991	1
FALSE	727	1	1989	1991	1
FALSE	792	1	1989	1991	1
FALSE	369	1	1995	1998	1
FALSE	745	1	1995	1998	1
FALSE	276	1	1998	2003	1
FALSE	725	1	1998	2003	1
FALSE	94	1	1998	2002	1
FALSE	148	1	1988	2001	1
FALSE	987	1	1988	2001	1
FALSE	289	1	1993	2003	1
FALSE	854	1	1993	2003	1
FALSE	698	1	1984	2002	1
FALSE	328	1	1994	1995	1
FALSE	820	1	1994	1995	1
FALSE	698	1	1994	1998	1
FALSE	30	1	1993	2003	1
FALSE	131	1	1993	2003	1
FALSE	833	1	1994	1995	1
FALSE	89	1	1998	2002	1
FALSE	219	1	1999	2002	1
FALSE	747	1	1999	2002	1
FALSE	689	1	1998	1999	1
FALSE	219	1	1999	2002	1
FALSE	887	1	1999	2002	1
FALSE	342	1	1993	1995	1
FALSE	1023	1	1993	1995	1
FALSE	879	1	1994	1995	1
FALSE	686	1	1992	2003	1
FALSE	289	1	1997	1999	1
FALSE	972	1	1996	2002	1

FALSE	276	1	1996	2003	1
FALSE	871	1	1996	2003	1
FALSE	698	1	1993	1998	1
FALSE	155	1	1995	2003	1
FALSE	689	1	1995	2003	1
FALSE	685	1	1986	1998	1
FALSE	698	1	1997	2003	1
FALSE	685	1	1985	2002	1
FALSE	103	1	1998	2001	1
FALSE	698	1	1998	2003	1
FALSE	633	1	1996	2000	1
FALSE	698	1	1996	2000	1
FALSE	776	1	1996	2000	1
FALSE	823	1	1999	2002	1
FALSE	417	1	1996	1997	1
FALSE	589	1	1996	1997	1
FALSE	861	1	1999	2002	1
FALSE	1019	1	1999	2002	1
FALSE	823	1	1994	1997	1
FALSE	886	1	1996	2003	1
FALSE	599	1	1997	2003	1
FALSE	613	1	1997	2003	1
FALSE	690	1	1996	2003	1
FALSE	276	1	1993	2002	1
FALSE	648	1	1993	2002	1
FALSE	698	1	1993	2002	1
FALSE	663	1	1992	1999	1
FALSE	823	1	1992	1999	1
FALSE	810	1	1996	2000	1
FALSE	663	1	1997	1999	1
FALSE	944	1	1997	1999	1
FALSE	987	1	1994	1999	1
FALSE	372	1	1990	1997	1
FALSE	698	1	1984	2003	1
FALSE	51	1	1989	2003	1
FALSE	698	1	1985	2003	1
FALSE	328	1	1993	1995	1
FALSE	698	1	1993	1995	1
FALSE	108	1	1991	2002	1
FALSE	792	1	1991	1994	1
FALSE	1010	1	1995	2001	1
FALSE	94	1	1992	2003	1
FALSE	178	1	1994	2002	1
FALSE	689	1	1998	1999	1
FALSE	94	1	1999	2000	1
FALSE	89	1	1996	2002	1
FALSE	260	1	1996	2002	1

FALSE	888	1	1997	2001	1
FALSE	698	1	1989	2003	1
FALSE	689	1	1988	2003	1
FALSE	542	1	1998	2000	1
FALSE	933	1	1993	1998	1
FALSE	51	1	1993	2003	1
FALSE	698	1	1985	2003	1
FALSE	272	1	1998	2000	1
FALSE	698	1	1998	2000	1
FALSE	987	1	1999	2001	1
FALSE	553	1	1997	2003	1
FALSE	698	1	1997	2003	1
FALSE	751	1	1997	2003	1
FALSE	685	1	1998	2001	1
FALSE	276	1	1991	1993	1
FALSE	698	1	1998	1999	1
FALSE	686	1	1984	2001	1
FALSE	698	1	1984	2001	1
FALSE	698	1	1987	2002	1
FALSE	698	1	1984	2002	1
FALSE	51	1	1997	2002	1
FALSE	94	1	1997	2002	1
FALSE	379	1	1997	2002	1
FALSE	611	1	1997	2002	1
FALSE	51	1	1998	2002	1
FALSE	698	1	1998	2002	1
FALSE	941	1	1998	2001	1
FALSE	698	1	1996	1997	1
FALSE	686	1	1995	2003	1
FALSE	698	1	1995	2003	1
FALSE	94	1	1999	2001	1
FALSE	289	1	1999	2001	1
FALSE	831	1	1999	2001	1
FALSE	855	1	1999	2001	1
FALSE	686	1	1997	1999	1
FALSE	94	1	1993	2000	1
FALSE	698	1	1994	2000	1
FALSE	89	1	1999	2000	1
FALSE	855	1	1999	2000	1
FALSE	376	1	1984	1998	1
FALSE	698	1	1997	2003	1
FALSE	786	1	1997	2003	1
FALSE	698	1	1989	2001	1
FALSE	715	1	1988	1993	1
FALSE	260	1	1997	2002	1
FALSE	94	1	1998	2002	1
FALSE	318	1	1997	1998	1

FALSE	51	1	1996	1997	1
FALSE	94	1	1996	1997	1
FALSE	89	1	1997	2003	1
FALSE	205	1	1997	2003	1
FALSE	93	1	1999	2002	1
FALSE	148	1	1999	2002	1
FALSE	775	1	1999	2002	1
FALSE	414	1	1997	2003	1
FALSE	698	1	1997	2003	1
FALSE	725	1	1997	2003	1
FALSE	146	1	1997	1998	1
FALSE	987	1	1997	1998	1
FALSE	148	1	1999	2003	1
FALSE	770	1	1999	2003	1
FALSE	822	1	1999	2003	1
FALSE	686	1	1998	2002	1
FALSE	698	1	1998	2002	1
FALSE	148	1	1998	2003	1
FALSE	276	1	1998	2003	1
FALSE	871	1	1998	2003	1
FALSE	289	1	1984	1994	1
FALSE	923	1	1984	1994	1
FALSE	803	1	1989	1991	1
FALSE	512	1	1992	1994	1
FALSE	698	1	1990	2003	1
FALSE	698	1	1999	2001	1
FALSE	148	1	1988	2002	1
FALSE	51	1	1992	1998	1
FALSE	208	1	1994	2003	1
FALSE	689	1	1998	1999	1
FALSE	987	1	1997	2001	1
FALSE	376	1	1995	2001	1
FALSE	805	1	1997	2001	1
FALSE	858	1	1997	2001	1
FALSE	376	1	1998	1999	1
FALSE	685	1	1998	1999	1
FALSE	775	1	1995	1996	1
FALSE	51	1	1998	2003	1
FALSE	822	1	1998	2003	1
FALSE	94	1	1986	2003	1
FALSE	689	1	1998	1999	1
FALSE	124	1	1988	1994	1
FALSE	471	1	1996	2003	1
FALSE	698	1	1996	2003	1
FALSE	698	1	1984	1989	1
FALSE	51	1	1997	2001	1
FALSE	148	1	1997	2000	1

FALSE	148	1	1995	2003	1
FALSE	698	1	1986	2002	1
FALSE	94	1	1987	2003	1
FALSE	698	1	1987	2003	1
FALSE	689	1	1997	1999	1
FALSE	130	1	1989	1997	1
FALSE	94	1	1996	1998	1
FALSE	325	1	1996	1998	1
FALSE	376	1	1996	1998	1
FALSE	689	1	1996	1998	1
FALSE	94	1	1998	2003	1
FALSE	385	1	1998	2003	1
FALSE	686	1	1996	2003	1
FALSE	698	1	1998	2000	1
FALSE	686	1	1992	2000	1
FALSE	686	1	1993	1996	1
FALSE	775	1	1993	1996	1
FALSE	698	1	1990	2003	1
FALSE	722	1	1990	2003	1
FALSE	58	1	1994	1995	1
FALSE	698	1	1994	1995	1
FALSE	108	1	1984	2002	1
FALSE	686	1	1984	2002	1
FALSE	963	1	1991	1999	1
FALSE	428	1	1990	1992	1
FALSE	1011	1	1990	1992	1
FALSE	94	1	1994	2002	1
FALSE	855	1	1994	2002	1
FALSE	698	1	1993	1996	1
FALSE	376	1	1989	2002	1
FALSE	405	1	1997	2001	1
FALSE	73	1	1998	2000	1
FALSE	689	1	1998	2000	1
FALSE	642	1	1985	1989	1
FALSE	698	1	1985	1989	1
FALSE	376	1	1993	2003	1
FALSE	289	1	1998	2001	1
FALSE	809	1	1998	2001	1
FALSE	103	1	1997	2000	1
FALSE	289	1	1997	2000	1
FALSE	686	1	1997	2000	1
FALSE	698	1	1994	2001	1
FALSE	718	1	1993	2003	1
FALSE	831	1	1995	2001	1
FALSE	855	1	1995	2001	1
FALSE	698	1	1996	2001	1
FALSE	51	1	1995	2002	1

FALSE	94	1	1995	2002	1
FALSE	685	1	1995	2003	1
FALSE	94	1	1990	2003	1
FALSE	719	1	1990	2003	1
FALSE	686	1	1989	2002	1
FALSE	689	1	1994	2003	1
FALSE	94	1	1998	2002	1
FALSE	686	1	1999	2003	1
FALSE	148	1	1996	2003	1
FALSE	545	1	1996	2003	1
FALSE	51	1	1985	2002	1
FALSE	276	1	1985	2002	1
FALSE	376	1	1993	2002	1
FALSE	698	1	1994	1996	1
FALSE	1050	1	1985	1988	1
FALSE	465	1	1991	1997	1
FALSE	51	1	1994	2000	1
FALSE	865	1	1994	2000	1
FALSE	865	1	1992	1998	1
FALSE	487	1	1993	1995	1
FALSE	449	1	1995	1996	1
FALSE	689	1	1995	1996	1
FALSE	698	1	1994	2002	1
FALSE	272	1	1984	2003	1
FALSE	689	1	1998	2003	1
FALSE	449	1	1997	2003	1
FALSE	452	1	1997	2003	1
FALSE	376	1	1999	2001	1
FALSE	685	1	1999	2003	1
FALSE	745	1	1999	2003	1
FALSE	451	1	1989	2003	1
FALSE	698	1	1989	2003	1
FALSE	42	1	1987	1991	1
FALSE	373	1	1992	2003	1
FALSE	428	1	1992	2003	1
FALSE	689	1	1992	2003	1
FALSE	912	1	1992	2003	1
FALSE	289	1	1997	2002	1
FALSE	698	1	1997	2002	1
FALSE	811	1	1986	1991	1
FALSE	698	1	1997	1998	1
FALSE	698	1	1999	2001	1
FALSE	690	1	1993	2003	1
FALSE	698	1	1993	2003	1
FALSE	698	1	1991	1992	1
FALSE	854	1	1991	1992	1
FALSE	822	1	1996	1997	1

FALSE	51	1	1997	2003	1
FALSE	698	1	1996	1999	1
FALSE	698	1	1993	1994	1
FALSE	933	1	1993	1994	1
FALSE	10	1	1995	2003	1
FALSE	690	1	1996	1997	1
FALSE	698	1	1996	1997	1
FALSE	89	1	1998	2003	1
FALSE	99	1	1987	1988	1
FALSE	698	1	1992	1994	1
FALSE	94	1	1999	2002	1
FALSE	147	1	1999	2002	1
FALSE	361	1	1999	2002	1
FALSE	698	1	1992	2000	1
FALSE	725	1	1992	2000	1
FALSE	276	1	1998	2001	1
FALSE	89	1	1991	2001	1
FALSE	749	1	1991	2001	1
FALSE	698	1	1998	2003	1
FALSE	94	1	1997	1999	1
FALSE	376	1	1997	1999	1
FALSE	289	1	1999	2003	1
FALSE	686	1	1999	2003	1
FALSE	698	1	1987	2002	1
FALSE	94	1	1998	2001	1
FALSE	385	1	1995	1999	1
FALSE	94	1	1997	2000	1
FALSE	698	1	1995	2003	1
FALSE	823	1	1999	2002	1
FALSE	94	1	1990	2003	1
FALSE	987	1	1990	2003	1
FALSE	379	1	1994	1996	1
FALSE	383	1	1994	1996	1
FALSE	685	1	1991	2001	1
FALSE	686	1	1991	2001	1
FALSE	698	1	1997	2003	1
FALSE	663	1	1994	2000	1
FALSE	94	1	1997	2003	1
FALSE	147	1	1999	2000	1
FALSE	698	1	1999	2000	1
FALSE	698	1	1992	1993	1
FALSE	101	1	1998	2001	1
FALSE	383	1	1997	1999	1
FALSE	935	1	1997	1999	1
FALSE	911	1	1993	2001	1
FALSE	686	1	1993	1994	1
FALSE	51	1	1999	2000	1

FALSE	94	1	1984	2003	1
FALSE	94	1	1989	2002	1
FALSE	698	1	1989	2002	1
FALSE	882	1	1994	1997	1
FALSE	112	1	1996	2003	1
FALSE	218	1	1996	2003	1
FALSE	823	1	1996	2003	1
FALSE	686	1	1996	2003	1
FALSE	689	1	1996	2003	1
FALSE	175	1	1984	1985	1
FALSE	513	1	1984	1985	1
FALSE	51	1	1991	2000	1
FALSE	94	1	1991	2000	1
FALSE	698	1	1991	2003	1
FALSE	94	1	1984	2003	1
FALSE	376	1	1997	2003	1
FALSE	971	1	1997	2003	1
FALSE	289	1	1999	2001	1
FALSE	698	1	1999	2001	1
FALSE	775	1	1998	2001	1
FALSE	94	1	1992	2003	1
FALSE	686	1	1992	2003	1
FALSE	289	1	1992	1996	1
FALSE	686	1	1996	2003	1
FALSE	242	1	1993	2003	1
FALSE	83	1	1994	1998	1
FALSE	698	1	1994	1998	1
FALSE	254	1	1997	1999	1
FALSE	383	1	1997	1999	1
FALSE	16	1	1993	2000	1
FALSE	17	1	1993	2000	1
FALSE	459	1	1991	1993	1
FALSE	698	1	1991	1993	1
FALSE	94	1	1998	2001	1
FALSE	698	1	1994	1997	1
FALSE	727	1	1994	1997	1
FALSE	820	1	1994	1997	1
FALSE	56	1	1996	1999	1
FALSE	698	1	1992	2003	1
FALSE	204	1	1994	2001	1
FALSE	987	1	1994	2001	1
FALSE	698	1	1992	1997	1
FALSE	289	1	1991	1995	1
FALSE	698	1	1991	1995	1
FALSE	51	1	1997	2000	1
FALSE	94	1	1996	2002	1
FALSE	698	1	1989	1991	1

FALSE	686	1	1989	2003	1
FALSE	698	1	1994	1995	1
FALSE	260	1	1999	2003	1
FALSE	855	1	1998	2003	1
FALSE	823	1	1996	1999	1
FALSE	990	1	1999	2001	1
FALSE	376	1	1998	1999	1
FALSE	1027	1	1998	1999	1
FALSE	51	1	1991	1996	1
FALSE	687	1	1996	2002	1
FALSE	89	1	1996	2003	1
FALSE	752	1	1996	2003	1
FALSE	261	1	1993	2003	1
FALSE	698	1	1993	2003	1
FALSE	752	1	1993	2003	1
FALSE	698	1	1986	1999	1
FALSE	698	1	1991	2003	1
FALSE	637	1	1986	1999	1
FALSE	698	1	1986	1999	1
FALSE	89	1	1997	2003	1
FALSE	698	1	1997	2003	1
FALSE	831	1	1997	2003	1
FALSE	855	1	1997	2003	1
FALSE	927	1	1997	2003	1
FALSE	276	1	1998	2003	1
FALSE	685	1	1994	2001	1
FALSE	148	1	1998	2002	1
FALSE	690	1	1998	2002	1
FALSE	725	1	1998	2002	1
FALSE	94	1	1992	2002	1
FALSE	94	1	1988	1999	1
FALSE	1063	1	1985	1993	1
FALSE	776	1	1995	2001	1
FALSE	488	1	1989	1995	1
FALSE	698	1	1991	2003	1
FALSE	480	1	1993	2001	1
FALSE	30	1	1999	2003	1
FALSE	686	1	1999	2003	1
FALSE	649	1	1996	1999	1
FALSE	823	1	1996	1999	1
FALSE	860	1	1996	1999	1
FALSE	510	1	1998	2002	1
FALSE	698	1	1998	2002	1
FALSE	437	1	1997	2000	1
FALSE	689	1	1997	2000	1
FALSE	533	1	1996	2003	1
FALSE	698	1	1996	2003	1

FALSE	930	1	1997	2001	1
FALSE	698	1	1997	2002	1
FALSE	112	1	1994	2003	1
FALSE	94	1	1995	2003	1
FALSE	94	1	1999	2003	1
FALSE	148	1	1999	2003	1
FALSE	376	1	1995	2000	1
FALSE	94	1	1987	1988	1
FALSE	289	1	1992	2000	1
FALSE	698	1	1984	2003	1
FALSE	698	1	1998	2002	1
FALSE	94	1	1988	2002	1
FALSE	135	1	1988	2002	1
FALSE	855	1	1999	2002	1
FALSE	855	1	1996	2001	1
FALSE	376	1	1993	1994	1
FALSE	698	1	1996	2001	1
FALSE	687	1	1995	1998	1
FALSE	974	1	1995	1998	1
FALSE	376	1	1985	2002	1
FALSE	94	1	1999	2002	1
FALSE	361	1	1999	2002	1
FALSE	688	1	1999	2002	1
FALSE	987	1	1999	2002	1
FALSE	94	1	1993	2003	1
FALSE	89	1	1993	2003	1
FALSE	728	1	1993	2003	1
FALSE	148	1	1997	1999	1
FALSE	902	1	1999	2002	1
FALSE	698	1	1995	1996	1
FALSE	844	1	1995	1996	1
FALSE	698	1	1995	1997	1
FALSE	94	1	1997	2003	1
FALSE	855	1	1997	2003	1
FALSE	688	1	1998	1999	1
FALSE	698	1	1992	1999	1
FALSE	272	1	1997	1998	1
FALSE	289	1	1992	2000	1
FALSE	698	1	1995	2003	1
FALSE	51	1	1999	2001	1
FALSE	682	1	1999	2001	1
FALSE	263	1	1999	2000	1
FALSE	1040	1	1999	2000	1
FALSE	276	1	1997	2003	1
FALSE	698	1	1997	2003	1
FALSE	89	1	1993	2001	1
FALSE	698	1	1995	1996	1

FALSE	395	1	1993	1997	1
FALSE	686	1	1993	2002	1
FALSE	698	1	1998	2002	1
FALSE	698	1	1993	1999	1
FALSE	798	1	1993	1999	1
FALSE	698	1	1989	2003	1
FALSE	823	1	1999	2000	1
FALSE	686	1	1986	1988	1
FALSE	698	1	1999	2002	1
FALSE	698	1	1995	2000	1
FALSE	698	1	1984	1985	1
FALSE	698	1	1992	2002	1
FALSE	135	1	1993	1995	1
FALSE	519	1	1993	1995	1
FALSE	89	1	1996	2000	1
FALSE	698	1	1984	1985	1
FALSE	686	1	1993	2002	1
FALSE	516	1	1988	2002	1
FALSE	698	1	1996	1999	1
FALSE	94	1	1993	2000	1
FALSE	686	1	1986	2003	1
FALSE	698	1	1986	2003	1
FALSE	148	1	1999	2002	1
FALSE	558	1	1999	2002	1
FALSE	698	1	1989	2003	1
FALSE	46	1	1998	2003	1
FALSE	698	1	1998	2003	1
FALSE	276	1	1994	1996	1
FALSE	51	1	1997	2001	1
FALSE	289	1	1993	2002	1
FALSE	376	1	1993	2002	1
FALSE	525	1	1986	2003	1
FALSE	698	1	1986	2003	1
FALSE	698	1	1996	2001	1
FALSE	698	1	1996	2003	1
FALSE	406	1	1999	2001	1
FALSE	698	1	1989	2002	1
FALSE	383	1	1992	1995	1
FALSE	289	1	1996	1997	1
FALSE	776	1	1996	1997	1
FALSE	698	1	1985	2002	1
FALSE	51	1	1998	2000	1
FALSE	685	1	1994	2003	1
FALSE	987	1	1999	2001	1
FALSE	94	1	1997	2000	1
FALSE	992	1	1997	2000	1
FALSE	686	1	1992	1998	1

FALSE	698	1	1992	1998	1
FALSE	936	1	1994	2003	1
FALSE	94	1	1995	2001	1
FALSE	51	1	1999	2001	1
FALSE	276	1	1999	2001	1
FALSE	698	1	1997	2001	1
FALSE	938	1	1996	2002	1
FALSE	698	1	1998	2001	1
FALSE	51	1	1988	2003	1
FALSE	94	1	1988	2003	1
FALSE	698	1	1988	2003	1
FALSE	289	1	1999	2000	1
FALSE	688	1	1999	2000	1
FALSE	89	1	1998	2000	1
FALSE	698	1	1998	2000	1
FALSE	94	1	1998	2001	1
FALSE	725	1	1998	2003	1
FALSE	289	1	1992	1994	1
FALSE	272	1	1997	2002	1
FALSE	686	1	1997	2002	1
FALSE	130	1	1989	1990	1
FALSE	370	1	1993	1995	1
FALSE	698	1	1994	1998	1
FALSE	10	1	1998	2003	1
FALSE	689	1	1998	2003	1
FALSE	94	1	1999	2001	1
FALSE	354	1	1999	2001	1
FALSE	500	1	1999	2001	1
FALSE	688	1	1999	2001	1
FALSE	652	1	1997	2003	1
FALSE	698	1	1997	2003	1
FALSE	663	1	1992	2003	1
FALSE	51	1	1999	2001	1
FALSE	51	1	1995	2003	1
FALSE	690	1	1993	1997	1
FALSE	376	1	1999	2003	1
FALSE	686	1	1999	2003	1
FALSE	698	1	1992	1995	1
FALSE	698	1	1991	2003	1
FALSE	698	1	1992	2003	1
FALSE	491	1	1991	2000	1
FALSE	509	1	1997	2003	1
FALSE	698	1	1997	2003	1
FALSE	698	1	1997	2002	1
FALSE	753	1	1997	2002	1
FALSE	994	1	1997	2002	1
FALSE	698	1	1999	2002	1

FALSE	996	1	1999	2002	1
FALSE	89	1	1991	1998	1
FALSE	639	1	1991	1998	1
FALSE	698	1	1987	1993	1
FALSE	89	1	1989	2003	1
FALSE	685	1	1989	2003	1
FALSE	940	1	1989	2003	1
FALSE	94	1	1996	1999	1
FALSE	689	1	1996	1999	1
FALSE	186	1	1999	2001	1
FALSE	689	1	1997	1999	1
FALSE	698	1	1997	2003	1
FALSE	855	1	1997	2003	1
FALSE	927	1	1997	2003	1
FALSE	698	1	1998	1999	1
FALSE	685	1	1998	2002	1
FALSE	698	1	1998	2002	1
FALSE	987	1	1998	2002	1
FALSE	376	1	1998	1999	1
FALSE	44	1	1999	2001	1
FALSE	116	1	1999	2001	1
FALSE	855	1	1999	2001	1
FALSE	51	1	1998	2003	1
FALSE	685	1	1998	2003	1
FALSE	254	1	1998	2000	1
FALSE	282	1	1998	2000	1
FALSE	352	1	1998	2000	1
FALSE	923	1	1998	2000	1
FALSE	689	1	1995	2003	1
FALSE	697	1	1993	2003	1
FALSE	94	1	1996	2001	1
FALSE	33	1	1995	2003	1
FALSE	94	1	1995	2003	1
FALSE	560	1	1995	2003	1
FALSE	51	1	1998	2000	1
FALSE	289	1	1998	2000	1
FALSE	698	1	1998	2000	1
FALSE	276	1	1994	2001	1
FALSE	698	1	1984	2003	1
FALSE	718	1	1997	2001	1
FALSE	698	1	1997	1998	1
FALSE	51	1	1998	2003	1
FALSE	379	1	1998	2003	1
FALSE	690	1	1998	2003	1
FALSE	289	1	1992	2001	1
FALSE	273	1	1999	2003	1
FALSE	276	1	1999	2003	1

FALSE	698	1	1990	2003	1
FALSE	319	1	1994	1997	1
FALSE	686	1	1994	1997	1
FALSE	698	1	1998	1999	1
FALSE	344	1	1999	2003	1
FALSE	829	1	1999	2003	1
FALSE	733	1	1992	1995	1
FALSE	613	1	1994	2000	1
FALSE	698	1	1994	2000	1
FALSE	686	1	1989	2003	1
FALSE	831	1	1995	2002	1
FALSE	886	1	1989	2002	1
FALSE	276	1	1999	2001	1
FALSE	576	1	1999	2001	1
FALSE	779	1	1999	2001	1
FALSE	588	1	1992	1994	1
FALSE	596	1	1992	1994	1
FALSE	744	1	1993	1997	1
FALSE	94	1	1998	2003	1
FALSE	686	1	1998	2003	1
FALSE	698	1	1995	1999	1
FALSE	46	1	1994	2002	1
FALSE	686	1	1994	2002	1
FALSE	698	1	1994	2002	1
FALSE	698	1	1990	2003	1
FALSE	698	1	1993	1996	1
FALSE	94	1	1988	2002	1
FALSE	94	1	1994	2003	1
FALSE	831	1	1994	2003	1
FALSE	987	1	1994	2003	1
FALSE	94	1	1991	2002	1
FALSE	51	1	1998	2000	1
FALSE	686	1	1998	2000	1
FALSE	690	1	1998	2000	1
FALSE	94	1	1999	2002	1
FALSE	831	1	1999	2002	1
FALSE	94	1	1994	2003	1
FALSE	376	1	1987	2003	1
FALSE	1027	1	1987	2003	1
FALSE	698	1	1996	2002	1
FALSE	94	1	1993	2003	1
FALSE	94	1	1992	2002	1
FALSE	992	1	1992	2002	1
FALSE	376	1	1990	1992	1
FALSE	689	1	1991	1992	1
FALSE	698	1	1998	2003	1
FALSE	803	1	1990	2001	1

FALSE	94	1	1993	2002	1
FALSE	698	1	1984	2003	1
FALSE	51	1	1988	2003	1
FALSE	698	1	1994	1999	1
FALSE	698	1	1989	2003	1
FALSE	628	1	1996	2002	1
FALSE	918	1	1997	1999	1
FALSE	451	1	1987	2003	1
FALSE	698	1	1987	2003	1
FALSE	103	1	1993	1995	1
FALSE	328	1	1993	1995	1
FALSE	698	1	1993	1995	1
FALSE	385	1	1996	2003	1
FALSE	698	1	1989	2003	1
FALSE	376	1	1994	1998	1
FALSE	698	1	1986	2003	1
FALSE	220	1	1993	1994	1
FALSE	221	1	1993	1994	1
FALSE	94	1	1987	1993	1
FALSE	1027	1	1987	1993	1
FALSE	161	1	1989	1997	1
FALSE	747	1	1998	1999	1
FALSE	464	1	1998	2000	1
FALSE	698	1	1998	2000	1
FALSE	994	1	1998	2000	1
FALSE	698	1	1991	2003	1
FALSE	994	1	1999	2000	1
FALSE	663	1	1995	1997	1
FALSE	254	1	1998	2000	1
FALSE	887	1	1998	2000	1
FALSE	708	1	1994	1996	1
FALSE	897	1	1994	1999	1
FALSE	698	1	1996	2000	1
FALSE	1065	1	1996	2000	1
FALSE	276	1	1989	2000	1
FALSE	276	1	1994	1998	1
FALSE	698	1	1997	1999	1
FALSE	760	1	1997	2001	1
FALSE	775	1	1997	2001	1
FALSE	491	1	1992	1993	1
FALSE	987	1	1992	1993	1
FALSE	500	1	1995	2000	1
FALSE	587	1	1999	2000	1
FALSE	51	1	1995	2001	1
FALSE	1011	1	1989	1996	1
FALSE	987	1	1987	2000	1
FALSE	686	1	1997	1999	1

FALSE	376	1	1998	2002	1
FALSE	831	1	1998	2002	1
FALSE	855	1	1998	2002	1
FALSE	148	1	1997	2001	1
FALSE	706	1	1990	1996	1
FALSE	676	1	1993	1994	1
FALSE	276	1	1993	2003	1
FALSE	689	1	1995	2002	1
FALSE	532	1	1995	1999	1
FALSE	51	1	1998	2003	1
FALSE	245	1	1998	2003	1
FALSE	698	1	1998	2003	1
FALSE	51	1	1998	2003	1
FALSE	728	1	1998	2003	1
FALSE	698	1	1996	1997	1
FALSE	982	1	1996	1997	1
FALSE	56	1	1996	1999	1
FALSE	89	1	1996	1999	1
FALSE	686	1	1999	2002	1
FALSE	727	1	1999	2002	1
FALSE	333	1	1985	2001	1
FALSE	276	1	1992	1994	1
FALSE	719	1	1992	1994	1
FALSE	94	1	1997	1998	1
FALSE	712	1	1997	1998	1
FALSE	698	1	1999	2002	1
FALSE	276	1	1996	1999	1
FALSE	190	1	1995	2003	1
FALSE	21	1	1991	1993	1
FALSE	698	1	1991	1993	1
FALSE	663	1	1989	1996	1
FALSE	879	1	1997	1999	1
FALSE	376	1	1994	2003	1
FALSE	613	1	1994	2003	1
FALSE	698	1	1996	1997	1
FALSE	698	1	1994	2003	1
FALSE	725	1	1994	2003	1
FALSE	698	1	1991	2000	1
FALSE	937	1	1991	2000	1
FALSE	686	1	1988	2002	1
FALSE	732	1	1988	2002	1
FALSE	289	1	1995	2001	1
FALSE	686	1	1995	2001	1
FALSE	698	1	1991	2003	1
FALSE	766	1	1991	2003	1
FALSE	94	1	1999	2002	1
FALSE	94	1	1992	2003	1

FALSE	676	1	1992	2003	1
FALSE	698	1	1991	1997	1
FALSE	336	1	1985	1987	1
FALSE	698	1	1985	1987	1
FALSE	698	1	1998	1999	1
FALSE	786	1	1998	1999	1
FALSE	698	1	1985	2001	1
FALSE	719	1	1985	2001	1
FALSE	686	1	1998	2001	1
FALSE	940	1	1998	2001	1
FALSE	823	1	1997	2002	1
FALSE	698	1	1991	2003	1
FALSE	276	1	1997	1998	1
FALSE	698	1	1998	2001	1
FALSE	940	1	1998	2001	1
FALSE	698	1	1995	2003	1
FALSE	698	1	1998	2000	1
FALSE	797	1	1998	2000	1
FALSE	697	1	1997	2000	1
FALSE	846	1	1994	1999	1
FALSE	385	1	1993	2003	1
FALSE	698	1	1993	2002	1
FALSE	250	1	1998	2000	1
FALSE	506	1	1998	2000	1
FALSE	148	1	1996	2000	1
FALSE	310	1	1996	2000	1
FALSE	376	1	1997	2003	1
FALSE	940	1	1995	1998	1
FALSE	148	1	1996	1998	1
FALSE	450	1	1996	1998	1
FALSE	89	1	1993	2002	1
FALSE	846	1	1993	2002	1
FALSE	750	1	1991	1993	1
FALSE	698	1	1999	2000	1
FALSE	690	1	1999	2003	1
FALSE	148	1	1999	2003	1
FALSE	846	1	1999	2003	1
FALSE	94	1	1997	2000	1
FALSE	698	1	1995	2000	1
FALSE	234	1	1995	1997	1
FALSE	18	1	1993	2003	1
FALSE	555	1	1993	2003	1
FALSE	640	1	1993	2003	1
FALSE	822	1	1993	2003	1
FALSE	698	1	1998	2001	1
FALSE	421	1	1999	2001	1
FALSE	833	1	1999	2001	1

FALSE	500	1	1999	2000	1
FALSE	289	1	1990	2001	1
FALSE	276	1	1995	2002	1
FALSE	533	1	1994	1997	1
FALSE	698	1	1994	1997	1
FALSE	276	1	1996	2003	1
FALSE	610	1	1996	2003	1
FALSE	51	1	1997	2001	1
FALSE	506	1	1997	2001	1
FALSE	94	1	1993	1997	1
FALSE	698	1	1997	1999	1
FALSE	725	1	1997	1999	1
FALSE	51	1	1999	2000	1
FALSE	555	1	1994	2002	1
FALSE	698	1	1994	2002	1
FALSE	266	1	1996	1997	1
FALSE	698	1	1996	1997	1
FALSE	9	1	1993	1996	1
FALSE	480	1	1995	2002	1
FALSE	698	1	1995	2002	1
FALSE	276	1	1998	1999	1
FALSE	533	1	1995	1999	1
FALSE	376	1	1995	2003	1
FALSE	219	1	1989	1997	1
FALSE	51	1	1993	2003	1
FALSE	698	1	1997	2002	1
FALSE	398	1	1996	2003	1
FALSE	94	1	1999	2003	1
FALSE	376	1	1999	2003	1
FALSE	28	1	1997	1999	1
FALSE	979	1	1997	1999	1
FALSE	980	1	1997	1999	1
FALSE	987	1	1997	1999	1
FALSE	878	1	1996	1997	1
FALSE	886	1	1996	1997	1
FALSE	280	1	1985	1988	1
FALSE	685	1	1995	2003	1
FALSE	74	1	1993	2003	1
FALSE	987	1	1993	2003	1
FALSE	51	1	1986	2000	1
FALSE	276	1	1997	1999	1
FALSE	276	1	1997	2003	1
FALSE	231	1	1996	2000	1
FALSE	685	1	1989	2001	1
FALSE	686	1	1989	2001	1
FALSE	320	1	1996	1997	1
FALSE	373	1	1996	1997	1

FALSE	854	1	1993	2002	1
FALSE	148	1	1986	2003	1
FALSE	135	1	1990	2002	1
FALSE	698	1	1997	2003	1
FALSE	470	1	1996	2002	1
FALSE	689	1	1996	2002	1
FALSE	749	1	1996	1997	1
FALSE	749	1	1998	2001	1
FALSE	698	1	1998	2000	1
FALSE	939	1	1995	1996	1
FALSE	252	1	1999	2002	1
FALSE	698	1	1999	2002	1
FALSE	148	1	1999	2002	1
FALSE	698	1	1991	2003	1
FALSE	775	1	1997	2000	1
FALSE	159	1	1997	2000	1
FALSE	378	1	1997	2000	1
FALSE	172	1	1995	1997	1
FALSE	230	1	1995	1997	1
FALSE	587	1	1996	2002	1
FALSE	749	1	1996	2002	1
FALSE	260	1	1996	2001	1
FALSE	698	1	1996	2001	1
FALSE	248	1	1995	2001	1
FALSE	698	1	1995	2001	1
FALSE	89	1	1996	2000	1
FALSE	698	1	1989	2003	1
FALSE	567	1	1998	2003	1
FALSE	698	1	1998	2003	1
FALSE	180	1	1997	2000	1
FALSE	362	1	1999	2002	1
FALSE	698	1	1999	2002	1
FALSE	383	1	1995	1997	1
FALSE	980	1	1995	1997	1
FALSE	404	1	1984	2002	1
FALSE	243	1	1998	2001	1
FALSE	698	1	1998	2001	1
FALSE	1037	1	1996	2001	1
FALSE	752	1	1984	2003	1
FALSE	734	1	1995	1999	1
FALSE	686	1	1999	2002	1
FALSE	89	1	1994	1997	1
FALSE	405	1	1995	2003	1
FALSE	788	1	1993	1998	1
FALSE	847	1	1993	2001	1
FALSE	848	1	1993	2001	1
FALSE	698	1	1992	1995	1

FALSE	698	1	1997	2002	1
FALSE	318	1	1989	2003	1
FALSE	449	1	1997	2003	1
FALSE	840	1	1997	2003	1
FALSE	361	1	1987	2002	1
FALSE	323	1	1999	2003	1
FALSE	745	1	1999	2003	1
FALSE	997	1	1999	2003	1
FALSE	698	1	1993	2001	1
FALSE	741	1	1993	2001	1
FALSE	148	1	1999	2000	1
FALSE	941	1	1994	1998	1
FALSE	94	1	1989	2000	1
FALSE	289	1	1992	2002	1
FALSE	698	1	1992	2002	1
FALSE	999	1	1992	2002	1
FALSE	94	1	1990	2002	1
FALSE	686	1	1989	2002	1
FALSE	376	1	1993	1994	1
FALSE	676	1	1993	1994	1
FALSE	698	1	1998	2003	1
FALSE	725	1	1998	2003	1
FALSE	51	1	1999	2001	1
FALSE	685	1	1993	2003	1
FALSE	693	1	1993	2003	1
FALSE	376	1	1999	2003	1
FALSE	376	1	1998	2003	1
FALSE	686	1	1997	1999	1
FALSE	685	1	1993	2003	1
FALSE	21	1	1999	2000	1
FALSE	94	1	1999	2000	1
FALSE	112	1	1988	1990	1
FALSE	262	1	1988	1990	1
FALSE	698	1	1988	1990	1
FALSE	698	1	1993	2003	1
FALSE	276	1	1993	1996	1
FALSE	698	1	1992	2003	1
FALSE	698	1	1989	2003	1
FALSE	698	1	1990	1995	1
FALSE	339	1	1995	2001	1
FALSE	53	1	1999	2000	1
FALSE	315	1	1999	2000	1
FALSE	698	1	1996	1999	1
FALSE	488	1	1989	2001	1
FALSE	698	1	1989	2001	1
FALSE	686	1	1996	2003	1
FALSE	94	1	1994	2002	1

FALSE	291	1	1994	2002	1
FALSE	855	1	1994	2002	1
FALSE	89	1	1992	1998	1
FALSE	130	1	1992	1998	1
FALSE	749	1	1992	1998	1
FALSE	698	1	1997	2001	1
FALSE	725	1	1997	2001	1
FALSE	698	1	1995	2003	1
FALSE	272	1	1997	1998	1
FALSE	685	1	1997	1998	1
FALSE	89	1	1995	1998	1
FALSE	639	1	1995	1998	1
FALSE	728	1	1995	1998	1
FALSE	51	1	1989	2003	1
FALSE	698	1	1992	2001	1
FALSE	148	1	1997	2003	1
FALSE	276	1	1997	2003	1
FALSE	383	1	1997	1999	1
FALSE	698	1	1997	1999	1
FALSE	935	1	1997	1999	1
FALSE	103	1	1998	2002	1
FALSE	30	1	1999	2000	1
FALSE	686	1	1999	2000	1
FALSE	493	1	1990	2001	1
FALSE	94	1	1998	2001	1
FALSE	689	1	1998	2001	1
FALSE	685	1	1991	1995	1
FALSE	686	1	1991	1995	1
FALSE	405	1	1995	2001	1
FALSE	697	1	1995	2001	1
FALSE	420	1	1996	2001	1
FALSE	89	1	1988	2003	1
FALSE	686	1	1987	2003	1
FALSE	725	1	1999	2000	1
FALSE	51	1	1992	2002	1
FALSE	51	1	1993	1994	1
FALSE	94	1	1996	2003	1
FALSE	135	1	1996	2003	1
FALSE	15	1	1993	2003	1
FALSE	51	1	1993	2003	1
FALSE	497	1	1993	2003	1
FALSE	697	1	1993	2003	1
FALSE	677	1	1997	1998	1
FALSE	148	1	1990	1999	1
FALSE	961	1	1990	1999	1
FALSE	148	1	1995	1998	1
FALSE	148	1	1996	2000	1

FALSE	148	1	1996	2003	1
FALSE	94	1	1991	2003	1
FALSE	670	1	1991	2003	1
FALSE	698	1	1991	2003	1
FALSE	89	1	1986	1995	1
FALSE	698	1	1999	2001	1
FALSE	241	1	1997	2001	1
FALSE	252	1	1998	1999	1
FALSE	698	1	1998	1999	1
FALSE	51	1	1993	1994	1
FALSE	58	1	1987	2003	1
FALSE	698	1	1987	2003	1
FALSE	839	1	1987	2003	1
FALSE	488	1	1999	2003	1
FALSE	971	1	1999	2003	1
FALSE	555	1	1995	2002	1
FALSE	698	1	1995	2002	1
FALSE	94	1	1995	2003	1
FALSE	971	1	1995	2003	1
FALSE	725	1	1996	2002	1
FALSE	872	1	1996	2002	1
FALSE	89	1	1999	2000	1
FALSE	987	1	1999	2000	1
FALSE	94	1	1993	2003	1
FALSE	686	1	1992	2001	1
FALSE	94	1	1998	2003	1
FALSE	170	1	1998	2003	1
FALSE	362	1	1998	2003	1
FALSE	752	1	1998	2003	1
FALSE	89	1	1987	2002	1
FALSE	749	1	1987	2002	1
FALSE	376	1	1998	2001	1
FALSE	51	1	1995	2002	1
FALSE	94	1	1995	2002	1
FALSE	448	1	1996	1999	1
FALSE	89	1	1990	2003	1
FALSE	698	1	1990	2003	1
FALSE	698	1	1991	2001	1
FALSE	686	1	1998	2001	1
FALSE	93	1	1995	2003	1
FALSE	94	1	1996	2001	1
FALSE	432	1	1996	2001	1
FALSE	698	1	1992	2002	1
FALSE	64	1	1985	2003	1
FALSE	94	1	1985	2003	1
FALSE	94	1	1996	1997	1
FALSE	369	1	1996	1997	1

FALSE	755	1	1985	2003	1
FALSE	698	1	1994	1996	1
FALSE	698	1	1990	2003	1
FALSE	698	1	1998	2000	1
FALSE	689	1	1990	2003	1
FALSE	698	1	1985	2003	1
FALSE	27	1	1994	2001	1
FALSE	1062	1	1994	2001	1
FALSE	825	1	1994	2001	1
FALSE	855	1	1994	2001	1
FALSE	94	1	1990	2001	1
FALSE	516	1	1990	2001	1
FALSE	887	1	1990	2001	1
FALSE	904	1	1990	2001	1
FALSE	698	1	1997	2001	1
FALSE	687	1	1998	2003	1
FALSE	351	1	1998	1999	1
FALSE	727	1	1998	1999	1
FALSE	698	1	1999	2000	1
FALSE	346	1	1990	2003	1
FALSE	383	1	1990	2003	1
FALSE	698	1	1990	2003	1
FALSE	750	1	1990	2003	1
FALSE	276	1	1994	2003	1
FALSE	289	1	1995	1998	1
FALSE	289	1	1996	2003	1
FALSE	997	1	1996	2003	1
FALSE	698	1	1991	2002	1
FALSE	202	1	1995	1997	1
FALSE	1011	1	1995	1997	1
FALSE	698	1	1995	1997	1
FALSE	1011	1	1995	1997	1
FALSE	276	1	1993	2002	1
FALSE	698	1	1991	1992	1
FALSE	813	1	1991	1992	1
FALSE	893	1	1995	2000	1
FALSE	376	1	1987	2003	1
FALSE	698	1	1994	2003	1
FALSE	240	1	1995	1999	1
FALSE	698	1	1994	1997	1
FALSE	706	1	1994	1997	1
FALSE	531	1	1999	2003	1
FALSE	698	1	1999	2003	1
FALSE	990	1	1999	2003	1
FALSE	94	1	1988	2003	1
FALSE	685	1	1988	2003	1
FALSE	89	1	1998	2003	1

FALSE	669	1	1998	2003	1
FALSE	94	1	1997	2002	1
FALSE	621	1	1988	1991	1
FALSE	94	1	1999	2002	1
FALSE	147	1	1999	2002	1
FALSE	686	1	1990	1996	1
FALSE	911	1	1990	1996	1
FALSE	686	1	1994	2000	1
FALSE	823	1	1998	1999	1
FALSE	276	1	1998	2002	1
FALSE	698	1	1995	2003	1
FALSE	725	1	1995	2003	1
FALSE	698	1	1997	2000	1
FALSE	893	1	1997	2000	1
FALSE	545	1	1997	1998	1
FALSE	985	1	1997	1998	1
FALSE	232	1	1990	1992	1
FALSE	1061	1	1990	1992	1
FALSE	517	1	1997	1999	1
FALSE	771	1	1997	1999	1
FALSE	698	1	1996	2003	1
FALSE	752	1	1996	2003	1
FALSE	1027	1	1986	1997	1
FALSE	690	1	1994	2003	1
FALSE	724	1	1994	2003	1
FALSE	725	1	1994	2003	1
FALSE	888	1	1996	1999	1
FALSE	686	1	1995	1997	1
FALSE	795	1	1995	1997	1
FALSE	130	1	1993	1996	1
FALSE	476	1	1993	1996	1
FALSE	94	1	1985	2003	1
FALSE	854	1	1985	2003	1
FALSE	715	1	1994	1999	1
FALSE	684	1	1988	1992	1
FALSE	698	1	1988	1992	1
FALSE	148	1	1999	2002	1
FALSE	376	1	1999	2003	1
FALSE	465	1	1998	2001	1
FALSE	698	1	1998	2001	1
FALSE	689	1	1999	2002	1
FALSE	94	1	1998	2001	1
FALSE	94	1	1989	2003	1
FALSE	89	1	1996	2003	1
FALSE	987	1	1996	2003	1
FALSE	891	1	1993	1994	1
FALSE	698	1	1987	2003	1

FALSE	289	1	1995	2003	1
FALSE	698	1	1995	2003	1
FALSE	94	1	1992	1999	1
FALSE	89	1	1990	2003	1
FALSE	130	1	1990	2003	1
FALSE	232	1	1990	2003	1
FALSE	698	1	1996	2003	1
FALSE	745	1	1996	2003	1
FALSE	698	1	1992	1999	1
FALSE	376	1	1999	2002	1
FALSE	698	1	1999	2002	1
FALSE	301	1	1995	2001	1
FALSE	689	1	1995	2001	1
FALSE	854	1	1995	2001	1
FALSE	94	1	1997	2003	1
FALSE	51	1	1999	2001	1
FALSE	276	1	1999	2001	1
FALSE	686	1	1990	2003	1
FALSE	698	1	1987	1994	1
FALSE	148	1	1993	2003	1
FALSE	698	1	1994	2001	1
FALSE	698	1	1987	2002	1
FALSE	51	1	1992	1994	1
FALSE	596	1	1992	1994	1
FALSE	51	1	1999	2003	1
FALSE	698	1	1996	2002	1
FALSE	376	1	1987	2003	1
FALSE	698	1	1987	2003	1
FALSE	51	1	1994	1999	1
FALSE	148	1	1999	2001	1
FALSE	275	1	1999	2001	1
FALSE	688	1	1999	2001	1
FALSE	698	1	1999	2001	1
FALSE	51	1	1994	2003	1
FALSE	94	1	1999	2002	1
FALSE	361	1	1999	2002	1
FALSE	900	1	1999	2002	1
FALSE	686	1	1997	2003	1
FALSE	89	1	1997	2003	1
FALSE	94	1	1999	2003	1
FALSE	94	1	1998	2001	1
FALSE	855	1	1998	2001	1
FALSE	148	1	1998	2003	1
FALSE	276	1	1998	2003	1
FALSE	799	1	1998	2003	1
FALSE	668	1	1994	1998	1
FALSE	698	1	1993	1996	1

FALSE	94	1	1989	2000	1
FALSE	51	1	1988	2003	1
FALSE	108	1	1995	1999	1
FALSE	698	1	1994	2003	1
FALSE	749	1	1989	1992	1
FALSE	130	1	1995	2003	1
FALSE	686	1	1995	2003	1
FALSE	94	1	1984	2003	1
FALSE	6	1	1997	2001	1
FALSE	987	1	1997	2001	1
FALSE	482	1	1993	2002	1
FALSE	698	1	1993	2002	1
FALSE	94	1	1984	2003	1
FALSE	698	1	1992	2003	1
FALSE	689	1	1992	2000	1
FALSE	748	1	1992	2000	1
FALSE	94	1	1994	2002	1
FALSE	89	1	1994	2002	1
FALSE	690	1	1999	2000	1
FALSE	689	1	1998	2000	1
FALSE	822	1	1995	2003	1
FALSE	698	1	1995	1997	1
FALSE	89	1	1996	2002	1
FALSE	918	1	1996	2002	1
FALSE	103	1	1990	1997	1
FALSE	698	1	1990	2003	1
FALSE	156	1	1995	2001	1
FALSE	698	1	1996	2003	1
FALSE	698	1	1985	1998	1
FALSE	51	1	1998	2001	1
FALSE	686	1	1987	2003	1
FALSE	698	1	1987	2003	1
FALSE	798	1	1998	1999	1
FALSE	94	1	1996	1999	1
FALSE	689	1	1993	2003	1
FALSE	698	1	1986	2002	1
FALSE	276	1	1998	2001	1
FALSE	94	1	1999	2003	1
FALSE	103	1	1999	2003	1
FALSE	949	1	1999	2003	1
FALSE	30	1	1995	1997	1
FALSE	686	1	1995	1997	1
FALSE	686	1	1995	2002	1
FALSE	148	1	1999	2000	1
FALSE	148	1	1998	2003	1
FALSE	558	1	1998	2003	1
FALSE	148	1	1994	1999	1

FALSE	450	1	1994	1999	1
FALSE	94	1	1997	2003	1
FALSE	698	1	1990	2001	1
FALSE	272	1	1986	2002	1
FALSE	94	1	1998	1999	1
FALSE	698	1	1998	1999	1
FALSE	92	1	1993	1999	1
FALSE	94	1	1985	2003	1
FALSE	51	1	1999	2003	1
FALSE	94	1	1999	2003	1
FALSE	194	1	1999	2003	1
FALSE	688	1	1999	2003	1
FALSE	173	1	1994	2000	1
FALSE	294	1	1994	2000	1
FALSE	686	1	1994	2000	1
FALSE	775	1	1995	2003	1
FALSE	148	1	1998	2003	1
FALSE	690	1	1998	2003	1
FALSE	725	1	1998	2003	1
FALSE	698	1	1995	1997	1
FALSE	940	1	1995	1997	1
FALSE	94	1	1984	1996	1
FALSE	94	1	1985	2003	1
FALSE	435	1	1997	1999	1
FALSE	1059	1	1997	1999	1
FALSE	130	1	1998	2001	1
FALSE	940	1	1998	2001	1
FALSE	82	1	1995	2001	1
FALSE	387	1	1995	2001	1
FALSE	698	1	1995	2001	1
FALSE	755	1	1999	2001	1
FALSE	923	1	1990	2001	1
FALSE	376	1	1999	2002	1
FALSE	47	1	1992	2003	1
FALSE	276	1	1992	2003	1
FALSE	613	1	1992	2003	1
FALSE	871	1	1992	2003	1
FALSE	698	1	1992	1994	1
FALSE	376	1	1992	2003	1
FALSE	698	1	1992	2003	1
FALSE	467	1	1997	2002	1
FALSE	698	1	1997	2002	1
FALSE	698	1	1998	1999	1
FALSE	686	1	1985	2003	1
FALSE	698	1	1985	2003	1
FALSE	698	1	1992	2001	1
FALSE	987	1	1992	2001	1

FALSE	686	1	1988	2001	1
FALSE	698	1	1988	2001	1
FALSE	727	1	1997	1998	1
FALSE	94	1	1996	2002	1
FALSE	712	1	1996	2002	1
FALSE	901	1	1996	2002	1
FALSE	1001	1	1996	2002	1
FALSE	698	1	1997	1998	1
FALSE	698	1	1996	2002	1
FALSE	276	1	1999	2002	1
FALSE	987	1	1994	2002	1
FALSE	698	1	1997	1999	1
FALSE	823	1	1996	2001	1
FALSE	698	1	1995	1999	1
FALSE	698	1	1994	1999	1
FALSE	51	1	1996	1998	1
FALSE	698	1	1996	1998	1
FALSE	276	1	1992	1993	1
FALSE	555	1	1992	1993	1
FALSE	753	1	1992	1993	1
FALSE	689	1	1990	1991	1
FALSE	887	1	1990	1991	1
FALSE	32	1	1998	2000	1
FALSE	698	1	1998	2000	1
FALSE	698	1	1988	1993	1
FALSE	94	1	1999	2003	1
FALSE	698	1	1988	2003	1
FALSE	698	1	1988	2001	1
FALSE	491	1	1987	1992	1
FALSE	89	1	1998	2000	1
FALSE	669	1	1998	2000	1
FALSE	389	1	1997	1999	1
FALSE	663	1	1997	1999	1
FALSE	94	1	1993	2003	1
FALSE	728	1	1993	2003	1
FALSE	686	1	1996	1997	1
FALSE	698	1	1994	2003	1
FALSE	51	1	1995	2001	1
FALSE	94	1	1995	2001	1
FALSE	272	1	1999	2002	1
FALSE	728	1	1995	1998	1
FALSE	376	1	1996	2002	1
FALSE	685	1	1996	2002	1
FALSE	376	1	1999	2001	1
FALSE	698	1	1990	2003	1
FALSE	94	1	1994	2003	1
FALSE	689	1	1994	2003	1

FALSE	840	1	1994	2003	1
FALSE	254	1	1997	1999	1
FALSE	383	1	1997	1999	1
FALSE	934	1	1997	1999	1
FALSE	987	1	1990	1991	1
FALSE	689	1	1995	2000	1
FALSE	698	1	1992	2002	1
FALSE	1011	1	1996	2003	1
FALSE	698	1	1987	2002	1
FALSE	698	1	1997	1999	1
FALSE	888	1	1997	1999	1
FALSE	822	1	1997	2003	1
FALSE	718	1	1995	1996	1
FALSE	318	1	1999	2000	1
FALSE	698	1	1999	2000	1
FALSE	344	1	1999	2001	1
FALSE	585	1	1999	2001	1
FALSE	94	1	1998	2000	1
FALSE	842	1	1998	2000	1
FALSE	89	1	1997	1999	1
FALSE	888	1	1997	1999	1
FALSE	1011	1	1995	2003	1
FALSE	954	1	1990	2003	1
FALSE	376	1	1995	2002	1
FALSE	337	1	1997	2001	1
FALSE	555	1	1993	1995	1
FALSE	698	1	1993	1995	1
FALSE	94	1	1987	1994	1
FALSE	1027	1	1987	1994	1
FALSE	698	1	1996	1999	1
FALSE	539	1	1994	2000	1
FALSE	698	1	1994	2000	1
FALSE	504	1	1999	2001	1
FALSE	94	1	1991	1998	1
FALSE	698	1	1990	1999	1
FALSE	728	1	1998	2000	1
FALSE	749	1	1998	2000	1
FALSE	686	1	1986	1990	1
FALSE	686	1	1997	1999	1
FALSE	94	1	1997	2003	1
FALSE	698	1	1989	2003	1
FALSE	686	1	1993	2003	1
FALSE	685	1	1989	2003	1
FALSE	686	1	1989	2003	1
FALSE	430	1	1999	2000	1
FALSE	148	1	1996	2003	1
FALSE	176	1	1995	2002	1

FALSE	672	1	1999	2001	1
FALSE	823	1	1999	2001	1
FALSE	982	1	1997	2000	1
FALSE	557	1	1998	2000	1
FALSE	720	1	1998	2000	1
FALSE	842	1	1998	2000	1
FALSE	297	1	1995	2001	1
FALSE	698	1	1998	1999	1
FALSE	289	1	1999	2001	1
FALSE	724	1	1999	2001	1
FALSE	94	1	1995	2003	1
FALSE	289	1	1999	2000	1
FALSE	376	1	1999	2000	1
FALSE	94	1	1992	2003	1
FALSE	698	1	1997	2003	1
FALSE	875	1	1996	2002	1
FALSE	148	1	1989	2003	1
FALSE	108	1	1994	2002	1
FALSE	686	1	1994	2002	1
FALSE	89	1	1998	2000	1
FALSE	902	1	1998	2000	1
FALSE	698	1	1992	2001	1
FALSE	94	1	1993	2003	1
FALSE	289	1	1998	2001	1
FALSE	698	1	1998	2003	1
FALSE	932	1	1998	2003	1
FALSE	94	1	1996	1998	1
FALSE	289	1	1996	1998	1
FALSE	686	1	1996	1998	1
FALSE	94	1	1996	2001	1
FALSE	686	1	1998	1999	1
FALSE	698	1	1991	2003	1
FALSE	939	1	1991	2003	1
FALSE	698	1	1998	1999	1
FALSE	148	1	1999	2002	1
FALSE	698	1	1990	2003	1
FALSE	94	1	1998	2000	1
FALSE	361	1	1998	2000	1
FALSE	698	1	1998	2000	1
FALSE	131	1	1985	1996	1
FALSE	94	1	1996	2003	1
FALSE	555	1	1995	2003	1
FALSE	698	1	1988	2003	1
FALSE	698	1	1987	2002	1
FALSE	435	1	1992	1998	1
FALSE	698	1	1992	1998	1
FALSE	687	1	1997	1999	1

FALSE	376	1	1990	2001	1
FALSE	227	1	1992	1993	1
FALSE	689	1	1992	1993	1
FALSE	690	1	1992	1993	1
FALSE	698	1	1996	2003	1
FALSE	376	1	1997	2001	1
FALSE	618	1	1997	2001	1
FALSE	619	1	1997	2001	1
FALSE	698	1	1996	2001	1
FALSE	698	1	1994	1997	1
FALSE	376	1	1998	1999	1
FALSE	1027	1	1998	1999	1
FALSE	94	1	1993	1995	1
FALSE	1027	1	1993	1995	1
FALSE	376	1	1990	1992	1
FALSE	698	1	1990	1992	1
FALSE	58	1	1995	1997	1
FALSE	148	1	1995	1997	1
FALSE	698	1	1998	2003	1
FALSE	130	1	1995	2003	1
FALSE	148	1	1995	2003	1
FALSE	685	1	1993	1999	1
FALSE	698	1	1995	2002	1
FALSE	925	1	1990	1996	1
FALSE	51	1	1996	2003	1
FALSE	272	1	1992	2001	1
FALSE	376	1	1996	1999	1
FALSE	698	1	1996	1999	1
FALSE	51	1	1987	2002	1
FALSE	689	1	1996	2003	1
FALSE	1035	1	1998	2001	1
FALSE	51	1	1993	2003	1
FALSE	963	1	1999	2002	1
FALSE	51	1	1995	2003	1
FALSE	698	1	1998	2000	1
FALSE	465	1	1996	2001	1
FALSE	698	1	1996	2001	1
FALSE	147	1	1999	2003	1
FALSE	698	1	1989	1992	1
FALSE	698	1	1998	2003	1
FALSE	130	1	1997	1998	1
FALSE	687	1	1997	1998	1
FALSE	686	1	1997	2003	1
FALSE	698	1	1997	2003	1
FALSE	725	1	1997	2003	1
FALSE	225	1	1994	2003	1
FALSE	338	1	1994	2003	1

FALSE	505	1	1994	2003	1
FALSE	727	1	1994	2003	1
FALSE	1004	1	1994	2003	1
FALSE	51	1	1998	2001	1
FALSE	148	1	1998	2001	1
FALSE	456	1	1996	2003	1
FALSE	689	1	1997	1999	1
FALSE	698	1	1993	1997	1
FALSE	698	1	1993	1999	1
FALSE	376	1	1996	1997	1
FALSE	698	1	1996	1997	1
FALSE	991	1	1999	2001	1
FALSE	94	1	1994	2001	1
FALSE	698	1	1998	2001	1
FALSE	698	1	1997	2001	1
FALSE	685	1	1992	2001	1
FALSE	689	1	1989	2002	1
FALSE	376	1	1999	2002	1
FALSE	698	1	1995	2001	1
FALSE	896	1	1995	2001	1
FALSE	73	1	1999	2002	1
FALSE	939	1	1999	2002	1
FALSE	102	1	1995	1998	1
FALSE	698	1	1996	1999	1
FALSE	94	1	1999	2000	1
FALSE	980	1	1999	2000	1
FALSE	94	1	1995	2001	1
FALSE	103	1	1994	2003	1
FALSE	698	1	1994	2003	1
FALSE	690	1	1998	2001	1
FALSE	698	1	1995	2001	1
FALSE	698	1	1998	1999	1
FALSE	982	1	1998	1999	1
FALSE	94	1	1998	2000	1
FALSE	688	1	1998	2000	1
FALSE	698	1	1986	1999	1
FALSE	698	1	1991	1994	1
FALSE	698	1	1985	1986	1
FALSE	719	1	1985	1986	1
FALSE	51	1	1990	2001	1
FALSE	376	1	1992	2003	1
FALSE	685	1	1992	2003	1
FALSE	148	1	1999	2001	1
FALSE	250	1	1999	2001	1
FALSE	822	1	1991	2000	1
FALSE	94	1	1996	2002	1
FALSE	689	1	1998	2000	1

FALSE	831	1	1998	2000	1
FALSE	698	1	1985	1987	1
FALSE	94	1	1998	2003	1
FALSE	361	1	1998	2003	1
FALSE	688	1	1998	2003	1
FALSE	689	1	1998	2003	1
FALSE	51	1	1992	2003	1
FALSE	89	1	1996	2003	1
FALSE	698	1	1996	2003	1
FALSE	987	1	1996	2003	1
FALSE	376	1	1996	1997	1
FALSE	64	1	1989	2001	1
FALSE	260	1	1996	2000	1
FALSE	254	1	1998	2000	1
FALSE	289	1	1998	2000	1
FALSE	887	1	1998	2000	1
FALSE	89	1	1998	2003	1
FALSE	698	1	1998	2003	1
FALSE	718	1	1998	2003	1
FALSE	902	1	1998	2003	1
FALSE	51	1	1992	2003	1
FALSE	276	1	1992	2003	1
FALSE	289	1	1998	2002	1
FALSE	686	1	1998	2002	1
FALSE	94	1	1999	2001	1
FALSE	628	1	1999	2001	1
FALSE	688	1	1999	2001	1
FALSE	935	1	1992	2002	1
FALSE	73	1	1991	1996	1
FALSE	698	1	1991	1996	1
FALSE	94	1	1996	2001	1
FALSE	6	1	1994	1995	1
FALSE	94	1	1996	2002	1
FALSE	698	1	1996	2002	1
FALSE	987	1	1996	2002	1
FALSE	686	1	1999	2002	1
FALSE	686	1	1986	2003	1
FALSE	94	1	1994	2001	1
FALSE	289	1	1995	1998	1
FALSE	613	1	1999	2003	1
FALSE	698	1	1996	2003	1
FALSE	790	1	1996	2003	1
FALSE	51	1	1996	1999	1
FALSE	716	1	1996	1999	1
FALSE	698	1	1999	2000	1
FALSE	686	1	1997	2001	1
FALSE	699	1	1997	2001	1

FALSE	980	1	1995	2003	1
FALSE	987	1	1995	2003	1
FALSE	698	1	1986	2000	1
FALSE	781	1	1986	2000	1
FALSE	1065	1	1986	2000	1
FALSE	456	1	1995	2003	1
FALSE	500	1	1995	2003	1
FALSE	698	1	1995	2003	1
FALSE	686	1	1997	2000	1
FALSE	698	1	1995	1997	1
FALSE	114	1	1994	2001	1
FALSE	572	1	1996	1997	1
FALSE	888	1	1996	1997	1
FALSE	587	1	1998	2000	1
FALSE	376	1	1999	2002	1
FALSE	500	1	1999	2002	1
FALSE	686	1	1994	2001	1
FALSE	639	1	1991	1996	1
FALSE	369	1	1988	2002	1
FALSE	689	1	1996	2003	1
FALSE	685	1	1990	1997	1
FALSE	689	1	1997	1999	1
FALSE	898	1	1994	1996	1
FALSE	318	1	1998	2003	1
FALSE	812	1	1997	1999	1
FALSE	698	1	1997	2003	1
FALSE	780	1	1997	2003	1
FALSE	831	1	1997	2003	1
FALSE	855	1	1997	2003	1
FALSE	927	1	1997	2003	1
FALSE	698	1	1991	1999	1
FALSE	89	1	1996	2003	1
FALSE	987	1	1996	2003	1
FALSE	698	1	1992	1995	1
FALSE	338	1	1996	1997	1
FALSE	698	1	1996	1997	1
FALSE	698	1	1998	1999	1
FALSE	94	1	1999	2003	1
FALSE	688	1	1999	2003	1
FALSE	51	1	1995	2003	1
FALSE	654	1	1988	2002	1
FALSE	376	1	1999	2001	1
FALSE	686	1	1999	2001	1
FALSE	637	1	1998	2002	1
FALSE	839	1	1998	2002	1
FALSE	376	1	1996	1999	1
FALSE	272	1	1991	2003	1

FALSE	707	1	1991	2003	1
FALSE	1009	1	1993	1999	1
FALSE	831	1	1995	2002	1
FALSE	94	1	1995	1996	1
FALSE	855	1	1995	1996	1
FALSE	604	1	1998	1999	1
FALSE	698	1	1998	1999	1
FALSE	559	1	1995	2003	1
FALSE	698	1	1995	2003	1
FALSE	900	1	1998	2000	1
FALSE	987	1	1998	2000	1
FALSE	820	1	1991	2003	1
FALSE	32	1	1999	2001	1
FALSE	376	1	1999	2001	1
FALSE	698	1	1994	2002	1
FALSE	706	1	1986	2003	1
FALSE	276	1	1995	1996	1
FALSE	93	1	1999	2003	1
FALSE	148	1	1999	2003	1
FALSE	94	1	1999	2000	1
FALSE	875	1	1999	2000	1
FALSE	693	1	1993	1995	1
FALSE	698	1	1997	2000	1
FALSE	51	1	1993	2003	1
FALSE	698	1	1998	2003	1
FALSE	89	1	1996	2003	1
FALSE	693	1	1994	1997	1
FALSE	686	1	1997	2001	1
FALSE	94	1	1991	2003	1
FALSE	987	1	1991	2003	1
FALSE	689	1	1994	1998	1
FALSE	840	1	1994	1998	1
FALSE	318	1	1997	1999	1
FALSE	94	1	1997	1998	1
FALSE	289	1	1997	1998	1
FALSE	529	1	1994	1997	1
FALSE	698	1	1994	1997	1
FALSE	1004	1	1990	1996	1
FALSE	698	1	1989	2002	1
FALSE	686	1	1996	2003	1
FALSE	698	1	1985	2001	1
FALSE	698	1	1998	2002	1
FALSE	276	1	1993	2003	1
FALSE	987	1	1996	2003	1
FALSE	698	1	1996	1997	1
FALSE	776	1	1996	1997	1
FALSE	89	1	1998	2002	1

FALSE	686	1	1996	2003	1
FALSE	89	1	1997	2003	1
FALSE	506	1	1997	2003	1
FALSE	388	1	1995	1996	1
FALSE	698	1	1995	1996	1
FALSE	698	1	1999	2002	1
FALSE	519	1	1999	2000	1
FALSE	698	1	1999	2000	1
FALSE	698	1	1992	2003	1
FALSE	698	1	1986	2001	1
FALSE	44	1	1989	2002	1
FALSE	987	1	1988	2002	1
FALSE	94	1	1998	2002	1
FALSE	376	1	1998	2002	1
FALSE	502	1	1998	2002	1
FALSE	94	1	1998	2000	1
FALSE	855	1	1998	2000	1
FALSE	732	1	1999	2003	1
FALSE	752	1	1999	2003	1
FALSE	698	1	1990	1998	1
FALSE	276	1	1996	1998	1
FALSE	822	1	1996	2003	1
FALSE	976	1	1996	2003	1
FALSE	977	1	1996	2003	1
FALSE	698	1	1995	2003	1
FALSE	94	1	1994	2003	1
FALSE	317	1	1994	2003	1
FALSE	317	1	1996	2003	1
FALSE	385	1	1996	2003	1
FALSE	823	1	1996	2003	1
FALSE	1023	1	1996	2003	1
FALSE	698	1	1999	2001	1
FALSE	688	1	1999	2002	1
FALSE	698	1	1999	2002	1
FALSE	309	1	1988	2003	1
FALSE	698	1	1988	2003	1
FALSE	770	1	1994	2000	1
FALSE	94	1	1988	2003	1
FALSE	698	1	1988	2003	1
FALSE	698	1	1995	2000	1
FALSE	659	1	1990	1991	1
FALSE	792	1	1990	1991	1
FALSE	698	1	1998	1999	1
FALSE	698	1	1984	2003	1
FALSE	94	1	1997	2003	1
FALSE	855	1	1997	2003	1
FALSE	987	1	1997	2003	1

FALSE	698	1	1998	2002	1
FALSE	839	1	1998	2002	1
FALSE	953	1	1998	2002	1
FALSE	89	1	1988	1995	1
FALSE	130	1	1988	1995	1
FALSE	698	1	1996	2003	1
FALSE	94	1	1999	2000	1
FALSE	698	1	1999	2000	1
FALSE	888	1	1999	2000	1
FALSE	978	1	1998	1999	1
FALSE	698	1	1985	2001	1
FALSE	698	1	1998	2003	1
FALSE	46	1	1998	2003	1
FALSE	51	1	1998	2003	1
FALSE	698	1	1995	2003	1
FALSE	89	1	1987	2003	1
FALSE	148	1	1995	2003	1
FALSE	686	1	1998	2002	1
FALSE	822	1	1992	1993	1
FALSE	698	1	1999	2003	1
FALSE	987	1	1999	2003	1
FALSE	685	1	1993	2002	1
FALSE	686	1	1993	2002	1
FALSE	51	1	1999	2003	1
FALSE	698	1	1999	2003	1
FALSE	686	1	1989	2001	1
FALSE	698	1	1993	1995	1
FALSE	376	1	1996	2002	1
FALSE	685	1	1996	2002	1
FALSE	30	1	1999	2000	1
FALSE	686	1	1999	2000	1
FALSE	51	1	1992	1999	1
FALSE	276	1	1992	1999	1
FALSE	698	1	1999	2000	1
FALSE	725	1	1999	2000	1
FALSE	89	1	1999	2003	1
FALSE	94	1	1999	2003	1
FALSE	698	1	1999	2003	1
FALSE	51	1	1985	2000	1
FALSE	94	1	1996	2002	1
FALSE	10	1	1999	2002	1
FALSE	276	1	1999	2002	1
FALSE	686	1	1987	2003	1
FALSE	94	1	1998	2000	1
FALSE	831	1	1998	2000	1
FALSE	855	1	1998	2000	1
FALSE	698	1	1998	2001	1

FALSE	689	1	1998	2003	1
FALSE	698	1	1996	2003	1
FALSE	987	1	1988	2001	1
FALSE	698	1	1990	2002	1
FALSE	854	1	1990	2002	1
FALSE	686	1	1994	1997	1
FALSE	148	1	1989	2003	1
FALSE	51	1	1990	2003	1
FALSE	94	1	1999	2003	1
FALSE	698	1	1999	2003	1
FALSE	685	1	1990	2003	1
FALSE	94	1	1994	2002	1
FALSE	823	1	1994	2002	1
FALSE	833	1	1994	2002	1
FALSE	689	1	1998	2003	1
FALSE	94	1	1998	2000	1
FALSE	376	1	1998	2000	1
FALSE	698	1	1991	1999	1
FALSE	698	1	1993	2000	1
FALSE	698	1	1996	2003	1
FALSE	727	1	1999	2001	1
FALSE	822	1	1999	2001	1
FALSE	686	1	1999	2002	1
FALSE	823	1	1998	2003	1
FALSE	698	1	1989	2001	1
FALSE	686	1	1998	2002	1
FALSE	698	1	1992	2003	1
FALSE	686	1	1995	1999	1
FALSE	698	1	1986	2003	1
FALSE	89	1	1993	2003	1
FALSE	551	1	1998	2001	1
FALSE	966	1	1988	1992	1
FALSE	94	1	1985	2003	1
FALSE	686	1	1996	2003	1
FALSE	823	1	1996	2003	1
FALSE	376	1	1989	1995	1
FALSE	693	1	1989	1995	1
FALSE	318	1	1995	2003	1
FALSE	698	1	1995	2003	1
FALSE	855	1	1998	2000	1
FALSE	894	1	1998	2000	1
FALSE	148	1	1998	2001	1
FALSE	273	1	1998	2001	1
FALSE	698	1	1997	2003	1
FALSE	888	1	1997	2003	1
FALSE	987	1	1995	2003	1
FALSE	987	1	1998	1999	1

FALSE	94	1	1996	2003	1
FALSE	689	1	1998	2002	1
FALSE	686	1	1994	1996	1
FALSE	698	1	1993	2003	1
FALSE	823	1	1993	2003	1
FALSE	1069	1	1991	2001	1
FALSE	51	1	1991	1998	1
FALSE	94	1	1996	1999	1
FALSE	688	1	1996	1999	1
FALSE	686	1	1994	2001	1
FALSE	949	1	1994	2001	1
FALSE	276	1	1999	2003	1
FALSE	792	1	1999	2003	1
FALSE	130	1	1999	2001	1
FALSE	304	1	1999	2001	1
FALSE	732	1	1999	2001	1
FALSE	51	1	1998	2002	1
FALSE	94	1	1998	2002	1
FALSE	718	1	1986	2003	1
FALSE	376	1	1998	2003	1
FALSE	449	1	1998	2003	1
FALSE	376	1	1998	2003	1
FALSE	698	1	1998	2003	1
FALSE	698	1	1992	1998	1
FALSE	383	1	1986	2001	1
FALSE	94	1	1997	2003	1
FALSE	971	1	1997	2003	1
FALSE	94	1	1994	2003	1
FALSE	194	1	1994	2003	1
FALSE	189	1	1996	2000	1
FALSE	698	1	1996	2000	1
FALSE	94	1	1986	2003	1
FALSE	689	1	1996	2002	1
FALSE	698	1	1996	2002	1
FALSE	698	1	1988	1993	1
FALSE	94	1	1998	2002	1
FALSE	698	1	1992	2003	1
FALSE	465	1	1998	2001	1
FALSE	990	1	1998	2001	1
FALSE	997	1	1986	1988	1
FALSE	1009	1	1986	1988	1
FALSE	118	1	1995	2003	1
FALSE	698	1	1988	2002	1
FALSE	888	1	1997	2000	1
FALSE	688	1	1999	2003	1
FALSE	698	1	1999	2003	1
FALSE	572	1	1992	2003	1

FALSE	686	1	1992	2003	1
FALSE	965	1	1992	2003	1
FALSE	698	1	1999	2003	1
FALSE	385	1	1992	1993	1
FALSE	720	1	1992	1993	1
FALSE	987	1	1991	1994	1
FALSE	685	1	1990	2003	1
FALSE	698	1	1990	2003	1
FALSE	89	1	1995	2001	1
FALSE	305	1	1995	2001	1
FALSE	94	1	1998	1999	1
FALSE	689	1	1998	1999	1
FALSE	698	1	1996	2003	1
FALSE	94	1	1995	1996	1
FALSE	686	1	1988	2003	1
FALSE	89	1	1997	2002	1
FALSE	698	1	1997	2002	1
FALSE	94	1	1992	2003	1
FALSE	749	1	1992	2003	1
FALSE	94	1	1999	2000	1
FALSE	686	1	1999	2000	1
FALSE	971	1	1999	2000	1
FALSE	94	1	1988	2001	1
FALSE	276	1	1993	1999	1
FALSE	272	1	1992	2003	1
FALSE	94	1	1997	2002	1
FALSE	1001	1	1997	2002	1
FALSE	725	1	1998	1999	1
FALSE	94	1	1996	2001	1
FALSE	94	1	1985	2000	1
FALSE	112	1	1985	2000	1
FALSE	94	1	1995	2001	1
FALSE	831	1	1995	2001	1
FALSE	727	1	1996	2003	1
FALSE	289	1	1992	2003	1
FALSE	999	1	1992	2003	1
FALSE	94	1	1998	2003	1
FALSE	376	1	1998	2003	1
FALSE	751	1	1998	2003	1
FALSE	749	1	1995	2002	1
FALSE	363	1	1996	2002	1
FALSE	418	1	1996	2002	1
FALSE	698	1	1996	2002	1
FALSE	148	1	1987	2002	1
FALSE	94	1	1987	1997	1
FALSE	376	1	1987	1997	1
FALSE	698	1	1987	1997	1

FALSE	881	1	1999	2001	1
FALSE	888	1	1999	2001	1
FALSE	732	1	1989	1993	1
FALSE	987	1	1996	1997	1
FALSE	289	1	1999	2000	1
FALSE	698	1	1999	2000	1
FALSE	837	1	1996	2003	1
FALSE	698	1	1992	2001	1
FALSE	855	1	1998	2002	1
FALSE	686	1	1996	2000	1
FALSE	982	1	1996	2000	1
FALSE	130	1	1997	2000	1
FALSE	775	1	1997	2000	1
FALSE	94	1	1999	2003	1
FALSE	376	1	1999	2003	1
FALSE	855	1	1999	2003	1
FALSE	254	1	1998	2000	1
FALSE	887	1	1998	2000	1
FALSE	698	1	1996	2002	1
FALSE	776	1	1996	2002	1
FALSE	376	1	1997	2001	1
FALSE	816	1	1996	1998	1
FALSE	1065	1	1996	1998	1
FALSE	982	1	1986	2002	1
FALSE	698	1	1999	2000	1
FALSE	470	1	1998	2001	1
FALSE	148	1	1994	2002	1
FALSE	450	1	1994	2002	1
FALSE	982	1	1994	2000	1
FALSE	698	1	1985	2003	1
FALSE	51	1	1999	2001	1
FALSE	481	1	1999	2001	1
FALSE	682	1	1999	2001	1
FALSE	94	1	1998	2001	1
FALSE	533	1	1988	2001	1
FALSE	51	1	1997	2000	1
FALSE	766	1	1995	1997	1
FALSE	94	1	1996	2000	1
FALSE	698	1	1996	2000	1
FALSE	698	1	1995	1996	1
FALSE	818	1	1995	1996	1
FALSE	533	1	1987	1989	1
FALSE	148	1	1995	2003	1
FALSE	135	1	1989	1991	1
FALSE	698	1	1992	2003	1
FALSE	289	1	1992	2003	1
FALSE	124	1	1987	1994	1

FALSE	923	1	1987	1994	1
FALSE	289	1	1984	1994	1
FALSE	923	1	1984	1994	1
FALSE	587	1	1997	2003	1
FALSE	276	1	1994	1998	1
FALSE	553	1	1994	1998	1
FALSE	148	1	1989	2003	1
FALSE	239	1	1994	1999	1
FALSE	585	1	1994	1999	1
FALSE	698	1	1994	1999	1
FALSE	322	1	1988	2000	1
FALSE	500	1	1998	2000	1
FALSE	698	1	1998	2000	1
FALSE	749	1	1997	2001	1
FALSE	488	1	1999	2000	1
FALSE	940	1	1996	1998	1
FALSE	963	1	1996	1998	1
FALSE	89	1	1994	2002	1
FALSE	698	1	1994	1998	1
FALSE	272	1	1999	2001	1
FALSE	306	1	1999	2001	1
FALSE	82	1	1995	1997	1
FALSE	387	1	1995	1997	1
FALSE	698	1	1995	1997	1
FALSE	276	1	1998	2002	1
FALSE	804	1	1998	1999	1
FALSE	51	1	1995	1997	1
FALSE	51	1	1999	2001	1
FALSE	94	1	1999	2001	1
FALSE	312	1	1999	2001	1
FALSE	51	1	1998	2003	1
FALSE	689	1	1997	2003	1
FALSE	698	1	1997	2003	1
FALSE	51	1	1990	2002	1
FALSE	94	1	1990	2002	1
FALSE	946	1	1997	1999	1
FALSE	987	1	1997	1999	1
FALSE	279	1	1996	1998	1
FALSE	321	1	1996	1998	1
FALSE	220	1	1995	2000	1
FALSE	698	1	1988	2003	1
FALSE	698	1	1995	1996	1
FALSE	706	1	1995	1996	1
FALSE	66	1	1991	1996	1
FALSE	81	1	1995	1998	1
FALSE	698	1	1993	2002	1
FALSE	488	1	1999	2002	1

FALSE	698	1	1999	2002	1
FALSE	130	1	1997	1998	1
FALSE	143	1	1997	1998	1
FALSE	686	1	1995	1998	1
FALSE	786	1	1996	2000	1
FALSE	387	1	1995	2002	1
FALSE	698	1	1995	2002	1
FALSE	685	1	1992	2003	1
FALSE	686	1	1992	2003	1
FALSE	823	1	1992	2003	1
FALSE	456	1	1995	2003	1
FALSE	500	1	1995	2003	1
FALSE	686	1	1995	2003	1
FALSE	698	1	1995	2003	1
FALSE	686	1	1992	1999	1
FALSE	148	1	1987	2003	1
FALSE	698	1	1992	2003	1
FALSE	1010	1	1992	2003	1
FALSE	689	1	1999	2003	1
FALSE	94	1	1998	2001	1
FALSE	94	1	1995	2003	1
FALSE	822	1	1995	2003	1
FALSE	686	1	1998	1999	1
FALSE	362	1	1992	1994	1
FALSE	572	1	1997	2000	1
FALSE	643	1	1996	1999	1
FALSE	698	1	1996	1999	1
FALSE	823	1	1997	2003	1
FALSE	94	1	1992	2003	1
FALSE	51	1	1998	2003	1
FALSE	94	1	1998	2003	1
FALSE	987	1	1998	2003	1
FALSE	89	1	1997	2003	1
FALSE	698	1	1997	2003	1
FALSE	89	1	1998	2003	1
FALSE	51	1	1989	1997	1
FALSE	698	1	1997	2001	1
FALSE	66	1	1998	2000	1
FALSE	1034	1	1998	2000	1
FALSE	94	1	1996	1999	1
FALSE	1017	1	1996	1997	1
FALSE	148	1	1996	2003	1
FALSE	148	1	1992	2002	1
FALSE	276	1	1998	2002	1
FALSE	725	1	1998	2002	1
FALSE	929	1	1998	2002	1
FALSE	698	1	1995	2002	1

FALSE	335	1	1993	1995	1
FALSE	698	1	1993	1995	1
FALSE	614	1	1994	2003	1
FALSE	148	1	1998	2003	1
FALSE	43	1	1994	1998	1
FALSE	285	1	1997	1998	1
FALSE	776	1	1997	1998	1
FALSE	94	1	1989	1995	1
FALSE	466	1	1989	1995	1
FALSE	749	1	1994	1998	1
FALSE	685	1	1984	2003	1
FALSE	706	1	1992	1994	1
FALSE	698	1	1994	2003	1
FALSE	235	1	1999	2002	1
FALSE	698	1	1998	2001	1
FALSE	996	1	1998	2001	1
FALSE	28	1	1995	1999	1
FALSE	808	1	1995	1999	1
FALSE	376	1	1999	2003	1
FALSE	698	1	1992	2003	1
FALSE	276	1	1991	1993	1
FALSE	698	1	1994	1998	1
FALSE	423	1	1993	1994	1
FALSE	424	1	1993	1994	1
FALSE	698	1	1994	2003	1
FALSE	698	1	1989	1995	1
FALSE	686	1	1993	1998	1
FALSE	425	1	1995	1999	1
FALSE	987	1	1990	1992	1
FALSE	94	1	1998	2003	1
FALSE	289	1	1999	2003	1
FALSE	698	1	1996	2003	1
FALSE	698	1	1992	2003	1
FALSE	78	1	1993	1994	1
FALSE	79	1	1993	1994	1
FALSE	987	1	1989	1992	1
FALSE	207	1	1999	2003	1
FALSE	698	1	1999	2003	1
FALSE	89	1	1986	2003	1
FALSE	276	1	1989	2003	1
FALSE	698	1	1992	1999	1
FALSE	686	1	1985	2002	1
FALSE	698	1	1994	2002	1
FALSE	676	1	1997	2002	1
FALSE	698	1	1997	2002	1
FALSE	753	1	1997	2002	1
FALSE	994	1	1997	2002	1

FALSE	51	1	1998	2001	1
FALSE	52	1	1998	2001	1
FALSE	276	1	1995	2000	1
FALSE	94	1	1993	2003	1
FALSE	94	1	1998	2000	1
FALSE	955	1	1998	2000	1
FALSE	647	1	1995	2002	1
FALSE	823	1	1994	2003	1
FALSE	148	1	1991	1998	1
FALSE	94	1	1995	1998	1
FALSE	712	1	1995	1998	1
FALSE	376	1	1999	2003	1
FALSE	94	1	1995	2003	1
FALSE	698	1	1995	2003	1
FALSE	89	1	1999	2003	1
FALSE	395	1	1999	2003	1
FALSE	698	1	1999	2003	1
FALSE	823	1	1999	2003	1
FALSE	888	1	1991	1997	1
FALSE	130	1	1988	2003	1
FALSE	698	1	1988	2003	1
FALSE	376	1	1999	2000	1
FALSE	267	1	1991	1999	1
FALSE	276	1	1991	1999	1
FALSE	621	1	1991	1999	1
FALSE	488	1	1995	2001	1
FALSE	823	1	1995	2001	1
FALSE	94	1	1993	2002	1
FALSE	687	1	1993	2003	1
FALSE	917	1	1990	1998	1
FALSE	698	1	1986	2001	1
FALSE	51	1	1992	2003	1
FALSE	831	1	1995	1998	1
FALSE	855	1	1995	1998	1
FALSE	698	1	1999	2000	1
FALSE	994	1	1999	2000	1
FALSE	698	1	1985	1989	1
FALSE	698	1	1989	2003	1
FALSE	572	1	1992	1996	1
FALSE	456	1	1995	2000	1
FALSE	500	1	1995	2000	1
FALSE	698	1	1995	2000	1
FALSE	394	1	1991	1998	1
FALSE	813	1	1991	1998	1
FALSE	816	1	1997	1998	1
FALSE	822	1	1997	2003	1
FALSE	376	1	1998	1999	1

FALSE	831	1	1998	1999	1
FALSE	376	1	1989	1991	1
FALSE	409	1	1999	2001	1
FALSE	535	1	1999	2001	1
FALSE	698	1	1995	2003	1
FALSE	997	1	1992	1998	1
FALSE	127	1	1994	1998	1
FALSE	689	1	1994	1998	1
FALSE	51	1	1995	2002	1
FALSE	539	1	1995	2002	1
FALSE	698	1	1995	2002	1
FALSE	840	1	1995	2002	1
FALSE	898	1	1995	2002	1
FALSE	376	1	1993	2002	1
FALSE	869	1	1995	2001	1
FALSE	987	1	1999	2002	1
FALSE	686	1	1997	2002	1
FALSE	823	1	1994	1997	1
FALSE	886	1	1994	1997	1
FALSE	94	1	1996	1998	1
FALSE	792	1	1996	1998	1
FALSE	376	1	1995	2002	1
FALSE	971	1	1995	2002	1
FALSE	94	1	1995	1999	1
FALSE	987	1	1994	1998	1
FALSE	698	1	1993	1999	1
FALSE	376	1	1996	2003	1
FALSE	341	1	1996	2003	1
FALSE	698	1	1996	2003	1
FALSE	698	1	1998	2003	1
FALSE	933	1	1998	2003	1
FALSE	327	1	1999	2000	1
FALSE	371	1	1999	2000	1
FALSE	37	1	1984	1985	1
FALSE	174	1	1984	1985	1
FALSE	698	1	1996	1997	1
FALSE	20	1	1986	1988	1
FALSE	70	1	1986	1988	1
FALSE	698	1	1985	2003	1
FALSE	94	1	1994	2003	1
FALSE	325	1	1994	1999	1
FALSE	796	1	1994	1999	1
FALSE	855	1	1994	1999	1
FALSE	94	1	1992	2002	1
FALSE	686	1	1996	2002	1
FALSE	686	1	1997	2003	1
FALSE	94	1	1995	1998	1

FALSE	102	1	1995	1998	1
FALSE	792	1	1991	1995	1
FALSE	865	1	1994	1998	1
FALSE	698	1	1990	1999	1
FALSE	318	1	1993	2002	1
FALSE	725	1	1993	2002	1
FALSE	698	1	1987	2003	1
FALSE	691	1	1998	2003	1
FALSE	698	1	1998	2003	1
FALSE	51	1	1999	2001	1
FALSE	148	1	1999	2001	1
FALSE	276	1	1995	2002	1
FALSE	686	1	1995	2002	1
FALSE	725	1	1997	2000	1
FALSE	698	1	1999	2002	1
FALSE	987	1	1999	2002	1
FALSE	698	1	1992	2000	1
FALSE	51	1	1999	2001	1
FALSE	276	1	1999	2001	1
FALSE	929	1	1999	2001	1
FALSE	1011	1	1991	2003	1
FALSE	923	1	1990	1997	1
FALSE	130	1	1989	1999	1
FALSE	376	1	1994	1999	1
FALSE	397	1	1994	2003	1
FALSE	376	1	1999	2001	1
FALSE	698	1	1995	1998	1
FALSE	465	1	1985	1987	1
FALSE	840	1	1985	1987	1
FALSE	698	1	1991	1998	1
FALSE	698	1	1988	2003	1
FALSE	698	1	1988	2002	1
FALSE	244	1	1992	1994	1
FALSE	94	1	1999	2002	1
FALSE	94	1	1990	2002	1
FALSE	276	1	1992	2003	1
FALSE	871	1	1992	2003	1
FALSE	383	1	1997	1999	1
FALSE	389	1	1997	1999	1
FALSE	935	1	1997	1999	1
FALSE	94	1	1995	2003	1
FALSE	831	1	1995	2003	1
FALSE	855	1	1995	2003	1
FALSE	148	1	1994	2003	1
FALSE	91	1	1999	2001	1
FALSE	148	1	1999	2001	1
FALSE	698	1	1990	2001	1

FALSE	706	1	1990	2001	1
FALSE	89	1	1998	2000	1
FALSE	587	1	1998	2000	1
FALSE	698	1	1998	2000	1
FALSE	120	1	1994	2003	1
FALSE	698	1	1994	2003	1
FALSE	823	1	1999	2000	1
FALSE	686	1	1994	2003	1
FALSE	888	1	1994	2003	1
FALSE	383	1	1995	1997	1
FALSE	763	1	1995	1997	1
FALSE	698	1	1992	2003	1
FALSE	698	1	1997	1999	1
FALSE	51	1	1996	1999	1
FALSE	823	1	1996	1999	1
FALSE	888	1	1996	1999	1
FALSE	276	1	1998	2003	1
FALSE	698	1	1998	2003	1
FALSE	89	1	1999	2003	1
FALSE	698	1	1999	2003	1
FALSE	718	1	1999	2003	1
FALSE	698	1	1993	2001	1
FALSE	698	1	1989	2002	1
FALSE	272	1	1998	1999	1
FALSE	686	1	1998	1999	1
FALSE	53	1	1996	2001	1
FALSE	690	1	1996	2001	1
FALSE	698	1	1995	1999	1
FALSE	51	1	1993	1997	1
FALSE	697	1	1993	1997	1
FALSE	88	1	1999	2002	1
FALSE	94	1	1999	2002	1
FALSE	94	1	1999	2003	1
FALSE	361	1	1999	2003	1
FALSE	688	1	1999	2003	1
FALSE	276	1	1993	2002	1
FALSE	488	1	1993	2002	1
FALSE	341	1	1992	1995	1
FALSE	698	1	1993	2000	1
FALSE	753	1	1993	2000	1
FALSE	94	1	1993	1994	1
FALSE	698	1	1984	1990	1
FALSE	698	1	1988	2003	1
FALSE	148	1	1993	2000	1
FALSE	376	1	1998	2002	1
FALSE	685	1	1998	2002	1
FALSE	698	1	1998	2002	1

FALSE	886	1	1997	2001	1
FALSE	698	1	1989	1999	1
FALSE	698	1	1997	2002	1
FALSE	698	1	1993	2001	1
FALSE	254	1	1998	2000	1
FALSE	887	1	1998	2000	1
FALSE	711	1	1993	2002	1
FALSE	94	1	1993	2002	1
FALSE	736	1	1988	1993	1
FALSE	468	1	1986	1987	1
FALSE	940	1	1994	1998	1
FALSE	751	1	1995	2001	1
FALSE	89	1	1996	2003	1
FALSE	698	1	1996	2003	1
FALSE	902	1	1996	2003	1
FALSE	147	1	1997	1998	1
FALSE	285	1	1997	1998	1
FALSE	1043	1	1997	1998	1
FALSE	698	1	1998	2001	1
FALSE	698	1	1999	2001	1
FALSE	51	1	1995	2001	1
FALSE	405	1	1995	2001	1
FALSE	410	1	1995	2001	1
FALSE	94	1	1995	1998	1
FALSE	94	1	1994	2000	1
FALSE	289	1	1991	2000	1
FALSE	376	1	1991	2000	1
FALSE	94	1	1988	2003	1
FALSE	376	1	1988	2003	1
FALSE	698	1	1996	2003	1
FALSE	1026	1	1996	2003	1
FALSE	1059	1	1996	2003	1
FALSE	685	1	1996	2003	1
FALSE	725	1	1996	2003	1
FALSE	21	1	1996	2002	1
FALSE	94	1	1996	2002	1
FALSE	396	1	1996	2002	1
FALSE	798	1	1996	2002	1
FALSE	775	1	1998	2001	1
FALSE	686	1	1984	2001	1
FALSE	698	1	1984	2001	1
FALSE	727	1	1984	2001	1
FALSE	68	1	1991	2001	1
FALSE	376	1	1991	2001	1
FALSE	698	1	1996	2002	1
FALSE	888	1	1996	2002	1
FALSE	553	1	1994	2002	1

FALSE	698	1	1994	2002	1
FALSE	94	1	1994	2000	1
FALSE	94	1	1997	2001	1
FALSE	548	1	1997	2001	1
FALSE	89	1	1992	2003	1
FALSE	130	1	1992	2003	1
FALSE	300	1	1992	2003	1
FALSE	94	1	1988	2003	1
FALSE	376	1	1988	2003	1
FALSE	148	1	1994	1999	1
FALSE	698	1	1985	2003	1
FALSE	1027	1	1998	1999	1
FALSE	94	1	1994	1997	1
FALSE	94	1	1999	2001	1
FALSE	698	1	1999	2001	1
FALSE	749	1	1999	2001	1
FALSE	674	1	1993	2002	1
FALSE	698	1	1993	2002	1
FALSE	698	1	1998	2000	1
FALSE	737	1	1998	2000	1
FALSE	753	1	1998	2000	1
FALSE	994	1	1998	2000	1
FALSE	698	1	1994	2001	1
FALSE	698	1	1994	1999	1
FALSE	906	1	1994	1999	1
FALSE	698	1	1991	1997	1
FALSE	466	1	1996	2001	1
FALSE	498	1	1996	2001	1
FALSE	515	1	1996	2001	1
FALSE	698	1	1995	1999	1
FALSE	94	1	1994	2003	1
FALSE	51	1	1993	2002	1

exap_b	ally	art_y21	entry_ii	exit_ii	y_in	art_y18	cit_y18
1	16	16	1988	2001	14	16	21
1	8	8	1987	1994	8	8	3
1	3	3	1994	1996	3	3	0
1	5	2	1993	1997	5	2	1
1	10	15	1993	2001	9	15	25
1	10	15	1993	2001	9	15	25
1	10	15	1993	2001	9	15	25
1	10	15	1993	2001	9	15	25
1	12	12	1992	2001	10	12	4
1	12	12	1992	2001	10	12	4
1	2	2	1997	1998	2	2	3
1	8	7	1993	2000	8	7	16
1	6	5	1998	2001	4	5	2
1	15	11	1987	2001	15	11	13
1	7	3	1997	2001	5	3	12
1	7	3	1997	2001	5	3	12
1	9	8	1995	2001	7	8	8
1	6	5	1996	2001	6	5	1
1	8	6	1991	1998	8	6	9
1	8	6	1991	1998	8	6	9
1	5	2	1995	1999	5	2	1
1	7	4	1994	2000	7	4	11
1	7	6	1996	2001	6	6	5
1	7	6	1996	2001	6	6	5
1	7	6	1996	2001	6	6	5
1	3	2	1997	1999	3	2	0
1	9	4	1995	2001	7	4	11
1	9	4	1995	2001	7	4	11
1	7	2	1991	1997	7	2	3
1	6	5	1998	2001	4	5	9
1	12	5	1992	2001	10	5	10
1	14	16	1990	2001	12	16	49
1	2	2	1999	2000	2	2	0
1	18	4	1986	2001	16	4	1
1	5	2	1998	2001	4	2	2
1	2	2	1991	1992	2	2	6
1	2	2	1991	1992	2	2	6
1	4	3	1996	1999	4	3	2
1	16	7	1988	2001	14	7	1
1	14	7	1989	2001	13	7	1
1	3	2	1998	2000	3	2	1
1	7	3	1993	1999	7	3	0
1	3	2	1999	2001	3	2	1
1	3	2	1999	2001	3	2	1
1	3	2	1999	2001	3	2	1
1	5	3	1990	1994	5	3	1

1	5	3	1990	1994	5	3	1
1	13	6	1988	2000	13	6	48
1	6	4	1997	2001	5	4	1
1	3	4	1998	2000	3	4	7
1	10	4	1990	1999	10	4	4
1	8	3	1988	1995	8	3	1
1	3	4	1998	2000	3	4	2
1	12	18	1992	2001	10	18	40
1	5	5	1999	2001	3	5	25
1	7	3	1995	2001	7	3	3
1	2	2	1998	1999	2	2	1
1	3	3	1998	2000	3	3	5
1	13	21	1989	2001	13	21	62
1	10	2	1994	2001	8	2	9
1	4	3	1998	2001	4	3	7
1	4	3	1998	2001	4	3	7
1	6	2	1998	2001	4	2	1
1	12	8	1991	2001	11	8	21
1	11	6	1993	2001	9	6	6
1	5	2	1994	1998	5	2	0
1	17	28	1987	2001	15	28	29
1	17	28	1987	2001	15	28	29
1	2	2	1992	1993	2	2	2
1	9	12	1995	2001	7	12	19
1	3	2	1996	1998	3	2	5
1	3	2	1996	1998	3	2	5
1	2	3	1994	1995	2	3	6
1	10	6	1990	1999	10	6	9
1	18	8	1986	2001	16	8	8
1	6	13	1998	2001	4	13	17
1	6	13	1998	2001	4	13	17
1	6	13	1998	2001	4	13	17
1	6	13	1998	2001	4	13	17
1	6	13	1998	2001	4	13	17
1	3	2	1990	1992	3	2	0
1	3	2	1990	1992	3	2	0
1	15	5	1986	2000	15	5	1
1	5	2	1989	1993	5	2	1
1	12	8	1992	2001	10	8	21
1	3	3	1999	2001	3	3	1
1	3	3	1999	2001	3	3	1
1	3	3	1999	2001	3	3	1
1	8	5	1989	1996	8	5	12
1	8	5	1989	1996	8	5	12
1	19	11	1985	2001	17	11	12
1	5	2	1997	2001	5	2	2
1	2	2	1994	1995	2	2	7

1	2	2	1994	1995	2	2	7
1	9	3	1995	2001	7	3	1
1	8	6	1996	2001	6	6	3
1	5	4	1998	2001	4	4	5
1	11	8	1993	2001	9	8	8
1	3	2	1998	2000	3	2	0
1	14	15	1990	2001	12	15	28
1	14	15	1990	2001	12	15	28
1	4	2	1998	2001	4	2	3
1	4	3	1997	2000	4	3	2
1	10	7	1993	2001	9	7	15
1	6	4	1996	2001	6	4	4
1	6	4	1996	2001	6	4	4
1	10	2	1993	2001	9	2	0
1	6	2	1996	2001	6	2	3
1	4	2	1998	2001	4	2	0
1	9	5	1995	2001	7	5	3
1	11	3	1989	1999	11	3	0
1	11	3	1989	1999	11	3	0
1	6	4	1996	2001	6	4	2
1	13	4	1991	2001	11	4	20
1	9	4	1994	2001	8	4	8
1	9	4	1994	2001	8	4	8
1	5	2	1988	1992	5	2	2
1	8	6	1993	2000	8	6	3
1	7	4	1997	2001	5	4	6
1	7	4	1997	2001	5	4	6
1	9	11	1995	2001	7	11	47
1	9	11	1995	2001	7	11	47
1	12	23	1992	2001	10	23	50
1	12	23	1992	2001	10	23	50
1	11	6	1992	2001	10	6	9
1	4	4	1998	2001	4	4	53
1	4	4	1998	2001	4	4	53
1	3	3	1999	2001	3	3	9
1	2	4	1992	1993	2	4	5
1	2	4	1992	1993	2	4	5
1	6	7	1998	2001	4	7	15
1	6	7	1998	2001	4	7	15
1	4	3	1998	2001	4	3	66
1	2	3	1997	1998	2	3	4
1	2	3	1997	1998	2	3	4
1	18	26	1986	2001	16	26	33
1	3	2	1999	2001	3	2	0
1	15	25	1989	2001	13	25	65
1	15	25	1989	2001	13	25	65
1	2	2	1995	1996	2	2	4

1	3	2	1995	1997	3	2	1
1	3	2	1995	1997	3	2	1
1	15	21	1989	2001	13	21	15
1	15	21	1989	2001	13	21	15
1	15	21	1989	2001	13	21	15
1	7	5	1991	1997	7	5	4
1	3	3	1999	2001	3	3	1
1	3	3	1999	2001	3	3	1
1	11	4	1993	2001	9	4	7
1	10	8	1994	2001	8	8	2
1	10	8	1994	2001	8	8	2
1	3	2	1999	2001	3	2	16
1	3	2	1999	2001	3	2	16
1	5	15	1999	2001	3	15	27
1	3	2	1999	2001	3	2	4
1	3	2	1999	2001	3	2	4
1	5	10	1998	2001	4	10	32
1	3	3	1997	1999	3	3	8
1	3	3	1997	1999	3	3	8
1	3	3	1997	1999	3	3	8
1	9	2	1992	2000	9	2	6
1	18	10	1985	2001	17	10	7
1	4	4	1998	2001	4	4	8
1	2	2	1985	1986	2	2	4
1	2	2	1985	1986	2	2	4
1	4	4	1998	2001	4	4	2
1	7	2	1991	1997	7	2	5
1	6	2	1990	1995	6	2	7
1	9	13	1995	2001	7	13	28
1	4	4	1991	1994	4	4	16
1	3	6	1997	1999	3	6	3
1	3	6	1997	1999	3	6	3
1	2	4	1999	2000	2	4	22
1	9	3	1995	2001	7	3	2
1	3	2	1995	1997	3	2	2
1	3	2	1995	1997	3	2	2
1	10	3	1994	2001	8	3	0
1	4	2	1998	2001	4	2	5
1	9	4	1994	2001	8	4	77
1	15	12	1988	2001	14	12	16
1	10	6	1994	2001	8	6	8
1	6	10	1998	2001	4	10	8
1	6	10	1998	2001	4	10	8
1	9	2	1992	2000	9	2	0
1	5	6	1997	2001	5	6	4
1	5	6	1997	2001	5	6	4
1	5	6	1997	2001	5	6	4

1	5	2	1999	2001	3	2	2
1	3	2	1991	1993	3	2	2
1	6	3	1998	2001	4	3	8
1	9	2	1995	2001	7	2	1
1	2	2	1992	1993	2	2	40
1	2	2	1992	1993	2	2	40
1	6	5	1997	2001	5	5	10
1	6	5	1997	2001	5	5	10
1	6	5	1997	2001	5	5	10
1	2	2	1999	2000	2	2	0
1	5	5	1999	2001	3	5	8
1	5	5	1999	2001	3	5	8
1	4	3	1999	2001	3	3	6
1	4	3	1999	2001	3	3	6
1	5	4	1996	2000	5	4	12
1	4	4	1999	2001	3	4	13
1	4	4	1999	2001	3	4	13
1	16	2	1987	2001	15	2	1
1	4	2	1998	2001	4	2	2
1	5	2	1999	2001	3	2	4
1	5	2	1999	2001	3	2	4
1	5	2	1992	1996	5	2	3
1	5	5	1997	2001	5	5	8
1	5	5	1997	2001	5	5	8
1	14	6	1990	2001	12	6	15
1	14	6	1990	2001	12	6	15
1	5	5	1996	2000	5	5	15
1	5	5	1996	2000	5	5	15
1	5	5	1996	2000	5	5	15
1	14	6	1990	2001	12	6	1
1	14	6	1990	2001	12	6	1
1	2	2	1999	2000	2	2	1
1	2	2	1999	2000	2	2	1
1	2	2	1999	2000	2	2	1
1	11	13	1993	2001	9	13	62
1	15	8	1989	2001	13	8	7
1	4	2	1999	2001	3	2	4
1	7	6	1997	2001	5	6	9
1	3	9	1996	1998	3	9	17
1	3	9	1996	1998	3	9	17
1	3	9	1996	1998	3	9	17
1	3	9	1996	1998	3	9	17
1	2	2	1994	1995	2	2	6
1	3	2	1992	1994	3	2	12
1	11	6	1993	2001	9	6	15
1	11	6	1993	2001	9	6	15
1	8	3	1990	1997	8	3	15

1	3	3	1998	2000	3	3	1
1	3	3	1998	2000	3	3	1
1	3	2	1996	1998	3	2	1
1	7	4	1997	2001	5	4	5
1	7	4	1997	2001	5	4	5
1	7	5	1997	2001	5	5	9
1	4	2	1999	2001	3	2	6
1	4	2	1999	2001	3	2	6
1	6	2	1998	2001	4	2	3
1	3	3	1994	1996	3	3	8
1	20	7	1984	2001	18	7	4
1	15	5	1986	2000	15	5	3
1	2	2	1989	1990	2	2	1
1	2	2	1989	1990	2	2	1
1	7	8	1997	2001	5	8	6
1	9	3	1995	2001	7	3	9
1	6	3	1998	2001	4	3	1
1	6	3	1998	2001	4	3	1
1	6	3	1998	2001	4	3	1
1	3	2	1995	1997	3	2	1
1	3	2	1995	1997	3	2	1
1	3	9	1998	2000	3	9	38
1	3	9	1998	2000	3	9	38
1	3	9	1998	2000	3	9	38
1	2	2	1997	1998	2	2	9
1	5	2	1991	1995	5	2	2
1	5	5	1996	2000	5	5	12
1	5	5	1996	2000	5	5	12
1	3	2	1995	1997	3	2	1
1	3	2	1995	1997	3	2	1
1	8	30	1995	2001	7	30	147
1	8	30	1995	2001	7	30	147
1	8	30	1995	2001	7	30	147
1	7	3	1995	2001	7	3	13
1	15	5	1986	2000	15	5	35
1	4	3	1997	2000	4	3	1
1	2	2	1995	1996	2	2	3
1	12	6	1992	2001	10	6	10
1	3	2	1999	2001	3	2	7
1	3	2	1999	2001	3	2	7
1	5	4	1999	2001	3	4	7
1	5	4	1999	2001	3	4	7
1	5	4	1999	2001	3	4	7
1	13	16	1991	2001	11	16	13
1	17	7	1985	2001	17	7	8
1	3	2	1999	2001	3	2	2
1	4	4	1999	2001	3	4	2

1	8	7	1993	2000	8	7	13
1	8	7	1993	2000	8	7	13
1	12	2	1989	2000	12	2	12
1	11	15	1992	2001	10	15	25
1	4	4	1998	2001	4	4	5
1	4	4	1998	2001	4	4	5
1	9	3	1990	1998	9	3	14
1	7	4	1992	1998	7	4	0
1	7	5	1997	2001	5	5	7
1	3	2	1999	2001	3	2	4
1	3	2	1999	2001	3	2	4
1	4	3	1994	1997	4	3	11
1	3	2	1999	2001	3	2	2
1	3	2	1999	2001	3	2	2
1	5	2	1997	2001	5	2	5
1	18	14	1986	2001	16	14	18
1	18	14	1986	2001	16	14	18
1	8	3	1994	2001	8	3	1
1	8	3	1994	2001	8	3	1
1	11	5	1991	2001	11	5	3
1	6	3	1984	1989	6	3	1
1	4	2	1993	1996	4	2	1
1	9	12	1995	2001	7	12	14
1	3	2	1999	2001	3	2	0
1	3	2	1999	2001	3	2	0
1	6	6	1998	2001	4	6	7
1	6	6	1998	2001	4	6	7
1	6	6	1998	2001	4	6	7
1	6	6	1998	2001	4	6	7
1	6	6	1998	2001	4	6	7
1	4	3	1996	1999	4	3	2
1	9	2	1989	1997	9	2	1
1	2	2	1995	1996	2	2	7
1	11	6	1992	2001	10	6	8
1	9	6	1984	1992	9	6	33
1	9	6	1984	1992	9	6	33
1	3	3	1997	1999	3	3	5
1	7	7	1997	2001	5	7	14
1	4	2	1999	2001	3	2	6
1	4	2	1999	2001	3	2	6
1	4	3	1998	2001	4	3	5
1	4	5	1998	2001	4	5	14
1	4	5	1998	2001	4	5	14
1	11	7	1990	2000	11	7	15
1	11	7	1990	2000	11	7	15
1	5	6	1995	1999	5	6	6
1	5	6	1995	1999	5	6	6

1	9	4	1992	2000	9	4	2
1	3	3	1989	1991	3	3	0
1	3	3	1989	1991	3	3	0
1	5	2	1997	2001	5	2	1
1	7	4	1990	1996	7	4	6
1	4	4	1997	2000	4	4	8
1	2	2	1998	1999	2	2	0
1	11	3	1993	2001	9	3	11
1	4	2	1998	2001	4	2	0
1	15	10	1989	2001	13	10	24
1	15	10	1989	2001	13	10	24
1	9	5	1991	1999	9	5	13
1	9	5	1991	1999	9	5	13
1	4	5	1999	2001	3	5	14
1	3	10	1998	2000	3	10	9
1	3	10	1998	2000	3	10	9
1	17	14	1987	2001	15	14	11
1	7	4	1995	2001	7	4	2
1	16	15	1988	2001	14	15	1
1	2	2	1999	2000	2	2	5
1	4	3	1999	2001	3	3	3
1	20	9	1984	2001	18	9	11
1	4	3	1997	2000	4	3	0
1	4	3	1997	2000	4	3	0
1	4	4	1999	2001	3	4	26
1	4	4	1999	2001	3	4	26
1	15	16	1989	2001	13	16	15
1	9	3	1994	2001	8	3	4
1	12	4	1990	2001	12	4	4
1	14	5	1989	2001	13	5	2
1	15	3	1989	2001	13	3	0
1	3	3	1999	2001	3	3	5
1	3	3	1999	2001	3	3	5
1	3	3	1999	2001	3	3	5
1	16	6	1987	2001	15	6	7
1	16	3	1987	2001	15	3	2
1	12	8	1992	2001	10	8	10
1	9	4	1995	2001	7	4	1
1	15	2	1989	2001	13	2	6
1	9	3	1986	1994	9	3	0
1	9	2	1995	2001	7	2	4
1	10	2	1989	1998	10	2	0
1	2	2	1994	1995	2	2	7
1	12	18	1992	2001	10	18	45
1	8	3	1996	2001	6	3	32
1	4	3	1999	2001	3	3	2
1	4	3	1999	2001	3	3	2

1	9	2	1990	1998	9	2	2
1	7	6	1987	1993	7	6	7
1	4	3	1998	2001	4	3	12
1	4	3	1998	2001	4	3	12
1	13	5	1986	1998	13	5	2
1	8	8	1995	2001	7	8	8
1	8	8	1995	2001	7	8	8
1	2	6	1996	1997	2	6	19
1	2	6	1996	1997	2	6	19
1	3	2	1998	2000	3	2	2
1	3	2	1998	2000	3	2	2
1	8	3	1993	2000	8	3	2
1	8	3	1993	2000	8	3	2
1	2	2	1998	1999	2	2	0
1	4	2	1998	2001	4	2	0
1	4	5	1995	1998	4	5	11
1	4	5	1995	1998	4	5	11
1	3	2	1999	2001	3	2	4
1	7	2	1995	2001	7	2	1
1	5	4	1997	2001	5	4	1
1	5	4	1997	2001	5	4	1
1	8	8	1996	2001	6	8	14
1	6	3	1988	1993	6	3	11
1	6	3	1988	1993	6	3	11
1	3	2	1995	1997	3	2	1
1	3	2	1995	1997	3	2	1
1	6	2	1998	2001	4	2	1
1	9	10	1995	2001	7	10	18
1	3	2	1984	1986	3	2	8
1	3	2	1984	1986	3	2	8
1	7	2	1996	2001	6	2	6
1	6	5	1996	2001	6	5	11
1	6	5	1996	2001	6	5	11
1	6	3	1998	2001	4	3	7
1	17	2	1986	2001	16	2	2
1	8	5	1995	2001	7	5	10
1	6	2	1998	2001	4	2	2
1	15	40	1989	2001	13	40	24
1	13	2	1984	1996	13	2	1
1	12	9	1992	2001	10	9	5
1	12	9	1992	2001	10	9	5
1	11	6	1993	2001	9	6	30
1	15	4	1988	2001	14	4	6
1	8	2	1995	2001	7	2	0
1	12	4	1992	2001	10	4	3
1	8	7	1989	1996	8	7	7
1	8	7	1989	1996	8	7	7

1	9	2	1989	1997	9	2	16
1	7	2	1997	2001	5	2	0
1	7	2	1997	2001	5	2	0
1	2	2	1999	2000	2	2	7
1	2	2	1999	2000	2	2	7
1	15	27	1989	2001	13	27	50
1	15	27	1989	2001	13	27	50
1	13	9	1989	2001	13	9	0
1	3	4	1996	1998	3	4	7
1	5	6	1997	2001	5	6	3
1	5	6	1997	2001	5	6	3
1	9	25	1995	2001	7	25	38
1	9	25	1995	2001	7	25	38
1	6	2	1988	1993	6	2	2
1	6	7	1997	2001	5	7	8
1	3	4	1989	1991	3	4	14
1	3	4	1989	1991	3	4	14
1	3	4	1989	1991	3	4	14
1	3	4	1989	1991	3	4	14
1	4	5	1995	1998	4	5	39
1	4	5	1995	1998	4	5	39
1	6	3	1998	2001	4	3	0
1	6	3	1998	2001	4	3	0
1	5	2	1998	2001	4	2	1
1	14	12	1988	2001	14	12	8
1	14	12	1988	2001	14	12	8
1	11	5	1993	2001	9	5	9
1	11	5	1993	2001	9	5	9
1	19	11	1984	2001	18	11	7
1	2	2	1994	1995	2	2	11
1	2	2	1994	1995	2	2	11
1	5	2	1994	1998	5	2	0
1	11	2	1993	2001	9	2	4
1	11	2	1993	2001	9	2	4
1	2	2	1994	1995	2	2	4
1	5	4	1998	2001	4	4	5
1	4	2	1999	2001	3	2	6
1	4	2	1999	2001	3	2	6
1	2	2	1998	1999	2	2	2
1	4	2	1999	2001	3	2	6
1	4	2	1999	2001	3	2	6
1	3	2	1993	1995	3	2	2
1	3	2	1993	1995	3	2	2
1	2	2	1994	1995	2	2	11
1	12	7	1992	2001	10	7	6
1	3	4	1997	1999	3	4	4
1	7	3	1996	2001	6	3	1

1	8	10	1996	2001	6	10	20
1	8	10	1996	2001	6	10	20
1	6	3	1993	1998	6	3	1
1	9	4	1995	2001	7	4	12
1	9	4	1995	2001	7	4	12
1	13	4	1986	1998	13	4	2
1	7	10	1997	2001	5	10	2
1	18	3	1985	2001	17	3	3
1	4	2	1998	2001	4	2	6
1	6	7	1998	2001	4	7	7
1	5	6	1996	2000	5	6	12
1	5	6	1996	2000	5	6	12
1	5	6	1996	2000	5	6	12
1	4	2	1999	2001	3	2	6
1	2	2	1996	1997	2	2	1
1	2	2	1996	1997	2	2	1
1	4	2	1999	2001	3	2	6
1	4	2	1999	2001	3	2	6
1	4	2	1994	1997	4	2	0
1	8	5	1996	2001	6	5	10
1	7	5	1997	2001	5	5	9
1	7	5	1997	2001	5	5	9
1	8	7	1996	2001	6	7	13
1	10	32	1993	2001	9	32	94
1	10	32	1993	2001	9	32	94
1	10	32	1993	2001	9	32	94
1	8	6	1992	1999	8	6	16
1	8	6	1992	1999	8	6	16
1	5	4	1996	2000	5	4	10
1	3	4	1997	1999	3	4	7
1	3	4	1997	1999	3	4	7
1	6	2	1994	1999	6	2	1
1	8	3	1990	1997	8	3	9
1	20	9	1984	2001	18	9	6
1	15	20	1989	2001	13	20	23
1	19	6	1985	2001	17	6	1
1	3	3	1993	1995	3	3	15
1	3	3	1993	1995	3	3	15
1	12	2	1991	2001	11	2	4
1	4	2	1991	1994	4	2	0
1	7	3	1995	2001	7	3	1
1	12	16	1992	2001	10	16	17
1	9	5	1994	2001	8	5	24
1	2	4	1998	1999	2	4	7
1	2	2	1999	2000	2	2	1
1	7	6	1996	2001	6	6	3
1	7	6	1996	2001	6	6	3

1	5	3	1997	2001	5	3	3
1	15	15	1989	2001	13	15	19
1	16	12	1988	2001	14	12	33
1	3	2	1998	2000	3	2	2
1	6	3	1993	1998	6	3	4
1	11	5	1993	2001	9	5	3
1	19	4	1985	2001	17	4	3
1	3	2	1998	2000	3	2	0
1	3	2	1998	2000	3	2	0
1	3	2	1999	2001	3	2	5
1	7	13	1997	2001	5	13	94
1	7	13	1997	2001	5	13	94
1	7	13	1997	2001	5	13	94
1	4	4	1998	2001	4	4	1
1	3	3	1991	1993	3	3	2
1	2	2	1998	1999	2	2	1
1	18	9	1984	2001	18	9	16
1	18	9	1984	2001	18	9	16
1	16	7	1987	2001	15	7	7
1	19	11	1984	2001	18	11	11
1	6	6	1997	2001	5	6	10
1	6	6	1997	2001	5	6	10
1	6	6	1997	2001	5	6	10
1	6	6	1997	2001	5	6	10
1	5	3	1998	2001	4	3	4
1	5	3	1998	2001	4	3	4
1	4	2	1998	2001	4	2	3
1	2	2	1996	1997	2	2	2
1	9	3	1995	2001	7	3	3
1	9	3	1995	2001	7	3	1
1	3	5	1999	2001	3	5	9
1	3	5	1999	2001	3	5	9
1	3	5	1999	2001	3	5	9
1	3	5	1999	2001	3	5	9
1	3	2	1997	1999	3	2	5
1	8	4	1993	2000	8	4	2
1	7	9	1994	2000	7	9	11
1	2	2	1999	2000	2	2	0
1	2	2	1999	2000	2	2	0
1	15	8	1984	1998	15	8	22
1	7	3	1997	2001	5	3	2
1	7	3	1997	2001	5	3	2
1	13	5	1989	2001	13	5	6
1	6	2	1988	1993	6	2	3
1	6	6	1997	2001	5	6	1
1	5	3	1998	2001	4	3	3
1	2	2	1997	1998	2	2	2

1	2	2	1996	1997	2	2	0
1	2	2	1996	1997	2	2	0
1	7	5	1997	2001	5	5	5
1	7	5	1997	2001	5	5	5
1	4	6	1999	2001	3	6	15
1	4	6	1999	2001	3	6	15
1	4	6	1999	2001	3	6	15
1	7	6	1997	2001	5	6	2
1	7	6	1997	2001	5	6	2
1	7	6	1997	2001	5	6	2
1	2	2	1997	1998	2	2	2
1	2	2	1997	1998	2	2	2
1	5	4	1999	2001	3	4	0
1	5	3	1999	2001	3	3	0
1	5	3	1999	2001	3	3	0
1	5	5	1998	2001	4	5	9
1	5	5	1998	2001	4	5	9
1	6	5	1998	2001	4	5	15
1	6	5	1998	2001	4	5	15
1	6	5	1998	2001	4	5	15
1	11	3	1984	1994	11	3	5
1	11	3	1984	1994	11	3	5
1	3	3	1989	1991	3	3	1
1	3	3	1992	1994	3	3	1
1	14	7	1990	2001	12	7	4
1	3	2	1999	2001	3	2	4
1	15	8	1988	2001	14	8	17
1	7	2	1992	1998	7	2	0
1	10	3	1994	2001	8	3	2
1	2	5	1998	1999	2	5	4
1	5	2	1997	2001	5	2	3
1	7	7	1995	2001	7	7	8
1	5	3	1997	2001	5	3	5
1	5	3	1997	2001	5	3	5
1	2	2	1998	1999	2	2	0
1	2	2	1998	1999	2	2	0
1	2	2	1995	1996	2	2	1
1	6	2	1998	2001	4	2	0
1	6	2	1998	2001	4	2	0
1	18	20	1986	2001	16	20	18
1	2	2	1998	1999	2	2	2
1	7	2	1988	1994	7	2	8
1	8	6	1996	2001	6	6	27
1	8	6	1996	2001	6	6	27
1	6	2	1984	1989	6	2	3
1	5	2	1997	2001	5	2	6
1	4	2	1997	2000	4	2	2

1	9	6	1995	2001	7	6	6
1	17	10	1986	2001	16	10	10
1	17	24	1987	2001	15	24	20
1	17	24	1987	2001	15	24	20
1	3	3	1997	1999	3	3	11
1	9	3	1989	1997	9	3	8
1	3	7	1996	1998	3	7	17
1	3	7	1996	1998	3	7	17
1	3	7	1996	1998	3	7	17
1	3	7	1996	1998	3	7	17
1	6	3	1998	2001	4	3	0
1	6	3	1998	2001	4	3	0
1	8	2	1996	2001	6	2	6
1	3	2	1998	2000	3	2	1
1	9	2	1992	2000	9	2	2
1	4	4	1993	1996	4	4	11
1	4	4	1993	1996	4	4	11
1	14	22	1990	2001	12	22	36
1	14	22	1990	2001	12	22	36
1	2	2	1994	1995	2	2	14
1	2	2	1994	1995	2	2	14
1	19	12	1984	2001	18	12	12
1	19	12	1984	2001	18	12	12
1	9	4	1991	1999	9	4	22
1	3	2	1990	1992	3	2	4
1	3	2	1990	1992	3	2	4
1	9	6	1994	2001	8	6	8
1	9	6	1994	2001	8	6	8
1	4	2	1993	1996	4	2	2
1	14	7	1989	2001	13	7	4
1	5	2	1997	2001	5	2	0
1	3	2	1998	2000	3	2	12
1	3	2	1998	2000	3	2	12
1	5	3	1985	1989	5	3	4
1	5	3	1985	1989	5	3	4
1	11	5	1993	2001	9	5	10
1	4	3	1998	2001	4	3	41
1	4	3	1998	2001	4	3	41
1	4	5	1997	2000	4	5	2
1	4	5	1997	2000	4	5	2
1	4	5	1997	2000	4	5	2
1	8	2	1994	2001	8	2	0
1	11	4	1993	2001	9	4	0
1	7	10	1995	2001	7	10	22
1	7	10	1995	2001	7	10	22
1	6	2	1996	2001	6	2	2
1	8	3	1995	2001	7	3	1

1	8	3	1995	2001	7	3	1
1	9	3	1995	2001	7	3	7
1	14	23	1990	2001	12	23	48
1	14	23	1990	2001	12	23	48
1	14	2	1989	2001	13	2	3
1	10	6	1994	2001	8	6	6
1	5	3	1998	2001	4	3	1
1	5	2	1999	2001	3	2	6
1	8	11	1996	2001	6	11	16
1	8	11	1996	2001	6	11	16
1	18	6	1985	2001	17	6	4
1	18	6	1985	2001	17	6	4
1	10	3	1993	2001	9	3	1
1	3	3	1994	1996	3	3	7
1	4	2	1985	1988	4	2	2
1	7	3	1991	1997	7	3	15
1	7	9	1994	2000	7	9	31
1	7	9	1994	2000	7	9	31
1	7	6	1992	1998	7	6	23
1	3	4	1993	1995	3	4	23
1	2	3	1995	1996	2	3	22
1	2	3	1995	1996	2	3	22
1	9	3	1994	2001	8	3	9
1	20	6	1984	2001	18	6	4
1	6	4	1998	2001	4	4	2
1	7	5	1997	2001	5	5	9
1	7	5	1997	2001	5	5	9
1	3	3	1999	2001	3	3	3
1	5	2	1999	2001	3	2	2
1	5	2	1999	2001	3	2	2
1	15	22	1989	2001	13	22	103
1	15	22	1989	2001	13	22	103
1	5	2	1987	1991	5	2	1
1	12	9	1992	2001	10	9	42
1	12	9	1992	2001	10	9	42
1	12	9	1992	2001	10	9	42
1	12	9	1992	2001	10	9	42
1	6	7	1997	2001	5	7	9
1	6	7	1997	2001	5	7	9
1	6	2	1986	1991	6	2	13
1	2	3	1997	1998	2	3	3
1	3	4	1999	2001	3	4	12
1	11	24	1993	2001	9	24	37
1	11	24	1993	2001	9	24	37
1	2	2	1991	1992	2	2	0
1	2	2	1991	1992	2	2	0
1	2	2	1996	1997	2	2	0

1	7	2	1997	2001	5	2	9
1	4	2	1996	1999	4	2	2
1	2	3	1993	1994	2	3	7
1	2	3	1993	1994	2	3	7
1	9	3	1995	2001	7	3	0
1	2	3	1996	1997	2	3	5
1	2	3	1996	1997	2	3	5
1	6	3	1998	2001	4	3	16
1	2	3	1987	1988	2	3	18
1	3	2	1992	1994	3	2	1
1	4	5	1999	2001	3	5	8
1	4	5	1999	2001	3	5	8
1	4	5	1999	2001	3	5	8
1	9	9	1992	2000	9	9	13
1	9	9	1992	2000	9	9	13
1	4	3	1998	2001	4	3	4
1	11	6	1991	2001	11	6	2
1	11	6	1991	2001	11	6	2
1	6	2	1998	2001	4	2	0
1	3	3	1997	1999	3	3	5
1	3	3	1997	1999	3	3	5
1	5	8	1999	2001	3	8	28
1	5	8	1999	2001	3	8	28
1	16	16	1987	2001	15	16	62
1	4	2	1998	2001	4	2	1
1	5	2	1995	1999	5	2	5
1	4	4	1997	2000	4	4	8
1	9	7	1995	2001	7	7	4
1	4	2	1999	2001	3	2	2
1	14	30	1990	2001	12	30	43
1	14	30	1990	2001	12	30	43
1	3	2	1994	1996	3	2	6
1	3	2	1994	1996	3	2	6
1	11	8	1991	2001	11	8	30
1	11	8	1991	2001	11	8	30
1	7	8	1997	2001	5	8	12
1	7	5	1994	2000	7	5	3
1	7	9	1997	2001	5	9	14
1	2	3	1999	2000	2	3	14
1	2	3	1999	2000	2	3	14
1	2	3	1992	1993	2	3	2
1	4	4	1998	2001	4	4	8
1	3	4	1997	1999	3	4	7
1	3	4	1997	1999	3	4	7
1	9	2	1993	2001	9	2	7
1	2	2	1993	1994	2	2	4
1	2	2	1999	2000	2	2	1

1	20	43	1984	2001	18	43	60
1	14	6	1989	2001	13	6	5
1	14	6	1989	2001	13	6	5
1	4	2	1994	1997	4	2	8
1	8	10	1996	2001	6	10	18
1	8	10	1996	2001	6	10	18
1	8	10	1996	2001	6	10	18
1	8	11	1996	2001	6	11	27
1	8	11	1996	2001	6	11	27
1	2	2	1984	1985	2	2	8
1	2	2	1984	1985	2	2	8
1	10	8	1991	2000	10	8	11
1	10	8	1991	2000	10	8	11
1	13	9	1991	2001	11	9	23
1	20	21	1984	2001	18	21	88
1	7	5	1997	2001	5	5	18
1	7	5	1997	2001	5	5	18
1	3	2	1999	2001	3	2	1
1	3	2	1999	2001	3	2	1
1	4	3	1998	2001	4	3	10
1	12	6	1992	2001	10	6	1
1	12	6	1992	2001	10	6	1
1	5	2	1992	1996	5	2	0
1	8	4	1996	2001	6	4	4
1	11	4	1993	2001	9	4	17
1	5	3	1994	1998	5	3	2
1	5	3	1994	1998	5	3	2
1	3	4	1997	1999	3	4	7
1	3	4	1997	1999	3	4	7
1	8	5	1993	2000	8	5	16
1	8	5	1993	2000	8	5	16
1	3	2	1991	1993	3	2	6
1	3	2	1991	1993	3	2	6
1	4	2	1998	2001	4	2	0
1	4	4	1994	1997	4	4	10
1	4	4	1994	1997	4	4	10
1	4	4	1994	1997	4	4	10
1	4	4	1996	1999	4	4	7
1	12	3	1992	2001	10	3	3
1	8	8	1994	2001	8	8	9
1	8	8	1994	2001	8	8	9
1	6	2	1992	1997	6	2	0
1	5	5	1991	1995	5	5	2
1	5	5	1991	1995	5	5	2
1	4	2	1997	2000	4	2	0
1	7	3	1996	2001	6	3	2
1	3	2	1989	1991	3	2	1

1	15	4	1989	2001	13	4	3
1	2	2	1994	1995	2	2	0
1	5	2	1999	2001	3	2	2
1	6	3	1998	2001	4	3	3
1	4	2	1996	1999	4	2	5
1	3	3	1999	2001	3	3	22
1	2	3	1998	1999	2	3	20
1	2	3	1998	1999	2	3	20
1	6	3	1991	1996	6	3	1
1	7	3	1996	2001	6	3	3
1	8	4	1996	2001	6	4	6
1	8	4	1996	2001	6	4	6
1	11	3	1993	2001	9	3	1
1	11	3	1993	2001	9	3	1
1	11	3	1993	2001	9	3	4
1	14	3	1986	1999	14	3	0
1	13	3	1991	2001	11	3	3
1	14	13	1986	1999	14	13	10
1	14	13	1986	1999	14	13	10
1	7	16	1997	2001	5	16	22
1	7	16	1997	2001	5	16	22
1	7	16	1997	2001	5	16	22
1	7	16	1997	2001	5	16	22
1	7	16	1997	2001	5	16	22
1	6	2	1998	2001	4	2	1
1	8	7	1994	2001	8	7	8
1	5	17	1998	2001	4	17	22
1	5	17	1998	2001	4	17	22
1	5	17	1998	2001	4	17	22
1	11	5	1992	2001	10	5	1
1	12	3	1988	1999	12	3	1
1	9	3	1985	1993	9	3	11
1	7	4	1995	2001	7	4	3
1	7	3	1989	1995	7	3	11
1	13	12	1991	2001	11	12	11
1	9	2	1993	2001	9	2	25
1	5	6	1999	2001	3	6	36
1	5	6	1999	2001	3	6	36
1	4	2	1996	1999	4	2	2
1	4	3	1996	1999	4	3	15
1	4	3	1996	1999	4	3	15
1	5	5	1998	2001	4	5	10
1	5	5	1998	2001	4	5	10
1	4	3	1997	2000	4	3	11
1	4	3	1997	2000	4	3	11
1	8	3	1996	2001	6	3	0
1	8	3	1996	2001	6	3	0

1	5	2	1997	2001	5	2	7
1	6	3	1997	2001	5	3	9
1	10	5	1994	2001	8	5	5
1	9	5	1995	2001	7	5	14
1	5	3	1999	2001	3	3	6
1	5	2	1999	2001	3	2	2
1	6	2	1995	2000	6	2	1
1	2	2	1987	1988	2	2	5
1	9	3	1992	2000	9	3	1
1	20	9	1984	2001	18	9	13
1	5	7	1998	2001	4	7	22
1	15	16	1988	2001	14	16	9
1	15	16	1988	2001	14	16	9
1	4	2	1999	2001	3	2	2
1	6	2	1996	2001	6	2	2
1	2	2	1993	1994	2	2	0
1	6	5	1996	2001	6	5	8
1	4	3	1995	1998	4	3	3
1	4	3	1995	1998	4	3	3
1	18	4	1985	2001	17	4	2
1	4	4	1999	2001	3	4	7
1	4	4	1999	2001	3	4	7
1	4	4	1999	2001	3	4	7
1	4	4	1999	2001	3	4	7
1	11	16	1993	2001	9	16	48
1	11	4	1993	2001	9	4	2
1	11	4	1993	2001	9	4	2
1	3	3	1997	1999	3	3	1
1	4	2	1999	2001	3	2	2
1	2	2	1995	1996	2	2	0
1	2	2	1995	1996	2	2	0
1	3	2	1995	1997	3	2	4
1	7	6	1997	2001	5	6	15
1	7	6	1997	2001	5	6	15
1	2	4	1998	1999	2	4	8
1	8	3	1992	1999	8	3	2
1	2	2	1997	1998	2	2	0
1	9	2	1992	2000	9	2	0
1	9	3	1995	2001	7	3	2
1	3	3	1999	2001	3	3	2
1	3	3	1999	2001	3	3	2
1	2	2	1999	2000	2	2	1
1	2	2	1999	2000	2	2	1
1	7	12	1997	2001	5	12	21
1	7	12	1997	2001	5	12	21
1	9	4	1993	2001	9	4	5
1	2	2	1995	1996	2	2	0

1	5	2	1993	1997	5	2	1
1	10	2	1993	2001	9	2	7
1	5	3	1998	2001	4	3	2
1	7	6	1993	1999	7	6	16
1	7	6	1993	1999	7	6	16
1	15	15	1989	2001	13	15	113
1	2	2	1999	2000	2	2	1
1	3	2	1986	1988	3	2	2
1	4	3	1999	2001	3	3	1
1	6	3	1995	2000	6	3	4
1	2	3	1984	1985	2	3	4
1	11	9	1992	2001	10	9	15
1	3	2	1993	1995	3	2	0
1	3	2	1993	1995	3	2	0
1	5	2	1996	2000	5	2	1
1	2	2	1984	1985	2	2	8
1	10	3	1993	2001	9	3	6
1	15	5	1988	2001	14	5	5
1	4	3	1996	1999	4	3	1
1	8	8	1993	2000	8	8	15
1	18	22	1986	2001	16	22	33
1	18	22	1986	2001	16	22	33
1	4	2	1999	2001	3	2	0
1	4	2	1999	2001	3	2	0
1	15	8	1989	2001	13	8	8
1	6	6	1998	2001	4	6	12
1	6	6	1998	2001	4	6	12
1	3	2	1994	1996	3	2	4
1	5	3	1997	2001	5	3	1
1	10	4	1993	2001	9	4	10
1	10	4	1993	2001	9	4	10
1	18	14	1986	2001	16	14	14
1	18	14	1986	2001	16	14	14
1	6	9	1996	2001	6	9	7
1	8	4	1996	2001	6	4	9
1	3	2	1999	2001	3	2	2
1	14	7	1989	2001	13	7	14
1	4	2	1992	1995	4	2	15
1	2	7	1996	1997	2	7	22
1	2	7	1996	1997	2	7	22
1	18	18	1985	2001	17	18	56
1	3	2	1998	2000	3	2	0
1	10	5	1994	2001	8	5	3
1	3	2	1999	2001	3	2	0
1	4	5	1997	2000	4	5	6
1	4	5	1997	2000	4	5	6
1	7	6	1992	1998	7	6	15

1	7	6	1992	1998	7	6	15
1	10	9	1994	2001	8	9	3
1	7	2	1995	2001	7	2	1
1	3	3	1999	2001	3	3	5
1	3	3	1999	2001	3	3	5
1	5	2	1997	2001	5	2	5
1	7	2	1996	2001	6	2	3
1	4	2	1998	2001	4	2	0
1	16	40	1988	2001	14	40	58
1	16	40	1988	2001	14	40	58
1	16	40	1988	2001	14	40	58
1	2	2	1999	2000	2	2	0
1	2	2	1999	2000	2	2	0
1	3	4	1998	2000	3	4	5
1	3	4	1998	2000	3	4	5
1	4	3	1998	2001	4	3	7
1	6	4	1998	2001	4	4	2
1	3	2	1992	1994	3	2	2
1	6	2	1997	2001	5	2	7
1	6	2	1997	2001	5	2	7
1	2	2	1989	1990	2	2	1
1	3	2	1993	1995	3	2	11
1	5	4	1994	1998	5	4	10
1	6	3	1998	2001	4	3	1
1	6	3	1998	2001	4	3	1
1	3	4	1999	2001	3	4	0
1	3	4	1999	2001	3	4	0
1	3	4	1999	2001	3	4	0
1	3	4	1999	2001	3	4	0
1	7	3	1997	2001	5	3	7
1	7	3	1997	2001	5	3	7
1	12	4	1992	2001	10	4	8
1	3	2	1999	2001	3	2	1
1	9	3	1995	2001	7	3	2
1	5	2	1993	1997	5	2	1
1	5	3	1999	2001	3	3	4
1	5	3	1999	2001	3	3	4
1	4	2	1992	1995	4	2	1
1	13	4	1991	2001	11	4	10
1	12	5	1992	2001	10	5	2
1	10	3	1991	2000	10	3	8
1	7	6	1997	2001	5	6	3
1	7	6	1997	2001	5	6	3
1	6	11	1997	2001	5	11	176
1	6	11	1997	2001	5	11	176
1	6	11	1997	2001	5	11	176
1	4	4	1999	2001	3	4	29

1	4	4	1999	2001	3	4	29
1	8	7	1991	1998	8	7	19
1	8	7	1991	1998	8	7	19
1	7	3	1987	1993	7	3	5
1	15	8	1989	2001	13	8	4
1	15	8	1989	2001	13	8	4
1	15	8	1989	2001	13	8	4
1	4	4	1996	1999	4	4	10
1	4	4	1996	1999	4	4	10
1	3	2	1999	2001	3	2	16
1	3	2	1997	1999	3	2	0
1	7	12	1997	2001	5	12	24
1	7	12	1997	2001	5	12	24
1	7	12	1997	2001	5	12	24
1	2	2	1998	1999	2	2	4
1	5	3	1998	2001	4	3	2
1	5	3	1998	2001	4	3	2
1	5	3	1998	2001	4	3	2
1	2	3	1998	1999	2	3	3
1	3	4	1999	2001	3	4	4
1	3	4	1999	2001	3	4	4
1	3	4	1999	2001	3	4	4
1	6	3	1998	2001	4	3	1
1	6	3	1998	2001	4	3	1
1	3	4	1998	2000	3	4	37
1	3	4	1998	2000	3	4	37
1	3	4	1998	2000	3	4	37
1	3	4	1998	2000	3	4	37
1	9	7	1995	2001	7	7	16
1	11	4	1993	2001	9	4	3
1	6	6	1996	2001	6	6	5
1	9	19	1995	2001	7	19	22
1	9	19	1995	2001	7	19	22
1	9	19	1995	2001	7	19	22
1	3	4	1998	2000	3	4	7
1	3	4	1998	2000	3	4	7
1	3	4	1998	2000	3	4	7
1	8	6	1994	2001	8	6	0
1	20	4	1984	2001	18	4	5
1	5	2	1997	2001	5	2	4
1	2	2	1997	1998	2	2	8
1	6	6	1998	2001	4	6	4
1	6	6	1998	2001	4	6	4
1	6	6	1998	2001	4	6	4
1	10	7	1992	2001	10	7	6
1	5	4	1999	2001	3	4	12
1	5	4	1999	2001	3	4	12

1	14	25	1990	2001	12	25	69
1	4	3	1994	1997	4	3	1
1	4	3	1994	1997	4	3	1
1	2	2	1998	1999	2	2	1
1	5	3	1999	2001	3	3	22
1	5	3	1999	2001	3	3	22
1	4	2	1992	1995	4	2	15
1	7	6	1994	2000	7	6	12
1	7	6	1994	2000	7	6	12
1	15	19	1989	2001	13	19	44
1	8	2	1995	2001	7	2	1
1	14	5	1989	2001	13	5	10
1	3	3	1999	2001	3	3	9
1	3	3	1999	2001	3	3	9
1	3	3	1999	2001	3	3	9
1	3	4	1992	1994	3	4	2
1	3	4	1992	1994	3	4	2
1	5	2	1993	1997	5	2	0
1	6	2	1998	2001	4	2	10
1	6	2	1998	2001	4	2	10
1	5	3	1995	1999	5	3	4
1	9	12	1994	2001	8	12	29
1	9	12	1994	2001	8	12	29
1	9	12	1994	2001	8	12	29
1	14	11	1990	2001	12	11	16
1	4	5	1993	1996	4	5	16
1	15	11	1988	2001	14	11	17
1	10	9	1994	2001	8	9	14
1	10	9	1994	2001	8	9	14
1	10	9	1994	2001	8	9	14
1	12	3	1991	2001	11	3	2
1	3	3	1998	2000	3	3	0
1	3	3	1998	2000	3	3	0
1	3	3	1998	2000	3	3	0
1	4	3	1999	2001	3	3	0
1	4	3	1999	2001	3	3	0
1	10	3	1994	2001	8	3	2
1	17	18	1987	2001	15	18	44
1	17	18	1987	2001	15	18	44
1	7	2	1996	2001	6	2	10
1	11	8	1993	2001	9	8	12
1	11	6	1992	2001	10	6	2
1	11	6	1992	2001	10	6	2
1	3	2	1990	1992	3	2	1
1	2	2	1991	1992	2	2	1
1	6	3	1998	2001	4	3	5
1	12	3	1990	2001	12	3	0

1	10	14	1993	2001	9	14	11
1	20	9	1984	2001	18	9	15
1	16	7	1988	2001	14	7	5
1	6	2	1994	1999	6	2	1
1	15	3	1989	2001	13	3	5
1	7	7	1996	2001	6	7	13
1	3	2	1997	1999	3	2	0
1	17	46	1987	2001	15	46	162
1	17	46	1987	2001	15	46	162
1	3	3	1993	1995	3	3	15
1	3	3	1993	1995	3	3	15
1	3	3	1993	1995	3	3	15
1	8	3	1996	2001	6	3	4
1	15	19	1989	2001	13	19	24
1	5	5	1994	1998	5	5	5
1	18	44	1986	2001	16	44	195
1	2	2	1993	1994	2	2	4
1	2	2	1993	1994	2	2	4
1	7	5	1987	1993	7	5	20
1	7	5	1987	1993	7	5	20
1	9	3	1989	1997	9	3	3
1	2	2	1998	1999	2	2	1
1	3	12	1998	2000	3	12	118
1	3	12	1998	2000	3	12	118
1	3	12	1998	2000	3	12	118
1	13	10	1991	2001	11	10	28
1	2	2	1999	2000	2	2	14
1	3	2	1995	1997	3	2	3
1	3	2	1998	2000	3	2	28
1	3	2	1998	2000	3	2	28
1	3	2	1994	1996	3	2	11
1	6	3	1994	1999	6	3	33
1	5	7	1996	2000	5	7	32
1	5	7	1996	2000	5	7	32
1	12	4	1989	2000	12	4	14
1	5	7	1994	1998	5	7	4
1	3	2	1997	1999	3	2	0
1	5	3	1997	2001	5	3	5
1	5	3	1997	2001	5	3	5
1	2	4	1992	1993	2	4	5
1	2	4	1992	1993	2	4	5
1	6	2	1995	2000	6	2	6
1	2	3	1999	2000	2	3	6
1	7	5	1995	2001	7	5	0
1	8	2	1989	1996	8	2	16
1	14	16	1987	2000	14	16	57
1	3	2	1997	1999	3	2	0

1	5	9	1998	2001	4	9	17
1	5	9	1998	2001	4	9	17
1	5	9	1998	2001	4	9	17
1	5	2	1997	2001	5	2	6
1	7	3	1990	1996	7	3	8
1	2	3	1993	1994	2	3	15
1	11	7	1993	2001	9	7	26
1	8	7	1995	2001	7	7	19
1	5	2	1995	1999	5	2	1
1	6	12	1998	2001	4	12	70
1	6	12	1998	2001	4	12	70
1	6	12	1998	2001	4	12	70
1	6	4	1998	2001	4	4	5
1	6	4	1998	2001	4	4	5
1	2	2	1996	1997	2	2	3
1	2	2	1996	1997	2	2	3
1	4	4	1996	1999	4	4	3
1	4	4	1996	1999	4	4	3
1	4	3	1999	2001	3	3	8
1	4	3	1999	2001	3	3	8
1	17	3	1985	2001	17	3	0
1	3	2	1992	1994	3	2	0
1	3	2	1992	1994	3	2	0
1	2	3	1997	1998	2	3	4
1	2	3	1997	1998	2	3	4
1	4	2	1999	2001	3	2	3
1	4	4	1996	1999	4	4	0
1	9	4	1995	2001	7	4	3
1	3	3	1991	1993	3	3	26
1	3	3	1991	1993	3	3	26
1	8	4	1989	1996	8	4	10
1	3	2	1997	1999	3	2	23
1	10	20	1994	2001	8	20	19
1	10	20	1994	2001	8	20	19
1	2	2	1996	1997	2	2	0
1	10	16	1994	2001	8	16	7
1	10	16	1994	2001	8	16	7
1	10	5	1991	2000	10	5	5
1	10	5	1991	2000	10	5	5
1	15	17	1988	2001	14	17	36
1	15	17	1988	2001	14	17	36
1	7	3	1995	2001	7	3	2
1	7	3	1995	2001	7	3	2
1	13	7	1991	2001	11	7	13
1	13	7	1991	2001	11	7	13
1	4	2	1999	2001	3	2	2
1	12	14	1992	2001	10	14	16

1	12	14	1992	2001	10	14	16
1	7	2	1991	1997	7	2	15
1	3	3	1985	1987	3	3	28
1	3	3	1985	1987	3	3	28
1	2	2	1998	1999	2	2	0
1	2	2	1998	1999	2	2	0
1	17	4	1985	2001	17	4	12
1	17	4	1985	2001	17	4	12
1	4	3	1998	2001	4	3	4
1	4	3	1998	2001	4	3	4
1	6	2	1997	2001	5	2	0
1	13	15	1991	2001	11	15	12
1	2	2	1997	1998	2	2	0
1	4	5	1998	2001	4	5	18
1	4	5	1998	2001	4	5	18
1	9	11	1995	2001	7	11	24
1	3	3	1998	2000	3	3	47
1	3	3	1998	2000	3	3	47
1	4	3	1997	2000	4	3	6
1	6	5	1994	1999	6	5	26
1	11	6	1993	2001	9	6	3
1	10	15	1993	2001	9	15	19
1	3	2	1998	2000	3	2	8
1	3	2	1998	2000	3	2	8
1	5	4	1996	2000	5	4	11
1	5	4	1996	2000	5	4	11
1	7	2	1997	2001	5	2	1
1	4	3	1995	1998	4	3	10
1	3	2	1996	1998	3	2	5
1	3	2	1996	1998	3	2	5
1	10	12	1993	2001	9	12	39
1	10	12	1993	2001	9	12	39
1	3	5	1991	1993	3	5	27
1	2	2	1999	2000	2	2	4
1	5	6	1999	2001	3	6	25
1	5	12	1999	2001	3	12	22
1	5	12	1999	2001	3	12	22
1	4	2	1997	2000	4	2	10
1	6	2	1995	2000	6	2	1
1	3	2	1995	1997	3	2	13
1	11	27	1993	2001	9	27	67
1	11	27	1993	2001	9	27	67
1	11	27	1993	2001	9	27	67
1	11	27	1993	2001	9	27	67
1	4	4	1998	2001	4	4	11
1	3	2	1999	2001	3	2	26
1	3	2	1999	2001	3	2	26

1	2	2	1999	2000	2	2	0
1	12	10	1990	2001	12	10	58
1	8	27	1995	2001	7	27	70
1	4	3	1994	1997	4	3	9
1	4	3	1994	1997	4	3	9
1	8	12	1996	2001	6	12	12
1	8	12	1996	2001	6	12	12
1	5	5	1997	2001	5	5	0
1	5	5	1997	2001	5	5	0
1	5	2	1993	1997	5	2	0
1	3	4	1997	1999	3	4	1
1	3	4	1997	1999	3	4	1
1	2	2	1999	2000	2	2	10
1	9	9	1994	2001	8	9	10
1	9	9	1994	2001	8	9	10
1	2	2	1996	1997	2	2	6
1	2	2	1996	1997	2	2	6
1	4	4	1993	1996	4	4	22
1	8	7	1995	2001	7	7	43
1	8	7	1995	2001	7	7	43
1	2	2	1998	1999	2	2	5
1	5	2	1995	1999	5	2	4
1	9	2	1995	2001	7	2	2
1	9	4	1989	1997	9	4	8
1	11	20	1993	2001	9	20	71
1	6	4	1997	2001	5	4	2
1	8	5	1996	2001	6	5	34
1	5	4	1999	2001	3	4	0
1	5	4	1999	2001	3	4	0
1	3	4	1997	1999	3	4	8
1	3	4	1997	1999	3	4	8
1	3	4	1997	1999	3	4	8
1	3	4	1997	1999	3	4	8
1	2	2	1996	1997	2	2	3
1	2	2	1996	1997	2	2	3
1	4	3	1985	1988	4	3	14
1	9	3	1995	2001	7	3	2
1	11	5	1993	2001	9	5	2
1	11	5	1993	2001	9	5	2
1	15	34	1986	2000	15	34	124
1	3	2	1997	1999	3	2	0
1	7	17	1997	2001	5	17	30
1	5	4	1996	2000	5	4	9
1	13	9	1989	2001	13	9	19
1	13	9	1989	2001	13	9	19
1	2	2	1996	1997	2	2	1
1	2	2	1996	1997	2	2	1

1	10	5	1993	2001	9	5	25
1	18	4	1986	2001	16	4	0
1	13	9	1990	2001	12	9	25
1	7	9	1997	2001	5	9	37
1	7	7	1996	2001	6	7	31
1	7	7	1996	2001	6	7	31
1	2	2	1996	1997	2	2	0
1	4	5	1998	2001	4	5	3
1	3	2	1998	2000	3	2	1
1	2	2	1995	1996	2	2	4
1	4	4	1999	2001	3	4	5
1	4	4	1999	2001	3	4	5
1	4	2	1999	2001	3	2	2
1	13	14	1991	2001	11	14	24
1	4	3	1997	2000	4	3	21
1	4	3	1997	2000	4	3	5
1	4	3	1997	2000	4	3	5
1	3	2	1995	1997	3	2	1
1	3	2	1995	1997	3	2	1
1	7	7	1996	2001	6	7	16
1	7	7	1996	2001	6	7	16
1	6	8	1996	2001	6	8	6
1	6	8	1996	2001	6	8	6
1	7	11	1995	2001	7	11	30
1	7	2	1995	2001	7	2	2
1	5	3	1996	2000	5	3	8
1	15	25	1989	2001	13	25	88
1	6	6	1998	2001	4	6	9
1	6	6	1998	2001	4	6	9
1	4	3	1997	2000	4	3	3
1	4	2	1999	2001	3	2	3
1	4	2	1999	2001	3	2	3
1	3	2	1995	1997	3	2	1
1	3	2	1995	1997	3	2	1
1	19	2	1984	2001	18	2	10
1	4	4	1998	2001	4	4	6
1	4	4	1998	2001	4	4	6
1	6	2	1996	2001	6	2	3
1	20	4	1984	2001	18	4	22
1	5	2	1995	1999	5	2	6
1	4	2	1999	2001	3	2	2
1	4	3	1994	1997	4	3	1
1	9	6	1995	2001	7	6	7
1	6	13	1993	1998	6	13	18
1	9	5	1993	2001	9	5	16
1	9	5	1993	2001	9	5	16
1	4	2	1992	1995	4	2	14

1	6	2	1997	2001	5	2	2
1	15	6	1989	2001	13	6	4
1	7	5	1997	2001	5	5	9
1	7	5	1997	2001	5	5	9
1	16	5	1987	2001	15	5	5
1	5	3	1999	2001	3	3	2
1	5	3	1999	2001	3	3	2
1	5	3	1999	2001	3	3	2
1	9	8	1993	2001	9	8	12
1	9	8	1993	2001	9	8	12
1	2	2	1999	2000	2	2	0
1	5	2	1994	1998	5	2	0
1	12	2	1989	2000	12	2	1
1	11	6	1992	2001	10	6	5
1	11	6	1992	2001	10	6	5
1	11	6	1992	2001	10	6	5
1	13	5	1990	2001	12	5	5
1	14	27	1989	2001	13	27	44
1	2	2	1993	1994	2	2	8
1	2	2	1993	1994	2	2	8
1	6	3	1998	2001	4	3	5
1	6	3	1998	2001	4	3	5
1	3	2	1999	2001	3	2	0
1	11	18	1993	2001	9	18	27
1	11	18	1993	2001	9	18	27
1	5	2	1999	2001	3	2	1
1	6	6	1998	2001	4	6	3
1	3	2	1997	1999	3	2	1
1	11	14	1993	2001	9	14	15
1	2	2	1999	2000	2	2	0
1	2	2	1999	2000	2	2	0
1	3	4	1988	1990	3	4	38
1	3	4	1988	1990	3	4	38
1	3	4	1988	1990	3	4	38
1	11	5	1993	2001	9	5	3
1	4	2	1993	1996	4	2	5
1	12	4	1992	2001	10	4	7
1	15	2	1989	2001	13	2	2
1	6	3	1990	1995	6	3	5
1	7	3	1995	2001	7	3	5
1	2	2	1999	2000	2	2	3
1	2	2	1999	2000	2	2	3
1	4	2	1996	1999	4	2	0
1	13	6	1989	2001	13	6	14
1	13	6	1989	2001	13	6	14
1	8	5	1996	2001	6	5	23
1	9	11	1994	2001	8	11	41

1	9	11	1994	2001	8	11	41
1	9	11	1994	2001	8	11	41
1	7	8	1992	1998	7	8	20
1	7	8	1992	1998	7	8	20
1	7	8	1992	1998	7	8	20
1	5	5	1997	2001	5	5	6
1	5	5	1997	2001	5	5	6
1	9	3	1995	2001	7	3	4
1	2	2	1997	1998	2	2	0
1	2	2	1997	1998	2	2	0
1	4	4	1995	1998	4	4	10
1	4	4	1995	1998	4	4	10
1	4	4	1995	1998	4	4	10
1	15	10	1989	2001	13	10	26
1	10	5	1992	2001	10	5	4
1	7	3	1997	2001	5	3	2
1	7	3	1997	2001	5	3	2
1	3	4	1997	1999	3	4	7
1	3	4	1997	1999	3	4	7
1	3	4	1997	1999	3	4	7
1	5	2	1998	2001	4	2	3
1	2	4	1999	2000	2	4	28
1	2	4	1999	2000	2	4	28
1	12	3	1990	2001	12	3	2
1	4	6	1998	2001	4	6	9
1	4	6	1998	2001	4	6	9
1	5	8	1991	1995	5	8	54
1	5	8	1991	1995	5	8	54
1	7	3	1995	2001	7	3	0
1	7	3	1995	2001	7	3	0
1	6	2	1996	2001	6	2	2
1	16	18	1988	2001	14	18	20
1	17	34	1987	2001	15	34	97
1	2	2	1999	2000	2	2	5
1	11	5	1992	2001	10	5	9
1	2	3	1993	1994	2	3	0
1	8	4	1996	2001	6	4	5
1	8	4	1996	2001	6	4	5
1	11	12	1993	2001	9	12	20
1	11	12	1993	2001	9	12	20
1	11	12	1993	2001	9	12	20
1	11	12	1993	2001	9	12	20
1	2	2	1997	1998	2	2	2
1	10	12	1990	1999	10	12	12
1	10	12	1990	1999	10	12	12
1	4	2	1995	1998	4	2	2
1	5	6	1996	2000	5	6	3

1	8	7	1996	2001	6	7	2
1	13	14	1991	2001	11	14	22
1	13	14	1991	2001	11	14	22
1	13	14	1991	2001	11	14	22
1	10	2	1986	1995	10	2	4
1	3	3	1999	2001	3	3	4
1	5	2	1997	2001	5	2	4
1	2	3	1998	1999	2	3	3
1	2	3	1998	1999	2	3	3
1	2	4	1993	1994	2	4	24
1	17	11	1987	2001	15	11	9
1	17	11	1987	2001	15	11	9
1	17	11	1987	2001	15	11	9
1	5	3	1999	2001	3	3	6
1	5	3	1999	2001	3	3	6
1	8	3	1995	2001	7	3	15
1	8	3	1995	2001	7	3	15
1	9	13	1995	2001	7	13	29
1	9	13	1995	2001	7	13	29
1	7	3	1996	2001	6	3	6
1	7	3	1996	2001	6	3	6
1	2	2	1999	2000	2	2	5
1	2	2	1999	2000	2	2	5
1	11	4	1993	2001	9	4	4
1	10	2	1992	2001	10	2	0
1	6	4	1998	2001	4	4	5
1	6	4	1998	2001	4	4	5
1	6	4	1998	2001	4	4	5
1	6	4	1998	2001	4	4	5
1	16	17	1987	2001	15	17	53
1	16	17	1987	2001	15	17	53
1	4	6	1998	2001	4	6	2
1	8	8	1995	2001	7	8	23
1	8	8	1995	2001	7	8	23
1	4	2	1996	1999	4	2	2
1	14	12	1990	2001	12	12	6
1	14	12	1990	2001	12	12	6
1	11	7	1991	2001	11	7	2
1	4	2	1998	2001	4	2	3
1	9	16	1995	2001	7	16	56
1	6	9	1996	2001	6	9	11
1	6	9	1996	2001	6	9	11
1	11	12	1992	2001	10	12	10
1	19	16	1985	2001	17	16	21
1	19	16	1985	2001	17	16	21
1	2	3	1996	1997	2	3	3
1	2	3	1996	1997	2	3	3

1	19	13	1985	2001	17	13	16
1	3	2	1994	1996	3	2	11
1	14	11	1990	2001	12	11	22
1	3	2	1998	2000	3	2	1
1	14	7	1990	2001	12	7	10
1	19	13	1985	2001	17	13	16
1	8	4	1994	2001	8	4	12
1	8	4	1994	2001	8	4	12
1	8	7	1994	2001	8	7	5
1	8	7	1994	2001	8	7	5
1	12	10	1990	2001	12	10	20
1	12	10	1990	2001	12	10	20
1	12	10	1990	2001	12	10	20
1	12	10	1990	2001	12	10	20
1	5	2	1997	2001	5	2	3
1	6	2	1998	2001	4	2	2
1	2	2	1998	1999	2	2	0
1	2	2	1998	1999	2	2	0
1	2	2	1999	2000	2	2	0
1	14	7	1990	2001	12	7	17
1	14	7	1990	2001	12	7	17
1	14	7	1990	2001	12	7	17
1	14	7	1990	2001	12	7	17
1	10	7	1994	2001	8	7	5
1	4	2	1995	1998	4	2	4
1	8	5	1996	2001	6	5	3
1	8	5	1996	2001	6	5	3
1	12	6	1991	2001	11	6	17
1	3	2	1995	1997	3	2	17
1	3	2	1995	1997	3	2	17
1	3	2	1995	1997	3	2	17
1	3	2	1995	1997	3	2	17
1	10	9	1993	2001	9	9	2
1	2	2	1991	1992	2	2	7
1	2	2	1991	1992	2	2	7
1	6	4	1995	2000	6	4	11
1	17	16	1987	2001	15	16	27
1	10	6	1994	2001	8	6	56
1	5	2	1995	1999	5	2	0
1	4	3	1994	1997	4	3	4
1	4	3	1994	1997	4	3	4
1	5	10	1999	2001	3	10	32
1	5	10	1999	2001	3	10	32
1	5	10	1999	2001	3	10	32
1	16	30	1988	2001	14	30	120
1	16	30	1988	2001	14	30	120
1	6	2	1998	2001	4	2	8

1	6	2	1998	2001	4	2	8
1	6	6	1997	2001	5	6	1
1	4	2	1988	1991	4	2	1
1	4	4	1999	2001	3	4	4
1	4	4	1999	2001	3	4	4
1	7	4	1990	1996	7	4	6
1	7	4	1990	1996	7	4	6
1	7	8	1994	2000	7	8	6
1	2	2	1998	1999	2	2	3
1	5	7	1998	2001	4	7	15
1	9	10	1995	2001	7	10	43
1	9	10	1995	2001	7	10	43
1	4	3	1997	2000	4	3	10
1	4	3	1997	2000	4	3	10
1	2	2	1997	1998	2	2	3
1	2	2	1997	1998	2	2	3
1	3	3	1990	1992	3	3	23
1	3	3	1990	1992	3	3	23
1	3	2	1997	1999	3	2	0
1	3	2	1997	1999	3	2	0
1	8	7	1996	2001	6	7	12
1	8	7	1996	2001	6	7	12
1	12	5	1986	1997	12	5	14
1	10	14	1994	2001	8	14	6
1	10	14	1994	2001	8	14	6
1	10	14	1994	2001	8	14	6
1	4	3	1996	1999	4	3	1
1	3	3	1995	1997	3	3	7
1	3	3	1995	1997	3	3	7
1	4	8	1993	1996	4	8	36
1	4	8	1993	1996	4	8	36
1	19	12	1985	2001	17	12	27
1	19	12	1985	2001	17	12	27
1	6	2	1994	1999	6	2	1
1	5	5	1988	1992	5	5	4
1	5	5	1988	1992	5	5	4
1	4	2	1999	2001	3	2	2
1	5	2	1999	2001	3	2	4
1	4	5	1998	2001	4	5	24
1	4	5	1998	2001	4	5	24
1	4	2	1999	2001	3	2	3
1	4	2	1998	2001	4	2	0
1	15	19	1989	2001	13	19	26
1	8	8	1996	2001	6	8	14
1	8	8	1996	2001	6	8	14
1	2	2	1993	1994	2	2	4
1	17	3	1987	2001	15	3	5

1	9	5	1995	2001	7	5	7
1	9	5	1995	2001	7	5	7
1	8	5	1992	1999	8	5	2
1	14	25	1990	2001	12	25	83
1	14	25	1990	2001	12	25	83
1	14	25	1990	2001	12	25	83
1	8	4	1996	2001	6	4	3
1	8	4	1996	2001	6	4	3
1	8	6	1992	1999	8	6	11
1	4	2	1999	2001	3	2	0
1	4	2	1999	2001	3	2	0
1	7	6	1995	2001	7	6	29
1	7	6	1995	2001	7	6	29
1	7	6	1995	2001	7	6	29
1	7	6	1997	2001	5	6	5
1	3	2	1999	2001	3	2	2
1	3	2	1999	2001	3	2	2
1	14	6	1990	2001	12	6	8
1	8	4	1987	1994	8	4	3
1	11	26	1993	2001	9	26	84
1	8	9	1994	2001	8	9	11
1	16	13	1987	2001	15	13	14
1	3	3	1992	1994	3	3	2
1	3	3	1992	1994	3	3	2
1	5	2	1999	2001	3	2	2
1	7	4	1996	2001	6	4	0
1	17	21	1987	2001	15	21	22
1	17	21	1987	2001	15	21	22
1	6	3	1994	1999	6	3	1
1	3	5	1999	2001	3	5	4
1	3	5	1999	2001	3	5	4
1	3	5	1999	2001	3	5	4
1	3	5	1999	2001	3	5	4
1	10	9	1994	2001	8	9	12
1	4	4	1999	2001	3	4	2
1	4	4	1999	2001	3	4	2
1	4	4	1999	2001	3	4	2
1	7	4	1997	2001	5	4	11
1	7	3	1997	2001	5	3	9
1	5	5	1999	2001	3	5	9
1	4	3	1998	2001	4	3	4
1	4	3	1998	2001	4	3	4
1	6	7	1998	2001	4	7	12
1	6	7	1998	2001	4	7	12
1	6	7	1998	2001	4	7	12
1	5	2	1994	1998	5	2	0
1	4	4	1993	1996	4	4	5

1	12	10	1989	2000	12	10	25
1	16	7	1988	2001	14	7	6
1	5	2	1995	1999	5	2	2
1	10	9	1994	2001	8	9	87
1	4	3	1989	1992	4	3	24
1	9	16	1995	2001	7	16	80
1	9	16	1995	2001	7	16	80
1	20	15	1984	2001	18	15	38
1	5	3	1997	2001	5	3	3
1	5	3	1997	2001	5	3	3
1	10	5	1993	2001	9	5	18
1	10	5	1993	2001	9	5	18
1	20	10	1984	2001	18	10	7
1	12	8	1992	2001	10	8	13
1	9	3	1992	2000	9	3	2
1	9	3	1992	2000	9	3	0
1	9	8	1994	2001	8	8	26
1	9	3	1994	2001	8	3	8
1	2	2	1999	2000	2	2	3
1	3	4	1998	2000	3	4	3
1	9	3	1995	2001	7	3	1
1	3	2	1995	1997	3	2	1
1	7	7	1996	2001	6	7	10
1	7	7	1996	2001	6	7	10
1	8	2	1990	1997	8	2	1
1	14	12	1990	2001	12	12	22
1	7	3	1995	2001	7	3	3
1	8	6	1996	2001	6	6	35
1	14	3	1985	1998	14	3	3
1	4	2	1998	2001	4	2	2
1	17	13	1987	2001	15	13	16
1	17	13	1987	2001	15	13	16
1	2	2	1998	1999	2	2	3
1	4	3	1996	1999	4	3	4
1	11	9	1993	2001	9	9	25
1	17	6	1986	2001	16	6	4
1	4	2	1998	2001	4	2	0
1	5	6	1999	2001	3	6	17
1	5	6	1999	2001	3	6	17
1	5	6	1999	2001	3	6	17
1	3	2	1995	1997	3	2	2
1	3	2	1995	1997	3	2	2
1	8	2	1995	2001	7	2	4
1	2	2	1999	2000	2	2	2
1	6	3	1998	2001	4	3	2
1	6	3	1998	2001	4	3	2
1	6	7	1994	1999	6	7	21

1	6	7	1994	1999	6	7	21
1	7	3	1997	2001	5	3	1
1	12	8	1990	2001	12	8	20
1	17	15	1986	2001	16	15	25
1	2	2	1998	1999	2	2	4
1	2	2	1998	1999	2	2	4
1	7	2	1993	1999	7	2	1
1	19	23	1985	2001	17	23	26
1	5	4	1999	2001	3	4	4
1	5	4	1999	2001	3	4	4
1	5	4	1999	2001	3	4	4
1	5	4	1999	2001	3	4	4
1	7	11	1994	2000	7	11	60
1	7	11	1994	2000	7	11	60
1	7	11	1994	2000	7	11	60
1	9	3	1995	2001	7	3	8
1	6	16	1998	2001	4	16	27
1	6	16	1998	2001	4	16	27
1	6	16	1998	2001	4	16	27
1	3	2	1995	1997	3	2	3
1	3	2	1995	1997	3	2	3
1	13	2	1984	1996	13	2	22
1	19	12	1985	2001	17	12	8
1	3	2	1997	1999	3	2	9
1	3	2	1997	1999	3	2	9
1	4	3	1998	2001	4	3	4
1	4	3	1998	2001	4	3	4
1	7	13	1995	2001	7	13	15
1	7	13	1995	2001	7	13	15
1	7	13	1995	2001	7	13	15
1	3	2	1999	2001	3	2	11
1	12	4	1990	2001	12	4	24
1	4	2	1999	2001	3	2	2
1	12	14	1992	2001	10	14	121
1	12	14	1992	2001	10	14	121
1	12	14	1992	2001	10	14	121
1	12	14	1992	2001	10	14	121
1	3	2	1992	1994	3	2	2
1	12	16	1992	2001	10	16	31
1	12	16	1992	2001	10	16	31
1	6	7	1997	2001	5	7	38
1	6	7	1997	2001	5	7	38
1	2	2	1998	1999	2	2	2
1	19	9	1985	2001	17	9	20
1	19	9	1985	2001	17	9	20
1	10	6	1992	2001	10	6	14
1	10	6	1992	2001	10	6	14

1	14	4	1988	2001	14	4	0
1	14	4	1988	2001	14	4	0
1	2	2	1997	1998	2	2	2
1	7	8	1996	2001	6	8	8
1	7	8	1996	2001	6	8	8
1	7	8	1996	2001	6	8	8
1	7	8	1996	2001	6	8	8
1	2	2	1997	1998	2	2	4
1	7	3	1996	2001	6	3	2
1	4	3	1999	2001	3	3	0
1	9	3	1994	2001	8	3	5
1	3	5	1997	1999	3	5	9
1	6	3	1996	2001	6	3	2
1	5	4	1995	1999	5	4	8
1	6	4	1994	1999	6	4	1
1	3	2	1996	1998	3	2	4
1	3	2	1996	1998	3	2	4
1	2	3	1992	1993	2	3	10
1	2	3	1992	1993	2	3	10
1	2	3	1992	1993	2	3	10
1	2	2	1990	1991	2	2	13
1	2	2	1990	1991	2	2	13
1	3	3	1998	2000	3	3	4
1	3	3	1998	2000	3	3	4
1	6	3	1988	1993	6	3	0
1	5	3	1999	2001	3	3	4
1	16	14	1988	2001	14	14	45
1	14	17	1988	2001	14	17	75
1	6	3	1987	1992	6	3	5
1	3	2	1998	2000	3	2	8
1	3	2	1998	2000	3	2	8
1	3	4	1997	1999	3	4	7
1	3	4	1997	1999	3	4	7
1	11	9	1993	2001	9	9	34
1	11	9	1993	2001	9	9	34
1	2	2	1996	1997	2	2	1
1	10	8	1994	2001	8	8	6
1	7	4	1995	2001	7	4	2
1	7	4	1995	2001	7	4	2
1	4	2	1999	2001	3	2	1
1	4	2	1995	1998	4	2	4
1	7	3	1996	2001	6	3	1
1	7	3	1996	2001	6	3	1
1	3	2	1999	2001	3	2	0
1	14	20	1990	2001	12	20	13
1	10	20	1994	2001	8	20	61
1	10	20	1994	2001	8	20	61

1	10	20	1994	2001	8	20	61
1	3	4	1997	1999	3	4	7
1	3	4	1997	1999	3	4	7
1	3	4	1997	1999	3	4	7
1	2	2	1990	1991	2	2	6
1	6	4	1995	2000	6	4	1
1	11	5	1992	2001	10	5	1
1	8	4	1996	2001	6	4	15
1	16	11	1987	2001	15	11	10
1	3	2	1997	1999	3	2	0
1	3	2	1997	1999	3	2	0
1	7	11	1997	2001	5	11	3
1	2	2	1995	1996	2	2	2
1	2	3	1999	2000	2	3	11
1	2	3	1999	2000	2	3	11
1	3	3	1999	2001	3	3	22
1	3	3	1999	2001	3	3	22
1	3	3	1998	2000	3	3	1
1	3	3	1998	2000	3	3	1
1	3	2	1997	1999	3	2	2
1	3	2	1997	1999	3	2	2
1	9	8	1995	2001	7	8	47
1	14	11	1990	2001	12	11	27
1	8	2	1995	2001	7	2	1
1	5	2	1997	2001	5	2	5
1	3	4	1993	1995	3	4	23
1	3	4	1993	1995	3	4	23
1	8	4	1987	1994	8	4	31
1	8	4	1987	1994	8	4	31
1	4	2	1996	1999	4	2	0
1	7	13	1994	2000	7	13	35
1	7	13	1994	2000	7	13	35
1	3	6	1999	2001	3	6	74
1	8	3	1991	1998	8	3	2
1	10	8	1990	1999	10	8	7
1	3	2	1998	2000	3	2	3
1	3	2	1998	2000	3	2	3
1	5	2	1986	1990	5	2	0
1	3	2	1997	1999	3	2	1
1	7	7	1997	2001	5	7	29
1	15	14	1989	2001	13	14	31
1	11	4	1993	2001	9	4	10
1	15	14	1989	2001	13	14	15
1	15	14	1989	2001	13	14	15
1	2	2	1999	2000	2	2	5
1	8	7	1996	2001	6	7	17
1	8	6	1995	2001	7	6	14

1	3	6	1999	2001	3	6	74
1	3	6	1999	2001	3	6	74
1	4	5	1997	2000	4	5	11
1	3	2	1998	2000	3	2	2
1	3	2	1998	2000	3	2	2
1	3	4	1998	2000	3	4	14
1	7	2	1995	2001	7	2	0
1	2	2	1998	1999	2	2	0
1	3	3	1999	2001	3	3	5
1	3	3	1999	2001	3	3	5
1	9	5	1995	2001	7	5	10
1	2	3	1999	2000	2	3	5
1	2	3	1999	2000	2	3	5
1	12	12	1992	2001	10	12	15
1	7	3	1997	2001	5	3	67
1	7	3	1996	2001	6	3	18
1	15	9	1989	2001	13	9	5
1	9	5	1994	2001	8	5	5
1	9	5	1994	2001	8	5	5
1	3	3	1998	2000	3	3	5
1	3	3	1998	2000	3	3	5
1	10	4	1992	2001	10	4	4
1	11	8	1993	2001	9	8	12
1	4	4	1998	2001	4	4	1
1	6	5	1998	2001	4	5	58
1	6	5	1998	2001	4	5	58
1	3	3	1996	1998	3	3	1
1	3	3	1996	1998	3	3	1
1	3	3	1996	1998	3	3	1
1	6	3	1996	2001	6	3	2
1	2	4	1998	1999	2	4	4
1	13	5	1991	2001	11	5	9
1	13	5	1991	2001	11	5	9
1	2	2	1998	1999	2	2	4
1	4	2	1999	2001	3	2	4
1	14	6	1990	2001	12	6	7
1	3	4	1998	2000	3	4	3
1	3	4	1998	2000	3	4	3
1	3	4	1998	2000	3	4	3
1	12	3	1985	1996	12	3	9
1	8	4	1996	2001	6	4	8
1	9	2	1995	2001	7	2	0
1	16	23	1988	2001	14	23	47
1	16	4	1987	2001	15	4	1
1	7	5	1992	1998	7	5	2
1	7	5	1992	1998	7	5	2
1	3	2	1997	1999	3	2	0

1	12	4	1990	2001	12	4	2
1	2	3	1992	1993	2	3	2
1	2	3	1992	1993	2	3	2
1	2	3	1992	1993	2	3	2
1	8	2	1996	2001	6	2	1
1	5	7	1997	2001	5	7	13
1	5	7	1997	2001	5	7	13
1	5	7	1997	2001	5	7	13
1	6	4	1996	2001	6	4	7
1	4	5	1994	1997	4	5	1
1	2	4	1998	1999	2	4	27
1	2	4	1998	1999	2	4	27
1	3	2	1993	1995	3	2	1
1	3	2	1993	1995	3	2	1
1	3	3	1990	1992	3	3	5
1	3	3	1990	1992	3	3	5
1	3	2	1995	1997	3	2	3
1	3	2	1995	1997	3	2	3
1	6	2	1998	2001	4	2	1
1	9	11	1995	2001	7	11	31
1	9	11	1995	2001	7	11	31
1	7	2	1993	1999	7	2	5
1	8	3	1995	2001	7	3	39
1	7	2	1990	1996	7	2	6
1	8	14	1996	2001	6	14	3
1	10	7	1992	2001	10	7	4
1	4	6	1996	1999	4	6	2
1	4	6	1996	1999	4	6	2
1	16	2	1987	2001	15	2	1
1	8	3	1996	2001	6	3	1
1	4	2	1998	2001	4	2	2
1	11	6	1993	2001	9	6	2
1	4	2	1999	2001	3	2	19
1	9	6	1995	2001	7	6	15
1	3	4	1998	2000	3	4	17
1	6	4	1996	2001	6	4	10
1	6	4	1996	2001	6	4	10
1	5	3	1999	2001	3	3	14
1	4	2	1989	1992	4	2	1
1	6	2	1998	2001	4	2	0
1	2	2	1997	1998	2	2	12
1	2	2	1997	1998	2	2	12
1	7	14	1997	2001	5	14	31
1	7	14	1997	2001	5	14	31
1	7	14	1997	2001	5	14	31
1	10	12	1994	2001	8	12	51
1	10	12	1994	2001	8	12	51

1	10	12	1994	2001	8	12	51
1	10	12	1994	2001	8	12	51
1	10	12	1994	2001	8	12	51
1	4	3	1998	2001	4	3	0
1	4	3	1998	2001	4	3	0
1	8	6	1996	2001	6	6	14
1	3	2	1997	1999	3	2	0
1	5	2	1993	1997	5	2	2
1	7	3	1993	1999	7	3	5
1	2	3	1996	1997	2	3	1
1	2	3	1996	1997	2	3	1
1	3	4	1999	2001	3	4	13
1	8	4	1994	2001	8	4	6
1	4	2	1998	2001	4	2	1
1	5	2	1997	2001	5	2	4
1	10	4	1992	2001	10	4	7
1	14	8	1989	2001	13	8	9
1	4	4	1999	2001	3	4	21
1	7	5	1995	2001	7	5	6
1	7	5	1995	2001	7	5	6
1	4	4	1999	2001	3	4	13
1	4	4	1999	2001	3	4	13
1	4	2	1995	1998	4	2	2
1	4	2	1996	1999	4	2	4
1	2	2	1999	2000	2	2	4
1	2	2	1999	2000	2	2	4
1	7	6	1995	2001	7	6	41
1	10	9	1994	2001	8	9	14
1	10	9	1994	2001	8	9	14
1	4	2	1998	2001	4	2	1
1	7	3	1995	2001	7	3	4
1	2	2	1998	1999	2	2	5
1	2	2	1998	1999	2	2	5
1	3	5	1998	2000	3	5	9
1	3	5	1998	2000	3	5	9
1	14	2	1986	1999	14	2	8
1	4	2	1991	1994	4	2	0
1	2	2	1985	1986	2	2	9
1	2	2	1985	1986	2	2	9
1	12	6	1990	2001	12	6	11
1	12	5	1992	2001	10	5	13
1	12	5	1992	2001	10	5	13
1	3	4	1999	2001	3	4	5
1	3	4	1999	2001	3	4	5
1	10	2	1991	2000	10	2	4
1	7	7	1996	2001	6	7	14
1	3	2	1998	2000	3	2	1

1	3	2	1998	2000	3	2	1
1	3	4	1985	1987	3	4	13
1	6	12	1998	2001	4	12	24
1	6	12	1998	2001	4	12	24
1	6	12	1998	2001	4	12	24
1	6	12	1998	2001	4	12	24
1	12	23	1992	2001	10	23	46
1	8	7	1996	2001	6	7	10
1	8	7	1996	2001	6	7	10
1	8	7	1996	2001	6	7	10
1	2	2	1996	1997	2	2	2
1	13	8	1989	2001	13	8	11
1	5	3	1996	2000	5	3	5
1	3	3	1998	2000	3	3	19
1	3	3	1998	2000	3	3	19
1	3	3	1998	2000	3	3	19
1	6	6	1998	2001	4	6	11
1	6	6	1998	2001	4	6	11
1	6	6	1998	2001	4	6	11
1	6	6	1998	2001	4	6	11
1	12	13	1992	2001	10	13	2
1	12	13	1992	2001	10	13	2
1	5	5	1998	2001	4	5	8
1	5	5	1998	2001	4	5	8
1	3	3	1999	2001	3	3	3
1	3	3	1999	2001	3	3	3
1	3	3	1999	2001	3	3	3
1	11	3	1992	2001	10	3	17
1	6	3	1991	1996	6	3	24
1	6	3	1991	1996	6	3	24
1	6	4	1996	2001	6	4	14
1	2	3	1994	1995	2	3	22
1	7	14	1996	2001	6	14	7
1	7	14	1996	2001	6	14	7
1	7	14	1996	2001	6	14	7
1	4	2	1999	2001	3	2	0
1	18	28	1986	2001	16	28	29
1	8	7	1994	2001	8	7	5
1	4	2	1995	1998	4	2	3
1	5	2	1999	2001	3	2	0
1	8	4	1996	2001	6	4	3
1	8	4	1996	2001	6	4	3
1	4	3	1996	1999	4	3	10
1	4	3	1996	1999	4	3	10
1	2	2	1999	2000	2	2	0
1	5	3	1997	2001	5	3	5
1	5	3	1997	2001	5	3	5

1	9	10	1995	2001	7	10	13
1	9	10	1995	2001	7	10	13
1	15	8	1986	2000	15	8	46
1	15	8	1986	2000	15	8	46
1	15	8	1986	2000	15	8	46
1	9	26	1995	2001	7	26	76
1	9	26	1995	2001	7	26	76
1	9	26	1995	2001	7	26	76
1	4	3	1997	2000	4	3	0
1	3	3	1995	1997	3	3	3
1	8	4	1994	2001	8	4	7
1	2	2	1996	1997	2	2	2
1	2	2	1996	1997	2	2	2
1	3	2	1998	2000	3	2	0
1	4	2	1999	2001	3	2	8
1	4	2	1999	2001	3	2	8
1	8	4	1994	2001	8	4	2
1	6	3	1991	1996	6	3	7
1	15	12	1988	2001	14	12	17
1	8	3	1996	2001	6	3	1
1	8	2	1990	1997	8	2	1
1	3	2	1997	1999	3	2	1
1	3	2	1994	1996	3	2	0
1	6	3	1998	2001	4	3	1
1	3	2	1997	1999	3	2	2
1	7	25	1997	2001	5	25	33
1	7	25	1997	2001	5	25	33
1	7	25	1997	2001	5	25	33
1	7	25	1997	2001	5	25	33
1	7	25	1997	2001	5	25	33
1	9	2	1991	1999	9	2	1
1	8	5	1996	2001	6	5	44
1	8	5	1996	2001	6	5	44
1	4	2	1992	1995	4	2	0
1	2	2	1996	1997	2	2	1
1	2	2	1996	1997	2	2	1
1	2	3	1998	1999	2	3	4
1	5	6	1999	2001	3	6	2
1	5	6	1999	2001	3	6	2
1	9	9	1995	2001	7	9	1
1	15	18	1988	2001	14	18	40
1	3	4	1999	2001	3	4	2
1	3	4	1999	2001	3	4	2
1	5	7	1998	2001	4	7	16
1	5	7	1998	2001	4	7	16
1	4	2	1996	1999	4	2	2
1	13	5	1991	2001	11	5	8

1	13	5	1991	2001	11	5	8
1	7	3	1993	1999	7	3	15
1	8	3	1995	2001	7	3	2
1	2	2	1995	1996	2	2	0
1	2	2	1995	1996	2	2	0
1	2	4	1998	1999	2	4	4
1	2	4	1998	1999	2	4	4
1	9	14	1995	2001	7	14	36
1	9	14	1995	2001	7	14	36
1	3	5	1998	2000	3	5	3
1	3	5	1998	2000	3	5	3
1	13	2	1991	2001	11	2	10
1	3	2	1999	2001	3	2	4
1	3	2	1999	2001	3	2	4
1	9	5	1994	2001	8	5	5
1	18	9	1986	2001	16	9	8
1	2	3	1995	1996	2	3	14
1	5	2	1999	2001	3	2	1
1	5	2	1999	2001	3	2	1
1	2	4	1999	2000	2	4	6
1	2	4	1999	2000	2	4	6
1	3	2	1993	1995	3	2	0
1	4	2	1997	2000	4	2	12
1	11	9	1993	2001	9	9	30
1	6	2	1998	2001	4	2	1
1	8	3	1996	2001	6	3	3
1	4	2	1994	1997	4	2	1
1	5	3	1997	2001	5	3	8
1	13	39	1991	2001	11	39	45
1	13	39	1991	2001	11	39	45
1	5	3	1994	1998	5	3	33
1	5	3	1994	1998	5	3	33
1	3	5	1997	1999	3	5	4
1	2	2	1997	1998	2	2	2
1	2	2	1997	1998	2	2	2
1	4	3	1994	1997	4	3	19
1	4	3	1994	1997	4	3	19
1	7	2	1990	1996	7	2	2
1	14	5	1989	2001	13	5	3
1	8	7	1996	2001	6	7	7
1	17	4	1985	2001	17	4	5
1	5	4	1998	2001	4	4	3
1	11	3	1993	2001	9	3	0
1	8	5	1996	2001	6	5	10
1	2	4	1996	1997	2	4	13
1	2	4	1996	1997	2	4	13
1	5	3	1998	2001	4	3	4

1	8	5	1996	2001	6	5	4
1	7	3	1997	2001	5	3	11
1	7	3	1997	2001	5	3	11
1	2	2	1995	1996	2	2	0
1	2	2	1995	1996	2	2	0
1	4	2	1999	2001	3	2	0
1	2	2	1999	2000	2	2	5
1	2	2	1999	2000	2	2	5
1	12	4	1992	2001	10	4	10
1	16	8	1986	2001	16	8	9
1	14	7	1989	2001	13	7	66
1	15	14	1988	2001	14	14	18
1	5	5	1998	2001	4	5	3
1	5	5	1998	2001	4	5	3
1	5	5	1998	2001	4	5	3
1	3	3	1998	2000	3	3	1
1	3	3	1998	2000	3	3	1
1	5	2	1999	2001	3	2	13
1	5	2	1999	2001	3	2	13
1	9	3	1990	1998	9	3	4
1	3	2	1996	1998	3	2	1
1	8	4	1996	2001	6	4	8
1	8	4	1996	2001	6	4	8
1	8	4	1996	2001	6	4	8
1	9	16	1995	2001	7	16	23
1	10	4	1994	2001	8	4	2
1	10	4	1994	2001	8	4	2
1	8	6	1996	2001	6	6	12
1	8	6	1996	2001	6	6	12
1	8	6	1996	2001	6	6	12
1	8	6	1996	2001	6	6	12
1	3	2	1999	2001	3	2	6
1	4	2	1999	2001	3	2	0
1	4	2	1999	2001	3	2	0
1	16	17	1988	2001	14	17	20
1	16	17	1988	2001	14	17	20
1	7	6	1994	2000	7	6	3
1	16	6	1988	2001	14	6	11
1	16	6	1988	2001	14	6	11
1	6	3	1995	2000	6	3	6
1	2	2	1990	1991	2	2	11
1	2	2	1990	1991	2	2	11
1	2	2	1998	1999	2	2	14
1	20	11	1984	2001	18	11	21
1	7	5	1997	2001	5	5	17
1	7	5	1997	2001	5	5	17
1	7	12	1997	2001	5	12	16

1	5	8	1998	2001	4	8	18
1	5	8	1998	2001	4	8	18
1	5	8	1998	2001	4	8	18
1	8	7	1988	1995	8	7	1
1	8	7	1988	1995	8	7	1
1	8	8	1996	2001	6	8	11
1	2	3	1999	2000	2	3	1
1	2	3	1999	2000	2	3	1
1	2	3	1999	2000	2	3	1
1	2	2	1998	1999	2	2	6
1	17	10	1985	2001	17	10	41
1	6	3	1998	2001	4	3	1
1	6	5	1998	2001	4	5	4
1	6	5	1998	2001	4	5	4
1	9	4	1995	2001	7	4	7
1	17	9	1987	2001	15	9	5
1	9	9	1995	2001	7	9	29
1	5	5	1998	2001	4	5	2
1	2	2	1992	1993	2	2	3
1	5	3	1999	2001	3	3	2
1	5	3	1999	2001	3	3	2
1	10	10	1993	2001	9	10	3
1	10	10	1993	2001	9	10	3
1	5	3	1999	2001	3	3	0
1	5	3	1999	2001	3	3	0
1	13	5	1989	2001	13	5	9
1	3	2	1993	1995	3	2	7
1	7	4	1996	2001	6	4	3
1	7	4	1996	2001	6	4	3
1	2	5	1999	2000	2	5	34
1	2	5	1999	2000	2	5	34
1	8	4	1992	1999	8	4	0
1	8	4	1992	1999	8	4	0
1	2	4	1999	2000	2	4	4
1	2	4	1999	2000	2	4	4
1	5	4	1999	2001	3	4	14
1	5	4	1999	2001	3	4	14
1	5	4	1999	2001	3	4	14
1	16	8	1985	2000	16	8	6
1	7	3	1996	2001	6	3	1
1	4	2	1999	2001	3	2	0
1	4	2	1999	2001	3	2	0
1	17	23	1987	2001	15	23	23
1	3	3	1998	2000	3	3	4
1	3	3	1998	2000	3	3	4
1	3	3	1998	2000	3	3	4
1	4	5	1998	2001	4	5	11

1	6	5	1998	2001	4	5	12
1	8	2	1996	2001	6	2	0
1	14	8	1988	2001	14	8	8
1	13	14	1990	2001	12	14	7
1	13	14	1990	2001	12	14	7
1	4	2	1994	1997	4	2	7
1	15	18	1989	2001	13	18	14
1	14	9	1990	2001	12	9	1
1	5	2	1999	2001	3	2	2
1	5	2	1999	2001	3	2	2
1	14	8	1990	2001	12	8	13
1	9	7	1994	2001	8	7	11
1	9	7	1994	2001	8	7	11
1	9	7	1994	2001	8	7	11
1	6	4	1998	2001	4	4	9
1	3	2	1998	2000	3	2	3
1	3	2	1998	2000	3	2	3
1	9	4	1991	1999	9	4	4
1	8	3	1993	2000	8	3	3
1	8	2	1996	2001	6	2	4
1	3	2	1999	2001	3	2	2
1	3	2	1999	2001	3	2	2
1	4	2	1999	2001	3	2	6
1	6	3	1998	2001	4	3	0
1	13	7	1989	2001	13	7	13
1	5	3	1998	2001	4	3	3
1	12	9	1992	2001	10	9	19
1	5	6	1995	1999	5	6	8
1	18	12	1986	2001	16	12	24
1	11	7	1993	2001	9	7	14
1	4	2	1998	2001	4	2	9
1	5	2	1988	1992	5	2	4
1	19	13	1985	2001	17	13	18
1	8	10	1996	2001	6	10	20
1	8	10	1996	2001	6	10	20
1	7	6	1989	1995	7	6	6
1	7	6	1989	1995	7	6	6
1	9	5	1995	2001	7	5	6
1	9	5	1995	2001	7	5	6
1	3	2	1998	2000	3	2	1
1	3	2	1998	2000	3	2	1
1	4	6	1998	2001	4	6	17
1	4	6	1998	2001	4	6	17
1	7	5	1997	2001	5	5	3
1	7	5	1997	2001	5	5	3
1	9	7	1995	2001	7	7	11
1	2	2	1998	1999	2	2	0

1	8	4	1996	2001	6	4	10
1	5	3	1998	2001	4	3	5
1	3	2	1994	1996	3	2	0
1	11	5	1993	2001	9	5	0
1	11	48	1993	2001	9	48	59
1	11	6	1991	2001	11	6	28
1	8	2	1991	1998	8	2	3
1	4	2	1996	1999	4	2	6
1	4	2	1996	1999	4	2	6
1	8	4	1994	2001	8	4	16
1	8	4	1994	2001	8	4	16
1	5	3	1999	2001	3	3	15
1	5	3	1999	2001	3	3	15
1	3	3	1999	2001	3	3	9
1	3	3	1999	2001	3	3	9
1	3	3	1999	2001	3	3	9
1	5	3	1998	2001	4	3	3
1	5	3	1998	2001	4	3	3
1	18	9	1986	2001	16	9	28
1	6	4	1998	2001	4	4	3
1	6	4	1998	2001	4	4	3
1	6	6	1998	2001	4	6	2
1	6	6	1998	2001	4	6	2
1	7	2	1992	1998	7	2	0
1	16	6	1986	2001	16	6	9
1	7	4	1997	2001	5	4	12
1	7	4	1997	2001	5	4	12
1	10	9	1994	2001	8	9	15
1	10	9	1994	2001	8	9	15
1	5	6	1996	2000	5	6	14
1	5	6	1996	2000	5	6	14
1	18	3	1986	2001	16	3	13
1	7	3	1996	2001	6	3	9
1	7	3	1996	2001	6	3	9
1	6	3	1988	1993	6	3	7
1	5	6	1998	2001	4	6	9
1	12	3	1992	2001	10	3	8
1	4	3	1998	2001	4	3	16
1	4	3	1998	2001	4	3	16
1	3	3	1986	1988	3	3	9
1	3	3	1986	1988	3	3	9
1	9	11	1995	2001	7	11	16
1	15	3	1988	2001	14	3	2
1	4	2	1997	2000	4	2	1
1	5	2	1999	2001	3	2	0
1	5	2	1999	2001	3	2	0
1	12	5	1992	2001	10	5	15

1	12	5	1992	2001	10	5	15
1	12	5	1992	2001	10	5	15
1	5	5	1999	2001	3	5	6
1	2	2	1992	1993	2	2	3
1	2	2	1992	1993	2	2	3
1	4	4	1991	1994	4	4	9
1	14	14	1990	2001	12	14	27
1	14	14	1990	2001	12	14	27
1	7	5	1995	2001	7	5	6
1	7	5	1995	2001	7	5	6
1	2	4	1998	1999	2	4	10
1	2	4	1998	1999	2	4	10
1	8	7	1996	2001	6	7	25
1	2	2	1995	1996	2	2	4
1	16	3	1988	2001	14	3	1
1	6	7	1997	2001	5	7	12
1	6	7	1997	2001	5	7	12
1	12	12	1992	2001	10	12	11
1	12	12	1992	2001	10	12	11
1	2	4	1999	2000	2	4	14
1	2	4	1999	2000	2	4	14
1	2	4	1999	2000	2	4	14
1	14	5	1988	2001	14	5	8
1	7	3	1993	1999	7	3	0
1	12	3	1992	2001	10	3	2
1	6	5	1997	2001	5	5	14
1	6	5	1997	2001	5	5	14
1	2	2	1998	1999	2	2	8
1	6	2	1996	2001	6	2	2
1	16	10	1985	2000	16	10	18
1	16	10	1985	2000	16	10	18
1	7	6	1995	2001	7	6	9
1	7	6	1995	2001	7	6	9
1	8	3	1996	2001	6	3	3
1	12	3	1992	2001	10	3	4
1	12	3	1992	2001	10	3	4
1	6	8	1998	2001	4	8	12
1	6	8	1998	2001	4	8	12
1	6	8	1998	2001	4	8	12
1	8	4	1995	2001	7	4	18
1	7	6	1996	2001	6	6	6
1	7	6	1996	2001	6	6	6
1	7	6	1996	2001	6	6	6
1	16	18	1987	2001	15	18	24
1	11	14	1987	1997	11	14	22
1	11	14	1987	1997	11	14	22
1	11	14	1987	1997	11	14	22

1	3	2	1999	2001	3	2	1
1	3	2	1999	2001	3	2	1
1	5	4	1989	1993	5	4	2
1	2	2	1996	1997	2	2	0
1	2	3	1999	2000	2	3	0
1	2	3	1999	2000	2	3	0
1	8	9	1996	2001	6	9	8
1	10	11	1992	2001	10	11	24
1	5	2	1998	2001	4	2	6
1	5	3	1996	2000	5	3	7
1	5	3	1996	2000	5	3	7
1	4	3	1997	2000	4	3	21
1	4	3	1997	2000	4	3	21
1	5	9	1999	2001	3	9	15
1	5	9	1999	2001	3	9	15
1	5	9	1999	2001	3	9	15
1	3	3	1998	2000	3	3	19
1	3	3	1998	2000	3	3	19
1	7	4	1996	2001	6	4	12
1	7	4	1996	2001	6	4	12
1	5	6	1997	2001	5	6	8
1	3	2	1996	1998	3	2	3
1	3	2	1996	1998	3	2	3
1	17	4	1986	2001	16	4	19
1	2	2	1999	2000	2	2	1
1	4	2	1998	2001	4	2	9
1	9	8	1994	2001	8	8	11
1	9	8	1994	2001	8	8	11
1	7	5	1994	2000	7	5	15
1	19	6	1985	2001	17	6	21
1	3	3	1999	2001	3	3	0
1	3	3	1999	2001	3	3	0
1	3	3	1999	2001	3	3	0
1	4	3	1998	2001	4	3	3
1	14	6	1988	2001	14	6	42
1	4	2	1997	2000	4	2	1
1	3	2	1995	1997	3	2	6
1	5	4	1996	2000	5	4	10
1	5	4	1996	2000	5	4	10
1	2	2	1995	1996	2	2	3
1	2	2	1995	1996	2	2	3
1	3	3	1987	1989	3	3	4
1	9	16	1995	2001	7	16	30
1	3	3	1989	1991	3	3	1
1	12	37	1992	2001	10	37	64
1	12	9	1992	2001	10	9	11
1	8	3	1987	1994	8	3	0

1	8	3	1987	1994	8	3	0
1	11	3	1984	1994	11	3	5
1	11	3	1984	1994	11	3	5
1	7	8	1997	2001	5	8	17
1	5	5	1994	1998	5	5	6
1	5	5	1994	1998	5	5	6
1	15	32	1989	2001	13	32	64
1	6	5	1994	1999	6	5	10
1	6	5	1994	1999	6	5	10
1	6	5	1994	1999	6	5	10
1	13	2	1988	2000	13	2	17
1	3	8	1998	2000	3	8	16
1	3	8	1998	2000	3	8	16
1	5	3	1997	2001	5	3	8
1	2	2	1999	2000	2	2	1
1	3	2	1996	1998	3	2	12
1	3	2	1996	1998	3	2	12
1	9	5	1994	2001	8	5	6
1	5	2	1994	1998	5	2	0
1	3	2	1999	2001	3	2	2
1	3	2	1999	2001	3	2	2
1	3	4	1995	1997	3	4	40
1	3	4	1995	1997	3	4	40
1	3	4	1995	1997	3	4	40
1	5	3	1998	2001	4	3	1
1	2	2	1998	1999	2	2	1
1	3	4	1995	1997	3	4	2
1	3	7	1999	2001	3	7	1
1	3	7	1999	2001	3	7	1
1	3	7	1999	2001	3	7	1
1	6	7	1998	2001	4	7	16
1	7	3	1997	2001	5	3	0
1	7	3	1997	2001	5	3	0
1	13	12	1990	2001	12	12	9
1	13	12	1990	2001	12	12	9
1	3	2	1997	1999	3	2	0
1	3	2	1997	1999	3	2	0
1	3	2	1996	1998	3	2	3
1	3	2	1996	1998	3	2	3
1	6	5	1995	2000	6	5	13
1	16	28	1988	2001	14	28	32
1	2	2	1995	1996	2	2	3
1	2	2	1995	1996	2	2	3
1	6	2	1991	1996	6	2	15
1	4	2	1995	1998	4	2	3
1	10	18	1993	2001	9	18	33
1	4	3	1999	2001	3	3	1

1	4	3	1999	2001	3	3	1
1	2	2	1997	1998	2	2	0
1	2	2	1997	1998	2	2	0
1	4	3	1995	1998	4	3	11
1	5	3	1996	2000	5	3	3
1	8	4	1995	2001	7	4	6
1	8	4	1995	2001	7	4	6
1	12	28	1992	2001	10	28	95
1	12	28	1992	2001	10	28	95
1	12	28	1992	2001	10	28	95
1	9	25	1995	2001	7	25	71
1	9	25	1995	2001	7	25	71
1	9	25	1995	2001	7	25	71
1	9	25	1995	2001	7	25	71
1	8	2	1992	1999	8	2	2
1	17	9	1987	2001	15	9	7
1	12	12	1992	2001	10	12	19
1	12	12	1992	2001	10	12	19
1	5	3	1999	2001	3	3	11
1	4	2	1998	2001	4	2	4
1	9	6	1995	2001	7	6	11
1	9	2	1995	2001	7	2	7
1	2	3	1998	1999	2	3	4
1	3	2	1992	1994	3	2	1
1	4	2	1997	2000	4	2	3
1	4	7	1996	1999	4	7	11
1	4	7	1996	1999	4	7	11
1	7	4	1997	2001	5	4	1
1	12	12	1992	2001	10	12	12
1	6	4	1998	2001	4	4	3
1	6	4	1998	2001	4	4	3
1	6	4	1998	2001	4	4	3
1	7	8	1997	2001	5	8	13
1	7	8	1997	2001	5	8	13
1	6	4	1998	2001	4	4	16
1	9	3	1989	1997	9	3	1
1	5	3	1997	2001	5	3	0
1	3	2	1998	2000	3	2	12
1	3	2	1998	2000	3	2	12
1	4	2	1996	1999	4	2	2
1	2	2	1996	1997	2	2	6
1	8	24	1996	2001	6	24	29
1	11	9	1992	2001	10	9	5
1	5	10	1998	2001	4	10	31
1	5	10	1998	2001	4	10	31
1	5	10	1998	2001	4	10	31
1	8	10	1995	2001	7	10	16

1	3	2	1993	1995	3	2	2
1	3	2	1993	1995	3	2	2
1	10	5	1994	2001	8	5	12
1	6	5	1998	2001	4	5	14
1	5	2	1994	1998	5	2	1
1	2	2	1997	1998	2	2	1
1	2	2	1997	1998	2	2	1
1	7	8	1989	1995	7	8	68
1	7	8	1989	1995	7	8	68
1	5	2	1994	1998	5	2	1
1	20	2	1984	2001	18	2	1
1	3	2	1992	1994	3	2	2
1	10	14	1994	2001	8	14	51
1	4	2	1999	2001	3	2	3
1	4	7	1998	2001	4	7	16
1	4	7	1998	2001	4	7	16
1	5	6	1995	1999	5	6	9
1	5	6	1995	1999	5	6	9
1	5	3	1999	2001	3	3	5
1	12	8	1992	2001	10	8	14
1	3	2	1991	1993	3	2	7
1	5	2	1994	1998	5	2	1
1	2	2	1993	1994	2	2	5
1	2	2	1993	1994	2	2	5
1	10	23	1994	2001	8	23	91
1	7	4	1989	1995	7	4	4
1	6	2	1993	1998	6	2	2
1	5	2	1995	1999	5	2	2
1	3	2	1990	1992	3	2	0
1	6	7	1998	2001	4	7	18
1	5	3	1999	2001	3	3	0
1	8	3	1996	2001	6	3	1
1	12	3	1992	2001	10	3	4
1	2	2	1993	1994	2	2	4
1	2	2	1993	1994	2	2	4
1	4	2	1989	1992	4	2	0
1	5	2	1999	2001	3	2	0
1	5	2	1999	2001	3	2	0
1	18	10	1986	2001	16	10	9
1	15	9	1989	2001	13	9	17
1	8	3	1992	1999	8	3	0
1	18	3	1985	2001	17	3	3
1	9	15	1994	2001	8	15	165
1	6	17	1997	2001	5	17	215
1	6	17	1997	2001	5	17	215
1	6	17	1997	2001	5	17	215
1	6	17	1997	2001	5	17	215

1	4	3	1998	2001	4	3	8
1	4	3	1998	2001	4	3	8
1	6	5	1995	2000	6	5	9
1	11	6	1993	2001	9	6	5
1	3	4	1998	2000	3	4	3
1	3	4	1998	2000	3	4	3
1	8	2	1995	2001	7	2	4
1	10	10	1994	2001	8	10	17
1	8	3	1991	1998	8	3	1
1	4	3	1995	1998	4	3	13
1	4	3	1995	1998	4	3	13
1	5	7	1999	2001	3	7	20
1	9	4	1995	2001	7	4	2
1	9	4	1995	2001	7	4	2
1	5	4	1999	2001	3	4	2
1	5	4	1999	2001	3	4	2
1	5	4	1999	2001	3	4	2
1	5	4	1999	2001	3	4	2
1	7	2	1991	1997	7	2	3
1	16	12	1988	2001	14	12	25
1	16	12	1988	2001	14	12	25
1	2	2	1999	2000	2	2	0
1	9	8	1991	1999	9	8	11
1	9	8	1991	1999	9	8	11
1	9	8	1991	1999	9	8	11
1	7	5	1995	2001	7	5	3
1	7	5	1995	2001	7	5	3
1	10	9	1993	2001	9	9	5
1	11	6	1993	2001	9	6	14
1	9	5	1990	1998	9	5	7
1	16	8	1986	2001	16	8	7
1	12	13	1992	2001	10	13	34
1	4	4	1995	1998	4	4	3
1	4	4	1995	1998	4	4	3
1	2	4	1999	2000	2	4	27
1	2	4	1999	2000	2	4	27
1	5	2	1985	1989	5	2	0
1	15	15	1989	2001	13	15	8
1	5	2	1992	1996	5	2	8
1	6	6	1995	2000	6	6	14
1	6	6	1995	2000	6	6	14
1	6	6	1995	2000	6	6	14
1	8	5	1991	1998	8	5	22
1	8	5	1991	1998	8	5	22
1	2	2	1997	1998	2	2	8
1	7	5	1997	2001	5	5	9
1	2	2	1998	1999	2	2	4

1	2	2	1998	1999	2	2	4
1	3	2	1989	1991	3	2	3
1	3	2	1999	2001	3	2	2
1	3	2	1999	2001	3	2	2
1	9	10	1995	2001	7	10	27
1	7	5	1992	1998	7	5	1
1	5	3	1994	1998	5	3	5
1	5	3	1994	1998	5	3	5
1	8	16	1995	2001	7	16	38
1	8	16	1995	2001	7	16	38
1	8	16	1995	2001	7	16	38
1	8	16	1995	2001	7	16	38
1	8	16	1995	2001	7	16	38
1	10	3	1993	2001	9	3	15
1	7	4	1995	2001	7	4	3
1	4	2	1999	2001	3	2	2
1	6	6	1997	2001	5	6	18
1	4	3	1994	1997	4	3	8
1	4	3	1994	1997	4	3	8
1	3	2	1996	1998	3	2	0
1	3	2	1996	1998	3	2	0
1	8	6	1995	2001	7	6	26
1	8	6	1995	2001	7	6	26
1	5	2	1995	1999	5	2	2
1	5	4	1994	1998	5	4	2
1	7	2	1993	1999	7	2	1
1	8	5	1996	2001	6	5	4
1	8	7	1996	2001	6	7	8
1	8	7	1996	2001	6	7	8
1	6	5	1998	2001	4	5	4
1	6	5	1998	2001	4	5	4
1	2	2	1999	2000	2	2	0
1	2	2	1999	2000	2	2	0
1	2	2	1984	1985	2	2	8
1	2	2	1984	1985	2	2	8
1	2	2	1996	1997	2	2	1
1	3	2	1986	1988	3	2	0
1	3	2	1986	1988	3	2	0
1	19	22	1985	2001	17	22	43
1	10	10	1994	2001	8	10	44
1	6	8	1994	1999	6	8	13
1	6	8	1994	1999	6	8	13
1	6	8	1994	1999	6	8	13
1	11	5	1992	2001	10	5	7
1	7	3	1996	2001	6	3	8
1	7	3	1997	2001	5	3	8
1	4	4	1995	1998	4	4	2

1	4	4	1995	1998	4	4	2
1	5	8	1991	1995	5	8	54
1	5	2	1994	1998	5	2	10
1	10	6	1990	1999	10	6	10
1	10	9	1993	2001	9	9	12
1	10	9	1993	2001	9	9	12
1	17	4	1987	2001	15	4	6
1	6	3	1998	2001	4	3	3
1	6	3	1998	2001	4	3	3
1	3	2	1999	2001	3	2	0
1	3	2	1999	2001	3	2	0
1	8	7	1995	2001	7	7	4
1	8	7	1995	2001	7	7	4
1	4	4	1997	2000	4	4	4
1	4	2	1999	2001	3	2	0
1	4	2	1999	2001	3	2	0
1	9	2	1992	2000	9	2	1
1	3	2	1999	2001	3	2	4
1	3	3	1999	2001	3	3	4
1	3	3	1999	2001	3	3	4
1	13	9	1991	2001	11	9	27
1	8	4	1990	1997	8	4	9
1	11	8	1989	1999	11	8	17
1	6	2	1994	1999	6	2	0
1	10	6	1994	2001	8	6	11
1	3	4	1999	2001	3	4	12
1	4	3	1995	1998	4	3	4
1	3	4	1985	1987	3	4	32
1	3	4	1985	1987	3	4	32
1	8	3	1991	1998	8	3	0
1	16	11	1988	2001	14	11	10
1	15	7	1988	2001	14	7	4
1	3	2	1992	1994	3	2	1
1	4	2	1999	2001	3	2	4
1	13	9	1990	2001	12	9	29
1	12	6	1992	2001	10	6	23
1	12	6	1992	2001	10	6	23
1	3	4	1997	1999	3	4	7
1	3	4	1997	1999	3	4	7
1	3	4	1997	1999	3	4	7
1	9	16	1995	2001	7	16	33
1	9	16	1995	2001	7	16	33
1	9	16	1995	2001	7	16	33
1	10	3	1994	2001	8	3	1
1	3	2	1999	2001	3	2	0
1	3	2	1999	2001	3	2	0
1	12	10	1990	2001	12	10	4

1	12	10	1990	2001	12	10	4
1	3	3	1998	2000	3	3	3
1	3	3	1998	2000	3	3	3
1	3	3	1998	2000	3	3	3
1	10	11	1994	2001	8	11	26
1	10	11	1994	2001	8	11	26
1	2	2	1999	2000	2	2	1
1	10	4	1994	2001	8	4	1
1	10	4	1994	2001	8	4	1
1	3	2	1995	1997	3	2	1
1	3	2	1995	1997	3	2	1
1	12	6	1992	2001	10	6	13
1	3	3	1997	1999	3	3	0
1	4	4	1996	1999	4	4	0
1	4	4	1996	1999	4	4	0
1	4	4	1996	1999	4	4	0
1	6	3	1998	2001	4	3	8
1	6	3	1998	2001	4	3	8
1	5	6	1999	2001	3	6	23
1	5	6	1999	2001	3	6	23
1	5	6	1999	2001	3	6	23
1	9	5	1993	2001	9	5	2
1	14	8	1989	2001	13	8	5
1	2	4	1998	1999	2	4	9
1	2	4	1998	1999	2	4	9
1	6	12	1996	2001	6	12	13
1	6	12	1996	2001	6	12	13
1	5	10	1995	1999	5	10	26
1	5	4	1993	1997	5	4	12
1	5	4	1993	1997	5	4	12
1	4	3	1999	2001	3	3	5
1	4	3	1999	2001	3	3	5
1	5	8	1999	2001	3	8	14
1	5	8	1999	2001	3	8	14
1	5	8	1999	2001	3	8	14
1	10	13	1993	2001	9	13	16
1	10	13	1993	2001	9	13	16
1	4	2	1992	1995	4	2	15
1	8	8	1993	2000	8	8	19
1	8	8	1993	2000	8	8	19
1	2	4	1993	1994	2	4	1
1	7	4	1984	1990	7	4	4
1	16	21	1988	2001	14	21	28
1	8	35	1993	2000	8	35	34
1	5	7	1998	2001	4	7	9
1	5	7	1998	2001	4	7	9
1	5	7	1998	2001	4	7	9

1	5	2	1997	2001	5	2	0
1	11	10	1989	1999	11	10	20
1	6	4	1997	2001	5	4	4
1	9	5	1993	2001	9	5	26
1	3	2	1998	2000	3	2	10
1	3	2	1998	2000	3	2	10
1	10	2	1993	2001	9	2	4
1	10	2	1993	2001	9	2	0
1	6	2	1988	1993	6	2	1
1	2	2	1986	1987	2	2	7
1	5	2	1994	1998	5	2	0
1	7	2	1995	2001	7	2	5
1	8	27	1996	2001	6	27	112
1	8	27	1996	2001	6	27	112
1	8	27	1996	2001	6	27	112
1	2	3	1997	1998	2	3	2
1	2	3	1997	1998	2	3	2
1	2	3	1997	1998	2	3	2
1	4	4	1998	2001	4	4	1
1	3	4	1999	2001	3	4	4
1	7	5	1995	2001	7	5	4
1	7	5	1995	2001	7	5	4
1	7	5	1995	2001	7	5	4
1	4	2	1995	1998	4	2	3
1	7	8	1994	2000	7	8	19
1	10	6	1991	2000	10	6	4
1	10	6	1991	2000	10	6	4
1	16	9	1988	2001	14	9	1
1	16	9	1988	2001	14	9	1
1	8	5	1996	2001	6	5	7
1	8	5	1996	2001	6	5	7
1	8	5	1996	2001	6	5	7
1	8	2	1996	2001	6	2	5
1	8	2	1996	2001	6	2	5
1	7	6	1996	2001	6	6	33
1	7	6	1996	2001	6	6	33
1	7	6	1996	2001	6	6	33
1	7	2	1996	2001	6	2	10
1	4	3	1998	2001	4	3	10
1	18	7	1984	2001	18	7	16
1	18	7	1984	2001	18	7	16
1	18	7	1984	2001	18	7	16
1	11	4	1991	2001	11	4	2
1	11	4	1991	2001	11	4	2
1	7	5	1996	2001	6	5	11
1	7	5	1996	2001	6	5	11
1	9	3	1994	2001	8	3	10

1	9	3	1994	2001	8	3	10
1	7	6	1994	2000	7	6	9
1	5	7	1997	2001	5	7	9
1	5	7	1997	2001	5	7	9
1	12	8	1992	2001	10	8	3
1	12	8	1992	2001	10	8	3
1	12	8	1992	2001	10	8	3
1	16	50	1988	2001	14	50	89
1	16	50	1988	2001	14	50	89
1	6	6	1994	1999	6	6	2
1	19	9	1985	2001	17	9	24
1	2	4	1998	1999	2	4	27
1	4	2	1994	1997	4	2	1
1	3	3	1999	2001	3	3	2
1	3	3	1999	2001	3	3	2
1	3	3	1999	2001	3	3	2
1	10	21	1993	2001	9	21	63
1	10	21	1993	2001	9	21	63
1	3	10	1998	2000	3	10	96
1	3	10	1998	2000	3	10	96
1	3	10	1998	2000	3	10	96
1	3	10	1998	2000	3	10	96
1	8	2	1994	2001	8	2	0
1	6	5	1994	1999	6	5	6
1	6	5	1994	1999	6	5	6
1	7	3	1991	1997	7	3	2
1	6	5	1996	2001	6	5	34
1	6	5	1996	2001	6	5	34
1	6	5	1996	2001	6	5	34
1	5	3	1995	1999	5	3	0
1	10	4	1994	2001	8	4	6
1	10	4	1993	2001	9	4	0

art_8401y	cit_8401y	one_one	nop_gostart	gostartall	gostartall_b	gostartyall	gostartyall_b
1.14	1.5	1		GOST-art-all	1	Not_GOST-art	0
1	0.38	1		Not_GOST-art	0	Not_GOST-art	0
1	0	1		Not_GOST-art	0	Not_GOST-art	0
0.4	0.2	1		Not_GOST-art	0	Not_GOST-art	0
1.67	2.78	1		GOST-art-all	1	GOST-art-Y-al	1
1.67	2.78	1		GOST-art-all	1	GOST-art-Y-al	1
1.67	2.78	1		GOST-art-all	1	GOST-art-Y-al	1
1.67	2.78	1		GOST-art-all	1	GOST-art-Y-al	1
1.2	0.4	1		Not_GOST-art	0	Not_GOST-art	0
1.2	0.4	1		Not_GOST-art	0	Not_GOST-art	0
1	1.5	1		Not_GOST-art	0	Not_GOST-art	0
0.88	2	1		Not_GOST-art	0	Not_GOST-art	0
1.25	0.5	1		Not_GOST-art	0	Not_GOST-art	0
0.73	0.87	1		Not_GOST-art	0	Not_GOST-art	0
0.6	2.4	1		Not_GOST-art	0	Not_GOST-art	0
0.6	2.4	1		Not_GOST-art	0	Not_GOST-art	0
1.14	1.14	1		Not_GOST-art	0	Not_GOST-art	0
0.83	0.17	1		Not_GOST-art	0	Not_GOST-art	0
0.75	1.13	1		Not_GOST-art	0	Not_GOST-art	0
0.75	1.13	1		Not_GOST-art	0	Not_GOST-art	0
0.4	0.2	1		Not_GOST-art	0	Not_GOST-art	0
0.57	1.57	1		Not_GOST-art	0	Not_GOST-art	0
1	0.83	1		Not_GOST-art	0	Not_GOST-art	0
1	0.83	1		Not_GOST-art	0	Not_GOST-art	0
1	0.83	1		Not_GOST-art	0	Not_GOST-art	0
0.67	0	1		Not_GOST-art	0	Not_GOST-art	0
0.57	1.57	1		Not_GOST-art	0	Not_GOST-art	0
0.57	1.57	1		Not_GOST-art	0	Not_GOST-art	0
0.29	0.43	1		Not_GOST-art	0	Not_GOST-art	0
1.25	2.25	1		Not_GOST-art	0	Not_GOST-art	0
0.5	1	1		Not_GOST-art	0	Not_GOST-art	0
1.33	4.08	1		GOST-art-all	1	Not_GOST-art	0
1	0	1		Not_GOST-art	0	Not_GOST-art	0
0.25	0.06	1		Not_GOST-art	0	Not_GOST-art	0
0.5	0.5	1		Not_GOST-art	0	Not_GOST-art	0
1	3	1		Not_GOST-art	0	Not_GOST-art	0
1	3	1		Not_GOST-art	0	Not_GOST-art	0
0.75	0.5	1		Not_GOST-art	0	Not_GOST-art	0
0.5	0.07	1		Not_GOST-art	0	Not_GOST-art	0
0.54	0.08	1		Not_GOST-art	0	Not_GOST-art	0
0.67	0.33	1		Not_GOST-art	0	Not_GOST-art	0
0.43	0	1		Not_GOST-art	0	Not_GOST-art	0
0.67	0.33	1		Not_GOST-art	0	Not_GOST-art	0
0.67	0.33	1		Not_GOST-art	0	Not_GOST-art	0
0.67	0.33	1		Not_GOST-art	0	Not_GOST-art	0
0.6	0.2	1		Not_GOST-art	0	Not_GOST-art	0

0.6	0.2	1	Not_GOST-art	0	Not_GOST-art	0
0.46	3.69	1	Not_GOST-art	0	Not_GOST-art	0
0.8	0.2	1	Not_GOST-art	0	Not_GOST-art	0
1.33	2.33	1	Not_GOST-art	0	Not_GOST-art	0
0.4	0.4	1	Not_GOST-art	0	Not_GOST-art	0
0.38	0.13	1	Not_GOST-art	0	Not_GOST-art	0
1.33	0.67	1	Not_GOST-art	0	Not_GOST-art	0
1.8	4	1	GOST-art-all	1	GOST-art-Y-al	1
1.67	8.33	1	Not_GOST-art	0	GOST-art-Y-al	1
0.43	0.43	1	Not_GOST-art	0	Not_GOST-art	0
1	0.5	1	Not_GOST-art	0	Not_GOST-art	0
1	1.67	1	Not_GOST-art	0	Not_GOST-art	0
1.62	4.77	1	GOST-art-all	1	GOST-art-Y-al	1
0.25	1.13	1	Not_GOST-art	0	Not_GOST-art	0
0.75	1.75	1	Not_GOST-art	0	Not_GOST-art	0
0.75	1.75	1	Not_GOST-art	0	Not_GOST-art	0
0.5	0.25	1	Not_GOST-art	0	Not_GOST-art	0
0.73	1.91	1	Not_GOST-art	0	Not_GOST-art	0
0.67	0.67	1	Not_GOST-art	0	Not_GOST-art	0
0.4	0	1	Not_GOST-art	0	Not_GOST-art	0
1.87	1.93	1	GOST-art-all	1	GOST-art-Y-al	1
1.87	1.93	1	GOST-art-all	1	GOST-art-Y-al	1
1	1	1	Not_GOST-art	0	Not_GOST-art	0
1.71	2.71	1	Not_GOST-art	0	GOST-art-Y-al	1
0.67	1.67	1	Not_GOST-art	0	Not_GOST-art	0
0.67	1.67	1	Not_GOST-art	0	Not_GOST-art	0
1.5	3	1	Not_GOST-art	0	Not_GOST-art	0
0.6	0.9	1	Not_GOST-art	0	Not_GOST-art	0
0.5	0.5	1	Not_GOST-art	0	Not_GOST-art	0
3.25	4.25	1	GOST-art-all	1	GOST-art-Y-al	1
3.25	4.25	1	GOST-art-all	1	GOST-art-Y-al	1
3.25	4.25	1	GOST-art-all	1	GOST-art-Y-al	1
3.25	4.25	1	GOST-art-all	1	GOST-art-Y-al	1
3.25	4.25	1	GOST-art-all	1	GOST-art-Y-al	1
0.67	0	1	Not_GOST-art	0	Not_GOST-art	0
0.67	0	1	Not_GOST-art	0	Not_GOST-art	0
0.33	0.07	1	Not_GOST-art	0	Not_GOST-art	0
0.4	0.2	1	Not_GOST-art	0	Not_GOST-art	0
0.8	2.1	1	Not_GOST-art	0	Not_GOST-art	0
1	0.33	1	Not_GOST-art	0	Not_GOST-art	0
1	0.33	1	Not_GOST-art	0	Not_GOST-art	0
1	0.33	1	Not_GOST-art	0	Not_GOST-art	0
0.63	1.5	1	Not_GOST-art	0	Not_GOST-art	0
0.63	1.5	1	Not_GOST-art	0	Not_GOST-art	0
0.65	0.71	1	Not_GOST-art	0	Not_GOST-art	0
0.4	0.4	1	Not_GOST-art	0	Not_GOST-art	0
1	3.5	1	Not_GOST-art	0	Not_GOST-art	0

1	3.5	1	Not_GOST-art	0	Not_GOST-art	0
0.43	0.14	1	Not_GOST-art	0	Not_GOST-art	0
1	0.5	1	Not_GOST-art	0	Not_GOST-art	0
1	1.25	1	Not_GOST-art	0	Not_GOST-art	0
0.89	0.89	1	Not_GOST-art	0	Not_GOST-art	0
0.67	0	1	Not_GOST-art	0	Not_GOST-art	0
1.25	2.33	1	GOST-art-all	1	Not_GOST-art	0
1.25	2.33	1	GOST-art-all	1	Not_GOST-art	0
0.5	0.75	1	Not_GOST-art	0	Not_GOST-art	0
0.75	0.5	1	Not_GOST-art	0	Not_GOST-art	0
0.78	1.67	1	Not_GOST-art	0	Not_GOST-art	0
0.67	0.67	1	Not_GOST-art	0	Not_GOST-art	0
0.67	0.67	1	Not_GOST-art	0	Not_GOST-art	0
0.22	0	1	Not_GOST-art	0	Not_GOST-art	0
0.33	0.5	1	Not_GOST-art	0	Not_GOST-art	0
0.5	0	1	Not_GOST-art	0	Not_GOST-art	0
0.71	0.43	1	Not_GOST-art	0	Not_GOST-art	0
0.27	0	1	Not_GOST-art	0	Not_GOST-art	0
0.27	0	1	Not_GOST-art	0	Not_GOST-art	0
0.67	0.33	1	Not_GOST-art	0	Not_GOST-art	0
0.36	1.82	1	Not_GOST-art	0	Not_GOST-art	0
0.5	1	1	Not_GOST-art	0	Not_GOST-art	0
0.5	1	1	Not_GOST-art	0	Not_GOST-art	0
0.4	0.4	1	Not_GOST-art	0	Not_GOST-art	0
0.75	0.38	1	Not_GOST-art	0	Not_GOST-art	0
0.8	1.2	1	Not_GOST-art	0	Not_GOST-art	0
0.8	1.2	1	Not_GOST-art	0	Not_GOST-art	0
1.57	6.71	1	Not_GOST-art	0	GOST-art-Y-al	1
1.57	6.71	1	Not_GOST-art	0	GOST-art-Y-al	1
2.3	5	1	GOST-art-all	1	GOST-art-Y-al	1
2.3	5	1	GOST-art-all	1	GOST-art-Y-al	1
0.6	0.9	1	Not_GOST-art	0	Not_GOST-art	0
1	13.25	1	Not_GOST-art	0	Not_GOST-art	0
1	13.25	1	Not_GOST-art	0	Not_GOST-art	0
1	3	1	Not_GOST-art	0	Not_GOST-art	0
2	2.5	1	Not_GOST-art	0	GOST-art-Y-al	1
2	2.5	1	Not_GOST-art	0	GOST-art-Y-al	1
1.75	3.75	1	Not_GOST-art	0	GOST-art-Y-al	1
1.75	3.75	1	Not_GOST-art	0	GOST-art-Y-al	1
0.75	16.5	1	Not_GOST-art	0	Not_GOST-art	0
1.5	2	1	Not_GOST-art	0	Not_GOST-art	0
1.5	2	1	Not_GOST-art	0	Not_GOST-art	0
1.63	2.06	1	GOST-art-all	1	GOST-art-Y-al	1
0.67	0	1	Not_GOST-art	0	Not_GOST-art	0
1.92	5	1	GOST-art-all	1	GOST-art-Y-al	1
1.92	5	1	GOST-art-all	1	GOST-art-Y-al	1
1	2	1	Not_GOST-art	0	Not_GOST-art	0

0.67	0.33	1	Not_GOST-art	0	Not_GOST-art	0
0.67	0.33	1	Not_GOST-art	0	Not_GOST-art	0
1.62	1.15	1	GOST-art-all	1	GOST-art-Y-al	1
1.62	1.15	1	GOST-art-all	1	GOST-art-Y-al	1
1.62	1.15	1	GOST-art-all	1	GOST-art-Y-al	1
0.71	0.57	1	Not_GOST-art	0	Not_GOST-art	0
1	0.33	1	Not_GOST-art	0	Not_GOST-art	0
1	0.33	1	Not_GOST-art	0	Not_GOST-art	0
0.44	0.78	1	Not_GOST-art	0	Not_GOST-art	0
1	0.25	1	Not_GOST-art	0	Not_GOST-art	0
1	0.25	1	Not_GOST-art	0	Not_GOST-art	0
0.67	5.33	1	Not_GOST-art	0	Not_GOST-art	0
0.67	5.33	1	Not_GOST-art	0	Not_GOST-art	0
5	9	1	GOST-art-all	1	GOST-art-Y-al	1
0.67	1.33	1	Not_GOST-art	0	Not_GOST-art	0
0.67	1.33	1	Not_GOST-art	0	Not_GOST-art	0
2.5	8	1	Not_GOST-art	0	GOST-art-Y-al	1
1	2.67	1	Not_GOST-art	0	Not_GOST-art	0
1	2.67	1	Not_GOST-art	0	Not_GOST-art	0
1	2.67	1	Not_GOST-art	0	Not_GOST-art	0
0.22	0.67	1	Not_GOST-art	0	Not_GOST-art	0
0.59	0.41	1	Not_GOST-art	0	Not_GOST-art	0
1	2	1	Not_GOST-art	0	Not_GOST-art	0
1	2	1	Not_GOST-art	0	Not_GOST-art	0
1	2	1	Not_GOST-art	0	Not_GOST-art	0
1	0.5	1	Not_GOST-art	0	Not_GOST-art	0
0.29	0.71	1	Not_GOST-art	0	Not_GOST-art	0
0.33	1.17	1	Not_GOST-art	0	Not_GOST-art	0
1.86	4	1	GOST-art-all	1	GOST-art-Y-al	1
1	4	1	Not_GOST-art	0	Not_GOST-art	0
2	1	1	Not_GOST-art	0	GOST-art-Y-al	1
2	1	1	Not_GOST-art	0	GOST-art-Y-al	1
2	11	1	Not_GOST-art	0	GOST-art-Y-al	1
0.43	0.29	1	Not_GOST-art	0	Not_GOST-art	0
0.67	0.67	1	Not_GOST-art	0	Not_GOST-art	0
0.67	0.67	1	Not_GOST-art	0	Not_GOST-art	0
0.38	0	1	Not_GOST-art	0	Not_GOST-art	0
0.5	1.25	1	Not_GOST-art	0	Not_GOST-art	0
0.5	9.63	1	Not_GOST-art	0	Not_GOST-art	0
0.86	1.14	1	Not_GOST-art	0	Not_GOST-art	0
0.75	1	1	Not_GOST-art	0	Not_GOST-art	0
2.5	2	1	Not_GOST-art	0	GOST-art-Y-al	1
2.5	2	1	Not_GOST-art	0	GOST-art-Y-al	1
0.22	0	1	Not_GOST-art	0	Not_GOST-art	0
1.2	0.8	1	Not_GOST-art	0	Not_GOST-art	0
1.2	0.8	1	Not_GOST-art	0	Not_GOST-art	0
1.2	0.8	1	Not_GOST-art	0	Not_GOST-art	0

0.67	0.67	1	Not_GOST-art	0	Not_GOST-art	0
0.67	0.67	1	Not_GOST-art	0	Not_GOST-art	0
0.75	2	1	Not_GOST-art	0	Not_GOST-art	0
0.29	0.14	1	Not_GOST-art	0	Not_GOST-art	0
1	20	1	Not_GOST-art	0	Not_GOST-art	0
1	20	1	Not_GOST-art	0	Not_GOST-art	0
1	2	1	Not_GOST-art	0	Not_GOST-art	0
1	2	1	Not_GOST-art	0	Not_GOST-art	0
1	2	1	Not_GOST-art	0	Not_GOST-art	0
1	0	1	Not_GOST-art	0	Not_GOST-art	0
1.67	2.67	1	Not_GOST-art	0	GOST-art-Y-al	1
1.67	2.67	1	Not_GOST-art	0	GOST-art-Y-al	1
1	2	1	Not_GOST-art	0	Not_GOST-art	0
1	2	1	Not_GOST-art	0	Not_GOST-art	0
0.8	2.4	1	Not_GOST-art	0	Not_GOST-art	0
1.33	4.33	1	Not_GOST-art	0	Not_GOST-art	0
1.33	4.33	1	Not_GOST-art	0	Not_GOST-art	0
0.13	0.07	1	Not_GOST-art	0	Not_GOST-art	0
0.5	0.5	1	Not_GOST-art	0	Not_GOST-art	0
0.67	1.33	1	Not_GOST-art	0	Not_GOST-art	0
0.67	1.33	1	Not_GOST-art	0	Not_GOST-art	0
0.4	0.6	1	Not_GOST-art	0	Not_GOST-art	0
1	1.6	1	Not_GOST-art	0	Not_GOST-art	0
1	1.6	1	Not_GOST-art	0	Not_GOST-art	0
0.5	1.25	1	Not_GOST-art	0	Not_GOST-art	0
0.5	1.25	1	Not_GOST-art	0	Not_GOST-art	0
1	3	1	Not_GOST-art	0	Not_GOST-art	0
1	3	1	Not_GOST-art	0	Not_GOST-art	0
1	3	1	Not_GOST-art	0	Not_GOST-art	0
0.5	0.08	1	Not_GOST-art	0	Not_GOST-art	0
0.5	0.08	1	Not_GOST-art	0	Not_GOST-art	0
1	0.5	1	Not_GOST-art	0	Not_GOST-art	0
1	0.5	1	Not_GOST-art	0	Not_GOST-art	0
1	0.5	1	Not_GOST-art	0	Not_GOST-art	0
1.44	6.89	1	GOST-art-all	1	Not_GOST-art	0
0.62	0.54	1	Not_GOST-art	0	Not_GOST-art	0
0.67	1.33	1	Not_GOST-art	0	Not_GOST-art	0
1.2	1.8	1	Not_GOST-art	0	Not_GOST-art	0
3	5.67	1	Not_GOST-art	0	GOST-art-Y-al	1
3	5.67	1	Not_GOST-art	0	GOST-art-Y-al	1
3	5.67	1	Not_GOST-art	0	GOST-art-Y-al	1
3	5.67	1	Not_GOST-art	0	GOST-art-Y-al	1
1	3	1	Not_GOST-art	0	Not_GOST-art	0
0.67	4	1	Not_GOST-art	0	Not_GOST-art	0
0.67	1.67	1	Not_GOST-art	0	Not_GOST-art	0
0.67	1.67	1	Not_GOST-art	0	Not_GOST-art	0
0.38	1.88	1	Not_GOST-art	0	Not_GOST-art	0

1	0.33	1	Not_GOST-art	0	Not_GOST-art	0
1	0.33	1	Not_GOST-art	0	Not_GOST-art	0
0.67	0.33	1	Not_GOST-art	0	Not_GOST-art	0
0.8	1	1	Not_GOST-art	0	Not_GOST-art	0
0.8	1	1	Not_GOST-art	0	Not_GOST-art	0
1	1.8	1	Not_GOST-art	0	Not_GOST-art	0
0.67	2	1	Not_GOST-art	0	Not_GOST-art	0
0.67	2	1	Not_GOST-art	0	Not_GOST-art	0
0.5	0.75	1	Not_GOST-art	0	Not_GOST-art	0
1	2.67	1	Not_GOST-art	0	Not_GOST-art	0
0.39	0.22	1	Not_GOST-art	0	Not_GOST-art	0
0.33	0.2	1	Not_GOST-art	0	Not_GOST-art	0
1	0.5	1	Not_GOST-art	0	Not_GOST-art	0
1	0.5	1	Not_GOST-art	0	Not_GOST-art	0
1.6	1.2	1	Not_GOST-art	0	GOST-art-Y-al	1
0.43	1.29	1	Not_GOST-art	0	Not_GOST-art	0
0.75	0.25	1	Not_GOST-art	0	Not_GOST-art	0
0.75	0.25	1	Not_GOST-art	0	Not_GOST-art	0
0.75	0.25	1	Not_GOST-art	0	Not_GOST-art	0
0.67	0.33	1	Not_GOST-art	0	Not_GOST-art	0
0.67	0.33	1	Not_GOST-art	0	Not_GOST-art	0
3	12.67	1	Not_GOST-art	0	GOST-art-Y-al	1
3	12.67	1	Not_GOST-art	0	GOST-art-Y-al	1
3	12.67	1	Not_GOST-art	0	GOST-art-Y-al	1
1	4.5	1	Not_GOST-art	0	Not_GOST-art	0
0.4	0.4	1	Not_GOST-art	0	Not_GOST-art	0
1	2.4	1	Not_GOST-art	0	Not_GOST-art	0
1	2.4	1	Not_GOST-art	0	Not_GOST-art	0
0.67	0.33	1	Not_GOST-art	0	Not_GOST-art	0
0.67	0.33	1	Not_GOST-art	0	Not_GOST-art	0
4.29	21	1	GOST-art-all	1	GOST-art-Y-al	1
4.29	21	1	GOST-art-all	1	GOST-art-Y-al	1
4.29	21	1	GOST-art-all	1	GOST-art-Y-al	1
0.43	1.86	1	Not_GOST-art	0	Not_GOST-art	0
0.33	2.33	1	Not_GOST-art	0	Not_GOST-art	0
0.75	0.25	1	Not_GOST-art	0	Not_GOST-art	0
1	1.5	1	Not_GOST-art	0	Not_GOST-art	0
0.6	1	1	Not_GOST-art	0	Not_GOST-art	0
0.67	2.33	1	Not_GOST-art	0	Not_GOST-art	0
0.67	2.33	1	Not_GOST-art	0	Not_GOST-art	0
1.33	2.33	1	Not_GOST-art	0	Not_GOST-art	0
1.33	2.33	1	Not_GOST-art	0	Not_GOST-art	0
1.33	2.33	1	Not_GOST-art	0	Not_GOST-art	0
1.45	1.18	1	GOST-art-all	1	Not_GOST-art	0
0.41	0.47	1	Not_GOST-art	0	Not_GOST-art	0
0.67	0.67	1	Not_GOST-art	0	Not_GOST-art	0
1.33	0.67	1	Not_GOST-art	0	Not_GOST-art	0

0.88	1.63	1	Not_GOST-art	0	Not_GOST-art	0
0.88	1.63	1	Not_GOST-art	0	Not_GOST-art	0
0.17	1	1	Not_GOST-art	0	Not_GOST-art	0
1.5	2.5	1	GOST-art-all	1	Not_GOST-art	0
1	1.25	1	Not_GOST-art	0	Not_GOST-art	0
1	1.25	1	Not_GOST-art	0	Not_GOST-art	0
0.33	1.56	1	Not_GOST-art	0	Not_GOST-art	0
0.57	0	1	Not_GOST-art	0	Not_GOST-art	0
1	1.4	1	Not_GOST-art	0	Not_GOST-art	0
0.67	1.33	1	Not_GOST-art	0	Not_GOST-art	0
0.67	1.33	1	Not_GOST-art	0	Not_GOST-art	0
0.75	2.75	1	Not_GOST-art	0	Not_GOST-art	0
0.67	0.67	1	Not_GOST-art	0	Not_GOST-art	0
0.67	0.67	1	Not_GOST-art	0	Not_GOST-art	0
0.4	1	1	Not_GOST-art	0	Not_GOST-art	0
0.88	1.13	1	GOST-art-all	1	Not_GOST-art	0
0.88	1.13	1	GOST-art-all	1	Not_GOST-art	0
0.38	0.13	1	Not_GOST-art	0	Not_GOST-art	0
0.38	0.13	1	Not_GOST-art	0	Not_GOST-art	0
0.45	0.27	1	Not_GOST-art	0	Not_GOST-art	0
0.5	0.17	1	Not_GOST-art	0	Not_GOST-art	0
0.5	0.25	1	Not_GOST-art	0	Not_GOST-art	0
1.71	2	1	Not_GOST-art	0	GOST-art-Y-al	1
0.67	0	1	Not_GOST-art	0	Not_GOST-art	0
0.67	0	1	Not_GOST-art	0	Not_GOST-art	0
1.5	1.75	1	Not_GOST-art	0	Not_GOST-art	0
1.5	1.75	1	Not_GOST-art	0	Not_GOST-art	0
1.5	1.75	1	Not_GOST-art	0	Not_GOST-art	0
1.5	1.75	1	Not_GOST-art	0	Not_GOST-art	0
1.5	1.75	1	Not_GOST-art	0	Not_GOST-art	0
0.75	0.5	1	Not_GOST-art	0	Not_GOST-art	0
0.22	0.11	1	Not_GOST-art	0	Not_GOST-art	0
1	3.5	1	Not_GOST-art	0	Not_GOST-art	0
0.6	0.8	1	Not_GOST-art	0	Not_GOST-art	0
0.67	3.67	1	Not_GOST-art	0	Not_GOST-art	0
0.67	3.67	1	Not_GOST-art	0	Not_GOST-art	0
1	1.67	1	Not_GOST-art	0	Not_GOST-art	0
1.4	2.8	1	Not_GOST-art	0	Not_GOST-art	0
0.67	2	1	Not_GOST-art	0	Not_GOST-art	0
0.67	2	1	Not_GOST-art	0	Not_GOST-art	0
0.75	1.25	1	Not_GOST-art	0	Not_GOST-art	0
1.25	3.5	1	Not_GOST-art	0	Not_GOST-art	0
1.25	3.5	1	Not_GOST-art	0	Not_GOST-art	0
0.64	1.36	1	Not_GOST-art	0	Not_GOST-art	0
0.64	1.36	1	Not_GOST-art	0	Not_GOST-art	0
1.2	1.2	1	Not_GOST-art	0	Not_GOST-art	0
1.2	1.2	1	Not_GOST-art	0	Not_GOST-art	0

0.44	0.22	1	Not_GOST-art	0	Not_GOST-art	0
1	0	1	Not_GOST-art	0	Not_GOST-art	0
1	0	1	Not_GOST-art	0	Not_GOST-art	0
0.4	0.2	1	Not_GOST-art	0	Not_GOST-art	0
0.57	0.86	1	Not_GOST-art	0	Not_GOST-art	0
1	2	1	Not_GOST-art	0	Not_GOST-art	0
1	0	1	Not_GOST-art	0	Not_GOST-art	0
0.33	1.22	1	Not_GOST-art	0	Not_GOST-art	0
0.5	0	1	Not_GOST-art	0	Not_GOST-art	0
0.77	1.85	1	Not_GOST-art	0	Not_GOST-art	0
0.77	1.85	1	Not_GOST-art	0	Not_GOST-art	0
0.56	1.44	1	Not_GOST-art	0	Not_GOST-art	0
0.56	1.44	1	Not_GOST-art	0	Not_GOST-art	0
1.67	4.67	1	Not_GOST-art	0	GOST-art-Y-al	1
3.33	3	1	Not_GOST-art	0	GOST-art-Y-al	1
3.33	3	1	Not_GOST-art	0	GOST-art-Y-al	1
0.93	0.73	1	GOST-art-all	1	Not_GOST-art	0
0.57	0.29	1	Not_GOST-art	0	Not_GOST-art	0
1.07	0.07	1	GOST-art-all	1	Not_GOST-art	0
1	2.5	1	Not_GOST-art	0	Not_GOST-art	0
1	1	1	Not_GOST-art	0	Not_GOST-art	0
0.5	0.61	1	Not_GOST-art	0	Not_GOST-art	0
0.75	0	1	Not_GOST-art	0	Not_GOST-art	0
0.75	0	1	Not_GOST-art	0	Not_GOST-art	0
1.33	8.67	1	Not_GOST-art	0	Not_GOST-art	0
1.33	8.67	1	Not_GOST-art	0	Not_GOST-art	0
1.23	1.15	1	GOST-art-all	1	Not_GOST-art	0
0.38	0.5	1	Not_GOST-art	0	Not_GOST-art	0
0.33	0.33	1	Not_GOST-art	0	Not_GOST-art	0
0.38	0.15	1	Not_GOST-art	0	Not_GOST-art	0
0.23	0	1	Not_GOST-art	0	Not_GOST-art	0
1	1.67	1	Not_GOST-art	0	Not_GOST-art	0
1	1.67	1	Not_GOST-art	0	Not_GOST-art	0
1	1.67	1	Not_GOST-art	0	Not_GOST-art	0
0.4	0.47	1	Not_GOST-art	0	Not_GOST-art	0
0.2	0.13	1	Not_GOST-art	0	Not_GOST-art	0
0.8	1	1	Not_GOST-art	0	Not_GOST-art	0
0.57	0.14	1	Not_GOST-art	0	Not_GOST-art	0
0.15	0.46	1	Not_GOST-art	0	Not_GOST-art	0
0.33	0	1	Not_GOST-art	0	Not_GOST-art	0
0.29	0.57	1	Not_GOST-art	0	Not_GOST-art	0
0.2	0	1	Not_GOST-art	0	Not_GOST-art	0
1	3.5	1	Not_GOST-art	0	Not_GOST-art	0
1.8	4.5	1	GOST-art-all	1	GOST-art-Y-al	1
0.5	5.33	1	Not_GOST-art	0	Not_GOST-art	0
1	0.67	1	Not_GOST-art	0	Not_GOST-art	0
1	0.67	1	Not_GOST-art	0	Not_GOST-art	0

0.22	0.22	1	Not_GOST-art	0	Not_GOST-art	0
0.86	1	1	Not_GOST-art	0	Not_GOST-art	0
0.75	3	1	Not_GOST-art	0	Not_GOST-art	0
0.75	3	1	Not_GOST-art	0	Not_GOST-art	0
0.38	0.15	1	Not_GOST-art	0	Not_GOST-art	0
1.14	1.14	1	Not_GOST-art	0	Not_GOST-art	0
1.14	1.14	1	Not_GOST-art	0	Not_GOST-art	0
3	9.5	1	Not_GOST-art	0	GOST-art-Y-al	1
3	9.5	1	Not_GOST-art	0	GOST-art-Y-al	1
0.67	0.67	1	Not_GOST-art	0	Not_GOST-art	0
0.67	0.67	1	Not_GOST-art	0	Not_GOST-art	0
0.38	0.25	1	Not_GOST-art	0	Not_GOST-art	0
0.38	0.25	1	Not_GOST-art	0	Not_GOST-art	0
1	0	1	Not_GOST-art	0	Not_GOST-art	0
0.5	0	1	Not_GOST-art	0	Not_GOST-art	0
1.25	2.75	1	Not_GOST-art	0	Not_GOST-art	0
1.25	2.75	1	Not_GOST-art	0	Not_GOST-art	0
0.67	1.33	1	Not_GOST-art	0	Not_GOST-art	0
0.29	0.14	1	Not_GOST-art	0	Not_GOST-art	0
0.8	0.2	1	Not_GOST-art	0	Not_GOST-art	0
0.8	0.2	1	Not_GOST-art	0	Not_GOST-art	0
1.33	2.33	1	Not_GOST-art	0	Not_GOST-art	0
0.5	1.83	1	Not_GOST-art	0	Not_GOST-art	0
0.5	1.83	1	Not_GOST-art	0	Not_GOST-art	0
0.67	0.33	1	Not_GOST-art	0	Not_GOST-art	0
0.67	0.33	1	Not_GOST-art	0	Not_GOST-art	0
0.5	0.25	1	Not_GOST-art	0	Not_GOST-art	0
1.43	2.57	1	Not_GOST-art	0	Not_GOST-art	0
0.67	2.67	1	Not_GOST-art	0	Not_GOST-art	0
0.67	2.67	1	Not_GOST-art	0	Not_GOST-art	0
0.33	1	1	Not_GOST-art	0	Not_GOST-art	0
0.83	1.83	1	Not_GOST-art	0	Not_GOST-art	0
0.83	1.83	1	Not_GOST-art	0	Not_GOST-art	0
0.75	1.75	1	Not_GOST-art	0	Not_GOST-art	0
0.13	0.13	1	Not_GOST-art	0	Not_GOST-art	0
0.71	1.43	1	Not_GOST-art	0	Not_GOST-art	0
0.5	0.5	1	Not_GOST-art	0	Not_GOST-art	0
3.08	1.85	1	GOST-art-all	1	GOST-art-Y-al	1
0.15	0.08	1	Not_GOST-art	0	Not_GOST-art	0
0.9	0.5	1	Not_GOST-art	0	Not_GOST-art	0
0.9	0.5	1	Not_GOST-art	0	Not_GOST-art	0
0.67	3.33	1	Not_GOST-art	0	Not_GOST-art	0
0.29	0.43	1	Not_GOST-art	0	Not_GOST-art	0
0.29	0	1	Not_GOST-art	0	Not_GOST-art	0
0.4	0.3	1	Not_GOST-art	0	Not_GOST-art	0
0.88	0.88	1	Not_GOST-art	0	Not_GOST-art	0
0.88	0.88	1	Not_GOST-art	0	Not_GOST-art	0

0.22	1.78	1	Not_GOST-art	0	Not_GOST-art	0
0.4	0	1	Not_GOST-art	0	Not_GOST-art	0
0.4	0	1	Not_GOST-art	0	Not_GOST-art	0
1	3.5	1	Not_GOST-art	0	Not_GOST-art	0
1	3.5	1	Not_GOST-art	0	Not_GOST-art	0
2.08	3.85	1	GOST-art-all	1	GOST-art-Y-al	1
2.08	3.85	1	GOST-art-all	1	GOST-art-Y-al	1
0.69	0	1	Not_GOST-art	0	Not_GOST-art	0
1.33	2.33	1	Not_GOST-art	0	Not_GOST-art	0
1.2	0.6	1	Not_GOST-art	0	Not_GOST-art	0
1.2	0.6	1	Not_GOST-art	0	Not_GOST-art	0
3.57	5.43	1	GOST-art-all	1	GOST-art-Y-al	1
3.57	5.43	1	GOST-art-all	1	GOST-art-Y-al	1
0.33	0.33	1	Not_GOST-art	0	Not_GOST-art	0
1.4	1.6	1	Not_GOST-art	0	Not_GOST-art	0
1.33	4.67	1	Not_GOST-art	0	Not_GOST-art	0
1.33	4.67	1	Not_GOST-art	0	Not_GOST-art	0
1.33	4.67	1	Not_GOST-art	0	Not_GOST-art	0
1.33	4.67	1	Not_GOST-art	0	Not_GOST-art	0
1.25	9.75	1	Not_GOST-art	0	Not_GOST-art	0
1.25	9.75	1	Not_GOST-art	0	Not_GOST-art	0
0.75	0	1	Not_GOST-art	0	Not_GOST-art	0
0.75	0	1	Not_GOST-art	0	Not_GOST-art	0
0.5	0.25	1	Not_GOST-art	0	Not_GOST-art	0
0.86	0.57	1	Not_GOST-art	0	Not_GOST-art	0
0.86	0.57	1	Not_GOST-art	0	Not_GOST-art	0
0.56	1	1	Not_GOST-art	0	Not_GOST-art	0
0.56	1	1	Not_GOST-art	0	Not_GOST-art	0
0.61	0.39	1	Not_GOST-art	0	Not_GOST-art	0
1	5.5	1	Not_GOST-art	0	Not_GOST-art	0
1	5.5	1	Not_GOST-art	0	Not_GOST-art	0
0.4	0	1	Not_GOST-art	0	Not_GOST-art	0
0.22	0.44	1	Not_GOST-art	0	Not_GOST-art	0
0.22	0.44	1	Not_GOST-art	0	Not_GOST-art	0
1	2	1	Not_GOST-art	0	Not_GOST-art	0
1	1.25	1	Not_GOST-art	0	Not_GOST-art	0
0.67	2	1	Not_GOST-art	0	Not_GOST-art	0
0.67	2	1	Not_GOST-art	0	Not_GOST-art	0
1	1	1	Not_GOST-art	0	Not_GOST-art	0
0.67	2	1	Not_GOST-art	0	Not_GOST-art	0
0.67	2	1	Not_GOST-art	0	Not_GOST-art	0
0.67	0.67	1	Not_GOST-art	0	Not_GOST-art	0
0.67	0.67	1	Not_GOST-art	0	Not_GOST-art	0
1	5.5	1	Not_GOST-art	0	Not_GOST-art	0
0.7	0.6	1	Not_GOST-art	0	Not_GOST-art	0
1.33	1.33	1	Not_GOST-art	0	Not_GOST-art	0
0.5	0.17	1	Not_GOST-art	0	Not_GOST-art	0

1.67	3.33	1	Not_GOST-art	0	GOST-art-Y-al	1
1.67	3.33	1	Not_GOST-art	0	GOST-art-Y-al	1
0.5	0.17	1	Not_GOST-art	0	Not_GOST-art	0
0.57	1.71	1	Not_GOST-art	0	Not_GOST-art	0
0.57	1.71	1	Not_GOST-art	0	Not_GOST-art	0
0.31	0.15	1	Not_GOST-art	0	Not_GOST-art	0
2	0.4	1	Not_GOST-art	0	GOST-art-Y-al	1
0.18	0.18	1	Not_GOST-art	0	Not_GOST-art	0
0.5	1.5	1	Not_GOST-art	0	Not_GOST-art	0
1.75	1.75	1	Not_GOST-art	0	GOST-art-Y-al	1
1.2	2.4	1	Not_GOST-art	0	Not_GOST-art	0
1.2	2.4	1	Not_GOST-art	0	Not_GOST-art	0
1.2	2.4	1	Not_GOST-art	0	Not_GOST-art	0
0.67	2	1	Not_GOST-art	0	Not_GOST-art	0
1	0.5	1	Not_GOST-art	0	Not_GOST-art	0
1	0.5	1	Not_GOST-art	0	Not_GOST-art	0
0.67	2	1	Not_GOST-art	0	Not_GOST-art	0
0.67	2	1	Not_GOST-art	0	Not_GOST-art	0
0.5	0	1	Not_GOST-art	0	Not_GOST-art	0
0.83	1.67	1	Not_GOST-art	0	Not_GOST-art	0
1	1.8	1	Not_GOST-art	0	Not_GOST-art	0
1	1.8	1	Not_GOST-art	0	Not_GOST-art	0
1.17	2.17	1	Not_GOST-art	0	Not_GOST-art	0
3.56	10.44	1	GOST-art-all	1	GOST-art-Y-al	1
3.56	10.44	1	GOST-art-all	1	GOST-art-Y-al	1
3.56	10.44	1	GOST-art-all	1	GOST-art-Y-al	1
0.75	2	1	Not_GOST-art	0	Not_GOST-art	0
0.75	2	1	Not_GOST-art	0	Not_GOST-art	0
0.8	2	1	Not_GOST-art	0	Not_GOST-art	0
1.33	2.33	1	Not_GOST-art	0	Not_GOST-art	0
1.33	2.33	1	Not_GOST-art	0	Not_GOST-art	0
0.33	0.17	1	Not_GOST-art	0	Not_GOST-art	0
0.38	1.13	1	Not_GOST-art	0	Not_GOST-art	0
0.5	0.33	1	Not_GOST-art	0	Not_GOST-art	0
1.54	1.77	1	GOST-art-all	1	Not_GOST-art	0
0.35	0.06	1	Not_GOST-art	0	Not_GOST-art	0
1	5	1	Not_GOST-art	0	Not_GOST-art	0
1	5	1	Not_GOST-art	0	Not_GOST-art	0
0.18	0.36	1	Not_GOST-art	0	Not_GOST-art	0
0.5	0	1	Not_GOST-art	0	Not_GOST-art	0
0.43	0.14	1	Not_GOST-art	0	Not_GOST-art	0
1.6	1.7	1	GOST-art-all	1	GOST-art-Y-al	1
0.63	3	1	Not_GOST-art	0	Not_GOST-art	0
2	3.5	1	Not_GOST-art	0	GOST-art-Y-al	1
1	0.5	1	Not_GOST-art	0	Not_GOST-art	0
1	0.5	1	Not_GOST-art	0	Not_GOST-art	0
1	0.5	1	Not_GOST-art	0	Not_GOST-art	0

0.6	0.6	1	Not_GOST-art	0	Not_GOST-art	0
1.15	1.46	1	GOST-art-all	1	Not_GOST-art	0
0.86	2.36	1	Not_GOST-art	0	Not_GOST-art	0
0.67	0.67	1	Not_GOST-art	0	Not_GOST-art	0
0.5	0.67	1	Not_GOST-art	0	Not_GOST-art	0
0.56	0.33	1	Not_GOST-art	0	Not_GOST-art	0
0.24	0.18	1	Not_GOST-art	0	Not_GOST-art	0
0.67	0	1	Not_GOST-art	0	Not_GOST-art	0
0.67	0	1	Not_GOST-art	0	Not_GOST-art	0
0.67	1.67	1	Not_GOST-art	0	Not_GOST-art	0
2.6	18.8	1	GOST-art-all	1	GOST-art-Y-al	1
2.6	18.8	1	GOST-art-all	1	GOST-art-Y-al	1
2.6	18.8	1	GOST-art-all	1	GOST-art-Y-al	1
1	0.25	1	Not_GOST-art	0	Not_GOST-art	0
1	0.67	1	Not_GOST-art	0	Not_GOST-art	0
1	0.5	1	Not_GOST-art	0	Not_GOST-art	0
0.5	0.89	1	Not_GOST-art	0	Not_GOST-art	0
0.5	0.89	1	Not_GOST-art	0	Not_GOST-art	0
0.47	0.47	1	Not_GOST-art	0	Not_GOST-art	0
0.61	0.61	1	Not_GOST-art	0	Not_GOST-art	0
1.2	2	1	Not_GOST-art	0	Not_GOST-art	0
1.2	2	1	Not_GOST-art	0	Not_GOST-art	0
1.2	2	1	Not_GOST-art	0	Not_GOST-art	0
1.2	2	1	Not_GOST-art	0	Not_GOST-art	0
0.75	1	1	Not_GOST-art	0	Not_GOST-art	0
0.75	1	1	Not_GOST-art	0	Not_GOST-art	0
0.5	0.75	1	Not_GOST-art	0	Not_GOST-art	0
1	1	1	Not_GOST-art	0	Not_GOST-art	0
0.43	0.43	1	Not_GOST-art	0	Not_GOST-art	0
0.43	0.14	1	Not_GOST-art	0	Not_GOST-art	0
1.67	3	1	Not_GOST-art	0	GOST-art-Y-al	1
1.67	3	1	Not_GOST-art	0	GOST-art-Y-al	1
1.67	3	1	Not_GOST-art	0	GOST-art-Y-al	1
1.67	3	1	Not_GOST-art	0	GOST-art-Y-al	1
0.67	1.67	1	Not_GOST-art	0	Not_GOST-art	0
0.5	0.25	1	Not_GOST-art	0	Not_GOST-art	0
1.29	1.57	1	Not_GOST-art	0	Not_GOST-art	0
1	0	1	Not_GOST-art	0	Not_GOST-art	0
1	0	1	Not_GOST-art	0	Not_GOST-art	0
0.53	1.47	1	Not_GOST-art	0	Not_GOST-art	0
0.6	0.4	1	Not_GOST-art	0	Not_GOST-art	0
0.6	0.4	1	Not_GOST-art	0	Not_GOST-art	0
0.38	0.46	1	Not_GOST-art	0	Not_GOST-art	0
0.33	0.5	1	Not_GOST-art	0	Not_GOST-art	0
1.2	0.2	1	Not_GOST-art	0	Not_GOST-art	0
0.75	0.75	1	Not_GOST-art	0	Not_GOST-art	0
1	1	1	Not_GOST-art	0	Not_GOST-art	0

1	0	1	Not_GOST-art	0	Not_GOST-art	0
1	0	1	Not_GOST-art	0	Not_GOST-art	0
1	1	1	Not_GOST-art	0	Not_GOST-art	0
1	1	1	Not_GOST-art	0	Not_GOST-art	0
2	5	1	Not_GOST-art	0	GOST-art-Y-al	1
2	5	1	Not_GOST-art	0	GOST-art-Y-al	1
2	5	1	Not_GOST-art	0	GOST-art-Y-al	1
1.2	0.4	1	Not_GOST-art	0	Not_GOST-art	0
1.2	0.4	1	Not_GOST-art	0	Not_GOST-art	0
1.2	0.4	1	Not_GOST-art	0	Not_GOST-art	0
1	1	1	Not_GOST-art	0	Not_GOST-art	0
1	1	1	Not_GOST-art	0	Not_GOST-art	0
1.33	0	1	Not_GOST-art	0	Not_GOST-art	0
1	0	1	Not_GOST-art	0	Not_GOST-art	0
1	0	1	Not_GOST-art	0	Not_GOST-art	0
1.25	2.25	1	Not_GOST-art	0	Not_GOST-art	0
1.25	2.25	1	Not_GOST-art	0	Not_GOST-art	0
1.25	3.75	1	Not_GOST-art	0	Not_GOST-art	0
1.25	3.75	1	Not_GOST-art	0	Not_GOST-art	0
1.25	3.75	1	Not_GOST-art	0	Not_GOST-art	0
0.27	0.45	1	Not_GOST-art	0	Not_GOST-art	0
0.27	0.45	1	Not_GOST-art	0	Not_GOST-art	0
1	0.33	1	Not_GOST-art	0	Not_GOST-art	0
1	0.33	1	Not_GOST-art	0	Not_GOST-art	0
0.58	0.33	1	Not_GOST-art	0	Not_GOST-art	0
0.67	1.33	1	Not_GOST-art	0	Not_GOST-art	0
0.57	1.21	1	Not_GOST-art	0	Not_GOST-art	0
0.29	0	1	Not_GOST-art	0	Not_GOST-art	0
0.38	0.25	1	Not_GOST-art	0	Not_GOST-art	0
2.5	2	1	Not_GOST-art	0	GOST-art-Y-al	1
0.4	0.6	1	Not_GOST-art	0	Not_GOST-art	0
1	1.14	1	Not_GOST-art	0	Not_GOST-art	0
0.6	1	1	Not_GOST-art	0	Not_GOST-art	0
0.6	1	1	Not_GOST-art	0	Not_GOST-art	0
1	0	1	Not_GOST-art	0	Not_GOST-art	0
1	0	1	Not_GOST-art	0	Not_GOST-art	0
1	0.5	1	Not_GOST-art	0	Not_GOST-art	0
0.5	0	1	Not_GOST-art	0	Not_GOST-art	0
0.5	0	1	Not_GOST-art	0	Not_GOST-art	0
1.25	1.13	1	GOST-art-all	1	Not_GOST-art	0
1	1	1	Not_GOST-art	0	Not_GOST-art	0
0.29	1.14	1	Not_GOST-art	0	Not_GOST-art	0
1	4.5	1	Not_GOST-art	0	Not_GOST-art	0
1	4.5	1	Not_GOST-art	0	Not_GOST-art	0
0.33	0.5	1	Not_GOST-art	0	Not_GOST-art	0
0.4	1.2	1	Not_GOST-art	0	Not_GOST-art	0
0.5	0.5	1	Not_GOST-art	0	Not_GOST-art	0

0.86	0.86	1	Not_GOST-art	0	Not_GOST-art	0
0.63	0.63	1	Not_GOST-art	0	Not_GOST-art	0
1.6	1.33	1	GOST-art-all	1	GOST-art-Y-al	1
1.6	1.33	1	GOST-art-all	1	GOST-art-Y-al	1
1	3.67	1	Not_GOST-art	0	Not_GOST-art	0
0.33	0.89	1	Not_GOST-art	0	Not_GOST-art	0
2.33	5.67	1	Not_GOST-art	0	GOST-art-Y-al	1
2.33	5.67	1	Not_GOST-art	0	GOST-art-Y-al	1
2.33	5.67	1	Not_GOST-art	0	GOST-art-Y-al	1
2.33	5.67	1	Not_GOST-art	0	GOST-art-Y-al	1
0.75	0	1	Not_GOST-art	0	Not_GOST-art	0
0.75	0	1	Not_GOST-art	0	Not_GOST-art	0
0.33	1	1	Not_GOST-art	0	Not_GOST-art	0
0.67	0.33	1	Not_GOST-art	0	Not_GOST-art	0
0.22	0.22	1	Not_GOST-art	0	Not_GOST-art	0
1	2.75	1	Not_GOST-art	0	Not_GOST-art	0
1	2.75	1	Not_GOST-art	0	Not_GOST-art	0
1.83	3	1	GOST-art-all	1	GOST-art-Y-al	1
1.83	3	1	GOST-art-all	1	GOST-art-Y-al	1
1	7	1	Not_GOST-art	0	Not_GOST-art	0
1	7	1	Not_GOST-art	0	Not_GOST-art	0
0.67	0.67	1	Not_GOST-art	0	Not_GOST-art	0
0.67	0.67	1	Not_GOST-art	0	Not_GOST-art	0
0.44	2.44	1	Not_GOST-art	0	Not_GOST-art	0
0.67	1.33	1	Not_GOST-art	0	Not_GOST-art	0
0.67	1.33	1	Not_GOST-art	0	Not_GOST-art	0
0.75	1	1	Not_GOST-art	0	Not_GOST-art	0
0.75	1	1	Not_GOST-art	0	Not_GOST-art	0
0.5	0.5	1	Not_GOST-art	0	Not_GOST-art	0
0.54	0.31	1	Not_GOST-art	0	Not_GOST-art	0
0.4	0	1	Not_GOST-art	0	Not_GOST-art	0
0.67	4	1	Not_GOST-art	0	Not_GOST-art	0
0.67	4	1	Not_GOST-art	0	Not_GOST-art	0
0.6	0.8	1	Not_GOST-art	0	Not_GOST-art	0
0.6	0.8	1	Not_GOST-art	0	Not_GOST-art	0
0.56	1.11	1	Not_GOST-art	0	Not_GOST-art	0
0.75	10.25	1	Not_GOST-art	0	Not_GOST-art	0
0.75	10.25	1	Not_GOST-art	0	Not_GOST-art	0
1.25	0.5	1	Not_GOST-art	0	Not_GOST-art	0
1.25	0.5	1	Not_GOST-art	0	Not_GOST-art	0
1.25	0.5	1	Not_GOST-art	0	Not_GOST-art	0
0.25	0	1	Not_GOST-art	0	Not_GOST-art	0
0.44	0	1	Not_GOST-art	0	Not_GOST-art	0
1.43	3.14	1	Not_GOST-art	0	Not_GOST-art	0
1.43	3.14	1	Not_GOST-art	0	Not_GOST-art	0
0.33	0.33	1	Not_GOST-art	0	Not_GOST-art	0
0.43	0.14	1	Not_GOST-art	0	Not_GOST-art	0

0.43	0.14	1	Not_GOST-art	0	Not_GOST-art	0
0.43	1	1	Not_GOST-art	0	Not_GOST-art	0
1.92	4	1	GOST-art-all	1	GOST-art-Y-al	1
1.92	4	1	GOST-art-all	1	GOST-art-Y-al	1
0.15	0.23	1	Not_GOST-art	0	Not_GOST-art	0
0.75	0.75	1	Not_GOST-art	0	Not_GOST-art	0
0.75	0.25	1	Not_GOST-art	0	Not_GOST-art	0
0.67	2	1	Not_GOST-art	0	Not_GOST-art	0
1.83	2.67	1	Not_GOST-art	0	GOST-art-Y-al	1
1.83	2.67	1	Not_GOST-art	0	GOST-art-Y-al	1
0.35	0.24	1	Not_GOST-art	0	Not_GOST-art	0
0.35	0.24	1	Not_GOST-art	0	Not_GOST-art	0
0.33	0.11	1	Not_GOST-art	0	Not_GOST-art	0
1	2.33	1	Not_GOST-art	0	Not_GOST-art	0
0.5	0.5	1	Not_GOST-art	0	Not_GOST-art	0
0.43	2.14	1	Not_GOST-art	0	Not_GOST-art	0
1.29	4.43	1	Not_GOST-art	0	Not_GOST-art	0
1.29	4.43	1	Not_GOST-art	0	Not_GOST-art	0
0.86	3.29	1	Not_GOST-art	0	Not_GOST-art	0
1.33	7.67	1	Not_GOST-art	0	Not_GOST-art	0
1.5	11	1	Not_GOST-art	0	Not_GOST-art	0
1.5	11	1	Not_GOST-art	0	Not_GOST-art	0
0.38	1.13	1	Not_GOST-art	0	Not_GOST-art	0
0.33	0.22	1	Not_GOST-art	0	Not_GOST-art	0
1	0.5	1	Not_GOST-art	0	Not_GOST-art	0
1	1.8	1	Not_GOST-art	0	Not_GOST-art	0
1	1.8	1	Not_GOST-art	0	Not_GOST-art	0
1	1	1	Not_GOST-art	0	Not_GOST-art	0
0.67	0.67	1	Not_GOST-art	0	Not_GOST-art	0
0.67	0.67	1	Not_GOST-art	0	Not_GOST-art	0
1.69	7.92	1	GOST-art-all	1	GOST-art-Y-al	1
1.69	7.92	1	GOST-art-all	1	GOST-art-Y-al	1
0.4	0.2	1	Not_GOST-art	0	Not_GOST-art	0
0.9	4.2	1	Not_GOST-art	0	Not_GOST-art	0
0.9	4.2	1	Not_GOST-art	0	Not_GOST-art	0
0.9	4.2	1	Not_GOST-art	0	Not_GOST-art	0
0.9	4.2	1	Not_GOST-art	0	Not_GOST-art	0
1.4	1.8	1	Not_GOST-art	0	Not_GOST-art	0
1.4	1.8	1	Not_GOST-art	0	Not_GOST-art	0
0.33	2.17	1	Not_GOST-art	0	Not_GOST-art	0
1.5	1.5	1	Not_GOST-art	0	Not_GOST-art	0
1.33	4	1	Not_GOST-art	0	Not_GOST-art	0
2.67	4.11	1	GOST-art-all	1	GOST-art-Y-al	1
2.67	4.11	1	GOST-art-all	1	GOST-art-Y-al	1
1	0	1	Not_GOST-art	0	Not_GOST-art	0
1	0	1	Not_GOST-art	0	Not_GOST-art	0
1	0	1	Not_GOST-art	0	Not_GOST-art	0

0.4	1.8	1	Not_GOST-art	0	Not_GOST-art	0
0.5	0.5	1	Not_GOST-art	0	Not_GOST-art	0
1.5	3.5	1	Not_GOST-art	0	Not_GOST-art	0
1.5	3.5	1	Not_GOST-art	0	Not_GOST-art	0
0.43	0	1	Not_GOST-art	0	Not_GOST-art	0
1.5	2.5	1	Not_GOST-art	0	Not_GOST-art	0
1.5	2.5	1	Not_GOST-art	0	Not_GOST-art	0
0.75	4	1	Not_GOST-art	0	Not_GOST-art	0
1.5	9	1	Not_GOST-art	0	Not_GOST-art	0
0.67	0.33	1	Not_GOST-art	0	Not_GOST-art	0
1.67	2.67	1	Not_GOST-art	0	GOST-art-Y-al	1
1.67	2.67	1	Not_GOST-art	0	GOST-art-Y-al	1
1.67	2.67	1	Not_GOST-art	0	GOST-art-Y-al	1
1	1.44	1	Not_GOST-art	0	Not_GOST-art	0
1	1.44	1	Not_GOST-art	0	Not_GOST-art	0
0.75	1	1	Not_GOST-art	0	Not_GOST-art	0
0.55	0.18	1	Not_GOST-art	0	Not_GOST-art	0
0.55	0.18	1	Not_GOST-art	0	Not_GOST-art	0
0.5	0	1	Not_GOST-art	0	Not_GOST-art	0
1	1.67	1	Not_GOST-art	0	Not_GOST-art	0
1	1.67	1	Not_GOST-art	0	Not_GOST-art	0
2.67	9.33	1	Not_GOST-art	0	GOST-art-Y-al	1
2.67	9.33	1	Not_GOST-art	0	GOST-art-Y-al	1
1.07	4.13	1	GOST-art-all	1	Not_GOST-art	0
0.5	0.25	1	Not_GOST-art	0	Not_GOST-art	0
0.4	1	1	Not_GOST-art	0	Not_GOST-art	0
1	2	1	Not_GOST-art	0	Not_GOST-art	0
1	0.57	1	Not_GOST-art	0	Not_GOST-art	0
0.67	0.67	1	Not_GOST-art	0	Not_GOST-art	0
2.5	3.58	1	GOST-art-all	1	GOST-art-Y-al	1
2.5	3.58	1	GOST-art-all	1	GOST-art-Y-al	1
0.67	2	1	Not_GOST-art	0	Not_GOST-art	0
0.67	2	1	Not_GOST-art	0	Not_GOST-art	0
0.73	2.73	1	Not_GOST-art	0	Not_GOST-art	0
0.73	2.73	1	Not_GOST-art	0	Not_GOST-art	0
1.6	2.4	1	Not_GOST-art	0	GOST-art-Y-al	1
0.71	0.43	1	Not_GOST-art	0	Not_GOST-art	0
1.8	2.8	1	Not_GOST-art	0	GOST-art-Y-al	1
1.5	7	1	Not_GOST-art	0	Not_GOST-art	0
1.5	7	1	Not_GOST-art	0	Not_GOST-art	0
1.5	1	1	Not_GOST-art	0	Not_GOST-art	0
1	2	1	Not_GOST-art	0	Not_GOST-art	0
1.33	2.33	1	Not_GOST-art	0	Not_GOST-art	0
1.33	2.33	1	Not_GOST-art	0	Not_GOST-art	0
0.22	0.78	1	Not_GOST-art	0	Not_GOST-art	0
1	2	1	Not_GOST-art	0	Not_GOST-art	0
1	0.5	1	Not_GOST-art	0	Not_GOST-art	0

2.39	3.33	1	GOST-art-all	1	GOST-art-Y-al	1
0.46	0.38	1	Not_GOST-art	0	Not_GOST-art	0
0.46	0.38	1	Not_GOST-art	0	Not_GOST-art	0
0.5	2	1	Not_GOST-art	0	Not_GOST-art	0
1.67	3	1	Not_GOST-art	0	GOST-art-Y-al	1
1.67	3	1	Not_GOST-art	0	GOST-art-Y-al	1
1.67	3	1	Not_GOST-art	0	GOST-art-Y-al	1
1.83	4.5	1	Not_GOST-art	0	GOST-art-Y-al	1
1.83	4.5	1	Not_GOST-art	0	GOST-art-Y-al	1
1	4	1	Not_GOST-art	0	Not_GOST-art	0
1	4	1	Not_GOST-art	0	Not_GOST-art	0
0.8	1.1	1	Not_GOST-art	0	Not_GOST-art	0
0.8	1.1	1	Not_GOST-art	0	Not_GOST-art	0
0.82	2.09	1	Not_GOST-art	0	Not_GOST-art	0
1.17	4.89	1	GOST-art-all	1	Not_GOST-art	0
1	3.6	1	Not_GOST-art	0	Not_GOST-art	0
1	3.6	1	Not_GOST-art	0	Not_GOST-art	0
0.67	0.33	1	Not_GOST-art	0	Not_GOST-art	0
0.67	0.33	1	Not_GOST-art	0	Not_GOST-art	0
0.75	2.5	1	Not_GOST-art	0	Not_GOST-art	0
0.6	0.1	1	Not_GOST-art	0	Not_GOST-art	0
0.6	0.1	1	Not_GOST-art	0	Not_GOST-art	0
0.4	0	1	Not_GOST-art	0	Not_GOST-art	0
0.67	0.67	1	Not_GOST-art	0	Not_GOST-art	0
0.44	1.89	1	Not_GOST-art	0	Not_GOST-art	0
0.6	0.4	1	Not_GOST-art	0	Not_GOST-art	0
0.6	0.4	1	Not_GOST-art	0	Not_GOST-art	0
1.33	2.33	1	Not_GOST-art	0	Not_GOST-art	0
1.33	2.33	1	Not_GOST-art	0	Not_GOST-art	0
0.63	2	1	Not_GOST-art	0	Not_GOST-art	0
0.63	2	1	Not_GOST-art	0	Not_GOST-art	0
0.67	2	1	Not_GOST-art	0	Not_GOST-art	0
0.67	2	1	Not_GOST-art	0	Not_GOST-art	0
0.5	0	1	Not_GOST-art	0	Not_GOST-art	0
1	2.5	1	Not_GOST-art	0	Not_GOST-art	0
1	2.5	1	Not_GOST-art	0	Not_GOST-art	0
1	2.5	1	Not_GOST-art	0	Not_GOST-art	0
1	1.75	1	Not_GOST-art	0	Not_GOST-art	0
0.3	0.3	1	Not_GOST-art	0	Not_GOST-art	0
1	1.13	1	Not_GOST-art	0	Not_GOST-art	0
1	1.13	1	Not_GOST-art	0	Not_GOST-art	0
0.33	0	1	Not_GOST-art	0	Not_GOST-art	0
1	0.4	1	Not_GOST-art	0	Not_GOST-art	0
1	0.4	1	Not_GOST-art	0	Not_GOST-art	0
0.5	0	1	Not_GOST-art	0	Not_GOST-art	0
0.5	0.33	1	Not_GOST-art	0	Not_GOST-art	0
0.67	0.33	1	Not_GOST-art	0	Not_GOST-art	0

0.31	0.23	1	Not_GOST-art	0	Not_GOST-art	0
1	0	1	Not_GOST-art	0	Not_GOST-art	0
0.67	0.67	1	Not_GOST-art	0	Not_GOST-art	0
0.75	0.75	1	Not_GOST-art	0	Not_GOST-art	0
0.5	1.25	1	Not_GOST-art	0	Not_GOST-art	0
1	7.33	1	Not_GOST-art	0	Not_GOST-art	0
1.5	10	1	Not_GOST-art	0	Not_GOST-art	0
1.5	10	1	Not_GOST-art	0	Not_GOST-art	0
0.5	0.17	1	Not_GOST-art	0	Not_GOST-art	0
0.5	0.5	1	Not_GOST-art	0	Not_GOST-art	0
0.67	1	1	Not_GOST-art	0	Not_GOST-art	0
0.67	1	1	Not_GOST-art	0	Not_GOST-art	0
0.33	0.11	1	Not_GOST-art	0	Not_GOST-art	0
0.33	0.11	1	Not_GOST-art	0	Not_GOST-art	0
0.33	0.44	1	Not_GOST-art	0	Not_GOST-art	0
0.21	0	1	Not_GOST-art	0	Not_GOST-art	0
0.27	0.27	1	Not_GOST-art	0	Not_GOST-art	0
0.93	0.71	1	GOST-art-all	1	Not_GOST-art	0
0.93	0.71	1	GOST-art-all	1	Not_GOST-art	0
3.2	4.4	1	GOST-art-all	1	GOST-art-Y-al	1
3.2	4.4	1	GOST-art-all	1	GOST-art-Y-al	1
3.2	4.4	1	GOST-art-all	1	GOST-art-Y-al	1
3.2	4.4	1	GOST-art-all	1	GOST-art-Y-al	1
3.2	4.4	1	GOST-art-all	1	GOST-art-Y-al	1
0.5	0.25	1	Not_GOST-art	0	Not_GOST-art	0
0.88	1	1	Not_GOST-art	0	Not_GOST-art	0
4.25	5.5	1	GOST-art-all	1	GOST-art-Y-al	1
4.25	5.5	1	GOST-art-all	1	GOST-art-Y-al	1
4.25	5.5	1	GOST-art-all	1	GOST-art-Y-al	1
0.5	0.1	1	Not_GOST-art	0	Not_GOST-art	0
0.25	0.08	1	Not_GOST-art	0	Not_GOST-art	0
0.33	1.22	1	Not_GOST-art	0	Not_GOST-art	0
0.57	0.43	1	Not_GOST-art	0	Not_GOST-art	0
0.43	1.57	1	Not_GOST-art	0	Not_GOST-art	0
1.09	1	1	Not_GOST-art	0	Not_GOST-art	0
0.22	2.78	1	Not_GOST-art	0	Not_GOST-art	0
2	12	1	Not_GOST-art	0	GOST-art-Y-al	1
2	12	1	Not_GOST-art	0	GOST-art-Y-al	1
0.5	0.5	1	Not_GOST-art	0	Not_GOST-art	0
0.75	3.75	1	Not_GOST-art	0	Not_GOST-art	0
0.75	3.75	1	Not_GOST-art	0	Not_GOST-art	0
1.25	2.5	1	Not_GOST-art	0	Not_GOST-art	0
1.25	2.5	1	Not_GOST-art	0	Not_GOST-art	0
0.75	2.75	1	Not_GOST-art	0	Not_GOST-art	0
0.75	2.75	1	Not_GOST-art	0	Not_GOST-art	0
0.5	0	1	Not_GOST-art	0	Not_GOST-art	0
0.5	0	1	Not_GOST-art	0	Not_GOST-art	0

0.4	1.4	1	Not_GOST-art	0	Not_GOST-art	0
0.6	1.8	1	Not_GOST-art	0	Not_GOST-art	0
0.63	0.63	1	Not_GOST-art	0	Not_GOST-art	0
0.71	2	1	Not_GOST-art	0	Not_GOST-art	0
1	2	1	Not_GOST-art	0	Not_GOST-art	0
0.67	0.67	1	Not_GOST-art	0	Not_GOST-art	0
0.33	0.17	1	Not_GOST-art	0	Not_GOST-art	0
1	2.5	1	Not_GOST-art	0	Not_GOST-art	0
0.33	0.11	1	Not_GOST-art	0	Not_GOST-art	0
0.5	0.72	1	Not_GOST-art	0	Not_GOST-art	0
1.75	5.5	1	Not_GOST-art	0	GOST-art-Y-al	1
1.14	0.64	1	GOST-art-all	1	Not_GOST-art	0
1.14	0.64	1	GOST-art-all	1	Not_GOST-art	0
0.67	0.67	1	Not_GOST-art	0	Not_GOST-art	0
0.33	0.33	1	Not_GOST-art	0	Not_GOST-art	0
1	0	1	Not_GOST-art	0	Not_GOST-art	0
0.83	1.33	1	Not_GOST-art	0	Not_GOST-art	0
0.75	0.75	1	Not_GOST-art	0	Not_GOST-art	0
0.75	0.75	1	Not_GOST-art	0	Not_GOST-art	0
0.24	0.12	1	Not_GOST-art	0	Not_GOST-art	0
1.33	2.33	1	Not_GOST-art	0	Not_GOST-art	0
1.33	2.33	1	Not_GOST-art	0	Not_GOST-art	0
1.33	2.33	1	Not_GOST-art	0	Not_GOST-art	0
1.33	2.33	1	Not_GOST-art	0	Not_GOST-art	0
1.78	5.33	1	GOST-art-all	1	GOST-art-Y-al	1
0.44	0.22	1	Not_GOST-art	0	Not_GOST-art	0
0.44	0.22	1	Not_GOST-art	0	Not_GOST-art	0
1	0.33	1	Not_GOST-art	0	Not_GOST-art	0
0.67	0.67	1	Not_GOST-art	0	Not_GOST-art	0
1	0	1	Not_GOST-art	0	Not_GOST-art	0
1	0	1	Not_GOST-art	0	Not_GOST-art	0
0.67	1.33	1	Not_GOST-art	0	Not_GOST-art	0
1.2	3	1	Not_GOST-art	0	Not_GOST-art	0
1.2	3	1	Not_GOST-art	0	Not_GOST-art	0
2	4	1	Not_GOST-art	0	GOST-art-Y-al	1
0.38	0.25	1	Not_GOST-art	0	Not_GOST-art	0
1	0	1	Not_GOST-art	0	Not_GOST-art	0
0.22	0	1	Not_GOST-art	0	Not_GOST-art	0
0.43	0.29	1	Not_GOST-art	0	Not_GOST-art	0
1	0.67	1	Not_GOST-art	0	Not_GOST-art	0
1	0.67	1	Not_GOST-art	0	Not_GOST-art	0
1	0.5	1	Not_GOST-art	0	Not_GOST-art	0
1	0.5	1	Not_GOST-art	0	Not_GOST-art	0
2.4	4.2	1	Not_GOST-art	0	GOST-art-Y-al	1
2.4	4.2	1	Not_GOST-art	0	GOST-art-Y-al	1
0.44	0.56	1	Not_GOST-art	0	Not_GOST-art	0
1	0	1	Not_GOST-art	0	Not_GOST-art	0

0.4	0.2	1	Not_GOST-art	0	Not_GOST-art	0
0.22	0.78	1	Not_GOST-art	0	Not_GOST-art	0
0.75	0.5	1	Not_GOST-art	0	Not_GOST-art	0
0.86	2.29	1	Not_GOST-art	0	Not_GOST-art	0
0.86	2.29	1	Not_GOST-art	0	Not_GOST-art	0
1.15	8.69	1	GOST-art-all	1	Not_GOST-art	0
1	0.5	1	Not_GOST-art	0	Not_GOST-art	0
0.67	0.67	1	Not_GOST-art	0	Not_GOST-art	0
1	0.33	1	Not_GOST-art	0	Not_GOST-art	0
0.5	0.67	1	Not_GOST-art	0	Not_GOST-art	0
1.5	2	1	Not_GOST-art	0	Not_GOST-art	0
0.9	1.5	1	Not_GOST-art	0	Not_GOST-art	0
0.67	0	1	Not_GOST-art	0	Not_GOST-art	0
0.67	0	1	Not_GOST-art	0	Not_GOST-art	0
0.4	0.2	1	Not_GOST-art	0	Not_GOST-art	0
1	4	1	Not_GOST-art	0	Not_GOST-art	0
0.33	0.67	1	Not_GOST-art	0	Not_GOST-art	0
0.36	0.36	1	Not_GOST-art	0	Not_GOST-art	0
0.75	0.25	1	Not_GOST-art	0	Not_GOST-art	0
1	1.88	1	Not_GOST-art	0	Not_GOST-art	0
1.38	2.06	1	GOST-art-all	1	Not_GOST-art	0
1.38	2.06	1	GOST-art-all	1	Not_GOST-art	0
0.67	0	1	Not_GOST-art	0	Not_GOST-art	0
0.67	0	1	Not_GOST-art	0	Not_GOST-art	0
0.62	0.62	1	Not_GOST-art	0	Not_GOST-art	0
1.5	3	1	Not_GOST-art	0	Not_GOST-art	0
1.5	3	1	Not_GOST-art	0	Not_GOST-art	0
0.67	1.33	1	Not_GOST-art	0	Not_GOST-art	0
0.6	0.2	1	Not_GOST-art	0	Not_GOST-art	0
0.44	1.11	1	Not_GOST-art	0	Not_GOST-art	0
0.44	1.11	1	Not_GOST-art	0	Not_GOST-art	0
0.88	0.88	1	GOST-art-all	1	Not_GOST-art	0
0.88	0.88	1	GOST-art-all	1	Not_GOST-art	0
1.5	1.17	1	Not_GOST-art	0	Not_GOST-art	0
0.67	1.5	1	Not_GOST-art	0	Not_GOST-art	0
0.67	0.67	1	Not_GOST-art	0	Not_GOST-art	0
0.54	1.08	1	Not_GOST-art	0	Not_GOST-art	0
0.5	3.75	1	Not_GOST-art	0	Not_GOST-art	0
3.5	11	1	Not_GOST-art	0	GOST-art-Y-al	1
3.5	11	1	Not_GOST-art	0	GOST-art-Y-al	1
1.06	3.29	1	GOST-art-all	1	Not_GOST-art	0
0.67	0	1	Not_GOST-art	0	Not_GOST-art	0
0.63	0.38	1	Not_GOST-art	0	Not_GOST-art	0
0.67	0	1	Not_GOST-art	0	Not_GOST-art	0
1.25	1.5	1	Not_GOST-art	0	Not_GOST-art	0
1.25	1.5	1	Not_GOST-art	0	Not_GOST-art	0
0.86	2.14	1	Not_GOST-art	0	Not_GOST-art	0

0.86	2.14	1	Not_GOST-art	0	Not_GOST-art	0
1.13	0.38	1	Not_GOST-art	0	Not_GOST-art	0
0.29	0.14	1	Not_GOST-art	0	Not_GOST-art	0
1	1.67	1	Not_GOST-art	0	Not_GOST-art	0
1	1.67	1	Not_GOST-art	0	Not_GOST-art	0
0.4	1	1	Not_GOST-art	0	Not_GOST-art	0
0.33	0.5	1	Not_GOST-art	0	Not_GOST-art	0
0.5	0	1	Not_GOST-art	0	Not_GOST-art	0
2.86	4.14	1	GOST-art-all	1	GOST-art-Y-al	1
2.86	4.14	1	GOST-art-all	1	GOST-art-Y-al	1
2.86	4.14	1	GOST-art-all	1	GOST-art-Y-al	1
1	0	1	Not_GOST-art	0	Not_GOST-art	0
1	0	1	Not_GOST-art	0	Not_GOST-art	0
1.33	1.67	1	Not_GOST-art	0	Not_GOST-art	0
1.33	1.67	1	Not_GOST-art	0	Not_GOST-art	0
0.75	1.75	1	Not_GOST-art	0	Not_GOST-art	0
1	0.5	1	Not_GOST-art	0	Not_GOST-art	0
0.67	0.67	1	Not_GOST-art	0	Not_GOST-art	0
0.4	1.4	1	Not_GOST-art	0	Not_GOST-art	0
0.4	1.4	1	Not_GOST-art	0	Not_GOST-art	0
1	0.5	1	Not_GOST-art	0	Not_GOST-art	0
0.67	3.67	1	Not_GOST-art	0	Not_GOST-art	0
0.8	2	1	Not_GOST-art	0	Not_GOST-art	0
0.75	0.25	1	Not_GOST-art	0	Not_GOST-art	0
0.75	0.25	1	Not_GOST-art	0	Not_GOST-art	0
1.33	0	1	Not_GOST-art	0	Not_GOST-art	0
1.33	0	1	Not_GOST-art	0	Not_GOST-art	0
1.33	0	1	Not_GOST-art	0	Not_GOST-art	0
1.33	0	1	Not_GOST-art	0	Not_GOST-art	0
0.6	1.4	1	Not_GOST-art	0	Not_GOST-art	0
0.6	1.4	1	Not_GOST-art	0	Not_GOST-art	0
0.4	0.8	1	Not_GOST-art	0	Not_GOST-art	0
0.67	0.33	1	Not_GOST-art	0	Not_GOST-art	0
0.43	0.29	1	Not_GOST-art	0	Not_GOST-art	0
0.4	0.2	1	Not_GOST-art	0	Not_GOST-art	0
1	1.33	1	Not_GOST-art	0	Not_GOST-art	0
1	1.33	1	Not_GOST-art	0	Not_GOST-art	0
0.5	0.25	1	Not_GOST-art	0	Not_GOST-art	0
0.36	0.91	1	Not_GOST-art	0	Not_GOST-art	0
0.5	0.2	1	Not_GOST-art	0	Not_GOST-art	0
0.3	0.8	1	Not_GOST-art	0	Not_GOST-art	0
1.2	0.6	1	Not_GOST-art	0	Not_GOST-art	0
1.2	0.6	1	Not_GOST-art	0	Not_GOST-art	0
2.2	35.2	1	Not_GOST-art	0	GOST-art-Y-al	1
2.2	35.2	1	Not_GOST-art	0	GOST-art-Y-al	1
2.2	35.2	1	Not_GOST-art	0	GOST-art-Y-al	1
1.33	9.67	1	Not_GOST-art	0	Not_GOST-art	0

1.33	9.67	1	Not_GOST-art	0	Not_GOST-art	0
0.88	2.38	1	Not_GOST-art	0	Not_GOST-art	0
0.88	2.38	1	Not_GOST-art	0	Not_GOST-art	0
0.43	0.71	1	Not_GOST-art	0	Not_GOST-art	0
0.62	0.31	1	Not_GOST-art	0	Not_GOST-art	0
0.62	0.31	1	Not_GOST-art	0	Not_GOST-art	0
0.62	0.31	1	Not_GOST-art	0	Not_GOST-art	0
1	2.5	1	Not_GOST-art	0	Not_GOST-art	0
1	2.5	1	Not_GOST-art	0	Not_GOST-art	0
0.67	5.33	1	Not_GOST-art	0	Not_GOST-art	0
0.67	0	1	Not_GOST-art	0	Not_GOST-art	0
2.4	4.8	1	Not_GOST-art	0	GOST-art-Y-al	1
2.4	4.8	1	Not_GOST-art	0	GOST-art-Y-al	1
2.4	4.8	1	Not_GOST-art	0	GOST-art-Y-al	1
1	2	1	Not_GOST-art	0	Not_GOST-art	0
0.75	0.5	1	Not_GOST-art	0	Not_GOST-art	0
0.75	0.5	1	Not_GOST-art	0	Not_GOST-art	0
0.75	0.5	1	Not_GOST-art	0	Not_GOST-art	0
1.5	1.5	1	Not_GOST-art	0	Not_GOST-art	0
1.33	1.33	1	Not_GOST-art	0	Not_GOST-art	0
1.33	1.33	1	Not_GOST-art	0	Not_GOST-art	0
1.33	1.33	1	Not_GOST-art	0	Not_GOST-art	0
0.75	0.25	1	Not_GOST-art	0	Not_GOST-art	0
0.75	0.25	1	Not_GOST-art	0	Not_GOST-art	0
1.33	12.33	1	Not_GOST-art	0	Not_GOST-art	0
1.33	12.33	1	Not_GOST-art	0	Not_GOST-art	0
1.33	12.33	1	Not_GOST-art	0	Not_GOST-art	0
1.33	12.33	1	Not_GOST-art	0	Not_GOST-art	0
1	2.29	1	Not_GOST-art	0	Not_GOST-art	0
0.44	0.33	1	Not_GOST-art	0	Not_GOST-art	0
1	0.83	1	Not_GOST-art	0	Not_GOST-art	0
2.71	3.14	1	GOST-art-all	1	GOST-art-Y-al	1
2.71	3.14	1	GOST-art-all	1	GOST-art-Y-al	1
2.71	3.14	1	GOST-art-all	1	GOST-art-Y-al	1
1.33	2.33	1	Not_GOST-art	0	Not_GOST-art	0
1.33	2.33	1	Not_GOST-art	0	Not_GOST-art	0
1.33	2.33	1	Not_GOST-art	0	Not_GOST-art	0
0.75	0	1	Not_GOST-art	0	Not_GOST-art	0
0.22	0.28	1	Not_GOST-art	0	Not_GOST-art	0
0.4	0.8	1	Not_GOST-art	0	Not_GOST-art	0
1	4	1	Not_GOST-art	0	Not_GOST-art	0
1.5	1	1	Not_GOST-art	0	Not_GOST-art	0
1.5	1	1	Not_GOST-art	0	Not_GOST-art	0
1.5	1	1	Not_GOST-art	0	Not_GOST-art	0
0.7	0.6	1	Not_GOST-art	0	Not_GOST-art	0
1.33	4	1	Not_GOST-art	0	Not_GOST-art	0
1.33	4	1	Not_GOST-art	0	Not_GOST-art	0

2.08	5.75	1	GOST-art-all	1	GOST-art-Y-al	1
0.75	0.25	1	Not_GOST-art	0	Not_GOST-art	0
0.75	0.25	1	Not_GOST-art	0	Not_GOST-art	0
1	0.5	1	Not_GOST-art	0	Not_GOST-art	0
1	7.33	1	Not_GOST-art	0	Not_GOST-art	0
1	7.33	1	Not_GOST-art	0	Not_GOST-art	0
0.5	3.75	1	Not_GOST-art	0	Not_GOST-art	0
0.86	1.71	1	Not_GOST-art	0	Not_GOST-art	0
0.86	1.71	1	Not_GOST-art	0	Not_GOST-art	0
1.46	3.38	1	GOST-art-all	1	Not_GOST-art	0
0.29	0.14	1	Not_GOST-art	0	Not_GOST-art	0
0.38	0.77	1	Not_GOST-art	0	Not_GOST-art	0
1	3	1	Not_GOST-art	0	Not_GOST-art	0
1	3	1	Not_GOST-art	0	Not_GOST-art	0
1	3	1	Not_GOST-art	0	Not_GOST-art	0
1.33	0.67	1	Not_GOST-art	0	Not_GOST-art	0
1.33	0.67	1	Not_GOST-art	0	Not_GOST-art	0
0.4	0	1	Not_GOST-art	0	Not_GOST-art	0
0.5	2.5	1	Not_GOST-art	0	Not_GOST-art	0
0.5	2.5	1	Not_GOST-art	0	Not_GOST-art	0
0.6	0.8	1	Not_GOST-art	0	Not_GOST-art	0
1.5	3.63	1	Not_GOST-art	0	Not_GOST-art	0
1.5	3.63	1	Not_GOST-art	0	Not_GOST-art	0
1.5	3.63	1	Not_GOST-art	0	Not_GOST-art	0
0.92	1.33	1	Not_GOST-art	0	Not_GOST-art	0
1.25	4	1	Not_GOST-art	0	Not_GOST-art	0
0.79	1.21	1	Not_GOST-art	0	Not_GOST-art	0
1.13	1.75	1	Not_GOST-art	0	Not_GOST-art	0
1.13	1.75	1	Not_GOST-art	0	Not_GOST-art	0
1.13	1.75	1	Not_GOST-art	0	Not_GOST-art	0
0.27	0.18	1	Not_GOST-art	0	Not_GOST-art	0
1	0	1	Not_GOST-art	0	Not_GOST-art	0
1	0	1	Not_GOST-art	0	Not_GOST-art	0
1	0	1	Not_GOST-art	0	Not_GOST-art	0
1	0	1	Not_GOST-art	0	Not_GOST-art	0
1	0	1	Not_GOST-art	0	Not_GOST-art	0
0.38	0.25	1	Not_GOST-art	0	Not_GOST-art	0
1.2	2.93	1	GOST-art-all	1	Not_GOST-art	0
1.2	2.93	1	GOST-art-all	1	Not_GOST-art	0
0.33	1.67	1	Not_GOST-art	0	Not_GOST-art	0
0.89	1.33	1	Not_GOST-art	0	Not_GOST-art	0
0.6	0.2	1	Not_GOST-art	0	Not_GOST-art	0
0.6	0.2	1	Not_GOST-art	0	Not_GOST-art	0
0.67	0.33	1	Not_GOST-art	0	Not_GOST-art	0
1	0.5	1	Not_GOST-art	0	Not_GOST-art	0
0.75	1.25	1	Not_GOST-art	0	Not_GOST-art	0
0.25	0	1	Not_GOST-art	0	Not_GOST-art	0

1.56	1.22	1	GOST-art-all	1	GOST-art-Y-al	1
0.5	0.83	1	Not_GOST-art	0	Not_GOST-art	0
0.5	0.36	1	Not_GOST-art	0	Not_GOST-art	0
0.33	0.17	1	Not_GOST-art	0	Not_GOST-art	0
0.23	0.38	1	Not_GOST-art	0	Not_GOST-art	0
1.17	2.17	1	Not_GOST-art	0	Not_GOST-art	0
0.67	0	1	Not_GOST-art	0	Not_GOST-art	0
3.07	10.8	1	GOST-art-all	1	GOST-art-Y-al	1
3.07	10.8	1	GOST-art-all	1	GOST-art-Y-al	1
1	5	1	Not_GOST-art	0	Not_GOST-art	0
1	5	1	Not_GOST-art	0	Not_GOST-art	0
1	5	1	Not_GOST-art	0	Not_GOST-art	0
0.5	0.67	1	Not_GOST-art	0	Not_GOST-art	0
1.46	1.85	1	GOST-art-all	1	Not_GOST-art	0
1	1	1	Not_GOST-art	0	Not_GOST-art	0
2.75	12.19	1	GOST-art-all	1	GOST-art-Y-al	1
1	2	1	Not_GOST-art	0	Not_GOST-art	0
1	2	1	Not_GOST-art	0	Not_GOST-art	0
0.71	2.86	1	Not_GOST-art	0	Not_GOST-art	0
0.71	2.86	1	Not_GOST-art	0	Not_GOST-art	0
0.33	0.33	1	Not_GOST-art	0	Not_GOST-art	0
1	0.5	1	Not_GOST-art	0	Not_GOST-art	0
4	39.33	1	Not_GOST-art	0	GOST-art-Y-al	1
4	39.33	1	Not_GOST-art	0	GOST-art-Y-al	1
4	39.33	1	Not_GOST-art	0	GOST-art-Y-al	1
0.91	2.55	1	Not_GOST-art	0	Not_GOST-art	0
1	7	1	Not_GOST-art	0	Not_GOST-art	0
0.67	1	1	Not_GOST-art	0	Not_GOST-art	0
0.67	9.33	1	Not_GOST-art	0	Not_GOST-art	0
0.67	9.33	1	Not_GOST-art	0	Not_GOST-art	0
0.67	3.67	1	Not_GOST-art	0	Not_GOST-art	0
0.5	5.5	1	Not_GOST-art	0	Not_GOST-art	0
1.4	6.4	1	Not_GOST-art	0	Not_GOST-art	0
1.4	6.4	1	Not_GOST-art	0	Not_GOST-art	0
0.33	1.17	1	Not_GOST-art	0	Not_GOST-art	0
1.4	0.8	1	Not_GOST-art	0	Not_GOST-art	0
0.67	0	1	Not_GOST-art	0	Not_GOST-art	0
0.6	1	1	Not_GOST-art	0	Not_GOST-art	0
0.6	1	1	Not_GOST-art	0	Not_GOST-art	0
2	2.5	1	Not_GOST-art	0	GOST-art-Y-al	1
2	2.5	1	Not_GOST-art	0	GOST-art-Y-al	1
0.33	1	1	Not_GOST-art	0	Not_GOST-art	0
1.5	3	1	Not_GOST-art	0	Not_GOST-art	0
0.71	0	1	Not_GOST-art	0	Not_GOST-art	0
0.25	2	1	Not_GOST-art	0	Not_GOST-art	0
1.14	4.07	1	GOST-art-all	1	Not_GOST-art	0
0.67	0	1	Not_GOST-art	0	Not_GOST-art	0

2.25	4.25	1	Not_GOST-art	0	GOST-art-Y-al	1
2.25	4.25	1	Not_GOST-art	0	GOST-art-Y-al	1
2.25	4.25	1	Not_GOST-art	0	GOST-art-Y-al	1
0.4	1.2	1	Not_GOST-art	0	Not_GOST-art	0
0.43	1.14	1	Not_GOST-art	0	Not_GOST-art	0
1.5	7.5	1	Not_GOST-art	0	Not_GOST-art	0
0.78	2.89	1	Not_GOST-art	0	Not_GOST-art	0
1	2.71	1	Not_GOST-art	0	Not_GOST-art	0
0.4	0.2	1	Not_GOST-art	0	Not_GOST-art	0
3	17.5	1	Not_GOST-art	0	GOST-art-Y-al	1
3	17.5	1	Not_GOST-art	0	GOST-art-Y-al	1
3	17.5	1	Not_GOST-art	0	GOST-art-Y-al	1
1	1.25	1	Not_GOST-art	0	Not_GOST-art	0
1	1.25	1	Not_GOST-art	0	Not_GOST-art	0
1	1.5	1	Not_GOST-art	0	Not_GOST-art	0
1	1.5	1	Not_GOST-art	0	Not_GOST-art	0
1	0.75	1	Not_GOST-art	0	Not_GOST-art	0
1	0.75	1	Not_GOST-art	0	Not_GOST-art	0
1	2.67	1	Not_GOST-art	0	Not_GOST-art	0
1	2.67	1	Not_GOST-art	0	Not_GOST-art	0
0.18	0	1	Not_GOST-art	0	Not_GOST-art	0
0.67	0	1	Not_GOST-art	0	Not_GOST-art	0
0.67	0	1	Not_GOST-art	0	Not_GOST-art	0
1.5	2	1	Not_GOST-art	0	Not_GOST-art	0
1.5	2	1	Not_GOST-art	0	Not_GOST-art	0
0.67	1	1	Not_GOST-art	0	Not_GOST-art	0
1	0	1	Not_GOST-art	0	Not_GOST-art	0
0.57	0.43	1	Not_GOST-art	0	Not_GOST-art	0
1	8.67	1	Not_GOST-art	0	Not_GOST-art	0
1	8.67	1	Not_GOST-art	0	Not_GOST-art	0
0.5	1.25	1	Not_GOST-art	0	Not_GOST-art	0
0.67	7.67	1	Not_GOST-art	0	Not_GOST-art	0
2.5	2.38	1	GOST-art-all	1	GOST-art-Y-al	1
2.5	2.38	1	GOST-art-all	1	GOST-art-Y-al	1
1	0	1	Not_GOST-art	0	Not_GOST-art	0
2	0.88	1	GOST-art-all	1	GOST-art-Y-al	1
2	0.88	1	GOST-art-all	1	GOST-art-Y-al	1
0.5	0.5	1	Not_GOST-art	0	Not_GOST-art	0
0.5	0.5	1	Not_GOST-art	0	Not_GOST-art	0
1.21	2.57	1	GOST-art-all	1	Not_GOST-art	0
1.21	2.57	1	GOST-art-all	1	Not_GOST-art	0
0.43	0.29	1	Not_GOST-art	0	Not_GOST-art	0
0.43	0.29	1	Not_GOST-art	0	Not_GOST-art	0
0.64	1.18	1	Not_GOST-art	0	Not_GOST-art	0
0.64	1.18	1	Not_GOST-art	0	Not_GOST-art	0
0.67	0.67	1	Not_GOST-art	0	Not_GOST-art	0
1.4	1.6	1	GOST-art-all	1	Not_GOST-art	0

1.4	1.6	1	GOST-art-all	1	Not_GOST-art	0
0.29	2.14	1	Not_GOST-art	0	Not_GOST-art	0
1	9.33	1	Not_GOST-art	0	Not_GOST-art	0
1	9.33	1	Not_GOST-art	0	Not_GOST-art	0
1	0	1	Not_GOST-art	0	Not_GOST-art	0
1	0	1	Not_GOST-art	0	Not_GOST-art	0
0.24	0.71	1	Not_GOST-art	0	Not_GOST-art	0
0.24	0.71	1	Not_GOST-art	0	Not_GOST-art	0
0.75	1	1	Not_GOST-art	0	Not_GOST-art	0
0.75	1	1	Not_GOST-art	0	Not_GOST-art	0
0.4	0	1	Not_GOST-art	0	Not_GOST-art	0
1.36	1.09	1	GOST-art-all	1	Not_GOST-art	0
1	0	1	Not_GOST-art	0	Not_GOST-art	0
1.25	4.5	1	Not_GOST-art	0	Not_GOST-art	0
1.25	4.5	1	Not_GOST-art	0	Not_GOST-art	0
1.57	3.43	1	Not_GOST-art	0	GOST-art-Y-al	1
1	15.67	1	Not_GOST-art	0	Not_GOST-art	0
1	15.67	1	Not_GOST-art	0	Not_GOST-art	0
0.75	1.5	1	Not_GOST-art	0	Not_GOST-art	0
0.83	4.33	1	Not_GOST-art	0	Not_GOST-art	0
0.67	0.33	1	Not_GOST-art	0	Not_GOST-art	0
1.67	2.11	1	GOST-art-all	1	GOST-art-Y-al	1
0.67	2.67	1	Not_GOST-art	0	Not_GOST-art	0
0.67	2.67	1	Not_GOST-art	0	Not_GOST-art	0
0.8	2.2	1	Not_GOST-art	0	Not_GOST-art	0
0.8	2.2	1	Not_GOST-art	0	Not_GOST-art	0
0.4	0.2	1	Not_GOST-art	0	Not_GOST-art	0
0.75	2.5	1	Not_GOST-art	0	Not_GOST-art	0
0.67	1.67	1	Not_GOST-art	0	Not_GOST-art	0
0.67	1.67	1	Not_GOST-art	0	Not_GOST-art	0
1.33	4.33	1	Not_GOST-art	0	Not_GOST-art	0
1.33	4.33	1	Not_GOST-art	0	Not_GOST-art	0
1.67	9	1	Not_GOST-art	0	GOST-art-Y-al	1
1	2	1	Not_GOST-art	0	Not_GOST-art	0
2	8.33	1	Not_GOST-art	0	GOST-art-Y-al	1
4	7.33	1	Not_GOST-art	0	GOST-art-Y-al	1
4	7.33	1	Not_GOST-art	0	GOST-art-Y-al	1
0.5	2.5	1	Not_GOST-art	0	Not_GOST-art	0
0.33	0.17	1	Not_GOST-art	0	Not_GOST-art	0
0.67	4.33	1	Not_GOST-art	0	Not_GOST-art	0
3	7.44	1	GOST-art-all	1	GOST-art-Y-al	1
3	7.44	1	GOST-art-all	1	GOST-art-Y-al	1
3	7.44	1	GOST-art-all	1	GOST-art-Y-al	1
3	7.44	1	GOST-art-all	1	GOST-art-Y-al	1
1	2.75	1	Not_GOST-art	0	Not_GOST-art	0
0.67	8.67	1	Not_GOST-art	0	Not_GOST-art	0
0.67	8.67	1	Not_GOST-art	0	Not_GOST-art	0

1	0	1	Not_GOST-art	0	Not_GOST-art	0
0.83	4.83	1	Not_GOST-art	0	Not_GOST-art	0
3.86	10	1	GOST-art-all	1	GOST-art-Y-al	1
0.75	2.25	1	Not_GOST-art	0	Not_GOST-art	0
0.75	2.25	1	Not_GOST-art	0	Not_GOST-art	0
2	2	1	Not_GOST-art	0	GOST-art-Y-al	1
2	2	1	Not_GOST-art	0	GOST-art-Y-al	1
1	0	1	Not_GOST-art	0	Not_GOST-art	0
1	0	1	Not_GOST-art	0	Not_GOST-art	0
0.4	0	1	Not_GOST-art	0	Not_GOST-art	0
1.33	0.33	1	Not_GOST-art	0	Not_GOST-art	0
1.33	0.33	1	Not_GOST-art	0	Not_GOST-art	0
1	5	1	Not_GOST-art	0	Not_GOST-art	0
1.13	1.25	1	Not_GOST-art	0	Not_GOST-art	0
1.13	1.25	1	Not_GOST-art	0	Not_GOST-art	0
1	3	1	Not_GOST-art	0	Not_GOST-art	0
1	3	1	Not_GOST-art	0	Not_GOST-art	0
1	5.5	1	Not_GOST-art	0	Not_GOST-art	0
1	6.14	1	Not_GOST-art	0	Not_GOST-art	0
1	6.14	1	Not_GOST-art	0	Not_GOST-art	0
1	2.5	1	Not_GOST-art	0	Not_GOST-art	0
0.4	0.8	1	Not_GOST-art	0	Not_GOST-art	0
0.29	0.29	1	Not_GOST-art	0	Not_GOST-art	0
0.44	0.89	1	Not_GOST-art	0	Not_GOST-art	0
2.22	7.89	1	GOST-art-all	1	GOST-art-Y-al	1
0.8	0.4	1	Not_GOST-art	0	Not_GOST-art	0
0.83	5.67	1	Not_GOST-art	0	Not_GOST-art	0
1.33	0	1	Not_GOST-art	0	Not_GOST-art	0
1.33	0	1	Not_GOST-art	0	Not_GOST-art	0
1.33	2.67	1	Not_GOST-art	0	Not_GOST-art	0
1.33	2.67	1	Not_GOST-art	0	Not_GOST-art	0
1.33	2.67	1	Not_GOST-art	0	Not_GOST-art	0
1.33	2.67	1	Not_GOST-art	0	Not_GOST-art	0
1	1.5	1	Not_GOST-art	0	Not_GOST-art	0
1	1.5	1	Not_GOST-art	0	Not_GOST-art	0
0.75	3.5	1	Not_GOST-art	0	Not_GOST-art	0
0.43	0.29	1	Not_GOST-art	0	Not_GOST-art	0
0.56	0.22	1	Not_GOST-art	0	Not_GOST-art	0
0.56	0.22	1	Not_GOST-art	0	Not_GOST-art	0
2.27	8.27	1	GOST-art-all	1	GOST-art-Y-al	1
0.67	0	1	Not_GOST-art	0	Not_GOST-art	0
3.4	6	1	GOST-art-all	1	GOST-art-Y-al	1
0.8	1.8	1	Not_GOST-art	0	Not_GOST-art	0
0.69	1.46	1	Not_GOST-art	0	Not_GOST-art	0
0.69	1.46	1	Not_GOST-art	0	Not_GOST-art	0
1	0.5	1	Not_GOST-art	0	Not_GOST-art	0
1	0.5	1	Not_GOST-art	0	Not_GOST-art	0

0.56	2.78	1	Not_GOST-art	0	Not_GOST-art	0
0.25	0	1	Not_GOST-art	0	Not_GOST-art	0
0.75	2.08	1	Not_GOST-art	0	Not_GOST-art	0
1.8	7.4	1	Not_GOST-art	0	GOST-art-Y-al	1
1.17	5.17	1	Not_GOST-art	0	Not_GOST-art	0
1.17	5.17	1	Not_GOST-art	0	Not_GOST-art	0
1	0	1	Not_GOST-art	0	Not_GOST-art	0
1.25	0.75	1	Not_GOST-art	0	Not_GOST-art	0
0.67	0.33	1	Not_GOST-art	0	Not_GOST-art	0
1	2	1	Not_GOST-art	0	Not_GOST-art	0
1.33	1.67	1	Not_GOST-art	0	Not_GOST-art	0
1.33	1.67	1	Not_GOST-art	0	Not_GOST-art	0
0.67	0.67	1	Not_GOST-art	0	Not_GOST-art	0
1.27	2.18	1	GOST-art-all	1	Not_GOST-art	0
0.75	5.25	1	Not_GOST-art	0	Not_GOST-art	0
0.75	1.25	1	Not_GOST-art	0	Not_GOST-art	0
0.75	1.25	1	Not_GOST-art	0	Not_GOST-art	0
0.67	0.33	1	Not_GOST-art	0	Not_GOST-art	0
0.67	0.33	1	Not_GOST-art	0	Not_GOST-art	0
1.17	2.67	1	Not_GOST-art	0	Not_GOST-art	0
1.17	2.67	1	Not_GOST-art	0	Not_GOST-art	0
1.33	1	1	Not_GOST-art	0	Not_GOST-art	0
1.33	1	1	Not_GOST-art	0	Not_GOST-art	0
1.57	4.29	1	Not_GOST-art	0	GOST-art-Y-al	1
0.29	0.29	1	Not_GOST-art	0	Not_GOST-art	0
0.6	1.6	1	Not_GOST-art	0	Not_GOST-art	0
1.92	6.77	1	GOST-art-all	1	GOST-art-Y-al	1
1.5	2.25	1	Not_GOST-art	0	Not_GOST-art	0
1.5	2.25	1	Not_GOST-art	0	Not_GOST-art	0
0.75	0.75	1	Not_GOST-art	0	Not_GOST-art	0
0.67	1	1	Not_GOST-art	0	Not_GOST-art	0
0.67	1	1	Not_GOST-art	0	Not_GOST-art	0
0.67	0.33	1	Not_GOST-art	0	Not_GOST-art	0
0.67	0.33	1	Not_GOST-art	0	Not_GOST-art	0
0.11	0.56	1	Not_GOST-art	0	Not_GOST-art	0
1	1.5	1	Not_GOST-art	0	Not_GOST-art	0
1	1.5	1	Not_GOST-art	0	Not_GOST-art	0
0.33	0.5	1	Not_GOST-art	0	Not_GOST-art	0
0.22	1.22	1	Not_GOST-art	0	Not_GOST-art	0
0.4	1.2	1	Not_GOST-art	0	Not_GOST-art	0
0.67	0.67	1	Not_GOST-art	0	Not_GOST-art	0
0.75	0.25	1	Not_GOST-art	0	Not_GOST-art	0
0.86	1	1	Not_GOST-art	0	Not_GOST-art	0
2.17	3	1	GOST-art-all	1	GOST-art-Y-al	1
0.56	1.78	1	Not_GOST-art	0	Not_GOST-art	0
0.56	1.78	1	Not_GOST-art	0	Not_GOST-art	0
0.5	3.5	1	Not_GOST-art	0	Not_GOST-art	0

0.4	0.4	1	Not_GOST-art	0	Not_GOST-art	0
0.46	0.31	1	Not_GOST-art	0	Not_GOST-art	0
1	1.8	1	Not_GOST-art	0	Not_GOST-art	0
1	1.8	1	Not_GOST-art	0	Not_GOST-art	0
0.33	0.33	1	Not_GOST-art	0	Not_GOST-art	0
1	0.67	1	Not_GOST-art	0	Not_GOST-art	0
1	0.67	1	Not_GOST-art	0	Not_GOST-art	0
1	0.67	1	Not_GOST-art	0	Not_GOST-art	0
0.89	1.33	1	Not_GOST-art	0	Not_GOST-art	0
0.89	1.33	1	Not_GOST-art	0	Not_GOST-art	0
1	0	1	Not_GOST-art	0	Not_GOST-art	0
0.4	0	1	Not_GOST-art	0	Not_GOST-art	0
0.17	0.08	1	Not_GOST-art	0	Not_GOST-art	0
0.6	0.5	1	Not_GOST-art	0	Not_GOST-art	0
0.6	0.5	1	Not_GOST-art	0	Not_GOST-art	0
0.6	0.5	1	Not_GOST-art	0	Not_GOST-art	0
0.42	0.42	1	Not_GOST-art	0	Not_GOST-art	0
2.08	3.38	1	GOST-art-all	1	GOST-art-Y-al	1
1	4	1	Not_GOST-art	0	Not_GOST-art	0
1	4	1	Not_GOST-art	0	Not_GOST-art	0
0.75	1.25	1	Not_GOST-art	0	Not_GOST-art	0
0.75	1.25	1	Not_GOST-art	0	Not_GOST-art	0
0.67	0	1	Not_GOST-art	0	Not_GOST-art	0
2	3	1	GOST-art-all	1	GOST-art-Y-al	1
2	3	1	GOST-art-all	1	GOST-art-Y-al	1
0.67	0.33	1	Not_GOST-art	0	Not_GOST-art	0
1.5	0.75	1	Not_GOST-art	0	Not_GOST-art	0
0.67	0.33	1	Not_GOST-art	0	Not_GOST-art	0
1.56	1.67	1	GOST-art-all	1	GOST-art-Y-al	1
1	0	1	Not_GOST-art	0	Not_GOST-art	0
1	0	1	Not_GOST-art	0	Not_GOST-art	0
1.33	12.67	1	Not_GOST-art	0	Not_GOST-art	0
1.33	12.67	1	Not_GOST-art	0	Not_GOST-art	0
1.33	12.67	1	Not_GOST-art	0	Not_GOST-art	0
0.56	0.33	1	Not_GOST-art	0	Not_GOST-art	0
0.5	1.25	1	Not_GOST-art	0	Not_GOST-art	0
0.4	0.7	1	Not_GOST-art	0	Not_GOST-art	0
0.15	0.15	1	Not_GOST-art	0	Not_GOST-art	0
0.5	0.83	1	Not_GOST-art	0	Not_GOST-art	0
0.43	0.71	1	Not_GOST-art	0	Not_GOST-art	0
1	1.5	1	Not_GOST-art	0	Not_GOST-art	0
1	1.5	1	Not_GOST-art	0	Not_GOST-art	0
0.5	0	1	Not_GOST-art	0	Not_GOST-art	0
0.46	1.08	1	Not_GOST-art	0	Not_GOST-art	0
0.46	1.08	1	Not_GOST-art	0	Not_GOST-art	0
0.83	3.83	1	Not_GOST-art	0	Not_GOST-art	0
1.38	5.13	1	Not_GOST-art	0	Not_GOST-art	0

1.38	5.13	1	Not_GOST-art	0	Not_GOST-art	0
1.38	5.13	1	Not_GOST-art	0	Not_GOST-art	0
1.14	2.86	1	Not_GOST-art	0	Not_GOST-art	0
1.14	2.86	1	Not_GOST-art	0	Not_GOST-art	0
1.14	2.86	1	Not_GOST-art	0	Not_GOST-art	0
1	1.2	1	Not_GOST-art	0	Not_GOST-art	0
1	1.2	1	Not_GOST-art	0	Not_GOST-art	0
0.43	0.57	1	Not_GOST-art	0	Not_GOST-art	0
1	0	1	Not_GOST-art	0	Not_GOST-art	0
1	0	1	Not_GOST-art	0	Not_GOST-art	0
1	2.5	1	Not_GOST-art	0	Not_GOST-art	0
1	2.5	1	Not_GOST-art	0	Not_GOST-art	0
1	2.5	1	Not_GOST-art	0	Not_GOST-art	0
0.77	2	1	Not_GOST-art	0	Not_GOST-art	0
0.5	0.4	1	Not_GOST-art	0	Not_GOST-art	0
0.6	0.4	1	Not_GOST-art	0	Not_GOST-art	0
0.6	0.4	1	Not_GOST-art	0	Not_GOST-art	0
1.33	2.33	1	Not_GOST-art	0	Not_GOST-art	0
1.33	2.33	1	Not_GOST-art	0	Not_GOST-art	0
1.33	2.33	1	Not_GOST-art	0	Not_GOST-art	0
0.5	0.75	1	Not_GOST-art	0	Not_GOST-art	0
2	14	1	Not_GOST-art	0	GOST-art-Y-al	1
2	14	1	Not_GOST-art	0	GOST-art-Y-al	1
0.25	0.17	1	Not_GOST-art	0	Not_GOST-art	0
1.5	2.25	1	Not_GOST-art	0	Not_GOST-art	0
1.5	2.25	1	Not_GOST-art	0	Not_GOST-art	0
1.6	10.8	1	Not_GOST-art	0	GOST-art-Y-al	1
1.6	10.8	1	Not_GOST-art	0	GOST-art-Y-al	1
0.43	0	1	Not_GOST-art	0	Not_GOST-art	0
0.43	0	1	Not_GOST-art	0	Not_GOST-art	0
0.33	0.33	1	Not_GOST-art	0	Not_GOST-art	0
1.29	1.43	1	GOST-art-all	1	Not_GOST-art	0
2.27	6.47	1	GOST-art-all	1	GOST-art-Y-al	1
1	2.5	1	Not_GOST-art	0	Not_GOST-art	0
0.5	0.9	1	Not_GOST-art	0	Not_GOST-art	0
1.5	0	1	Not_GOST-art	0	Not_GOST-art	0
0.67	0.83	1	Not_GOST-art	0	Not_GOST-art	0
0.67	0.83	1	Not_GOST-art	0	Not_GOST-art	0
1.33	2.22	1	Not_GOST-art	0	Not_GOST-art	0
1.33	2.22	1	Not_GOST-art	0	Not_GOST-art	0
1.33	2.22	1	Not_GOST-art	0	Not_GOST-art	0
1.33	2.22	1	Not_GOST-art	0	Not_GOST-art	0
1	1	1	Not_GOST-art	0	Not_GOST-art	0
1.2	1.2	1	Not_GOST-art	0	Not_GOST-art	0
1.2	1.2	1	Not_GOST-art	0	Not_GOST-art	0
0.5	0.5	1	Not_GOST-art	0	Not_GOST-art	0
1.2	0.6	1	Not_GOST-art	0	Not_GOST-art	0

1.17	0.33	1	Not_GOST-art	0	Not_GOST-art	0
1.27	2	1	GOST-art-all	1	Not_GOST-art	0
1.27	2	1	GOST-art-all	1	Not_GOST-art	0
1.27	2	1	GOST-art-all	1	Not_GOST-art	0
0.2	0.4	1	Not_GOST-art	0	Not_GOST-art	0
1	1.33	1	Not_GOST-art	0	Not_GOST-art	0
0.4	0.8	1	Not_GOST-art	0	Not_GOST-art	0
1.5	1.5	1	Not_GOST-art	0	Not_GOST-art	0
1.5	1.5	1	Not_GOST-art	0	Not_GOST-art	0
2	12	1	Not_GOST-art	0	GOST-art-Y-al	1
0.73	0.6	1	Not_GOST-art	0	Not_GOST-art	0
0.73	0.6	1	Not_GOST-art	0	Not_GOST-art	0
0.73	0.6	1	Not_GOST-art	0	Not_GOST-art	0
1	2	1	Not_GOST-art	0	Not_GOST-art	0
1	2	1	Not_GOST-art	0	Not_GOST-art	0
0.43	2.14	1	Not_GOST-art	0	Not_GOST-art	0
0.43	2.14	1	Not_GOST-art	0	Not_GOST-art	0
1.86	4.14	1	GOST-art-all	1	GOST-art-Y-al	1
1.86	4.14	1	GOST-art-all	1	GOST-art-Y-al	1
0.5	1	1	Not_GOST-art	0	Not_GOST-art	0
0.5	1	1	Not_GOST-art	0	Not_GOST-art	0
1	2.5	1	Not_GOST-art	0	Not_GOST-art	0
1	2.5	1	Not_GOST-art	0	Not_GOST-art	0
0.44	0.44	1	Not_GOST-art	0	Not_GOST-art	0
0.2	0	1	Not_GOST-art	0	Not_GOST-art	0
1	1.25	1	Not_GOST-art	0	Not_GOST-art	0
1	1.25	1	Not_GOST-art	0	Not_GOST-art	0
1	1.25	1	Not_GOST-art	0	Not_GOST-art	0
1	1.25	1	Not_GOST-art	0	Not_GOST-art	0
1.13	3.53	1	GOST-art-all	1	Not_GOST-art	0
1.13	3.53	1	GOST-art-all	1	Not_GOST-art	0
1.5	0.5	1	Not_GOST-art	0	Not_GOST-art	0
1.14	3.29	1	Not_GOST-art	0	Not_GOST-art	0
1.14	3.29	1	Not_GOST-art	0	Not_GOST-art	0
0.5	0.5	1	Not_GOST-art	0	Not_GOST-art	0
1	0.5	1	Not_GOST-art	0	Not_GOST-art	0
1	0.5	1	Not_GOST-art	0	Not_GOST-art	0
0.64	0.18	1	Not_GOST-art	0	Not_GOST-art	0
0.5	0.75	1	Not_GOST-art	0	Not_GOST-art	0
2.29	8	1	GOST-art-all	1	GOST-art-Y-al	1
1.5	1.83	1	Not_GOST-art	0	Not_GOST-art	0
1.5	1.83	1	Not_GOST-art	0	Not_GOST-art	0
1.2	1	1	Not_GOST-art	0	Not_GOST-art	0
0.94	1.24	1	GOST-art-all	1	Not_GOST-art	0
0.94	1.24	1	GOST-art-all	1	Not_GOST-art	0
1.5	1.5	1	Not_GOST-art	0	Not_GOST-art	0
1.5	1.5	1	Not_GOST-art	0	Not_GOST-art	0

0.76	0.94	1	GOST-art-all	1	Not_GOST-art	0
0.67	3.67	1	Not_GOST-art	0	Not_GOST-art	0
0.92	1.83	1	Not_GOST-art	0	Not_GOST-art	0
0.67	0.33	1	Not_GOST-art	0	Not_GOST-art	0
0.58	0.83	1	Not_GOST-art	0	Not_GOST-art	0
0.76	0.94	1	GOST-art-all	1	Not_GOST-art	0
0.5	1.5	1	Not_GOST-art	0	Not_GOST-art	0
0.5	1.5	1	Not_GOST-art	0	Not_GOST-art	0
0.88	0.63	1	Not_GOST-art	0	Not_GOST-art	0
0.88	0.63	1	Not_GOST-art	0	Not_GOST-art	0
0.83	1.67	1	Not_GOST-art	0	Not_GOST-art	0
0.83	1.67	1	Not_GOST-art	0	Not_GOST-art	0
0.83	1.67	1	Not_GOST-art	0	Not_GOST-art	0
0.83	1.67	1	Not_GOST-art	0	Not_GOST-art	0
0.4	0.6	1	Not_GOST-art	0	Not_GOST-art	0
0.5	0.5	1	Not_GOST-art	0	Not_GOST-art	0
1	0	1	Not_GOST-art	0	Not_GOST-art	0
1	0	1	Not_GOST-art	0	Not_GOST-art	0
1	0	1	Not_GOST-art	0	Not_GOST-art	0
0.58	1.42	1	Not_GOST-art	0	Not_GOST-art	0
0.58	1.42	1	Not_GOST-art	0	Not_GOST-art	0
0.58	1.42	1	Not_GOST-art	0	Not_GOST-art	0
0.58	1.42	1	Not_GOST-art	0	Not_GOST-art	0
0.88	0.63	1	Not_GOST-art	0	Not_GOST-art	0
0.5	1	1	Not_GOST-art	0	Not_GOST-art	0
0.83	0.5	1	Not_GOST-art	0	Not_GOST-art	0
0.83	0.5	1	Not_GOST-art	0	Not_GOST-art	0
0.55	1.55	1	Not_GOST-art	0	Not_GOST-art	0
0.67	5.67	1	Not_GOST-art	0	Not_GOST-art	0
0.67	5.67	1	Not_GOST-art	0	Not_GOST-art	0
0.67	5.67	1	Not_GOST-art	0	Not_GOST-art	0
0.67	5.67	1	Not_GOST-art	0	Not_GOST-art	0
1	0.22	1	Not_GOST-art	0	Not_GOST-art	0
1	3.5	1	Not_GOST-art	0	Not_GOST-art	0
1	3.5	1	Not_GOST-art	0	Not_GOST-art	0
0.67	1.83	1	Not_GOST-art	0	Not_GOST-art	0
1.07	1.8	1	GOST-art-all	1	Not_GOST-art	0
0.75	7	1	Not_GOST-art	0	Not_GOST-art	0
0.4	0	1	Not_GOST-art	0	Not_GOST-art	0
0.75	1	1	Not_GOST-art	0	Not_GOST-art	0
0.75	1	1	Not_GOST-art	0	Not_GOST-art	0
3.33	10.67	1	Not_GOST-art	0	GOST-art-Y-al	1
3.33	10.67	1	Not_GOST-art	0	GOST-art-Y-al	1
3.33	10.67	1	Not_GOST-art	0	GOST-art-Y-al	1
2.14	8.57	1	GOST-art-all	1	GOST-art-Y-al	1
2.14	8.57	1	GOST-art-all	1	GOST-art-Y-al	1
0.5	2	1	Not_GOST-art	0	Not_GOST-art	0

0.5	2	1	Not_GOST-art	0	Not_GOST-art	0
1.2	0.2	1	Not_GOST-art	0	Not_GOST-art	0
0.5	0.25	1	Not_GOST-art	0	Not_GOST-art	0
1.33	1.33	1	Not_GOST-art	0	Not_GOST-art	0
1.33	1.33	1	Not_GOST-art	0	Not_GOST-art	0
0.57	0.86	1	Not_GOST-art	0	Not_GOST-art	0
0.57	0.86	1	Not_GOST-art	0	Not_GOST-art	0
1.14	0.86	1	Not_GOST-art	0	Not_GOST-art	0
1	1.5	1	Not_GOST-art	0	Not_GOST-art	0
1.75	3.75	1	Not_GOST-art	0	GOST-art-Y-al	1
1.43	6.14	1	Not_GOST-art	0	Not_GOST-art	0
1.43	6.14	1	Not_GOST-art	0	Not_GOST-art	0
0.75	2.5	1	Not_GOST-art	0	Not_GOST-art	0
0.75	2.5	1	Not_GOST-art	0	Not_GOST-art	0
1	1.5	1	Not_GOST-art	0	Not_GOST-art	0
1	1.5	1	Not_GOST-art	0	Not_GOST-art	0
1	7.67	1	Not_GOST-art	0	Not_GOST-art	0
1	7.67	1	Not_GOST-art	0	Not_GOST-art	0
0.67	0	1	Not_GOST-art	0	Not_GOST-art	0
0.67	0	1	Not_GOST-art	0	Not_GOST-art	0
1.17	2	1	Not_GOST-art	0	Not_GOST-art	0
1.17	2	1	Not_GOST-art	0	Not_GOST-art	0
0.42	1.17	1	Not_GOST-art	0	Not_GOST-art	0
1.75	0.75	1	GOST-art-all	1	GOST-art-Y-al	1
1.75	0.75	1	GOST-art-all	1	GOST-art-Y-al	1
1.75	0.75	1	GOST-art-all	1	GOST-art-Y-al	1
0.75	0.25	1	Not_GOST-art	0	Not_GOST-art	0
1	2.33	1	Not_GOST-art	0	Not_GOST-art	0
1	2.33	1	Not_GOST-art	0	Not_GOST-art	0
2	9	1	Not_GOST-art	0	GOST-art-Y-al	1
2	9	1	Not_GOST-art	0	GOST-art-Y-al	1
0.71	1.59	1	Not_GOST-art	0	Not_GOST-art	0
0.71	1.59	1	Not_GOST-art	0	Not_GOST-art	0
0.33	0.17	1	Not_GOST-art	0	Not_GOST-art	0
1	0.8	1	Not_GOST-art	0	Not_GOST-art	0
1	0.8	1	Not_GOST-art	0	Not_GOST-art	0
0.67	0.67	1	Not_GOST-art	0	Not_GOST-art	0
0.67	1.33	1	Not_GOST-art	0	Not_GOST-art	0
1.25	6	1	Not_GOST-art	0	Not_GOST-art	0
1.25	6	1	Not_GOST-art	0	Not_GOST-art	0
0.67	1	1	Not_GOST-art	0	Not_GOST-art	0
0.5	0	1	Not_GOST-art	0	Not_GOST-art	0
1.46	2	1	GOST-art-all	1	Not_GOST-art	0
1.33	2.33	1	Not_GOST-art	0	Not_GOST-art	0
1.33	2.33	1	Not_GOST-art	0	Not_GOST-art	0
1	2	1	Not_GOST-art	0	Not_GOST-art	0
0.2	0.33	1	Not_GOST-art	0	Not_GOST-art	0

0.71	1	1	Not_GOST-art	0	Not_GOST-art	0
0.71	1	1	Not_GOST-art	0	Not_GOST-art	0
0.63	0.25	1	Not_GOST-art	0	Not_GOST-art	0
2.08	6.92	1	GOST-art-all	1	GOST-art-Y-al	1
2.08	6.92	1	GOST-art-all	1	GOST-art-Y-al	1
2.08	6.92	1	GOST-art-all	1	GOST-art-Y-al	1
0.67	0.5	1	Not_GOST-art	0	Not_GOST-art	0
0.67	0.5	1	Not_GOST-art	0	Not_GOST-art	0
0.75	1.38	1	Not_GOST-art	0	Not_GOST-art	0
0.67	0	1	Not_GOST-art	0	Not_GOST-art	0
0.67	0	1	Not_GOST-art	0	Not_GOST-art	0
0.86	4.14	1	Not_GOST-art	0	Not_GOST-art	0
0.86	4.14	1	Not_GOST-art	0	Not_GOST-art	0
0.86	4.14	1	Not_GOST-art	0	Not_GOST-art	0
1.2	1	1	Not_GOST-art	0	Not_GOST-art	0
0.67	0.67	1	Not_GOST-art	0	Not_GOST-art	0
0.67	0.67	1	Not_GOST-art	0	Not_GOST-art	0
0.5	0.67	1	Not_GOST-art	0	Not_GOST-art	0
0.5	0.38	1	Not_GOST-art	0	Not_GOST-art	0
2.89	9.33	1	GOST-art-all	1	GOST-art-Y-al	1
1.13	1.38	1	Not_GOST-art	0	Not_GOST-art	0
0.87	0.93	1	GOST-art-all	1	Not_GOST-art	0
1	0.67	1	Not_GOST-art	0	Not_GOST-art	0
1	0.67	1	Not_GOST-art	0	Not_GOST-art	0
0.67	0.67	1	Not_GOST-art	0	Not_GOST-art	0
0.67	0	1	Not_GOST-art	0	Not_GOST-art	0
1.4	1.47	1	GOST-art-all	1	Not_GOST-art	0
1.4	1.47	1	GOST-art-all	1	Not_GOST-art	0
0.5	0.17	1	Not_GOST-art	0	Not_GOST-art	0
1.67	1.33	1	Not_GOST-art	0	GOST-art-Y-al	1
1.67	1.33	1	Not_GOST-art	0	GOST-art-Y-al	1
1.67	1.33	1	Not_GOST-art	0	GOST-art-Y-al	1
1.67	1.33	1	Not_GOST-art	0	GOST-art-Y-al	1
1.13	1.5	1	Not_GOST-art	0	Not_GOST-art	0
1.33	0.67	1	Not_GOST-art	0	Not_GOST-art	0
1.33	0.67	1	Not_GOST-art	0	Not_GOST-art	0
1.33	0.67	1	Not_GOST-art	0	Not_GOST-art	0
0.8	2.2	1	Not_GOST-art	0	Not_GOST-art	0
0.6	1.8	1	Not_GOST-art	0	Not_GOST-art	0
1.67	3	1	Not_GOST-art	0	GOST-art-Y-al	1
0.75	1	1	Not_GOST-art	0	Not_GOST-art	0
0.75	1	1	Not_GOST-art	0	Not_GOST-art	0
1.75	3	1	Not_GOST-art	0	GOST-art-Y-al	1
1.75	3	1	Not_GOST-art	0	GOST-art-Y-al	1
1.75	3	1	Not_GOST-art	0	GOST-art-Y-al	1
0.4	0	1	Not_GOST-art	0	Not_GOST-art	0
1	1.25	1	Not_GOST-art	0	Not_GOST-art	0

0.83	2.08	1	Not_GOST-art	0	Not_GOST-art	0
0.5	0.43	1	Not_GOST-art	0	Not_GOST-art	0
0.4	0.4	1	Not_GOST-art	0	Not_GOST-art	0
1.13	10.88	1	Not_GOST-art	0	Not_GOST-art	0
0.75	6	1	Not_GOST-art	0	Not_GOST-art	0
2.29	11.43	1	GOST-art-all	1	GOST-art-Y-al	1
2.29	11.43	1	GOST-art-all	1	GOST-art-Y-al	1
0.83	2.11	1	GOST-art-all	1	Not_GOST-art	0
0.6	0.6	1	Not_GOST-art	0	Not_GOST-art	0
0.6	0.6	1	Not_GOST-art	0	Not_GOST-art	0
0.56	2	1	Not_GOST-art	0	Not_GOST-art	0
0.56	2	1	Not_GOST-art	0	Not_GOST-art	0
0.56	0.39	1	Not_GOST-art	0	Not_GOST-art	0
0.8	1.3	1	Not_GOST-art	0	Not_GOST-art	0
0.33	0.22	1	Not_GOST-art	0	Not_GOST-art	0
0.33	0	1	Not_GOST-art	0	Not_GOST-art	0
1	3.25	1	Not_GOST-art	0	Not_GOST-art	0
0.38	1	1	Not_GOST-art	0	Not_GOST-art	0
1	1.5	1	Not_GOST-art	0	Not_GOST-art	0
1.33	1	1	Not_GOST-art	0	Not_GOST-art	0
0.43	0.14	1	Not_GOST-art	0	Not_GOST-art	0
0.67	0.33	1	Not_GOST-art	0	Not_GOST-art	0
1.17	1.67	1	Not_GOST-art	0	Not_GOST-art	0
1.17	1.67	1	Not_GOST-art	0	Not_GOST-art	0
0.25	0.13	1	Not_GOST-art	0	Not_GOST-art	0
1	1.83	1	Not_GOST-art	0	Not_GOST-art	0
0.43	0.43	1	Not_GOST-art	0	Not_GOST-art	0
1	5.83	1	Not_GOST-art	0	Not_GOST-art	0
0.21	0.21	1	Not_GOST-art	0	Not_GOST-art	0
0.5	0.5	1	Not_GOST-art	0	Not_GOST-art	0
0.87	1.07	1	GOST-art-all	1	Not_GOST-art	0
0.87	1.07	1	GOST-art-all	1	Not_GOST-art	0
1	1.5	1	Not_GOST-art	0	Not_GOST-art	0
0.75	1	1	Not_GOST-art	0	Not_GOST-art	0
1	2.78	1	Not_GOST-art	0	Not_GOST-art	0
0.38	0.25	1	Not_GOST-art	0	Not_GOST-art	0
0.5	0	1	Not_GOST-art	0	Not_GOST-art	0
2	5.67	1	Not_GOST-art	0	GOST-art-Y-al	1
2	5.67	1	Not_GOST-art	0	GOST-art-Y-al	1
2	5.67	1	Not_GOST-art	0	GOST-art-Y-al	1
0.67	0.67	1	Not_GOST-art	0	Not_GOST-art	0
0.67	0.67	1	Not_GOST-art	0	Not_GOST-art	0
0.29	0.57	1	Not_GOST-art	0	Not_GOST-art	0
1	1	1	Not_GOST-art	0	Not_GOST-art	0
0.75	0.5	1	Not_GOST-art	0	Not_GOST-art	0
0.75	0.5	1	Not_GOST-art	0	Not_GOST-art	0
1.17	3.5	1	Not_GOST-art	0	Not_GOST-art	0

1.17	3.5	1	Not_GOST-art	0	Not_GOST-art	0
0.6	0.2	1	Not_GOST-art	0	Not_GOST-art	0
0.67	1.67	1	Not_GOST-art	0	Not_GOST-art	0
0.94	1.56	1	GOST-art-all	1	Not_GOST-art	0
1	2	1	Not_GOST-art	0	Not_GOST-art	0
1	2	1	Not_GOST-art	0	Not_GOST-art	0
0.29	0.14	1	Not_GOST-art	0	Not_GOST-art	0
1.35	1.53	1	GOST-art-all	1	Not_GOST-art	0
1.33	1.33	1	Not_GOST-art	0	Not_GOST-art	0
1.33	1.33	1	Not_GOST-art	0	Not_GOST-art	0
1.33	1.33	1	Not_GOST-art	0	Not_GOST-art	0
1.33	1.33	1	Not_GOST-art	0	Not_GOST-art	0
1.57	8.57	1	Not_GOST-art	0	GOST-art-Y-al	1
1.57	8.57	1	Not_GOST-art	0	GOST-art-Y-al	1
1.57	8.57	1	Not_GOST-art	0	GOST-art-Y-al	1
0.43	1.14	1	Not_GOST-art	0	Not_GOST-art	0
4	6.75	1	GOST-art-all	1	GOST-art-Y-al	1
4	6.75	1	GOST-art-all	1	GOST-art-Y-al	1
4	6.75	1	GOST-art-all	1	GOST-art-Y-al	1
0.67	1	1	Not_GOST-art	0	Not_GOST-art	0
0.67	1	1	Not_GOST-art	0	Not_GOST-art	0
0.15	1.69	1	Not_GOST-art	0	Not_GOST-art	0
0.71	0.47	1	Not_GOST-art	0	Not_GOST-art	0
0.67	3	1	Not_GOST-art	0	Not_GOST-art	0
0.67	3	1	Not_GOST-art	0	Not_GOST-art	0
0.75	1	1	Not_GOST-art	0	Not_GOST-art	0
0.75	1	1	Not_GOST-art	0	Not_GOST-art	0
1.86	2.14	1	GOST-art-all	1	GOST-art-Y-al	1
1.86	2.14	1	GOST-art-all	1	GOST-art-Y-al	1
1.86	2.14	1	GOST-art-all	1	GOST-art-Y-al	1
0.67	3.67	1	Not_GOST-art	0	Not_GOST-art	0
0.33	2	1	Not_GOST-art	0	Not_GOST-art	0
0.67	0.67	1	Not_GOST-art	0	Not_GOST-art	0
1.4	12.1	1	GOST-art-all	1	Not_GOST-art	0
1.4	12.1	1	GOST-art-all	1	Not_GOST-art	0
1.4	12.1	1	GOST-art-all	1	Not_GOST-art	0
1.4	12.1	1	GOST-art-all	1	Not_GOST-art	0
0.67	0.67	1	Not_GOST-art	0	Not_GOST-art	0
1.6	3.1	1	GOST-art-all	1	GOST-art-Y-al	1
1.6	3.1	1	GOST-art-all	1	GOST-art-Y-al	1
1.4	7.6	1	Not_GOST-art	0	Not_GOST-art	0
1.4	7.6	1	Not_GOST-art	0	Not_GOST-art	0
1	1	1	Not_GOST-art	0	Not_GOST-art	0
0.53	1.18	1	Not_GOST-art	0	Not_GOST-art	0
0.53	1.18	1	Not_GOST-art	0	Not_GOST-art	0
0.6	1.4	1	Not_GOST-art	0	Not_GOST-art	0
0.6	1.4	1	Not_GOST-art	0	Not_GOST-art	0

0.29	0	1	Not_GOST-art	0	Not_GOST-art	0
0.29	0	1	Not_GOST-art	0	Not_GOST-art	0
1	1	1	Not_GOST-art	0	Not_GOST-art	0
1.33	1.33	1	Not_GOST-art	0	Not_GOST-art	0
1.33	1.33	1	Not_GOST-art	0	Not_GOST-art	0
1.33	1.33	1	Not_GOST-art	0	Not_GOST-art	0
1.33	1.33	1	Not_GOST-art	0	Not_GOST-art	0
1	2	1	Not_GOST-art	0	Not_GOST-art	0
0.5	0.33	1	Not_GOST-art	0	Not_GOST-art	0
1	0	1	Not_GOST-art	0	Not_GOST-art	0
0.38	0.63	1	Not_GOST-art	0	Not_GOST-art	0
1.67	3	1	Not_GOST-art	0	GOST-art-Y-al	1
0.5	0.33	1	Not_GOST-art	0	Not_GOST-art	0
0.8	1.6	1	Not_GOST-art	0	Not_GOST-art	0
0.67	0.17	1	Not_GOST-art	0	Not_GOST-art	0
0.67	1.33	1	Not_GOST-art	0	Not_GOST-art	0
0.67	1.33	1	Not_GOST-art	0	Not_GOST-art	0
1.5	5	1	Not_GOST-art	0	Not_GOST-art	0
1.5	5	1	Not_GOST-art	0	Not_GOST-art	0
1.5	5	1	Not_GOST-art	0	Not_GOST-art	0
1	6.5	1	Not_GOST-art	0	Not_GOST-art	0
1	6.5	1	Not_GOST-art	0	Not_GOST-art	0
1	1.33	1	Not_GOST-art	0	Not_GOST-art	0
1	1.33	1	Not_GOST-art	0	Not_GOST-art	0
0.5	0	1	Not_GOST-art	0	Not_GOST-art	0
1	1.33	1	Not_GOST-art	0	Not_GOST-art	0
1	3.21	1	GOST-art-all	1	Not_GOST-art	0
1.21	5.36	1	GOST-art-all	1	Not_GOST-art	0
0.5	0.83	1	Not_GOST-art	0	Not_GOST-art	0
0.67	2.67	1	Not_GOST-art	0	Not_GOST-art	0
0.67	2.67	1	Not_GOST-art	0	Not_GOST-art	0
1.33	2.33	1	Not_GOST-art	0	Not_GOST-art	0
1.33	2.33	1	Not_GOST-art	0	Not_GOST-art	0
1	3.78	1	Not_GOST-art	0	Not_GOST-art	0
1	3.78	1	Not_GOST-art	0	Not_GOST-art	0
1	0.5	1	Not_GOST-art	0	Not_GOST-art	0
1	0.75	1	Not_GOST-art	0	Not_GOST-art	0
0.57	0.29	1	Not_GOST-art	0	Not_GOST-art	0
0.57	0.29	1	Not_GOST-art	0	Not_GOST-art	0
0.67	0.33	1	Not_GOST-art	0	Not_GOST-art	0
0.5	1	1	Not_GOST-art	0	Not_GOST-art	0
0.5	0.17	1	Not_GOST-art	0	Not_GOST-art	0
0.5	0.17	1	Not_GOST-art	0	Not_GOST-art	0
0.67	0	1	Not_GOST-art	0	Not_GOST-art	0
1.67	1.08	1	GOST-art-all	1	GOST-art-Y-al	1
2.5	7.63	1	GOST-art-all	1	GOST-art-Y-al	1
2.5	7.63	1	GOST-art-all	1	GOST-art-Y-al	1

2.5	7.63	1	GOST-art-all	1	GOST-art-Y-al	1
1.33	2.33	1	Not_GOST-art	0	Not_GOST-art	0
1.33	2.33	1	Not_GOST-art	0	Not_GOST-art	0
1.33	2.33	1	Not_GOST-art	0	Not_GOST-art	0
1	3	1	Not_GOST-art	0	Not_GOST-art	0
0.67	0.17	1	Not_GOST-art	0	Not_GOST-art	0
0.5	0.1	1	Not_GOST-art	0	Not_GOST-art	0
0.67	2.5	1	Not_GOST-art	0	Not_GOST-art	0
0.73	0.67	1	Not_GOST-art	0	Not_GOST-art	0
0.67	0	1	Not_GOST-art	0	Not_GOST-art	0
0.67	0	1	Not_GOST-art	0	Not_GOST-art	0
2.2	0.6	1	Not_GOST-art	0	GOST-art-Y-al	1
1	1	1	Not_GOST-art	0	Not_GOST-art	0
1.5	5.5	1	Not_GOST-art	0	Not_GOST-art	0
1.5	5.5	1	Not_GOST-art	0	Not_GOST-art	0
1	7.33	1	Not_GOST-art	0	Not_GOST-art	0
1	7.33	1	Not_GOST-art	0	Not_GOST-art	0
1	0.33	1	Not_GOST-art	0	Not_GOST-art	0
1	0.33	1	Not_GOST-art	0	Not_GOST-art	0
0.67	0.67	1	Not_GOST-art	0	Not_GOST-art	0
0.67	0.67	1	Not_GOST-art	0	Not_GOST-art	0
1.14	6.71	1	Not_GOST-art	0	Not_GOST-art	0
0.92	2.25	1	Not_GOST-art	0	Not_GOST-art	0
0.29	0.14	1	Not_GOST-art	0	Not_GOST-art	0
0.4	1	1	Not_GOST-art	0	Not_GOST-art	0
1.33	7.67	1	Not_GOST-art	0	Not_GOST-art	0
1.33	7.67	1	Not_GOST-art	0	Not_GOST-art	0
0.5	3.88	1	Not_GOST-art	0	Not_GOST-art	0
0.5	3.88	1	Not_GOST-art	0	Not_GOST-art	0
0.5	0	1	Not_GOST-art	0	Not_GOST-art	0
1.86	5	1	GOST-art-all	1	GOST-art-Y-al	1
1.86	5	1	GOST-art-all	1	GOST-art-Y-al	1
2	24.67	1	Not_GOST-art	0	GOST-art-Y-al	1
0.38	0.25	1	Not_GOST-art	0	Not_GOST-art	0
0.8	0.7	1	Not_GOST-art	0	Not_GOST-art	0
0.67	1	1	Not_GOST-art	0	Not_GOST-art	0
0.67	1	1	Not_GOST-art	0	Not_GOST-art	0
0.4	0	1	Not_GOST-art	0	Not_GOST-art	0
0.67	0.33	1	Not_GOST-art	0	Not_GOST-art	0
1.4	5.8	1	Not_GOST-art	0	Not_GOST-art	0
1.08	2.38	1	GOST-art-all	1	Not_GOST-art	0
0.44	1.11	1	Not_GOST-art	0	Not_GOST-art	0
1.08	1.15	1	GOST-art-all	1	Not_GOST-art	0
1.08	1.15	1	GOST-art-all	1	Not_GOST-art	0
1	2.5	1	Not_GOST-art	0	Not_GOST-art	0
1.17	2.83	1	Not_GOST-art	0	Not_GOST-art	0
0.86	2	1	Not_GOST-art	0	Not_GOST-art	0

2	24.67	1	Not_GOST-art	0	GOST-art-Y-al	1
2	24.67	1	Not_GOST-art	0	GOST-art-Y-al	1
1.25	2.75	1	Not_GOST-art	0	Not_GOST-art	0
0.67	0.67	1	Not_GOST-art	0	Not_GOST-art	0
0.67	0.67	1	Not_GOST-art	0	Not_GOST-art	0
1.33	4.67	1	Not_GOST-art	0	Not_GOST-art	0
0.29	0	1	Not_GOST-art	0	Not_GOST-art	0
1	0	1	Not_GOST-art	0	Not_GOST-art	0
1	1.67	1	Not_GOST-art	0	Not_GOST-art	0
1	1.67	1	Not_GOST-art	0	Not_GOST-art	0
0.71	1.43	1	Not_GOST-art	0	Not_GOST-art	0
1.5	2.5	1	Not_GOST-art	0	Not_GOST-art	0
1.5	2.5	1	Not_GOST-art	0	Not_GOST-art	0
1.2	1.5	1	Not_GOST-art	0	Not_GOST-art	0
0.6	13.4	1	Not_GOST-art	0	Not_GOST-art	0
0.5	3	1	Not_GOST-art	0	Not_GOST-art	0
0.69	0.38	1	Not_GOST-art	0	Not_GOST-art	0
0.63	0.63	1	Not_GOST-art	0	Not_GOST-art	0
0.63	0.63	1	Not_GOST-art	0	Not_GOST-art	0
1	1.67	1	Not_GOST-art	0	Not_GOST-art	0
1	1.67	1	Not_GOST-art	0	Not_GOST-art	0
0.4	0.4	1	Not_GOST-art	0	Not_GOST-art	0
0.89	1.33	1	Not_GOST-art	0	Not_GOST-art	0
1	0.25	1	Not_GOST-art	0	Not_GOST-art	0
1.25	14.5	1	Not_GOST-art	0	Not_GOST-art	0
1.25	14.5	1	Not_GOST-art	0	Not_GOST-art	0
1	0.33	1	Not_GOST-art	0	Not_GOST-art	0
1	0.33	1	Not_GOST-art	0	Not_GOST-art	0
1	0.33	1	Not_GOST-art	0	Not_GOST-art	0
0.5	0.33	1	Not_GOST-art	0	Not_GOST-art	0
2	2	1	Not_GOST-art	0	GOST-art-Y-al	1
0.45	0.82	1	Not_GOST-art	0	Not_GOST-art	0
0.45	0.82	1	Not_GOST-art	0	Not_GOST-art	0
1	2	1	Not_GOST-art	0	Not_GOST-art	0
0.67	1.33	1	Not_GOST-art	0	Not_GOST-art	0
0.5	0.58	1	Not_GOST-art	0	Not_GOST-art	0
1.33	1	1	Not_GOST-art	0	Not_GOST-art	0
1.33	1	1	Not_GOST-art	0	Not_GOST-art	0
1.33	1	1	Not_GOST-art	0	Not_GOST-art	0
0.25	0.75	1	Not_GOST-art	0	Not_GOST-art	0
0.67	1.33	1	Not_GOST-art	0	Not_GOST-art	0
0.29	0	1	Not_GOST-art	0	Not_GOST-art	0
1.64	3.36	1	GOST-art-all	1	GOST-art-Y-al	1
0.27	0.07	1	Not_GOST-art	0	Not_GOST-art	0
0.71	0.29	1	Not_GOST-art	0	Not_GOST-art	0
0.71	0.29	1	Not_GOST-art	0	Not_GOST-art	0
0.67	0	1	Not_GOST-art	0	Not_GOST-art	0

0.33	0.17	1	Not_GOST-art	0	Not_GOST-art	0
1.5	1	1	Not_GOST-art	0	Not_GOST-art	0
1.5	1	1	Not_GOST-art	0	Not_GOST-art	0
1.5	1	1	Not_GOST-art	0	Not_GOST-art	0
0.33	0.17	1	Not_GOST-art	0	Not_GOST-art	0
1.4	2.6	1	Not_GOST-art	0	Not_GOST-art	0
1.4	2.6	1	Not_GOST-art	0	Not_GOST-art	0
1.4	2.6	1	Not_GOST-art	0	Not_GOST-art	0
0.67	1.17	1	Not_GOST-art	0	Not_GOST-art	0
1.25	0.25	1	Not_GOST-art	0	Not_GOST-art	0
2	13.5	1	Not_GOST-art	0	GOST-art-Y-al	1
2	13.5	1	Not_GOST-art	0	GOST-art-Y-al	1
0.67	0.33	1	Not_GOST-art	0	Not_GOST-art	0
0.67	0.33	1	Not_GOST-art	0	Not_GOST-art	0
1	1.67	1	Not_GOST-art	0	Not_GOST-art	0
1	1.67	1	Not_GOST-art	0	Not_GOST-art	0
0.67	1	1	Not_GOST-art	0	Not_GOST-art	0
0.67	1	1	Not_GOST-art	0	Not_GOST-art	0
0.5	0.25	1	Not_GOST-art	0	Not_GOST-art	0
1.57	4.43	1	Not_GOST-art	0	GOST-art-Y-al	1
1.57	4.43	1	Not_GOST-art	0	GOST-art-Y-al	1
0.29	0.71	1	Not_GOST-art	0	Not_GOST-art	0
0.43	5.57	1	Not_GOST-art	0	Not_GOST-art	0
0.29	0.86	1	Not_GOST-art	0	Not_GOST-art	0
2.33	0.5	1	GOST-art-all	1	GOST-art-Y-al	1
0.7	0.4	1	Not_GOST-art	0	Not_GOST-art	0
1.5	0.5	1	Not_GOST-art	0	Not_GOST-art	0
1.5	0.5	1	Not_GOST-art	0	Not_GOST-art	0
0.13	0.07	1	Not_GOST-art	0	Not_GOST-art	0
0.5	0.17	1	Not_GOST-art	0	Not_GOST-art	0
0.5	0.5	1	Not_GOST-art	0	Not_GOST-art	0
0.67	0.22	1	Not_GOST-art	0	Not_GOST-art	0
0.67	6.33	1	Not_GOST-art	0	Not_GOST-art	0
0.86	2.14	1	Not_GOST-art	0	Not_GOST-art	0
1.33	5.67	1	Not_GOST-art	0	Not_GOST-art	0
0.67	1.67	1	Not_GOST-art	0	Not_GOST-art	0
0.67	1.67	1	Not_GOST-art	0	Not_GOST-art	0
1	4.67	1	Not_GOST-art	0	Not_GOST-art	0
0.5	0.25	1	Not_GOST-art	0	Not_GOST-art	0
0.5	0	1	Not_GOST-art	0	Not_GOST-art	0
1	6	1	Not_GOST-art	0	Not_GOST-art	0
1	6	1	Not_GOST-art	0	Not_GOST-art	0
2.8	6.2	1	GOST-art-all	1	GOST-art-Y-al	1
2.8	6.2	1	GOST-art-all	1	GOST-art-Y-al	1
2.8	6.2	1	GOST-art-all	1	GOST-art-Y-al	1
1.5	6.38	1	Not_GOST-art	0	Not_GOST-art	0
1.5	6.38	1	Not_GOST-art	0	Not_GOST-art	0

1.5	6.38	1	Not_GOST-art	0	Not_GOST-art	0
1.5	6.38	1	Not_GOST-art	0	Not_GOST-art	0
1.5	6.38	1	Not_GOST-art	0	Not_GOST-art	0
0.75	0	1	Not_GOST-art	0	Not_GOST-art	0
0.75	0	1	Not_GOST-art	0	Not_GOST-art	0
1	2.33	1	Not_GOST-art	0	Not_GOST-art	0
0.67	0	1	Not_GOST-art	0	Not_GOST-art	0
0.4	0.4	1	Not_GOST-art	0	Not_GOST-art	0
0.43	0.71	1	Not_GOST-art	0	Not_GOST-art	0
1.5	0.5	1	Not_GOST-art	0	Not_GOST-art	0
1.5	0.5	1	Not_GOST-art	0	Not_GOST-art	0
1.33	4.33	1	Not_GOST-art	0	Not_GOST-art	0
0.5	0.75	1	Not_GOST-art	0	Not_GOST-art	0
0.5	0.25	1	Not_GOST-art	0	Not_GOST-art	0
0.4	0.8	1	Not_GOST-art	0	Not_GOST-art	0
0.4	0.7	1	Not_GOST-art	0	Not_GOST-art	0
0.62	0.69	1	Not_GOST-art	0	Not_GOST-art	0
1.33	7	1	Not_GOST-art	0	Not_GOST-art	0
0.71	0.86	1	Not_GOST-art	0	Not_GOST-art	0
0.71	0.86	1	Not_GOST-art	0	Not_GOST-art	0
1.33	4.33	1	Not_GOST-art	0	Not_GOST-art	0
1.33	4.33	1	Not_GOST-art	0	Not_GOST-art	0
0.5	0.5	1	Not_GOST-art	0	Not_GOST-art	0
0.5	1	1	Not_GOST-art	0	Not_GOST-art	0
1	2	1	Not_GOST-art	0	Not_GOST-art	0
1	2	1	Not_GOST-art	0	Not_GOST-art	0
0.86	5.86	1	Not_GOST-art	0	Not_GOST-art	0
1.13	1.75	1	Not_GOST-art	0	Not_GOST-art	0
1.13	1.75	1	Not_GOST-art	0	Not_GOST-art	0
0.5	0.25	1	Not_GOST-art	0	Not_GOST-art	0
0.43	0.57	1	Not_GOST-art	0	Not_GOST-art	0
1	2.5	1	Not_GOST-art	0	Not_GOST-art	0
1	2.5	1	Not_GOST-art	0	Not_GOST-art	0
1.67	3	1	Not_GOST-art	0	GOST-art-Y-al	1
1.67	3	1	Not_GOST-art	0	GOST-art-Y-al	1
0.14	0.57	1	Not_GOST-art	0	Not_GOST-art	0
0.5	0	1	Not_GOST-art	0	Not_GOST-art	0
1	4.5	1	Not_GOST-art	0	Not_GOST-art	0
1	4.5	1	Not_GOST-art	0	Not_GOST-art	0
0.5	0.92	1	Not_GOST-art	0	Not_GOST-art	0
0.5	1.3	1	Not_GOST-art	0	Not_GOST-art	0
0.5	1.3	1	Not_GOST-art	0	Not_GOST-art	0
1.33	1.67	1	Not_GOST-art	0	Not_GOST-art	0
1.33	1.67	1	Not_GOST-art	0	Not_GOST-art	0
0.2	0.4	1	Not_GOST-art	0	Not_GOST-art	0
1.17	2.33	1	Not_GOST-art	0	Not_GOST-art	0
0.67	0.33	1	Not_GOST-art	0	Not_GOST-art	0

0.67	0.33	1	Not_GOST-art	0	Not_GOST-art	0
1.33	4.33	1	Not_GOST-art	0	Not_GOST-art	0
3	6	1	Not_GOST-art	0	GOST-art-Y-al	1
3	6	1	Not_GOST-art	0	GOST-art-Y-al	1
3	6	1	Not_GOST-art	0	GOST-art-Y-al	1
3	6	1	Not_GOST-art	0	GOST-art-Y-al	1
2.3	4.6	1	GOST-art-all	1	GOST-art-Y-al	1
1.17	1.67	1	Not_GOST-art	0	Not_GOST-art	0
1.17	1.67	1	Not_GOST-art	0	Not_GOST-art	0
1.17	1.67	1	Not_GOST-art	0	Not_GOST-art	0
1	1	1	Not_GOST-art	0	Not_GOST-art	0
0.62	0.85	1	Not_GOST-art	0	Not_GOST-art	0
0.6	1	1	Not_GOST-art	0	Not_GOST-art	0
1	6.33	1	Not_GOST-art	0	Not_GOST-art	0
1	6.33	1	Not_GOST-art	0	Not_GOST-art	0
1	6.33	1	Not_GOST-art	0	Not_GOST-art	0
1.5	2.75	1	Not_GOST-art	0	Not_GOST-art	0
1.5	2.75	1	Not_GOST-art	0	Not_GOST-art	0
1.5	2.75	1	Not_GOST-art	0	Not_GOST-art	0
1.5	2.75	1	Not_GOST-art	0	Not_GOST-art	0
1.3	0.2	1	GOST-art-all	1	Not_GOST-art	0
1.3	0.2	1	GOST-art-all	1	Not_GOST-art	0
1.25	2	1	Not_GOST-art	0	Not_GOST-art	0
1.25	2	1	Not_GOST-art	0	Not_GOST-art	0
1	1	1	Not_GOST-art	0	Not_GOST-art	0
1	1	1	Not_GOST-art	0	Not_GOST-art	0
1	1	1	Not_GOST-art	0	Not_GOST-art	0
0.3	1.7	1	Not_GOST-art	0	Not_GOST-art	0
0.5	4	1	Not_GOST-art	0	Not_GOST-art	0
0.5	4	1	Not_GOST-art	0	Not_GOST-art	0
0.67	2.33	1	Not_GOST-art	0	Not_GOST-art	0
1.5	11	1	Not_GOST-art	0	Not_GOST-art	0
2.33	1.17	1	GOST-art-all	1	GOST-art-Y-al	1
2.33	1.17	1	GOST-art-all	1	GOST-art-Y-al	1
2.33	1.17	1	GOST-art-all	1	GOST-art-Y-al	1
0.67	0	1	Not_GOST-art	0	Not_GOST-art	0
1.75	1.81	1	GOST-art-all	1	GOST-art-Y-al	1
0.88	0.63	1	Not_GOST-art	0	Not_GOST-art	0
0.5	0.75	1	Not_GOST-art	0	Not_GOST-art	0
0.67	0	1	Not_GOST-art	0	Not_GOST-art	0
0.67	0.5	1	Not_GOST-art	0	Not_GOST-art	0
0.67	0.5	1	Not_GOST-art	0	Not_GOST-art	0
0.75	2.5	1	Not_GOST-art	0	Not_GOST-art	0
0.75	2.5	1	Not_GOST-art	0	Not_GOST-art	0
1	0	1	Not_GOST-art	0	Not_GOST-art	0
0.6	1	1	Not_GOST-art	0	Not_GOST-art	0
0.6	1	1	Not_GOST-art	0	Not_GOST-art	0

1.43	1.86	1	Not_GOST-art	0	Not_GOST-art	0
1.43	1.86	1	Not_GOST-art	0	Not_GOST-art	0
0.53	3.07	1	Not_GOST-art	0	Not_GOST-art	0
0.53	3.07	1	Not_GOST-art	0	Not_GOST-art	0
0.53	3.07	1	Not_GOST-art	0	Not_GOST-art	0
3.71	10.86	1	GOST-art-all	1	GOST-art-Y-al	1
3.71	10.86	1	GOST-art-all	1	GOST-art-Y-al	1
3.71	10.86	1	GOST-art-all	1	GOST-art-Y-al	1
0.75	0	1	Not_GOST-art	0	Not_GOST-art	0
1	1	1	Not_GOST-art	0	Not_GOST-art	0
0.5	0.88	1	Not_GOST-art	0	Not_GOST-art	0
1	1	1	Not_GOST-art	0	Not_GOST-art	0
1	1	1	Not_GOST-art	0	Not_GOST-art	0
0.67	0	1	Not_GOST-art	0	Not_GOST-art	0
0.67	2.67	1	Not_GOST-art	0	Not_GOST-art	0
0.67	2.67	1	Not_GOST-art	0	Not_GOST-art	0
0.5	0.25	1	Not_GOST-art	0	Not_GOST-art	0
0.5	1.17	1	Not_GOST-art	0	Not_GOST-art	0
0.86	1.21	1	Not_GOST-art	0	Not_GOST-art	0
0.5	0.17	1	Not_GOST-art	0	Not_GOST-art	0
0.25	0.13	1	Not_GOST-art	0	Not_GOST-art	0
0.67	0.33	1	Not_GOST-art	0	Not_GOST-art	0
0.67	0	1	Not_GOST-art	0	Not_GOST-art	0
0.75	0.25	1	Not_GOST-art	0	Not_GOST-art	0
0.67	0.67	1	Not_GOST-art	0	Not_GOST-art	0
5	6.6	1	GOST-art-all	1	GOST-art-Y-al	1
5	6.6	1	GOST-art-all	1	GOST-art-Y-al	1
5	6.6	1	GOST-art-all	1	GOST-art-Y-al	1
5	6.6	1	GOST-art-all	1	GOST-art-Y-al	1
5	6.6	1	GOST-art-all	1	GOST-art-Y-al	1
0.22	0.11	1	Not_GOST-art	0	Not_GOST-art	0
0.83	7.33	1	Not_GOST-art	0	Not_GOST-art	0
0.83	7.33	1	Not_GOST-art	0	Not_GOST-art	0
0.5	0	1	Not_GOST-art	0	Not_GOST-art	0
1	0.5	1	Not_GOST-art	0	Not_GOST-art	0
1	0.5	1	Not_GOST-art	0	Not_GOST-art	0
1.5	2	1	Not_GOST-art	0	Not_GOST-art	0
2	0.67	1	Not_GOST-art	0	GOST-art-Y-al	1
2	0.67	1	Not_GOST-art	0	GOST-art-Y-al	1
1.29	0.14	1	Not_GOST-art	0	Not_GOST-art	0
1.29	2.86	1	GOST-art-all	1	Not_GOST-art	0
1.33	0.67	1	Not_GOST-art	0	Not_GOST-art	0
1.33	0.67	1	Not_GOST-art	0	Not_GOST-art	0
1.75	4	1	Not_GOST-art	0	GOST-art-Y-al	1
1.75	4	1	Not_GOST-art	0	GOST-art-Y-al	1
0.5	0.5	1	Not_GOST-art	0	Not_GOST-art	0
0.45	0.73	1	Not_GOST-art	0	Not_GOST-art	0

0.45	0.73	1	Not_GOST-art	0	Not_GOST-art	0
0.43	2.14	1	Not_GOST-art	0	Not_GOST-art	0
0.43	0.29	1	Not_GOST-art	0	Not_GOST-art	0
1	0	1	Not_GOST-art	0	Not_GOST-art	0
1	0	1	Not_GOST-art	0	Not_GOST-art	0
2	2	1	Not_GOST-art	0	GOST-art-Y-al	1
2	2	1	Not_GOST-art	0	GOST-art-Y-al	1
2	5.14	1	GOST-art-all	1	GOST-art-Y-al	1
2	5.14	1	GOST-art-all	1	GOST-art-Y-al	1
1.67	1	1	Not_GOST-art	0	GOST-art-Y-al	1
1.67	1	1	Not_GOST-art	0	GOST-art-Y-al	1
0.18	0.91	1	Not_GOST-art	0	Not_GOST-art	0
0.67	1.33	1	Not_GOST-art	0	Not_GOST-art	0
0.67	1.33	1	Not_GOST-art	0	Not_GOST-art	0
0.63	0.63	1	Not_GOST-art	0	Not_GOST-art	0
0.56	0.5	1	Not_GOST-art	0	Not_GOST-art	0
1.5	7	1	Not_GOST-art	0	Not_GOST-art	0
0.67	0.33	1	Not_GOST-art	0	Not_GOST-art	0
0.67	0.33	1	Not_GOST-art	0	Not_GOST-art	0
2	3	1	Not_GOST-art	0	GOST-art-Y-al	1
2	3	1	Not_GOST-art	0	GOST-art-Y-al	1
0.67	0	1	Not_GOST-art	0	Not_GOST-art	0
0.5	3	1	Not_GOST-art	0	Not_GOST-art	0
1	3.33	1	Not_GOST-art	0	Not_GOST-art	0
0.5	0.25	1	Not_GOST-art	0	Not_GOST-art	0
0.5	0.5	1	Not_GOST-art	0	Not_GOST-art	0
0.5	0.25	1	Not_GOST-art	0	Not_GOST-art	0
0.6	1.6	1	Not_GOST-art	0	Not_GOST-art	0
3.55	4.09	1	GOST-art-all	1	GOST-art-Y-al	1
3.55	4.09	1	GOST-art-all	1	GOST-art-Y-al	1
0.6	6.6	1	Not_GOST-art	0	Not_GOST-art	0
0.6	6.6	1	Not_GOST-art	0	Not_GOST-art	0
1.67	1.33	1	Not_GOST-art	0	GOST-art-Y-al	1
1	1	1	Not_GOST-art	0	Not_GOST-art	0
1	1	1	Not_GOST-art	0	Not_GOST-art	0
0.75	4.75	1	Not_GOST-art	0	Not_GOST-art	0
0.75	4.75	1	Not_GOST-art	0	Not_GOST-art	0
0.29	0.29	1	Not_GOST-art	0	Not_GOST-art	0
0.38	0.23	1	Not_GOST-art	0	Not_GOST-art	0
1.17	1.17	1	Not_GOST-art	0	Not_GOST-art	0
0.24	0.29	1	Not_GOST-art	0	Not_GOST-art	0
1	0.75	1	Not_GOST-art	0	Not_GOST-art	0
0.33	0	1	Not_GOST-art	0	Not_GOST-art	0
0.83	1.67	1	Not_GOST-art	0	Not_GOST-art	0
2	6.5	1	Not_GOST-art	0	GOST-art-Y-al	1
2	6.5	1	Not_GOST-art	0	GOST-art-Y-al	1
0.75	1	1	Not_GOST-art	0	Not_GOST-art	0

0.83	0.67	1	Not_GOST-art	0	Not_GOST-art	0
0.6	2.2	1	Not_GOST-art	0	Not_GOST-art	0
0.6	2.2	1	Not_GOST-art	0	Not_GOST-art	0
1	0	1	Not_GOST-art	0	Not_GOST-art	0
1	0	1	Not_GOST-art	0	Not_GOST-art	0
0.67	0	1	Not_GOST-art	0	Not_GOST-art	0
1	2.5	1	Not_GOST-art	0	Not_GOST-art	0
1	2.5	1	Not_GOST-art	0	Not_GOST-art	0
0.4	1	1	Not_GOST-art	0	Not_GOST-art	0
0.5	0.56	1	Not_GOST-art	0	Not_GOST-art	0
0.54	5.08	1	Not_GOST-art	0	Not_GOST-art	0
1	1.29	1	GOST-art-all	1	Not_GOST-art	0
1.25	0.75	1	Not_GOST-art	0	Not_GOST-art	0
1.25	0.75	1	Not_GOST-art	0	Not_GOST-art	0
1.25	0.75	1	Not_GOST-art	0	Not_GOST-art	0
1	0.33	1	Not_GOST-art	0	Not_GOST-art	0
1	0.33	1	Not_GOST-art	0	Not_GOST-art	0
0.67	4.33	1	Not_GOST-art	0	Not_GOST-art	0
0.67	4.33	1	Not_GOST-art	0	Not_GOST-art	0
0.33	0.44	1	Not_GOST-art	0	Not_GOST-art	0
0.67	0.33	1	Not_GOST-art	0	Not_GOST-art	0
0.67	1.33	1	Not_GOST-art	0	Not_GOST-art	0
0.67	1.33	1	Not_GOST-art	0	Not_GOST-art	0
0.67	1.33	1	Not_GOST-art	0	Not_GOST-art	0
2.29	3.29	1	GOST-art-all	1	GOST-art-Y-al	1
0.5	0.25	1	Not_GOST-art	0	Not_GOST-art	0
0.5	0.25	1	Not_GOST-art	0	Not_GOST-art	0
1	2	1	Not_GOST-art	0	Not_GOST-art	0
1	2	1	Not_GOST-art	0	Not_GOST-art	0
1	2	1	Not_GOST-art	0	Not_GOST-art	0
1	2	1	Not_GOST-art	0	Not_GOST-art	0
0.67	2	1	Not_GOST-art	0	Not_GOST-art	0
0.67	0	1	Not_GOST-art	0	Not_GOST-art	0
0.67	0	1	Not_GOST-art	0	Not_GOST-art	0
1.21	1.43	1	GOST-art-all	1	Not_GOST-art	0
1.21	1.43	1	GOST-art-all	1	Not_GOST-art	0
0.86	0.43	1	Not_GOST-art	0	Not_GOST-art	0
0.43	0.79	1	Not_GOST-art	0	Not_GOST-art	0
0.43	0.79	1	Not_GOST-art	0	Not_GOST-art	0
0.5	1	1	Not_GOST-art	0	Not_GOST-art	0
1	5.5	1	Not_GOST-art	0	Not_GOST-art	0
1	5.5	1	Not_GOST-art	0	Not_GOST-art	0
1	7	1	Not_GOST-art	0	Not_GOST-art	0
0.61	1.17	1	Not_GOST-art	0	Not_GOST-art	0
1	3.4	1	Not_GOST-art	0	Not_GOST-art	0
1	3.4	1	Not_GOST-art	0	Not_GOST-art	0
2.4	3.2	1	Not_GOST-art	0	GOST-art-Y-al	1

2	4.5	1	Not_GOST-art	0	GOST-art-Y-al	1
2	4.5	1	Not_GOST-art	0	GOST-art-Y-al	1
2	4.5	1	Not_GOST-art	0	GOST-art-Y-al	1
0.88	0.13	1	Not_GOST-art	0	Not_GOST-art	0
0.88	0.13	1	Not_GOST-art	0	Not_GOST-art	0
1.33	1.83	1	Not_GOST-art	0	Not_GOST-art	0
1.5	0.5	1	Not_GOST-art	0	Not_GOST-art	0
1.5	0.5	1	Not_GOST-art	0	Not_GOST-art	0
1.5	0.5	1	Not_GOST-art	0	Not_GOST-art	0
1	3	1	Not_GOST-art	0	Not_GOST-art	0
0.59	2.41	1	Not_GOST-art	0	Not_GOST-art	0
0.75	0.25	1	Not_GOST-art	0	Not_GOST-art	0
1.25	1	1	Not_GOST-art	0	Not_GOST-art	0
1.25	1	1	Not_GOST-art	0	Not_GOST-art	0
0.57	1	1	Not_GOST-art	0	Not_GOST-art	0
0.6	0.33	1	Not_GOST-art	0	Not_GOST-art	0
1.29	4.14	1	Not_GOST-art	0	Not_GOST-art	0
1.25	0.5	1	Not_GOST-art	0	Not_GOST-art	0
1	1.5	1	Not_GOST-art	0	Not_GOST-art	0
1	0.67	1	Not_GOST-art	0	Not_GOST-art	0
1	0.67	1	Not_GOST-art	0	Not_GOST-art	0
1.11	0.33	1	Not_GOST-art	0	Not_GOST-art	0
1.11	0.33	1	Not_GOST-art	0	Not_GOST-art	0
1	0	1	Not_GOST-art	0	Not_GOST-art	0
1	0	1	Not_GOST-art	0	Not_GOST-art	0
0.38	0.69	1	Not_GOST-art	0	Not_GOST-art	0
0.67	2.33	1	Not_GOST-art	0	Not_GOST-art	0
0.67	0.5	1	Not_GOST-art	0	Not_GOST-art	0
0.67	0.5	1	Not_GOST-art	0	Not_GOST-art	0
2.5	17	1	Not_GOST-art	0	GOST-art-Y-al	1
2.5	17	1	Not_GOST-art	0	GOST-art-Y-al	1
0.5	0	1	Not_GOST-art	0	Not_GOST-art	0
0.5	0	1	Not_GOST-art	0	Not_GOST-art	0
2	2	1	Not_GOST-art	0	GOST-art-Y-al	1
2	2	1	Not_GOST-art	0	GOST-art-Y-al	1
1.33	4.67	1	Not_GOST-art	0	Not_GOST-art	0
1.33	4.67	1	Not_GOST-art	0	Not_GOST-art	0
1.33	4.67	1	Not_GOST-art	0	Not_GOST-art	0
0.5	0.38	1	Not_GOST-art	0	Not_GOST-art	0
0.5	0.17	1	Not_GOST-art	0	Not_GOST-art	0
0.67	0	1	Not_GOST-art	0	Not_GOST-art	0
0.67	0	1	Not_GOST-art	0	Not_GOST-art	0
1.53	1.53	1	GOST-art-all	1	Not_GOST-art	0
1	1.33	1	Not_GOST-art	0	Not_GOST-art	0
1	1.33	1	Not_GOST-art	0	Not_GOST-art	0
1	1.33	1	Not_GOST-art	0	Not_GOST-art	0
1.25	2.75	1	Not_GOST-art	0	Not_GOST-art	0

1.25	3	1	Not_GOST-art	0	Not_GOST-art	0
0.33	0	1	Not_GOST-art	0	Not_GOST-art	0
0.57	0.57	1	Not_GOST-art	0	Not_GOST-art	0
1.17	0.58	1	GOST-art-all	1	Not_GOST-art	0
1.17	0.58	1	GOST-art-all	1	Not_GOST-art	0
0.5	1.75	1	Not_GOST-art	0	Not_GOST-art	0
1.38	1.08	1	GOST-art-all	1	Not_GOST-art	0
0.75	0.08	1	Not_GOST-art	0	Not_GOST-art	0
0.67	0.67	1	Not_GOST-art	0	Not_GOST-art	0
0.67	0.67	1	Not_GOST-art	0	Not_GOST-art	0
0.67	1.08	1	Not_GOST-art	0	Not_GOST-art	0
0.88	1.38	1	Not_GOST-art	0	Not_GOST-art	0
0.88	1.38	1	Not_GOST-art	0	Not_GOST-art	0
0.88	1.38	1	Not_GOST-art	0	Not_GOST-art	0
1	2.25	1	Not_GOST-art	0	Not_GOST-art	0
0.67	1	1	Not_GOST-art	0	Not_GOST-art	0
0.67	1	1	Not_GOST-art	0	Not_GOST-art	0
0.44	0.44	1	Not_GOST-art	0	Not_GOST-art	0
0.38	0.38	1	Not_GOST-art	0	Not_GOST-art	0
0.33	0.67	1	Not_GOST-art	0	Not_GOST-art	0
0.67	0.67	1	Not_GOST-art	0	Not_GOST-art	0
0.67	0.67	1	Not_GOST-art	0	Not_GOST-art	0
0.67	2	1	Not_GOST-art	0	Not_GOST-art	0
0.75	0	1	Not_GOST-art	0	Not_GOST-art	0
0.54	1	1	Not_GOST-art	0	Not_GOST-art	0
0.75	0.75	1	Not_GOST-art	0	Not_GOST-art	0
0.9	1.9	1	Not_GOST-art	0	Not_GOST-art	0
1.2	1.6	1	Not_GOST-art	0	Not_GOST-art	0
0.75	1.5	1	Not_GOST-art	0	Not_GOST-art	0
0.78	1.56	1	Not_GOST-art	0	Not_GOST-art	0
0.5	2.25	1	Not_GOST-art	0	Not_GOST-art	0
0.4	0.8	1	Not_GOST-art	0	Not_GOST-art	0
0.76	1.06	1	GOST-art-all	1	Not_GOST-art	0
1.67	3.33	1	Not_GOST-art	0	GOST-art-Y-al	1
1.67	3.33	1	Not_GOST-art	0	GOST-art-Y-al	1
0.86	0.86	1	Not_GOST-art	0	Not_GOST-art	0
0.86	0.86	1	Not_GOST-art	0	Not_GOST-art	0
0.71	0.86	1	Not_GOST-art	0	Not_GOST-art	0
0.71	0.86	1	Not_GOST-art	0	Not_GOST-art	0
0.67	0.33	1	Not_GOST-art	0	Not_GOST-art	0
0.67	0.33	1	Not_GOST-art	0	Not_GOST-art	0
1.5	4.25	1	Not_GOST-art	0	Not_GOST-art	0
1.5	4.25	1	Not_GOST-art	0	Not_GOST-art	0
1	0.6	1	Not_GOST-art	0	Not_GOST-art	0
1	0.6	1	Not_GOST-art	0	Not_GOST-art	0
1	1.57	1	Not_GOST-art	0	Not_GOST-art	0
1	0	1	Not_GOST-art	0	Not_GOST-art	0

0.67	1.67	1	Not_GOST-art	0	Not_GOST-art	0
0.75	1.25	1	Not_GOST-art	0	Not_GOST-art	0
0.67	0	1	Not_GOST-art	0	Not_GOST-art	0
0.56	0	1	Not_GOST-art	0	Not_GOST-art	0
5.33	6.56	1	GOST-art-all	1	GOST-art-Y-al	1
0.55	2.55	1	Not_GOST-art	0	Not_GOST-art	0
0.25	0.38	1	Not_GOST-art	0	Not_GOST-art	0
0.5	1.5	1	Not_GOST-art	0	Not_GOST-art	0
0.5	1.5	1	Not_GOST-art	0	Not_GOST-art	0
0.5	2	1	Not_GOST-art	0	Not_GOST-art	0
0.5	2	1	Not_GOST-art	0	Not_GOST-art	0
1	5	1	Not_GOST-art	0	Not_GOST-art	0
1	5	1	Not_GOST-art	0	Not_GOST-art	0
1	3	1	Not_GOST-art	0	Not_GOST-art	0
1	3	1	Not_GOST-art	0	Not_GOST-art	0
1	3	1	Not_GOST-art	0	Not_GOST-art	0
0.75	0.75	1	Not_GOST-art	0	Not_GOST-art	0
0.75	0.75	1	Not_GOST-art	0	Not_GOST-art	0
0.56	1.75	1	Not_GOST-art	0	Not_GOST-art	0
1	0.75	1	Not_GOST-art	0	Not_GOST-art	0
1	0.75	1	Not_GOST-art	0	Not_GOST-art	0
1.5	0.5	1	Not_GOST-art	0	Not_GOST-art	0
1.5	0.5	1	Not_GOST-art	0	Not_GOST-art	0
0.29	0	1	Not_GOST-art	0	Not_GOST-art	0
0.38	0.56	1	Not_GOST-art	0	Not_GOST-art	0
0.8	2.4	1	Not_GOST-art	0	Not_GOST-art	0
0.8	2.4	1	Not_GOST-art	0	Not_GOST-art	0
1.13	1.88	1	Not_GOST-art	0	Not_GOST-art	0
1.13	1.88	1	Not_GOST-art	0	Not_GOST-art	0
1.2	2.8	1	Not_GOST-art	0	Not_GOST-art	0
1.2	2.8	1	Not_GOST-art	0	Not_GOST-art	0
0.19	0.81	1	Not_GOST-art	0	Not_GOST-art	0
0.5	1.5	1	Not_GOST-art	0	Not_GOST-art	0
0.5	1.5	1	Not_GOST-art	0	Not_GOST-art	0
0.5	1.17	1	Not_GOST-art	0	Not_GOST-art	0
1.5	2.25	1	Not_GOST-art	0	Not_GOST-art	0
0.3	0.8	1	Not_GOST-art	0	Not_GOST-art	0
0.75	4	1	Not_GOST-art	0	Not_GOST-art	0
0.75	4	1	Not_GOST-art	0	Not_GOST-art	0
1	3	1	Not_GOST-art	0	Not_GOST-art	0
1	3	1	Not_GOST-art	0	Not_GOST-art	0
1.57	2.29	1	Not_GOST-art	0	GOST-art-Y-al	1
0.21	0.14	1	Not_GOST-art	0	Not_GOST-art	0
0.5	0.25	1	Not_GOST-art	0	Not_GOST-art	0
0.67	0	1	Not_GOST-art	0	Not_GOST-art	0
0.67	0	1	Not_GOST-art	0	Not_GOST-art	0
0.5	1.5	1	Not_GOST-art	0	Not_GOST-art	0

0.5	1.5	1	Not_GOST-art	0	Not_GOST-art	0
0.5	1.5	1	Not_GOST-art	0	Not_GOST-art	0
1.67	2	1	Not_GOST-art	0	GOST-art-Y-al	1
1	1.5	1	Not_GOST-art	0	Not_GOST-art	0
1	1.5	1	Not_GOST-art	0	Not_GOST-art	0
1	2.25	1	Not_GOST-art	0	Not_GOST-art	0
1.17	2.25	1	GOST-art-all	1	Not_GOST-art	0
1.17	2.25	1	GOST-art-all	1	Not_GOST-art	0
0.71	0.86	1	Not_GOST-art	0	Not_GOST-art	0
0.71	0.86	1	Not_GOST-art	0	Not_GOST-art	0
2	5	1	Not_GOST-art	0	GOST-art-Y-al	1
2	5	1	Not_GOST-art	0	GOST-art-Y-al	1
1.17	4.17	1	Not_GOST-art	0	Not_GOST-art	0
1	2	1	Not_GOST-art	0	Not_GOST-art	0
0.21	0.07	1	Not_GOST-art	0	Not_GOST-art	0
1.4	2.4	1	Not_GOST-art	0	Not_GOST-art	0
1.4	2.4	1	Not_GOST-art	0	Not_GOST-art	0
1.2	1.1	1	Not_GOST-art	0	Not_GOST-art	0
1.2	1.1	1	Not_GOST-art	0	Not_GOST-art	0
2	7	1	Not_GOST-art	0	GOST-art-Y-al	1
2	7	1	Not_GOST-art	0	GOST-art-Y-al	1
2	7	1	Not_GOST-art	0	GOST-art-Y-al	1
0.36	0.57	1	Not_GOST-art	0	Not_GOST-art	0
0.43	0	1	Not_GOST-art	0	Not_GOST-art	0
0.3	0.2	1	Not_GOST-art	0	Not_GOST-art	0
1	2.8	1	Not_GOST-art	0	Not_GOST-art	0
1	2.8	1	Not_GOST-art	0	Not_GOST-art	0
1	4	1	Not_GOST-art	0	Not_GOST-art	0
0.33	0.33	1	Not_GOST-art	0	Not_GOST-art	0
0.63	1.13	1	Not_GOST-art	0	Not_GOST-art	0
0.63	1.13	1	Not_GOST-art	0	Not_GOST-art	0
0.86	1.29	1	Not_GOST-art	0	Not_GOST-art	0
0.86	1.29	1	Not_GOST-art	0	Not_GOST-art	0
0.5	0.5	1	Not_GOST-art	0	Not_GOST-art	0
0.3	0.4	1	Not_GOST-art	0	Not_GOST-art	0
0.3	0.4	1	Not_GOST-art	0	Not_GOST-art	0
2	3	1	Not_GOST-art	0	GOST-art-Y-al	1
2	3	1	Not_GOST-art	0	GOST-art-Y-al	1
2	3	1	Not_GOST-art	0	GOST-art-Y-al	1
0.57	2.57	1	Not_GOST-art	0	Not_GOST-art	0
1	1	1	Not_GOST-art	0	Not_GOST-art	0
1	1	1	Not_GOST-art	0	Not_GOST-art	0
1	1	1	Not_GOST-art	0	Not_GOST-art	0
1.2	1.6	1	GOST-art-all	1	Not_GOST-art	0
1.27	2	1	GOST-art-all	1	Not_GOST-art	0
1.27	2	1	GOST-art-all	1	Not_GOST-art	0
1.27	2	1	GOST-art-all	1	Not_GOST-art	0

0.67	0.33	1	Not_GOST-art	0	Not_GOST-art	0
0.67	0.33	1	Not_GOST-art	0	Not_GOST-art	0
0.8	0.4	1	Not_GOST-art	0	Not_GOST-art	0
1	0	1	Not_GOST-art	0	Not_GOST-art	0
1.5	0	1	Not_GOST-art	0	Not_GOST-art	0
1.5	0	1	Not_GOST-art	0	Not_GOST-art	0
1.5	1.33	1	Not_GOST-art	0	Not_GOST-art	0
1.1	2.4	1	Not_GOST-art	0	Not_GOST-art	0
0.5	1.5	1	Not_GOST-art	0	Not_GOST-art	0
0.6	1.4	1	Not_GOST-art	0	Not_GOST-art	0
0.6	1.4	1	Not_GOST-art	0	Not_GOST-art	0
0.75	5.25	1	Not_GOST-art	0	Not_GOST-art	0
0.75	5.25	1	Not_GOST-art	0	Not_GOST-art	0
3	5	1	Not_GOST-art	0	GOST-art-Y-al	1
3	5	1	Not_GOST-art	0	GOST-art-Y-al	1
3	5	1	Not_GOST-art	0	GOST-art-Y-al	1
1	6.33	1	Not_GOST-art	0	Not_GOST-art	0
1	6.33	1	Not_GOST-art	0	Not_GOST-art	0
0.67	2	1	Not_GOST-art	0	Not_GOST-art	0
0.67	2	1	Not_GOST-art	0	Not_GOST-art	0
1.2	1.6	1	Not_GOST-art	0	Not_GOST-art	0
0.67	1	1	Not_GOST-art	0	Not_GOST-art	0
0.67	1	1	Not_GOST-art	0	Not_GOST-art	0
0.25	1.19	1	Not_GOST-art	0	Not_GOST-art	0
1	0.5	1	Not_GOST-art	0	Not_GOST-art	0
0.5	2.25	1	Not_GOST-art	0	Not_GOST-art	0
1	1.38	1	Not_GOST-art	0	Not_GOST-art	0
1	1.38	1	Not_GOST-art	0	Not_GOST-art	0
0.71	2.14	1	Not_GOST-art	0	Not_GOST-art	0
0.35	1.24	1	Not_GOST-art	0	Not_GOST-art	0
1	0	1	Not_GOST-art	0	Not_GOST-art	0
1	0	1	Not_GOST-art	0	Not_GOST-art	0
1	0	1	Not_GOST-art	0	Not_GOST-art	0
0.75	0.75	1	Not_GOST-art	0	Not_GOST-art	0
0.43	3	1	Not_GOST-art	0	Not_GOST-art	0
0.5	0.25	1	Not_GOST-art	0	Not_GOST-art	0
0.67	2	1	Not_GOST-art	0	Not_GOST-art	0
0.8	2	1	Not_GOST-art	0	Not_GOST-art	0
0.8	2	1	Not_GOST-art	0	Not_GOST-art	0
1	1.5	1	Not_GOST-art	0	Not_GOST-art	0
1	1.5	1	Not_GOST-art	0	Not_GOST-art	0
1	1.33	1	Not_GOST-art	0	Not_GOST-art	0
2.29	4.29	1	GOST-art-all	1	GOST-art-Y-al	1
1	0.33	1	Not_GOST-art	0	Not_GOST-art	0
3.7	6.4	1	GOST-art-all	1	GOST-art-Y-al	1
0.9	1.1	1	Not_GOST-art	0	Not_GOST-art	0
0.38	0	1	Not_GOST-art	0	Not_GOST-art	0

0.38	0	1	Not_GOST-art	0	Not_GOST-art	0
0.27	0.45	1	Not_GOST-art	0	Not_GOST-art	0
0.27	0.45	1	Not_GOST-art	0	Not_GOST-art	0
1.6	3.4	1	Not_GOST-art	0	GOST-art-Y-al	1
1	1.2	1	Not_GOST-art	0	Not_GOST-art	0
1	1.2	1	Not_GOST-art	0	Not_GOST-art	0
2.46	4.92	1	GOST-art-all	1	GOST-art-Y-al	1
0.83	1.67	1	Not_GOST-art	0	Not_GOST-art	0
0.83	1.67	1	Not_GOST-art	0	Not_GOST-art	0
0.83	1.67	1	Not_GOST-art	0	Not_GOST-art	0
0.15	1.31	1	Not_GOST-art	0	Not_GOST-art	0
2.67	5.33	1	Not_GOST-art	0	GOST-art-Y-al	1
2.67	5.33	1	Not_GOST-art	0	GOST-art-Y-al	1
0.6	1.6	1	Not_GOST-art	0	Not_GOST-art	0
1	0.5	1	Not_GOST-art	0	Not_GOST-art	0
0.67	4	1	Not_GOST-art	0	Not_GOST-art	0
0.67	4	1	Not_GOST-art	0	Not_GOST-art	0
0.63	0.75	1	Not_GOST-art	0	Not_GOST-art	0
0.4	0	1	Not_GOST-art	0	Not_GOST-art	0
0.67	0.67	1	Not_GOST-art	0	Not_GOST-art	0
0.67	0.67	1	Not_GOST-art	0	Not_GOST-art	0
1.33	13.33	1	Not_GOST-art	0	Not_GOST-art	0
1.33	13.33	1	Not_GOST-art	0	Not_GOST-art	0
1.33	13.33	1	Not_GOST-art	0	Not_GOST-art	0
0.75	0.25	1	Not_GOST-art	0	Not_GOST-art	0
1	0.5	1	Not_GOST-art	0	Not_GOST-art	0
1.33	0.67	1	Not_GOST-art	0	Not_GOST-art	0
2.33	0.33	1	Not_GOST-art	0	GOST-art-Y-al	1
2.33	0.33	1	Not_GOST-art	0	GOST-art-Y-al	1
2.33	0.33	1	Not_GOST-art	0	GOST-art-Y-al	1
1.75	4	1	Not_GOST-art	0	GOST-art-Y-al	1
0.6	0	1	Not_GOST-art	0	Not_GOST-art	0
0.6	0	1	Not_GOST-art	0	Not_GOST-art	0
1	0.75	1	Not_GOST-art	0	Not_GOST-art	0
1	0.75	1	Not_GOST-art	0	Not_GOST-art	0
0.67	0	1	Not_GOST-art	0	Not_GOST-art	0
0.67	0	1	Not_GOST-art	0	Not_GOST-art	0
0.67	1	1	Not_GOST-art	0	Not_GOST-art	0
0.67	1	1	Not_GOST-art	0	Not_GOST-art	0
0.83	2.17	1	Not_GOST-art	0	Not_GOST-art	0
2	2.29	1	GOST-art-all	1	GOST-art-Y-al	1
1	1.5	1	Not_GOST-art	0	Not_GOST-art	0
1	1.5	1	Not_GOST-art	0	Not_GOST-art	0
0.33	2.5	1	Not_GOST-art	0	Not_GOST-art	0
0.5	0.75	1	Not_GOST-art	0	Not_GOST-art	0
2	3.67	1	GOST-art-all	1	GOST-art-Y-al	1
1	0.33	1	Not_GOST-art	0	Not_GOST-art	0

1	0.33	1	Not_GOST-art	0	Not_GOST-art	0
1	0	1	Not_GOST-art	0	Not_GOST-art	0
1	0	1	Not_GOST-art	0	Not_GOST-art	0
0.75	2.75	1	Not_GOST-art	0	Not_GOST-art	0
0.6	0.6	1	Not_GOST-art	0	Not_GOST-art	0
0.57	0.86	1	Not_GOST-art	0	Not_GOST-art	0
0.57	0.86	1	Not_GOST-art	0	Not_GOST-art	0
2.8	9.5	1	GOST-art-all	1	GOST-art-Y-al	1
2.8	9.5	1	GOST-art-all	1	GOST-art-Y-al	1
2.8	9.5	1	GOST-art-all	1	GOST-art-Y-al	1
3.57	10.14	1	GOST-art-all	1	GOST-art-Y-al	1
3.57	10.14	1	GOST-art-all	1	GOST-art-Y-al	1
3.57	10.14	1	GOST-art-all	1	GOST-art-Y-al	1
3.57	10.14	1	GOST-art-all	1	GOST-art-Y-al	1
0.25	0.25	1	Not_GOST-art	0	Not_GOST-art	0
0.6	0.47	1	Not_GOST-art	0	Not_GOST-art	0
1.2	1.9	1	Not_GOST-art	0	Not_GOST-art	0
1.2	1.9	1	Not_GOST-art	0	Not_GOST-art	0
1	3.67	1	Not_GOST-art	0	Not_GOST-art	0
0.5	1	1	Not_GOST-art	0	Not_GOST-art	0
0.86	1.57	1	Not_GOST-art	0	Not_GOST-art	0
0.29	1	1	Not_GOST-art	0	Not_GOST-art	0
1.5	2	1	Not_GOST-art	0	Not_GOST-art	0
0.67	0.33	1	Not_GOST-art	0	Not_GOST-art	0
0.5	0.75	1	Not_GOST-art	0	Not_GOST-art	0
1.75	2.75	1	Not_GOST-art	0	GOST-art-Y-al	1
1.75	2.75	1	Not_GOST-art	0	GOST-art-Y-al	1
0.8	0.2	1	Not_GOST-art	0	Not_GOST-art	0
1.2	1.2	1	Not_GOST-art	0	Not_GOST-art	0
1	0.75	1	Not_GOST-art	0	Not_GOST-art	0
1	0.75	1	Not_GOST-art	0	Not_GOST-art	0
1	0.75	1	Not_GOST-art	0	Not_GOST-art	0
1.6	2.6	1	Not_GOST-art	0	GOST-art-Y-al	1
1.6	2.6	1	Not_GOST-art	0	GOST-art-Y-al	1
1	4	1	Not_GOST-art	0	Not_GOST-art	0
0.33	0.11	1	Not_GOST-art	0	Not_GOST-art	0
0.6	0	1	Not_GOST-art	0	Not_GOST-art	0
0.67	4	1	Not_GOST-art	0	Not_GOST-art	0
0.67	4	1	Not_GOST-art	0	Not_GOST-art	0
0.5	0.5	1	Not_GOST-art	0	Not_GOST-art	0
1	3	1	Not_GOST-art	0	Not_GOST-art	0
4	4.83	1	GOST-art-all	1	GOST-art-Y-al	1
0.9	0.5	1	Not_GOST-art	0	Not_GOST-art	0
2.5	7.75	1	Not_GOST-art	0	GOST-art-Y-al	1
2.5	7.75	1	Not_GOST-art	0	GOST-art-Y-al	1
2.5	7.75	1	Not_GOST-art	0	GOST-art-Y-al	1
1.43	2.29	1	Not_GOST-art	0	Not_GOST-art	0

0.67	0.67	1	Not_GOST-art	0	Not_GOST-art	0
0.67	0.67	1	Not_GOST-art	0	Not_GOST-art	0
0.63	1.5	1	Not_GOST-art	0	Not_GOST-art	0
1.25	3.5	1	Not_GOST-art	0	Not_GOST-art	0
0.4	0.2	1	Not_GOST-art	0	Not_GOST-art	0
1	0.5	1	Not_GOST-art	0	Not_GOST-art	0
1	0.5	1	Not_GOST-art	0	Not_GOST-art	0
1.14	9.71	1	Not_GOST-art	0	Not_GOST-art	0
1.14	9.71	1	Not_GOST-art	0	Not_GOST-art	0
0.4	0.2	1	Not_GOST-art	0	Not_GOST-art	0
0.11	0.06	1	Not_GOST-art	0	Not_GOST-art	0
0.67	0.67	1	Not_GOST-art	0	Not_GOST-art	0
1.75	6.38	1	GOST-art-all	1	GOST-art-Y-al	1
0.67	1	1	Not_GOST-art	0	Not_GOST-art	0
1.75	4	1	Not_GOST-art	0	GOST-art-Y-al	1
1.75	4	1	Not_GOST-art	0	GOST-art-Y-al	1
1.2	1.8	1	Not_GOST-art	0	Not_GOST-art	0
1.2	1.8	1	Not_GOST-art	0	Not_GOST-art	0
1	1.67	1	Not_GOST-art	0	Not_GOST-art	0
0.8	1.4	1	Not_GOST-art	0	Not_GOST-art	0
0.67	2.33	1	Not_GOST-art	0	Not_GOST-art	0
0.4	0.2	1	Not_GOST-art	0	Not_GOST-art	0
1	2.5	1	Not_GOST-art	0	Not_GOST-art	0
1	2.5	1	Not_GOST-art	0	Not_GOST-art	0
2.88	11.38	1	GOST-art-all	1	GOST-art-Y-al	1
0.57	0.57	1	Not_GOST-art	0	Not_GOST-art	0
0.33	0.33	1	Not_GOST-art	0	Not_GOST-art	0
0.4	0.4	1	Not_GOST-art	0	Not_GOST-art	0
0.67	0	1	Not_GOST-art	0	Not_GOST-art	0
1.75	4.5	1	Not_GOST-art	0	GOST-art-Y-al	1
1	0	1	Not_GOST-art	0	Not_GOST-art	0
0.5	0.17	1	Not_GOST-art	0	Not_GOST-art	0
0.3	0.4	1	Not_GOST-art	0	Not_GOST-art	0
1	2	1	Not_GOST-art	0	Not_GOST-art	0
1	2	1	Not_GOST-art	0	Not_GOST-art	0
0.5	0	1	Not_GOST-art	0	Not_GOST-art	0
0.67	0	1	Not_GOST-art	0	Not_GOST-art	0
0.67	0	1	Not_GOST-art	0	Not_GOST-art	0
0.63	0.56	1	Not_GOST-art	0	Not_GOST-art	0
0.69	1.31	1	Not_GOST-art	0	Not_GOST-art	0
0.38	0	1	Not_GOST-art	0	Not_GOST-art	0
0.18	0.18	1	Not_GOST-art	0	Not_GOST-art	0
1.88	20.63	1	GOST-art-all	1	GOST-art-Y-al	1
3.4	43	1	GOST-art-all	1	GOST-art-Y-al	1
3.4	43	1	GOST-art-all	1	GOST-art-Y-al	1
3.4	43	1	GOST-art-all	1	GOST-art-Y-al	1
3.4	43	1	GOST-art-all	1	GOST-art-Y-al	1

0.75	2	1	Not_GOST-art	0	Not_GOST-art	0
0.75	2	1	Not_GOST-art	0	Not_GOST-art	0
0.83	1.5	1	Not_GOST-art	0	Not_GOST-art	0
0.67	0.56	1	Not_GOST-art	0	Not_GOST-art	0
1.33	1	1	Not_GOST-art	0	Not_GOST-art	0
1.33	1	1	Not_GOST-art	0	Not_GOST-art	0
0.29	0.57	1	Not_GOST-art	0	Not_GOST-art	0
1.25	2.13	1	Not_GOST-art	0	Not_GOST-art	0
0.38	0.13	1	Not_GOST-art	0	Not_GOST-art	0
0.75	3.25	1	Not_GOST-art	0	Not_GOST-art	0
0.75	3.25	1	Not_GOST-art	0	Not_GOST-art	0
2.33	6.67	1	Not_GOST-art	0	GOST-art-Y-al	1
0.57	0.29	1	Not_GOST-art	0	Not_GOST-art	0
0.57	0.29	1	Not_GOST-art	0	Not_GOST-art	0
1.33	0.67	1	Not_GOST-art	0	Not_GOST-art	0
1.33	0.67	1	Not_GOST-art	0	Not_GOST-art	0
1.33	0.67	1	Not_GOST-art	0	Not_GOST-art	0
1.33	0.67	1	Not_GOST-art	0	Not_GOST-art	0
0.29	0.43	1	Not_GOST-art	0	Not_GOST-art	0
0.86	1.79	1	Not_GOST-art	0	Not_GOST-art	0
0.86	1.79	1	Not_GOST-art	0	Not_GOST-art	0
1	0	1	Not_GOST-art	0	Not_GOST-art	0
0.89	1.22	1	Not_GOST-art	0	Not_GOST-art	0
0.89	1.22	1	Not_GOST-art	0	Not_GOST-art	0
0.89	1.22	1	Not_GOST-art	0	Not_GOST-art	0
0.71	0.43	1	Not_GOST-art	0	Not_GOST-art	0
0.71	0.43	1	Not_GOST-art	0	Not_GOST-art	0
1	0.56	1	Not_GOST-art	0	Not_GOST-art	0
0.67	1.56	1	Not_GOST-art	0	Not_GOST-art	0
0.56	0.78	1	Not_GOST-art	0	Not_GOST-art	0
0.5	0.44	1	Not_GOST-art	0	Not_GOST-art	0
1.3	3.4	1	GOST-art-all	1	Not_GOST-art	0
1	0.75	1	Not_GOST-art	0	Not_GOST-art	0
1	0.75	1	Not_GOST-art	0	Not_GOST-art	0
2	13.5	1	Not_GOST-art	0	GOST-art-Y-al	1
2	13.5	1	Not_GOST-art	0	GOST-art-Y-al	1
0.4	0	1	Not_GOST-art	0	Not_GOST-art	0
1.15	0.62	1	GOST-art-all	1	Not_GOST-art	0
0.4	1.6	1	Not_GOST-art	0	Not_GOST-art	0
1	2.33	1	Not_GOST-art	0	Not_GOST-art	0
1	2.33	1	Not_GOST-art	0	Not_GOST-art	0
1	2.33	1	Not_GOST-art	0	Not_GOST-art	0
0.63	2.75	1	Not_GOST-art	0	Not_GOST-art	0
0.63	2.75	1	Not_GOST-art	0	Not_GOST-art	0
1	4	1	Not_GOST-art	0	Not_GOST-art	0
1	1.8	1	Not_GOST-art	0	Not_GOST-art	0
1	2	1	Not_GOST-art	0	Not_GOST-art	0

1	2	1	Not_GOST-art	0	Not_GOST-art	0
0.67	1	1	Not_GOST-art	0	Not_GOST-art	0
0.67	0.67	1	Not_GOST-art	0	Not_GOST-art	0
0.67	0.67	1	Not_GOST-art	0	Not_GOST-art	0
1.43	3.86	1	Not_GOST-art	0	Not_GOST-art	0
0.71	0.14	1	Not_GOST-art	0	Not_GOST-art	0
0.6	1	1	Not_GOST-art	0	Not_GOST-art	0
0.6	1	1	Not_GOST-art	0	Not_GOST-art	0
2.29	5.43	1	GOST-art-all	1	GOST-art-Y-al	1
2.29	5.43	1	GOST-art-all	1	GOST-art-Y-al	1
2.29	5.43	1	GOST-art-all	1	GOST-art-Y-al	1
2.29	5.43	1	GOST-art-all	1	GOST-art-Y-al	1
2.29	5.43	1	GOST-art-all	1	GOST-art-Y-al	1
0.33	1.67	1	Not_GOST-art	0	Not_GOST-art	0
0.57	0.43	1	Not_GOST-art	0	Not_GOST-art	0
0.67	0.67	1	Not_GOST-art	0	Not_GOST-art	0
1.2	3.6	1	Not_GOST-art	0	Not_GOST-art	0
0.75	2	1	Not_GOST-art	0	Not_GOST-art	0
0.75	2	1	Not_GOST-art	0	Not_GOST-art	0
0.67	0	1	Not_GOST-art	0	Not_GOST-art	0
0.67	0	1	Not_GOST-art	0	Not_GOST-art	0
0.86	3.71	1	Not_GOST-art	0	Not_GOST-art	0
0.86	3.71	1	Not_GOST-art	0	Not_GOST-art	0
0.4	0.4	1	Not_GOST-art	0	Not_GOST-art	0
0.8	0.4	1	Not_GOST-art	0	Not_GOST-art	0
0.29	0.14	1	Not_GOST-art	0	Not_GOST-art	0
0.83	0.67	1	Not_GOST-art	0	Not_GOST-art	0
1.17	1.33	1	Not_GOST-art	0	Not_GOST-art	0
1.17	1.33	1	Not_GOST-art	0	Not_GOST-art	0
1.25	1	1	Not_GOST-art	0	Not_GOST-art	0
1.25	1	1	Not_GOST-art	0	Not_GOST-art	0
1	0	1	Not_GOST-art	0	Not_GOST-art	0
1	0	1	Not_GOST-art	0	Not_GOST-art	0
1	4	1	Not_GOST-art	0	Not_GOST-art	0
1	4	1	Not_GOST-art	0	Not_GOST-art	0
1	0.5	1	Not_GOST-art	0	Not_GOST-art	0
0.67	0	1	Not_GOST-art	0	Not_GOST-art	0
0.67	0	1	Not_GOST-art	0	Not_GOST-art	0
1.29	2.53	1	GOST-art-all	1	Not_GOST-art	0
1.25	5.5	1	Not_GOST-art	0	Not_GOST-art	0
1.33	2.17	1	Not_GOST-art	0	Not_GOST-art	0
1.33	2.17	1	Not_GOST-art	0	Not_GOST-art	0
1.33	2.17	1	Not_GOST-art	0	Not_GOST-art	0
0.5	0.7	1	Not_GOST-art	0	Not_GOST-art	0
0.5	1.33	1	Not_GOST-art	0	Not_GOST-art	0
0.6	1.6	1	Not_GOST-art	0	Not_GOST-art	0
1	0.5	1	Not_GOST-art	0	Not_GOST-art	0

1	0.5	1	Not_GOST-art	0	Not_GOST-art	0
1.6	10.8	1	Not_GOST-art	0	GOST-art-Y-al	1
0.4	2	1	Not_GOST-art	0	Not_GOST-art	0
0.6	1	1	Not_GOST-art	0	Not_GOST-art	0
1	1.33	1	Not_GOST-art	0	Not_GOST-art	0
1	1.33	1	Not_GOST-art	0	Not_GOST-art	0
0.27	0.4	1	Not_GOST-art	0	Not_GOST-art	0
0.75	0.75	1	Not_GOST-art	0	Not_GOST-art	0
0.75	0.75	1	Not_GOST-art	0	Not_GOST-art	0
0.67	0	1	Not_GOST-art	0	Not_GOST-art	0
0.67	0	1	Not_GOST-art	0	Not_GOST-art	0
1	0.57	1	Not_GOST-art	0	Not_GOST-art	0
1	0.57	1	Not_GOST-art	0	Not_GOST-art	0
1	1	1	Not_GOST-art	0	Not_GOST-art	0
0.67	0	1	Not_GOST-art	0	Not_GOST-art	0
0.67	0	1	Not_GOST-art	0	Not_GOST-art	0
0.22	0.11	1	Not_GOST-art	0	Not_GOST-art	0
0.67	1.33	1	Not_GOST-art	0	Not_GOST-art	0
1	1.33	1	Not_GOST-art	0	Not_GOST-art	0
1	1.33	1	Not_GOST-art	0	Not_GOST-art	0
0.82	2.45	1	Not_GOST-art	0	Not_GOST-art	0
0.5	1.13	1	Not_GOST-art	0	Not_GOST-art	0
0.73	1.55	1	Not_GOST-art	0	Not_GOST-art	0
0.33	0	1	Not_GOST-art	0	Not_GOST-art	0
0.75	1.38	1	Not_GOST-art	0	Not_GOST-art	0
1.33	4	1	Not_GOST-art	0	Not_GOST-art	0
0.75	1	1	Not_GOST-art	0	Not_GOST-art	0
1.33	10.67	1	Not_GOST-art	0	Not_GOST-art	0
1.33	10.67	1	Not_GOST-art	0	Not_GOST-art	0
0.38	0	1	Not_GOST-art	0	Not_GOST-art	0
0.79	0.71	1	Not_GOST-art	0	Not_GOST-art	0
0.5	0.29	1	Not_GOST-art	0	Not_GOST-art	0
0.67	0.33	1	Not_GOST-art	0	Not_GOST-art	0
0.67	1.33	1	Not_GOST-art	0	Not_GOST-art	0
0.75	2.42	1	Not_GOST-art	0	Not_GOST-art	0
0.6	2.3	1	Not_GOST-art	0	Not_GOST-art	0
0.6	2.3	1	Not_GOST-art	0	Not_GOST-art	0
1.33	2.33	1	Not_GOST-art	0	Not_GOST-art	0
1.33	2.33	1	Not_GOST-art	0	Not_GOST-art	0
1.33	2.33	1	Not_GOST-art	0	Not_GOST-art	0
2.29	4.71	1	GOST-art-all	1	GOST-art-Y-al	1
2.29	4.71	1	GOST-art-all	1	GOST-art-Y-al	1
2.29	4.71	1	GOST-art-all	1	GOST-art-Y-al	1
0.38	0.13	1	Not_GOST-art	0	Not_GOST-art	0
0.67	0	1	Not_GOST-art	0	Not_GOST-art	0
0.67	0	1	Not_GOST-art	0	Not_GOST-art	0
0.83	0.33	1	Not_GOST-art	0	Not_GOST-art	0

0.83	0.33	1	Not_GOST-art	0	Not_GOST-art	0
1	1	1	Not_GOST-art	0	Not_GOST-art	0
1	1	1	Not_GOST-art	0	Not_GOST-art	0
1	1	1	Not_GOST-art	0	Not_GOST-art	0
1.38	3.25	1	Not_GOST-art	0	Not_GOST-art	0
1.38	3.25	1	Not_GOST-art	0	Not_GOST-art	0
1	0.5	1	Not_GOST-art	0	Not_GOST-art	0
0.5	0.13	1	Not_GOST-art	0	Not_GOST-art	0
0.5	0.13	1	Not_GOST-art	0	Not_GOST-art	0
0.67	0.33	1	Not_GOST-art	0	Not_GOST-art	0
0.67	0.33	1	Not_GOST-art	0	Not_GOST-art	0
0.6	1.3	1	Not_GOST-art	0	Not_GOST-art	0
1	0	1	Not_GOST-art	0	Not_GOST-art	0
1	0	1	Not_GOST-art	0	Not_GOST-art	0
1	0	1	Not_GOST-art	0	Not_GOST-art	0
1	0	1	Not_GOST-art	0	Not_GOST-art	0
0.75	2	1	Not_GOST-art	0	Not_GOST-art	0
0.75	2	1	Not_GOST-art	0	Not_GOST-art	0
2	7.67	1	Not_GOST-art	0	GOST-art-Y-al	1
2	7.67	1	Not_GOST-art	0	GOST-art-Y-al	1
2	7.67	1	Not_GOST-art	0	GOST-art-Y-al	1
0.56	0.22	1	Not_GOST-art	0	Not_GOST-art	0
0.62	0.38	1	Not_GOST-art	0	Not_GOST-art	0
2	4.5	1	Not_GOST-art	0	GOST-art-Y-al	1
2	4.5	1	Not_GOST-art	0	GOST-art-Y-al	1
2	2.17	1	Not_GOST-art	0	GOST-art-Y-al	1
2	2.17	1	Not_GOST-art	0	GOST-art-Y-al	1
2	5.2	1	Not_GOST-art	0	GOST-art-Y-al	1
0.8	2.4	1	Not_GOST-art	0	Not_GOST-art	0
0.8	2.4	1	Not_GOST-art	0	Not_GOST-art	0
1	1.67	1	Not_GOST-art	0	Not_GOST-art	0
1	1.67	1	Not_GOST-art	0	Not_GOST-art	0
2.67	4.67	1	Not_GOST-art	0	GOST-art-Y-al	1
2.67	4.67	1	Not_GOST-art	0	GOST-art-Y-al	1
2.67	4.67	1	Not_GOST-art	0	GOST-art-Y-al	1
1.44	1.78	1	GOST-art-all	1	Not_GOST-art	0
1.44	1.78	1	GOST-art-all	1	Not_GOST-art	0
0.5	3.75	1	Not_GOST-art	0	Not_GOST-art	0
1	2.38	1	Not_GOST-art	0	Not_GOST-art	0
1	2.38	1	Not_GOST-art	0	Not_GOST-art	0
2	0.5	1	Not_GOST-art	0	GOST-art-Y-al	1
0.57	0.57	1	Not_GOST-art	0	Not_GOST-art	0
1.5	2	1	GOST-art-all	1	Not_GOST-art	0
4.38	4.25	1	GOST-art-all	1	GOST-art-Y-al	1
1.75	2.25	1	Not_GOST-art	0	GOST-art-Y-al	1
1.75	2.25	1	Not_GOST-art	0	GOST-art-Y-al	1
1.75	2.25	1	Not_GOST-art	0	GOST-art-Y-al	1

0.4	0	1	Not_GOST-art	0	Not_GOST-art	0
0.91	1.82	1	Not_GOST-art	0	Not_GOST-art	0
0.8	0.8	1	Not_GOST-art	0	Not_GOST-art	0
0.56	2.89	1	Not_GOST-art	0	Not_GOST-art	0
0.67	3.33	1	Not_GOST-art	0	Not_GOST-art	0
0.67	3.33	1	Not_GOST-art	0	Not_GOST-art	0
0.22	0.44	1	Not_GOST-art	0	Not_GOST-art	0
0.22	0	1	Not_GOST-art	0	Not_GOST-art	0
0.33	0.17	1	Not_GOST-art	0	Not_GOST-art	0
1	3.5	1	Not_GOST-art	0	Not_GOST-art	0
0.4	0	1	Not_GOST-art	0	Not_GOST-art	0
0.29	0.71	1	Not_GOST-art	0	Not_GOST-art	0
4.5	18.67	1	GOST-art-all	1	GOST-art-Y-al	1
4.5	18.67	1	GOST-art-all	1	GOST-art-Y-al	1
4.5	18.67	1	GOST-art-all	1	GOST-art-Y-al	1
1.5	1	1	Not_GOST-art	0	Not_GOST-art	0
1.5	1	1	Not_GOST-art	0	Not_GOST-art	0
1.5	1	1	Not_GOST-art	0	Not_GOST-art	0
1	0.25	1	Not_GOST-art	0	Not_GOST-art	0
1.33	1.33	1	Not_GOST-art	0	Not_GOST-art	0
0.71	0.57	1	Not_GOST-art	0	Not_GOST-art	0
0.71	0.57	1	Not_GOST-art	0	Not_GOST-art	0
0.71	0.57	1	Not_GOST-art	0	Not_GOST-art	0
0.5	0.75	1	Not_GOST-art	0	Not_GOST-art	0
1.14	2.71	1	Not_GOST-art	0	Not_GOST-art	0
0.6	0.4	1	Not_GOST-art	0	Not_GOST-art	0
0.6	0.4	1	Not_GOST-art	0	Not_GOST-art	0
0.64	0.07	1	Not_GOST-art	0	Not_GOST-art	0
0.64	0.07	1	Not_GOST-art	0	Not_GOST-art	0
0.83	1.17	1	Not_GOST-art	0	Not_GOST-art	0
0.83	1.17	1	Not_GOST-art	0	Not_GOST-art	0
0.83	1.17	1	Not_GOST-art	0	Not_GOST-art	0
0.33	0.83	1	Not_GOST-art	0	Not_GOST-art	0
0.33	0.83	1	Not_GOST-art	0	Not_GOST-art	0
1	5.5	1	Not_GOST-art	0	Not_GOST-art	0
1	5.5	1	Not_GOST-art	0	Not_GOST-art	0
1	5.5	1	Not_GOST-art	0	Not_GOST-art	0
0.33	1.67	1	Not_GOST-art	0	Not_GOST-art	0
0.75	2.5	1	Not_GOST-art	0	Not_GOST-art	0
0.39	0.89	1	Not_GOST-art	0	Not_GOST-art	0
0.39	0.89	1	Not_GOST-art	0	Not_GOST-art	0
0.39	0.89	1	Not_GOST-art	0	Not_GOST-art	0
0.36	0.18	1	Not_GOST-art	0	Not_GOST-art	0
0.36	0.18	1	Not_GOST-art	0	Not_GOST-art	0
0.83	1.83	1	Not_GOST-art	0	Not_GOST-art	0
0.83	1.83	1	Not_GOST-art	0	Not_GOST-art	0
0.38	1.25	1	Not_GOST-art	0	Not_GOST-art	0

0.38	1.25	1	Not_GOST-art	0	Not_GOST-art	0
0.86	1.29	1	Not_GOST-art	0	Not_GOST-art	0
1.4	1.8	1	Not_GOST-art	0	Not_GOST-art	0
1.4	1.8	1	Not_GOST-art	0	Not_GOST-art	0
0.8	0.3	1	Not_GOST-art	0	Not_GOST-art	0
0.8	0.3	1	Not_GOST-art	0	Not_GOST-art	0
0.8	0.3	1	Not_GOST-art	0	Not_GOST-art	0
3.57	6.36	1	GOST-art-all	1	GOST-art-Y-al	1
3.57	6.36	1	GOST-art-all	1	GOST-art-Y-al	1
1	0.33	1	Not_GOST-art	0	Not_GOST-art	0
0.53	1.41	1	Not_GOST-art	0	Not_GOST-art	0
2	13.5	1	Not_GOST-art	0	GOST-art-Y-al	1
0.5	0.25	1	Not_GOST-art	0	Not_GOST-art	0
1	0.67	1	Not_GOST-art	0	Not_GOST-art	0
1	0.67	1	Not_GOST-art	0	Not_GOST-art	0
1	0.67	1	Not_GOST-art	0	Not_GOST-art	0
2.33	7	1	GOST-art-all	1	GOST-art-Y-al	1
2.33	7	1	GOST-art-all	1	GOST-art-Y-al	1
3.33	32	1	Not_GOST-art	0	GOST-art-Y-al	1
3.33	32	1	Not_GOST-art	0	GOST-art-Y-al	1
3.33	32	1	Not_GOST-art	0	GOST-art-Y-al	1
3.33	32	1	Not_GOST-art	0	GOST-art-Y-al	1
0.25	0	1	Not_GOST-art	0	Not_GOST-art	0
0.83	1	1	Not_GOST-art	0	Not_GOST-art	0
0.83	1	1	Not_GOST-art	0	Not_GOST-art	0
0.43	0.29	1	Not_GOST-art	0	Not_GOST-art	0
0.83	5.67	1	Not_GOST-art	0	Not_GOST-art	0
0.83	5.67	1	Not_GOST-art	0	Not_GOST-art	0
0.83	5.67	1	Not_GOST-art	0	Not_GOST-art	0
0.6	0	1	Not_GOST-art	0	Not_GOST-art	0
0.5	0.75	1	Not_GOST-art	0	Not_GOST-art	0
0.44	0	1	Not_GOST-art	0	Not_GOST-art	0

nop_gostcit	gostcitall	gostcitall_b	gostcityall	gostcityall_b	nop_gostart_	gostartall_2	gostartall_b_
	Not_GOST-cit	0	Not_GOST-cit	0			
	Not_GOST-cit	0	Not_GOST-cit	0			
	Not_GOST-cit	0	Not_GOST-cit	0			
	Not_GOST-cit	0	Not_GOST-cit	0			
	Not_GOST-cit	0	Not_GOST-cit	0			
	Not_GOST-cit	0	Not_GOST-cit	0			
	Not_GOST-cit	0	Not_GOST-cit	0			
	Not_GOST-cit	0	Not_GOST-cit	0			
	Not_GOST-cit	0	Not_GOST-cit	0			
	Not_GOST-cit	0	Not_GOST-cit	0			
	Not_GOST-cit	0	Not_GOST-cit	0			
	Not_GOST-cit	0	Not_GOST-cit	0			
	Not_GOST-cit	0	Not_GOST-cit	0			
	Not_GOST-cit	0	Not_GOST-cit	0			
	Not_GOST-cit	0	Not_GOST-cit	0			
	Not_GOST-cit	0	Not_GOST-cit	0			
	Not_GOST-cit	0	Not_GOST-cit	0			
	Not_GOST-cit	0	Not_GOST-cit	0			
	Not_GOST-cit	0	Not_GOST-cit	0			
	Not_GOST-cit	0	Not_GOST-cit	0			
	Not_GOST-cit	0	Not_GOST-cit	0			
	Not_GOST-cit	0	Not_GOST-cit	0			
	Not_GOST-cit	0	Not_GOST-cit	0			
	Not_GOST-cit	0	Not_GOST-cit	0			
	Not_GOST-cit	0	Not_GOST-cit	0			
	Not_GOST-cit	0	Not_GOST-cit	0			
	Not_GOST-cit	0	Not_GOST-cit	0			
	Not_GOST-cit	0	Not_GOST-cit	0			
	Not_GOST-cit	0	Not_GOST-cit	0			
	Not_GOST-cit	0	Not_GOST-cit	0			
	Not_GOST-cit	0	Not_GOST-cit	0			
	GOST-cit-all	1	Not_GOST-cit	0			
	Not_GOST-cit	0	Not_GOST-cit	0			
	Not_GOST-cit	0	Not_GOST-cit	0			
	Not_GOST-cit	0	Not_GOST-cit	0			
	Not_GOST-cit	0	Not_GOST-cit	0			
	Not_GOST-cit	0	Not_GOST-cit	0			
	Not_GOST-cit	0	Not_GOST-cit	0			
	Not_GOST-cit	0	Not_GOST-cit	0			
	Not_GOST-cit	0	Not_GOST-cit	0			
	Not_GOST-cit	0	Not_GOST-cit	0			
	Not_GOST-cit	0	Not_GOST-cit	0			
	Not_GOST-cit	0	Not_GOST-cit	0			
	Not_GOST-cit	0	Not_GOST-cit	0			
	Not_GOST-cit	0	Not_GOST-cit	0			
	Not_GOST-cit	0	Not_GOST-cit	0			

Not_GOST-cit	0	Not_GOST-cit	0
GOST-cit-all	1	Not_GOST-cit	0
Not_GOST-cit	0	Not_GOST-cit	0
Not_GOST-cit	0	Not_GOST-cit	0
Not_GOST-cit	0	Not_GOST-cit	0
Not_GOST-cit	0	Not_GOST-cit	0
Not_GOST-cit	0	Not_GOST-cit	0
GOST-cit-all	1	Not_GOST-cit	0
Not_GOST-cit	0	GOST-cit-Y-all	1
Not_GOST-cit	0	Not_GOST-cit	0
Not_GOST-cit	0	Not_GOST-cit	0
Not_GOST-cit	0	Not_GOST-cit	0
GOST-cit-all	1	Not_GOST-cit	0
Not_GOST-cit	0	Not_GOST-cit	0
Not_GOST-cit	0	Not_GOST-cit	0
Not_GOST-cit	0	Not_GOST-cit	0
Not_GOST-cit	0	Not_GOST-cit	0
Not_GOST-cit	0	Not_GOST-cit	0
Not_GOST-cit	0	Not_GOST-cit	0
Not_GOST-cit	0	Not_GOST-cit	0
Not_GOST-cit	0	Not_GOST-cit	0
Not_GOST-cit	0	Not_GOST-cit	0
Not_GOST-cit	0	Not_GOST-cit	0
Not_GOST-cit	0	Not_GOST-cit	0
Not_GOST-cit	0	Not_GOST-cit	0
Not_GOST-cit	0	Not_GOST-cit	0
Not_GOST-cit	0	Not_GOST-cit	0
Not_GOST-cit	0	Not_GOST-cit	0
Not_GOST-cit	0	Not_GOST-cit	0
Not_GOST-cit	0	Not_GOST-cit	0
Not_GOST-cit	0	Not_GOST-cit	0
Not_GOST-cit	0	Not_GOST-cit	0
Not_GOST-cit	0	Not_GOST-cit	0
Not_GOST-cit	0	Not_GOST-cit	0
Not_GOST-cit	0	Not_GOST-cit	0
Not_GOST-cit	0	Not_GOST-cit	0
Not_GOST-cit	0	Not_GOST-cit	0
Not_GOST-cit	0	Not_GOST-cit	0
Not_GOST-cit	0	Not_GOST-cit	0
Not_GOST-cit	0	Not_GOST-cit	0
Not_GOST-cit	0	Not_GOST-cit	0
Not_GOST-cit	0	Not_GOST-cit	0
Not_GOST-cit	0	Not_GOST-cit	0
Not_GOST-cit	0	Not_GOST-cit	0
Not_GOST-cit	0	Not_GOST-cit	0
Not_GOST-cit	0	Not_GOST-cit	0
Not_GOST-cit	0	Not_GOST-cit	0

Not_GOST-cit	0	Not_GOST-cit	0
Not_GOST-cit	0	Not_GOST-cit	0
Not_GOST-cit	0	Not_GOST-cit	0
Not_GOST-cit	0	Not_GOST-cit	0
Not_GOST-cit	0	Not_GOST-cit	0
Not_GOST-cit	0	Not_GOST-cit	0
Not_GOST-cit	0	Not_GOST-cit	0
Not_GOST-cit	0	Not_GOST-cit	0
Not_GOST-cit	0	Not_GOST-cit	0
Not_GOST-cit	0	Not_GOST-cit	0
Not_GOST-cit	0	Not_GOST-cit	0
Not_GOST-cit	0	Not_GOST-cit	0
Not_GOST-cit	0	Not_GOST-cit	0
Not_GOST-cit	0	Not_GOST-cit	0
Not_GOST-cit	0	Not_GOST-cit	0
Not_GOST-cit	0	Not_GOST-cit	0
Not_GOST-cit	0	Not_GOST-cit	0
Not_GOST-cit	0	Not_GOST-cit	0
Not_GOST-cit	0	Not_GOST-cit	0
Not_GOST-cit	0	Not_GOST-cit	0
Not_GOST-cit	0	Not_GOST-cit	0
Not_GOST-cit	0	Not_GOST-cit	0
Not_GOST-cit	0	Not_GOST-cit	0
Not_GOST-cit	0	Not_GOST-cit	0
Not_GOST-cit	0	Not_GOST-cit	0
Not_GOST-cit	0	Not_GOST-cit	0
Not_GOST-cit	0	Not_GOST-cit	0
GOST-cit-all	1	GOST-cit-Y-all	1
GOST-cit-all	1	GOST-cit-Y-all	1
GOST-cit-all	1	Not_GOST-cit	0
GOST-cit-all	1	Not_GOST-cit	0
Not_GOST-cit	0	Not_GOST-cit	0
GOST-cit-all	1	GOST-cit-Y-all	1
GOST-cit-all	1	GOST-cit-Y-all	1
Not_GOST-cit	0	Not_GOST-cit	0
Not_GOST-cit	0	Not_GOST-cit	0
Not_GOST-cit	0	Not_GOST-cit	0
Not_GOST-cit	0	Not_GOST-cit	0
Not_GOST-cit	0	Not_GOST-cit	0
GOST-cit-all	1	GOST-cit-Y-all	1
Not_GOST-cit	0	Not_GOST-cit	0
Not_GOST-cit	0	Not_GOST-cit	0
Not_GOST-cit	0	Not_GOST-cit	0
Not_GOST-cit	0	Not_GOST-cit	0
GOST-cit-all	1	Not_GOST-cit	0
GOST-cit-all	1	Not_GOST-cit	0
Not_GOST-cit	0	Not_GOST-cit	0

Not_GOST-cit	0	Not_GOST-cit	0
Not_GOST-cit	0	Not_GOST-cit	0
Not_GOST-cit	0	Not_GOST-cit	0
Not_GOST-cit	0	Not_GOST-cit	0
Not_GOST-cit	0	Not_GOST-cit	0
Not_GOST-cit	0	Not_GOST-cit	0
Not_GOST-cit	0	Not_GOST-cit	0
Not_GOST-cit	0	Not_GOST-cit	0
Not_GOST-cit	0	Not_GOST-cit	0
Not_GOST-cit	0	Not_GOST-cit	0
Not_GOST-cit	0	Not_GOST-cit	0
Not_GOST-cit	0	GOST-cit-Y-all	1
Not_GOST-cit	0	GOST-cit-Y-all	1
Not_GOST-cit	0	GOST-cit-Y-all	1
Not_GOST-cit	0	Not_GOST-cit	0
Not_GOST-cit	0	Not_GOST-cit	0
Not_GOST-cit	0	GOST-cit-Y-all	1
Not_GOST-cit	0	Not_GOST-cit	0
Not_GOST-cit	0	Not_GOST-cit	0
Not_GOST-cit	0	Not_GOST-cit	0
Not_GOST-cit	0	Not_GOST-cit	0
Not_GOST-cit	0	Not_GOST-cit	0
Not_GOST-cit	0	Not_GOST-cit	0
Not_GOST-cit	0	Not_GOST-cit	0
Not_GOST-cit	0	Not_GOST-cit	0
Not_GOST-cit	0	Not_GOST-cit	0
Not_GOST-cit	0	Not_GOST-cit	0
Not_GOST-cit	0	Not_GOST-cit	0
Not_GOST-cit	0	Not_GOST-cit	0
Not_GOST-cit	0	Not_GOST-cit	0
Not_GOST-cit	0	Not_GOST-cit	0
Not_GOST-cit	0	Not_GOST-cit	0
Not_GOST-cit	0	GOST-cit-Y-all	1
Not_GOST-cit	0	Not_GOST-cit	0
Not_GOST-cit	0	Not_GOST-cit	0
Not_GOST-cit	0	Not_GOST-cit	0
Not_GOST-cit	0	Not_GOST-cit	0
Not_GOST-cit	0	Not_GOST-cit	0
GOST-cit-all	1	GOST-cit-Y-all	1
Not_GOST-cit	0	Not_GOST-cit	0
Not_GOST-cit	0	Not_GOST-cit	0
Not_GOST-cit	0	Not_GOST-cit	0
Not_GOST-cit	0	Not_GOST-cit	0
Not_GOST-cit	0	Not_GOST-cit	0
Not_GOST-cit	0	Not_GOST-cit	0
Not_GOST-cit	0	Not_GOST-cit	0
Not_GOST-cit	0	Not_GOST-cit	0

Not_GOST-cit	0	Not_GOST-cit	0
Not_GOST-cit	0	Not_GOST-cit	0
Not_GOST-cit	0	Not_GOST-cit	0
Not_GOST-cit	0	Not_GOST-cit	0
GOST-cit-all	1	GOST-cit-Y-all	1
GOST-cit-all	1	GOST-cit-Y-all	1
Not_GOST-cit	0	Not_GOST-cit	0
Not_GOST-cit	0	Not_GOST-cit	0
Not_GOST-cit	0	Not_GOST-cit	0
Not_GOST-cit	0	Not_GOST-cit	0
Not_GOST-cit	0	Not_GOST-cit	0
Not_GOST-cit	0	Not_GOST-cit	0
Not_GOST-cit	0	Not_GOST-cit	0
Not_GOST-cit	0	Not_GOST-cit	0
Not_GOST-cit	0	Not_GOST-cit	0
Not_GOST-cit	0	Not_GOST-cit	0
Not_GOST-cit	0	Not_GOST-cit	0
Not_GOST-cit	0	Not_GOST-cit	0
Not_GOST-cit	0	Not_GOST-cit	0
Not_GOST-cit	0	Not_GOST-cit	0
Not_GOST-cit	0	Not_GOST-cit	0
Not_GOST-cit	0	Not_GOST-cit	0
Not_GOST-cit	0	Not_GOST-cit	0
Not_GOST-cit	0	Not_GOST-cit	0
Not_GOST-cit	0	Not_GOST-cit	0
Not_GOST-cit	0	Not_GOST-cit	0
Not_GOST-cit	0	Not_GOST-cit	0
Not_GOST-cit	0	Not_GOST-cit	0
Not_GOST-cit	0	Not_GOST-cit	0
Not_GOST-cit	0	Not_GOST-cit	0
Not_GOST-cit	0	Not_GOST-cit	0
Not_GOST-cit	0	Not_GOST-cit	0
Not_GOST-cit	0	Not_GOST-cit	0
Not_GOST-cit	0	Not_GOST-cit	0
Not_GOST-cit	0	Not_GOST-cit	0
GOST-cit-all	1	GOST-cit-Y-all	1
Not_GOST-cit	0	Not_GOST-cit	0
Not_GOST-cit	0	Not_GOST-cit	0
Not_GOST-cit	0	Not_GOST-cit	0
Not_GOST-cit	0	GOST-cit-Y-all	1
Not_GOST-cit	0	GOST-cit-Y-all	1
Not_GOST-cit	0	GOST-cit-Y-all	1
Not_GOST-cit	0	GOST-cit-Y-all	1
Not_GOST-cit	0	Not_GOST-cit	0
Not_GOST-cit	0	Not_GOST-cit	0
Not_GOST-cit	0	Not_GOST-cit	0
Not_GOST-cit	0	Not_GOST-cit	0
Not_GOST-cit	0	Not_GOST-cit	0

Not_GOST-cit	0	Not_GOST-cit	0
Not_GOST-cit	0	Not_GOST-cit	0
Not_GOST-cit	0	Not_GOST-cit	0
Not_GOST-cit	0	Not_GOST-cit	0
Not_GOST-cit	0	Not_GOST-cit	0
Not_GOST-cit	0	Not_GOST-cit	0
Not_GOST-cit	0	Not_GOST-cit	0
Not_GOST-cit	0	Not_GOST-cit	0
Not_GOST-cit	0	Not_GOST-cit	0
Not_GOST-cit	0	Not_GOST-cit	0
Not_GOST-cit	0	Not_GOST-cit	0
Not_GOST-cit	0	Not_GOST-cit	0
Not_GOST-cit	0	Not_GOST-cit	0
Not_GOST-cit	0	Not_GOST-cit	0
Not_GOST-cit	0	Not_GOST-cit	0
Not_GOST-cit	0	Not_GOST-cit	0
Not_GOST-cit	0	Not_GOST-cit	0
Not_GOST-cit	0	Not_GOST-cit	0
Not_GOST-cit	0	Not_GOST-cit	0
Not_GOST-cit	0	Not_GOST-cit	0
Not_GOST-cit	0	Not_GOST-cit	0
GOST-cit-all	1	GOST-cit-Y-all	1
GOST-cit-all	1	GOST-cit-Y-all	1
GOST-cit-all	1	GOST-cit-Y-all	1
Not_GOST-cit	0	Not_GOST-cit	0
Not_GOST-cit	0	Not_GOST-cit	0
Not_GOST-cit	0	Not_GOST-cit	0
Not_GOST-cit	0	Not_GOST-cit	0
Not_GOST-cit	0	Not_GOST-cit	0
Not_GOST-cit	0	Not_GOST-cit	0
GOST-cit-all	1	GOST-cit-Y-all	1
GOST-cit-all	1	GOST-cit-Y-all	1
GOST-cit-all	1	GOST-cit-Y-all	1
Not_GOST-cit	0	Not_GOST-cit	0
GOST-cit-all	1	Not_GOST-cit	0
Not_GOST-cit	0	Not_GOST-cit	0
Not_GOST-cit	0	Not_GOST-cit	0
Not_GOST-cit	0	Not_GOST-cit	0
Not_GOST-cit	0	Not_GOST-cit	0
Not_GOST-cit	0	Not_GOST-cit	0
Not_GOST-cit	0	Not_GOST-cit	0
Not_GOST-cit	0	Not_GOST-cit	0
Not_GOST-cit	0	Not_GOST-cit	0
Not_GOST-cit	0	Not_GOST-cit	0
Not_GOST-cit	0	Not_GOST-cit	0
Not_GOST-cit	0	Not_GOST-cit	0
Not_GOST-cit	0	Not_GOST-cit	0

Not_GOST-cit	0	Not_GOST-cit	0
Not_GOST-cit	0	Not_GOST-cit	0
Not_GOST-cit	0	Not_GOST-cit	0
Not_GOST-cit	0	Not_GOST-cit	0
Not_GOST-cit	0	Not_GOST-cit	0
Not_GOST-cit	0	Not_GOST-cit	0
Not_GOST-cit	0	Not_GOST-cit	0
Not_GOST-cit	0	Not_GOST-cit	0
Not_GOST-cit	0	Not_GOST-cit	0
Not_GOST-cit	0	Not_GOST-cit	0
Not_GOST-cit	0	Not_GOST-cit	0
Not_GOST-cit	0	Not_GOST-cit	0
Not_GOST-cit	0	Not_GOST-cit	0
Not_GOST-cit	0	Not_GOST-cit	0
Not_GOST-cit	0	Not_GOST-cit	0
Not_GOST-cit	0	Not_GOST-cit	0
Not_GOST-cit	0	Not_GOST-cit	0
Not_GOST-cit	0	Not_GOST-cit	0
Not_GOST-cit	0	Not_GOST-cit	0
Not_GOST-cit	0	Not_GOST-cit	0
Not_GOST-cit	0	Not_GOST-cit	0
Not_GOST-cit	0	Not_GOST-cit	0
Not_GOST-cit	0	Not_GOST-cit	0
Not_GOST-cit	0	Not_GOST-cit	0
Not_GOST-cit	0	Not_GOST-cit	0
Not_GOST-cit	0	Not_GOST-cit	0
Not_GOST-cit	0	Not_GOST-cit	0
Not_GOST-cit	0	Not_GOST-cit	0
Not_GOST-cit	0	Not_GOST-cit	0
Not_GOST-cit	0	Not_GOST-cit	0
Not_GOST-cit	0	Not_GOST-cit	0
Not_GOST-cit	0	Not_GOST-cit	0
Not_GOST-cit	0	Not_GOST-cit	0
Not_GOST-cit	0	Not_GOST-cit	0
Not_GOST-cit	0	Not_GOST-cit	0
Not_GOST-cit	0	Not_GOST-cit	0
Not_GOST-cit	0	Not_GOST-cit	0
Not_GOST-cit	0	Not_GOST-cit	0
Not_GOST-cit	0	Not_GOST-cit	0
Not_GOST-cit	0	Not_GOST-cit	0
Not_GOST-cit	0	Not_GOST-cit	0
Not_GOST-cit	0	Not_GOST-cit	0
Not_GOST-cit	0	Not_GOST-cit	0
Not_GOST-cit	0	Not_GOST-cit	0
Not_GOST-cit	0	Not_GOST-cit	0
Not_GOST-cit	0	Not_GOST-cit	0
Not_GOST-cit	0	Not_GOST-cit	0

Not_GOST-cit	0	Not_GOST-cit	0
Not_GOST-cit	0	Not_GOST-cit	0
Not_GOST-cit	0	Not_GOST-cit	0
Not_GOST-cit	0	Not_GOST-cit	0
Not_GOST-cit	0	Not_GOST-cit	0
Not_GOST-cit	0	Not_GOST-cit	0
Not_GOST-cit	0	Not_GOST-cit	0
Not_GOST-cit	0	Not_GOST-cit	0
Not_GOST-cit	0	Not_GOST-cit	0
Not_GOST-cit	0	Not_GOST-cit	0
Not_GOST-cit	0	Not_GOST-cit	0
Not_GOST-cit	0	Not_GOST-cit	0
Not_GOST-cit	0	Not_GOST-cit	0
Not_GOST-cit	0	Not_GOST-cit	0
Not_GOST-cit	0	Not_GOST-cit	0
Not_GOST-cit	0	Not_GOST-cit	0
Not_GOST-cit	0	Not_GOST-cit	0
Not_GOST-cit	0	Not_GOST-cit	0
Not_GOST-cit	0	Not_GOST-cit	0
Not_GOST-cit	0	Not_GOST-cit	0
Not_GOST-cit	0	Not_GOST-cit	0
Not_GOST-cit	0	Not_GOST-cit	0
Not_GOST-cit	0	Not_GOST-cit	0
Not_GOST-cit	0	Not_GOST-cit	0
Not_GOST-cit	0	GOST-cit-Y-all	1
Not_GOST-cit	0	GOST-cit-Y-all	1
Not_GOST-cit	0	Not_GOST-cit	0
Not_GOST-cit	0	Not_GOST-cit	0
Not_GOST-cit	0	Not_GOST-cit	0
Not_GOST-cit	0	Not_GOST-cit	0
Not_GOST-cit	0	Not_GOST-cit	0
Not_GOST-cit	0	Not_GOST-cit	0
Not_GOST-cit	0	Not_GOST-cit	0
Not_GOST-cit	0	Not_GOST-cit	0
Not_GOST-cit	0	Not_GOST-cit	0
Not_GOST-cit	0	Not_GOST-cit	0
Not_GOST-cit	0	Not_GOST-cit	0
Not_GOST-cit	0	Not_GOST-cit	0
Not_GOST-cit	0	Not_GOST-cit	0
Not_GOST-cit	0	Not_GOST-cit	0
Not_GOST-cit	0	Not_GOST-cit	0
Not_GOST-cit	0	Not_GOST-cit	0
Not_GOST-cit	0	Not_GOST-cit	0
GOST-cit-all	1	Not_GOST-cit	0
Not_GOST-cit	0	GOST-cit-Y-all	1
Not_GOST-cit	0	Not_GOST-cit	0
Not_GOST-cit	0	Not_GOST-cit	0

Not_GOST-cit	0	Not_GOST-cit	0
Not_GOST-cit	0	Not_GOST-cit	0
Not_GOST-cit	0	Not_GOST-cit	0
Not_GOST-cit	0	Not_GOST-cit	0
Not_GOST-cit	0	Not_GOST-cit	0
Not_GOST-cit	0	Not_GOST-cit	0
Not_GOST-cit	0	Not_GOST-cit	0
Not_GOST-cit	0	GOST-cit-Y-all	1
Not_GOST-cit	0	GOST-cit-Y-all	1
Not_GOST-cit	0	Not_GOST-cit	0
Not_GOST-cit	0	Not_GOST-cit	0
Not_GOST-cit	0	Not_GOST-cit	0
Not_GOST-cit	0	Not_GOST-cit	0
Not_GOST-cit	0	Not_GOST-cit	0
Not_GOST-cit	0	Not_GOST-cit	0
Not_GOST-cit	0	Not_GOST-cit	0
Not_GOST-cit	0	Not_GOST-cit	0
Not_GOST-cit	0	Not_GOST-cit	0
Not_GOST-cit	0	Not_GOST-cit	0
Not_GOST-cit	0	Not_GOST-cit	0
Not_GOST-cit	0	Not_GOST-cit	0
Not_GOST-cit	0	Not_GOST-cit	0
Not_GOST-cit	0	Not_GOST-cit	0
Not_GOST-cit	0	Not_GOST-cit	0
Not_GOST-cit	0	Not_GOST-cit	0
Not_GOST-cit	0	Not_GOST-cit	0
Not_GOST-cit	0	Not_GOST-cit	0
Not_GOST-cit	0	Not_GOST-cit	0
Not_GOST-cit	0	Not_GOST-cit	0
Not_GOST-cit	0	Not_GOST-cit	0
Not_GOST-cit	0	Not_GOST-cit	0
Not_GOST-cit	0	Not_GOST-cit	0
Not_GOST-cit	0	Not_GOST-cit	0
Not_GOST-cit	0	Not_GOST-cit	0
Not_GOST-cit	0	Not_GOST-cit	0
Not_GOST-cit	0	Not_GOST-cit	0
Not_GOST-cit	0	Not_GOST-cit	0
Not_GOST-cit	0	Not_GOST-cit	0
Not_GOST-cit	0	Not_GOST-cit	0
Not_GOST-cit	0	Not_GOST-cit	0
Not_GOST-cit	0	Not_GOST-cit	0
Not_GOST-cit	0	Not_GOST-cit	0
Not_GOST-cit	0	Not_GOST-cit	0
Not_GOST-cit	0	Not_GOST-cit	0
Not_GOST-cit	0	Not_GOST-cit	0
Not_GOST-cit	0	Not_GOST-cit	0
Not_GOST-cit	0	Not_GOST-cit	0

Not_GOST-cit	0	Not_GOST-cit	0
Not_GOST-cit	0	Not_GOST-cit	0
Not_GOST-cit	0	Not_GOST-cit	0
Not_GOST-cit	0	Not_GOST-cit	0
Not_GOST-cit	0	Not_GOST-cit	0
GOST-cit-all	1	Not_GOST-cit	0
GOST-cit-all	1	Not_GOST-cit	0
Not_GOST-cit	0	Not_GOST-cit	0
Not_GOST-cit	0	Not_GOST-cit	0
Not_GOST-cit	0	Not_GOST-cit	0
Not_GOST-cit	0	Not_GOST-cit	0
GOST-cit-all	1	GOST-cit-Y-all	1
GOST-cit-all	1	GOST-cit-Y-all	1
Not_GOST-cit	0	Not_GOST-cit	0
Not_GOST-cit	0	Not_GOST-cit	0
Not_GOST-cit	0	Not_GOST-cit	0
Not_GOST-cit	0	Not_GOST-cit	0
Not_GOST-cit	0	Not_GOST-cit	0
Not_GOST-cit	0	Not_GOST-cit	0
GOST-cit-all	1	GOST-cit-Y-all	1
GOST-cit-all	1	GOST-cit-Y-all	1
Not_GOST-cit	0	Not_GOST-cit	0
Not_GOST-cit	0	Not_GOST-cit	0
Not_GOST-cit	0	Not_GOST-cit	0
Not_GOST-cit	0	Not_GOST-cit	0
Not_GOST-cit	0	Not_GOST-cit	0
Not_GOST-cit	0	Not_GOST-cit	0
Not_GOST-cit	0	Not_GOST-cit	0
Not_GOST-cit	0	Not_GOST-cit	0
Not_GOST-cit	0	GOST-cit-Y-all	1
Not_GOST-cit	0	GOST-cit-Y-all	1
Not_GOST-cit	0	Not_GOST-cit	0
Not_GOST-cit	0	Not_GOST-cit	0
Not_GOST-cit	0	Not_GOST-cit	0
Not_GOST-cit	0	Not_GOST-cit	0
Not_GOST-cit	0	Not_GOST-cit	0
Not_GOST-cit	0	Not_GOST-cit	0
Not_GOST-cit	0	Not_GOST-cit	0
Not_GOST-cit	0	Not_GOST-cit	0
Not_GOST-cit	0	Not_GOST-cit	0
Not_GOST-cit	0	Not_GOST-cit	0
Not_GOST-cit	0	Not_GOST-cit	0
Not_GOST-cit	0	Not_GOST-cit	0
Not_GOST-cit	0	GOST-cit-Y-all	1
Not_GOST-cit	0	Not_GOST-cit	0
Not_GOST-cit	0	Not_GOST-cit	0
Not_GOST-cit	0	Not_GOST-cit	0

Not_GOST-cit	0	Not_GOST-cit	0
Not_GOST-cit	0	Not_GOST-cit	0
Not_GOST-cit	0	Not_GOST-cit	0
Not_GOST-cit	0	Not_GOST-cit	0
Not_GOST-cit	0	Not_GOST-cit	0
Not_GOST-cit	0	Not_GOST-cit	0
Not_GOST-cit	0	Not_GOST-cit	0
Not_GOST-cit	0	Not_GOST-cit	0
Not_GOST-cit	0	Not_GOST-cit	0
Not_GOST-cit	0	Not_GOST-cit	0
Not_GOST-cit	0	Not_GOST-cit	0
Not_GOST-cit	0	Not_GOST-cit	0
Not_GOST-cit	0	Not_GOST-cit	0
Not_GOST-cit	0	Not_GOST-cit	0
Not_GOST-cit	0	Not_GOST-cit	0
Not_GOST-cit	0	Not_GOST-cit	0
Not_GOST-cit	0	Not_GOST-cit	0
Not_GOST-cit	0	Not_GOST-cit	0
Not_GOST-cit	0	Not_GOST-cit	0
Not_GOST-cit	0	Not_GOST-cit	0
Not_GOST-cit	0	Not_GOST-cit	0
Not_GOST-cit	0	Not_GOST-cit	0
Not_GOST-cit	0	Not_GOST-cit	0
GOST-cit-all	1	GOST-cit-Y-all	1
GOST-cit-all	1	GOST-cit-Y-all	1
GOST-cit-all	1	GOST-cit-Y-all	1
Not_GOST-cit	0	Not_GOST-cit	0
Not_GOST-cit	0	Not_GOST-cit	0
Not_GOST-cit	0	Not_GOST-cit	0
Not_GOST-cit	0	Not_GOST-cit	0
Not_GOST-cit	0	Not_GOST-cit	0
Not_GOST-cit	0	Not_GOST-cit	0
Not_GOST-cit	0	Not_GOST-cit	0
Not_GOST-cit	0	Not_GOST-cit	0
Not_GOST-cit	0	Not_GOST-cit	0
Not_GOST-cit	0	Not_GOST-cit	0
Not_GOST-cit	0	Not_GOST-cit	0
Not_GOST-cit	0	Not_GOST-cit	0
Not_GOST-cit	0	Not_GOST-cit	0
Not_GOST-cit	0	Not_GOST-cit	0
Not_GOST-cit	0	Not_GOST-cit	0
Not_GOST-cit	0	Not_GOST-cit	0
Not_GOST-cit	0	Not_GOST-cit	0
Not_GOST-cit	0	Not_GOST-cit	0
Not_GOST-cit	0	Not_GOST-cit	0
Not_GOST-cit	0	Not_GOST-cit	0
Not_GOST-cit	0	Not_GOST-cit	0

Not_GOST-cit	0	Not_GOST-cit	0
Not_GOST-cit	0	Not_GOST-cit	0
Not_GOST-cit	0	Not_GOST-cit	0
Not_GOST-cit	0	Not_GOST-cit	0
Not_GOST-cit	0	Not_GOST-cit	0
Not_GOST-cit	0	Not_GOST-cit	0
Not_GOST-cit	0	Not_GOST-cit	0
Not_GOST-cit	0	Not_GOST-cit	0
Not_GOST-cit	0	Not_GOST-cit	0
Not_GOST-cit	0	Not_GOST-cit	0
GOST-cit-all	1	GOST-cit-Y-all	1
GOST-cit-all	1	GOST-cit-Y-all	1
GOST-cit-all	1	GOST-cit-Y-all	1
Not_GOST-cit	0	Not_GOST-cit	0
Not_GOST-cit	0	Not_GOST-cit	0
Not_GOST-cit	0	Not_GOST-cit	0
Not_GOST-cit	0	Not_GOST-cit	0
Not_GOST-cit	0	Not_GOST-cit	0
Not_GOST-cit	0	Not_GOST-cit	0
Not_GOST-cit	0	Not_GOST-cit	0
Not_GOST-cit	0	Not_GOST-cit	0
Not_GOST-cit	0	Not_GOST-cit	0
Not_GOST-cit	0	Not_GOST-cit	0
Not_GOST-cit	0	Not_GOST-cit	0
Not_GOST-cit	0	Not_GOST-cit	0
Not_GOST-cit	0	Not_GOST-cit	0
Not_GOST-cit	0	Not_GOST-cit	0
Not_GOST-cit	0	Not_GOST-cit	0
Not_GOST-cit	0	Not_GOST-cit	0
Not_GOST-cit	0	Not_GOST-cit	0
Not_GOST-cit	0	Not_GOST-cit	0
Not_GOST-cit	0	Not_GOST-cit	0
Not_GOST-cit	0	Not_GOST-cit	0
Not_GOST-cit	0	Not_GOST-cit	0
Not_GOST-cit	0	Not_GOST-cit	0
Not_GOST-cit	0	Not_GOST-cit	0
Not_GOST-cit	0	Not_GOST-cit	0
Not_GOST-cit	0	Not_GOST-cit	0
Not_GOST-cit	0	Not_GOST-cit	0
Not_GOST-cit	0	Not_GOST-cit	0
Not_GOST-cit	0	Not_GOST-cit	0
Not_GOST-cit	0	Not_GOST-cit	0
Not_GOST-cit	0	Not_GOST-cit	0
Not_GOST-cit	0	Not_GOST-cit	0
Not_GOST-cit	0	Not_GOST-cit	0
Not_GOST-cit	0	Not_GOST-cit	0
Not_GOST-cit	0	Not_GOST-cit	0

Not_GOST-cit	0	Not_GOST-cit	0
Not_GOST-cit	0	Not_GOST-cit	0
Not_GOST-cit	0	Not_GOST-cit	0
Not_GOST-cit	0	Not_GOST-cit	0
Not_GOST-cit	0	Not_GOST-cit	0
Not_GOST-cit	0	Not_GOST-cit	0
Not_GOST-cit	0	Not_GOST-cit	0
Not_GOST-cit	0	Not_GOST-cit	0
Not_GOST-cit	0	Not_GOST-cit	0
Not_GOST-cit	0	Not_GOST-cit	0
Not_GOST-cit	0	Not_GOST-cit	0
Not_GOST-cit	0	Not_GOST-cit	0
Not_GOST-cit	0	Not_GOST-cit	0
Not_GOST-cit	0	Not_GOST-cit	0
Not_GOST-cit	0	Not_GOST-cit	0
Not_GOST-cit	0	Not_GOST-cit	0
Not_GOST-cit	0	Not_GOST-cit	0
Not_GOST-cit	0	Not_GOST-cit	0
Not_GOST-cit	0	Not_GOST-cit	0
Not_GOST-cit	0	Not_GOST-cit	0
Not_GOST-cit	0	Not_GOST-cit	0
Not_GOST-cit	0	Not_GOST-cit	0
Not_GOST-cit	0	Not_GOST-cit	0
Not_GOST-cit	0	Not_GOST-cit	0
Not_GOST-cit	0	Not_GOST-cit	0
Not_GOST-cit	0	Not_GOST-cit	0
Not_GOST-cit	0	Not_GOST-cit	0
Not_GOST-cit	0	Not_GOST-cit	0
Not_GOST-cit	0	Not_GOST-cit	0
Not_GOST-cit	0	Not_GOST-cit	0
Not_GOST-cit	0	Not_GOST-cit	0
Not_GOST-cit	0	Not_GOST-cit	0
Not_GOST-cit	0	Not_GOST-cit	0
Not_GOST-cit	0	Not_GOST-cit	0
Not_GOST-cit	0	Not_GOST-cit	0
Not_GOST-cit	0	Not_GOST-cit	0
Not_GOST-cit	0	Not_GOST-cit	0
Not_GOST-cit	0	Not_GOST-cit	0
Not_GOST-cit	0	Not_GOST-cit	0
Not_GOST-cit	0	Not_GOST-cit	0
Not_GOST-cit	0	Not_GOST-cit	0
Not_GOST-cit	0	Not_GOST-cit	0
Not_GOST-cit	0	Not_GOST-cit	0
Not_GOST-cit	0	Not_GOST-cit	0
Not_GOST-cit	0	Not_GOST-cit	0
Not_GOST-cit	0	Not_GOST-cit	0
Not_GOST-cit	0	Not_GOST-cit	0

Not_GOST-cit	0	Not_GOST-cit	0
Not_GOST-cit	0	Not_GOST-cit	0
Not_GOST-cit	0	Not_GOST-cit	0
Not_GOST-cit	0	Not_GOST-cit	0
Not_GOST-cit	0	Not_GOST-cit	0
Not_GOST-cit	0	Not_GOST-cit	0
Not_GOST-cit	0	GOST-cit-Y-all	1
Not_GOST-cit	0	GOST-cit-Y-all	1
Not_GOST-cit	0	GOST-cit-Y-all	1
Not_GOST-cit	0	GOST-cit-Y-all	1
Not_GOST-cit	0	Not_GOST-cit	0
Not_GOST-cit	0	Not_GOST-cit	0
Not_GOST-cit	0	Not_GOST-cit	0
Not_GOST-cit	0	Not_GOST-cit	0
Not_GOST-cit	0	Not_GOST-cit	0
Not_GOST-cit	0	Not_GOST-cit	0
Not_GOST-cit	0	Not_GOST-cit	0
GOST-cit-all	1	Not_GOST-cit	0
GOST-cit-all	1	Not_GOST-cit	0
Not_GOST-cit	0	GOST-cit-Y-all	1
Not_GOST-cit	0	GOST-cit-Y-all	1
Not_GOST-cit	0	Not_GOST-cit	0
Not_GOST-cit	0	Not_GOST-cit	0
Not_GOST-cit	0	Not_GOST-cit	0
Not_GOST-cit	0	Not_GOST-cit	0
Not_GOST-cit	0	Not_GOST-cit	0
Not_GOST-cit	0	Not_GOST-cit	0
Not_GOST-cit	0	Not_GOST-cit	0
Not_GOST-cit	0	Not_GOST-cit	0
Not_GOST-cit	0	Not_GOST-cit	0
Not_GOST-cit	0	Not_GOST-cit	0
Not_GOST-cit	0	Not_GOST-cit	0
Not_GOST-cit	0	Not_GOST-cit	0
Not_GOST-cit	0	Not_GOST-cit	0
Not_GOST-cit	0	Not_GOST-cit	0
Not_GOST-cit	0	Not_GOST-cit	0
GOST-cit-all	1	GOST-cit-Y-all	1
GOST-cit-all	1	GOST-cit-Y-all	1
Not_GOST-cit	0	Not_GOST-cit	0
Not_GOST-cit	0	Not_GOST-cit	0
Not_GOST-cit	0	Not_GOST-cit	0
Not_GOST-cit	0	Not_GOST-cit	0
Not_GOST-cit	0	Not_GOST-cit	0
Not_GOST-cit	0	Not_GOST-cit	0
Not_GOST-cit	0	Not_GOST-cit	0
Not_GOST-cit	0	Not_GOST-cit	0
Not_GOST-cit	0	Not_GOST-cit	0

Not_GOST-cit	0	Not_GOST-cit	0
Not_GOST-cit	0	Not_GOST-cit	0
GOST-cit-all	1	Not_GOST-cit	0
GOST-cit-all	1	Not_GOST-cit	0
Not_GOST-cit	0	Not_GOST-cit	0
Not_GOST-cit	0	Not_GOST-cit	0
Not_GOST-cit	0	Not_GOST-cit	0
Not_GOST-cit	0	Not_GOST-cit	0
Not_GOST-cit	0	Not_GOST-cit	0
Not_GOST-cit	0	Not_GOST-cit	0
Not_GOST-cit	0	Not_GOST-cit	0
Not_GOST-cit	0	Not_GOST-cit	0
Not_GOST-cit	0	Not_GOST-cit	0
Not_GOST-cit	0	Not_GOST-cit	0
Not_GOST-cit	0	Not_GOST-cit	0
Not_GOST-cit	0	Not_GOST-cit	0
Not_GOST-cit	0	Not_GOST-cit	0
Not_GOST-cit	0	Not_GOST-cit	0
Not_GOST-cit	0	Not_GOST-cit	0
Not_GOST-cit	0	GOST-cit-Y-all	1
Not_GOST-cit	0	GOST-cit-Y-all	1
Not_GOST-cit	0	GOST-cit-Y-all	1
Not_GOST-cit	0	Not_GOST-cit	0
Not_GOST-cit	0	Not_GOST-cit	0
Not_GOST-cit	0	Not_GOST-cit	0
Not_GOST-cit	0	Not_GOST-cit	0
Not_GOST-cit	0	Not_GOST-cit	0
Not_GOST-cit	0	Not_GOST-cit	0
Not_GOST-cit	0	Not_GOST-cit	0
Not_GOST-cit	0	Not_GOST-cit	0
GOST-cit-all	1	GOST-cit-Y-all	1
GOST-cit-all	1	GOST-cit-Y-all	1
Not_GOST-cit	0	Not_GOST-cit	0
GOST-cit-all	1	Not_GOST-cit	0
GOST-cit-all	1	Not_GOST-cit	0
GOST-cit-all	1	Not_GOST-cit	0
GOST-cit-all	1	Not_GOST-cit	0
Not_GOST-cit	0	Not_GOST-cit	0
Not_GOST-cit	0	Not_GOST-cit	0
Not_GOST-cit	0	Not_GOST-cit	0
Not_GOST-cit	0	Not_GOST-cit	0
Not_GOST-cit	0	Not_GOST-cit	0
GOST-cit-all	1	Not_GOST-cit	0
GOST-cit-all	1	Not_GOST-cit	0
Not_GOST-cit	0	Not_GOST-cit	0
Not_GOST-cit	0	Not_GOST-cit	0
Not_GOST-cit	0	Not_GOST-cit	0

Not_GOST-cit	0	Not_GOST-cit	0
Not_GOST-cit	0	Not_GOST-cit	0
Not_GOST-cit	0	Not_GOST-cit	0
Not_GOST-cit	0	Not_GOST-cit	0
Not_GOST-cit	0	Not_GOST-cit	0
Not_GOST-cit	0	Not_GOST-cit	0
Not_GOST-cit	0	Not_GOST-cit	0
Not_GOST-cit	0	Not_GOST-cit	0
Not_GOST-cit	0	GOST-cit-Y-all	1
Not_GOST-cit	0	Not_GOST-cit	0
Not_GOST-cit	0	Not_GOST-cit	0
Not_GOST-cit	0	Not_GOST-cit	0
Not_GOST-cit	0	Not_GOST-cit	0
Not_GOST-cit	0	Not_GOST-cit	0
Not_GOST-cit	0	Not_GOST-cit	0
Not_GOST-cit	0	Not_GOST-cit	0
Not_GOST-cit	0	Not_GOST-cit	0
Not_GOST-cit	0	Not_GOST-cit	0
Not_GOST-cit	0	Not_GOST-cit	0
Not_GOST-cit	0	Not_GOST-cit	0
Not_GOST-cit	0	Not_GOST-cit	0
Not_GOST-cit	0	GOST-cit-Y-all	1
Not_GOST-cit	0	GOST-cit-Y-all	1
GOST-cit-all	1	Not_GOST-cit	0
Not_GOST-cit	0	Not_GOST-cit	0
Not_GOST-cit	0	Not_GOST-cit	0
Not_GOST-cit	0	Not_GOST-cit	0
Not_GOST-cit	0	Not_GOST-cit	0
Not_GOST-cit	0	Not_GOST-cit	0
GOST-cit-all	1	Not_GOST-cit	0
GOST-cit-all	1	Not_GOST-cit	0
Not_GOST-cit	0	Not_GOST-cit	0
Not_GOST-cit	0	Not_GOST-cit	0
Not_GOST-cit	0	Not_GOST-cit	0
Not_GOST-cit	0	Not_GOST-cit	0
Not_GOST-cit	0	Not_GOST-cit	0
Not_GOST-cit	0	Not_GOST-cit	0
Not_GOST-cit	0	Not_GOST-cit	0
Not_GOST-cit	0	GOST-cit-Y-all	1
Not_GOST-cit	0	GOST-cit-Y-all	1
Not_GOST-cit	0	Not_GOST-cit	0
Not_GOST-cit	0	Not_GOST-cit	0
Not_GOST-cit	0	Not_GOST-cit	0
Not_GOST-cit	0	Not_GOST-cit	0
Not_GOST-cit	0	Not_GOST-cit	0
Not_GOST-cit	0	Not_GOST-cit	0
Not_GOST-cit	0	Not_GOST-cit	0

GOST-cit-all	1	Not_GOST-cit	0
Not_GOST-cit	0	Not_GOST-cit	0
Not_GOST-cit	0	Not_GOST-cit	0
Not_GOST-cit	0	Not_GOST-cit	0
Not_GOST-cit	0	Not_GOST-cit	0
Not_GOST-cit	0	Not_GOST-cit	0
Not_GOST-cit	0	Not_GOST-cit	0
Not_GOST-cit	0	Not_GOST-cit	0
Not_GOST-cit	0	Not_GOST-cit	0
Not_GOST-cit	0	Not_GOST-cit	0
Not_GOST-cit	0	Not_GOST-cit	0
Not_GOST-cit	0	Not_GOST-cit	0
Not_GOST-cit	0	Not_GOST-cit	0
Not_GOST-cit	0	Not_GOST-cit	0
GOST-cit-all	1	Not_GOST-cit	0
Not_GOST-cit	0	Not_GOST-cit	0
Not_GOST-cit	0	Not_GOST-cit	0
Not_GOST-cit	0	Not_GOST-cit	0
Not_GOST-cit	0	Not_GOST-cit	0
Not_GOST-cit	0	Not_GOST-cit	0
Not_GOST-cit	0	Not_GOST-cit	0
Not_GOST-cit	0	Not_GOST-cit	0
Not_GOST-cit	0	Not_GOST-cit	0
Not_GOST-cit	0	Not_GOST-cit	0
Not_GOST-cit	0	Not_GOST-cit	0
Not_GOST-cit	0	Not_GOST-cit	0
Not_GOST-cit	0	Not_GOST-cit	0
Not_GOST-cit	0	Not_GOST-cit	0
Not_GOST-cit	0	Not_GOST-cit	0
Not_GOST-cit	0	Not_GOST-cit	0
Not_GOST-cit	0	Not_GOST-cit	0
Not_GOST-cit	0	Not_GOST-cit	0
Not_GOST-cit	0	Not_GOST-cit	0
Not_GOST-cit	0	Not_GOST-cit	0
Not_GOST-cit	0	Not_GOST-cit	0
Not_GOST-cit	0	Not_GOST-cit	0
Not_GOST-cit	0	Not_GOST-cit	0
Not_GOST-cit	0	Not_GOST-cit	0
Not_GOST-cit	0	Not_GOST-cit	0
Not_GOST-cit	0	Not_GOST-cit	0
Not_GOST-cit	0	Not_GOST-cit	0
Not_GOST-cit	0	Not_GOST-cit	0
Not_GOST-cit	0	Not_GOST-cit	0
Not_GOST-cit	0	Not_GOST-cit	0
Not_GOST-cit	0	Not_GOST-cit	0
Not_GOST-cit	0	Not_GOST-cit	0
Not_GOST-cit	0	Not_GOST-cit	0

Not_GOST-cit	0	Not_GOST-cit	0
Not_GOST-cit	0	Not_GOST-cit	0
Not_GOST-cit	0	Not_GOST-cit	0
Not_GOST-cit	0	Not_GOST-cit	0
Not_GOST-cit	0	Not_GOST-cit	0
Not_GOST-cit	0	GOST-cit-Y-all	1
Not_GOST-cit	0	GOST-cit-Y-all	1
Not_GOST-cit	0	GOST-cit-Y-all	1
Not_GOST-cit	0	Not_GOST-cit	0
Not_GOST-cit	0	Not_GOST-cit	0
Not_GOST-cit	0	Not_GOST-cit	0
Not_GOST-cit	0	Not_GOST-cit	0
Not_GOST-cit	0	Not_GOST-cit	0
Not_GOST-cit	0	Not_GOST-cit	0
Not_GOST-cit	0	Not_GOST-cit	0
Not_GOST-cit	0	Not_GOST-cit	0
Not_GOST-cit	0	Not_GOST-cit	0
Not_GOST-cit	0	Not_GOST-cit	0
Not_GOST-cit	0	Not_GOST-cit	0
Not_GOST-cit	0	Not_GOST-cit	0
Not_GOST-cit	0	Not_GOST-cit	0
Not_GOST-cit	0	Not_GOST-cit	0
Not_GOST-cit	0	Not_GOST-cit	0
Not_GOST-cit	0	Not_GOST-cit	0
Not_GOST-cit	0	Not_GOST-cit	0
Not_GOST-cit	0	Not_GOST-cit	0
Not_GOST-cit	0	GOST-cit-Y-all	1
Not_GOST-cit	0	GOST-cit-Y-all	1
Not_GOST-cit	0	GOST-cit-Y-all	1
Not_GOST-cit	0	Not_GOST-cit	0
Not_GOST-cit	0	Not_GOST-cit	0
Not_GOST-cit	0	Not_GOST-cit	0
Not_GOST-cit	0	Not_GOST-cit	0
Not_GOST-cit	0	Not_GOST-cit	0
Not_GOST-cit	0	Not_GOST-cit	0
Not_GOST-cit	0	Not_GOST-cit	0
GOST-cit-all	1	GOST-cit-Y-all	1
GOST-cit-all	1	GOST-cit-Y-all	1
Not_GOST-cit	0	Not_GOST-cit	0
Not_GOST-cit	0	Not_GOST-cit	0
Not_GOST-cit	0	Not_GOST-cit	0
Not_GOST-cit	0	Not_GOST-cit	0
Not_GOST-cit	0	Not_GOST-cit	0
Not_GOST-cit	0	Not_GOST-cit	0
Not_GOST-cit	0	Not_GOST-cit	0
Not_GOST-cit	0	Not_GOST-cit	0
Not_GOST-cit	0	Not_GOST-cit	0

Not_GOST-cit	0	Not_GOST-cit	0
Not_GOST-cit	0	Not_GOST-cit	0
Not_GOST-cit	0	Not_GOST-cit	0
Not_GOST-cit	0	Not_GOST-cit	0
Not_GOST-cit	0	Not_GOST-cit	0
Not_GOST-cit	0	Not_GOST-cit	0
Not_GOST-cit	0	Not_GOST-cit	0
Not_GOST-cit	0	Not_GOST-cit	0
Not_GOST-cit	0	Not_GOST-cit	0
Not_GOST-cit	0	Not_GOST-cit	0
Not_GOST-cit	0	GOST-cit-Y-all	1
Not_GOST-cit	0	Not_GOST-cit	0
Not_GOST-cit	0	Not_GOST-cit	0
Not_GOST-cit	0	Not_GOST-cit	0
Not_GOST-cit	0	Not_GOST-cit	0
Not_GOST-cit	0	Not_GOST-cit	0
Not_GOST-cit	0	Not_GOST-cit	0
Not_GOST-cit	0	Not_GOST-cit	0
Not_GOST-cit	0	Not_GOST-cit	0
Not_GOST-cit	0	Not_GOST-cit	0
Not_GOST-cit	0	Not_GOST-cit	0
Not_GOST-cit	0	Not_GOST-cit	0
Not_GOST-cit	0	Not_GOST-cit	0
Not_GOST-cit	0	Not_GOST-cit	0
GOST-cit-all	1	GOST-cit-Y-all	1
Not_GOST-cit	0	Not_GOST-cit	0
Not_GOST-cit	0	Not_GOST-cit	0
Not_GOST-cit	0	Not_GOST-cit	0
Not_GOST-cit	0	Not_GOST-cit	0
Not_GOST-cit	0	Not_GOST-cit	0
Not_GOST-cit	0	Not_GOST-cit	0
Not_GOST-cit	0	Not_GOST-cit	0
Not_GOST-cit	0	Not_GOST-cit	0
Not_GOST-cit	0	Not_GOST-cit	0
Not_GOST-cit	0	Not_GOST-cit	0
Not_GOST-cit	0	Not_GOST-cit	0
Not_GOST-cit	0	Not_GOST-cit	0
Not_GOST-cit	0	Not_GOST-cit	0
Not_GOST-cit	0	Not_GOST-cit	0
Not_GOST-cit	0	Not_GOST-cit	0
Not_GOST-cit	0	Not_GOST-cit	0
Not_GOST-cit	0	Not_GOST-cit	0
Not_GOST-cit	0	Not_GOST-cit	0
Not_GOST-cit	0	Not_GOST-cit	0
Not_GOST-cit	0	Not_GOST-cit	0
Not_GOST-cit	0	Not_GOST-cit	0
Not_GOST-cit	0	Not_GOST-cit	0

Not_GOST-cit	0	Not_GOST-cit	0
Not_GOST-cit	0	Not_GOST-cit	0
Not_GOST-cit	0	Not_GOST-cit	0
Not_GOST-cit	0	Not_GOST-cit	0
Not_GOST-cit	0	Not_GOST-cit	0
GOST-cit-all	1	GOST-cit-Y-all	1
Not_GOST-cit	0	Not_GOST-cit	0
Not_GOST-cit	0	Not_GOST-cit	0
Not_GOST-cit	0	Not_GOST-cit	0
Not_GOST-cit	0	Not_GOST-cit	0
Not_GOST-cit	0	Not_GOST-cit	0
Not_GOST-cit	0	Not_GOST-cit	0
Not_GOST-cit	0	Not_GOST-cit	0
Not_GOST-cit	0	Not_GOST-cit	0
Not_GOST-cit	0	Not_GOST-cit	0
Not_GOST-cit	0	Not_GOST-cit	0
Not_GOST-cit	0	Not_GOST-cit	0
Not_GOST-cit	0	Not_GOST-cit	0
Not_GOST-cit	0	Not_GOST-cit	0
Not_GOST-cit	0	Not_GOST-cit	0
Not_GOST-cit	0	Not_GOST-cit	0
Not_GOST-cit	0	Not_GOST-cit	0
Not_GOST-cit	0	Not_GOST-cit	0
Not_GOST-cit	0	Not_GOST-cit	0
Not_GOST-cit	0	Not_GOST-cit	0
Not_GOST-cit	0	Not_GOST-cit	0
Not_GOST-cit	0	Not_GOST-cit	0
Not_GOST-cit	0	Not_GOST-cit	0
Not_GOST-cit	0	Not_GOST-cit	0
Not_GOST-cit	0	Not_GOST-cit	0
Not_GOST-cit	0	Not_GOST-cit	0
Not_GOST-cit	0	Not_GOST-cit	0
Not_GOST-cit	0	Not_GOST-cit	0
Not_GOST-cit	0	Not_GOST-cit	0
Not_GOST-cit	0	Not_GOST-cit	0
Not_GOST-cit	0	Not_GOST-cit	0
Not_GOST-cit	0	Not_GOST-cit	0
Not_GOST-cit	0	Not_GOST-cit	0
Not_GOST-cit	0	Not_GOST-cit	0
Not_GOST-cit	0	Not_GOST-cit	0
Not_GOST-cit	0	Not_GOST-cit	0
Not_GOST-cit	0	GOST-cit-Y-all	1
Not_GOST-cit	0	GOST-cit-Y-all	1
GOST-cit-all	1	Not_GOST-cit	0
Not_GOST-cit	0	Not_GOST-cit	0
Not_GOST-cit	0	Not_GOST-cit	0
Not_GOST-cit	0	Not_GOST-cit	0
Not_GOST-cit	0	Not_GOST-cit	0
Not_GOST-cit	0	Not_GOST-cit	0
Not_GOST-cit	0	Not_GOST-cit	0

Not_GOST-cit	0	Not_GOST-cit	0
Not_GOST-cit	0	Not_GOST-cit	0
Not_GOST-cit	0	Not_GOST-cit	0
Not_GOST-cit	0	Not_GOST-cit	0
Not_GOST-cit	0	Not_GOST-cit	0
Not_GOST-cit	0	Not_GOST-cit	0
Not_GOST-cit	0	Not_GOST-cit	0
Not_GOST-cit	0	Not_GOST-cit	0
GOST-cit-all	1	Not_GOST-cit	0
GOST-cit-all	1	Not_GOST-cit	0
GOST-cit-all	1	Not_GOST-cit	0
Not_GOST-cit	0	Not_GOST-cit	0
Not_GOST-cit	0	Not_GOST-cit	0
Not_GOST-cit	0	Not_GOST-cit	0
Not_GOST-cit	0	Not_GOST-cit	0
Not_GOST-cit	0	Not_GOST-cit	0
Not_GOST-cit	0	Not_GOST-cit	0
Not_GOST-cit	0	Not_GOST-cit	0
Not_GOST-cit	0	Not_GOST-cit	0
Not_GOST-cit	0	Not_GOST-cit	0
Not_GOST-cit	0	Not_GOST-cit	0
Not_GOST-cit	0	Not_GOST-cit	0
Not_GOST-cit	0	Not_GOST-cit	0
Not_GOST-cit	0	Not_GOST-cit	0
Not_GOST-cit	0	Not_GOST-cit	0
Not_GOST-cit	0	Not_GOST-cit	0
Not_GOST-cit	0	Not_GOST-cit	0
Not_GOST-cit	0	Not_GOST-cit	0
Not_GOST-cit	0	Not_GOST-cit	0
Not_GOST-cit	0	Not_GOST-cit	0
Not_GOST-cit	0	Not_GOST-cit	0
Not_GOST-cit	0	Not_GOST-cit	0
Not_GOST-cit	0	Not_GOST-cit	0
Not_GOST-cit	0	Not_GOST-cit	0
Not_GOST-cit	0	Not_GOST-cit	0
Not_GOST-cit	0	Not_GOST-cit	0
Not_GOST-cit	0	Not_GOST-cit	0
Not_GOST-cit	0	Not_GOST-cit	0
Not_GOST-cit	0	Not_GOST-cit	0
Not_GOST-cit	0	Not_GOST-cit	0
Not_GOST-cit	0	Not_GOST-cit	0
Not_GOST-cit	0	Not_GOST-cit	0
Not_GOST-cit	0	Not_GOST-cit	0
GOST-cit-all	1	GOST-cit-Y-all	1
GOST-cit-all	1	GOST-cit-Y-all	1
GOST-cit-all	1	GOST-cit-Y-all	1
Not_GOST-cit	0	GOST-cit-Y-all	1

Not_GOST-cit	0	GOST-cit-Y-all	1
Not_GOST-cit	0	Not_GOST-cit	0
Not_GOST-cit	0	Not_GOST-cit	0
Not_GOST-cit	0	Not_GOST-cit	0
Not_GOST-cit	0	Not_GOST-cit	0
Not_GOST-cit	0	Not_GOST-cit	0
Not_GOST-cit	0	Not_GOST-cit	0
Not_GOST-cit	0	Not_GOST-cit	0
Not_GOST-cit	0	Not_GOST-cit	0
Not_GOST-cit	0	GOST-cit-Y-all	1
Not_GOST-cit	0	Not_GOST-cit	0
Not_GOST-cit	0	Not_GOST-cit	0
Not_GOST-cit	0	Not_GOST-cit	0
Not_GOST-cit	0	Not_GOST-cit	0
Not_GOST-cit	0	Not_GOST-cit	0
Not_GOST-cit	0	Not_GOST-cit	0
Not_GOST-cit	0	Not_GOST-cit	0
Not_GOST-cit	0	Not_GOST-cit	0
Not_GOST-cit	0	Not_GOST-cit	0
Not_GOST-cit	0	Not_GOST-cit	0
Not_GOST-cit	0	Not_GOST-cit	0
Not_GOST-cit	0	Not_GOST-cit	0
Not_GOST-cit	0	Not_GOST-cit	0
Not_GOST-cit	0	Not_GOST-cit	0
GOST-cit-all	1	GOST-cit-Y-all	1
GOST-cit-all	1	GOST-cit-Y-all	1
GOST-cit-all	1	GOST-cit-Y-all	1
GOST-cit-all	1	GOST-cit-Y-all	1
Not_GOST-cit	0	Not_GOST-cit	0
Not_GOST-cit	0	Not_GOST-cit	0
Not_GOST-cit	0	Not_GOST-cit	0
Not_GOST-cit	0	Not_GOST-cit	0
Not_GOST-cit	0	Not_GOST-cit	0
Not_GOST-cit	0	Not_GOST-cit	0
Not_GOST-cit	0	Not_GOST-cit	0
Not_GOST-cit	0	Not_GOST-cit	0
Not_GOST-cit	0	Not_GOST-cit	0
Not_GOST-cit	0	Not_GOST-cit	0
Not_GOST-cit	0	Not_GOST-cit	0
Not_GOST-cit	0	Not_GOST-cit	0
Not_GOST-cit	0	Not_GOST-cit	0
Not_GOST-cit	0	Not_GOST-cit	0
Not_GOST-cit	0	Not_GOST-cit	0
Not_GOST-cit	0	Not_GOST-cit	0
Not_GOST-cit	0	Not_GOST-cit	0
Not_GOST-cit	0	Not_GOST-cit	0
Not_GOST-cit	0	Not_GOST-cit	0

GOST-cit-all	1	GOST-cit-Y-all	1
Not_GOST-cit	0	Not_GOST-cit	0
Not_GOST-cit	0	Not_GOST-cit	0
Not_GOST-cit	0	Not_GOST-cit	0
Not_GOST-cit	0	GOST-cit-Y-all	1
Not_GOST-cit	0	GOST-cit-Y-all	1
Not_GOST-cit	0	Not_GOST-cit	0
Not_GOST-cit	0	Not_GOST-cit	0
Not_GOST-cit	0	Not_GOST-cit	0
GOST-cit-all	1	Not_GOST-cit	0
Not_GOST-cit	0	Not_GOST-cit	0
Not_GOST-cit	0	Not_GOST-cit	0
Not_GOST-cit	0	Not_GOST-cit	0
Not_GOST-cit	0	Not_GOST-cit	0
Not_GOST-cit	0	Not_GOST-cit	0
Not_GOST-cit	0	Not_GOST-cit	0
Not_GOST-cit	0	Not_GOST-cit	0
Not_GOST-cit	0	Not_GOST-cit	0
Not_GOST-cit	0	Not_GOST-cit	0
Not_GOST-cit	0	Not_GOST-cit	0
Not_GOST-cit	0	Not_GOST-cit	0
Not_GOST-cit	0	Not_GOST-cit	0
Not_GOST-cit	0	Not_GOST-cit	0
Not_GOST-cit	0	Not_GOST-cit	0
Not_GOST-cit	0	Not_GOST-cit	0
Not_GOST-cit	0	Not_GOST-cit	0
Not_GOST-cit	0	Not_GOST-cit	0
Not_GOST-cit	0	Not_GOST-cit	0
Not_GOST-cit	0	Not_GOST-cit	0
Not_GOST-cit	0	Not_GOST-cit	0
Not_GOST-cit	0	Not_GOST-cit	0
Not_GOST-cit	0	Not_GOST-cit	0
Not_GOST-cit	0	Not_GOST-cit	0
Not_GOST-cit	0	Not_GOST-cit	0
Not_GOST-cit	0	Not_GOST-cit	0
Not_GOST-cit	0	Not_GOST-cit	0
Not_GOST-cit	0	Not_GOST-cit	0
Not_GOST-cit	0	Not_GOST-cit	0
GOST-cit-all	1	Not_GOST-cit	0
GOST-cit-all	1	Not_GOST-cit	0
Not_GOST-cit	0	Not_GOST-cit	0
Not_GOST-cit	0	Not_GOST-cit	0
Not_GOST-cit	0	Not_GOST-cit	0
Not_GOST-cit	0	Not_GOST-cit	0
Not_GOST-cit	0	Not_GOST-cit	0
Not_GOST-cit	0	Not_GOST-cit	0
Not_GOST-cit	0	Not_GOST-cit	0
Not_GOST-cit	0	Not_GOST-cit	0

Not_GOST-cit	0	Not_GOST-cit	0
Not_GOST-cit	0	Not_GOST-cit	0
Not_GOST-cit	0	Not_GOST-cit	0
Not_GOST-cit	0	Not_GOST-cit	0
Not_GOST-cit	0	Not_GOST-cit	0
Not_GOST-cit	0	Not_GOST-cit	0
Not_GOST-cit	0	Not_GOST-cit	0
GOST-cit-all	1	GOST-cit-Y-all	1
GOST-cit-all	1	GOST-cit-Y-all	1
Not_GOST-cit	0	Not_GOST-cit	0
Not_GOST-cit	0	Not_GOST-cit	0
Not_GOST-cit	0	Not_GOST-cit	0
Not_GOST-cit	0	Not_GOST-cit	0
Not_GOST-cit	0	Not_GOST-cit	0
Not_GOST-cit	0	Not_GOST-cit	0
GOST-cit-all	1	GOST-cit-Y-all	1
Not_GOST-cit	0	Not_GOST-cit	0
Not_GOST-cit	0	Not_GOST-cit	0
Not_GOST-cit	0	Not_GOST-cit	0
Not_GOST-cit	0	Not_GOST-cit	0
Not_GOST-cit	0	Not_GOST-cit	0
Not_GOST-cit	0	Not_GOST-cit	0
GOST-cit-all	1	GOST-cit-Y-all	1
GOST-cit-all	1	GOST-cit-Y-all	1
GOST-cit-all	1	GOST-cit-Y-all	1
Not_GOST-cit	0	Not_GOST-cit	0
Not_GOST-cit	0	GOST-cit-Y-all	1
Not_GOST-cit	0	Not_GOST-cit	0
Not_GOST-cit	0	GOST-cit-Y-all	1
Not_GOST-cit	0	GOST-cit-Y-all	1
Not_GOST-cit	0	Not_GOST-cit	0
Not_GOST-cit	0	GOST-cit-Y-all	1
Not_GOST-cit	0	GOST-cit-Y-all	1
Not_GOST-cit	0	GOST-cit-Y-all	1
Not_GOST-cit	0	Not_GOST-cit	0
Not_GOST-cit	0	Not_GOST-cit	0
Not_GOST-cit	0	Not_GOST-cit	0
Not_GOST-cit	0	Not_GOST-cit	0
Not_GOST-cit	0	Not_GOST-cit	0
Not_GOST-cit	0	Not_GOST-cit	0
Not_GOST-cit	0	Not_GOST-cit	0
Not_GOST-cit	0	Not_GOST-cit	0
Not_GOST-cit	0	Not_GOST-cit	0
Not_GOST-cit	0	Not_GOST-cit	0
Not_GOST-cit	0	Not_GOST-cit	0
GOST-cit-all	1	Not_GOST-cit	0
Not_GOST-cit	0	Not_GOST-cit	0

Not_GOST-cit	0	Not_GOST-cit	0
Not_GOST-cit	0	Not_GOST-cit	0
Not_GOST-cit	0	Not_GOST-cit	0
Not_GOST-cit	0	Not_GOST-cit	0
Not_GOST-cit	0	Not_GOST-cit	0
Not_GOST-cit	0	GOST-cit-Y-all	1
Not_GOST-cit	0	Not_GOST-cit	0
Not_GOST-cit	0	Not_GOST-cit	0
Not_GOST-cit	0	Not_GOST-cit	0
GOST-cit-all	1	GOST-cit-Y-all	1
GOST-cit-all	1	GOST-cit-Y-all	1
GOST-cit-all	1	GOST-cit-Y-all	1
Not_GOST-cit	0	Not_GOST-cit	0
Not_GOST-cit	0	Not_GOST-cit	0
Not_GOST-cit	0	Not_GOST-cit	0
Not_GOST-cit	0	Not_GOST-cit	0
Not_GOST-cit	0	Not_GOST-cit	0
Not_GOST-cit	0	Not_GOST-cit	0
Not_GOST-cit	0	Not_GOST-cit	0
Not_GOST-cit	0	Not_GOST-cit	0
Not_GOST-cit	0	Not_GOST-cit	0
Not_GOST-cit	0	Not_GOST-cit	0
Not_GOST-cit	0	Not_GOST-cit	0
Not_GOST-cit	0	Not_GOST-cit	0
Not_GOST-cit	0	Not_GOST-cit	0
Not_GOST-cit	0	Not_GOST-cit	0
Not_GOST-cit	0	Not_GOST-cit	0
Not_GOST-cit	0	Not_GOST-cit	0
Not_GOST-cit	0	GOST-cit-Y-all	1
Not_GOST-cit	0	GOST-cit-Y-all	1
Not_GOST-cit	0	Not_GOST-cit	0
Not_GOST-cit	0	GOST-cit-Y-all	1
Not_GOST-cit	0	Not_GOST-cit	0
Not_GOST-cit	0	Not_GOST-cit	0
Not_GOST-cit	0	Not_GOST-cit	0
Not_GOST-cit	0	Not_GOST-cit	0
Not_GOST-cit	0	Not_GOST-cit	0
Not_GOST-cit	0	Not_GOST-cit	0
Not_GOST-cit	0	Not_GOST-cit	0
GOST-cit-all	1	Not_GOST-cit	0
GOST-cit-all	1	Not_GOST-cit	0
Not_GOST-cit	0	Not_GOST-cit	0
Not_GOST-cit	0	Not_GOST-cit	0
Not_GOST-cit	0	Not_GOST-cit	0
Not_GOST-cit	0	Not_GOST-cit	0
Not_GOST-cit	0	Not_GOST-cit	0
Not_GOST-cit	0	Not_GOST-cit	0

Not_GOST-cit	0	Not_GOST-cit	0
Not_GOST-cit	0	Not_GOST-cit	0
Not_GOST-cit	0	GOST-cit-Y-all	1
Not_GOST-cit	0	GOST-cit-Y-all	1
Not_GOST-cit	0	Not_GOST-cit	0
Not_GOST-cit	0	Not_GOST-cit	0
Not_GOST-cit	0	Not_GOST-cit	0
Not_GOST-cit	0	Not_GOST-cit	0
Not_GOST-cit	0	Not_GOST-cit	0
Not_GOST-cit	0	Not_GOST-cit	0
Not_GOST-cit	0	Not_GOST-cit	0
Not_GOST-cit	0	Not_GOST-cit	0
Not_GOST-cit	0	Not_GOST-cit	0
Not_GOST-cit	0	Not_GOST-cit	0
Not_GOST-cit	0	Not_GOST-cit	0
Not_GOST-cit	0	Not_GOST-cit	0
GOST-cit-all	1	GOST-cit-Y-all	1
GOST-cit-all	1	GOST-cit-Y-all	1
Not_GOST-cit	0	Not_GOST-cit	0
Not_GOST-cit	0	Not_GOST-cit	0
Not_GOST-cit	0	Not_GOST-cit	0
Not_GOST-cit	0	Not_GOST-cit	0
Not_GOST-cit	0	Not_GOST-cit	0
Not_GOST-cit	0	Not_GOST-cit	0
Not_GOST-cit	0	Not_GOST-cit	0
Not_GOST-cit	0	Not_GOST-cit	0
Not_GOST-cit	0	Not_GOST-cit	0
Not_GOST-cit	0	Not_GOST-cit	0
Not_GOST-cit	0	Not_GOST-cit	0
Not_GOST-cit	0	Not_GOST-cit	0
GOST-cit-all	1	Not_GOST-cit	0
GOST-cit-all	1	Not_GOST-cit	0
Not_GOST-cit	0	GOST-cit-Y-all	1
Not_GOST-cit	0	Not_GOST-cit	0
Not_GOST-cit	0	GOST-cit-Y-all	1
Not_GOST-cit	0	GOST-cit-Y-all	1
Not_GOST-cit	0	GOST-cit-Y-all	1
Not_GOST-cit	0	Not_GOST-cit	0
Not_GOST-cit	0	Not_GOST-cit	0
Not_GOST-cit	0	Not_GOST-cit	0
GOST-cit-all	1	GOST-cit-Y-all	1
GOST-cit-all	1	GOST-cit-Y-all	1
GOST-cit-all	1	GOST-cit-Y-all	1
GOST-cit-all	1	GOST-cit-Y-all	1
Not_GOST-cit	0	Not_GOST-cit	0
Not_GOST-cit	0	GOST-cit-Y-all	1
Not_GOST-cit	0	GOST-cit-Y-all	1

Not_GOST-cit	0	Not_GOST-cit	0
GOST-cit-all	1	Not_GOST-cit	0
GOST-cit-all	1	GOST-cit-Y-all	1
Not_GOST-cit	0	Not_GOST-cit	0
Not_GOST-cit	0	Not_GOST-cit	0
Not_GOST-cit	0	Not_GOST-cit	0
Not_GOST-cit	0	Not_GOST-cit	0
Not_GOST-cit	0	Not_GOST-cit	0
Not_GOST-cit	0	Not_GOST-cit	0
Not_GOST-cit	0	Not_GOST-cit	0
Not_GOST-cit	0	Not_GOST-cit	0
Not_GOST-cit	0	Not_GOST-cit	0
Not_GOST-cit	0	Not_GOST-cit	0
Not_GOST-cit	0	Not_GOST-cit	0
Not_GOST-cit	0	Not_GOST-cit	0
Not_GOST-cit	0	Not_GOST-cit	0
Not_GOST-cit	0	Not_GOST-cit	0
Not_GOST-cit	0	GOST-cit-Y-all	1
GOST-cit-all	1	GOST-cit-Y-all	1
GOST-cit-all	1	GOST-cit-Y-all	1
Not_GOST-cit	0	Not_GOST-cit	0
Not_GOST-cit	0	Not_GOST-cit	0
Not_GOST-cit	0	Not_GOST-cit	0
Not_GOST-cit	0	Not_GOST-cit	0
GOST-cit-all	1	GOST-cit-Y-all	1
Not_GOST-cit	0	Not_GOST-cit	0
Not_GOST-cit	0	GOST-cit-Y-all	1
Not_GOST-cit	0	Not_GOST-cit	0
Not_GOST-cit	0	Not_GOST-cit	0
Not_GOST-cit	0	Not_GOST-cit	0
Not_GOST-cit	0	Not_GOST-cit	0
Not_GOST-cit	0	Not_GOST-cit	0
Not_GOST-cit	0	Not_GOST-cit	0
Not_GOST-cit	0	Not_GOST-cit	0
Not_GOST-cit	0	Not_GOST-cit	0
Not_GOST-cit	0	Not_GOST-cit	0
Not_GOST-cit	0	Not_GOST-cit	0
Not_GOST-cit	0	Not_GOST-cit	0
Not_GOST-cit	0	Not_GOST-cit	0
GOST-cit-all	1	GOST-cit-Y-all	1
Not_GOST-cit	0	Not_GOST-cit	0
Not_GOST-cit	0	GOST-cit-Y-all	1
Not_GOST-cit	0	Not_GOST-cit	0
Not_GOST-cit	0	Not_GOST-cit	0
Not_GOST-cit	0	Not_GOST-cit	0
Not_GOST-cit	0	Not_GOST-cit	0
Not_GOST-cit	0	Not_GOST-cit	0

Not_GOST-cit	0	Not_GOST-cit	0
Not_GOST-cit	0	Not_GOST-cit	0
Not_GOST-cit	0	Not_GOST-cit	0
GOST-cit-all	1	GOST-cit-Y-all	1
Not_GOST-cit	0	GOST-cit-Y-all	1
Not_GOST-cit	0	GOST-cit-Y-all	1
Not_GOST-cit	0	Not_GOST-cit	0
Not_GOST-cit	0	Not_GOST-cit	0
Not_GOST-cit	0	Not_GOST-cit	0
Not_GOST-cit	0	Not_GOST-cit	0
Not_GOST-cit	0	Not_GOST-cit	0
Not_GOST-cit	0	Not_GOST-cit	0
Not_GOST-cit	0	Not_GOST-cit	0
Not_GOST-cit	0	Not_GOST-cit	0
Not_GOST-cit	0	GOST-cit-Y-all	1
Not_GOST-cit	0	Not_GOST-cit	0
Not_GOST-cit	0	Not_GOST-cit	0
Not_GOST-cit	0	Not_GOST-cit	0
Not_GOST-cit	0	Not_GOST-cit	0
Not_GOST-cit	0	Not_GOST-cit	0
Not_GOST-cit	0	Not_GOST-cit	0
Not_GOST-cit	0	Not_GOST-cit	0
Not_GOST-cit	0	Not_GOST-cit	0
Not_GOST-cit	0	Not_GOST-cit	0
Not_GOST-cit	0	Not_GOST-cit	0
Not_GOST-cit	0	Not_GOST-cit	0
GOST-cit-all	1	GOST-cit-Y-all	1
Not_GOST-cit	0	Not_GOST-cit	0
Not_GOST-cit	0	Not_GOST-cit	0
Not_GOST-cit	0	Not_GOST-cit	0
Not_GOST-cit	0	Not_GOST-cit	0
Not_GOST-cit	0	Not_GOST-cit	0
Not_GOST-cit	0	Not_GOST-cit	0
Not_GOST-cit	0	Not_GOST-cit	0
Not_GOST-cit	0	Not_GOST-cit	0
Not_GOST-cit	0	Not_GOST-cit	0
Not_GOST-cit	0	Not_GOST-cit	0
Not_GOST-cit	0	Not_GOST-cit	0
Not_GOST-cit	0	Not_GOST-cit	0
Not_GOST-cit	0	Not_GOST-cit	0
Not_GOST-cit	0	Not_GOST-cit	0
Not_GOST-cit	0	Not_GOST-cit	0
Not_GOST-cit	0	Not_GOST-cit	0
Not_GOST-cit	0	Not_GOST-cit	0
Not_GOST-cit	0	Not_GOST-cit	0
Not_GOST-cit	0	Not_GOST-cit	0
Not_GOST-cit	0	Not_GOST-cit	0

Not_GOST-cit	0	Not_GOST-cit	0
Not_GOST-cit	0	Not_GOST-cit	0
Not_GOST-cit	0	Not_GOST-cit	0
Not_GOST-cit	0	Not_GOST-cit	0
Not_GOST-cit	0	Not_GOST-cit	0
Not_GOST-cit	0	Not_GOST-cit	0
Not_GOST-cit	0	Not_GOST-cit	0
Not_GOST-cit	0	Not_GOST-cit	0
Not_GOST-cit	0	Not_GOST-cit	0
Not_GOST-cit	0	Not_GOST-cit	0
Not_GOST-cit	0	Not_GOST-cit	0
Not_GOST-cit	0	Not_GOST-cit	0
Not_GOST-cit	0	Not_GOST-cit	0
Not_GOST-cit	0	Not_GOST-cit	0
Not_GOST-cit	0	Not_GOST-cit	0
Not_GOST-cit	0	Not_GOST-cit	0
Not_GOST-cit	0	Not_GOST-cit	0
GOST-cit-all	1	Not_GOST-cit	0
Not_GOST-cit	0	Not_GOST-cit	0
Not_GOST-cit	0	Not_GOST-cit	0
Not_GOST-cit	0	Not_GOST-cit	0
Not_GOST-cit	0	Not_GOST-cit	0
Not_GOST-cit	0	Not_GOST-cit	0
Not_GOST-cit	0	Not_GOST-cit	0
Not_GOST-cit	0	Not_GOST-cit	0
Not_GOST-cit	0	Not_GOST-cit	0
Not_GOST-cit	0	Not_GOST-cit	0
Not_GOST-cit	0	Not_GOST-cit	0
Not_GOST-cit	0	Not_GOST-cit	0
Not_GOST-cit	0	Not_GOST-cit	0
Not_GOST-cit	0	Not_GOST-cit	0
GOST-cit-all	1	GOST-cit-Y-all	1
GOST-cit-all	1	GOST-cit-Y-all	1
GOST-cit-all	1	GOST-cit-Y-all	1
Not_GOST-cit	0	Not_GOST-cit	0
Not_GOST-cit	0	Not_GOST-cit	0
Not_GOST-cit	0	Not_GOST-cit	0
Not_GOST-cit	0	Not_GOST-cit	0
Not_GOST-cit	0	Not_GOST-cit	0
Not_GOST-cit	0	Not_GOST-cit	0
Not_GOST-cit	0	Not_GOST-cit	0
Not_GOST-cit	0	Not_GOST-cit	0
Not_GOST-cit	0	Not_GOST-cit	0
Not_GOST-cit	0	Not_GOST-cit	0
Not_GOST-cit	0	Not_GOST-cit	0
Not_GOST-cit	0	Not_GOST-cit	0
GOST-cit-all	1	GOST-cit-Y-all	1

GOST-cit-all	1	GOST-cit-Y-all	1
GOST-cit-all	1	GOST-cit-Y-all	1
Not_GOST-cit	0	Not_GOST-cit	0
Not_GOST-cit	0	Not_GOST-cit	0
Not_GOST-cit	0	Not_GOST-cit	0
Not_GOST-cit	0	Not_GOST-cit	0
Not_GOST-cit	0	Not_GOST-cit	0
Not_GOST-cit	0	Not_GOST-cit	0
Not_GOST-cit	0	Not_GOST-cit	0
Not_GOST-cit	0	Not_GOST-cit	0
Not_GOST-cit	0	Not_GOST-cit	0
Not_GOST-cit	0	Not_GOST-cit	0
Not_GOST-cit	0	Not_GOST-cit	0
Not_GOST-cit	0	Not_GOST-cit	0
Not_GOST-cit	0	Not_GOST-cit	0
Not_GOST-cit	0	Not_GOST-cit	0
Not_GOST-cit	0	Not_GOST-cit	0
Not_GOST-cit	0	Not_GOST-cit	0
Not_GOST-cit	0	Not_GOST-cit	0
Not_GOST-cit	0	Not_GOST-cit	0
Not_GOST-cit	0	Not_GOST-cit	0
Not_GOST-cit	0	GOST-cit-Y-all	1
Not_GOST-cit	0	GOST-cit-Y-all	1
Not_GOST-cit	0	Not_GOST-cit	0
Not_GOST-cit	0	Not_GOST-cit	0
Not_GOST-cit	0	Not_GOST-cit	0
GOST-cit-all	1	GOST-cit-Y-all	1
GOST-cit-all	1	GOST-cit-Y-all	1
Not_GOST-cit	0	Not_GOST-cit	0
Not_GOST-cit	0	Not_GOST-cit	0
Not_GOST-cit	0	Not_GOST-cit	0
Not_GOST-cit	0	Not_GOST-cit	0
GOST-cit-all	1	GOST-cit-Y-all	1
Not_GOST-cit	0	Not_GOST-cit	0
Not_GOST-cit	0	Not_GOST-cit	0
Not_GOST-cit	0	Not_GOST-cit	0
Not_GOST-cit	0	Not_GOST-cit	0
Not_GOST-cit	0	Not_GOST-cit	0
Not_GOST-cit	0	Not_GOST-cit	0
Not_GOST-cit	0	Not_GOST-cit	0
Not_GOST-cit	0	Not_GOST-cit	0
Not_GOST-cit	0	Not_GOST-cit	0
Not_GOST-cit	0	Not_GOST-cit	0
Not_GOST-cit	0	Not_GOST-cit	0
Not_GOST-cit	0	Not_GOST-cit	0
Not_GOST-cit	0	Not_GOST-cit	0
Not_GOST-cit	0	Not_GOST-cit	0

Not_GOST-cit	0	Not_GOST-cit	0
Not_GOST-cit	0	Not_GOST-cit	0
Not_GOST-cit	0	Not_GOST-cit	0
Not_GOST-cit	0	Not_GOST-cit	0
Not_GOST-cit	0	Not_GOST-cit	0
Not_GOST-cit	0	Not_GOST-cit	0
Not_GOST-cit	0	Not_GOST-cit	0
Not_GOST-cit	0	Not_GOST-cit	0
Not_GOST-cit	0	Not_GOST-cit	0
Not_GOST-cit	0	GOST-cit-Y-all	1
Not_GOST-cit	0	Not_GOST-cit	0
Not_GOST-cit	0	Not_GOST-cit	0
Not_GOST-cit	0	Not_GOST-cit	0
Not_GOST-cit	0	Not_GOST-cit	0
Not_GOST-cit	0	Not_GOST-cit	0
Not_GOST-cit	0	Not_GOST-cit	0
Not_GOST-cit	0	Not_GOST-cit	0
Not_GOST-cit	0	Not_GOST-cit	0
Not_GOST-cit	0	Not_GOST-cit	0
Not_GOST-cit	0	Not_GOST-cit	0
Not_GOST-cit	0	Not_GOST-cit	0
Not_GOST-cit	0	Not_GOST-cit	0
Not_GOST-cit	0	Not_GOST-cit	0
Not_GOST-cit	0	Not_GOST-cit	0
Not_GOST-cit	0	Not_GOST-cit	0
Not_GOST-cit	0	Not_GOST-cit	0
Not_GOST-cit	0	Not_GOST-cit	0
Not_GOST-cit	0	Not_GOST-cit	0
Not_GOST-cit	0	Not_GOST-cit	0
GOST-cit-all	1	Not_GOST-cit	0
GOST-cit-all	1	Not_GOST-cit	0
Not_GOST-cit	0	Not_GOST-cit	0
Not_GOST-cit	0	Not_GOST-cit	0
Not_GOST-cit	0	Not_GOST-cit	0
Not_GOST-cit	0	Not_GOST-cit	0
Not_GOST-cit	0	Not_GOST-cit	0
Not_GOST-cit	0	Not_GOST-cit	0
Not_GOST-cit	0	Not_GOST-cit	0
Not_GOST-cit	0	Not_GOST-cit	0
GOST-cit-all	1	GOST-cit-Y-all	1
Not_GOST-cit	0	Not_GOST-cit	0
Not_GOST-cit	0	Not_GOST-cit	0
Not_GOST-cit	0	Not_GOST-cit	0
Not_GOST-cit	0	Not_GOST-cit	0
Not_GOST-cit	0	Not_GOST-cit	0
Not_GOST-cit	0	Not_GOST-cit	0
Not_GOST-cit	0	Not_GOST-cit	0

Not_GOST-cit	0	Not_GOST-cit	0
Not_GOST-cit	0	Not_GOST-cit	0
Not_GOST-cit	0	Not_GOST-cit	0
Not_GOST-cit	0	Not_GOST-cit	0
Not_GOST-cit	0	Not_GOST-cit	0
Not_GOST-cit	0	Not_GOST-cit	0
Not_GOST-cit	0	Not_GOST-cit	0
Not_GOST-cit	0	Not_GOST-cit	0
Not_GOST-cit	0	Not_GOST-cit	0
Not_GOST-cit	0	Not_GOST-cit	0
Not_GOST-cit	0	Not_GOST-cit	0
Not_GOST-cit	0	Not_GOST-cit	0
Not_GOST-cit	0	Not_GOST-cit	0
Not_GOST-cit	0	Not_GOST-cit	0
Not_GOST-cit	0	Not_GOST-cit	0
Not_GOST-cit	0	Not_GOST-cit	0
Not_GOST-cit	0	Not_GOST-cit	0
Not_GOST-cit	0	Not_GOST-cit	0
Not_GOST-cit	0	Not_GOST-cit	0
Not_GOST-cit	0	Not_GOST-cit	0
Not_GOST-cit	0	Not_GOST-cit	0
Not_GOST-cit	0	Not_GOST-cit	0
Not_GOST-cit	0	Not_GOST-cit	0
Not_GOST-cit	0	Not_GOST-cit	0
Not_GOST-cit	0	Not_GOST-cit	0
Not_GOST-cit	0	Not_GOST-cit	0
Not_GOST-cit	0	Not_GOST-cit	0
Not_GOST-cit	0	Not_GOST-cit	0
Not_GOST-cit	0	GOST-cit-Y-all	1
Not_GOST-cit	0	GOST-cit-Y-all	1
Not_GOST-cit	0	GOST-cit-Y-all	1
Not_GOST-cit	0	GOST-cit-Y-all	1
Not_GOST-cit	0	Not_GOST-cit	0
Not_GOST-cit	0	Not_GOST-cit	0
Not_GOST-cit	0	Not_GOST-cit	0
Not_GOST-cit	0	Not_GOST-cit	0
Not_GOST-cit	0	Not_GOST-cit	0
GOST-cit-all	1	GOST-cit-Y-all	1
Not_GOST-cit	0	Not_GOST-cit	0
Not_GOST-cit	0	Not_GOST-cit	0
Not_GOST-cit	0	Not_GOST-cit	0
Not_GOST-cit	0	GOST-cit-Y-all	1
Not_GOST-cit	0	GOST-cit-Y-all	1
Not_GOST-cit	0	GOST-cit-Y-all	1
GOST-cit-all	1	GOST-cit-Y-all	1
GOST-cit-all	1	GOST-cit-Y-all	1
Not_GOST-cit	0	Not_GOST-cit	0

Not_GOST-cit	0	Not_GOST-cit	0
Not_GOST-cit	0	Not_GOST-cit	0
Not_GOST-cit	0	Not_GOST-cit	0
Not_GOST-cit	0	Not_GOST-cit	0
Not_GOST-cit	0	Not_GOST-cit	0
Not_GOST-cit	0	Not_GOST-cit	0
Not_GOST-cit	0	Not_GOST-cit	0
Not_GOST-cit	0	Not_GOST-cit	0
Not_GOST-cit	0	Not_GOST-cit	0
Not_GOST-cit	0	Not_GOST-cit	0
GOST-cit-all	1	GOST-cit-Y-all	1
GOST-cit-all	1	GOST-cit-Y-all	1
Not_GOST-cit	0	Not_GOST-cit	0
Not_GOST-cit	0	Not_GOST-cit	0
Not_GOST-cit	0	Not_GOST-cit	0
Not_GOST-cit	0	Not_GOST-cit	0
Not_GOST-cit	0	GOST-cit-Y-all	1
Not_GOST-cit	0	GOST-cit-Y-all	1
Not_GOST-cit	0	Not_GOST-cit	0
Not_GOST-cit	0	Not_GOST-cit	0
Not_GOST-cit	0	Not_GOST-cit	0
Not_GOST-cit	0	Not_GOST-cit	0
Not_GOST-cit	0	Not_GOST-cit	0
Not_GOST-cit	0	Not_GOST-cit	0
Not_GOST-cit	0	Not_GOST-cit	0
Not_GOST-cit	0	Not_GOST-cit	0
Not_GOST-cit	0	Not_GOST-cit	0
Not_GOST-cit	0	Not_GOST-cit	0
Not_GOST-cit	0	Not_GOST-cit	0
GOST-cit-all	1	GOST-cit-Y-all	1
GOST-cit-all	1	GOST-cit-Y-all	1
Not_GOST-cit	0	Not_GOST-cit	0
Not_GOST-cit	0	Not_GOST-cit	0
Not_GOST-cit	0	Not_GOST-cit	0
Not_GOST-cit	0	Not_GOST-cit	0
Not_GOST-cit	0	Not_GOST-cit	0
Not_GOST-cit	0	Not_GOST-cit	0
Not_GOST-cit	0	Not_GOST-cit	0
Not_GOST-cit	0	GOST-cit-Y-all	1
Not_GOST-cit	0	GOST-cit-Y-all	1
Not_GOST-cit	0	Not_GOST-cit	0
Not_GOST-cit	0	Not_GOST-cit	0
Not_GOST-cit	0	Not_GOST-cit	0
Not_GOST-cit	0	Not_GOST-cit	0
Not_GOST-cit	0	Not_GOST-cit	0
Not_GOST-cit	0	Not_GOST-cit	0
Not_GOST-cit	0	Not_GOST-cit	0

Not_GOST-cit	0	Not_GOST-cit	0
Not_GOST-cit	0	Not_GOST-cit	0
Not_GOST-cit	0	Not_GOST-cit	0
GOST-cit-all	1	GOST-cit-Y-all	1
GOST-cit-all	1	GOST-cit-Y-all	1
GOST-cit-all	1	GOST-cit-Y-all	1
Not_GOST-cit	0	Not_GOST-cit	0
Not_GOST-cit	0	Not_GOST-cit	0
Not_GOST-cit	0	Not_GOST-cit	0
Not_GOST-cit	0	Not_GOST-cit	0
Not_GOST-cit	0	Not_GOST-cit	0
Not_GOST-cit	0	Not_GOST-cit	0
Not_GOST-cit	0	Not_GOST-cit	0
Not_GOST-cit	0	Not_GOST-cit	0
Not_GOST-cit	0	Not_GOST-cit	0
Not_GOST-cit	0	Not_GOST-cit	0
Not_GOST-cit	0	Not_GOST-cit	0
Not_GOST-cit	0	Not_GOST-cit	0
Not_GOST-cit	0	Not_GOST-cit	0
GOST-cit-all	1	GOST-cit-Y-all	1
Not_GOST-cit	0	Not_GOST-cit	0
Not_GOST-cit	0	Not_GOST-cit	0
Not_GOST-cit	0	Not_GOST-cit	0
Not_GOST-cit	0	Not_GOST-cit	0
Not_GOST-cit	0	Not_GOST-cit	0
Not_GOST-cit	0	Not_GOST-cit	0
Not_GOST-cit	0	Not_GOST-cit	0
Not_GOST-cit	0	Not_GOST-cit	0
Not_GOST-cit	0	Not_GOST-cit	0
Not_GOST-cit	0	Not_GOST-cit	0
Not_GOST-cit	0	Not_GOST-cit	0
Not_GOST-cit	0	Not_GOST-cit	0
Not_GOST-cit	0	Not_GOST-cit	0
Not_GOST-cit	0	Not_GOST-cit	0
Not_GOST-cit	0	Not_GOST-cit	0
Not_GOST-cit	0	Not_GOST-cit	0
Not_GOST-cit	0	Not_GOST-cit	0
Not_GOST-cit	0	Not_GOST-cit	0
Not_GOST-cit	0	Not_GOST-cit	0
Not_GOST-cit	0	Not_GOST-cit	0
Not_GOST-cit	0	Not_GOST-cit	0
Not_GOST-cit	0	Not_GOST-cit	0
Not_GOST-cit	0	Not_GOST-cit	0
Not_GOST-cit	0	Not_GOST-cit	0
Not_GOST-cit	0	Not_GOST-cit	0
Not_GOST-cit	0	Not_GOST-cit	0
Not_GOST-cit	0	Not_GOST-cit	0

Not_GOST-cit	0	Not_GOST-cit	0
Not_GOST-cit	0	Not_GOST-cit	0
Not_GOST-cit	0	Not_GOST-cit	0
GOST-cit-all	1	GOST-cit-Y-all	1
Not_GOST-cit	0	GOST-cit-Y-all	1
GOST-cit-all	1	GOST-cit-Y-all	1
GOST-cit-all	1	GOST-cit-Y-all	1
GOST-cit-all	1	Not_GOST-cit	0
Not_GOST-cit	0	Not_GOST-cit	0
Not_GOST-cit	0	Not_GOST-cit	0
Not_GOST-cit	0	Not_GOST-cit	0
Not_GOST-cit	0	Not_GOST-cit	0
Not_GOST-cit	0	Not_GOST-cit	0
Not_GOST-cit	0	Not_GOST-cit	0
Not_GOST-cit	0	Not_GOST-cit	0
Not_GOST-cit	0	Not_GOST-cit	0
Not_GOST-cit	0	Not_GOST-cit	0
Not_GOST-cit	0	Not_GOST-cit	0
Not_GOST-cit	0	Not_GOST-cit	0
Not_GOST-cit	0	Not_GOST-cit	0
Not_GOST-cit	0	Not_GOST-cit	0
Not_GOST-cit	0	Not_GOST-cit	0
Not_GOST-cit	0	Not_GOST-cit	0
Not_GOST-cit	0	Not_GOST-cit	0
Not_GOST-cit	0	Not_GOST-cit	0
Not_GOST-cit	0	Not_GOST-cit	0
Not_GOST-cit	0	Not_GOST-cit	0
GOST-cit-all	1	GOST-cit-Y-all	1
Not_GOST-cit	0	Not_GOST-cit	0
Not_GOST-cit	0	Not_GOST-cit	0
Not_GOST-cit	0	Not_GOST-cit	0
Not_GOST-cit	0	Not_GOST-cit	0
Not_GOST-cit	0	Not_GOST-cit	0
Not_GOST-cit	0	Not_GOST-cit	0
Not_GOST-cit	0	Not_GOST-cit	0
Not_GOST-cit	0	Not_GOST-cit	0
Not_GOST-cit	0	Not_GOST-cit	0
Not_GOST-cit	0	GOST-cit-Y-all	1
Not_GOST-cit	0	GOST-cit-Y-all	1
Not_GOST-cit	0	GOST-cit-Y-all	1
Not_GOST-cit	0	Not_GOST-cit	0
Not_GOST-cit	0	Not_GOST-cit	0
Not_GOST-cit	0	Not_GOST-cit	0
Not_GOST-cit	0	Not_GOST-cit	0
Not_GOST-cit	0	Not_GOST-cit	0
Not_GOST-cit	0	Not_GOST-cit	0
Not_GOST-cit	0	Not_GOST-cit	0

Not_GOST-cit	0	Not_GOST-cit	0
Not_GOST-cit	0	Not_GOST-cit	0
Not_GOST-cit	0	Not_GOST-cit	0
Not_GOST-cit	0	Not_GOST-cit	0
Not_GOST-cit	0	Not_GOST-cit	0
Not_GOST-cit	0	Not_GOST-cit	0
Not_GOST-cit	0	Not_GOST-cit	0
Not_GOST-cit	0	Not_GOST-cit	0
Not_GOST-cit	0	Not_GOST-cit	0
Not_GOST-cit	0	Not_GOST-cit	0
Not_GOST-cit	0	Not_GOST-cit	0
Not_GOST-cit	0	Not_GOST-cit	0
GOST-cit-all	1	GOST-cit-Y-all	1
GOST-cit-all	1	GOST-cit-Y-all	1
GOST-cit-all	1	GOST-cit-Y-all	1
Not_GOST-cit	0	Not_GOST-cit	0
Not_GOST-cit	0	GOST-cit-Y-all	1
Not_GOST-cit	0	GOST-cit-Y-all	1
Not_GOST-cit	0	GOST-cit-Y-all	1
Not_GOST-cit	0	Not_GOST-cit	0
Not_GOST-cit	0	Not_GOST-cit	0
Not_GOST-cit	0	Not_GOST-cit	0
Not_GOST-cit	0	Not_GOST-cit	0
Not_GOST-cit	0	Not_GOST-cit	0
Not_GOST-cit	0	Not_GOST-cit	0
Not_GOST-cit	0	Not_GOST-cit	0
Not_GOST-cit	0	Not_GOST-cit	0
Not_GOST-cit	0	Not_GOST-cit	0
Not_GOST-cit	0	Not_GOST-cit	0
Not_GOST-cit	0	Not_GOST-cit	0
Not_GOST-cit	0	Not_GOST-cit	0
Not_GOST-cit	0	Not_GOST-cit	0
Not_GOST-cit	0	Not_GOST-cit	0
GOST-cit-all	1	GOST-cit-Y-all	1
GOST-cit-all	1	GOST-cit-Y-all	1
GOST-cit-all	1	GOST-cit-Y-all	1
GOST-cit-all	1	GOST-cit-Y-all	1
Not_GOST-cit	0	Not_GOST-cit	0
Not_GOST-cit	0	Not_GOST-cit	0
Not_GOST-cit	0	Not_GOST-cit	0
GOST-cit-all	1	GOST-cit-Y-all	1
GOST-cit-all	1	GOST-cit-Y-all	1
Not_GOST-cit	0	Not_GOST-cit	0
Not_GOST-cit	0	Not_GOST-cit	0
Not_GOST-cit	0	Not_GOST-cit	0
Not_GOST-cit	0	Not_GOST-cit	0
Not_GOST-cit	0	Not_GOST-cit	0

Not_GOST-cit	0	Not_GOST-cit	0
Not_GOST-cit	0	Not_GOST-cit	0
Not_GOST-cit	0	Not_GOST-cit	0
Not_GOST-cit	0	Not_GOST-cit	0
Not_GOST-cit	0	Not_GOST-cit	0
Not_GOST-cit	0	Not_GOST-cit	0
Not_GOST-cit	0	Not_GOST-cit	0
Not_GOST-cit	0	Not_GOST-cit	0
Not_GOST-cit	0	Not_GOST-cit	0
Not_GOST-cit	0	Not_GOST-cit	0
Not_GOST-cit	0	Not_GOST-cit	0
Not_GOST-cit	0	Not_GOST-cit	0
Not_GOST-cit	0	Not_GOST-cit	0
Not_GOST-cit	0	Not_GOST-cit	0
Not_GOST-cit	0	Not_GOST-cit	0
Not_GOST-cit	0	Not_GOST-cit	0
Not_GOST-cit	0	Not_GOST-cit	0
Not_GOST-cit	0	Not_GOST-cit	0
Not_GOST-cit	0	Not_GOST-cit	0
Not_GOST-cit	0	Not_GOST-cit	0
Not_GOST-cit	0	GOST-cit-Y-all	1
Not_GOST-cit	0	GOST-cit-Y-all	1
Not_GOST-cit	0	Not_GOST-cit	0
Not_GOST-cit	0	Not_GOST-cit	0
Not_GOST-cit	0	Not_GOST-cit	0
Not_GOST-cit	0	Not_GOST-cit	0
GOST-cit-all	1	Not_GOST-cit	0
GOST-cit-all	1	GOST-cit-Y-all	1
Not_GOST-cit	0	Not_GOST-cit	0
Not_GOST-cit	0	Not_GOST-cit	0
Not_GOST-cit	0	Not_GOST-cit	0
Not_GOST-cit	0	Not_GOST-cit	0
Not_GOST-cit	0	Not_GOST-cit	0
Not_GOST-cit	0	Not_GOST-cit	0
Not_GOST-cit	0	Not_GOST-cit	0
Not_GOST-cit	0	Not_GOST-cit	0
Not_GOST-cit	0	Not_GOST-cit	0
Not_GOST-cit	0	Not_GOST-cit	0
Not_GOST-cit	0	Not_GOST-cit	0
Not_GOST-cit	0	Not_GOST-cit	0
Not_GOST-cit	0	Not_GOST-cit	0
Not_GOST-cit	0	Not_GOST-cit	0
Not_GOST-cit	0	Not_GOST-cit	0
Not_GOST-cit	0	Not_GOST-cit	0
Not_GOST-cit	0	Not_GOST-cit	0
GOST-cit-all	1	GOST-cit-Y-all	1
GOST-cit-all	1	GOST-cit-Y-all	1

GOST-cit-all	1	GOST-cit-Y-all	1
Not_GOST-cit	0	Not_GOST-cit	0
Not_GOST-cit	0	Not_GOST-cit	0
Not_GOST-cit	0	Not_GOST-cit	0
Not_GOST-cit	0	Not_GOST-cit	0
Not_GOST-cit	0	Not_GOST-cit	0
Not_GOST-cit	0	Not_GOST-cit	0
Not_GOST-cit	0	Not_GOST-cit	0
Not_GOST-cit	0	Not_GOST-cit	0
Not_GOST-cit	0	Not_GOST-cit	0
Not_GOST-cit	0	Not_GOST-cit	0
Not_GOST-cit	0	Not_GOST-cit	0
Not_GOST-cit	0	Not_GOST-cit	0
Not_GOST-cit	0	GOST-cit-Y-all	1
Not_GOST-cit	0	GOST-cit-Y-all	1
Not_GOST-cit	0	GOST-cit-Y-all	1
Not_GOST-cit	0	GOST-cit-Y-all	1
Not_GOST-cit	0	Not_GOST-cit	0
Not_GOST-cit	0	Not_GOST-cit	0
Not_GOST-cit	0	Not_GOST-cit	0
Not_GOST-cit	0	Not_GOST-cit	0
GOST-cit-all	1	GOST-cit-Y-all	1
Not_GOST-cit	0	Not_GOST-cit	0
Not_GOST-cit	0	Not_GOST-cit	0
Not_GOST-cit	0	Not_GOST-cit	0
Not_GOST-cit	0	GOST-cit-Y-all	1
Not_GOST-cit	0	GOST-cit-Y-all	1
Not_GOST-cit	0	Not_GOST-cit	0
Not_GOST-cit	0	Not_GOST-cit	0
Not_GOST-cit	0	Not_GOST-cit	0
GOST-cit-all	1	Not_GOST-cit	0
GOST-cit-all	1	Not_GOST-cit	0
GOST-cit-all	1	GOST-cit-Y-all	1
Not_GOST-cit	0	Not_GOST-cit	0
Not_GOST-cit	0	Not_GOST-cit	0
Not_GOST-cit	0	Not_GOST-cit	0
Not_GOST-cit	0	Not_GOST-cit	0
Not_GOST-cit	0	Not_GOST-cit	0
Not_GOST-cit	0	Not_GOST-cit	0
Not_GOST-cit	0	GOST-cit-Y-all	1
Not_GOST-cit	0	Not_GOST-cit	0
Not_GOST-cit	0	Not_GOST-cit	0
Not_GOST-cit	0	Not_GOST-cit	0
Not_GOST-cit	0	Not_GOST-cit	0
Not_GOST-cit	0	Not_GOST-cit	0
Not_GOST-cit	0	Not_GOST-cit	0
Not_GOST-cit	0	Not_GOST-cit	0

GOST-cit-all	1	GOST-cit-Y-all	1
GOST-cit-all	1	GOST-cit-Y-all	1
Not_GOST-cit	0	Not_GOST-cit	0
Not_GOST-cit	0	Not_GOST-cit	0
Not_GOST-cit	0	Not_GOST-cit	0
Not_GOST-cit	0	Not_GOST-cit	0
Not_GOST-cit	0	Not_GOST-cit	0
Not_GOST-cit	0	Not_GOST-cit	0
Not_GOST-cit	0	Not_GOST-cit	0
Not_GOST-cit	0	Not_GOST-cit	0
Not_GOST-cit	0	Not_GOST-cit	0
Not_GOST-cit	0	Not_GOST-cit	0
Not_GOST-cit	0	Not_GOST-cit	0
Not_GOST-cit	0	Not_GOST-cit	0
GOST-cit-all	1	GOST-cit-Y-all	1
Not_GOST-cit	0	Not_GOST-cit	0
Not_GOST-cit	0	Not_GOST-cit	0
Not_GOST-cit	0	Not_GOST-cit	0
Not_GOST-cit	0	Not_GOST-cit	0
Not_GOST-cit	0	Not_GOST-cit	0
Not_GOST-cit	0	Not_GOST-cit	0
Not_GOST-cit	0	Not_GOST-cit	0
Not_GOST-cit	0	Not_GOST-cit	0
Not_GOST-cit	0	Not_GOST-cit	0
GOST-cit-all	1	GOST-cit-Y-all	1
GOST-cit-all	1	GOST-cit-Y-all	1
Not_GOST-cit	0	Not_GOST-cit	0
Not_GOST-cit	0	Not_GOST-cit	0
Not_GOST-cit	0	Not_GOST-cit	0
Not_GOST-cit	0	Not_GOST-cit	0
Not_GOST-cit	0	Not_GOST-cit	0
Not_GOST-cit	0	Not_GOST-cit	0
Not_GOST-cit	0	Not_GOST-cit	0
Not_GOST-cit	0	Not_GOST-cit	0
Not_GOST-cit	0	Not_GOST-cit	0
Not_GOST-cit	0	Not_GOST-cit	0
Not_GOST-cit	0	Not_GOST-cit	0
Not_GOST-cit	0	Not_GOST-cit	0
Not_GOST-cit	0	Not_GOST-cit	0
Not_GOST-cit	0	Not_GOST-cit	0
Not_GOST-cit	0	Not_GOST-cit	0
Not_GOST-cit	0	Not_GOST-cit	0
GOST-cit-all	1	Not_GOST-cit	0
Not_GOST-cit	0	Not_GOST-cit	0
Not_GOST-cit	0	Not_GOST-cit	0
Not_GOST-cit	0	Not_GOST-cit	0
Not_GOST-cit	0	Not_GOST-cit	0

Not_GOST-cit	0	Not_GOST-cit	0
Not_GOST-cit	0	Not_GOST-cit	0
Not_GOST-cit	0	Not_GOST-cit	0
Not_GOST-cit	0	Not_GOST-cit	0
Not_GOST-cit	0	Not_GOST-cit	0
Not_GOST-cit	0	Not_GOST-cit	0
Not_GOST-cit	0	Not_GOST-cit	0
Not_GOST-cit	0	Not_GOST-cit	0
Not_GOST-cit	0	Not_GOST-cit	0
Not_GOST-cit	0	Not_GOST-cit	0
Not_GOST-cit	0	GOST-cit-Y-all	1
Not_GOST-cit	0	GOST-cit-Y-all	1
Not_GOST-cit	0	Not_GOST-cit	0
Not_GOST-cit	0	Not_GOST-cit	0
Not_GOST-cit	0	Not_GOST-cit	0
Not_GOST-cit	0	Not_GOST-cit	0
Not_GOST-cit	0	Not_GOST-cit	0
Not_GOST-cit	0	Not_GOST-cit	0
Not_GOST-cit	0	Not_GOST-cit	0
Not_GOST-cit	0	Not_GOST-cit	0
Not_GOST-cit	0	Not_GOST-cit	0
Not_GOST-cit	0	Not_GOST-cit	0
GOST-cit-all	1	GOST-cit-Y-all	1
Not_GOST-cit	0	Not_GOST-cit	0
Not_GOST-cit	0	Not_GOST-cit	0
Not_GOST-cit	0	Not_GOST-cit	0
Not_GOST-cit	0	Not_GOST-cit	0
Not_GOST-cit	0	Not_GOST-cit	0
Not_GOST-cit	0	Not_GOST-cit	0
Not_GOST-cit	0	Not_GOST-cit	0
Not_GOST-cit	0	Not_GOST-cit	0
Not_GOST-cit	0	Not_GOST-cit	0
Not_GOST-cit	0	GOST-cit-Y-all	1
Not_GOST-cit	0	Not_GOST-cit	0
Not_GOST-cit	0	GOST-cit-Y-all	1
Not_GOST-cit	0	Not_GOST-cit	0
Not_GOST-cit	0	Not_GOST-cit	0
Not_GOST-cit	0	Not_GOST-cit	0
Not_GOST-cit	0	Not_GOST-cit	0
Not_GOST-cit	0	Not_GOST-cit	0
Not_GOST-cit	0	GOST-cit-Y-all	1
Not_GOST-cit	0	GOST-cit-Y-all	1
Not_GOST-cit	0	GOST-cit-Y-all	1
Not_GOST-cit	0	GOST-cit-Y-all	1
Not_GOST-cit	0	GOST-cit-Y-all	1
GOST-cit-all	1	GOST-cit-Y-all	1
GOST-cit-all	1	GOST-cit-Y-all	1

GOST-cit-all	1	GOST-cit-Y-all	1
GOST-cit-all	1	GOST-cit-Y-all	1
GOST-cit-all	1	GOST-cit-Y-all	1
Not_GOST-cit	0	Not_GOST-cit	0
Not_GOST-cit	0	Not_GOST-cit	0
Not_GOST-cit	0	Not_GOST-cit	0
Not_GOST-cit	0	Not_GOST-cit	0
Not_GOST-cit	0	Not_GOST-cit	0
Not_GOST-cit	0	Not_GOST-cit	0
Not_GOST-cit	0	Not_GOST-cit	0
Not_GOST-cit	0	Not_GOST-cit	0
Not_GOST-cit	0	Not_GOST-cit	0
Not_GOST-cit	0	Not_GOST-cit	0
Not_GOST-cit	0	Not_GOST-cit	0
Not_GOST-cit	0	Not_GOST-cit	0
Not_GOST-cit	0	Not_GOST-cit	0
Not_GOST-cit	0	Not_GOST-cit	0
Not_GOST-cit	0	GOST-cit-Y-all	1
Not_GOST-cit	0	Not_GOST-cit	0
Not_GOST-cit	0	Not_GOST-cit	0
Not_GOST-cit	0	Not_GOST-cit	0
Not_GOST-cit	0	Not_GOST-cit	0
Not_GOST-cit	0	Not_GOST-cit	0
Not_GOST-cit	0	Not_GOST-cit	0
Not_GOST-cit	0	Not_GOST-cit	0
Not_GOST-cit	0	Not_GOST-cit	0
GOST-cit-all	1	GOST-cit-Y-all	1
Not_GOST-cit	0	Not_GOST-cit	0
Not_GOST-cit	0	Not_GOST-cit	0
Not_GOST-cit	0	Not_GOST-cit	0
Not_GOST-cit	0	Not_GOST-cit	0
Not_GOST-cit	0	Not_GOST-cit	0
Not_GOST-cit	0	Not_GOST-cit	0
Not_GOST-cit	0	Not_GOST-cit	0
Not_GOST-cit	0	Not_GOST-cit	0
Not_GOST-cit	0	Not_GOST-cit	0
Not_GOST-cit	0	Not_GOST-cit	0
Not_GOST-cit	0	Not_GOST-cit	0
Not_GOST-cit	0	Not_GOST-cit	0
Not_GOST-cit	0	Not_GOST-cit	0
Not_GOST-cit	0	Not_GOST-cit	0
Not_GOST-cit	0	Not_GOST-cit	0
Not_GOST-cit	0	Not_GOST-cit	0
Not_GOST-cit	0	Not_GOST-cit	0
Not_GOST-cit	0	Not_GOST-cit	0
Not_GOST-cit	0	Not_GOST-cit	0
Not_GOST-cit	0	Not_GOST-cit	0

Not_GOST-cit	0	Not_GOST-cit	0
Not_GOST-cit	0	Not_GOST-cit	0
Not_GOST-cit	0	GOST-cit-Y-all	1
Not_GOST-cit	0	GOST-cit-Y-all	1
Not_GOST-cit	0	GOST-cit-Y-all	1
Not_GOST-cit	0	GOST-cit-Y-all	1
GOST-cit-all	1	Not_GOST-cit	0
Not_GOST-cit	0	Not_GOST-cit	0
Not_GOST-cit	0	Not_GOST-cit	0
Not_GOST-cit	0	Not_GOST-cit	0
Not_GOST-cit	0	Not_GOST-cit	0
Not_GOST-cit	0	Not_GOST-cit	0
Not_GOST-cit	0	Not_GOST-cit	0
Not_GOST-cit	0	GOST-cit-Y-all	1
Not_GOST-cit	0	GOST-cit-Y-all	1
Not_GOST-cit	0	GOST-cit-Y-all	1
Not_GOST-cit	0	Not_GOST-cit	0
Not_GOST-cit	0	Not_GOST-cit	0
Not_GOST-cit	0	Not_GOST-cit	0
Not_GOST-cit	0	Not_GOST-cit	0
Not_GOST-cit	0	Not_GOST-cit	0
Not_GOST-cit	0	Not_GOST-cit	0
Not_GOST-cit	0	Not_GOST-cit	0
Not_GOST-cit	0	Not_GOST-cit	0
Not_GOST-cit	0	Not_GOST-cit	0
Not_GOST-cit	0	Not_GOST-cit	0
Not_GOST-cit	0	Not_GOST-cit	0
Not_GOST-cit	0	Not_GOST-cit	0
Not_GOST-cit	0	Not_GOST-cit	0
Not_GOST-cit	0	Not_GOST-cit	0
Not_GOST-cit	0	Not_GOST-cit	0
Not_GOST-cit	0	GOST-cit-Y-all	1
Not_GOST-cit	0	Not_GOST-cit	0
Not_GOST-cit	0	Not_GOST-cit	0
Not_GOST-cit	0	Not_GOST-cit	0
Not_GOST-cit	0	Not_GOST-cit	0
Not_GOST-cit	0	Not_GOST-cit	0
Not_GOST-cit	0	Not_GOST-cit	0
Not_GOST-cit	0	Not_GOST-cit	0
Not_GOST-cit	0	Not_GOST-cit	0
Not_GOST-cit	0	Not_GOST-cit	0
Not_GOST-cit	0	Not_GOST-cit	0
Not_GOST-cit	0	Not_GOST-cit	0
Not_GOST-cit	0	Not_GOST-cit	0
Not_GOST-cit	0	Not_GOST-cit	0
Not_GOST-cit	0	Not_GOST-cit	0
Not_GOST-cit	0	Not_GOST-cit	0

Not_GOST-cit	0	Not_GOST-cit	0
Not_GOST-cit	0	Not_GOST-cit	0
GOST-cit-all	1	Not_GOST-cit	0
GOST-cit-all	1	Not_GOST-cit	0
GOST-cit-all	1	Not_GOST-cit	0
GOST-cit-all	1	GOST-cit-Y-all	1
GOST-cit-all	1	GOST-cit-Y-all	1
GOST-cit-all	1	GOST-cit-Y-all	1
Not_GOST-cit	0	Not_GOST-cit	0
Not_GOST-cit	0	Not_GOST-cit	0
Not_GOST-cit	0	Not_GOST-cit	0
Not_GOST-cit	0	Not_GOST-cit	0
Not_GOST-cit	0	Not_GOST-cit	0
Not_GOST-cit	0	Not_GOST-cit	0
Not_GOST-cit	0	Not_GOST-cit	0
Not_GOST-cit	0	Not_GOST-cit	0
Not_GOST-cit	0	Not_GOST-cit	0
Not_GOST-cit	0	Not_GOST-cit	0
Not_GOST-cit	0	Not_GOST-cit	0
Not_GOST-cit	0	Not_GOST-cit	0
Not_GOST-cit	0	Not_GOST-cit	0
Not_GOST-cit	0	Not_GOST-cit	0
Not_GOST-cit	0	Not_GOST-cit	0
Not_GOST-cit	0	Not_GOST-cit	0
Not_GOST-cit	0	Not_GOST-cit	0
Not_GOST-cit	0	GOST-cit-Y-all	1
Not_GOST-cit	0	GOST-cit-Y-all	1
Not_GOST-cit	0	GOST-cit-Y-all	1
Not_GOST-cit	0	GOST-cit-Y-all	1
Not_GOST-cit	0	GOST-cit-Y-all	1
Not_GOST-cit	0	Not_GOST-cit	0
GOST-cit-all	1	GOST-cit-Y-all	1
GOST-cit-all	1	GOST-cit-Y-all	1
Not_GOST-cit	0	Not_GOST-cit	0
Not_GOST-cit	0	Not_GOST-cit	0
Not_GOST-cit	0	Not_GOST-cit	0
Not_GOST-cit	0	Not_GOST-cit	0
Not_GOST-cit	0	Not_GOST-cit	0
Not_GOST-cit	0	Not_GOST-cit	0
Not_GOST-cit	0	Not_GOST-cit	0
GOST-cit-all	1	Not_GOST-cit	0
Not_GOST-cit	0	Not_GOST-cit	0
Not_GOST-cit	0	Not_GOST-cit	0
Not_GOST-cit	0	Not_GOST-cit	0
Not_GOST-cit	0	Not_GOST-cit	0
Not_GOST-cit	0	Not_GOST-cit	0
Not_GOST-cit	0	Not_GOST-cit	0

Not_GOST-cit	0	Not_GOST-cit	0
Not_GOST-cit	0	Not_GOST-cit	0
Not_GOST-cit	0	Not_GOST-cit	0
Not_GOST-cit	0	Not_GOST-cit	0
Not_GOST-cit	0	Not_GOST-cit	0
Not_GOST-cit	0	Not_GOST-cit	0
Not_GOST-cit	0	Not_GOST-cit	0
GOST-cit-all	1	GOST-cit-Y-all	1
GOST-cit-all	1	GOST-cit-Y-all	1
Not_GOST-cit	0	Not_GOST-cit	0
Not_GOST-cit	0	Not_GOST-cit	0
Not_GOST-cit	0	Not_GOST-cit	0
Not_GOST-cit	0	Not_GOST-cit	0
Not_GOST-cit	0	Not_GOST-cit	0
Not_GOST-cit	0	Not_GOST-cit	0
Not_GOST-cit	0	Not_GOST-cit	0
Not_GOST-cit	0	GOST-cit-Y-all	1
Not_GOST-cit	0	Not_GOST-cit	0
Not_GOST-cit	0	Not_GOST-cit	0
Not_GOST-cit	0	Not_GOST-cit	0
Not_GOST-cit	0	Not_GOST-cit	0
Not_GOST-cit	0	Not_GOST-cit	0
Not_GOST-cit	0	Not_GOST-cit	0
Not_GOST-cit	0	Not_GOST-cit	0
Not_GOST-cit	0	Not_GOST-cit	0
Not_GOST-cit	0	Not_GOST-cit	0
Not_GOST-cit	0	Not_GOST-cit	0
Not_GOST-cit	0	Not_GOST-cit	0
GOST-cit-all	1	Not_GOST-cit	0
GOST-cit-all	1	Not_GOST-cit	0
Not_GOST-cit	0	GOST-cit-Y-all	1
Not_GOST-cit	0	GOST-cit-Y-all	1
Not_GOST-cit	0	Not_GOST-cit	0
Not_GOST-cit	0	Not_GOST-cit	0
Not_GOST-cit	0	Not_GOST-cit	0
Not_GOST-cit	0	Not_GOST-cit	0
Not_GOST-cit	0	Not_GOST-cit	0
Not_GOST-cit	0	Not_GOST-cit	0
Not_GOST-cit	0	Not_GOST-cit	0
Not_GOST-cit	0	Not_GOST-cit	0
Not_GOST-cit	0	Not_GOST-cit	0
Not_GOST-cit	0	Not_GOST-cit	0
Not_GOST-cit	0	Not_GOST-cit	0
Not_GOST-cit	0	Not_GOST-cit	0
Not_GOST-cit	0	GOST-cit-Y-all	1
Not_GOST-cit	0	GOST-cit-Y-all	1
Not_GOST-cit	0	Not_GOST-cit	0

Not_GOST-cit	0	Not_GOST-cit	0
Not_GOST-cit	0	Not_GOST-cit	0
Not_GOST-cit	0	Not_GOST-cit	0
Not_GOST-cit	0	Not_GOST-cit	0
Not_GOST-cit	0	Not_GOST-cit	0
Not_GOST-cit	0	Not_GOST-cit	0
Not_GOST-cit	0	Not_GOST-cit	0
Not_GOST-cit	0	Not_GOST-cit	0
Not_GOST-cit	0	Not_GOST-cit	0
Not_GOST-cit	0	Not_GOST-cit	0
GOST-cit-all	1	GOST-cit-Y-all	1
Not_GOST-cit	0	Not_GOST-cit	0
Not_GOST-cit	0	Not_GOST-cit	0
Not_GOST-cit	0	Not_GOST-cit	0
Not_GOST-cit	0	Not_GOST-cit	0
Not_GOST-cit	0	Not_GOST-cit	0
Not_GOST-cit	0	Not_GOST-cit	0
Not_GOST-cit	0	Not_GOST-cit	0
Not_GOST-cit	0	Not_GOST-cit	0
Not_GOST-cit	0	Not_GOST-cit	0
Not_GOST-cit	0	Not_GOST-cit	0
Not_GOST-cit	0	Not_GOST-cit	0
Not_GOST-cit	0	Not_GOST-cit	0
Not_GOST-cit	0	Not_GOST-cit	0
Not_GOST-cit	0	Not_GOST-cit	0
Not_GOST-cit	0	Not_GOST-cit	0
Not_GOST-cit	0	Not_GOST-cit	0
Not_GOST-cit	0	Not_GOST-cit	0
Not_GOST-cit	0	Not_GOST-cit	0
Not_GOST-cit	0	Not_GOST-cit	0
Not_GOST-cit	0	Not_GOST-cit	0
Not_GOST-cit	0	Not_GOST-cit	0
Not_GOST-cit	0	Not_GOST-cit	0
Not_GOST-cit	0	Not_GOST-cit	0
Not_GOST-cit	0	Not_GOST-cit	0
Not_GOST-cit	0	Not_GOST-cit	0
Not_GOST-cit	0	Not_GOST-cit	0
Not_GOST-cit	0	Not_GOST-cit	0
Not_GOST-cit	0	Not_GOST-cit	0
Not_GOST-cit	0	Not_GOST-cit	0
Not_GOST-cit	0	GOST-cit-Y-all	1
Not_GOST-cit	0	GOST-cit-Y-all	1
Not_GOST-cit	0	GOST-cit-Y-all	1
Not_GOST-cit	0	Not_GOST-cit	0
Not_GOST-cit	0	Not_GOST-cit	0
Not_GOST-cit	0	Not_GOST-cit	0
Not_GOST-cit	0	Not_GOST-cit	0

Not_GOST-cit	0	Not_GOST-cit	0
Not_GOST-cit	0	Not_GOST-cit	0
Not_GOST-cit	0	Not_GOST-cit	0
Not_GOST-cit	0	Not_GOST-cit	0
Not_GOST-cit	0	Not_GOST-cit	0
Not_GOST-cit	0	Not_GOST-cit	0
Not_GOST-cit	0	Not_GOST-cit	0
Not_GOST-cit	0	Not_GOST-cit	0
Not_GOST-cit	0	Not_GOST-cit	0
Not_GOST-cit	0	Not_GOST-cit	0
GOST-cit-all	1	Not_GOST-cit	0
Not_GOST-cit	0	Not_GOST-cit	0
Not_GOST-cit	0	Not_GOST-cit	0
Not_GOST-cit	0	Not_GOST-cit	0
Not_GOST-cit	0	Not_GOST-cit	0
Not_GOST-cit	0	Not_GOST-cit	0
Not_GOST-cit	0	Not_GOST-cit	0
Not_GOST-cit	0	Not_GOST-cit	0
Not_GOST-cit	0	Not_GOST-cit	0
Not_GOST-cit	0	Not_GOST-cit	0
Not_GOST-cit	0	Not_GOST-cit	0
Not_GOST-cit	0	Not_GOST-cit	0
Not_GOST-cit	0	Not_GOST-cit	0
Not_GOST-cit	0	Not_GOST-cit	0
Not_GOST-cit	0	Not_GOST-cit	0
Not_GOST-cit	0	Not_GOST-cit	0
Not_GOST-cit	0	Not_GOST-cit	0
Not_GOST-cit	0	Not_GOST-cit	0
Not_GOST-cit	0	Not_GOST-cit	0
Not_GOST-cit	0	GOST-cit-Y-all	1
Not_GOST-cit	0	GOST-cit-Y-all	1
Not_GOST-cit	0	Not_GOST-cit	0
Not_GOST-cit	0	Not_GOST-cit	0
Not_GOST-cit	0	Not_GOST-cit	0
Not_GOST-cit	0	Not_GOST-cit	0
Not_GOST-cit	0	Not_GOST-cit	0
Not_GOST-cit	0	Not_GOST-cit	0
Not_GOST-cit	0	Not_GOST-cit	0
Not_GOST-cit	0	Not_GOST-cit	0
Not_GOST-cit	0	Not_GOST-cit	0
Not_GOST-cit	0	Not_GOST-cit	0
Not_GOST-cit	0	Not_GOST-cit	0
Not_GOST-cit	0	Not_GOST-cit	0
Not_GOST-cit	0	Not_GOST-cit	0
Not_GOST-cit	0	Not_GOST-cit	0
Not_GOST-cit	0	Not_GOST-cit	0
Not_GOST-cit	0	Not_GOST-cit	0

Not_GOST-cit	0	Not_GOST-cit	0
Not_GOST-cit	0	Not_GOST-cit	0
Not_GOST-cit	0	Not_GOST-cit	0
Not_GOST-cit	0	Not_GOST-cit	0
Not_GOST-cit	0	Not_GOST-cit	0
Not_GOST-cit	0	Not_GOST-cit	0
Not_GOST-cit	0	Not_GOST-cit	0
Not_GOST-cit	0	Not_GOST-cit	0
Not_GOST-cit	0	Not_GOST-cit	0
Not_GOST-cit	0	Not_GOST-cit	0
Not_GOST-cit	0	Not_GOST-cit	0
Not_GOST-cit	0	Not_GOST-cit	0
Not_GOST-cit	0	Not_GOST-cit	0
Not_GOST-cit	0	Not_GOST-cit	0
Not_GOST-cit	0	Not_GOST-cit	0
Not_GOST-cit	0	Not_GOST-cit	0
Not_GOST-cit	0	Not_GOST-cit	0
Not_GOST-cit	0	Not_GOST-cit	0
Not_GOST-cit	0	Not_GOST-cit	0
Not_GOST-cit	0	Not_GOST-cit	0
Not_GOST-cit	0	Not_GOST-cit	0
Not_GOST-cit	0	Not_GOST-cit	0
Not_GOST-cit	0	Not_GOST-cit	0
Not_GOST-cit	0	Not_GOST-cit	0
Not_GOST-cit	0	Not_GOST-cit	0
Not_GOST-cit	0	Not_GOST-cit	0
Not_GOST-cit	0	Not_GOST-cit	0
Not_GOST-cit	0	Not_GOST-cit	0
Not_GOST-cit	0	Not_GOST-cit	0
Not_GOST-cit	0	Not_GOST-cit	0
Not_GOST-cit	0	Not_GOST-cit	0
Not_GOST-cit	0	Not_GOST-cit	0
Not_GOST-cit	0	Not_GOST-cit	0
Not_GOST-cit	0	Not_GOST-cit	0
Not_GOST-cit	0	Not_GOST-cit	0
Not_GOST-cit	0	Not_GOST-cit	0
Not_GOST-cit	0	Not_GOST-cit	0
Not_GOST-cit	0	Not_GOST-cit	0
Not_GOST-cit	0	Not_GOST-cit	0
Not_GOST-cit	0	Not_GOST-cit	0
Not_GOST-cit	0	Not_GOST-cit	0
Not_GOST-cit	0	Not_GOST-cit	0
Not_GOST-cit	0	Not_GOST-cit	0
Not_GOST-cit	0	Not_GOST-cit	0
Not_GOST-cit	0	Not_GOST-cit	0
Not_GOST-cit	0	Not_GOST-cit	0
Not_GOST-cit	0	Not_GOST-cit	0

Not_GOST-cit	0	Not_GOST-cit	0
Not_GOST-cit	0	Not_GOST-cit	0
Not_GOST-cit	0	Not_GOST-cit	0
Not_GOST-cit	0	Not_GOST-cit	0
GOST-cit-all	1	GOST-cit-Y-all	1
Not_GOST-cit	0	Not_GOST-cit	0
Not_GOST-cit	0	Not_GOST-cit	0
Not_GOST-cit	0	Not_GOST-cit	0
Not_GOST-cit	0	Not_GOST-cit	0
Not_GOST-cit	0	Not_GOST-cit	0
Not_GOST-cit	0	Not_GOST-cit	0
Not_GOST-cit	0	Not_GOST-cit	0
Not_GOST-cit	0	Not_GOST-cit	0
Not_GOST-cit	0	Not_GOST-cit	0
Not_GOST-cit	0	Not_GOST-cit	0
Not_GOST-cit	0	Not_GOST-cit	0
Not_GOST-cit	0	Not_GOST-cit	0
Not_GOST-cit	0	Not_GOST-cit	0
Not_GOST-cit	0	Not_GOST-cit	0
Not_GOST-cit	0	Not_GOST-cit	0
Not_GOST-cit	0	Not_GOST-cit	0
Not_GOST-cit	0	Not_GOST-cit	0
Not_GOST-cit	0	Not_GOST-cit	0
Not_GOST-cit	0	Not_GOST-cit	0
Not_GOST-cit	0	Not_GOST-cit	0
Not_GOST-cit	0	Not_GOST-cit	0
Not_GOST-cit	0	Not_GOST-cit	0
Not_GOST-cit	0	Not_GOST-cit	0
Not_GOST-cit	0	Not_GOST-cit	0
Not_GOST-cit	0	Not_GOST-cit	0
Not_GOST-cit	0	Not_GOST-cit	0
Not_GOST-cit	0	Not_GOST-cit	0
Not_GOST-cit	0	Not_GOST-cit	0
Not_GOST-cit	0	Not_GOST-cit	0
Not_GOST-cit	0	Not_GOST-cit	0
Not_GOST-cit	0	Not_GOST-cit	0
Not_GOST-cit	0	Not_GOST-cit	0
Not_GOST-cit	0	Not_GOST-cit	0
Not_GOST-cit	0	Not_GOST-cit	0
Not_GOST-cit	0	Not_GOST-cit	0
Not_GOST-cit	0	Not_GOST-cit	0
Not_GOST-cit	0	Not_GOST-cit	0
Not_GOST-cit	0	Not_GOST-cit	0
Not_GOST-cit	0	Not_GOST-cit	0
Not_GOST-cit	0	Not_GOST-cit	0
Not_GOST-cit	0	Not_GOST-cit	0
Not_GOST-cit	0	Not_GOST-cit	0

Not_GOST-cit	0	Not_GOST-cit	0
Not_GOST-cit	0	Not_GOST-cit	0
Not_GOST-cit	0	Not_GOST-cit	0
Not_GOST-cit	0	Not_GOST-cit	0
Not_GOST-cit	0	Not_GOST-cit	0
Not_GOST-cit	0	Not_GOST-cit	0
Not_GOST-cit	0	Not_GOST-cit	0
Not_GOST-cit	0	Not_GOST-cit	0
Not_GOST-cit	0	Not_GOST-cit	0
Not_GOST-cit	0	Not_GOST-cit	0
Not_GOST-cit	0	Not_GOST-cit	0
Not_GOST-cit	0	Not_GOST-cit	0
Not_GOST-cit	0	Not_GOST-cit	0
Not_GOST-cit	0	Not_GOST-cit	0
Not_GOST-cit	0	Not_GOST-cit	0
Not_GOST-cit	0	Not_GOST-cit	0
Not_GOST-cit	0	Not_GOST-cit	0
Not_GOST-cit	0	Not_GOST-cit	0
Not_GOST-cit	0	Not_GOST-cit	0
Not_GOST-cit	0	GOST-cit-Y-all	1
Not_GOST-cit	0	GOST-cit-Y-all	1
Not_GOST-cit	0	GOST-cit-Y-all	1
Not_GOST-cit	0	Not_GOST-cit	0
Not_GOST-cit	0	Not_GOST-cit	0
Not_GOST-cit	0	Not_GOST-cit	0
Not_GOST-cit	0	Not_GOST-cit	0
Not_GOST-cit	0	Not_GOST-cit	0
Not_GOST-cit	0	Not_GOST-cit	0
Not_GOST-cit	0	Not_GOST-cit	0
Not_GOST-cit	0	Not_GOST-cit	0
Not_GOST-cit	0	Not_GOST-cit	0
Not_GOST-cit	0	Not_GOST-cit	0
Not_GOST-cit	0	Not_GOST-cit	0
Not_GOST-cit	0	Not_GOST-cit	0
Not_GOST-cit	0	Not_GOST-cit	0
Not_GOST-cit	0	Not_GOST-cit	0
Not_GOST-cit	0	Not_GOST-cit	0
Not_GOST-cit	0	Not_GOST-cit	0
Not_GOST-cit	0	Not_GOST-cit	0
Not_GOST-cit	0	Not_GOST-cit	0
Not_GOST-cit	0	Not_GOST-cit	0
Not_GOST-cit	0	Not_GOST-cit	0
Not_GOST-cit	0	Not_GOST-cit	0
Not_GOST-cit	0	Not_GOST-cit	0
Not_GOST-cit	0	Not_GOST-cit	0
Not_GOST-cit	0	Not_GOST-cit	0
Not_GOST-cit	0	Not_GOST-cit	0

Not_GOST-cit	0	Not_GOST-cit	0
Not_GOST-cit	0	Not_GOST-cit	0
Not_GOST-cit	0	Not_GOST-cit	0
Not_GOST-cit	0	Not_GOST-cit	0
Not_GOST-cit	0	Not_GOST-cit	0
Not_GOST-cit	0	Not_GOST-cit	0
Not_GOST-cit	0	Not_GOST-cit	0
Not_GOST-cit	0	Not_GOST-cit	0
Not_GOST-cit	0	Not_GOST-cit	0
Not_GOST-cit	0	Not_GOST-cit	0
Not_GOST-cit	0	Not_GOST-cit	0
Not_GOST-cit	0	GOST-cit-Y-all	1
Not_GOST-cit	0	GOST-cit-Y-all	1
Not_GOST-cit	0	Not_GOST-cit	0
Not_GOST-cit	0	Not_GOST-cit	0
Not_GOST-cit	0	Not_GOST-cit	0
Not_GOST-cit	0	GOST-cit-Y-all	1
Not_GOST-cit	0	GOST-cit-Y-all	1
Not_GOST-cit	0	Not_GOST-cit	0
Not_GOST-cit	0	Not_GOST-cit	0
Not_GOST-cit	0	Not_GOST-cit	0
Not_GOST-cit	0	Not_GOST-cit	0
Not_GOST-cit	0	Not_GOST-cit	0
Not_GOST-cit	0	Not_GOST-cit	0
Not_GOST-cit	0	Not_GOST-cit	0
Not_GOST-cit	0	Not_GOST-cit	0
Not_GOST-cit	0	Not_GOST-cit	0
Not_GOST-cit	0	Not_GOST-cit	0
Not_GOST-cit	0	Not_GOST-cit	0
Not_GOST-cit	0	Not_GOST-cit	0
Not_GOST-cit	0	Not_GOST-cit	0
Not_GOST-cit	0	Not_GOST-cit	0
Not_GOST-cit	0	Not_GOST-cit	0
Not_GOST-cit	0	Not_GOST-cit	0
GOST-cit-all	1	Not_GOST-cit	0
Not_GOST-cit	0	Not_GOST-cit	0
Not_GOST-cit	0	Not_GOST-cit	0
Not_GOST-cit	0	Not_GOST-cit	0
Not_GOST-cit	0	Not_GOST-cit	0
Not_GOST-cit	0	Not_GOST-cit	0
Not_GOST-cit	0	Not_GOST-cit	0
Not_GOST-cit	0	Not_GOST-cit	0
Not_GOST-cit	0	Not_GOST-cit	0
Not_GOST-cit	0	Not_GOST-cit	0
GOST-cit-all	1	GOST-cit-Y-all	1
Not_GOST-cit	0	Not_GOST-cit	0
Not_GOST-cit	0	Not_GOST-cit	0

Not_GOST-cit	0	Not_GOST-cit	0
Not_GOST-cit	0	Not_GOST-cit	0
Not_GOST-cit	0	Not_GOST-cit	0
Not_GOST-cit	0	Not_GOST-cit	0
Not_GOST-cit	0	Not_GOST-cit	0
Not_GOST-cit	0	Not_GOST-cit	0
GOST-cit-all	1	Not_GOST-cit	0
Not_GOST-cit	0	Not_GOST-cit	0
Not_GOST-cit	0	Not_GOST-cit	0
Not_GOST-cit	0	Not_GOST-cit	0
Not_GOST-cit	0	Not_GOST-cit	0
Not_GOST-cit	0	GOST-cit-Y-all	1
Not_GOST-cit	0	GOST-cit-Y-all	1
Not_GOST-cit	0	Not_GOST-cit	0
Not_GOST-cit	0	Not_GOST-cit	0
Not_GOST-cit	0	Not_GOST-cit	0
Not_GOST-cit	0	Not_GOST-cit	0
Not_GOST-cit	0	Not_GOST-cit	0
Not_GOST-cit	0	Not_GOST-cit	0
Not_GOST-cit	0	Not_GOST-cit	0
Not_GOST-cit	0	Not_GOST-cit	0
GOST-cit-all	1	GOST-cit-Y-all	1
GOST-cit-all	1	GOST-cit-Y-all	1
GOST-cit-all	1	GOST-cit-Y-all	1
Not_GOST-cit	0	Not_GOST-cit	0
Not_GOST-cit	0	Not_GOST-cit	0
Not_GOST-cit	0	Not_GOST-cit	0
Not_GOST-cit	0	Not_GOST-cit	0
Not_GOST-cit	0	Not_GOST-cit	0
Not_GOST-cit	0	Not_GOST-cit	0
Not_GOST-cit	0	Not_GOST-cit	0
Not_GOST-cit	0	Not_GOST-cit	0
Not_GOST-cit	0	Not_GOST-cit	0
Not_GOST-cit	0	Not_GOST-cit	0
Not_GOST-cit	0	Not_GOST-cit	0
Not_GOST-cit	0	Not_GOST-cit	0
Not_GOST-cit	0	Not_GOST-cit	0
Not_GOST-cit	0	Not_GOST-cit	0
Not_GOST-cit	0	Not_GOST-cit	0
Not_GOST-cit	0	Not_GOST-cit	0
Not_GOST-cit	0	Not_GOST-cit	0
Not_GOST-cit	0	Not_GOST-cit	0
Not_GOST-cit	0	Not_GOST-cit	0
Not_GOST-cit	0	Not_GOST-cit	0
Not_GOST-cit	0	Not_GOST-cit	0
Not_GOST-cit	0	Not_GOST-cit	0
Not_GOST-cit	0	Not_GOST-cit	0

Not_GOST-cit	0	Not_GOST-cit	0
Not_GOST-cit	0	Not_GOST-cit	0
Not_GOST-cit	0	Not_GOST-cit	0
Not_GOST-cit	0	Not_GOST-cit	0
Not_GOST-cit	0	Not_GOST-cit	0
Not_GOST-cit	0	Not_GOST-cit	0
Not_GOST-cit	0	Not_GOST-cit	0
GOST-cit-all	1	GOST-cit-Y-all	1
GOST-cit-all	1	GOST-cit-Y-all	1
GOST-cit-all	1	GOST-cit-Y-all	1
GOST-cit-all	1	GOST-cit-Y-all	1
GOST-cit-all	1	GOST-cit-Y-all	1
GOST-cit-all	1	GOST-cit-Y-all	1
GOST-cit-all	1	GOST-cit-Y-all	1
Not_GOST-cit	0	Not_GOST-cit	0
Not_GOST-cit	0	Not_GOST-cit	0
Not_GOST-cit	0	Not_GOST-cit	0
Not_GOST-cit	0	Not_GOST-cit	0
Not_GOST-cit	0	Not_GOST-cit	0
Not_GOST-cit	0	Not_GOST-cit	0
Not_GOST-cit	0	Not_GOST-cit	0
Not_GOST-cit	0	Not_GOST-cit	0
Not_GOST-cit	0	Not_GOST-cit	0
Not_GOST-cit	0	Not_GOST-cit	0
Not_GOST-cit	0	Not_GOST-cit	0
Not_GOST-cit	0	Not_GOST-cit	0
Not_GOST-cit	0	Not_GOST-cit	0
Not_GOST-cit	0	Not_GOST-cit	0
Not_GOST-cit	0	Not_GOST-cit	0
Not_GOST-cit	0	Not_GOST-cit	0
Not_GOST-cit	0	Not_GOST-cit	0
Not_GOST-cit	0	Not_GOST-cit	0
Not_GOST-cit	0	Not_GOST-cit	0
Not_GOST-cit	0	Not_GOST-cit	0
Not_GOST-cit	0	Not_GOST-cit	0
Not_GOST-cit	0	Not_GOST-cit	0
Not_GOST-cit	0	Not_GOST-cit	0
Not_GOST-cit	0	Not_GOST-cit	0
Not_GOST-cit	0	Not_GOST-cit	0
Not_GOST-cit	0	Not_GOST-cit	0
Not_GOST-cit	0	Not_GOST-cit	0
Not_GOST-cit	0	Not_GOST-cit	0
Not_GOST-cit	0	Not_GOST-cit	0
Not_GOST-cit	0	Not_GOST-cit	0
Not_GOST-cit	0	Not_GOST-cit	0
Not_GOST-cit	0	GOST-cit-Y-all	1
Not_GOST-cit	0	GOST-cit-Y-all	1
Not_GOST-cit	0	GOST-cit-Y-all	1
Not_GOST-cit	0	Not_GOST-cit	0

Not_GOST-cit	0	Not_GOST-cit	0
Not_GOST-cit	0	Not_GOST-cit	0
Not_GOST-cit	0	Not_GOST-cit	0
Not_GOST-cit	0	Not_GOST-cit	0
Not_GOST-cit	0	Not_GOST-cit	0
Not_GOST-cit	0	Not_GOST-cit	0
Not_GOST-cit	0	Not_GOST-cit	0
GOST-cit-all	1	GOST-cit-Y-all	1
GOST-cit-all	1	GOST-cit-Y-all	1
Not_GOST-cit	0	Not_GOST-cit	0
Not_GOST-cit	0	Not_GOST-cit	0
Not_GOST-cit	0	Not_GOST-cit	0
GOST-cit-all	1	GOST-cit-Y-all	1
Not_GOST-cit	0	Not_GOST-cit	0
Not_GOST-cit	0	Not_GOST-cit	0
Not_GOST-cit	0	Not_GOST-cit	0
Not_GOST-cit	0	Not_GOST-cit	0
Not_GOST-cit	0	Not_GOST-cit	0
Not_GOST-cit	0	Not_GOST-cit	0
Not_GOST-cit	0	Not_GOST-cit	0
Not_GOST-cit	0	Not_GOST-cit	0
Not_GOST-cit	0	Not_GOST-cit	0
Not_GOST-cit	0	Not_GOST-cit	0
Not_GOST-cit	0	Not_GOST-cit	0
GOST-cit-all	1	GOST-cit-Y-all	1
Not_GOST-cit	0	Not_GOST-cit	0
Not_GOST-cit	0	Not_GOST-cit	0
Not_GOST-cit	0	Not_GOST-cit	0
Not_GOST-cit	0	Not_GOST-cit	0
Not_GOST-cit	0	Not_GOST-cit	0
Not_GOST-cit	0	Not_GOST-cit	0
Not_GOST-cit	0	Not_GOST-cit	0
Not_GOST-cit	0	Not_GOST-cit	0
Not_GOST-cit	0	Not_GOST-cit	0
Not_GOST-cit	0	Not_GOST-cit	0
Not_GOST-cit	0	Not_GOST-cit	0
Not_GOST-cit	0	Not_GOST-cit	0
Not_GOST-cit	0	Not_GOST-cit	0
Not_GOST-cit	0	Not_GOST-cit	0
Not_GOST-cit	0	Not_GOST-cit	0
Not_GOST-cit	0	Not_GOST-cit	0
Not_GOST-cit	0	Not_GOST-cit	0
GOST-cit-all	1	GOST-cit-Y-all	1
GOST-cit-all	1	GOST-cit-Y-all	1
GOST-cit-all	1	GOST-cit-Y-all	1
GOST-cit-all	1	GOST-cit-Y-all	1
GOST-cit-all	1	GOST-cit-Y-all	1

Not_GOST-cit	0	Not_GOST-cit	0
Not_GOST-cit	0	Not_GOST-cit	0
Not_GOST-cit	0	Not_GOST-cit	0
Not_GOST-cit	0	Not_GOST-cit	0
Not_GOST-cit	0	Not_GOST-cit	0
Not_GOST-cit	0	Not_GOST-cit	0
Not_GOST-cit	0	Not_GOST-cit	0
Not_GOST-cit	0	Not_GOST-cit	0
Not_GOST-cit	0	Not_GOST-cit	0
Not_GOST-cit	0	Not_GOST-cit	0
Not_GOST-cit	0	Not_GOST-cit	0
Not_GOST-cit	0	GOST-cit-Y-all	1
Not_GOST-cit	0	Not_GOST-cit	0
Not_GOST-cit	0	Not_GOST-cit	0
Not_GOST-cit	0	Not_GOST-cit	0
Not_GOST-cit	0	Not_GOST-cit	0
Not_GOST-cit	0	Not_GOST-cit	0
Not_GOST-cit	0	Not_GOST-cit	0
Not_GOST-cit	0	Not_GOST-cit	0
Not_GOST-cit	0	Not_GOST-cit	0
Not_GOST-cit	0	Not_GOST-cit	0
Not_GOST-cit	0	Not_GOST-cit	0
Not_GOST-cit	0	Not_GOST-cit	0
Not_GOST-cit	0	Not_GOST-cit	0
Not_GOST-cit	0	Not_GOST-cit	0
Not_GOST-cit	0	Not_GOST-cit	0
Not_GOST-cit	0	Not_GOST-cit	0
Not_GOST-cit	0	Not_GOST-cit	0
Not_GOST-cit	0	Not_GOST-cit	0
Not_GOST-cit	0	Not_GOST-cit	0
Not_GOST-cit	0	Not_GOST-cit	0
Not_GOST-cit	0	Not_GOST-cit	0
Not_GOST-cit	0	Not_GOST-cit	0
Not_GOST-cit	0	Not_GOST-cit	0
Not_GOST-cit	0	GOST-cit-Y-all	1
Not_GOST-cit	0	GOST-cit-Y-all	1
Not_GOST-cit	0	Not_GOST-cit	0
Not_GOST-cit	0	Not_GOST-cit	0
Not_GOST-cit	0	Not_GOST-cit	0
Not_GOST-cit	0	Not_GOST-cit	0
Not_GOST-cit	0	Not_GOST-cit	0
Not_GOST-cit	0	Not_GOST-cit	0
Not_GOST-cit	0	Not_GOST-cit	0
Not_GOST-cit	0	Not_GOST-cit	0
Not_GOST-cit	0	Not_GOST-cit	0
Not_GOST-cit	0	Not_GOST-cit	0
Not_GOST-cit	0	Not_GOST-cit	0

Not_GOST-cit	0	Not_GOST-cit	0
Not_GOST-cit	0	Not_GOST-cit	0
Not_GOST-cit	0	Not_GOST-cit	0
Not_GOST-cit	0	Not_GOST-cit	0
Not_GOST-cit	0	Not_GOST-cit	0
Not_GOST-cit	0	Not_GOST-cit	0
Not_GOST-cit	0	Not_GOST-cit	0
Not_GOST-cit	0	Not_GOST-cit	0
GOST-cit-all	1	GOST-cit-Y-all	1
GOST-cit-all	1	GOST-cit-Y-all	1
GOST-cit-all	1	GOST-cit-Y-all	1
GOST-cit-all	1	GOST-cit-Y-all	1
GOST-cit-all	1	GOST-cit-Y-all	1
Not_GOST-cit	0	Not_GOST-cit	0
Not_GOST-cit	0	Not_GOST-cit	0
Not_GOST-cit	0	Not_GOST-cit	0
Not_GOST-cit	0	Not_GOST-cit	0
Not_GOST-cit	0	Not_GOST-cit	0
Not_GOST-cit	0	Not_GOST-cit	0
Not_GOST-cit	0	Not_GOST-cit	0
Not_GOST-cit	0	Not_GOST-cit	0
Not_GOST-cit	0	Not_GOST-cit	0
Not_GOST-cit	0	Not_GOST-cit	0
Not_GOST-cit	0	Not_GOST-cit	0
Not_GOST-cit	0	Not_GOST-cit	0
Not_GOST-cit	0	Not_GOST-cit	0
Not_GOST-cit	0	Not_GOST-cit	0
Not_GOST-cit	0	Not_GOST-cit	0
Not_GOST-cit	0	Not_GOST-cit	0
Not_GOST-cit	0	Not_GOST-cit	0
Not_GOST-cit	0	Not_GOST-cit	0
Not_GOST-cit	0	Not_GOST-cit	0
Not_GOST-cit	0	Not_GOST-cit	0
Not_GOST-cit	0	Not_GOST-cit	0
Not_GOST-cit	0	Not_GOST-cit	0
Not_GOST-cit	0	Not_GOST-cit	0
Not_GOST-cit	0	Not_GOST-cit	0
Not_GOST-cit	0	Not_GOST-cit	0
GOST-cit-all	1	Not_GOST-cit	0
GOST-cit-all	1	GOST-cit-Y-all	1
Not_GOST-cit	0	Not_GOST-cit	0
Not_GOST-cit	0	Not_GOST-cit	0
Not_GOST-cit	0	Not_GOST-cit	0
Not_GOST-cit	0	Not_GOST-cit	0
Not_GOST-cit	0	Not_GOST-cit	0
Not_GOST-cit	0	Not_GOST-cit	0
Not_GOST-cit	0	Not_GOST-cit	0

Not_GOST-cit	0	Not_GOST-cit	0
GOST-cit-all	1	GOST-cit-Y-all	1
Not_GOST-cit	0	Not_GOST-cit	0
Not_GOST-cit	0	Not_GOST-cit	0
Not_GOST-cit	0	Not_GOST-cit	0
Not_GOST-cit	0	Not_GOST-cit	0
Not_GOST-cit	0	Not_GOST-cit	0
Not_GOST-cit	0	Not_GOST-cit	0
Not_GOST-cit	0	Not_GOST-cit	0
Not_GOST-cit	0	Not_GOST-cit	0
Not_GOST-cit	0	Not_GOST-cit	0
Not_GOST-cit	0	Not_GOST-cit	0
Not_GOST-cit	0	Not_GOST-cit	0
Not_GOST-cit	0	Not_GOST-cit	0
Not_GOST-cit	0	Not_GOST-cit	0
Not_GOST-cit	0	Not_GOST-cit	0
Not_GOST-cit	0	Not_GOST-cit	0
Not_GOST-cit	0	Not_GOST-cit	0
Not_GOST-cit	0	Not_GOST-cit	0
Not_GOST-cit	0	Not_GOST-cit	0
Not_GOST-cit	0	Not_GOST-cit	0
Not_GOST-cit	0	Not_GOST-cit	0
Not_GOST-cit	0	Not_GOST-cit	0
Not_GOST-cit	0	Not_GOST-cit	0
Not_GOST-cit	0	Not_GOST-cit	0
Not_GOST-cit	0	Not_GOST-cit	0
Not_GOST-cit	0	Not_GOST-cit	0
Not_GOST-cit	0	GOST-cit-Y-all	1
Not_GOST-cit	0	GOST-cit-Y-all	1
Not_GOST-cit	0	Not_GOST-cit	0
Not_GOST-cit	0	Not_GOST-cit	0
Not_GOST-cit	0	Not_GOST-cit	0
Not_GOST-cit	0	Not_GOST-cit	0
Not_GOST-cit	0	Not_GOST-cit	0
Not_GOST-cit	0	Not_GOST-cit	0
Not_GOST-cit	0	Not_GOST-cit	0
Not_GOST-cit	0	Not_GOST-cit	0
Not_GOST-cit	0	Not_GOST-cit	0
Not_GOST-cit	0	Not_GOST-cit	0
Not_GOST-cit	0	Not_GOST-cit	0
Not_GOST-cit	0	Not_GOST-cit	0
Not_GOST-cit	0	Not_GOST-cit	0
Not_GOST-cit	0	Not_GOST-cit	0
Not_GOST-cit	0	Not_GOST-cit	0
Not_GOST-cit	0	Not_GOST-cit	0
Not_GOST-cit	0	Not_GOST-cit	0
Not_GOST-cit	0	Not_GOST-cit	0

Not_GOST-cit	0	Not_GOST-cit	0
Not_GOST-cit	0	Not_GOST-cit	0
Not_GOST-cit	0	Not_GOST-cit	0
Not_GOST-cit	0	Not_GOST-cit	0
Not_GOST-cit	0	Not_GOST-cit	0
Not_GOST-cit	0	Not_GOST-cit	0
Not_GOST-cit	0	Not_GOST-cit	0
Not_GOST-cit	0	Not_GOST-cit	0
Not_GOST-cit	0	Not_GOST-cit	0
Not_GOST-cit	0	Not_GOST-cit	0
Not_GOST-cit	0	Not_GOST-cit	0
Not_GOST-cit	0	Not_GOST-cit	0
Not_GOST-cit	0	Not_GOST-cit	0
Not_GOST-cit	0	Not_GOST-cit	0
Not_GOST-cit	0	Not_GOST-cit	0
Not_GOST-cit	0	Not_GOST-cit	0
Not_GOST-cit	0	Not_GOST-cit	0
Not_GOST-cit	0	Not_GOST-cit	0
Not_GOST-cit	0	GOST-cit-Y-all	1
Not_GOST-cit	0	GOST-cit-Y-all	1
Not_GOST-cit	0	GOST-cit-Y-all	1
Not_GOST-cit	0	Not_GOST-cit	0
Not_GOST-cit	0	Not_GOST-cit	0
Not_GOST-cit	0	Not_GOST-cit	0
Not_GOST-cit	0	Not_GOST-cit	0
Not_GOST-cit	0	Not_GOST-cit	0
Not_GOST-cit	0	Not_GOST-cit	0
Not_GOST-cit	0	GOST-cit-Y-all	1
Not_GOST-cit	0	Not_GOST-cit	0
Not_GOST-cit	0	Not_GOST-cit	0
Not_GOST-cit	0	Not_GOST-cit	0
Not_GOST-cit	0	Not_GOST-cit	0
Not_GOST-cit	0	Not_GOST-cit	0
Not_GOST-cit	0	Not_GOST-cit	0
Not_GOST-cit	0	Not_GOST-cit	0
Not_GOST-cit	0	Not_GOST-cit	0
Not_GOST-cit	0	Not_GOST-cit	0
Not_GOST-cit	0	Not_GOST-cit	0
Not_GOST-cit	0	Not_GOST-cit	0
Not_GOST-cit	0	Not_GOST-cit	0
Not_GOST-cit	0	Not_GOST-cit	0
Not_GOST-cit	0	Not_GOST-cit	0
Not_GOST-cit	0	Not_GOST-cit	0
Not_GOST-cit	0	Not_GOST-cit	0
Not_GOST-cit	0	Not_GOST-cit	0
Not_GOST-cit	0	Not_GOST-cit	0
Not_GOST-cit	0	Not_GOST-cit	0

Not_GOST-cit	0	Not_GOST-cit	0
Not_GOST-cit	0	Not_GOST-cit	0
Not_GOST-cit	0	Not_GOST-cit	0
Not_GOST-cit	0	Not_GOST-cit	0
Not_GOST-cit	0	Not_GOST-cit	0
Not_GOST-cit	0	Not_GOST-cit	0
Not_GOST-cit	0	Not_GOST-cit	0
Not_GOST-cit	0	Not_GOST-cit	0
Not_GOST-cit	0	Not_GOST-cit	0
Not_GOST-cit	0	Not_GOST-cit	0
Not_GOST-cit	0	Not_GOST-cit	0
Not_GOST-cit	0	Not_GOST-cit	0
GOST-cit-all	1	GOST-cit-Y-all	1
GOST-cit-all	1	GOST-cit-Y-all	1
GOST-cit-all	1	GOST-cit-Y-all	1
Not_GOST-cit	0	Not_GOST-cit	0
Not_GOST-cit	0	Not_GOST-cit	0
Not_GOST-cit	0	Not_GOST-cit	0
Not_GOST-cit	0	Not_GOST-cit	0
Not_GOST-cit	0	Not_GOST-cit	0
Not_GOST-cit	0	Not_GOST-cit	0
Not_GOST-cit	0	Not_GOST-cit	0
Not_GOST-cit	0	Not_GOST-cit	0
Not_GOST-cit	0	Not_GOST-cit	0
Not_GOST-cit	0	Not_GOST-cit	0
Not_GOST-cit	0	Not_GOST-cit	0
Not_GOST-cit	0	Not_GOST-cit	0
Not_GOST-cit	0	Not_GOST-cit	0
Not_GOST-cit	0	Not_GOST-cit	0
Not_GOST-cit	0	Not_GOST-cit	0
Not_GOST-cit	0	Not_GOST-cit	0
Not_GOST-cit	0	Not_GOST-cit	0
Not_GOST-cit	0	Not_GOST-cit	0
Not_GOST-cit	0	Not_GOST-cit	0
Not_GOST-cit	0	GOST-cit-Y-all	1
Not_GOST-cit	0	GOST-cit-Y-all	1
Not_GOST-cit	0	GOST-cit-Y-all	1
Not_GOST-cit	0	Not_GOST-cit	0
Not_GOST-cit	0	Not_GOST-cit	0
Not_GOST-cit	0	Not_GOST-cit	0
Not_GOST-cit	0	Not_GOST-cit	0
Not_GOST-cit	0	Not_GOST-cit	0
Not_GOST-cit	0	Not_GOST-cit	0
Not_GOST-cit	0	Not_GOST-cit	0
Not_GOST-cit	0	Not_GOST-cit	0
Not_GOST-cit	0	Not_GOST-cit	0
Not_GOST-cit	0	Not_GOST-cit	0

Not_GOST-cit	0	Not_GOST-cit	0
Not_GOST-cit	0	Not_GOST-cit	0
Not_GOST-cit	0	Not_GOST-cit	0
Not_GOST-cit	0	Not_GOST-cit	0
Not_GOST-cit	0	Not_GOST-cit	0
Not_GOST-cit	0	Not_GOST-cit	0
Not_GOST-cit	0	Not_GOST-cit	0
GOST-cit-all	1	GOST-cit-Y-all	1
GOST-cit-all	1	GOST-cit-Y-all	1
Not_GOST-cit	0	Not_GOST-cit	0
Not_GOST-cit	0	Not_GOST-cit	0
Not_GOST-cit	0	GOST-cit-Y-all	1
Not_GOST-cit	0	Not_GOST-cit	0
Not_GOST-cit	0	Not_GOST-cit	0
Not_GOST-cit	0	Not_GOST-cit	0
Not_GOST-cit	0	Not_GOST-cit	0
GOST-cit-all	1	GOST-cit-Y-all	1
GOST-cit-all	1	GOST-cit-Y-all	1
GOST-cit-all	1	GOST-cit-Y-all	1
GOST-cit-all	1	GOST-cit-Y-all	1
GOST-cit-all	1	GOST-cit-Y-all	1
GOST-cit-all	1	GOST-cit-Y-all	1
Not_GOST-cit	0	Not_GOST-cit	0
Not_GOST-cit	0	Not_GOST-cit	0
Not_GOST-cit	0	Not_GOST-cit	0
Not_GOST-cit	0	Not_GOST-cit	0
Not_GOST-cit	0	GOST-cit-Y-all	1
Not_GOST-cit	0	GOST-cit-Y-all	1
Not_GOST-cit	0	GOST-cit-Y-all	1
Not_GOST-cit	0	Not_GOST-cit	0
Not_GOST-cit	0	Not_GOST-cit	0
Not_GOST-cit	0	Not_GOST-cit	0

gostartyall_2	gostartyall_b_	nop_gostcit_2	gostcitall_2	gostcitall_b_2	gostcityall_2	gostcityall_b_	nop_egostart
Not_GOST-art	0				Not_GOST-cit	0	
Not_GOST-art	0				Not_GOST-cit	0	
Not_GOST-art	0				Not_GOST-cit	0	
Not_GOST-art	0				Not_GOST-cit	0	
Not_GOST-art	0				Not_GOST-cit	0	
Not_GOST-art	0				Not_GOST-cit	0	
Not_GOST-art	0				Not_GOST-cit	0	
Not_GOST-art	0				Not_GOST-cit	0	
Not_GOST-art	0				Not_GOST-cit	0	
Not_GOST-art	0				Not_GOST-cit	0	
Not_GOST-art	0				Not_GOST-cit	0	
Not_GOST-art	0				Not_GOST-cit	0	
Not_GOST-art	0				Not_GOST-cit	0	
Not_GOST-art	0				Not_GOST-cit	0	
Not_GOST-art	0				Not_GOST-cit	0	
Not_GOST-art	0				Not_GOST-cit	0	
Not_GOST-art	0				Not_GOST-cit	0	
Not_GOST-art	0				Not_GOST-cit	0	
Not_GOST-art	0				Not_GOST-cit	0	
Not_GOST-art	0				Not_GOST-cit	0	
Not_GOST-art	0				Not_GOST-cit	0	
Not_GOST-art	0				Not_GOST-cit	0	
Not_GOST-art	0				Not_GOST-cit	0	
Not_GOST-art	0				Not_GOST-cit	0	
Not_GOST-art	0				Not_GOST-cit	0	
Not_GOST-art	0				Not_GOST-cit	0	
Not_GOST-art	0				Not_GOST-cit	0	
Not_GOST-art	0				Not_GOST-cit	0	
Not_GOST-art	0				Not_GOST-cit	0	
Not_GOST-art	0				Not_GOST-cit	0	
Not_GOST-art	0				Not_GOST-cit	0	
Not_GOST-art	0				Not_GOST-cit	0	
Not_GOST-art	0				Not_GOST-cit	0	
Not_GOST-art	0				Not_GOST-cit	0	
Not_GOST-art	0				Not_GOST-cit	0	
Not_GOST-art	0				Not_GOST-cit	0	
Not_GOST-art	0				Not_GOST-cit	0	
Not_GOST-art	0				Not_GOST-cit	0	
Not_GOST-art	0				Not_GOST-cit	0	
Not_GOST-art	0				Not_GOST-cit	0	
Not_GOST-art	0				Not_GOST-cit	0	
Not_GOST-art	0				Not_GOST-cit	0	
Not_GOST-art	0				Not_GOST-cit	0	
Not_GOST-art	0				Not_GOST-cit	0	
Not_GOST-art	0				Not_GOST-cit	0	
Not_GOST-art	0				Not_GOST-cit	0	

Not_GOST-art	0	Not_GOST-cit	0
Not_GOST-art	0	Not_GOST-cit	0
Not_GOST-art	0	Not_GOST-cit	0
Not_GOST-art	0	Not_GOST-cit	0
Not_GOST-art	0	Not_GOST-cit	0
Not_GOST-art	0	Not_GOST-cit	0
Not_GOST-art	0	Not_GOST-cit	0
Not_GOST-art	0	Not_GOST-cit	0
Not_GOST-art	0	GOST-cit-Y-all	1
Not_GOST-art	0	Not_GOST-cit	0
Not_GOST-art	0	Not_GOST-cit	0
Not_GOST-art	0	Not_GOST-cit	0
Not_GOST-art	0	Not_GOST-cit	0
Not_GOST-art	0	Not_GOST-cit	0
Not_GOST-art	0	Not_GOST-cit	0
Not_GOST-art	0	Not_GOST-cit	0
Not_GOST-art	0	Not_GOST-cit	0
Not_GOST-art	0	Not_GOST-cit	0
Not_GOST-art	0	Not_GOST-cit	0
Not_GOST-art	0	Not_GOST-cit	0
Not_GOST-art	0	Not_GOST-cit	0
Not_GOST-art	0	Not_GOST-cit	0
Not_GOST-art	0	Not_GOST-cit	0
Not_GOST-art	0	Not_GOST-cit	0
Not_GOST-art	0	Not_GOST-cit	0
Not_GOST-art	0	Not_GOST-cit	0
Not_GOST-art	0	Not_GOST-cit	0
Not_GOST-art	0	Not_GOST-cit	0
Not_GOST-art	0	Not_GOST-cit	0
GOST-art-Y-al	1	Not_GOST-cit	0
GOST-art-Y-al	1	Not_GOST-cit	0
GOST-art-Y-al	1	Not_GOST-cit	0
GOST-art-Y-al	1	Not_GOST-cit	0
GOST-art-Y-al	1	Not_GOST-cit	0
Not_GOST-art	0	Not_GOST-cit	0
Not_GOST-art	0	Not_GOST-cit	0
Not_GOST-art	0	Not_GOST-cit	0
Not_GOST-art	0	Not_GOST-cit	0
Not_GOST-art	0	Not_GOST-cit	0
Not_GOST-art	0	Not_GOST-cit	0
Not_GOST-art	0	Not_GOST-cit	0
Not_GOST-art	0	Not_GOST-cit	0
Not_GOST-art	0	Not_GOST-cit	0
Not_GOST-art	0	Not_GOST-cit	0
Not_GOST-art	0	Not_GOST-cit	0
Not_GOST-art	0	Not_GOST-cit	0
Not_GOST-art	0	Not_GOST-cit	0

Not_GOST-art	0	Not_GOST-cit	0
Not_GOST-art	0	Not_GOST-cit	0
Not_GOST-art	0	Not_GOST-cit	0
Not_GOST-art	0	Not_GOST-cit	0
Not_GOST-art	0	Not_GOST-cit	0
Not_GOST-art	0	Not_GOST-cit	0
Not_GOST-art	0	Not_GOST-cit	0
Not_GOST-art	0	Not_GOST-cit	0
Not_GOST-art	0	Not_GOST-cit	0
Not_GOST-art	0	Not_GOST-cit	0
Not_GOST-art	0	Not_GOST-cit	0
Not_GOST-art	0	Not_GOST-cit	0
Not_GOST-art	0	Not_GOST-cit	0
Not_GOST-art	0	Not_GOST-cit	0
Not_GOST-art	0	Not_GOST-cit	0
Not_GOST-art	0	Not_GOST-cit	0
Not_GOST-art	0	Not_GOST-cit	0
Not_GOST-art	0	Not_GOST-cit	0
Not_GOST-art	0	Not_GOST-cit	0
Not_GOST-art	0	Not_GOST-cit	0
Not_GOST-art	0	Not_GOST-cit	0
Not_GOST-art	0	Not_GOST-cit	0
Not_GOST-art	0	Not_GOST-cit	0
Not_GOST-art	0	Not_GOST-cit	0
Not_GOST-art	0	Not_GOST-cit	0
Not_GOST-art	0	Not_GOST-cit	0
Not_GOST-art	0	Not_GOST-cit	0
Not_GOST-art	0	Not_GOST-cit	0
Not_GOST-art	0	Not_GOST-cit	0
GOST-art-Y-al	1	Not_GOST-cit	0
GOST-art-Y-al	1	Not_GOST-cit	0
Not_GOST-art	0	Not_GOST-cit	0
Not_GOST-art	0	GOST-cit-Y-all	1
Not_GOST-art	0	GOST-cit-Y-all	1
Not_GOST-art	0	Not_GOST-cit	0
Not_GOST-art	0	Not_GOST-cit	0
Not_GOST-art	0	Not_GOST-cit	0
Not_GOST-art	0	Not_GOST-cit	0
Not_GOST-art	0	Not_GOST-cit	0
Not_GOST-art	0	GOST-cit-Y-all	1
Not_GOST-art	0	Not_GOST-cit	0
Not_GOST-art	0	Not_GOST-cit	0
Not_GOST-art	0	Not_GOST-cit	0
Not_GOST-art	0	Not_GOST-cit	0
Not_GOST-art	0	Not_GOST-cit	0
Not_GOST-art	0	Not_GOST-cit	0
Not_GOST-art	0	Not_GOST-cit	0

Not_GOST-art	0	Not_GOST-cit	0
Not_GOST-art	0	Not_GOST-cit	0
Not_GOST-art	0	Not_GOST-cit	0
Not_GOST-art	0	Not_GOST-cit	0
Not_GOST-art	0	Not_GOST-cit	0
Not_GOST-art	0	Not_GOST-cit	0
Not_GOST-art	0	Not_GOST-cit	0
Not_GOST-art	0	Not_GOST-cit	0
Not_GOST-art	0	Not_GOST-cit	0
Not_GOST-art	0	Not_GOST-cit	0
Not_GOST-art	0	Not_GOST-cit	0
Not_GOST-art	0	Not_GOST-cit	0
Not_GOST-art	0	Not_GOST-cit	0
GOST-art-Y-al	1	GOST-cit-Y-all	1
Not_GOST-art	0	Not_GOST-cit	0
Not_GOST-art	0	Not_GOST-cit	0
GOST-art-Y-al	1	Not_GOST-cit	0
Not_GOST-art	0	Not_GOST-cit	0
Not_GOST-art	0	Not_GOST-cit	0
Not_GOST-art	0	Not_GOST-cit	0
Not_GOST-art	0	Not_GOST-cit	0
Not_GOST-art	0	Not_GOST-cit	0
Not_GOST-art	0	Not_GOST-cit	0
Not_GOST-art	0	Not_GOST-cit	0
Not_GOST-art	0	Not_GOST-cit	0
Not_GOST-art	0	Not_GOST-cit	0
Not_GOST-art	0	Not_GOST-cit	0
Not_GOST-art	0	Not_GOST-cit	0
Not_GOST-art	0	Not_GOST-cit	0
Not_GOST-art	0	Not_GOST-cit	0
Not_GOST-art	0	Not_GOST-cit	0
Not_GOST-art	0	Not_GOST-cit	0
Not_GOST-art	0	GOST-cit-Y-all	1
Not_GOST-art	0	Not_GOST-cit	0
Not_GOST-art	0	Not_GOST-cit	0
Not_GOST-art	0	Not_GOST-cit	0
Not_GOST-art	0	Not_GOST-cit	0
Not_GOST-art	0	Not_GOST-cit	0
Not_GOST-art	0	GOST-cit-Y-all	1
Not_GOST-art	0	Not_GOST-cit	0
Not_GOST-art	0	Not_GOST-cit	0
GOST-art-Y-al	1	Not_GOST-cit	0
GOST-art-Y-al	1	Not_GOST-cit	0
Not_GOST-art	0	Not_GOST-cit	0
Not_GOST-art	0	Not_GOST-cit	0
Not_GOST-art	0	Not_GOST-cit	0
Not_GOST-art	0	Not_GOST-cit	0

Not_GOST-art	0	Not_GOST-cit	0
Not_GOST-art	0	Not_GOST-cit	0
Not_GOST-art	0	Not_GOST-cit	0
Not_GOST-art	0	Not_GOST-cit	0
Not_GOST-art	0	GOST-cit-Y-all	1
Not_GOST-art	0	GOST-cit-Y-all	1
Not_GOST-art	0	Not_GOST-cit	0
Not_GOST-art	0	Not_GOST-cit	0
Not_GOST-art	0	Not_GOST-cit	0
Not_GOST-art	0	Not_GOST-cit	0
Not_GOST-art	0	Not_GOST-cit	0
Not_GOST-art	0	Not_GOST-cit	0
Not_GOST-art	0	Not_GOST-cit	0
Not_GOST-art	0	Not_GOST-cit	0
Not_GOST-art	0	Not_GOST-cit	0
Not_GOST-art	0	Not_GOST-cit	0
Not_GOST-art	0	Not_GOST-cit	0
Not_GOST-art	0	Not_GOST-cit	0
Not_GOST-art	0	Not_GOST-cit	0
Not_GOST-art	0	Not_GOST-cit	0
Not_GOST-art	0	Not_GOST-cit	0
Not_GOST-art	0	Not_GOST-cit	0
Not_GOST-art	0	Not_GOST-cit	0
Not_GOST-art	0	Not_GOST-cit	0
Not_GOST-art	0	Not_GOST-cit	0
Not_GOST-art	0	Not_GOST-cit	0
Not_GOST-art	0	Not_GOST-cit	0
Not_GOST-art	0	Not_GOST-cit	0
Not_GOST-art	0	Not_GOST-cit	0
Not_GOST-art	0	Not_GOST-cit	0
Not_GOST-art	0	Not_GOST-cit	0
Not_GOST-art	0	Not_GOST-cit	0
Not_GOST-art	0	Not_GOST-cit	0
Not_GOST-art	0	Not_GOST-cit	0
Not_GOST-art	0	Not_GOST-cit	0
Not_GOST-art	0	Not_GOST-cit	0
Not_GOST-art	0	Not_GOST-cit	0
Not_GOST-art	0	Not_GOST-cit	0
GOST-art-Y-al	1	Not_GOST-cit	0
GOST-art-Y-al	1	Not_GOST-cit	0
GOST-art-Y-al	1	Not_GOST-cit	0
GOST-art-Y-al	1	Not_GOST-cit	0
Not_GOST-art	0	Not_GOST-cit	0
Not_GOST-art	0	Not_GOST-cit	0
Not_GOST-art	0	Not_GOST-cit	0
Not_GOST-art	0	Not_GOST-cit	0
Not_GOST-art	0	Not_GOST-cit	0

Not_GOST-art	0	Not_GOST-cit	0
Not_GOST-art	0	Not_GOST-cit	0
Not_GOST-art	0	Not_GOST-cit	0
Not_GOST-art	0	Not_GOST-cit	0
Not_GOST-art	0	Not_GOST-cit	0
Not_GOST-art	0	Not_GOST-cit	0
Not_GOST-art	0	Not_GOST-cit	0
Not_GOST-art	0	Not_GOST-cit	0
Not_GOST-art	0	Not_GOST-cit	0
Not_GOST-art	0	Not_GOST-cit	0
Not_GOST-art	0	Not_GOST-cit	0
Not_GOST-art	0	Not_GOST-cit	0
Not_GOST-art	0	Not_GOST-cit	0
Not_GOST-art	0	Not_GOST-cit	0
Not_GOST-art	0	Not_GOST-cit	0
Not_GOST-art	0	Not_GOST-cit	0
Not_GOST-art	0	Not_GOST-cit	0
Not_GOST-art	0	Not_GOST-cit	0
Not_GOST-art	0	Not_GOST-cit	0
Not_GOST-art	0	Not_GOST-cit	0
Not_GOST-art	0	Not_GOST-cit	0
GOST-art-Y-al	1	GOST-cit-Y-all	1
GOST-art-Y-al	1	GOST-cit-Y-all	1
GOST-art-Y-al	1	GOST-cit-Y-all	1
Not_GOST-art	0	Not_GOST-cit	0
Not_GOST-art	0	Not_GOST-cit	0
Not_GOST-art	0	Not_GOST-cit	0
Not_GOST-art	0	Not_GOST-cit	0
Not_GOST-art	0	Not_GOST-cit	0
Not_GOST-art	0	Not_GOST-cit	0
GOST-art-Y-al	1	GOST-cit-Y-all	1
GOST-art-Y-al	1	GOST-cit-Y-all	1
GOST-art-Y-al	1	GOST-cit-Y-all	1
Not_GOST-art	0	Not_GOST-cit	0
Not_GOST-art	0	Not_GOST-cit	0
Not_GOST-art	0	Not_GOST-cit	0
Not_GOST-art	0	Not_GOST-cit	0
Not_GOST-art	0	Not_GOST-cit	0
Not_GOST-art	0	Not_GOST-cit	0
Not_GOST-art	0	Not_GOST-cit	0
Not_GOST-art	0	Not_GOST-cit	0
Not_GOST-art	0	Not_GOST-cit	0
Not_GOST-art	0	Not_GOST-cit	0
Not_GOST-art	0	Not_GOST-cit	0
Not_GOST-art	0	Not_GOST-cit	0
Not_GOST-art	0	Not_GOST-cit	0
Not_GOST-art	0	Not_GOST-cit	0

Not_GOST-art	0	Not_GOST-cit	0
Not_GOST-art	0	Not_GOST-cit	0
Not_GOST-art	0	Not_GOST-cit	0
Not_GOST-art	0	Not_GOST-cit	0
Not_GOST-art	0	Not_GOST-cit	0
Not_GOST-art	0	Not_GOST-cit	0
Not_GOST-art	0	Not_GOST-cit	0
Not_GOST-art	0	Not_GOST-cit	0
Not_GOST-art	0	Not_GOST-cit	0
Not_GOST-art	0	Not_GOST-cit	0
Not_GOST-art	0	Not_GOST-cit	0
Not_GOST-art	0	Not_GOST-cit	0
Not_GOST-art	0	Not_GOST-cit	0
Not_GOST-art	0	Not_GOST-cit	0
Not_GOST-art	0	Not_GOST-cit	0
Not_GOST-art	0	Not_GOST-cit	0
Not_GOST-art	0	Not_GOST-cit	0
Not_GOST-art	0	Not_GOST-cit	0
Not_GOST-art	0	Not_GOST-cit	0
Not_GOST-art	0	Not_GOST-cit	0
Not_GOST-art	0	Not_GOST-cit	0
Not_GOST-art	0	Not_GOST-cit	0
Not_GOST-art	0	Not_GOST-cit	0
Not_GOST-art	0	Not_GOST-cit	0
Not_GOST-art	0	Not_GOST-cit	0
Not_GOST-art	0	Not_GOST-cit	0
Not_GOST-art	0	Not_GOST-cit	0
Not_GOST-art	0	Not_GOST-cit	0
Not_GOST-art	0	Not_GOST-cit	0
Not_GOST-art	0	Not_GOST-cit	0
Not_GOST-art	0	Not_GOST-cit	0
Not_GOST-art	0	Not_GOST-cit	0
Not_GOST-art	0	Not_GOST-cit	0
Not_GOST-art	0	Not_GOST-cit	0
Not_GOST-art	0	Not_GOST-cit	0
Not_GOST-art	0	Not_GOST-cit	0
Not_GOST-art	0	Not_GOST-cit	0
Not_GOST-art	0	Not_GOST-cit	0
Not_GOST-art	0	Not_GOST-cit	0
Not_GOST-art	0	Not_GOST-cit	0
Not_GOST-art	0	Not_GOST-cit	0
Not_GOST-art	0	Not_GOST-cit	0
Not_GOST-art	0	Not_GOST-cit	0
Not_GOST-art	0	Not_GOST-cit	0
Not_GOST-art	0	Not_GOST-cit	0
Not_GOST-art	0	Not_GOST-cit	0
Not_GOST-art	0	Not_GOST-cit	0

Not_GOST-art	0	Not_GOST-cit	0
Not_GOST-art	0	Not_GOST-cit	0
Not_GOST-art	0	Not_GOST-cit	0
Not_GOST-art	0	Not_GOST-cit	0
Not_GOST-art	0	Not_GOST-cit	0
Not_GOST-art	0	Not_GOST-cit	0
Not_GOST-art	0	Not_GOST-cit	0
Not_GOST-art	0	Not_GOST-cit	0
Not_GOST-art	0	Not_GOST-cit	0
Not_GOST-art	0	Not_GOST-cit	0
Not_GOST-art	0	Not_GOST-cit	0
Not_GOST-art	0	Not_GOST-cit	0
Not_GOST-art	0	Not_GOST-cit	0
Not_GOST-art	0	Not_GOST-cit	0
GOST-art-Y-al	1	Not_GOST-cit	0
GOST-art-Y-al	1	Not_GOST-cit	0
Not_GOST-art	0	Not_GOST-cit	0
Not_GOST-art	0	Not_GOST-cit	0
Not_GOST-art	0	Not_GOST-cit	0
Not_GOST-art	0	Not_GOST-cit	0
Not_GOST-art	0	Not_GOST-cit	0
Not_GOST-art	0	Not_GOST-cit	0
Not_GOST-art	0	Not_GOST-cit	0
Not_GOST-art	0	Not_GOST-cit	0
Not_GOST-art	0	GOST-cit-Y-all	1
Not_GOST-art	0	GOST-cit-Y-all	1
Not_GOST-art	0	Not_GOST-cit	0
Not_GOST-art	0	Not_GOST-cit	0
Not_GOST-art	0	Not_GOST-cit	0
Not_GOST-art	0	Not_GOST-cit	0
Not_GOST-art	0	Not_GOST-cit	0
Not_GOST-art	0	Not_GOST-cit	0
Not_GOST-art	0	Not_GOST-cit	0
Not_GOST-art	0	Not_GOST-cit	0
Not_GOST-art	0	Not_GOST-cit	0
Not_GOST-art	0	Not_GOST-cit	0
Not_GOST-art	0	Not_GOST-cit	0
Not_GOST-art	0	Not_GOST-cit	0
Not_GOST-art	0	Not_GOST-cit	0
Not_GOST-art	0	Not_GOST-cit	0
Not_GOST-art	0	Not_GOST-cit	0
Not_GOST-art	0	Not_GOST-cit	0
Not_GOST-art	0	Not_GOST-cit	0
Not_GOST-art	0	Not_GOST-cit	0
Not_GOST-art	0	Not_GOST-cit	0
Not_GOST-art	0	Not_GOST-cit	0
Not_GOST-art	0	Not_GOST-cit	0

Not_GOST-art	0	Not_GOST-cit	0
Not_GOST-art	0	Not_GOST-cit	0
Not_GOST-art	0	Not_GOST-cit	0
Not_GOST-art	0	Not_GOST-cit	0
Not_GOST-art	0	Not_GOST-cit	0
Not_GOST-art	0	Not_GOST-cit	0
Not_GOST-art	0	Not_GOST-cit	0
GOST-art-Y-al	1	GOST-cit-Y-all	1
GOST-art-Y-al	1	GOST-cit-Y-all	1
Not_GOST-art	0	Not_GOST-cit	0
Not_GOST-art	0	Not_GOST-cit	0
Not_GOST-art	0	Not_GOST-cit	0
Not_GOST-art	0	Not_GOST-cit	0
Not_GOST-art	0	Not_GOST-cit	0
Not_GOST-art	0	Not_GOST-cit	0
Not_GOST-art	0	Not_GOST-cit	0
Not_GOST-art	0	Not_GOST-cit	0
Not_GOST-art	0	Not_GOST-cit	0
Not_GOST-art	0	Not_GOST-cit	0
Not_GOST-art	0	Not_GOST-cit	0
Not_GOST-art	0	Not_GOST-cit	0
Not_GOST-art	0	Not_GOST-cit	0
Not_GOST-art	0	Not_GOST-cit	0
Not_GOST-art	0	Not_GOST-cit	0
Not_GOST-art	0	Not_GOST-cit	0
Not_GOST-art	0	Not_GOST-cit	0
Not_GOST-art	0	Not_GOST-cit	0
Not_GOST-art	0	Not_GOST-cit	0
Not_GOST-art	0	Not_GOST-cit	0
Not_GOST-art	0	Not_GOST-cit	0
Not_GOST-art	0	Not_GOST-cit	0
Not_GOST-art	0	Not_GOST-cit	0
Not_GOST-art	0	Not_GOST-cit	0
Not_GOST-art	0	Not_GOST-cit	0
Not_GOST-art	0	Not_GOST-cit	0
Not_GOST-art	0	Not_GOST-cit	0
Not_GOST-art	0	Not_GOST-cit	0
GOST-art-Y-al	1	Not_GOST-cit	0
Not_GOST-art	0	Not_GOST-cit	0
Not_GOST-art	0	Not_GOST-cit	0
Not_GOST-art	0	Not_GOST-cit	0
Not_GOST-art	0	Not_GOST-cit	0
Not_GOST-art	0	Not_GOST-cit	0
Not_GOST-art	0	Not_GOST-cit	0
Not_GOST-art	0	Not_GOST-cit	0
Not_GOST-art	0	Not_GOST-cit	0
Not_GOST-art	0	Not_GOST-cit	0

Not_GOST-art	0	Not_GOST-cit	0
Not_GOST-art	0	Not_GOST-cit	0
Not_GOST-art	0	Not_GOST-cit	0
Not_GOST-art	0	Not_GOST-cit	0
Not_GOST-art	0	Not_GOST-cit	0
Not_GOST-art	0	Not_GOST-cit	0
Not_GOST-art	0	Not_GOST-cit	0
Not_GOST-art	0	Not_GOST-cit	0
Not_GOST-art	0	Not_GOST-cit	0
Not_GOST-art	0	Not_GOST-cit	0
Not_GOST-art	0	Not_GOST-cit	0
GOST-art-Y-al	1	Not_GOST-cit	0
GOST-art-Y-al	1	Not_GOST-cit	0
Not_GOST-art	0	Not_GOST-cit	0
Not_GOST-art	0	Not_GOST-cit	0
Not_GOST-art	0	Not_GOST-cit	0
Not_GOST-art	0	Not_GOST-cit	0
Not_GOST-art	0	Not_GOST-cit	0
Not_GOST-art	0	Not_GOST-cit	0
Not_GOST-art	0	GOST-cit-Y-all	1
Not_GOST-art	0	GOST-cit-Y-all	1
Not_GOST-art	0	Not_GOST-cit	0
Not_GOST-art	0	Not_GOST-cit	0
Not_GOST-art	0	Not_GOST-cit	0
Not_GOST-art	0	Not_GOST-cit	0
Not_GOST-art	0	Not_GOST-cit	0
Not_GOST-art	0	Not_GOST-cit	0
Not_GOST-art	0	Not_GOST-cit	0
Not_GOST-art	0	Not_GOST-cit	0
Not_GOST-art	0	Not_GOST-cit	0
Not_GOST-art	0	Not_GOST-cit	0
Not_GOST-art	0	Not_GOST-cit	0
Not_GOST-art	0	Not_GOST-cit	0
Not_GOST-art	0	Not_GOST-cit	0
Not_GOST-art	0	Not_GOST-cit	0
Not_GOST-art	0	Not_GOST-cit	0
Not_GOST-art	0	Not_GOST-cit	0
Not_GOST-art	0	Not_GOST-cit	0
Not_GOST-art	0	Not_GOST-cit	0
Not_GOST-art	0	Not_GOST-cit	0
Not_GOST-art	0	Not_GOST-cit	0
Not_GOST-art	0	Not_GOST-cit	0
Not_GOST-art	0	Not_GOST-cit	0
Not_GOST-art	0	Not_GOST-cit	0
Not_GOST-art	0	Not_GOST-cit	0
Not_GOST-art	0	Not_GOST-cit	0
Not_GOST-art	0	Not_GOST-cit	0

Not_GOST-art	0	Not_GOST-cit	0
Not_GOST-art	0	Not_GOST-cit	0
Not_GOST-art	0	Not_GOST-cit	0
Not_GOST-art	0	Not_GOST-cit	0
Not_GOST-art	0	Not_GOST-cit	0
Not_GOST-art	0	Not_GOST-cit	0
Not_GOST-art	0	Not_GOST-cit	0
Not_GOST-art	0	Not_GOST-cit	0
Not_GOST-art	0	Not_GOST-cit	0
Not_GOST-art	0	Not_GOST-cit	0
Not_GOST-art	0	Not_GOST-cit	0
Not_GOST-art	0	Not_GOST-cit	0
Not_GOST-art	0	Not_GOST-cit	0
Not_GOST-art	0	Not_GOST-cit	0
Not_GOST-art	0	Not_GOST-cit	0
Not_GOST-art	0	Not_GOST-cit	0
Not_GOST-art	0	Not_GOST-cit	0
Not_GOST-art	0	Not_GOST-cit	0
Not_GOST-art	0	Not_GOST-cit	0
Not_GOST-art	0	Not_GOST-cit	0
Not_GOST-art	0	Not_GOST-cit	0
Not_GOST-art	0	Not_GOST-cit	0
Not_GOST-art	0	Not_GOST-cit	0
GOST-art-Y-al	1	GOST-cit-Y-all	1
GOST-art-Y-al	1	GOST-cit-Y-all	1
GOST-art-Y-al	1	GOST-cit-Y-all	1
Not_GOST-art	0	Not_GOST-cit	0
Not_GOST-art	0	Not_GOST-cit	0
Not_GOST-art	0	Not_GOST-cit	0
Not_GOST-art	0	Not_GOST-cit	0
Not_GOST-art	0	Not_GOST-cit	0
Not_GOST-art	0	Not_GOST-cit	0
Not_GOST-art	0	Not_GOST-cit	0
Not_GOST-art	0	Not_GOST-cit	0
Not_GOST-art	0	Not_GOST-cit	0
Not_GOST-art	0	Not_GOST-cit	0
Not_GOST-art	0	Not_GOST-cit	0
Not_GOST-art	0	Not_GOST-cit	0
Not_GOST-art	0	Not_GOST-cit	0
Not_GOST-art	0	Not_GOST-cit	0
Not_GOST-art	0	Not_GOST-cit	0
Not_GOST-art	0	Not_GOST-cit	0
Not_GOST-art	0	Not_GOST-cit	0
Not_GOST-art	0	Not_GOST-cit	0
Not_GOST-art	0	Not_GOST-cit	0
Not_GOST-art	0	Not_GOST-cit	0
Not_GOST-art	0	Not_GOST-cit	0

Not_GOST-art	0	Not_GOST-cit	0
Not_GOST-art	0	Not_GOST-cit	0
Not_GOST-art	0	Not_GOST-cit	0
Not_GOST-art	0	Not_GOST-cit	0
Not_GOST-art	0	Not_GOST-cit	0
Not_GOST-art	0	Not_GOST-cit	0
Not_GOST-art	0	Not_GOST-cit	0
Not_GOST-art	0	Not_GOST-cit	0
Not_GOST-art	0	Not_GOST-cit	0
Not_GOST-art	0	Not_GOST-cit	0
GOST-art-Y-al	1	GOST-cit-Y-all	1
GOST-art-Y-al	1	GOST-cit-Y-all	1
GOST-art-Y-al	1	GOST-cit-Y-all	1
Not_GOST-art	0	Not_GOST-cit	0
Not_GOST-art	0	Not_GOST-cit	0
Not_GOST-art	0	Not_GOST-cit	0
Not_GOST-art	0	Not_GOST-cit	0
Not_GOST-art	0	Not_GOST-cit	0
Not_GOST-art	0	Not_GOST-cit	0
Not_GOST-art	0	Not_GOST-cit	0
Not_GOST-art	0	Not_GOST-cit	0
Not_GOST-art	0	Not_GOST-cit	0
Not_GOST-art	0	Not_GOST-cit	0
Not_GOST-art	0	Not_GOST-cit	0
Not_GOST-art	0	Not_GOST-cit	0
Not_GOST-art	0	Not_GOST-cit	0
Not_GOST-art	0	Not_GOST-cit	0
Not_GOST-art	0	Not_GOST-cit	0
Not_GOST-art	0	Not_GOST-cit	0
Not_GOST-art	0	Not_GOST-cit	0
Not_GOST-art	0	Not_GOST-cit	0
Not_GOST-art	0	Not_GOST-cit	0
Not_GOST-art	0	Not_GOST-cit	0
Not_GOST-art	0	Not_GOST-cit	0
Not_GOST-art	0	Not_GOST-cit	0
Not_GOST-art	0	Not_GOST-cit	0
Not_GOST-art	0	Not_GOST-cit	0
Not_GOST-art	0	Not_GOST-cit	0
Not_GOST-art	0	Not_GOST-cit	0
Not_GOST-art	0	Not_GOST-cit	0
Not_GOST-art	0	Not_GOST-cit	0
Not_GOST-art	0	Not_GOST-cit	0
Not_GOST-art	0	Not_GOST-cit	0
Not_GOST-art	0	Not_GOST-cit	0
Not_GOST-art	0	Not_GOST-cit	0
Not_GOST-art	0	Not_GOST-cit	0
Not_GOST-art	0	Not_GOST-cit	0

Not_GOST-art	0	Not_GOST-cit	0
Not_GOST-art	0	Not_GOST-cit	0
Not_GOST-art	0	Not_GOST-cit	0
Not_GOST-art	0	Not_GOST-cit	0
Not_GOST-art	0	Not_GOST-cit	0
Not_GOST-art	0	Not_GOST-cit	0
Not_GOST-art	0	Not_GOST-cit	0
Not_GOST-art	0	Not_GOST-cit	0
Not_GOST-art	0	Not_GOST-cit	0
Not_GOST-art	0	Not_GOST-cit	0
Not_GOST-art	0	Not_GOST-cit	0
Not_GOST-art	0	Not_GOST-cit	0
Not_GOST-art	0	Not_GOST-cit	0
Not_GOST-art	0	Not_GOST-cit	0
Not_GOST-art	0	Not_GOST-cit	0
Not_GOST-art	0	Not_GOST-cit	0
Not_GOST-art	0	Not_GOST-cit	0
Not_GOST-art	0	Not_GOST-cit	0
Not_GOST-art	0	Not_GOST-cit	0
Not_GOST-art	0	Not_GOST-cit	0
Not_GOST-art	0	Not_GOST-cit	0
Not_GOST-art	0	Not_GOST-cit	0
Not_GOST-art	0	Not_GOST-cit	0
Not_GOST-art	0	Not_GOST-cit	0
Not_GOST-art	0	Not_GOST-cit	0
Not_GOST-art	0	Not_GOST-cit	0
Not_GOST-art	0	Not_GOST-cit	0
Not_GOST-art	0	Not_GOST-cit	0
Not_GOST-art	0	Not_GOST-cit	0
GOST-art-Y-al	1	Not_GOST-cit	0
Not_GOST-art	0	Not_GOST-cit	0
Not_GOST-art	0	Not_GOST-cit	0
Not_GOST-art	0	Not_GOST-cit	0
Not_GOST-art	0	Not_GOST-cit	0
Not_GOST-art	0	Not_GOST-cit	0
Not_GOST-art	0	Not_GOST-cit	0
Not_GOST-art	0	Not_GOST-cit	0
Not_GOST-art	0	Not_GOST-cit	0
Not_GOST-art	0	Not_GOST-cit	0
Not_GOST-art	0	Not_GOST-cit	0
Not_GOST-art	0	Not_GOST-cit	0
Not_GOST-art	0	Not_GOST-cit	0
Not_GOST-art	0	Not_GOST-cit	0
Not_GOST-art	0	Not_GOST-cit	0
Not_GOST-art	0	Not_GOST-cit	0
Not_GOST-art	0	Not_GOST-cit	0
Not_GOST-art	0	Not_GOST-cit	0

Not_GOST-art	0	Not_GOST-cit	0
Not_GOST-art	0	Not_GOST-cit	0
Not_GOST-art	0	Not_GOST-cit	0
Not_GOST-art	0	Not_GOST-cit	0
Not_GOST-art	0	Not_GOST-cit	0
Not_GOST-art	0	Not_GOST-cit	0
GOST-art-Y-al	1	Not_GOST-cit	0
GOST-art-Y-al	1	Not_GOST-cit	0
GOST-art-Y-al	1	Not_GOST-cit	0
GOST-art-Y-al	1	Not_GOST-cit	0
Not_GOST-art	0	Not_GOST-cit	0
Not_GOST-art	0	Not_GOST-cit	0
Not_GOST-art	0	Not_GOST-cit	0
Not_GOST-art	0	Not_GOST-cit	0
Not_GOST-art	0	Not_GOST-cit	0
Not_GOST-art	0	Not_GOST-cit	0
Not_GOST-art	0	Not_GOST-cit	0
Not_GOST-art	0	Not_GOST-cit	0
Not_GOST-art	0	Not_GOST-cit	0
Not_GOST-art	0	Not_GOST-cit	0
Not_GOST-art	0	Not_GOST-cit	0
Not_GOST-art	0	Not_GOST-cit	0
Not_GOST-art	0	Not_GOST-cit	0
Not_GOST-art	0	Not_GOST-cit	0
Not_GOST-art	0	Not_GOST-cit	0
Not_GOST-art	0	Not_GOST-cit	0
Not_GOST-art	0	Not_GOST-cit	0
Not_GOST-art	0	Not_GOST-cit	0
Not_GOST-art	0	Not_GOST-cit	0
Not_GOST-art	0	Not_GOST-cit	0
Not_GOST-art	0	Not_GOST-cit	0
Not_GOST-art	0	Not_GOST-cit	0
Not_GOST-art	0	Not_GOST-cit	0
Not_GOST-art	0	Not_GOST-cit	0
Not_GOST-art	0	Not_GOST-cit	0
Not_GOST-art	0	GOST-cit-Y-all	1
Not_GOST-art	0	GOST-cit-Y-all	1
Not_GOST-art	0	Not_GOST-cit	0
Not_GOST-art	0	Not_GOST-cit	0
Not_GOST-art	0	Not_GOST-cit	0
Not_GOST-art	0	Not_GOST-cit	0
Not_GOST-art	0	Not_GOST-cit	0
Not_GOST-art	0	Not_GOST-cit	0
Not_GOST-art	0	Not_GOST-cit	0
Not_GOST-art	0	Not_GOST-cit	0
Not_GOST-art	0	Not_GOST-cit	0

Not_GOST-art	0	Not_GOST-cit	0
Not_GOST-art	0	Not_GOST-cit	0
Not_GOST-art	0	Not_GOST-cit	0
Not_GOST-art	0	Not_GOST-cit	0
Not_GOST-art	0	Not_GOST-cit	0
Not_GOST-art	0	Not_GOST-cit	0
Not_GOST-art	0	Not_GOST-cit	0
Not_GOST-art	0	Not_GOST-cit	0
Not_GOST-art	0	Not_GOST-cit	0
Not_GOST-art	0	Not_GOST-cit	0
Not_GOST-art	0	Not_GOST-cit	0
Not_GOST-art	0	Not_GOST-cit	0
Not_GOST-art	0	Not_GOST-cit	0
Not_GOST-art	0	Not_GOST-cit	0
Not_GOST-art	0	Not_GOST-cit	0
Not_GOST-art	0	Not_GOST-cit	0
Not_GOST-art	0	Not_GOST-cit	0
Not_GOST-art	0	Not_GOST-cit	0
Not_GOST-art	0	Not_GOST-cit	0
Not_GOST-art	0	Not_GOST-cit	0
Not_GOST-art	0	GOST-cit-Y-all	1
Not_GOST-art	0	GOST-cit-Y-all	1
Not_GOST-art	0	Not_GOST-cit	0
Not_GOST-art	0	Not_GOST-cit	0
Not_GOST-art	0	Not_GOST-cit	0
Not_GOST-art	0	Not_GOST-cit	0
Not_GOST-art	0	Not_GOST-cit	0
Not_GOST-art	0	Not_GOST-cit	0
Not_GOST-art	0	Not_GOST-cit	0
Not_GOST-art	0	Not_GOST-cit	0
Not_GOST-art	0	Not_GOST-cit	0
Not_GOST-art	0	Not_GOST-cit	0
Not_GOST-art	0	Not_GOST-cit	0
Not_GOST-art	0	Not_GOST-cit	0
Not_GOST-art	0	Not_GOST-cit	0
Not_GOST-art	0	Not_GOST-cit	0
Not_GOST-art	0	Not_GOST-cit	0
Not_GOST-art	0	Not_GOST-cit	0
Not_GOST-art	0	Not_GOST-cit	0
Not_GOST-art	0	Not_GOST-cit	0
Not_GOST-art	0	Not_GOST-cit	0
Not_GOST-art	0	Not_GOST-cit	0
GOST-art-Y-al	1	Not_GOST-cit	0
GOST-art-Y-al	1	Not_GOST-cit	0
Not_GOST-art	0	Not_GOST-cit	0
Not_GOST-art	0	Not_GOST-cit	0
Not_GOST-art	0	Not_GOST-cit	0

Not_GOST-art	0	Not_GOST-cit	0
Not_GOST-art	0	Not_GOST-cit	0
Not_GOST-art	0	Not_GOST-cit	0
Not_GOST-art	0	Not_GOST-cit	0
Not_GOST-art	0	Not_GOST-cit	0
Not_GOST-art	0	Not_GOST-cit	0
Not_GOST-art	0	Not_GOST-cit	0
Not_GOST-art	0	Not_GOST-cit	0
Not_GOST-art	0	GOST-cit-Y-all	1
Not_GOST-art	0	Not_GOST-cit	0
Not_GOST-art	0	Not_GOST-cit	0
Not_GOST-art	0	Not_GOST-cit	0
Not_GOST-art	0	Not_GOST-cit	0
Not_GOST-art	0	Not_GOST-cit	0
Not_GOST-art	0	Not_GOST-cit	0
Not_GOST-art	0	Not_GOST-cit	0
Not_GOST-art	0	Not_GOST-cit	0
Not_GOST-art	0	Not_GOST-cit	0
Not_GOST-art	0	Not_GOST-cit	0
Not_GOST-art	0	Not_GOST-cit	0
Not_GOST-art	0	Not_GOST-cit	0
GOST-art-Y-al	1	GOST-cit-Y-all	1
GOST-art-Y-al	1	GOST-cit-Y-all	1
Not_GOST-art	0	Not_GOST-cit	0
Not_GOST-art	0	Not_GOST-cit	0
Not_GOST-art	0	Not_GOST-cit	0
Not_GOST-art	0	Not_GOST-cit	0
Not_GOST-art	0	Not_GOST-cit	0
Not_GOST-art	0	Not_GOST-cit	0
GOST-art-Y-al	1	Not_GOST-cit	0
GOST-art-Y-al	1	Not_GOST-cit	0
Not_GOST-art	0	Not_GOST-cit	0
Not_GOST-art	0	Not_GOST-cit	0
Not_GOST-art	0	Not_GOST-cit	0
Not_GOST-art	0	Not_GOST-cit	0
Not_GOST-art	0	Not_GOST-cit	0
Not_GOST-art	0	Not_GOST-cit	0
Not_GOST-art	0	Not_GOST-cit	0
Not_GOST-art	0	Not_GOST-cit	0
Not_GOST-art	0	Not_GOST-cit	0
Not_GOST-art	0	Not_GOST-cit	0
Not_GOST-art	0	Not_GOST-cit	0
Not_GOST-art	0	Not_GOST-cit	0
Not_GOST-art	0	Not_GOST-cit	0
Not_GOST-art	0	Not_GOST-cit	0
Not_GOST-art	0	Not_GOST-cit	0
Not_GOST-art	0	Not_GOST-cit	0

GOST-art-Y-al	1	Not_GOST-cit	0
Not_GOST-art	0	Not_GOST-cit	0
Not_GOST-art	0	Not_GOST-cit	0
Not_GOST-art	0	Not_GOST-cit	0
Not_GOST-art	0	Not_GOST-cit	0
Not_GOST-art	0	Not_GOST-cit	0
Not_GOST-art	0	Not_GOST-cit	0
Not_GOST-art	0	Not_GOST-cit	0
Not_GOST-art	0	Not_GOST-cit	0
Not_GOST-art	0	Not_GOST-cit	0
Not_GOST-art	0	Not_GOST-cit	0
Not_GOST-art	0	Not_GOST-cit	0
Not_GOST-art	0	Not_GOST-cit	0
Not_GOST-art	0	Not_GOST-cit	0
Not_GOST-art	0	Not_GOST-cit	0
Not_GOST-art	0	Not_GOST-cit	0
Not_GOST-art	0	Not_GOST-cit	0
Not_GOST-art	0	Not_GOST-cit	0
Not_GOST-art	0	Not_GOST-cit	0
Not_GOST-art	0	Not_GOST-cit	0
Not_GOST-art	0	Not_GOST-cit	0
Not_GOST-art	0	Not_GOST-cit	0
Not_GOST-art	0	Not_GOST-cit	0
Not_GOST-art	0	Not_GOST-cit	0
Not_GOST-art	0	Not_GOST-cit	0
Not_GOST-art	0	Not_GOST-cit	0
Not_GOST-art	0	Not_GOST-cit	0
Not_GOST-art	0	Not_GOST-cit	0
Not_GOST-art	0	Not_GOST-cit	0
Not_GOST-art	0	Not_GOST-cit	0
Not_GOST-art	0	Not_GOST-cit	0
Not_GOST-art	0	Not_GOST-cit	0
Not_GOST-art	0	Not_GOST-cit	0
Not_GOST-art	0	Not_GOST-cit	0
Not_GOST-art	0	Not_GOST-cit	0
Not_GOST-art	0	Not_GOST-cit	0
Not_GOST-art	0	Not_GOST-cit	0
Not_GOST-art	0	Not_GOST-cit	0
Not_GOST-art	0	Not_GOST-cit	0
Not_GOST-art	0	Not_GOST-cit	0
Not_GOST-art	0	Not_GOST-cit	0
Not_GOST-art	0	Not_GOST-cit	0
Not_GOST-art	0	Not_GOST-cit	0
Not_GOST-art	0	Not_GOST-cit	0
Not_GOST-art	0	Not_GOST-cit	0
Not_GOST-art	0	Not_GOST-cit	0
Not_GOST-art	0	Not_GOST-cit	0

Not_GOST-art	0	Not_GOST-cit	0
Not_GOST-art	0	Not_GOST-cit	0
Not_GOST-art	0	Not_GOST-cit	0
Not_GOST-art	0	Not_GOST-cit	0
Not_GOST-art	0	Not_GOST-cit	0
Not_GOST-art	0	Not_GOST-cit	0
Not_GOST-art	0	GOST-cit-Y-all	1
Not_GOST-art	0	GOST-cit-Y-all	1
Not_GOST-art	0	Not_GOST-cit	0
Not_GOST-art	0	Not_GOST-cit	0
Not_GOST-art	0	Not_GOST-cit	0
Not_GOST-art	0	Not_GOST-cit	0
Not_GOST-art	0	Not_GOST-cit	0
Not_GOST-art	0	Not_GOST-cit	0
Not_GOST-art	0	Not_GOST-cit	0
Not_GOST-art	0	Not_GOST-cit	0
Not_GOST-art	0	Not_GOST-cit	0
Not_GOST-art	0	Not_GOST-cit	0
Not_GOST-art	0	Not_GOST-cit	0
GOST-art-Y-al	1	Not_GOST-cit	0
GOST-art-Y-al	1	Not_GOST-cit	0
GOST-art-Y-al	1	Not_GOST-cit	0
GOST-art-Y-al	1	Not_GOST-cit	0
GOST-art-Y-al	1	Not_GOST-cit	0
Not_GOST-art	0	Not_GOST-cit	0
Not_GOST-art	0	Not_GOST-cit	0
GOST-art-Y-al	1	Not_GOST-cit	0
GOST-art-Y-al	1	Not_GOST-cit	0
GOST-art-Y-al	1	Not_GOST-cit	0
Not_GOST-art	0	Not_GOST-cit	0
Not_GOST-art	0	Not_GOST-cit	0
Not_GOST-art	0	Not_GOST-cit	0
Not_GOST-art	0	Not_GOST-cit	0
Not_GOST-art	0	Not_GOST-cit	0
Not_GOST-art	0	Not_GOST-cit	0
Not_GOST-art	0	Not_GOST-cit	0
Not_GOST-art	0	GOST-cit-Y-all	1
Not_GOST-art	0	GOST-cit-Y-all	1
Not_GOST-art	0	Not_GOST-cit	0
Not_GOST-art	0	Not_GOST-cit	0
Not_GOST-art	0	Not_GOST-cit	0
Not_GOST-art	0	Not_GOST-cit	0
Not_GOST-art	0	Not_GOST-cit	0
Not_GOST-art	0	Not_GOST-cit	0
Not_GOST-art	0	Not_GOST-cit	0
Not_GOST-art	0	Not_GOST-cit	0
Not_GOST-art	0	Not_GOST-cit	0

Not_GOST-art	0	Not_GOST-cit	0
Not_GOST-art	0	Not_GOST-cit	0
Not_GOST-art	0	Not_GOST-cit	0
Not_GOST-art	0	Not_GOST-cit	0
Not_GOST-art	0	Not_GOST-cit	0
Not_GOST-art	0	Not_GOST-cit	0
Not_GOST-art	0	Not_GOST-cit	0
Not_GOST-art	0	Not_GOST-cit	0
Not_GOST-art	0	Not_GOST-cit	0
Not_GOST-art	0	Not_GOST-cit	0
Not_GOST-art	0	Not_GOST-cit	0
Not_GOST-art	0	Not_GOST-cit	0
Not_GOST-art	0	Not_GOST-cit	0
Not_GOST-art	0	Not_GOST-cit	0
Not_GOST-art	0	Not_GOST-cit	0
Not_GOST-art	0	Not_GOST-cit	0
Not_GOST-art	0	Not_GOST-cit	0
Not_GOST-art	0	Not_GOST-cit	0
Not_GOST-art	0	Not_GOST-cit	0
Not_GOST-art	0	Not_GOST-cit	0
Not_GOST-art	0	Not_GOST-cit	0
Not_GOST-art	0	Not_GOST-cit	0
Not_GOST-art	0	Not_GOST-cit	0
Not_GOST-art	0	Not_GOST-cit	0
Not_GOST-art	0	Not_GOST-cit	0
Not_GOST-art	0	Not_GOST-cit	0
Not_GOST-art	0	Not_GOST-cit	0
Not_GOST-art	0	Not_GOST-cit	0
Not_GOST-art	0	Not_GOST-cit	0
Not_GOST-art	0	Not_GOST-cit	0
Not_GOST-art	0	Not_GOST-cit	0
Not_GOST-art	0	Not_GOST-cit	0
Not_GOST-art	0	Not_GOST-cit	0
Not_GOST-art	0	Not_GOST-cit	0
Not_GOST-art	0	Not_GOST-cit	0
Not_GOST-art	0	Not_GOST-cit	0
Not_GOST-art	0	Not_GOST-cit	0
Not_GOST-art	0	Not_GOST-cit	0
Not_GOST-art	0	Not_GOST-cit	0
Not_GOST-art	0	Not_GOST-cit	0
Not_GOST-art	0	Not_GOST-cit	0
Not_GOST-art	0	Not_GOST-cit	0
Not_GOST-art	0	Not_GOST-cit	0
GOST-art-Y-al	1	Not_GOST-cit	0
GOST-art-Y-al	1	Not_GOST-cit	0
Not_GOST-art	0	Not_GOST-cit	0
Not_GOST-art	0	Not_GOST-cit	0

Not_GOST-art	0	Not_GOST-cit	0
Not_GOST-art	0	Not_GOST-cit	0
Not_GOST-art	0	Not_GOST-cit	0
Not_GOST-art	0	Not_GOST-cit	0
Not_GOST-art	0	Not_GOST-cit	0
Not_GOST-art	0	GOST-cit-Y-all	1
Not_GOST-art	0	Not_GOST-cit	0
Not_GOST-art	0	Not_GOST-cit	0
Not_GOST-art	0	Not_GOST-cit	0
Not_GOST-art	0	Not_GOST-cit	0
Not_GOST-art	0	Not_GOST-cit	0
Not_GOST-art	0	Not_GOST-cit	0
Not_GOST-art	0	Not_GOST-cit	0
Not_GOST-art	0	Not_GOST-cit	0
Not_GOST-art	0	Not_GOST-cit	0
Not_GOST-art	0	Not_GOST-cit	0
Not_GOST-art	0	Not_GOST-cit	0
Not_GOST-art	0	Not_GOST-cit	0
Not_GOST-art	0	Not_GOST-cit	0
Not_GOST-art	0	Not_GOST-cit	0
Not_GOST-art	0	Not_GOST-cit	0
Not_GOST-art	0	Not_GOST-cit	0
Not_GOST-art	0	Not_GOST-cit	0
Not_GOST-art	0	Not_GOST-cit	0
Not_GOST-art	0	Not_GOST-cit	0
Not_GOST-art	0	Not_GOST-cit	0
Not_GOST-art	0	Not_GOST-cit	0
Not_GOST-art	0	Not_GOST-cit	0
Not_GOST-art	0	Not_GOST-cit	0
Not_GOST-art	0	Not_GOST-cit	0
Not_GOST-art	0	Not_GOST-cit	0
Not_GOST-art	0	Not_GOST-cit	0
Not_GOST-art	0	Not_GOST-cit	0
Not_GOST-art	0	Not_GOST-cit	0
Not_GOST-art	0	Not_GOST-cit	0
Not_GOST-art	0	Not_GOST-cit	0
Not_GOST-art	0	Not_GOST-cit	0
Not_GOST-art	0	Not_GOST-cit	0
GOST-art-Y-al	1	GOST-cit-Y-all	1
GOST-art-Y-al	1	GOST-cit-Y-all	1
Not_GOST-art	0	Not_GOST-cit	0
Not_GOST-art	0	Not_GOST-cit	0
Not_GOST-art	0	Not_GOST-cit	0
Not_GOST-art	0	Not_GOST-cit	0
Not_GOST-art	0	Not_GOST-cit	0
Not_GOST-art	0	Not_GOST-cit	0
Not_GOST-art	0	Not_GOST-cit	0

Not_GOST-art	0	Not_GOST-cit	0
Not_GOST-art	0	Not_GOST-cit	0
Not_GOST-art	0	Not_GOST-cit	0
Not_GOST-art	0	Not_GOST-cit	0
Not_GOST-art	0	Not_GOST-cit	0
Not_GOST-art	0	Not_GOST-cit	0
Not_GOST-art	0	Not_GOST-cit	0
Not_GOST-art	0	Not_GOST-cit	0
GOST-art-Y-al	1	Not_GOST-cit	0
GOST-art-Y-al	1	Not_GOST-cit	0
GOST-art-Y-al	1	Not_GOST-cit	0
Not_GOST-art	0	Not_GOST-cit	0
Not_GOST-art	0	Not_GOST-cit	0
Not_GOST-art	0	Not_GOST-cit	0
Not_GOST-art	0	Not_GOST-cit	0
Not_GOST-art	0	Not_GOST-cit	0
Not_GOST-art	0	Not_GOST-cit	0
Not_GOST-art	0	Not_GOST-cit	0
Not_GOST-art	0	Not_GOST-cit	0
Not_GOST-art	0	Not_GOST-cit	0
Not_GOST-art	0	Not_GOST-cit	0
Not_GOST-art	0	Not_GOST-cit	0
Not_GOST-art	0	Not_GOST-cit	0
Not_GOST-art	0	Not_GOST-cit	0
Not_GOST-art	0	Not_GOST-cit	0
Not_GOST-art	0	Not_GOST-cit	0
Not_GOST-art	0	Not_GOST-cit	0
Not_GOST-art	0	Not_GOST-cit	0
Not_GOST-art	0	Not_GOST-cit	0
Not_GOST-art	0	Not_GOST-cit	0
Not_GOST-art	0	Not_GOST-cit	0
Not_GOST-art	0	Not_GOST-cit	0
Not_GOST-art	0	Not_GOST-cit	0
Not_GOST-art	0	Not_GOST-cit	0
Not_GOST-art	0	Not_GOST-cit	0
Not_GOST-art	0	Not_GOST-cit	0
Not_GOST-art	0	Not_GOST-cit	0
Not_GOST-art	0	Not_GOST-cit	0
Not_GOST-art	0	Not_GOST-cit	0
Not_GOST-art	0	Not_GOST-cit	0
Not_GOST-art	0	Not_GOST-cit	0
Not_GOST-art	0	Not_GOST-cit	0
Not_GOST-art	0	Not_GOST-cit	0
GOST-art-Y-al	1	GOST-cit-Y-all	1
GOST-art-Y-al	1	GOST-cit-Y-all	1
GOST-art-Y-al	1	GOST-cit-Y-all	1
Not_GOST-art	0	GOST-cit-Y-all	1

Not_GOST-art	0	GOST-cit-Y-all	1
Not_GOST-art	0	Not_GOST-cit	0
Not_GOST-art	0	Not_GOST-cit	0
Not_GOST-art	0	Not_GOST-cit	0
Not_GOST-art	0	Not_GOST-cit	0
Not_GOST-art	0	Not_GOST-cit	0
Not_GOST-art	0	Not_GOST-cit	0
Not_GOST-art	0	Not_GOST-cit	0
Not_GOST-art	0	Not_GOST-cit	0
Not_GOST-art	0	Not_GOST-cit	0
Not_GOST-art	0	Not_GOST-cit	0
GOST-art-Y-al	1	Not_GOST-cit	0
GOST-art-Y-al	1	Not_GOST-cit	0
GOST-art-Y-al	1	Not_GOST-cit	0
Not_GOST-art	0	Not_GOST-cit	0
Not_GOST-art	0	Not_GOST-cit	0
Not_GOST-art	0	Not_GOST-cit	0
Not_GOST-art	0	Not_GOST-cit	0
Not_GOST-art	0	Not_GOST-cit	0
Not_GOST-art	0	Not_GOST-cit	0
Not_GOST-art	0	Not_GOST-cit	0
Not_GOST-art	0	Not_GOST-cit	0
Not_GOST-art	0	Not_GOST-cit	0
Not_GOST-art	0	Not_GOST-cit	0
Not_GOST-art	0	GOST-cit-Y-all	1
Not_GOST-art	0	GOST-cit-Y-all	1
Not_GOST-art	0	GOST-cit-Y-all	1
Not_GOST-art	0	GOST-cit-Y-all	1
Not_GOST-art	0	Not_GOST-cit	0
Not_GOST-art	0	Not_GOST-cit	0
Not_GOST-art	0	Not_GOST-cit	0
GOST-art-Y-al	1	Not_GOST-cit	0
GOST-art-Y-al	1	Not_GOST-cit	0
GOST-art-Y-al	1	Not_GOST-cit	0
Not_GOST-art	0	Not_GOST-cit	0
Not_GOST-art	0	Not_GOST-cit	0
Not_GOST-art	0	Not_GOST-cit	0
Not_GOST-art	0	Not_GOST-cit	0
Not_GOST-art	0	Not_GOST-cit	0
Not_GOST-art	0	Not_GOST-cit	0
Not_GOST-art	0	Not_GOST-cit	0
Not_GOST-art	0	Not_GOST-cit	0
Not_GOST-art	0	Not_GOST-cit	0
Not_GOST-art	0	Not_GOST-cit	0
Not_GOST-art	0	Not_GOST-cit	0
Not_GOST-art	0	Not_GOST-cit	0
Not_GOST-art	0	Not_GOST-cit	0

Not_GOST-art	0	Not_GOST-cit	0
Not_GOST-art	0	Not_GOST-cit	0
Not_GOST-art	0	Not_GOST-cit	0
Not_GOST-art	0	Not_GOST-cit	0
Not_GOST-art	0	Not_GOST-cit	0
Not_GOST-art	0	Not_GOST-cit	0
Not_GOST-art	0	Not_GOST-cit	0
Not_GOST-art	0	Not_GOST-cit	0
Not_GOST-art	0	Not_GOST-cit	0
Not_GOST-art	0	Not_GOST-cit	0
Not_GOST-art	0	Not_GOST-cit	0
Not_GOST-art	0	Not_GOST-cit	0
Not_GOST-art	0	Not_GOST-cit	0
Not_GOST-art	0	Not_GOST-cit	0
Not_GOST-art	0	Not_GOST-cit	0
Not_GOST-art	0	Not_GOST-cit	0
Not_GOST-art	0	Not_GOST-cit	0
Not_GOST-art	0	Not_GOST-cit	0
Not_GOST-art	0	Not_GOST-cit	0
Not_GOST-art	0	Not_GOST-cit	0
Not_GOST-art	0	Not_GOST-cit	0
Not_GOST-art	0	Not_GOST-cit	0
Not_GOST-art	0	Not_GOST-cit	0
Not_GOST-art	0	Not_GOST-cit	0
Not_GOST-art	0	Not_GOST-cit	0
Not_GOST-art	0	Not_GOST-cit	0
Not_GOST-art	0	Not_GOST-cit	0
Not_GOST-art	0	Not_GOST-cit	0
Not_GOST-art	0	Not_GOST-cit	0
Not_GOST-art	0	Not_GOST-cit	0
Not_GOST-art	0	Not_GOST-cit	0
Not_GOST-art	0	Not_GOST-cit	0
Not_GOST-art	0	Not_GOST-cit	0
Not_GOST-art	0	Not_GOST-cit	0
Not_GOST-art	0	Not_GOST-cit	0
Not_GOST-art	0	Not_GOST-cit	0
Not_GOST-art	0	Not_GOST-cit	0
Not_GOST-art	0	Not_GOST-cit	0
Not_GOST-art	0	Not_GOST-cit	0
Not_GOST-art	0	Not_GOST-cit	0
Not_GOST-art	0	Not_GOST-cit	0
Not_GOST-art	0	Not_GOST-cit	0
Not_GOST-art	0	Not_GOST-cit	0
Not_GOST-art	0	Not_GOST-cit	0
Not_GOST-art	0	Not_GOST-cit	0
Not_GOST-art	0	Not_GOST-cit	0
Not_GOST-art	0	Not_GOST-cit	0

Not_GOST-art	0	Not_GOST-cit	0
Not_GOST-art	0	Not_GOST-cit	0
Not_GOST-art	0	Not_GOST-cit	0
Not_GOST-art	0	Not_GOST-cit	0
Not_GOST-art	0	Not_GOST-cit	0
Not_GOST-art	0	Not_GOST-cit	0
Not_GOST-art	0	Not_GOST-cit	0
GOST-art-Y-al	1	GOST-cit-Y-all	1
GOST-art-Y-al	1	GOST-cit-Y-all	1
Not_GOST-art	0	Not_GOST-cit	0
Not_GOST-art	0	Not_GOST-cit	0
Not_GOST-art	0	Not_GOST-cit	0
Not_GOST-art	0	Not_GOST-cit	0
Not_GOST-art	0	Not_GOST-cit	0
Not_GOST-art	0	Not_GOST-cit	0
GOST-art-Y-al	1	GOST-cit-Y-all	1
Not_GOST-art	0	Not_GOST-cit	0
Not_GOST-art	0	Not_GOST-cit	0
Not_GOST-art	0	Not_GOST-cit	0
Not_GOST-art	0	Not_GOST-cit	0
Not_GOST-art	0	Not_GOST-cit	0
Not_GOST-art	0	Not_GOST-cit	0
GOST-art-Y-al	1	GOST-cit-Y-all	1
GOST-art-Y-al	1	GOST-cit-Y-all	1
GOST-art-Y-al	1	GOST-cit-Y-all	1
Not_GOST-art	0	Not_GOST-cit	0
Not_GOST-art	0	Not_GOST-cit	0
Not_GOST-art	0	Not_GOST-cit	0
Not_GOST-art	0	GOST-cit-Y-all	1
Not_GOST-art	0	GOST-cit-Y-all	1
Not_GOST-art	0	Not_GOST-cit	0
Not_GOST-art	0	Not_GOST-cit	0
Not_GOST-art	0	Not_GOST-cit	0
Not_GOST-art	0	Not_GOST-cit	0
Not_GOST-art	0	Not_GOST-cit	0
Not_GOST-art	0	Not_GOST-cit	0
Not_GOST-art	0	Not_GOST-cit	0
Not_GOST-art	0	Not_GOST-cit	0
Not_GOST-art	0	Not_GOST-cit	0
Not_GOST-art	0	Not_GOST-cit	0
Not_GOST-art	0	Not_GOST-cit	0
Not_GOST-art	0	Not_GOST-cit	0
Not_GOST-art	0	Not_GOST-cit	0
Not_GOST-art	0	Not_GOST-cit	0
Not_GOST-art	0	Not_GOST-cit	0
Not_GOST-art	0	Not_GOST-cit	0
Not_GOST-art	0	Not_GOST-cit	0

GOST-art-Y-al	1	Not_GOST-cit	0
GOST-art-Y-al	1	Not_GOST-cit	0
GOST-art-Y-al	1	Not_GOST-cit	0
Not_GOST-art	0	Not_GOST-cit	0
Not_GOST-art	0	Not_GOST-cit	0
Not_GOST-art	0	Not_GOST-cit	0
Not_GOST-art	0	Not_GOST-cit	0
Not_GOST-art	0	Not_GOST-cit	0
Not_GOST-art	0	Not_GOST-cit	0
GOST-art-Y-al	1	GOST-cit-Y-all	1
GOST-art-Y-al	1	GOST-cit-Y-all	1
GOST-art-Y-al	1	GOST-cit-Y-all	1
Not_GOST-art	0	Not_GOST-cit	0
Not_GOST-art	0	Not_GOST-cit	0
Not_GOST-art	0	Not_GOST-cit	0
Not_GOST-art	0	Not_GOST-cit	0
Not_GOST-art	0	Not_GOST-cit	0
Not_GOST-art	0	Not_GOST-cit	0
Not_GOST-art	0	Not_GOST-cit	0
Not_GOST-art	0	Not_GOST-cit	0
Not_GOST-art	0	Not_GOST-cit	0
Not_GOST-art	0	Not_GOST-cit	0
Not_GOST-art	0	Not_GOST-cit	0
Not_GOST-art	0	Not_GOST-cit	0
Not_GOST-art	0	Not_GOST-cit	0
Not_GOST-art	0	Not_GOST-cit	0
Not_GOST-art	0	Not_GOST-cit	0
Not_GOST-art	0	Not_GOST-cit	0
Not_GOST-art	0	GOST-cit-Y-all	1
Not_GOST-art	0	GOST-cit-Y-all	1
Not_GOST-art	0	Not_GOST-cit	0
Not_GOST-art	0	Not_GOST-cit	0
GOST-art-Y-al	1	Not_GOST-cit	0
GOST-art-Y-al	1	Not_GOST-cit	0
Not_GOST-art	0	Not_GOST-cit	0
Not_GOST-art	0	Not_GOST-cit	0
Not_GOST-art	0	Not_GOST-cit	0
Not_GOST-art	0	Not_GOST-cit	0
Not_GOST-art	0	Not_GOST-cit	0
Not_GOST-art	0	Not_GOST-cit	0
Not_GOST-art	0	Not_GOST-cit	0
Not_GOST-art	0	Not_GOST-cit	0
Not_GOST-art	0	Not_GOST-cit	0
Not_GOST-art	0	Not_GOST-cit	0
Not_GOST-art	0	Not_GOST-cit	0
Not_GOST-art	0	Not_GOST-cit	0
Not_GOST-art	0	Not_GOST-cit	0

Not_GOST-art	0	Not_GOST-cit	0
Not_GOST-art	0	Not_GOST-cit	0
Not_GOST-art	0	GOST-cit-Y-all	1
Not_GOST-art	0	GOST-cit-Y-all	1
Not_GOST-art	0	Not_GOST-cit	0
Not_GOST-art	0	Not_GOST-cit	0
Not_GOST-art	0	Not_GOST-cit	0
Not_GOST-art	0	Not_GOST-cit	0
Not_GOST-art	0	Not_GOST-cit	0
Not_GOST-art	0	Not_GOST-cit	0
Not_GOST-art	0	Not_GOST-cit	0
Not_GOST-art	0	Not_GOST-cit	0
Not_GOST-art	0	Not_GOST-cit	0
Not_GOST-art	0	Not_GOST-cit	0
Not_GOST-art	0	Not_GOST-cit	0
Not_GOST-art	0	Not_GOST-cit	0
Not_GOST-art	0	GOST-cit-Y-all	1
Not_GOST-art	0	GOST-cit-Y-all	1
Not_GOST-art	0	Not_GOST-cit	0
Not_GOST-art	0	Not_GOST-cit	0
Not_GOST-art	0	Not_GOST-cit	0
Not_GOST-art	0	Not_GOST-cit	0
Not_GOST-art	0	Not_GOST-cit	0
Not_GOST-art	0	Not_GOST-cit	0
Not_GOST-art	0	Not_GOST-cit	0
Not_GOST-art	0	Not_GOST-cit	0
Not_GOST-art	0	Not_GOST-cit	0
Not_GOST-art	0	Not_GOST-cit	0
Not_GOST-art	0	Not_GOST-cit	0
Not_GOST-art	0	Not_GOST-cit	0
Not_GOST-art	0	Not_GOST-cit	0
Not_GOST-art	0	Not_GOST-cit	0
Not_GOST-art	0	GOST-cit-Y-all	1
Not_GOST-art	0	Not_GOST-cit	0
Not_GOST-art	0	GOST-cit-Y-all	1
GOST-art-Y-al	1	Not_GOST-cit	0
GOST-art-Y-al	1	Not_GOST-cit	0
Not_GOST-art	0	Not_GOST-cit	0
Not_GOST-art	0	Not_GOST-cit	0
Not_GOST-art	0	Not_GOST-cit	0
GOST-art-Y-al	1	Not_GOST-cit	0
GOST-art-Y-al	1	Not_GOST-cit	0
GOST-art-Y-al	1	Not_GOST-cit	0
GOST-art-Y-al	1	Not_GOST-cit	0
Not_GOST-art	0	Not_GOST-cit	0
Not_GOST-art	0	GOST-cit-Y-all	1
Not_GOST-art	0	GOST-cit-Y-all	1

Not_GOST-art	0	Not_GOST-cit	0
Not_GOST-art	0	Not_GOST-cit	0
GOST-art-Y-al	1	GOST-cit-Y-all	1
Not_GOST-art	0	Not_GOST-cit	0
Not_GOST-art	0	Not_GOST-cit	0
Not_GOST-art	0	Not_GOST-cit	0
Not_GOST-art	0	Not_GOST-cit	0
Not_GOST-art	0	Not_GOST-cit	0
Not_GOST-art	0	Not_GOST-cit	0
Not_GOST-art	0	Not_GOST-cit	0
Not_GOST-art	0	Not_GOST-cit	0
Not_GOST-art	0	Not_GOST-cit	0
Not_GOST-art	0	Not_GOST-cit	0
Not_GOST-art	0	Not_GOST-cit	0
Not_GOST-art	0	Not_GOST-cit	0
Not_GOST-art	0	Not_GOST-cit	0
Not_GOST-art	0	Not_GOST-cit	0
Not_GOST-art	0	Not_GOST-cit	0
Not_GOST-art	0	Not_GOST-cit	0
Not_GOST-art	0	Not_GOST-cit	0
Not_GOST-art	0	Not_GOST-cit	0
Not_GOST-art	0	Not_GOST-cit	0
Not_GOST-art	0	Not_GOST-cit	0
Not_GOST-art	0	Not_GOST-cit	0
GOST-art-Y-al	1	Not_GOST-cit	0
Not_GOST-art	0	Not_GOST-cit	0
Not_GOST-art	0	Not_GOST-cit	0
Not_GOST-art	0	Not_GOST-cit	0
Not_GOST-art	0	Not_GOST-cit	0
Not_GOST-art	0	Not_GOST-cit	0
Not_GOST-art	0	Not_GOST-cit	0
Not_GOST-art	0	Not_GOST-cit	0
Not_GOST-art	0	Not_GOST-cit	0
Not_GOST-art	0	Not_GOST-cit	0
Not_GOST-art	0	Not_GOST-cit	0
Not_GOST-art	0	Not_GOST-cit	0
Not_GOST-art	0	Not_GOST-cit	0
Not_GOST-art	0	Not_GOST-cit	0
Not_GOST-art	0	Not_GOST-cit	0
GOST-art-Y-al	1	GOST-cit-Y-all	1
Not_GOST-art	0	Not_GOST-cit	0
GOST-art-Y-al	1	Not_GOST-cit	0
Not_GOST-art	0	Not_GOST-cit	0
Not_GOST-art	0	Not_GOST-cit	0
Not_GOST-art	0	Not_GOST-cit	0
Not_GOST-art	0	Not_GOST-cit	0
Not_GOST-art	0	Not_GOST-cit	0

Not_GOST-art	0	Not_GOST-cit	0
Not_GOST-art	0	Not_GOST-cit	0
Not_GOST-art	0	Not_GOST-cit	0
Not_GOST-art	0	Not_GOST-cit	0
Not_GOST-art	0	Not_GOST-cit	0
Not_GOST-art	0	Not_GOST-cit	0
Not_GOST-art	0	Not_GOST-cit	0
Not_GOST-art	0	Not_GOST-cit	0
Not_GOST-art	0	Not_GOST-cit	0
Not_GOST-art	0	Not_GOST-cit	0
Not_GOST-art	0	Not_GOST-cit	0
Not_GOST-art	0	Not_GOST-cit	0
Not_GOST-art	0	Not_GOST-cit	0
Not_GOST-art	0	Not_GOST-cit	0
Not_GOST-art	0	Not_GOST-cit	0
Not_GOST-art	0	Not_GOST-cit	0
Not_GOST-art	0	Not_GOST-cit	0
Not_GOST-art	0	Not_GOST-cit	0
Not_GOST-art	0	Not_GOST-cit	0
Not_GOST-art	0	Not_GOST-cit	0
Not_GOST-art	0	Not_GOST-cit	0
Not_GOST-art	0	Not_GOST-cit	0
Not_GOST-art	0	Not_GOST-cit	0
Not_GOST-art	0	Not_GOST-cit	0
Not_GOST-art	0	Not_GOST-cit	0
Not_GOST-art	0	Not_GOST-cit	0
Not_GOST-art	0	Not_GOST-cit	0
Not_GOST-art	0	Not_GOST-cit	0
Not_GOST-art	0	Not_GOST-cit	0
Not_GOST-art	0	Not_GOST-cit	0
Not_GOST-art	0	Not_GOST-cit	0
Not_GOST-art	0	Not_GOST-cit	0
Not_GOST-art	0	Not_GOST-cit	0
Not_GOST-art	0	Not_GOST-cit	0
Not_GOST-art	0	Not_GOST-cit	0
Not_GOST-art	0	Not_GOST-cit	0
Not_GOST-art	0	Not_GOST-cit	0
Not_GOST-art	0	Not_GOST-cit	0
Not_GOST-art	0	Not_GOST-cit	0
Not_GOST-art	0	Not_GOST-cit	0
Not_GOST-art	0	Not_GOST-cit	0
Not_GOST-art	0	Not_GOST-cit	0
Not_GOST-art	0	Not_GOST-cit	0
Not_GOST-art	0	Not_GOST-cit	0
Not_GOST-art	0	Not_GOST-cit	0
Not_GOST-art	0	Not_GOST-cit	0
Not_GOST-art	0	Not_GOST-cit	0

Not_GOST-art	0	Not_GOST-cit	0
Not_GOST-art	0	Not_GOST-cit	0
Not_GOST-art	0	Not_GOST-cit	0
Not_GOST-art	0	Not_GOST-cit	0
Not_GOST-art	0	Not_GOST-cit	0
Not_GOST-art	0	Not_GOST-cit	0
Not_GOST-art	0	Not_GOST-cit	0
Not_GOST-art	0	Not_GOST-cit	0
Not_GOST-art	0	Not_GOST-cit	0
Not_GOST-art	0	Not_GOST-cit	0
Not_GOST-art	0	Not_GOST-cit	0
Not_GOST-art	0	Not_GOST-cit	0
Not_GOST-art	0	Not_GOST-cit	0
Not_GOST-art	0	Not_GOST-cit	0
Not_GOST-art	0	Not_GOST-cit	0
Not_GOST-art	0	Not_GOST-cit	0
Not_GOST-art	0	Not_GOST-cit	0
Not_GOST-art	0	Not_GOST-cit	0
Not_GOST-art	0	Not_GOST-cit	0
Not_GOST-art	0	Not_GOST-cit	0
Not_GOST-art	0	Not_GOST-cit	0
Not_GOST-art	0	Not_GOST-cit	0
Not_GOST-art	0	Not_GOST-cit	0
Not_GOST-art	0	Not_GOST-cit	0
Not_GOST-art	0	Not_GOST-cit	0
Not_GOST-art	0	Not_GOST-cit	0
Not_GOST-art	0	Not_GOST-cit	0
Not_GOST-art	0	Not_GOST-cit	0
Not_GOST-art	0	Not_GOST-cit	0
Not_GOST-art	0	Not_GOST-cit	0
Not_GOST-art	0	Not_GOST-cit	0
Not_GOST-art	0	GOST-cit-Y-all	1
Not_GOST-art	0	GOST-cit-Y-all	1
Not_GOST-art	0	GOST-cit-Y-all	1
Not_GOST-art	0	Not_GOST-cit	0
Not_GOST-art	0	Not_GOST-cit	0
Not_GOST-art	0	Not_GOST-cit	0
Not_GOST-art	0	Not_GOST-cit	0
Not_GOST-art	0	Not_GOST-cit	0
Not_GOST-art	0	Not_GOST-cit	0
Not_GOST-art	0	Not_GOST-cit	0
Not_GOST-art	0	Not_GOST-cit	0
Not_GOST-art	0	Not_GOST-cit	0
Not_GOST-art	0	Not_GOST-cit	0
Not_GOST-art	0	Not_GOST-cit	0
Not_GOST-art	0	Not_GOST-cit	0
Not_GOST-art	0	Not_GOST-cit	0

Not_GOST-art	0	Not_GOST-cit	0
Not_GOST-art	0	Not_GOST-cit	0
Not_GOST-art	0	Not_GOST-cit	0
Not_GOST-art	0	Not_GOST-cit	0
Not_GOST-art	0	Not_GOST-cit	0
Not_GOST-art	0	Not_GOST-cit	0
Not_GOST-art	0	Not_GOST-cit	0
Not_GOST-art	0	Not_GOST-cit	0
Not_GOST-art	0	Not_GOST-cit	0
Not_GOST-art	0	Not_GOST-cit	0
Not_GOST-art	0	Not_GOST-cit	0
Not_GOST-art	0	Not_GOST-cit	0
Not_GOST-art	0	Not_GOST-cit	0
Not_GOST-art	0	Not_GOST-cit	0
Not_GOST-art	0	Not_GOST-cit	0
Not_GOST-art	0	Not_GOST-cit	0
Not_GOST-art	0	Not_GOST-cit	0
Not_GOST-art	0	Not_GOST-cit	0
Not_GOST-art	0	Not_GOST-cit	0
Not_GOST-art	0	Not_GOST-cit	0
Not_GOST-art	0	Not_GOST-cit	0
Not_GOST-art	0	GOST-cit-Y-all	1
Not_GOST-art	0	GOST-cit-Y-all	1
Not_GOST-art	0	Not_GOST-cit	0
Not_GOST-art	0	Not_GOST-cit	0
Not_GOST-art	0	Not_GOST-cit	0
Not_GOST-art	0	GOST-cit-Y-all	1
Not_GOST-art	0	GOST-cit-Y-all	1
Not_GOST-art	0	Not_GOST-cit	0
Not_GOST-art	0	Not_GOST-cit	0
Not_GOST-art	0	Not_GOST-cit	0
Not_GOST-art	0	Not_GOST-cit	0
GOST-art-Y-al	1	Not_GOST-cit	0
Not_GOST-art	0	Not_GOST-cit	0
Not_GOST-art	0	Not_GOST-cit	0
Not_GOST-art	0	Not_GOST-cit	0
Not_GOST-art	0	Not_GOST-cit	0
Not_GOST-art	0	Not_GOST-cit	0
Not_GOST-art	0	Not_GOST-cit	0
Not_GOST-art	0	Not_GOST-cit	0
Not_GOST-art	0	Not_GOST-cit	0
Not_GOST-art	0	Not_GOST-cit	0
Not_GOST-art	0	Not_GOST-cit	0
Not_GOST-art	0	Not_GOST-cit	0
Not_GOST-art	0	Not_GOST-cit	0
Not_GOST-art	0	Not_GOST-cit	0
Not_GOST-art	0	Not_GOST-cit	0

Not_GOST-art	0	Not_GOST-cit	0
Not_GOST-art	0	Not_GOST-cit	0
Not_GOST-art	0	Not_GOST-cit	0
Not_GOST-art	0	Not_GOST-cit	0
Not_GOST-art	0	Not_GOST-cit	0
Not_GOST-art	0	Not_GOST-cit	0
Not_GOST-art	0	Not_GOST-cit	0
Not_GOST-art	0	Not_GOST-cit	0
Not_GOST-art	0	Not_GOST-cit	0
Not_GOST-art	0	GOST-cit-Y-all	1
Not_GOST-art	0	Not_GOST-cit	0
Not_GOST-art	0	Not_GOST-cit	0
Not_GOST-art	0	Not_GOST-cit	0
Not_GOST-art	0	Not_GOST-cit	0
Not_GOST-art	0	Not_GOST-cit	0
Not_GOST-art	0	Not_GOST-cit	0
Not_GOST-art	0	Not_GOST-cit	0
Not_GOST-art	0	Not_GOST-cit	0
Not_GOST-art	0	Not_GOST-cit	0
Not_GOST-art	0	Not_GOST-cit	0
Not_GOST-art	0	Not_GOST-cit	0
Not_GOST-art	0	Not_GOST-cit	0
Not_GOST-art	0	Not_GOST-cit	0
Not_GOST-art	0	Not_GOST-cit	0
Not_GOST-art	0	Not_GOST-cit	0
Not_GOST-art	0	Not_GOST-cit	0
Not_GOST-art	0	Not_GOST-cit	0
Not_GOST-art	0	Not_GOST-cit	0
Not_GOST-art	0	Not_GOST-cit	0
Not_GOST-art	0	Not_GOST-cit	0
Not_GOST-art	0	Not_GOST-cit	0
Not_GOST-art	0	Not_GOST-cit	0
Not_GOST-art	0	Not_GOST-cit	0
Not_GOST-art	0	Not_GOST-cit	0
Not_GOST-art	0	Not_GOST-cit	0
Not_GOST-art	0	Not_GOST-cit	0
Not_GOST-art	0	Not_GOST-cit	0
Not_GOST-art	0	Not_GOST-cit	0
Not_GOST-art	0	Not_GOST-cit	0
GOST-art-Y-al	1	Not_GOST-cit	0
Not_GOST-art	0	Not_GOST-cit	0
Not_GOST-art	0	Not_GOST-cit	0
Not_GOST-art	0	Not_GOST-cit	0
Not_GOST-art	0	Not_GOST-cit	0
Not_GOST-art	0	Not_GOST-cit	0
Not_GOST-art	0	Not_GOST-cit	0
Not_GOST-art	0	Not_GOST-cit	0

Not_GOST-art	0	Not_GOST-cit	0
Not_GOST-art	0	Not_GOST-cit	0
Not_GOST-art	0	Not_GOST-cit	0
Not_GOST-art	0	Not_GOST-cit	0
Not_GOST-art	0	Not_GOST-cit	0
Not_GOST-art	0	Not_GOST-cit	0
Not_GOST-art	0	Not_GOST-cit	0
Not_GOST-art	0	Not_GOST-cit	0
Not_GOST-art	0	Not_GOST-cit	0
Not_GOST-art	0	Not_GOST-cit	0
Not_GOST-art	0	Not_GOST-cit	0
Not_GOST-art	0	Not_GOST-cit	0
Not_GOST-art	0	Not_GOST-cit	0
Not_GOST-art	0	Not_GOST-cit	0
Not_GOST-art	0	Not_GOST-cit	0
Not_GOST-art	0	Not_GOST-cit	0
Not_GOST-art	0	Not_GOST-cit	0
Not_GOST-art	0	Not_GOST-cit	0
Not_GOST-art	0	Not_GOST-cit	0
Not_GOST-art	0	Not_GOST-cit	0
Not_GOST-art	0	Not_GOST-cit	0
Not_GOST-art	0	Not_GOST-cit	0
Not_GOST-art	0	Not_GOST-cit	0
Not_GOST-art	0	Not_GOST-cit	0
Not_GOST-art	0	Not_GOST-cit	0
Not_GOST-art	0	Not_GOST-cit	0
Not_GOST-art	0	Not_GOST-cit	0
Not_GOST-art	0	Not_GOST-cit	0
Not_GOST-art	0	Not_GOST-cit	0
Not_GOST-art	0	Not_GOST-cit	0
Not_GOST-art	0	Not_GOST-cit	0
Not_GOST-art	0	Not_GOST-cit	0
Not_GOST-art	0	Not_GOST-cit	0
Not_GOST-art	0	Not_GOST-cit	0
Not_GOST-art	0	Not_GOST-cit	0
Not_GOST-art	0	Not_GOST-cit	0
Not_GOST-art	0	Not_GOST-cit	0
Not_GOST-art	0	Not_GOST-cit	0
Not_GOST-art	0	Not_GOST-cit	0
Not_GOST-art	0	Not_GOST-cit	0
Not_GOST-art	0	Not_GOST-cit	0
GOST-art-Y-al	1	GOST-cit-Y-all	1
GOST-art-Y-al	1	GOST-cit-Y-all	1
GOST-art-Y-al	1	GOST-cit-Y-all	1
Not_GOST-art	0	GOST-cit-Y-all	1
Not_GOST-art	0	GOST-cit-Y-all	1
Not_GOST-art	0	Not_GOST-cit	0

Not_GOST-art	0	Not_GOST-cit	0
Not_GOST-art	0	Not_GOST-cit	0
Not_GOST-art	0	Not_GOST-cit	0
Not_GOST-art	0	Not_GOST-cit	0
Not_GOST-art	0	Not_GOST-cit	0
Not_GOST-art	0	Not_GOST-cit	0
Not_GOST-art	0	Not_GOST-cit	0
Not_GOST-art	0	Not_GOST-cit	0
Not_GOST-art	0	Not_GOST-cit	0
Not_GOST-art	0	Not_GOST-cit	0
Not_GOST-art	0	Not_GOST-cit	0
Not_GOST-art	0	Not_GOST-cit	0
Not_GOST-art	0	Not_GOST-cit	0
Not_GOST-art	0	Not_GOST-cit	0
Not_GOST-art	0	Not_GOST-cit	0
Not_GOST-art	0	Not_GOST-cit	0
Not_GOST-art	0	Not_GOST-cit	0
Not_GOST-art	0	Not_GOST-cit	0
Not_GOST-art	0	Not_GOST-cit	0
Not_GOST-art	0	Not_GOST-cit	0
Not_GOST-art	0	Not_GOST-cit	0
Not_GOST-art	0	Not_GOST-cit	0
Not_GOST-art	0	Not_GOST-cit	0
Not_GOST-art	0	Not_GOST-cit	0
Not_GOST-art	0	Not_GOST-cit	0
Not_GOST-art	0	Not_GOST-cit	0
Not_GOST-art	0	Not_GOST-cit	0
Not_GOST-art	0	Not_GOST-cit	0
Not_GOST-art	0	Not_GOST-cit	0
Not_GOST-art	0	GOST-cit-Y-all	1
Not_GOST-art	0	GOST-cit-Y-all	1
Not_GOST-art	0	Not_GOST-cit	0
Not_GOST-art	0	Not_GOST-cit	0
Not_GOST-art	0	Not_GOST-cit	0
Not_GOST-art	0	Not_GOST-cit	0
Not_GOST-art	0	Not_GOST-cit	0
Not_GOST-art	0	Not_GOST-cit	0
Not_GOST-art	0	Not_GOST-cit	0
Not_GOST-art	0	Not_GOST-cit	0
Not_GOST-art	0	Not_GOST-cit	0
Not_GOST-art	0	Not_GOST-cit	0
Not_GOST-art	0	Not_GOST-cit	0
Not_GOST-art	0	Not_GOST-cit	0
Not_GOST-art	0	Not_GOST-cit	0
Not_GOST-art	0	Not_GOST-cit	0
Not_GOST-art	0	Not_GOST-cit	0
Not_GOST-art	0	Not_GOST-cit	0

Not_GOST-art	0	Not_GOST-cit	0
Not_GOST-art	0	Not_GOST-cit	0
Not_GOST-art	0	Not_GOST-cit	0
Not_GOST-art	0	Not_GOST-cit	0
Not_GOST-art	0	Not_GOST-cit	0
Not_GOST-art	0	Not_GOST-cit	0
Not_GOST-art	0	Not_GOST-cit	0
Not_GOST-art	0	Not_GOST-cit	0
Not_GOST-art	0	Not_GOST-cit	0
Not_GOST-art	0	Not_GOST-cit	0
Not_GOST-art	0	Not_GOST-cit	0
Not_GOST-art	0	Not_GOST-cit	0
Not_GOST-art	0	Not_GOST-cit	0
Not_GOST-art	0	Not_GOST-cit	0
Not_GOST-art	0	Not_GOST-cit	0
Not_GOST-art	0	Not_GOST-cit	0
Not_GOST-art	0	Not_GOST-cit	0
Not_GOST-art	0	Not_GOST-cit	0
Not_GOST-art	0	Not_GOST-cit	0
GOST-art-Y-al	1	GOST-cit-Y-all	1
Not_GOST-art	0	Not_GOST-cit	0
Not_GOST-art	0	Not_GOST-cit	0
Not_GOST-art	0	Not_GOST-cit	0
Not_GOST-art	0	Not_GOST-cit	0
Not_GOST-art	0	Not_GOST-cit	0
Not_GOST-art	0	Not_GOST-cit	0
Not_GOST-art	0	Not_GOST-cit	0
Not_GOST-art	0	Not_GOST-cit	0
Not_GOST-art	0	Not_GOST-cit	0
Not_GOST-art	0	Not_GOST-cit	0
Not_GOST-art	0	Not_GOST-cit	0
Not_GOST-art	0	Not_GOST-cit	0
Not_GOST-art	0	Not_GOST-cit	0
Not_GOST-art	0	Not_GOST-cit	0
Not_GOST-art	0	Not_GOST-cit	0
Not_GOST-art	0	Not_GOST-cit	0
Not_GOST-art	0	Not_GOST-cit	0
Not_GOST-art	0	Not_GOST-cit	0
Not_GOST-art	0	Not_GOST-cit	0
Not_GOST-art	0	Not_GOST-cit	0
Not_GOST-art	0	Not_GOST-cit	0
Not_GOST-art	0	Not_GOST-cit	0
Not_GOST-art	0	Not_GOST-cit	0
Not_GOST-art	0	Not_GOST-cit	0
Not_GOST-art	0	Not_GOST-cit	0
Not_GOST-art	0	Not_GOST-cit	0
Not_GOST-art	0	Not_GOST-cit	0

Not_GOST-art	0	Not_GOST-cit	0
Not_GOST-art	0	Not_GOST-cit	0
Not_GOST-art	0	Not_GOST-cit	0
Not_GOST-art	0	GOST-cit-Y-all	1
Not_GOST-art	0	Not_GOST-cit	0
GOST-art-Y-al	1	GOST-cit-Y-all	1
GOST-art-Y-al	1	GOST-cit-Y-all	1
Not_GOST-art	0	Not_GOST-cit	0
Not_GOST-art	0	Not_GOST-cit	0
Not_GOST-art	0	Not_GOST-cit	0
Not_GOST-art	0	Not_GOST-cit	0
Not_GOST-art	0	Not_GOST-cit	0
Not_GOST-art	0	Not_GOST-cit	0
Not_GOST-art	0	Not_GOST-cit	0
Not_GOST-art	0	Not_GOST-cit	0
Not_GOST-art	0	Not_GOST-cit	0
Not_GOST-art	0	Not_GOST-cit	0
Not_GOST-art	0	Not_GOST-cit	0
Not_GOST-art	0	Not_GOST-cit	0
Not_GOST-art	0	Not_GOST-cit	0
Not_GOST-art	0	Not_GOST-cit	0
Not_GOST-art	0	Not_GOST-cit	0
Not_GOST-art	0	Not_GOST-cit	0
Not_GOST-art	0	Not_GOST-cit	0
Not_GOST-art	0	Not_GOST-cit	0
Not_GOST-art	0	Not_GOST-cit	0
Not_GOST-art	0	Not_GOST-cit	0
Not_GOST-art	0	Not_GOST-cit	0
Not_GOST-art	0	Not_GOST-cit	0
Not_GOST-art	0	Not_GOST-cit	0
Not_GOST-art	0	Not_GOST-cit	0
Not_GOST-art	0	Not_GOST-cit	0
Not_GOST-art	0	Not_GOST-cit	0
Not_GOST-art	0	Not_GOST-cit	0
Not_GOST-art	0	Not_GOST-cit	0
Not_GOST-art	0	Not_GOST-cit	0
Not_GOST-art	0	Not_GOST-cit	0
Not_GOST-art	0	Not_GOST-cit	0
Not_GOST-art	0	Not_GOST-cit	0
Not_GOST-art	0	Not_GOST-cit	0
Not_GOST-art	0	Not_GOST-cit	0
Not_GOST-art	0	Not_GOST-cit	0
Not_GOST-art	0	Not_GOST-cit	0
Not_GOST-art	0	Not_GOST-cit	0
Not_GOST-art	0	Not_GOST-cit	0
Not_GOST-art	0	Not_GOST-cit	0
Not_GOST-art	0	Not_GOST-cit	0

Not_GOST-art	0	Not_GOST-cit	0
Not_GOST-art	0	Not_GOST-cit	0
Not_GOST-art	0	Not_GOST-cit	0
Not_GOST-art	0	Not_GOST-cit	0
Not_GOST-art	0	Not_GOST-cit	0
Not_GOST-art	0	Not_GOST-cit	0
Not_GOST-art	0	Not_GOST-cit	0
Not_GOST-art	0	Not_GOST-cit	0
Not_GOST-art	0	Not_GOST-cit	0
Not_GOST-art	0	Not_GOST-cit	0
Not_GOST-art	0	Not_GOST-cit	0
Not_GOST-art	0	Not_GOST-cit	0
Not_GOST-art	0	GOST-cit-Y-all	1
Not_GOST-art	0	GOST-cit-Y-all	1
Not_GOST-art	0	GOST-cit-Y-all	1
Not_GOST-art	0	Not_GOST-cit	0
GOST-art-Y-al	1	Not_GOST-cit	0
GOST-art-Y-al	1	Not_GOST-cit	0
GOST-art-Y-al	1	Not_GOST-cit	0
Not_GOST-art	0	Not_GOST-cit	0
Not_GOST-art	0	Not_GOST-cit	0
Not_GOST-art	0	Not_GOST-cit	0
Not_GOST-art	0	Not_GOST-cit	0
Not_GOST-art	0	Not_GOST-cit	0
Not_GOST-art	0	Not_GOST-cit	0
Not_GOST-art	0	Not_GOST-cit	0
Not_GOST-art	0	Not_GOST-cit	0
Not_GOST-art	0	Not_GOST-cit	0
Not_GOST-art	0	Not_GOST-cit	0
Not_GOST-art	0	Not_GOST-cit	0
Not_GOST-art	0	Not_GOST-cit	0
Not_GOST-art	0	Not_GOST-cit	0
Not_GOST-art	0	Not_GOST-cit	0
Not_GOST-art	0	GOST-cit-Y-all	1
Not_GOST-art	0	GOST-cit-Y-all	1
Not_GOST-art	0	GOST-cit-Y-all	1
Not_GOST-art	0	GOST-cit-Y-all	1
Not_GOST-art	0	Not_GOST-cit	0
Not_GOST-art	0	Not_GOST-cit	0
Not_GOST-art	0	Not_GOST-cit	0
Not_GOST-art	0	Not_GOST-cit	0
Not_GOST-art	0	Not_GOST-cit	0
Not_GOST-art	0	Not_GOST-cit	0
Not_GOST-art	0	Not_GOST-cit	0
Not_GOST-art	0	Not_GOST-cit	0
Not_GOST-art	0	Not_GOST-cit	0
Not_GOST-art	0	Not_GOST-cit	0

Not_GOST-art	0	Not_GOST-cit	0
Not_GOST-art	0	Not_GOST-cit	0
Not_GOST-art	0	Not_GOST-cit	0
Not_GOST-art	0	Not_GOST-cit	0
Not_GOST-art	0	Not_GOST-cit	0
Not_GOST-art	0	Not_GOST-cit	0
Not_GOST-art	0	Not_GOST-cit	0
Not_GOST-art	0	Not_GOST-cit	0
Not_GOST-art	0	Not_GOST-cit	0
Not_GOST-art	0	Not_GOST-cit	0
Not_GOST-art	0	Not_GOST-cit	0
Not_GOST-art	0	Not_GOST-cit	0
Not_GOST-art	0	Not_GOST-cit	0
Not_GOST-art	0	Not_GOST-cit	0
Not_GOST-art	0	Not_GOST-cit	0
Not_GOST-art	0	Not_GOST-cit	0
Not_GOST-art	0	Not_GOST-cit	0
Not_GOST-art	0	Not_GOST-cit	0
Not_GOST-art	0	Not_GOST-cit	0
Not_GOST-art	0	Not_GOST-cit	0
Not_GOST-art	0	Not_GOST-cit	0
Not_GOST-art	0	Not_GOST-cit	0
Not_GOST-art	0	Not_GOST-cit	0
Not_GOST-art	0	Not_GOST-cit	0
Not_GOST-art	0	Not_GOST-cit	0
Not_GOST-art	0	Not_GOST-cit	0
Not_GOST-art	0	Not_GOST-cit	0
Not_GOST-art	0	Not_GOST-cit	0
Not_GOST-art	0	Not_GOST-cit	0
Not_GOST-art	0	Not_GOST-cit	0
Not_GOST-art	0	Not_GOST-cit	0
Not_GOST-art	0	Not_GOST-cit	0
Not_GOST-art	0	Not_GOST-cit	0
Not_GOST-art	0	Not_GOST-cit	0
Not_GOST-art	0	Not_GOST-cit	0
Not_GOST-art	0	Not_GOST-cit	0
Not_GOST-art	0	Not_GOST-cit	0
Not_GOST-art	0	Not_GOST-cit	0
Not_GOST-art	0	Not_GOST-cit	0
Not_GOST-art	0	Not_GOST-cit	0
Not_GOST-art	0	Not_GOST-cit	0
Not_GOST-art	0	Not_GOST-cit	0
Not_GOST-art	0	Not_GOST-cit	0
Not_GOST-art	0	Not_GOST-cit	0
Not_GOST-art	0	Not_GOST-cit	0
GOST-art-Y-al	1	Not_GOST-cit	0
GOST-art-Y-al	1	Not_GOST-cit	0

GOST-art-Y-al	1	Not_GOST-cit	0
Not_GOST-art	0	Not_GOST-cit	0
Not_GOST-art	0	Not_GOST-cit	0
Not_GOST-art	0	Not_GOST-cit	0
Not_GOST-art	0	Not_GOST-cit	0
Not_GOST-art	0	Not_GOST-cit	0
Not_GOST-art	0	Not_GOST-cit	0
Not_GOST-art	0	Not_GOST-cit	0
Not_GOST-art	0	Not_GOST-cit	0
Not_GOST-art	0	Not_GOST-cit	0
Not_GOST-art	0	Not_GOST-cit	0
GOST-art-Y-al	1	Not_GOST-cit	0
Not_GOST-art	0	Not_GOST-cit	0
Not_GOST-art	0	Not_GOST-cit	0
Not_GOST-art	0	Not_GOST-cit	0
Not_GOST-art	0	Not_GOST-cit	0
Not_GOST-art	0	Not_GOST-cit	0
Not_GOST-art	0	Not_GOST-cit	0
Not_GOST-art	0	Not_GOST-cit	0
Not_GOST-art	0	Not_GOST-cit	0
Not_GOST-art	0	Not_GOST-cit	0
Not_GOST-art	0	Not_GOST-cit	0
Not_GOST-art	0	Not_GOST-cit	0
Not_GOST-art	0	Not_GOST-cit	0
Not_GOST-art	0	Not_GOST-cit	0
Not_GOST-art	0	Not_GOST-cit	0
Not_GOST-art	0	Not_GOST-cit	0
Not_GOST-art	0	Not_GOST-cit	0
Not_GOST-art	0	Not_GOST-cit	0
Not_GOST-art	0	Not_GOST-cit	0
Not_GOST-art	0	Not_GOST-cit	0
Not_GOST-art	0	Not_GOST-cit	0
Not_GOST-art	0	GOST-cit-Y-all	1
Not_GOST-art	0	Not_GOST-cit	0
Not_GOST-art	0	Not_GOST-cit	0
Not_GOST-art	0	Not_GOST-cit	0
Not_GOST-art	0	Not_GOST-cit	0
Not_GOST-art	0	Not_GOST-cit	0
Not_GOST-art	0	Not_GOST-cit	0
Not_GOST-art	0	Not_GOST-cit	0
Not_GOST-art	0	Not_GOST-cit	0
Not_GOST-art	0	Not_GOST-cit	0
Not_GOST-art	0	Not_GOST-cit	0
Not_GOST-art	0	Not_GOST-cit	0
Not_GOST-art	0	Not_GOST-cit	0
Not_GOST-art	0	Not_GOST-cit	0
Not_GOST-art	0	Not_GOST-cit	0

Not_GOST-art	0	GOST-cit-Y-all	1
Not_GOST-art	0	GOST-cit-Y-all	1
Not_GOST-art	0	Not_GOST-cit	0
Not_GOST-art	0	Not_GOST-cit	0
Not_GOST-art	0	Not_GOST-cit	0
Not_GOST-art	0	Not_GOST-cit	0
Not_GOST-art	0	Not_GOST-cit	0
Not_GOST-art	0	Not_GOST-cit	0
Not_GOST-art	0	Not_GOST-cit	0
Not_GOST-art	0	Not_GOST-cit	0
Not_GOST-art	0	Not_GOST-cit	0
Not_GOST-art	0	Not_GOST-cit	0
Not_GOST-art	0	Not_GOST-cit	0
Not_GOST-art	0	Not_GOST-cit	0
Not_GOST-art	0	GOST-cit-Y-all	1
Not_GOST-art	0	Not_GOST-cit	0
Not_GOST-art	0	Not_GOST-cit	0
Not_GOST-art	0	Not_GOST-cit	0
Not_GOST-art	0	Not_GOST-cit	0
Not_GOST-art	0	Not_GOST-cit	0
Not_GOST-art	0	Not_GOST-cit	0
Not_GOST-art	0	Not_GOST-cit	0
Not_GOST-art	0	Not_GOST-cit	0
Not_GOST-art	0	Not_GOST-cit	0
Not_GOST-art	0	GOST-cit-Y-all	1
Not_GOST-art	0	GOST-cit-Y-all	1
Not_GOST-art	0	Not_GOST-cit	0
Not_GOST-art	0	Not_GOST-cit	0
Not_GOST-art	0	Not_GOST-cit	0
Not_GOST-art	0	Not_GOST-cit	0
Not_GOST-art	0	Not_GOST-cit	0
Not_GOST-art	0	Not_GOST-cit	0
Not_GOST-art	0	Not_GOST-cit	0
Not_GOST-art	0	Not_GOST-cit	0
Not_GOST-art	0	Not_GOST-cit	0
Not_GOST-art	0	Not_GOST-cit	0
Not_GOST-art	0	Not_GOST-cit	0
Not_GOST-art	0	Not_GOST-cit	0
Not_GOST-art	0	Not_GOST-cit	0
Not_GOST-art	0	Not_GOST-cit	0
Not_GOST-art	0	Not_GOST-cit	0
Not_GOST-art	0	Not_GOST-cit	0
Not_GOST-art	0	Not_GOST-cit	0
Not_GOST-art	0	Not_GOST-cit	0
Not_GOST-art	0	Not_GOST-cit	0
Not_GOST-art	0	Not_GOST-cit	0
Not_GOST-art	0	Not_GOST-cit	0

Not_GOST-art	0	Not_GOST-cit	0
Not_GOST-art	0	Not_GOST-cit	0
Not_GOST-art	0	Not_GOST-cit	0
Not_GOST-art	0	Not_GOST-cit	0
Not_GOST-art	0	Not_GOST-cit	0
Not_GOST-art	0	Not_GOST-cit	0
Not_GOST-art	0	Not_GOST-cit	0
Not_GOST-art	0	Not_GOST-cit	0
Not_GOST-art	0	Not_GOST-cit	0
Not_GOST-art	0	Not_GOST-cit	0
Not_GOST-art	0	GOST-cit-Y-all	1
Not_GOST-art	0	GOST-cit-Y-all	1
Not_GOST-art	0	Not_GOST-cit	0
Not_GOST-art	0	Not_GOST-cit	0
Not_GOST-art	0	Not_GOST-cit	0
Not_GOST-art	0	Not_GOST-cit	0
Not_GOST-art	0	Not_GOST-cit	0
Not_GOST-art	0	Not_GOST-cit	0
Not_GOST-art	0	Not_GOST-cit	0
Not_GOST-art	0	Not_GOST-cit	0
Not_GOST-art	0	Not_GOST-cit	0
Not_GOST-art	0	Not_GOST-cit	0
Not_GOST-art	0	Not_GOST-cit	0
Not_GOST-art	0	Not_GOST-cit	0
GOST-art-Y-al	1	Not_GOST-cit	0
Not_GOST-art	0	Not_GOST-cit	0
Not_GOST-art	0	Not_GOST-cit	0
Not_GOST-art	0	Not_GOST-cit	0
Not_GOST-art	0	Not_GOST-cit	0
Not_GOST-art	0	Not_GOST-cit	0
Not_GOST-art	0	Not_GOST-cit	0
Not_GOST-art	0	Not_GOST-cit	0
Not_GOST-art	0	Not_GOST-cit	0
Not_GOST-art	0	Not_GOST-cit	0
Not_GOST-art	0	Not_GOST-cit	0
Not_GOST-art	0	Not_GOST-cit	0
Not_GOST-art	0	Not_GOST-cit	0
Not_GOST-art	0	Not_GOST-cit	0
Not_GOST-art	0	Not_GOST-cit	0
Not_GOST-art	0	Not_GOST-cit	0
Not_GOST-art	0	Not_GOST-cit	0
Not_GOST-art	0	Not_GOST-cit	0
GOST-art-Y-al	1	Not_GOST-cit	0
GOST-art-Y-al	1	Not_GOST-cit	0
GOST-art-Y-al	1	Not_GOST-cit	0
Not_GOST-art	0	Not_GOST-cit	0
Not_GOST-art	0	Not_GOST-cit	0

Not_GOST-art	0	Not_GOST-cit	0
Not_GOST-art	0	Not_GOST-cit	0
Not_GOST-art	0	Not_GOST-cit	0
Not_GOST-art	0	Not_GOST-cit	0
Not_GOST-art	0	Not_GOST-cit	0
Not_GOST-art	0	Not_GOST-cit	0
Not_GOST-art	0	Not_GOST-cit	0
Not_GOST-art	0	Not_GOST-cit	0
Not_GOST-art	0	Not_GOST-cit	0
Not_GOST-art	0	Not_GOST-cit	0
Not_GOST-art	0	Not_GOST-cit	0
Not_GOST-art	0	Not_GOST-cit	0
Not_GOST-art	0	Not_GOST-cit	0
Not_GOST-art	0	Not_GOST-cit	0
Not_GOST-art	0	Not_GOST-cit	0
Not_GOST-art	0	Not_GOST-cit	0
Not_GOST-art	0	Not_GOST-cit	0
Not_GOST-art	0	Not_GOST-cit	0
Not_GOST-art	0	Not_GOST-cit	0
Not_GOST-art	0	Not_GOST-cit	0
Not_GOST-art	0	Not_GOST-cit	0
Not_GOST-art	0	Not_GOST-cit	0
Not_GOST-art	0	Not_GOST-cit	0
Not_GOST-art	0	Not_GOST-cit	0
Not_GOST-art	0	Not_GOST-cit	0
Not_GOST-art	0	Not_GOST-cit	0
Not_GOST-art	0	Not_GOST-cit	0
Not_GOST-art	0	Not_GOST-cit	0
Not_GOST-art	0	Not_GOST-cit	0
Not_GOST-art	0	Not_GOST-cit	0
Not_GOST-art	0	Not_GOST-cit	0
Not_GOST-art	0	Not_GOST-cit	0
Not_GOST-art	0	Not_GOST-cit	0
Not_GOST-art	0	Not_GOST-cit	0
Not_GOST-art	0	Not_GOST-cit	0
Not_GOST-art	0	Not_GOST-cit	0
Not_GOST-art	0	Not_GOST-cit	0
Not_GOST-art	0	Not_GOST-cit	0
Not_GOST-art	0	Not_GOST-cit	0
Not_GOST-art	0	Not_GOST-cit	0
Not_GOST-art	0	Not_GOST-cit	0
Not_GOST-art	0	Not_GOST-cit	0
Not_GOST-art	0	Not_GOST-cit	0
Not_GOST-art	0	Not_GOST-cit	0
Not_GOST-art	0	Not_GOST-cit	0
Not_GOST-art	0	Not_GOST-cit	0
Not_GOST-art	0	Not_GOST-cit	0

Not_GOST-art	0	Not_GOST-cit	0
Not_GOST-art	0	Not_GOST-cit	0
GOST-art-Y-al	1	Not_GOST-cit	0
GOST-art-Y-al	1	Not_GOST-cit	0
GOST-art-Y-al	1	Not_GOST-cit	0
GOST-art-Y-al	1	Not_GOST-cit	0
GOST-art-Y-al	1	Not_GOST-cit	0
Not_GOST-art	0	Not_GOST-cit	0
Not_GOST-art	0	Not_GOST-cit	0
Not_GOST-art	0	Not_GOST-cit	0
Not_GOST-art	0	Not_GOST-cit	0
Not_GOST-art	0	Not_GOST-cit	0
Not_GOST-art	0	Not_GOST-cit	0
Not_GOST-art	0	Not_GOST-cit	0
Not_GOST-art	0	Not_GOST-cit	0
Not_GOST-art	0	Not_GOST-cit	0
Not_GOST-art	0	Not_GOST-cit	0
Not_GOST-art	0	Not_GOST-cit	0
Not_GOST-art	0	Not_GOST-cit	0
Not_GOST-art	0	Not_GOST-cit	0
Not_GOST-art	0	Not_GOST-cit	0
Not_GOST-art	0	Not_GOST-cit	0
Not_GOST-art	0	Not_GOST-cit	0
Not_GOST-art	0	Not_GOST-cit	0
Not_GOST-art	0	Not_GOST-cit	0
Not_GOST-art	0	Not_GOST-cit	0
Not_GOST-art	0	Not_GOST-cit	0
Not_GOST-art	0	Not_GOST-cit	0
Not_GOST-art	0	Not_GOST-cit	0
Not_GOST-art	0	Not_GOST-cit	0
Not_GOST-art	0	Not_GOST-cit	0
Not_GOST-art	0	GOST-cit-Y-all	1
GOST-art-Y-al	1	Not_GOST-cit	0
GOST-art-Y-al	1	Not_GOST-cit	0
GOST-art-Y-al	1	Not_GOST-cit	0
Not_GOST-art	0	Not_GOST-cit	0
Not_GOST-art	0	Not_GOST-cit	0
Not_GOST-art	0	Not_GOST-cit	0
Not_GOST-art	0	Not_GOST-cit	0
Not_GOST-art	0	Not_GOST-cit	0
Not_GOST-art	0	Not_GOST-cit	0
Not_GOST-art	0	Not_GOST-cit	0
Not_GOST-art	0	Not_GOST-cit	0
Not_GOST-art	0	Not_GOST-cit	0
Not_GOST-art	0	Not_GOST-cit	0
Not_GOST-art	0	Not_GOST-cit	0
Not_GOST-art	0	Not_GOST-cit	0

Not_GOST-art	0	Not_GOST-cit	0
Not_GOST-art	0	Not_GOST-cit	0
Not_GOST-art	0	Not_GOST-cit	0
Not_GOST-art	0	Not_GOST-cit	0
Not_GOST-art	0	Not_GOST-cit	0
GOST-art-Y-al	1	GOST-cit-Y-all	1
GOST-art-Y-al	1	GOST-cit-Y-all	1
GOST-art-Y-al	1	GOST-cit-Y-all	1
Not_GOST-art	0	Not_GOST-cit	0
Not_GOST-art	0	Not_GOST-cit	0
Not_GOST-art	0	Not_GOST-cit	0
Not_GOST-art	0	Not_GOST-cit	0
Not_GOST-art	0	Not_GOST-cit	0
Not_GOST-art	0	Not_GOST-cit	0
Not_GOST-art	0	Not_GOST-cit	0
Not_GOST-art	0	Not_GOST-cit	0
Not_GOST-art	0	Not_GOST-cit	0
Not_GOST-art	0	Not_GOST-cit	0
Not_GOST-art	0	Not_GOST-cit	0
Not_GOST-art	0	Not_GOST-cit	0
Not_GOST-art	0	Not_GOST-cit	0
Not_GOST-art	0	Not_GOST-cit	0
Not_GOST-art	0	Not_GOST-cit	0
Not_GOST-art	0	Not_GOST-cit	0
Not_GOST-art	0	Not_GOST-cit	0
GOST-art-Y-al	1	Not_GOST-cit	0
GOST-art-Y-al	1	Not_GOST-cit	0
GOST-art-Y-al	1	Not_GOST-cit	0
GOST-art-Y-al	1	Not_GOST-cit	0
GOST-art-Y-al	1	Not_GOST-cit	0
Not_GOST-art	0	Not_GOST-cit	0
Not_GOST-art	0	Not_GOST-cit	0
Not_GOST-art	0	Not_GOST-cit	0
Not_GOST-art	0	Not_GOST-cit	0
Not_GOST-art	0	Not_GOST-cit	0
Not_GOST-art	0	Not_GOST-cit	0
Not_GOST-art	0	Not_GOST-cit	0
Not_GOST-art	0	Not_GOST-cit	0
Not_GOST-art	0	Not_GOST-cit	0
Not_GOST-art	0	Not_GOST-cit	0
Not_GOST-art	0	Not_GOST-cit	0
Not_GOST-art	0	Not_GOST-cit	0
Not_GOST-art	0	Not_GOST-cit	0
Not_GOST-art	0	Not_GOST-cit	0
Not_GOST-art	0	Not_GOST-cit	0
Not_GOST-art	0	Not_GOST-cit	0
Not_GOST-art	0	Not_GOST-cit	0

Not_GOST-art	0	Not_GOST-cit	0
Not_GOST-art	0	Not_GOST-cit	0
Not_GOST-art	0	Not_GOST-cit	0
Not_GOST-art	0	Not_GOST-cit	0
Not_GOST-art	0	Not_GOST-cit	0
Not_GOST-art	0	Not_GOST-cit	0
Not_GOST-art	0	Not_GOST-cit	0
Not_GOST-art	0	Not_GOST-cit	0
Not_GOST-art	0	Not_GOST-cit	0
Not_GOST-art	0	Not_GOST-cit	0
Not_GOST-art	0	Not_GOST-cit	0
Not_GOST-art	0	Not_GOST-cit	0
Not_GOST-art	0	Not_GOST-cit	0
Not_GOST-art	0	Not_GOST-cit	0
Not_GOST-art	0	Not_GOST-cit	0
Not_GOST-art	0	Not_GOST-cit	0
Not_GOST-art	0	Not_GOST-cit	0
Not_GOST-art	0	Not_GOST-cit	0
Not_GOST-art	0	Not_GOST-cit	0
Not_GOST-art	0	Not_GOST-cit	0
Not_GOST-art	0	Not_GOST-cit	0
Not_GOST-art	0	Not_GOST-cit	0
Not_GOST-art	0	Not_GOST-cit	0
Not_GOST-art	0	Not_GOST-cit	0
Not_GOST-art	0	Not_GOST-cit	0
Not_GOST-art	0	Not_GOST-cit	0
Not_GOST-art	0	Not_GOST-cit	0
Not_GOST-art	0	Not_GOST-cit	0
GOST-art-Y-al	1	Not_GOST-cit	0
GOST-art-Y-al	1	Not_GOST-cit	0
Not_GOST-art	0	Not_GOST-cit	0
Not_GOST-art	0	Not_GOST-cit	0
Not_GOST-art	0	Not_GOST-cit	0
Not_GOST-art	0	Not_GOST-cit	0
Not_GOST-art	0	Not_GOST-cit	0
Not_GOST-art	0	Not_GOST-cit	0
Not_GOST-art	0	Not_GOST-cit	0
Not_GOST-art	0	Not_GOST-cit	0
Not_GOST-art	0	Not_GOST-cit	0
Not_GOST-art	0	Not_GOST-cit	0
Not_GOST-art	0	Not_GOST-cit	0
Not_GOST-art	0	Not_GOST-cit	0
Not_GOST-art	0	Not_GOST-cit	0
Not_GOST-art	0	Not_GOST-cit	0
Not_GOST-art	0	Not_GOST-cit	0
Not_GOST-art	0	Not_GOST-cit	0
Not_GOST-art	0	Not_GOST-cit	0

Not_GOST-art	0	Not_GOST-cit	0
Not_GOST-art	0	Not_GOST-cit	0
Not_GOST-art	0	Not_GOST-cit	0
Not_GOST-art	0	Not_GOST-cit	0
Not_GOST-art	0	Not_GOST-cit	0
Not_GOST-art	0	Not_GOST-cit	0
Not_GOST-art	0	Not_GOST-cit	0
Not_GOST-art	0	Not_GOST-cit	0
Not_GOST-art	0	Not_GOST-cit	0
Not_GOST-art	0	Not_GOST-cit	0
Not_GOST-art	0	Not_GOST-cit	0
Not_GOST-art	0	Not_GOST-cit	0
Not_GOST-art	0	Not_GOST-cit	0
Not_GOST-art	0	Not_GOST-cit	0
Not_GOST-art	0	Not_GOST-cit	0
Not_GOST-art	0	Not_GOST-cit	0
Not_GOST-art	0	Not_GOST-cit	0
Not_GOST-art	0	Not_GOST-cit	0
Not_GOST-art	0	Not_GOST-cit	0
Not_GOST-art	0	Not_GOST-cit	0
Not_GOST-art	0	Not_GOST-cit	0
Not_GOST-art	0	Not_GOST-cit	0
Not_GOST-art	0	Not_GOST-cit	0
Not_GOST-art	0	Not_GOST-cit	0
GOST-art-Y-al	1	Not_GOST-cit	0
Not_GOST-art	0	Not_GOST-cit	0
Not_GOST-art	0	Not_GOST-cit	0
Not_GOST-art	0	Not_GOST-cit	0
Not_GOST-art	0	Not_GOST-cit	0
Not_GOST-art	0	Not_GOST-cit	0
Not_GOST-art	0	Not_GOST-cit	0
Not_GOST-art	0	Not_GOST-cit	0
Not_GOST-art	0	Not_GOST-cit	0
Not_GOST-art	0	Not_GOST-cit	0
Not_GOST-art	0	Not_GOST-cit	0
Not_GOST-art	0	Not_GOST-cit	0
Not_GOST-art	0	Not_GOST-cit	0
Not_GOST-art	0	Not_GOST-cit	0
Not_GOST-art	0	Not_GOST-cit	0
Not_GOST-art	0	Not_GOST-cit	0
Not_GOST-art	0	Not_GOST-cit	0
Not_GOST-art	0	Not_GOST-cit	0
Not_GOST-art	0	Not_GOST-cit	0
Not_GOST-art	0	Not_GOST-cit	0
Not_GOST-art	0	Not_GOST-cit	0
Not_GOST-art	0	Not_GOST-cit	0
GOST-art-Y-al	1	Not_GOST-cit	0

Not_GOST-art	0	Not_GOST-cit	0
Not_GOST-art	0	Not_GOST-cit	0
Not_GOST-art	0	Not_GOST-cit	0
Not_GOST-art	0	Not_GOST-cit	0
Not_GOST-art	0	Not_GOST-cit	0
Not_GOST-art	0	Not_GOST-cit	0
Not_GOST-art	0	Not_GOST-cit	0
Not_GOST-art	0	Not_GOST-cit	0
Not_GOST-art	0	Not_GOST-cit	0
Not_GOST-art	0	Not_GOST-cit	0
Not_GOST-art	0	Not_GOST-cit	0
Not_GOST-art	0	Not_GOST-cit	0
Not_GOST-art	0	Not_GOST-cit	0
Not_GOST-art	0	Not_GOST-cit	0
Not_GOST-art	0	Not_GOST-cit	0
Not_GOST-art	0	Not_GOST-cit	0
Not_GOST-art	0	Not_GOST-cit	0
Not_GOST-art	0	Not_GOST-cit	0
Not_GOST-art	0	Not_GOST-cit	0
Not_GOST-art	0	Not_GOST-cit	0
Not_GOST-art	0	Not_GOST-cit	0
Not_GOST-art	0	Not_GOST-cit	0
Not_GOST-art	0	Not_GOST-cit	0
Not_GOST-art	0	Not_GOST-cit	0
Not_GOST-art	0	Not_GOST-cit	0
Not_GOST-art	0	Not_GOST-cit	0
Not_GOST-art	0	Not_GOST-cit	0
Not_GOST-art	0	Not_GOST-cit	0
Not_GOST-art	0	Not_GOST-cit	0
GOST-art-Y-al	1	GOST-cit-Y-all	1
GOST-art-Y-al	1	GOST-cit-Y-all	1
Not_GOST-art	0	Not_GOST-cit	0
Not_GOST-art	0	Not_GOST-cit	0
Not_GOST-art	0	Not_GOST-cit	0
Not_GOST-art	0	Not_GOST-cit	0
Not_GOST-art	0	Not_GOST-cit	0
Not_GOST-art	0	Not_GOST-cit	0
Not_GOST-art	0	Not_GOST-cit	0
Not_GOST-art	0	Not_GOST-cit	0
Not_GOST-art	0	Not_GOST-cit	0
Not_GOST-art	0	Not_GOST-cit	0
Not_GOST-art	0	Not_GOST-cit	0
Not_GOST-art	0	Not_GOST-cit	0
Not_GOST-art	0	Not_GOST-cit	0
Not_GOST-art	0	Not_GOST-cit	0
Not_GOST-art	0	Not_GOST-cit	0
Not_GOST-art	0	Not_GOST-cit	0

Not_GOST-art	0	Not_GOST-cit	0
Not_GOST-art	0	Not_GOST-cit	0
Not_GOST-art	0	Not_GOST-cit	0
Not_GOST-art	0	Not_GOST-cit	0
Not_GOST-art	0	Not_GOST-cit	0
Not_GOST-art	0	Not_GOST-cit	0
Not_GOST-art	0	Not_GOST-cit	0
Not_GOST-art	0	Not_GOST-cit	0
Not_GOST-art	0	Not_GOST-cit	0
Not_GOST-art	0	Not_GOST-cit	0
Not_GOST-art	0	Not_GOST-cit	0
Not_GOST-art	0	Not_GOST-cit	0
Not_GOST-art	0	Not_GOST-cit	0
Not_GOST-art	0	Not_GOST-cit	0
Not_GOST-art	0	Not_GOST-cit	0
Not_GOST-art	0	Not_GOST-cit	0
Not_GOST-art	0	Not_GOST-cit	0
Not_GOST-art	0	Not_GOST-cit	0
Not_GOST-art	0	Not_GOST-cit	0
Not_GOST-art	0	Not_GOST-cit	0
Not_GOST-art	0	Not_GOST-cit	0
Not_GOST-art	0	Not_GOST-cit	0
Not_GOST-art	0	Not_GOST-cit	0
Not_GOST-art	0	Not_GOST-cit	0
Not_GOST-art	0	Not_GOST-cit	0
Not_GOST-art	0	Not_GOST-cit	0
Not_GOST-art	0	Not_GOST-cit	0
Not_GOST-art	0	Not_GOST-cit	0
Not_GOST-art	0	Not_GOST-cit	0
Not_GOST-art	0	Not_GOST-cit	0
Not_GOST-art	0	Not_GOST-cit	0
Not_GOST-art	0	Not_GOST-cit	0
Not_GOST-art	0	Not_GOST-cit	0
Not_GOST-art	0	Not_GOST-cit	0
Not_GOST-art	0	Not_GOST-cit	0
Not_GOST-art	0	Not_GOST-cit	0
Not_GOST-art	0	Not_GOST-cit	0
Not_GOST-art	0	Not_GOST-cit	0
Not_GOST-art	0	Not_GOST-cit	0
Not_GOST-art	0	Not_GOST-cit	0
Not_GOST-art	0	Not_GOST-cit	0
Not_GOST-art	0	Not_GOST-cit	0
Not_GOST-art	0	Not_GOST-cit	0
Not_GOST-art	0	Not_GOST-cit	0
Not_GOST-art	0	Not_GOST-cit	0
Not_GOST-art	0	Not_GOST-cit	0
Not_GOST-art	0	Not_GOST-cit	0

Not_GOST-art	0	Not_GOST-cit	0
Not_GOST-art	0	Not_GOST-cit	0
Not_GOST-art	0	Not_GOST-cit	0
Not_GOST-art	0	Not_GOST-cit	0
GOST-art-Y-al	1	Not_GOST-cit	0
Not_GOST-art	0	Not_GOST-cit	0
Not_GOST-art	0	Not_GOST-cit	0
Not_GOST-art	0	Not_GOST-cit	0
Not_GOST-art	0	Not_GOST-cit	0
Not_GOST-art	0	Not_GOST-cit	0
Not_GOST-art	0	Not_GOST-cit	0
Not_GOST-art	0	Not_GOST-cit	0
Not_GOST-art	0	Not_GOST-cit	0
Not_GOST-art	0	Not_GOST-cit	0
Not_GOST-art	0	Not_GOST-cit	0
Not_GOST-art	0	Not_GOST-cit	0
Not_GOST-art	0	Not_GOST-cit	0
Not_GOST-art	0	Not_GOST-cit	0
Not_GOST-art	0	Not_GOST-cit	0
Not_GOST-art	0	Not_GOST-cit	0
Not_GOST-art	0	Not_GOST-cit	0
Not_GOST-art	0	Not_GOST-cit	0
Not_GOST-art	0	Not_GOST-cit	0
Not_GOST-art	0	Not_GOST-cit	0
Not_GOST-art	0	Not_GOST-cit	0
Not_GOST-art	0	Not_GOST-cit	0
Not_GOST-art	0	Not_GOST-cit	0
Not_GOST-art	0	Not_GOST-cit	0
Not_GOST-art	0	Not_GOST-cit	0
Not_GOST-art	0	Not_GOST-cit	0
Not_GOST-art	0	Not_GOST-cit	0
Not_GOST-art	0	Not_GOST-cit	0
Not_GOST-art	0	Not_GOST-cit	0
Not_GOST-art	0	Not_GOST-cit	0
Not_GOST-art	0	Not_GOST-cit	0
Not_GOST-art	0	Not_GOST-cit	0
Not_GOST-art	0	Not_GOST-cit	0
Not_GOST-art	0	Not_GOST-cit	0
Not_GOST-art	0	Not_GOST-cit	0
Not_GOST-art	0	Not_GOST-cit	0
Not_GOST-art	0	Not_GOST-cit	0
Not_GOST-art	0	Not_GOST-cit	0
Not_GOST-art	0	Not_GOST-cit	0
Not_GOST-art	0	Not_GOST-cit	0
Not_GOST-art	0	Not_GOST-cit	0
Not_GOST-art	0	Not_GOST-cit	0
Not_GOST-art	0	Not_GOST-cit	0

Not_GOST-art	0	Not_GOST-cit	0
Not_GOST-art	0	Not_GOST-cit	0
Not_GOST-art	0	Not_GOST-cit	0
Not_GOST-art	0	Not_GOST-cit	0
Not_GOST-art	0	Not_GOST-cit	0
Not_GOST-art	0	Not_GOST-cit	0
Not_GOST-art	0	Not_GOST-cit	0
Not_GOST-art	0	Not_GOST-cit	0
Not_GOST-art	0	Not_GOST-cit	0
Not_GOST-art	0	Not_GOST-cit	0
Not_GOST-art	0	Not_GOST-cit	0
Not_GOST-art	0	Not_GOST-cit	0
Not_GOST-art	0	Not_GOST-cit	0
Not_GOST-art	0	Not_GOST-cit	0
Not_GOST-art	0	Not_GOST-cit	0
Not_GOST-art	0	Not_GOST-cit	0
Not_GOST-art	0	Not_GOST-cit	0
Not_GOST-art	0	Not_GOST-cit	0
Not_GOST-art	0	Not_GOST-cit	0
Not_GOST-art	0	Not_GOST-cit	0
Not_GOST-art	0	Not_GOST-cit	0
Not_GOST-art	0	Not_GOST-cit	0
Not_GOST-art	0	Not_GOST-cit	0
Not_GOST-art	0	Not_GOST-cit	0
Not_GOST-art	0	Not_GOST-cit	0
Not_GOST-art	0	Not_GOST-cit	0
Not_GOST-art	0	Not_GOST-cit	0
Not_GOST-art	0	Not_GOST-cit	0
Not_GOST-art	0	Not_GOST-cit	0
Not_GOST-art	0	Not_GOST-cit	0
Not_GOST-art	0	Not_GOST-cit	0
Not_GOST-art	0	Not_GOST-cit	0
Not_GOST-art	0	Not_GOST-cit	0
Not_GOST-art	0	Not_GOST-cit	0
Not_GOST-art	0	Not_GOST-cit	0
Not_GOST-art	0	Not_GOST-cit	0
Not_GOST-art	0	Not_GOST-cit	0
Not_GOST-art	0	Not_GOST-cit	0
Not_GOST-art	0	Not_GOST-cit	0
Not_GOST-art	0	Not_GOST-cit	0
Not_GOST-art	0	Not_GOST-cit	0
Not_GOST-art	0	Not_GOST-cit	0
Not_GOST-art	0	Not_GOST-cit	0
Not_GOST-art	0	Not_GOST-cit	0
Not_GOST-art	0	Not_GOST-cit	0
Not_GOST-art	0	Not_GOST-cit	0
Not_GOST-art	0	Not_GOST-cit	0

Not_GOST-art	0	Not_GOST-cit	0
Not_GOST-art	0	Not_GOST-cit	0
Not_GOST-art	0	Not_GOST-cit	0
Not_GOST-art	0	Not_GOST-cit	0
Not_GOST-art	0	Not_GOST-cit	0
Not_GOST-art	0	Not_GOST-cit	0
Not_GOST-art	0	Not_GOST-cit	0
Not_GOST-art	0	Not_GOST-cit	0
Not_GOST-art	0	Not_GOST-cit	0
Not_GOST-art	0	Not_GOST-cit	0
Not_GOST-art	0	Not_GOST-cit	0
Not_GOST-art	0	Not_GOST-cit	0
Not_GOST-art	0	Not_GOST-cit	0
GOST-art-Y-al	1	Not_GOST-cit	0
GOST-art-Y-al	1	Not_GOST-cit	0
GOST-art-Y-al	1	Not_GOST-cit	0
Not_GOST-art	0	Not_GOST-cit	0
Not_GOST-art	0	Not_GOST-cit	0
Not_GOST-art	0	Not_GOST-cit	0
Not_GOST-art	0	Not_GOST-cit	0
Not_GOST-art	0	Not_GOST-cit	0
Not_GOST-art	0	Not_GOST-cit	0
Not_GOST-art	0	Not_GOST-cit	0
Not_GOST-art	0	Not_GOST-cit	0
Not_GOST-art	0	Not_GOST-cit	0
Not_GOST-art	0	Not_GOST-cit	0
Not_GOST-art	0	Not_GOST-cit	0
Not_GOST-art	0	Not_GOST-cit	0
Not_GOST-art	0	Not_GOST-cit	0
Not_GOST-art	0	Not_GOST-cit	0
Not_GOST-art	0	Not_GOST-cit	0
Not_GOST-art	0	Not_GOST-cit	0
Not_GOST-art	0	Not_GOST-cit	0
Not_GOST-art	0	Not_GOST-cit	0
Not_GOST-art	0	Not_GOST-cit	0
Not_GOST-art	0	Not_GOST-cit	0
Not_GOST-art	0	Not_GOST-cit	0
Not_GOST-art	0	Not_GOST-cit	0
Not_GOST-art	0	Not_GOST-cit	0
Not_GOST-art	0	Not_GOST-cit	0
Not_GOST-art	0	Not_GOST-cit	0
Not_GOST-art	0	Not_GOST-cit	0
GOST-art-Y-al	1	Not_GOST-cit	0
Not_GOST-art	0	Not_GOST-cit	0
GOST-art-Y-al	1	Not_GOST-cit	0
Not_GOST-art	0	Not_GOST-cit	0
Not_GOST-art	0	Not_GOST-cit	0

Not_GOST-art	0	Not_GOST-cit	0
Not_GOST-art	0	Not_GOST-cit	0
Not_GOST-art	0	Not_GOST-cit	0
Not_GOST-art	0	Not_GOST-cit	0
Not_GOST-art	0	Not_GOST-cit	0
Not_GOST-art	0	Not_GOST-cit	0
GOST-art-Y-al	1	Not_GOST-cit	0
Not_GOST-art	0	Not_GOST-cit	0
Not_GOST-art	0	Not_GOST-cit	0
Not_GOST-art	0	Not_GOST-cit	0
Not_GOST-art	0	Not_GOST-cit	0
GOST-art-Y-al	1	Not_GOST-cit	0
GOST-art-Y-al	1	Not_GOST-cit	0
Not_GOST-art	0	Not_GOST-cit	0
Not_GOST-art	0	Not_GOST-cit	0
Not_GOST-art	0	Not_GOST-cit	0
Not_GOST-art	0	Not_GOST-cit	0
Not_GOST-art	0	Not_GOST-cit	0
Not_GOST-art	0	Not_GOST-cit	0
Not_GOST-art	0	Not_GOST-cit	0
Not_GOST-art	0	Not_GOST-cit	0
Not_GOST-art	0	GOST-cit-Y-all	1
Not_GOST-art	0	GOST-cit-Y-all	1
Not_GOST-art	0	GOST-cit-Y-all	1
Not_GOST-art	0	Not_GOST-cit	0
Not_GOST-art	0	Not_GOST-cit	0
Not_GOST-art	0	Not_GOST-cit	0
GOST-art-Y-al	1	Not_GOST-cit	0
GOST-art-Y-al	1	Not_GOST-cit	0
GOST-art-Y-al	1	Not_GOST-cit	0
Not_GOST-art	0	Not_GOST-cit	0
Not_GOST-art	0	Not_GOST-cit	0
Not_GOST-art	0	Not_GOST-cit	0
Not_GOST-art	0	Not_GOST-cit	0
Not_GOST-art	0	Not_GOST-cit	0
Not_GOST-art	0	Not_GOST-cit	0
Not_GOST-art	0	Not_GOST-cit	0
Not_GOST-art	0	Not_GOST-cit	0
Not_GOST-art	0	Not_GOST-cit	0
Not_GOST-art	0	Not_GOST-cit	0
Not_GOST-art	0	Not_GOST-cit	0
Not_GOST-art	0	Not_GOST-cit	0
Not_GOST-art	0	Not_GOST-cit	0
Not_GOST-art	0	Not_GOST-cit	0
Not_GOST-art	0	Not_GOST-cit	0
Not_GOST-art	0	Not_GOST-cit	0
Not_GOST-art	0	Not_GOST-cit	0

Not_GOST-art	0	Not_GOST-cit	0
Not_GOST-art	0	Not_GOST-cit	0
Not_GOST-art	0	Not_GOST-cit	0
Not_GOST-art	0	Not_GOST-cit	0
Not_GOST-art	0	Not_GOST-cit	0
Not_GOST-art	0	Not_GOST-cit	0
Not_GOST-art	0	Not_GOST-cit	0
GOST-art-Y-al	1	GOST-cit-Y-all	1
GOST-art-Y-al	1	GOST-cit-Y-all	1
GOST-art-Y-al	1	GOST-cit-Y-all	1
GOST-art-Y-al	1	GOST-cit-Y-all	1
GOST-art-Y-al	1	GOST-cit-Y-all	1
GOST-art-Y-al	1	GOST-cit-Y-all	1
GOST-art-Y-al	1	GOST-cit-Y-all	1
Not_GOST-art	0	Not_GOST-cit	0
Not_GOST-art	0	Not_GOST-cit	0
Not_GOST-art	0	Not_GOST-cit	0
Not_GOST-art	0	Not_GOST-cit	0
Not_GOST-art	0	Not_GOST-cit	0
Not_GOST-art	0	Not_GOST-cit	0
Not_GOST-art	0	Not_GOST-cit	0
Not_GOST-art	0	Not_GOST-cit	0
Not_GOST-art	0	Not_GOST-cit	0
Not_GOST-art	0	Not_GOST-cit	0
Not_GOST-art	0	Not_GOST-cit	0
Not_GOST-art	0	Not_GOST-cit	0
Not_GOST-art	0	Not_GOST-cit	0
Not_GOST-art	0	Not_GOST-cit	0
Not_GOST-art	0	Not_GOST-cit	0
Not_GOST-art	0	Not_GOST-cit	0
Not_GOST-art	0	Not_GOST-cit	0
Not_GOST-art	0	Not_GOST-cit	0
Not_GOST-art	0	Not_GOST-cit	0
Not_GOST-art	0	Not_GOST-cit	0
Not_GOST-art	0	Not_GOST-cit	0
Not_GOST-art	0	Not_GOST-cit	0
Not_GOST-art	0	Not_GOST-cit	0
Not_GOST-art	0	Not_GOST-cit	0
Not_GOST-art	0	Not_GOST-cit	0
Not_GOST-art	0	Not_GOST-cit	0
Not_GOST-art	0	Not_GOST-cit	0
GOST-art-Y-al	1	Not_GOST-cit	0
Not_GOST-art	0	Not_GOST-cit	0
GOST-art-Y-al	1	Not_GOST-cit	0
GOST-art-Y-al	1	Not_GOST-cit	0
GOST-art-Y-al	1	Not_GOST-cit	0
Not_GOST-art	0	Not_GOST-cit	0

Not_GOST-art	0	Not_GOST-cit	0
Not_GOST-art	0	Not_GOST-cit	0
Not_GOST-art	0	Not_GOST-cit	0
Not_GOST-art	0	Not_GOST-cit	0
Not_GOST-art	0	Not_GOST-cit	0
Not_GOST-art	0	Not_GOST-cit	0
Not_GOST-art	0	Not_GOST-cit	0
Not_GOST-art	0	GOST-cit-Y-all	1
Not_GOST-art	0	GOST-cit-Y-all	1
Not_GOST-art	0	Not_GOST-cit	0
Not_GOST-art	0	Not_GOST-cit	0
Not_GOST-art	0	Not_GOST-cit	0
Not_GOST-art	0	Not_GOST-cit	0
Not_GOST-art	0	Not_GOST-cit	0
Not_GOST-art	0	Not_GOST-cit	0
Not_GOST-art	0	Not_GOST-cit	0
Not_GOST-art	0	Not_GOST-cit	0
Not_GOST-art	0	Not_GOST-cit	0
Not_GOST-art	0	Not_GOST-cit	0
Not_GOST-art	0	Not_GOST-cit	0
Not_GOST-art	0	Not_GOST-cit	0
Not_GOST-art	0	Not_GOST-cit	0
Not_GOST-art	0	Not_GOST-cit	0
Not_GOST-art	0	Not_GOST-cit	0
GOST-art-Y-al	1	GOST-cit-Y-all	1
Not_GOST-art	0	Not_GOST-cit	0
Not_GOST-art	0	Not_GOST-cit	0
Not_GOST-art	0	Not_GOST-cit	0
Not_GOST-art	0	Not_GOST-cit	0
Not_GOST-art	0	Not_GOST-cit	0
Not_GOST-art	0	Not_GOST-cit	0
Not_GOST-art	0	Not_GOST-cit	0
Not_GOST-art	0	Not_GOST-cit	0
Not_GOST-art	0	Not_GOST-cit	0
Not_GOST-art	0	Not_GOST-cit	0
Not_GOST-art	0	Not_GOST-cit	0
Not_GOST-art	0	Not_GOST-cit	0
Not_GOST-art	0	Not_GOST-cit	0
Not_GOST-art	0	Not_GOST-cit	0
Not_GOST-art	0	Not_GOST-cit	0
Not_GOST-art	0	Not_GOST-cit	0
Not_GOST-art	0	Not_GOST-cit	0
Not_GOST-art	0	GOST-cit-Y-all	1
GOST-art-Y-al	1	GOST-cit-Y-all	1
GOST-art-Y-al	1	GOST-cit-Y-all	1
GOST-art-Y-al	1	GOST-cit-Y-all	1
GOST-art-Y-al	1	GOST-cit-Y-all	1

Not_GOST-art	0	Not_GOST-cit	0
Not_GOST-art	0	Not_GOST-cit	0
Not_GOST-art	0	Not_GOST-cit	0
Not_GOST-art	0	Not_GOST-cit	0
Not_GOST-art	0	Not_GOST-cit	0
Not_GOST-art	0	Not_GOST-cit	0
Not_GOST-art	0	Not_GOST-cit	0
Not_GOST-art	0	Not_GOST-cit	0
Not_GOST-art	0	Not_GOST-cit	0
Not_GOST-art	0	Not_GOST-cit	0
Not_GOST-art	0	Not_GOST-cit	0
GOST-art-Y-al	1	Not_GOST-cit	0
Not_GOST-art	0	Not_GOST-cit	0
Not_GOST-art	0	Not_GOST-cit	0
Not_GOST-art	0	Not_GOST-cit	0
Not_GOST-art	0	Not_GOST-cit	0
Not_GOST-art	0	Not_GOST-cit	0
Not_GOST-art	0	Not_GOST-cit	0
Not_GOST-art	0	Not_GOST-cit	0
Not_GOST-art	0	Not_GOST-cit	0
Not_GOST-art	0	Not_GOST-cit	0
Not_GOST-art	0	Not_GOST-cit	0
Not_GOST-art	0	Not_GOST-cit	0
Not_GOST-art	0	Not_GOST-cit	0
Not_GOST-art	0	Not_GOST-cit	0
Not_GOST-art	0	Not_GOST-cit	0
Not_GOST-art	0	Not_GOST-cit	0
Not_GOST-art	0	Not_GOST-cit	0
Not_GOST-art	0	Not_GOST-cit	0
Not_GOST-art	0	Not_GOST-cit	0
Not_GOST-art	0	Not_GOST-cit	0
Not_GOST-art	0	Not_GOST-cit	0
Not_GOST-art	0	Not_GOST-cit	0
Not_GOST-art	0	Not_GOST-cit	0
Not_GOST-art	0	GOST-cit-Y-all	1
Not_GOST-art	0	GOST-cit-Y-all	1
Not_GOST-art	0	Not_GOST-cit	0
Not_GOST-art	0	Not_GOST-cit	0
Not_GOST-art	0	Not_GOST-cit	0
Not_GOST-art	0	Not_GOST-cit	0
Not_GOST-art	0	Not_GOST-cit	0
Not_GOST-art	0	Not_GOST-cit	0
Not_GOST-art	0	Not_GOST-cit	0
Not_GOST-art	0	Not_GOST-cit	0
Not_GOST-art	0	Not_GOST-cit	0
Not_GOST-art	0	Not_GOST-cit	0
Not_GOST-art	0	Not_GOST-cit	0

Not_GOST-art	0	Not_GOST-cit	0
Not_GOST-art	0	Not_GOST-cit	0
Not_GOST-art	0	Not_GOST-cit	0
Not_GOST-art	0	Not_GOST-cit	0
Not_GOST-art	0	Not_GOST-cit	0
Not_GOST-art	0	Not_GOST-cit	0
Not_GOST-art	0	Not_GOST-cit	0
Not_GOST-art	0	Not_GOST-cit	0
GOST-art-Y-al	1	Not_GOST-cit	0
GOST-art-Y-al	1	Not_GOST-cit	0
GOST-art-Y-al	1	Not_GOST-cit	0
GOST-art-Y-al	1	Not_GOST-cit	0
GOST-art-Y-al	1	Not_GOST-cit	0
Not_GOST-art	0	Not_GOST-cit	0
Not_GOST-art	0	Not_GOST-cit	0
Not_GOST-art	0	Not_GOST-cit	0
Not_GOST-art	0	Not_GOST-cit	0
Not_GOST-art	0	Not_GOST-cit	0
Not_GOST-art	0	Not_GOST-cit	0
Not_GOST-art	0	Not_GOST-cit	0
Not_GOST-art	0	Not_GOST-cit	0
Not_GOST-art	0	Not_GOST-cit	0
Not_GOST-art	0	Not_GOST-cit	0
Not_GOST-art	0	Not_GOST-cit	0
Not_GOST-art	0	Not_GOST-cit	0
Not_GOST-art	0	Not_GOST-cit	0
Not_GOST-art	0	Not_GOST-cit	0
Not_GOST-art	0	Not_GOST-cit	0
Not_GOST-art	0	Not_GOST-cit	0
Not_GOST-art	0	Not_GOST-cit	0
Not_GOST-art	0	Not_GOST-cit	0
Not_GOST-art	0	Not_GOST-cit	0
Not_GOST-art	0	Not_GOST-cit	0
Not_GOST-art	0	Not_GOST-cit	0
Not_GOST-art	0	Not_GOST-cit	0
Not_GOST-art	0	Not_GOST-cit	0
Not_GOST-art	0	Not_GOST-cit	0
Not_GOST-art	0	Not_GOST-cit	0
Not_GOST-art	0	Not_GOST-cit	0
Not_GOST-art	0	Not_GOST-cit	0
Not_GOST-art	0	Not_GOST-cit	0
Not_GOST-art	0	Not_GOST-cit	0
Not_GOST-art	0	Not_GOST-cit	0
Not_GOST-art	0	Not_GOST-cit	0
Not_GOST-art	0	Not_GOST-cit	0
Not_GOST-art	0	Not_GOST-cit	0
Not_GOST-art	0	Not_GOST-cit	0

Not_GOST-art	0	Not_GOST-cit	0
Not_GOST-art	0	GOST-cit-Y-all	1
Not_GOST-art	0	Not_GOST-cit	0
Not_GOST-art	0	Not_GOST-cit	0
Not_GOST-art	0	Not_GOST-cit	0
Not_GOST-art	0	Not_GOST-cit	0
Not_GOST-art	0	Not_GOST-cit	0
Not_GOST-art	0	Not_GOST-cit	0
Not_GOST-art	0	Not_GOST-cit	0
Not_GOST-art	0	Not_GOST-cit	0
Not_GOST-art	0	Not_GOST-cit	0
Not_GOST-art	0	Not_GOST-cit	0
Not_GOST-art	0	Not_GOST-cit	0
Not_GOST-art	0	Not_GOST-cit	0
Not_GOST-art	0	Not_GOST-cit	0
Not_GOST-art	0	Not_GOST-cit	0
Not_GOST-art	0	Not_GOST-cit	0
Not_GOST-art	0	Not_GOST-cit	0
Not_GOST-art	0	Not_GOST-cit	0
Not_GOST-art	0	Not_GOST-cit	0
Not_GOST-art	0	Not_GOST-cit	0
Not_GOST-art	0	Not_GOST-cit	0
Not_GOST-art	0	Not_GOST-cit	0
Not_GOST-art	0	Not_GOST-cit	0
Not_GOST-art	0	Not_GOST-cit	0
Not_GOST-art	0	Not_GOST-cit	0
Not_GOST-art	0	Not_GOST-cit	0
Not_GOST-art	0	GOST-cit-Y-all	1
Not_GOST-art	0	GOST-cit-Y-all	1
Not_GOST-art	0	Not_GOST-cit	0
Not_GOST-art	0	Not_GOST-cit	0
Not_GOST-art	0	Not_GOST-cit	0
Not_GOST-art	0	Not_GOST-cit	0
Not_GOST-art	0	Not_GOST-cit	0
Not_GOST-art	0	Not_GOST-cit	0
Not_GOST-art	0	Not_GOST-cit	0
Not_GOST-art	0	Not_GOST-cit	0
Not_GOST-art	0	Not_GOST-cit	0
Not_GOST-art	0	Not_GOST-cit	0
Not_GOST-art	0	Not_GOST-cit	0
GOST-art-Y-al	1	Not_GOST-cit	0
GOST-art-Y-al	1	Not_GOST-cit	0
GOST-art-Y-al	1	Not_GOST-cit	0
Not_GOST-art	0	Not_GOST-cit	0
Not_GOST-art	0	Not_GOST-cit	0
Not_GOST-art	0	Not_GOST-cit	0
Not_GOST-art	0	Not_GOST-cit	0

Not_GOST-art	0	Not_GOST-cit	0
Not_GOST-art	0	Not_GOST-cit	0
Not_GOST-art	0	Not_GOST-cit	0
Not_GOST-art	0	Not_GOST-cit	0
Not_GOST-art	0	Not_GOST-cit	0
Not_GOST-art	0	Not_GOST-cit	0
Not_GOST-art	0	Not_GOST-cit	0
Not_GOST-art	0	Not_GOST-cit	0
Not_GOST-art	0	Not_GOST-cit	0
Not_GOST-art	0	Not_GOST-cit	0
Not_GOST-art	0	Not_GOST-cit	0
Not_GOST-art	0	Not_GOST-cit	0
Not_GOST-art	0	Not_GOST-cit	0
Not_GOST-art	0	Not_GOST-cit	0
Not_GOST-art	0	Not_GOST-cit	0
Not_GOST-art	0	Not_GOST-cit	0
Not_GOST-art	0	Not_GOST-cit	0
Not_GOST-art	0	Not_GOST-cit	0
Not_GOST-art	0	Not_GOST-cit	0
Not_GOST-art	0	Not_GOST-cit	0
Not_GOST-art	0	Not_GOST-cit	0
Not_GOST-art	0	Not_GOST-cit	0
Not_GOST-art	0	Not_GOST-cit	0
Not_GOST-art	0	Not_GOST-cit	0
Not_GOST-art	0	Not_GOST-cit	0
Not_GOST-art	0	Not_GOST-cit	0
Not_GOST-art	0	Not_GOST-cit	0
Not_GOST-art	0	Not_GOST-cit	0
Not_GOST-art	0	Not_GOST-cit	0
Not_GOST-art	0	Not_GOST-cit	0
Not_GOST-art	0	Not_GOST-cit	0
Not_GOST-art	0	Not_GOST-cit	0
GOST-art-Y-al	1	Not_GOST-cit	0
GOST-art-Y-al	1	Not_GOST-cit	0
GOST-art-Y-al	1	Not_GOST-cit	0
Not_GOST-art	0	Not_GOST-cit	0
Not_GOST-art	0	Not_GOST-cit	0
Not_GOST-art	0	Not_GOST-cit	0
Not_GOST-art	0	Not_GOST-cit	0
Not_GOST-art	0	Not_GOST-cit	0
Not_GOST-art	0	Not_GOST-cit	0
Not_GOST-art	0	Not_GOST-cit	0
Not_GOST-art	0	Not_GOST-cit	0
GOST-art-Y-al	1	Not_GOST-cit	0
Not_GOST-art	0	Not_GOST-cit	0
Not_GOST-art	0	Not_GOST-cit	0
Not_GOST-art	0	Not_GOST-cit	0

Not_GOST-art	0	Not_GOST-cit	0
Not_GOST-art	0	Not_GOST-cit	0
Not_GOST-art	0	Not_GOST-cit	0
Not_GOST-art	0	Not_GOST-cit	0
Not_GOST-art	0	Not_GOST-cit	0
Not_GOST-art	0	Not_GOST-cit	0
Not_GOST-art	0	Not_GOST-cit	0
Not_GOST-art	0	Not_GOST-cit	0
Not_GOST-art	0	Not_GOST-cit	0
Not_GOST-art	0	Not_GOST-cit	0
Not_GOST-art	0	Not_GOST-cit	0
Not_GOST-art	0	Not_GOST-cit	0
GOST-art-Y-al	1	GOST-cit-Y-all	1
GOST-art-Y-al	1	GOST-cit-Y-all	1
GOST-art-Y-al	1	GOST-cit-Y-all	1
Not_GOST-art	0	Not_GOST-cit	0
Not_GOST-art	0	Not_GOST-cit	0
Not_GOST-art	0	Not_GOST-cit	0
Not_GOST-art	0	Not_GOST-cit	0
Not_GOST-art	0	Not_GOST-cit	0
Not_GOST-art	0	Not_GOST-cit	0
Not_GOST-art	0	Not_GOST-cit	0
Not_GOST-art	0	Not_GOST-cit	0
Not_GOST-art	0	Not_GOST-cit	0
Not_GOST-art	0	Not_GOST-cit	0
Not_GOST-art	0	Not_GOST-cit	0
Not_GOST-art	0	Not_GOST-cit	0
Not_GOST-art	0	Not_GOST-cit	0
Not_GOST-art	0	Not_GOST-cit	0
Not_GOST-art	0	Not_GOST-cit	0
Not_GOST-art	0	Not_GOST-cit	0
Not_GOST-art	0	Not_GOST-cit	0
Not_GOST-art	0	Not_GOST-cit	0
Not_GOST-art	0	Not_GOST-cit	0
Not_GOST-art	0	Not_GOST-cit	0
Not_GOST-art	0	Not_GOST-cit	0
Not_GOST-art	0	Not_GOST-cit	0
Not_GOST-art	0	Not_GOST-cit	0
Not_GOST-art	0	Not_GOST-cit	0
Not_GOST-art	0	Not_GOST-cit	0
Not_GOST-art	0	Not_GOST-cit	0
Not_GOST-art	0	Not_GOST-cit	0
Not_GOST-art	0	Not_GOST-cit	0
Not_GOST-art	0	Not_GOST-cit	0
Not_GOST-art	0	Not_GOST-cit	0
Not_GOST-art	0	Not_GOST-cit	0
Not_GOST-art	0	Not_GOST-cit	0

Not_GOST-art	0	Not_GOST-cit	0
Not_GOST-art	0	Not_GOST-cit	0
Not_GOST-art	0	Not_GOST-cit	0
Not_GOST-art	0	Not_GOST-cit	0
Not_GOST-art	0	Not_GOST-cit	0
Not_GOST-art	0	Not_GOST-cit	0
Not_GOST-art	0	Not_GOST-cit	0
GOST-art-Y-al	1	Not_GOST-cit	0
GOST-art-Y-al	1	Not_GOST-cit	0
Not_GOST-art	0	Not_GOST-cit	0
Not_GOST-art	0	Not_GOST-cit	0
Not_GOST-art	0	GOST-cit-Y-all	1
Not_GOST-art	0	Not_GOST-cit	0
Not_GOST-art	0	Not_GOST-cit	0
Not_GOST-art	0	Not_GOST-cit	0
Not_GOST-art	0	Not_GOST-cit	0
GOST-art-Y-al	1	Not_GOST-cit	0
GOST-art-Y-al	1	Not_GOST-cit	0
GOST-art-Y-al	1	GOST-cit-Y-all	1
GOST-art-Y-al	1	GOST-cit-Y-all	1
GOST-art-Y-al	1	GOST-cit-Y-all	1
GOST-art-Y-al	1	GOST-cit-Y-all	1
Not_GOST-art	0	Not_GOST-cit	0
Not_GOST-art	0	Not_GOST-cit	0
Not_GOST-art	0	Not_GOST-cit	0
Not_GOST-art	0	Not_GOST-cit	0
Not_GOST-art	0	Not_GOST-cit	0
Not_GOST-art	0	Not_GOST-cit	0
Not_GOST-art	0	Not_GOST-cit	0
Not_GOST-art	0	Not_GOST-cit	0
Not_GOST-art	0	Not_GOST-cit	0
Not_GOST-art	0	Not_GOST-cit	0

easaarty	easaarty_b	ewagostart	ewagostart_b	only_ewagost	only_ewagost	nop_egostcit	easacity
	0		0		0		
	0		0		0		
	0		0		0		
	0		0		0		
E-ASA-art-Y	1		0		0		
E-ASA-art-Y	1		0		0		
E-ASA-art-Y	1		0		0		
E-ASA-art-Y	1		0		0		
	0		0		0		
	0		0		0		
	0	Ew/a-GOST-ar	1	ONLY_E-w/a-(	1		
	0		0		0		
	0		0		0		
	0		0		0		
	0		0		0		
	0		0		0		
	0		0		0		
	0		0		0		
	0		0		0		
	0		0		0		
	0		0		0		
	0		0		0		
	0	Ew/a-GOST-ar	1	ONLY_E-w/a-(	1		
	0	Ew/a-GOST-ar	1	ONLY_E-w/a-(	1		
	0	Ew/a-GOST-ar	1	ONLY_E-w/a-(	1		
	0		0		0		
	0		0		0		
	0		0		0		
	0		0		0		
	0	Ew/a-GOST-ar	1	ONLY_E-w/a-(	1		
	0		0		0		
	0		0		0		
	0		0		0		
	0		0		0		
	0		0		0		
	0		0		0		
	0		0		0		
	0		0		0		
	0		0		0		
	0		0		0		
	0		0		0		
	0		0		0		
	0	Ew/a-GOST-ar	1	ONLY_E-w/a-(	1		
	0	Ew/a-GOST-ar	1	ONLY_E-w/a-(	1		
	0	Ew/a-GOST-ar	1	ONLY_E-w/a-(	1		
	0		0		0		

	0		0		0	
	0		0		0	
	0	Ew/a-GOST-ar	1	ONLY_E-w/a-(	1	
	0		0		0	
	0	Ew/a-GOST-ar	1	ONLY_E-w/a-(	1	
	0		0		0	
	0		0		0	
E-ASA-art-Y	1	Ew/a-GOST-ar	1		0	
E-ASA-art-Y	1	Ew/a-GOST-ar	1		0	E-ASA-cit-Y
	0		0		0	
	0		0		0	
	0		0		0	
E-ASA-art-Y	1		0		0	
	0	Ew/a-GOST-ar	1	ONLY_E-w/a-(	1	
	0	Ew/a-GOST-ar	1	ONLY_E-w/a-(	1	
	0	Ew/a-GOST-ar	1	ONLY_E-w/a-(	1	
	0		0		0	
	0		0		0	
	0		0		0	
	0		0		0	
E-ASA-art-Y	1		0		0	
E-ASA-art-Y	1		0		0	
	0		0		0	
E-ASA-art-Y	1		0		0	
	0	Ew/a-GOST-ar	1	ONLY_E-w/a-(	1	
	0	Ew/a-GOST-ar	1	ONLY_E-w/a-(	1	
	0		0		0	
	0		0		0	
	0		0		0	
E-ASA-art-Y	1	Ew/a-GOST-ar	1		0	
E-ASA-art-Y	1	Ew/a-GOST-ar	1		0	
E-ASA-art-Y	1	Ew/a-GOST-ar	1		0	
E-ASA-art-Y	1	Ew/a-GOST-ar	1		0	
E-ASA-art-Y	1	Ew/a-GOST-ar	1		0	
	0		0		0	
	0		0		0	
	0		0		0	
	0		0		0	
	0		0		0	
	0		0		0	
	0		0		0	
	0		0		0	
	0		0		0	
	0		0		0	
	0		0		0	
	0	Ew/a-GOST-ar	1	ONLY_E-w/a-(	1	
	0		0		0	

	0		0		0	
	0		0		0	
	0		0		0	
	0	Ew/a-GOST-a	1	ONLY_E-w/a-	1	
	0		0		0	
	0		0		0	
	0		0		0	
	0		0		0	
	0	Ew/a-GOST-a	1	ONLY_E-w/a-	1	
	0		0		0	
	0		0		0	
	0		0		0	
	0		0		0	
	0	Ew/a-GOST-a	1	ONLY_E-w/a-	1	
	0		0		0	
	0		0		0	
	0		0		0	
	0		0		0	
	0		0		0	
	0		0		0	
	0	Ew/a-GOST-a	1	ONLY_E-w/a-	1	
	0		0		0	
	0		0		0	
	0		0		0	
	0	Ew/a-GOST-a	1	ONLY_E-w/a-	1	
	0	Ew/a-GOST-a	1	ONLY_E-w/a-	1	
	0	Ew/a-GOST-a	1	ONLY_E-w/a-	1	
E-ASA-art-Y	1		0		0	E-ASA-cit-Y
E-ASA-art-Y	1		0		0	E-ASA-cit-Y
E-ASA-art-Y	1	Ew/a-GOST-a	1		0	
E-ASA-art-Y	1	Ew/a-GOST-a	1		0	
	0		0		0	
	0		0		0	E-ASA-cit-Y
	0		0		0	E-ASA-cit-Y
	0	Ew/a-GOST-a	1	ONLY_E-w/a-	1	
E-ASA-art-Y	1	Ew/a-GOST-a	1		0	
E-ASA-art-Y	1	Ew/a-GOST-a	1		0	
E-ASA-art-Y	1	Ew/a-GOST-a	1		0	
E-ASA-art-Y	1	Ew/a-GOST-a	1		0	
	0	Ew/a-GOST-a	1	ONLY_E-w/a-	1	E-ASA-cit-Y
	0	Ew/a-GOST-a	1	ONLY_E-w/a-	1	
	0	Ew/a-GOST-a	1	ONLY_E-w/a-	1	
E-ASA-art-Y	1		0		0	
	0		0		0	
E-ASA-art-Y	1		0		0	
E-ASA-art-Y	1		0		0	
	0		0		0	

	0		0		0	
	0		0		0	
E-ASA-art-Y	1		0		0	
E-ASA-art-Y	1		0		0	
E-ASA-art-Y	1		0		0	
	0		0		0	
	0	Ew/a-GOST-a	1	ONLY_E-w/a-	1	
	0	Ew/a-GOST-a	1	ONLY_E-w/a-	1	
	0		0		0	
	0		0		0	
	0		0		0	
	0		0		0	E-ASA-cit-Y
	0		0		0	E-ASA-cit-Y
E-ASA-art-Y	1		0		0	E-ASA-cit-Y
	0		0		0	
	0		0		0	
E-ASA-art-Y	1	Ew/a-GOST-a	1		0	E-ASA-cit-Y
	0		0		0	
	0		0		0	
	0		0		0	
	0		0		0	
	0		0		0	
	0	Ew/a-GOST-a	1	ONLY_E-w/a-	1	
	0		0		0	
	0		0		0	
	0		0		0	
	0		0		0	
	0	Ew/a-GOST-a	1	ONLY_E-w/a-	1	
E-ASA-art-Y	1		0		0	
	0	Ew/a-GOST-a	1	ONLY_E-w/a-	1	
E-ASA-art-Y	1		0		0	
E-ASA-art-Y	1		0		0	
E-ASA-art-Y	1		0		0	E-ASA-cit-Y
	0		0		0	
	0	Ew/a-GOST-a	1	ONLY_E-w/a-	1	
	0	Ew/a-GOST-a	1	ONLY_E-w/a-	1	
	0		0		0	
	0	Ew/a-GOST-a	1	ONLY_E-w/a-	1	
	0	Ew/a-GOST-a	1	ONLY_E-w/a-	1	E-ASA-cit-Y
	0		0		0	
	0	Ew/a-GOST-a	1	ONLY_E-w/a-	1	
E-ASA-art-Y	1	Ew/a-GOST-a	1		0	
E-ASA-art-Y	1	Ew/a-GOST-a	1		0	
	0		0		0	
	0		0		0	
	0		0		0	
	0		0		0	

	0		0		0	
	0		0		0	
	0	Ew/a-GOST-ar	1	ONLY_E-w/a-(	1	
	0		0		0	
	0		0		0	E-ASA-cit-Y
	0		0		0	E-ASA-cit-Y
	0		0		0	
	0		0		0	
	0		0		0	
	0	Ew/a-GOST-ar	1	ONLY_E-w/a-(	1	
E-ASA-art-Y	1	Ew/a-GOST-ar	1		0	
E-ASA-art-Y	1	Ew/a-GOST-ar	1		0	
	0	Ew/a-GOST-ar	1	ONLY_E-w/a-(	1	
	0	Ew/a-GOST-ar	1	ONLY_E-w/a-(	1	
	0		0		0	
	0	Ew/a-GOST-ar	1	ONLY_E-w/a-(	1	
	0	Ew/a-GOST-ar	1	ONLY_E-w/a-(	1	
	0		0		0	
	0		0		0	
	0		0		0	
	0		0		0	
	0		0		0	
	0	Ew/a-GOST-ar	1	ONLY_E-w/a-(	1	
	0	Ew/a-GOST-ar	1	ONLY_E-w/a-(	1	
	0	Ew/a-GOST-ar	1	ONLY_E-w/a-(	1	
	0	Ew/a-GOST-ar	1	ONLY_E-w/a-(	1	
	0	Ew/a-GOST-ar	1	ONLY_E-w/a-(	1	
	0	Ew/a-GOST-ar	1	ONLY_E-w/a-(	1	
	0	Ew/a-GOST-ar	1	ONLY_E-w/a-(	1	
	0		0		0	
	0		0		0	
	0		0		0	
	0		0		0	
	0		0		0	
	0		0		0	E-ASA-cit-Y
	0		0		0	
	0	Ew/a-GOST-ar	1	ONLY_E-w/a-(	1	
	0	Ew/a-GOST-ar	1	ONLY_E-w/a-(	1	
E-ASA-art-Y	1	Ew/a-GOST-ar	1		0	E-ASA-cit-Y
E-ASA-art-Y	1	Ew/a-GOST-ar	1		0	E-ASA-cit-Y
E-ASA-art-Y	1	Ew/a-GOST-ar	1		0	E-ASA-cit-Y
E-ASA-art-Y	1	Ew/a-GOST-ar	1		0	E-ASA-cit-Y
	0		0		0	
	0	Ew/a-GOST-ar	1	ONLY_E-w/a-(	1	
	0		0		0	
	0		0		0	
	0		0		0	

	0	Ew/a-GOST-a	1	ONLY_E-w/a-	1	
	0	Ew/a-GOST-a	1	ONLY_E-w/a-	1	
	0		0		0	
	0	Ew/a-GOST-a	1	ONLY_E-w/a-	1	
	0	Ew/a-GOST-a	1	ONLY_E-w/a-	1	
	0		0		0	
	0	Ew/a-GOST-a	1	ONLY_E-w/a-	1	
	0	Ew/a-GOST-a	1	ONLY_E-w/a-	1	
	0	Ew/a-GOST-a	1	ONLY_E-w/a-	1	
	0	Ew/a-GOST-a	1	ONLY_E-w/a-	1	
	0		0		0	
	0		0		0	
	0		0		0	
	0		0		0	
E-ASA-art-Y	1		0		0	
	0		0		0	
	0	Ew/a-GOST-a	1	ONLY_E-w/a-	1	
	0	Ew/a-GOST-a	1	ONLY_E-w/a-	1	
	0	Ew/a-GOST-a	1	ONLY_E-w/a-	1	
	0		0		0	
	0		0		0	
E-ASA-art-Y	1	Ew/a-GOST-a	1		0	E-ASA-cit-Y
E-ASA-art-Y	1	Ew/a-GOST-a	1		0	E-ASA-cit-Y
E-ASA-art-Y	1	Ew/a-GOST-a	1		0	E-ASA-cit-Y
	0		0		0	
	0		0		0	
	0	Ew/a-GOST-a	1	ONLY_E-w/a-	1	
	0	Ew/a-GOST-a	1	ONLY_E-w/a-	1	
	0		0		0	
	0		0		0	
E-ASA-art-Y	1	Ew/a-GOST-a	1		0	E-ASA-cit-Y
E-ASA-art-Y	1	Ew/a-GOST-a	1		0	E-ASA-cit-Y
E-ASA-art-Y	1	Ew/a-GOST-a	1		0	E-ASA-cit-Y
	0		0		0	
	0		0		0	
	0		0		0	
	0		0		0	
	0		0		0	
	0	Ew/a-GOST-a	1	ONLY_E-w/a-	1	
	0	Ew/a-GOST-a	1	ONLY_E-w/a-	1	
	0		0		0	
	0		0		0	
	0		0		0	
	0		0		0	
	0		0		0	
	0	Ew/a-GOST-a	1	ONLY_E-w/a-	1	
	0		0		0	

	0	Ew/a-GOST-ar	1	ONLY_E-w/a-(	1
	0	Ew/a-GOST-ar	1	ONLY_E-w/a-(	1
	0		0		0
	0		0		0
	0	Ew/a-GOST-ar	1	ONLY_E-w/a-(	1
	0	Ew/a-GOST-ar	1	ONLY_E-w/a-(	1
	0	Ew/a-GOST-ar	1	ONLY_E-w/a-(	1
	0		0		0
	0		0		0
	0	Ew/a-GOST-ar	1	ONLY_E-w/a-(	1
	0	Ew/a-GOST-ar	1	ONLY_E-w/a-(	1
	0		0		0
	0		0		0
	0		0		0
	0		0		0
	0		0		0
	0		0		0
	0		0		0
	0		0		0
	0		0		0
	0		0		0
	0		0		0
E-ASA-art-Y	1		0		0
	0	Ew/a-GOST-ar	1	ONLY_E-w/a-(	1
	0	Ew/a-GOST-ar	1	ONLY_E-w/a-(	1
	0	Ew/a-GOST-ar	1	ONLY_E-w/a-(	1
	0	Ew/a-GOST-ar	1	ONLY_E-w/a-(	1
	0	Ew/a-GOST-ar	1	ONLY_E-w/a-(	1
	0	Ew/a-GOST-ar	1	ONLY_E-w/a-(	1
	0	Ew/a-GOST-ar	1	ONLY_E-w/a-(	1
	0		0		0
	0		0		0
	0		0		0
	0		0		0
	0		0		0
	0		0		0
	0		0		0
	0		0		0
	0		0		0
	0		0		0
	0		0		0
	0		0		0
	0		0		0
	0		0		0
	0		0		0
	0		0		0
	0		0		0

	0		0		0	
	0		0		0	
	0		0		0	
	0		0		0	
	0		0		0	
	0	Ew/a-GOST-ar	1	ONLY_E-w/a-(	1	
	0	Ew/a-GOST-ar	1	ONLY_E-w/a-(	1	
	0		0		0	
	0		0		0	
	0		0		0	
	0		0		0	
	0		0		0	
	0		0		0	
E-ASA-art-Y	1	Ew/a-GOST-ar	1		0	
E-ASA-art-Y	1	Ew/a-GOST-ar	1		0	
E-ASA-art-Y	1	Ew/a-GOST-ar	1		0	
	0		0		0	
	0	Ew/a-GOST-ar	1	ONLY_E-w/a-(	1	
	0		0		0	
	0	Ew/a-GOST-ar	1	ONLY_E-w/a-(	1	
	0		0		0	
	0		0		0	
	0		0		0	
	0		0		0	
	0	Ew/a-GOST-ar	1	ONLY_E-w/a-(	1	E-ASA-cit-Y
	0	Ew/a-GOST-ar	1	ONLY_E-w/a-(	1	E-ASA-cit-Y
	0		0		0	
	0		0		0	
	0		0		0	
	0		0		0	
	0		0		0	
	0		0		0	
	0		0		0	
	0		0		0	
	0		0		0	
	0		0		0	
	0		0		0	
	0		0		0	
	0		0		0	
	0		0		0	
	0	Ew/a-GOST-ar	1	ONLY_E-w/a-(	1	
	0		0		0	
	0		0		0	
E-ASA-art-Y	1		0		0	
	0		0		0	E-ASA-cit-Y
	0	Ew/a-GOST-ar	1	ONLY_E-w/a-(	1	
	0	Ew/a-GOST-ar	1	ONLY_E-w/a-(	1	

	0		0		0	
	0		0		0	
	0		0		0	
	0		0		0	
	0		0		0	
	0		0		0	
	0		0		0	
E-ASA-art-Y	1	Ew/a-GOST-ar	1		0	E-ASA-cit-Y
E-ASA-art-Y	1	Ew/a-GOST-ar	1		0	E-ASA-cit-Y
	0		0		0	
	0		0		0	
	0	Ew/a-GOST-ar	1	ONLY_E-w/a-	1	
	0	Ew/a-GOST-ar	1	ONLY_E-w/a-	1	
	0	Ew/a-GOST-ar	1	ONLY_E-w/a-	1	
	0		0		0	
	0		0		0	
	0		0		0	
	0		0		0	
	0		0		0	
	0		0		0	
	0		0		0	
	0	Ew/a-GOST-ar	1	ONLY_E-w/a-	1	
	0		0		0	
	0		0		0	
	0		0		0	
	0		0		0	
	0		0		0	
	0		0		0	
	0	Ew/a-GOST-ar	1	ONLY_E-w/a-	1	
	0	Ew/a-GOST-ar	1	ONLY_E-w/a-	1	
	0		0		0	
	0	Ew/a-GOST-ar	1	ONLY_E-w/a-	1	
	0	Ew/a-GOST-ar	1	ONLY_E-w/a-	1	
	0		0		0	
	0	Ew/a-GOST-ar	1	ONLY_E-w/a-	1	
	0		0		0	
	0		0		0	
E-ASA-art-Y	1		0		0	
	0		0		0	
	0		0		0	
	0		0		0	
	0		0		0	
	0		0		0	
	0		0		0	
	0		0		0	
	0		0		0	
	0		0		0	

	0		0		0	
	0	Ew/a-GOST-ar	1	ONLY_E-w/a-	1	
	0	Ew/a-GOST-ar	1	ONLY_E-w/a-	1	
	0	Ew/a-GOST-ar	1	ONLY_E-w/a-	1	
	0	Ew/a-GOST-ar	1	ONLY_E-w/a-	1	
E-ASA-art-Y	1		0		0	
E-ASA-art-Y	1		0		0	
	0		0		0	
	0	Ew/a-GOST-ar	1	ONLY_E-w/a-	1	
	0		0		0	
	0		0		0	
E-ASA-art-Y	1		0		0	E-ASA-cit-Y
E-ASA-art-Y	1		0		0	E-ASA-cit-Y
	0		0		0	
	0		0		0	
	0		0		0	
	0		0		0	
	0		0		0	
	0		0		0	
	0		0		0	E-ASA-cit-Y
	0		0		0	E-ASA-cit-Y
	0		0		0	
	0		0		0	
	0	Ew/a-GOST-ar	1	ONLY_E-w/a-	1	
	0		0		0	
	0		0		0	
	0		0		0	
	0		0		0	
	0		0		0	
	0		0		0	E-ASA-cit-Y
	0		0		0	E-ASA-cit-Y
	0		0		0	
	0		0		0	
	0		0		0	
	0		0		0	
	0		0		0	
	0		0		0	
	0		0		0	
	0		0		0	
	0		0		0	
	0		0		0	
	0		0		0	
	0		0		0	
	0		0		0	E-ASA-cit-Y
	0	Ew/a-GOST-ar	1	ONLY_E-w/a-	1	
	0	Ew/a-GOST-ar	1	ONLY_E-w/a-	1	
	0		0		0	

E-ASA-art-Y	1		0		0	
E-ASA-art-Y	1		0		0	
	0		0		0	
	0		0		0	
	0		0		0	
	0		0		0	
E-ASA-art-Y	1		0		0	
	0		0		0	
	0		0		0	
E-ASA-art-Y	1		0		0	
	0	Ew/a-GOST-a	1	ONLY_E-w/a-	1	
	0	Ew/a-GOST-a	1	ONLY_E-w/a-	1	
	0	Ew/a-GOST-a	1	ONLY_E-w/a-	1	
	0		0		0	
	0		0		0	
	0		0		0	
	0		0		0	
	0		0		0	
	0		0		0	
	0		0		0	
	0	Ew/a-GOST-a	1	ONLY_E-w/a-	1	
	0	Ew/a-GOST-a	1	ONLY_E-w/a-	1	
	0		0		0	
E-ASA-art-Y	1		0		0	E-ASA-cit-Y
E-ASA-art-Y	1		0		0	E-ASA-cit-Y
E-ASA-art-Y	1		0		0	E-ASA-cit-Y
	0		0		0	
	0		0		0	
	0		0		0	
	0		0		0	
	0		0		0	
	0		0		0	
	0		0		0	
	0		0		0	
	0	Ew/a-GOST-a	1	ONLY_E-w/a-	1	
	0		0		0	
	0		0		0	
	0		0		0	
	0		0		0	
	0		0		0	
	0	Ew/a-GOST-a	1	ONLY_E-w/a-	1	
E-ASA-art-Y	1		0		0	
	0		0		0	
E-ASA-art-Y	1	Ew/a-GOST-a	1		0	
	0		0		0	
	0	Ew/a-GOST-a	1	ONLY_E-w/a-	1	
	0	Ew/a-GOST-a	1	ONLY_E-w/a-	1	

	0		0		0	
	0		0		0	
	0		0		0	
	0		0		0	
	0		0		0	
	0		0		0	
	0		0		0	
	0	Ew/a-GOST-ar	1	ONLY_E-w/a-(	1	
	0	Ew/a-GOST-ar	1	ONLY_E-w/a-(	1	
	0		0		0	
E-ASA-art-Y	1		0		0	E-ASA-cit-Y
E-ASA-art-Y	1		0		0	E-ASA-cit-Y
E-ASA-art-Y	1		0		0	E-ASA-cit-Y
	0		0		0	
	0		0		0	
	0		0		0	
	0		0		0	
	0		0		0	
	0		0		0	
	0		0		0	
	0	Ew/a-GOST-ar	1	ONLY_E-w/a-(	1	
	0	Ew/a-GOST-ar	1	ONLY_E-w/a-(	1	
	0	Ew/a-GOST-ar	1	ONLY_E-w/a-(	1	
	0	Ew/a-GOST-ar	1	ONLY_E-w/a-(	1	
	0		0		0	
	0		0		0	
	0	Ew/a-GOST-ar	1	ONLY_E-w/a-(	1	
	0		0		0	
	0		0		0	
	0		0		0	
E-ASA-art-Y	1	Ew/a-GOST-ar	1		0	
E-ASA-art-Y	1	Ew/a-GOST-ar	1		0	
E-ASA-art-Y	1	Ew/a-GOST-ar	1		0	
E-ASA-art-Y	1	Ew/a-GOST-ar	1		0	
	0		0		0	
	0		0		0	
	0		0		0	
	0	Ew/a-GOST-ar	1	ONLY_E-w/a-(	1	
	0	Ew/a-GOST-ar	1	ONLY_E-w/a-(	1	
	0		0		0	
	0		0		0	
	0		0		0	
	0	Ew/a-GOST-ar	1	ONLY_E-w/a-(	1	
	0		0		0	
	0	Ew/a-GOST-ar	1	ONLY_E-w/a-(	1	
	0	Ew/a-GOST-ar	1	ONLY_E-w/a-(	1	
	0		0		0	

	0	Ew/a-GOST-a	1	ONLY_E-w/a-	1
	0	Ew/a-GOST-a	1	ONLY_E-w/a-	1
	0		0		0
	0		0		0
E-ASA-art-Y	1	Ew/a-GOST-a	1		0
E-ASA-art-Y	1	Ew/a-GOST-a	1		0
E-ASA-art-Y	1	Ew/a-GOST-a	1		0
	0		0		0
	0		0		0
	0		0		0
	0		0		0
	0		0		0
	0	Ew/a-GOST-a	1	ONLY_E-w/a-	1
	0		0		0
	0		0		0
	0	Ew/a-GOST-a	1	ONLY_E-w/a-	1
	0	Ew/a-GOST-a	1	ONLY_E-w/a-	1
	0		0		0
	0		0		0
	0		0		0
	0		0		0
	0		0		0
	0		0		0
	0		0		0
	0		0		0
	0	Ew/a-GOST-a	1	ONLY_E-w/a-	1
	0		0		0
	0		0		0
	0		0		0
E-ASA-art-Y	1	Ew/a-GOST-a	1		0
	0		0		0
	0		0		0
	0		0		0
	0		0		0
	0		0		0
	0		0		0
	0		0		0
	0	Ew/a-GOST-a	1	ONLY_E-w/a-	1
	0	Ew/a-GOST-a	1	ONLY_E-w/a-	1
	0		0		0
	0	Ew/a-GOST-a	1	ONLY_E-w/a-	1
	0		0		0
	0		0		0
	0		0		0
	0		0		0
	0		0		0
	0		0		0

	0		0		0	
	0		0		0	
E-ASA-art-Y	1	Ew/a-GOST-a	1		0	
E-ASA-art-Y	1	Ew/a-GOST-a	1		0	
	0		0		0	
	0		0		0	
E-ASA-art-Y	1	Ew/a-GOST-a	1		0	E-ASA-cit-Y
E-ASA-art-Y	1	Ew/a-GOST-a	1		0	E-ASA-cit-Y
E-ASA-art-Y	1	Ew/a-GOST-a	1		0	E-ASA-cit-Y
E-ASA-art-Y	1	Ew/a-GOST-a	1		0	E-ASA-cit-Y
	0		0		0	
	0		0		0	
	0		0		0	
	0		0		0	
	0		0		0	
	0		0		0	
	0		0		0	
E-ASA-art-Y	1		0		0	
E-ASA-art-Y	1		0		0	
	0		0		0	E-ASA-cit-Y
	0		0		0	E-ASA-cit-Y
	0	Ew/a-GOST-a	1	ONLY_E-w/a-	1	
	0	Ew/a-GOST-a	1	ONLY_E-w/a-	1	
	0	Ew/a-GOST-a	1	ONLY_E-w/a-	1	
	0	Ew/a-GOST-a	1	ONLY_E-w/a-	1	
	0	Ew/a-GOST-a	1	ONLY_E-w/a-	1	
	0		0		0	
	0		0		0	
	0		0		0	
	0		0		0	
	0		0		0	
	0	Ew/a-GOST-a	1	ONLY_E-w/a-	1	
	0	Ew/a-GOST-a	1	ONLY_E-w/a-	1	
	0		0		0	
	0		0		0	
	0		0		0	
	0		0		0	E-ASA-cit-Y
	0		0		0	E-ASA-cit-Y
	0	Ew/a-GOST-a	1	ONLY_E-w/a-	1	
	0	Ew/a-GOST-a	1	ONLY_E-w/a-	1	
	0	Ew/a-GOST-a	1	ONLY_E-w/a-	1	
	0		0		0	
	0	Ew/a-GOST-a	1	ONLY_E-w/a-	1	
	0		0		0	
	0		0		0	
	0		0		0	
	0		0		0	

	0		0		0	
	0		0		0	
E-ASA-art-Y	1		0		0	
E-ASA-art-Y	1		0		0	
	0		0		0	
	0		0		0	
	0		0		0	
	0	Ew/a-GOST-ar	1	ONLY_E-w/a-G	1	
E-ASA-art-Y	1		0		0	
E-ASA-art-Y	1		0		0	
	0		0		0	
	0		0		0	
	0		0		0	
	0		0		0	
	0	Ew/a-GOST-ar	1	ONLY_E-w/a-G	1	
	0		0		0	
	0		0		0	
	0		0		0	
	0		0		0	
	0	Ew/a-GOST-ar	1	ONLY_E-w/a-G	1	E-ASA-cit-Y
	0		0		0	E-ASA-cit-Y
	0		0		0	E-ASA-cit-Y
	0		0		0	
	0		0		0	
	0		0		0	
	0		0		0	
	0		0		0	
	0	Ew/a-GOST-ar	1	ONLY_E-w/a-G	1	
	0		0		0	
	0		0		0	
E-ASA-art-Y	1		0		0	E-ASA-cit-Y
E-ASA-art-Y	1		0		0	E-ASA-cit-Y
	0		0		0	
	0	Ew/a-GOST-ar	1	ONLY_E-w/a-G	1	
	0	Ew/a-GOST-ar	1	ONLY_E-w/a-G	1	
	0	Ew/a-GOST-ar	1	ONLY_E-w/a-G	1	
	0	Ew/a-GOST-ar	1	ONLY_E-w/a-G	1	
	0		0		0	
	0		0		0	
	0		0		0	
	0		0		0	
	0		0		0	
E-ASA-art-Y	1		0		0	
E-ASA-art-Y	1		0		0	
	0		0		0	
	0		0		0	
	0		0		0	

	0		0		0	
	0		0		0	
	0		0		0	
	0		0		0	
	0	Ew/a-GOST-a	1	ONLY_E-w/a-	1	
	0		0		0	
	0		0		0	
	0		0		0	
	0	Ew/a-GOST-a	1	ONLY_E-w/a-	1	E-ASA-cit-Y
	0		0		0	
E-ASA-art-Y	1	Ew/a-GOST-a	1		0	
E-ASA-art-Y	1	Ew/a-GOST-a	1		0	
E-ASA-art-Y	1	Ew/a-GOST-a	1		0	
	0		0		0	
	0		0		0	
	0	Ew/a-GOST-a	1	ONLY_E-w/a-	1	
	0		0		0	
	0		0		0	
	0		0		0	
	0	Ew/a-GOST-a	1	ONLY_E-w/a-	1	
	0	Ew/a-GOST-a	1	ONLY_E-w/a-	1	
E-ASA-art-Y	1	Ew/a-GOST-a	1		0	E-ASA-cit-Y
E-ASA-art-Y	1	Ew/a-GOST-a	1		0	E-ASA-cit-Y
	0		0		0	
	0	Ew/a-GOST-a	1	ONLY_E-w/a-	1	
	0		0		0	
	0		0		0	
	0		0		0	
	0	Ew/a-GOST-a	1	ONLY_E-w/a-	1	
E-ASA-art-Y	1	Ew/a-GOST-a	1		0	
E-ASA-art-Y	1	Ew/a-GOST-a	1		0	
	0	Ew/a-GOST-a	1	ONLY_E-w/a-	1	
	0	Ew/a-GOST-a	1	ONLY_E-w/a-	1	
	0		0		0	
	0		0		0	
E-ASA-art-Y	1		0		0	
	0		0		0	
E-ASA-art-Y	1		0		0	
	0		0		0	E-ASA-cit-Y
	0		0		0	E-ASA-cit-Y
	0		0		0	
	0	Ew/a-GOST-a	1	ONLY_E-w/a-	1	
	0		0		0	
	0		0		0	
	0		0		0	
	0		0		0	
	0		0		0	

E-ASA-art-Y	1		0		0
	0		0		0
	0		0		0
	0		0		0
E-ASA-art-Y	1		0		0
E-ASA-art-Y	1		0		0
E-ASA-art-Y	1		0		0
E-ASA-art-Y	1		0		0
E-ASA-art-Y	1		0		0
	0		0		0
	0		0		0
	0		0		0
	0		0		0
	0		0		0
	0		0		0
	0		0		0
	0		0		0
	0	Ew/a-GOST-ar	1	ONLY_E-w/a-(	1
	0	Ew/a-GOST-ar	1	ONLY_E-w/a-(	1
	0		0		0
	0		0		0
	0		0		0
	0		0		0
	0		0		0
	0		0		0
	0		0		0
	0		0		0
	0		0		0
	0		0		0
	0		0		0
	0		0		0
	0		0		0
	0		0		0
	0		0		0
	0		0		0
	0		0		0
	0		0		0
	0		0		0
	0		0		0
	0		0		0
	0		0		0
	0		0		0
	0		0		0
	0		0		0
	0	Ew/a-GOST-ar	1	ONLY_E-w/a-(	1
	0		0		0

	0		0		0	
	0		0		0	
	0		0		0	
	0	Ew/a-GOST-ar	1	ONLY_E-w/a-	1	
	0		0		0	
	0		0		0	E-ASA-cit-Y
	0	Ew/a-GOST-ar	1	ONLY_E-w/a-	1	E-ASA-cit-Y
	0	Ew/a-GOST-ar	1	ONLY_E-w/a-	1	E-ASA-cit-Y
	0		0		0	
	0		0		0	
	0		0		0	
	0		0		0	
	0		0		0	
	0		0		0	
	0	Ew/a-GOST-ar	1	ONLY_E-w/a-	1	
	0		0		0	
	0		0		0	
	0		0		0	
	0		0		0	
E-ASA-art-Y	1	Ew/a-GOST-ar	1		0	
E-ASA-art-Y	1	Ew/a-GOST-ar	1		0	
E-ASA-art-Y	1	Ew/a-GOST-ar	1		0	
E-ASA-art-Y	1	Ew/a-GOST-ar	1		0	
E-ASA-art-Y	1	Ew/a-GOST-ar	1		0	
	0		0		0	
	0	Ew/a-GOST-ar	1	ONLY_E-w/a-	1	
E-ASA-art-Y	1	Ew/a-GOST-ar	1		0	E-ASA-cit-Y
E-ASA-art-Y	1	Ew/a-GOST-ar	1		0	E-ASA-cit-Y
E-ASA-art-Y	1	Ew/a-GOST-ar	1		0	E-ASA-cit-Y
	0		0		0	
	0		0		0	
	0		0		0	
	0	Ew/a-GOST-ar	1	ONLY_E-w/a-	1	
	0		0		0	
	0		0		0	
	0		0		0	
E-ASA-art-Y	1	Ew/a-GOST-ar	1		0	E-ASA-cit-Y
E-ASA-art-Y	1	Ew/a-GOST-ar	1		0	E-ASA-cit-Y
	0	Ew/a-GOST-ar	1	ONLY_E-w/a-	1	
	0	Ew/a-GOST-ar	1	ONLY_E-w/a-	1	
	0	Ew/a-GOST-ar	1	ONLY_E-w/a-	1	
	0	Ew/a-GOST-ar	1	ONLY_E-w/a-	1	
	0	Ew/a-GOST-ar	1	ONLY_E-w/a-	1	
	0		0		0	
	0		0		0	
	0		0		0	
	0		0		0	

	0	Ew/a-GOST-ar	1	ONLY_E-w/a-	1	
	0	Ew/a-GOST-ar	1	ONLY_E-w/a-	1	
	0		0		0	
	0		0		0	
	0		0		0	
	0		0		0	
	0		0		0	
	0		0		0	
	0		0		0	
	0		0		0	
E-ASA-art-Y	1		0		0	E-ASA-cit-Y
	0	Ew/a-GOST-ar	1	ONLY_E-w/a-	1	
	0	Ew/a-GOST-ar	1	ONLY_E-w/a-	1	
	0	Ew/a-GOST-ar	1	ONLY_E-w/a-	1	
	0		0		0	
	0		0		0	
	0		0		0	
	0		0		0	
	0		0		0	
	0		0		0	
	0	Ew/a-GOST-ar	1	ONLY_E-w/a-	1	
	0	Ew/a-GOST-ar	1	ONLY_E-w/a-	1	
	0	Ew/a-GOST-ar	1	ONLY_E-w/a-	1	
	0	Ew/a-GOST-ar	1	ONLY_E-w/a-	1	
E-ASA-art-Y	1		0		0	E-ASA-cit-Y
	0		0		0	
	0		0		0	
	0		0		0	
	0		0		0	
	0		0		0	
	0		0		0	
	0		0		0	
	0	Ew/a-GOST-ar	1	ONLY_E-w/a-	1	
	0	Ew/a-GOST-ar	1	ONLY_E-w/a-	1	
E-ASA-art-Y	1	Ew/a-GOST-ar	1		0	
	0		0		0	
	0		0		0	
	0		0		0	
	0		0		0	
	0	Ew/a-GOST-ar	1	ONLY_E-w/a-	1	
	0	Ew/a-GOST-ar	1	ONLY_E-w/a-	1	
	0		0		0	
	0		0		0	
E-ASA-art-Y	1		0		0	
E-ASA-art-Y	1		0		0	
	0		0		0	
	0		0		0	

	0		0		0	
	0		0		0	
	0		0		0	
	0		0		0	
	0		0		0	
	0	Ew/a-GOST-a	1	ONLY_E-w/a-	1	E-ASA-cit-Y
	0		0		0	
	0		0		0	
	0		0		0	
	0		0		0	
	0		0		0	
	0	Ew/a-GOST-a	1	ONLY_E-w/a-	1	
	0	Ew/a-GOST-a	1	ONLY_E-w/a-	1	
	0	Ew/a-GOST-a	1	ONLY_E-w/a-	1	
	0		0		0	
	0		0		0	
	0		0		0	
	0	Ew/a-GOST-a	1	ONLY_E-w/a-	1	
	0		0		0	
	0		0		0	
	0		0		0	
	0		0		0	
	0	Ew/a-GOST-a	1	ONLY_E-w/a-	1	
	0	Ew/a-GOST-a	1	ONLY_E-w/a-	1	
	0	Ew/a-GOST-a	1	ONLY_E-w/a-	1	
	0	Ew/a-GOST-a	1	ONLY_E-w/a-	1	
	0	Ew/a-GOST-a	1	ONLY_E-w/a-	1	
	0		0		0	
	0		0		0	
	0		0		0	
	0		0		0	
	0		0		0	
	0		0		0	
	0		0		0	
	0		0		0	
	0	Ew/a-GOST-a	1	ONLY_E-w/a-	1	
	0		0		0	
	0		0		0	
E-ASA-art-Y	1	Ew/a-GOST-a	1		0	E-ASA-cit-Y
E-ASA-art-Y	1	Ew/a-GOST-a	1		0	E-ASA-cit-Y
	0		0		0	
	0		0		0	
	0	Ew/a-GOST-a	1	ONLY_E-w/a-	1	
	0		0		0	
	0		0		0	
	0		0		0	
	0		0		0	

	0		0		0	
	0		0		0	
	0		0		0	
	0	Ew/a-GOST-a	1	ONLY_E-w/a-	1	
	0	Ew/a-GOST-a	1	ONLY_E-w/a-	1	
	0		0		0	
	0		0		0	
	0		0		0	
E-ASA-art-Y	1	Ew/a-GOST-a	1		0	
E-ASA-art-Y	1	Ew/a-GOST-a	1		0	
E-ASA-art-Y	1	Ew/a-GOST-a	1		0	
	0	Ew/a-GOST-a	1	ONLY_E-w/a-	1	
	0	Ew/a-GOST-a	1	ONLY_E-w/a-	1	
	0	Ew/a-GOST-a	1	ONLY_E-w/a-	1	
	0	Ew/a-GOST-a	1	ONLY_E-w/a-	1	
	0		0		0	
	0		0		0	
	0		0		0	
	0		0		0	
	0		0		0	
	0		0		0	
	0	Ew/a-GOST-a	1	ONLY_E-w/a-	1	
	0		0		0	
	0		0		0	
	0		0		0	
	0	Ew/a-GOST-a	1	ONLY_E-w/a-	1	
	0	Ew/a-GOST-a	1	ONLY_E-w/a-	1	
	0	Ew/a-GOST-a	1	ONLY_E-w/a-	1	
	0	Ew/a-GOST-a	1	ONLY_E-w/a-	1	
	0		0		0	
	0		0		0	
	0		0		0	
	0		0		0	
	0		0		0	
	0		0		0	
	0		0		0	
	0		0		0	
	0		0		0	
	0		0		0	
	0		0		0	
	0		0		0	
	0		0		0	
	0		0		0	
E-ASA-art-Y	1	Ew/a-GOST-a	1		0	E-ASA-cit-Y
E-ASA-art-Y	1	Ew/a-GOST-a	1		0	E-ASA-cit-Y
E-ASA-art-Y	1	Ew/a-GOST-a	1		0	E-ASA-cit-Y
	0		0		0	E-ASA-cit-Y

	0		0		0	E-ASA-cit-Y
	0		0		0	
	0		0		0	
	0		0		0	
	0		0		0	
	0		0		0	
	0		0		0	
	0	Ew/a-GOST-a	1	ONLY_E-w/a-	1	
	0	Ew/a-GOST-a	1	ONLY_E-w/a-	1	
	0		0		0	E-ASA-cit-Y
	0		0		0	
E-ASA-art-Y	1	Ew/a-GOST-a	1		0	
E-ASA-art-Y	1	Ew/a-GOST-a	1		0	
E-ASA-art-Y	1	Ew/a-GOST-a	1		0	
	0		0		0	
	0		0		0	
	0		0		0	
	0		0		0	
	0		0		0	
	0	Ew/a-GOST-a	1	ONLY_E-w/a-	1	
	0	Ew/a-GOST-a	1	ONLY_E-w/a-	1	
	0	Ew/a-GOST-a	1	ONLY_E-w/a-	1	
	0		0		0	
	0		0		0	
	0		0		0	E-ASA-cit-Y
	0		0		0	E-ASA-cit-Y
	0		0		0	E-ASA-cit-Y
	0		0		0	E-ASA-cit-Y
	0		0		0	
	0	Ew/a-GOST-a	1	ONLY_E-w/a-	1	
	0		0		0	
E-ASA-art-Y	1		0		0	
E-ASA-art-Y	1		0		0	
E-ASA-art-Y	1		0		0	
	0		0		0	
	0		0		0	
	0		0		0	
	0		0		0	
	0		0		0	
	0		0		0	
	0		0		0	
	0		0		0	
	0		0		0	
	0		0		0	
	0		0		0	
	0	Ew/a-GOST-a	1	ONLY_E-w/a-	1	
	0	Ew/a-GOST-a	1	ONLY_E-w/a-	1	

E-ASA-art-Y	1		0		0	E-ASA-cit-Y
	0		0		0	
	0		0		0	
	0		0		0	
	0		0		0	E-ASA-cit-Y
	0		0		0	E-ASA-cit-Y
	0		0		0	
	0	Ew/a-GOST-a	1	ONLY_E-w/a-	1	
	0	Ew/a-GOST-a	1	ONLY_E-w/a-	1	
	0		0		0	
	0		0		0	
	0		0		0	
	0	Ew/a-GOST-a	1	ONLY_E-w/a-	1	
	0	Ew/a-GOST-a	1	ONLY_E-w/a-	1	
	0	Ew/a-GOST-a	1	ONLY_E-w/a-	1	
	0		0		0	
	0		0		0	
	0		0		0	
	0	Ew/a-GOST-a	1	ONLY_E-w/a-	1	
	0	Ew/a-GOST-a	1	ONLY_E-w/a-	1	
	0	Ew/a-GOST-a	1	ONLY_E-w/a-	1	
	0		0		0	
	0		0		0	
	0		0		0	
	0		0		0	
	0	Ew/a-GOST-a	1	ONLY_E-w/a-	1	
	0		0		0	
	0		0		0	
	0		0		0	
	0		0		0	
	0	Ew/a-GOST-a	1	ONLY_E-w/a-	1	
	0		0		0	
	0		0		0	
	0		0		0	
	0	Ew/a-GOST-a	1	ONLY_E-w/a-	1	
	0	Ew/a-GOST-a	1	ONLY_E-w/a-	1	
	0		0		0	
	0		0		0	
	0		0		0	
	0	Ew/a-GOST-a	1	ONLY_E-w/a-	1	
	0		0		0	
	0		0		0	
	0		0		0	
	0		0		0	
	0		0		0	
	0		0		0	
	0	Ew/a-GOST-a	1	ONLY_E-w/a-	1	

E-ASA-art-Y	1		0		0	
	0		0		0	
	0		0		0	
	0		0		0	
	0	Ew/a-GOST-a	1	ONLY_E-w/a-	1	
	0		0		0	
	0		0		0	
E-ASA-art-Y	1	Ew/a-GOST-a	1		0	E-ASA-cit-Y
E-ASA-art-Y	1	Ew/a-GOST-a	1		0	E-ASA-cit-Y
	0		0		0	
	0		0		0	
	0		0		0	
	0		0		0	
	0		0		0	
	0		0		0	
E-ASA-art-Y	1		0		0	E-ASA-cit-Y
	0		0		0	
	0		0		0	
	0		0		0	
	0		0		0	
	0		0		0	
	0	Ew/a-GOST-a	1	ONLY_E-w/a-	1	
E-ASA-art-Y	1	Ew/a-GOST-a	1		0	E-ASA-cit-Y
E-ASA-art-Y	1	Ew/a-GOST-a	1		0	E-ASA-cit-Y
E-ASA-art-Y	1	Ew/a-GOST-a	1		0	E-ASA-cit-Y
	0		0		0	
	0	Ew/a-GOST-a	1	ONLY_E-w/a-	1	E-ASA-cit-Y
	0		0		0	
	0		0		0	E-ASA-cit-Y
	0		0		0	E-ASA-cit-Y
	0		0		0	
	0		0		0	E-ASA-cit-Y
	0	Ew/a-GOST-a	1	ONLY_E-w/a-	1	E-ASA-cit-Y
	0	Ew/a-GOST-a	1	ONLY_E-w/a-	1	E-ASA-cit-Y
	0	Ew/a-GOST-a	1	ONLY_E-w/a-	1	
	0	Ew/a-GOST-a	1	ONLY_E-w/a-	1	
	0		0		0	
	0		0		0	
	0		0		0	
E-ASA-art-Y	1	Ew/a-GOST-a	1		0	
E-ASA-art-Y	1	Ew/a-GOST-a	1		0	
	0	Ew/a-GOST-a	1	ONLY_E-w/a-	1	
	0	Ew/a-GOST-a	1	ONLY_E-w/a-	1	
	0		0		0	
	0		0		0	
	0		0		0	
	0		0		0	

E-ASA-art-Y	1	Ew/a-GOST-ar	1		0	
E-ASA-art-Y	1	Ew/a-GOST-ar	1		0	
E-ASA-art-Y	1	Ew/a-GOST-ar	1		0	
	0		0		0	
	0		0		0	
	0		0		0	E-ASA-cit-Y
	0		0		0	
	0		0		0	
	0	Ew/a-GOST-ar	1	ONLY_E-w/a-(	1	
E-ASA-art-Y	1		0		0	E-ASA-cit-Y
E-ASA-art-Y	1		0		0	E-ASA-cit-Y
E-ASA-art-Y	1		0		0	E-ASA-cit-Y
	0		0		0	
	0		0		0	
	0		0		0	
	0		0		0	
	0		0		0	
	0		0		0	
	0		0		0	
	0		0		0	
	0		0		0	
	0		0		0	
	0		0		0	
	0		0		0	
	0		0		0	
	0	Ew/a-GOST-ar	1	ONLY_E-w/a-(	1	
	0		0		0	
	0		0		0	
	0		0		0	E-ASA-cit-Y
	0		0		0	E-ASA-cit-Y
	0		0		0	
	0	Ew/a-GOST-ar	1	ONLY_E-w/a-(	1	E-ASA-cit-Y
E-ASA-art-Y	1		0		0	
E-ASA-art-Y	1		0		0	
	0		0		0	
E-ASA-art-Y	1		0		0	
E-ASA-art-Y	1		0		0	
	0		0		0	
	0		0		0	
	0		0		0	
	0		0		0	
	0		0		0	
	0		0		0	
	0		0		0	
	0		0		0	
	0		0		0	
	0		0		0	

	0		0		0	
	0		0		0	
	0		0		0	E-ASA-cit-Y
	0		0		0	E-ASA-cit-Y
	0		0		0	
	0		0		0	
	0		0		0	
	0		0		0	
	0		0		0	
	0		0		0	
	0		0		0	
	0		0		0	
	0		0		0	
	0		0		0	
	0		0		0	
E-ASA-art-Y	1		0		0	
	0	Ew/a-GOST-ar	1	ONLY_E-w/a-	1	E-ASA-cit-Y
	0	Ew/a-GOST-ar	1	ONLY_E-w/a-	1	E-ASA-cit-Y
	0		0		0	
	0		0		0	
	0		0		0	
E-ASA-art-Y	1		0		0	
	0		0		0	
	0		0		0	
	0		0		0	
	0		0		0	
	0		0		0	
	0		0		0	
	0		0		0	
	0		0		0	
	0		0		0	
	0		0		0	
E-ASA-art-Y	1	Ew/a-GOST-ar	1		0	E-ASA-cit-Y
	0		0		0	
E-ASA-art-Y	1		0		0	E-ASA-cit-Y
E-ASA-art-Y	1		0		0	E-ASA-cit-Y
E-ASA-art-Y	1		0		0	E-ASA-cit-Y
	0		0		0	
	0		0		0	
	0		0		0	
E-ASA-art-Y	1		0		0	E-ASA-cit-Y
E-ASA-art-Y	1		0		0	E-ASA-cit-Y
E-ASA-art-Y	1		0		0	E-ASA-cit-Y
E-ASA-art-Y	1		0		0	E-ASA-cit-Y
	0		0		0	
	0		0		0	E-ASA-cit-Y
	0		0		0	E-ASA-cit-Y

	0	Ew/a-GOST-ar	1	ONLY_E-w/a-(	1	
	0		0		0	
E-ASA-art-Y	1		0		0	E-ASA-cit-Y
	0		0		0	
	0		0		0	
E-ASA-art-Y	1		0		0	
E-ASA-art-Y	1		0		0	
	0	Ew/a-GOST-ar	1	ONLY_E-w/a-(	1	
	0	Ew/a-GOST-ar	1	ONLY_E-w/a-(	1	
	0		0		0	
	0	Ew/a-GOST-ar	1	ONLY_E-w/a-(	1	
	0	Ew/a-GOST-ar	1	ONLY_E-w/a-(	1	
	0	Ew/a-GOST-ar	1	ONLY_E-w/a-(	1	
	0		0		0	
	0		0		0	
	0	Ew/a-GOST-ar	1	ONLY_E-w/a-(	1	
	0	Ew/a-GOST-ar	1	ONLY_E-w/a-(	1	
	0	Ew/a-GOST-ar	1	ONLY_E-w/a-(	1	E-ASA-cit-Y
	0	Ew/a-GOST-ar	1	ONLY_E-w/a-(	1	E-ASA-cit-Y
	0	Ew/a-GOST-ar	1	ONLY_E-w/a-(	1	E-ASA-cit-Y
	0		0		0	
	0		0		0	
	0		0		0	
	0		0		0	
E-ASA-art-Y	1		0		0	E-ASA-cit-Y
	0	Ew/a-GOST-ar	1	ONLY_E-w/a-(	1	
	0		0		0	E-ASA-cit-Y
	0		0		0	
	0		0		0	
	0		0		0	
	0		0		0	
	0		0		0	
	0		0		0	
	0	Ew/a-GOST-ar	1	ONLY_E-w/a-(	1	
	0	Ew/a-GOST-ar	1	ONLY_E-w/a-(	1	
	0	Ew/a-GOST-ar	1	ONLY_E-w/a-(	1	
	0		0		0	
	0	Ew/a-GOST-ar	1	ONLY_E-w/a-(	1	
	0	Ew/a-GOST-ar	1	ONLY_E-w/a-(	1	
E-ASA-art-Y	1		0		0	E-ASA-cit-Y
	0		0		0	
E-ASA-art-Y	1		0		0	E-ASA-cit-Y
	0		0		0	
	0		0		0	
	0		0		0	
	0		0		0	
	0		0		0	

	0	Ew/a-GOST-ar	1	ONLY_E-w/a-(	1	
	0		0		0	
	0	Ew/a-GOST-ar	1	ONLY_E-w/a-(	1	
E-ASA-art-Y	1		0		0	E-ASA-cit-Y
	0		0		0	E-ASA-cit-Y
	0		0		0	E-ASA-cit-Y
	0		0		0	
	0		0		0	
	0		0		0	
	0		0		0	
	0	Ew/a-GOST-ar	1	ONLY_E-w/a-(	1	
	0	Ew/a-GOST-ar	1	ONLY_E-w/a-(	1	
	0		0		0	
	0		0		0	
	0		0		0	E-ASA-cit-Y
	0	Ew/a-GOST-ar	1	ONLY_E-w/a-(	1	
	0	Ew/a-GOST-ar	1	ONLY_E-w/a-(	1	
	0		0		0	
	0		0		0	
	0	Ew/a-GOST-ar	1	ONLY_E-w/a-(	1	
	0	Ew/a-GOST-ar	1	ONLY_E-w/a-(	1	
	0	Ew/a-GOST-ar	1	ONLY_E-w/a-(	1	
	0	Ew/a-GOST-ar	1	ONLY_E-w/a-(	1	
E-ASA-art-Y	1	Ew/a-GOST-ar	1		0	
	0		0		0	
	0	Ew/a-GOST-ar	1	ONLY_E-w/a-(	1	
E-ASA-art-Y	1		0		0	E-ASA-cit-Y
	0		0		0	
	0		0		0	
	0		0		0	
	0		0		0	
	0		0		0	
	0		0		0	
	0		0		0	
	0		0		0	
	0		0		0	
	0		0		0	
	0		0		0	
	0		0		0	
	0	Ew/a-GOST-ar	1	ONLY_E-w/a-(	1	
	0		0		0	
	0		0		0	
	0	Ew/a-GOST-ar	1	ONLY_E-w/a-(	1	
E-ASA-art-Y	1	Ew/a-GOST-ar	1		0	
	0		0		0	
	0		0		0	
	0		0		0	

	0		0		0	
	0		0		0	
	0		0		0	
	0		0		0	
	0		0		0	
	0		0		0	
	0		0		0	
	0		0		0	
	0	Ew/a-GOST-ar	1	ONLY_E-w/a-(	1	
	0	Ew/a-GOST-ar	1	ONLY_E-w/a-(	1	
	0		0		0	
	0		0		0	
	0		0		0	
	0		0		0	
	0		0		0	
	0		0		0	
	0	Ew/a-GOST-ar	1	ONLY_E-w/a-(	1	
E-ASA-art-Y	1		0		0	
	0		0		0	
	0		0		0	
	0	Ew/a-GOST-ar	1	ONLY_E-w/a-(	1	
	0	Ew/a-GOST-ar	1	ONLY_E-w/a-(	1	
	0		0		0	
E-ASA-art-Y	1		0		0	
E-ASA-art-Y	1		0		0	
	0		0		0	
	0	Ew/a-GOST-ar	1	ONLY_E-w/a-(	1	
	0		0		0	
E-ASA-art-Y	1		0		0	
	0		0		0	
	0		0		0	
	0		0		0	E-ASA-cit-Y
	0		0		0	E-ASA-cit-Y
	0		0		0	E-ASA-cit-Y
	0		0		0	
	0	Ew/a-GOST-ar	1	ONLY_E-w/a-(	1	
	0		0		0	
	0	Ew/a-GOST-ar	1	ONLY_E-w/a-(	1	
	0		0		0	
	0		0		0	
	0	Ew/a-GOST-ar	1	ONLY_E-w/a-(	1	
	0	Ew/a-GOST-ar	1	ONLY_E-w/a-(	1	
	0		0		0	
	0		0		0	
	0		0		0	
	0		0		0	
	0	Ew/a-GOST-ar	1	ONLY_E-w/a-(	1	E-ASA-cit-Y

	0	Ew/a-GOST-a	1	ONLY_E-w/a-	1	E-ASA-cit-Y
	0	Ew/a-GOST-a	1	ONLY_E-w/a-	1	E-ASA-cit-Y
	0	Ew/a-GOST-a	1	ONLY_E-w/a-	1	
	0	Ew/a-GOST-a	1	ONLY_E-w/a-	1	
	0	Ew/a-GOST-a	1	ONLY_E-w/a-	1	
	0	Ew/a-GOST-a	1	ONLY_E-w/a-	1	
	0	Ew/a-GOST-a	1	ONLY_E-w/a-	1	
	0	Ew/a-GOST-a	1	ONLY_E-w/a-	1	
	0		0		0	
	0		0		0	
	0	Ew/a-GOST-a	1	ONLY_E-w/a-	1	
	0	Ew/a-GOST-a	1	ONLY_E-w/a-	1	
	0	Ew/a-GOST-a	1	ONLY_E-w/a-	1	
	0		0		0	
	0		0		0	
	0		0		0	
	0		0		0	
	0		0		0	
	0		0		0	
	0		0		0	
	0		0		0	
E-ASA-art-Y	1	Ew/a-GOST-a	1		0	E-ASA-cit-Y
E-ASA-art-Y	1	Ew/a-GOST-a	1		0	E-ASA-cit-Y
	0		0		0	
	0	Ew/a-GOST-a	1	ONLY_E-w/a-	1	
	0	Ew/a-GOST-a	1	ONLY_E-w/a-	1	
E-ASA-art-Y	1	Ew/a-GOST-a	1		0	E-ASA-cit-Y
E-ASA-art-Y	1	Ew/a-GOST-a	1		0	E-ASA-cit-Y
	0	Ew/a-GOST-a	1	ONLY_E-w/a-	1	
	0	Ew/a-GOST-a	1	ONLY_E-w/a-	1	
	0	Ew/a-GOST-a	1	ONLY_E-w/a-	1	
	0	Ew/a-GOST-a	1	ONLY_E-w/a-	1	
E-ASA-art-Y	1	Ew/a-GOST-a	1		0	E-ASA-cit-Y
	0	Ew/a-GOST-a	1	ONLY_E-w/a-	1	
	0		0		0	
	0		0		0	
	0	Ew/a-GOST-a	1	ONLY_E-w/a-	1	
	0	Ew/a-GOST-a	1	ONLY_E-w/a-	1	
	0	Ew/a-GOST-a	1	ONLY_E-w/a-	1	
	0	Ew/a-GOST-a	1	ONLY_E-w/a-	1	
	0	Ew/a-GOST-a	1	ONLY_E-w/a-	1	
	0	Ew/a-GOST-a	1	ONLY_E-w/a-	1	
	0		0		0	
	0		0		0	
	0		0		0	
	0		0		0	
	0		0		0	

	0		0		0	
	0	Ew/a-GOST-ar	1	ONLY_E-w/a-	1	
	0	Ew/a-GOST-ar	1	ONLY_E-w/a-	1	
	0	Ew/a-GOST-ar	1	ONLY_E-w/a-	1	
	0		0		0	
	0	Ew/a-GOST-ar	1	ONLY_E-w/a-	1	
	0		0		0	
	0	Ew/a-GOST-ar	1	ONLY_E-w/a-	1	
	0	Ew/a-GOST-ar	1	ONLY_E-w/a-	1	
E-ASA-art-Y	1	Ew/a-GOST-ar	1		0	E-ASA-cit-Y
	0		0		0	
	0		0		0	
	0		0		0	
	0	Ew/a-GOST-ar	1	ONLY_E-w/a-	1	
	0	Ew/a-GOST-ar	1	ONLY_E-w/a-	1	
	0		0		0	
	0		0		0	
E-ASA-art-Y	1		0		0	
E-ASA-art-Y	1		0		0	
	0	Ew/a-GOST-ar	1	ONLY_E-w/a-	1	
	0	Ew/a-GOST-ar	1	ONLY_E-w/a-	1	
	0	Ew/a-GOST-ar	1	ONLY_E-w/a-	1	
	0	Ew/a-GOST-ar	1	ONLY_E-w/a-	1	
	0		0		0	
	0		0		0	
	0		0		0	
	0		0		0	
	0		0		0	
	0		0		0	
	0	Ew/a-GOST-ar	1	ONLY_E-w/a-	1	
	0	Ew/a-GOST-ar	1	ONLY_E-w/a-	1	
	0		0		0	
	0		0		0	
	0		0		0	
	0	Ew/a-GOST-ar	1	ONLY_E-w/a-	1	
	0		0		0	
	0		0		0	
	0		0		0	
	0		0		0	
E-ASA-art-Y	1		0		0	E-ASA-cit-Y
	0		0		0	
	0		0		0	
	0		0		0	
	0		0		0	
	0		0		0	
	0		0		0	
	0		0		0	

	0		0		0	
	0		0		0	
	0		0		0	
	0		0		0	
	0		0		0	
	0		0		0	
	0		0		0	
	0		0		0	
	0		0		0	
	0		0		0	
	0	Ew/a-GOST-ar	1	ONLY_E-w/a-	1	
	0	Ew/a-GOST-ar	1	ONLY_E-w/a-	1	
	0	Ew/a-GOST-ar	1	ONLY_E-w/a-	1	
	0	Ew/a-GOST-ar	1	ONLY_E-w/a-	1	
	0		0		0	
	0		0		0	
	0	Ew/a-GOST-ar	1	ONLY_E-w/a-	1	
	0	Ew/a-GOST-ar	1	ONLY_E-w/a-	1	
	0		0		0	
	0		0		0	
	0		0		0	
	0		0		0	
	0		0		0	
	0		0		0	
	0		0		0	
	0		0		0	
	0		0		0	
	0		0		0	
	0		0		0	E-ASA-cit-Y
	0		0		0	E-ASA-cit-Y
	0		0		0	E-ASA-cit-Y
	0		0		0	E-ASA-cit-Y
	0		0		0	
	0		0		0	
	0		0		0	
	0	Ew/a-GOST-ar	1	ONLY_E-w/a-	1	
	0	Ew/a-GOST-ar	1	ONLY_E-w/a-	1	
	0		0		0	E-ASA-cit-Y
	0		0		0	
	0		0		0	
	0		0		0	
E-ASA-art-Y	1	Ew/a-GOST-ar	1		0	E-ASA-cit-Y
E-ASA-art-Y	1	Ew/a-GOST-ar	1		0	E-ASA-cit-Y
E-ASA-art-Y	1	Ew/a-GOST-ar	1		0	E-ASA-cit-Y
E-ASA-art-Y	1		0		0	E-ASA-cit-Y
E-ASA-art-Y	1		0		0	E-ASA-cit-Y
	0		0		0	

	0		0		0	
	0	Ew/a-GOST-ar	1	ONLY_E-w/a-(	1	
	0		0		0	
	0		0		0	
	0		0		0	
	0		0		0	
	0		0		0	
	0		0		0	
	0		0		0	
E-ASA-art-Y	1	Ew/a-GOST-ar	1		0	
	0	Ew/a-GOST-ar	1	ONLY_E-w/a-(	1	E-ASA-cit-Y
	0	Ew/a-GOST-ar	1	ONLY_E-w/a-(	1	E-ASA-cit-Y
	0		0		0	
	0		0		0	
	0		0		0	
	0		0		0	
	0	Ew/a-GOST-ar	1	ONLY_E-w/a-(	1	E-ASA-cit-Y
	0	Ew/a-GOST-ar	1	ONLY_E-w/a-(	1	E-ASA-cit-Y
	0		0		0	
	0		0		0	
	0	Ew/a-GOST-ar	1	ONLY_E-w/a-(	1	
	0	Ew/a-GOST-ar	1	ONLY_E-w/a-(	1	
	0	Ew/a-GOST-ar	1	ONLY_E-w/a-(	1	
E-ASA-art-Y	1		0		0	
E-ASA-art-Y	1		0		0	
E-ASA-art-Y	1		0		0	
	0		0		0	
	0		0		0	
	0		0		0	
E-ASA-art-Y	1	Ew/a-GOST-ar	1		0	E-ASA-cit-Y
E-ASA-art-Y	1	Ew/a-GOST-ar	1		0	E-ASA-cit-Y
	0		0		0	
	0		0		0	
	0		0		0	
	0		0		0	
	0		0		0	
	0	Ew/a-GOST-ar	1	ONLY_E-w/a-(	1	
	0		0		0	
	0		0		0	E-ASA-cit-Y
	0		0		0	E-ASA-cit-Y
	0		0		0	
	0		0		0	
	0		0		0	
	0		0		0	
	0		0		0	
	0		0		0	
	0		0		0	

	0		0		0	
	0		0		0	
	0	Ew/a-GOST-ar	1	ONLY_E-w/a-	1	
E-ASA-art-Y	1		0		0	E-ASA-cit-Y
E-ASA-art-Y	1		0		0	E-ASA-cit-Y
E-ASA-art-Y	1		0		0	E-ASA-cit-Y
	0		0		0	
	0		0		0	
	0	Ew/a-GOST-ar	1	ONLY_E-w/a-	1	
	0		0		0	
	0		0		0	
	0		0		0	
	0		0		0	
	0		0		0	
	0		0		0	
	0		0		0	
	0		0		0	
	0		0		0	
	0		0		0	
E-ASA-art-Y	1	Ew/a-GOST-ar	1		0	E-ASA-cit-Y
	0		0		0	
	0		0		0	
	0		0		0	
	0		0		0	
	0		0		0	
	0		0		0	
	0	Ew/a-GOST-ar	1	ONLY_E-w/a-	1	
	0	Ew/a-GOST-ar	1	ONLY_E-w/a-	1	
	0	Ew/a-GOST-ar	1	ONLY_E-w/a-	1	
E-ASA-art-Y	1	Ew/a-GOST-ar	1		0	
E-ASA-art-Y	1	Ew/a-GOST-ar	1		0	
E-ASA-art-Y	1	Ew/a-GOST-ar	1		0	
E-ASA-art-Y	1	Ew/a-GOST-ar	1		0	
	0		0		0	
	0	Ew/a-GOST-ar	1	ONLY_E-w/a-	1	
	0	Ew/a-GOST-ar	1	ONLY_E-w/a-	1	
	0	Ew/a-GOST-ar	1	ONLY_E-w/a-	1	
	0		0		0	
	0		0		0	
E-ASA-art-Y	1	Ew/a-GOST-ar	1		0	
	0		0		0	
	0		0		0	
E-ASA-art-Y	1	Ew/a-GOST-ar	1		0	
E-ASA-art-Y	1	Ew/a-GOST-ar	1		0	
E-ASA-art-Y	1	Ew/a-GOST-ar	1		0	
	0		0		0	
	0		0		0	

	0		0		0	
	0		0		0	
	0	Ew/a-GOST-a	1	ONLY_E-w/a-	1	
	0		0		0	E-ASA-cit-Y
	0		0		0	E-ASA-cit-Y
E-ASA-art-Y	1	Ew/a-GOST-a	1		0	E-ASA-cit-Y
E-ASA-art-Y	1	Ew/a-GOST-a	1		0	E-ASA-cit-Y
	0		0		0	
	0		0		0	
	0		0		0	
	0		0		0	
	0		0		0	
	0		0		0	
	0		0		0	
	0		0		0	
	0		0		0	
	0		0		0	
	0		0		0	
	0	Ew/a-GOST-a	1	ONLY_E-w/a-	1	
	0	Ew/a-GOST-a	1	ONLY_E-w/a-	1	
	0		0		0	
	0		0		0	
	0		0		0	
	0		0		0	
	0	Ew/a-GOST-a	1	ONLY_E-w/a-	1	
	0		0		0	
	0		0		0	
	0		0		0	E-ASA-cit-Y
	0		0		0	
	0	Ew/a-GOST-a	1	ONLY_E-w/a-	1	
	0		0		0	
	0		0		0	
	0		0		0	
	0	Ew/a-GOST-a	1	ONLY_E-w/a-	1	
	0		0		0	
	0		0		0	
	0		0		0	
E-ASA-art-Y	1	Ew/a-GOST-a	1		0	E-ASA-cit-Y
E-ASA-art-Y	1	Ew/a-GOST-a	1		0	E-ASA-cit-Y
E-ASA-art-Y	1	Ew/a-GOST-a	1		0	E-ASA-cit-Y
	0		0		0	
	0		0		0	
	0		0		0	
	0	Ew/a-GOST-a	1	ONLY_E-w/a-	1	
	0	Ew/a-GOST-a	1	ONLY_E-w/a-	1	
	0	Ew/a-GOST-a	1	ONLY_E-w/a-	1	
	0		0		0	

	0		0		0	
	0	Ew/a-GOST-a	1	ONLY_E-w/a-	1	
	0		0		0	
	0		0		0	
	0	Ew/a-GOST-a	1	ONLY_E-w/a-	1	
	0	Ew/a-GOST-a	1	ONLY_E-w/a-	1	
	0		0		0	
	0		0		0	
	0	Ew/a-GOST-a	1	ONLY_E-w/a-	1	
	0	Ew/a-GOST-a	1	ONLY_E-w/a-	1	
	0	Ew/a-GOST-a	1	ONLY_E-w/a-	1	
	0	Ew/a-GOST-a	1	ONLY_E-w/a-	1	
E-ASA-art-Y	1		0		0	E-ASA-cit-Y
E-ASA-art-Y	1		0		0	E-ASA-cit-Y
E-ASA-art-Y	1		0		0	E-ASA-cit-Y
	0		0		0	
E-ASA-art-Y	1	Ew/a-GOST-a	1		0	E-ASA-cit-Y
E-ASA-art-Y	1	Ew/a-GOST-a	1		0	E-ASA-cit-Y
E-ASA-art-Y	1	Ew/a-GOST-a	1		0	E-ASA-cit-Y
	0		0		0	
	0		0		0	
	0		0		0	
	0		0		0	
	0		0		0	
	0		0		0	
	0		0		0	
	0		0		0	
E-ASA-art-Y	1		0		0	
E-ASA-art-Y	1		0		0	
E-ASA-art-Y	1		0		0	
	0	Ew/a-GOST-a	1	ONLY_E-w/a-	1	
	0		0		0	
	0		0		0	
	0		0		0	E-ASA-cit-Y
	0		0		0	E-ASA-cit-Y
	0		0		0	E-ASA-cit-Y
	0		0		0	E-ASA-cit-Y
	0		0		0	
E-ASA-art-Y	1		0		0	
E-ASA-art-Y	1		0		0	
	0		0		0	E-ASA-cit-Y
	0		0		0	E-ASA-cit-Y
	0	Ew/a-GOST-a	1	ONLY_E-w/a-	1	
	0		0		0	
	0		0		0	
	0		0		0	
	0		0		0	

	0		0		0		
	0		0		0		
	0		0		0		
	0	Ew/a-GOST-ar	1	ONLY_E-w/a-(	1		
	0	Ew/a-GOST-ar	1	ONLY_E-w/a-(	1		
	0	Ew/a-GOST-ar	1	ONLY_E-w/a-(	1		
	0	Ew/a-GOST-ar	1	ONLY_E-w/a-(	1		
	0	Ew/a-GOST-ar	1	ONLY_E-w/a-(	1		
	0		0		0		
	0		0		0		
	0		0		0		
E-ASA-art-Y	1	Ew/a-GOST-ar	1		0		
	0		0		0		
	0		0		0		
	0		0		0		
	0		0		0		
	0		0		0		
	0		0		0		
	0		0		0		
	0		0		0		
	0		0		0	E-ASA-cit-Y	
	0		0		0	E-ASA-cit-Y	
	0	Ew/a-GOST-ar	1	ONLY_E-w/a-(	1		
	0	Ew/a-GOST-ar	1	ONLY_E-w/a-(	1		
	0		0		0		
	0		0		0		
	0		0		0		
	0		0		0	E-ASA-cit-Y	
	0		0		0		
	0		0		0		
	0		0		0		
	0		0		0		
	0		0		0		
	0		0		0		
	0		0		0		
	0		0		0		
	0		0		0		
	0		0		0		
	0		0		0		
	0	Ew/a-GOST-ar	1	ONLY_E-w/a-(	1		
	0	Ew/a-GOST-ar	1	ONLY_E-w/a-(	1		
	0		0		0		
	0		0		0		
	0		0		0		
E-ASA-art-Y	1		0		0		
E-ASA-art-Y	1		0		0	E-ASA-cit-Y	
E-ASA-art-Y	1		0		0	E-ASA-cit-Y	

E-ASA-art-Y	1		0		0	E-ASA-cit-Y
	0		0		0	
	0		0		0	
	0		0		0	
	0		0		0	
	0		0		0	
	0		0		0	
	0		0		0	
	0		0		0	
	0		0		0	
	0		0		0	
E-ASA-art-Y	1		0		0	
	0		0		0	
	0	Ew/a-GOST-a	1	ONLY_E-w/a-	1	E-ASA-cit-Y
	0	Ew/a-GOST-a	1	ONLY_E-w/a-	1	E-ASA-cit-Y
	0		0		0	E-ASA-cit-Y
	0		0		0	E-ASA-cit-Y
	0		0		0	
	0		0		0	
	0	Ew/a-GOST-a	1	ONLY_E-w/a-	1	
	0	Ew/a-GOST-a	1	ONLY_E-w/a-	1	
	0		0		0	E-ASA-cit-Y
	0		0		0	
	0		0		0	
	0		0		0	
	0	Ew/a-GOST-a	1	ONLY_E-w/a-	1	E-ASA-cit-Y
	0	Ew/a-GOST-a	1	ONLY_E-w/a-	1	E-ASA-cit-Y
	0		0		0	
	0		0		0	
	0	Ew/a-GOST-a	1	ONLY_E-w/a-	1	
E-ASA-art-Y	1		0		0	
E-ASA-art-Y	1		0		0	
E-ASA-art-Y	1	Ew/a-GOST-a	1		0	E-ASA-cit-Y
	0		0		0	
	0		0		0	
	0	Ew/a-GOST-a	1	ONLY_E-w/a-	1	
	0	Ew/a-GOST-a	1	ONLY_E-w/a-	1	
	0		0		0	
	0		0		0	
	0	Ew/a-GOST-a	1	ONLY_E-w/a-	1	E-ASA-cit-Y
	0		0		0	
	0		0		0	
	0		0		0	
	0		0		0	
	0	Ew/a-GOST-a	1	ONLY_E-w/a-	1	
	0		0		0	
	0		0		0	

E-ASA-art-Y	1	Ew/a-GOST-a	1		0	E-ASA-cit-Y
E-ASA-art-Y	1	Ew/a-GOST-a	1		0	E-ASA-cit-Y
	0		0		0	
	0		0		0	
	0		0		0	
	0		0		0	
	0		0		0	
	0		0		0	
	0	Ew/a-GOST-a	1	ONLY_E-w/a-	1	
	0	Ew/a-GOST-a	1	ONLY_E-w/a-	1	
	0		0		0	
	0		0		0	
	0		0		0	
	0		0		0	
	0	Ew/a-GOST-a	1	ONLY_E-w/a-	1	E-ASA-cit-Y
	0		0		0	
	0		0		0	
	0	Ew/a-GOST-a	1	ONLY_E-w/a-	1	
	0	Ew/a-GOST-a	1	ONLY_E-w/a-	1	
	0	Ew/a-GOST-a	1	ONLY_E-w/a-	1	
	0	Ew/a-GOST-a	1	ONLY_E-w/a-	1	
	0		0		0	
	0	Ew/a-GOST-a	1	ONLY_E-w/a-	1	
	0		0		0	
	0		0		0	E-ASA-cit-Y
	0		0		0	E-ASA-cit-Y
	0		0		0	
	0		0		0	
	0		0		0	
	0	Ew/a-GOST-a	1	ONLY_E-w/a-	1	
E-ASA-art-Y	1		0		0	
	0		0		0	
	0		0		0	
	0	Ew/a-GOST-a	1	ONLY_E-w/a-	1	
	0		0		0	
	0		0		0	
	0	Ew/a-GOST-a	1	ONLY_E-w/a-	1	
	0	Ew/a-GOST-a	1	ONLY_E-w/a-	1	
	0	Ew/a-GOST-a	1	ONLY_E-w/a-	1	
	0		0		0	
	0		0		0	
	0		0		0	
E-ASA-art-Y	1	Ew/a-GOST-a	1		0	
	0		0		0	
	0		0		0	
	0		0		0	
	0		0		0	

	0		0		0	
	0		0		0	
	0		0		0	
	0		0		0	
	0		0		0	
	0	Ew/a-GOST-ar	1	ONLY_E-w/a-	1	
	0	Ew/a-GOST-ar	1	ONLY_E-w/a-	1	
	0	Ew/a-GOST-ar	1	ONLY_E-w/a-	1	
	0		0		0	
	0		0		0	
E-ASA-art-Y	1	Ew/a-GOST-ar	1		0	E-ASA-cit-Y
E-ASA-art-Y	1	Ew/a-GOST-ar	1		0	E-ASA-cit-Y
	0		0		0	
	0		0		0	
	0		0		0	
	0		0		0	
	0	Ew/a-GOST-ar	1	ONLY_E-w/a-	1	
	0	Ew/a-GOST-ar	1	ONLY_E-w/a-	1	
	0		0		0	
E-ASA-art-Y	1		0		0	
E-ASA-art-Y	1		0		0	
	0		0		0	
	0		0		0	E-ASA-cit-Y
	0		0		0	
E-ASA-art-Y	1		0		0	
	0		0		0	
	0		0		0	
	0		0		0	
	0		0		0	
	0		0		0	
	0		0		0	
	0		0		0	
	0		0		0	E-ASA-cit-Y
	0		0		0	
	0	Ew/a-GOST-ar	1	ONLY_E-w/a-	1	E-ASA-cit-Y
	0		0		0	
	0		0		0	
	0		0		0	
	0		0		0	
	0		0		0	
	0		0		0	E-ASA-cit-Y
	0		0		0	E-ASA-cit-Y
E-ASA-art-Y	1	Ew/a-GOST-ar	1		0	E-ASA-cit-Y
E-ASA-art-Y	1	Ew/a-GOST-ar	1		0	E-ASA-cit-Y
E-ASA-art-Y	1	Ew/a-GOST-ar	1		0	E-ASA-cit-Y
	0	Ew/a-GOST-ar	1	ONLY_E-w/a-	1	E-ASA-cit-Y
	0	Ew/a-GOST-ar	1	ONLY_E-w/a-	1	E-ASA-cit-Y

	0	Ew/a-GOST-a	1	ONLY_E-w/a-	1	E-ASA-cit-Y
	0	Ew/a-GOST-a	1	ONLY_E-w/a-	1	E-ASA-cit-Y
	0	Ew/a-GOST-a	1	ONLY_E-w/a-	1	E-ASA-cit-Y
	0		0		0	
	0		0		0	
	0	Ew/a-GOST-a	1	ONLY_E-w/a-	1	
	0		0		0	
	0		0		0	
	0		0		0	
	0		0		0	
	0		0		0	
	0		0		0	
	0	Ew/a-GOST-a	1	ONLY_E-w/a-	1	
	0		0		0	
	0		0		0	
	0		0		0	
	0	Ew/a-GOST-a	1	ONLY_E-w/a-	1	
	0		0		0	E-ASA-cit-Y
	0		0		0	
	0		0		0	
	0	Ew/a-GOST-a	1	ONLY_E-w/a-	1	
	0	Ew/a-GOST-a	1	ONLY_E-w/a-	1	
	0		0		0	
	0		0		0	
	0	Ew/a-GOST-a	1	ONLY_E-w/a-	1	
	0	Ew/a-GOST-a	1	ONLY_E-w/a-	1	
	0		0		0	E-ASA-cit-Y
	0	Ew/a-GOST-a	1	ONLY_E-w/a-	1	
	0	Ew/a-GOST-a	1	ONLY_E-w/a-	1	
	0	Ew/a-GOST-a	1	ONLY_E-w/a-	1	
	0		0		0	
	0	Ew/a-GOST-a	1	ONLY_E-w/a-	1	
	0	Ew/a-GOST-a	1	ONLY_E-w/a-	1	
E-ASA-art-Y	1	Ew/a-GOST-a	1		0	
E-ASA-art-Y	1	Ew/a-GOST-a	1		0	
	0		0		0	
	0		0		0	
	0		0		0	
	0		0		0	
	0		0		0	
	0		0		0	
	0		0		0	
	0		0		0	
	0		0		0	
	0		0		0	
	0		0		0	
	0		0		0	

	0		0		0	
	0		0		0	
E-ASA-art-Y	1	Ew/a-GOST-a	1		0	E-ASA-cit-Y
E-ASA-art-Y	1	Ew/a-GOST-a	1		0	E-ASA-cit-Y
E-ASA-art-Y	1	Ew/a-GOST-a	1		0	E-ASA-cit-Y
E-ASA-art-Y	1	Ew/a-GOST-a	1		0	E-ASA-cit-Y
E-ASA-art-Y	1	Ew/a-GOST-a	1		0	
	0		0		0	
	0		0		0	
	0		0		0	
	0		0		0	
	0	Ew/a-GOST-a	1	ONLY_E-w/a-	1	
	0		0		0	
	0		0		0	E-ASA-cit-Y
	0		0		0	E-ASA-cit-Y
	0		0		0	E-ASA-cit-Y
	0	Ew/a-GOST-a	1	ONLY_E-w/a-	1	
	0	Ew/a-GOST-a	1	ONLY_E-w/a-	1	
	0	Ew/a-GOST-a	1	ONLY_E-w/a-	1	
	0	Ew/a-GOST-a	1	ONLY_E-w/a-	1	
	0		0		0	
	0		0		0	
	0		0		0	
	0		0		0	
	0		0		0	
	0		0		0	
	0		0		0	
	0		0		0	
	0	Ew/a-GOST-a	1	ONLY_E-w/a-	1	
	0	Ew/a-GOST-a	1	ONLY_E-w/a-	1	
	0		0		0	
	0	Ew/a-GOST-a	1	ONLY_E-w/a-	1	E-ASA-cit-Y
E-ASA-art-Y	1	Ew/a-GOST-a	1		0	
E-ASA-art-Y	1	Ew/a-GOST-a	1		0	
E-ASA-art-Y	1	Ew/a-GOST-a	1		0	
	0		0		0	
E-ASA-art-Y	1		0		0	
	0		0		0	
	0		0		0	
	0	Ew/a-GOST-a	1	ONLY_E-w/a-	1	
	0		0		0	
	0		0		0	
	0	Ew/a-GOST-a	1	ONLY_E-w/a-	1	
	0	Ew/a-GOST-a	1	ONLY_E-w/a-	1	
	0		0		0	
	0		0		0	
	0		0		0	

	0		0		0	
	0		0		0	
	0	Ew/a-GOST-a	1	ONLY_E-w/a-	1	
	0	Ew/a-GOST-a	1	ONLY_E-w/a-	1	
	0	Ew/a-GOST-a	1	ONLY_E-w/a-	1	
E-ASA-art-Y	1	Ew/a-GOST-a	1		0	E-ASA-cit-Y
E-ASA-art-Y	1	Ew/a-GOST-a	1		0	E-ASA-cit-Y
E-ASA-art-Y	1	Ew/a-GOST-a	1		0	E-ASA-cit-Y
	0		0		0	
	0		0		0	
	0	Ew/a-GOST-a	1	ONLY_E-w/a-	1	
	0		0		0	
	0		0		0	
	0		0		0	
	0	Ew/a-GOST-a	1	ONLY_E-w/a-	1	
	0	Ew/a-GOST-a	1	ONLY_E-w/a-	1	
	0		0		0	
	0	Ew/a-GOST-a	1	ONLY_E-w/a-	1	
	0		0		0	
	0		0		0	
	0		0		0	
	0		0		0	
	0		0		0	
	0		0		0	
	0		0		0	
E-ASA-art-Y	1	Ew/a-GOST-a	1		0	E-ASA-cit-Y
E-ASA-art-Y	1	Ew/a-GOST-a	1		0	E-ASA-cit-Y
E-ASA-art-Y	1	Ew/a-GOST-a	1		0	E-ASA-cit-Y
E-ASA-art-Y	1	Ew/a-GOST-a	1		0	E-ASA-cit-Y
E-ASA-art-Y	1	Ew/a-GOST-a	1		0	E-ASA-cit-Y
	0		0		0	
	0		0		0	E-ASA-cit-Y
	0		0		0	E-ASA-cit-Y
	0	Ew/a-GOST-a	1	ONLY_E-w/a-	1	
	0		0		0	
	0		0		0	
	0		0		0	
E-ASA-art-Y	1	Ew/a-GOST-a	1		0	
E-ASA-art-Y	1	Ew/a-GOST-a	1		0	
	0		0		0	
	0	Ew/a-GOST-a	1	ONLY_E-w/a-	1	
	0	Ew/a-GOST-a	1	ONLY_E-w/a-	1	
	0	Ew/a-GOST-a	1	ONLY_E-w/a-	1	
E-ASA-art-Y	1	Ew/a-GOST-a	1		0	
E-ASA-art-Y	1	Ew/a-GOST-a	1		0	
	0	Ew/a-GOST-a	1	ONLY_E-w/a-	1	
	0		0		0	

	0		0		0	
	0	Ew/a-GOST-a	1	ONLY_E-w/a-	1	
	0		0		0	
	0		0		0	
	0		0		0	
E-ASA-art-Y	1		0		0	
E-ASA-art-Y	1		0		0	
E-ASA-art-Y	1		0		0	E-ASA-cit-Y
E-ASA-art-Y	1		0		0	E-ASA-cit-Y
E-ASA-art-Y	1		0		0	
E-ASA-art-Y	1		0		0	
	0	Ew/a-GOST-a	1	ONLY_E-w/a-	1	
	0	Ew/a-GOST-a	1	ONLY_E-w/a-	1	
	0	Ew/a-GOST-a	1	ONLY_E-w/a-	1	
	0		0		0	
	0		0		0	
	0		0		0	E-ASA-cit-Y
	0	Ew/a-GOST-a	1	ONLY_E-w/a-	1	
	0	Ew/a-GOST-a	1	ONLY_E-w/a-	1	
E-ASA-art-Y	1	Ew/a-GOST-a	1		0	
E-ASA-art-Y	1	Ew/a-GOST-a	1		0	
	0		0		0	
	0		0		0	
	0		0		0	
	0		0		0	
	0	Ew/a-GOST-a	1	ONLY_E-w/a-	1	
	0		0		0	
	0		0		0	
E-ASA-art-Y	1		0		0	
E-ASA-art-Y	1		0		0	
	0	Ew/a-GOST-a	1	ONLY_E-w/a-	1	E-ASA-cit-Y
	0	Ew/a-GOST-a	1	ONLY_E-w/a-	1	E-ASA-cit-Y
E-ASA-art-Y	1		0		0	
	0		0		0	
	0		0		0	
	0		0		0	
	0		0		0	
	0		0		0	
	0		0		0	
	0		0		0	
	0		0		0	
	0		0		0	
	0		0		0	
	0	Ew/a-GOST-a	1	ONLY_E-w/a-	1	
E-ASA-art-Y	1	Ew/a-GOST-a	1		0	E-ASA-cit-Y
E-ASA-art-Y	1	Ew/a-GOST-a	1		0	E-ASA-cit-Y
	0		0		0	

	0		0		0	
	0		0		0	
	0		0		0	
	0		0		0	
	0		0		0	
	0		0		0	
	0	Ew/a-GOST-a	1	ONLY_E-w/a-	1	
	0	Ew/a-GOST-a	1	ONLY_E-w/a-	1	
	0		0		0	
	0		0		0	
	0	Ew/a-GOST-a	1	ONLY_E-w/a-	1	E-ASA-cit-Y
	0	Ew/a-GOST-a	1	ONLY_E-w/a-	1	
	0		0		0	
	0		0		0	
	0		0		0	
	0	Ew/a-GOST-a	1	ONLY_E-w/a-	1	
	0	Ew/a-GOST-a	1	ONLY_E-w/a-	1	
	0		0		0	
	0		0		0	
	0		0		0	
	0		0		0	
	0	Ew/a-GOST-a	1	ONLY_E-w/a-	1	
	0	Ew/a-GOST-a	1	ONLY_E-w/a-	1	
	0	Ew/a-GOST-a	1	ONLY_E-w/a-	1	
E-ASA-art-Y	1		0		0	
	0	Ew/a-GOST-a	1	ONLY_E-w/a-	1	
	0	Ew/a-GOST-a	1	ONLY_E-w/a-	1	
	0		0		0	
	0		0		0	
	0		0		0	
	0		0		0	
	0		0		0	
	0	Ew/a-GOST-a	1	ONLY_E-w/a-	1	
	0	Ew/a-GOST-a	1	ONLY_E-w/a-	1	
	0		0		0	
	0		0		0	
	0		0		0	
	0	Ew/a-GOST-a	1	ONLY_E-w/a-	1	
	0	Ew/a-GOST-a	1	ONLY_E-w/a-	1	
	0		0		0	
	0		0		0	E-ASA-cit-Y
	0		0		0	E-ASA-cit-Y
	0		0		0	E-ASA-cit-Y
	0		0		0	
	0	Ew/a-GOST-a	1	ONLY_E-w/a-	1	
	0	Ew/a-GOST-a	1	ONLY_E-w/a-	1	
E-ASA-art-Y	1		0		0	

E-ASA-art-Y	1	Ew/a-GOST-ar	1		0	
E-ASA-art-Y	1	Ew/a-GOST-ar	1		0	
E-ASA-art-Y	1	Ew/a-GOST-ar	1		0	
	0		0		0	
	0		0		0	
	0		0		0	
	0	Ew/a-GOST-ar	1	ONLY_E-w/a-	1	
	0	Ew/a-GOST-ar	1	ONLY_E-w/a-	1	
	0	Ew/a-GOST-ar	1	ONLY_E-w/a-	1	
	0	Ew/a-GOST-ar	1	ONLY_E-w/a-	1	
	0		0		0	
	0	Ew/a-GOST-ar	1	ONLY_E-w/a-	1	
	0		0		0	
	0		0		0	
	0		0		0	
	0		0		0	
	0	Ew/a-GOST-ar	1	ONLY_E-w/a-	1	
	0		0		0	
	0		0		0	
	0		0		0	
	0		0		0	
	0		0		0	
	0		0		0	
	0		0		0	
	0		0		0	
	0		0		0	
	0		0		0	
	0		0		0	
	0		0		0	
E-ASA-art-Y	1	Ew/a-GOST-ar	1		0	E-ASA-cit-Y
E-ASA-art-Y	1	Ew/a-GOST-ar	1		0	E-ASA-cit-Y
	0		0		0	
	0		0		0	
E-ASA-art-Y	1	Ew/a-GOST-ar	1		0	
E-ASA-art-Y	1	Ew/a-GOST-ar	1		0	
	0	Ew/a-GOST-ar	1	ONLY_E-w/a-	1	
	0	Ew/a-GOST-ar	1	ONLY_E-w/a-	1	
	0	Ew/a-GOST-ar	1	ONLY_E-w/a-	1	
	0		0		0	
	0	Ew/a-GOST-ar	1	ONLY_E-w/a-	1	
	0	Ew/a-GOST-ar	1	ONLY_E-w/a-	1	
	0	Ew/a-GOST-ar	1	ONLY_E-w/a-	1	
	0		0		0	
	0	Ew/a-GOST-ar	1	ONLY_E-w/a-	1	
	0	Ew/a-GOST-ar	1	ONLY_E-w/a-	1	
	0	Ew/a-GOST-ar	1	ONLY_E-w/a-	1	
	0		0		0	

	0	Ew/a-GOST-ar	1	ONLY_E-w/a-(	1
	0		0		0
	0		0		0
	0		0		0
	0		0		0
	0		0		0
	0		0		0
	0		0		0
	0		0		0
	0		0		0
	0		0		0
	0		0		0
	0		0		0
	0		0		0
	0	Ew/a-GOST-ar	1	ONLY_E-w/a-(	1
	0	Ew/a-GOST-ar	1	ONLY_E-w/a-(	1
	0	Ew/a-GOST-ar	1	ONLY_E-w/a-(	1
	0		0		0
	0		0		0
	0		0		0
	0		0		0
	0		0		0
	0		0		0
	0	Ew/a-GOST-ar	1	ONLY_E-w/a-(	1
	0		0		0
	0		0		0
	0		0		0
	0		0		0
	0		0		0
	0		0		0
	0		0		0
	0		0		0
	0		0		0
E-ASA-art-Y	1	Ew/a-GOST-ar	1		0
E-ASA-art-Y	1	Ew/a-GOST-ar	1		0
	0		0		0
	0		0		0
	0	Ew/a-GOST-ar	1	ONLY_E-w/a-(	1
	0	Ew/a-GOST-ar	1	ONLY_E-w/a-(	1
	0	Ew/a-GOST-ar	1	ONLY_E-w/a-(	1
	0	Ew/a-GOST-ar	1	ONLY_E-w/a-(	1
	0		0		0
	0		0		0
	0		0		0
	0		0		0
	0		0		0
	0	Ew/a-GOST-ar	1	ONLY_E-w/a-(	1

	0		0		0	
	0		0		0	
	0		0		0	
	0		0		0	
E-ASA-art-Y	1		0		0	E-ASA-cit-Y
	0		0		0	
	0	Ew/a-GOST-a	1	ONLY_E-w/a-	1	
	0		0		0	
	0		0		0	
	0	Ew/a-GOST-a	1	ONLY_E-w/a-	1	
	0	Ew/a-GOST-a	1	ONLY_E-w/a-	1	
	0		0		0	
	0		0		0	
	0		0		0	
	0		0		0	
	0		0		0	
	0	Ew/a-GOST-a	1	ONLY_E-w/a-	1	
	0	Ew/a-GOST-a	1	ONLY_E-w/a-	1	
	0		0		0	
	0		0		0	
	0		0		0	
	0		0		0	
	0		0		0	
	0		0		0	
	0		0		0	
	0	Ew/a-GOST-a	1	ONLY_E-w/a-	1	
	0	Ew/a-GOST-a	1	ONLY_E-w/a-	1	
	0	Ew/a-GOST-a	1	ONLY_E-w/a-	1	
	0	Ew/a-GOST-a	1	ONLY_E-w/a-	1	
	0		0		0	
	0		0		0	
	0		0		0	
	0		0		0	
	0		0		0	
	0		0		0	
	0		0		0	
	0		0		0	
	0		0		0	
	0		0		0	
	0		0		0	
	0		0		0	
E-ASA-art-Y	1	Ew/a-GOST-a	1		0	
	0		0		0	
	0	Ew/a-GOST-a	1	ONLY_E-w/a-	1	
	0	Ew/a-GOST-a	1	ONLY_E-w/a-	1	
	0	Ew/a-GOST-a	1	ONLY_E-w/a-	1	
	0		0		0	

	0		0		0	
	0		0		0	
E-ASA-art-Y	1		0		0	
	0		0		0	
	0		0		0	
	0		0		0	
	0		0		0	
	0		0		0	
	0	Ew/a-GOST-ar	1	ONLY_E-w/a-(	1	
	0	Ew/a-GOST-ar	1	ONLY_E-w/a-(	1	
E-ASA-art-Y	1	Ew/a-GOST-ar	1		0	
E-ASA-art-Y	1	Ew/a-GOST-ar	1		0	
	0		0		0	
	0		0		0	
	0		0		0	
	0	Ew/a-GOST-ar	1	ONLY_E-w/a-(	1	
	0	Ew/a-GOST-ar	1	ONLY_E-w/a-(	1	
	0	Ew/a-GOST-ar	1	ONLY_E-w/a-(	1	
	0	Ew/a-GOST-ar	1	ONLY_E-w/a-(	1	
E-ASA-art-Y	1	Ew/a-GOST-ar	1		0	E-ASA-cit-Y
E-ASA-art-Y	1	Ew/a-GOST-ar	1		0	E-ASA-cit-Y
E-ASA-art-Y	1	Ew/a-GOST-ar	1		0	E-ASA-cit-Y
	0	Ew/a-GOST-ar	1	ONLY_E-w/a-(	1	
	0		0		0	
	0	Ew/a-GOST-ar	1	ONLY_E-w/a-(	1	
	0	Ew/a-GOST-ar	1	ONLY_E-w/a-(	1	
	0	Ew/a-GOST-ar	1	ONLY_E-w/a-(	1	
	0	Ew/a-GOST-ar	1	ONLY_E-w/a-(	1	
	0		0		0	
	0		0		0	
	0		0		0	
	0		0		0	
	0		0		0	
	0	Ew/a-GOST-ar	1	ONLY_E-w/a-(	1	
	0		0		0	
	0		0		0	
E-ASA-art-Y	1	Ew/a-GOST-ar	1		0	
E-ASA-art-Y	1	Ew/a-GOST-ar	1		0	
E-ASA-art-Y	1	Ew/a-GOST-ar	1		0	
	0	Ew/a-GOST-ar	1	ONLY_E-w/a-(	1	
	0		0		0	
	0		0		0	
	0		0		0	
	0		0		0	
	0	Ew/a-GOST-ar	1	ONLY_E-w/a-(	1	
	0	Ew/a-GOST-ar	1	ONLY_E-w/a-(	1	
	0	Ew/a-GOST-ar	1	ONLY_E-w/a-(	1	

	0	Ew/a-GOST-ar	1	ONLY_E-w/a-(	1	
	0	Ew/a-GOST-ar	1	ONLY_E-w/a-(	1	
	0		0		0	
	0		0		0	
	0		0		0	
	0		0		0	
	0	Ew/a-GOST-ar	1	ONLY_E-w/a-(	1	
	0		0		0	
	0		0		0	
	0		0		0	
	0		0		0	
	0		0		0	E-ASA-cit-Y
	0		0		0	E-ASA-cit-Y
E-ASA-art-Y	1		0		0	
E-ASA-art-Y	1		0		0	
E-ASA-art-Y	1		0		0	
	0		0		0	E-ASA-cit-Y
	0		0		0	E-ASA-cit-Y
	0		0		0	
	0		0		0	
	0		0		0	
	0	Ew/a-GOST-ar	1	ONLY_E-w/a-(	1	
	0	Ew/a-GOST-ar	1	ONLY_E-w/a-(	1	
	0		0		0	
	0		0		0	
	0		0		0	
	0		0		0	
	0		0		0	
	0		0		0	
	0		0		0	
	0		0		0	
	0		0		0	
	0		0		0	
	0		0		0	
	0		0		0	
	0		0		0	
	0		0		0	
	0		0		0	
	0		0		0	
	0	Ew/a-GOST-ar	1	ONLY_E-w/a-(	1	
	0	Ew/a-GOST-ar	1	ONLY_E-w/a-(	1	
	0		0		0	
E-ASA-art-Y	1		0		0	
	0		0		0	
E-ASA-art-Y	1		0		0	E-ASA-cit-Y
	0		0		0	
	0		0		0	

	0		0		0	
	0		0		0	
	0		0		0	
E-ASA-art-Y	1		0		0	
	0	Ew/a-GOST-ar	1	ONLY_E-w/a-(	1	
	0	Ew/a-GOST-ar	1	ONLY_E-w/a-(	1	
E-ASA-art-Y	1		0		0	
	0		0		0	
	0		0		0	
	0		0		0	
	0	Ew/a-GOST-ar	1	ONLY_E-w/a-(	1	
E-ASA-art-Y	1	Ew/a-GOST-ar	1		0	E-ASA-cit-Y
E-ASA-art-Y	1	Ew/a-GOST-ar	1		0	E-ASA-cit-Y
	0	Ew/a-GOST-ar	1	ONLY_E-w/a-(	1	
	0	Ew/a-GOST-ar	1	ONLY_E-w/a-(	1	
	0	Ew/a-GOST-ar	1	ONLY_E-w/a-(	1	
	0	Ew/a-GOST-ar	1	ONLY_E-w/a-(	1	
	0		0		0	
	0		0		0	
	0		0		0	
	0		0		0	
	0		0		0	E-ASA-cit-Y
	0		0		0	E-ASA-cit-Y
	0		0		0	E-ASA-cit-Y
	0		0		0	
	0		0		0	
	0		0		0	
E-ASA-art-Y	1		0		0	
E-ASA-art-Y	1		0		0	
E-ASA-art-Y	1		0		0	
E-ASA-art-Y	1	Ew/a-GOST-ar	1		0	
	0	Ew/a-GOST-ar	1	ONLY_E-w/a-(	1	
	0	Ew/a-GOST-ar	1	ONLY_E-w/a-(	1	
	0	Ew/a-GOST-ar	1	ONLY_E-w/a-(	1	
	0	Ew/a-GOST-ar	1	ONLY_E-w/a-(	1	
	0		0		0	
	0		0		0	
	0		0		0	
	0		0		0	
	0		0		0	
E-ASA-art-Y	1		0		0	
	0		0		0	
	0		0		0	
	0		0		0	
	0		0		0	
E-ASA-art-Y	1		0		0	
	0	Ew/a-GOST-ar	1	ONLY_E-w/a-(	1	

	0	Ew/a-GOST-ar	1	ONLY_E-w/a-(	1	
	0		0		0	
	0		0		0	
	0		0		0	
	0		0		0	
	0	Ew/a-GOST-ar	1	ONLY_E-w/a-(	1	
	0	Ew/a-GOST-ar	1	ONLY_E-w/a-(	1	
E-ASA-art-Y	1		0		0	E-ASA-cit-Y
E-ASA-art-Y	1		0		0	E-ASA-cit-Y
E-ASA-art-Y	1		0		0	E-ASA-cit-Y
E-ASA-art-Y	1	Ew/a-GOST-ar	1		0	E-ASA-cit-Y
E-ASA-art-Y	1	Ew/a-GOST-ar	1		0	E-ASA-cit-Y
E-ASA-art-Y	1	Ew/a-GOST-ar	1		0	E-ASA-cit-Y
E-ASA-art-Y	1	Ew/a-GOST-ar	1		0	E-ASA-cit-Y
	0		0		0	
	0		0		0	
	0		0		0	
	0		0		0	
	0	Ew/a-GOST-ar	1	ONLY_E-w/a-(	1	
	0	Ew/a-GOST-ar	1	ONLY_E-w/a-(	1	
	0		0		0	
	0		0		0	
	0		0		0	
	0		0		0	
	0		0		0	
E-ASA-art-Y	1		0		0	
E-ASA-art-Y	1		0		0	
	0		0		0	
	0		0		0	
	0	Ew/a-GOST-ar	1	ONLY_E-w/a-(	1	
	0	Ew/a-GOST-ar	1	ONLY_E-w/a-(	1	
	0	Ew/a-GOST-ar	1	ONLY_E-w/a-(	1	
E-ASA-art-Y	1		0		0	
E-ASA-art-Y	1		0		0	
	0		0		0	
	0		0		0	
	0		0		0	
	0	Ew/a-GOST-ar	1	ONLY_E-w/a-(	1	
	0	Ew/a-GOST-ar	1	ONLY_E-w/a-(	1	
	0		0		0	
	0		0		0	
E-ASA-art-Y	1		0		0	
	0		0		0	
E-ASA-art-Y	1	Ew/a-GOST-ar	1		0	E-ASA-cit-Y
E-ASA-art-Y	1	Ew/a-GOST-ar	1		0	E-ASA-cit-Y
E-ASA-art-Y	1	Ew/a-GOST-ar	1		0	E-ASA-cit-Y
	0		0		0	

	0		0		0	
	0		0		0	
	0	Ew/a-GOST-a	1	ONLY_E-w/a-	1	
	0	Ew/a-GOST-a	1	ONLY_E-w/a-	1	
	0		0		0	
	0	Ew/a-GOST-a	1	ONLY_E-w/a-	1	
	0	Ew/a-GOST-a	1	ONLY_E-w/a-	1	
	0		0		0	E-ASA-cit-Y
	0		0		0	E-ASA-cit-Y
	0		0		0	
	0		0		0	
	0		0		0	
E-ASA-art-Y	1		0		0	E-ASA-cit-Y
	0		0		0	
E-ASA-art-Y	1	Ew/a-GOST-a	1		0	
E-ASA-art-Y	1	Ew/a-GOST-a	1		0	
	0		0		0	
	0		0		0	
	0	Ew/a-GOST-a	1	ONLY_E-w/a-	1	
	0		0		0	
	0		0		0	
	0	Ew/a-GOST-a	1	ONLY_E-w/a-	1	
	0	Ew/a-GOST-a	1	ONLY_E-w/a-	1	
	0	Ew/a-GOST-a	1	ONLY_E-w/a-	1	
E-ASA-art-Y	1		0		0	E-ASA-cit-Y
	0		0		0	
	0		0		0	
	0		0		0	
	0		0		0	
E-ASA-art-Y	1		0		0	
	0	Ew/a-GOST-a	1	ONLY_E-w/a-	1	
	0		0		0	
	0		0		0	
	0		0		0	
	0		0		0	
	0	Ew/a-GOST-a	1	ONLY_E-w/a-	1	
	0		0		0	
	0		0		0	
	0		0		0	
	0		0		0	
	0		0		0	
	0		0		0	
E-ASA-art-Y	1		0		0	E-ASA-cit-Y
E-ASA-art-Y	1	Ew/a-GOST-a	1		0	E-ASA-cit-Y
E-ASA-art-Y	1	Ew/a-GOST-a	1		0	E-ASA-cit-Y
E-ASA-art-Y	1	Ew/a-GOST-a	1		0	E-ASA-cit-Y
E-ASA-art-Y	1	Ew/a-GOST-a	1		0	E-ASA-cit-Y

	0	Ew/a-GOST-ar	1	ONLY_E-w/a-(	1	
	0	Ew/a-GOST-ar	1	ONLY_E-w/a-(	1	
	0		0		0	
	0		0		0	
	0	Ew/a-GOST-ar	1	ONLY_E-w/a-(	1	
	0	Ew/a-GOST-ar	1	ONLY_E-w/a-(	1	
	0	Ew/a-GOST-ar	1	ONLY_E-w/a-(	1	
	0		0		0	
	0		0		0	
	0	Ew/a-GOST-ar	1	ONLY_E-w/a-(	1	
	0	Ew/a-GOST-ar	1	ONLY_E-w/a-(	1	
E-ASA-art-Y	1		0		0	E-ASA-cit-Y
	0	Ew/a-GOST-ar	1	ONLY_E-w/a-(	1	
	0	Ew/a-GOST-ar	1	ONLY_E-w/a-(	1	
	0	Ew/a-GOST-ar	1	ONLY_E-w/a-(	1	
	0	Ew/a-GOST-ar	1	ONLY_E-w/a-(	1	
	0	Ew/a-GOST-ar	1	ONLY_E-w/a-(	1	
	0	Ew/a-GOST-ar	1	ONLY_E-w/a-(	1	
	0		0		0	
	0		0		0	
	0		0		0	
	0		0		0	
	0		0		0	
	0		0		0	
	0		0		0	
	0		0		0	
	0		0		0	
	0		0		0	
	0	Ew/a-GOST-ar	1	ONLY_E-w/a-(	1	
	0		0		0	
	0		0		0	
	0		0		0	
	0	Ew/a-GOST-ar	1	ONLY_E-w/a-(	1	
	0	Ew/a-GOST-ar	1	ONLY_E-w/a-(	1	
E-ASA-art-Y	1	Ew/a-GOST-ar	1		0	E-ASA-cit-Y
E-ASA-art-Y	1	Ew/a-GOST-ar	1		0	E-ASA-cit-Y
	0		0		0	
	0		0		0	
	0		0		0	
	0	Ew/a-GOST-ar	1	ONLY_E-w/a-(	1	
	0	Ew/a-GOST-ar	1	ONLY_E-w/a-(	1	
	0	Ew/a-GOST-ar	1	ONLY_E-w/a-(	1	
	0		0		0	
	0		0		0	
	0		0		0	
	0		0		0	
	0		0		0	

	0		0		0	
	0		0		0	
	0	Ew/a-GOST-ar	1	ONLY_E-w/a-(	1	
	0	Ew/a-GOST-ar	1	ONLY_E-w/a-(	1	
	0		0		0	
	0		0		0	
	0		0		0	
	0		0		0	
E-ASA-art-Y	1		0		0	E-ASA-cit-Y
E-ASA-art-Y	1		0		0	E-ASA-cit-Y
E-ASA-art-Y	1		0		0	E-ASA-cit-Y
E-ASA-art-Y	1		0		0	E-ASA-cit-Y
E-ASA-art-Y	1		0		0	E-ASA-cit-Y
	0		0		0	
	0		0		0	
	0		0		0	
	0		0		0	
	0		0		0	
	0		0		0	
	0		0		0	
	0		0		0	
	0		0		0	
	0		0		0	
	0		0		0	
	0		0		0	
	0		0		0	
	0		0		0	
	0		0		0	
	0		0		0	
	0		0		0	
	0		0		0	
	0		0		0	
	0		0		0	
	0		0		0	
	0		0		0	
	0		0		0	
	0		0		0	
	0		0		0	
	0		0		0	E-ASA-cit-Y
	0	Ew/a-GOST-ar	1	ONLY_E-w/a-(	1	
	0	Ew/a-GOST-ar	1	ONLY_E-w/a-(	1	
	0	Ew/a-GOST-ar	1	ONLY_E-w/a-(	1	
	0		0		0	
	0		0		0	
	0		0		0	
	0		0		0	

	0		0		0	
E-ASA-art-Y	1	Ew/a-GOST-ar	1		0	E-ASA-cit-Y
	0		0		0	
	0		0		0	
	0	Ew/a-GOST-ar	1	ONLY_E-w/a-(	1	
	0	Ew/a-GOST-ar	1	ONLY_E-w/a-(	1	
	0		0		0	
	0	Ew/a-GOST-ar	1	ONLY_E-w/a-(	1	
	0	Ew/a-GOST-ar	1	ONLY_E-w/a-(	1	
	0		0		0	
	0		0		0	
	0		0		0	
	0		0		0	
	0	Ew/a-GOST-ar	1	ONLY_E-w/a-(	1	
	0		0		0	
	0		0		0	
	0		0		0	
	0		0		0	
	0	Ew/a-GOST-ar	1	ONLY_E-w/a-(	1	
	0	Ew/a-GOST-ar	1	ONLY_E-w/a-(	1	
	0	Ew/a-GOST-ar	1	ONLY_E-w/a-(	1	
	0		0		0	
	0		0		0	
	0		0		0	
	0	Ew/a-GOST-ar	1	ONLY_E-w/a-(	1	
	0		0		0	
	0		0		0	
	0		0		0	E-ASA-cit-Y
	0		0		0	E-ASA-cit-Y
	0		0		0	
	0		0		0	
	0		0		0	
	0		0		0	
	0	Ew/a-GOST-ar	1	ONLY_E-w/a-(	1	
	0		0		0	
	0		0		0	
	0		0		0	
	0		0		0	
	0		0		0	
	0		0		0	
E-ASA-art-Y	1	Ew/a-GOST-ar	1		0	
E-ASA-art-Y	1	Ew/a-GOST-ar	1		0	
E-ASA-art-Y	1	Ew/a-GOST-ar	1		0	
	0	Ew/a-GOST-ar	1	ONLY_E-w/a-(	1	
	0	Ew/a-GOST-ar	1	ONLY_E-w/a-(	1	
	0	Ew/a-GOST-ar	1	ONLY_E-w/a-(	1	
	0		0		0	

	0		0		0	
	0	Ew/a-GOST-a	1	ONLY_E-w/a-	1	
	0	Ew/a-GOST-a	1	ONLY_E-w/a-	1	
	0	Ew/a-GOST-a	1	ONLY_E-w/a-	1	
	0		0		0	
	0		0		0	
	0		0		0	
	0		0		0	
	0		0		0	
	0		0		0	
	0		0		0	
	0		0		0	
	0		0		0	
	0		0		0	
	0		0		0	
	0		0		0	
	0	Ew/a-GOST-a	1	ONLY_E-w/a-	1	
	0	Ew/a-GOST-a	1	ONLY_E-w/a-	1	
E-ASA-art-Y	1		0		0	E-ASA-cit-Y
E-ASA-art-Y	1		0		0	E-ASA-cit-Y
E-ASA-art-Y	1		0		0	E-ASA-cit-Y
	0		0		0	
	0		0		0	
E-ASA-art-Y	1		0		0	
E-ASA-art-Y	1		0		0	
E-ASA-art-Y	1		0		0	
E-ASA-art-Y	1		0		0	
E-ASA-art-Y	1		0		0	E-ASA-cit-Y
	0		0		0	
	0		0		0	
	0	Ew/a-GOST-a	1	ONLY_E-w/a-	1	
	0	Ew/a-GOST-a	1	ONLY_E-w/a-	1	
E-ASA-art-Y	1	Ew/a-GOST-a	1		0	
E-ASA-art-Y	1	Ew/a-GOST-a	1		0	
E-ASA-art-Y	1	Ew/a-GOST-a	1		0	
	0		0		0	
	0		0		0	
	0		0		0	
	0		0		0	
	0		0		0	
E-ASA-art-Y	1		0		0	
	0		0		0	
	0		0		0	
E-ASA-art-Y	1		0		0	
E-ASA-art-Y	1	Ew/a-GOST-a	1		0	
E-ASA-art-Y	1	Ew/a-GOST-a	1		0	
E-ASA-art-Y	1	Ew/a-GOST-a	1		0	

	0		0		0	
	0		0		0	
	0		0		0	
	0		0		0	
	0		0		0	
	0		0		0	
	0		0		0	
	0	Ew/a-GOST-ar	1	ONLY_E-w/a-	1	
	0		0		0	
	0		0		0	
	0		0		0	
	0		0		0	
E-ASA-art-Y	1		0		0	E-ASA-cit-Y
E-ASA-art-Y	1		0		0	E-ASA-cit-Y
E-ASA-art-Y	1		0		0	E-ASA-cit-Y
	0	Ew/a-GOST-ar	1	ONLY_E-w/a-	1	
	0	Ew/a-GOST-ar	1	ONLY_E-w/a-	1	
	0	Ew/a-GOST-ar	1	ONLY_E-w/a-	1	
	0		0		0	
	0		0		0	
	0	Ew/a-GOST-ar	1	ONLY_E-w/a-	1	
	0	Ew/a-GOST-ar	1	ONLY_E-w/a-	1	
	0	Ew/a-GOST-ar	1	ONLY_E-w/a-	1	
	0		0		0	
	0		0		0	
	0		0		0	
	0		0		0	
	0		0		0	
	0		0		0	
	0		0		0	
	0		0		0	
	0		0		0	
	0		0		0	
	0		0		0	
	0		0		0	E-ASA-cit-Y
	0		0		0	E-ASA-cit-Y
	0		0		0	E-ASA-cit-Y
	0		0		0	
	0		0		0	
	0		0		0	
	0		0		0	
	0		0		0	
	0		0		0	
	0		0		0	
	0		0		0	
	0		0		0	
	0		0		0	

	0		0		0	
	0		0		0	
	0		0		0	
	0		0		0	
	0		0		0	
	0		0		0	
	0		0		0	
E-ASA-art-Y	1		0		0	E-ASA-cit-Y
E-ASA-art-Y	1		0		0	E-ASA-cit-Y
	0		0		0	
	0		0		0	
E-ASA-art-Y	1	Ew/a-GOST-a	1		0	E-ASA-cit-Y
	0		0		0	
	0	Ew/a-GOST-a	1	ONLY_E-w/a-	1	
	0	Ew/a-GOST-a	1	ONLY_E-w/a-	1	
	0	Ew/a-GOST-a	1	ONLY_E-w/a-	1	
E-ASA-art-Y	1		0		0	E-ASA-cit-Y
E-ASA-art-Y	1		0		0	E-ASA-cit-Y
E-ASA-art-Y	1	Ew/a-GOST-a	1		0	E-ASA-cit-Y
E-ASA-art-Y	1	Ew/a-GOST-a	1		0	E-ASA-cit-Y
E-ASA-art-Y	1	Ew/a-GOST-a	1		0	E-ASA-cit-Y
E-ASA-art-Y	1	Ew/a-GOST-a	1		0	E-ASA-cit-Y
	0		0		0	
	0		0		0	
	0		0		0	
	0		0		0	
	0		0		0	E-ASA-cit-Y
	0		0		0	E-ASA-cit-Y
	0		0		0	E-ASA-cit-Y
	0		0		0	
	0		0		0	
	0		0		0	

easacity_b	ewagostcit	ewagostcit_b	only_ewagost	only_ewagost	v62	easaarty_2	easaarty_b_2
0		0		0			0
0		0		0			0
0		0		0			0
0		0		0			0
0		0		0			0
0		0		0			0
0		0		0			0
0		0		0			0
0		0		0			0
0		0		0			0
0	Ew/a-GOST-ci	1	ONLY_E-w/a-(	1			0
0		0		0			0
0		0		0			0
0		0		0			0
0		0		0			0
0		0		0			0
0		0		0			0
0		0		0			0
0		0		0			0
0		0		0			0
0		0		0			0
0		0		0			0
0		0		0			0
0		0		0			0
0		0		0			0
0		0		0			0
0		0		0			0
0		0		0			0
0		0		0			0
0		0		0			0
0		0		0			0
0		0		0			0
0		0		0			0
0		0		0			0
0		0		0			0
0		0		0			0
0		0		0			0
0		0		0			0
0		0		0			0
0		0		0			0
0		0		0			0
0		0		0			0
0		0		0			0
0		0		0			0
0		0		0			0
0		0		0			0

0		0		0	0
0		0		0	0
0	Ew/a-GOST-ci	1	ONLY_E-w/a-(	1	0
0		0		0	0
0		0		0	0
0		0		0	0
0		0		0	0
0		0		0	0
1	Ew/a-GOST-ci	1		0	0
0		0		0	0
0		0		0	0
0		0		0	0
0		0		0	0
0	Ew/a-GOST-ci	1	ONLY_E-w/a-(	1	0
0	Ew/a-GOST-ci	1	ONLY_E-w/a-(	1	0
0	Ew/a-GOST-ci	1	ONLY_E-w/a-(	1	0
0		0		0	0
0		0		0	0
0		0		0	0
0		0		0	0
0		0		0	0
0		0		0	0
0		0		0	0
0		0		0	0
0	Ew/a-GOST-ci	1	ONLY_E-w/a-(	1	0
0	Ew/a-GOST-ci	1	ONLY_E-w/a-(	1	0
0		0		0	0
0		0		0	0
0		0		0	0
0	Ew/a-GOST-ci	1	ONLY_E-w/a-(	1	1
0	Ew/a-GOST-ci	1	ONLY_E-w/a-(	1	1
0	Ew/a-GOST-ci	1	ONLY_E-w/a-(	1	1
0	Ew/a-GOST-ci	1	ONLY_E-w/a-(	1	1
0	Ew/a-GOST-ci	1	ONLY_E-w/a-(	1	1
0		0		0	0
0		0		0	0
0		0		0	0
0		0		0	0
0		0		0	0
0		0		0	0
0		0		0	0
0		0		0	0
0		0		0	0
0		0		0	0
0		0		0	0
0		0		0	0
0		0		0	0

0		0		0	0
0		0		0	0
0		0		0	0
0	Ew/a-GOST-ci	1	ONLY_E-w/a-(	1	0
0		0		0	0
0		0		0	0
0		0		0	0
0		0		0	0
0		0		0	0
0		0		0	0
0		0		0	0
0		0		0	0
0		0		0	0
0	Ew/a-GOST-ci	1	ONLY_E-w/a-(	1	0
0		0		0	0
0		0		0	0
0		0		0	0
0		0		0	0
0		0		0	0
0		0		0	0
0	Ew/a-GOST-ci	1	ONLY_E-w/a-(	1	0
0		0		0	0
0		0		0	0
0		0		0	0
0		0		0	0
0		0		0	0
0		0		0	0
1		0		0	0
1		0		0	0
0	Ew/a-GOST-ci	1	ONLY_E-w/a-(	1	1
0	Ew/a-GOST-ci	1	ONLY_E-w/a-(	1	1
0		0		0	0
1	Ew/a-GOST-ci	1		0	0
1	Ew/a-GOST-ci	1		0	0
0	Ew/a-GOST-ci	1	ONLY_E-w/a-(	1	0
0		0		0	0
0		0		0	0
0		0		0	0
0		0		0	0
1	Ew/a-GOST-ci	1		0	0
0		0		0	0
0		0		0	0
0		0		0	0
0		0		0	0
0		0		0	0
0		0		0	0
0		0		0	0

0		0		0	0
0		0		0	0
0	Ew/a-GOST-ci	1	ONLY_E-w/a-(	1	0
0	Ew/a-GOST-ci	1	ONLY_E-w/a-(	1	0
0	Ew/a-GOST-ci	1	ONLY_E-w/a-(	1	0
0		0		0	0
0		0		0	0
0		0		0	0
0		0		0	0
0		0		0	0
0		0		0	0
1	Ew/a-GOST-ci	1		0	0
1	Ew/a-GOST-ci	1		0	0
1		0		0	1
0		0		0	0
0		0		0	0
1	Ew/a-GOST-ci	1		0	1
0		0		0	0
0		0		0	0
0		0		0	0
0		0		0	0
0		0		0	0
0		0		0	0
0		0		0	0
0		0		0	0
0		0		0	0
0		0		0	0
0	Ew/a-GOST-ci	1	ONLY_E-w/a-(	1	0
0		0		0	0
0	Ew/a-GOST-ci	1	ONLY_E-w/a-(	1	0
0		0		0	0
0		0		0	0
1	Ew/a-GOST-ci	1		0	0
0		0		0	0
0		0		0	0
0		0		0	0
0		0		0	0
0	Ew/a-GOST-ci	1	ONLY_E-w/a-(	1	0
1	Ew/a-GOST-ci	1		0	0
0		0		0	0
0		0		0	0
0		0		0	1
0		0		0	1
0		0		0	0
0		0		0	0
0		0		0	0
0		0		0	0

0		0		0	0
0		0		0	0
0		0		0	0
0		0		0	0
1		0		0	0
1		0		0	0
0		0		0	0
0		0		0	0
0		0		0	0
0		0		0	0
0		0		0	0
0		0		0	0
0	Ew/a-GOST-ci	1	ONLY_E-w/a-	1	0
0	Ew/a-GOST-ci	1	ONLY_E-w/a-	1	0
0		0		0	0
0		0		0	0
0		0		0	0
0		0		0	0
0		0		0	0
0		0		0	0
0		0		0	0
0		0		0	0
0	Ew/a-GOST-ci	1	ONLY_E-w/a-	1	0
0	Ew/a-GOST-ci	1	ONLY_E-w/a-	1	0
0	Ew/a-GOST-ci	1	ONLY_E-w/a-	1	0
0	Ew/a-GOST-ci	1	ONLY_E-w/a-	1	0
0		0		0	0
0		0		0	0
0		0		0	0
0		0		0	0
0		0		0	0
0		0		0	0
0		0		0	0
0		0		0	0
1		0		0	0
0		0		0	0
0	Ew/a-GOST-ci	1	ONLY_E-w/a-	1	0
0		0		0	0
1	Ew/a-GOST-ci	1		0	1
1	Ew/a-GOST-ci	1		0	1
1	Ew/a-GOST-ci	1		0	1
1	Ew/a-GOST-ci	1		0	1
0		0		0	0
0	Ew/a-GOST-ci	1	ONLY_E-w/a-	1	0
0	Ew/a-GOST-ci	1	ONLY_E-w/a-	1	0
0	Ew/a-GOST-ci	1	ONLY_E-w/a-	1	0
0		0		0	0

0		0		0	0
0		0		0	0
0		0		0	0
0		0		0	0
0		0		0	0
0		0		0	0
0		0		0	0
0		0		0	0
0	Ew/a-GOST-ci	1	ONLY_E-w/a-(	1	0
0		0		0	0
0		0		0	0
0		0		0	0
0		0		0	0
0		0		0	0
0		0		0	0
0		0		0	0
0		0		0	0
0		0		0	0
0		0		0	0
0		0		0	0
0		0		0	0
1	Ew/a-GOST-ci	1		0	1
1	Ew/a-GOST-ci	1		0	1
1	Ew/a-GOST-ci	1		0	1
0		0		0	0
0		0		0	0
0	Ew/a-GOST-ci	1	ONLY_E-w/a-(	1	0
0	Ew/a-GOST-ci	1	ONLY_E-w/a-(	1	0
0		0		0	0
0		0		0	0
1	Ew/a-GOST-ci	1		0	1
1	Ew/a-GOST-ci	1		0	1
1	Ew/a-GOST-ci	1		0	1
0		0		0	0
0		0		0	0
0		0		0	0
0		0		0	0
0		0		0	0
0	Ew/a-GOST-ci	1	ONLY_E-w/a-(	1	0
0	Ew/a-GOST-ci	1	ONLY_E-w/a-(	1	0
0		0		0	0
0		0		0	0
0		0		0	0
0		0		0	0
0		0		0	0
0	Ew/a-GOST-ci	1	ONLY_E-w/a-(	1	0
0		0		0	0

0	Ew/a-GOST-ci	1	ONLY_E-w/a-(	1	0
0	Ew/a-GOST-ci	1	ONLY_E-w/a-(	1	0
0	Ew/a-GOST-ci	1	ONLY_E-w/a-(	1	0
0		0		0	0
0	Ew/a-GOST-ci	1	ONLY_E-w/a-(	1	0
0	Ew/a-GOST-ci	1	ONLY_E-w/a-(	1	0
0	Ew/a-GOST-ci	1	ONLY_E-w/a-(	1	0
0		0		0	0
0		0		0	0
0	Ew/a-GOST-ci	1	ONLY_E-w/a-(	1	0
0	Ew/a-GOST-ci	1	ONLY_E-w/a-(	1	0
0		0		0	0
0		0		0	0
0		0		0	0
0		0		0	0
0		0		0	0
0		0		0	0
0		0		0	0
0		0		0	0
0		0		0	0
0		0		0	0
0		0		0	0
0		0		0	0
0		0		0	0
0		0		0	0
0	Ew/a-GOST-ci	1	ONLY_E-w/a-(	1	0
0	Ew/a-GOST-ci	1	ONLY_E-w/a-(	1	0
0	Ew/a-GOST-ci	1	ONLY_E-w/a-(	1	0
0	Ew/a-GOST-ci	1	ONLY_E-w/a-(	1	0
0	Ew/a-GOST-ci	1	ONLY_E-w/a-(	1	0
0		0		0	0
0		0		0	0
0	Ew/a-GOST-ci	1	ONLY_E-w/a-(	1	0
0		0		0	0
0		0		0	0
0		0		0	0
0		0		0	0
0		0		0	0
0		0		0	0
0		0		0	0
0		0		0	0
0		0		0	0
0		0		0	0
0		0		0	0
0		0		0	0
0		0		0	0
0		0		0	0

0		0		0	0
0		0		0	0
0		0		0	0
0		0		0	0
0		0		0	0
0	Ew/a-GOST-ci	1	ONLY_E-w/a-(	1	0
0	Ew/a-GOST-ci	1	ONLY_E-w/a-(	1	0
0		0		0	0
0		0		0	0
0		0		0	0
0		0		0	0
0		0		0	0
0		0		0	0
0		0		0	0
0	Ew/a-GOST-ci	1	ONLY_E-w/a-(	1	1
0	Ew/a-GOST-ci	1	ONLY_E-w/a-(	1	1
0		0		0	0
0		0		0	0
0		0		0	0
0	Ew/a-GOST-ci	1	ONLY_E-w/a-(	1	0
0		0		0	0
0		0		0	0
0		0		0	0
0		0		0	0
1	Ew/a-GOST-ci	1		0	0
1	Ew/a-GOST-ci	1		0	0
0		0		0	0
0		0		0	0
0		0		0	0
0		0		0	0
0		0		0	0
0		0		0	0
0		0		0	0
0		0		0	0
0		0		0	0
0		0		0	0
0		0		0	0
0		0		0	0
0		0		0	0
0		0		0	0
0	Ew/a-GOST-ci	1	ONLY_E-w/a-(	1	0
0		0		0	0
0		0		0	0
0		0		0	0
1		0		0	0
0		0		0	0
0		0		0	0

0		0		0	0
0		0		0	0
0	Ew/a-GOST-ci	1	ONLY_E-w/a-(	1	0
0	Ew/a-GOST-ci	1	ONLY_E-w/a-(	1	0
0		0		0	0
0		0		0	0
0		0		0	0
1	Ew/a-GOST-ci	1		0	1
1	Ew/a-GOST-ci	1		0	1
0		0		0	0
0		0		0	0
0	Ew/a-GOST-ci	1	ONLY_E-w/a-(	1	0
0	Ew/a-GOST-ci	1	ONLY_E-w/a-(	1	0
0	Ew/a-GOST-ci	1	ONLY_E-w/a-(	1	0
0		0		0	0
0		0		0	0
0		0		0	0
0		0		0	0
0		0		0	0
0		0		0	0
0		0		0	0
0	Ew/a-GOST-ci	1	ONLY_E-w/a-(	1	0
0		0		0	0
0		0		0	0
0		0		0	0
0		0		0	0
0		0		0	0
0		0		0	0
0	Ew/a-GOST-ci	1	ONLY_E-w/a-(	1	0
0	Ew/a-GOST-ci	1	ONLY_E-w/a-(	1	0
0		0		0	0
0		0		0	0
0		0		0	0
0		0		0	0
0		0		0	0
0		0		0	0
0		0		0	0
0		0		0	1
0		0		0	0
0		0		0	0
0		0		0	0
0		0		0	0
0		0		0	0
0		0		0	0
0		0		0	0
0		0		0	0
0		0		0	0

0		0		0	0
0	Ew/a-GOST-ci	1	ONLY_E-w/a-(	1	0
0	Ew/a-GOST-ci	1	ONLY_E-w/a-(	1	0
0	Ew/a-GOST-ci	1	ONLY_E-w/a-(	1	0
0	Ew/a-GOST-ci	1	ONLY_E-w/a-(	1	0
0		0		0	0
0		0		0	0
0		0		0	0
0	Ew/a-GOST-ci	1	ONLY_E-w/a-(	1	0
0		0		0	0
0		0		0	0
1		0		0	1
1		0		0	1
0		0		0	0
0		0		0	0
0		0		0	0
0		0		0	0
0		0		0	0
0		0		0	0
1	Ew/a-GOST-ci	1		0	0
1	Ew/a-GOST-ci	1		0	0
0		0		0	0
0		0		0	0
0		0		0	0
0		0		0	0
0		0		0	0
0		0		0	0
0		0		0	0
0		0		0	0
1	Ew/a-GOST-ci	1		0	0
1	Ew/a-GOST-ci	1		0	0
0		0		0	0
0		0		0	0
0		0		0	0
0		0		0	0
0		0		0	0
0		0		0	0
0		0		0	0
0		0		0	0
0		0		0	0
0		0		0	0
0		0		0	0
0		0		0	0
1	Ew/a-GOST-ci	1		0	0
0		0		0	0
0		0		0	0
0		0		0	0

0		0		0	0
0		0		0	0
0		0		0	0
0		0		0	0
0		0		0	0
0		0		0	0
0		0		0	0
0		0		0	0
0		0		0	0
0		0		0	0
0	Ew/a-GOST-ci	1	ONLY_E-w/a-(	1	0
0	Ew/a-GOST-ci	1	ONLY_E-w/a-(	1	0
0	Ew/a-GOST-ci	1	ONLY_E-w/a-(	1	0
0		0		0	0
0		0		0	0
0		0		0	0
0		0		0	0
0		0		0	0
0		0		0	0
0		0		0	0
0		0		0	0
0		0		0	0
0		0		0	0
1	Ew/a-GOST-ci	1		0	1
1	Ew/a-GOST-ci	1		0	1
1	Ew/a-GOST-ci	1		0	1
0		0		0	0
0		0		0	0
0		0		0	0
0		0		0	0
0		0		0	0
0		0		0	0
0		0		0	0
0		0		0	0
0	Ew/a-GOST-ci	1	ONLY_E-w/a-(	1	0
0		0		0	0
0	Ew/a-GOST-ci	1	ONLY_E-w/a-(	1	0
0	Ew/a-GOST-ci	1	ONLY_E-w/a-(	1	0
0		0		0	0
0		0		0	0
0	Ew/a-GOST-ci	1	ONLY_E-w/a-(	1	0
0		0		0	0
0		0		0	0
0	Ew/a-GOST-ci	1	ONLY_E-w/a-(	1	0
0		0		0	0
0		0		0	0
0		0		0	0

0		0		0	0
0		0		0	0
0		0		0	0
0		0		0	0
0		0		0	0
0		0		0	0
0		0		0	0
0		0		0	0
0		0		0	0
0		0		0	0
1		0		0	1
1		0		0	1
1		0		0	1
0		0		0	0
0		0		0	0
0		0		0	0
0		0		0	0
0		0		0	0
0		0		0	0
0		0		0	0
0		0		0	0
0		0		0	0
0		0		0	0
0		0		0	0
0		0		0	0
0		0		0	0
0		0		0	0
0		0		0	0
0		0		0	0
0		0		0	0
0		0		0	0
0		0		0	0
0		0		0	0
0		0		0	0
0		0		0	0
0		0		0	0
0		0		0	0
0		0		0	0
0		0		0	0
0		0		0	0
0		0		0	0
0		0		0	0
0		0		0	0
0		0		0	0
0	Ew/a-GOST-ci	1	ONLY_E-w/a-[illegible]	1	0
0		0		0	0
0		0		0	0

0	Ew/a-GOST-ci	1	ONLY_E-w/a-(	1	0
0	Ew/a-GOST-ci	1	ONLY_E-w/a-(	1	0
0		0		0	0
0		0		0	0
0		0		0	0
0		0		0	0
0		0		0	0
0		0		0	0
0		0		0	0
0		0		0	0
0		0		0	0
0		0		0	0
0	Ew/a-GOST-ci	1	ONLY_E-w/a-(	1	0
0		0		0	0
0		0		0	0
0	Ew/a-GOST-ci	1	ONLY_E-w/a-(	1	0
0	Ew/a-GOST-ci	1	ONLY_E-w/a-(	1	0
0		0		0	0
0		0		0	0
0		0		0	0
0		0		0	0
0		0		0	0
0		0		0	0
0		0		0	0
0		0		0	0
0		0		0	0
0		0		0	0
0		0		0	0
0		0		0	0
0	Ew/a-GOST-ci	1	ONLY_E-w/a-(	1	1
0		0		0	0
0		0		0	0
0		0		0	0
0		0		0	0
0		0		0	0
0		0		0	0
0		0		0	0
0	Ew/a-GOST-ci	1	ONLY_E-w/a-(	1	0
0	Ew/a-GOST-ci	1	ONLY_E-w/a-(	1	0
0		0		0	0
0		0		0	0
0		0		0	0
0		0		0	0
0		0		0	0
0		0		0	0
0		0		0	0
0		0		0	0

0		0		0	0
0		0		0	0
0	Ew/a-GOST-ci	1	ONLY_E-w/a-(	1	0
0	Ew/a-GOST-ci	1	ONLY_E-w/a-(	1	0
0	Ew/a-GOST-ci	1	ONLY_E-w/a-(	1	0
0		0		0	0
1	Ew/a-GOST-ci	1		0	1
1	Ew/a-GOST-ci	1		0	1
1	Ew/a-GOST-ci	1		0	1
1	Ew/a-GOST-ci	1		0	1
0		0		0	0
0		0		0	0
0		0		0	0
0		0		0	0
0		0		0	0
0		0		0	0
0		0		0	0
0		0		0	0
0		0		0	0
1		0		0	0
1		0		0	0
0	Ew/a-GOST-ci	1	ONLY_E-w/a-(	1	0
0	Ew/a-GOST-ci	1	ONLY_E-w/a-(	1	0
0	Ew/a-GOST-ci	1	ONLY_E-w/a-(	1	0
0	Ew/a-GOST-ci	1	ONLY_E-w/a-(	1	0
0	Ew/a-GOST-ci	1	ONLY_E-w/a-(	1	0
0		0		0	0
0		0		0	0
0		0		0	0
0		0		0	0
0		0		0	0
0		0		0	0
0		0		0	0
0		0		0	0
0		0		0	0
0		0		0	0
1	Ew/a-GOST-ci	1		0	0
1	Ew/a-GOST-ci	1		0	0
0		0		0	0
0		0		0	0
0		0		0	0
0		0		0	0
0		0		0	0
0		0		0	0
0		0		0	0
0		0		0	0
0		0		0	0

0		0		0	0
0		0		0	0
0		0		0	0
0		0		0	0
0		0		0	0
0		0		0	0
0		0		0	0
0		0		0	0
0		0		0	0
0		0		0	0
0		0		0	0
0		0		0	0
0		0		0	0
0		0		0	0
0	Ew/a-GOST-ci	1	ONLY_E-w/a-(	1	0
0	Ew/a-GOST-ci	1	ONLY_E-w/a-(	1	0
0		0		0	0
0		0		0	0
0		0		0	0
1	Ew/a-GOST-ci	1		0	0
1		0		0	0
1		0		0	0
0		0		0	0
0		0		0	0
0		0		0	0
0		0		0	0
0		0		0	0
0	Ew/a-GOST-ci	1	ONLY_E-w/a-(	1	0
0		0		0	0
0		0		0	0
1		0		0	0
1		0		0	0
0		0		0	0
0	Ew/a-GOST-ci	1	ONLY_E-w/a-(	1	0
0	Ew/a-GOST-ci	1	ONLY_E-w/a-(	1	0
0	Ew/a-GOST-ci	1	ONLY_E-w/a-(	1	0
0	Ew/a-GOST-ci	1	ONLY_E-w/a-(	1	0
0		0		0	0
0		0		0	0
0		0		0	0
0		0		0	0
0		0		0	0
0		0		0	1
0		0		0	1
0		0		0	0
0		0		0	0
0		0		0	0

0		0		0	0
0		0		0	0
0		0		0	0
0		0		0	0
0		0		0	0
0		0		0	0
0		0		0	0
0		0		0	0
1	Ew/a-GOST-ci	1		0	0
0		0		0	0
0		0		0	0
0		0		0	0
0		0		0	0
0		0		0	0
0		0		0	0
0	Ew/a-GOST-ci	1	ONLY_E-w/a-(	1	0
0		0		0	0
0		0		0	0
0		0		0	0
0	Ew/a-GOST-ci	1	ONLY_E-w/a-(	1	0
0	Ew/a-GOST-ci	1	ONLY_E-w/a-(	1	0
1	Ew/a-GOST-ci	1		0	1
1	Ew/a-GOST-ci	1		0	1
0		0		0	0
0		0		0	0
0		0		0	0
0		0		0	0
0		0		0	0
0	Ew/a-GOST-ci	1	ONLY_E-w/a-(	1	0
0	Ew/a-GOST-ci	1	ONLY_E-w/a-(	1	1
0	Ew/a-GOST-ci	1	ONLY_E-w/a-(	1	1
0	Ew/a-GOST-ci	1	ONLY_E-w/a-(	1	0
0	Ew/a-GOST-ci	1	ONLY_E-w/a-(	1	0
0		0		0	0
0		0		0	0
0		0		0	0
0		0		0	0
0		0		0	0
1		0		0	0
1		0		0	0
0		0		0	0
0		0		0	0
0		0		0	0
0		0		0	0
0		0		0	0
0		0		0	0
0		0		0	0

0		0		0	1
0		0		0	0
0		0		0	0
0		0		0	0
0		0		0	0
0		0		0	0
0		0		0	0
0		0		0	0
0		0		0	0
0		0		0	0
0		0		0	0
0		0		0	0
0		0		0	0
0		0		0	0
0		0		0	0
0		0		0	0
0		0		0	0
0		0		0	0
0		0		0	0
0		0		0	0
0		0		0	0
0		0		0	0
0		0		0	0
0		0		0	0
0		0		0	0
0		0		0	0
0		0		0	0
0		0		0	0
0		0		0	0
0		0		0	0
0		0		0	0
0		0		0	0
0		0		0	0
0		0		0	0
0		0		0	0
0		0		0	0
0		0		0	0
0		0		0	0
0		0		0	0
0		0		0	0
0		0		0	0
0		0		0	0
0		0		0	0
0		0		0	0
0		0		0	0
0	Ew/a-GOST-ci	1	ONLY_E-w/a-(	1	0
0		0		0	0

0		0		0	0
0		0		0	0
0		0		0	0
0	Ew/a-GOST-ci	1	ONLY_E-w/a-(	1	0
0		0		0	0
1	Ew/a-GOST-ci	1		0	0
1	Ew/a-GOST-ci	1		0	0
1	Ew/a-GOST-ci	1		0	0
0		0		0	0
0		0		0	0
0		0		0	0
0		0		0	0
0		0		0	0
0		0		0	0
0		0		0	0
0		0		0	0
0		0		0	0
0		0		0	0
0		0		0	0
0	Ew/a-GOST-ci	1	ONLY_E-w/a-(	1	1
0	Ew/a-GOST-ci	1	ONLY_E-w/a-(	1	1
0	Ew/a-GOST-ci	1	ONLY_E-w/a-(	1	1
0	Ew/a-GOST-ci	1	ONLY_E-w/a-(	1	1
0	Ew/a-GOST-ci	1	ONLY_E-w/a-(	1	1
0		0		0	0
0		0		0	0
1	Ew/a-GOST-ci	1		0	1
1	Ew/a-GOST-ci	1		0	1
1	Ew/a-GOST-ci	1		0	1
0		0		0	0
0		0		0	0
0		0		0	0
0	Ew/a-GOST-ci	1	ONLY_E-w/a-(	1	0
0		0		0	0
0		0		0	0
0		0		0	0
1	Ew/a-GOST-ci	1		0	0
1	Ew/a-GOST-ci	1		0	0
0		0		0	0
0	Ew/a-GOST-ci	1	ONLY_E-w/a-(	1	0
0	Ew/a-GOST-ci	1	ONLY_E-w/a-(	1	0
0	Ew/a-GOST-ci	1	ONLY_E-w/a-(	1	0
0	Ew/a-GOST-ci	1	ONLY_E-w/a-(	1	0
0		0		0	0
0		0		0	0
0		0		0	0
0		0		0	0

0		0		0	0
0		0		0	0
0		0		0	0
0		0		0	0
0		0		0	0
0		0		0	0
0		0		0	0
0	Ew/a-GOST-ci	1	ONLY_E-w/a-(	1	0
0		0		0	0
0		0		0	0
1		0		0	0
0		0		0	0
0		0		0	0
0	Ew/a-GOST-ci	1	ONLY_E-w/a-(	1	0
0		0		0	0
0		0		0	0
0		0		0	0
0		0		0	0
0		0		0	0
0		0		0	0
0	Ew/a-GOST-ci	1	ONLY_E-w/a-(	1	0
0	Ew/a-GOST-ci	1	ONLY_E-w/a-(	1	0
0	Ew/a-GOST-ci	1	ONLY_E-w/a-(	1	0
0	Ew/a-GOST-ci	1	ONLY_E-w/a-(	1	0
1		0		0	0
0		0		0	0
0		0		0	0
0		0		0	0
0		0		0	0
0		0		0	0
0		0		0	0
0		0		0	0
0		0		0	0
0		0		0	0
0	Ew/a-GOST-ci	1	ONLY_E-w/a-(	1	0
0		0		0	0
0		0		0	0
0		0		0	0
0		0		0	0
0		0		0	0
0		0		0	0
0		0		0	0
0		0		0	0
0		0		0	1
0		0		0	1
0		0		0	0
0		0		0	0

0		0		0	0
0		0		0	0
0		0		0	0
0		0		0	0
0		0		0	0
1		0		0	0
0		0		0	0
0		0		0	0
0		0		0	0
0		0		0	0
0		0		0	0
0	Ew/a-GOST-ci	1	ONLY_E-w/a-(	1	0
0	Ew/a-GOST-ci	1	ONLY_E-w/a-(	1	0
0	Ew/a-GOST-ci	1	ONLY_E-w/a-(	1	0
0		0		0	0
0		0		0	0
0		0		0	0
0		0		0	0
0		0		0	0
0		0		0	0
0		0		0	0
0		0		0	0
0		0		0	0
0		0		0	0
0	Ew/a-GOST-ci	1	ONLY_E-w/a-(	1	0
0		0		0	0
0		0		0	0
0		0		0	0
0		0		0	0
0		0		0	0
0		0		0	0
0		0		0	0
0		0		0	0
0		0		0	0
0		0		0	0
0	Ew/a-GOST-ci	1	ONLY_E-w/a-(	1	0
0	Ew/a-GOST-ci	1	ONLY_E-w/a-(	1	0
0		0		0	0
1	Ew/a-GOST-ci	1		0	1
1	Ew/a-GOST-ci	1		0	1
0		0		0	0
0		0		0	0
0		0		0	0
0		0		0	0
0		0		0	0
0		0		0	0
0		0		0	0

0		0		0	0
0		0		0	0
0		0		0	0
0		0		0	0
0		0		0	0
0		0		0	0
0		0		0	0
0		0		0	0
0		0		0	1
0		0		0	1
0		0		0	1
0		0		0	0
0		0		0	0
0	Ew/a-GOST-ci	1	ONLY_E-w/a-(	1	0
0	Ew/a-GOST-ci	1	ONLY_E-w/a-(	1	0
0		0		0	0
0		0		0	0
0		0		0	0
0		0		0	0
0		0		0	0
0		0		0	0
0	Ew/a-GOST-ci	1	ONLY_E-w/a-(	1	0
0		0		0	0
0		0		0	0
0		0		0	0
0		0		0	0
0		0		0	0
0		0		0	0
0		0		0	0
0	Ew/a-GOST-ci	1	ONLY_E-w/a-(	1	0
0	Ew/a-GOST-ci	1	ONLY_E-w/a-(	1	0
0		0		0	0
0		0		0	0
0		0		0	0
0		0		0	0
0		0		0	0
0		0		0	0
0		0		0	0
0		0		0	0
0		0		0	0
0		0		0	0
0		0		0	0
0		0		0	0
1	Ew/a-GOST-ci	1		0	1
1	Ew/a-GOST-ci	1		0	1
1	Ew/a-GOST-ci	1		0	1
1		0		0	0

1		0		0	0
0	Ew/a-GOST-ci	1	ONLY_E-w/a-(	1	0
0	Ew/a-GOST-ci	1	ONLY_E-w/a-(	1	0
0		0		0	0
0		0		0	0
0		0		0	0
0		0		0	0
0	Ew/a-GOST-ci	1	ONLY_E-w/a-(	1	0
0	Ew/a-GOST-ci	1	ONLY_E-w/a-(	1	0
1		0		0	0
0		0		0	0
0	Ew/a-GOST-ci	1	ONLY_E-w/a-(	1	1
0	Ew/a-GOST-ci	1	ONLY_E-w/a-(	1	1
0	Ew/a-GOST-ci	1	ONLY_E-w/a-(	1	1
0		0		0	0
0		0		0	0
0		0		0	0
0		0		0	0
0		0		0	0
0		0		0	0
0		0		0	0
0		0		0	0
0		0		0	0
0		0		0	0
1	Ew/a-GOST-ci	1		0	0
1	Ew/a-GOST-ci	1		0	0
1	Ew/a-GOST-ci	1		0	0
1	Ew/a-GOST-ci	1		0	0
0		0		0	0
0	Ew/a-GOST-ci	1	ONLY_E-w/a-(	1	0
0		0		0	0
0		0		0	1
0		0		0	1
0		0		0	1
0		0		0	0
0		0		0	0
0		0		0	0
0		0		0	0
0		0		0	0
0		0		0	0
0		0		0	0
0		0		0	0
0		0		0	0
0		0		0	0
0		0		0	0
0		0		0	0
0		0		0	0

1		0		0	0
0		0		0	0
0		0		0	0
0		0		0	0
1	Ew/a-GOST-ci	1		0	0
1	Ew/a-GOST-ci	1		0	0
0		0		0	0
0		0		0	0
0		0		0	0
0		0		0	0
0		0		0	0
0		0		0	0
0	Ew/a-GOST-ci	1	ONLY_E-w/a-(	1	0
0	Ew/a-GOST-ci	1	ONLY_E-w/a-(	1	0
0	Ew/a-GOST-ci	1	ONLY_E-w/a-(	1	0
0		0		0	0
0		0		0	0
0		0		0	0
0	Ew/a-GOST-ci	1	ONLY_E-w/a-(	1	0
0	Ew/a-GOST-ci	1	ONLY_E-w/a-(	1	0
0	Ew/a-GOST-ci	1	ONLY_E-w/a-(	1	0
0		0		0	0
0		0		0	0
0		0		0	0
0		0		0	0
0	Ew/a-GOST-ci	1	ONLY_E-w/a-(	1	0
0		0		0	0
0		0		0	0
0		0		0	0
0		0		0	0
0		0		0	0
0		0		0	0
0		0		0	0
0		0		0	0
0	Ew/a-GOST-ci	1	ONLY_E-w/a-(	1	0
0	Ew/a-GOST-ci	1	ONLY_E-w/a-(	1	0
0		0		0	0
0		0		0	0
0		0		0	0
0		0		0	0
0		0		0	0
0		0		0	0
0		0		0	0
0		0		0	0
0		0		0	0
0		0		0	0
0	Ew/a-GOST-ci	1	ONLY_E-w/a-(	1	0

0		0		0	0
0		0		0	0
0		0		0	0
0		0		0	0
0		0		0	0
0		0		0	0
0		0		0	0
1	Ew/a-GOST-ci	1		0	1
1	Ew/a-GOST-ci	1		0	1
0	Ew/a-GOST-ci	1	ONLY_E-w/a-(	1	0
0	Ew/a-GOST-ci	1	ONLY_E-w/a-(	1	0
0	Ew/a-GOST-ci	1	ONLY_E-w/a-(	1	0
0		0		0	0
0		0		0	0
0		0		0	0
1		0		0	1
0		0		0	0
0		0		0	0
0		0		0	0
0		0		0	0
0		0		0	0
0	Ew/a-GOST-ci	1	ONLY_E-w/a-(	1	0
1	Ew/a-GOST-ci	1		0	1
1	Ew/a-GOST-ci	1		0	1
1	Ew/a-GOST-ci	1		0	1
0		0		0	0
1	Ew/a-GOST-ci	1		0	0
0	Ew/a-GOST-ci	1	ONLY_E-w/a-(	1	0
1	Ew/a-GOST-ci	1		0	0
1	Ew/a-GOST-ci	1		0	0
0		0		0	0
1		0		0	0
1	Ew/a-GOST-ci	1		0	0
1	Ew/a-GOST-ci	1		0	0
0	Ew/a-GOST-ci	1	ONLY_E-w/a-(	1	0
0		0		0	0
0		0		0	0
0		0		0	0
0		0		0	0
0		0		0	0
0		0		0	0
0		0		0	0
0	Ew/a-GOST-ci	1	ONLY_E-w/a-(	1	0
0		0		0	0
0		0		0	0
0		0		0	0
0		0		0	0

0	Ew/a-GOST-ci	1	ONLY_E-w/a-(	1	1
0	Ew/a-GOST-ci	1	ONLY_E-w/a-(	1	1
0	Ew/a-GOST-ci	1	ONLY_E-w/a-(	1	1
0		0		0	0
0		0		0	0
1		0		0	0
0		0		0	0
0		0		0	0
0		0		0	0
1		0		0	1
1		0		0	1
1		0		0	1
0		0		0	0
0		0		0	0
0		0		0	0
0		0		0	0
0		0		0	0
0		0		0	0
0		0		0	0
0		0		0	0
0		0		0	0
0		0		0	0
0		0		0	0
0		0		0	0
0		0		0	0
0		0		0	0
0		0		0	0
0		0		0	0
1	Ew/a-GOST-ci	1		0	0
1	Ew/a-GOST-ci	1		0	0
0		0		0	0
1	Ew/a-GOST-ci	1		0	0
0		0		0	1
0		0		0	1
0		0		0	0
0		0		0	0
0		0		0	0
0		0		0	0
0		0		0	0
0		0		0	0
0		0		0	0
0		0		0	0
0		0		0	0
0		0		0	0
0		0		0	0
0		0		0	0
0		0		0	0

0	0	0	0
0	0	0	0
1	0	0	0
1	0	0	0
0	0	0	0
0	0	0	0
0	0	0	0
0	0	0	0
0	0	0	0
0	0	0	0
0	0	0	0
0	0	0	0
0	0	0	0
0	0	0	0
0	0	0	0
0	0	0	0
1 Ew/a-GOST-ci	1	0	0
1 Ew/a-GOST-ci	1	0	0
0	0	0	0
0	0	0	0
0	0	0	0
0	0	0	0
0	0	0	0
0	0	0	0
0	0	0	0
0	0	0	0
0	0	0	0
0	0	0	0
0	0	0	0
0	0	0	0
0	0	0	0
0	0	0	0
1	0	0	0
0	0	0	0
1	0	0	0
1	0	0	1
1	0	0	1
0	0	0	0
0	0	0	0
0	0	0	0
1 Ew/a-GOST-ci	1	0	1
1 Ew/a-GOST-ci	1	0	1
1 Ew/a-GOST-ci	1	0	1
1 Ew/a-GOST-ci	1	0	1
0	0	0	0
1 Ew/a-GOST-ci	1	0	0
1 Ew/a-GOST-ci	1	0	0

0		0		0	0
0		0		0	0
1		0		0	1
0		0		0	0
0		0		0	0
0		0		0	0
0		0		0	0
0		0		0	0
0		0		0	0
0		0		0	0
0		0		0	0
0		0		0	0
0	Ew/a-GOST-ci	1	ONLY_E-w/a-(	1	0
0		0		0	0
0		0		0	0
0	Ew/a-GOST-ci	1	ONLY_E-w/a-(	1	0
0	Ew/a-GOST-ci	1	ONLY_E-w/a-(	1	0
1	Ew/a-GOST-ci	1		0	0
1	Ew/a-GOST-ci	1		0	0
1	Ew/a-GOST-ci	1		0	0
0		0		0	0
0		0		0	0
0		0		0	0
0		0		0	0
1		0		0	1
0	Ew/a-GOST-ci	1	ONLY_E-w/a-(	1	0
1	Ew/a-GOST-ci	1		0	0
0		0		0	0
0		0		0	0
0		0		0	0
0		0		0	0
0		0		0	0
0		0		0	0
0	Ew/a-GOST-ci	1	ONLY_E-w/a-(	1	0
0	Ew/a-GOST-ci	1	ONLY_E-w/a-(	1	0
0	Ew/a-GOST-ci	1	ONLY_E-w/a-(	1	0
0		0		0	0
0	Ew/a-GOST-ci	1	ONLY_E-w/a-(	1	0
0	Ew/a-GOST-ci	1	ONLY_E-w/a-(	1	0
1		0		0	1
0		0		0	0
1		0		0	1
0		0		0	0
0		0		0	0
0		0		0	0
0		0		0	0
0		0		0	0

0	Ew/a-GOST-ci	1	ONLY_E-w/a-(	1	0
0		0		0	0
0	Ew/a-GOST-ci	1	ONLY_E-w/a-(	1	0
1	Ew/a-GOST-ci	1		0	0
1		0		0	0
1		0		0	0
0		0		0	0
0		0		0	0
0		0		0	0
0		0		0	0
0	Ew/a-GOST-ci	1	ONLY_E-w/a-(	1	0
0	Ew/a-GOST-ci	1	ONLY_E-w/a-(	1	0
0		0		0	0
0		0		0	0
1	Ew/a-GOST-ci	1		0	0
0		0		0	0
0		0		0	0
0		0		0	0
0		0		0	0
0	Ew/a-GOST-ci	1	ONLY_E-w/a-(	1	0
0	Ew/a-GOST-ci	1	ONLY_E-w/a-(	1	0
0	Ew/a-GOST-ci	1	ONLY_E-w/a-(	1	0
0	Ew/a-GOST-ci	1	ONLY_E-w/a-(	1	0
0	Ew/a-GOST-ci	1	ONLY_E-w/a-(	1	0
0		0		0	0
0	Ew/a-GOST-ci	1	ONLY_E-w/a-(	1	0
1	Ew/a-GOST-ci	1		0	0
0		0		0	0
0		0		0	0
0		0		0	0
0		0		0	0
0		0		0	0
0		0		0	0
0		0		0	0
0		0		0	0
0		0		0	0
0		0		0	0
0		0		0	0
0		0		0	0
0		0		0	0
0		0		0	0
0		0		0	0
0		0		0	0
0	Ew/a-GOST-ci	1	ONLY_E-w/a-(	1	0
0	Ew/a-GOST-ci	1	ONLY_E-w/a-(	1	0
0	Ew/a-GOST-ci	1	ONLY_E-w/a-(	1	0
0		0		0	0

0		0		0	0
0		0		0	0
0		0		0	0
0		0		0	0
0		0		0	0
0		0		0	0
0		0		0	0
0		0		0	0
0	Ew/a-GOST-ci	1	ONLY_E-w/a-(	1	0
0	Ew/a-GOST-ci	1	ONLY_E-w/a-(	1	0
0		0		0	0
0		0		0	0
0		0		0	0
0		0		0	0
0		0		0	0
0		0		0	0
0		0		0	0
0		0		0	0
0		0		0	0
0		0		0	0
0	Ew/a-GOST-ci	1	ONLY_E-w/a-(	1	0
0	Ew/a-GOST-ci	1	ONLY_E-w/a-(	1	0
0		0		0	0
0		0		0	0
0		0		0	0
0		0		0	0
0	Ew/a-GOST-ci	1	ONLY_E-w/a-(	1	0
0		0		0	0
0		0		0	0
0		0		0	0
0		0		0	0
1		0		0	0
1		0		0	0
1		0		0	0
0		0		0	0
0	Ew/a-GOST-ci	1	ONLY_E-w/a-(	1	0
0		0		0	0
0		0		0	0
0		0		0	0
0		0		0	0
0		0		0	0
0		0		0	0
0		0		0	0
0		0		0	0
0		0		0	0
0		0		0	0
1	Ew/a-GOST-ci	1		0	0

1	Ew/a-GOST-ci	1		0	0
1	Ew/a-GOST-ci	1		0	0
0	Ew/a-GOST-ci	1	ONLY_E-w/a-(	1	0
0	Ew/a-GOST-ci	1	ONLY_E-w/a-(	1	0
0	Ew/a-GOST-ci	1	ONLY_E-w/a-(	1	0
0		0		0	0
0		0		0	0
0		0		0	0
0		0		0	0
0		0		0	0
0	Ew/a-GOST-ci	1	ONLY_E-w/a-(	1	0
0	Ew/a-GOST-ci	1	ONLY_E-w/a-(	1	0
0	Ew/a-GOST-ci	1	ONLY_E-w/a-(	1	0
0		0		0	0
0		0		0	0
0		0		0	0
0		0		0	0
0		0		0	0
0		0		0	0
0		0		0	0
0		0		0	0
1	Ew/a-GOST-ci	1		0	0
1	Ew/a-GOST-ci	1		0	0
0		0		0	0
0		0		0	0
0		0		0	0
1	Ew/a-GOST-ci	1		0	0
1	Ew/a-GOST-ci	1		0	0
0		0		0	0
0		0		0	0
0		0		0	0
0		0		0	0
1	Ew/a-GOST-ci	1		0	1
0	Ew/a-GOST-ci	1	ONLY_E-w/a-(	1	0
0		0		0	0
0		0		0	0
0		0		0	0
0		0		0	0
0		0		0	0
0		0		0	0
0		0		0	0
0		0		0	0
0		0		0	0
0		0		0	0
0		0		0	0
0		0		0	0
0		0		0	0

0		0		0	0
0		0		0	0
0		0		0	0
0		0		0	0
0		0		0	0
0	Ew/a-GOST-ci	1	ONLY_E-w/a-(	1	0
0		0		0	0
0	Ew/a-GOST-ci	1	ONLY_E-w/a-(	1	0
0	Ew/a-GOST-ci	1	ONLY_E-w/a-(	1	0
1	Ew/a-GOST-ci	1		0	0
0		0		0	0
0		0		0	0
0		0		0	0
0	Ew/a-GOST-ci	1	ONLY_E-w/a-(	1	0
0	Ew/a-GOST-ci	1	ONLY_E-w/a-(	1	0
0	Ew/a-GOST-ci	1	ONLY_E-w/a-(	1	0
0	Ew/a-GOST-ci	1	ONLY_E-w/a-(	1	0
0		0		0	0
0		0		0	0
0	Ew/a-GOST-ci	1	ONLY_E-w/a-(	1	0
0	Ew/a-GOST-ci	1	ONLY_E-w/a-(	1	0
0		0		0	0
0		0		0	0
0		0		0	0
0		0		0	0
0		0		0	0
0		0		0	0
0		0		0	0
0		0		0	0
0	Ew/a-GOST-ci	1	ONLY_E-w/a-(	1	0
0	Ew/a-GOST-ci	1	ONLY_E-w/a-(	1	0
0		0		0	0
0		0		0	0
0		0		0	0
0		0		0	0
0		0		0	0
0		0		0	0
0		0		0	0
0		0		0	0
1		0		0	1
0		0		0	0
0		0		0	0
0		0		0	0
0		0		0	0
0		0		0	0
0		0		0	0
0		0		0	0

0		0		0	0
0		0		0	0
0		0		0	0
0		0		0	0
0		0		0	0
0		0		0	0
0		0		0	0
0		0		0	0
0		0		0	0
0		0		0	0
0		0		0	0
0		0		0	0
0		0		0	0
0		0		0	0
0		0		0	0
0		0		0	0
0		0		0	0
0		0		0	0
0		0		0	0
0		0		0	0
0		0		0	0
0		0		0	0
0		0		0	0
0		0		0	0
0		0		0	0
0		0		0	0
0		0		0	0
0		0		0	0
1	Ew/a-GOST-ci	1		0	0
1	Ew/a-GOST-ci	1		0	0
1	Ew/a-GOST-ci	1		0	0
1	Ew/a-GOST-ci	1		0	0
0		0		0	0
0		0		0	0
0		0		0	0
0	Ew/a-GOST-ci	1	ONLY_E-w/a-	1	0
0	Ew/a-GOST-ci	1	ONLY_E-w/a-	1	0
1		0		0	0
0		0		0	0
0		0		0	0
0		0		0	0
1	Ew/a-GOST-ci	1		0	1
1	Ew/a-GOST-ci	1		0	1
1	Ew/a-GOST-ci	1		0	1
1		0		0	0
1		0		0	0
0		0		0	0

0		0		0	0
0		0		0	0
0		0		0	0
0		0		0	0
0		0		0	0
0		0		0	0
0		0		0	0
0		0		0	0
0		0		0	0
0	Ew/a-GOST-ci	1	ONLY_E-w/a-(	1	0
1	Ew/a-GOST-ci	1		0	0
1	Ew/a-GOST-ci	1		0	0
0		0		0	0
0		0		0	0
0		0		0	0
0		0		0	0
1	Ew/a-GOST-ci	1		0	0
1	Ew/a-GOST-ci	1		0	0
0		0		0	0
0		0		0	0
0		0		0	0
0		0		0	0
0	Ew/a-GOST-ci	1	ONLY_E-w/a-(	1	0
0		0		0	0
0		0		0	0
0		0		0	0
0		0		0	0
0		0		0	0
0		0		0	0
1	Ew/a-GOST-ci	1		0	0
1	Ew/a-GOST-ci	1		0	0
0		0		0	0
0		0		0	0
0		0		0	0
0		0		0	0
0		0		0	0
0	Ew/a-GOST-ci	1	ONLY_E-w/a-(	1	0
0		0		0	0
1	Ew/a-GOST-ci	1		0	0
1	Ew/a-GOST-ci	1		0	0
0		0		0	0
0		0		0	0
0		0		0	0
0		0		0	0
0		0		0	0
0		0		0	0
0		0		0	0

0	0	0	0
0	0	0	0
0	0	0	0
1 Ew/a-GOST-ci	1	0	0
1 Ew/a-GOST-ci	1	0	0
1 Ew/a-GOST-ci	1	0	0
0	0	0	0
0	0	0	0
0	0	0	0
0	0	0	0
0	0	0	0
0	0	0	0
0	0	0	0
0	0	0	0
0	0	0	0
0	0	0	0
0	0	0	0
0	0	0	0
0	0	0	0
1 Ew/a-GOST-ci	1	0	1
0	0	0	0
0	0	0	0
0	0	0	0
0	0	0	0
0	0	0	0
0	0	0	0
0	0	0	0
0	0	0	0
0	0	0	0
0	0	0	0
0	0	0	0
0	0	0	0
0	0	0	0
0	0	0	0
0	0	0	0
0	0	0	0
0	0	0	0
0	0	0	0
0	0	0	0
0	0	0	0
0	0	0	0
0	0	0	0
0	0	0	0
0	0	0	0
0	0	0	0
0	0	0	0
0	0	0	0

0		0		0	0
0		0		0	0
0	Ew/a-GOST-ci	1	ONLY_E-w/a-(	1	0
1		0		0	0
1		0		0	0
1	Ew/a-GOST-ci	1		0	1
1	Ew/a-GOST-ci	1		0	1
0		0		0	0
0		0		0	0
0		0		0	0
0		0		0	0
0		0		0	0
0		0		0	0
0		0		0	0
0		0		0	0
0		0		0	0
0		0		0	0
0		0		0	0
0		0		0	0
0		0		0	0
0		0		0	0
0		0		0	0
0		0		0	0
0		0		0	0
0	Ew/a-GOST-ci	1	ONLY_E-w/a-(	1	0
0		0		0	0
0		0		0	0
1	Ew/a-GOST-ci	1		0	0
0		0		0	0
0	Ew/a-GOST-ci	1	ONLY_E-w/a-(	1	0
0		0		0	0
0		0		0	0
0		0		0	0
0		0		0	0
0		0		0	0
0		0		0	0
0		0		0	0
1	Ew/a-GOST-ci	1		0	0
1	Ew/a-GOST-ci	1		0	0
1	Ew/a-GOST-ci	1		0	0
0		0		0	0
0		0		0	0
0		0		0	0
0		0		0	0
0		0		0	0
0		0		0	0
0		0		0	0

0		0		0	0
0	Ew/a-GOST-ci	1	ONLY_E-w/a-(	1	0
0		0		0	0
0		0		0	0
0	Ew/a-GOST-ci	1	ONLY_E-w/a-(	1	0
0	Ew/a-GOST-ci	1	ONLY_E-w/a-(	1	0
0		0		0	0
0		0		0	0
0		0		0	0
0		0		0	0
0		0		0	0
0		0		0	0
1		0		0	0
1		0		0	0
1		0		0	0
0	Ew/a-GOST-ci	1	ONLY_E-w/a-(	1	0
1	Ew/a-GOST-ci	1		0	1
1	Ew/a-GOST-ci	1		0	1
1	Ew/a-GOST-ci	1		0	1
0		0		0	0
0		0		0	0
0		0		0	0
0		0		0	0
0		0		0	0
0		0		0	0
0		0		0	0
0		0		0	0
0	Ew/a-GOST-ci	1	ONLY_E-w/a-(	1	0
0	Ew/a-GOST-ci	1	ONLY_E-w/a-(	1	0
0	Ew/a-GOST-ci	1	ONLY_E-w/a-(	1	0
0		0		0	0
0		0		0	0
0		0		0	0
1		0		0	0
1		0		0	0
1		0		0	0
1		0		0	0
0		0		0	0
0		0		0	0
0		0		0	0
1		0		0	0
1		0		0	0
0		0		0	0
0		0		0	0
0		0		0	0
0		0		0	0
0		0		0	0

0		0		0	0
0		0		0	0
0		0		0	0
0		0		0	0
0		0		0	0
0		0		0	0
0		0		0	0
0	Ew/a-GOST-ci	1	ONLY_E-w/a-(	1	0
0		0		0	0
0		0		0	0
0		0		0	0
0	Ew/a-GOST-ci	1	ONLY_E-w/a-(	1	0
0		0		0	0
0		0		0	0
0		0		0	0
0		0		0	0
0		0		0	0
0		0		0	0
0		0		0	0
0		0		0	0
1		0		0	0
1		0		0	0
0	Ew/a-GOST-ci	1	ONLY_E-w/a-(	1	0
0	Ew/a-GOST-ci	1	ONLY_E-w/a-(	1	0
0		0		0	0
0		0		0	0
0		0		0	0
1		0		0	0
0		0		0	0
0		0		0	0
0		0		0	0
0		0		0	0
0		0		0	0
0		0		0	0
0		0		0	0
0		0		0	0
0		0		0	0
0		0		0	0
0		0		0	0
0		0		0	0
0	Ew/a-GOST-ci	1	ONLY_E-w/a-(	1	0
0		0		0	0
0		0		0	0
0		0		0	0
0		0		0	0
1	Ew/a-GOST-ci	1		0	1
1	Ew/a-GOST-ci	1		0	1

1	Ew/a-GOST-ci	1		0	1
0		0		0	0
0		0		0	0
0		0		0	0
0		0		0	0
0		0		0	0
0		0		0	0
0		0		0	0
0		0		0	0
0		0		0	0
0		0		0	0
0		0		0	1
0		0		0	0
1		0		0	0
1		0		0	0
1	Ew/a-GOST-ci	1		0	0
1	Ew/a-GOST-ci	1		0	0
0		0		0	0
0		0		0	0
0	Ew/a-GOST-ci	1	ONLY_E-w/a-(	1	0
0	Ew/a-GOST-ci	1	ONLY_E-w/a-(	1	0
1		0		0	0
0		0		0	0
0		0		0	0
0		0		0	0
1	Ew/a-GOST-ci	1		0	0
1	Ew/a-GOST-ci	1		0	0
0		0		0	0
0		0		0	0
0		0		0	0
0		0		0	0
0		0		0	0
1	Ew/a-GOST-ci	1		0	0
0		0		0	0
0		0		0	0
0	Ew/a-GOST-ci	1	ONLY_E-w/a-(	1	0
0	Ew/a-GOST-ci	1	ONLY_E-w/a-(	1	0
0		0		0	0
0		0		0	0
1	Ew/a-GOST-ci	1		0	0
0		0		0	0
0		0		0	0
0		0		0	0
0		0		0	0
0	Ew/a-GOST-ci	1	ONLY_E-w/a-(	1	0
0		0		0	0
0		0		0	0

1	Ew/a-GOST-ci	1		0	0
1	Ew/a-GOST-ci	1		0	0
0		0		0	0
0		0		0	0
0		0		0	0
0		0		0	0
0		0		0	0
0		0		0	0
0		0		0	0
0		0		0	0
0		0		0	0
0		0		0	0
0		0		0	0
0		0		0	0
1	Ew/a-GOST-ci	1		0	0
0		0		0	0
0		0		0	0
0	Ew/a-GOST-ci	1	ONLY_E-w/a-	1	0
0	Ew/a-GOST-ci	1	ONLY_E-w/a-	1	0
0		0		0	0
0		0		0	0
0		0		0	0
0		0		0	0
0		0		0	0
1	Ew/a-GOST-ci	1		0	0
1	Ew/a-GOST-ci	1		0	0
0		0		0	0
0		0		0	0
0		0		0	0
0	Ew/a-GOST-ci	1	ONLY_E-w/a-	1	0
0		0		0	0
0		0		0	0
0		0		0	0
0	Ew/a-GOST-ci	1	ONLY_E-w/a-	1	0
0		0		0	0
0		0		0	0
0		0		0	0
0		0		0	0
0		0		0	0
0		0		0	0
0		0		0	0
0		0		0	0
0		0		0	0
0		0		0	0
0		0		0	0
0		0		0	0
0		0		0	0

0		0		0	0
0		0		0	0
0		0		0	0
0		0		0	0
0		0		0	0
0		0		0	0
0		0		0	0
0		0		0	0
0		0		0	0
0		0		0	0
1	Ew/a-GOST-ci	1		0	0
1	Ew/a-GOST-ci	1		0	0
0		0		0	0
0		0		0	0
0		0		0	0
0		0		0	0
0		0		0	0
0		0		0	0
0		0		0	0
0		0		0	0
0		0		0	0
0		0		0	0
1		0		0	0
0		0		0	0
0		0		0	1
0		0		0	0
0		0		0	0
0		0		0	0
0		0		0	0
0		0		0	0
0		0		0	0
0		0		0	0
1		0		0	0
0		0		0	0
1		0		0	0
0		0		0	0
0		0		0	0
0	Ew/a-GOST-ci	1	ONLY_E-w/a-(	1	0
0		0		0	0
0		0		0	0
1	Ew/a-GOST-ci	1		0	0
1	Ew/a-GOST-ci	1		0	0
1		0		0	1
1		0		0	1
1		0		0	1
1	Ew/a-GOST-ci	1		0	0
1	Ew/a-GOST-ci	1		0	0

1	Ew/a-GOST-ci	1		0	0
1	Ew/a-GOST-ci	1		0	0
1	Ew/a-GOST-ci	1		0	0
0		0		0	0
0		0		0	0
0	Ew/a-GOST-ci	1	ONLY_E-w/a-(	1	0
0		0		0	0
0		0		0	0
0		0		0	0
0		0		0	0
0		0		0	0
0		0		0	0
0		0		0	0
0		0		0	0
0		0		0	0
0		0		0	0
0	Ew/a-GOST-ci	1	ONLY_E-w/a-(	1	0
1		0		0	0
0		0		0	0
0		0		0	0
0		0		0	0
0		0		0	0
0		0		0	0
0		0		0	0
0		0		0	0
0		0		0	0
1	Ew/a-GOST-ci	1		0	0
0		0		0	0
0		0		0	0
0	Ew/a-GOST-ci	1	ONLY_E-w/a-(	1	0
0		0		0	0
0	Ew/a-GOST-ci	1	ONLY_E-w/a-(	1	0
0	Ew/a-GOST-ci	1	ONLY_E-w/a-(	1	0
0	Ew/a-GOST-ci	1	ONLY_E-w/a-(	1	0
0	Ew/a-GOST-ci	1	ONLY_E-w/a-(	1	0
0		0		0	0
0		0		0	0
0		0		0	0
0		0		0	0
0		0		0	0
0		0		0	0
0		0		0	0
0		0		0	0
0		0		0	0
0		0		0	0
0		0		0	0
0		0		0	0

0		0		0	0
0		0		0	0
1		0		0	1
1		0		0	1
1		0		0	1
1		0		0	1
0	Ew/a-GOST-ci	1	ONLY_E-w/a-(	1	1
0		0		0	0
0		0		0	0
0		0		0	0
0		0		0	0
0		0		0	0
0		0		0	0
1	Ew/a-GOST-ci	1		0	0
1	Ew/a-GOST-ci	1		0	0
1	Ew/a-GOST-ci	1		0	0
0		0		0	0
0		0		0	0
0		0		0	0
0		0		0	0
0		0		0	0
0		0		0	0
0		0		0	0
0		0		0	0
0		0		0	0
0		0		0	0
0		0		0	0
0		0		0	0
0	Ew/a-GOST-ci	1	ONLY_E-w/a-(	1	0
0	Ew/a-GOST-ci	1	ONLY_E-w/a-(	1	0
0		0		0	0
1	Ew/a-GOST-ci	1		0	0
0		0		0	1
0		0		0	1
0		0		0	1
0		0		0	0
0		0		0	0
0		0		0	0
0		0		0	0
0		0		0	0
0		0		0	0
0		0		0	0
0		0		0	0
0		0		0	0
0		0		0	0
0		0		0	0
0		0		0	0

0		0		0	0
0		0		0	0
0	Ew/a-GOST-ci	1	ONLY_E-w/a-(	1	0
0	Ew/a-GOST-ci	1	ONLY_E-w/a-(	1	0
0	Ew/a-GOST-ci	1	ONLY_E-w/a-(	1	0
1	Ew/a-GOST-ci	1		0	1
1	Ew/a-GOST-ci	1		0	1
1	Ew/a-GOST-ci	1		0	1
0		0		0	0
0		0		0	0
0		0		0	0
0		0		0	0
0		0		0	0
0		0		0	0
0	Ew/a-GOST-ci	1	ONLY_E-w/a-(	1	0
0	Ew/a-GOST-ci	1	ONLY_E-w/a-(	1	0
0		0		0	0
0	Ew/a-GOST-ci	1	ONLY_E-w/a-(	1	0
0		0		0	0
0		0		0	0
0		0		0	0
0		0		0	0
0		0		0	0
0		0		0	0
0		0		0	0
1		0		0	1
1		0		0	1
1		0		0	1
1		0		0	1
1		0		0	1
0		0		0	0
1	Ew/a-GOST-ci	1		0	0
1	Ew/a-GOST-ci	1		0	0
0		0		0	0
0		0		0	0
0		0		0	0
0		0		0	0
0		0		0	0
0		0		0	0
0		0		0	0
0	Ew/a-GOST-ci	1	ONLY_E-w/a-(	1	0
0		0		0	0
0		0		0	0
0		0		0	0
0		0		0	0
0		0		0	0
0		0		0	0

0		0		0	0
0	Ew/a-GOST-ci	1	ONLY_E-w/a-(	1	0
0		0		0	0
0		0		0	0
0		0		0	0
0		0		0	0
0		0		0	0
1		0		0	0
1		0		0	0
0		0		0	0
0		0		0	0
0	Ew/a-GOST-ci	1	ONLY_E-w/a-(	1	0
0		0		0	0
0		0		0	0
0		0		0	0
0		0		0	0
1		0		0	0
0	Ew/a-GOST-ci	1	ONLY_E-w/a-(	1	0
0	Ew/a-GOST-ci	1	ONLY_E-w/a-(	1	0
0	Ew/a-GOST-ci	1	ONLY_E-w/a-(	1	0
0	Ew/a-GOST-ci	1	ONLY_E-w/a-(	1	0
0		0		0	0
0		0		0	0
0		0		0	0
0		0		0	0
0	Ew/a-GOST-ci	1	ONLY_E-w/a-(	1	0
0		0		0	0
0		0		0	0
0		0		0	1
0		0		0	1
1	Ew/a-GOST-ci	1		0	0
1	Ew/a-GOST-ci	1		0	0
0		0		0	0
0		0		0	0
0		0		0	0
0		0		0	0
0		0		0	0
0		0		0	0
0		0		0	0
0		0		0	0
0		0		0	0
0		0		0	0
0		0		0	0
0	Ew/a-GOST-ci	1	ONLY_E-w/a-(	1	0
1	Ew/a-GOST-ci	1		0	0
1	Ew/a-GOST-ci	1		0	0
0		0		0	0

0		0		0	0
0		0		0	0
0		0		0	0
0		0		0	0
0		0		0	0
0		0		0	0
0	Ew/a-GOST-ci	1	ONLY_E-w/a-(	1	0
0	Ew/a-GOST-ci	1	ONLY_E-w/a-(	1	0
0		0		0	0
0		0		0	0
1	Ew/a-GOST-ci	1		0	0
0		0		0	0
0		0		0	0
0		0		0	0
0		0		0	0
0		0		0	0
0		0		0	0
0		0		0	0
0		0		0	0
0		0		0	0
0		0		0	0
0	Ew/a-GOST-ci	1	ONLY_E-w/a-(	1	0
0	Ew/a-GOST-ci	1	ONLY_E-w/a-(	1	0
0	Ew/a-GOST-ci	1	ONLY_E-w/a-(	1	0
0		0		0	1
0	Ew/a-GOST-ci	1	ONLY_E-w/a-(	1	0
0	Ew/a-GOST-ci	1	ONLY_E-w/a-(	1	0
0		0		0	0
0		0		0	0
0		0		0	0
0		0		0	0
0		0		0	0
0		0		0	0
0		0		0	0
0		0		0	0
0		0		0	0
0		0		0	0
0		0		0	0
0		0		0	0
0		0		0	0
1		0		0	0
1		0		0	0
1	Ew/a-GOST-ci	1		0	0
0		0		0	0
0		0		0	0
0		0		0	0
0		0		0	1

0		0		0	0
0		0		0	0
0		0		0	0
0		0		0	0
0		0		0	0
0		0		0	0
0	Ew/a-GOST-ci	1	ONLY_E-w/a-(	1	0
0	Ew/a-GOST-ci	1	ONLY_E-w/a-(	1	0
0	Ew/a-GOST-ci	1	ONLY_E-w/a-(	1	0
0	Ew/a-GOST-ci	1	ONLY_E-w/a-(	1	0
0		0		0	0
0	Ew/a-GOST-ci	1	ONLY_E-w/a-(	1	0
0		0		0	0
0		0		0	0
0		0		0	0
0		0		0	0
0	Ew/a-GOST-ci	1	ONLY_E-w/a-(	1	0
0		0		0	0
0		0		0	0
0		0		0	0
0		0		0	0
0		0		0	0
0		0		0	0
0		0		0	0
0		0		0	0
0		0		0	0
0		0		0	0
0		0		0	0
0		0		0	0
1	Ew/a-GOST-ci	1		0	1
1	Ew/a-GOST-ci	1		0	1
0		0		0	0
0		0		0	0
0	Ew/a-GOST-ci	1	ONLY_E-w/a-(	1	0
0	Ew/a-GOST-ci	1	ONLY_E-w/a-(	1	0
0		0		0	0
0		0		0	0
0		0		0	0
0		0		0	0
0	Ew/a-GOST-ci	1	ONLY_E-w/a-(	1	0
0	Ew/a-GOST-ci	1	ONLY_E-w/a-(	1	0
0	Ew/a-GOST-ci	1	ONLY_E-w/a-(	1	0
0		0		0	0
0	Ew/a-GOST-ci	1	ONLY_E-w/a-(	1	0
0	Ew/a-GOST-ci	1	ONLY_E-w/a-(	1	0
0	Ew/a-GOST-ci	1	ONLY_E-w/a-(	1	0
0		0		0	0

0	Ew/a-GOST-ci	1	ONLY_E-w/a-(	1	0
0		0		0	0
0		0		0	0
0		0		0	0
0		0		0	0
0		0		0	0
0		0		0	0
0		0		0	0
0		0		0	0
0		0		0	0
0		0		0	0
0		0		0	0
0		0		0	0
0		0		0	0
0	Ew/a-GOST-ci	1	ONLY_E-w/a-(	1	0
0		0		0	0
0		0		0	0
0		0		0	0
0		0		0	0
0		0		0	0
0		0		0	0
0		0		0	0
0		0		0	0
0	Ew/a-GOST-ci	1	ONLY_E-w/a-(	1	0
0		0		0	0
0		0		0	0
0		0		0	0
0		0		0	0
0		0		0	0
0		0		0	0
0	Ew/a-GOST-ci	1	ONLY_E-w/a-(	1	0
0		0		0	0
0		0		0	0
0	Ew/a-GOST-ci	1	ONLY_E-w/a-(	1	0
0	Ew/a-GOST-ci	1	ONLY_E-w/a-(	1	0
0		0		0	0
0		0		0	0
0		0		0	0
0		0		0	0
0	Ew/a-GOST-ci	1	ONLY_E-w/a-(	1	0
0	Ew/a-GOST-ci	1	ONLY_E-w/a-(	1	0
0		0		0	0
0		0		0	0
0		0		0	0
0		0		0	0
0		0		0	0
0		0		0	0

0		0		0	0
0		0		0	0
0		0		0	0
0		0		0	0
1		0		0	1
0	Ew/a-GOST-ci	1	ONLY_E-w/a-(	1	0
0	Ew/a-GOST-ci	1	ONLY_E-w/a-(	1	0
0		0		0	0
0		0		0	0
0	Ew/a-GOST-ci	1	ONLY_E-w/a-(	1	0
0	Ew/a-GOST-ci	1	ONLY_E-w/a-(	1	0
0	Ew/a-GOST-ci	1	ONLY_E-w/a-(	1	0
0	Ew/a-GOST-ci	1	ONLY_E-w/a-(	1	0
0		0		0	0
0		0		0	0
0		0		0	0
0	Ew/a-GOST-ci	1	ONLY_E-w/a-(	1	0
0	Ew/a-GOST-ci	1	ONLY_E-w/a-(	1	0
0		0		0	0
0		0		0	0
0		0		0	0
0		0		0	0
0		0		0	0
0		0		0	0
0		0		0	0
0		0		0	0
0		0		0	0
0		0		0	0
0		0		0	0
0		0		0	0
0		0		0	0
0		0		0	0
0		0		0	0
0		0		0	0
0		0		0	0
0		0		0	0
0		0		0	0
0	Ew/a-GOST-ci	1	ONLY_E-w/a-(	1	0
0	Ew/a-GOST-ci	1	ONLY_E-w/a-(	1	0
0		0		0	0
0		0		0	0
0	Ew/a-GOST-ci	1	ONLY_E-w/a-(	1	0
0		0		0	0
0		0		0	0
0		0		0	0
0		0		0	0
0		0		0	0

0		0		0	0
0		0		0	0
0		0		0	0
0		0		0	0
0		0		0	0
0		0		0	0
0		0		0	0
0		0		0	0
0		0		0	0
0		0		0	0
0	Ew/a-GOST-ci	1	ONLY_E-w/a-(	1	0
0	Ew/a-GOST-ci	1	ONLY_E-w/a-(	1	0
0		0		0	0
0		0		0	0
0		0		0	0
0		0		0	0
0		0		0	0
0		0		0	0
0		0		0	0
1		0		0	0
1		0		0	0
1		0		0	0
0		0		0	0
0		0		0	0
0		0		0	0
0		0		0	0
0		0		0	0
0		0		0	0
0		0		0	0
0		0		0	0
0		0		0	0
0		0		0	0
0		0		0	0
0		0		0	0
0		0		0	0
0		0		0	0
0	Ew/a-GOST-ci	1	ONLY_E-w/a-(	1	0
0	Ew/a-GOST-ci	1	ONLY_E-w/a-(	1	0
0	Ew/a-GOST-ci	1	ONLY_E-w/a-(	1	0
0	Ew/a-GOST-ci	1	ONLY_E-w/a-(	1	0
0		0		0	0
0		0		0	0
0		0		0	0
0		0		0	0
0		0		0	0
0		0		0	0
0		0		0	0

0		0		0	0
0		0		0	0
0		0		0	0
0		0		0	0
0		0		0	0
0		0		0	0
0	Ew/a-GOST-ci	1	ONLY_E-w/a-(	1	0
0		0		0	0
0		0		0	0
0		0		0	0
0		0		0	0
1	Ew/a-GOST-ci	1		0	0
1	Ew/a-GOST-ci	1		0	0
0		0		0	1
0		0		0	1
0		0		0	1
1	Ew/a-GOST-ci	1		0	0
1	Ew/a-GOST-ci	1		0	0
0		0		0	0
0		0		0	0
0		0		0	0
0	Ew/a-GOST-ci	1	ONLY_E-w/a-(	1	0
0	Ew/a-GOST-ci	1	ONLY_E-w/a-(	1	0
0		0		0	0
0		0		0	0
0	Ew/a-GOST-ci	1	ONLY_E-w/a-(	1	0
0		0		0	0
0		0		0	0
0		0		0	0
0		0		0	0
0		0		0	0
0		0		0	0
0		0		0	0
0	Ew/a-GOST-ci	1	ONLY_E-w/a-(	1	0
0	Ew/a-GOST-ci	1	ONLY_E-w/a-(	1	0
0		0		0	0
0		0		0	0
0		0		0	0
0		0		0	0
0	Ew/a-GOST-ci	1	ONLY_E-w/a-(	1	0
0	Ew/a-GOST-ci	1	ONLY_E-w/a-(	1	0
0		0		0	0
0		0		0	1
0		0		0	0
1		0		0	1
0		0		0	0
0		0		0	0

0		0		0	0
0		0		0	0
0		0		0	0
0		0		0	0
0	Ew/a-GOST-ci	1	ONLY_E-w/a-(	1	0
0	Ew/a-GOST-ci	1	ONLY_E-w/a-(	1	0
0		0		0	1
0		0		0	0
0		0		0	0
0		0		0	0
0	Ew/a-GOST-ci	1	ONLY_E-w/a-(	1	0
1	Ew/a-GOST-ci	1		0	1
1	Ew/a-GOST-ci	1		0	1
0	Ew/a-GOST-ci	1	ONLY_E-w/a-(	1	0
0	Ew/a-GOST-ci	1	ONLY_E-w/a-(	1	0
0		0		0	0
0		0		0	0
0		0		0	0
0		0		0	0
0		0		0	0
0		0		0	0
1	Ew/a-GOST-ci	1		0	0
1	Ew/a-GOST-ci	1		0	0
1	Ew/a-GOST-ci	1		0	0
0		0		0	0
0		0		0	0
0		0		0	0
0		0		0	1
0		0		0	1
0		0		0	1
0		0		0	0
0		0		0	0
0		0		0	0
0	Ew/a-GOST-ci	1	ONLY_E-w/a-(	1	0
0	Ew/a-GOST-ci	1	ONLY_E-w/a-(	1	0
0		0		0	0
0		0		0	0
0	Ew/a-GOST-ci	1	ONLY_E-w/a-(	1	0
0	Ew/a-GOST-ci	1	ONLY_E-w/a-(	1	0
0		0		0	0
0		0		0	0
0		0		0	0
0		0		0	0
0		0		0	0
0		0		0	0
0		0		0	0
0	Ew/a-GOST-ci	1	ONLY_E-w/a-(	1	0

0	Ew/a-GOST-ci	1	ONLY_E-w/a-(	1	0
0		0		0	0
0		0		0	0
0		0		0	0
0		0		0	0
0	Ew/a-GOST-ci	1	ONLY_E-w/a-(	1	0
0	Ew/a-GOST-ci	1	ONLY_E-w/a-(	1	0
1		0		0	1
1		0		0	1
1		0		0	1
1	Ew/a-GOST-ci	1		0	1
1	Ew/a-GOST-ci	1		0	1
1	Ew/a-GOST-ci	1		0	1
1	Ew/a-GOST-ci	1		0	1
0		0		0	0
0		0		0	0
0		0		0	0
0		0		0	0
0		0		0	0
0	Ew/a-GOST-ci	1	ONLY_E-w/a-(	1	0
0		0		0	0
0		0		0	0
0		0		0	0
0		0		0	0
0		0		0	0
0		0		0	0
0		0		0	0
0		0		0	0
0		0		0	0
0		0		0	0
0		0		0	0
0		0		0	0
0		0		0	0
0		0		0	0
0		0		0	0
0		0		0	0
0		0		0	0
0		0		0	0
0		0		0	0
0		0		0	0
0		0		0	0
0		0		0	1
0		0		0	0
1	Ew/a-GOST-ci	1		0	1
1	Ew/a-GOST-ci	1		0	1
1	Ew/a-GOST-ci	1		0	1
0		0		0	0

0		0		0	0
0		0		0	0
0		0		0	0
0		0		0	0
0		0		0	0
0	Ew/a-GOST-ci	1	ONLY_E-w/a-(	1	0
0	Ew/a-GOST-ci	1	ONLY_E-w/a-(	1	0
1		0		0	0
1		0		0	0
0		0		0	0
0		0		0	0
0		0		0	0
1		0		0	0
0		0		0	0
0	Ew/a-GOST-ci	1	ONLY_E-w/a-(	1	0
0	Ew/a-GOST-ci	1	ONLY_E-w/a-(	1	0
0		0		0	0
0		0		0	0
0		0		0	0
0		0		0	0
0		0		0	0
0		0		0	0
0	Ew/a-GOST-ci	1	ONLY_E-w/a-(	1	0
0	Ew/a-GOST-ci	1	ONLY_E-w/a-(	1	0
1		0		0	1
0		0		0	0
0		0		0	0
0		0		0	0
0		0		0	0
0		0		0	0
0		0		0	0
0		0		0	0
0		0		0	0
0		0		0	0
0		0		0	0
0	Ew/a-GOST-ci	1	ONLY_E-w/a-(	1	0
0		0		0	0
0		0		0	0
0		0		0	0
0		0		0	0
0		0		0	0
0		0		0	0
1		0		0	0
1	Ew/a-GOST-ci	1		0	1
1	Ew/a-GOST-ci	1		0	1
1	Ew/a-GOST-ci	1		0	1
1	Ew/a-GOST-ci	1		0	1

0	Ew/a-GOST-ci	1	ONLY_E-w/a-(	1	0
0	Ew/a-GOST-ci	1	ONLY_E-w/a-(	1	0
0		0		0	0
0		0		0	0
0		0		0	0
0		0		0	0
0		0		0	0
0		0		0	0
0		0		0	0
0	Ew/a-GOST-ci	1	ONLY_E-w/a-(	1	0
0	Ew/a-GOST-ci	1	ONLY_E-w/a-(	1	0
1		0		0	1
0		0		0	0
0		0		0	0
0		0		0	0
0		0		0	0
0		0		0	0
0		0		0	0
0		0		0	0
0		0		0	0
0		0		0	0
0		0		0	0
0		0		0	0
0		0		0	0
0		0		0	0
0		0		0	0
0		0		0	0
0		0		0	0
0		0		0	0
0		0		0	0
0		0		0	0
0		0		0	0
0	Ew/a-GOST-ci	1	ONLY_E-w/a-(	1	0
0	Ew/a-GOST-ci	1	ONLY_E-w/a-(	1	0
1	Ew/a-GOST-ci	1		0	0
1	Ew/a-GOST-ci	1		0	0
0		0		0	0
0		0		0	0
0		0		0	0
0	Ew/a-GOST-ci	1	ONLY_E-w/a-(	1	0
0	Ew/a-GOST-ci	1	ONLY_E-w/a-(	1	0
0	Ew/a-GOST-ci	1	ONLY_E-w/a-(	1	0
0		0		0	0
0		0		0	0
0		0		0	0
0		0		0	0
0		0		0	0

0		0		0	0
0		0		0	0
0	Ew/a-GOST-ci	1	ONLY_E-w/a-(	1	0
0	Ew/a-GOST-ci	1	ONLY_E-w/a-(	1	0
0		0		0	0
0		0		0	0
0		0		0	0
0		0		0	0
1		0		0	1
1		0		0	1
1		0		0	1
1		0		0	1
1		0		0	1
0	Ew/a-GOST-ci	1	ONLY_E-w/a-(	1	0
0		0		0	0
0		0		0	0
0		0		0	0
0		0		0	0
0		0		0	0
0		0		0	0
0		0		0	0
0		0		0	0
0		0		0	0
0		0		0	0
0		0		0	0
0		0		0	0
0		0		0	0
0	Ew/a-GOST-ci	1	ONLY_E-w/a-(	1	0
0	Ew/a-GOST-ci	1	ONLY_E-w/a-(	1	0
0		0		0	0
0		0		0	0
0		0		0	0
0		0		0	0
0		0		0	0
0		0		0	0
0		0		0	0
0		0		0	0
0		0		0	0
0		0		0	0
1		0		0	0
0	Ew/a-GOST-ci	1	ONLY_E-w/a-(	1	0
0	Ew/a-GOST-ci	1	ONLY_E-w/a-(	1	0
0	Ew/a-GOST-ci	1	ONLY_E-w/a-(	1	0
0		0		0	0
0		0		0	0
0		0		0	0
0		0		0	0

0		0		0	0
1	Ew/a-GOST-ci	1		0	0
0		0		0	0
0		0		0	0
0		0		0	0
0		0		0	0
0		0		0	0
0		0		0	0
0		0		0	0
0		0		0	0
0		0		0	0
0		0		0	0
0		0		0	0
0		0		0	0
0		0		0	0
0		0		0	0
0		0		0	0
0		0		0	0
0	Ew/a-GOST-ci	1	ONLY_E-w/a-(	1	0
0	Ew/a-GOST-ci	1	ONLY_E-w/a-(	1	0
0	Ew/a-GOST-ci	1	ONLY_E-w/a-(	1	0
0		0		0	0
0		0		0	0
0		0		0	0
0	Ew/a-GOST-ci	1	ONLY_E-w/a-(	1	0
0		0		0	0
0		0		0	0
1	Ew/a-GOST-ci	1		0	0
1	Ew/a-GOST-ci	1		0	0
0		0		0	0
0		0		0	0
0		0		0	0
0		0		0	0
0		0		0	0
0		0		0	0
0	Ew/a-GOST-ci	1	ONLY_E-w/a-(	1	0
0	Ew/a-GOST-ci	1	ONLY_E-w/a-(	1	0
0		0		0	0
0		0		0	0
0		0		0	0
0	Ew/a-GOST-ci	1	ONLY_E-w/a-(	1	1
0	Ew/a-GOST-ci	1	ONLY_E-w/a-(	1	1
0	Ew/a-GOST-ci	1	ONLY_E-w/a-(	1	1
0		0		0	0
0		0		0	0
0		0		0	0
0		0		0	0

0		0		0	0
0	Ew/a-GOST-ci	1	ONLY_E-w/a-	1	0
0	Ew/a-GOST-ci	1	ONLY_E-w/a-	1	0
0	Ew/a-GOST-ci	1	ONLY_E-w/a-	1	0
0		0		0	0
0		0		0	0
0		0		0	0
0		0		0	0
0		0		0	0
0		0		0	0
0		0		0	0
0		0		0	0
0		0		0	0
0		0		0	0
0		0		0	0
0		0		0	0
0		0		0	0
0		0		0	0
1		0		0	0
1		0		0	0
1		0		0	0
0		0		0	0
0		0		0	0
0		0		0	0
0		0		0	0
0		0		0	0
0		0		0	0
1		0		0	0
0		0		0	0
0		0		0	0
0		0		0	0
0		0		0	0
0		0		0	1
0		0		0	1
0		0		0	1
0		0		0	0
0		0		0	0
0		0		0	0
0		0		0	0
0		0		0	0
0		0		0	0
0		0		0	0
0		0		0	0
0		0		0	1
0		0		0	0
0		0		0	0
0		0		0	0

0		0		0	0
0		0		0	0
0		0		0	0
0	Ew/a-GOST-ci	1	ONLY_E-w/a-(	1	0
0	Ew/a-GOST-ci	1	ONLY_E-w/a-(	1	0
0	Ew/a-GOST-ci	1	ONLY_E-w/a-(	1	0
0		0		0	0
0	Ew/a-GOST-ci	1	ONLY_E-w/a-(	1	0
0		0		0	0
0		0		0	0
0		0		0	0
0		0		0	0
1		0		0	1
1		0		0	1
1		0		0	1
0	Ew/a-GOST-ci	1	ONLY_E-w/a-(	1	0
0	Ew/a-GOST-ci	1	ONLY_E-w/a-(	1	0
0	Ew/a-GOST-ci	1	ONLY_E-w/a-(	1	0
0		0		0	0
0		0		0	0
0		0		0	0
0		0		0	0
0		0		0	0
0		0		0	0
0		0		0	0
0		0		0	0
0		0		0	0
0		0		0	0
0		0		0	0
0		0		0	0
0		0		0	0
0		0		0	0
0		0		0	0
0		0		0	0
1		0		0	0
1		0		0	0
1		0		0	0
0		0		0	0
0		0		0	0
0		0		0	0
0		0		0	0
0		0		0	0
0		0		0	0
0		0		0	0
0		0		0	0
0		0		0	0
0		0		0	0

0		0	0	0
0		0	0	0
0		0	0	0
0		0	0	0
0		0	0	0
0		0	0	0
0		0	0	0
1		0	0	1
1		0	0	1
0		0	0	0
0		0	0	0
1	Ew/a-GOST-ci	1	0	0
0		0	0	0
0		0	0	0
0		0	0	0
0		0	0	0
1		0	0	1
1		0	0	1
1	Ew/a-GOST-ci	1	0	1
1	Ew/a-GOST-ci	1	0	1
1	Ew/a-GOST-ci	1	0	1
1	Ew/a-GOST-ci	1	0	1
0		0	0	0
0		0	0	0
0		0	0	0
0		0	0	0
1	Ew/a-GOST-ci	1	0	0
1	Ew/a-GOST-ci	1	0	0
1	Ew/a-GOST-ci	1	0	0
0		0	0	0
0		0	0	0
0		0	0	0

ewagostart_2	ewagostart_b	only_ewagost	only_ewagost	nop_egostcit_	easacity_2	easacity_b_2	ewagostcit_2
	0		0			0	
	0		0			0	
	0		0			0	
	0		0			0	
	0		0			0	
	0		0			0	
	0		0			0	
	0		0			0	
	0		0			0	
	0		0			0	
	1		1			0	
	0		0			0	
	0		0			0	
	0		0			0	
	0		0			0	
	0		0			0	
	0		0			0	
	0		0			0	
	0		0			0	
	0		0			0	
	0		0			0	
	0		0			0	
	1		1			0	
	1		1			0	
	1		1			0	
	0		0			0	
	0		0			0	
	0		0			0	
	0		0			0	
	1		1			0	
	0		0			0	
	0		0			0	
	0		0			0	
	0		0			0	
	0		0			0	
	0		0			0	
	0		0			0	
	0		0			0	
	0		0			0	
	0		0			0	
	0		0			0	
	0		0			0	
	1		1			0	
	1		1			0	
	1		1			0	
	0		0			0	

0	0	0
0	0	0
1	1	0
0	0	0
1	1	0
0	0	0
0	0	0
0	0	0
0	0	1
0	0	0
0	0	0
0	0	0
0	0	0
0	0	0
0	0	0
0	0	0
0	0	0
0	0	0
0	0	0
0	0	0
0	0	0
0	0	0
0	0	0
0	0	0
1	1	0
1	1	0
0	0	0
0	0	0
0	0	0
1	0	0
1	0	0
1	0	0
1	0	0
1	0	0
0	0	0
0	0	0
0	0	0
0	0	0
0	0	0
0	0	0
0	0	0
0	0	0
0	0	0
0	0	0
0	0	0
0	0	0
0	0	0

0	0	0
0	0	0
0	0	0
1	1	0
0	0	0
0	0	0
0	0	0
0	0	0
1	1	0
0	0	0
0	0	0
0	0	0
0	0	0
1	1	0
0	0	0
0	0	0
0	0	0
0	0	0
0	0	0
0	0	0
1	1	0
0	0	0
0	0	0
0	0	0
0	0	0
1	1	0
1	1	0
0	0	0
0	0	0
1	0	0
1	0	0
0	0	0
0	0	1
0	0	1
1	1	0
0	0	0
0	0	0
1	1	0
1	1	0
1	1	1
0	0	0
0	0	0
0	0	0
0	0	0
0	0	0
0	0	0
0	0	0

0	0	0
0	0	0
0	0	0
0	0	0
0	0	0
0	0	0
0	0	0
0	0	0
0	0	0
0	0	0
0	0	0
0	0	0
0	0	0
0	0	1
0	0	0
0	0	0
1	0	0
0	0	0
0	0	0
0	0	0
0	0	0
0	0	0
0	0	0
0	0	0
0	0	0
0	0	0
0	0	0
0	0	0
0	0	0
0	0	0
0	0	0
0	0	0
0	0	1
0	0	0
1	1	0
1	1	0
0	0	0
0	0	0
0	0	1
0	0	0
0	0	0
1	0	0
1	0	0
0	0	0
0	0	0
0	0	0
0	0	0

0	0	0
0	0	0
0	0	0
0	0	0
0	0	1
0	0	1
0	0	0
0	0	0
0	0	0
1	1	0
0	0	0
0	0	0
1	1	0
1	1	0
0	0	0
0	0	0
0	0	0
0	0	0
0	0	0
0	0	0
0	0	0
0	0	0
1	1	0
1	1	0
0	0	0
0	0	0
0	0	0
0	0	0
0	0	0
0	0	0
0	0	0
0	0	0
0	0	0
0	0	0
0	0	0
0	0	0
1	1	0
0	0	0
1	0	0
1	0	0
1	0	0
1	0	0
0	0	0
0	0	0
0	0	0
0	0	0
0	0	0

1	1	0
1	1	0
0	0	0
0	0	0
0	0	0
0	0	0
1	1	0
1	1	0
1	1	0
0	0	0
0	0	0
0	0	0
0	0	0
0	0	0
0	0	0
0	0	0
1	1	0
1	1	0
1	1	0
0	0	0
0	0	0
1	0	1
1	0	1
1	0	1
0	0	0
0	0	0
1	1	0
1	1	0
0	0	0
0	0	0
1	0	1
1	0	1
1	0	1
0	0	0
0	0	0
0	0	0
0	0	0
0	0	0
1	1	0
1	1	0
0	0	0
0	0	0
0	0	0
0	0	0
0	0	0
1	1	0
0	0	0

1	1	0
1	1	0
0	0	0
0	0	0
1	1	0
1	1	0
0	0	0
0	0	0
0	0	0
1	1	0
1	1	0
0	0	0
0	0	0
0	0	0
0	0	0
0	0	0
0	0	0
0	0	0
0	0	0
0	0	0
0	0	0
0	0	0
0	0	0
0	0	0
0	0	0
1	1	0
1	1	0
1	1	0
1	1	0
1	1	0
0	0	0
0	0	0
0	0	0
0	0	0
0	0	0
0	0	0
0	0	0
0	0	0
0	0	0
0	0	0
0	0	0
0	0	0
0	0	0
0	0	0
0	0	0
0	0	0
0	0	0

0	0	0
0	0	0
0	0	0
0	0	0
0	0	0
1	1	0
1	1	0
0	0	0
0	0	0
0	0	0
0	0	0
0	0	0
0	0	0
0	0	0
1	0	0
1	0	0
0	0	0
1	1	0
0	0	0
0	0	0
0	0	0
0	0	0
0	0	0
0	0	0
1	1	1
1	1	1
0	0	0
0	0	0
0	0	0
0	0	0
0	0	0
0	0	0
0	0	0
0	0	0
0	0	0
0	0	0
0	0	0
0	0	0
0	0	0
0	0	0
1	1	0
0	0	0
0	0	0
0	0	0
0	0	0
1	1	0
1	1	0

0	0	0
0	0	0
0	0	0
0	0	0
0	0	0
0	0	0
0	0	0
1	0	1
1	0	1
0	0	0
0	0	0
1	1	0
1	1	0
0	0	0
0	0	0
0	0	0
0	0	0
0	0	0
0	0	0
0	0	0
0	0	0
1	1	0
0	0	0
0	0	0
0	0	0
0	0	0
0	0	0
0	0	0
1	1	0
1	1	0
0	0	0
0	0	0
0	0	0
0	0	0
0	0	0
0	0	0
0	0	0
0	0	0
0	0	0
0	0	0
0	0	0
0	0	0
0	0	0
0	0	0
0	0	0
0	0	0
0	0	0

0	0	0
1	1	0
1	1	0
1	1	0
1	1	0
0	0	0
0	0	0
0	0	0
1	1	0
0	0	0
0	0	0
0	0	0
0	0	0
0	0	0
0	0	0
0	0	0
0	0	0
0	0	0
0	0	0
0	0	1
0	0	1
0	0	0
0	0	0
0	0	0
0	0	0
0	0	0
0	0	0
0	0	0
0	0	0
0	0	0
0	0	0
0	0	0
0	0	0
0	0	0
0	0	0
0	0	0
0	0	0
0	0	0
0	0	0
0	0	0
0	0	0
0	0	0
0	0	0
0	0	0
0	0	0
0	0	0
0	0	0

0	0	0
0	0	0
0	0	0
0	0	0
0	0	0
0	0	0
0	0	0
0	0	0
0	0	0
0	0	0
1	1	0
1	1	0
1	1	0
0	0	0
0	0	0
0	0	0
0	0	0
0	0	0
0	0	0
0	0	0
1	1	0
1	1	0
0	0	0
0	0	1
0	0	1
0	0	1
0	0	0
0	0	0
0	0	0
0	0	0
0	0	0
0	0	0
0	0	0
0	0	0
1	1	0
0	0	0
0	0	0
0	0	0
0	0	0
0	0	0
1	1	0
0	0	0
0	0	0
1	1	0
0	0	0
0	0	0
0	0	0

0	0	0
0	0	0
0	0	0
0	0	0
0	0	0
0	0	0
0	0	0
0	0	0
0	0	0
0	0	0
0	0	1
0	0	1
0	0	1
0	0	0
0	0	0
0	0	0
0	0	0
0	0	0
0	0	0
0	0	0
0	0	0
0	0	0
0	0	0
0	0	0
0	0	0
0	0	0
0	0	0
0	0	0
0	0	0
0	0	0
0	0	0
0	0	0
0	0	0
0	0	0
0	0	0
0	0	0
0	0	0
0	0	0
0	0	0
0	0	0
0	0	0
0	0	0
0	0	0
0	0	0
1	1	0
0	0	0
0	0	0

1	1	0
1	1	0
0	0	0
0	0	0
1	1	0
1	1	0
1	1	0
0	0	0
0	0	0
0	0	0
0	0	0
0	0	0
1	1	0
0	0	0
0	0	0
1	1	0
1	1	0
0	0	0
0	0	0
0	0	0
0	0	0
0	0	0
0	0	0
0	0	0
0	0	0
0	0	0
0	0	0
0	0	0
0	0	0
1	0	0
0	0	0
0	0	0
0	0	0
0	0	0
0	0	0
0	0	0
0	0	0
1	1	0
1	1	0
0	0	0
1	1	0
0	0	0
0	0	0
0	0	0
0	0	0
0	0	0
0	0	0

0	0	0
0	0	0
1	1	0
1	1	0
0	0	0
0	0	0
1	0	0
1	0	0
1	0	0
1	0	0
0	0	0
0	0	0
0	0	0
0	0	0
0	0	0
0	0	0
0	0	0
0	0	0
0	0	0
0	0	0
0	0	0
1	1	0
1	1	0
1	1	0
0	0	0
0	0	0
0	0	0
0	0	0
0	0	0
0	0	0
0	0	0
0	0	0
0	0	0
0	0	0
0	0	0
0	0	0
0	0	1
0	0	1
0	0	0
0	0	0
0	0	0
0	0	0
0	0	0
0	0	0
0	0	0
0	0	0
0	0	0

0	0	0
0	0	0
0	0	0
0	0	0
0	0	0
0	0	0
0	0	0
0	0	0
0	0	0
0	0	0
0	0	0
0	0	0
0	0	0
0	0	0
1	1	0
0	0	0
0	0	0
0	0	0
0	0	0
1	1	0
0	0	1
0	0	1
0	0	0
0	0	0
0	0	0
0	0	0
0	0	0
1	1	0
0	0	0
0	0	0
0	0	0
0	0	0
0	0	0
0	0	0
0	0	0
0	0	0
0	0	0
0	0	0
0	0	0
0	0	0
0	0	0
0	0	0
0	0	0
0	0	0
0	0	0
0	0	0
0	0	0

0	0	0
0	0	0
0	0	0
0	0	0
0	0	0
0	0	0
0	0	0
0	0	0
1	1	1
0	0	0
0	0	0
0	0	0
0	0	0
0	0	0
0	0	0
1	1	0
0	0	0
0	0	0
0	0	0
1	1	0
1	1	0
0	0	1
0	0	1
0	0	0
1	1	0
0	0	0
0	0	0
0	0	0
1	1	0
0	0	0
0	0	0
0	0	0
0	0	0
0	0	0
0	0	0
0	0	0
0	0	0
0	0	0
0	0	0
0	0	0
0	0	0
0	0	0
0	0	0
0	0	0
0	0	0
0	0	0
0	0	0

0	0	0
0	0	0
0	0	0
0	0	0
0	0	0
0	0	0
0	0	0
0	0	0
0	0	0
0	0	0
0	0	0
0	0	0
0	0	0
0	0	0
0	0	0
0	0	0
0	0	0
0	0	0
0	0	0
0	0	0
0	0	0
0	0	0
0	0	0
0	0	0
0	0	0
0	0	0
0	0	0
0	0	0
0	0	0
0	0	0
0	0	0
0	0	0
0	0	0
0	0	0
0	0	0
0	0	0
0	0	0
0	0	0
0	0	0
0	0	0
0	0	0
0	0	0
0	0	0
0	0	0
0	0	0
1	1	0
0	0	0

0	0	0
0	0	0
0	0	0
1	1	0
0	0	0
0	0	0
1	1	1
1	1	1
0	0	0
0	0	0
0	0	0
0	0	0
0	0	0
0	0	0
0	0	0
0	0	0
0	0	0
0	0	0
0	0	0
1	0	0
1	0	0
1	0	0
1	0	0
1	0	0
0	0	0
0	0	0
1	0	0
1	0	0
1	0	0
0	0	0
0	0	0
0	0	0
1	1	0
0	0	0
0	0	0
0	0	0
1	1	1
1	1	1
0	0	0
0	0	0
0	0	0
1	1	0
1	1	0
0	0	0
0	0	0
0	0	0
0	0	0

1	1	0
0	0	0
0	0	0
0	0	0
0	0	0
0	0	0
0	0	0
0	0	0
0	0	0
0	0	0
0	0	0
1	1	0
1	1	0
1	1	0
0	0	0
0	0	0
0	0	0
0	0	0
0	0	0
0	0	0
1	1	0
1	1	0
1	1	0
1	1	0
0	0	0
0	0	0
0	0	0
0	0	0
0	0	0
0	0	0
0	0	0
0	0	0
1	1	0
1	1	0
1	1	0
0	0	0
0	0	0
0	0	0
0	0	0
1	1	0
1	1	0
0	0	0
0	0	0
0	0	0
0	0	0
0	0	0
0	0	0

0	0	0
0	0	0
0	0	0
0	0	0
0	0	0
0	0	1
0	0	0
0	0	0
0	0	0
0	0	0
0	0	0
1	1	0
0	0	0
0	0	0
0	0	0
0	0	0
0	0	0
0	0	0
0	0	0
0	0	0
0	0	0
0	0	0
1	1	0
1	1	0
1	1	0
1	1	0
1	1	0
0	0	0
0	0	0
0	0	0
0	0	0
0	0	0
0	0	0
0	0	0
0	0	0
1	1	0
0	0	0
0	0	0
1	0	1
1	0	1
0	0	0
0	0	0
0	0	0
0	0	0
0	0	0
0	0	0
0	0	0

0	0	0
0	0	0
0	0	0
1	1	0
1	1	0
0	0	0
0	0	0
0	0	0
0	0	0
0	0	0
0	0	0
1	1	0
1	1	0
1	1	0
1	1	0
0	0	0
0	0	0
0	0	0
0	0	0
0	0	0
0	0	0
1	1	0
0	0	0
0	0	0
0	0	0
1	1	0
1	1	0
1	1	0
1	1	0
0	0	0
0	0	0
0	0	0
0	0	0
0	0	0
0	0	0
0	0	0
0	0	0
0	0	0
0	0	0
0	0	0
0	0	0
0	0	0
0	0	0
1	0	1
1	0	1
1	0	1
0	0	1

0	0	1
0	0	0
0	0	0
0	0	0
0	0	0
0	0	0
0	0	0
1	1	0
1	1	0
0	0	0
0	0	0
1	0	0
1	0	0
1	0	0
0	0	0
0	0	0
0	0	0
0	0	0
0	0	0
0	0	0
0	0	0
0	0	0
0	0	0
0	0	0
0	0	1
0	0	1
0	0	1
0	0	1
0	0	0
1	1	0
0	0	0
0	0	0
0	0	0
0	0	0
0	0	0
0	0	0
0	0	0
0	0	0
0	0	0
0	0	0
0	0	0
0	0	0
0	0	0
0	0	0
0	0	0
0	0	0
0	0	0

0	0	0
0	0	0
0	0	0
0	0	0
0	0	0
0	0	0
0	0	0
1	1	0
1	1	0
0	0	0
0	0	0
0	0	0
1	1	0
1	1	0
1	1	0
0	0	0
0	0	0
0	0	0
1	1	0
1	1	0
1	1	0
0	0	0
0	0	0
0	0	0
0	0	0
0	0	0
0	0	0
0	0	0
0	0	0
0	0	0
0	0	0
0	0	0
0	0	0
0	0	0
1	1	0
1	1	0
0	0	0
0	0	0
0	0	0
0	0	0
0	0	0
0	0	0
0	0	0
0	0	0
0	0	0
0	0	0
1	1	0

0	0	0
0	0	0
0	0	0
0	0	0
0	0	0
0	0	0
0	0	0
1	0	1
1	0	1
0	0	0
0	0	0
0	0	0
0	0	0
0	0	0
0	0	0
0	0	1
0	0	0
0	0	0
0	0	0
0	0	0
0	0	0
1	1	0
1	0	1
1	0	1
1	0	1
0	0	0
1	1	0
0	0	0
0	0	1
0	0	1
0	0	0
0	0	0
1	1	0
1	1	0
1	1	0
1	1	0
0	0	0
0	0	0
0	0	0
0	0	0
0	0	0
1	1	0
1	1	0
0	0	0
0	0	0
0	0	0
0	0	0

1	0	0
1	0	0
1	0	0
0	0	0
0	0	0
0	0	0
0	0	0
0	0	0
0	0	0
0	0	1
0	0	1
0	0	1
0	0	0
0	0	0
0	0	0
0	0	0
0	0	0
0	0	0
0	0	0
0	0	0
0	0	0
0	0	0
0	0	0
0	0	0
0	0	0
0	0	0
0	0	0
0	0	0
0	0	1
0	0	1
0	0	0
0	0	0
0	0	0
0	0	0
0	0	0
0	0	0
0	0	0
0	0	0
0	0	0
0	0	0
0	0	0
0	0	0
0	0	0
0	0	0
0	0	0
0	0	0
0	0	0

0	0	0
0	0	0
0	0	1
0	0	1
0	0	0
0	0	0
0	0	0
0	0	0
0	0	0
0	0	0
0	0	0
0	0	0
0	0	0
0	0	0
0	0	0
0	0	0
1	1	1
1	1	1
0	0	0
0	0	0
0	0	0
0	0	0
0	0	0
0	0	0
0	0	0
0	0	0
0	0	0
0	0	0
0	0	0
0	0	0
0	0	0
0	0	0
0	0	1
0	0	0
0	0	1
0	0	0
0	0	0
0	0	0
0	0	0
0	0	0
0	0	0
0	0	0
0	0	0
0	0	0
0	0	0
0	0	1
0	0	1

0	0	0
0	0	0
0	0	1
0	0	0
0	0	0
0	0	0
0	0	0
1	1	0
1	1	0
0	0	0
0	0	0
0	0	0
1	1	0
0	0	0
0	0	0
1	1	0
1	1	0
1	1	0
0	0	0
0	0	0
0	0	0
0	0	0
0	0	0
0	0	0
0	0	0
0	0	0
0	0	0
0	0	0
0	0	0
0	0	0
0	0	0
0	0	0
0	0	0
1	1	0
1	1	0
0	0	0
0	0	0
0	0	0
0	0	0
0	0	1
0	0	0
0	0	0
0	0	0
0	0	0
0	0	0
0	0	0
0	0	0

1	1	0
0	0	0
1	1	0
0	0	0
0	0	0
0	0	0
0	0	0
0	0	0
0	0	0
0	0	0
1	1	0
1	1	0
0	0	0
0	0	0
0	0	0
1	1	0
1	1	0
0	0	0
0	0	0
0	0	0
0	0	0
1	1	0
1	1	0
1	1	0
0	0	0
0	0	0
0	0	0
0	0	0
0	0	0
0	0	0
0	0	0
0	0	0
0	0	0
0	0	0
0	0	0
0	0	0
0	0	0
0	0	0
0	0	0
0	0	0
0	0	0
0	0	0
1	1	0
1	1	0
0	0	0
0	0	0
0	0	0

0	0	0
0	0	0
0	0	0
0	0	0
0	0	0
0	0	0
0	0	0
0	0	0
1	1	0
1	1	0
0	0	0
0	0	0
0	0	0
0	0	0
0	0	0
0	0	0
1	1	0
0	0	0
0	0	0
0	0	0
1	1	0
1	1	0
0	0	0
0	0	0
0	0	0
0	0	0
1	1	0
0	0	0
0	0	0
0	0	0
0	0	0
0	0	1
0	0	1
0	0	1
0	0	0
1	1	0
0	0	0
0	0	0
0	0	0
0	0	0
0	0	0
0	0	0
0	0	0
0	0	0
0	0	0
0	0	0
1	1	0

1	1	0
1	1	0
0	0	0
0	0	0
0	0	0
0	0	0
0	0	0
0	0	0
0	0	0
0	0	0
0	0	0
0	0	0
0	0	0
0	0	0
0	0	0
0	0	0
0	0	0
0	0	0
0	0	0
0	0	0
0	0	0
1	1	1
1	1	1
0	0	0
0	0	0
0	0	0
1	1	1
1	1	1
1	1	0
1	1	0
0	0	0
0	0	0
0	0	0
1	1	0
0	0	0
0	0	0
0	0	0
0	0	0
1	1	0
1	1	0
1	1	0
1	1	0
0	0	0
0	0	0
0	0	0
0	0	0
0	0	0

0	0	0
0	0	0
0	0	0
0	0	0
0	0	0
1	1	0
0	0	0
1	1	0
1	1	0
0	0	1
0	0	0
0	0	0
0	0	0
0	0	0
0	0	0
0	0	0
0	0	0
0	0	0
0	0	0
1	1	0
1	1	0
1	1	0
1	1	0
0	0	0
0	0	0
0	0	0
0	0	0
0	0	0
0	0	0
0	0	0
0	0	0
0	0	0
0	0	0
0	0	0
0	0	0
0	0	0
0	0	0
0	0	0
0	0	0
0	0	0
0	0	0
0	0	0
0	0	0
0	0	0
0	0	0
0	0	0
0	0	0

0	0	0
0	0	0
0	0	0
0	0	0
0	0	0
0	0	0
0	0	0
0	0	0
0	0	0
0	0	0
0	0	0
0	0	0
0	0	0
0	0	0
0	0	0
0	0	0
1	1	0
1	1	0
0	0	0
0	0	0
0	0	0
0	0	0
0	0	0
0	0	0
0	0	0
0	0	0
0	0	0
0	0	0
0	0	0
0	0	0
0	0	0
0	0	0
0	0	0
0	0	0
0	0	0
0	0	0
0	0	0
0	0	0
0	0	0
0	0	0
0	0	0
1	0	1
1	0	1
1	0	1
0	0	1
0	0	1
0	0	0

0	0	0
0	0	0
0	0	0
0	0	0
0	0	0
0	0	0
0	0	0
0	0	0
0	0	0
1	1	0
1	1	0
1	1	0
0	0	0
0	0	0
0	0	0
0	0	0
0	0	0
0	0	0
0	0	0
0	0	0
0	0	0
0	0	0
0	0	0
0	0	0
0	0	0
0	0	0
0	0	0
0	0	0
0	0	0
0	0	1
0	0	1
0	0	0
0	0	0
0	0	0
0	0	0
0	0	0
1	1	0
0	0	0
0	0	0
0	0	0
0	0	0
0	0	0
0	0	0
0	0	0
0	0	0
0	0	0
0	0	0

0	0	0
0	0	0
0	0	0
0	0	0
0	0	0
0	0	0
0	0	0
0	0	0
0	0	0
0	0	0
0	0	0
0	0	0
0	0	0
0	0	0
0	0	0
0	0	0
0	0	0
0	0	0
0	0	0
1	0	1
0	0	0
0	0	0
0	0	0
0	0	0
0	0	0
0	0	0
0	0	0
0	0	0
0	0	0
1	1	0
1	1	0
1	1	0
1	1	0
0	0	0
1	1	0
1	1	0
1	1	0
0	0	0
0	0	0
0	0	0
0	0	0
0	0	0
0	0	0
0	0	0
0	0	0
0	0	0
0	0	0

0	0	0
0	0	0
1	1	0
0	0	1
0	0	0
1	0	1
1	0	1
0	0	0
0	0	0
0	0	0
0	0	0
0	0	0
0	0	0
0	0	0
0	0	0
0	0	0
0	0	0
0	0	0
0	0	0
1	1	0
0	0	0
0	0	0
0	0	0
0	0	0
0	0	0
0	0	0
0	0	0
0	0	0
0	0	0
1	1	0
0	0	0
0	0	0
0	0	0
0	0	0
0	0	0
0	0	0
0	0	0
0	0	0
0	0	0
0	0	0
0	0	0
0	0	0
0	0	0
1	1	0
1	1	0
1	1	0
0	0	0

0	0	0
0	0	0
0	0	0
0	0	0
1	1	0
1	1	0
0	0	0
0	0	0
1	1	0
1	1	0
1	1	0
1	1	0
0	0	1
0	0	1
0	0	1
0	0	0
1	0	0
1	0	0
1	0	0
0	0	0
0	0	0
0	0	0
0	0	0
0	0	0
0	0	0
0	0	0
0	0	0
0	0	0
0	0	0
0	0	0
0	0	0
0	0	0
0	0	0
0	0	1
0	0	1
0	0	1
0	0	1
0	0	0
0	0	0
0	0	0
0	0	0
0	0	0
1	1	0
0	0	0
0	0	0
0	0	0
0	0	0

0	0	0
0	0	0
0	0	0
0	0	0
0	0	0
0	0	0
0	0	0
1	1	0
0	0	0
0	0	0
0	0	0
0	0	0
0	0	0
0	0	0
0	0	0
0	0	0
0	0	0
0	0	0
0	0	0
0	0	0
0	0	0
0	0	0
0	0	0
0	0	0
0	0	0
0	0	0
0	0	0
0	0	0
0	0	0
0	0	0
0	0	0
0	0	0
0	0	0
0	0	0
0	0	0
0	0	0
0	0	0
0	0	0
0	0	0
0	0	0
0	0	0
0	0	0
0	0	0
0	0	0
0	0	0
0	0	0
0	0	0

0	0	0
0	0	0
0	0	0
0	0	0
0	0	0
0	0	0
0	0	0
0	0	0
0	0	0
0	0	0
0	0	0
0	0	0
0	0	0
1	1	0
1	1	0
0	0	0
0	0	0
0	0	0
0	0	0
1	1	0
1	1	0
0	0	0
0	0	0
0	0	0
0	0	0
1	1	0
1	1	0
0	0	0
0	0	0
0	0	0
0	0	0
0	0	0
0	0	1
0	0	0
0	0	0
0	0	0
0	0	0
0	0	0
0	0	0
0	0	0
0	0	0
0	0	0
0	0	0
0	0	0
1	1	0
0	0	0
0	0	0

0	0	1
0	0	1
0	0	0
0	0	0
0	0	0
0	0	0
0	0	0
0	0	0
0	0	0
0	0	0
0	0	0
0	0	0
0	0	0
0	0	0
1	1	1
0	0	0
0	0	0
1	1	0
1	1	0
0	0	0
0	0	0
0	0	0
0	0	0
0	0	0
0	0	1
0	0	1
0	0	0
0	0	0
0	0	0
1	1	0
0	0	0
0	0	0
0	0	0
0	0	0
0	0	0
0	0	0
0	0	0
0	0	0
0	0	0
0	0	0
0	0	0
0	0	0
1	1	0
0	0	0
0	0	0
0	0	0
0	0	0

0	0	0
0	0	0
0	0	0
0	0	0
0	0	0
0	0	0
0	0	0
0	0	0
0	0	0
0	0	0
1	1	1
1	1	1
0	0	0
0	0	0
0	0	0
0	0	0
1	1	0
1	1	0
0	0	0
0	0	0
0	0	0
0	0	0
0	0	0
0	0	0
0	0	0
0	0	0
0	0	0
0	0	0
0	0	0
0	0	0
0	0	0
0	0	0
0	0	0
0	0	0
0	0	0
0	0	0
0	0	0
0	0	0
0	0	0
0	0	0
0	0	0
0	0	0
0	0	0
0	0	0
0	0	0
0	0	0
0	0	0

0	0	0
0	0	0
0	0	0
0	0	0
0	0	0
1	1	0
0	0	0
0	0	0
0	0	0
0	0	0
0	0	0
0	0	0
1	1	0
0	0	0
0	0	0
0	0	0
1	1	0
0	0	0
0	0	0
0	0	0
0	0	0
0	0	0
0	0	0
0	0	0
0	0	0
0	0	0
0	0	0
0	0	0
0	0	0
1	1	0
0	0	0
1	1	0
1	1	0
1	1	0
1	1	0
0	0	0
0	0	0
0	0	0
0	0	0
0	0	0
0	0	0
0	0	0
0	0	0
0	0	0
0	0	0
0	0	0
0	0	0

0	0	0
0	0	0
1	0	0
1	0	0
1	0	0
1	0	0
0	0	0
0	0	0
0	0	0
0	0	0
0	0	0
0	0	0
0	0	0
0	0	0
0	0	0
0	0	0
0	0	0
0	0	0
0	0	0
0	0	0
0	0	0
0	0	0
0	0	0
0	0	0
0	0	0
0	0	0
0	0	0
0	0	0
0	0	0
0	0	0
0	0	0
0	0	1
1	0	0
1	0	0
1	0	0
0	0	0
0	0	0
0	0	0
0	0	0
1	1	0
0	0	0
0	0	0
1	1	0
1	1	0
0	0	0
0	0	0
0	0	0

0	0	0
0	0	0
1	1	0
1	1	0
1	1	0
1	0	1
1	0	1
1	0	1
0	0	0
0	0	0
1	1	0
0	0	0
0	0	0
0	0	0
1	1	0
1	1	0
0	0	0
0	0	0
0	0	0
0	0	0
0	0	0
0	0	0
0	0	0
0	0	0
0	0	0
1	0	0
1	0	0
1	0	0
1	0	0
1	0	0
0	0	0
0	0	0
0	0	0
0	0	0
0	0	0
0	0	0
0	0	0
1	1	0
1	1	0
0	0	0
1	1	0
0	0	0
0	0	0
0	0	0
0	0	0
1	1	0
0	0	0

0	0	0
0	0	0
0	0	0
0	0	0
0	0	0
0	0	0
0	0	0
0	0	0
0	0	0
0	0	0
0	0	0
0	0	0
0	0	0
0	0	0
0	0	0
0	0	0
0	0	0
1	1	0
1	1	0
1	1	0
1	1	0
0	0	0
0	0	0
0	0	0
0	0	0
1	1	0
0	0	0
0	0	0
0	0	0
0	0	0
1	1	0
1	1	0
0	0	0
0	0	0
0	0	0
0	0	0
0	0	0
0	0	0
0	0	0
0	0	0
0	0	0
0	0	0
0	0	0
0	0	0
1	1	0
1	1	0
0	0	0

0	0	0
0	0	0
0	0	0
0	0	0
0	0	0
0	0	0
1	1	0
1	1	0
0	0	0
0	0	0
0	0	0
0	0	0
0	0	0
0	0	0
0	0	0
0	0	0
0	0	0
0	0	0
0	0	0
0	0	0
0	0	0
1	1	0
1	1	0
1	1	0
0	0	0
1	1	0
1	1	0
0	0	0
0	0	0
0	0	0
0	0	0
0	0	0
1	1	0
1	1	0
0	0	0
0	0	0
0	0	0
1	1	0
1	1	0
0	0	0
0	0	0
0	0	0
0	0	0
0	0	0
1	1	0
1	1	0
0	0	0

0	0	0
0	0	0
0	0	0
0	0	0
0	0	0
0	0	0
0	0	0
0	0	0
0	0	0
0	0	0
0	0	0
1	1	0
0	0	0
0	0	0
0	0	0
0	0	0
1	1	0
0	0	0
0	0	0
0	0	0
0	0	0
0	0	0
0	0	0
0	0	0
0	0	0
0	0	0
0	0	0
0	0	0
0	0	0
0	0	1
0	0	1
0	0	0
0	0	0
1	1	0
1	1	0
0	0	0
0	0	0
0	0	0
0	0	0
1	1	0
1	1	0
1	1	0
0	0	0
1	1	0
1	1	0
1	1	0
0	0	0

0	0	0
0	0	0
0	0	0
0	0	0
0	0	0
0	0	0
0	0	0
0	0	0
0	0	0
0	0	0
0	0	0
0	0	0
0	0	0
0	0	0
1	1	0
0	0	0
0	0	0
0	0	0
0	0	0
0	0	0
0	0	0
0	0	0
0	0	0
1	1	0
0	0	0
0	0	0
0	0	0
0	0	0
0	0	0
0	0	0
0	0	0
0	0	0
0	0	0
0	0	0
0	0	0
0	0	0
0	0	0
1	1	0
1	1	0
1	1	0
1	1	0
0	0	0
0	0	0
0	0	0
0	0	0
0	0	0
1	1	0

0	0	0
0	0	0
0	0	0
0	0	0
0	0	0
0	0	0
1	1	0
0	0	0
0	0	0
0	0	0
0	0	0
0	0	0
0	0	0
0	0	0
0	0	0
0	0	0
1	1	0
1	1	0
0	0	0
0	0	0
0	0	0
0	0	0
0	0	0
0	0	0
0	0	0
0	0	0
0	0	0
1	1	0
1	1	0
0	0	0
0	0	0
0	0	0
0	0	0
0	0	0
0	0	0
0	0	0
0	0	0
0	0	0
0	0	0
0	0	0
0	0	0
1	1	0
0	0	0
1	1	0
1	1	0
1	1	0
0	0	0

0	0	0
0	0	0
0	0	0
0	0	0
0	0	0
0	0	0
0	0	0
0	0	0
0	0	0
0	0	0
1	1	0
1	1	0
0	0	0
0	0	0
0	0	0
0	0	0
0	0	0
0	0	0
0	0	0
0	0	0
0	0	0
0	0	0
1	1	0
0	0	0
0	0	0
0	0	0
0	0	0
0	0	0
0	0	0
0	0	0
0	0	0
0	0	0
0	0	0
1	1	0
0	0	0
0	0	0
1	1	0
1	1	0
1	1	0
0	0	0
0	0	0
0	0	0
0	0	0
0	0	0
0	0	0
0	0	0
0	0	0

0	0	0
0	0	0
0	0	0
0	0	0
0	0	0
0	0	0
1	1	0
0	0	0
0	0	0
0	0	0
0	0	0
0	0	0
0	0	0
0	0	0
0	0	0
0	0	0
0	0	0
0	0	0
0	0	0
0	0	0
0	0	0
1	1	0
1	1	0
0	0	0
0	0	0
0	0	0
0	0	0
0	0	0
0	0	0
0	0	0
0	0	0
0	0	0
0	0	0
0	0	0
0	0	0
0	0	0
0	0	0
0	0	0
0	0	0
1	1	0
1	1	0
0	0	0
0	0	0
0	0	0
0	0	0
0	0	0
0	0	0

0	0	0
0	0	0
0	0	0
0	0	0
1	1	0
1	1	0
0	0	0
0	0	0
0	0	0
0	0	0
0	0	0
1	0	0
1	0	0
0	0	0
0	0	0
0	0	0
0	0	0
0	0	0
0	0	0
0	0	0
0	0	0
0	0	1
0	0	1
0	0	1
0	0	0
0	0	0
0	0	0
0	0	0
0	0	0
0	0	0
1	1	0
1	1	0
1	1	0
1	1	0
1	1	0
0	0	0
0	0	0
0	0	0
0	0	0
0	0	0
0	0	0
0	0	0
0	0	0
0	0	0
0	0	0
0	0	0
0	0	0

0	0	0
0	0	0
0	0	0
0	0	0
0	0	0
0	0	0
0	0	0
0	0	1
0	0	1
0	0	1
1	0	1
1	0	1
1	0	1
1	0	1
0	0	0
0	0	0
0	0	0
0	0	0
0	0	0
1	1	0
0	0	0
0	0	0
0	0	0
0	0	0
0	0	0
0	0	0
0	0	0
0	0	0
0	0	0
0	0	0
0	0	0
0	0	0
0	0	0
0	0	0
0	0	0
0	0	0
0	0	0
0	0	0
0	0	0
0	0	0
0	0	0
0	0	0
0	0	0
1	0	0
1	0	0
1	0	0
0	0	0

0	0	0
0	0	0
1	1	0
0	0	0
0	0	0
1	1	0
1	1	0
0	0	1
0	0	1
0	0	0
0	0	0
0	0	0
0	0	0
0	0	0
1	1	0
1	1	0
0	0	0
0	0	0
0	0	0
0	0	0
0	0	0
0	0	0
1	1	0
1	1	0
0	0	1
0	0	0
0	0	0
0	0	0
0	0	0
0	0	0
0	0	0
0	0	0
0	0	0
0	0	0
0	0	0
1	1	0
0	0	0
0	0	0
0	0	0
0	0	0
0	0	0
0	0	0
0	0	1
1	0	1
1	0	1
1	0	1
1	0	1

1	1	0
1	1	0
0	0	0
0	0	0
0	0	0
0	0	0
1	1	0
0	0	0
0	0	0
1	1	0
1	1	0
0	0	0
1	1	0
1	1	0
0	0	0
0	0	0
0	0	0
0	0	0
0	0	0
0	0	0
0	0	0
0	0	0
0	0	0
0	0	0
0	0	0
0	0	0
0	0	0
0	0	0
0	0	0
0	0	0
0	0	0
0	0	0
1	1	0
1	1	0
1	1	1
1	1	1
0	0	0
0	0	0
0	0	0
1	1	0
1	1	0
1	1	0
0	0	0
0	0	0
0	0	0
0	0	0
0	0	0

0	0	0
0	0	0
1	1	0
1	1	0
0	0	0
0	0	0
0	0	0
0	0	0
0	0	0
0	0	0
0	0	0
0	0	0
0	0	0
0	0	0
0	0	0
0	0	0
0	0	0
0	0	0
0	0	0
0	0	0
0	0	0
0	0	0
0	0	0
0	0	0
0	0	0
0	0	0
0	0	0
0	0	0
0	0	0
0	0	0
0	0	0
0	0	0
0	0	0
0	0	0
0	0	0
0	0	0
0	0	0
0	0	0
0	0	0
0	0	0
1	1	0
1	1	0
1	1	0
0	0	0
0	0	0
0	0	0
0	0	0

0	0	0
1	1	1
0	0	0
0	0	0
0	0	0
0	0	0
0	0	0
0	0	0
0	0	0
0	0	0
0	0	0
0	0	0
0	0	0
0	0	0
0	0	0
0	0	0
0	0	0
0	0	0
1	1	0
1	1	0
0	0	0
0	0	0
0	0	0
0	0	0
1	1	0
0	0	0
0	0	0
0	0	1
0	0	1
0	0	0
0	0	0
0	0	0
0	0	0
1	1	0
0	0	0
0	0	0
0	0	0
0	0	0
0	0	0
0	0	0
1	0	0
1	0	0
1	0	0
0	0	0
1	1	0
1	1	0
0	0	0

0	0	0
1	1	0
1	1	0
1	1	0
0	0	0
0	0	0
0	0	0
0	0	0
0	0	0
0	0	0
0	0	0
0	0	0
0	0	0
0	0	0
0	0	0
0	0	0
1	1	0
1	1	0
0	0	0
0	0	0
0	0	0
0	0	0
0	0	0
0	0	0
0	0	0
0	0	0
0	0	0
0	0	0
0	0	0
0	0	0
1	1	0
1	1	0
1	0	0
1	0	0
1	0	0
0	0	0
0	0	0
0	0	0
0	0	0
0	0	0
0	0	0
0	0	0
0	0	0
0	0	0
0	0	0
0	0	0
0	0	0

0	0	0
0	0	0
0	0	0
0	0	0
0	0	0
0	0	0
0	0	0
0	0	0
0	0	0
0	0	0
0	0	0
0	0	0
0	0	1
0	0	1
0	0	1
1	1	0
1	1	0
1	1	0
0	0	0
0	0	0
1	1	0
1	1	0
1	1	0
0	0	0
0	0	0
0	0	0
0	0	0
0	0	0
0	0	0
0	0	0
0	0	0
0	0	0
0	0	0
0	0	0
0	0	0
0	0	0
0	0	0
0	0	0
0	0	0
0	0	0
0	0	0
0	0	0
0	0	0
0	0	0
0	0	0
0	0	0
0	0	0

0	0	0
0	0	0
0	0	0
0	0	0
0	0	0
0	0	0
0	0	0
0	0	0
0	0	0
0	0	0
0	0	0
1	1	1
0	0	0
0	0	0
0	0	0
0	0	0
0	0	0
0	0	0
1	0	1
1	0	1
1	0	1
1	0	1
0	0	0
0	0	0
0	0	0
0	0	0
0	0	0
0	0	0
0	0	0
0	0	0
0	0	0
0	0	0

ewagostcit_b	only_ewagost	only_ewagost	nop_lostart	lost_art	lost_art_b	lost3_art	lost3_art_b
0		0			0		0
0		0			0	LOST-3_art	1
0		0		#N/A	0	#N/A	0
0		0		#N/A	0	#N/A	0
0		0		LOST_art	1	LOST-3_art	1
0		0		LOST_art	1	LOST-3_art	1
0		0		LOST_art	1	LOST-3_art	1
0		0		LOST_art	1	LOST-3_art	1
0		0		LOST_art	1	LOST-3_art	1
0		0		LOST_art	1	LOST-3_art	1
1		1		#N/A	0	#N/A	0
0		0		#N/A	0	#N/A	0
0		0		#N/A	0	#N/A	0
0		0			0		0
0		0		#N/A	0	#N/A	0
0		0		#N/A	0	#N/A	0
0		0		#N/A	0	#N/A	0
0		0		#N/A	0	#N/A	0
0		0		LOST_art	1	LOST-3_art	1
0		0		LOST_art	1	LOST-3_art	1
0		0		#N/A	0	#N/A	0
0		0		#N/A	0	#N/A	0
0		0		#N/A	0	#N/A	0
0		0		#N/A	0	#N/A	0
0		0		#N/A	0	#N/A	0
0		0		#N/A	0	#N/A	0
0		0		#N/A	0	#N/A	0
0		0		#N/A	0	#N/A	0
0		0		#N/A	0	#N/A	0
0		0			0	LOST-3_art	1
0		0			0		0
0		0		LOST_art	1	LOST-3_art	1
0		0		#N/A	0	#N/A	0
0		0		#N/A	0	#N/A	0
0		0		#N/A	0	#N/A	0
0		0		#N/A	0	#N/A	0
0		0		#N/A	0	#N/A	0
0		0		#N/A	0	#N/A	0
0		0		#N/A	0	#N/A	0
0		0		#N/A	0	#N/A	0
0		0		#N/A	0	#N/A	0
0		0		#N/A	0	#N/A	0
0		0		#N/A	0	#N/A	0
0		0		#N/A	0	#N/A	0
0		0		#N/A	0	#N/A	0
0		0		#N/A	0	#N/A	0

0	0	#N/A	0	#N/A	0
0	0	#N/A	0	#N/A	0
1	1	#N/A	0	#N/A	0
0	0	#N/A	0	#N/A	0
0	0	#N/A	0	#N/A	0
0	0	#N/A	0	#N/A	0
0	0	#N/A	0	#N/A	0
0	0		0		0
0	0		0	LOST-3_art	1
0	0	#N/A	0	#N/A	0
0	0	#N/A	0	#N/A	0
0	0	#N/A	0	#N/A	0
0	0		0		0
0	0	#N/A	0	#N/A	0
0	0	#N/A	0	#N/A	0
0	0	#N/A	0	#N/A	0
0	0	#N/A	0	#N/A	0
0	0	#N/A	0	#N/A	0
0	0	#N/A	0	#N/A	0
0	0	#N/A	0	#N/A	0
0	0		0		0
0	0		0		0
0	0	#N/A	0	#N/A	0
0	0		0		0
0	0	#N/A	0	#N/A	0
0	0	#N/A	0	#N/A	0
0	0	LOST_art	1	LOST-3_art	1
0	0	#N/A	0	#N/A	0
0	0	#N/A	0	#N/A	0
0	0	LOST_art	1	LOST-3_art	1
0	0	LOST_art	1	LOST-3_art	1
0	0	LOST_art	1	LOST-3_art	1
0	0	LOST_art	1	LOST-3_art	1
0	0	LOST_art	1	LOST-3_art	1
0	0	#N/A	0	#N/A	0
0	0	#N/A	0	#N/A	0
0	0	#N/A	0	#N/A	0
0	0	#N/A	0	#N/A	0
0	0	#N/A	0	#N/A	0
0	0	#N/A	0	#N/A	0
0	0	#N/A	0	#N/A	0
0	0	#N/A	0	#N/A	0
0	0	#N/A	0	#N/A	0
0	0	#N/A	0	#N/A	0
0	0	#N/A	0	#N/A	0
0	0	#N/A	0	#N/A	0
0	0	#N/A	0	#N/A	0

0	0	#N/A	0	#N/A	0
0	0	#N/A	0	#N/A	0
0	0	#N/A	0	#N/A	0
1	1	#N/A	0	#N/A	0
0	0	#N/A	0	#N/A	0
0	0	#N/A	0	#N/A	0
0	0	LOST_art	1	LOST-3_art	1
0	0	LOST_art	1	LOST-3_art	1
0	0	#N/A	0	#N/A	0
0	0	#N/A	0	#N/A	0
0	0	#N/A	0	#N/A	0
0	0	#N/A	0	#N/A	0
0	0	#N/A	0	#N/A	0
0	0	#N/A	0	#N/A	0
0	0	#N/A	0	#N/A	0
0	0	#N/A	0	#N/A	0
0	0	#N/A	0	#N/A	0
0	0	#N/A	0	#N/A	0
0	0	#N/A	0	#N/A	0
0	0	#N/A	0	#N/A	0
1	1	#N/A	0	#N/A	0
0	0	#N/A	0	#N/A	0
0	0	#N/A	0	#N/A	0
0	0	#N/A	0	#N/A	0
0	0	#N/A	0	#N/A	0
0	0	#N/A	0	#N/A	0
0	0	#N/A	0	#N/A	0
0	0		0	LOST-3_art	1
0	0		0	LOST-3_art	1
0	0	LOST_art	1	LOST-3_art	1
0	0	LOST_art	1	LOST-3_art	1
0	0	#N/A	0	#N/A	0
1	0	#N/A	0	#N/A	0
1	0	#N/A	0	#N/A	0
1	1	#N/A	0	#N/A	0
0	0	LOST_art	1	LOST-3_art	1
0	0	LOST_art	1	LOST-3_art	1
0	0		0	LOST-3_art	1
0	0		0	LOST-3_art	1
1	0	#N/A	0	#N/A	0
0	0	#N/A	0	#N/A	0
0	0	#N/A	0	#N/A	0
0	0		0	LOST-3_art	1
0	0	#N/A	0	#N/A	0
0	0	LOST_art	1	LOST-3_art	1
0	0	LOST_art	1	LOST-3_art	1
0	0	#N/A	0	#N/A	0

0	0	#N/A	0	#N/A	0
0	0	#N/A	0	#N/A	0
1	1		0	LOST-3_art	1
1	1		0	LOST-3_art	1
1	1		0	LOST-3_art	1
0	0	#N/A	0	#N/A	0
0	0	#N/A	0	#N/A	0
0	0	#N/A	0	#N/A	0
0	0	#N/A	0	#N/A	0
0	0	#N/A	0	#N/A	0
0	0	#N/A	0	#N/A	0
0	0	#N/A	0	#N/A	0
0	0	#N/A	0	#N/A	0
0	0	LOST_art	1	LOST-3_art	1
0	0	#N/A	0	#N/A	0
0	0	#N/A	0	#N/A	0
1	1	LOST_art	1	LOST-3_art	1
0	0	#N/A	0	#N/A	0
0	0	#N/A	0	#N/A	0
0	0	#N/A	0	#N/A	0
0	0	#N/A	0	#N/A	0
0	0	#N/A	0	#N/A	0
0	0		0		0
0	0	#N/A	0	#N/A	0
0	0	#N/A	0	#N/A	0
0	0	#N/A	0	#N/A	0
0	0	#N/A	0	#N/A	0
1	1	#N/A	0	#N/A	0
0	0	LOST_art	1	LOST-3_art	1
0	0	#N/A	0	#N/A	0
0	0	LOST_art	1	LOST-3_art	1
0	0	LOST_art	1	LOST-3_art	1
0	0	#N/A	0	#N/A	0
0	0	#N/A	0	#N/A	0
0	0	#N/A	0	#N/A	0
0	0	#N/A	0	#N/A	0
0	0	#N/A	0	#N/A	0
0	0	#N/A	0	#N/A	0
1	0	#N/A	0	#N/A	0
0	0	#N/A	0	#N/A	0
0	0	#N/A	0	#N/A	0
0	0	LOST_art	1	LOST-3_art	1
0	0	LOST_art	1	LOST-3_art	1
0	0	#N/A	0	#N/A	0
0	0	#N/A	0	#N/A	0
0	0	#N/A	0	#N/A	0
0	0	#N/A	0	#N/A	0

0	0	#N/A	0	#N/A	0
0	0	#N/A	0	#N/A	0
0	0	#N/A	0	#N/A	0
0	0	#N/A	0	#N/A	0
0	0	#N/A	0	#N/A	0
0	0	#N/A	0	#N/A	0
0	0	#N/A	0	#N/A	0
0	0	#N/A	0	#N/A	0
0	0	#N/A	0	#N/A	0
0	0	#N/A	0	#N/A	0
0	0	#N/A	0	#N/A	0
0	0	#N/A	0	#N/A	0
0	0	#N/A	0	#N/A	0
0	0	#N/A	0	#N/A	0
0	0	#N/A	0	#N/A	0
0	0		0	LOST-3_art	1
0	0		0	LOST-3_art	1
0	0	#N/A	0	#N/A	0
0	0	#N/A	0	#N/A	0
0	0	#N/A	0	#N/A	0
0	0	#N/A	0	#N/A	0
0	0	#N/A	0	#N/A	0
0	0	#N/A	0	#N/A	0
0	0	#N/A	0	#N/A	0
1	1	LOST_art	1	LOST-3_art	1
1	1	LOST_art	1	LOST-3_art	1
0	0	#N/A	0	#N/A	0
0	0	#N/A	0	#N/A	0
0	0	#N/A	0	#N/A	0
0	0	#N/A	0	#N/A	0
0	0	#N/A	0	#N/A	0
0	0	#N/A	0	#N/A	0
0	0	#N/A	0	#N/A	0
0	0	#N/A	0	#N/A	0
0	0		0		0
0	0	#N/A	0	#N/A	0
0	0	#N/A	0	#N/A	0
0	0		0		0
1	1	LOST_art	1	LOST-3_art	1
1	1	LOST_art	1	LOST-3_art	1
1	1	LOST_art	1	LOST-3_art	1
1	1	LOST_art	1	LOST-3_art	1
0	0	#N/A	0	#N/A	0
0	0	#N/A	0	#N/A	0
0	0	#N/A	0	#N/A	0
0	0	#N/A	0	#N/A	0
0	0	#N/A	0	#N/A	0

0	0	#N/A	0	#N/A	0
0	0	#N/A	0	#N/A	0
0	0	#N/A	0	#N/A	0
0	0	#N/A	0	#N/A	0
0	0	#N/A	0	#N/A	0
0	0	#N/A	0	#N/A	0
0	0	#N/A	0	#N/A	0
0	0	#N/A	0	#N/A	0
0	0	#N/A	0	#N/A	0
0	0		0	LOST-3_art	1
0	0		0		0
0	0	#N/A	0	#N/A	0
0	0	#N/A	0	#N/A	0
0	0	#N/A	0	#N/A	0
0	0	#N/A	0	#N/A	0
0	0		0		0
0	0	#N/A	0	#N/A	0
0	0	#N/A	0	#N/A	0
0	0	#N/A	0	#N/A	0
0	0	#N/A	0	#N/A	0
0	0	#N/A	0	#N/A	0
1	0	LOST_art	1	LOST-3_art	1
1	0	LOST_art	1	LOST-3_art	1
1	0	LOST_art	1	LOST-3_art	1
0	0	#N/A	0	#N/A	0
0	0	#N/A	0	#N/A	0
0	0	#N/A	0	#N/A	0
0	0	#N/A	0	#N/A	0
0	0	#N/A	0	#N/A	0
0	0	#N/A	0	#N/A	0
1	0	LOST_art	1	LOST-3_art	1
1	0	LOST_art	1	LOST-3_art	1
1	0	LOST_art	1	LOST-3_art	1
0	0	#N/A	0	#N/A	0
0	0	#N/A	0	#N/A	0
0	0	#N/A	0	#N/A	0
0	0	#N/A	0	#N/A	0
0	0	#N/A	0	#N/A	0
1	1	#N/A	0	#N/A	0
1	1	#N/A	0	#N/A	0
0	0	#N/A	0	#N/A	0
0	0	#N/A	0	#N/A	0
0	0	#N/A	0	#N/A	0
0	0		0		0
0	0	#N/A	0	#N/A	0
0	0	#N/A	0	#N/A	0
0	0	#N/A	0	#N/A	0

0	0	#N/A	0	#N/A	0
0	0	#N/A	0	#N/A	0
1	1	#N/A	0	#N/A	0
0	0		0		0
0	0	#N/A	0	#N/A	0
0	0	#N/A	0	#N/A	0
0	0	#N/A	0	#N/A	0
0	0	#N/A	0	#N/A	0
0	0	#N/A	0	#N/A	0
1	1	#N/A	0	#N/A	0
1	1	#N/A	0	#N/A	0
0	0	#N/A	0	#N/A	0
0	0	#N/A	0	#N/A	0
0	0	#N/A	0	#N/A	0
0	0	#N/A	0	#N/A	0
0	0		0		0
0	0		0		0
0	0	#N/A	0	#N/A	0
0	0	#N/A	0	#N/A	0
0	0	#N/A	0	#N/A	0
0	0	#N/A	0	#N/A	0
0	0	#N/A	0	#N/A	0
0	0		0	LOST-3_art	1
0	0	#N/A	0	#N/A	0
0	0	#N/A	0	#N/A	0
1	1	#N/A	0	#N/A	0
1	1	#N/A	0	#N/A	0
1	1	#N/A	0	#N/A	0
1	1	#N/A	0	#N/A	0
1	1	#N/A	0	#N/A	0
0	0	#N/A	0	#N/A	0
0	0	#N/A	0	#N/A	0
1	1	#N/A	0	#N/A	0
0	0	#N/A	0	#N/A	0
0	0	LOST_art	1	LOST-3_art	1
0	0	LOST_art	1	LOST-3_art	1
0	0	#N/A	0	#N/A	0
0	0		0		0
0	0	#N/A	0	#N/A	0
0	0	#N/A	0	#N/A	0
0	0	#N/A	0	#N/A	0
0	0	#N/A	0	#N/A	0
0	0	#N/A	0	#N/A	0
0	0	#N/A	0	#N/A	0
0	0	#N/A	0	#N/A	0
0	0	#N/A	0	#N/A	0
0	0	#N/A	0	#N/A	0

0	0	#N/A	0	#N/A	0
0	0		0	LOST-3_art	1
0	0		0	LOST-3_art	1
0	0	#N/A	0	#N/A	0
0	0	#N/A	0	#N/A	0
0	0	#N/A	0	#N/A	0
0	0	#N/A	0	#N/A	0
0	0	#N/A	0	#N/A	0
0	0	#N/A	0	#N/A	0
0	0	#N/A	0	#N/A	0
0	0	#N/A	0	#N/A	0
0	0	#N/A	0	#N/A	0
0	0	#N/A	0	#N/A	0
0	0		0	LOST-3_art	1
0	0	LOST_art	1	LOST-3_art	1
0	0	LOST_art	1	LOST-3_art	1
0	0		0		0
0	0	#N/A	0	#N/A	0
0	0		0	LOST-3_art	1
0	0	#N/A	0	#N/A	0
0	0	#N/A	0	#N/A	0
0	0		0		0
0	0	#N/A	0	#N/A	0
0	0	#N/A	0	#N/A	0
1	0	#N/A	0	#N/A	0
1	0	#N/A	0	#N/A	0
0	0		0		0
0	0	#N/A	0	#N/A	0
0	0	#N/A	0	#N/A	0
0	0	#N/A	0	#N/A	0
0	0	#N/A	0	#N/A	0
0	0	#N/A	0	#N/A	0
0	0	#N/A	0	#N/A	0
0	0	#N/A	0	#N/A	0
0	0	#N/A	0	#N/A	0
0	0	#N/A	0	#N/A	0
0	0		0		0
0	0	#N/A	0	#N/A	0
0	0	#N/A	0	#N/A	0
0	0	#N/A	0	#N/A	0
1	1	#N/A	0	#N/A	0
0	0	#N/A	0	#N/A	0
0	0	#N/A	0	#N/A	0
0	0	LOST_art	1	LOST-3_art	1
0	0	#N/A	0	#N/A	0
0	0	#N/A	0	#N/A	0
0	0	#N/A	0	#N/A	0

0	0	#N/A	0	#N/A	0
0	0		0		0
0	0	#N/A	0	#N/A	0
0	0	#N/A	0	#N/A	0
0	0	#N/A	0	#N/A	0
0	0		0		0
0	0		0		0
1	0	LOST_art	1	LOST-3_art	1
1	0	LOST_art	1	LOST-3_art	1
0	0	#N/A	0	#N/A	0
0	0	#N/A	0	#N/A	0
0	0	#N/A	0	#N/A	0
0	0	#N/A	0	#N/A	0
0	0	#N/A	0	#N/A	0
0	0	#N/A	0	#N/A	0
0	0	#N/A	0	#N/A	0
0	0	#N/A	0	#N/A	0
0	0	#N/A	0	#N/A	0
0	0	#N/A	0	#N/A	0
0	0	#N/A	0	#N/A	0
0	0	#N/A	0	#N/A	0
1	1	LOST_art	1	LOST-3_art	1
0	0	#N/A	0	#N/A	0
0	0	#N/A	0	#N/A	0
0	0	#N/A	0	#N/A	0
0	0	#N/A	0	#N/A	0
0	0	#N/A	0	#N/A	0
0	0		0		0
0	0	#N/A	0	#N/A	0
0	0	#N/A	0	#N/A	0
0	0	#N/A	0	#N/A	0
0	0	#N/A	0	#N/A	0
0	0	#N/A	0	#N/A	0
0	0	#N/A	0	#N/A	0
0	0	#N/A	0	#N/A	0
0	0	#N/A	0	#N/A	0
0	0	#N/A	0	#N/A	0
0	0	LOST_art	1	LOST-3_art	1
0	0	#N/A	0	#N/A	0
0	0	#N/A	0	#N/A	0
0	0	#N/A	0	#N/A	0
0	0	#N/A	0	#N/A	0
0	0	#N/A	0	#N/A	0
0	0	#N/A	0	#N/A	0
0	0	#N/A	0	#N/A	0
0	0	LOST_art	1	LOST-3_art	1
0	0	LOST_art	1	LOST-3_art	1

0	0	#N/A	0	#N/A	0
0	0	#N/A	0	#N/A	0
0	0	#N/A	0	#N/A	0
0	0	#N/A	0	#N/A	0
0	0	#N/A	0	#N/A	0
0	0	LOST_art	1	LOST-3_art	1
0	0	LOST_art	1	LOST-3_art	1
0	0	#N/A	0	#N/A	0
1	1	#N/A	0	#N/A	0
0	0	#N/A	0	#N/A	0
0	0	#N/A	0	#N/A	0
0	0		0	LOST-3_art	1
0	0		0	LOST-3_art	1
0	0	#N/A	0	#N/A	0
0	0		0		0
0	0	LOST_art	1	LOST-3_art	1
0	0	LOST_art	1	LOST-3_art	1
0	0	LOST_art	1	LOST-3_art	1
0	0	LOST_art	1	LOST-3_art	1
0	0	#N/A	0	#N/A	0
0	0	#N/A	0	#N/A	0
0	0	#N/A	0	#N/A	0
0	0	#N/A	0	#N/A	0
0	0	#N/A	0	#N/A	0
0	0		0		0
0	0		0		0
0	0	#N/A	0	#N/A	0
0	0	#N/A	0	#N/A	0
0	0		0	LOST-3_art	1
0	0	#N/A	0	#N/A	0
0	0	#N/A	0	#N/A	0
0	0	#N/A	0	#N/A	0
0	0	#N/A	0	#N/A	0
0	0	#N/A	0	#N/A	0
0	0	#N/A	0	#N/A	0
0	0	#N/A	0	#N/A	0
0	0	#N/A	0	#N/A	0
0	0	#N/A	0	#N/A	0
0	0	#N/A	0	#N/A	0
0	0	#N/A	0	#N/A	0
0	0	#N/A	0	#N/A	0
0	0	#N/A	0	#N/A	0
0	0	#N/A	0	#N/A	0
0	0	#N/A	0	#N/A	0
0	0	#N/A	0	#N/A	0
0	0	#N/A	0	#N/A	0
0	0	#N/A	0	#N/A	0

0	0		0	LOST-3_art	1
0	0		0	LOST-3_art	1
0	0	#N/A	0	#N/A	0
0	0	#N/A	0	#N/A	0
0	0	#N/A	0	#N/A	0
0	0	#N/A	0	#N/A	0
0	0		0	LOST-3_art	1
0	0	#N/A	0	#N/A	0
0	0	#N/A	0	#N/A	0
0	0		0		0
1	1	#N/A	0	#N/A	0
1	1	#N/A	0	#N/A	0
1	1	#N/A	0	#N/A	0
0	0	#N/A	0	#N/A	0
0	0	#N/A	0	#N/A	0
0	0	#N/A	0	#N/A	0
0	0	#N/A	0	#N/A	0
0	0	#N/A	0	#N/A	0
0	0	#N/A	0	#N/A	0
0	0	#N/A	0	#N/A	0
0	0	#N/A	0	#N/A	0
0	0	#N/A	0	#N/A	0
0	0		0	LOST-3_art	1
0	0	LOST_art	1	LOST-3_art	1
0	0	LOST_art	1	LOST-3_art	1
0	0	LOST_art	1	LOST-3_art	1
0	0	#N/A	0	#N/A	0
0	0	#N/A	0	#N/A	0
0	0	#N/A	0	#N/A	0
0	0	#N/A	0	#N/A	0
0	0	#N/A	0	#N/A	0
0	0	#N/A	0	#N/A	0
0	0	#N/A	0	#N/A	0
0	0	#N/A	0	#N/A	0
0	0	LOST_art	1	LOST-3_art	1
0	0	#N/A	0	#N/A	0
0	0	#N/A	0	#N/A	0
0	0	#N/A	0	#N/A	0
0	0	#N/A	0	#N/A	0
0	0	#N/A	0	#N/A	0
1	1	#N/A	0	#N/A	0
0	0		0		0
0	0	#N/A	0	#N/A	0
0	0		0	LOST-3_art	1
0	0	#N/A	0	#N/A	0
0	0	#N/A	0	#N/A	0
0	0	#N/A	0	#N/A	0

0	0	#N/A	0	#N/A	0
0	0		0		0
0	0	#N/A	0	#N/A	0
0	0	#N/A	0	#N/A	0
0	0	#N/A	0	#N/A	0
0	0	#N/A	0	#N/A	0
0	0	#N/A	0	#N/A	0
0	0	#N/A	0	#N/A	0
0	0	#N/A	0	#N/A	0
0	0	#N/A	0	#N/A	0
0	0	LOST_art	1	LOST-3_art	1
0	0	LOST_art	1	LOST-3_art	1
0	0	LOST_art	1	LOST-3_art	1
0	0	#N/A	0	#N/A	0
0	0	#N/A	0	#N/A	0
0	0	#N/A	0	#N/A	0
0	0	#N/A	0	#N/A	0
0	0	#N/A	0	#N/A	0
0	0	#N/A	0	#N/A	0
0	0		0		0
0	0	#N/A	0	#N/A	0
0	0	#N/A	0	#N/A	0
0	0	#N/A	0	#N/A	0
0	0	#N/A	0	#N/A	0
0	0	#N/A	0	#N/A	0
0	0	#N/A	0	#N/A	0
0	0	#N/A	0	#N/A	0
0	0	#N/A	0	#N/A	0
0	0	#N/A	0	#N/A	0
0	0	#N/A	0	#N/A	0
0	0	#N/A	0	#N/A	0
0	0	#N/A	0	#N/A	0
0	0	#N/A	0	#N/A	0
0	0	#N/A	0	#N/A	0
0	0	#N/A	0	#N/A	0
0	0	#N/A	0	#N/A	0
0	0		0		0
0	0	#N/A	0	#N/A	0
0	0	#N/A	0	#N/A	0
0	0		0		0
0	0	#N/A	0	#N/A	0
0	0	#N/A	0	#N/A	0
0	0	#N/A	0	#N/A	0
0	0	#N/A	0	#N/A	0
1	1	#N/A	0	#N/A	0
0	0	#N/A	0	#N/A	0
0	0	#N/A	0	#N/A	0

1	1	#N/A	0	#N/A	0
1	1	#N/A	0	#N/A	0
0	0	#N/A	0	#N/A	0
0	0	#N/A	0	#N/A	0
0	0	#N/A	0	#N/A	0
0	0	#N/A	0	#N/A	0
0	0	#N/A	0	#N/A	0
0	0	#N/A	0	#N/A	0
0	0	#N/A	0	#N/A	0
0	0	#N/A	0	#N/A	0
0	0	#N/A	0	#N/A	0
0	0	#N/A	0	#N/A	0
0	0	#N/A	0	#N/A	0
0	0	#N/A	0	#N/A	0
0	0	#N/A	0	#N/A	0
0	0	#N/A	0	#N/A	0
0	0	#N/A	0	#N/A	0
0	0	#N/A	0	#N/A	0
0	0	#N/A	0	#N/A	0
0	0	#N/A	0	#N/A	0
0	0	#N/A	0	#N/A	0
0	0	#N/A	0	#N/A	0
0	0	#N/A	0	#N/A	0
0	0		0	LOST-3_art	1
0	0	#N/A	0	#N/A	0
0	0	#N/A	0	#N/A	0
0	0	#N/A	0	#N/A	0
0	0	#N/A	0	#N/A	0
0	0	#N/A	0	#N/A	0
0	0	LOST_art	1	LOST-3_art	1
0	0	#N/A	0	#N/A	0
0	0	#N/A	0	#N/A	0
0	0	#N/A	0	#N/A	0
0	0	#N/A	0	#N/A	0
0	0	#N/A	0	#N/A	0
0	0	#N/A	0	#N/A	0
0	0	#N/A	0	#N/A	0
1	1	#N/A	0	#N/A	0
1	1	#N/A	0	#N/A	0
0	0		0		0
0	0	#N/A	0	#N/A	0
0	0	#N/A	0	#N/A	0
0	0	#N/A	0	#N/A	0
0	0	#N/A	0	#N/A	0
0	0	#N/A	0	#N/A	0
0	0	#N/A	0	#N/A	0
0	0	#N/A	0	#N/A	0

0	0		0		0
0	0		0	LOST-3_art	1
0	0	LOST_art	1	LOST-3_art	1
0	0	LOST_art	1	LOST-3_art	1
0	0	#N/A	0	#N/A	0
0	0	#N/A	0	#N/A	0
0	0	LOST_art	1	LOST-3_art	1
0	0	LOST_art	1	LOST-3_art	1
0	0	LOST_art	1	LOST-3_art	1
0	0	LOST_art	1	LOST-3_art	1
0	0	#N/A	0	#N/A	0
0	0	#N/A	0	#N/A	0
0	0	#N/A	0	#N/A	0
0	0	#N/A	0	#N/A	0
0	0	#N/A	0	#N/A	0
0	0	#N/A	0	#N/A	0
0	0	#N/A	0	#N/A	0
0	0		0	LOST-3_art	1
0	0		0	LOST-3_art	1
0	0	#N/A	0	#N/A	0
0	0	#N/A	0	#N/A	0
0	0	#N/A	0	#N/A	0
0	0	#N/A	0	#N/A	0
0	0	#N/A	0	#N/A	0
0	0	#N/A	0	#N/A	0
0	0	#N/A	0	#N/A	0
0	0	#N/A	0	#N/A	0
0	0	#N/A	0	#N/A	0
0	0	#N/A	0	#N/A	0
0	0	#N/A	0	#N/A	0
0	0	#N/A	0	#N/A	0
0	0	#N/A	0	#N/A	0
0	0	#N/A	0	#N/A	0
0	0	#N/A	0	#N/A	0
0	0	#N/A	0	#N/A	0
0	0	#N/A	0	#N/A	0
1	0	#N/A	0	#N/A	0
1	0	#N/A	0	#N/A	0
0	0	#N/A	0	#N/A	0
0	0	#N/A	0	#N/A	0
0	0	#N/A	0	#N/A	0
0	0	#N/A	0	#N/A	0
0	0	#N/A	0	#N/A	0
0	0	#N/A	0	#N/A	0
0	0	#N/A	0	#N/A	0
0	0	#N/A	0	#N/A	0
0	0	#N/A	0	#N/A	0

0	0	#N/A	0	#N/A	0
0	0	#N/A	0	#N/A	0
0	0	LOST_art	1	LOST-3_art	1
0	0	LOST_art	1	LOST-3_art	1
0	0	#N/A	0	#N/A	0
0	0	#N/A	0	#N/A	0
0	0	#N/A	0	#N/A	0
0	0	#N/A	0	#N/A	0
0	0		0		0
0	0		0		0
0	0	LOST_art	1	LOST-3_art	1
0	0	LOST_art	1	LOST-3_art	1
0	0	#N/A	0	#N/A	0
0	0	#N/A	0	#N/A	0
1	1	#N/A	0	#N/A	0
0	0	#N/A	0	#N/A	0
0	0		0	LOST-3_art	1
0	0		0	LOST-3_art	1
0	0		0		0
0	0	#N/A	0	#N/A	0
0	0		0	LOST-3_art	1
0	0		0	LOST-3_art	1
0	0	#N/A	0	#N/A	0
0	0	#N/A	0	#N/A	0
0	0	#N/A	0	#N/A	0
0	0	#N/A	0	#N/A	0
0	0	#N/A	0	#N/A	0
0	0	#N/A	0	#N/A	0
0	0	#N/A	0	#N/A	0
0	0	#N/A	0	#N/A	0
0	0		0		0
0	0		0		0
0	0	#N/A	0	#N/A	0
0	0	LOST_art	1	LOST-3_art	1
0	0	LOST_art	1	LOST-3_art	1
0	0	LOST_art	1	LOST-3_art	1
0	0	LOST_art	1	LOST-3_art	1
0	0	LOST_art	1	LOST-3_art	1
0	0	LOST_art	1	LOST-3_art	1
0	0	#N/A	0	#N/A	0
0	0	#N/A	0	#N/A	0
0	0		0	LOST-3_art	1
0	0		0	LOST-3_art	1
0	0		0	LOST-3_art	1
0	0	#N/A	0	#N/A	0
0	0	#N/A	0	#N/A	0
0	0	#N/A	0	#N/A	0

0	0	#N/A	0	#N/A	0
0	0	#N/A	0	#N/A	0
0	0	LOST_art	1	LOST-3_art	1
0	0	LOST_art	1	LOST-3_art	1
0	0	#N/A	0	#N/A	0
0	0		0	LOST-3_art	1
0	0		0	LOST-3_art	1
0	0	#N/A	0	#N/A	0
1	0	LOST_art	1	LOST-3_art	1
0	0	#N/A	0	#N/A	0
0	0		0	LOST-3_art	1
0	0		0	LOST-3_art	1
0	0		0	LOST-3_art	1
0	0		0		0
0	0		0		0
0	0	#N/A	0	#N/A	0
0	0	#N/A	0	#N/A	0
0	0	#N/A	0	#N/A	0
0	0	#N/A	0	#N/A	0
0	0	#N/A	0	#N/A	0
0	0	#N/A	0	#N/A	0
1	0	LOST_art	1	LOST-3_art	1
1	0	LOST_art	1	LOST-3_art	1
0	0	LOST_art	1	LOST-3_art	1
0	0	#N/A	0	#N/A	0
0	0	#N/A	0	#N/A	0
0	0	#N/A	0	#N/A	0
0	0	LOST_art	1	LOST-3_art	1
0	0	#N/A	0	#N/A	0
0	0		0	LOST-3_art	1
0	0		0	LOST-3_art	1
1	1	#N/A	0	#N/A	0
1	1	#N/A	0	#N/A	0
0	0	#N/A	0	#N/A	0
0	0	#N/A	0	#N/A	0
0	0		0		0
0	0	#N/A	0	#N/A	0
0	0		0		0
0	0	#N/A	0	#N/A	0
0	0	#N/A	0	#N/A	0
0	0	LOST_art	1	LOST-3_art	1
0	0		0		0
0	0	#N/A	0	#N/A	0
0	0	#N/A	0	#N/A	0
0	0	#N/A	0	#N/A	0
0	0	#N/A	0	#N/A	0
0	0	#N/A	0	#N/A	0

0	0		0	LOST-3_art	1
0	0		0		0
0	0		0		0
0	0	#N/A	0	#N/A	0
0	0		0	LOST-3_art	1
0	0		0	LOST-3_art	1
0	0		0	LOST-3_art	1
0	0		0	LOST-3_art	1
0	0		0	LOST-3_art	1
0	0	#N/A	0	#N/A	0
0	0	#N/A	0	#N/A	0
0	0	LOST_art	1	LOST-3_art	1
0	0	LOST_art	1	LOST-3_art	1
0	0	#N/A	0	#N/A	0
0	0		0		0
0	0	#N/A	0	#N/A	0
0	0	#N/A	0	#N/A	0
0	0	#N/A	0	#N/A	0
0	0	#N/A	0	#N/A	0
0	0	#N/A	0	#N/A	0
0	0	#N/A	0	#N/A	0
0	0	#N/A	0	#N/A	0
0	0	#N/A	0	#N/A	0
0	0	#N/A	0	#N/A	0
0	0	#N/A	0	#N/A	0
0	0	#N/A	0	#N/A	0
0	0	#N/A	0	#N/A	0
0	0	#N/A	0	#N/A	0
0	0	#N/A	0	#N/A	0
0	0	#N/A	0	#N/A	0
0	0	#N/A	0	#N/A	0
0	0	#N/A	0	#N/A	0
0	0	#N/A	0	#N/A	0
0	0	#N/A	0	#N/A	0
0	0	#N/A	0	#N/A	0
0	0	#N/A	0	#N/A	0
0	0	#N/A	0	#N/A	0
0	0		0		0
0	0	#N/A	0	#N/A	0
0	0		0		0
0	0		0		0
0	0	#N/A	0	#N/A	0
0	0	LOST_art	1	LOST-3_art	1
0	0	LOST_art	1	LOST-3_art	1
0	0	#N/A	0	#N/A	0
1	1	#N/A	0	#N/A	0
0	0	#N/A	0	#N/A	0

0	0	#N/A	0	#N/A	0
0	0	#N/A	0	#N/A	0
0	0	#N/A	0	#N/A	0
0	0	#N/A	0	#N/A	0
0	0	#N/A	0	#N/A	0
0	0	#N/A	0	#N/A	0
1	0	#N/A	0	#N/A	0
1	0	#N/A	0	#N/A	0
0	0	#N/A	0	#N/A	0
0	0	#N/A	0	#N/A	0
0	0	#N/A	0	#N/A	0
0	0	#N/A	0	#N/A	0
0	0	#N/A	0	#N/A	0
0	0	#N/A	0	#N/A	0
0	0	#N/A	0	#N/A	0
0	0	#N/A	0	#N/A	0
0	0	#N/A	0	#N/A	0
0	0	LOST_art	1	LOST-3_art	1
0	0	LOST_art	1	LOST-3_art	1
0	0	LOST_art	1	LOST-3_art	1
0	0	LOST_art	1	LOST-3_art	1
0	0	LOST_art	1	LOST-3_art	1
0	0	LOST_art	1	LOST-3_art	1
0	0	LOST_art	1	LOST-3_art	1
0	0	#N/A	0	#N/A	0
0	0	#N/A	0	#N/A	0
0	0	LOST_art	1	LOST-3_art	1
0	0	LOST_art	1	LOST-3_art	1
0	0	LOST_art	1	LOST-3_art	1
0	0	#N/A	0	#N/A	0
0	0	#N/A	0	#N/A	0
0	0	#N/A	0	#N/A	0
0	0	#N/A	0	#N/A	0
0	0		0		0
0	0	#N/A	0	#N/A	0
0	0	#N/A	0	#N/A	0
1	0	#N/A	0	#N/A	0
1	0	#N/A	0	#N/A	0
0	0	#N/A	0	#N/A	0
1	1	#N/A	0	#N/A	0
1	1	#N/A	0	#N/A	0
0	0	#N/A	0	#N/A	0
0	0	#N/A	0	#N/A	0
0	0	#N/A	0	#N/A	0
0	0	#N/A	0	#N/A	0
0	0	#N/A	0	#N/A	0
0	0	#N/A	0	#N/A	0

0	0	#N/A	0	#N/A	0
0	0	#N/A	0	#N/A	0
0	0	#N/A	0	#N/A	0
0	0		0		0
0	0	#N/A	0	#N/A	0
0	0	#N/A	0	#N/A	0
0	0	#N/A	0	#N/A	0
1	1	#N/A	0	#N/A	0
0	0	#N/A	0	#N/A	0
0	0	#N/A	0	#N/A	0
0	0	#N/A	0	#N/A	0
0	0		0		0
0	0		0		0
1	1	#N/A	0	#N/A	0
0	0	#N/A	0	#N/A	0
0	0	#N/A	0	#N/A	0
0	0	#N/A	0	#N/A	0
0	0	#N/A	0	#N/A	0
0	0	#N/A	0	#N/A	0
0	0	#N/A	0	#N/A	0
0	0	#N/A	0	#N/A	0
0	0	#N/A	0	#N/A	0
0	0	#N/A	0	#N/A	0
0	0	#N/A	0	#N/A	0
0	0		0		0
0	0	#N/A	0	#N/A	0
0	0	#N/A	0	#N/A	0
0	0	#N/A	0	#N/A	0
0	0	#N/A	0	#N/A	0
0	0	#N/A	0	#N/A	0
0	0	#N/A	0	#N/A	0
0	0	#N/A	0	#N/A	0
0	0		0		0
0	0		0		0
0	0	#N/A	0	#N/A	0
0	0	#N/A	0	#N/A	0
0	0	#N/A	0	#N/A	0
0	0	#N/A	0	#N/A	0
0	0	#N/A	0	#N/A	0
0	0	#N/A	0	#N/A	0
0	0	#N/A	0	#N/A	0
0	0	#N/A	0	#N/A	0
0	0	#N/A	0	#N/A	0
0	0	LOST_art	1	LOST-3_art	1
0	0	LOST_art	1	LOST-3_art	1
0	0	#N/A	0	#N/A	0
0	0	#N/A	0	#N/A	0

0	0	#N/A	0	#N/A	0
0	0	#N/A	0	#N/A	0
0	0	#N/A	0	#N/A	0
0	0	#N/A	0	#N/A	0
0	0	#N/A	0	#N/A	0
0	0	LOST_art	1	LOST-3_art	1
0	0	#N/A	0	#N/A	0
0	0	#N/A	0	#N/A	0
0	0	#N/A	0	#N/A	0
0	0	#N/A	0	#N/A	0
0	0	LOST_art	1	LOST-3_art	1
0	0	#N/A	0	#N/A	0
1	1	#N/A	0	#N/A	0
1	1	#N/A	0	#N/A	0
0	0	#N/A	0	#N/A	0
0	0	#N/A	0	#N/A	0
0	0	#N/A	0	#N/A	0
0	0	#N/A	0	#N/A	0
0	0	#N/A	0	#N/A	0
0	0		0		0
0	0	LOST_art	1	LOST-3_art	1
0	0	LOST_art	1	LOST-3_art	1
0	0	#N/A	0	#N/A	0
0	0	#N/A	0	#N/A	0
0	0	#N/A	0	#N/A	0
0	0	#N/A	0	#N/A	0
0	0	#N/A	0	#N/A	0
0	0	#N/A	0	#N/A	0
0	0	#N/A	0	#N/A	0
0	0	#N/A	0	#N/A	0
0	0	#N/A	0	#N/A	0
0	0		0		0
0	0		0		0
0	0		0		0
0	0	#N/A	0	#N/A	0
0	0	#N/A	0	#N/A	0
1	1	#N/A	0	#N/A	0
0	0	#N/A	0	#N/A	0
1	0	LOST_art	1	LOST-3_art	1
1	0	LOST_art	1	LOST-3_art	1
0	0		0		0
0	0	#N/A	0	#N/A	0
0	0	#N/A	0	#N/A	0
0	0	#N/A	0	#N/A	0
0	0	#N/A	0	#N/A	0
0	0	#N/A	0	#N/A	0
0	0	#N/A	0	#N/A	0

0	0	#N/A	0	#N/A	0
0	0		0		0
0	0	#N/A	0	#N/A	0
0	0	#N/A	0	#N/A	0
0	0	#N/A	0	#N/A	0
0	0	#N/A	0	#N/A	0
0	0	#N/A	0	#N/A	0
0	0	#N/A	0	#N/A	0
0	0	LOST_art	1	LOST-3_art	1
0	0	LOST_art	1	LOST-3_art	1
0	0	LOST_art	1	LOST-3_art	1
0	0	#N/A	0	#N/A	0
0	0	#N/A	0	#N/A	0
1	1	#N/A	0	#N/A	0
1	1	#N/A	0	#N/A	0
0	0	#N/A	0	#N/A	0
0	0	#N/A	0	#N/A	0
0	0	#N/A	0	#N/A	0
0	0	#N/A	0	#N/A	0
0	0	#N/A	0	#N/A	0
0	0	#N/A	0	#N/A	0
0	0	#N/A	0	#N/A	0
0	0	#N/A	0	#N/A	0
0	0	#N/A	0	#N/A	0
0	0	#N/A	0	#N/A	0
0	0	#N/A	0	#N/A	0
0	0	#N/A	0	#N/A	0
0	0	#N/A	0	#N/A	0
0	0	#N/A	0	#N/A	0
0	0	#N/A	0	#N/A	0
0	0	#N/A	0	#N/A	0
0	0	#N/A	0	#N/A	0
0	0	#N/A	0	#N/A	0
0	0	#N/A	0	#N/A	0
0	0	#N/A	0	#N/A	0
0	0	#N/A	0	#N/A	0
0	0	#N/A	0	#N/A	0
0	0	#N/A	0	#N/A	0
0	0	#N/A	0	#N/A	0
0	0	#N/A	0	#N/A	0
0	0	#N/A	0	#N/A	0
0	0	#N/A	0	#N/A	0
0	0		0		0
0	0		0		0
1	0	LOST_art	1	LOST-3_art	1
1	0	LOST_art	1	LOST-3_art	1
1	0	LOST_art	1	LOST-3_art	1
0	0	#N/A	0	#N/A	0

0	0	#N/A	0	#N/A	0
0	0	LOST_art	1	LOST-3_art	1
0	0	LOST_art	1	LOST-3_art	1
0	0	#N/A	0	#N/A	0
0	0		0	LOST-3_art	1
0	0		0	LOST-3_art	1
0	0		0	LOST-3_art	1
0	0	#N/A	0	#N/A	0
0	0	#N/A	0	#N/A	0
0	0	#N/A	0	#N/A	0
0	0	#N/A	0	#N/A	0
0	0	LOST_art	1	LOST-3_art	1
0	0	LOST_art	1	LOST-3_art	1
0	0	LOST_art	1	LOST-3_art	1
0	0	#N/A	0	#N/A	0
0	0	#N/A	0	#N/A	0
0	0	#N/A	0	#N/A	0
0	0	#N/A	0	#N/A	0
0	0	#N/A	0	#N/A	0
0	0	#N/A	0	#N/A	0
0	0	#N/A	0	#N/A	0
0	0	#N/A	0	#N/A	0
0	0	#N/A	0	#N/A	0
0	0	#N/A	0	#N/A	0
1	0	#N/A	0	#N/A	0
1	0	#N/A	0	#N/A	0
1	0	#N/A	0	#N/A	0
1	0	#N/A	0	#N/A	0
0	0		0		0
0	0	#N/A	0	#N/A	0
0	0	#N/A	0	#N/A	0
0	0		0	LOST-3_art	1
0	0		0	LOST-3_art	1
0	0		0	LOST-3_art	1
0	0	#N/A	0	#N/A	0
0	0	#N/A	0	#N/A	0
0	0	#N/A	0	#N/A	0
0	0	#N/A	0	#N/A	0
0	0	#N/A	0	#N/A	0
0	0	#N/A	0	#N/A	0
0	0	#N/A	0	#N/A	0
0	0	#N/A	0	#N/A	0
0	0	#N/A	0	#N/A	0
0	0	#N/A	0	#N/A	0
0	0	#N/A	0	#N/A	0
0	0	#N/A	0	#N/A	0
0	0	#N/A	0	#N/A	0

0	0		0	LOST-3_art	1
0	0	#N/A	0	#N/A	0
0	0	#N/A	0	#N/A	0
0	0	#N/A	0	#N/A	0
0	0	#N/A	0	#N/A	0
0	0	#N/A	0	#N/A	0
0	0	#N/A	0	#N/A	0
0	0	#N/A	0	#N/A	0
0	0	#N/A	0	#N/A	0
0	0		0		0
0	0	#N/A	0	#N/A	0
0	0	#N/A	0	#N/A	0
0	0	#N/A	0	#N/A	0
0	0	#N/A	0	#N/A	0
0	0	#N/A	0	#N/A	0
0	0		0	LOST-3_art	1
0	0		0	LOST-3_art	1
0	0	#N/A	0	#N/A	0
1	1	#N/A	0	#N/A	0
1	1	#N/A	0	#N/A	0
1	1	#N/A	0	#N/A	0
0	0	LOST_art	1	LOST-3_art	1
0	0	LOST_art	1	LOST-3_art	1
0	0	LOST_art	1	LOST-3_art	1
0	0		0		0
1	1		0	LOST-3_art	1
0	0		0		0
0	0	#N/A	0	#N/A	0
0	0	#N/A	0	#N/A	0
0	0	#N/A	0	#N/A	0
0	0	#N/A	0	#N/A	0
0	0	#N/A	0	#N/A	0
0	0	#N/A	0	#N/A	0
0	0	#N/A	0	#N/A	0
0	0	#N/A	0	#N/A	0
0	0	#N/A	0	#N/A	0
0	0	#N/A	0	#N/A	0
0	0		0		0
0	0		0		0
0	0	#N/A	0	#N/A	0
0	0		0	LOST-3_art	1
0	0	#N/A	0	#N/A	0
0	0	#N/A	0	#N/A	0
0	0	#N/A	0	#N/A	0
0	0	#N/A	0	#N/A	0
0	0	#N/A	0	#N/A	0
0	0	#N/A	0	#N/A	0

0	0		0	LOST-3_art	1
0	0	#N/A	0	#N/A	0
0	0	#N/A	0	#N/A	0
0	0	#N/A	0	#N/A	0
0	0	#N/A	0	#N/A	0
0	0	#N/A	0	#N/A	0
0	0	#N/A	0	#N/A	0
1	0		0		0
1	0		0		0
0	0	#N/A	0	#N/A	0
0	0	#N/A	0	#N/A	0
0	0	#N/A	0	#N/A	0
0	0	#N/A	0	#N/A	0
0	0		0		0
0	0	#N/A	0	#N/A	0
0	0		0		0
0	0	#N/A	0	#N/A	0
0	0	#N/A	0	#N/A	0
0	0	#N/A	0	#N/A	0
0	0	#N/A	0	#N/A	0
0	0	#N/A	0	#N/A	0
0	0	#N/A	0	#N/A	0
1	0	LOST_art	1	LOST-3_art	1
1	0	LOST_art	1	LOST-3_art	1
1	0	LOST_art	1	LOST-3_art	1
0	0	LOST_art	1	LOST-3_art	1
1	1	#N/A	0	#N/A	0
0	0	#N/A	0	#N/A	0
1	0	#N/A	0	#N/A	0
1	0	#N/A	0	#N/A	0
0	0	#N/A	0	#N/A	0
0	0	#N/A	0	#N/A	0
1	1	#N/A	0	#N/A	0
1	1	#N/A	0	#N/A	0
0	0	#N/A	0	#N/A	0
0	0	LOST_art	1	LOST-3_art	1
0	0	#N/A	0	#N/A	0
0	0	#N/A	0	#N/A	0
0	0	#N/A	0	#N/A	0
0	0	LOST_art	1	LOST-3_art	1
0	0	LOST_art	1	LOST-3_art	1
0	0	#N/A	0	#N/A	0
0	0	#N/A	0	#N/A	0
0	0	#N/A	0	#N/A	0
0	0	#N/A	0	#N/A	0
0	0	LOST_art	1	LOST-3_art	1
0	0	#N/A	0	#N/A	0

0	0	LOST_art	1	LOST-3_art	1
0	0	LOST_art	1	LOST-3_art	1
0	0	LOST_art	1	LOST-3_art	1
0	0	#N/A	0	#N/A	0
0	0	#N/A	0	#N/A	0
0	0	LOST_art	1	LOST-3_art	1
0	0	#N/A	0	#N/A	0
0	0	#N/A	0	#N/A	0
0	0	#N/A	0	#N/A	0
0	0	LOST_art	1	LOST-3_art	1
0	0	LOST_art	1	LOST-3_art	1
0	0	LOST_art	1	LOST-3_art	1
0	0	#N/A	0	#N/A	0
0	0	#N/A	0	#N/A	0
0	0	#N/A	0	#N/A	0
0	0	#N/A	0	#N/A	0
0	0	#N/A	0	#N/A	0
0	0	#N/A	0	#N/A	0
0	0	#N/A	0	#N/A	0
0	0	#N/A	0	#N/A	0
0	0	#N/A	0	#N/A	0
0	0	#N/A	0	#N/A	0
0	0	#N/A	0	#N/A	0
0	0	#N/A	0	#N/A	0
0	0	#N/A	0	#N/A	0
0	0	#N/A	0	#N/A	0
0	0	#N/A	0	#N/A	0
0	0	#N/A	0	#N/A	0
1	0	#N/A	0	#N/A	0
1	0	#N/A	0	#N/A	0
0	0	#N/A	0	#N/A	0
0	0	#N/A	0	#N/A	0
0	0		0	LOST-3_art	1
0	0		0	LOST-3_art	1
0	0	#N/A	0	#N/A	0
0	0		0	LOST-3_art	1
0	0		0	LOST-3_art	1
0	0	#N/A	0	#N/A	0
0	0	#N/A	0	#N/A	0
0	0		0		0
0	0		0		0
0	0	#N/A	0	#N/A	0
0	0	#N/A	0	#N/A	0
0	0	#N/A	0	#N/A	0
0	0	#N/A	0	#N/A	0
0	0	#N/A	0	#N/A	0
0	0		0		0

0	0		0		0
0	0	#N/A	0	#N/A	0
0	0	#N/A	0	#N/A	0
0	0	#N/A	0	#N/A	0
0	0	#N/A	0	#N/A	0
0	0	#N/A	0	#N/A	0
0	0	#N/A	0	#N/A	0
0	0	#N/A	0	#N/A	0
0	0	#N/A	0	#N/A	0
0	0	#N/A	0	#N/A	0
0	0	#N/A	0	#N/A	0
0	0	LOST_art	1	LOST-3_art	1
0	0	#N/A	0	#N/A	0
0	0	#N/A	0	#N/A	0
0	0	#N/A	0	#N/A	0
0	0		0		0
1	0	#N/A	0	#N/A	0
1	0	#N/A	0	#N/A	0
0	0	#N/A	0	#N/A	0
0	0	#N/A	0	#N/A	0
0	0	#N/A	0	#N/A	0
0	0		0	LOST-3_art	1
0	0	#N/A	0	#N/A	0
0	0	#N/A	0	#N/A	0
0	0	#N/A	0	#N/A	0
0	0	#N/A	0	#N/A	0
0	0	#N/A	0	#N/A	0
0	0	#N/A	0	#N/A	0
0	0	#N/A	0	#N/A	0
0	0	#N/A	0	#N/A	0
0	0	#N/A	0	#N/A	0
0	0	#N/A	0	#N/A	0
0	0	LOST_art	1	LOST-3_art	1
0	0	#N/A	0	#N/A	0
0	0		0	LOST-3_art	1
0	0	LOST_art	1	LOST-3_art	1
0	0	LOST_art	1	LOST-3_art	1
0	0	#N/A	0	#N/A	0
0	0	#N/A	0	#N/A	0
0	0	#N/A	0	#N/A	0
0	0	LOST_art	1	LOST-3_art	1
0	0	LOST_art	1	LOST-3_art	1
0	0	LOST_art	1	LOST-3_art	1
0	0	LOST_art	1	LOST-3_art	1
0	0	#N/A	0	#N/A	0
0	0	#N/A	0	#N/A	0
0	0	#N/A	0	#N/A	0

0	0	#N/A	0	#N/A	0
0	0		0		0
0	0	LOST_art	1	LOST-3_art	1
0	0	#N/A	0	#N/A	0
0	0	#N/A	0	#N/A	0
0	0		0	LOST-3_art	1
0	0		0	LOST-3_art	1
0	0		0		0
0	0		0		0
0	0	#N/A	0	#N/A	0
0	0	#N/A	0	#N/A	0
0	0	#N/A	0	#N/A	0
1	1	#N/A	0	#N/A	0
0	0	#N/A	0	#N/A	0
0	0	#N/A	0	#N/A	0
1	1	#N/A	0	#N/A	0
1	1	#N/A	0	#N/A	0
1	1	#N/A	0	#N/A	0
1	1	LOST_art	1	LOST-3_art	1
1	1	LOST_art	1	LOST-3_art	1
0	0	#N/A	0	#N/A	0
0	0	#N/A	0	#N/A	0
0	0	#N/A	0	#N/A	0
0	0	#N/A	0	#N/A	0
0	0		0	LOST-3_art	1
0	0	#N/A	0	#N/A	0
0	0	#N/A	0	#N/A	0
0	0	#N/A	0	#N/A	0
0	0	#N/A	0	#N/A	0
0	0	#N/A	0	#N/A	0
0	0	#N/A	0	#N/A	0
0	0	#N/A	0	#N/A	0
0	0	#N/A	0	#N/A	0
1	1	#N/A	0	#N/A	0
1	1	#N/A	0	#N/A	0
1	1		0		0
0	0	#N/A	0	#N/A	0
0	0	#N/A	0	#N/A	0
0	0	#N/A	0	#N/A	0
0	0	LOST_art	1	LOST-3_art	1
0	0	#N/A	0	#N/A	0
0	0	LOST_art	1	LOST-3_art	1
0	0	#N/A	0	#N/A	0
0	0	#N/A	0	#N/A	0
0	0	#N/A	0	#N/A	0
0	0	#N/A	0	#N/A	0
0	0	#N/A	0	#N/A	0

0	0	#N/A	0	#N/A	0
0	0	#N/A	0	#N/A	0
0	0	#N/A	0	#N/A	0
0	0		0	LOST-3_art	1
0	0	#N/A	0	#N/A	0
0	0	#N/A	0	#N/A	0
0	0	#N/A	0	#N/A	0
0	0	#N/A	0	#N/A	0
0	0	#N/A	0	#N/A	0
0	0	#N/A	0	#N/A	0
0	0	#N/A	0	#N/A	0
0	0	#N/A	0	#N/A	0
0	0	#N/A	0	#N/A	0
0	0		0		0
0	0	#N/A	0	#N/A	0
0	0	#N/A	0	#N/A	0
0	0	#N/A	0	#N/A	0
0	0	#N/A	0	#N/A	0
0	0	#N/A	0	#N/A	0
0	0	#N/A	0	#N/A	0
0	0	#N/A	0	#N/A	0
1	1	#N/A	0	#N/A	0
1	1	#N/A	0	#N/A	0
1	1		0	LOST-3_art	1
0	0	#N/A	0	#N/A	0
0	0	#N/A	0	#N/A	0
0	0	LOST_art	1	LOST-3_art	1
0	0		0		0
0	0		0		0
0	0	#N/A	0	#N/A	0
0	0	#N/A	0	#N/A	0
0	0	#N/A	0	#N/A	0
0	0	#N/A	0	#N/A	0
0	0	#N/A	0	#N/A	0
0	0	#N/A	0	#N/A	0
0	0	#N/A	0	#N/A	0
0	0	#N/A	0	#N/A	0
0	0	#N/A	0	#N/A	0
0	0	#N/A	0	#N/A	0
0	0	#N/A	0	#N/A	0
0	0	#N/A	0	#N/A	0
0	0	#N/A	0	#N/A	0
0	0		0		0
0	0	LOST_art	1	LOST-3_art	1
0	0	#N/A	0	#N/A	0
0	0	#N/A	0	#N/A	0
0	0	#N/A	0	#N/A	0

0	0	#N/A	0	#N/A	0
0	0	#N/A	0	#N/A	0
0	0	#N/A	0	#N/A	0
0	0	#N/A	0	#N/A	0
0	0	#N/A	0	#N/A	0
0	0	#N/A	0	#N/A	0
0	0	#N/A	0	#N/A	0
0	0	#N/A	0	#N/A	0
1	1	#N/A	0	#N/A	0
1	1	#N/A	0	#N/A	0
0	0	#N/A	0	#N/A	0
0	0	#N/A	0	#N/A	0
0	0	#N/A	0	#N/A	0
0	0	#N/A	0	#N/A	0
0	0	#N/A	0	#N/A	0
0	0	#N/A	0	#N/A	0
0	0	#N/A	0	#N/A	0
0	0		0	LOST-3_art	1
0	0	#N/A	0	#N/A	0
0	0	#N/A	0	#N/A	0
0	0	#N/A	0	#N/A	0
0	0	#N/A	0	#N/A	0
0	0	#N/A	0	#N/A	0
0	0	LOST_art	1	LOST-3_art	1
0	0	LOST_art	1	LOST-3_art	1
0	0	#N/A	0	#N/A	0
0	0	#N/A	0	#N/A	0
0	0	#N/A	0	#N/A	0
0	0		0		0
0	0	#N/A	0	#N/A	0
0	0	#N/A	0	#N/A	0
0	0	LOST_art	1	LOST-3_art	1
0	0	LOST_art	1	LOST-3_art	1
0	0	LOST_art	1	LOST-3_art	1
0	0	#N/A	0	#N/A	0
0	0	#N/A	0	#N/A	0
0	0	#N/A	0	#N/A	0
0	0	#N/A	0	#N/A	0
0	0	#N/A	0	#N/A	0
0	0	#N/A	0	#N/A	0
0	0	#N/A	0	#N/A	0
0	0	#N/A	0	#N/A	0
0	0	#N/A	0	#N/A	0
0	0	#N/A	0	#N/A	0
0	0	#N/A	0	#N/A	0
0	0	#N/A	0	#N/A	0
0	0	#N/A	0	#N/A	0

0	0	#N/A	0	#N/A	0
0	0	#N/A	0	#N/A	0
0	0	#N/A	0	#N/A	0
0	0	#N/A	0	#N/A	0
0	0	#N/A	0	#N/A	0
0	0	#N/A	0	#N/A	0
0	0	#N/A	0	#N/A	0
0	0	#N/A	0	#N/A	0
0	0	#N/A	0	#N/A	0
0	0	#N/A	0	#N/A	0
0	0	#N/A	0	#N/A	0
0	0	#N/A	0	#N/A	0
0	0	#N/A	0	#N/A	0
0	0		0		0
0	0	#N/A	0	#N/A	0
0	0	#N/A	0	#N/A	0
0	0	#N/A	0	#N/A	0
0	0	#N/A	0	#N/A	0
0	0	#N/A	0	#N/A	0
0	0	#N/A	0	#N/A	0
0	0	#N/A	0	#N/A	0
1	0	#N/A	0	#N/A	0
1	0	#N/A	0	#N/A	0
0	0	#N/A	0	#N/A	0
0	0		0	LOST-3_art	1
0	0		0	LOST-3_art	1
1	0		0	LOST-3_art	1
1	0		0	LOST-3_art	1
0	0	#N/A	0	#N/A	0
0	0	#N/A	0	#N/A	0
0	0	#N/A	0	#N/A	0
0	0		0		0
1	1		0		0
0	0	#N/A	0	#N/A	0
0	0	#N/A	0	#N/A	0
0	0	LOST_art	1	LOST-3_art	1
0	0	#N/A	0	#N/A	0
0	0	#N/A	0	#N/A	0
0	0	LOST_art	1	LOST-3_art	1
0	0	LOST_art	1	LOST-3_art	1
0	0	LOST_art	1	LOST-3_art	1
0	0	LOST_art	1	LOST-3_art	1
0	0	#N/A	0	#N/A	0
0	0	LOST_art	1	LOST-3_art	1
0	0	LOST_art	1	LOST-3_art	1
0	0	#N/A	0	#N/A	0
0	0	#N/A	0	#N/A	0

0	0	#N/A	0	#N/A	0
0	0		0		0
0	0		0		0
0	0		0		0
0	0	#N/A	0	#N/A	0
1	1	#N/A	0	#N/A	0
0	0	#N/A	0	#N/A	0
0	0	#N/A	0	#N/A	0
0	0	#N/A	0	#N/A	0
1	0	LOST_art	1	LOST-3_art	1
0	0	#N/A	0	#N/A	0
0	0	#N/A	0	#N/A	0
0	0	#N/A	0	#N/A	0
0	0	#N/A	0	#N/A	0
0	0	#N/A	0	#N/A	0
0	0	#N/A	0	#N/A	0
0	0	#N/A	0	#N/A	0
0	0		0	LOST-3_art	1
0	0		0	LOST-3_art	1
1	1	#N/A	0	#N/A	0
1	1	#N/A	0	#N/A	0
0	0	#N/A	0	#N/A	0
0	0	#N/A	0	#N/A	0
0	0	#N/A	0	#N/A	0
0	0	#N/A	0	#N/A	0
0	0	#N/A	0	#N/A	0
0	0	#N/A	0	#N/A	0
0	0	#N/A	0	#N/A	0
0	0	#N/A	0	#N/A	0
0	0		0		0
0	0		0		0
0	0	#N/A	0	#N/A	0
0	0	#N/A	0	#N/A	0
0	0	#N/A	0	#N/A	0
0	0	#N/A	0	#N/A	0
0	0	LOST_art	1	LOST-3_art	1
0	0	LOST_art	1	LOST-3_art	1
0	0	#N/A	0	#N/A	0
0	0	#N/A	0	#N/A	0
0	0	LOST_art	1	LOST-3_art	1
0	0		0		0
0	0		0		0
0	0	LOST_art	1	LOST-3_art	1
0	0		0		0
0	0		0		0
0	0	#N/A	0	#N/A	0
0	0	#N/A	0	#N/A	0

0	0		0		0
0	0	#N/A	0	#N/A	0
0	0		0		0
0	0	#N/A	0	#N/A	0
0	0		0		0
0	0		0	LOST-3_art	1
0	0	#N/A	0	#N/A	0
0	0	#N/A	0	#N/A	0
0	0	#N/A	0	#N/A	0
0	0	#N/A	0	#N/A	0
0	0	LOST_art	1	LOST-3_art	1
0	0	LOST_art	1	LOST-3_art	1
0	0	LOST_art	1	LOST-3_art	1
0	0	LOST_art	1	LOST-3_art	1
0	0	#N/A	0	#N/A	0
0	0	#N/A	0	#N/A	0
0	0	#N/A	0	#N/A	0
0	0	#N/A	0	#N/A	0
0	0	#N/A	0	#N/A	0
0	0	LOST_art	1	LOST-3_art	1
0	0	LOST_art	1	LOST-3_art	1
0	0	LOST_art	1	LOST-3_art	1
0	0	LOST_art	1	LOST-3_art	1
0	0	#N/A	0	#N/A	0
0	0	#N/A	0	#N/A	0
0	0	#N/A	0	#N/A	0
0	0	#N/A	0	#N/A	0
0	0	#N/A	0	#N/A	0
0	0	#N/A	0	#N/A	0
0	0	#N/A	0	#N/A	0
0	0	#N/A	0	#N/A	0
0	0	#N/A	0	#N/A	0
0	0		0		0
0	0	#N/A	0	#N/A	0
0	0	#N/A	0	#N/A	0
0	0	#N/A	0	#N/A	0
0	0		0		0
0	0	#N/A	0	#N/A	0
0	0	#N/A	0	#N/A	0
0	0	#N/A	0	#N/A	0
0	0	#N/A	0	#N/A	0
0	0	LOST_art	1	LOST-3_art	1
0	0	LOST_art	1	LOST-3_art	1
0	0	LOST_art	1	LOST-3_art	1
0	0		0	LOST-3_art	1
0	0		0	LOST-3_art	1
0	0	#N/A	0	#N/A	0

0	0	#N/A	0	#N/A	0
0	0		0		0
0	0	#N/A	0	#N/A	0
0	0	#N/A	0	#N/A	0
0	0	#N/A	0	#N/A	0
0	0	#N/A	0	#N/A	0
0	0	#N/A	0	#N/A	0
0	0		0		0
0	0	#N/A	0	#N/A	0
1	1	#N/A	0	#N/A	0
1	1		0	LOST-3_art	1
1	1		0	LOST-3_art	1
0	0	#N/A	0	#N/A	0
0	0	#N/A	0	#N/A	0
0	0	#N/A	0	#N/A	0
0	0	#N/A	0	#N/A	0
0	0	#N/A	0	#N/A	0
0	0	#N/A	0	#N/A	0
0	0	#N/A	0	#N/A	0
0	0	#N/A	0	#N/A	0
0	0	#N/A	0	#N/A	0
0	0	#N/A	0	#N/A	0
1	1	#N/A	0	#N/A	0
0	0		0	LOST-3_art	1
0	0		0	LOST-3_art	1
0	0		0	LOST-3_art	1
0	0	#N/A	0	#N/A	0
0	0	#N/A	0	#N/A	0
0	0	#N/A	0	#N/A	0
1	0		0	LOST-3_art	1
1	0		0	LOST-3_art	1
0	0		0		0
0	0		0		0
0	0	#N/A	0	#N/A	0
0	0	#N/A	0	#N/A	0
0	0	#N/A	0	#N/A	0
1	1	#N/A	0	#N/A	0
0	0	#N/A	0	#N/A	0
0	0		0		0
0	0		0		0
0	0	#N/A	0	#N/A	0
0	0	#N/A	0	#N/A	0
0	0		0		0
0	0	#N/A	0	#N/A	0
0	0	#N/A	0	#N/A	0
0	0	#N/A	0	#N/A	0
0	0	#N/A	0	#N/A	0

0	0	#N/A	0	#N/A	0
0	0	#N/A	0	#N/A	0
0	0		0		0
0	0	LOST_art	1	LOST-3_art	1
0	0	LOST_art	1	LOST-3_art	1
0	0	LOST_art	1	LOST-3_art	1
0	0	#N/A	0	#N/A	0
0	0	#N/A	0	#N/A	0
0	0	#N/A	0	#N/A	0
0	0	#N/A	0	#N/A	0
0	0	#N/A	0	#N/A	0
0	0	#N/A	0	#N/A	0
0	0	#N/A	0	#N/A	0
0	0	#N/A	0	#N/A	0
0	0	#N/A	0	#N/A	0
0	0	#N/A	0	#N/A	0
0	0	#N/A	0	#N/A	0
0	0	#N/A	0	#N/A	0
0	0	#N/A	0	#N/A	0
0	0	LOST_art	1	LOST-3_art	1
0	0	#N/A	0	#N/A	0
0	0	#N/A	0	#N/A	0
0	0	#N/A	0	#N/A	0
0	0	#N/A	0	#N/A	0
0	0	#N/A	0	#N/A	0
0	0	#N/A	0	#N/A	0
0	0		0		0
0	0		0		0
0	0	#N/A	0	#N/A	0
0	0	#N/A	0	#N/A	0
0	0	#N/A	0	#N/A	0
0	0	#N/A	0	#N/A	0
0	0	#N/A	0	#N/A	0
0	0	#N/A	0	#N/A	0
0	0	#N/A	0	#N/A	0
0	0	#N/A	0	#N/A	0
0	0	#N/A	0	#N/A	0
0	0	#N/A	0	#N/A	0
0	0	#N/A	0	#N/A	0
0	0	#N/A	0	#N/A	0
0	0	#N/A	0	#N/A	0
0	0	#N/A	0	#N/A	0
0	0	#N/A	0	#N/A	0
0	0	#N/A	0	#N/A	0
0	0	#N/A	0	#N/A	0
0	0	#N/A	0	#N/A	0
0	0		0		0

0	0		0		0
0	0	#N/A	0	#N/A	0
0	0	#N/A	0	#N/A	0
0	0	#N/A	0	#N/A	0
0	0	#N/A	0	#N/A	0
0	0		0	LOST-3_art	1
0	0		0	LOST-3_art	1
0	0	LOST_art	1	LOST-3_art	1
0	0	#N/A	0	#N/A	0
0	0	#N/A	0	#N/A	0
0	0	#N/A	0	#N/A	0
0	0	#N/A	0	#N/A	0
0	0		0		0
0	0	#N/A	0	#N/A	0
0	0	#N/A	0	#N/A	0
0	0	#N/A	0	#N/A	0
0	0	#N/A	0	#N/A	0
0	0		0		0
0	0	#N/A	0	#N/A	0
0	0	#N/A	0	#N/A	0
0	0	#N/A	0	#N/A	0
0	0	#N/A	0	#N/A	0
0	0	#N/A	0	#N/A	0
0	0	#N/A	0	#N/A	0
0	0	#N/A	0	#N/A	0
0	0	#N/A	0	#N/A	0
0	0	#N/A	0	#N/A	0
0	0	#N/A	0	#N/A	0
0	0	#N/A	0	#N/A	0
1	1	#N/A	0	#N/A	0
0	0		0		0
0	0		0		0
0	0	#N/A	0	#N/A	0
0	0	#N/A	0	#N/A	0
0	0	#N/A	0	#N/A	0
0	0	#N/A	0	#N/A	0
0	0	#N/A	0	#N/A	0
1	1		0	LOST-3_art	1
1	1		0	LOST-3_art	1
1	1		0	LOST-3_art	1
0	0	#N/A	0	#N/A	0
0	0	#N/A	0	#N/A	0
0	0	#N/A	0	#N/A	0
0	0	#N/A	0	#N/A	0
0	0	#N/A	0	#N/A	0
0	0	#N/A	0	#N/A	0
0	0	#N/A	0	#N/A	0

0	0	#N/A	0	#N/A	0
1	1	#N/A	0	#N/A	0
0	0	#N/A	0	#N/A	0
0	0	#N/A	0	#N/A	0
0	0	#N/A	0	#N/A	0
0	0	#N/A	0	#N/A	0
0	0	#N/A	0	#N/A	0
0	0		0		0
0	0	#N/A	0	#N/A	0
0	0	#N/A	0	#N/A	0
0	0	#N/A	0	#N/A	0
0	0	#N/A	0	#N/A	0
0	0	LOST_art	1	LOST-3_art	1
0	0	LOST_art	1	LOST-3_art	1
0	0	LOST_art	1	LOST-3_art	1
0	0	#N/A	0	#N/A	0
0	0	LOST_art	1	LOST-3_art	1
0	0	LOST_art	1	LOST-3_art	1
0	0	LOST_art	1	LOST-3_art	1
0	0	#N/A	0	#N/A	0
0	0	#N/A	0	#N/A	0
0	0	#N/A	0	#N/A	0
0	0		0		0
0	0	#N/A	0	#N/A	0
0	0	#N/A	0	#N/A	0
0	0	#N/A	0	#N/A	0
0	0	#N/A	0	#N/A	0
0	0	LOST_art	1	LOST-3_art	1
0	0	LOST_art	1	LOST-3_art	1
0	0	LOST_art	1	LOST-3_art	1
0	0	#N/A	0	#N/A	0
0	0	#N/A	0	#N/A	0
0	0	#N/A	0	#N/A	0
0	0		0		0
0	0		0		0
0	0		0		0
0	0		0		0
0	0	#N/A	0	#N/A	0
0	0		0		0
0	0		0		0
0	0	#N/A	0	#N/A	0
0	0	#N/A	0	#N/A	0
0	0	#N/A	0	#N/A	0
0	0	#N/A	0	#N/A	0
0	0	#N/A	0	#N/A	0
0	0	#N/A	0	#N/A	0
0	0	#N/A	0	#N/A	0

0	0	#N/A	0	#N/A	0
0	0	#N/A	0	#N/A	0
0	0	#N/A	0	#N/A	0
0	0	LOST_art	1	LOST-3_art	1
0	0	LOST_art	1	LOST-3_art	1
0	0	LOST_art	1	LOST-3_art	1
0	0	LOST_art	1	LOST-3_art	1
0	0	#N/A	0	#N/A	0
0	0	#N/A	0	#N/A	0
0	0	#N/A	0	#N/A	0
0	0	#N/A	0	#N/A	0
0	0	#N/A	0	#N/A	0
0	0	#N/A	0	#N/A	0
0	0	#N/A	0	#N/A	0
0	0	#N/A	0	#N/A	0
0	0	#N/A	0	#N/A	0
0	0	#N/A	0	#N/A	0
0	0	LOST_art	1	LOST-3_art	1
0	0	LOST_art	1	LOST-3_art	1
0	0	LOST_art	1	LOST-3_art	1
0	0	#N/A	0	#N/A	0
0	0	#N/A	0	#N/A	0
0	0	#N/A	0	#N/A	0
0	0	#N/A	0	#N/A	0
0	0	#N/A	0	#N/A	0
0	0	#N/A	0	#N/A	0
0	0	#N/A	0	#N/A	0
0	0	LOST_art	1	LOST-3_art	1
0	0		0		0
0	0	#N/A	0	#N/A	0
0	0	#N/A	0	#N/A	0
0	0	#N/A	0	#N/A	0
0	0	#N/A	0	#N/A	0
0	0	#N/A	0	#N/A	0
0	0	#N/A	0	#N/A	0
0	0	#N/A	0	#N/A	0
0	0		0		0
0	0	#N/A	0	#N/A	0
0	0	#N/A	0	#N/A	0
0	0	#N/A	0	#N/A	0
0	0	#N/A	0	#N/A	0
0	0	#N/A	0	#N/A	0
0	0	#N/A	0	#N/A	0
0	0	#N/A	0	#N/A	0
0	0		0		0
0	0		0	LOST-3_art	1
0	0		0	LOST-3_art	1

0	0		0	LOST-3_art	1
0	0	#N/A	0	#N/A	0
0	0	#N/A	0	#N/A	0
0	0	#N/A	0	#N/A	0
0	0	#N/A	0	#N/A	0
0	0	#N/A	0	#N/A	0
0	0	#N/A	0	#N/A	0
0	0	#N/A	0	#N/A	0
0	0		0		0
0	0	#N/A	0	#N/A	0
0	0	#N/A	0	#N/A	0
0	0	LOST_art	1	LOST-3_art	1
0	0	#N/A	0	#N/A	0
0	0	#N/A	0	#N/A	0
0	0	#N/A	0	#N/A	0
0	0	#N/A	0	#N/A	0
0	0	#N/A	0	#N/A	0
0	0	#N/A	0	#N/A	0
0	0	#N/A	0	#N/A	0
1	1	#N/A	0	#N/A	0
1	1	#N/A	0	#N/A	0
0	0		0		0
0	0		0		0
0	0	#N/A	0	#N/A	0
0	0	#N/A	0	#N/A	0
0	0	#N/A	0	#N/A	0
0	0	#N/A	0	#N/A	0
0	0	#N/A	0	#N/A	0
0	0	#N/A	0	#N/A	0
0	0	#N/A	0	#N/A	0
0	0	LOST_art	1	LOST-3_art	1
0	0	LOST_art	1	LOST-3_art	1
1	0	#N/A	0	#N/A	0
0	0	#N/A	0	#N/A	0
0	0	#N/A	0	#N/A	0
0	0	#N/A	0	#N/A	0
0	0	#N/A	0	#N/A	0
0	0	#N/A	0	#N/A	0
0	0	#N/A	0	#N/A	0
0	0	#N/A	0	#N/A	0
0	0		0		0
0	0	#N/A	0	#N/A	0
0	0		0		0
0	0		0		0
0	0	#N/A	0	#N/A	0
0	0	#N/A	0	#N/A	0
0	0		0		0

1	0	#N/A	0	#N/A	0
1	0	#N/A	0	#N/A	0
0	0	#N/A	0	#N/A	0
0	0	#N/A	0	#N/A	0
0	0	#N/A	0	#N/A	0
0	0	#N/A	0	#N/A	0
0	0	#N/A	0	#N/A	0
0	0	#N/A	0	#N/A	0
0	0	#N/A	0	#N/A	0
0	0	#N/A	0	#N/A	0
0	0	#N/A	0	#N/A	0
0	0	#N/A	0	#N/A	0
0	0	#N/A	0	#N/A	0
0	0		0		0
1	0	#N/A	0	#N/A	0
0	0	#N/A	0	#N/A	0
0	0	#N/A	0	#N/A	0
0	0	#N/A	0	#N/A	0
0	0	#N/A	0	#N/A	0
0	0	#N/A	0	#N/A	0
0	0	#N/A	0	#N/A	0
0	0	#N/A	0	#N/A	0
0	0		0		0
0	0	#N/A	0	#N/A	0
1	0	#N/A	0	#N/A	0
1	0	#N/A	0	#N/A	0
0	0	#N/A	0	#N/A	0
0	0	#N/A	0	#N/A	0
0	0	#N/A	0	#N/A	0
0	0	#N/A	0	#N/A	0
0	0	#N/A	0	#N/A	0
0	0	#N/A	0	#N/A	0
0	0	#N/A	0	#N/A	0
0	0	#N/A	0	#N/A	0
0	0	#N/A	0	#N/A	0
0	0	#N/A	0	#N/A	0
0	0	#N/A	0	#N/A	0
0	0	#N/A	0	#N/A	0
0	0	#N/A	0	#N/A	0
0	0	#N/A	0	#N/A	0
0	0	#N/A	0	#N/A	0
0	0	#N/A	0	#N/A	0
0	0	LOST_art	1	LOST-3_art	1
0	0	#N/A	0	#N/A	0
0	0	#N/A	0	#N/A	0
0	0	#N/A	0	#N/A	0
0	0	#N/A	0	#N/A	0

0	0	#N/A	0	#N/A	0
0	0	LOST_art	1	LOST-3_art	1
0	0	LOST_art	1	LOST-3_art	1
0	0	LOST_art	1	LOST-3_art	1
0	0	#N/A	0	#N/A	0
0	0	LOST_art	1	LOST-3_art	1
0	0	LOST_art	1	LOST-3_art	1
0	0	LOST_art	1	LOST-3_art	1
0	0	#N/A	0	#N/A	0
0	0	#N/A	0	#N/A	0
1	0	#N/A	0	#N/A	0
1	0	#N/A	0	#N/A	0
0	0	#N/A	0	#N/A	0
0	0	#N/A	0	#N/A	0
0	0	#N/A	0	#N/A	0
0	0	#N/A	0	#N/A	0
0	0	#N/A	0	#N/A	0
0	0	#N/A	0	#N/A	0
0	0	#N/A	0	#N/A	0
0	0		0	LOST-3_art	1
0	0		0	LOST-3_art	1
0	0	#N/A	0	#N/A	0
0	0	#N/A	0	#N/A	0
0	0	#N/A	0	#N/A	0
0	0		0		0
0	0	#N/A	0	#N/A	0
0	0		0		0
0	0		0		0
0	0	#N/A	0	#N/A	0
0	0	#N/A	0	#N/A	0
0	0	#N/A	0	#N/A	0
0	0	#N/A	0	#N/A	0
0	0	#N/A	0	#N/A	0
0	0	#N/A	0	#N/A	0
0	0	#N/A	0	#N/A	0
0	0	#N/A	0	#N/A	0
0	0	#N/A	0	#N/A	0
0	0	#N/A	0	#N/A	0
0	0	#N/A	0	#N/A	0
0	0	#N/A	0	#N/A	0
0	0	#N/A	0	#N/A	0
0	0	#N/A	0	#N/A	0
0	0	LOST_art	1	LOST-3_art	1
0	0	LOST_art	1	LOST-3_art	1
0	0	LOST_art	1	LOST-3_art	1
1	1	LOST_art	1	LOST-3_art	1
1	1	LOST_art	1	LOST-3_art	1

1	1	LOST_art	1	LOST-3_art	1
1	1	LOST_art	1	LOST-3_art	1
1	1	LOST_art	1	LOST-3_art	1
0	0	#N/A	0	#N/A	0
0	0	#N/A	0	#N/A	0
1	1	#N/A	0	#N/A	0
0	0	#N/A	0	#N/A	0
0	0	#N/A	0	#N/A	0
0	0	#N/A	0	#N/A	0
0	0		0	LOST-3_art	1
0	0		0	LOST-3_art	1
0	0	#N/A	0	#N/A	0
0	0	#N/A	0	#N/A	0
0	0	#N/A	0	#N/A	0
0	0	#N/A	0	#N/A	0
0	0	#N/A	0	#N/A	0
0	0	#N/A	0	#N/A	0
0	0	#N/A	0	#N/A	0
0	0	#N/A	0	#N/A	0
0	0	#N/A	0	#N/A	0
0	0		0	LOST-3_art	1
0	0		0	LOST-3_art	1
0	0	#N/A	0	#N/A	0
0	0	#N/A	0	#N/A	0
0	0	#N/A	0	#N/A	0
0	0	#N/A	0	#N/A	0
1	1	#N/A	0	#N/A	0
0	0	#N/A	0	#N/A	0
0	0	#N/A	0	#N/A	0
0	0	#N/A	0	#N/A	0
0	0	#N/A	0	#N/A	0
1	1	#N/A	0	#N/A	0
1	1	#N/A	0	#N/A	0
0	0		0	LOST-3_art	1
0	0		0	LOST-3_art	1
0	0	#N/A	0	#N/A	0
0	0	#N/A	0	#N/A	0
0	0	#N/A	0	#N/A	0
0	0	#N/A	0	#N/A	0
0	0	#N/A	0	#N/A	0
0	0	#N/A	0	#N/A	0
0	0	#N/A	0	#N/A	0
0	0	#N/A	0	#N/A	0
0	0	#N/A	0	#N/A	0
0	0	#N/A	0	#N/A	0
0	0		0	LOST-3_art	1
0	0	#N/A	0	#N/A	0

0	0	#N/A	0	#N/A	0
0	0	LOST_art	1	LOST-3_art	1
0	0	LOST_art	1	LOST-3_art	1
0	0	LOST_art	1	LOST-3_art	1
0	0	LOST_art	1	LOST-3_art	1
0	0	LOST_art	1	LOST-3_art	1
0	0		0	LOST-3_art	1
0	0	LOST_art	1	LOST-3_art	1
0	0	LOST_art	1	LOST-3_art	1
0	0	LOST_art	1	LOST-3_art	1
0	0	#N/A	0	#N/A	0
0	0	#N/A	0	#N/A	0
0	0	#N/A	0	#N/A	0
1	1	#N/A	0	#N/A	0
1	1	#N/A	0	#N/A	0
1	1	#N/A	0	#N/A	0
0	0	#N/A	0	#N/A	0
0	0	#N/A	0	#N/A	0
0	0	#N/A	0	#N/A	0
0	0	#N/A	0	#N/A	0
0	0	LOST_art	1	LOST-3_art	1
0	0	LOST_art	1	LOST-3_art	1
0	0	#N/A	0	#N/A	0
0	0	#N/A	0	#N/A	0
0	0	#N/A	0	#N/A	0
0	0	#N/A	0	#N/A	0
0	0	#N/A	0	#N/A	0
0	0	#N/A	0	#N/A	0
1	1	#N/A	0	#N/A	0
1	1	#N/A	0	#N/A	0
0	0	#N/A	0	#N/A	0
0	0	LOST_art	1	LOST-3_art	1
0	0	LOST_art	1	LOST-3_art	1
0	0	LOST_art	1	LOST-3_art	1
0	0	LOST_art	1	LOST-3_art	1
0	0	#N/A	0	#N/A	0
0	0		0		0
0	0	#N/A	0	#N/A	0
0	0	#N/A	0	#N/A	0
0	0	#N/A	0	#N/A	0
0	0	#N/A	0	#N/A	0
0	0	#N/A	0	#N/A	0
0	0	#N/A	0	#N/A	0
0	0	#N/A	0	#N/A	0
0	0	#N/A	0	#N/A	0
0	0	#N/A	0	#N/A	0
0	0	#N/A	0	#N/A	0

0	0	#N/A	0	#N/A	0
0	0	#N/A	0	#N/A	0
1	1	#N/A	0	#N/A	0
1	1	#N/A	0	#N/A	0
1	1	#N/A	0	#N/A	0
1	0	LOST_art	1	LOST-3_art	1
1	0	LOST_art	1	LOST-3_art	1
1	0	LOST_art	1	LOST-3_art	1
0	0	#N/A	0	#N/A	0
0	0	#N/A	0	#N/A	0
0	0	#N/A	0	#N/A	0
0	0	#N/A	0	#N/A	0
0	0	#N/A	0	#N/A	0
0	0	#N/A	0	#N/A	0
0	0	#N/A	0	#N/A	0
0	0	#N/A	0	#N/A	0
0	0	#N/A	0	#N/A	0
0	0	#N/A	0	#N/A	0
0	0	#N/A	0	#N/A	0
0	0	#N/A	0	#N/A	0
0	0	#N/A	0	#N/A	0
0	0	#N/A	0	#N/A	0
0	0	#N/A	0	#N/A	0
0	0	#N/A	0	#N/A	0
0	0	#N/A	0	#N/A	0
0	0	LOST_art	1	LOST-3_art	1
0	0	LOST_art	1	LOST-3_art	1
0	0	LOST_art	1	LOST-3_art	1
0	0	LOST_art	1	LOST-3_art	1
0	0	LOST_art	1	LOST-3_art	1
0	0	#N/A	0	#N/A	0
1	1	#N/A	0	#N/A	0
1	1	#N/A	0	#N/A	0
0	0	#N/A	0	#N/A	0
0	0	#N/A	0	#N/A	0
0	0	#N/A	0	#N/A	0
0	0	#N/A	0	#N/A	0
0	0		0	LOST-3_art	1
0	0		0	LOST-3_art	1
0	0		0		0
0	0		0		0
0	0	#N/A	0	#N/A	0
0	0	#N/A	0	#N/A	0
0	0		0	LOST-3_art	1
0	0		0	LOST-3_art	1
0	0	#N/A	0	#N/A	0
0	0	#N/A	0	#N/A	0

0	0	#N/A	0	#N/A	0
1	1	#N/A	0	#N/A	0
0	0	#N/A	0	#N/A	0
0	0	#N/A	0	#N/A	0
0	0	#N/A	0	#N/A	0
0	0	#N/A	0	#N/A	0
0	0	#N/A	0	#N/A	0
0	0		0	LOST-3_art	1
0	0		0	LOST-3_art	1
0	0	#N/A	0	#N/A	0
0	0	#N/A	0	#N/A	0
0	0	#N/A	0	#N/A	0
0	0	#N/A	0	#N/A	0
0	0	#N/A	0	#N/A	0
0	0	#N/A	0	#N/A	0
0	0		0		0
0	0		0	LOST-3_art	1
0	0	#N/A	0	#N/A	0
0	0	#N/A	0	#N/A	0
0	0	#N/A	0	#N/A	0
0	0	#N/A	0	#N/A	0
0	0	#N/A	0	#N/A	0
0	0	#N/A	0	#N/A	0
0	0	#N/A	0	#N/A	0
0	0	#N/A	0	#N/A	0
0	0	#N/A	0	#N/A	0
0	0	#N/A	0	#N/A	0
0	0	#N/A	0	#N/A	0
0	0	LOST_art	1	LOST-3_art	1
0	0	LOST_art	1	LOST-3_art	1
0	0	#N/A	0	#N/A	0
0	0	#N/A	0	#N/A	0
0	0	#N/A	0	#N/A	0
0	0	#N/A	0	#N/A	0
0	0	#N/A	0	#N/A	0
0	0	#N/A	0	#N/A	0
0	0	#N/A	0	#N/A	0
0	0	#N/A	0	#N/A	0
0	0	#N/A	0	#N/A	0
0	0	#N/A	0	#N/A	0
0	0	#N/A	0	#N/A	0
0	0	#N/A	0	#N/A	0
0	0	#N/A	0	#N/A	0
0	0	#N/A	0	#N/A	0
1	1	LOST_art	1	LOST-3_art	1
1	1	LOST_art	1	LOST-3_art	1
0	0	#N/A	0	#N/A	0

0	0	#N/A	0	#N/A	0
0	0	#N/A	0	#N/A	0
0	0	#N/A	0	#N/A	0
0	0	#N/A	0	#N/A	0
0	0	#N/A	0	#N/A	0
0	0	#N/A	0	#N/A	0
1	1	#N/A	0	#N/A	0
1	1	#N/A	0	#N/A	0
0	0	#N/A	0	#N/A	0
0	0	#N/A	0	#N/A	0
0	0		0	LOST-3_art	1
0	0		0		0
0	0	#N/A	0	#N/A	0
0	0	#N/A	0	#N/A	0
0	0	#N/A	0	#N/A	0
0	0	#N/A	0	#N/A	0
0	0	#N/A	0	#N/A	0
0	0	#N/A	0	#N/A	0
0	0	#N/A	0	#N/A	0
0	0	#N/A	0	#N/A	0
0	0	#N/A	0	#N/A	0
1	1	#N/A	0	#N/A	0
1	1	#N/A	0	#N/A	0
1	1	#N/A	0	#N/A	0
0	0		0	LOST-3_art	1
0	0	#N/A	0	#N/A	0
0	0	#N/A	0	#N/A	0
0	0	LOST_art	1	LOST-3_art	1
0	0	LOST_art	1	LOST-3_art	1
0	0	LOST_art	1	LOST-3_art	1
0	0	LOST_art	1	LOST-3_art	1
0	0	#N/A	0	#N/A	0
0	0	#N/A	0	#N/A	0
0	0	#N/A	0	#N/A	0
0	0	LOST_art	1	LOST-3_art	1
0	0	LOST_art	1	LOST-3_art	1
0	0	#N/A	0	#N/A	0
0	0	LOST_art	1	LOST-3_art	1
0	0	LOST_art	1	LOST-3_art	1
0	0	#N/A	0	#N/A	0
0	0	#N/A	0	#N/A	0
0	0	#N/A	0	#N/A	0
0	0	#N/A	0	#N/A	0
0	0	#N/A	0	#N/A	0
0	0	#N/A	0	#N/A	0
0	0	#N/A	0	#N/A	0
0	0	LOST_art	1	LOST-3_art	1

0	0		0	LOST-3_art	1
0	0		0	LOST-3_art	1
0	0		0	LOST-3_art	1
0	0		0	LOST-3_art	1
0	0		0	LOST-3_art	1
0	0	#N/A	0	#N/A	0
1	1	#N/A	0	#N/A	0
1	1	#N/A	0	#N/A	0
1	1	#N/A	0	#N/A	0
0	0	#N/A	0	#N/A	0
0	0	#N/A	0	#N/A	0
1	1	#N/A	0	#N/A	0
0	0	#N/A	0	#N/A	0
0	0	#N/A	0	#N/A	0
0	0	#N/A	0	#N/A	0
0	0		0		0
0	0	#N/A	0	#N/A	0
0	0	#N/A	0	#N/A	0
0	0	#N/A	0	#N/A	0
0	0	#N/A	0	#N/A	0
0	0	#N/A	0	#N/A	0
0	0	#N/A	0	#N/A	0
0	0	#N/A	0	#N/A	0
0	0	#N/A	0	#N/A	0
0	0	#N/A	0	#N/A	0
0	0	#N/A	0	#N/A	0
0	0	#N/A	0	#N/A	0
0	0	#N/A	0	#N/A	0
0	0	#N/A	0	#N/A	0
1	0	LOST_art	1	LOST-3_art	1
1	0	LOST_art	1	LOST-3_art	1
0	0	#N/A	0	#N/A	0
0	0	#N/A	0	#N/A	0
0	0	#N/A	0	#N/A	0
0	0	#N/A	0	#N/A	0
0	0	#N/A	0	#N/A	0
0	0	#N/A	0	#N/A	0
0	0	#N/A	0	#N/A	0
0	0	#N/A	0	#N/A	0
0	0	#N/A	0	#N/A	0
1	1	#N/A	0	#N/A	0
1	1	#N/A	0	#N/A	0
0	0		0		0
0	0	#N/A	0	#N/A	0
0	0	#N/A	0	#N/A	0
0	0	#N/A	0	#N/A	0
0	0	#N/A	0	#N/A	0

0	0	#N/A	0	#N/A	0
0	0	#N/A	0	#N/A	0
0	0	#N/A	0	#N/A	0
0	0	LOST_art	1	LOST-3_art	1
0	0	LOST_art	1	LOST-3_art	1
0	0	#N/A	0	#N/A	0
0	0		0		0
0	0	#N/A	0	#N/A	0
0	0	#N/A	0	#N/A	0
0	0	#N/A	0	#N/A	0
0	0		0		0
0	0	LOST_art	1	LOST-3_art	1
0	0	LOST_art	1	LOST-3_art	1
0	0	LOST_art	1	LOST-3_art	1
0	0	#N/A	0	#N/A	0
0	0	#N/A	0	#N/A	0
0	0	#N/A	0	#N/A	0
0	0	#N/A	0	#N/A	0
0	0	#N/A	0	#N/A	0
0	0	#N/A	0	#N/A	0
0	0	#N/A	0	#N/A	0
0	0	#N/A	0	#N/A	0
0	0	#N/A	0	#N/A	0
1	1	#N/A	0	#N/A	0
0	0	#N/A	0	#N/A	0
0	0		0		0
0	0	#N/A	0	#N/A	0
0	0	#N/A	0	#N/A	0
0	0		0		0
0	0	#N/A	0	#N/A	0
0	0	#N/A	0	#N/A	0
0	0	#N/A	0	#N/A	0
0	0		0		0
1	1		0	LOST-3_art	1
1	1		0	LOST-3_art	1
0	0		0		0
0	0		0		0
0	0	#N/A	0	#N/A	0
0	0	#N/A	0	#N/A	0
0	0	#N/A	0	#N/A	0
0	0	#N/A	0	#N/A	0
0	0	#N/A	0	#N/A	0
0	0	#N/A	0	#N/A	0
0	0	#N/A	0	#N/A	0
0	0	#N/A	0	#N/A	0
0	0	#N/A	0	#N/A	0
0	0	#N/A	0	#N/A	0

0	0	#N/A	0	#N/A	0
0	0	#N/A	0	#N/A	0
0	0	#N/A	0	#N/A	0
0	0	#N/A	0	#N/A	0
0	0	LOST_art	1	LOST-3_art	1
0	0	#N/A	0	#N/A	0
1	1	#N/A	0	#N/A	0
0	0	#N/A	0	#N/A	0
0	0	#N/A	0	#N/A	0
1	1	#N/A	0	#N/A	0
1	1	#N/A	0	#N/A	0
1	1	#N/A	0	#N/A	0
1	1	#N/A	0	#N/A	0
0	0	#N/A	0	#N/A	0
0	0	#N/A	0	#N/A	0
0	0	#N/A	0	#N/A	0
0	0	#N/A	0	#N/A	0
0	0	#N/A	0	#N/A	0
0	0	#N/A	0	#N/A	0
0	0	#N/A	0	#N/A	0
0	0	#N/A	0	#N/A	0
0	0	#N/A	0	#N/A	0
0	0	#N/A	0	#N/A	0
0	0	#N/A	0	#N/A	0
0	0	#N/A	0	#N/A	0
0	0	#N/A	0	#N/A	0
0	0	#N/A	0	#N/A	0
0	0		0		0
0	0		0		0
0	0	#N/A	0	#N/A	0
0	0	#N/A	0	#N/A	0
0	0	#N/A	0	#N/A	0
0	0	#N/A	0	#N/A	0
0	0	#N/A	0	#N/A	0
0	0	#N/A	0	#N/A	0
0	0	#N/A	0	#N/A	0
0	0	#N/A	0	#N/A	0
0	0	#N/A	0	#N/A	0
0	0	#N/A	0	#N/A	0
0	0	#N/A	0	#N/A	0
0	0	#N/A	0	#N/A	0
1	1		0	LOST-3_art	1
0	0	#N/A	0	#N/A	0
0	0	#N/A	0	#N/A	0
0	0	#N/A	0	#N/A	0
0	0	#N/A	0	#N/A	0
0	0	#N/A	0	#N/A	0

0	0	#N/A	0	#N/A	0
0	0	#N/A	0	#N/A	0
0	0	#N/A	0	#N/A	0
0	0	#N/A	0	#N/A	0
0	0	#N/A	0	#N/A	0
0	0		0	LOST-3_art	1
0	0	#N/A	0	#N/A	0
0	0	#N/A	0	#N/A	0
0	0	#N/A	0	#N/A	0
0	0	#N/A	0	#N/A	0
0	0		0	LOST-3_art	1
0	0		0	LOST-3_art	1
0	0	#N/A	0	#N/A	0
0	0	#N/A	0	#N/A	0
0	0	#N/A	0	#N/A	0
0	0	#N/A	0	#N/A	0
0	0	#N/A	0	#N/A	0
0	0	#N/A	0	#N/A	0
0	0	#N/A	0	#N/A	0
0	0		0	LOST-3_art	1
0	0		0	LOST-3_art	1
0	0		0	LOST-3_art	1
0	0	#N/A	0	#N/A	0
0	0	#N/A	0	#N/A	0
0	0	#N/A	0	#N/A	0
0	0	#N/A	0	#N/A	0
0	0	#N/A	0	#N/A	0
0	0	#N/A	0	#N/A	0
0	0	#N/A	0	#N/A	0
0	0	#N/A	0	#N/A	0
0	0	#N/A	0	#N/A	0
0	0	#N/A	0	#N/A	0
0	0	#N/A	0	#N/A	0
0	0	#N/A	0	#N/A	0
0	0	#N/A	0	#N/A	0
0	0	#N/A	0	#N/A	0
1	1	LOST_art	1	LOST-3_art	1
1	1	LOST_art	1	LOST-3_art	1
1	1	LOST_art	1	LOST-3_art	1
0	0	#N/A	0	#N/A	0
0	0	#N/A	0	#N/A	0
0	0	#N/A	0	#N/A	0
0	0	#N/A	0	#N/A	0
0	0		0		0
0	0	LOST_art	1	LOST-3_art	1
0	0	LOST_art	1	LOST-3_art	1
0	0	LOST_art	1	LOST-3_art	1

0	0	#N/A	0	#N/A	0
0	0	#N/A	0	#N/A	0
0	0		0		0
0	0	#N/A	0	#N/A	0
0	0	#N/A	0	#N/A	0
0	0	#N/A	0	#N/A	0
0	0		0		0
0	0	LOST_art	1	LOST-3_art	1
0	0	#N/A	0	#N/A	0
0	0	#N/A	0	#N/A	0
0	0	#N/A	0	#N/A	0
0	0	#N/A	0	#N/A	0
0	0	#N/A	0	#N/A	0
0	0	LOST_art	1	LOST-3_art	1
0	0	LOST_art	1	LOST-3_art	1
0	0	LOST_art	1	LOST-3_art	1
1	1	#N/A	0	#N/A	0
1	1	#N/A	0	#N/A	0
0	0	#N/A	0	#N/A	0
0	0	#N/A	0	#N/A	0
0	0		0		0
1	1	#N/A	0	#N/A	0
1	1	#N/A	0	#N/A	0
0	0	#N/A	0	#N/A	0
0	0	#N/A	0	#N/A	0
0	0	#N/A	0	#N/A	0
0	0	#N/A	0	#N/A	0
0	0	#N/A	0	#N/A	0
0	0	#N/A	0	#N/A	0
0	0	#N/A	0	#N/A	0
0	0	#N/A	0	#N/A	0
0	0	#N/A	0	#N/A	0
0	0	#N/A	0	#N/A	0
0	0	#N/A	0	#N/A	0
0	0	#N/A	0	#N/A	0
0	0	#N/A	0	#N/A	0
0	0	#N/A	0	#N/A	0
0	0	#N/A	0	#N/A	0
0	0	#N/A	0	#N/A	0
1	1	#N/A	0	#N/A	0
1	1	#N/A	0	#N/A	0
0	0		0	LOST-3_art	1
0	0	LOST_art	1	LOST-3_art	1
0	0	#N/A	0	#N/A	0
0	0	LOST_art	1	LOST-3_art	1
0	0	#N/A	0	#N/A	0
0	0	#N/A	0	#N/A	0

0	0	#N/A	0	#N/A	0
0	0	#N/A	0	#N/A	0
0	0	#N/A	0	#N/A	0
0	0		0		0
0	0	#N/A	0	#N/A	0
0	0	#N/A	0	#N/A	0
0	0		0		0
0	0	#N/A	0	#N/A	0
0	0	#N/A	0	#N/A	0
0	0	#N/A	0	#N/A	0
1	1	#N/A	0	#N/A	0
1	1	LOST_art	1	LOST-3_art	1
1	1	LOST_art	1	LOST-3_art	1
0	0	#N/A	0	#N/A	0
1	1	#N/A	0	#N/A	0
0	0	#N/A	0	#N/A	0
0	0	#N/A	0	#N/A	0
0	0	#N/A	0	#N/A	0
0	0	#N/A	0	#N/A	0
0	0	#N/A	0	#N/A	0
0	0	#N/A	0	#N/A	0
0	0		0	LOST-3_art	1
0	0		0	LOST-3_art	1
0	0		0	LOST-3_art	1
0	0	#N/A	0	#N/A	0
0	0	#N/A	0	#N/A	0
0	0		0	LOST-3_art	1
0	0	LOST_art	1	LOST-3_art	1
0	0	LOST_art	1	LOST-3_art	1
0	0	LOST_art	1	LOST-3_art	1
0	0	#N/A	0	#N/A	0
0	0	#N/A	0	#N/A	0
0	0	#N/A	0	#N/A	0
1	1		0		0
1	1		0		0
0	0	#N/A	0	#N/A	0
0	0	#N/A	0	#N/A	0
1	1	#N/A	0	#N/A	0
1	1	#N/A	0	#N/A	0
0	0	#N/A	0	#N/A	0
0	0		0		0
0	0	#N/A	0	#N/A	0
0	0	#N/A	0	#N/A	0
0	0	#N/A	0	#N/A	0
0	0	#N/A	0	#N/A	0
0	0	LOST_art	1	LOST-3_art	1
1	1	#N/A	0	#N/A	0

1	1	#N/A	0	#N/A	0
0	0	#N/A	0	#N/A	0
0	0	#N/A	0	#N/A	0
0	0	#N/A	0	#N/A	0
0	0	#N/A	0	#N/A	0
0	0	#N/A	0	#N/A	0
0	0	#N/A	0	#N/A	0
0	0		0	LOST-3_art	1
0	0		0	LOST-3_art	1
0	0		0	LOST-3_art	1
1	0	LOST_art	1	LOST-3_art	1
1	0	LOST_art	1	LOST-3_art	1
1	0	LOST_art	1	LOST-3_art	1
1	0	LOST_art	1	LOST-3_art	1
0	0	#N/A	0	#N/A	0
0	0		0		0
0	0		0		0
0	0		0		0
0	0	#N/A	0	#N/A	0
0	0	#N/A	0	#N/A	0
0	0	#N/A	0	#N/A	0
0	0	#N/A	0	#N/A	0
0	0	#N/A	0	#N/A	0
0	0	#N/A	0	#N/A	0
0	0	#N/A	0	#N/A	0
0	0	LOST_art	1	LOST-3_art	1
0	0	LOST_art	1	LOST-3_art	1
0	0	#N/A	0	#N/A	0
0	0		0		0
0	0	#N/A	0	#N/A	0
0	0	#N/A	0	#N/A	0
0	0	#N/A	0	#N/A	0
0	0	LOST_art	1	LOST-3_art	1
0	0	LOST_art	1	LOST-3_art	1
0	0	#N/A	0	#N/A	0
0	0	#N/A	0	#N/A	0
0	0	#N/A	0	#N/A	0
0	0	#N/A	0	#N/A	0
0	0	#N/A	0	#N/A	0
0	0	#N/A	0	#N/A	0
0	0	#N/A	0	#N/A	0
0	0	LOST_art	1	LOST-3_art	1
0	0	#N/A	0	#N/A	0
1	1	LOST_art	1	LOST-3_art	1
1	1	LOST_art	1	LOST-3_art	1
1	1	LOST_art	1	LOST-3_art	1
0	0	LOST_art	1	LOST-3_art	1

0	0	#N/A	0	#N/A	0
0	0	#N/A	0	#N/A	0
0	0	#N/A	0	#N/A	0
0	0	#N/A	0	#N/A	0
0	0	#N/A	0	#N/A	0
0	0	#N/A	0	#N/A	0
0	0	#N/A	0	#N/A	0
0	0	LOST_art	1	LOST-3_art	1
0	0	LOST_art	1	LOST-3_art	1
0	0	#N/A	0	#N/A	0
0	0	#N/A	0	#N/A	0
0	0	#N/A	0	#N/A	0
0	0		0		0
0	0	#N/A	0	#N/A	0
1	1	#N/A	0	#N/A	0
1	1	#N/A	0	#N/A	0
0	0	#N/A	0	#N/A	0
0	0	#N/A	0	#N/A	0
0	0	#N/A	0	#N/A	0
0	0		0		0
0	0	#N/A	0	#N/A	0
0	0	#N/A	0	#N/A	0
0	0	#N/A	0	#N/A	0
0	0	#N/A	0	#N/A	0
0	0	LOST_art	1	LOST-3_art	1
0	0	#N/A	0	#N/A	0
0	0	#N/A	0	#N/A	0
0	0	#N/A	0	#N/A	0
0	0	#N/A	0	#N/A	0
0	0		0	LOST-3_art	1
0	0	#N/A	0	#N/A	0
0	0	#N/A	0	#N/A	0
0	0	#N/A	0	#N/A	0
0	0	#N/A	0	#N/A	0
0	0	#N/A	0	#N/A	0
1	1	#N/A	0	#N/A	0
0	0	#N/A	0	#N/A	0
0	0	#N/A	0	#N/A	0
0	0		0		0
0	0	#N/A	0	#N/A	0
0	0	#N/A	0	#N/A	0
0	0	#N/A	0	#N/A	0
0	0		0		0
1	0	LOST_art	1	LOST-3_art	1
1	0	LOST_art	1	LOST-3_art	1
1	0	LOST_art	1	LOST-3_art	1
1	0	LOST_art	1	LOST-3_art	1

1	1	#N/A	0	#N/A	0
1	1	#N/A	0	#N/A	0
0	0	#N/A	0	#N/A	0
0	0	#N/A	0	#N/A	0
0	0	#N/A	0	#N/A	0
0	0	#N/A	0	#N/A	0
0	0	#N/A	0	#N/A	0
0	0		0		0
0	0	#N/A	0	#N/A	0
0	0	#N/A	0	#N/A	0
0	0	#N/A	0	#N/A	0
0	0	LOST_art	1	LOST-3_art	1
0	0	#N/A	0	#N/A	0
0	0	#N/A	0	#N/A	0
0	0	#N/A	0	#N/A	0
0	0	#N/A	0	#N/A	0
0	0	#N/A	0	#N/A	0
0	0	#N/A	0	#N/A	0
0	0	#N/A	0	#N/A	0
0	0		0		0
0	0		0		0
0	0	#N/A	0	#N/A	0
0	0	LOST_art	1	LOST-3_art	1
0	0	LOST_art	1	LOST-3_art	1
0	0	LOST_art	1	LOST-3_art	1
0	0		0		0
0	0		0		0
0	0		0		0
0	0	#N/A	0	#N/A	0
0	0	#N/A	0	#N/A	0
0	0	#N/A	0	#N/A	0
0	0		0		0
1	1	#N/A	0	#N/A	0
1	1	#N/A	0	#N/A	0
1	0	#N/A	0	#N/A	0
1	0	#N/A	0	#N/A	0
0	0	#N/A	0	#N/A	0
0	0		0		0
0	0	#N/A	0	#N/A	0
1	1	#N/A	0	#N/A	0
1	1	#N/A	0	#N/A	0
1	1	#N/A	0	#N/A	0
0	0	#N/A	0	#N/A	0
0	0	#N/A	0	#N/A	0
0	0	#N/A	0	#N/A	0
0	0	#N/A	0	#N/A	0
0	0	#N/A	0	#N/A	0

0	0	#N/A	0	#N/A	0
0	0	#N/A	0	#N/A	0
0	0	#N/A	0	#N/A	0
0	0	#N/A	0	#N/A	0
0	0		0		0
0	0		0		0
0	0	#N/A	0	#N/A	0
0	0	#N/A	0	#N/A	0
0	0	LOST_art	1	LOST-3_art	1
0	0	LOST_art	1	LOST-3_art	1
0	0	LOST_art	1	LOST-3_art	1
0	0	LOST_art	1	LOST-3_art	1
0	0	LOST_art	1	LOST-3_art	1
0	0	#N/A	0	#N/A	0
0	0	#N/A	0	#N/A	0
0	0	#N/A	0	#N/A	0
0	0	#N/A	0	#N/A	0
0	0	#N/A	0	#N/A	0
0	0	#N/A	0	#N/A	0
0	0	#N/A	0	#N/A	0
0	0	#N/A	0	#N/A	0
0	0	#N/A	0	#N/A	0
0	0	#N/A	0	#N/A	0
0	0	#N/A	0	#N/A	0
0	0	#N/A	0	#N/A	0
0	0	#N/A	0	#N/A	0
0	0	#N/A	0	#N/A	0
0	0		0		0
0	0		0		0
0	0	#N/A	0	#N/A	0
0	0	#N/A	0	#N/A	0
0	0	#N/A	0	#N/A	0
0	0	#N/A	0	#N/A	0
0	0	#N/A	0	#N/A	0
0	0	#N/A	0	#N/A	0
0	0	#N/A	0	#N/A	0
0	0	#N/A	0	#N/A	0
0	0	#N/A	0	#N/A	0
0	0		0	LOST-3_art	1
0	0		0		0
0	0		0	LOST-3_art	1
0	0		0	LOST-3_art	1
0	0		0	LOST-3_art	1
0	0	#N/A	0	#N/A	0
0	0	#N/A	0	#N/A	0
0	0	#N/A	0	#N/A	0
0	0	#N/A	0	#N/A	0

0	0	#N/A	0	#N/A	0
1	0		0	LOST-3_art	1
0	0	#N/A	0	#N/A	0
0	0	#N/A	0	#N/A	0
0	0	#N/A	0	#N/A	0
0	0	#N/A	0	#N/A	0
0	0	#N/A	0	#N/A	0
0	0	#N/A	0	#N/A	0
0	0	#N/A	0	#N/A	0
0	0	#N/A	0	#N/A	0
0	0	#N/A	0	#N/A	0
0	0	#N/A	0	#N/A	0
0	0	#N/A	0	#N/A	0
0	0	#N/A	0	#N/A	0
0	0	#N/A	0	#N/A	0
0	0	#N/A	0	#N/A	0
0	0	#N/A	0	#N/A	0
0	0	#N/A	0	#N/A	0
1	1	#N/A	0	#N/A	0
1	1	#N/A	0	#N/A	0
0	0	#N/A	0	#N/A	0
0	0	#N/A	0	#N/A	0
0	0	#N/A	0	#N/A	0
0	0	#N/A	0	#N/A	0
1	1	#N/A	0	#N/A	0
0	0	#N/A	0	#N/A	0
0	0	#N/A	0	#N/A	0
0	0	LOST_art	1	LOST-3_art	1
0	0	LOST_art	1	LOST-3_art	1
0	0	#N/A	0	#N/A	0
0	0	#N/A	0	#N/A	0
0	0	#N/A	0	#N/A	0
0	0	#N/A	0	#N/A	0
0	0	#N/A	0	#N/A	0
0	0	#N/A	0	#N/A	0
1	1	#N/A	0	#N/A	0
1	1	#N/A	0	#N/A	0
0	0	#N/A	0	#N/A	0
0	0	#N/A	0	#N/A	0
0	0	#N/A	0	#N/A	0
0	0		0		0
0	0		0		0
0	0		0		0
0	0	#N/A	0	#N/A	0
0	0	#N/A	0	#N/A	0
0	0	#N/A	0	#N/A	0
0	0	LOST_art	1	LOST-3_art	1

0	0	LOST_art	1	LOST-3_art	1
1	1	#N/A	0	#N/A	0
1	1	#N/A	0	#N/A	0
1	1	#N/A	0	#N/A	0
0	0		0		0
0	0		0		0
0	0	#N/A	0	#N/A	0
0	0	#N/A	0	#N/A	0
0	0	#N/A	0	#N/A	0
0	0	#N/A	0	#N/A	0
0	0	#N/A	0	#N/A	0
0	0	#N/A	0	#N/A	0
0	0	#N/A	0	#N/A	0
0	0	#N/A	0	#N/A	0
0	0	#N/A	0	#N/A	0
0	0	#N/A	0	#N/A	0
0	0	#N/A	0	#N/A	0
0	0	#N/A	0	#N/A	0
0	0		0	LOST-3_art	1
0	0		0	LOST-3_art	1
0	0		0	LOST-3_art	1
0	0	#N/A	0	#N/A	0
0	0	LOST_art	1	LOST-3_art	1
0	0		0	LOST-3_art	1
0	0		0	LOST-3_art	1
0	0		0	LOST-3_art	1
0	0		0	LOST-3_art	1
0	0	LOST_art	1	LOST-3_art	1
0	0	#N/A	0	#N/A	0
0	0	#N/A	0	#N/A	0
0	0	#N/A	0	#N/A	0
0	0	#N/A	0	#N/A	0
0	0	LOST_art	1	LOST-3_art	1
0	0	LOST_art	1	LOST-3_art	1
0	0	LOST_art	1	LOST-3_art	1
0	0		0		0
0	0		0		0
0	0	#N/A	0	#N/A	0
0	0	LOST_art	1	LOST-3_art	1
0	0	LOST_art	1	LOST-3_art	1
0	0	LOST_art	1	LOST-3_art	1
0	0		0		0
0	0		0		0
0	0	LOST_art	1	LOST-3_art	1
0	0	LOST_art	1	LOST-3_art	1
0	0	LOST_art	1	LOST-3_art	1
0	0	LOST_art	1	LOST-3_art	1

0	0	#N/A	0	#N/A	0
0	0		0		0
0	0	#N/A	0	#N/A	0
0	0	#N/A	0	#N/A	0
1	1	#N/A	0	#N/A	0
1	1	#N/A	0	#N/A	0
0	0	#N/A	0	#N/A	0
1	1	#N/A	0	#N/A	0
0	0	#N/A	0	#N/A	0
0	0	#N/A	0	#N/A	0
0	0	#N/A	0	#N/A	0
0	0	#N/A	0	#N/A	0
0	0	LOST_art	1	LOST-3_art	1
0	0	LOST_art	1	LOST-3_art	1
0	0	LOST_art	1	LOST-3_art	1
0	0	#N/A	0	#N/A	0
0	0	#N/A	0	#N/A	0
0	0	#N/A	0	#N/A	0
0	0	#N/A	0	#N/A	0
0	0	#N/A	0	#N/A	0
0	0	#N/A	0	#N/A	0
0	0	#N/A	0	#N/A	0
0	0	#N/A	0	#N/A	0
0	0	#N/A	0	#N/A	0
0	0	#N/A	0	#N/A	0
0	0	#N/A	0	#N/A	0
0	0	#N/A	0	#N/A	0
0	0		0	LOST-3_art	1
0	0		0	LOST-3_art	1
0	0	#N/A	0	#N/A	0
0	0	#N/A	0	#N/A	0
0	0	#N/A	0	#N/A	0
0	0	#N/A	0	#N/A	0
0	0	#N/A	0	#N/A	0
0	0	#N/A	0	#N/A	0
0	0	#N/A	0	#N/A	0
0	0	#N/A	0	#N/A	0
0	0	#N/A	0	#N/A	0
0	0	#N/A	0	#N/A	0
0	0	LOST_art	1	LOST-3_art	1
0	0	LOST_art	1	LOST-3_art	1
0	0	LOST_art	1	LOST-3_art	1
0	0	#N/A	0	#N/A	0
0	0	#N/A	0	#N/A	0
0	0	#N/A	0	#N/A	0
0	0	#N/A	0	#N/A	0
0	0	#N/A	0	#N/A	0

0	0	#N/A	0	#N/A	0
0	0	#N/A	0	#N/A	0
0	0	#N/A	0	#N/A	0
0	0	#N/A	0	#N/A	0
0	0	#N/A	0	#N/A	0
0	0	#N/A	0	#N/A	0
0	0	#N/A	0	#N/A	0
0	0		0		0
0	0		0		0
0	0	#N/A	0	#N/A	0
0	0		0		0
1	0	#N/A	0	#N/A	0
0	0	#N/A	0	#N/A	0
0	0	#N/A	0	#N/A	0
0	0	#N/A	0	#N/A	0
0	0	#N/A	0	#N/A	0
0	0		0	LOST-3_art	1
0	0		0	LOST-3_art	1
1	0	LOST_art	1	LOST-3_art	1
1	0	LOST_art	1	LOST-3_art	1
1	0	LOST_art	1	LOST-3_art	1
1	0	LOST_art	1	LOST-3_art	1
0	0	#N/A	0	#N/A	0
0	0	#N/A	0	#N/A	0
0	0	#N/A	0	#N/A	0
0	0	#N/A	0	#N/A	0
0	0	#N/A	0	#N/A	0
0	0	#N/A	0	#N/A	0
0	0	#N/A	0	#N/A	0
0	0	#N/A	0	#N/A	0
0	0	#N/A	0	#N/A	0
0	0	#N/A	0	#N/A	0

lost6_art	lost6_art_b	lost_7_art	lost_7_art_b	nop_elostart	ewalostart	ewalostart_b	ewplostart
	0	LOST>6_art	1			0	
	0	LOST>6_art	1			0	
#N/A	0	#N/A	0		#N/A	0	#N/A
#N/A	0	#N/A	0		#N/A	0	#N/A
LOST-6_art	1	LOST>6_art	1		Ew/a-LOST-ar	1	
LOST-6_art	1	LOST>6_art	1		Ew/a-LOST-ar	1	
LOST-6_art	1	LOST>6_art	1		Ew/a-LOST-ar	1	
LOST-6_art	1	LOST>6_art	1		Ew/a-LOST-ar	1	
	0	LOST>6_art	1		Ew/a-LOST-ar	1	
	0	LOST>6_art	1		Ew/a-LOST-ar	1	
#N/A	0	#N/A	0		#N/A	0	#N/A
#N/A	0	#N/A	0		#N/A	0	#N/A
#N/A	0	#N/A	0		#N/A	0	#N/A
LOST-6_art	1		0			0	
#N/A	0	#N/A	0		#N/A	0	#N/A
#N/A	0	#N/A	0		#N/A	0	#N/A
#N/A	0	#N/A	0		#N/A	0	#N/A
#N/A	0	#N/A	0		#N/A	0	#N/A
	0		0		Ew/a-LOST-ar	1	Ew/p-LOST-ar
	0		0		Ew/a-LOST-ar	1	Ew/p-LOST-ar
#N/A	0	#N/A	0		#N/A	0	#N/A
#N/A	0	#N/A	0		#N/A	0	#N/A
#N/A	0	#N/A	0		#N/A	0	#N/A
#N/A	0	#N/A	0		#N/A	0	#N/A
#N/A	0	#N/A	0		#N/A	0	#N/A
#N/A	0	#N/A	0		#N/A	0	#N/A
#N/A	0	#N/A	0		#N/A	0	#N/A
#N/A	0	#N/A	0		#N/A	0	#N/A
#N/A	0	#N/A	0		#N/A	0	#N/A
LOST-6_art	1		0		Ew/a-LOST-ar	1	Ew/p-LOST-ar
	0	LOST>6_art	1			0	
LOST-6_art	1		0			0	
#N/A	0	#N/A	0		#N/A	0	#N/A
#N/A	0	#N/A	0		#N/A	0	#N/A
#N/A	0	#N/A	0		#N/A	0	#N/A
#N/A	0	#N/A	0		#N/A	0	#N/A
#N/A	0	#N/A	0		#N/A	0	#N/A
#N/A	0	#N/A	0		#N/A	0	#N/A
#N/A	0	#N/A	0		#N/A	0	#N/A
#N/A	0	#N/A	0		#N/A	0	#N/A
#N/A	0	#N/A	0		#N/A	0	#N/A
#N/A	0	#N/A	0		#N/A	0	#N/A
#N/A	0	#N/A	0		#N/A	0	#N/A
#N/A	0	#N/A	0		#N/A	0	#N/A
#N/A	0	#N/A	0		#N/A	0	#N/A
#N/A	0	#N/A	0		#N/A	0	#N/A

#N/A	0	#N/A	0	#N/A	0	#N/A
#N/A	0	#N/A	0	#N/A	0	#N/A
#N/A	0	#N/A	0	#N/A	0	#N/A
#N/A	0	#N/A	0	#N/A	0	#N/A
#N/A	0	#N/A	0	#N/A	0	#N/A
#N/A	0	#N/A	0	#N/A	0	#N/A
#N/A	0	#N/A	0	#N/A	0	#N/A
LOST-6_art	1	LOST>6_art	1		0	Ew/p-LOST-ar
	0		0	Ew/a-LOST-ar	1	Ew/p-LOST-ar
#N/A	0	#N/A	0	#N/A	0	#N/A
#N/A	0	#N/A	0	#N/A	0	#N/A
#N/A	0	#N/A	0	#N/A	0	#N/A
LOST-6_art	1	LOST>6_art	1		0	Ew/p-LOST-ar
#N/A	0	#N/A	0	#N/A	0	#N/A
#N/A	0	#N/A	0	#N/A	0	#N/A
#N/A	0	#N/A	0	#N/A	0	#N/A
#N/A	0	#N/A	0	#N/A	0	#N/A
#N/A	0	#N/A	0	#N/A	0	#N/A
#N/A	0	#N/A	0	#N/A	0	#N/A
#N/A	0	#N/A	0	#N/A	0	#N/A
	0	LOST>6_art	1	Ew/a-LOST-ar	1	
	0	LOST>6_art	1	Ew/a-LOST-ar	1	
#N/A	0	#N/A	0	#N/A	0	#N/A
LOST-6_art	1	LOST>6_art	1		0	
#N/A	0	#N/A	0	#N/A	0	#N/A
#N/A	0	#N/A	0	#N/A	0	#N/A
	0		0		0	
#N/A	0	#N/A	0	#N/A	0	#N/A
#N/A	0	#N/A	0	#N/A	0	#N/A
	0		0	Ew/a-LOST-ar	1	Ew/p-LOST-ar
	0		0	Ew/a-LOST-ar	1	Ew/p-LOST-ar
	0		0	Ew/a-LOST-ar	1	Ew/p-LOST-ar
	0		0	Ew/a-LOST-ar	1	Ew/p-LOST-ar
	0		0	Ew/a-LOST-ar	1	Ew/p-LOST-ar
#N/A	0	#N/A	0	#N/A	0	#N/A
#N/A	0	#N/A	0	#N/A	0	#N/A
#N/A	0	#N/A	0	#N/A	0	#N/A
#N/A	0	#N/A	0	#N/A	0	#N/A
#N/A	0	#N/A	0	#N/A	0	#N/A
#N/A	0	#N/A	0	#N/A	0	#N/A
#N/A	0	#N/A	0	#N/A	0	#N/A
#N/A	0	#N/A	0	#N/A	0	#N/A
#N/A	0	#N/A	0	#N/A	0	#N/A
#N/A	0	#N/A	0	#N/A	0	#N/A
#N/A	0	#N/A	0	#N/A	0	#N/A
#N/A	0	#N/A	0	#N/A	0	#N/A
#N/A	0	#N/A	0	#N/A	0	#N/A

#N/A	0	#N/A	0	#N/A	0	#N/A
#N/A	0	#N/A	0	#N/A	0	#N/A
#N/A	0	#N/A	0	#N/A	0	#N/A
#N/A	0	#N/A	0	#N/A	0	#N/A
#N/A	0	#N/A	0	#N/A	0	#N/A
#N/A	0	#N/A	0	#N/A	0	#N/A
LOST-6_art	1		0	Ew/a-LOST-ar	1	Ew/p-LOST-ar
LOST-6_art	1		0	Ew/a-LOST-ar	1	Ew/p-LOST-ar
#N/A	0	#N/A	0	#N/A	0	#N/A
#N/A	0	#N/A	0	#N/A	0	#N/A
#N/A	0	#N/A	0	#N/A	0	#N/A
#N/A	0	#N/A	0	#N/A	0	#N/A
#N/A	0	#N/A	0	#N/A	0	#N/A
#N/A	0	#N/A	0	#N/A	0	#N/A
#N/A	0	#N/A	0	#N/A	0	#N/A
#N/A	0	#N/A	0	#N/A	0	#N/A
#N/A	0	#N/A	0	#N/A	0	#N/A
#N/A	0	#N/A	0	#N/A	0	#N/A
#N/A	0	#N/A	0	#N/A	0	#N/A
#N/A	0	#N/A	0	#N/A	0	#N/A
#N/A	0	#N/A	0	#N/A	0	#N/A
#N/A	0	#N/A	0	#N/A	0	#N/A
#N/A	0	#N/A	0	#N/A	0	#N/A
#N/A	0	#N/A	0	#N/A	0	#N/A
#N/A	0	#N/A	0	#N/A	0	#N/A
#N/A	0	#N/A	0	#N/A	0	#N/A
#N/A	0	#N/A	0	#N/A	0	#N/A
LOST-6_art	1	LOST>6_art	1		0	
LOST-6_art	1	LOST>6_art	1		0	
LOST-6_art	1	LOST>6_art	1	Ew/a-LOST-ar	1	Ew/p-LOST-ar
LOST-6_art	1	LOST>6_art	1	Ew/a-LOST-ar	1	Ew/p-LOST-ar
#N/A	0	#N/A	0	#N/A	0	#N/A
#N/A	0	#N/A	0	#N/A	0	#N/A
#N/A	0	#N/A	0	#N/A	0	#N/A
#N/A	0	#N/A	0	#N/A	0	#N/A
	0		0	Ew/a-LOST-ar	1	
	0		0	Ew/a-LOST-ar	1	
LOST-6_art	1		0	Ew/a-LOST-ar	1	Ew/p-LOST-ar
LOST-6_art	1		0	Ew/a-LOST-ar	1	Ew/p-LOST-ar
#N/A	0	#N/A	0	#N/A	0	#N/A
#N/A	0	#N/A	0	#N/A	0	#N/A
#N/A	0	#N/A	0	#N/A	0	#N/A
LOST-6_art	1	LOST>6_art	1		0	
#N/A	0	#N/A	0	#N/A	0	#N/A
LOST-6_art	1	LOST>6_art	1		0	
LOST-6_art	1	LOST>6_art	1		0	
#N/A	0	#N/A	0	#N/A	0	#N/A

#N/A	0	#N/A	0	#N/A	0	#N/A
#N/A	0	#N/A	0	#N/A	0	#N/A
LOST-6_art	1	LOST>6_art	1	Ew/a-LOST-ar	1	
LOST-6_art	1	LOST>6_art	1	Ew/a-LOST-ar	1	
LOST-6_art	1	LOST>6_art	1	Ew/a-LOST-ar	1	
#N/A	0	#N/A	0	#N/A	0	#N/A
#N/A	0	#N/A	0	#N/A	0	#N/A
#N/A	0	#N/A	0	#N/A	0	#N/A
#N/A	0	#N/A	0	#N/A	0	#N/A
#N/A	0	#N/A	0	#N/A	0	#N/A
#N/A	0	#N/A	0	#N/A	0	#N/A
#N/A	0	#N/A	0	#N/A	0	#N/A
#N/A	0	#N/A	0	#N/A	0	#N/A
	0		0		0	Ew/p-LOST-ar
#N/A	0	#N/A	0	#N/A	0	#N/A
#N/A	0	#N/A	0	#N/A	0	#N/A
	0		0	Ew/a-LOST-ar	1	Ew/p-LOST-ar
#N/A	0	#N/A	0	#N/A	0	#N/A
#N/A	0	#N/A	0	#N/A	0	#N/A
#N/A	0	#N/A	0	#N/A	0	#N/A
#N/A	0	#N/A	0	#N/A	0	#N/A
#N/A	0	#N/A	0	#N/A	0	#N/A
LOST-6_art	1		0		0	Ew/p-LOST-ar
#N/A	0	#N/A	0	#N/A	0	#N/A
#N/A	0	#N/A	0	#N/A	0	#N/A
#N/A	0	#N/A	0	#N/A	0	#N/A
#N/A	0	#N/A	0	#N/A	0	#N/A
#N/A	0	#N/A	0	#N/A	0	#N/A
LOST-6_art	1		0		0	
#N/A	0	#N/A	0	#N/A	0	#N/A
	0		0		0	
	0		0		0	
#N/A	0	#N/A	0	#N/A	0	#N/A
#N/A	0	#N/A	0	#N/A	0	#N/A
#N/A	0	#N/A	0	#N/A	0	#N/A
#N/A	0	#N/A	0	#N/A	0	#N/A
#N/A	0	#N/A	0	#N/A	0	#N/A
#N/A	0	#N/A	0	#N/A	0	#N/A
#N/A	0	#N/A	0	#N/A	0	#N/A
#N/A	0	#N/A	0	#N/A	0	#N/A
#N/A	0	#N/A	0	#N/A	0	#N/A
	0		0	Ew/a-LOST-ar	1	Ew/p-LOST-ar
	0		0	Ew/a-LOST-ar	1	Ew/p-LOST-ar
#N/A	0	#N/A	0	#N/A	0	#N/A
#N/A	0	#N/A	0	#N/A	0	#N/A
#N/A	0	#N/A	0	#N/A	0	#N/A
#N/A	0	#N/A	0	#N/A	0	#N/A

#N/A	0	#N/A	0	#N/A	0	#N/A
#N/A	0	#N/A	0	#N/A	0	#N/A
#N/A	0	#N/A	0	#N/A	0	#N/A
#N/A	0	#N/A	0	#N/A	0	#N/A
#N/A	0	#N/A	0	#N/A	0	#N/A
#N/A	0	#N/A	0	#N/A	0	#N/A
#N/A	0	#N/A	0	#N/A	0	#N/A
#N/A	0	#N/A	0	#N/A	0	#N/A
#N/A	0	#N/A	0	#N/A	0	#N/A
#N/A	0	#N/A	0	#N/A	0	#N/A
#N/A	0	#N/A	0	#N/A	0	#N/A
#N/A	0	#N/A	0	#N/A	0	#N/A
#N/A	0	#N/A	0	#N/A	0	#N/A
#N/A	0	#N/A	0	#N/A	0	#N/A
#N/A	0	#N/A	0	#N/A	0	#N/A
	0		0		0	
	0		0		0	
#N/A	0	#N/A	0	#N/A	0	#N/A
#N/A	0	#N/A	0	#N/A	0	#N/A
#N/A	0	#N/A	0	#N/A	0	#N/A
#N/A	0	#N/A	0	#N/A	0	#N/A
#N/A	0	#N/A	0	#N/A	0	#N/A
#N/A	0	#N/A	0	#N/A	0	#N/A
#N/A	0	#N/A	0	#N/A	0	#N/A
	0		0	Ew/a-LOST-ar	1	
	0		0	Ew/a-LOST-ar	1	
#N/A	0	#N/A	0	#N/A	0	#N/A
#N/A	0	#N/A	0	#N/A	0	#N/A
#N/A	0	#N/A	0	#N/A	0	#N/A
#N/A	0	#N/A	0	#N/A	0	#N/A
#N/A	0	#N/A	0	#N/A	0	#N/A
#N/A	0	#N/A	0	#N/A	0	#N/A
#N/A	0	#N/A	0	#N/A	0	#N/A
#N/A	0	#N/A	0	#N/A	0	#N/A
LOST-6_art	1	LOST>6_art	1		0	
#N/A	0	#N/A	0	#N/A	0	#N/A
#N/A	0	#N/A	0	#N/A	0	#N/A
LOST-6_art	1		0		0	
	0		0	Ew/a-LOST-ar	1	Ew/p-LOST-ar
	0		0	Ew/a-LOST-ar	1	Ew/p-LOST-ar
	0		0	Ew/a-LOST-ar	1	Ew/p-LOST-ar
	0		0	Ew/a-LOST-ar	1	Ew/p-LOST-ar
#N/A	0	#N/A	0	#N/A	0	#N/A
#N/A	0	#N/A	0	#N/A	0	#N/A
#N/A	0	#N/A	0	#N/A	0	#N/A
#N/A	0	#N/A	0	#N/A	0	#N/A
#N/A	0	#N/A	0	#N/A	0	#N/A

#N/A	0	#N/A	0	#N/A	0	#N/A
#N/A	0	#N/A	0	#N/A	0	#N/A
#N/A	0	#N/A	0	#N/A	0	#N/A
#N/A	0	#N/A	0	#N/A	0	#N/A
#N/A	0	#N/A	0	#N/A	0	#N/A
#N/A	0	#N/A	0	#N/A	0	#N/A
#N/A	0	#N/A	0	#N/A	0	#N/A
#N/A	0	#N/A	0	#N/A	0	#N/A
#N/A	0	#N/A	0	#N/A	0	#N/A
	0		0	Ew/a-LOST-ar	1	Ew/p-LOST-ar
LOST-6_art	1		0		0	
#N/A	0	#N/A	0	#N/A	0	#N/A
#N/A	0	#N/A	0	#N/A	0	#N/A
#N/A	0	#N/A	0	#N/A	0	#N/A
#N/A	0	#N/A	0	#N/A	0	#N/A
	0	LOST>6_art	1		0	
#N/A	0	#N/A	0	#N/A	0	#N/A
#N/A	0	#N/A	0	#N/A	0	#N/A
#N/A	0	#N/A	0	#N/A	0	#N/A
#N/A	0	#N/A	0	#N/A	0	#N/A
#N/A	0	#N/A	0	#N/A	0	#N/A
	0		0	Ew/a-LOST-ar	1	Ew/p-LOST-ar
	0		0	Ew/a-LOST-ar	1	Ew/p-LOST-ar
	0		0	Ew/a-LOST-ar	1	Ew/p-LOST-ar
#N/A	0	#N/A	0	#N/A	0	#N/A
#N/A	0	#N/A	0	#N/A	0	#N/A
#N/A	0	#N/A	0	#N/A	0	#N/A
#N/A	0	#N/A	0	#N/A	0	#N/A
#N/A	0	#N/A	0	#N/A	0	#N/A
#N/A	0	#N/A	0	#N/A	0	#N/A
LOST-6_art	1	LOST>6_art	1	Ew/a-LOST-ar	1	Ew/p-LOST-ar
LOST-6_art	1	LOST>6_art	1	Ew/a-LOST-ar	1	Ew/p-LOST-ar
LOST-6_art	1	LOST>6_art	1	Ew/a-LOST-ar	1	Ew/p-LOST-ar
#N/A	0	#N/A	0	#N/A	0	#N/A
#N/A	0	#N/A	0	#N/A	0	#N/A
#N/A	0	#N/A	0	#N/A	0	#N/A
#N/A	0	#N/A	0	#N/A	0	#N/A
#N/A	0	#N/A	0	#N/A	0	#N/A
#N/A	0	#N/A	0	#N/A	0	#N/A
#N/A	0	#N/A	0	#N/A	0	#N/A
#N/A	0	#N/A	0	#N/A	0	#N/A
#N/A	0	#N/A	0	#N/A	0	#N/A
#N/A	0	#N/A	0	#N/A	0	#N/A
LOST-6_art	1	LOST>6_art	1		0	
#N/A	0	#N/A	0	#N/A	0	#N/A
#N/A	0	#N/A	0	#N/A	0	#N/A
#N/A	0	#N/A	0	#N/A	0	#N/A

#N/A	0	#N/A	0	#N/A	0	#N/A
#N/A	0	#N/A	0	#N/A	0	#N/A
#N/A	0	#N/A	0	#N/A	0	#N/A
	0	LOST>6_art	1		0	
#N/A	0	#N/A	0	#N/A	0	#N/A
#N/A	0	#N/A	0	#N/A	0	#N/A
#N/A	0	#N/A	0	#N/A	0	#N/A
#N/A	0	#N/A	0	#N/A	0	#N/A
#N/A	0	#N/A	0	#N/A	0	#N/A
#N/A	0	#N/A	0	#N/A	0	#N/A
#N/A	0	#N/A	0	#N/A	0	#N/A
#N/A	0	#N/A	0	#N/A	0	#N/A
#N/A	0	#N/A	0	#N/A	0	#N/A
#N/A	0	#N/A	0	#N/A	0	#N/A
#N/A	0	#N/A	0	#N/A	0	#N/A
	0	LOST>6_art	1		0	
	0	LOST>6_art	1		0	
#N/A	0	#N/A	0	#N/A	0	#N/A
#N/A	0	#N/A	0	#N/A	0	#N/A
#N/A	0	#N/A	0	#N/A	0	#N/A
#N/A	0	#N/A	0	#N/A	0	#N/A
#N/A	0	#N/A	0	#N/A	0	#N/A
LOST-6_art	1		0		0	
#N/A	0	#N/A	0	#N/A	0	#N/A
#N/A	0	#N/A	0	#N/A	0	#N/A
#N/A	0	#N/A	0	#N/A	0	#N/A
#N/A	0	#N/A	0	#N/A	0	#N/A
#N/A	0	#N/A	0	#N/A	0	#N/A
#N/A	0	#N/A	0	#N/A	0	#N/A
#N/A	0	#N/A	0	#N/A	0	#N/A
#N/A	0	#N/A	0	#N/A	0	#N/A
#N/A	0	#N/A	0	#N/A	0	#N/A
#N/A	0	#N/A	0	#N/A	0	#N/A
#N/A	0	#N/A	0	#N/A	0	#N/A
	0		0		0	
	0		0		0	
#N/A	0	#N/A	0	#N/A	0	#N/A
LOST-6_art	1		0		0	
#N/A	0	#N/A	0	#N/A	0	#N/A
#N/A	0	#N/A	0	#N/A	0	#N/A
#N/A	0	#N/A	0	#N/A	0	#N/A
#N/A	0	#N/A	0	#N/A	0	#N/A
#N/A	0	#N/A	0	#N/A	0	#N/A
#N/A	0	#N/A	0	#N/A	0	#N/A
#N/A	0	#N/A	0	#N/A	0	#N/A
#N/A	0	#N/A	0	#N/A	0	#N/A
#N/A	0	#N/A	0	#N/A	0	#N/A

#N/A	0	#N/A	0	#N/A	0	#N/A
	0		0	Ew/a-LOST-ar	1	
	0		0	Ew/a-LOST-ar	1	
#N/A	0	#N/A	0	#N/A	0	#N/A
#N/A	0	#N/A	0	#N/A	0	#N/A
#N/A	0	#N/A	0	#N/A	0	#N/A
#N/A	0	#N/A	0	#N/A	0	#N/A
#N/A	0	#N/A	0	#N/A	0	#N/A
#N/A	0	#N/A	0	#N/A	0	#N/A
#N/A	0	#N/A	0	#N/A	0	#N/A
#N/A	0	#N/A	0	#N/A	0	#N/A
#N/A	0	#N/A	0	#N/A	0	#N/A
#N/A	0	#N/A	0	#N/A	0	#N/A
	0		0		0	
	0		0	Ew/a-LOST-ar	1	Ew/p-LOST-ar
	0		0	Ew/a-LOST-ar	1	Ew/p-LOST-ar
	0	LOST>6_art	1		0	
#N/A	0	#N/A	0	#N/A	0	#N/A
	0		0		0	
#N/A	0	#N/A	0	#N/A	0	#N/A
#N/A	0	#N/A	0	#N/A	0	#N/A
	0	LOST>6_art	1		0	
#N/A	0	#N/A	0	#N/A	0	#N/A
#N/A	0	#N/A	0	#N/A	0	#N/A
#N/A	0	#N/A	0	#N/A	0	#N/A
#N/A	0	#N/A	0	#N/A	0	#N/A
	0	LOST>6_art	1		0	Ew/p-LOST-ar
#N/A	0	#N/A	0	#N/A	0	#N/A
#N/A	0	#N/A	0	#N/A	0	#N/A
#N/A	0	#N/A	0	#N/A	0	#N/A
#N/A	0	#N/A	0	#N/A	0	#N/A
#N/A	0	#N/A	0	#N/A	0	#N/A
#N/A	0	#N/A	0	#N/A	0	#N/A
#N/A	0	#N/A	0	#N/A	0	#N/A
#N/A	0	#N/A	0	#N/A	0	#N/A
#N/A	0	#N/A	0	#N/A	0	#N/A
	0	LOST>6_art	1		0	
#N/A	0	#N/A	0	#N/A	0	#N/A
#N/A	0	#N/A	0	#N/A	0	#N/A
#N/A	0	#N/A	0	#N/A	0	#N/A
#N/A	0	#N/A	0	#N/A	0	#N/A
#N/A	0	#N/A	0	#N/A	0	#N/A
#N/A	0	#N/A	0	#N/A	0	#N/A
LOST-6_art	1	LOST>6_art	1		0	
#N/A	0	#N/A	0	#N/A	0	#N/A
#N/A	0	#N/A	0	#N/A	0	#N/A
#N/A	0	#N/A	0	#N/A	0	#N/A

#N/A	0	#N/A	0	#N/A	0	#N/A
LOST-6_art	1	LOST>6_art	1		0	
#N/A	0	#N/A	0	#N/A	0	#N/A
#N/A	0	#N/A	0	#N/A	0	#N/A
#N/A	0	#N/A	0	#N/A	0	#N/A
LOST-6_art	1	LOST>6_art	1		0	
LOST-6_art	1	LOST>6_art	1		0	
	0		0	Ew/a-LOST-ar	1	Ew/p-LOST-ar
	0		0	Ew/a-LOST-ar	1	Ew/p-LOST-ar
#N/A	0	#N/A	0	#N/A	0	#N/A
#N/A	0	#N/A	0	#N/A	0	#N/A
#N/A	0	#N/A	0	#N/A	0	#N/A
#N/A	0	#N/A	0	#N/A	0	#N/A
#N/A	0	#N/A	0	#N/A	0	#N/A
#N/A	0	#N/A	0	#N/A	0	#N/A
#N/A	0	#N/A	0	#N/A	0	#N/A
#N/A	0	#N/A	0	#N/A	0	#N/A
#N/A	0	#N/A	0	#N/A	0	#N/A
#N/A	0	#N/A	0	#N/A	0	#N/A
#N/A	0	#N/A	0	#N/A	0	#N/A
#N/A	0	#N/A	0	#N/A	0	#N/A
LOST-6_art	1		0	Ew/a-LOST-ar	1	Ew/p-LOST-ar
#N/A	0	#N/A	0	#N/A	0	#N/A
#N/A	0	#N/A	0	#N/A	0	#N/A
#N/A	0	#N/A	0	#N/A	0	#N/A
#N/A	0	#N/A	0	#N/A	0	#N/A
#N/A	0	#N/A	0	#N/A	0	#N/A
LOST-6_art	1	LOST>6_art	1		0	
#N/A	0	#N/A	0	#N/A	0	#N/A
#N/A	0	#N/A	0	#N/A	0	#N/A
#N/A	0	#N/A	0	#N/A	0	#N/A
#N/A	0	#N/A	0	#N/A	0	#N/A
#N/A	0	#N/A	0	#N/A	0	#N/A
#N/A	0	#N/A	0	#N/A	0	#N/A
#N/A	0	#N/A	0	#N/A	0	#N/A
#N/A	0	#N/A	0	#N/A	0	#N/A
#N/A	0	#N/A	0	#N/A	0	#N/A
	0	LOST>6_art	1		0	
#N/A	0	#N/A	0	#N/A	0	#N/A
#N/A	0	#N/A	0	#N/A	0	#N/A
#N/A	0	#N/A	0	#N/A	0	#N/A
#N/A	0	#N/A	0	#N/A	0	#N/A
#N/A	0	#N/A	0	#N/A	0	#N/A
#N/A	0	#N/A	0	#N/A	0	#N/A
#N/A	0	#N/A	0	#N/A	0	#N/A
	0		0		0	
	0		0		0	

#N/A	0	#N/A	0	#N/A	0	#N/A
#N/A	0	#N/A	0	#N/A	0	#N/A
#N/A	0	#N/A	0	#N/A	0	#N/A
#N/A	0	#N/A	0	#N/A	0	#N/A
#N/A	0	#N/A	0	#N/A	0	#N/A
LOST-6_art	1	LOST>6_art	1		0	
LOST-6_art	1	LOST>6_art	1		0	
#N/A	0	#N/A	0	#N/A	0	#N/A
#N/A	0	#N/A	0	#N/A	0	#N/A
#N/A	0	#N/A	0	#N/A	0	#N/A
#N/A	0	#N/A	0	#N/A	0	#N/A
LOST-6_art	1	LOST>6_art	1		0	
LOST-6_art	1	LOST>6_art	1		0	
#N/A	0	#N/A	0	#N/A	0	#N/A
LOST-6_art	1		0		0	
	0		0	Ew/a-LOST-ar	1	Ew/p-LOST-ar
	0		0	Ew/a-LOST-ar	1	Ew/p-LOST-ar
	0		0	Ew/a-LOST-ar	1	Ew/p-LOST-ar
	0		0	Ew/a-LOST-ar	1	Ew/p-LOST-ar
#N/A	0	#N/A	0	#N/A	0	#N/A
#N/A	0	#N/A	0	#N/A	0	#N/A
#N/A	0	#N/A	0	#N/A	0	#N/A
#N/A	0	#N/A	0	#N/A	0	#N/A
#N/A	0	#N/A	0	#N/A	0	#N/A
	0	LOST>6_art	1		0	
	0	LOST>6_art	1		0	
#N/A	0	#N/A	0	#N/A	0	#N/A
#N/A	0	#N/A	0	#N/A	0	#N/A
	0		0		0	
#N/A	0	#N/A	0	#N/A	0	#N/A
#N/A	0	#N/A	0	#N/A	0	#N/A
#N/A	0	#N/A	0	#N/A	0	#N/A
#N/A	0	#N/A	0	#N/A	0	#N/A
#N/A	0	#N/A	0	#N/A	0	#N/A
#N/A	0	#N/A	0	#N/A	0	#N/A
#N/A	0	#N/A	0	#N/A	0	#N/A
#N/A	0	#N/A	0	#N/A	0	#N/A
#N/A	0	#N/A	0	#N/A	0	#N/A
#N/A	0	#N/A	0	#N/A	0	#N/A
#N/A	0	#N/A	0	#N/A	0	#N/A
#N/A	0	#N/A	0	#N/A	0	#N/A
#N/A	0	#N/A	0	#N/A	0	#N/A
#N/A	0	#N/A	0	#N/A	0	#N/A
#N/A	0	#N/A	0	#N/A	0	#N/A
#N/A	0	#N/A	0	#N/A	0	#N/A
#N/A	0	#N/A	0	#N/A	0	#N/A
#N/A	0	#N/A	0	#N/A	0	#N/A

LOST-6_art	1		0		0	
LOST-6_art	1		0		0	
#N/A	0	#N/A	0	#N/A	0	#N/A
#N/A	0	#N/A	0	#N/A	0	#N/A
#N/A	0	#N/A	0	#N/A	0	#N/A
#N/A	0	#N/A	0	#N/A	0	#N/A
	0		0		0	
#N/A	0	#N/A	0	#N/A	0	#N/A
#N/A	0	#N/A	0	#N/A	0	#N/A
LOST-6_art	1		0		0	Ew/p-LOST-ar
#N/A	0	#N/A	0	#N/A	0	#N/A
#N/A	0	#N/A	0	#N/A	0	#N/A
#N/A	0	#N/A	0	#N/A	0	#N/A
#N/A	0	#N/A	0	#N/A	0	#N/A
#N/A	0	#N/A	0	#N/A	0	#N/A
#N/A	0	#N/A	0	#N/A	0	#N/A
#N/A	0	#N/A	0	#N/A	0	#N/A
#N/A	0	#N/A	0	#N/A	0	#N/A
#N/A	0	#N/A	0	#N/A	0	#N/A
#N/A	0	#N/A	0	#N/A	0	#N/A
#N/A	0	#N/A	0	#N/A	0	#N/A
#N/A	0	#N/A	0	#N/A	0	#N/A
	0		0		0	
LOST-6_art	1	LOST>6_art	1		0	
LOST-6_art	1	LOST>6_art	1		0	
LOST-6_art	1	LOST>6_art	1		0	
#N/A	0	#N/A	0	#N/A	0	#N/A
#N/A	0	#N/A	0	#N/A	0	#N/A
#N/A	0	#N/A	0	#N/A	0	#N/A
#N/A	0	#N/A	0	#N/A	0	#N/A
#N/A	0	#N/A	0	#N/A	0	#N/A
#N/A	0	#N/A	0	#N/A	0	#N/A
#N/A	0	#N/A	0	#N/A	0	#N/A
#N/A	0	#N/A	0	#N/A	0	#N/A
	0	LOST>6_art	1	Ew/a-LOST-ar	1	Ew/p-LOST-ar
#N/A	0	#N/A	0	#N/A	0	#N/A
#N/A	0	#N/A	0	#N/A	0	#N/A
#N/A	0	#N/A	0	#N/A	0	#N/A
#N/A	0	#N/A	0	#N/A	0	#N/A
#N/A	0	#N/A	0	#N/A	0	#N/A
#N/A	0	#N/A	0	#N/A	0	#N/A
LOST-6_art	1		0		0	
#N/A	0	#N/A	0	#N/A	0	#N/A
	0		0	Ew/a-LOST-ar	1	Ew/p-LOST-ar
#N/A	0	#N/A	0	#N/A	0	#N/A
#N/A	0	#N/A	0	#N/A	0	#N/A
#N/A	0	#N/A	0	#N/A	0	#N/A

#N/A	0	#N/A	0	#N/A	0	#N/A
	0	LOST>6_art	1	Ew/a-LOST-ar	1	
#N/A	0	#N/A	0	#N/A	0	#N/A
#N/A	0	#N/A	0	#N/A	0	#N/A
#N/A	0	#N/A	0	#N/A	0	#N/A
#N/A	0	#N/A	0	#N/A	0	#N/A
#N/A	0	#N/A	0	#N/A	0	#N/A
#N/A	0	#N/A	0	#N/A	0	#N/A
#N/A	0	#N/A	0	#N/A	0	#N/A
#N/A	0	#N/A	0	#N/A	0	#N/A
LOST-6_art	1		0		0	
LOST-6_art	1		0		0	
LOST-6_art	1		0		0	
#N/A	0	#N/A	0	#N/A	0	#N/A
#N/A	0	#N/A	0	#N/A	0	#N/A
#N/A	0	#N/A	0	#N/A	0	#N/A
#N/A	0	#N/A	0	#N/A	0	#N/A
#N/A	0	#N/A	0	#N/A	0	#N/A
#N/A	0	#N/A	0	#N/A	0	#N/A
	0	LOST>6_art	1		0	
#N/A	0	#N/A	0	#N/A	0	#N/A
#N/A	0	#N/A	0	#N/A	0	#N/A
#N/A	0	#N/A	0	#N/A	0	#N/A
#N/A	0	#N/A	0	#N/A	0	#N/A
#N/A	0	#N/A	0	#N/A	0	#N/A
#N/A	0	#N/A	0	#N/A	0	#N/A
#N/A	0	#N/A	0	#N/A	0	#N/A
#N/A	0	#N/A	0	#N/A	0	#N/A
#N/A	0	#N/A	0	#N/A	0	#N/A
#N/A	0	#N/A	0	#N/A	0	#N/A
#N/A	0	#N/A	0	#N/A	0	#N/A
#N/A	0	#N/A	0	#N/A	0	#N/A
#N/A	0	#N/A	0	#N/A	0	#N/A
#N/A	0	#N/A	0	#N/A	0	#N/A
#N/A	0	#N/A	0	#N/A	0	#N/A
#N/A	0	#N/A	0	#N/A	0	#N/A
LOST-6_art	1		0		0	
#N/A	0	#N/A	0	#N/A	0	#N/A
#N/A	0	#N/A	0	#N/A	0	#N/A
	0	LOST>6_art	1		0	Ew/p-LOST-ar
#N/A	0	#N/A	0	#N/A	0	#N/A
#N/A	0	#N/A	0	#N/A	0	#N/A
#N/A	0	#N/A	0	#N/A	0	#N/A
#N/A	0	#N/A	0	#N/A	0	#N/A
#N/A	0	#N/A	0	#N/A	0	#N/A
#N/A	0	#N/A	0	#N/A	0	#N/A
#N/A	0	#N/A	0	#N/A	0	#N/A

#N/A	0	#N/A	0	#N/A	0	#N/A
#N/A	0	#N/A	0	#N/A	0	#N/A
#N/A	0	#N/A	0	#N/A	0	#N/A
#N/A	0	#N/A	0	#N/A	0	#N/A
#N/A	0	#N/A	0	#N/A	0	#N/A
#N/A	0	#N/A	0	#N/A	0	#N/A
#N/A	0	#N/A	0	#N/A	0	#N/A
#N/A	0	#N/A	0	#N/A	0	#N/A
#N/A	0	#N/A	0	#N/A	0	#N/A
#N/A	0	#N/A	0	#N/A	0	#N/A
#N/A	0	#N/A	0	#N/A	0	#N/A
#N/A	0	#N/A	0	#N/A	0	#N/A
#N/A	0	#N/A	0	#N/A	0	#N/A
#N/A	0	#N/A	0	#N/A	0	#N/A
#N/A	0	#N/A	0	#N/A	0	#N/A
#N/A	0	#N/A	0	#N/A	0	#N/A
#N/A	0	#N/A	0	#N/A	0	#N/A
#N/A	0	#N/A	0	#N/A	0	#N/A
#N/A	0	#N/A	0	#N/A	0	#N/A
#N/A	0	#N/A	0	#N/A	0	#N/A
#N/A	0	#N/A	0	#N/A	0	#N/A
#N/A	0	#N/A	0	#N/A	0	#N/A
#N/A	0	#N/A	0	#N/A	0	#N/A
	0		0		0	
#N/A	0	#N/A	0	#N/A	0	#N/A
#N/A	0	#N/A	0	#N/A	0	#N/A
#N/A	0	#N/A	0	#N/A	0	#N/A
#N/A	0	#N/A	0	#N/A	0	#N/A
#N/A	0	#N/A	0	#N/A	0	#N/A
	0		0	Ew/a-LOST-ar	1	Ew/p-LOST-ar
#N/A	0	#N/A	0	#N/A	0	#N/A
#N/A	0	#N/A	0	#N/A	0	#N/A
#N/A	0	#N/A	0	#N/A	0	#N/A
#N/A	0	#N/A	0	#N/A	0	#N/A
#N/A	0	#N/A	0	#N/A	0	#N/A
#N/A	0	#N/A	0	#N/A	0	#N/A
#N/A	0	#N/A	0	#N/A	0	#N/A
#N/A	0	#N/A	0	#N/A	0	#N/A
#N/A	0	#N/A	0	#N/A	0	#N/A
LOST-6_art	1	LOST>6_art	1		0	
#N/A	0	#N/A	0	#N/A	0	#N/A
#N/A	0	#N/A	0	#N/A	0	#N/A
#N/A	0	#N/A	0	#N/A	0	#N/A
#N/A	0	#N/A	0	#N/A	0	#N/A
#N/A	0	#N/A	0	#N/A	0	#N/A
#N/A	0	#N/A	0	#N/A	0	#N/A
#N/A	0	#N/A	0	#N/A	0	#N/A

	0	LOST>6_art	1		0	
	0		0		0	
LOST-6_art	1	LOST>6_art	1	Ew/a-LOST-ar	1	Ew/p-LOST-ar
LOST-6_art	1	LOST>6_art	1	Ew/a-LOST-ar	1	Ew/p-LOST-ar
#N/A	0	#N/A	0	#N/A	0	#N/A
#N/A	0	#N/A	0	#N/A	0	#N/A
	0		0	Ew/a-LOST-ar	1	Ew/p-LOST-ar
	0		0	Ew/a-LOST-ar	1	Ew/p-LOST-ar
	0		0	Ew/a-LOST-ar	1	Ew/p-LOST-ar
	0		0	Ew/a-LOST-ar	1	Ew/p-LOST-ar
#N/A	0	#N/A	0	#N/A	0	#N/A
#N/A	0	#N/A	0	#N/A	0	#N/A
#N/A	0	#N/A	0	#N/A	0	#N/A
#N/A	0	#N/A	0	#N/A	0	#N/A
#N/A	0	#N/A	0	#N/A	0	#N/A
#N/A	0	#N/A	0	#N/A	0	#N/A
#N/A	0	#N/A	0	#N/A	0	#N/A
LOST-6_art	1	LOST>6_art	1		0	Ew/p-LOST-ar
LOST-6_art	1	LOST>6_art	1		0	Ew/p-LOST-ar
#N/A	0	#N/A	0	#N/A	0	#N/A
#N/A	0	#N/A	0	#N/A	0	#N/A
#N/A	0	#N/A	0	#N/A	0	#N/A
#N/A	0	#N/A	0	#N/A	0	#N/A
#N/A	0	#N/A	0	#N/A	0	#N/A
#N/A	0	#N/A	0	#N/A	0	#N/A
#N/A	0	#N/A	0	#N/A	0	#N/A
#N/A	0	#N/A	0	#N/A	0	#N/A
#N/A	0	#N/A	0	#N/A	0	#N/A
#N/A	0	#N/A	0	#N/A	0	#N/A
#N/A	0	#N/A	0	#N/A	0	#N/A
#N/A	0	#N/A	0	#N/A	0	#N/A
#N/A	0	#N/A	0	#N/A	0	#N/A
#N/A	0	#N/A	0	#N/A	0	#N/A
#N/A	0	#N/A	0	#N/A	0	#N/A
#N/A	0	#N/A	0	#N/A	0	#N/A
#N/A	0	#N/A	0	#N/A	0	#N/A
#N/A	0	#N/A	0	#N/A	0	#N/A
#N/A	0	#N/A	0	#N/A	0	#N/A
#N/A	0	#N/A	0	#N/A	0	#N/A
#N/A	0	#N/A	0	#N/A	0	#N/A
#N/A	0	#N/A	0	#N/A	0	#N/A
#N/A	0	#N/A	0	#N/A	0	#N/A
#N/A	0	#N/A	0	#N/A	0	#N/A
#N/A	0	#N/A	0	#N/A	0	#N/A
#N/A	0	#N/A	0	#N/A	0	#N/A
#N/A	0	#N/A	0	#N/A	0	#N/A

#N/A	0	#N/A	0	#N/A	0	#N/A
#N/A	0	#N/A	0	#N/A	0	#N/A
LOST-6_art	1	LOST>6_art	1	Ew/a-LOST-ar	1	
LOST-6_art	1	LOST>6_art	1	Ew/a-LOST-ar	1	
#N/A	0	#N/A	0	#N/A	0	#N/A
#N/A	0	#N/A	0	#N/A	0	#N/A
#N/A	0	#N/A	0	#N/A	0	#N/A
#N/A	0	#N/A	0	#N/A	0	#N/A
LOST-6_art	1		0		0	
LOST-6_art	1		0		0	
	0		0	Ew/a-LOST-ar	1	Ew/p-LOST-ar
	0		0	Ew/a-LOST-ar	1	Ew/p-LOST-ar
#N/A	0	#N/A	0	#N/A	0	#N/A
#N/A	0	#N/A	0	#N/A	0	#N/A
#N/A	0	#N/A	0	#N/A	0	#N/A
#N/A	0	#N/A	0	#N/A	0	#N/A
LOST-6_art	1		0		0	
LOST-6_art	1		0		0	
LOST-6_art	1	LOST>6_art	1		0	Ew/p-LOST-ar
#N/A	0	#N/A	0	#N/A	0	#N/A
	0		0		0	
	0		0		0	
#N/A	0	#N/A	0	#N/A	0	#N/A
#N/A	0	#N/A	0	#N/A	0	#N/A
#N/A	0	#N/A	0	#N/A	0	#N/A
#N/A	0	#N/A	0	#N/A	0	#N/A
#N/A	0	#N/A	0	#N/A	0	#N/A
#N/A	0	#N/A	0	#N/A	0	#N/A
#N/A	0	#N/A	0	#N/A	0	#N/A
#N/A	0	#N/A	0	#N/A	0	#N/A
	0	LOST>6_art	1		0	
	0	LOST>6_art	1		0	
#N/A	0	#N/A	0	#N/A	0	#N/A
	0		0	Ew/a-LOST-ar	1	Ew/p-LOST-ar
	0		0	Ew/a-LOST-ar	1	Ew/p-LOST-ar
	0		0	Ew/a-LOST-ar	1	Ew/p-LOST-ar
	0		0	Ew/a-LOST-ar	1	Ew/p-LOST-ar
	0		0		0	
	0		0		0	
#N/A	0	#N/A	0	#N/A	0	#N/A
#N/A	0	#N/A	0	#N/A	0	#N/A
	0		0		0	
LOST-6_art	1		0		0	
LOST-6_art	1		0		0	
#N/A	0	#N/A	0	#N/A	0	#N/A
#N/A	0	#N/A	0	#N/A	0	#N/A
#N/A	0	#N/A	0	#N/A	0	#N/A

#N/A	0	#N/A	0	#N/A	0	#N/A
#N/A	0	#N/A	0	#N/A	0	#N/A
	0		0	Ew/a-LOST-ar	1	Ew/p-LOST-ar
	0		0	Ew/a-LOST-ar	1	Ew/p-LOST-ar
#N/A	0	#N/A	0	#N/A	0	#N/A
	0		0	Ew/a-LOST-ar	1	Ew/p-LOST-ar
	0		0	Ew/a-LOST-ar	1	Ew/p-LOST-ar
#N/A	0	#N/A	0	#N/A	0	#N/A
	0		0	Ew/a-LOST-ar	1	Ew/p-LOST-ar
#N/A	0	#N/A	0	#N/A	0	#N/A
	0		0		0	
	0		0		0	
	0		0		0	
	0	LOST>6_art	1		0	
	0	LOST>6_art	1		0	
#N/A	0	#N/A	0	#N/A	0	#N/A
#N/A	0	#N/A	0	#N/A	0	#N/A
#N/A	0	#N/A	0	#N/A	0	#N/A
#N/A	0	#N/A	0	#N/A	0	#N/A
#N/A	0	#N/A	0	#N/A	0	#N/A
#N/A	0	#N/A	0	#N/A	0	#N/A
	0		0	Ew/a-LOST-ar	1	Ew/p-LOST-ar
	0		0	Ew/a-LOST-ar	1	Ew/p-LOST-ar
LOST-6_art	1	LOST>6_art	1		0	
#N/A	0	#N/A	0	#N/A	0	#N/A
#N/A	0	#N/A	0	#N/A	0	#N/A
#N/A	0	#N/A	0	#N/A	0	#N/A
	0		0		0	
#N/A	0	#N/A	0	#N/A	0	#N/A
LOST-6_art	1	LOST>6_art	1		0	Ew/p-LOST-ar
LOST-6_art	1	LOST>6_art	1		0	Ew/p-LOST-ar
#N/A	0	#N/A	0	#N/A	0	#N/A
#N/A	0	#N/A	0	#N/A	0	#N/A
#N/A	0	#N/A	0	#N/A	0	#N/A
#N/A	0	#N/A	0	#N/A	0	#N/A
LOST-6_art	1		0		0	
#N/A	0	#N/A	0	#N/A	0	#N/A
LOST-6_art	1		0		0	
#N/A	0	#N/A	0	#N/A	0	#N/A
#N/A	0	#N/A	0	#N/A	0	#N/A
	0		0		0	
LOST-6_art	1		0		0	Ew/p-LOST-ar
#N/A	0	#N/A	0	#N/A	0	#N/A
#N/A	0	#N/A	0	#N/A	0	#N/A
#N/A	0	#N/A	0	#N/A	0	#N/A
#N/A	0	#N/A	0	#N/A	0	#N/A
#N/A	0	#N/A	0	#N/A	0	#N/A

	0	LOST>6_art	1		0	
	0	LOST>6_art	1		0	
	0	LOST>6_art	1		0	
#N/A	0	#N/A	0	#N/A	0	#N/A
LOST-6_art	1		0		0	
LOST-6_art	1		0		0	
LOST-6_art	1		0		0	
	0		0		0	
	0		0		0	
#N/A	0	#N/A	0	#N/A	0	#N/A
#N/A	0	#N/A	0	#N/A	0	#N/A
	0		0		0	
	0		0		0	
#N/A	0	#N/A	0	#N/A	0	#N/A
LOST-6_art	1	LOST>6_art	1		0	
#N/A	0	#N/A	0	#N/A	0	#N/A
#N/A	0	#N/A	0	#N/A	0	#N/A
#N/A	0	#N/A	0	#N/A	0	#N/A
#N/A	0	#N/A	0	#N/A	0	#N/A
#N/A	0	#N/A	0	#N/A	0	#N/A
#N/A	0	#N/A	0	#N/A	0	#N/A
#N/A	0	#N/A	0	#N/A	0	#N/A
#N/A	0	#N/A	0	#N/A	0	#N/A
#N/A	0	#N/A	0	#N/A	0	#N/A
#N/A	0	#N/A	0	#N/A	0	#N/A
#N/A	0	#N/A	0	#N/A	0	#N/A
#N/A	0	#N/A	0	#N/A	0	#N/A
#N/A	0	#N/A	0	#N/A	0	#N/A
#N/A	0	#N/A	0	#N/A	0	#N/A
#N/A	0	#N/A	0	#N/A	0	#N/A
#N/A	0	#N/A	0	#N/A	0	#N/A
#N/A	0	#N/A	0	#N/A	0	#N/A
#N/A	0	#N/A	0	#N/A	0	#N/A
#N/A	0	#N/A	0	#N/A	0	#N/A
#N/A	0	#N/A	0	#N/A	0	#N/A
#N/A	0	#N/A	0	#N/A	0	#N/A
#N/A	0	#N/A	0	#N/A	0	#N/A
LOST-6_art	1		0		0	
#N/A	0	#N/A	0	#N/A	0	#N/A
	0	LOST>6_art	1		0	
	0	LOST>6_art	1		0	
#N/A	0	#N/A	0	#N/A	0	#N/A
	0		0		0	
	0		0		0	
#N/A	0	#N/A	0	#N/A	0	#N/A
#N/A	0	#N/A	0	#N/A	0	#N/A
#N/A	0	#N/A	0	#N/A	0	#N/A

#N/A	0	#N/A	0	#N/A	0	#N/A
#N/A	0	#N/A	0	#N/A	0	#N/A
#N/A	0	#N/A	0	#N/A	0	#N/A
#N/A	0	#N/A	0	#N/A	0	#N/A
#N/A	0	#N/A	0	#N/A	0	#N/A
#N/A	0	#N/A	0	#N/A	0	#N/A
#N/A	0	#N/A	0	#N/A	0	#N/A
#N/A	0	#N/A	0	#N/A	0	#N/A
#N/A	0	#N/A	0	#N/A	0	#N/A
#N/A	0	#N/A	0	#N/A	0	#N/A
#N/A	0	#N/A	0	#N/A	0	#N/A
#N/A	0	#N/A	0	#N/A	0	#N/A
#N/A	0	#N/A	0	#N/A	0	#N/A
#N/A	0	#N/A	0	#N/A	0	#N/A
#N/A	0	#N/A	0	#N/A	0	#N/A
#N/A	0	#N/A	0	#N/A	0	#N/A
#N/A	0	#N/A	0	#N/A	0	#N/A
LOST-6_art	1		0	Ew/a-LOST-ar	1	
LOST-6_art	1		0	Ew/a-LOST-ar	1	
LOST-6_art	1		0	Ew/a-LOST-ar	1	
LOST-6_art	1		0	Ew/a-LOST-ar	1	
LOST-6_art	1		0	Ew/a-LOST-ar	1	
LOST-6_art	1		0	Ew/a-LOST-ar	1	
LOST-6_art	1		0	Ew/a-LOST-ar	1	
#N/A	0	#N/A	0	#N/A	0	#N/A
#N/A	0	#N/A	0	#N/A	0	#N/A
LOST-6_art	1		0	Ew/a-LOST-ar	1	Ew/p-LOST-ar
LOST-6_art	1		0	Ew/a-LOST-ar	1	Ew/p-LOST-ar
LOST-6_art	1		0	Ew/a-LOST-ar	1	Ew/p-LOST-ar
#N/A	0	#N/A	0	#N/A	0	#N/A
#N/A	0	#N/A	0	#N/A	0	#N/A
#N/A	0	#N/A	0	#N/A	0	#N/A
#N/A	0	#N/A	0	#N/A	0	#N/A
	0	LOST>6_art	1		0	
#N/A	0	#N/A	0	#N/A	0	#N/A
#N/A	0	#N/A	0	#N/A	0	#N/A
#N/A	0	#N/A	0	#N/A	0	#N/A
#N/A	0	#N/A	0	#N/A	0	#N/A
#N/A	0	#N/A	0	#N/A	0	#N/A
#N/A	0	#N/A	0	#N/A	0	#N/A
#N/A	0	#N/A	0	#N/A	0	#N/A
#N/A	0	#N/A	0	#N/A	0	#N/A
#N/A	0	#N/A	0	#N/A	0	#N/A
#N/A	0	#N/A	0	#N/A	0	#N/A
#N/A	0	#N/A	0	#N/A	0	#N/A
#N/A	0	#N/A	0	#N/A	0	#N/A
#N/A	0	#N/A	0	#N/A	0	#N/A

#N/A	0	#N/A	0	#N/A	0	#N/A
#N/A	0	#N/A	0	#N/A	0	#N/A
#N/A	0	#N/A	0	#N/A	0	#N/A
	0	LOST>6_art	1	Ew/a-LOST-ar	1	
#N/A	0	#N/A	0	#N/A	0	#N/A
#N/A	0	#N/A	0	#N/A	0	#N/A
#N/A	0	#N/A	0	#N/A	0	#N/A
#N/A	0	#N/A	0	#N/A	0	#N/A
#N/A	0	#N/A	0	#N/A	0	#N/A
#N/A	0	#N/A	0	#N/A	0	#N/A
#N/A	0	#N/A	0	#N/A	0	#N/A
	0	LOST>6_art	1	Ew/a-LOST-ar	1	Ew/p-LOST-ar
	0	LOST>6_art	1	Ew/a-LOST-ar	1	Ew/p-LOST-ar
#N/A	0	#N/A	0	#N/A	0	#N/A
#N/A	0	#N/A	0	#N/A	0	#N/A
#N/A	0	#N/A	0	#N/A	0	#N/A
#N/A	0	#N/A	0	#N/A	0	#N/A
#N/A	0	#N/A	0	#N/A	0	#N/A
#N/A	0	#N/A	0	#N/A	0	#N/A
#N/A	0	#N/A	0	#N/A	0	#N/A
#N/A	0	#N/A	0	#N/A	0	#N/A
#N/A	0	#N/A	0	#N/A	0	#N/A
#N/A	0	#N/A	0	#N/A	0	#N/A
#N/A	0	#N/A	0	#N/A	0	#N/A
LOST-6_art	1	LOST>6_art	1		0	
#N/A	0	#N/A	0	#N/A	0	#N/A
#N/A	0	#N/A	0	#N/A	0	#N/A
#N/A	0	#N/A	0	#N/A	0	#N/A
#N/A	0	#N/A	0	#N/A	0	#N/A
#N/A	0	#N/A	0	#N/A	0	#N/A
#N/A	0	#N/A	0	#N/A	0	#N/A
#N/A	0	#N/A	0	#N/A	0	#N/A
LOST-6_art	1		0		0	
LOST-6_art	1		0		0	
#N/A	0	#N/A	0	#N/A	0	#N/A
#N/A	0	#N/A	0	#N/A	0	#N/A
#N/A	0	#N/A	0	#N/A	0	#N/A
#N/A	0	#N/A	0	#N/A	0	#N/A
#N/A	0	#N/A	0	#N/A	0	#N/A
#N/A	0	#N/A	0	#N/A	0	#N/A
#N/A	0	#N/A	0	#N/A	0	#N/A
#N/A	0	#N/A	0	#N/A	0	#N/A
#N/A	0	#N/A	0	#N/A	0	#N/A
	0		0		0	
	0		0		0	
#N/A	0	#N/A	0	#N/A	0	#N/A
#N/A	0	#N/A	0	#N/A	0	#N/A

#N/A	0	#N/A	0	#N/A	0	#N/A
#N/A	0	#N/A	0	#N/A	0	#N/A
#N/A	0	#N/A	0	#N/A	0	#N/A
#N/A	0	#N/A	0	#N/A	0	#N/A
#N/A	0	#N/A	0	#N/A	0	#N/A
	0	LOST>6_art	1		0	
#N/A	0	#N/A	0	#N/A	0	#N/A
#N/A	0	#N/A	0	#N/A	0	#N/A
#N/A	0	#N/A	0	#N/A	0	#N/A
#N/A	0	#N/A	0	#N/A	0	#N/A
	0		0		0	
#N/A	0	#N/A	0	#N/A	0	#N/A
#N/A	0	#N/A	0	#N/A	0	#N/A
#N/A	0	#N/A	0	#N/A	0	#N/A
#N/A	0	#N/A	0	#N/A	0	#N/A
#N/A	0	#N/A	0	#N/A	0	#N/A
#N/A	0	#N/A	0	#N/A	0	#N/A
#N/A	0	#N/A	0	#N/A	0	#N/A
#N/A	0	#N/A	0	#N/A	0	#N/A
LOST-6_art	1		0	Ew/a-LOST-ar	1	Ew/p-LOST-ar
LOST-6_art	1	LOST>6_art	1	Ew/a-LOST-ar	1	
LOST-6_art	1	LOST>6_art	1	Ew/a-LOST-ar	1	
#N/A	0	#N/A	0	#N/A	0	#N/A
#N/A	0	#N/A	0	#N/A	0	#N/A
#N/A	0	#N/A	0	#N/A	0	#N/A
#N/A	0	#N/A	0	#N/A	0	#N/A
#N/A	0	#N/A	0	#N/A	0	#N/A
#N/A	0	#N/A	0	#N/A	0	#N/A
#N/A	0	#N/A	0	#N/A	0	#N/A
#N/A	0	#N/A	0	#N/A	0	#N/A
#N/A	0	#N/A	0	#N/A	0	#N/A
	0	LOST>6_art	1		0	
	0	LOST>6_art	1		0	
LOST-6_art	1		0		0	
#N/A	0	#N/A	0	#N/A	0	#N/A
#N/A	0	#N/A	0	#N/A	0	#N/A
#N/A	0	#N/A	0	#N/A	0	#N/A
#N/A	0	#N/A	0	#N/A	0	#N/A
	0		0	Ew/a-LOST-ar	1	Ew/p-LOST-ar
	0		0	Ew/a-LOST-ar	1	Ew/p-LOST-ar
	0	LOST>6_art	1		0	
#N/A	0	#N/A	0	#N/A	0	#N/A
#N/A	0	#N/A	0	#N/A	0	#N/A
#N/A	0	#N/A	0	#N/A	0	#N/A
#N/A	0	#N/A	0	#N/A	0	#N/A
#N/A	0	#N/A	0	#N/A	0	#N/A
#N/A	0	#N/A	0	#N/A	0	#N/A

#N/A	0	#N/A	0	#N/A	0	#N/A
LOST-6_art	1		0		0	
#N/A	0	#N/A	0	#N/A	0	#N/A
#N/A	0	#N/A	0	#N/A	0	#N/A
#N/A	0	#N/A	0	#N/A	0	#N/A
#N/A	0	#N/A	0	#N/A	0	#N/A
#N/A	0	#N/A	0	#N/A	0	#N/A
#N/A	0	#N/A	0	#N/A	0	#N/A
LOST-6_art	1	LOST>6_art	1	Ew/a-LOST-ar	1	
LOST-6_art	1	LOST>6_art	1	Ew/a-LOST-ar	1	
LOST-6_art	1	LOST>6_art	1	Ew/a-LOST-ar	1	
#N/A	0	#N/A	0	#N/A	0	#N/A
#N/A	0	#N/A	0	#N/A	0	#N/A
#N/A	0	#N/A	0	#N/A	0	#N/A
#N/A	0	#N/A	0	#N/A	0	#N/A
#N/A	0	#N/A	0	#N/A	0	#N/A
#N/A	0	#N/A	0	#N/A	0	#N/A
#N/A	0	#N/A	0	#N/A	0	#N/A
#N/A	0	#N/A	0	#N/A	0	#N/A
#N/A	0	#N/A	0	#N/A	0	#N/A
#N/A	0	#N/A	0	#N/A	0	#N/A
#N/A	0	#N/A	0	#N/A	0	#N/A
#N/A	0	#N/A	0	#N/A	0	#N/A
#N/A	0	#N/A	0	#N/A	0	#N/A
#N/A	0	#N/A	0	#N/A	0	#N/A
#N/A	0	#N/A	0	#N/A	0	#N/A
#N/A	0	#N/A	0	#N/A	0	#N/A
#N/A	0	#N/A	0	#N/A	0	#N/A
#N/A	0	#N/A	0	#N/A	0	#N/A
#N/A	0	#N/A	0	#N/A	0	#N/A
#N/A	0	#N/A	0	#N/A	0	#N/A
#N/A	0	#N/A	0	#N/A	0	#N/A
#N/A	0	#N/A	0	#N/A	0	#N/A
#N/A	0	#N/A	0	#N/A	0	#N/A
#N/A	0	#N/A	0	#N/A	0	#N/A
#N/A	0	#N/A	0	#N/A	0	#N/A
#N/A	0	#N/A	0	#N/A	0	#N/A
#N/A	0	#N/A	0	#N/A	0	#N/A
#N/A	0	#N/A	0	#N/A	0	#N/A
#N/A	0	#N/A	0	#N/A	0	#N/A
#N/A	0	#N/A	0	#N/A	0	#N/A
LOST-6_art	1		0		0	
LOST-6_art	1		0		0	
LOST-6_art	1		0	Ew/a-LOST-ar	1	
LOST-6_art	1		0	Ew/a-LOST-ar	1	
LOST-6_art	1		0	Ew/a-LOST-ar	1	
#N/A	0	#N/A	0	#N/A	0	#N/A

#N/A	0	#N/A	0	#N/A	0	#N/A
	0		0	Ew/a-LOST-ar	1	Ew/p-LOST-ar
	0		0	Ew/a-LOST-ar	1	Ew/p-LOST-ar
#N/A	0	#N/A	0	#N/A	0	#N/A
	0		0		0	
	0		0		0	
	0		0		0	
#N/A	0	#N/A	0	#N/A	0	#N/A
#N/A	0	#N/A	0	#N/A	0	#N/A
#N/A	0	#N/A	0	#N/A	0	#N/A
#N/A	0	#N/A	0	#N/A	0	#N/A
	0		0	Ew/a-LOST-ar	1	
	0		0	Ew/a-LOST-ar	1	
	0		0	Ew/a-LOST-ar	1	
#N/A	0	#N/A	0	#N/A	0	#N/A
#N/A	0	#N/A	0	#N/A	0	#N/A
#N/A	0	#N/A	0	#N/A	0	#N/A
#N/A	0	#N/A	0	#N/A	0	#N/A
#N/A	0	#N/A	0	#N/A	0	#N/A
#N/A	0	#N/A	0	#N/A	0	#N/A
#N/A	0	#N/A	0	#N/A	0	#N/A
#N/A	0	#N/A	0	#N/A	0	#N/A
#N/A	0	#N/A	0	#N/A	0	#N/A
#N/A	0	#N/A	0	#N/A	0	#N/A
#N/A	0	#N/A	0	#N/A	0	#N/A
#N/A	0	#N/A	0	#N/A	0	#N/A
#N/A	0	#N/A	0	#N/A	0	#N/A
#N/A	0	#N/A	0	#N/A	0	#N/A
LOST-6_art	1	LOST>6_art	1		0	
#N/A	0	#N/A	0	#N/A	0	#N/A
#N/A	0	#N/A	0	#N/A	0	#N/A
LOST-6_art	1	LOST>6_art	1		0	
LOST-6_art	1	LOST>6_art	1		0	
LOST-6_art	1	LOST>6_art	1		0	
#N/A	0	#N/A	0	#N/A	0	#N/A
#N/A	0	#N/A	0	#N/A	0	#N/A
#N/A	0	#N/A	0	#N/A	0	#N/A
#N/A	0	#N/A	0	#N/A	0	#N/A
#N/A	0	#N/A	0	#N/A	0	#N/A
#N/A	0	#N/A	0	#N/A	0	#N/A
#N/A	0	#N/A	0	#N/A	0	#N/A
#N/A	0	#N/A	0	#N/A	0	#N/A
#N/A	0	#N/A	0	#N/A	0	#N/A
#N/A	0	#N/A	0	#N/A	0	#N/A
#N/A	0	#N/A	0	#N/A	0	#N/A
#N/A	0	#N/A	0	#N/A	0	#N/A
#N/A	0	#N/A	0	#N/A	0	#N/A

LOST-6_art	1	LOST>6_art	1		0	
#N/A	0	#N/A	0	#N/A	0	#N/A
#N/A	0	#N/A	0	#N/A	0	#N/A
#N/A	0	#N/A	0	#N/A	0	#N/A
#N/A	0	#N/A	0	#N/A	0	#N/A
#N/A	0	#N/A	0	#N/A	0	#N/A
#N/A	0	#N/A	0	#N/A	0	#N/A
#N/A	0	#N/A	0	#N/A	0	#N/A
#N/A	0	#N/A	0	#N/A	0	#N/A
LOST-6_art	1	LOST>6_art	1		0	
#N/A	0	#N/A	0	#N/A	0	#N/A
#N/A	0	#N/A	0	#N/A	0	#N/A
#N/A	0	#N/A	0	#N/A	0	#N/A
#N/A	0	#N/A	0	#N/A	0	#N/A
#N/A	0	#N/A	0	#N/A	0	#N/A
	0		0		0	
	0		0		0	
#N/A	0	#N/A	0	#N/A	0	#N/A
#N/A	0	#N/A	0	#N/A	0	#N/A
#N/A	0	#N/A	0	#N/A	0	#N/A
#N/A	0	#N/A	0	#N/A	0	#N/A
	0		0		0	
	0		0		0	
	0		0		0	
LOST-6_art	1		0		0	
LOST-6_art	1		0	Ew/a-LOST-ar	1	
	0	LOST>6_art	1		0	
#N/A	0	#N/A	0	#N/A	0	#N/A
#N/A	0	#N/A	0	#N/A	0	#N/A
#N/A	0	#N/A	0	#N/A	0	#N/A
#N/A	0	#N/A	0	#N/A	0	#N/A
#N/A	0	#N/A	0	#N/A	0	#N/A
#N/A	0	#N/A	0	#N/A	0	#N/A
#N/A	0	#N/A	0	#N/A	0	#N/A
#N/A	0	#N/A	0	#N/A	0	#N/A
#N/A	0	#N/A	0	#N/A	0	#N/A
#N/A	0	#N/A	0	#N/A	0	#N/A
	0	LOST>6_art	1		0	
	0	LOST>6_art	1		0	
#N/A	0	#N/A	0	#N/A	0	#N/A
LOST-6_art	1		0		0	
#N/A	0	#N/A	0	#N/A	0	#N/A
#N/A	0	#N/A	0	#N/A	0	#N/A
#N/A	0	#N/A	0	#N/A	0	#N/A
#N/A	0	#N/A	0	#N/A	0	#N/A
#N/A	0	#N/A	0	#N/A	0	#N/A
#N/A	0	#N/A	0	#N/A	0	#N/A

LOST-6_art	1	LOST>6_art	1		0	
#N/A	0	#N/A	0	#N/A	0	#N/A
#N/A	0	#N/A	0	#N/A	0	#N/A
#N/A	0	#N/A	0	#N/A	0	#N/A
#N/A	0	#N/A	0	#N/A	0	#N/A
#N/A	0	#N/A	0	#N/A	0	#N/A
#N/A	0	#N/A	0	#N/A	0	#N/A
LOST-6_art	1	LOST>6_art	1		0	Ew/p-LOST-ar
LOST-6_art	1	LOST>6_art	1		0	Ew/p-LOST-ar
#N/A	0	#N/A	0	#N/A	0	#N/A
#N/A	0	#N/A	0	#N/A	0	#N/A
#N/A	0	#N/A	0	#N/A	0	#N/A
#N/A	0	#N/A	0	#N/A	0	#N/A
LOST-6_art	1	LOST>6_art	1		0	
#N/A	0	#N/A	0	#N/A	0	#N/A
	0	LOST>6_art	1		0	
#N/A	0	#N/A	0	#N/A	0	#N/A
#N/A	0	#N/A	0	#N/A	0	#N/A
#N/A	0	#N/A	0	#N/A	0	#N/A
#N/A	0	#N/A	0	#N/A	0	#N/A
#N/A	0	#N/A	0	#N/A	0	#N/A
#N/A	0	#N/A	0	#N/A	0	#N/A
	0		0	Ew/a-LOST-ar	1	Ew/p-LOST-ar
	0		0	Ew/a-LOST-ar	1	Ew/p-LOST-ar
	0		0	Ew/a-LOST-ar	1	Ew/p-LOST-ar
	0		0		0	
#N/A	0	#N/A	0	#N/A	0	#N/A
#N/A	0	#N/A	0	#N/A	0	#N/A
#N/A	0	#N/A	0	#N/A	0	#N/A
#N/A	0	#N/A	0	#N/A	0	#N/A
#N/A	0	#N/A	0	#N/A	0	#N/A
#N/A	0	#N/A	0	#N/A	0	#N/A
#N/A	0	#N/A	0	#N/A	0	#N/A
#N/A	0	#N/A	0	#N/A	0	#N/A
#N/A	0	#N/A	0	#N/A	0	#N/A
	0		0	Ew/a-LOST-ar	1	Ew/p-LOST-ar
#N/A	0	#N/A	0	#N/A	0	#N/A
#N/A	0	#N/A	0	#N/A	0	#N/A
#N/A	0	#N/A	0	#N/A	0	#N/A
	0		0	Ew/a-LOST-ar	1	
	0		0	Ew/a-LOST-ar	1	
#N/A	0	#N/A	0	#N/A	0	#N/A
#N/A	0	#N/A	0	#N/A	0	#N/A
#N/A	0	#N/A	0	#N/A	0	#N/A
#N/A	0	#N/A	0	#N/A	0	#N/A
LOST-6_art	1		0		0	
#N/A	0	#N/A	0	#N/A	0	#N/A

	0		0	Ew/a-LOST-ar	1	Ew/p-LOST-ar
	0		0	Ew/a-LOST-ar	1	Ew/p-LOST-ar
	0		0	Ew/a-LOST-ar	1	Ew/p-LOST-ar
#N/A	0	#N/A	0	#N/A	0	#N/A
#N/A	0	#N/A	0	#N/A	0	#N/A
	0		0		0	
#N/A	0	#N/A	0	#N/A	0	#N/A
#N/A	0	#N/A	0	#N/A	0	#N/A
#N/A	0	#N/A	0	#N/A	0	#N/A
	0		0		0	
	0		0		0	
	0		0		0	
#N/A	0	#N/A	0	#N/A	0	#N/A
#N/A	0	#N/A	0	#N/A	0	#N/A
#N/A	0	#N/A	0	#N/A	0	#N/A
#N/A	0	#N/A	0	#N/A	0	#N/A
#N/A	0	#N/A	0	#N/A	0	#N/A
#N/A	0	#N/A	0	#N/A	0	#N/A
#N/A	0	#N/A	0	#N/A	0	#N/A
#N/A	0	#N/A	0	#N/A	0	#N/A
#N/A	0	#N/A	0	#N/A	0	#N/A
#N/A	0	#N/A	0	#N/A	0	#N/A
#N/A	0	#N/A	0	#N/A	0	#N/A
#N/A	0	#N/A	0	#N/A	0	#N/A
#N/A	0	#N/A	0	#N/A	0	#N/A
#N/A	0	#N/A	0	#N/A	0	#N/A
#N/A	0	#N/A	0	#N/A	0	#N/A
#N/A	0	#N/A	0	#N/A	0	#N/A
#N/A	0	#N/A	0	#N/A	0	#N/A
#N/A	0	#N/A	0	#N/A	0	#N/A
#N/A	0	#N/A	0	#N/A	0	#N/A
#N/A	0	#N/A	0	#N/A	0	#N/A
LOST-6_art	1	LOST>6_art	1		0	
LOST-6_art	1	LOST>6_art	1		0	
#N/A	0	#N/A	0	#N/A	0	#N/A
LOST-6_art	1		0		0	
LOST-6_art	1		0		0	
#N/A	0	#N/A	0	#N/A	0	#N/A
#N/A	0	#N/A	0	#N/A	0	#N/A
	0	LOST>6_art	1		0	
	0	LOST>6_art	1		0	
#N/A	0	#N/A	0	#N/A	0	#N/A
#N/A	0	#N/A	0	#N/A	0	#N/A
#N/A	0	#N/A	0	#N/A	0	#N/A
#N/A	0	#N/A	0	#N/A	0	#N/A
#N/A	0	#N/A	0	#N/A	0	#N/A
	0	LOST>6_art	1		0	

	0	LOST>6_art	1		0	
#N/A	0	#N/A	0	#N/A	0	#N/A
#N/A	0	#N/A	0	#N/A	0	#N/A
#N/A	0	#N/A	0	#N/A	0	#N/A
#N/A	0	#N/A	0	#N/A	0	#N/A
#N/A	0	#N/A	0	#N/A	0	#N/A
#N/A	0	#N/A	0	#N/A	0	#N/A
#N/A	0	#N/A	0	#N/A	0	#N/A
#N/A	0	#N/A	0	#N/A	0	#N/A
#N/A	0	#N/A	0	#N/A	0	#N/A
#N/A	0	#N/A	0	#N/A	0	#N/A
	0		0		0	
#N/A	0	#N/A	0	#N/A	0	#N/A
#N/A	0	#N/A	0	#N/A	0	#N/A
#N/A	0	#N/A	0	#N/A	0	#N/A
LOST-6_art	1		0		0	
#N/A	0	#N/A	0	#N/A	0	#N/A
#N/A	0	#N/A	0	#N/A	0	#N/A
#N/A	0	#N/A	0	#N/A	0	#N/A
#N/A	0	#N/A	0	#N/A	0	#N/A
#N/A	0	#N/A	0	#N/A	0	#N/A
LOST-6_art	1		0		0	
#N/A	0	#N/A	0	#N/A	0	#N/A
#N/A	0	#N/A	0	#N/A	0	#N/A
#N/A	0	#N/A	0	#N/A	0	#N/A
#N/A	0	#N/A	0	#N/A	0	#N/A
#N/A	0	#N/A	0	#N/A	0	#N/A
#N/A	0	#N/A	0	#N/A	0	#N/A
#N/A	0	#N/A	0	#N/A	0	#N/A
#N/A	0	#N/A	0	#N/A	0	#N/A
#N/A	0	#N/A	0	#N/A	0	#N/A
#N/A	0	#N/A	0	#N/A	0	#N/A
	0		0	Ew/a-LOST-ar	1	Ew/p-LOST-ar
#N/A	0	#N/A	0	#N/A	0	#N/A
	0		0		0	
	0		0	Ew/a-LOST-ar	1	Ew/p-LOST-ar
	0		0	Ew/a-LOST-ar	1	Ew/p-LOST-ar
#N/A	0	#N/A	0	#N/A	0	#N/A
#N/A	0	#N/A	0	#N/A	0	#N/A
#N/A	0	#N/A	0	#N/A	0	#N/A
LOST-6_art	1	LOST>6_art	1		0	
LOST-6_art	1	LOST>6_art	1		0	
LOST-6_art	1	LOST>6_art	1		0	
LOST-6_art	1	LOST>6_art	1		0	
#N/A	0	#N/A	0	#N/A	0	#N/A
#N/A	0	#N/A	0	#N/A	0	#N/A
#N/A	0	#N/A	0	#N/A	0	#N/A

#N/A	0	#N/A	0	#N/A	0	#N/A
	0	LOST>6_art	1		0	
LOST-6_art	1	LOST>6_art	1		0	
#N/A	0	#N/A	0	#N/A	0	#N/A
#N/A	0	#N/A	0	#N/A	0	#N/A
LOST-6_art	1		0		0	
LOST-6_art	1		0		0	
LOST-6_art	1		0		0	
LOST-6_art	1		0		0	
#N/A	0	#N/A	0	#N/A	0	#N/A
#N/A	0	#N/A	0	#N/A	0	#N/A
#N/A	0	#N/A	0	#N/A	0	#N/A
#N/A	0	#N/A	0	#N/A	0	#N/A
#N/A	0	#N/A	0	#N/A	0	#N/A
#N/A	0	#N/A	0	#N/A	0	#N/A
#N/A	0	#N/A	0	#N/A	0	#N/A
#N/A	0	#N/A	0	#N/A	0	#N/A
#N/A	0	#N/A	0	#N/A	0	#N/A
	0		0	Ew/a-LOST-ar	1	
	0		0	Ew/a-LOST-ar	1	
#N/A	0	#N/A	0	#N/A	0	#N/A
#N/A	0	#N/A	0	#N/A	0	#N/A
#N/A	0	#N/A	0	#N/A	0	#N/A
#N/A	0	#N/A	0	#N/A	0	#N/A
LOST-6_art	1	LOST>6_art	1		0	
#N/A	0	#N/A	0	#N/A	0	#N/A
#N/A	0	#N/A	0	#N/A	0	#N/A
#N/A	0	#N/A	0	#N/A	0	#N/A
#N/A	0	#N/A	0	#N/A	0	#N/A
#N/A	0	#N/A	0	#N/A	0	#N/A
#N/A	0	#N/A	0	#N/A	0	#N/A
#N/A	0	#N/A	0	#N/A	0	#N/A
#N/A	0	#N/A	0	#N/A	0	#N/A
#N/A	0	#N/A	0	#N/A	0	#N/A
#N/A	0	#N/A	0	#N/A	0	#N/A
LOST-6_art	1		0	Ew/a-LOST-ar	1	Ew/p-LOST-ar
#N/A	0	#N/A	0	#N/A	0	#N/A
#N/A	0	#N/A	0	#N/A	0	#N/A
#N/A	0	#N/A	0	#N/A	0	#N/A
	0	LOST>6_art	1		0	
#N/A	0	#N/A	0	#N/A	0	#N/A
LOST-6_art	1		0		0	
#N/A	0	#N/A	0	#N/A	0	#N/A
#N/A	0	#N/A	0	#N/A	0	#N/A
#N/A	0	#N/A	0	#N/A	0	#N/A
#N/A	0	#N/A	0	#N/A	0	#N/A
#N/A	0	#N/A	0	#N/A	0	#N/A

#N/A	0	#N/A	0	#N/A	0	#N/A
#N/A	0	#N/A	0	#N/A	0	#N/A
#N/A	0	#N/A	0	#N/A	0	#N/A
LOST-6_art	1		0		0	
#N/A	0	#N/A	0	#N/A	0	#N/A
#N/A	0	#N/A	0	#N/A	0	#N/A
#N/A	0	#N/A	0	#N/A	0	#N/A
#N/A	0	#N/A	0	#N/A	0	#N/A
#N/A	0	#N/A	0	#N/A	0	#N/A
#N/A	0	#N/A	0	#N/A	0	#N/A
#N/A	0	#N/A	0	#N/A	0	#N/A
#N/A	0	#N/A	0	#N/A	0	#N/A
#N/A	0	#N/A	0	#N/A	0	#N/A
	0	LOST>6_art	1		0	
#N/A	0	#N/A	0	#N/A	0	#N/A
#N/A	0	#N/A	0	#N/A	0	#N/A
#N/A	0	#N/A	0	#N/A	0	#N/A
#N/A	0	#N/A	0	#N/A	0	#N/A
#N/A	0	#N/A	0	#N/A	0	#N/A
#N/A	0	#N/A	0	#N/A	0	#N/A
#N/A	0	#N/A	0	#N/A	0	#N/A
#N/A	0	#N/A	0	#N/A	0	#N/A
#N/A	0	#N/A	0	#N/A	0	#N/A
	0		0	Ew/a-LOST-ar	1	
#N/A	0	#N/A	0	#N/A	0	#N/A
#N/A	0	#N/A	0	#N/A	0	#N/A
LOST-6_art	1	LOST>6_art	1		0	
LOST-6_art	1		0		0	
LOST-6_art	1		0		0	
#N/A	0	#N/A	0	#N/A	0	#N/A
#N/A	0	#N/A	0	#N/A	0	#N/A
#N/A	0	#N/A	0	#N/A	0	#N/A
#N/A	0	#N/A	0	#N/A	0	#N/A
#N/A	0	#N/A	0	#N/A	0	#N/A
#N/A	0	#N/A	0	#N/A	0	#N/A
#N/A	0	#N/A	0	#N/A	0	#N/A
#N/A	0	#N/A	0	#N/A	0	#N/A
#N/A	0	#N/A	0	#N/A	0	#N/A
#N/A	0	#N/A	0	#N/A	0	#N/A
#N/A	0	#N/A	0	#N/A	0	#N/A
#N/A	0	#N/A	0	#N/A	0	#N/A
#N/A	0	#N/A	0	#N/A	0	#N/A
LOST-6_art	1	LOST>6_art	1		0	
	0		0	Ew/a-LOST-ar	1	Ew/p-LOST-ar
#N/A	0	#N/A	0	#N/A	0	#N/A
#N/A	0	#N/A	0	#N/A	0	#N/A
#N/A	0	#N/A	0	#N/A	0	#N/A

#N/A	0	#N/A	0	#N/A	0	#N/A
#N/A	0	#N/A	0	#N/A	0	#N/A
#N/A	0	#N/A	0	#N/A	0	#N/A
#N/A	0	#N/A	0	#N/A	0	#N/A
#N/A	0	#N/A	0	#N/A	0	#N/A
#N/A	0	#N/A	0	#N/A	0	#N/A
#N/A	0	#N/A	0	#N/A	0	#N/A
#N/A	0	#N/A	0	#N/A	0	#N/A
#N/A	0	#N/A	0	#N/A	0	#N/A
#N/A	0	#N/A	0	#N/A	0	#N/A
#N/A	0	#N/A	0	#N/A	0	#N/A
#N/A	0	#N/A	0	#N/A	0	#N/A
#N/A	0	#N/A	0	#N/A	0	#N/A
#N/A	0	#N/A	0	#N/A	0	#N/A
#N/A	0	#N/A	0	#N/A	0	#N/A
#N/A	0	#N/A	0	#N/A	0	#N/A
#N/A	0	#N/A	0	#N/A	0	#N/A
LOST-6_art	1	LOST>6_art	1		0	
#N/A	0	#N/A	0	#N/A	0	#N/A
#N/A	0	#N/A	0	#N/A	0	#N/A
#N/A	0	#N/A	0	#N/A	0	#N/A
#N/A	0	#N/A	0	#N/A	0	#N/A
#N/A	0	#N/A	0	#N/A	0	#N/A
LOST-6_art	1	LOST>6_art	1		0	
LOST-6_art	1	LOST>6_art	1		0	
#N/A	0	#N/A	0	#N/A	0	#N/A
#N/A	0	#N/A	0	#N/A	0	#N/A
#N/A	0	#N/A	0	#N/A	0	#N/A
LOST-6_art	1	LOST>6_art	1		0	
#N/A	0	#N/A	0	#N/A	0	#N/A
#N/A	0	#N/A	0	#N/A	0	#N/A
	0		0		0	
	0		0		0	
	0		0		0	
#N/A	0	#N/A	0	#N/A	0	#N/A
#N/A	0	#N/A	0	#N/A	0	#N/A
#N/A	0	#N/A	0	#N/A	0	#N/A
#N/A	0	#N/A	0	#N/A	0	#N/A
#N/A	0	#N/A	0	#N/A	0	#N/A
#N/A	0	#N/A	0	#N/A	0	#N/A
#N/A	0	#N/A	0	#N/A	0	#N/A
#N/A	0	#N/A	0	#N/A	0	#N/A
#N/A	0	#N/A	0	#N/A	0	#N/A
#N/A	0	#N/A	0	#N/A	0	#N/A
#N/A	0	#N/A	0	#N/A	0	#N/A
#N/A	0	#N/A	0	#N/A	0	#N/A
#N/A	0	#N/A	0	#N/A	0	#N/A

#N/A	0	#N/A	0	#N/A	0	#N/A
#N/A	0	#N/A	0	#N/A	0	#N/A
#N/A	0	#N/A	0	#N/A	0	#N/A
#N/A	0	#N/A	0	#N/A	0	#N/A
#N/A	0	#N/A	0	#N/A	0	#N/A
#N/A	0	#N/A	0	#N/A	0	#N/A
#N/A	0	#N/A	0	#N/A	0	#N/A
#N/A	0	#N/A	0	#N/A	0	#N/A
#N/A	0	#N/A	0	#N/A	0	#N/A
#N/A	0	#N/A	0	#N/A	0	#N/A
#N/A	0	#N/A	0	#N/A	0	#N/A
#N/A	0	#N/A	0	#N/A	0	#N/A
#N/A	0	#N/A	0	#N/A	0	#N/A
	0	LOST>6_art	1		0	
#N/A	0	#N/A	0	#N/A	0	#N/A
#N/A	0	#N/A	0	#N/A	0	#N/A
#N/A	0	#N/A	0	#N/A	0	#N/A
#N/A	0	#N/A	0	#N/A	0	#N/A
#N/A	0	#N/A	0	#N/A	0	#N/A
#N/A	0	#N/A	0	#N/A	0	#N/A
#N/A	0	#N/A	0	#N/A	0	#N/A
#N/A	0	#N/A	0	#N/A	0	#N/A
#N/A	0	#N/A	0	#N/A	0	#N/A
#N/A	0	#N/A	0	#N/A	0	#N/A
	0		0	Ew/a-LOST-ar	1	
	0		0	Ew/a-LOST-ar	1	
LOST-6_art	1		0	Ew/a-LOST-ar	1	Ew/p-LOST-ar
LOST-6_art	1		0	Ew/a-LOST-ar	1	Ew/p-LOST-ar
#N/A	0	#N/A	0	#N/A	0	#N/A
#N/A	0	#N/A	0	#N/A	0	#N/A
#N/A	0	#N/A	0	#N/A	0	#N/A
	0	LOST>6_art	1		0	
LOST-6_art	1	LOST>6_art	1		0	
#N/A	0	#N/A	0	#N/A	0	#N/A
#N/A	0	#N/A	0	#N/A	0	#N/A
	0		0		0	
#N/A	0	#N/A	0	#N/A	0	#N/A
#N/A	0	#N/A	0	#N/A	0	#N/A
	0		0		0	
	0		0		0	
	0		0		0	
	0		0		0	
#N/A	0	#N/A	0	#N/A	0	#N/A
	0		0		0	
	0		0		0	
#N/A	0	#N/A	0	#N/A	0	#N/A
#N/A	0	#N/A	0	#N/A	0	#N/A

#N/A	0	#N/A	0	#N/A	0	#N/A
	0	LOST>6_art	1		0	Ew/p-LOST-ar
	0	LOST>6_art	1		0	Ew/p-LOST-ar
	0	LOST>6_art	1		0	Ew/p-LOST-ar
#N/A	0	#N/A	0	#N/A	0	#N/A
#N/A	0	#N/A	0	#N/A	0	#N/A
#N/A	0	#N/A	0	#N/A	0	#N/A
#N/A	0	#N/A	0	#N/A	0	#N/A
#N/A	0	#N/A	0	#N/A	0	#N/A
	0		0		0	
#N/A	0	#N/A	0	#N/A	0	#N/A
#N/A	0	#N/A	0	#N/A	0	#N/A
#N/A	0	#N/A	0	#N/A	0	#N/A
#N/A	0	#N/A	0	#N/A	0	#N/A
#N/A	0	#N/A	0	#N/A	0	#N/A
#N/A	0	#N/A	0	#N/A	0	#N/A
#N/A	0	#N/A	0	#N/A	0	#N/A
LOST-6_art	1		0	Ew/a-LOST-ar	1	
LOST-6_art	1		0	Ew/a-LOST-ar	1	
#N/A	0	#N/A	0	#N/A	0	#N/A
#N/A	0	#N/A	0	#N/A	0	#N/A
#N/A	0	#N/A	0	#N/A	0	#N/A
#N/A	0	#N/A	0	#N/A	0	#N/A
#N/A	0	#N/A	0	#N/A	0	#N/A
#N/A	0	#N/A	0	#N/A	0	#N/A
#N/A	0	#N/A	0	#N/A	0	#N/A
#N/A	0	#N/A	0	#N/A	0	#N/A
#N/A	0	#N/A	0	#N/A	0	#N/A
#N/A	0	#N/A	0	#N/A	0	#N/A
LOST-6_art	1	LOST>6_art	1	Ew/a-LOST-ar	1	Ew/p-LOST-ar
LOST-6_art	1	LOST>6_art	1	Ew/a-LOST-ar	1	Ew/p-LOST-ar
#N/A	0	#N/A	0	#N/A	0	#N/A
#N/A	0	#N/A	0	#N/A	0	#N/A
#N/A	0	#N/A	0	#N/A	0	#N/A
#N/A	0	#N/A	0	#N/A	0	#N/A
	0		0	Ew/a-LOST-ar	1	
	0		0	Ew/a-LOST-ar	1	
#N/A	0	#N/A	0	#N/A	0	#N/A
#N/A	0	#N/A	0	#N/A	0	#N/A
LOST-6_art	1		0		0	
LOST-6_art	1		0		0	
LOST-6_art	1		0		0	
	0		0		0	
LOST-6_art	1	LOST>6_art	1		0	Ew/p-LOST-ar
LOST-6_art	1	LOST>6_art	1		0	Ew/p-LOST-ar
#N/A	0	#N/A	0	#N/A	0	#N/A
#N/A	0	#N/A	0	#N/A	0	#N/A

	0	LOST>6_art	1		0	
#N/A	0	#N/A	0	#N/A	0	#N/A
	0	LOST>6_art	1		0	
#N/A	0	#N/A	0	#N/A	0	#N/A
	0	LOST>6_art	1		0	
LOST-6_art	1		0	Ew/a-LOST-ar	1	Ew/p-LOST-ar
#N/A	0	#N/A	0	#N/A	0	#N/A
#N/A	0	#N/A	0	#N/A	0	#N/A
#N/A	0	#N/A	0	#N/A	0	#N/A
#N/A	0	#N/A	0	#N/A	0	#N/A
	0		0	Ew/a-LOST-ar	1	
	0		0	Ew/a-LOST-ar	1	
	0		0	Ew/a-LOST-ar	1	
	0		0	Ew/a-LOST-ar	1	
#N/A	0	#N/A	0	#N/A	0	#N/A
#N/A	0	#N/A	0	#N/A	0	#N/A
#N/A	0	#N/A	0	#N/A	0	#N/A
#N/A	0	#N/A	0	#N/A	0	#N/A
#N/A	0	#N/A	0	#N/A	0	#N/A
	0		0		0	
	0		0		0	
	0		0		0	
	0		0		0	
#N/A	0	#N/A	0	#N/A	0	#N/A
#N/A	0	#N/A	0	#N/A	0	#N/A
#N/A	0	#N/A	0	#N/A	0	#N/A
#N/A	0	#N/A	0	#N/A	0	#N/A
#N/A	0	#N/A	0	#N/A	0	#N/A
#N/A	0	#N/A	0	#N/A	0	#N/A
#N/A	0	#N/A	0	#N/A	0	#N/A
#N/A	0	#N/A	0	#N/A	0	#N/A
#N/A	0	#N/A	0	#N/A	0	#N/A
	0	LOST>6_art	1		0	
#N/A	0	#N/A	0	#N/A	0	#N/A
#N/A	0	#N/A	0	#N/A	0	#N/A
#N/A	0	#N/A	0	#N/A	0	#N/A
LOST-6_art	1	LOST>6_art	1	Ew/a-LOST-ar	1	Ew/p-LOST-ar
#N/A	0	#N/A	0	#N/A	0	#N/A
#N/A	0	#N/A	0	#N/A	0	#N/A
#N/A	0	#N/A	0	#N/A	0	#N/A
#N/A	0	#N/A	0	#N/A	0	#N/A
	0		0	Ew/a-LOST-ar	1	Ew/p-LOST-ar
	0		0	Ew/a-LOST-ar	1	Ew/p-LOST-ar
	0		0	Ew/a-LOST-ar	1	Ew/p-LOST-ar
LOST-6_art	1	LOST>6_art	1		0	
LOST-6_art	1	LOST>6_art	1		0	
#N/A	0	#N/A	0	#N/A	0	#N/A

#N/A	0	#N/A	0	#N/A	0	#N/A
LOST-6_art	1		0	Ew/a-LOST-ar	1	Ew/p-LOST-ar
#N/A	0	#N/A	0	#N/A	0	#N/A
#N/A	0	#N/A	0	#N/A	0	#N/A
#N/A	0	#N/A	0	#N/A	0	#N/A
#N/A	0	#N/A	0	#N/A	0	#N/A
#N/A	0	#N/A	0	#N/A	0	#N/A
LOST-6_art	1		0		0	
#N/A	0	#N/A	0	#N/A	0	#N/A
#N/A	0	#N/A	0	#N/A	0	#N/A
LOST-6_art	1		0	Ew/a-LOST-ar	1	Ew/p-LOST-ar
LOST-6_art	1		0	Ew/a-LOST-ar	1	Ew/p-LOST-ar
#N/A	0	#N/A	0	#N/A	0	#N/A
#N/A	0	#N/A	0	#N/A	0	#N/A
#N/A	0	#N/A	0	#N/A	0	#N/A
#N/A	0	#N/A	0	#N/A	0	#N/A
#N/A	0	#N/A	0	#N/A	0	#N/A
#N/A	0	#N/A	0	#N/A	0	#N/A
#N/A	0	#N/A	0	#N/A	0	#N/A
#N/A	0	#N/A	0	#N/A	0	#N/A
#N/A	0	#N/A	0	#N/A	0	#N/A
#N/A	0	#N/A	0	#N/A	0	#N/A
#N/A	0	#N/A	0	#N/A	0	#N/A
LOST-6_art	1		0		0	
LOST-6_art	1		0		0	
LOST-6_art	1		0		0	
#N/A	0	#N/A	0	#N/A	0	#N/A
#N/A	0	#N/A	0	#N/A	0	#N/A
#N/A	0	#N/A	0	#N/A	0	#N/A
LOST-6_art	1		0	Ew/a-LOST-ar	1	
LOST-6_art	1		0	Ew/a-LOST-ar	1	
	0	LOST>6_art	1		0	
	0	LOST>6_art	1		0	
#N/A	0	#N/A	0	#N/A	0	#N/A
#N/A	0	#N/A	0	#N/A	0	#N/A
#N/A	0	#N/A	0	#N/A	0	#N/A
#N/A	0	#N/A	0	#N/A	0	#N/A
#N/A	0	#N/A	0	#N/A	0	#N/A
LOST-6_art	1		0		0	
LOST-6_art	1		0		0	
#N/A	0	#N/A	0	#N/A	0	#N/A
#N/A	0	#N/A	0	#N/A	0	#N/A
	0	LOST>6_art	1		0	
#N/A	0	#N/A	0	#N/A	0	#N/A
#N/A	0	#N/A	0	#N/A	0	#N/A
#N/A	0	#N/A	0	#N/A	0	#N/A
#N/A	0	#N/A	0	#N/A	0	#N/A

#N/A	0	#N/A	0	#N/A	0	#N/A
#N/A	0	#N/A	0	#N/A	0	#N/A
	0	LOST>6_art	1		0	Ew/p-LOST-ar
LOST-6_art	1	LOST>6_art	1	Ew/a-LOST-ar	1	
LOST-6_art	1	LOST>6_art	1	Ew/a-LOST-ar	1	
LOST-6_art	1	LOST>6_art	1	Ew/a-LOST-ar	1	
#N/A	0	#N/A	0	#N/A	0	#N/A
#N/A	0	#N/A	0	#N/A	0	#N/A
#N/A	0	#N/A	0	#N/A	0	#N/A
#N/A	0	#N/A	0	#N/A	0	#N/A
#N/A	0	#N/A	0	#N/A	0	#N/A
#N/A	0	#N/A	0	#N/A	0	#N/A
#N/A	0	#N/A	0	#N/A	0	#N/A
#N/A	0	#N/A	0	#N/A	0	#N/A
#N/A	0	#N/A	0	#N/A	0	#N/A
#N/A	0	#N/A	0	#N/A	0	#N/A
#N/A	0	#N/A	0	#N/A	0	#N/A
#N/A	0	#N/A	0	#N/A	0	#N/A
#N/A	0	#N/A	0	#N/A	0	#N/A
LOST-6_art	1	LOST>6_art	1	Ew/a-LOST-ar	1	Ew/p-LOST-ar
#N/A	0	#N/A	0	#N/A	0	#N/A
#N/A	0	#N/A	0	#N/A	0	#N/A
#N/A	0	#N/A	0	#N/A	0	#N/A
#N/A	0	#N/A	0	#N/A	0	#N/A
#N/A	0	#N/A	0	#N/A	0	#N/A
#N/A	0	#N/A	0	#N/A	0	#N/A
	0	LOST>6_art	1	Ew/a-LOST-ar	1	
	0	LOST>6_art	1	Ew/a-LOST-ar	1	
#N/A	0	#N/A	0	#N/A	0	#N/A
#N/A	0	#N/A	0	#N/A	0	#N/A
#N/A	0	#N/A	0	#N/A	0	#N/A
#N/A	0	#N/A	0	#N/A	0	#N/A
#N/A	0	#N/A	0	#N/A	0	#N/A
#N/A	0	#N/A	0	#N/A	0	#N/A
#N/A	0	#N/A	0	#N/A	0	#N/A
#N/A	0	#N/A	0	#N/A	0	#N/A
#N/A	0	#N/A	0	#N/A	0	#N/A
#N/A	0	#N/A	0	#N/A	0	#N/A
#N/A	0	#N/A	0	#N/A	0	#N/A
#N/A	0	#N/A	0	#N/A	0	#N/A
#N/A	0	#N/A	0	#N/A	0	#N/A
#N/A	0	#N/A	0	#N/A	0	#N/A
#N/A	0	#N/A	0	#N/A	0	#N/A
#N/A	0	#N/A	0	#N/A	0	#N/A
#N/A	0	#N/A	0	#N/A	0	#N/A
#N/A	0	#N/A	0	#N/A	0	#N/A
LOST-6_art	1		0		0	

	0	LOST>6_art	1		0	
#N/A	0	#N/A	0	#N/A	0	#N/A
#N/A	0	#N/A	0	#N/A	0	#N/A
#N/A	0	#N/A	0	#N/A	0	#N/A
#N/A	0	#N/A	0	#N/A	0	#N/A
LOST-6_art	1	LOST>6_art	1	Ew/a-LOST-ar	1	Ew/p-LOST-ar
LOST-6_art	1	LOST>6_art	1	Ew/a-LOST-ar	1	Ew/p-LOST-ar
	0		0		0	
#N/A	0	#N/A	0	#N/A	0	#N/A
#N/A	0	#N/A	0	#N/A	0	#N/A
#N/A	0	#N/A	0	#N/A	0	#N/A
#N/A	0	#N/A	0	#N/A	0	#N/A
	0	LOST>6_art	1		0	
#N/A	0	#N/A	0	#N/A	0	#N/A
#N/A	0	#N/A	0	#N/A	0	#N/A
#N/A	0	#N/A	0	#N/A	0	#N/A
#N/A	0	#N/A	0	#N/A	0	#N/A
	0	LOST>6_art	1		0	
#N/A	0	#N/A	0	#N/A	0	#N/A
#N/A	0	#N/A	0	#N/A	0	#N/A
#N/A	0	#N/A	0	#N/A	0	#N/A
#N/A	0	#N/A	0	#N/A	0	#N/A
#N/A	0	#N/A	0	#N/A	0	#N/A
#N/A	0	#N/A	0	#N/A	0	#N/A
#N/A	0	#N/A	0	#N/A	0	#N/A
#N/A	0	#N/A	0	#N/A	0	#N/A
#N/A	0	#N/A	0	#N/A	0	#N/A
#N/A	0	#N/A	0	#N/A	0	#N/A
#N/A	0	#N/A	0	#N/A	0	#N/A
#N/A	0	#N/A	0	#N/A	0	#N/A
	0	LOST>6_art	1		0	Ew/p-LOST-ar
	0	LOST>6_art	1		0	Ew/p-LOST-ar
#N/A	0	#N/A	0	#N/A	0	#N/A
#N/A	0	#N/A	0	#N/A	0	#N/A
#N/A	0	#N/A	0	#N/A	0	#N/A
#N/A	0	#N/A	0	#N/A	0	#N/A
#N/A	0	#N/A	0	#N/A	0	#N/A
	0		0	Ew/a-LOST-ar	1	Ew/p-LOST-ar
	0		0	Ew/a-LOST-ar	1	Ew/p-LOST-ar
	0		0	Ew/a-LOST-ar	1	Ew/p-LOST-ar
#N/A	0	#N/A	0	#N/A	0	#N/A
#N/A	0	#N/A	0	#N/A	0	#N/A
#N/A	0	#N/A	0	#N/A	0	#N/A
#N/A	0	#N/A	0	#N/A	0	#N/A
#N/A	0	#N/A	0	#N/A	0	#N/A
#N/A	0	#N/A	0	#N/A	0	#N/A
#N/A	0	#N/A	0	#N/A	0	#N/A

#N/A	0	#N/A	0	#N/A	0	#N/A
#N/A	0	#N/A	0	#N/A	0	#N/A
#N/A	0	#N/A	0	#N/A	0	#N/A
#N/A	0	#N/A	0	#N/A	0	#N/A
#N/A	0	#N/A	0	#N/A	0	#N/A
#N/A	0	#N/A	0	#N/A	0	#N/A
#N/A	0	#N/A	0	#N/A	0	#N/A
	0	LOST>6_art	1		0	
#N/A	0	#N/A	0	#N/A	0	#N/A
#N/A	0	#N/A	0	#N/A	0	#N/A
#N/A	0	#N/A	0	#N/A	0	#N/A
#N/A	0	#N/A	0	#N/A	0	#N/A
LOST-6_art	1	LOST>6_art	1		0	
LOST-6_art	1	LOST>6_art	1		0	
LOST-6_art	1	LOST>6_art	1		0	
#N/A	0	#N/A	0	#N/A	0	#N/A
LOST-6_art	1		0	Ew/a-LOST-ar	1	
LOST-6_art	1		0	Ew/a-LOST-ar	1	
LOST-6_art	1		0	Ew/a-LOST-ar	1	
#N/A	0	#N/A	0	#N/A	0	#N/A
#N/A	0	#N/A	0	#N/A	0	#N/A
#N/A	0	#N/A	0	#N/A	0	#N/A
	0	LOST>6_art	1		0	
#N/A	0	#N/A	0	#N/A	0	#N/A
#N/A	0	#N/A	0	#N/A	0	#N/A
#N/A	0	#N/A	0	#N/A	0	#N/A
#N/A	0	#N/A	0	#N/A	0	#N/A
	0		0	Ew/a-LOST-ar	1	
	0		0	Ew/a-LOST-ar	1	
	0		0	Ew/a-LOST-ar	1	
#N/A	0	#N/A	0	#N/A	0	#N/A
#N/A	0	#N/A	0	#N/A	0	#N/A
#N/A	0	#N/A	0	#N/A	0	#N/A
	0	LOST>6_art	1		0	
	0	LOST>6_art	1		0	
	0	LOST>6_art	1		0	
	0	LOST>6_art	1		0	
#N/A	0	#N/A	0	#N/A	0	#N/A
LOST-6_art	1	LOST>6_art	1		0	
LOST-6_art	1	LOST>6_art	1		0	
#N/A	0	#N/A	0	#N/A	0	#N/A
#N/A	0	#N/A	0	#N/A	0	#N/A
#N/A	0	#N/A	0	#N/A	0	#N/A
#N/A	0	#N/A	0	#N/A	0	#N/A
#N/A	0	#N/A	0	#N/A	0	#N/A
#N/A	0	#N/A	0	#N/A	0	#N/A
#N/A	0	#N/A	0	#N/A	0	#N/A

#N/A	0	#N/A	0	#N/A	0	#N/A
#N/A	0	#N/A	0	#N/A	0	#N/A
#N/A	0	#N/A	0	#N/A	0	#N/A
	0		0	Ew/a-LOST-ar	1	Ew/p-LOST-ar
	0		0	Ew/a-LOST-ar	1	Ew/p-LOST-ar
	0		0	Ew/a-LOST-ar	1	Ew/p-LOST-ar
	0		0	Ew/a-LOST-ar	1	Ew/p-LOST-ar
#N/A	0	#N/A	0	#N/A	0	#N/A
#N/A	0	#N/A	0	#N/A	0	#N/A
#N/A	0	#N/A	0	#N/A	0	#N/A
#N/A	0	#N/A	0	#N/A	0	#N/A
#N/A	0	#N/A	0	#N/A	0	#N/A
#N/A	0	#N/A	0	#N/A	0	#N/A
#N/A	0	#N/A	0	#N/A	0	#N/A
#N/A	0	#N/A	0	#N/A	0	#N/A
#N/A	0	#N/A	0	#N/A	0	#N/A
#N/A	0	#N/A	0	#N/A	0	#N/A
	0		0		0	
	0		0		0	
	0		0		0	
#N/A	0	#N/A	0	#N/A	0	#N/A
#N/A	0	#N/A	0	#N/A	0	#N/A
#N/A	0	#N/A	0	#N/A	0	#N/A
#N/A	0	#N/A	0	#N/A	0	#N/A
#N/A	0	#N/A	0	#N/A	0	#N/A
#N/A	0	#N/A	0	#N/A	0	#N/A
#N/A	0	#N/A	0	#N/A	0	#N/A
LOST-6_art	1	LOST>6_art	1		0	
LOST-6_art	1		0	Ew/a-LOST-ar	1	
#N/A	0	#N/A	0	#N/A	0	#N/A
#N/A	0	#N/A	0	#N/A	0	#N/A
#N/A	0	#N/A	0	#N/A	0	#N/A
#N/A	0	#N/A	0	#N/A	0	#N/A
#N/A	0	#N/A	0	#N/A	0	#N/A
#N/A	0	#N/A	0	#N/A	0	#N/A
#N/A	0	#N/A	0	#N/A	0	#N/A
	0	LOST>6_art	1		0	Ew/p-LOST-ar
#N/A	0	#N/A	0	#N/A	0	#N/A
#N/A	0	#N/A	0	#N/A	0	#N/A
#N/A	0	#N/A	0	#N/A	0	#N/A
#N/A	0	#N/A	0	#N/A	0	#N/A
#N/A	0	#N/A	0	#N/A	0	#N/A
#N/A	0	#N/A	0	#N/A	0	#N/A
#N/A	0	#N/A	0	#N/A	0	#N/A
	0	LOST>6_art	1		0	
LOST-6_art	1	LOST>6_art	1	Ew/a-LOST-ar	1	
LOST-6_art	1	LOST>6_art	1	Ew/a-LOST-ar	1	

LOST-6_art	1	LOST>6_art	1	Ew/a-LOST-ar	1	
#N/A	0	#N/A	0	#N/A	0	#N/A
#N/A	0	#N/A	0	#N/A	0	#N/A
#N/A	0	#N/A	0	#N/A	0	#N/A
#N/A	0	#N/A	0	#N/A	0	#N/A
#N/A	0	#N/A	0	#N/A	0	#N/A
#N/A	0	#N/A	0	#N/A	0	#N/A
#N/A	0	#N/A	0	#N/A	0	#N/A
LOST-6_art	1		0		0	
#N/A	0	#N/A	0	#N/A	0	#N/A
#N/A	0	#N/A	0	#N/A	0	#N/A
	0		0		0	
#N/A	0	#N/A	0	#N/A	0	#N/A
#N/A	0	#N/A	0	#N/A	0	#N/A
#N/A	0	#N/A	0	#N/A	0	#N/A
#N/A	0	#N/A	0	#N/A	0	#N/A
#N/A	0	#N/A	0	#N/A	0	#N/A
#N/A	0	#N/A	0	#N/A	0	#N/A
#N/A	0	#N/A	0	#N/A	0	#N/A
#N/A	0	#N/A	0	#N/A	0	#N/A
#N/A	0	#N/A	0	#N/A	0	#N/A
LOST-6_art	1		0		0	Ew/p-LOST-ar
LOST-6_art	1	LOST>6_art	1		0	Ew/p-LOST-ar
#N/A	0	#N/A	0	#N/A	0	#N/A
#N/A	0	#N/A	0	#N/A	0	#N/A
#N/A	0	#N/A	0	#N/A	0	#N/A
#N/A	0	#N/A	0	#N/A	0	#N/A
#N/A	0	#N/A	0	#N/A	0	#N/A
#N/A	0	#N/A	0	#N/A	0	#N/A
#N/A	0	#N/A	0	#N/A	0	#N/A
LOST-6_art	1		0		0	
LOST-6_art	1		0		0	
#N/A	0	#N/A	0	#N/A	0	#N/A
#N/A	0	#N/A	0	#N/A	0	#N/A
#N/A	0	#N/A	0	#N/A	0	#N/A
#N/A	0	#N/A	0	#N/A	0	#N/A
#N/A	0	#N/A	0	#N/A	0	#N/A
#N/A	0	#N/A	0	#N/A	0	#N/A
#N/A	0	#N/A	0	#N/A	0	#N/A
#N/A	0	#N/A	0	#N/A	0	#N/A
	0	LOST>6_art	1		0	
#N/A	0	#N/A	0	#N/A	0	#N/A
	0	LOST>6_art	1		0	
	0	LOST>6_art	1		0	
#N/A	0	#N/A	0	#N/A	0	#N/A
#N/A	0	#N/A	0	#N/A	0	#N/A
	0	LOST>6_art	1	Ew/a-LOST-ar	1	

#N/A	0	#N/A	0	#N/A	0	#N/A
#N/A	0	#N/A	0	#N/A	0	#N/A
#N/A	0	#N/A	0	#N/A	0	#N/A
#N/A	0	#N/A	0	#N/A	0	#N/A
#N/A	0	#N/A	0	#N/A	0	#N/A
#N/A	0	#N/A	0	#N/A	0	#N/A
#N/A	0	#N/A	0	#N/A	0	#N/A
#N/A	0	#N/A	0	#N/A	0	#N/A
#N/A	0	#N/A	0	#N/A	0	#N/A
#N/A	0	#N/A	0	#N/A	0	#N/A
#N/A	0	#N/A	0	#N/A	0	#N/A
#N/A	0	#N/A	0	#N/A	0	#N/A
#N/A	0	#N/A	0	#N/A	0	#N/A
	0	LOST>6_art	1		0	
#N/A	0	#N/A	0	#N/A	0	#N/A
#N/A	0	#N/A	0	#N/A	0	#N/A
#N/A	0	#N/A	0	#N/A	0	#N/A
#N/A	0	#N/A	0	#N/A	0	#N/A
#N/A	0	#N/A	0	#N/A	0	#N/A
#N/A	0	#N/A	0	#N/A	0	#N/A
#N/A	0	#N/A	0	#N/A	0	#N/A
#N/A	0	#N/A	0	#N/A	0	#N/A
	0	LOST>6_art	1		0	Ew/p-LOST-ar
#N/A	0	#N/A	0	#N/A	0	#N/A
#N/A	0	#N/A	0	#N/A	0	#N/A
#N/A	0	#N/A	0	#N/A	0	#N/A
#N/A	0	#N/A	0	#N/A	0	#N/A
#N/A	0	#N/A	0	#N/A	0	#N/A
#N/A	0	#N/A	0	#N/A	0	#N/A
#N/A	0	#N/A	0	#N/A	0	#N/A
#N/A	0	#N/A	0	#N/A	0	#N/A
#N/A	0	#N/A	0	#N/A	0	#N/A
#N/A	0	#N/A	0	#N/A	0	#N/A
#N/A	0	#N/A	0	#N/A	0	#N/A
#N/A	0	#N/A	0	#N/A	0	#N/A
#N/A	0	#N/A	0	#N/A	0	#N/A
#N/A	0	#N/A	0	#N/A	0	#N/A
#N/A	0	#N/A	0	#N/A	0	#N/A
#N/A	0	#N/A	0	#N/A	0	#N/A
#N/A	0	#N/A	0	#N/A	0	#N/A
#N/A	0	#N/A	0	#N/A	0	#N/A
#N/A	0	#N/A	0	#N/A	0	#N/A
LOST-6_art	1	LOST>6_art	1		0	Ew/p-LOST-ar
#N/A	0	#N/A	0	#N/A	0	#N/A
#N/A	0	#N/A	0	#N/A	0	#N/A
#N/A	0	#N/A	0	#N/A	0	#N/A
#N/A	0	#N/A	0	#N/A	0	#N/A

#N/A	0	#N/A	0	#N/A	0	#N/A
	0		0		0	
	0		0		0	
	0		0		0	
#N/A	0	#N/A	0	#N/A	0	#N/A
	0		0	Ew/a-LOST-ar	1	Ew/p-LOST-ar
	0		0	Ew/a-LOST-ar	1	Ew/p-LOST-ar
	0		0	Ew/a-LOST-ar	1	Ew/p-LOST-ar
#N/A	0	#N/A	0	#N/A	0	#N/A
#N/A	0	#N/A	0	#N/A	0	#N/A
#N/A	0	#N/A	0	#N/A	0	#N/A
#N/A	0	#N/A	0	#N/A	0	#N/A
#N/A	0	#N/A	0	#N/A	0	#N/A
#N/A	0	#N/A	0	#N/A	0	#N/A
#N/A	0	#N/A	0	#N/A	0	#N/A
#N/A	0	#N/A	0	#N/A	0	#N/A
#N/A	0	#N/A	0	#N/A	0	#N/A
#N/A	0	#N/A	0	#N/A	0	#N/A
#N/A	0	#N/A	0	#N/A	0	#N/A
LOST-6_art	1	LOST>6_art	1		0	
LOST-6_art	1	LOST>6_art	1		0	
#N/A	0	#N/A	0	#N/A	0	#N/A
#N/A	0	#N/A	0	#N/A	0	#N/A
#N/A	0	#N/A	0	#N/A	0	#N/A
LOST-6_art	1		0		0	
#N/A	0	#N/A	0	#N/A	0	#N/A
LOST-6_art	1		0		0	
LOST-6_art	1		0		0	
#N/A	0	#N/A	0	#N/A	0	#N/A
#N/A	0	#N/A	0	#N/A	0	#N/A
#N/A	0	#N/A	0	#N/A	0	#N/A
#N/A	0	#N/A	0	#N/A	0	#N/A
#N/A	0	#N/A	0	#N/A	0	#N/A
#N/A	0	#N/A	0	#N/A	0	#N/A
#N/A	0	#N/A	0	#N/A	0	#N/A
#N/A	0	#N/A	0	#N/A	0	#N/A
#N/A	0	#N/A	0	#N/A	0	#N/A
#N/A	0	#N/A	0	#N/A	0	#N/A
#N/A	0	#N/A	0	#N/A	0	#N/A
#N/A	0	#N/A	0	#N/A	0	#N/A
#N/A	0	#N/A	0	#N/A	0	#N/A
#N/A	0	#N/A	0	#N/A	0	#N/A
LOST-6_art	1		0	Ew/a-LOST-ar	1	Ew/p-LOST-ar
LOST-6_art	1		0	Ew/a-LOST-ar	1	Ew/p-LOST-ar
LOST-6_art	1		0	Ew/a-LOST-ar	1	Ew/p-LOST-ar
LOST-6_art	1	LOST>6_art	1	Ew/a-LOST-ar	1	Ew/p-LOST-ar
LOST-6_art	1	LOST>6_art	1	Ew/a-LOST-ar	1	Ew/p-LOST-ar

LOST-6_art	1	LOST>6_art	1	Ew/a-LOST-ar	1	Ew/p-LOST-ar
LOST-6_art	1	LOST>6_art	1	Ew/a-LOST-ar	1	Ew/p-LOST-ar
LOST-6_art	1	LOST>6_art	1	Ew/a-LOST-ar	1	Ew/p-LOST-ar
#N/A	0	#N/A	0	#N/A	0	#N/A
#N/A	0	#N/A	0	#N/A	0	#N/A
#N/A	0	#N/A	0	#N/A	0	#N/A
#N/A	0	#N/A	0	#N/A	0	#N/A
#N/A	0	#N/A	0	#N/A	0	#N/A
#N/A	0	#N/A	0	#N/A	0	#N/A
	0		0		0	
	0		0		0	
#N/A	0	#N/A	0	#N/A	0	#N/A
#N/A	0	#N/A	0	#N/A	0	#N/A
#N/A	0	#N/A	0	#N/A	0	#N/A
#N/A	0	#N/A	0	#N/A	0	#N/A
#N/A	0	#N/A	0	#N/A	0	#N/A
#N/A	0	#N/A	0	#N/A	0	#N/A
#N/A	0	#N/A	0	#N/A	0	#N/A
#N/A	0	#N/A	0	#N/A	0	#N/A
#N/A	0	#N/A	0	#N/A	0	#N/A
	0		0		0	
	0		0		0	
#N/A	0	#N/A	0	#N/A	0	#N/A
#N/A	0	#N/A	0	#N/A	0	#N/A
#N/A	0	#N/A	0	#N/A	0	#N/A
#N/A	0	#N/A	0	#N/A	0	#N/A
#N/A	0	#N/A	0	#N/A	0	#N/A
#N/A	0	#N/A	0	#N/A	0	#N/A
#N/A	0	#N/A	0	#N/A	0	#N/A
#N/A	0	#N/A	0	#N/A	0	#N/A
#N/A	0	#N/A	0	#N/A	0	#N/A
#N/A	0	#N/A	0	#N/A	0	#N/A
#N/A	0	#N/A	0	#N/A	0	#N/A
	0		0	Ew/a-LOST-ar	1	Ew/p-LOST-ar
	0		0	Ew/a-LOST-ar	1	Ew/p-LOST-ar
#N/A	0	#N/A	0	#N/A	0	#N/A
#N/A	0	#N/A	0	#N/A	0	#N/A
#N/A	0	#N/A	0	#N/A	0	#N/A
#N/A	0	#N/A	0	#N/A	0	#N/A
#N/A	0	#N/A	0	#N/A	0	#N/A
#N/A	0	#N/A	0	#N/A	0	#N/A
#N/A	0	#N/A	0	#N/A	0	#N/A
#N/A	0	#N/A	0	#N/A	0	#N/A
#N/A	0	#N/A	0	#N/A	0	#N/A
#N/A	0	#N/A	0	#N/A	0	#N/A
	0		0		0	
#N/A	0	#N/A	0	#N/A	0	#N/A

#N/A	0	#N/A	0	#N/A	0	#N/A
	0		0		0	
	0		0	Ew/a-LOST-ar	1	Ew/p-LOST-ar
	0		0	Ew/a-LOST-ar	1	Ew/p-LOST-ar
	0		0	Ew/a-LOST-ar	1	Ew/p-LOST-ar
	0		0	Ew/a-LOST-ar	1	Ew/p-LOST-ar
LOST-6_art	1	LOST>6_art	1	Ew/a-LOST-ar	1	Ew/p-LOST-ar
	0		0		0	
	0		0		0	
	0		0		0	
#N/A	0	#N/A	0	#N/A	0	#N/A
#N/A	0	#N/A	0	#N/A	0	#N/A
#N/A	0	#N/A	0	#N/A	0	#N/A
#N/A	0	#N/A	0	#N/A	0	#N/A
#N/A	0	#N/A	0	#N/A	0	#N/A
#N/A	0	#N/A	0	#N/A	0	#N/A
#N/A	0	#N/A	0	#N/A	0	#N/A
#N/A	0	#N/A	0	#N/A	0	#N/A
#N/A	0	#N/A	0	#N/A	0	#N/A
#N/A	0	#N/A	0	#N/A	0	#N/A
	0		0	Ew/a-LOST-ar	1	
	0		0	Ew/a-LOST-ar	1	
#N/A	0	#N/A	0	#N/A	0	#N/A
#N/A	0	#N/A	0	#N/A	0	#N/A
#N/A	0	#N/A	0	#N/A	0	#N/A
#N/A	0	#N/A	0	#N/A	0	#N/A
#N/A	0	#N/A	0	#N/A	0	#N/A
#N/A	0	#N/A	0	#N/A	0	#N/A
#N/A	0	#N/A	0	#N/A	0	#N/A
#N/A	0	#N/A	0	#N/A	0	#N/A
#N/A	0	#N/A	0	#N/A	0	#N/A
	0		0	Ew/a-LOST-ar	1	Ew/p-LOST-ar
LOST-6_art	1		0	Ew/a-LOST-ar	1	Ew/p-LOST-ar
LOST-6_art	1		0	Ew/a-LOST-ar	1	Ew/p-LOST-ar
LOST-6_art	1		0	Ew/a-LOST-ar	1	Ew/p-LOST-ar
#N/A	0	#N/A	0	#N/A	0	#N/A
	0	LOST>6_art	1		0	
#N/A	0	#N/A	0	#N/A	0	#N/A
#N/A	0	#N/A	0	#N/A	0	#N/A
#N/A	0	#N/A	0	#N/A	0	#N/A
#N/A	0	#N/A	0	#N/A	0	#N/A
#N/A	0	#N/A	0	#N/A	0	#N/A
#N/A	0	#N/A	0	#N/A	0	#N/A
#N/A	0	#N/A	0	#N/A	0	#N/A
#N/A	0	#N/A	0	#N/A	0	#N/A
#N/A	0	#N/A	0	#N/A	0	#N/A
#N/A	0	#N/A	0	#N/A	0	#N/A

#N/A	0	#N/A	0	#N/A	0	#N/A
#N/A	0	#N/A	0	#N/A	0	#N/A
#N/A	0	#N/A	0	#N/A	0	#N/A
#N/A	0	#N/A	0	#N/A	0	#N/A
#N/A	0	#N/A	0	#N/A	0	#N/A
LOST-6_art	1	LOST>6_art	1	Ew/a-LOST-ar	1	Ew/p-LOST-ar
LOST-6_art	1	LOST>6_art	1	Ew/a-LOST-ar	1	Ew/p-LOST-ar
LOST-6_art	1	LOST>6_art	1	Ew/a-LOST-ar	1	Ew/p-LOST-ar
#N/A	0	#N/A	0	#N/A	0	#N/A
#N/A	0	#N/A	0	#N/A	0	#N/A
#N/A	0	#N/A	0	#N/A	0	#N/A
#N/A	0	#N/A	0	#N/A	0	#N/A
#N/A	0	#N/A	0	#N/A	0	#N/A
#N/A	0	#N/A	0	#N/A	0	#N/A
#N/A	0	#N/A	0	#N/A	0	#N/A
#N/A	0	#N/A	0	#N/A	0	#N/A
#N/A	0	#N/A	0	#N/A	0	#N/A
#N/A	0	#N/A	0	#N/A	0	#N/A
#N/A	0	#N/A	0	#N/A	0	#N/A
#N/A	0	#N/A	0	#N/A	0	#N/A
#N/A	0	#N/A	0	#N/A	0	#N/A
#N/A	0	#N/A	0	#N/A	0	#N/A
#N/A	0	#N/A	0	#N/A	0	#N/A
#N/A	0	#N/A	0	#N/A	0	#N/A
LOST-6_art	1		0	Ew/a-LOST-ar	1	
LOST-6_art	1		0	Ew/a-LOST-ar	1	
LOST-6_art	1		0	Ew/a-LOST-ar	1	
LOST-6_art	1		0	Ew/a-LOST-ar	1	
LOST-6_art	1		0	Ew/a-LOST-ar	1	
#N/A	0	#N/A	0	#N/A	0	#N/A
#N/A	0	#N/A	0	#N/A	0	#N/A
#N/A	0	#N/A	0	#N/A	0	#N/A
#N/A	0	#N/A	0	#N/A	0	#N/A
#N/A	0	#N/A	0	#N/A	0	#N/A
#N/A	0	#N/A	0	#N/A	0	#N/A
#N/A	0	#N/A	0	#N/A	0	#N/A
	0		0	Ew/a-LOST-ar	1	Ew/p-LOST-ar
	0		0	Ew/a-LOST-ar	1	Ew/p-LOST-ar
LOST-6_art	1		0		0	
LOST-6_art	1	LOST>6_art	1	Ew/a-LOST-ar	1	Ew/p-LOST-ar
#N/A	0	#N/A	0	#N/A	0	#N/A
#N/A	0	#N/A	0	#N/A	0	#N/A
	0		0	Ew/a-LOST-ar	1	Ew/p-LOST-ar
	0		0	Ew/a-LOST-ar	1	Ew/p-LOST-ar
#N/A	0	#N/A	0	#N/A	0	#N/A
#N/A	0	#N/A	0	#N/A	0	#N/A

#N/A	0	#N/A	0	#N/A	0	#N/A
#N/A	0	#N/A	0	#N/A	0	#N/A
#N/A	0	#N/A	0	#N/A	0	#N/A
#N/A	0	#N/A	0	#N/A	0	#N/A
#N/A	0	#N/A	0	#N/A	0	#N/A
#N/A	0	#N/A	0	#N/A	0	#N/A
#N/A	0	#N/A	0	#N/A	0	#N/A
LOST-6_art	1	LOST>6_art	1		0	
LOST-6_art	1	LOST>6_art	1		0	
#N/A	0	#N/A	0	#N/A	0	#N/A
#N/A	0	#N/A	0	#N/A	0	#N/A
#N/A	0	#N/A	0	#N/A	0	#N/A
#N/A	0	#N/A	0	#N/A	0	#N/A
#N/A	0	#N/A	0	#N/A	0	#N/A
#N/A	0	#N/A	0	#N/A	0	#N/A
	0	LOST>6_art	1		0	
	0		0		0	
#N/A	0	#N/A	0	#N/A	0	#N/A
#N/A	0	#N/A	0	#N/A	0	#N/A
#N/A	0	#N/A	0	#N/A	0	#N/A
#N/A	0	#N/A	0	#N/A	0	#N/A
#N/A	0	#N/A	0	#N/A	0	#N/A
#N/A	0	#N/A	0	#N/A	0	#N/A
#N/A	0	#N/A	0	#N/A	0	#N/A
#N/A	0	#N/A	0	#N/A	0	#N/A
#N/A	0	#N/A	0	#N/A	0	#N/A
#N/A	0	#N/A	0	#N/A	0	#N/A
#N/A	0	#N/A	0	#N/A	0	#N/A
LOST-6_art	1	LOST>6_art	1	Ew/a-LOST-ar	1	Ew/p-LOST-ar
LOST-6_art	1	LOST>6_art	1	Ew/a-LOST-ar	1	Ew/p-LOST-ar
#N/A	0	#N/A	0	#N/A	0	#N/A
#N/A	0	#N/A	0	#N/A	0	#N/A
#N/A	0	#N/A	0	#N/A	0	#N/A
#N/A	0	#N/A	0	#N/A	0	#N/A
#N/A	0	#N/A	0	#N/A	0	#N/A
#N/A	0	#N/A	0	#N/A	0	#N/A
#N/A	0	#N/A	0	#N/A	0	#N/A
#N/A	0	#N/A	0	#N/A	0	#N/A
#N/A	0	#N/A	0	#N/A	0	#N/A
#N/A	0	#N/A	0	#N/A	0	#N/A
#N/A	0	#N/A	0	#N/A	0	#N/A
#N/A	0	#N/A	0	#N/A	0	#N/A
#N/A	0	#N/A	0	#N/A	0	#N/A
#N/A	0	#N/A	0	#N/A	0	#N/A
	0		0	Ew/a-LOST-ar	1	
	0		0	Ew/a-LOST-ar	1	
#N/A	0	#N/A	0	#N/A	0	#N/A

#N/A	0	#N/A	0	#N/A	0	#N/A
#N/A	0	#N/A	0	#N/A	0	#N/A
#N/A	0	#N/A	0	#N/A	0	#N/A
#N/A	0	#N/A	0	#N/A	0	#N/A
#N/A	0	#N/A	0	#N/A	0	#N/A
#N/A	0	#N/A	0	#N/A	0	#N/A
#N/A	0	#N/A	0	#N/A	0	#N/A
#N/A	0	#N/A	0	#N/A	0	#N/A
#N/A	0	#N/A	0	#N/A	0	#N/A
#N/A	0	#N/A	0	#N/A	0	#N/A
	0		0	Ew/a-LOST-ar	1	
LOST-6_art	1		0		0	
#N/A	0	#N/A	0	#N/A	0	#N/A
#N/A	0	#N/A	0	#N/A	0	#N/A
#N/A	0	#N/A	0	#N/A	0	#N/A
#N/A	0	#N/A	0	#N/A	0	#N/A
#N/A	0	#N/A	0	#N/A	0	#N/A
#N/A	0	#N/A	0	#N/A	0	#N/A
#N/A	0	#N/A	0	#N/A	0	#N/A
#N/A	0	#N/A	0	#N/A	0	#N/A
#N/A	0	#N/A	0	#N/A	0	#N/A
#N/A	0	#N/A	0	#N/A	0	#N/A
#N/A	0	#N/A	0	#N/A	0	#N/A
#N/A	0	#N/A	0	#N/A	0	#N/A
LOST-6_art	1	LOST>6_art	1		0	
#N/A	0	#N/A	0	#N/A	0	#N/A
#N/A	0	#N/A	0	#N/A	0	#N/A
	0		0		0	Ew/p-LOST-ar
	0		0		0	Ew/p-LOST-ar
	0		0		0	Ew/p-LOST-ar
	0		0		0	Ew/p-LOST-ar
#N/A	0	#N/A	0	#N/A	0	#N/A
#N/A	0	#N/A	0	#N/A	0	#N/A
#N/A	0	#N/A	0	#N/A	0	#N/A
LOST-6_art	1		0		0	
LOST-6_art	1		0		0	
#N/A	0	#N/A	0	#N/A	0	#N/A
	0		0	Ew/a-LOST-ar	1	
	0		0	Ew/a-LOST-ar	1	
#N/A	0	#N/A	0	#N/A	0	#N/A
#N/A	0	#N/A	0	#N/A	0	#N/A
#N/A	0	#N/A	0	#N/A	0	#N/A
#N/A	0	#N/A	0	#N/A	0	#N/A
#N/A	0	#N/A	0	#N/A	0	#N/A
#N/A	0	#N/A	0	#N/A	0	#N/A
#N/A	0	#N/A	0	#N/A	0	#N/A
	0		0	Ew/a-LOST-ar	1	

	0		0	Ew/a-LOST-ar	1	Ew/p-LOST-ar
	0		0	Ew/a-LOST-ar	1	Ew/p-LOST-ar
	0		0	Ew/a-LOST-ar	1	Ew/p-LOST-ar
	0		0		0	
	0		0		0	
#N/A	0	#N/A	0	#N/A	0	#N/A
#N/A	0	#N/A	0	#N/A	0	#N/A
#N/A	0	#N/A	0	#N/A	0	#N/A
#N/A	0	#N/A	0	#N/A	0	#N/A
#N/A	0	#N/A	0	#N/A	0	#N/A
#N/A	0	#N/A	0	#N/A	0	#N/A
#N/A	0	#N/A	0	#N/A	0	#N/A
#N/A	0	#N/A	0	#N/A	0	#N/A
#N/A	0	#N/A	0	#N/A	0	#N/A
#N/A	0	#N/A	0	#N/A	0	#N/A
LOST-6_art	1		0		0	
#N/A	0	#N/A	0	#N/A	0	#N/A
#N/A	0	#N/A	0	#N/A	0	#N/A
#N/A	0	#N/A	0	#N/A	0	#N/A
#N/A	0	#N/A	0	#N/A	0	#N/A
#N/A	0	#N/A	0	#N/A	0	#N/A
#N/A	0	#N/A	0	#N/A	0	#N/A
#N/A	0	#N/A	0	#N/A	0	#N/A
#N/A	0	#N/A	0	#N/A	0	#N/A
#N/A	0	#N/A	0	#N/A	0	#N/A
#N/A	0	#N/A	0	#N/A	0	#N/A
#N/A	0	#N/A	0	#N/A	0	#N/A
#N/A	0	#N/A	0	#N/A	0	#N/A
#N/A	0	#N/A	0	#N/A	0	#N/A
	0		0		0	
	0		0		0	
#N/A	0	#N/A	0	#N/A	0	#N/A
#N/A	0	#N/A	0	#N/A	0	#N/A
#N/A	0	#N/A	0	#N/A	0	#N/A
#N/A	0	#N/A	0	#N/A	0	#N/A
#N/A	0	#N/A	0	#N/A	0	#N/A
#N/A	0	#N/A	0	#N/A	0	#N/A
#N/A	0	#N/A	0	#N/A	0	#N/A
#N/A	0	#N/A	0	#N/A	0	#N/A
#N/A	0	#N/A	0	#N/A	0	#N/A
#N/A	0	#N/A	0	#N/A	0	#N/A
#N/A	0	#N/A	0	#N/A	0	#N/A
LOST-6_art	1	LOST>6_art	1		0	
#N/A	0	#N/A	0	#N/A	0	#N/A
#N/A	0	#N/A	0	#N/A	0	#N/A
#N/A	0	#N/A	0	#N/A	0	#N/A
#N/A	0	#N/A	0	#N/A	0	#N/A

#N/A	0	#N/A	0	#N/A	0	#N/A
#N/A	0	#N/A	0	#N/A	0	#N/A
#N/A	0	#N/A	0	#N/A	0	#N/A
	0		0	Ew/a-LOST-ar	1	Ew/p-LOST-ar
	0		0	Ew/a-LOST-ar	1	Ew/p-LOST-ar
#N/A	0	#N/A	0	#N/A	0	#N/A
	0	LOST>6_art	1	Ew/a-LOST-ar	1	Ew/p-LOST-ar
#N/A	0	#N/A	0	#N/A	0	#N/A
#N/A	0	#N/A	0	#N/A	0	#N/A
#N/A	0	#N/A	0	#N/A	0	#N/A
	0	LOST>6_art	1		0	
	0		0		0	
	0		0		0	
	0		0		0	
#N/A	0	#N/A	0	#N/A	0	#N/A
#N/A	0	#N/A	0	#N/A	0	#N/A
#N/A	0	#N/A	0	#N/A	0	#N/A
#N/A	0	#N/A	0	#N/A	0	#N/A
#N/A	0	#N/A	0	#N/A	0	#N/A
#N/A	0	#N/A	0	#N/A	0	#N/A
#N/A	0	#N/A	0	#N/A	0	#N/A
#N/A	0	#N/A	0	#N/A	0	#N/A
#N/A	0	#N/A	0	#N/A	0	#N/A
#N/A	0	#N/A	0	#N/A	0	#N/A
#N/A	0	#N/A	0	#N/A	0	#N/A
LOST-6_art	1		0	Ew/a-LOST-ar	1	Ew/p-LOST-ar
#N/A	0	#N/A	0	#N/A	0	#N/A
#N/A	0	#N/A	0	#N/A	0	#N/A
LOST-6_art	1	LOST>6_art	1		0	
#N/A	0	#N/A	0	#N/A	0	#N/A
#N/A	0	#N/A	0	#N/A	0	#N/A
#N/A	0	#N/A	0	#N/A	0	#N/A
	0	LOST>6_art	1		0	
LOST-6_art	1		0	Ew/a-LOST-ar	1	Ew/p-LOST-ar
LOST-6_art	1		0	Ew/a-LOST-ar	1	Ew/p-LOST-ar
LOST-6_art	1		0		0	
LOST-6_art	1		0		0	
#N/A	0	#N/A	0	#N/A	0	#N/A
#N/A	0	#N/A	0	#N/A	0	#N/A
#N/A	0	#N/A	0	#N/A	0	#N/A
#N/A	0	#N/A	0	#N/A	0	#N/A
#N/A	0	#N/A	0	#N/A	0	#N/A
#N/A	0	#N/A	0	#N/A	0	#N/A
#N/A	0	#N/A	0	#N/A	0	#N/A
#N/A	0	#N/A	0	#N/A	0	#N/A
#N/A	0	#N/A	0	#N/A	0	#N/A
#N/A	0	#N/A	0	#N/A	0	#N/A

#N/A	0	#N/A	0	#N/A	0	#N/A	
#N/A	0	#N/A	0	#N/A	0	#N/A	
#N/A	0	#N/A	0	#N/A	0	#N/A	
#N/A	0	#N/A	0	#N/A	0	#N/A	
LOST-6_art	1	LOST>6_art	1		0		
#N/A	0	#N/A	0	#N/A	0	#N/A	
#N/A	0	#N/A	0	#N/A	0	#N/A	
#N/A	0	#N/A	0	#N/A	0	#N/A	
#N/A	0	#N/A	0	#N/A	0	#N/A	
#N/A	0	#N/A	0	#N/A	0	#N/A	
#N/A	0	#N/A	0	#N/A	0	#N/A	
#N/A	0	#N/A	0	#N/A	0	#N/A	
#N/A	0	#N/A	0	#N/A	0	#N/A	
#N/A	0	#N/A	0	#N/A	0	#N/A	
#N/A	0	#N/A	0	#N/A	0	#N/A	
#N/A	0	#N/A	0	#N/A	0	#N/A	
#N/A	0	#N/A	0	#N/A	0	#N/A	
#N/A	0	#N/A	0	#N/A	0	#N/A	
#N/A	0	#N/A	0	#N/A	0	#N/A	
#N/A	0	#N/A	0	#N/A	0	#N/A	
#N/A	0	#N/A	0	#N/A	0	#N/A	
#N/A	0	#N/A	0	#N/A	0	#N/A	
#N/A	0	#N/A	0	#N/A	0	#N/A	
#N/A	0	#N/A	0	#N/A	0	#N/A	
#N/A	0	#N/A	0	#N/A	0	#N/A	
#N/A	0	#N/A	0	#N/A	0	#N/A	
#N/A	0	#N/A	0	#N/A	0	#N/A	
	0	LOST>6_art	1		0	Ew/p-LOST-ar	
	0	LOST>6_art	1		0	Ew/p-LOST-ar	
#N/A	0	#N/A	0	#N/A	0	#N/A	
#N/A	0	#N/A	0	#N/A	0	#N/A	
#N/A	0	#N/A	0	#N/A	0	#N/A	
#N/A	0	#N/A	0	#N/A	0	#N/A	
#N/A	0	#N/A	0	#N/A	0	#N/A	
#N/A	0	#N/A	0	#N/A	0	#N/A	
#N/A	0	#N/A	0	#N/A	0	#N/A	
#N/A	0	#N/A	0	#N/A	0	#N/A	
#N/A	0	#N/A	0	#N/A	0	#N/A	
#N/A	0	#N/A	0	#N/A	0	#N/A	
#N/A	0	#N/A	0	#N/A	0	#N/A	
#N/A	0	#N/A	0	#N/A	0	#N/A	
LOST-6_art	1		0	Ew/a-LOST-ar	1	Ew/p-LOST-ar	
#N/A	0	#N/A	0	#N/A	0	#N/A	
#N/A	0	#N/A	0	#N/A	0	#N/A	
#N/A	0	#N/A	0	#N/A	0	#N/A	
#N/A	0	#N/A	0	#N/A	0	#N/A	
#N/A	0	#N/A	0	#N/A	0	#N/A	

#N/A	0	#N/A	0	#N/A	0	#N/A
#N/A	0	#N/A	0	#N/A	0	#N/A
#N/A	0	#N/A	0	#N/A	0	#N/A
#N/A	0	#N/A	0	#N/A	0	#N/A
#N/A	0	#N/A	0	#N/A	0	#N/A
	0		0		0	
#N/A	0	#N/A	0	#N/A	0	#N/A
#N/A	0	#N/A	0	#N/A	0	#N/A
#N/A	0	#N/A	0	#N/A	0	#N/A
#N/A	0	#N/A	0	#N/A	0	#N/A
	0		0	Ew/a-LOST-ar	1	Ew/p-LOST-ar
	0		0	Ew/a-LOST-ar	1	Ew/p-LOST-ar
#N/A	0	#N/A	0	#N/A	0	#N/A
#N/A	0	#N/A	0	#N/A	0	#N/A
#N/A	0	#N/A	0	#N/A	0	#N/A
#N/A	0	#N/A	0	#N/A	0	#N/A
#N/A	0	#N/A	0	#N/A	0	#N/A
#N/A	0	#N/A	0	#N/A	0	#N/A
#N/A	0	#N/A	0	#N/A	0	#N/A
	0		0	Ew/a-LOST-ar	1	Ew/p-LOST-ar
	0		0	Ew/a-LOST-ar	1	Ew/p-LOST-ar
	0		0	Ew/a-LOST-ar	1	Ew/p-LOST-ar
#N/A	0	#N/A	0	#N/A	0	#N/A
#N/A	0	#N/A	0	#N/A	0	#N/A
#N/A	0	#N/A	0	#N/A	0	#N/A
#N/A	0	#N/A	0	#N/A	0	#N/A
#N/A	0	#N/A	0	#N/A	0	#N/A
#N/A	0	#N/A	0	#N/A	0	#N/A
#N/A	0	#N/A	0	#N/A	0	#N/A
#N/A	0	#N/A	0	#N/A	0	#N/A
#N/A	0	#N/A	0	#N/A	0	#N/A
#N/A	0	#N/A	0	#N/A	0	#N/A
#N/A	0	#N/A	0	#N/A	0	#N/A
#N/A	0	#N/A	0	#N/A	0	#N/A
#N/A	0	#N/A	0	#N/A	0	#N/A
#N/A	0	#N/A	0	#N/A	0	#N/A
	0		0	Ew/a-LOST-ar	1	Ew/p-LOST-ar
	0		0	Ew/a-LOST-ar	1	Ew/p-LOST-ar
	0		0	Ew/a-LOST-ar	1	Ew/p-LOST-ar
#N/A	0	#N/A	0	#N/A	0	#N/A
#N/A	0	#N/A	0	#N/A	0	#N/A
#N/A	0	#N/A	0	#N/A	0	#N/A
#N/A	0	#N/A	0	#N/A	0	#N/A
LOST-6_art	1	LOST>6_art	1		0	
LOST-6_art	1		0		0	
LOST-6_art	1		0		0	
LOST-6_art	1		0		0	

#N/A	0	#N/A	0	#N/A	0	#N/A
#N/A	0	#N/A	0	#N/A	0	#N/A
LOST-6_art	1		0	Ew/a-LOST-ar	1	
#N/A	0	#N/A	0	#N/A	0	#N/A
#N/A	0	#N/A	0	#N/A	0	#N/A
#N/A	0	#N/A	0	#N/A	0	#N/A
LOST-6_art	1		0	Ew/a-LOST-ar	1	Ew/p-LOST-ar
	0		0		0	Ew/p-LOST-ar
#N/A	0	#N/A	0	#N/A	0	#N/A
#N/A	0	#N/A	0	#N/A	0	#N/A
#N/A	0	#N/A	0	#N/A	0	#N/A
#N/A	0	#N/A	0	#N/A	0	#N/A
#N/A	0	#N/A	0	#N/A	0	#N/A
	0		0	Ew/a-LOST-ar	1	Ew/p-LOST-ar
	0		0	Ew/a-LOST-ar	1	Ew/p-LOST-ar
	0		0	Ew/a-LOST-ar	1	Ew/p-LOST-ar
#N/A	0	#N/A	0	#N/A	0	#N/A
#N/A	0	#N/A	0	#N/A	0	#N/A
#N/A	0	#N/A	0	#N/A	0	#N/A
#N/A	0	#N/A	0	#N/A	0	#N/A
LOST-6_art	1		0		0	
#N/A	0	#N/A	0	#N/A	0	#N/A
#N/A	0	#N/A	0	#N/A	0	#N/A
#N/A	0	#N/A	0	#N/A	0	#N/A
#N/A	0	#N/A	0	#N/A	0	#N/A
#N/A	0	#N/A	0	#N/A	0	#N/A
#N/A	0	#N/A	0	#N/A	0	#N/A
#N/A	0	#N/A	0	#N/A	0	#N/A
#N/A	0	#N/A	0	#N/A	0	#N/A
#N/A	0	#N/A	0	#N/A	0	#N/A
#N/A	0	#N/A	0	#N/A	0	#N/A
#N/A	0	#N/A	0	#N/A	0	#N/A
#N/A	0	#N/A	0	#N/A	0	#N/A
#N/A	0	#N/A	0	#N/A	0	#N/A
#N/A	0	#N/A	0	#N/A	0	#N/A
#N/A	0	#N/A	0	#N/A	0	#N/A
#N/A	0	#N/A	0	#N/A	0	#N/A
#N/A	0	#N/A	0	#N/A	0	#N/A
#N/A	0	#N/A	0	#N/A	0	#N/A
#N/A	0	#N/A	0	#N/A	0	#N/A
#N/A	0	#N/A	0	#N/A	0	#N/A
	0		0	Ew/a-LOST-ar	1	
	0		0		0	
#N/A	0	#N/A	0	#N/A	0	#N/A
LOST-6_art	1	LOST>6_art	1		0	
#N/A	0	#N/A	0	#N/A	0	#N/A
#N/A	0	#N/A	0	#N/A	0	#N/A

#N/A	0	#N/A	0	#N/A	0	#N/A
#N/A	0	#N/A	0	#N/A	0	#N/A
#N/A	0	#N/A	0	#N/A	0	#N/A
LOST-6_art	1		0		0	
#N/A	0	#N/A	0	#N/A	0	#N/A
#N/A	0	#N/A	0	#N/A	0	#N/A
LOST-6_art	1	LOST>6_art	1		0	
#N/A	0	#N/A	0	#N/A	0	#N/A
#N/A	0	#N/A	0	#N/A	0	#N/A
#N/A	0	#N/A	0	#N/A	0	#N/A
#N/A	0	#N/A	0	#N/A	0	#N/A
	0		0	Ew/a-LOST-ar	1	Ew/p-LOST-ar
	0		0	Ew/a-LOST-ar	1	Ew/p-LOST-ar
#N/A	0	#N/A	0	#N/A	0	#N/A
#N/A	0	#N/A	0	#N/A	0	#N/A
#N/A	0	#N/A	0	#N/A	0	#N/A
#N/A	0	#N/A	0	#N/A	0	#N/A
#N/A	0	#N/A	0	#N/A	0	#N/A
#N/A	0	#N/A	0	#N/A	0	#N/A
#N/A	0	#N/A	0	#N/A	0	#N/A
#N/A	0	#N/A	0	#N/A	0	#N/A
	0		0	Ew/a-LOST-ar	1	
	0		0	Ew/a-LOST-ar	1	
	0		0	Ew/a-LOST-ar	1	
#N/A	0	#N/A	0	#N/A	0	#N/A
#N/A	0	#N/A	0	#N/A	0	#N/A
	0		0		0	
	0		0	Ew/a-LOST-ar	1	
	0		0	Ew/a-LOST-ar	1	
	0		0	Ew/a-LOST-ar	1	
#N/A	0	#N/A	0	#N/A	0	#N/A
#N/A	0	#N/A	0	#N/A	0	#N/A
#N/A	0	#N/A	0	#N/A	0	#N/A
LOST-6_art	1		0	Ew/a-LOST-ar	1	Ew/p-LOST-ar
LOST-6_art	1		0	Ew/a-LOST-ar	1	Ew/p-LOST-ar
#N/A	0	#N/A	0	#N/A	0	#N/A
#N/A	0	#N/A	0	#N/A	0	#N/A
#N/A	0	#N/A	0	#N/A	0	#N/A
#N/A	0	#N/A	0	#N/A	0	#N/A
#N/A	0	#N/A	0	#N/A	0	#N/A
	0	LOST>6_art	1		0	
#N/A	0	#N/A	0	#N/A	0	#N/A
#N/A	0	#N/A	0	#N/A	0	#N/A
#N/A	0	#N/A	0	#N/A	0	#N/A
#N/A	0	#N/A	0	#N/A	0	#N/A
LOST-6_art	1	LOST>6_art	1		0	
#N/A	0	#N/A	0	#N/A	0	#N/A

#N/A	0	#N/A	0	#N/A	0	#N/A
#N/A	0	#N/A	0	#N/A	0	#N/A
#N/A	0	#N/A	0	#N/A	0	#N/A
#N/A	0	#N/A	0	#N/A	0	#N/A
#N/A	0	#N/A	0	#N/A	0	#N/A
#N/A	0	#N/A	0	#N/A	0	#N/A
#N/A	0	#N/A	0	#N/A	0	#N/A
LOST-6_art	1	LOST>6_art	1		0	
LOST-6_art	1	LOST>6_art	1		0	
LOST-6_art	1	LOST>6_art	1		0	
LOST-6_art	1		0	Ew/a-LOST-ar	1	Ew/p-LOST-ar
LOST-6_art	1		0	Ew/a-LOST-ar	1	Ew/p-LOST-ar
LOST-6_art	1		0	Ew/a-LOST-ar	1	Ew/p-LOST-ar
LOST-6_art	1		0	Ew/a-LOST-ar	1	Ew/p-LOST-ar
#N/A	0	#N/A	0	#N/A	0	#N/A
	0	LOST>6_art	1		0	
	0	LOST>6_art	1		0	
	0	LOST>6_art	1		0	
#N/A	0	#N/A	0	#N/A	0	#N/A
#N/A	0	#N/A	0	#N/A	0	#N/A
#N/A	0	#N/A	0	#N/A	0	#N/A
#N/A	0	#N/A	0	#N/A	0	#N/A
#N/A	0	#N/A	0	#N/A	0	#N/A
#N/A	0	#N/A	0	#N/A	0	#N/A
#N/A	0	#N/A	0	#N/A	0	#N/A
	0		0		0	
	0		0		0	
#N/A	0	#N/A	0	#N/A	0	#N/A
	0	LOST>6_art	1		0	
#N/A	0	#N/A	0	#N/A	0	#N/A
#N/A	0	#N/A	0	#N/A	0	#N/A
#N/A	0	#N/A	0	#N/A	0	#N/A
	0		0		0	
	0		0		0	
#N/A	0	#N/A	0	#N/A	0	#N/A
#N/A	0	#N/A	0	#N/A	0	#N/A
#N/A	0	#N/A	0	#N/A	0	#N/A
#N/A	0	#N/A	0	#N/A	0	#N/A
#N/A	0	#N/A	0	#N/A	0	#N/A
#N/A	0	#N/A	0	#N/A	0	#N/A
#N/A	0	#N/A	0	#N/A	0	#N/A
LOST-6_art	1		0		0	
#N/A	0	#N/A	0	#N/A	0	#N/A
	0		0	Ew/a-LOST-ar	1	Ew/p-LOST-ar
	0		0	Ew/a-LOST-ar	1	Ew/p-LOST-ar
	0		0	Ew/a-LOST-ar	1	Ew/p-LOST-ar
	0		0		0	

#N/A	0	#N/A	0	#N/A	0	#N/A
#N/A	0	#N/A	0	#N/A	0	#N/A
#N/A	0	#N/A	0	#N/A	0	#N/A
#N/A	0	#N/A	0	#N/A	0	#N/A
#N/A	0	#N/A	0	#N/A	0	#N/A
#N/A	0	#N/A	0	#N/A	0	#N/A
#N/A	0	#N/A	0	#N/A	0	#N/A
	0		0		0	
	0		0		0	
#N/A	0	#N/A	0	#N/A	0	#N/A
#N/A	0	#N/A	0	#N/A	0	#N/A
#N/A	0	#N/A	0	#N/A	0	#N/A
LOST-6_art	1	LOST>6_art	1		0	
#N/A	0	#N/A	0	#N/A	0	#N/A
#N/A	0	#N/A	0	#N/A	0	#N/A
#N/A	0	#N/A	0	#N/A	0	#N/A
#N/A	0	#N/A	0	#N/A	0	#N/A
#N/A	0	#N/A	0	#N/A	0	#N/A
#N/A	0	#N/A	0	#N/A	0	#N/A
	0	LOST>6_art	1		0	
#N/A	0	#N/A	0	#N/A	0	#N/A
#N/A	0	#N/A	0	#N/A	0	#N/A
#N/A	0	#N/A	0	#N/A	0	#N/A
#N/A	0	#N/A	0	#N/A	0	#N/A
LOST-6_art	1	LOST>6_art	1		0	
#N/A	0	#N/A	0	#N/A	0	#N/A
#N/A	0	#N/A	0	#N/A	0	#N/A
#N/A	0	#N/A	0	#N/A	0	#N/A
#N/A	0	#N/A	0	#N/A	0	#N/A
LOST-6_art	1		0		0	Ew/p-LOST-ar
#N/A	0	#N/A	0	#N/A	0	#N/A
#N/A	0	#N/A	0	#N/A	0	#N/A
#N/A	0	#N/A	0	#N/A	0	#N/A
#N/A	0	#N/A	0	#N/A	0	#N/A
#N/A	0	#N/A	0	#N/A	0	#N/A
#N/A	0	#N/A	0	#N/A	0	#N/A
#N/A	0	#N/A	0	#N/A	0	#N/A
#N/A	0	#N/A	0	#N/A	0	#N/A
LOST-6_art	1		0		0	
#N/A	0	#N/A	0	#N/A	0	#N/A
#N/A	0	#N/A	0	#N/A	0	#N/A
#N/A	0	#N/A	0	#N/A	0	#N/A
LOST-6_art	1	LOST>6_art	1		0	
LOST-6_art	1		0	Ew/a-LOST-ar	1	Ew/p-LOST-ar
LOST-6_art	1		0	Ew/a-LOST-ar	1	Ew/p-LOST-ar
LOST-6_art	1		0	Ew/a-LOST-ar	1	Ew/p-LOST-ar
LOST-6_art	1		0	Ew/a-LOST-ar	1	Ew/p-LOST-ar

#N/A	0	#N/A	0	#N/A	0	#N/A
#N/A	0	#N/A	0	#N/A	0	#N/A
#N/A	0	#N/A	0	#N/A	0	#N/A
#N/A	0	#N/A	0	#N/A	0	#N/A
#N/A	0	#N/A	0	#N/A	0	#N/A
#N/A	0	#N/A	0	#N/A	0	#N/A
#N/A	0	#N/A	0	#N/A	0	#N/A
LOST-6_art	1		0		0	
#N/A	0	#N/A	0	#N/A	0	#N/A
#N/A	0	#N/A	0	#N/A	0	#N/A
#N/A	0	#N/A	0	#N/A	0	#N/A
	0		0		0	
#N/A	0	#N/A	0	#N/A	0	#N/A
#N/A	0	#N/A	0	#N/A	0	#N/A
#N/A	0	#N/A	0	#N/A	0	#N/A
#N/A	0	#N/A	0	#N/A	0	#N/A
#N/A	0	#N/A	0	#N/A	0	#N/A
#N/A	0	#N/A	0	#N/A	0	#N/A
#N/A	0	#N/A	0	#N/A	0	#N/A
LOST-6_art	1	LOST>6_art	1	Ew/a-LOST-ar	1	
LOST-6_art	1	LOST>6_art	1	Ew/a-LOST-ar	1	
#N/A	0	#N/A	0	#N/A	0	#N/A
	0		0	Ew/a-LOST-ar	1	
	0		0	Ew/a-LOST-ar	1	
	0		0	Ew/a-LOST-ar	1	
	0	LOST>6_art	1		0	Ew/p-LOST-ar
	0	LOST>6_art	1		0	Ew/p-LOST-ar
	0	LOST>6_art	1	Ew/a-LOST-ar	1	Ew/p-LOST-ar
#N/A	0	#N/A	0	#N/A	0	#N/A
#N/A	0	#N/A	0	#N/A	0	#N/A
#N/A	0	#N/A	0	#N/A	0	#N/A
	0	LOST>6_art	1	Ew/a-LOST-ar	1	Ew/p-LOST-ar
#N/A	0	#N/A	0	#N/A	0	#N/A
#N/A	0	#N/A	0	#N/A	0	#N/A
#N/A	0	#N/A	0	#N/A	0	#N/A
#N/A	0	#N/A	0	#N/A	0	#N/A
#N/A	0	#N/A	0	#N/A	0	#N/A
LOST-6_art	1	LOST>6_art	1		0	
#N/A	0	#N/A	0	#N/A	0	#N/A
#N/A	0	#N/A	0	#N/A	0	#N/A
#N/A	0	#N/A	0	#N/A	0	#N/A
#N/A	0	#N/A	0	#N/A	0	#N/A
#N/A	0	#N/A	0	#N/A	0	#N/A
#N/A	0	#N/A	0	#N/A	0	#N/A
#N/A	0	#N/A	0	#N/A	0	#N/A
#N/A	0	#N/A	0	#N/A	0	#N/A
#N/A	0	#N/A	0	#N/A	0	#N/A

#N/A	0	#N/A	0	#N/A	0	#N/A
#N/A	0	#N/A	0	#N/A	0	#N/A
#N/A	0	#N/A	0	#N/A	0	#N/A
#N/A	0	#N/A	0	#N/A	0	#N/A
LOST-6_art	1		0		0	
	0	LOST>6_art	1		0	Ew/p-LOST-ar
#N/A	0	#N/A	0	#N/A	0	#N/A
#N/A	0	#N/A	0	#N/A	0	#N/A
LOST-6_art	1	LOST>6_art	1		0	Ew/p-LOST-ar
LOST-6_art	1	LOST>6_art	1		0	Ew/p-LOST-ar
LOST-6_art	1	LOST>6_art	1		0	Ew/p-LOST-ar
LOST-6_art	1	LOST>6_art	1		0	Ew/p-LOST-ar
LOST-6_art	1	LOST>6_art	1		0	Ew/p-LOST-ar
#N/A	0	#N/A	0	#N/A	0	#N/A
#N/A	0	#N/A	0	#N/A	0	#N/A
#N/A	0	#N/A	0	#N/A	0	#N/A
#N/A	0	#N/A	0	#N/A	0	#N/A
#N/A	0	#N/A	0	#N/A	0	#N/A
#N/A	0	#N/A	0	#N/A	0	#N/A
#N/A	0	#N/A	0	#N/A	0	#N/A
#N/A	0	#N/A	0	#N/A	0	#N/A
#N/A	0	#N/A	0	#N/A	0	#N/A
#N/A	0	#N/A	0	#N/A	0	#N/A
#N/A	0	#N/A	0	#N/A	0	#N/A
#N/A	0	#N/A	0	#N/A	0	#N/A
#N/A	0	#N/A	0	#N/A	0	#N/A
#N/A	0	#N/A	0	#N/A	0	#N/A
LOST-6_art	1		0		0	Ew/p-LOST-ar
LOST-6_art	1		0		0	Ew/p-LOST-ar
#N/A	0	#N/A	0	#N/A	0	#N/A
#N/A	0	#N/A	0	#N/A	0	#N/A
#N/A	0	#N/A	0	#N/A	0	#N/A
#N/A	0	#N/A	0	#N/A	0	#N/A
#N/A	0	#N/A	0	#N/A	0	#N/A
#N/A	0	#N/A	0	#N/A	0	#N/A
#N/A	0	#N/A	0	#N/A	0	#N/A
#N/A	0	#N/A	0	#N/A	0	#N/A
#N/A	0	#N/A	0	#N/A	0	#N/A
LOST-6_art	1	LOST>6_art	1	Ew/a-LOST-ar	1	
LOST-6_art	1	LOST>6_art	1		0	
	0		0	Ew/a-LOST-ar	1	Ew/p-LOST-ar
	0		0	Ew/a-LOST-ar	1	Ew/p-LOST-ar
	0		0	Ew/a-LOST-ar	1	Ew/p-LOST-ar
#N/A	0	#N/A	0	#N/A	0	#N/A
#N/A	0	#N/A	0	#N/A	0	#N/A
#N/A	0	#N/A	0	#N/A	0	#N/A
#N/A	0	#N/A	0	#N/A	0	#N/A

#N/A	0	#N/A	0	#N/A	0	#N/A
LOST-6_art	1		0	Ew/a-LOST-ar	1	Ew/p-LOST-ar
#N/A	0	#N/A	0	#N/A	0	#N/A
#N/A	0	#N/A	0	#N/A	0	#N/A
#N/A	0	#N/A	0	#N/A	0	#N/A
#N/A	0	#N/A	0	#N/A	0	#N/A
#N/A	0	#N/A	0	#N/A	0	#N/A
#N/A	0	#N/A	0	#N/A	0	#N/A
#N/A	0	#N/A	0	#N/A	0	#N/A
#N/A	0	#N/A	0	#N/A	0	#N/A
#N/A	0	#N/A	0	#N/A	0	#N/A
#N/A	0	#N/A	0	#N/A	0	#N/A
#N/A	0	#N/A	0	#N/A	0	#N/A
#N/A	0	#N/A	0	#N/A	0	#N/A
#N/A	0	#N/A	0	#N/A	0	#N/A
#N/A	0	#N/A	0	#N/A	0	#N/A
#N/A	0	#N/A	0	#N/A	0	#N/A
#N/A	0	#N/A	0	#N/A	0	#N/A
#N/A	0	#N/A	0	#N/A	0	#N/A
#N/A	0	#N/A	0	#N/A	0	#N/A
#N/A	0	#N/A	0	#N/A	0	#N/A
#N/A	0	#N/A	0	#N/A	0	#N/A
#N/A	0	#N/A	0	#N/A	0	#N/A
#N/A	0	#N/A	0	#N/A	0	#N/A
#N/A	0	#N/A	0	#N/A	0	#N/A
#N/A	0	#N/A	0	#N/A	0	#N/A
#N/A	0	#N/A	0	#N/A	0	#N/A
	0		0	Ew/a-LOST-ar	1	
	0		0	Ew/a-LOST-ar	1	
#N/A	0	#N/A	0	#N/A	0	#N/A
#N/A	0	#N/A	0	#N/A	0	#N/A
#N/A	0	#N/A	0	#N/A	0	#N/A
#N/A	0	#N/A	0	#N/A	0	#N/A
#N/A	0	#N/A	0	#N/A	0	#N/A
#N/A	0	#N/A	0	#N/A	0	#N/A
#N/A	0	#N/A	0	#N/A	0	#N/A
#N/A	0	#N/A	0	#N/A	0	#N/A
#N/A	0	#N/A	0	#N/A	0	#N/A
#N/A	0	#N/A	0	#N/A	0	#N/A
#N/A	0	#N/A	0	#N/A	0	#N/A
LOST-6_art	1		0	Ew/a-LOST-ar	1	Ew/p-LOST-ar
LOST-6_art	1		0	Ew/a-LOST-ar	1	Ew/p-LOST-ar
LOST-6_art	1		0	Ew/a-LOST-ar	1	Ew/p-LOST-ar
#N/A	0	#N/A	0	#N/A	0	#N/A
#N/A	0	#N/A	0	#N/A	0	#N/A
#N/A	0	#N/A	0	#N/A	0	#N/A
	0		0		0	

	0		0		0	
#N/A	0	#N/A	0	#N/A	0	#N/A
#N/A	0	#N/A	0	#N/A	0	#N/A
#N/A	0	#N/A	0	#N/A	0	#N/A
	0	LOST>6_art	1		0	
	0	LOST>6_art	1		0	
#N/A	0	#N/A	0	#N/A	0	#N/A
#N/A	0	#N/A	0	#N/A	0	#N/A
#N/A	0	#N/A	0	#N/A	0	#N/A
#N/A	0	#N/A	0	#N/A	0	#N/A
#N/A	0	#N/A	0	#N/A	0	#N/A
#N/A	0	#N/A	0	#N/A	0	#N/A
#N/A	0	#N/A	0	#N/A	0	#N/A
#N/A	0	#N/A	0	#N/A	0	#N/A
#N/A	0	#N/A	0	#N/A	0	#N/A
#N/A	0	#N/A	0	#N/A	0	#N/A
#N/A	0	#N/A	0	#N/A	0	#N/A
#N/A	0	#N/A	0	#N/A	0	#N/A
	0		0		0	Ew/p-LOST-ar
	0		0		0	Ew/p-LOST-ar
	0		0		0	Ew/p-LOST-ar
#N/A	0	#N/A	0	#N/A	0	#N/A
	0		0		0	
	0		0		0	
	0		0		0	
LOST-6_art	1		0		0	Ew/p-LOST-ar
LOST-6_art	1		0		0	Ew/p-LOST-ar
LOST-6_art	1		0		0	
#N/A	0	#N/A	0	#N/A	0	#N/A
#N/A	0	#N/A	0	#N/A	0	#N/A
#N/A	0	#N/A	0	#N/A	0	#N/A
#N/A	0	#N/A	0	#N/A	0	#N/A
	0		0	Ew/a-LOST-ar	1	Ew/p-LOST-ar
	0		0	Ew/a-LOST-ar	1	Ew/p-LOST-ar
	0		0	Ew/a-LOST-ar	1	Ew/p-LOST-ar
	0	LOST>6_art	1		0	
	0	LOST>6_art	1		0	
#N/A	0	#N/A	0	#N/A	0	#N/A
	0		0		0	
	0		0		0	
	0		0		0	
LOST-6_art	1	LOST>6_art	1		0	
LOST-6_art	1	LOST>6_art	1		0	
LOST-6_art	1		0		0	
	0		0	Ew/a-LOST-ar	1	Ew/p-LOST-ar
	0		0	Ew/a-LOST-ar	1	Ew/p-LOST-ar
	0		0	Ew/a-LOST-ar	1	Ew/p-LOST-ar

#N/A	0	#N/A	0	#N/A	0	#N/A
	0	LOST>6_art	1	Ew/a-LOST-ar	1	Ew/p-LOST-ar
#N/A	0	#N/A	0	#N/A	0	#N/A
#N/A	0	#N/A	0	#N/A	0	#N/A
#N/A	0	#N/A	0	#N/A	0	#N/A
#N/A	0	#N/A	0	#N/A	0	#N/A
#N/A	0	#N/A	0	#N/A	0	#N/A
#N/A	0	#N/A	0	#N/A	0	#N/A
#N/A	0	#N/A	0	#N/A	0	#N/A
#N/A	0	#N/A	0	#N/A	0	#N/A
#N/A	0	#N/A	0	#N/A	0	#N/A
#N/A	0	#N/A	0	#N/A	0	#N/A
LOST-6_art	1		0		0	
LOST-6_art	1		0		0	
LOST-6_art	1		0		0	
#N/A	0	#N/A	0	#N/A	0	#N/A
#N/A	0	#N/A	0	#N/A	0	#N/A
#N/A	0	#N/A	0	#N/A	0	#N/A
#N/A	0	#N/A	0	#N/A	0	#N/A
#N/A	0	#N/A	0	#N/A	0	#N/A
#N/A	0	#N/A	0	#N/A	0	#N/A
#N/A	0	#N/A	0	#N/A	0	#N/A
#N/A	0	#N/A	0	#N/A	0	#N/A
#N/A	0	#N/A	0	#N/A	0	#N/A
#N/A	0	#N/A	0	#N/A	0	#N/A
#N/A	0	#N/A	0	#N/A	0	#N/A
#N/A	0	#N/A	0	#N/A	0	#N/A
	0		0		0	
	0		0		0	
#N/A	0	#N/A	0	#N/A	0	#N/A
#N/A	0	#N/A	0	#N/A	0	#N/A
#N/A	0	#N/A	0	#N/A	0	#N/A
#N/A	0	#N/A	0	#N/A	0	#N/A
#N/A	0	#N/A	0	#N/A	0	#N/A
#N/A	0	#N/A	0	#N/A	0	#N/A
#N/A	0	#N/A	0	#N/A	0	#N/A
#N/A	0	#N/A	0	#N/A	0	#N/A
#N/A	0	#N/A	0	#N/A	0	#N/A
#N/A	0	#N/A	0	#N/A	0	#N/A
	0		0	Ew/a-LOST-ar	1	Ew/p-LOST-ar
	0		0	Ew/a-LOST-ar	1	Ew/p-LOST-ar
	0		0	Ew/a-LOST-ar	1	Ew/p-LOST-ar
#N/A	0	#N/A	0	#N/A	0	#N/A
#N/A	0	#N/A	0	#N/A	0	#N/A
#N/A	0	#N/A	0	#N/A	0	#N/A
#N/A	0	#N/A	0	#N/A	0	#N/A
#N/A	0	#N/A	0	#N/A	0	#N/A

#N/A	0	#N/A	0	#N/A	0	#N/A	
#N/A	0	#N/A	0	#N/A	0	#N/A	
#N/A	0	#N/A	0	#N/A	0	#N/A	
#N/A	0	#N/A	0	#N/A	0	#N/A	
#N/A	0	#N/A	0	#N/A	0	#N/A	
#N/A	0	#N/A	0	#N/A	0	#N/A	
#N/A	0	#N/A	0	#N/A	0	#N/A	
LOST-6_art	1	LOST>6_art	1	Ew/a-LOST-ar	1	Ew/p-LOST-ar	
LOST-6_art	1	LOST>6_art	1	Ew/a-LOST-ar	1	Ew/p-LOST-ar	
#N/A	0	#N/A	0	#N/A	0	#N/A	
LOST-6_art	1	LOST>6_art	1		0	Ew/p-LOST-ar	
#N/A	0	#N/A	0	#N/A	0	#N/A	
#N/A	0	#N/A	0	#N/A	0	#N/A	
#N/A	0	#N/A	0	#N/A	0	#N/A	
#N/A	0	#N/A	0	#N/A	0	#N/A	
#N/A	0	#N/A	0	#N/A	0	#N/A	
LOST-6_art	1	LOST>6_art	1		0		
LOST-6_art	1	LOST>6_art	1		0		
	0		0	Ew/a-LOST-ar	1	Ew/p-LOST-ar	
	0		0	Ew/a-LOST-ar	1	Ew/p-LOST-ar	
	0		0	Ew/a-LOST-ar	1	Ew/p-LOST-ar	
	0		0	Ew/a-LOST-ar	1	Ew/p-LOST-ar	
#N/A	0	#N/A	0	#N/A	0	#N/A	
#N/A	0	#N/A	0	#N/A	0	#N/A	
#N/A	0	#N/A	0	#N/A	0	#N/A	
#N/A	0	#N/A	0	#N/A	0	#N/A	
#N/A	0	#N/A	0	#N/A	0	#N/A	
#N/A	0	#N/A	0	#N/A	0	#N/A	
#N/A	0	#N/A	0	#N/A	0	#N/A	
#N/A	0	#N/A	0	#N/A	0	#N/A	
#N/A	0	#N/A	0	#N/A	0	#N/A	
#N/A	0	#N/A	0	#N/A	0	#N/A	

ewplostart_b	ewaplostart	ewaplostart_l	nop_lostcit	lost_cit	lost_cit_b	lost3_cit	lost3_cit_b
0		0		#N/A	0	#N/A	0
0		0		#N/A	0	#N/A	0
0	#N/A	0		#N/A	0	#N/A	0
0	#N/A	0		#N/A	0	#N/A	0
0	Ew/a-p-LOST-	1			0	LOST-3_cit	1
0	Ew/a-p-LOST-	1			0	LOST-3_cit	1
0	Ew/a-p-LOST-	1			0	LOST-3_cit	1
0	Ew/a-p-LOST-	1			0	LOST-3_cit	1
0	Ew/a-p-LOST-	1		#N/A	0	#N/A	0
0	Ew/a-p-LOST-	1		#N/A	0	#N/A	0
0	#N/A	0		#N/A	0	#N/A	0
0	#N/A	0		#N/A	0	#N/A	0
0	#N/A	0		#N/A	0	#N/A	0
0		0			0		0
0	#N/A	0		#N/A	0	#N/A	0
0	#N/A	0		#N/A	0	#N/A	0
0	#N/A	0		#N/A	0	#N/A	0
0	#N/A	0		#N/A	0	#N/A	0
1	Ew/a-p-LOST-	1		#N/A	0	#N/A	0
1	Ew/a-p-LOST-	1		#N/A	0	#N/A	0
0	#N/A	0		#N/A	0	#N/A	0
0	#N/A	0		#N/A	0	#N/A	0
0	#N/A	0		#N/A	0	#N/A	0
0	#N/A	0		#N/A	0	#N/A	0
0	#N/A	0		#N/A	0	#N/A	0
0	#N/A	0		#N/A	0	#N/A	0
0	#N/A	0		#N/A	0	#N/A	0
0	#N/A	0		#N/A	0	#N/A	0
0	#N/A	0		#N/A	0	#N/A	0
1	Ew/a-p-LOST-	1		#N/A	0	#N/A	0
0		0		#N/A	0	#N/A	0
0		0			0		0
0	#N/A	0		#N/A	0	#N/A	0
0	#N/A	0		#N/A	0	#N/A	0
0	#N/A	0		#N/A	0	#N/A	0
0	#N/A	0			0	LOST-3_cit	1
0	#N/A	0			0	LOST-3_cit	1
0	#N/A	0		#N/A	0	#N/A	0
0	#N/A	0		#N/A	0	#N/A	0
0	#N/A	0		#N/A	0	#N/A	0
0	#N/A	0		#N/A	0	#N/A	0
0	#N/A	0		#N/A	0	#N/A	0
0	#N/A	0		#N/A	0	#N/A	0
0	#N/A	0		#N/A	0	#N/A	0
0	#N/A	0		#N/A	0	#N/A	0
0	#N/A	0		#N/A	0	#N/A	0

0	#N/A	0	#N/A	0	#N/A	0
0	#N/A	0		0		0
0	#N/A	0	#N/A	0	#N/A	0
0	#N/A	0	#N/A	0	#N/A	0
0	#N/A	0	#N/A	0	#N/A	0
0	#N/A	0	#N/A	0	#N/A	0
0	#N/A	0	#N/A	0	#N/A	0
1	Ew/a-p-LOST-	1		0		0
1	Ew/a-p-LOST-	1	LOST_cit	1	LOST-3_cit	1
0	#N/A	0	#N/A	0	#N/A	0
0	#N/A	0	#N/A	0	#N/A	0
0	#N/A	0	#N/A	0	#N/A	0
1	Ew/a-p-LOST-	1		0		0
0	#N/A	0	#N/A	0	#N/A	0
0	#N/A	0	#N/A	0	#N/A	0
0	#N/A	0	#N/A	0	#N/A	0
0	#N/A	0	#N/A	0	#N/A	0
0	#N/A	0	#N/A	0	#N/A	0
0	#N/A	0	#N/A	0	#N/A	0
0	#N/A	0	#N/A	0	#N/A	0
0	Ew/a-p-LOST-	1	#N/A	0	#N/A	0
0	Ew/a-p-LOST-	1	#N/A	0	#N/A	0
0	#N/A	0	#N/A	0	#N/A	0
0		0	#N/A	0	#N/A	0
0	#N/A	0	#N/A	0	#N/A	0
0	#N/A	0	#N/A	0	#N/A	0
0		0	#N/A	0	#N/A	0
0	#N/A	0	#N/A	0	#N/A	0
0	#N/A	0	#N/A	0	#N/A	0
1	Ew/a-p-LOST-	1	#N/A	0	#N/A	0
1	Ew/a-p-LOST-	1	#N/A	0	#N/A	0
1	Ew/a-p-LOST-	1	#N/A	0	#N/A	0
1	Ew/a-p-LOST-	1	#N/A	0	#N/A	0
1	Ew/a-p-LOST-	1	#N/A	0	#N/A	0
0	#N/A	0	#N/A	0	#N/A	0
0	#N/A	0	#N/A	0	#N/A	0
0	#N/A	0	#N/A	0	#N/A	0
0	#N/A	0	#N/A	0	#N/A	0
0	#N/A	0		0		0
0	#N/A	0	#N/A	0	#N/A	0
0	#N/A	0	#N/A	0	#N/A	0
0	#N/A	0	#N/A	0	#N/A	0
0	#N/A	0	#N/A	0	#N/A	0
0	#N/A	0	#N/A	0	#N/A	0
0	#N/A	0	#N/A	0	#N/A	0
0	#N/A	0	#N/A	0	#N/A	0
0	#N/A	0		0	LOST-3_cit	1

0	#N/A	0		0	LOST-3_cit	1
0	#N/A	0	#N/A	0	#N/A	0
0	#N/A	0	#N/A	0	#N/A	0
0	#N/A	0	#N/A	0	#N/A	0
0	#N/A	0	#N/A	0	#N/A	0
0	#N/A	0	#N/A	0	#N/A	0
1	Ew/a-p-LOST-	1	LOST_cit	1	LOST-3_cit	1
1	Ew/a-p-LOST-	1	LOST_cit	1	LOST-3_cit	1
0	#N/A	0	#N/A	0	#N/A	0
0	#N/A	0	#N/A	0	#N/A	0
0	#N/A	0	#N/A	0	#N/A	0
0	#N/A	0	#N/A	0	#N/A	0
0	#N/A	0	#N/A	0	#N/A	0
0	#N/A	0	#N/A	0	#N/A	0
0	#N/A	0	#N/A	0	#N/A	0
0	#N/A	0	#N/A	0	#N/A	0
0	#N/A	0	#N/A	0	#N/A	0
0	#N/A	0	#N/A	0	#N/A	0
0	#N/A	0	#N/A	0	#N/A	0
0	#N/A	0	#N/A	0	#N/A	0
0	#N/A	0	LOST_cit	1	LOST-3_cit	1
0	#N/A	0	#N/A	0	#N/A	0
0	#N/A	0	#N/A	0	#N/A	0
0	#N/A	0	#N/A	0	#N/A	0
0	#N/A	0	#N/A	0	#N/A	0
0	#N/A	0	#N/A	0	#N/A	0
0	#N/A	0	#N/A	0	#N/A	0
0		0		0	LOST-3_cit	1
0		0		0	LOST-3_cit	1
1	Ew/a-p-LOST-	1	LOST_cit	1	LOST-3_cit	1
1	Ew/a-p-LOST-	1	LOST_cit	1	LOST-3_cit	1
0	#N/A	0	#N/A	0	#N/A	0
0	#N/A	0	LOST_cit	1	LOST-3_cit	1
0	#N/A	0	LOST_cit	1	LOST-3_cit	1
0	#N/A	0		0	LOST-3_cit	1
0	Ew/a-p-LOST-	1	#N/A	0	#N/A	0
0	Ew/a-p-LOST-	1	#N/A	0	#N/A	0
1	Ew/a-p-LOST-	1	#N/A	0	#N/A	0
1	Ew/a-p-LOST-	1	#N/A	0	#N/A	0
0	#N/A	0	LOST_cit	1	LOST-3_cit	1
0	#N/A	0	#N/A	0	#N/A	0
0	#N/A	0	#N/A	0	#N/A	0
0		0	#N/A	0	#N/A	0
0	#N/A	0	#N/A	0	#N/A	0
0		0		0		0
0		0		0		0
0	#N/A	0	#N/A	0	#N/A	0

0	#N/A	0	#N/A	0	#N/A	0
0	#N/A	0	#N/A	0	#N/A	0
0	Ew/a-p-LOST-	1	#N/A	0	#N/A	0
0	Ew/a-p-LOST-	1	#N/A	0	#N/A	0
0	Ew/a-p-LOST-	1	#N/A	0	#N/A	0
0	#N/A	0	#N/A	0	#N/A	0
0	#N/A	0	#N/A	0	#N/A	0
0	#N/A	0	#N/A	0	#N/A	0
0	#N/A	0	#N/A	0	#N/A	0
0	#N/A	0	#N/A	0	#N/A	0
0	#N/A	0	#N/A	0	#N/A	0
0	#N/A	0		0	LOST-3_cit	1
0	#N/A	0		0	LOST-3_cit	1
1	Ew/a-p-LOST-	1	LOST_cit	1	LOST-3_cit	1
0	#N/A	0	#N/A	0	#N/A	0
0	#N/A	0	#N/A	0	#N/A	0
1	Ew/a-p-LOST-	1	LOST_cit	1	LOST-3_cit	1
0	#N/A	0	#N/A	0	#N/A	0
0	#N/A	0	#N/A	0	#N/A	0
0	#N/A	0	#N/A	0	#N/A	0
0	#N/A	0	#N/A	0	#N/A	0
0	#N/A	0	#N/A	0	#N/A	0
1	Ew/a-p-LOST-	1		0		0
0	#N/A	0	#N/A	0	#N/A	0
0	#N/A	0	#N/A	0	#N/A	0
0	#N/A	0	#N/A	0	#N/A	0
0	#N/A	0	#N/A	0	#N/A	0
0	#N/A	0	#N/A	0	#N/A	0
0		0	#N/A	0	#N/A	0
0	#N/A	0	LOST_cit	1	LOST-3_cit	1
0		0	#N/A	0	#N/A	0
0		0	#N/A	0	#N/A	0
0	#N/A	0	LOST_cit	1	LOST-3_cit	1
0	#N/A	0	#N/A	0	#N/A	0
0	#N/A	0	#N/A	0	#N/A	0
0	#N/A	0	#N/A	0	#N/A	0
0	#N/A	0	#N/A	0	#N/A	0
0	#N/A	0	#N/A	0	#N/A	0
0	#N/A	0	LOST_cit	1	LOST-3_cit	1
0	#N/A	0	#N/A	0	#N/A	0
0	#N/A	0	#N/A	0	#N/A	0
1	Ew/a-p-LOST-	1	#N/A	0	#N/A	0
1	Ew/a-p-LOST-	1	#N/A	0	#N/A	0
0	#N/A	0	#N/A	0	#N/A	0
0	#N/A	0	#N/A	0	#N/A	0
0	#N/A	0	#N/A	0	#N/A	0
0	#N/A	0	#N/A	0	#N/A	0

0	#N/A	0	#N/A	0	#N/A	0
0	#N/A	0	#N/A	0	#N/A	0
0	#N/A	0	#N/A	0	#N/A	0
0	#N/A	0	#N/A	0	#N/A	0
0	#N/A	0	LOST_cit	1	LOST-3_cit	1
0	#N/A	0	LOST_cit	1	LOST-3_cit	1
0	#N/A	0	#N/A	0	#N/A	0
0	#N/A	0	#N/A	0	#N/A	0
0	#N/A	0	#N/A	0	#N/A	0
0	#N/A	0	#N/A	0	#N/A	0
0	#N/A	0	#N/A	0	#N/A	0
0	#N/A	0	#N/A	0	#N/A	0
0	#N/A	0	#N/A	0	#N/A	0
0	#N/A	0	#N/A	0	#N/A	0
0	#N/A	0	#N/A	0	#N/A	0
0		0		0	LOST-3_cit	1
0		0		0	LOST-3_cit	1
0	#N/A	0	#N/A	0	#N/A	0
0	#N/A	0	#N/A	0	#N/A	0
0	#N/A	0	#N/A	0	#N/A	0
0	#N/A	0	#N/A	0	#N/A	0
0	#N/A	0	#N/A	0	#N/A	0
0	#N/A	0	#N/A	0	#N/A	0
0	#N/A	0	#N/A	0	#N/A	0
0	Ew/a-p-LOST-	1	LOST_cit	1	LOST-3_cit	1
0	Ew/a-p-LOST-	1	LOST_cit	1	LOST-3_cit	1
0	#N/A	0	#N/A	0	#N/A	0
0	#N/A	0	#N/A	0	#N/A	0
0	#N/A	0	#N/A	0	#N/A	0
0	#N/A	0	#N/A	0	#N/A	0
0	#N/A	0	#N/A	0	#N/A	0
0	#N/A	0	#N/A	0	#N/A	0
0	#N/A	0	#N/A	0	#N/A	0
0	#N/A	0	#N/A	0	#N/A	0
0		0		0		0
0	#N/A	0	#N/A	0	#N/A	0
0	#N/A	0	#N/A	0	#N/A	0
0		0	#N/A	0	#N/A	0
1	Ew/a-p-LOST-	1	LOST_cit	1	LOST-3_cit	1
1	Ew/a-p-LOST-	1	LOST_cit	1	LOST-3_cit	1
1	Ew/a-p-LOST-	1	LOST_cit	1	LOST-3_cit	1
1	Ew/a-p-LOST-	1	LOST_cit	1	LOST-3_cit	1
0	#N/A	0	#N/A	0	#N/A	0
0	#N/A	0		0	LOST-3_cit	1
0	#N/A	0	#N/A	0	#N/A	0
0	#N/A	0	#N/A	0	#N/A	0
0	#N/A	0	#N/A	0	#N/A	0

0	#N/A	0	#N/A	0	#N/A	0
0	#N/A	0	#N/A	0	#N/A	0
0	#N/A	0	#N/A	0	#N/A	0
0	#N/A	0	#N/A	0	#N/A	0
0	#N/A	0	#N/A	0	#N/A	0
0	#N/A	0	#N/A	0	#N/A	0
0	#N/A	0	#N/A	0	#N/A	0
0	#N/A	0	#N/A	0	#N/A	0
0	#N/A	0	#N/A	0	#N/A	0
1	Ew/a-p-LOST-	1	#N/A	0	#N/A	0
0		0	#N/A	0	#N/A	0
0	#N/A	0	#N/A	0	#N/A	0
0	#N/A	0	#N/A	0	#N/A	0
0	#N/A	0	#N/A	0	#N/A	0
0	#N/A	0	#N/A	0	#N/A	0
0		0	#N/A	0	#N/A	0
0	#N/A	0	#N/A	0	#N/A	0
0	#N/A	0	#N/A	0	#N/A	0
0	#N/A	0	#N/A	0	#N/A	0
0	#N/A	0	#N/A	0	#N/A	0
0	#N/A	0	#N/A	0	#N/A	0
1	Ew/a-p-LOST-	1	LOST_cit	1	LOST-3_cit	1
1	Ew/a-p-LOST-	1	LOST_cit	1	LOST-3_cit	1
1	Ew/a-p-LOST-	1	LOST_cit	1	LOST-3_cit	1
0	#N/A	0	#N/A	0	#N/A	0
0	#N/A	0	#N/A	0	#N/A	0
0	#N/A	0	#N/A	0	#N/A	0
0	#N/A	0	#N/A	0	#N/A	0
0	#N/A	0	#N/A	0	#N/A	0
0	#N/A	0	#N/A	0	#N/A	0
1	Ew/a-p-LOST-	1	LOST_cit	1	LOST-3_cit	1
1	Ew/a-p-LOST-	1	LOST_cit	1	LOST-3_cit	1
1	Ew/a-p-LOST-	1	LOST_cit	1	LOST-3_cit	1
0	#N/A	0	#N/A	0	#N/A	0
0	#N/A	0	LOST_cit	1	LOST-3_cit	1
0	#N/A	0	#N/A	0	#N/A	0
0	#N/A	0	#N/A	0	#N/A	0
0	#N/A	0	#N/A	0	#N/A	0
0	#N/A	0	#N/A	0	#N/A	0
0	#N/A	0	#N/A	0	#N/A	0
0	#N/A	0	#N/A	0	#N/A	0
0	#N/A	0	#N/A	0	#N/A	0
0	#N/A	0	#N/A	0	#N/A	0
0		0	#N/A	0	#N/A	0
0	#N/A	0		0		0
0	#N/A	0	#N/A	0	#N/A	0
0	#N/A	0	#N/A	0	#N/A	0

0	#N/A	0	#N/A	0	#N/A	0
0	#N/A	0	#N/A	0	#N/A	0
0	#N/A	0	LOST_cit	1	LOST-3_cit	1
0		0		0		0
0	#N/A	0	#N/A	0	#N/A	0
0	#N/A	0	#N/A	0	#N/A	0
0	#N/A	0		0		0
0	#N/A	0	#N/A	0	#N/A	0
0	#N/A	0	#N/A	0	#N/A	0
0	#N/A	0	#N/A	0	#N/A	0
0	#N/A	0	#N/A	0	#N/A	0
0	#N/A	0	#N/A	0	#N/A	0
0	#N/A	0	#N/A	0	#N/A	0
0	#N/A	0	#N/A	0	#N/A	0
0	#N/A	0	#N/A	0	#N/A	0
0		0	#N/A	0	#N/A	0
0		0	#N/A	0	#N/A	0
0	#N/A	0	#N/A	0	#N/A	0
0	#N/A	0	#N/A	0	#N/A	0
0	#N/A	0	#N/A	0	#N/A	0
0	#N/A	0	#N/A	0	#N/A	0
0	#N/A	0	#N/A	0	#N/A	0
0		0	#N/A	0	#N/A	0
0	#N/A	0	#N/A	0	#N/A	0
0	#N/A	0	#N/A	0	#N/A	0
0	#N/A	0	#N/A	0	#N/A	0
0	#N/A	0	#N/A	0	#N/A	0
0	#N/A	0	#N/A	0	#N/A	0
0	#N/A	0	#N/A	0	#N/A	0
0	#N/A	0	#N/A	0	#N/A	0
0	#N/A	0	#N/A	0	#N/A	0
0	#N/A	0	#N/A	0	#N/A	0
0	#N/A	0	#N/A	0	#N/A	0
0	#N/A	0	#N/A	0	#N/A	0
0		0		0		0
0		0		0		0
0	#N/A	0	#N/A	0	#N/A	0
0		0	#N/A	0	#N/A	0
0	#N/A	0	#N/A	0	#N/A	0
0	#N/A	0	#N/A	0	#N/A	0
0	#N/A	0	#N/A	0	#N/A	0
0	#N/A	0	#N/A	0	#N/A	0
0	#N/A	0	#N/A	0	#N/A	0
0	#N/A	0		0	LOST-3_cit	1
0	#N/A	0		0	LOST-3_cit	1
0	#N/A	0	#N/A	0	#N/A	0
0	#N/A	0	#N/A	0	#N/A	0

0	#N/A	0	#N/A	0	#N/A	0
0	Ew/a-p-LOST-	1	#N/A	0	#N/A	0
0	Ew/a-p-LOST-	1	#N/A	0	#N/A	0
0	#N/A	0	#N/A	0	#N/A	0
0	#N/A	0	#N/A	0	#N/A	0
0	#N/A	0	#N/A	0	#N/A	0
0	#N/A	0	#N/A	0	#N/A	0
0	#N/A	0	#N/A	0	#N/A	0
0	#N/A	0	#N/A	0	#N/A	0
0	#N/A	0	#N/A	0	#N/A	0
0	#N/A	0	#N/A	0	#N/A	0
0	#N/A	0	#N/A	0	#N/A	0
0	#N/A	0	#N/A	0	#N/A	0
0		0		0	LOST-3_cit	1
1	Ew/a-p-LOST-	1	#N/A	0	#N/A	0
1	Ew/a-p-LOST-	1	#N/A	0	#N/A	0
0		0	#N/A	0	#N/A	0
0	#N/A	0	#N/A	0	#N/A	0
0		0	#N/A	0	#N/A	0
0	#N/A	0	#N/A	0	#N/A	0
0	#N/A	0	#N/A	0	#N/A	0
0		0	#N/A	0	#N/A	0
0	#N/A	0	#N/A	0	#N/A	0
0	#N/A	0	#N/A	0	#N/A	0
0	#N/A	0	LOST_cit	1	LOST-3_cit	1
0	#N/A	0	LOST_cit	1	LOST-3_cit	1
1	Ew/a-p-LOST-	1	#N/A	0	#N/A	0
0	#N/A	0	#N/A	0	#N/A	0
0	#N/A	0	#N/A	0	#N/A	0
0	#N/A	0	#N/A	0	#N/A	0
0	#N/A	0	#N/A	0	#N/A	0
0	#N/A	0	#N/A	0	#N/A	0
0	#N/A	0	#N/A	0	#N/A	0
0	#N/A	0	#N/A	0	#N/A	0
0	#N/A	0	#N/A	0	#N/A	0
0	#N/A	0	#N/A	0	#N/A	0
0		0	#N/A	0	#N/A	0
0	#N/A	0	#N/A	0	#N/A	0
0	#N/A	0	#N/A	0	#N/A	0
0	#N/A	0	#N/A	0	#N/A	0
0	#N/A	0	#N/A	0	#N/A	0
0	#N/A	0	#N/A	0	#N/A	0
0	#N/A	0	#N/A	0	#N/A	0
0		0	LOST_cit	1	LOST-3_cit	1
0	#N/A	0	LOST_cit	1	LOST-3_cit	1
0	#N/A	0	#N/A	0	#N/A	0
0	#N/A	0	#N/A	0	#N/A	0

0	#N/A	0	#N/A	0	#N/A	0
0		0	#N/A	0	#N/A	0
0	#N/A	0		0		0
0	#N/A	0		0		0
0	#N/A	0	#N/A	0	#N/A	0
0		0	#N/A	0	#N/A	0
0		0	#N/A	0	#N/A	0
1	Ew/a-p-LOST-	1	LOST_cit	1	LOST-3_cit	1
1	Ew/a-p-LOST-	1	LOST_cit	1	LOST-3_cit	1
0	#N/A	0	#N/A	0	#N/A	0
0	#N/A	0	#N/A	0	#N/A	0
0	#N/A	0	#N/A	0	#N/A	0
0	#N/A	0	#N/A	0	#N/A	0
0	#N/A	0	#N/A	0	#N/A	0
0	#N/A	0	#N/A	0	#N/A	0
0	#N/A	0	#N/A	0	#N/A	0
0	#N/A	0	#N/A	0	#N/A	0
0	#N/A	0	#N/A	0	#N/A	0
0	#N/A	0	#N/A	0	#N/A	0
0	#N/A	0	#N/A	0	#N/A	0
0	#N/A	0	#N/A	0	#N/A	0
1	Ew/a-p-LOST-	1	#N/A	0	#N/A	0
0	#N/A	0	LOST_cit	1	LOST-3_cit	1
0	#N/A	0	LOST_cit	1	LOST-3_cit	1
0	#N/A	0	#N/A	0	#N/A	0
0	#N/A	0	#N/A	0	#N/A	0
0	#N/A	0	#N/A	0	#N/A	0
0		0	#N/A	0	#N/A	0
0	#N/A	0	#N/A	0	#N/A	0
0	#N/A	0	#N/A	0	#N/A	0
0	#N/A	0	#N/A	0	#N/A	0
0	#N/A	0	#N/A	0	#N/A	0
0	#N/A	0	#N/A	0	#N/A	0
0	#N/A	0	#N/A	0	#N/A	0
0	#N/A	0	#N/A	0	#N/A	0
0	#N/A	0	#N/A	0	#N/A	0
0	#N/A	0	#N/A	0	#N/A	0
0		0		0		0
0	#N/A	0	#N/A	0	#N/A	0
0	#N/A	0	#N/A	0	#N/A	0
0	#N/A	0	#N/A	0	#N/A	0
0	#N/A	0	LOST_cit	1	LOST-3_cit	1
0	#N/A	0	#N/A	0	#N/A	0
0	#N/A	0	#N/A	0	#N/A	0
0	#N/A	0	#N/A	0	#N/A	0
0		0	#N/A	0	#N/A	0
0		0	#N/A	0	#N/A	0

0	#N/A	0	LOST_cit	1	LOST-3_cit	1
0	#N/A	0	#N/A	0	#N/A	0
0	#N/A	0	#N/A	0	#N/A	0
0	#N/A	0	#N/A	0	#N/A	0
0	#N/A	0	#N/A	0	#N/A	0
0		0		0		0
0		0		0		0
0	#N/A	0	#N/A	0	#N/A	0
0	#N/A	0	#N/A	0	#N/A	0
0	#N/A	0	#N/A	0	#N/A	0
0	#N/A	0	#N/A	0	#N/A	0
0		0		0	LOST-3_cit	1
0		0		0	LOST-3_cit	1
0	#N/A	0	#N/A	0	#N/A	0
0		0		0		0
1	Ew/a-p-LOST-	1	LOST_cit	1	LOST-3_cit	1
1	Ew/a-p-LOST-	1	LOST_cit	1	LOST-3_cit	1
1	Ew/a-p-LOST-	1	LOST_cit	1	LOST-3_cit	1
1	Ew/a-p-LOST-	1	LOST_cit	1	LOST-3_cit	1
0	#N/A	0	LOST_cit	1	LOST-3_cit	1
0	#N/A	0	LOST_cit	1	LOST-3_cit	1
0	#N/A	0	#N/A	0	#N/A	0
0	#N/A	0	#N/A	0	#N/A	0
0	#N/A	0	#N/A	0	#N/A	0
0		0	#N/A	0	#N/A	0
0		0	#N/A	0	#N/A	0
0	#N/A	0	#N/A	0	#N/A	0
0	#N/A	0	#N/A	0	#N/A	0
0		0	#N/A	0	#N/A	0
0	#N/A	0	LOST_cit	1	LOST-3_cit	1
0	#N/A	0	LOST_cit	1	LOST-3_cit	1
0	#N/A	0	#N/A	0	#N/A	0
0	#N/A	0	#N/A	0	#N/A	0
0	#N/A	0	#N/A	0	#N/A	0
0	#N/A	0	#N/A	0	#N/A	0
0	#N/A	0	#N/A	0	#N/A	0
0	#N/A	0	#N/A	0	#N/A	0
0	#N/A	0	#N/A	0	#N/A	0
0	#N/A	0	#N/A	0	#N/A	0
0	#N/A	0	#N/A	0	#N/A	0
0	#N/A	0	#N/A	0	#N/A	0
0	#N/A	0	#N/A	0	#N/A	0
0	#N/A	0	#N/A	0	#N/A	0
0	#N/A	0	LOST_cit	1	LOST-3_cit	1
0	#N/A	0	#N/A	0	#N/A	0
0	#N/A	0	#N/A	0	#N/A	0
0	#N/A	0	#N/A	0	#N/A	0

0		0		0		0
0		0		0		0
0	#N/A	0	#N/A	0	#N/A	0
0	#N/A	0	#N/A	0	#N/A	0
0	#N/A	0	#N/A	0	#N/A	0
0	#N/A	0	#N/A	0	#N/A	0
0		0	#N/A	0	#N/A	0
0	#N/A	0	#N/A	0	#N/A	0
0	#N/A	0	#N/A	0	#N/A	0
1	Ew/a-p-LOST-	1	#N/A	0	#N/A	0
0	#N/A	0	#N/A	0	#N/A	0
0	#N/A	0	#N/A	0	#N/A	0
0	#N/A	0	#N/A	0	#N/A	0
0	#N/A	0	#N/A	0	#N/A	0
0	#N/A	0	#N/A	0	#N/A	0
0	#N/A	0	#N/A	0	#N/A	0
0	#N/A	0	#N/A	0	#N/A	0
0	#N/A	0	#N/A	0	#N/A	0
0	#N/A	0	#N/A	0	#N/A	0
0	#N/A	0	#N/A	0	#N/A	0
0	#N/A	0	#N/A	0	#N/A	0
0	#N/A	0	#N/A	0	#N/A	0
0		0	#N/A	0	#N/A	0
0		0	LOST_cit	1	LOST-3_cit	1
0		0	LOST_cit	1	LOST-3_cit	1
0		0	LOST_cit	1	LOST-3_cit	1
0	#N/A	0	#N/A	0	#N/A	0
0	#N/A	0	#N/A	0	#N/A	0
0	#N/A	0	#N/A	0	#N/A	0
0	#N/A	0	#N/A	0	#N/A	0
0	#N/A	0	#N/A	0	#N/A	0
0	#N/A	0	#N/A	0	#N/A	0
0	#N/A	0	#N/A	0	#N/A	0
0	#N/A	0	#N/A	0	#N/A	0
1	Ew/a-p-LOST-	1	#N/A	0	#N/A	0
0	#N/A	0	#N/A	0	#N/A	0
0	#N/A	0	LOST_cit	1	LOST-3_cit	1
0	#N/A	0	LOST_cit	1	LOST-3_cit	1
0	#N/A	0	#N/A	0	#N/A	0
0	#N/A	0	#N/A	0	#N/A	0
0	#N/A	0	#N/A	0	#N/A	0
0		0	#N/A	0	#N/A	0
0	#N/A	0		0	LOST-3_cit	1
1	Ew/a-p-LOST-	1	#N/A	0	#N/A	0
0	#N/A	0	#N/A	0	#N/A	0
0	#N/A	0	#N/A	0	#N/A	0
0	#N/A	0	#N/A	0	#N/A	0

0	#N/A	0	#N/A	0	#N/A	0
0	Ew/a-p-LOST-	1	#N/A	0	#N/A	0
0	#N/A	0		0		0
0	#N/A	0	#N/A	0	#N/A	0
0	#N/A	0	#N/A	0	#N/A	0
0	#N/A	0	#N/A	0	#N/A	0
0	#N/A	0	#N/A	0	#N/A	0
0	#N/A	0	#N/A	0	#N/A	0
0	#N/A	0	#N/A	0	#N/A	0
0	#N/A	0	#N/A	0	#N/A	0
0		0	LOST_cit	1	LOST-3_cit	1
0		0	LOST_cit	1	LOST-3_cit	1
0		0	LOST_cit	1	LOST-3_cit	1
0	#N/A	0	#N/A	0	#N/A	0
0	#N/A	0	#N/A	0	#N/A	0
0	#N/A	0	#N/A	0	#N/A	0
0	#N/A	0	#N/A	0	#N/A	0
0	#N/A	0	#N/A	0	#N/A	0
0	#N/A	0	#N/A	0	#N/A	0
0		0	#N/A	0	#N/A	0
0	#N/A	0	#N/A	0	#N/A	0
0	#N/A	0	#N/A	0	#N/A	0
0	#N/A	0	#N/A	0	#N/A	0
0	#N/A	0	#N/A	0	#N/A	0
0	#N/A	0	#N/A	0	#N/A	0
0	#N/A	0	#N/A	0	#N/A	0
0	#N/A	0	#N/A	0	#N/A	0
0	#N/A	0	#N/A	0	#N/A	0
0	#N/A	0	#N/A	0	#N/A	0
0	#N/A	0	#N/A	0	#N/A	0
0	#N/A	0	#N/A	0	#N/A	0
0	#N/A	0	#N/A	0	#N/A	0
0	#N/A	0	#N/A	0	#N/A	0
0	#N/A	0	#N/A	0	#N/A	0
0	#N/A	0	#N/A	0	#N/A	0
0	#N/A	0	#N/A	0	#N/A	0
0		0	#N/A	0	#N/A	0
0	#N/A	0	#N/A	0	#N/A	0
0	#N/A	0	#N/A	0	#N/A	0
1	Ew/a-p-LOST-	1		0		0
0	#N/A	0	#N/A	0	#N/A	0
0	#N/A	0	#N/A	0	#N/A	0
0	#N/A	0	#N/A	0	#N/A	0
0	#N/A	0	#N/A	0	#N/A	0
0	#N/A	0	#N/A	0	#N/A	0
0	#N/A	0	#N/A	0	#N/A	0
0	#N/A	0	#N/A	0	#N/A	0

0	#N/A	0	#N/A	0	#N/A	0
0	#N/A	0	#N/A	0	#N/A	0
0	#N/A	0	#N/A	0	#N/A	0
0	#N/A	0	#N/A	0	#N/A	0
0	#N/A	0	#N/A	0	#N/A	0
0	#N/A	0	#N/A	0	#N/A	0
0	#N/A	0	#N/A	0	#N/A	0
0	#N/A	0	#N/A	0	#N/A	0
0	#N/A	0	#N/A	0	#N/A	0
0	#N/A	0	#N/A	0	#N/A	0
0	#N/A	0	#N/A	0	#N/A	0
0	#N/A	0	#N/A	0	#N/A	0
0	#N/A	0	#N/A	0	#N/A	0
0	#N/A	0	#N/A	0	#N/A	0
0	#N/A	0	#N/A	0	#N/A	0
0	#N/A	0	#N/A	0	#N/A	0
0	#N/A	0	#N/A	0	#N/A	0
0	#N/A	0	#N/A	0	#N/A	0
0	#N/A	0	#N/A	0	#N/A	0
0	#N/A	0	#N/A	0	#N/A	0
0	#N/A	0	#N/A	0	#N/A	0
0	#N/A	0	#N/A	0	#N/A	0
0	#N/A	0	#N/A	0	#N/A	0
0		0	#N/A	0	#N/A	0
0	#N/A	0	#N/A	0	#N/A	0
0	#N/A	0	#N/A	0	#N/A	0
0	#N/A	0	#N/A	0	#N/A	0
0	#N/A	0	#N/A	0	#N/A	0
0	#N/A	0	#N/A	0	#N/A	0
1	Ew/a-p-LOST-	1	#N/A	0	#N/A	0
0	#N/A	0	#N/A	0	#N/A	0
0	#N/A	0	#N/A	0	#N/A	0
0	#N/A	0	#N/A	0	#N/A	0
0	#N/A	0	#N/A	0	#N/A	0
0	#N/A	0	#N/A	0	#N/A	0
0	#N/A	0	#N/A	0	#N/A	0
0	#N/A	0	#N/A	0	#N/A	0
0	#N/A	0	#N/A	0	#N/A	0
0	#N/A	0	#N/A	0	#N/A	0
0		0	#N/A	0	#N/A	0
0	#N/A	0	#N/A	0	#N/A	0
0	#N/A	0	#N/A	0	#N/A	0
0	#N/A	0		0		0
0	#N/A	0		0		0
0	#N/A	0	#N/A	0	#N/A	0
0	#N/A	0		0		0
0	#N/A	0	#N/A	0	#N/A	0

0		0	#N/A	0	#N/A	0
0		0	#N/A	0	#N/A	0
1	Ew/a-p-LOST-	1	#N/A	0	#N/A	0
1	Ew/a-p-LOST-	1	#N/A	0	#N/A	0
0	#N/A	0	#N/A	0	#N/A	0
0	#N/A	0	#N/A	0	#N/A	0
1	Ew/a-p-LOST-	1	LOST_cit	1	LOST-3_cit	1
1	Ew/a-p-LOST-	1	LOST_cit	1	LOST-3_cit	1
1	Ew/a-p-LOST-	1	LOST_cit	1	LOST-3_cit	1
1	Ew/a-p-LOST-	1	LOST_cit	1	LOST-3_cit	1
0	#N/A	0	#N/A	0	#N/A	0
0	#N/A	0	#N/A	0	#N/A	0
0	#N/A	0	#N/A	0	#N/A	0
0	#N/A	0	#N/A	0	#N/A	0
0	#N/A	0	#N/A	0	#N/A	0
0	#N/A	0	#N/A	0	#N/A	0
0	#N/A	0	#N/A	0	#N/A	0
1	Ew/a-p-LOST-	1		0	LOST-3_cit	1
1	Ew/a-p-LOST-	1		0	LOST-3_cit	1
0	#N/A	0	LOST_cit	1	LOST-3_cit	1
0	#N/A	0	LOST_cit	1	LOST-3_cit	1
0	#N/A	0	#N/A	0	#N/A	0
0	#N/A	0	#N/A	0	#N/A	0
0	#N/A	0		0		0
0	#N/A	0	#N/A	0	#N/A	0
0	#N/A	0	#N/A	0	#N/A	0
0	#N/A	0	#N/A	0	#N/A	0
0	#N/A	0	#N/A	0	#N/A	0
0	#N/A	0	#N/A	0	#N/A	0
0	#N/A	0	#N/A	0	#N/A	0
0	#N/A	0	#N/A	0	#N/A	0
0	#N/A	0		0	LOST-3_cit	1
0	#N/A	0		0	LOST-3_cit	1
0	#N/A	0	#N/A	0	#N/A	0
0	#N/A	0	#N/A	0	#N/A	0
0	#N/A	0	#N/A	0	#N/A	0
0	#N/A	0	LOST_cit	1	LOST-3_cit	1
0	#N/A	0	LOST_cit	1	LOST-3_cit	1
0	#N/A	0	#N/A	0	#N/A	0
0	#N/A	0	#N/A	0	#N/A	0
0	#N/A	0	#N/A	0	#N/A	0
0	#N/A	0	#N/A	0	#N/A	0
0	#N/A	0	#N/A	0	#N/A	0
0	#N/A	0	#N/A	0	#N/A	0
0	#N/A	0	#N/A	0	#N/A	0
0	#N/A	0	#N/A	0	#N/A	0
0	#N/A	0	#N/A	0	#N/A	0

0	#N/A	0	#N/A	0	#N/A	0
0	#N/A	0	#N/A	0	#N/A	0
0	Ew/a-p-LOST-	1		0		0
0	Ew/a-p-LOST-	1		0		0
0	#N/A	0	#N/A	0	#N/A	0
0	#N/A	0	#N/A	0	#N/A	0
0	#N/A	0	#N/A	0	#N/A	0
0	#N/A	0	#N/A	0	#N/A	0
0		0	#N/A	0	#N/A	0
0		0	#N/A	0	#N/A	0
1	Ew/a-p-LOST-	1	#N/A	0	#N/A	0
1	Ew/a-p-LOST-	1	#N/A	0	#N/A	0
0	#N/A	0	#N/A	0	#N/A	0
0	#N/A	0	#N/A	0	#N/A	0
0	#N/A	0	#N/A	0	#N/A	0
0	#N/A	0		0		0
0		0	LOST_cit	1	LOST-3_cit	1
0		0	LOST_cit	1	LOST-3_cit	1
1	Ew/a-p-LOST-	1	LOST_cit	1	LOST-3_cit	1
0	#N/A	0	LOST_cit	1	LOST-3_cit	1
0		0	LOST_cit	1	LOST-3_cit	1
0		0	LOST_cit	1	LOST-3_cit	1
0	#N/A	0	#N/A	0	#N/A	0
0	#N/A	0	#N/A	0	#N/A	0
0	#N/A	0	#N/A	0	#N/A	0
0	#N/A	0	#N/A	0	#N/A	0
0	#N/A	0	#N/A	0	#N/A	0
0	#N/A	0	#N/A	0	#N/A	0
0	#N/A	0	#N/A	0	#N/A	0
0	#N/A	0	#N/A	0	#N/A	0
0		0	LOST_cit	1	LOST-3_cit	1
0		0	LOST_cit	1	LOST-3_cit	1
0	#N/A	0	#N/A	0	#N/A	0
1	Ew/a-p-LOST-	1	LOST_cit	1	LOST-3_cit	1
1	Ew/a-p-LOST-	1	LOST_cit	1	LOST-3_cit	1
1	Ew/a-p-LOST-	1	LOST_cit	1	LOST-3_cit	1
1	Ew/a-p-LOST-	1	LOST_cit	1	LOST-3_cit	1
0		0	#N/A	0	#N/A	0
0		0	#N/A	0	#N/A	0
0	#N/A	0	LOST_cit	1	LOST-3_cit	1
0	#N/A	0	#N/A	0	#N/A	0
0		0		0	LOST-3_cit	1
0		0	LOST_cit	1	LOST-3_cit	1
0		0	LOST_cit	1	LOST-3_cit	1
0	#N/A	0	#N/A	0	#N/A	0
0	#N/A	0	#N/A	0	#N/A	0
0	#N/A	0	#N/A	0	#N/A	0

0	#N/A	0	#N/A	0	#N/A	0
0	#N/A	0	#N/A	0	#N/A	0
1	Ew/a-p-LOST-	1	#N/A	0	#N/A	0
1	Ew/a-p-LOST-	1	#N/A	0	#N/A	0
0	#N/A	0	#N/A	0	#N/A	0
1	Ew/a-p-LOST-	1	#N/A	0	#N/A	0
1	Ew/a-p-LOST-	1	#N/A	0	#N/A	0
0	#N/A	0	#N/A	0	#N/A	0
1	Ew/a-p-LOST-	1	LOST_cit	1	LOST-3_cit	1
0	#N/A	0	#N/A	0	#N/A	0
0		0	#N/A	0	#N/A	0
0		0	#N/A	0	#N/A	0
0		0	#N/A	0	#N/A	0
0		0	#N/A	0	#N/A	0
0		0	#N/A	0	#N/A	0
0	#N/A	0	#N/A	0	#N/A	0
0	#N/A	0	#N/A	0	#N/A	0
0	#N/A	0	#N/A	0	#N/A	0
0	#N/A	0	#N/A	0	#N/A	0
0	#N/A	0	#N/A	0	#N/A	0
0	#N/A	0	#N/A	0	#N/A	0
1	Ew/a-p-LOST-	1	LOST_cit	1	LOST-3_cit	1
1	Ew/a-p-LOST-	1	LOST_cit	1	LOST-3_cit	1
0		0	LOST_cit	1	LOST-3_cit	1
0	#N/A	0	#N/A	0	#N/A	0
0	#N/A	0	#N/A	0	#N/A	0
0	#N/A	0	#N/A	0	#N/A	0
0		0	#N/A	0	#N/A	0
0	#N/A	0	#N/A	0	#N/A	0
1	Ew/a-p-LOST-	1		0		0
1	Ew/a-p-LOST-	1		0		0
0	#N/A	0	#N/A	0	#N/A	0
0	#N/A	0	#N/A	0	#N/A	0
0	#N/A	0	LOST_cit	1	LOST-3_cit	1
0	#N/A	0	LOST_cit	1	LOST-3_cit	1
0		0	#N/A	0	#N/A	0
0	#N/A	0	#N/A	0	#N/A	0
0		0		0		0
0	#N/A	0	LOST_cit	1	LOST-3_cit	1
0	#N/A	0	LOST_cit	1	LOST-3_cit	1
0		0	#N/A	0	#N/A	0
1	Ew/a-p-LOST-	1		0		0
0	#N/A	0	#N/A	0	#N/A	0
0	#N/A	0	#N/A	0	#N/A	0
0	#N/A	0	#N/A	0	#N/A	0
0	#N/A	0	#N/A	0	#N/A	0
0	#N/A	0	#N/A	0	#N/A	0

0		0		0		0
0		0	#N/A	0	#N/A	0
0		0	#N/A	0	#N/A	0
0	#N/A	0	#N/A	0	#N/A	0
0		0	#N/A	0	#N/A	0
0		0	#N/A	0	#N/A	0
0		0	#N/A	0	#N/A	0
0		0		0	LOST-3_cit	1
0		0		0	LOST-3_cit	1
0	#N/A	0		0	LOST-3_cit	1
0	#N/A	0		0	LOST-3_cit	1
0		0	#N/A	0	#N/A	0
0		0	#N/A	0	#N/A	0
0	#N/A	0		0		0
0		0		0		0
0	#N/A	0		0		0
0	#N/A	0		0		0
0	#N/A	0	#N/A	0	#N/A	0
0	#N/A	0	#N/A	0	#N/A	0
0	#N/A	0	#N/A	0	#N/A	0
0	#N/A	0	#N/A	0	#N/A	0
0	#N/A	0	#N/A	0	#N/A	0
0	#N/A	0	#N/A	0	#N/A	0
0	#N/A	0	#N/A	0	#N/A	0
0	#N/A	0	LOST_cit	1	LOST-3_cit	1
0	#N/A	0	#N/A	0	#N/A	0
0	#N/A	0	#N/A	0	#N/A	0
0	#N/A	0	#N/A	0	#N/A	0
0	#N/A	0	#N/A	0	#N/A	0
0	#N/A	0	#N/A	0	#N/A	0
0	#N/A	0	#N/A	0	#N/A	0
0	#N/A	0	#N/A	0	#N/A	0
0	#N/A	0	#N/A	0	#N/A	0
0	#N/A	0	#N/A	0	#N/A	0
0	#N/A	0	#N/A	0	#N/A	0
0	#N/A	0	#N/A	0	#N/A	0
0	#N/A	0	#N/A	0	#N/A	0
0		0		0		0
0	#N/A	0	#N/A	0	#N/A	0
0		0		0		0
0		0		0		0
0	#N/A	0	#N/A	0	#N/A	0
0		0	#N/A	0	#N/A	0
0		0	#N/A	0	#N/A	0
0	#N/A	0	#N/A	0	#N/A	0
0	#N/A	0	#N/A	0	#N/A	0
0	#N/A	0	#N/A	0	#N/A	0

0	#N/A	0	#N/A	0	#N/A	0
0	#N/A	0	#N/A	0	#N/A	0
0	#N/A	0	#N/A	0	#N/A	0
0	#N/A	0	#N/A	0	#N/A	0
0	#N/A	0	#N/A	0	#N/A	0
0	#N/A	0	LOST_cit	1	LOST-3_cit	1
0	#N/A	0	LOST_cit	1	LOST-3_cit	1
0	#N/A	0	LOST_cit	1	LOST-3_cit	1
0	#N/A	0	#N/A	0	#N/A	0
0	#N/A	0	#N/A	0	#N/A	0
0	#N/A	0	#N/A	0	#N/A	0
0	#N/A	0	#N/A	0	#N/A	0
0	#N/A	0	#N/A	0	#N/A	0
0	#N/A	0	#N/A	0	#N/A	0
0	#N/A	0	#N/A	0	#N/A	0
0	#N/A	0	#N/A	0	#N/A	0
0	#N/A	0	#N/A	0	#N/A	0
0	Ew/a-p-LOST-	1	#N/A	0	#N/A	0
0	Ew/a-p-LOST-	1	#N/A	0	#N/A	0
0	Ew/a-p-LOST-	1	LOST_cit	1	LOST-3_cit	1
0	Ew/a-p-LOST-	1	LOST_cit	1	LOST-3_cit	1
0	Ew/a-p-LOST-	1	LOST_cit	1	LOST-3_cit	1
0	Ew/a-p-LOST-	1	LOST_cit	1	LOST-3_cit	1
0	Ew/a-p-LOST-	1	LOST_cit	1	LOST-3_cit	1
0	#N/A	0	#N/A	0	#N/A	0
0	#N/A	0	#N/A	0	#N/A	0
1	Ew/a-p-LOST-	1	LOST_cit	1	LOST-3_cit	1
1	Ew/a-p-LOST-	1	LOST_cit	1	LOST-3_cit	1
1	Ew/a-p-LOST-	1	LOST_cit	1	LOST-3_cit	1
0	#N/A	0	#N/A	0	#N/A	0
0	#N/A	0	#N/A	0	#N/A	0
0	#N/A	0		0		0
0	#N/A	0	#N/A	0	#N/A	0
0		0		0		0
0	#N/A	0	#N/A	0	#N/A	0
0	#N/A	0	LOST_cit	1	LOST-3_cit	1
0	#N/A	0	LOST_cit	1	LOST-3_cit	1
0	#N/A	0	LOST_cit	1	LOST-3_cit	1
0	#N/A	0	#N/A	0	#N/A	0
0	#N/A	0	#N/A	0	#N/A	0
0	#N/A	0	#N/A	0	#N/A	0
0	#N/A	0	#N/A	0	#N/A	0
0	#N/A	0	#N/A	0	#N/A	0
0	#N/A	0	#N/A	0	#N/A	0
0	#N/A	0	#N/A	0	#N/A	0
0	#N/A	0	#N/A	0	#N/A	0
0	#N/A	0	#N/A	0	#N/A	0

0	#N/A	0	#N/A	0	#N/A	0
0	#N/A	0	#N/A	0	#N/A	0
0	#N/A	0	#N/A	0	#N/A	0
0	Ew/a-p-LOST-	1		0		0
0	#N/A	0	#N/A	0	#N/A	0
0	#N/A	0	#N/A	0	#N/A	0
0	#N/A	0	#N/A	0	#N/A	0
0	#N/A	0	#N/A	0	#N/A	0
0	#N/A	0	#N/A	0	#N/A	0
0	#N/A	0	#N/A	0	#N/A	0
0	#N/A	0	LOST_cit	1	LOST-3_cit	1
1	Ew/a-p-LOST-	1	#N/A	0	#N/A	0
1	Ew/a-p-LOST-	1	#N/A	0	#N/A	0
0	#N/A	0	#N/A	0	#N/A	0
0	#N/A	0	#N/A	0	#N/A	0
0	#N/A	0	#N/A	0	#N/A	0
0	#N/A	0	#N/A	0	#N/A	0
0	#N/A	0	#N/A	0	#N/A	0
0	#N/A	0	#N/A	0	#N/A	0
0	#N/A	0	#N/A	0	#N/A	0
0	#N/A	0	#N/A	0	#N/A	0
0	#N/A	0	#N/A	0	#N/A	0
0	#N/A	0	#N/A	0	#N/A	0
0	#N/A	0	#N/A	0	#N/A	0
0		0		0		0
0	#N/A	0	#N/A	0	#N/A	0
0	#N/A	0	#N/A	0	#N/A	0
0	#N/A	0	#N/A	0	#N/A	0
0	#N/A	0	#N/A	0	#N/A	0
0	#N/A	0	#N/A	0	#N/A	0
0	#N/A	0	#N/A	0	#N/A	0
0	#N/A	0	#N/A	0	#N/A	0
0		0	#N/A	0	#N/A	0
0		0	#N/A	0	#N/A	0
0	#N/A	0		0	LOST-3_cit	1
0	#N/A	0	#N/A	0	#N/A	0
0	#N/A	0	#N/A	0	#N/A	0
0	#N/A	0	#N/A	0	#N/A	0
0	#N/A	0	#N/A	0	#N/A	0
0	#N/A	0	#N/A	0	#N/A	0
0	#N/A	0	#N/A	0	#N/A	0
0	#N/A	0	#N/A	0	#N/A	0
0	#N/A	0	#N/A	0	#N/A	0
0		0	LOST_cit	1	LOST-3_cit	1
0		0	LOST_cit	1	LOST-3_cit	1
0	#N/A	0	#N/A	0	#N/A	0
0	#N/A	0	#N/A	0	#N/A	0

0	#N/A	0	#N/A	0	#N/A	0
0	#N/A	0	#N/A	0	#N/A	0
0	#N/A	0	#N/A	0	#N/A	0
0	#N/A	0	#N/A	0	#N/A	0
0	#N/A	0	#N/A	0	#N/A	0
0		0	LOST_cit	1	LOST-3_cit	1
0	#N/A	0	#N/A	0	#N/A	0
0	#N/A	0	#N/A	0	#N/A	0
0	#N/A	0	#N/A	0	#N/A	0
0	#N/A	0	#N/A	0	#N/A	0
0		0	#N/A	0	#N/A	0
0	#N/A	0	#N/A	0	#N/A	0
0	#N/A	0	#N/A	0	#N/A	0
0	#N/A	0	#N/A	0	#N/A	0
0	#N/A	0	#N/A	0	#N/A	0
0	#N/A	0		0	LOST-3_cit	1
0	#N/A	0	#N/A	0	#N/A	0
0	#N/A	0	#N/A	0	#N/A	0
0	#N/A	0	#N/A	0	#N/A	0
1	Ew/a-p-LOST-	1	#N/A	0	#N/A	0
0	Ew/a-p-LOST-	1		0		0
0	Ew/a-p-LOST-	1		0		0
0	#N/A	0	#N/A	0	#N/A	0
0	#N/A	0	#N/A	0	#N/A	0
0	#N/A	0	#N/A	0	#N/A	0
0	#N/A	0	#N/A	0	#N/A	0
0	#N/A	0	#N/A	0	#N/A	0
0	#N/A	0	#N/A	0	#N/A	0
0	#N/A	0	#N/A	0	#N/A	0
0	#N/A	0	#N/A	0	#N/A	0
0	#N/A	0	#N/A	0	#N/A	0
0		0	#N/A	0	#N/A	0
0		0	#N/A	0	#N/A	0
0		0	#N/A	0	#N/A	0
0	#N/A	0	#N/A	0	#N/A	0
0	#N/A	0	#N/A	0	#N/A	0
0	#N/A	0	LOST_cit	1	LOST-3_cit	1
0	#N/A	0	LOST_cit	1	LOST-3_cit	1
1	Ew/a-p-LOST-	1	LOST_cit	1	LOST-3_cit	1
1	Ew/a-p-LOST-	1	LOST_cit	1	LOST-3_cit	1
0		0		0		0
0	#N/A	0	#N/A	0	#N/A	0
0	#N/A	0	#N/A	0	#N/A	0
0	#N/A	0	#N/A	0	#N/A	0
0	#N/A	0	#N/A	0	#N/A	0
0	#N/A	0	#N/A	0	#N/A	0
0	#N/A	0		0		0

0	#N/A	0		0		0
0		0	#N/A	0	#N/A	0
0	#N/A	0	#N/A	0	#N/A	0
0	#N/A	0	#N/A	0	#N/A	0
0	#N/A	0	#N/A	0	#N/A	0
0	#N/A	0	#N/A	0	#N/A	0
0	#N/A	0	#N/A	0	#N/A	0
0	#N/A	0	#N/A	0	#N/A	0
0	Ew/a-p-LOST-	1	LOST_cit	1	LOST-3_cit	1
0	Ew/a-p-LOST-	1	LOST_cit	1	LOST-3_cit	1
0	Ew/a-p-LOST-	1	LOST_cit	1	LOST-3_cit	1
0	#N/A	0	#N/A	0	#N/A	0
0	#N/A	0	#N/A	0	#N/A	0
0	#N/A	0	#N/A	0	#N/A	0
0	#N/A	0	#N/A	0	#N/A	0
0	#N/A	0	#N/A	0	#N/A	0
0	#N/A	0	#N/A	0	#N/A	0
0	#N/A	0	#N/A	0	#N/A	0
0	#N/A	0	#N/A	0	#N/A	0
0	#N/A	0	#N/A	0	#N/A	0
0	#N/A	0	#N/A	0	#N/A	0
0	#N/A	0		0	LOST-3_cit	1
0	#N/A	0	#N/A	0	#N/A	0
0	#N/A	0	#N/A	0	#N/A	0
0	#N/A	0	#N/A	0	#N/A	0
0	#N/A	0	#N/A	0	#N/A	0
0	#N/A	0	#N/A	0	#N/A	0
0	#N/A	0	#N/A	0	#N/A	0
0	#N/A	0	#N/A	0	#N/A	0
0	#N/A	0	#N/A	0	#N/A	0
0	#N/A	0	#N/A	0	#N/A	0
0	#N/A	0	#N/A	0	#N/A	0
0	#N/A	0	#N/A	0	#N/A	0
0	#N/A	0	#N/A	0	#N/A	0
0	#N/A	0	#N/A	0	#N/A	0
0	#N/A	0	#N/A	0	#N/A	0
0	#N/A	0	#N/A	0	#N/A	0
0	#N/A	0	#N/A	0	#N/A	0
0	#N/A	0	#N/A	0	#N/A	0
0	#N/A	0	#N/A	0	#N/A	0
0	#N/A	0	#N/A	0	#N/A	0
0		0	#N/A	0	#N/A	0
0		0	#N/A	0	#N/A	0
0	Ew/a-p-LOST-	1	LOST_cit	1	LOST-3_cit	1
0	Ew/a-p-LOST-	1	LOST_cit	1	LOST-3_cit	1
0	Ew/a-p-LOST-	1	LOST_cit	1	LOST-3_cit	1
0	#N/A	0	LOST_cit	1	LOST-3_cit	1

0	#N/A	0	LOST_cit	1	LOST-3_cit	1
1	Ew/a-p-LOST-	1	#N/A	0	#N/A	0
1	Ew/a-p-LOST-	1	#N/A	0	#N/A	0
0	#N/A	0	#N/A	0	#N/A	0
0		0	#N/A	0	#N/A	0
0		0	#N/A	0	#N/A	0
0		0	#N/A	0	#N/A	0
0	#N/A	0	#N/A	0	#N/A	0
0	#N/A	0	#N/A	0	#N/A	0
0	#N/A	0		0	LOST-3_cit	1
0	#N/A	0	#N/A	0	#N/A	0
0	Ew/a-p-LOST-	1	LOST_cit	1	LOST-3_cit	1
0	Ew/a-p-LOST-	1	LOST_cit	1	LOST-3_cit	1
0	Ew/a-p-LOST-	1	LOST_cit	1	LOST-3_cit	1
0	#N/A	0	#N/A	0	#N/A	0
0	#N/A	0	#N/A	0	#N/A	0
0	#N/A	0	#N/A	0	#N/A	0
0	#N/A	0	#N/A	0	#N/A	0
0	#N/A	0	#N/A	0	#N/A	0
0	#N/A	0	#N/A	0	#N/A	0
0	#N/A	0	#N/A	0	#N/A	0
0	#N/A	0	#N/A	0	#N/A	0
0	#N/A	0	#N/A	0	#N/A	0
0	#N/A	0	#N/A	0	#N/A	0
0	#N/A	0	LOST_cit	1	LOST-3_cit	1
0	#N/A	0	LOST_cit	1	LOST-3_cit	1
0	#N/A	0	LOST_cit	1	LOST-3_cit	1
0	#N/A	0	LOST_cit	1	LOST-3_cit	1
0		0	#N/A	0	#N/A	0
0	#N/A	0	#N/A	0	#N/A	0
0	#N/A	0	#N/A	0	#N/A	0
0		0	#N/A	0	#N/A	0
0		0	#N/A	0	#N/A	0
0		0	#N/A	0	#N/A	0
0	#N/A	0	#N/A	0	#N/A	0
0	#N/A	0	#N/A	0	#N/A	0
0	#N/A	0	#N/A	0	#N/A	0
0	#N/A	0	#N/A	0	#N/A	0
0	#N/A	0	#N/A	0	#N/A	0
0	#N/A	0	#N/A	0	#N/A	0
0	#N/A	0	#N/A	0	#N/A	0
0	#N/A	0	#N/A	0	#N/A	0
0	#N/A	0	#N/A	0	#N/A	0
0	#N/A	0	#N/A	0	#N/A	0
0	#N/A	0	#N/A	0	#N/A	0
0	#N/A	0	#N/A	0	#N/A	0
0	#N/A	0	#N/A	0	#N/A	0

0		0		0	LOST-3_cit	1
0	#N/A	0	#N/A	0	#N/A	0
0	#N/A	0	#N/A	0	#N/A	0
0	#N/A	0	#N/A	0	#N/A	0
0	#N/A	0	LOST_cit	1	LOST-3_cit	1
0	#N/A	0	LOST_cit	1	LOST-3_cit	1
0	#N/A	0	LOST_cit	1	LOST-3_cit	1
0	#N/A	0	#N/A	0	#N/A	0
0	#N/A	0	#N/A	0	#N/A	0
0		0		0		0
0	#N/A	0	#N/A	0	#N/A	0
0	#N/A	0	#N/A	0	#N/A	0
0	#N/A	0	#N/A	0	#N/A	0
0	#N/A	0	#N/A	0	#N/A	0
0	#N/A	0	#N/A	0	#N/A	0
0		0	#N/A	0	#N/A	0
0		0	#N/A	0	#N/A	0
0	#N/A	0	#N/A	0	#N/A	0
0	#N/A	0	#N/A	0	#N/A	0
0	#N/A	0	#N/A	0	#N/A	0
0	#N/A	0	#N/A	0	#N/A	0
0		0	LOST_cit	1	LOST-3_cit	1
0		0	LOST_cit	1	LOST-3_cit	1
0		0	LOST_cit	1	LOST-3_cit	1
0		0	#N/A	0	#N/A	0
0	Ew/a-p-LOST-	1		0	LOST-3_cit	1
0		0	#N/A	0	#N/A	0
0	#N/A	0	#N/A	0	#N/A	0
0	#N/A	0	#N/A	0	#N/A	0
0	#N/A	0	#N/A	0	#N/A	0
0	#N/A	0	#N/A	0	#N/A	0
0	#N/A	0	#N/A	0	#N/A	0
0	#N/A	0	#N/A	0	#N/A	0
0	#N/A	0	#N/A	0	#N/A	0
0	#N/A	0	#N/A	0	#N/A	0
0	#N/A	0	#N/A	0	#N/A	0
0	#N/A	0	#N/A	0	#N/A	0
0		0	LOST_cit	1	LOST-3_cit	1
0		0	LOST_cit	1	LOST-3_cit	1
0	#N/A	0	#N/A	0	#N/A	0
0		0	#N/A	0	#N/A	0
0	#N/A	0	#N/A	0	#N/A	0
0	#N/A	0	#N/A	0	#N/A	0
0	#N/A	0	#N/A	0	#N/A	0
0	#N/A	0	#N/A	0	#N/A	0
0	#N/A	0	#N/A	0	#N/A	0
0	#N/A	0	#N/A	0	#N/A	0

0		0	#N/A	0	#N/A	0
0	#N/A	0	#N/A	0	#N/A	0
0	#N/A	0	#N/A	0	#N/A	0
0	#N/A	0	#N/A	0	#N/A	0
0	#N/A	0	#N/A	0	#N/A	0
0	#N/A	0	#N/A	0	#N/A	0
0	#N/A	0	#N/A	0	#N/A	0
1	Ew/a-p-LOST-	1		0	LOST-3_cit	1
1	Ew/a-p-LOST-	1		0	LOST-3_cit	1
0	#N/A	0	LOST_cit	1	LOST-3_cit	1
0	#N/A	0	LOST_cit	1	LOST-3_cit	1
0	#N/A	0	LOST_cit	1	LOST-3_cit	1
0	#N/A	0	#N/A	0	#N/A	0
0		0	#N/A	0	#N/A	0
0	#N/A	0	#N/A	0	#N/A	0
0		0		0		0
0	#N/A	0	#N/A	0	#N/A	0
0	#N/A	0	#N/A	0	#N/A	0
0	#N/A	0	LOST_cit	1	LOST-3_cit	1
0	#N/A	0	LOST_cit	1	LOST-3_cit	1
0	#N/A	0	#N/A	0	#N/A	0
0	#N/A	0	#N/A	0	#N/A	0
1	Ew/a-p-LOST-	1	LOST_cit	1	LOST-3_cit	1
1	Ew/a-p-LOST-	1	LOST_cit	1	LOST-3_cit	1
1	Ew/a-p-LOST-	1	LOST_cit	1	LOST-3_cit	1
0		0	#N/A	0	#N/A	0
0	#N/A	0	LOST_cit	1	LOST-3_cit	1
0	#N/A	0	#N/A	0	#N/A	0
0	#N/A	0	LOST_cit	1	LOST-3_cit	1
0	#N/A	0	LOST_cit	1	LOST-3_cit	1
0	#N/A	0	#N/A	0	#N/A	0
0	#N/A	0		0		0
0	#N/A	0	LOST_cit	1	LOST-3_cit	1
0	#N/A	0	LOST_cit	1	LOST-3_cit	1
0	#N/A	0		0		0
1	Ew/a-p-LOST-	1	#N/A	0	#N/A	0
0	#N/A	0	#N/A	0	#N/A	0
0	#N/A	0	#N/A	0	#N/A	0
0	#N/A	0	#N/A	0	#N/A	0
0	Ew/a-p-LOST-	1	#N/A	0	#N/A	0
0	Ew/a-p-LOST-	1	#N/A	0	#N/A	0
0	#N/A	0	#N/A	0	#N/A	0
0	#N/A	0	#N/A	0	#N/A	0
0	#N/A	0	#N/A	0	#N/A	0
0	#N/A	0		0		0
0		0	LOST_cit	1	LOST-3_cit	1
0	#N/A	0	#N/A	0	#N/A	0

1	Ew/a-p-LOST-	1	#N/A	0	#N/A	0
1	Ew/a-p-LOST-	1	#N/A	0	#N/A	0
1	Ew/a-p-LOST-	1	#N/A	0	#N/A	0
0	#N/A	0		0		0
0	#N/A	0	#N/A	0	#N/A	0
0		0	LOST_cit	1	LOST-3_cit	1
0	#N/A	0		0	LOST-3_cit	1
0	#N/A	0	#N/A	0	#N/A	0
0	#N/A	0	#N/A	0	#N/A	0
0		0	LOST_cit	1	LOST-3_cit	1
0		0	LOST_cit	1	LOST-3_cit	1
0		0	LOST_cit	1	LOST-3_cit	1
0	#N/A	0	#N/A	0	#N/A	0
0	#N/A	0	#N/A	0	#N/A	0
0	#N/A	0	#N/A	0	#N/A	0
0	#N/A	0	#N/A	0	#N/A	0
0	#N/A	0	#N/A	0	#N/A	0
0	#N/A	0	#N/A	0	#N/A	0
0	#N/A	0	#N/A	0	#N/A	0
0	#N/A	0	#N/A	0	#N/A	0
0	#N/A	0	#N/A	0	#N/A	0
0	#N/A	0	#N/A	0	#N/A	0
0	#N/A	0	#N/A	0	#N/A	0
0	#N/A	0	#N/A	0	#N/A	0
0	#N/A	0	#N/A	0	#N/A	0
0	#N/A	0	#N/A	0	#N/A	0
0	#N/A	0	#N/A	0	#N/A	0
0	#N/A	0	#N/A	0	#N/A	0
0	#N/A	0	LOST_cit	1	LOST-3_cit	1
0	#N/A	0	LOST_cit	1	LOST-3_cit	1
0	#N/A	0	#N/A	0	#N/A	0
0	#N/A	0	LOST_cit	1	LOST-3_cit	1
0		0	#N/A	0	#N/A	0
0		0	#N/A	0	#N/A	0
0	#N/A	0	#N/A	0	#N/A	0
0		0	#N/A	0	#N/A	0
0		0	#N/A	0	#N/A	0
0	#N/A	0	#N/A	0	#N/A	0
0	#N/A	0	#N/A	0	#N/A	0
0		0	LOST_cit	1	LOST-3_cit	1
0		0	LOST_cit	1	LOST-3_cit	1
0	#N/A	0	#N/A	0	#N/A	0
0	#N/A	0	#N/A	0	#N/A	0
0	#N/A	0	#N/A	0	#N/A	0
0	#N/A	0	#N/A	0	#N/A	0
0	#N/A	0	#N/A	0	#N/A	0
0		0	#N/A	0	#N/A	0

0		0	#N/A	0	#N/A	0
0	#N/A	0	LOST_cit	1	LOST-3_cit	1
0	#N/A	0	LOST_cit	1	LOST-3_cit	1
0	#N/A	0	LOST_cit	1	LOST-3_cit	1
0	#N/A	0	#N/A	0	#N/A	0
0	#N/A	0	#N/A	0	#N/A	0
0	#N/A	0	#N/A	0	#N/A	0
0	#N/A	0	#N/A	0	#N/A	0
0	#N/A	0	#N/A	0	#N/A	0
0	#N/A	0	#N/A	0	#N/A	0
0	#N/A	0	#N/A	0	#N/A	0
0		0	#N/A	0	#N/A	0
0	#N/A	0	#N/A	0	#N/A	0
0	#N/A	0		0	LOST-3_cit	1
0	#N/A	0		0	LOST-3_cit	1
0		0	#N/A	0	#N/A	0
0	#N/A	0	LOST_cit	1	LOST-3_cit	1
0	#N/A	0	LOST_cit	1	LOST-3_cit	1
0	#N/A	0	#N/A	0	#N/A	0
0	#N/A	0		0		0
0	#N/A	0	#N/A	0	#N/A	0
0		0	#N/A	0	#N/A	0
0	#N/A	0	#N/A	0	#N/A	0
0	#N/A	0	#N/A	0	#N/A	0
0	#N/A	0	#N/A	0	#N/A	0
0	#N/A	0	#N/A	0	#N/A	0
0	#N/A	0	#N/A	0	#N/A	0
0	#N/A	0	#N/A	0	#N/A	0
0	#N/A	0	#N/A	0	#N/A	0
0	#N/A	0	#N/A	0	#N/A	0
0	#N/A	0		0		0
0	#N/A	0		0		0
1	Ew/a-p-LOST-	1	LOST_cit	1	LOST-3_cit	1
0	#N/A	0	#N/A	0	#N/A	0
0		0	LOST_cit	1	LOST-3_cit	1
1	Ew/a-p-LOST-	1	LOST_cit	1	LOST-3_cit	1
1	Ew/a-p-LOST-	1	LOST_cit	1	LOST-3_cit	1
0	#N/A	0		0		0
0	#N/A	0	#N/A	0	#N/A	0
0	#N/A	0	#N/A	0	#N/A	0
0		0	LOST_cit	1	LOST-3_cit	1
0		0	LOST_cit	1	LOST-3_cit	1
0		0	LOST_cit	1	LOST-3_cit	1
0		0	LOST_cit	1	LOST-3_cit	1
0	#N/A	0		0		0
0	#N/A	0	LOST_cit	1	LOST-3_cit	1
0	#N/A	0	LOST_cit	1	LOST-3_cit	1

0	#N/A	0	#N/A	0	#N/A	0
0		0		0		0
0		0	LOST_cit	1	LOST-3_cit	1
0	#N/A	0	#N/A	0	#N/A	0
0	#N/A	0	#N/A	0	#N/A	0
0		0	#N/A	0	#N/A	0
0		0	#N/A	0	#N/A	0
0		0	#N/A	0	#N/A	0
0		0	#N/A	0	#N/A	0
0	#N/A	0	#N/A	0	#N/A	0
0	#N/A	0	#N/A	0	#N/A	0
0	#N/A	0	#N/A	0	#N/A	0
0	#N/A	0		0	LOST-3_cit	1
0	#N/A	0	#N/A	0	#N/A	0
0	#N/A	0	#N/A	0	#N/A	0
0	#N/A	0	#N/A	0	#N/A	0
0	#N/A	0	#N/A	0	#N/A	0
0	#N/A	0	LOST_cit	1	LOST-3_cit	1
0	Ew/a-p-LOST-	1	LOST_cit	1	LOST-3_cit	1
0	Ew/a-p-LOST-	1	LOST_cit	1	LOST-3_cit	1
0	#N/A	0	#N/A	0	#N/A	0
0	#N/A	0	#N/A	0	#N/A	0
0	#N/A	0	#N/A	0	#N/A	0
0	#N/A	0	#N/A	0	#N/A	0
0		0		0		0
0	#N/A	0	#N/A	0	#N/A	0
0	#N/A	0		0	LOST-3_cit	1
0	#N/A	0	#N/A	0	#N/A	0
0	#N/A	0	#N/A	0	#N/A	0
0	#N/A	0	#N/A	0	#N/A	0
0	#N/A	0	#N/A	0	#N/A	0
0	#N/A	0	#N/A	0	#N/A	0
0	#N/A	0	#N/A	0	#N/A	0
0	#N/A	0	#N/A	0	#N/A	0
0	#N/A	0	#N/A	0	#N/A	0
1	Ew/a-p-LOST-	1		0	LOST-3_cit	1
0	#N/A	0	#N/A	0	#N/A	0
0	#N/A	0	#N/A	0	#N/A	0
0	#N/A	0	#N/A	0	#N/A	0
0		0	LOST_cit	1	LOST-3_cit	1
0	#N/A	0	#N/A	0	#N/A	0
0		0	LOST_cit	1	LOST-3_cit	1
0	#N/A	0	#N/A	0	#N/A	0
0	#N/A	0	#N/A	0	#N/A	0
0	#N/A	0	#N/A	0	#N/A	0
0	#N/A	0	#N/A	0	#N/A	0
0	#N/A	0	#N/A	0	#N/A	0

0	#N/A	0		0		0
0	#N/A	0	#N/A	0	#N/A	0
0	#N/A	0		0		0
0		0	LOST_cit	1	LOST-3_cit	1
0	#N/A	0		0		0
0	#N/A	0		0		0
0	#N/A	0	#N/A	0	#N/A	0
0	#N/A	0	#N/A	0	#N/A	0
0	#N/A	0	#N/A	0	#N/A	0
0	#N/A	0	#N/A	0	#N/A	0
0	#N/A	0	#N/A	0	#N/A	0
0	#N/A	0	#N/A	0	#N/A	0
0	#N/A	0	#N/A	0	#N/A	0
0		0		0		0
0	#N/A	0		0		0
0	#N/A	0	#N/A	0	#N/A	0
0	#N/A	0	#N/A	0	#N/A	0
0	#N/A	0	#N/A	0	#N/A	0
0	#N/A	0	#N/A	0	#N/A	0
0	#N/A	0	#N/A	0	#N/A	0
0	#N/A	0	#N/A	0	#N/A	0
0	#N/A	0	#N/A	0	#N/A	0
0	#N/A	0	#N/A	0	#N/A	0
0	Ew/a-p-LOST-	1	LOST_cit	1	LOST-3_cit	1
0	#N/A	0	#N/A	0	#N/A	0
0	#N/A	0	#N/A	0	#N/A	0
0		0	LOST_cit	1	LOST-3_cit	1
0		0	#N/A	0	#N/A	0
0		0	#N/A	0	#N/A	0
0	#N/A	0	#N/A	0	#N/A	0
0	#N/A	0	#N/A	0	#N/A	0
0	#N/A	0	#N/A	0	#N/A	0
0	#N/A	0	#N/A	0	#N/A	0
0	#N/A	0	#N/A	0	#N/A	0
0	#N/A	0	#N/A	0	#N/A	0
0	#N/A	0	#N/A	0	#N/A	0
0	#N/A	0	#N/A	0	#N/A	0
0	#N/A	0	#N/A	0	#N/A	0
0	#N/A	0	#N/A	0	#N/A	0
0	#N/A	0	#N/A	0	#N/A	0
0	#N/A	0	#N/A	0	#N/A	0
0	#N/A	0	#N/A	0	#N/A	0
0		0	#N/A	0	#N/A	0
1	Ew/a-p-LOST-	1	#N/A	0	#N/A	0
0	#N/A	0	#N/A	0	#N/A	0
0	#N/A	0	#N/A	0	#N/A	0
0	#N/A	0	LOST_cit	1	LOST-3_cit	1

0	#N/A	0	#N/A	0	#N/A	0
0	#N/A	0	#N/A	0	#N/A	0
0	#N/A	0	#N/A	0	#N/A	0
0	#N/A	0	#N/A	0	#N/A	0
0	#N/A	0	#N/A	0	#N/A	0
0	#N/A	0	#N/A	0	#N/A	0
0	#N/A	0	#N/A	0	#N/A	0
0	#N/A	0	#N/A	0	#N/A	0
0	#N/A	0	#N/A	0	#N/A	0
0	#N/A	0	#N/A	0	#N/A	0
0	#N/A	0	#N/A	0	#N/A	0
0	#N/A	0	#N/A	0	#N/A	0
0	#N/A	0	#N/A	0	#N/A	0
0	#N/A	0	#N/A	0	#N/A	0
0	#N/A	0	#N/A	0	#N/A	0
0	#N/A	0	#N/A	0	#N/A	0
0	#N/A	0	#N/A	0	#N/A	0
0		0	LOST_cit	1	LOST-3_cit	1
0	#N/A	0	#N/A	0	#N/A	0
0	#N/A	0	#N/A	0	#N/A	0
0	#N/A	0	#N/A	0	#N/A	0
0	#N/A	0	#N/A	0	#N/A	0
0	#N/A	0	#N/A	0	#N/A	0
0		0	#N/A	0	#N/A	0
0		0	#N/A	0	#N/A	0
0	#N/A	0	#N/A	0	#N/A	0
0	#N/A	0	#N/A	0	#N/A	0
0	#N/A	0	#N/A	0	#N/A	0
0		0	#N/A	0	#N/A	0
0	#N/A	0	#N/A	0	#N/A	0
0	#N/A	0	#N/A	0	#N/A	0
0		0	LOST_cit	1	LOST-3_cit	1
0		0	LOST_cit	1	LOST-3_cit	1
0		0	LOST_cit	1	LOST-3_cit	1
0	#N/A	0	#N/A	0	#N/A	0
0	#N/A	0	#N/A	0	#N/A	0
0	#N/A	0	#N/A	0	#N/A	0
0	#N/A	0	#N/A	0	#N/A	0
0	#N/A	0	#N/A	0	#N/A	0
0	#N/A	0	#N/A	0	#N/A	0
0	#N/A	0	#N/A	0	#N/A	0
0	#N/A	0	#N/A	0	#N/A	0
0	#N/A	0	#N/A	0	#N/A	0
0	#N/A	0	#N/A	0	#N/A	0
0	#N/A	0	#N/A	0	#N/A	0
0	#N/A	0	LOST_cit	1	LOST-3_cit	1
0	#N/A	0	LOST_cit	1	LOST-3_cit	1

0	#N/A	0	LOST_cit	1	LOST-3_cit	1
0	#N/A	0	LOST_cit	1	LOST-3_cit	1
0	#N/A	0	#N/A	0	#N/A	0
0	#N/A	0	#N/A	0	#N/A	0
0	#N/A	0	#N/A	0	#N/A	0
0	#N/A	0	#N/A	0	#N/A	0
0	#N/A	0	#N/A	0	#N/A	0
0	#N/A	0	#N/A	0	#N/A	0
0	#N/A	0	#N/A	0	#N/A	0
0	#N/A	0	#N/A	0	#N/A	0
0	#N/A	0	#N/A	0	#N/A	0
0	#N/A	0	#N/A	0	#N/A	0
0	#N/A	0	#N/A	0	#N/A	0
0		0		0		0
0	#N/A	0	#N/A	0	#N/A	0
0	#N/A	0	#N/A	0	#N/A	0
0	#N/A	0	#N/A	0	#N/A	0
0	#N/A	0	#N/A	0	#N/A	0
0	#N/A	0	#N/A	0	#N/A	0
0	#N/A	0	#N/A	0	#N/A	0
0	#N/A	0	#N/A	0	#N/A	0
0	#N/A	0	LOST_cit	1	LOST-3_cit	1
0	#N/A	0	LOST_cit	1	LOST-3_cit	1
0	#N/A	0	#N/A	0	#N/A	0
0	Ew/a-p-LOST-	1	#N/A	0	#N/A	0
0	Ew/a-p-LOST-	1	#N/A	0	#N/A	0
1	Ew/a-p-LOST-	1	LOST_cit	1	LOST-3_cit	1
1	Ew/a-p-LOST-	1	LOST_cit	1	LOST-3_cit	1
0	#N/A	0	#N/A	0	#N/A	0
0	#N/A	0	#N/A	0	#N/A	0
0	#N/A	0	#N/A	0	#N/A	0
0		0	#N/A	0	#N/A	0
0		0		0		0
0	#N/A	0	#N/A	0	#N/A	0
0	#N/A	0	#N/A	0	#N/A	0
0		0	#N/A	0	#N/A	0
0	#N/A	0	#N/A	0	#N/A	0
0	#N/A	0	#N/A	0	#N/A	0
0		0	LOST_cit	1	LOST-3_cit	1
0		0	LOST_cit	1	LOST-3_cit	1
0		0	LOST_cit	1	LOST-3_cit	1
0		0	LOST_cit	1	LOST-3_cit	1
0	#N/A	0	#N/A	0	#N/A	0
0		0	#N/A	0	#N/A	0
0		0	#N/A	0	#N/A	0
0	#N/A	0	#N/A	0	#N/A	0
0	#N/A	0	#N/A	0	#N/A	0

0	#N/A	0	#N/A	0	#N/A	0
1	Ew/a-p-LOST-	1	#N/A	0	#N/A	0
1	Ew/a-p-LOST-	1	#N/A	0	#N/A	0
1	Ew/a-p-LOST-	1	#N/A	0	#N/A	0
0	#N/A	0	#N/A	0	#N/A	0
0	#N/A	0	#N/A	0	#N/A	0
0	#N/A	0	#N/A	0	#N/A	0
0	#N/A	0	#N/A	0	#N/A	0
0	#N/A	0	#N/A	0	#N/A	0
0		0	LOST_cit	1	LOST-3_cit	1
0	#N/A	0	#N/A	0	#N/A	0
0	#N/A	0	#N/A	0	#N/A	0
0	#N/A	0	#N/A	0	#N/A	0
0	#N/A	0	#N/A	0	#N/A	0
0	#N/A	0	#N/A	0	#N/A	0
0	#N/A	0	#N/A	0	#N/A	0
0	#N/A	0	#N/A	0	#N/A	0
0	Ew/a-p-LOST-	1		0		0
0	Ew/a-p-LOST-	1		0		0
0	#N/A	0	#N/A	0	#N/A	0
0	#N/A	0	#N/A	0	#N/A	0
0	#N/A	0	#N/A	0	#N/A	0
0	#N/A	0	#N/A	0	#N/A	0
0	#N/A	0	#N/A	0	#N/A	0
0	#N/A	0	#N/A	0	#N/A	0
0	#N/A	0	#N/A	0	#N/A	0
0	#N/A	0	#N/A	0	#N/A	0
0	#N/A	0	#N/A	0	#N/A	0
0	#N/A	0	#N/A	0	#N/A	0
1	Ew/a-p-LOST-	1		0	LOST-3_cit	1
1	Ew/a-p-LOST-	1		0	LOST-3_cit	1
0	#N/A	0	#N/A	0	#N/A	0
0	#N/A	0		0	LOST-3_cit	1
0	#N/A	0		0	LOST-3_cit	1
0	#N/A	0	#N/A	0	#N/A	0
0	Ew/a-p-LOST-	1	#N/A	0	#N/A	0
0	Ew/a-p-LOST-	1	#N/A	0	#N/A	0
0	#N/A	0	#N/A	0	#N/A	0
0	#N/A	0	#N/A	0	#N/A	0
0		0	LOST_cit	1	LOST-3_cit	1
0		0	#N/A	0	#N/A	0
0		0	#N/A	0	#N/A	0
0		0	#N/A	0	#N/A	0
1	Ew/a-p-LOST-	1	#N/A	0	#N/A	0
1	Ew/a-p-LOST-	1	#N/A	0	#N/A	0
0	#N/A	0	#N/A	0	#N/A	0
0	#N/A	0	#N/A	0	#N/A	0

0		0	#N/A	0	#N/A	0
0	#N/A	0	#N/A	0	#N/A	0
0		0	#N/A	0	#N/A	0
0	#N/A	0	#N/A	0	#N/A	0
0		0	#N/A	0	#N/A	0
1	Ew/a-p-LOST-	1	#N/A	0	#N/A	0
0	#N/A	0		0		0
0	#N/A	0		0		0
0	#N/A	0	#N/A	0	#N/A	0
0	#N/A	0	#N/A	0	#N/A	0
0	Ew/a-p-LOST-	1	#N/A	0	#N/A	0
0	Ew/a-p-LOST-	1	#N/A	0	#N/A	0
0	Ew/a-p-LOST-	1	#N/A	0	#N/A	0
0	Ew/a-p-LOST-	1	#N/A	0	#N/A	0
0	#N/A	0	#N/A	0	#N/A	0
0	#N/A	0	#N/A	0	#N/A	0
0	#N/A	0	#N/A	0	#N/A	0
0	#N/A	0	#N/A	0	#N/A	0
0	#N/A	0	#N/A	0	#N/A	0
0		0	LOST_cit	1	LOST-3_cit	1
0		0	LOST_cit	1	LOST-3_cit	1
0		0	LOST_cit	1	LOST-3_cit	1
0		0	LOST_cit	1	LOST-3_cit	1
0	#N/A	0	#N/A	0	#N/A	0
0	#N/A	0	#N/A	0	#N/A	0
0	#N/A	0	#N/A	0	#N/A	0
0	#N/A	0	#N/A	0	#N/A	0
0	#N/A	0	#N/A	0	#N/A	0
0	#N/A	0	LOST_cit	1	LOST-3_cit	1
0	#N/A	0	LOST_cit	1	LOST-3_cit	1
0	#N/A	0	LOST_cit	1	LOST-3_cit	1
0	#N/A	0	LOST_cit	1	LOST-3_cit	1
0		0	#N/A	0	#N/A	0
0	#N/A	0	#N/A	0	#N/A	0
0	#N/A	0	#N/A	0	#N/A	0
0	#N/A	0	#N/A	0	#N/A	0
1	Ew/a-p-LOST-	1	#N/A	0	#N/A	0
0	#N/A	0		0		0
0	#N/A	0	#N/A	0	#N/A	0
0	#N/A	0	#N/A	0	#N/A	0
0	#N/A	0	#N/A	0	#N/A	0
1	Ew/a-p-LOST-	1	LOST_cit	1	LOST-3_cit	1
1	Ew/a-p-LOST-	1	LOST_cit	1	LOST-3_cit	1
1	Ew/a-p-LOST-	1	LOST_cit	1	LOST-3_cit	1
0		0		0	LOST-3_cit	1
0		0		0	LOST-3_cit	1
0	#N/A	0	#N/A	0	#N/A	0

0	#N/A	0	#N/A	0	#N/A	0
1	Ew/a-p-LOST-	1	#N/A	0	#N/A	0
0	#N/A	0	#N/A	0	#N/A	0
0	#N/A	0	#N/A	0	#N/A	0
0	#N/A	0	#N/A	0	#N/A	0
0	#N/A	0	#N/A	0	#N/A	0
0	#N/A	0	#N/A	0	#N/A	0
0		0	#N/A	0	#N/A	0
0	#N/A	0	#N/A	0	#N/A	0
0	#N/A	0	#N/A	0	#N/A	0
1	Ew/a-p-LOST-	1		0	LOST-3_cit	1
1	Ew/a-p-LOST-	1		0	LOST-3_cit	1
0	#N/A	0	#N/A	0	#N/A	0
0	#N/A	0	#N/A	0	#N/A	0
0	#N/A	0	#N/A	0	#N/A	0
0	#N/A	0	#N/A	0	#N/A	0
0	#N/A	0	LOST_cit	1	LOST-3_cit	1
0	#N/A	0	LOST_cit	1	LOST-3_cit	1
0	#N/A	0	#N/A	0	#N/A	0
0	#N/A	0	#N/A	0	#N/A	0
0	#N/A	0	#N/A	0	#N/A	0
0	#N/A	0	#N/A	0	#N/A	0
0	#N/A	0		0		0
0		0	#N/A	0	#N/A	0
0		0	#N/A	0	#N/A	0
0		0	#N/A	0	#N/A	0
0	#N/A	0	#N/A	0	#N/A	0
0	#N/A	0	#N/A	0	#N/A	0
0	#N/A	0	#N/A	0	#N/A	0
0	Ew/a-p-LOST-	1	LOST_cit	1	LOST-3_cit	1
0	Ew/a-p-LOST-	1	LOST_cit	1	LOST-3_cit	1
0		0	#N/A	0	#N/A	0
0		0	#N/A	0	#N/A	0
0	#N/A	0	#N/A	0	#N/A	0
0	#N/A	0	#N/A	0	#N/A	0
0	#N/A	0	#N/A	0	#N/A	0
0	#N/A	0	#N/A	0	#N/A	0
0	#N/A	0	#N/A	0	#N/A	0
0		0		0		0
0		0		0		0
0	#N/A	0	#N/A	0	#N/A	0
0	#N/A	0	#N/A	0	#N/A	0
0		0	#N/A	0	#N/A	0
0	#N/A	0	#N/A	0	#N/A	0
0	#N/A	0	#N/A	0	#N/A	0
0	#N/A	0	#N/A	0	#N/A	0
0	#N/A	0	#N/A	0	#N/A	0

0	#N/A	0	#N/A	0	#N/A	0
0	#N/A	0	#N/A	0	#N/A	0
1	Ew/a-p-LOST-	1	#N/A	0	#N/A	0
0	Ew/a-p-LOST-	1	LOST_cit	1	LOST-3_cit	1
0	Ew/a-p-LOST-	1	LOST_cit	1	LOST-3_cit	1
0	Ew/a-p-LOST-	1	LOST_cit	1	LOST-3_cit	1
0	#N/A	0	#N/A	0	#N/A	0
0	#N/A	0	#N/A	0	#N/A	0
0	#N/A	0	#N/A	0	#N/A	0
0	#N/A	0	#N/A	0	#N/A	0
0	#N/A	0	#N/A	0	#N/A	0
0	#N/A	0	LOST_cit	1	LOST-3_cit	1
0	#N/A	0	LOST_cit	1	LOST-3_cit	1
0	#N/A	0	LOST_cit	1	LOST-3_cit	1
0	#N/A	0	#N/A	0	#N/A	0
0	#N/A	0	#N/A	0	#N/A	0
0	#N/A	0	#N/A	0	#N/A	0
0	#N/A	0	#N/A	0	#N/A	0
0	#N/A	0	#N/A	0	#N/A	0
1	Ew/a-p-LOST-	1		0	LOST-3_cit	1
0	#N/A	0	#N/A	0	#N/A	0
0	#N/A	0	#N/A	0	#N/A	0
0	#N/A	0	#N/A	0	#N/A	0
0	#N/A	0	#N/A	0	#N/A	0
0	#N/A	0	#N/A	0	#N/A	0
0	#N/A	0	#N/A	0	#N/A	0
0	Ew/a-p-LOST-	1	#N/A	0	#N/A	0
0	Ew/a-p-LOST-	1	#N/A	0	#N/A	0
0	#N/A	0	#N/A	0	#N/A	0
0	#N/A	0	#N/A	0	#N/A	0
0	#N/A	0	#N/A	0	#N/A	0
0	#N/A	0	#N/A	0	#N/A	0
0	#N/A	0	#N/A	0	#N/A	0
0	#N/A	0	#N/A	0	#N/A	0
0	#N/A	0	#N/A	0	#N/A	0
0	#N/A	0	#N/A	0	#N/A	0
0	#N/A	0	#N/A	0	#N/A	0
0	#N/A	0	#N/A	0	#N/A	0
0	#N/A	0	#N/A	0	#N/A	0
0	#N/A	0	#N/A	0	#N/A	0
0	#N/A	0	#N/A	0	#N/A	0
0	#N/A	0	#N/A	0	#N/A	0
0	#N/A	0	#N/A	0	#N/A	0
0	#N/A	0	#N/A	0	#N/A	0
0	#N/A	0	#N/A	0	#N/A	0
0	#N/A	0	#N/A	0	#N/A	0
0		0	#N/A	0	#N/A	0

0		0	LOST_cit	1	LOST-3_cit	1
0	#N/A	0	#N/A	0	#N/A	0
0	#N/A	0	#N/A	0	#N/A	0
0	#N/A	0		0	LOST-3_cit	1
0	#N/A	0	LOST_cit	1	LOST-3_cit	1
1	Ew/a-p-LOST-	1	LOST_cit	1	LOST-3_cit	1
1	Ew/a-p-LOST-	1	LOST_cit	1	LOST-3_cit	1
0		0		0		0
0	#N/A	0	#N/A	0	#N/A	0
0	#N/A	0	#N/A	0	#N/A	0
0	#N/A	0	LOST_cit	1	LOST-3_cit	1
0	#N/A	0	LOST_cit	1	LOST-3_cit	1
0		0	#N/A	0	#N/A	0
0	#N/A	0	#N/A	0	#N/A	0
0	#N/A	0	#N/A	0	#N/A	0
0	#N/A	0	#N/A	0	#N/A	0
0	#N/A	0		0		0
0		0	#N/A	0	#N/A	0
0	#N/A	0	#N/A	0	#N/A	0
0	#N/A	0	#N/A	0	#N/A	0
0	#N/A	0	#N/A	0	#N/A	0
0	#N/A	0	#N/A	0	#N/A	0
0	#N/A	0	#N/A	0	#N/A	0
0	#N/A	0	#N/A	0	#N/A	0
0	#N/A	0	#N/A	0	#N/A	0
0	#N/A	0	#N/A	0	#N/A	0
0	#N/A	0	#N/A	0	#N/A	0
0	#N/A	0		0	LOST-3_cit	1
0	#N/A	0	#N/A	0	#N/A	0
0	#N/A	0	#N/A	0	#N/A	0
1	Ew/a-p-LOST-	1	#N/A	0	#N/A	0
1	Ew/a-p-LOST-	1	#N/A	0	#N/A	0
0	#N/A	0	#N/A	0	#N/A	0
0	#N/A	0	#N/A	0	#N/A	0
0	#N/A	0		0	LOST-3_cit	1
0	#N/A	0	#N/A	0	#N/A	0
0	#N/A	0	#N/A	0	#N/A	0
1	Ew/a-p-LOST-	1	#N/A	0	#N/A	0
1	Ew/a-p-LOST-	1	#N/A	0	#N/A	0
1	Ew/a-p-LOST-	1	#N/A	0	#N/A	0
0	#N/A	0	#N/A	0	#N/A	0
0	#N/A	0	#N/A	0	#N/A	0
0	#N/A	0	#N/A	0	#N/A	0
0	#N/A	0	#N/A	0	#N/A	0
0	#N/A	0	#N/A	0	#N/A	0
0	#N/A	0	#N/A	0	#N/A	0
0	#N/A	0	LOST_cit	1	LOST-3_cit	1

0	#N/A	0	LOST_cit	1	LOST-3_cit	1
0	#N/A	0	#N/A	0	#N/A	0
0	#N/A	0	#N/A	0	#N/A	0
0	#N/A	0	#N/A	0	#N/A	0
0	#N/A	0	#N/A	0	#N/A	0
0	#N/A	0	#N/A	0	#N/A	0
0	#N/A	0	#N/A	0	#N/A	0
0		0		0		0
0	#N/A	0	#N/A	0	#N/A	0
0	#N/A	0	#N/A	0	#N/A	0
0	#N/A	0	#N/A	0	#N/A	0
0	#N/A	0	#N/A	0	#N/A	0
0		0	LOST_cit	1	LOST-3_cit	1
0		0	LOST_cit	1	LOST-3_cit	1
0		0	LOST_cit	1	LOST-3_cit	1
0	#N/A	0	#N/A	0	#N/A	0
0	Ew/a-p-LOST-	1	LOST_cit	1	LOST-3_cit	1
0	Ew/a-p-LOST-	1	LOST_cit	1	LOST-3_cit	1
0	Ew/a-p-LOST-	1	LOST_cit	1	LOST-3_cit	1
0	#N/A	0	#N/A	0	#N/A	0
0	#N/A	0	#N/A	0	#N/A	0
0	#N/A	0	LOST_cit	1	LOST-3_cit	1
0		0	#N/A	0	#N/A	0
0	#N/A	0		0	LOST-3_cit	1
0	#N/A	0		0	LOST-3_cit	1
0	#N/A	0	#N/A	0	#N/A	0
0	#N/A	0	#N/A	0	#N/A	0
0	Ew/a-p-LOST-	1	#N/A	0	#N/A	0
0	Ew/a-p-LOST-	1	#N/A	0	#N/A	0
0	Ew/a-p-LOST-	1	#N/A	0	#N/A	0
0	#N/A	0	#N/A	0	#N/A	0
0	#N/A	0		0		0
0	#N/A	0	#N/A	0	#N/A	0
0		0	LOST_cit	1	LOST-3_cit	1
0		0	LOST_cit	1	LOST-3_cit	1
0		0	LOST_cit	1	LOST-3_cit	1
0		0	LOST_cit	1	LOST-3_cit	1
0	#N/A	0	#N/A	0	#N/A	0
0		0		0		0
0		0		0		0
0	#N/A	0	LOST_cit	1	LOST-3_cit	1
0	#N/A	0	LOST_cit	1	LOST-3_cit	1
0	#N/A	0	#N/A	0	#N/A	0
0	#N/A	0	LOST_cit	1	LOST-3_cit	1
0	#N/A	0	LOST_cit	1	LOST-3_cit	1
0	#N/A	0		0		0
0	#N/A	0		0		0

0	#N/A	0	#N/A	0	#N/A	0
0	#N/A	0	#N/A	0	#N/A	0
0	#N/A	0	#N/A	0	#N/A	0
1	Ew/a-p-LOST-	1	#N/A	0	#N/A	0
1	Ew/a-p-LOST-	1	#N/A	0	#N/A	0
1	Ew/a-p-LOST-	1	#N/A	0	#N/A	0
1	Ew/a-p-LOST-	1	#N/A	0	#N/A	0
0	#N/A	0	#N/A	0	#N/A	0
0	#N/A	0	#N/A	0	#N/A	0
0	#N/A	0	#N/A	0	#N/A	0
0	#N/A	0	#N/A	0	#N/A	0
0	#N/A	0	#N/A	0	#N/A	0
0	#N/A	0	#N/A	0	#N/A	0
0	#N/A	0	#N/A	0	#N/A	0
0	#N/A	0	#N/A	0	#N/A	0
0	#N/A	0	#N/A	0	#N/A	0
0	#N/A	0	#N/A	0	#N/A	0
0		0	LOST_cit	1	LOST-3_cit	1
0		0	LOST_cit	1	LOST-3_cit	1
0		0	LOST_cit	1	LOST-3_cit	1
0	#N/A	0	LOST_cit	1	LOST-3_cit	1
0	#N/A	0	LOST_cit	1	LOST-3_cit	1
0	#N/A	0	#N/A	0	#N/A	0
0	#N/A	0	#N/A	0	#N/A	0
0	#N/A	0	#N/A	0	#N/A	0
0	#N/A	0	#N/A	0	#N/A	0
0	#N/A	0	LOST_cit	1	LOST-3_cit	1
0		0	LOST_cit	1	LOST-3_cit	1
0	Ew/a-p-LOST-	1	#N/A	0	#N/A	0
0	#N/A	0	#N/A	0	#N/A	0
0	#N/A	0	#N/A	0	#N/A	0
0	#N/A	0	#N/A	0	#N/A	0
0	#N/A	0	#N/A	0	#N/A	0
0	#N/A	0		0		0
0	#N/A	0		0		0
0	#N/A	0	#N/A	0	#N/A	0
1	Ew/a-p-LOST-	1	#N/A	0	#N/A	0
0	#N/A	0	#N/A	0	#N/A	0
0	#N/A	0	#N/A	0	#N/A	0
0	#N/A	0	#N/A	0	#N/A	0
0	#N/A	0	#N/A	0	#N/A	0
0	#N/A	0	#N/A	0	#N/A	0
0	#N/A	0	#N/A	0	#N/A	0
0	#N/A	0	#N/A	0	#N/A	0
0		0	#N/A	0	#N/A	0
0	Ew/a-p-LOST-	1	LOST_cit	1	LOST-3_cit	1
0	Ew/a-p-LOST-	1	LOST_cit	1	LOST-3_cit	1

0	Ew/a-p-LOST-	1	LOST_cit	1	LOST-3_cit	1
0	#N/A	0	#N/A	0	#N/A	0
0	#N/A	0	#N/A	0	#N/A	0
0	#N/A	0	#N/A	0	#N/A	0
0	#N/A	0		0	LOST-3_cit	1
0	#N/A	0	#N/A	0	#N/A	0
0	#N/A	0	#N/A	0	#N/A	0
0	#N/A	0	#N/A	0	#N/A	0
0		0	#N/A	0	#N/A	0
0	#N/A	0	#N/A	0	#N/A	0
0	#N/A	0	#N/A	0	#N/A	0
0		0	#N/A	0	#N/A	0
0	#N/A	0	#N/A	0	#N/A	0
0	#N/A	0	#N/A	0	#N/A	0
0	#N/A	0	#N/A	0	#N/A	0
0	#N/A	0	LOST_cit	1	LOST-3_cit	1
0	#N/A	0	LOST_cit	1	LOST-3_cit	1
0	#N/A	0	#N/A	0	#N/A	0
0	#N/A	0	#N/A	0	#N/A	0
0	#N/A	0	#N/A	0	#N/A	0
0	#N/A	0	#N/A	0	#N/A	0
1	Ew/a-p-LOST-	1		0	LOST-3_cit	1
1	Ew/a-p-LOST-	1		0		0
0	#N/A	0	#N/A	0	#N/A	0
0	#N/A	0	#N/A	0	#N/A	0
0	#N/A	0	LOST_cit	1	LOST-3_cit	1
0	#N/A	0	LOST_cit	1	LOST-3_cit	1
0	#N/A	0	LOST_cit	1	LOST-3_cit	1
0	#N/A	0	LOST_cit	1	LOST-3_cit	1
0	#N/A	0	#N/A	0	#N/A	0
0		0	LOST_cit	1	LOST-3_cit	1
0		0	LOST_cit	1	LOST-3_cit	1
0	#N/A	0	LOST_cit	1	LOST-3_cit	1
0	#N/A	0	#N/A	0	#N/A	0
0	#N/A	0	#N/A	0	#N/A	0
0	#N/A	0	#N/A	0	#N/A	0
0	#N/A	0	#N/A	0	#N/A	0
0	#N/A	0	#N/A	0	#N/A	0
0	#N/A	0	#N/A	0	#N/A	0
0	#N/A	0		0	LOST-3_cit	1
0		0		0		0
0	#N/A	0	#N/A	0	#N/A	0
0		0	#N/A	0	#N/A	0
0		0	#N/A	0	#N/A	0
0	#N/A	0	#N/A	0	#N/A	0
0	#N/A	0	#N/A	0	#N/A	0
0	Ew/a-p-LOST-	1		0		0

0	#N/A	0	LOST_cit	1	LOST-3_cit	1
0	#N/A	0	LOST_cit	1	LOST-3_cit	1
0	#N/A	0	#N/A	0	#N/A	0
0	#N/A	0	#N/A	0	#N/A	0
0	#N/A	0	#N/A	0	#N/A	0
0	#N/A	0	#N/A	0	#N/A	0
0	#N/A	0	#N/A	0	#N/A	0
0	#N/A	0	#N/A	0	#N/A	0
0	#N/A	0	#N/A	0	#N/A	0
0	#N/A	0	#N/A	0	#N/A	0
0	#N/A	0	#N/A	0	#N/A	0
0	#N/A	0	#N/A	0	#N/A	0
0	#N/A	0	#N/A	0	#N/A	0
0		0	#N/A	0	#N/A	0
0	#N/A	0	LOST_cit	1	LOST-3_cit	1
0	#N/A	0	#N/A	0	#N/A	0
0	#N/A	0	#N/A	0	#N/A	0
0	#N/A	0	#N/A	0	#N/A	0
0	#N/A	0	#N/A	0	#N/A	0
0	#N/A	0	#N/A	0	#N/A	0
0	#N/A	0	#N/A	0	#N/A	0
0	#N/A	0	#N/A	0	#N/A	0
1	Ew/a-p-LOST-	1	#N/A	0	#N/A	0
0	#N/A	0	#N/A	0	#N/A	0
0	#N/A	0	LOST_cit	1	LOST-3_cit	1
0	#N/A	0	LOST_cit	1	LOST-3_cit	1
0	#N/A	0	#N/A	0	#N/A	0
0	#N/A	0	#N/A	0	#N/A	0
0	#N/A	0	#N/A	0	#N/A	0
0	#N/A	0	#N/A	0	#N/A	0
0	#N/A	0	#N/A	0	#N/A	0
0	#N/A	0	#N/A	0	#N/A	0
0	#N/A	0	#N/A	0	#N/A	0
0	#N/A	0	#N/A	0	#N/A	0
0	#N/A	0	#N/A	0	#N/A	0
0	#N/A	0	#N/A	0	#N/A	0
0	#N/A	0	#N/A	0	#N/A	0
0	#N/A	0	#N/A	0	#N/A	0
0	#N/A	0	#N/A	0	#N/A	0
0	#N/A	0		0		0
0	#N/A	0	#N/A	0	#N/A	0
0	#N/A	0	#N/A	0	#N/A	0
1	Ew/a-p-LOST-	1		0	LOST-3_cit	1
0	#N/A	0	#N/A	0	#N/A	0
0	#N/A	0	#N/A	0	#N/A	0
0	#N/A	0	#N/A	0	#N/A	0
0	#N/A	0	#N/A	0	#N/A	0

0	#N/A	0	#N/A	0	#N/A	0
0		0	#N/A	0	#N/A	0
0		0	#N/A	0	#N/A	0
0		0	#N/A	0	#N/A	0
0	#N/A	0	#N/A	0	#N/A	0
1	Ew/a-p-LOST-	1	#N/A	0	#N/A	0
1	Ew/a-p-LOST-	1	#N/A	0	#N/A	0
1	Ew/a-p-LOST-	1	#N/A	0	#N/A	0
0	#N/A	0	#N/A	0	#N/A	0
0	#N/A	0	#N/A	0	#N/A	0
0	#N/A	0	LOST_cit	1	LOST-3_cit	1
0	#N/A	0	LOST_cit	1	LOST-3_cit	1
0	#N/A	0	#N/A	0	#N/A	0
0	#N/A	0	#N/A	0	#N/A	0
0	#N/A	0	#N/A	0	#N/A	0
0	#N/A	0	#N/A	0	#N/A	0
0	#N/A	0	#N/A	0	#N/A	0
0	#N/A	0	#N/A	0	#N/A	0
0	#N/A	0	#N/A	0	#N/A	0
0		0		0	LOST-3_cit	1
0		0		0	LOST-3_cit	1
0	#N/A	0	#N/A	0	#N/A	0
0	#N/A	0		0		0
0	#N/A	0	#N/A	0	#N/A	0
0		0	#N/A	0	#N/A	0
0	#N/A	0	#N/A	0	#N/A	0
0		0	#N/A	0	#N/A	0
0		0	#N/A	0	#N/A	0
0	#N/A	0	#N/A	0	#N/A	0
0	#N/A	0	#N/A	0	#N/A	0
0	#N/A	0	#N/A	0	#N/A	0
0	#N/A	0	#N/A	0	#N/A	0
0	#N/A	0	LOST_cit	1	LOST-3_cit	1
0	#N/A	0	#N/A	0	#N/A	0
0	#N/A	0		0	LOST-3_cit	1
0	#N/A	0	#N/A	0	#N/A	0
0	#N/A	0	#N/A	0	#N/A	0
0	#N/A	0	#N/A	0	#N/A	0
0	#N/A	0	#N/A	0	#N/A	0
0	#N/A	0	#N/A	0	#N/A	0
0	#N/A	0	LOST_cit	1	LOST-3_cit	1
0	#N/A	0	LOST_cit	1	LOST-3_cit	1
1	Ew/a-p-LOST-	1	LOST_cit	1	LOST-3_cit	1
1	Ew/a-p-LOST-	1	LOST_cit	1	LOST-3_cit	1
1	Ew/a-p-LOST-	1	LOST_cit	1	LOST-3_cit	1
1	Ew/a-p-LOST-	1	LOST_cit	1	LOST-3_cit	1
1	Ew/a-p-LOST-	1	LOST_cit	1	LOST-3_cit	1

1	Ew/a-p-LOST-	1	LOST_cit	1	LOST-3_cit	1
1	Ew/a-p-LOST-	1	LOST_cit	1	LOST-3_cit	1
1	Ew/a-p-LOST-	1	LOST_cit	1	LOST-3_cit	1
0	#N/A	0	#N/A	0	#N/A	0
0	#N/A	0	#N/A	0	#N/A	0
0	#N/A	0	#N/A	0	#N/A	0
0	#N/A	0	#N/A	0	#N/A	0
0	#N/A	0	#N/A	0	#N/A	0
0	#N/A	0	#N/A	0	#N/A	0
0		0	#N/A	0	#N/A	0
0		0	#N/A	0	#N/A	0
0	#N/A	0	#N/A	0	#N/A	0
0	#N/A	0	#N/A	0	#N/A	0
0	#N/A	0	#N/A	0	#N/A	0
0	#N/A	0	#N/A	0	#N/A	0
0	#N/A	0	#N/A	0	#N/A	0
0	#N/A	0	#N/A	0	#N/A	0
0	#N/A	0	LOST_cit	1	LOST-3_cit	1
0	#N/A	0	#N/A	0	#N/A	0
0	#N/A	0	#N/A	0	#N/A	0
0		0		0	LOST-3_cit	1
0		0		0	LOST-3_cit	1
0	#N/A	0	#N/A	0	#N/A	0
0	#N/A	0	#N/A	0	#N/A	0
0	#N/A	0	#N/A	0	#N/A	0
0	#N/A	0	#N/A	0	#N/A	0
0	#N/A	0	LOST_cit	1	LOST-3_cit	1
0	#N/A	0	#N/A	0	#N/A	0
0	#N/A	0	#N/A	0	#N/A	0
0	#N/A	0	#N/A	0	#N/A	0
0	#N/A	0	#N/A	0	#N/A	0
0	#N/A	0	#N/A	0	#N/A	0
0	#N/A	0	#N/A	0	#N/A	0
1	Ew/a-p-LOST-	1		0	LOST-3_cit	1
1	Ew/a-p-LOST-	1		0	LOST-3_cit	1
0	#N/A	0	#N/A	0	#N/A	0
0	#N/A	0	#N/A	0	#N/A	0
0	#N/A	0	LOST_cit	1	LOST-3_cit	1
0	#N/A	0	LOST_cit	1	LOST-3_cit	1
0	#N/A	0	#N/A	0	#N/A	0
0	#N/A	0	#N/A	0	#N/A	0
0	#N/A	0	#N/A	0	#N/A	0
0	#N/A	0	#N/A	0	#N/A	0
0	#N/A	0	#N/A	0	#N/A	0
0	#N/A	0	#N/A	0	#N/A	0
0		0	#N/A	0	#N/A	0
0	#N/A	0	#N/A	0	#N/A	0

0	#N/A	0	#N/A	0	#N/A	0
0		0	LOST_cit	1	LOST-3_cit	1
1	Ew/a-p-LOST-	1	LOST_cit	1	LOST-3_cit	1
1	Ew/a-p-LOST-	1	LOST_cit	1	LOST-3_cit	1
1	Ew/a-p-LOST-	1	LOST_cit	1	LOST-3_cit	1
1	Ew/a-p-LOST-	1	LOST_cit	1	LOST-3_cit	1
1	Ew/a-p-LOST-	1		0		0
0		0	#N/A	0	#N/A	0
0		0	#N/A	0	#N/A	0
0		0	#N/A	0	#N/A	0
0	#N/A	0	#N/A	0	#N/A	0
0	#N/A	0	#N/A	0	#N/A	0
0	#N/A	0	#N/A	0	#N/A	0
0	#N/A	0	LOST_cit	1	LOST-3_cit	1
0	#N/A	0	LOST_cit	1	LOST-3_cit	1
0	#N/A	0	LOST_cit	1	LOST-3_cit	1
0	#N/A	0	#N/A	0	#N/A	0
0	#N/A	0	#N/A	0	#N/A	0
0	#N/A	0	#N/A	0	#N/A	0
0	#N/A	0	#N/A	0	#N/A	0
0	Ew/a-p-LOST-	1	#N/A	0	#N/A	0
0	Ew/a-p-LOST-	1	#N/A	0	#N/A	0
0	#N/A	0	#N/A	0	#N/A	0
0	#N/A	0	#N/A	0	#N/A	0
0	#N/A	0	#N/A	0	#N/A	0
0	#N/A	0	#N/A	0	#N/A	0
0	#N/A	0	#N/A	0	#N/A	0
0	#N/A	0	#N/A	0	#N/A	0
0	#N/A	0	LOST_cit	1	LOST-3_cit	1
0	#N/A	0	LOST_cit	1	LOST-3_cit	1
0	#N/A	0	#N/A	0	#N/A	0
1	Ew/a-p-LOST-	1	LOST_cit	1	LOST-3_cit	1
1	Ew/a-p-LOST-	1	#N/A	0	#N/A	0
1	Ew/a-p-LOST-	1	#N/A	0	#N/A	0
1	Ew/a-p-LOST-	1	#N/A	0	#N/A	0
0	#N/A	0	#N/A	0	#N/A	0
0		0	#N/A	0	#N/A	0
0	#N/A	0	#N/A	0	#N/A	0
0	#N/A	0	#N/A	0	#N/A	0
0	#N/A	0	#N/A	0	#N/A	0
0	#N/A	0	#N/A	0	#N/A	0
0	#N/A	0	#N/A	0	#N/A	0
0	#N/A	0	#N/A	0	#N/A	0
0	#N/A	0	#N/A	0	#N/A	0
0	#N/A	0	#N/A	0	#N/A	0
0	#N/A	0	#N/A	0	#N/A	0
0	#N/A	0	#N/A	0	#N/A	0

0	#N/A	0	#N/A	0	#N/A	0
0	#N/A	0	#N/A	0	#N/A	0
0	#N/A	0	LOST_cit	1	LOST-3_cit	1
0	#N/A	0	LOST_cit	1	LOST-3_cit	1
0	#N/A	0	LOST_cit	1	LOST-3_cit	1
1	Ew/a-p-LOST-	1	LOST_cit	1	LOST-3_cit	1
1	Ew/a-p-LOST-	1	LOST_cit	1	LOST-3_cit	1
1	Ew/a-p-LOST-	1	LOST_cit	1	LOST-3_cit	1
0	#N/A	0	#N/A	0	#N/A	0
0	#N/A	0	#N/A	0	#N/A	0
0	#N/A	0	#N/A	0	#N/A	0
0	#N/A	0	#N/A	0	#N/A	0
0	#N/A	0	#N/A	0	#N/A	0
0	#N/A	0	#N/A	0	#N/A	0
0	#N/A	0	#N/A	0	#N/A	0
0	#N/A	0	#N/A	0	#N/A	0
0	#N/A	0	#N/A	0	#N/A	0
0	#N/A	0	#N/A	0	#N/A	0
0	#N/A	0	#N/A	0	#N/A	0
0	#N/A	0	#N/A	0	#N/A	0
0	#N/A	0	#N/A	0	#N/A	0
0	#N/A	0	#N/A	0	#N/A	0
0	#N/A	0	#N/A	0	#N/A	0
0	#N/A	0	#N/A	0	#N/A	0
0	#N/A	0	#N/A	0	#N/A	0
0	Ew/a-p-LOST-	1	LOST_cit	1	LOST-3_cit	1
0	Ew/a-p-LOST-	1	LOST_cit	1	LOST-3_cit	1
0	Ew/a-p-LOST-	1	LOST_cit	1	LOST-3_cit	1
0	Ew/a-p-LOST-	1	LOST_cit	1	LOST-3_cit	1
0	Ew/a-p-LOST-	1	LOST_cit	1	LOST-3_cit	1
0	#N/A	0	#N/A	0	#N/A	0
0	#N/A	0	LOST_cit	1	LOST-3_cit	1
0	#N/A	0	LOST_cit	1	LOST-3_cit	1
0	#N/A	0	#N/A	0	#N/A	0
0	#N/A	0	#N/A	0	#N/A	0
0	#N/A	0	#N/A	0	#N/A	0
0	#N/A	0	#N/A	0	#N/A	0
1	Ew/a-p-LOST-	1	#N/A	0	#N/A	0
1	Ew/a-p-LOST-	1	#N/A	0	#N/A	0
0		0	#N/A	0	#N/A	0
1	Ew/a-p-LOST-	1		0		0
0	#N/A	0	#N/A	0	#N/A	0
0	#N/A	0	#N/A	0	#N/A	0
1	Ew/a-p-LOST-	1		0		0
1	Ew/a-p-LOST-	1		0		0
0	#N/A	0	#N/A	0	#N/A	0
0	#N/A	0	#N/A	0	#N/A	0

0	#N/A	0	#N/A	0	#N/A	0
0	#N/A	0	#N/A	0	#N/A	0
0	#N/A	0	#N/A	0	#N/A	0
0	#N/A	0	#N/A	0	#N/A	0
0	#N/A	0	#N/A	0	#N/A	0
0	#N/A	0	#N/A	0	#N/A	0
0	#N/A	0	#N/A	0	#N/A	0
0		0		0		0
0		0		0		0
0	#N/A	0	#N/A	0	#N/A	0
0	#N/A	0	#N/A	0	#N/A	0
0	#N/A	0	#N/A	0	#N/A	0
0	#N/A	0	#N/A	0	#N/A	0
0	#N/A	0	#N/A	0	#N/A	0
0	#N/A	0	#N/A	0	#N/A	0
0		0	#N/A	0	#N/A	0
0		0	LOST_cit	1	LOST-3_cit	1
0	#N/A	0	#N/A	0	#N/A	0
0	#N/A	0	#N/A	0	#N/A	0
0	#N/A	0	#N/A	0	#N/A	0
0	#N/A	0	#N/A	0	#N/A	0
0	#N/A	0	#N/A	0	#N/A	0
0	#N/A	0		0		0
0	#N/A	0		0		0
0	#N/A	0	#N/A	0	#N/A	0
0	#N/A	0	#N/A	0	#N/A	0
0	#N/A	0	#N/A	0	#N/A	0
0	#N/A	0	#N/A	0	#N/A	0
1	Ew/a-p-LOST-	1		0		0
1	Ew/a-p-LOST-	1		0		0
0	#N/A	0	LOST_cit	1	LOST-3_cit	1
0	#N/A	0	LOST_cit	1	LOST-3_cit	1
0	#N/A	0	#N/A	0	#N/A	0
0	#N/A	0	#N/A	0	#N/A	0
0	#N/A	0	#N/A	0	#N/A	0
0	#N/A	0	LOST_cit	1	LOST-3_cit	1
0	#N/A	0	LOST_cit	1	LOST-3_cit	1
0	#N/A	0	#N/A	0	#N/A	0
0	#N/A	0	#N/A	0	#N/A	0
0	#N/A	0	#N/A	0	#N/A	0
0	#N/A	0	#N/A	0	#N/A	0
0	#N/A	0	#N/A	0	#N/A	0
0	#N/A	0	#N/A	0	#N/A	0
0	#N/A	0	#N/A	0	#N/A	0
0	Ew/a-p-LOST-	1	LOST_cit	1	LOST-3_cit	1
0	Ew/a-p-LOST-	1	LOST_cit	1	LOST-3_cit	1
0	#N/A	0	#N/A	0	#N/A	0

0	#N/A	0	#N/A	0	#N/A	0
0	#N/A	0	#N/A	0	#N/A	0
0	#N/A	0	#N/A	0	#N/A	0
0	#N/A	0	#N/A	0	#N/A	0
0	#N/A	0	#N/A	0	#N/A	0
0	#N/A	0	#N/A	0	#N/A	0
0	#N/A	0	#N/A	0	#N/A	0
0	#N/A	0	#N/A	0	#N/A	0
0	#N/A	0	#N/A	0	#N/A	0
0	#N/A	0	#N/A	0	#N/A	0
0	Ew/a-p-LOST-	1	LOST_cit	1	LOST-3_cit	1
0		0	#N/A	0	#N/A	0
0	#N/A	0	#N/A	0	#N/A	0
0	#N/A	0	#N/A	0	#N/A	0
0	#N/A	0	#N/A	0	#N/A	0
0	#N/A	0	#N/A	0	#N/A	0
0	#N/A	0	#N/A	0	#N/A	0
0	#N/A	0	#N/A	0	#N/A	0
0	#N/A	0	#N/A	0	#N/A	0
0	#N/A	0	#N/A	0	#N/A	0
0	#N/A	0	#N/A	0	#N/A	0
0	#N/A	0	#N/A	0	#N/A	0
0	#N/A	0	#N/A	0	#N/A	0
0	#N/A	0	#N/A	0	#N/A	0
0		0	#N/A	0	#N/A	0
0	#N/A	0	#N/A	0	#N/A	0
0	#N/A	0	#N/A	0	#N/A	0
1	Ew/a-p-LOST-	1	#N/A	0	#N/A	0
1	Ew/a-p-LOST-	1	#N/A	0	#N/A	0
1	Ew/a-p-LOST-	1	#N/A	0	#N/A	0
1	Ew/a-p-LOST-	1	#N/A	0	#N/A	0
0	#N/A	0	#N/A	0	#N/A	0
0	#N/A	0	#N/A	0	#N/A	0
0	#N/A	0	#N/A	0	#N/A	0
0		0	LOST_cit	1	LOST-3_cit	1
0		0	LOST_cit	1	LOST-3_cit	1
0	#N/A	0	#N/A	0	#N/A	0
0	Ew/a-p-LOST-	1	#N/A	0	#N/A	0
0	Ew/a-p-LOST-	1	#N/A	0	#N/A	0
0	#N/A	0	#N/A	0	#N/A	0
0	#N/A	0	LOST_cit	1	LOST-3_cit	1
0	#N/A	0	LOST_cit	1	LOST-3_cit	1
0	#N/A	0	LOST_cit	1	LOST-3_cit	1
0	#N/A	0	#N/A	0	#N/A	0
0	#N/A	0	#N/A	0	#N/A	0
0	#N/A	0	#N/A	0	#N/A	0
0	Ew/a-p-LOST-	1	#N/A	0	#N/A	0

1	Ew/a-p-LOST-	1		0		0
1	Ew/a-p-LOST-	1		0		0
1	Ew/a-p-LOST-	1		0		0
0		0	#N/A	0	#N/A	0
0		0	#N/A	0	#N/A	0
0	#N/A	0	#N/A	0	#N/A	0
0	#N/A	0	#N/A	0	#N/A	0
0	#N/A	0	#N/A	0	#N/A	0
0	#N/A	0	#N/A	0	#N/A	0
0	#N/A	0	#N/A	0	#N/A	0
0	#N/A	0		0		0
0	#N/A	0	#N/A	0	#N/A	0
0	#N/A	0	#N/A	0	#N/A	0
0	#N/A	0	#N/A	0	#N/A	0
0	#N/A	0	#N/A	0	#N/A	0
0		0	#N/A	0	#N/A	0
0	#N/A	0	LOST_cit	1	LOST-3_cit	1
0	#N/A	0	#N/A	0	#N/A	0
0	#N/A	0	#N/A	0	#N/A	0
0	#N/A	0	#N/A	0	#N/A	0
0	#N/A	0	#N/A	0	#N/A	0
0	#N/A	0	#N/A	0	#N/A	0
0	#N/A	0	#N/A	0	#N/A	0
0	#N/A	0	#N/A	0	#N/A	0
0	#N/A	0	#N/A	0	#N/A	0
0	#N/A	0	#N/A	0	#N/A	0
0	#N/A	0		0	LOST-3_cit	1
0	#N/A	0	#N/A	0	#N/A	0
0	#N/A	0	#N/A	0	#N/A	0
0		0	LOST_cit	1	LOST-3_cit	1
0		0	LOST_cit	1	LOST-3_cit	1
0	#N/A	0	#N/A	0	#N/A	0
0	#N/A	0	#N/A	0	#N/A	0
0	#N/A	0	#N/A	0	#N/A	0
0	#N/A	0	#N/A	0	#N/A	0
0	#N/A	0	#N/A	0	#N/A	0
0	#N/A	0	#N/A	0	#N/A	0
0	#N/A	0	#N/A	0	#N/A	0
0	#N/A	0	#N/A	0	#N/A	0
0	#N/A	0	#N/A	0	#N/A	0
0	#N/A	0	#N/A	0	#N/A	0
0	#N/A	0	#N/A	0	#N/A	0
0		0		0		0
0	#N/A	0	#N/A	0	#N/A	0
0	#N/A	0	#N/A	0	#N/A	0
0	#N/A	0	#N/A	0	#N/A	0
0	#N/A	0	#N/A	0	#N/A	0

0	#N/A	0	#N/A	0	#N/A	0
0	#N/A	0	#N/A	0	#N/A	0
0	#N/A	0	#N/A	0	#N/A	0
1	Ew/a-p-LOST-	1	#N/A	0	#N/A	0
1	Ew/a-p-LOST-	1	#N/A	0	#N/A	0
0	#N/A	0	#N/A	0	#N/A	0
1	Ew/a-p-LOST-	1	#N/A	0	#N/A	0
0	#N/A	0	#N/A	0	#N/A	0
0	#N/A	0	#N/A	0	#N/A	0
0	#N/A	0	#N/A	0	#N/A	0
0		0	#N/A	0	#N/A	0
0		0	#N/A	0	#N/A	0
0		0	#N/A	0	#N/A	0
0		0	#N/A	0	#N/A	0
0	#N/A	0	#N/A	0	#N/A	0
0	#N/A	0	#N/A	0	#N/A	0
0	#N/A	0	#N/A	0	#N/A	0
0	#N/A	0	#N/A	0	#N/A	0
0	#N/A	0	#N/A	0	#N/A	0
0	#N/A	0	#N/A	0	#N/A	0
0	#N/A	0	#N/A	0	#N/A	0
0	#N/A	0	#N/A	0	#N/A	0
0	#N/A	0	#N/A	0	#N/A	0
0	#N/A	0	#N/A	0	#N/A	0
0	#N/A	0	LOST_cit	1	LOST-3_cit	1
1	Ew/a-p-LOST-	1	#N/A	0	#N/A	0
0	#N/A	0	#N/A	0	#N/A	0
0	#N/A	0	#N/A	0	#N/A	0
0		0		0		0
0	#N/A	0	#N/A	0	#N/A	0
0	#N/A	0		0		0
0	#N/A	0	#N/A	0	#N/A	0
0		0		0	LOST-3_cit	1
1	Ew/a-p-LOST-	1	#N/A	0	#N/A	0
1	Ew/a-p-LOST-	1	#N/A	0	#N/A	0
0		0	#N/A	0	#N/A	0
0		0	#N/A	0	#N/A	0
0	#N/A	0	#N/A	0	#N/A	0
0	#N/A	0	#N/A	0	#N/A	0
0	#N/A	0	#N/A	0	#N/A	0
0	#N/A	0	#N/A	0	#N/A	0
0	#N/A	0		0		0
0	#N/A	0		0		0
0	#N/A	0	#N/A	0	#N/A	0
0	#N/A	0	#N/A	0	#N/A	0
0	#N/A	0	#N/A	0	#N/A	0
0	#N/A	0	#N/A	0	#N/A	0

0	#N/A	0	#N/A	0	#N/A	0
0	#N/A	0	#N/A	0	#N/A	0
0	#N/A	0	#N/A	0	#N/A	0
0	#N/A	0	#N/A	0	#N/A	0
0		0		0	LOST-3_cit	1
0	#N/A	0		0		0
0	#N/A	0	#N/A	0	#N/A	0
0	#N/A	0	#N/A	0	#N/A	0
0	#N/A	0	#N/A	0	#N/A	0
0	#N/A	0	#N/A	0	#N/A	0
0	#N/A	0	#N/A	0	#N/A	0
0	#N/A	0	#N/A	0	#N/A	0
0	#N/A	0	#N/A	0	#N/A	0
0	#N/A	0		0	LOST-3_cit	1
0	#N/A	0		0	LOST-3_cit	1
0	#N/A	0		0	LOST-3_cit	1
0	#N/A	0	#N/A	0	#N/A	0
0	#N/A	0	#N/A	0	#N/A	0
0	#N/A	0		0		0
0	#N/A	0	#N/A	0	#N/A	0
0	#N/A	0	#N/A	0	#N/A	0
0	#N/A	0	#N/A	0	#N/A	0
0	#N/A	0	#N/A	0	#N/A	0
0	#N/A	0	#N/A	0	#N/A	0
0	#N/A	0	#N/A	0	#N/A	0
0	#N/A	0	#N/A	0	#N/A	0
0	#N/A	0	#N/A	0	#N/A	0
1	Ew/a-p-LOST-	1	#N/A	0	#N/A	0
1	Ew/a-p-LOST-	1	#N/A	0	#N/A	0
0	#N/A	0	#N/A	0	#N/A	0
0	#N/A	0	#N/A	0	#N/A	0
0	#N/A	0	#N/A	0	#N/A	0
0	#N/A	0	#N/A	0	#N/A	0
0	#N/A	0	#N/A	0	#N/A	0
0	#N/A	0	#N/A	0	#N/A	0
0	#N/A	0	#N/A	0	#N/A	0
0	#N/A	0	#N/A	0	#N/A	0
0	#N/A	0	#N/A	0	#N/A	0
0	#N/A	0	#N/A	0	#N/A	0
0	#N/A	0	#N/A	0	#N/A	0
0	#N/A	0	#N/A	0	#N/A	0
1	Ew/a-p-LOST-	1	#N/A	0	#N/A	0
0	#N/A	0	#N/A	0	#N/A	0
0	#N/A	0	#N/A	0	#N/A	0
0	#N/A	0	#N/A	0	#N/A	0
0	#N/A	0	#N/A	0	#N/A	0
0	#N/A	0	#N/A	0	#N/A	0

0	#N/A	0	#N/A	0	#N/A	0
0	#N/A	0	#N/A	0	#N/A	0
0	#N/A	0	#N/A	0	#N/A	0
0	#N/A	0	#N/A	0	#N/A	0
0	#N/A	0	#N/A	0	#N/A	0
0		0	#N/A	0	#N/A	0
0	#N/A	0		0		0
0	#N/A	0		0		0
0	#N/A	0	#N/A	0	#N/A	0
0	#N/A	0	#N/A	0	#N/A	0
1	Ew/a-p-LOST-	1		0	LOST-3_cit	1
1	Ew/a-p-LOST-	1		0	LOST-3_cit	1
0	#N/A	0	#N/A	0	#N/A	0
0	#N/A	0	#N/A	0	#N/A	0
0	#N/A	0	#N/A	0	#N/A	0
0	#N/A	0	#N/A	0	#N/A	0
0	#N/A	0	#N/A	0	#N/A	0
0	#N/A	0	#N/A	0	#N/A	0
0	#N/A	0	#N/A	0	#N/A	0
1	Ew/a-p-LOST-	1	LOST_cit	1	LOST-3_cit	1
1	Ew/a-p-LOST-	1	LOST_cit	1	LOST-3_cit	1
1	Ew/a-p-LOST-	1	LOST_cit	1	LOST-3_cit	1
0	#N/A	0	#N/A	0	#N/A	0
0	#N/A	0	#N/A	0	#N/A	0
0	#N/A	0	#N/A	0	#N/A	0
0	#N/A	0	#N/A	0	#N/A	0
0	#N/A	0	#N/A	0	#N/A	0
0	#N/A	0	#N/A	0	#N/A	0
0	#N/A	0	#N/A	0	#N/A	0
0	#N/A	0	#N/A	0	#N/A	0
0	#N/A	0	#N/A	0	#N/A	0
0	#N/A	0	#N/A	0	#N/A	0
0	#N/A	0	#N/A	0	#N/A	0
0	#N/A	0	#N/A	0	#N/A	0
0	#N/A	0	#N/A	0	#N/A	0
0	#N/A	0	#N/A	0	#N/A	0
1	Ew/a-p-LOST-	1	#N/A	0	#N/A	0
1	Ew/a-p-LOST-	1	#N/A	0	#N/A	0
1	Ew/a-p-LOST-	1	#N/A	0	#N/A	0
0	#N/A	0	#N/A	0	#N/A	0
0	#N/A	0	#N/A	0	#N/A	0
0	#N/A	0	#N/A	0	#N/A	0
0	#N/A	0	#N/A	0	#N/A	0
0		0	#N/A	0	#N/A	0
0		0		0		0
0		0		0		0
0		0		0		0

0	#N/A	0	#N/A	0	#N/A	0
0	#N/A	0	#N/A	0	#N/A	0
0	Ew/a-p-LOST-	1	#N/A	0	#N/A	0
0	#N/A	0	#N/A	0	#N/A	0
0	#N/A	0	#N/A	0	#N/A	0
0	#N/A	0	#N/A	0	#N/A	0
1	Ew/a-p-LOST-	1	#N/A	0	#N/A	0
1	Ew/a-p-LOST-	1		0		0
0	#N/A	0	#N/A	0	#N/A	0
0	#N/A	0	#N/A	0	#N/A	0
0	#N/A	0	#N/A	0	#N/A	0
0	#N/A	0		0		0
0	#N/A	0		0		0
1	Ew/a-p-LOST-	1	#N/A	0	#N/A	0
1	Ew/a-p-LOST-	1	#N/A	0	#N/A	0
1	Ew/a-p-LOST-	1	#N/A	0	#N/A	0
0	#N/A	0	LOST_cit	1	LOST-3_cit	1
0	#N/A	0	LOST_cit	1	LOST-3_cit	1
0	#N/A	0	#N/A	0	#N/A	0
0	#N/A	0	#N/A	0	#N/A	0
0		0		0		0
0	#N/A	0	#N/A	0	#N/A	0
0	#N/A	0	#N/A	0	#N/A	0
0	#N/A	0	LOST_cit	1	LOST-3_cit	1
0	#N/A	0	#N/A	0	#N/A	0
0	#N/A	0		0		0
0	#N/A	0	#N/A	0	#N/A	0
0	#N/A	0	#N/A	0	#N/A	0
0	#N/A	0	#N/A	0	#N/A	0
0	#N/A	0	#N/A	0	#N/A	0
0	#N/A	0	#N/A	0	#N/A	0
0	#N/A	0	#N/A	0	#N/A	0
0	#N/A	0	#N/A	0	#N/A	0
0	#N/A	0	#N/A	0	#N/A	0
0	#N/A	0	LOST_cit	1	LOST-3_cit	1
0	#N/A	0	#N/A	0	#N/A	0
0	#N/A	0	#N/A	0	#N/A	0
0	#N/A	0	#N/A	0	#N/A	0
0	#N/A	0	#N/A	0	#N/A	0
0	#N/A	0	#N/A	0	#N/A	0
0	#N/A	0	#N/A	0	#N/A	0
0	Ew/a-p-LOST-	1	#N/A	0	#N/A	0
0		0	LOST_cit	1	LOST-3_cit	1
0	#N/A	0	#N/A	0	#N/A	0
0		0		0	LOST-3_cit	1
0	#N/A	0	#N/A	0	#N/A	0
0	#N/A	0	#N/A	0	#N/A	0

0	#N/A	0	#N/A	0	#N/A	0
0	#N/A	0	#N/A	0	#N/A	0
0	#N/A	0	#N/A	0	#N/A	0
0		0	#N/A	0	#N/A	0
0	#N/A	0	#N/A	0	#N/A	0
0	#N/A	0	#N/A	0	#N/A	0
0		0		0		0
0	#N/A	0	#N/A	0	#N/A	0
0	#N/A	0	#N/A	0	#N/A	0
0	#N/A	0	#N/A	0	#N/A	0
0	#N/A	0	LOST_cit	1	LOST-3_cit	1
1	Ew/a-p-LOST-	1	#N/A	0	#N/A	0
1	Ew/a-p-LOST-	1	#N/A	0	#N/A	0
0	#N/A	0	#N/A	0	#N/A	0
0	#N/A	0	#N/A	0	#N/A	0
0	#N/A	0	#N/A	0	#N/A	0
0	#N/A	0	#N/A	0	#N/A	0
0	#N/A	0	#N/A	0	#N/A	0
0	#N/A	0	#N/A	0	#N/A	0
0	#N/A	0	#N/A	0	#N/A	0
0	#N/A	0	#N/A	0	#N/A	0
0	Ew/a-p-LOST-	1	LOST_cit	1	LOST-3_cit	1
0	Ew/a-p-LOST-	1	LOST_cit	1	LOST-3_cit	1
0	Ew/a-p-LOST-	1	LOST_cit	1	LOST-3_cit	1
0	#N/A	0	#N/A	0	#N/A	0
0	#N/A	0	#N/A	0	#N/A	0
0		0	#N/A	0	#N/A	0
0	Ew/a-p-LOST-	1	#N/A	0	#N/A	0
0	Ew/a-p-LOST-	1	#N/A	0	#N/A	0
0	Ew/a-p-LOST-	1	#N/A	0	#N/A	0
0	#N/A	0	#N/A	0	#N/A	0
0	#N/A	0	#N/A	0	#N/A	0
0	#N/A	0	#N/A	0	#N/A	0
1	Ew/a-p-LOST-	1	#N/A	0	#N/A	0
1	Ew/a-p-LOST-	1	#N/A	0	#N/A	0
0	#N/A	0	#N/A	0	#N/A	0
0	#N/A	0	#N/A	0	#N/A	0
0	#N/A	0	#N/A	0	#N/A	0
0	#N/A	0	#N/A	0	#N/A	0
0	#N/A	0	#N/A	0	#N/A	0
0		0		0		0
0	#N/A	0	#N/A	0	#N/A	0
0	#N/A	0	#N/A	0	#N/A	0
0	#N/A	0		0		0
0	#N/A	0	#N/A	0	#N/A	0
0		0		0	LOST-3_cit	1
0	#N/A	0	#N/A	0	#N/A	0

0	#N/A	0	#N/A	0	#N/A	0
0	#N/A	0	#N/A	0	#N/A	0
0	#N/A	0	#N/A	0	#N/A	0
0	#N/A	0	#N/A	0	#N/A	0
0	#N/A	0	#N/A	0	#N/A	0
0	#N/A	0	#N/A	0	#N/A	0
0	#N/A	0	#N/A	0	#N/A	0
0		0	LOST_cit	1	LOST-3_cit	1
0		0	LOST_cit	1	LOST-3_cit	1
0		0	LOST_cit	1	LOST-3_cit	1
1	Ew/a-p-LOST-	1	LOST_cit	1	LOST-3_cit	1
1	Ew/a-p-LOST-	1	LOST_cit	1	LOST-3_cit	1
1	Ew/a-p-LOST-	1	LOST_cit	1	LOST-3_cit	1
1	Ew/a-p-LOST-	1	LOST_cit	1	LOST-3_cit	1
0	#N/A	0	#N/A	0	#N/A	0
0		0	#N/A	0	#N/A	0
0		0	#N/A	0	#N/A	0
0		0	#N/A	0	#N/A	0
0	#N/A	0	#N/A	0	#N/A	0
0	#N/A	0	#N/A	0	#N/A	0
0	#N/A	0	#N/A	0	#N/A	0
0	#N/A	0	#N/A	0	#N/A	0
0	#N/A	0	#N/A	0	#N/A	0
0	#N/A	0	#N/A	0	#N/A	0
0	#N/A	0	#N/A	0	#N/A	0
0		0	#N/A	0	#N/A	0
0		0	#N/A	0	#N/A	0
0	#N/A	0	#N/A	0	#N/A	0
0		0	#N/A	0	#N/A	0
0	#N/A	0	#N/A	0	#N/A	0
0	#N/A	0	#N/A	0	#N/A	0
0	#N/A	0	#N/A	0	#N/A	0
0		0		0		0
0		0		0		0
0	#N/A	0	#N/A	0	#N/A	0
0	#N/A	0	#N/A	0	#N/A	0
0	#N/A	0	#N/A	0	#N/A	0
0	#N/A	0		0	LOST-3_cit	1
0	#N/A	0		0	LOST-3_cit	1
0	#N/A	0	#N/A	0	#N/A	0
0	#N/A	0	#N/A	0	#N/A	0
0		0		0	LOST-3_cit	1
0	#N/A	0	#N/A	0	#N/A	0
1	Ew/a-p-LOST-	1	LOST_cit	1	LOST-3_cit	1
1	Ew/a-p-LOST-	1	LOST_cit	1	LOST-3_cit	1
1	Ew/a-p-LOST-	1	LOST_cit	1	LOST-3_cit	1
0		0	#N/A	0	#N/A	0

0	#N/A	0	#N/A	0	#N/A	0
0	#N/A	0	#N/A	0	#N/A	0
0	#N/A	0	#N/A	0	#N/A	0
0	#N/A	0	#N/A	0	#N/A	0
0	#N/A	0	#N/A	0	#N/A	0
0	#N/A	0	#N/A	0	#N/A	0
0	#N/A	0	#N/A	0	#N/A	0
0		0	LOST_cit	1	LOST-3_cit	1
0		0	LOST_cit	1	LOST-3_cit	1
0	#N/A	0	#N/A	0	#N/A	0
0	#N/A	0	#N/A	0	#N/A	0
0	#N/A	0	#N/A	0	#N/A	0
0		0		0		0
0	#N/A	0	#N/A	0	#N/A	0
0	#N/A	0	#N/A	0	#N/A	0
0	#N/A	0	#N/A	0	#N/A	0
0	#N/A	0	#N/A	0	#N/A	0
0	#N/A	0	#N/A	0	#N/A	0
0	#N/A	0	#N/A	0	#N/A	0
0		0	#N/A	0	#N/A	0
0	#N/A	0		0	LOST-3_cit	1
0	#N/A	0	#N/A	0	#N/A	0
0	#N/A	0		0	LOST-3_cit	1
0	#N/A	0		0	LOST-3_cit	1
0		0	LOST_cit	1	LOST-3_cit	1
0	#N/A	0	#N/A	0	#N/A	0
0	#N/A	0	#N/A	0	#N/A	0
0	#N/A	0	#N/A	0	#N/A	0
0	#N/A	0	#N/A	0	#N/A	0
1	Ew/a-p-LOST-	1		0		0
0	#N/A	0	#N/A	0	#N/A	0
0	#N/A	0	#N/A	0	#N/A	0
0	#N/A	0	#N/A	0	#N/A	0
0	#N/A	0	#N/A	0	#N/A	0
0	#N/A	0	#N/A	0	#N/A	0
0	#N/A	0	#N/A	0	#N/A	0
0	#N/A	0	#N/A	0	#N/A	0
0	#N/A	0	#N/A	0	#N/A	0
0		0	#N/A	0	#N/A	0
0	#N/A	0	#N/A	0	#N/A	0
0	#N/A	0	#N/A	0	#N/A	0
0	#N/A	0	#N/A	0	#N/A	0
0		0		0	LOST-3_cit	1
1	Ew/a-p-LOST-	1	LOST_cit	1	LOST-3_cit	1
1	Ew/a-p-LOST-	1	LOST_cit	1	LOST-3_cit	1
1	Ew/a-p-LOST-	1	LOST_cit	1	LOST-3_cit	1
1	Ew/a-p-LOST-	1	LOST_cit	1	LOST-3_cit	1

0	#N/A	0	#N/A	0	#N/A	0
0	#N/A	0	#N/A	0	#N/A	0
0	#N/A	0	#N/A	0	#N/A	0
0	#N/A	0	#N/A	0	#N/A	0
0	#N/A	0	#N/A	0	#N/A	0
0	#N/A	0	#N/A	0	#N/A	0
0	#N/A	0	#N/A	0	#N/A	0
0		0	#N/A	0	#N/A	0
0	#N/A	0	#N/A	0	#N/A	0
0	#N/A	0	#N/A	0	#N/A	0
0	#N/A	0	#N/A	0	#N/A	0
0		0	LOST_cit	1	LOST-3_cit	1
0	#N/A	0	#N/A	0	#N/A	0
0	#N/A	0	#N/A	0	#N/A	0
0	#N/A	0	#N/A	0	#N/A	0
0	#N/A	0	#N/A	0	#N/A	0
0	#N/A	0	#N/A	0	#N/A	0
0	#N/A	0	#N/A	0	#N/A	0
0	#N/A	0	#N/A	0	#N/A	0
0	Ew/a-p-LOST-	1	#N/A	0	#N/A	0
0	Ew/a-p-LOST-	1	#N/A	0	#N/A	0
0	#N/A	0	#N/A	0	#N/A	0
0	Ew/a-p-LOST-	1	#N/A	0	#N/A	0
0	Ew/a-p-LOST-	1	#N/A	0	#N/A	0
0	Ew/a-p-LOST-	1	#N/A	0	#N/A	0
1	Ew/a-p-LOST-	1	#N/A	0	#N/A	0
1	Ew/a-p-LOST-	1	#N/A	0	#N/A	0
1	Ew/a-p-LOST-	1	#N/A	0	#N/A	0
0	#N/A	0	#N/A	0	#N/A	0
0	#N/A	0	#N/A	0	#N/A	0
0	#N/A	0	#N/A	0	#N/A	0
1	Ew/a-p-LOST-	1	#N/A	0	#N/A	0
0	#N/A	0	#N/A	0	#N/A	0
0	#N/A	0	#N/A	0	#N/A	0
0	#N/A	0	LOST_cit	1	LOST-3_cit	1
0	#N/A	0	LOST_cit	1	LOST-3_cit	1
0	#N/A	0	#N/A	0	#N/A	0
0		0	#N/A	0	#N/A	0
0	#N/A	0	#N/A	0	#N/A	0
0	#N/A	0	LOST_cit	1	LOST-3_cit	1
0	#N/A	0	LOST_cit	1	LOST-3_cit	1
0	#N/A	0	LOST_cit	1	LOST-3_cit	1
0	#N/A	0		0	LOST-3_cit	1
0	#N/A	0		0	LOST-3_cit	1
0	#N/A	0	#N/A	0	#N/A	0
0	#N/A	0	#N/A	0	#N/A	0
0	#N/A	0	#N/A	0	#N/A	0

0	#N/A	0	#N/A	0	#N/A	0
0	#N/A	0	#N/A	0	#N/A	0
0	#N/A	0	#N/A	0	#N/A	0
0	#N/A	0	#N/A	0	#N/A	0
0		0		0	LOST-3_cit	1
1	Ew/a-p-LOST-	1	#N/A	0	#N/A	0
0	#N/A	0	#N/A	0	#N/A	0
0	#N/A	0	#N/A	0	#N/A	0
1	Ew/a-p-LOST-	1		0		0
1	Ew/a-p-LOST-	1		0		0
1	Ew/a-p-LOST-	1		0		0
1	Ew/a-p-LOST-	1		0		0
1	Ew/a-p-LOST-	1		0		0
0	#N/A	0	LOST_cit	1	LOST-3_cit	1
0	#N/A	0	#N/A	0	#N/A	0
0	#N/A	0	#N/A	0	#N/A	0
0	#N/A	0	#N/A	0	#N/A	0
0	#N/A	0	#N/A	0	#N/A	0
0	#N/A	0	#N/A	0	#N/A	0
0	#N/A	0	#N/A	0	#N/A	0
0	#N/A	0	#N/A	0	#N/A	0
0	#N/A	0		0		0
0	#N/A	0		0		0
0	#N/A	0	#N/A	0	#N/A	0
0	#N/A	0	#N/A	0	#N/A	0
0	#N/A	0	#N/A	0	#N/A	0
0	#N/A	0	#N/A	0	#N/A	0
1	Ew/a-p-LOST-	1		0		0
1	Ew/a-p-LOST-	1		0		0
0	#N/A	0	#N/A	0	#N/A	0
0	#N/A	0	#N/A	0	#N/A	0
0	#N/A	0	#N/A	0	#N/A	0
0	#N/A	0	#N/A	0	#N/A	0
0	#N/A	0		0	LOST-3_cit	1
0	#N/A	0		0	LOST-3_cit	1
0	#N/A	0	#N/A	0	#N/A	0
0	#N/A	0	#N/A	0	#N/A	0
0	#N/A	0	#N/A	0	#N/A	0
0	Ew/a-p-LOST-	1		0		0
0		0		0		0
1	Ew/a-p-LOST-	1	#N/A	0	#N/A	0
1	Ew/a-p-LOST-	1	#N/A	0	#N/A	0
1	Ew/a-p-LOST-	1	#N/A	0	#N/A	0
0	#N/A	0	#N/A	0	#N/A	0
0	#N/A	0	#N/A	0	#N/A	0
0	#N/A	0	#N/A	0	#N/A	0
0	#N/A	0	#N/A	0	#N/A	0

0	#N/A	0	#N/A	0	#N/A	0
1	Ew/a-p-LOST-	1	LOST_cit	1	LOST-3_cit	1
0	#N/A	0	#N/A	0	#N/A	0
0	#N/A	0	#N/A	0	#N/A	0
0	#N/A	0	#N/A	0	#N/A	0
0	#N/A	0	#N/A	0	#N/A	0
0	#N/A	0	#N/A	0	#N/A	0
0	#N/A	0	#N/A	0	#N/A	0
0	#N/A	0	#N/A	0	#N/A	0
0	#N/A	0	#N/A	0	#N/A	0
0	#N/A	0	#N/A	0	#N/A	0
0	#N/A	0	#N/A	0	#N/A	0
0	#N/A	0	#N/A	0	#N/A	0
0	#N/A	0	#N/A	0	#N/A	0
0	#N/A	0	#N/A	0	#N/A	0
0	#N/A	0	#N/A	0	#N/A	0
0	#N/A	0	#N/A	0	#N/A	0
0	#N/A	0	#N/A	0	#N/A	0
0	#N/A	0	#N/A	0	#N/A	0
0	#N/A	0	#N/A	0	#N/A	0
0	#N/A	0	LOST_cit	1	LOST-3_cit	1
0	#N/A	0	#N/A	0	#N/A	0
0	#N/A	0		0	LOST-3_cit	1
0	#N/A	0	#N/A	0	#N/A	0
0	#N/A	0	#N/A	0	#N/A	0
0	#N/A	0		0	LOST-3_cit	1
0	#N/A	0	#N/A	0	#N/A	0
0	Ew/a-p-LOST-	1	LOST_cit	1	LOST-3_cit	1
0	Ew/a-p-LOST-	1	LOST_cit	1	LOST-3_cit	1
0	#N/A	0	#N/A	0	#N/A	0
0	#N/A	0	#N/A	0	#N/A	0
0	#N/A	0	#N/A	0	#N/A	0
0	#N/A	0	#N/A	0	#N/A	0
0	#N/A	0	#N/A	0	#N/A	0
0	#N/A	0	#N/A	0	#N/A	0
0	#N/A	0	#N/A	0	#N/A	0
0	#N/A	0	#N/A	0	#N/A	0
0	#N/A	0	#N/A	0	#N/A	0
0	#N/A	0	#N/A	0	#N/A	0
0	#N/A	0	#N/A	0	#N/A	0
1	Ew/a-p-LOST-	1		0		0
1	Ew/a-p-LOST-	1		0		0
1	Ew/a-p-LOST-	1		0		0
0	#N/A	0	#N/A	0	#N/A	0
0	#N/A	0	#N/A	0	#N/A	0
0	#N/A	0	#N/A	0	#N/A	0
0		0	#N/A	0	#N/A	0

0		0	#N/A	0	#N/A	0
0	#N/A	0	#N/A	0	#N/A	0
0	#N/A	0	#N/A	0	#N/A	0
0	#N/A	0	#N/A	0	#N/A	0
0		0		0		0
0		0		0		0
0	#N/A	0	#N/A	0	#N/A	0
0	#N/A	0	#N/A	0	#N/A	0
0	#N/A	0	#N/A	0	#N/A	0
0	#N/A	0	#N/A	0	#N/A	0
0	#N/A	0	#N/A	0	#N/A	0
0	#N/A	0	#N/A	0	#N/A	0
0	#N/A	0	#N/A	0	#N/A	0
0	#N/A	0	#N/A	0	#N/A	0
0	#N/A	0	#N/A	0	#N/A	0
0	#N/A	0	#N/A	0	#N/A	0
0	#N/A	0	#N/A	0	#N/A	0
0	#N/A	0	#N/A	0	#N/A	0
1	Ew/a-p-LOST-	1	LOST_cit	1	LOST-3_cit	1
1	Ew/a-p-LOST-	1	LOST_cit	1	LOST-3_cit	1
1	Ew/a-p-LOST-	1	LOST_cit	1	LOST-3_cit	1
0	#N/A	0	#N/A	0	#N/A	0
0		0	#N/A	0	#N/A	0
0		0	#N/A	0	#N/A	0
0		0	#N/A	0	#N/A	0
1	Ew/a-p-LOST-	1	#N/A	0	#N/A	0
1	Ew/a-p-LOST-	1	#N/A	0	#N/A	0
0		0	LOST_cit	1	LOST-3_cit	1
0	#N/A	0	#N/A	0	#N/A	0
0	#N/A	0	#N/A	0	#N/A	0
0	#N/A	0	#N/A	0	#N/A	0
0	#N/A	0	#N/A	0	#N/A	0
1	Ew/a-p-LOST-	1	#N/A	0	#N/A	0
1	Ew/a-p-LOST-	1	#N/A	0	#N/A	0
1	Ew/a-p-LOST-	1	#N/A	0	#N/A	0
0		0		0		0
0		0		0		0
0	#N/A	0	LOST_cit	1	LOST-3_cit	1
0		0	#N/A	0	#N/A	0
0		0	#N/A	0	#N/A	0
0		0	#N/A	0	#N/A	0
0		0	#N/A	0	#N/A	0
0		0	#N/A	0	#N/A	0
0		0	LOST_cit	1	LOST-3_cit	1
1	Ew/a-p-LOST-	1	#N/A	0	#N/A	0
1	Ew/a-p-LOST-	1	#N/A	0	#N/A	0
1	Ew/a-p-LOST-	1	#N/A	0	#N/A	0

0	#N/A	0	#N/A	0	#N/A	0
1	Ew/a-p-LOST-	1	#N/A	0	#N/A	0
0	#N/A	0	#N/A	0	#N/A	0
0	#N/A	0		0		0
0	#N/A	0	#N/A	0	#N/A	0
0	#N/A	0	#N/A	0	#N/A	0
0	#N/A	0	#N/A	0	#N/A	0
0	#N/A	0	#N/A	0	#N/A	0
0	#N/A	0	#N/A	0	#N/A	0
0	#N/A	0	#N/A	0	#N/A	0
0	#N/A	0	#N/A	0	#N/A	0
0	#N/A	0	#N/A	0	#N/A	0
0		0	LOST_cit	1	LOST-3_cit	1
0		0	LOST_cit	1	LOST-3_cit	1
0		0	LOST_cit	1	LOST-3_cit	1
0	#N/A	0	#N/A	0	#N/A	0
0	#N/A	0	#N/A	0	#N/A	0
0	#N/A	0	#N/A	0	#N/A	0
0	#N/A	0	#N/A	0	#N/A	0
0	#N/A	0	#N/A	0	#N/A	0
0	#N/A	0	#N/A	0	#N/A	0
0	#N/A	0	#N/A	0	#N/A	0
0	#N/A	0	#N/A	0	#N/A	0
0	#N/A	0	#N/A	0	#N/A	0
0	#N/A	0		0		0
0	#N/A	0	#N/A	0	#N/A	0
0	#N/A	0	#N/A	0	#N/A	0
0		0	#N/A	0	#N/A	0
0		0	#N/A	0	#N/A	0
0	#N/A	0	#N/A	0	#N/A	0
0	#N/A	0	#N/A	0	#N/A	0
0	#N/A	0	#N/A	0	#N/A	0
0	#N/A	0	#N/A	0	#N/A	0
0	#N/A	0	#N/A	0	#N/A	0
0	#N/A	0	LOST_cit	1	LOST-3_cit	1
0	#N/A	0	LOST_cit	1	LOST-3_cit	1
0	#N/A	0	LOST_cit	1	LOST-3_cit	1
0	#N/A	0	#N/A	0	#N/A	0
0	#N/A	0	#N/A	0	#N/A	0
1	Ew/a-p-LOST-	1	#N/A	0	#N/A	0
1	Ew/a-p-LOST-	1	#N/A	0	#N/A	0
1	Ew/a-p-LOST-	1	#N/A	0	#N/A	0
0	#N/A	0	#N/A	0	#N/A	0
0	#N/A	0	#N/A	0	#N/A	0
0	#N/A	0	#N/A	0	#N/A	0
0	#N/A	0	#N/A	0	#N/A	0
0	#N/A	0	#N/A	0	#N/A	0

0	#N/A	0	#N/A	0	#N/A	0
0	#N/A	0	#N/A	0	#N/A	0
0	#N/A	0	#N/A	0	#N/A	0
0	#N/A	0	#N/A	0	#N/A	0
0	#N/A	0	#N/A	0	#N/A	0
0	#N/A	0	#N/A	0	#N/A	0
0	#N/A	0	#N/A	0	#N/A	0
1	Ew/a-p-LOST-	1		0		0
1	Ew/a-p-LOST-	1		0		0
0	#N/A	0	#N/A	0	#N/A	0
1	Ew/a-p-LOST-	1		0		0
0	#N/A	0	LOST_cit	1	LOST-3_cit	1
0	#N/A	0	#N/A	0	#N/A	0
0	#N/A	0	#N/A	0	#N/A	0
0	#N/A	0	#N/A	0	#N/A	0
0	#N/A	0	#N/A	0	#N/A	0
0		0	LOST_cit	1	LOST-3_cit	1
0		0	LOST_cit	1	LOST-3_cit	1
1	Ew/a-p-LOST-	1	LOST_cit	1	LOST-3_cit	1
1	Ew/a-p-LOST-	1	LOST_cit	1	LOST-3_cit	1
1	Ew/a-p-LOST-	1	LOST_cit	1	LOST-3_cit	1
1	Ew/a-p-LOST-	1	LOST_cit	1	LOST-3_cit	1
0	#N/A	0	#N/A	0	#N/A	0
0	#N/A	0	#N/A	0	#N/A	0
0	#N/A	0	#N/A	0	#N/A	0
0	#N/A	0	#N/A	0	#N/A	0
0	#N/A	0		0	LOST-3_cit	1
0	#N/A	0		0	LOST-3_cit	1
0	#N/A	0		0	LOST-3_cit	1
0	#N/A	0	#N/A	0	#N/A	0
0	#N/A	0	#N/A	0	#N/A	0
0	#N/A	0	#N/A	0	#N/A	0

lost6_cit	lost6_cit_b	lost_7_cit	lost_7_cit_b	nop_elostcit	ewalostcit	ewalostcit_b	ewplostcit
#N/A	0	#N/A	0		#N/A	0	#N/A
#N/A	0	#N/A	0		#N/A	0	#N/A
#N/A	0	#N/A	0		#N/A	0	#N/A
#N/A	0	#N/A	0		#N/A	0	#N/A
	0		0			0	
	0		0			0	
	0		0			0	
	0		0			0	
#N/A	0	#N/A	0		#N/A	0	#N/A
#N/A	0	#N/A	0		#N/A	0	#N/A
#N/A	0	#N/A	0		#N/A	0	#N/A
#N/A	0	#N/A	0		#N/A	0	#N/A
#N/A	0	#N/A	0		#N/A	0	#N/A
	0	LOST>6_cit	1			0	
#N/A	0	#N/A	0		#N/A	0	#N/A
#N/A	0	#N/A	0		#N/A	0	#N/A
#N/A	0	#N/A	0		#N/A	0	#N/A
#N/A	0	#N/A	0		#N/A	0	#N/A
#N/A	0	#N/A	0		#N/A	0	#N/A
#N/A	0	#N/A	0		#N/A	0	#N/A
#N/A	0	#N/A	0		#N/A	0	#N/A
#N/A	0	#N/A	0		#N/A	0	#N/A
#N/A	0	#N/A	0		#N/A	0	#N/A
#N/A	0	#N/A	0		#N/A	0	#N/A
#N/A	0	#N/A	0		#N/A	0	#N/A
#N/A	0	#N/A	0		#N/A	0	#N/A
#N/A	0	#N/A	0		#N/A	0	#N/A
#N/A	0	#N/A	0		#N/A	0	#N/A
#N/A	0	#N/A	0		#N/A	0	#N/A
#N/A	0	#N/A	0		#N/A	0	#N/A
#N/A	0	#N/A	0		#N/A	0	#N/A
	0	LOST>6_cit	1			0	
#N/A	0	#N/A	0		#N/A	0	#N/A
#N/A	0	#N/A	0		#N/A	0	#N/A
#N/A	0	#N/A	0		#N/A	0	#N/A
	0		0			0	
	0		0			0	
#N/A	0	#N/A	0		#N/A	0	#N/A
#N/A	0	#N/A	0		#N/A	0	#N/A
#N/A	0	#N/A	0		#N/A	0	#N/A
#N/A	0	#N/A	0		#N/A	0	#N/A
#N/A	0	#N/A	0		#N/A	0	#N/A
#N/A	0	#N/A	0		#N/A	0	#N/A
#N/A	0	#N/A	0		#N/A	0	#N/A
#N/A	0	#N/A	0		#N/A	0	#N/A
#N/A	0	#N/A	0		#N/A	0	#N/A

#N/A	0	#N/A	0	#N/A	0	#N/A
	0	LOST>6_cit	1		0	Ew/p-LOST-cit
#N/A	0	#N/A	0	#N/A	0	#N/A
#N/A	0	#N/A	0	#N/A	0	#N/A
#N/A	0	#N/A	0	#N/A	0	#N/A
#N/A	0	#N/A	0	#N/A	0	#N/A
#N/A	0	#N/A	0	#N/A	0	#N/A
LOST-6_cit	1	LOST>6_cit	1		0	Ew/p-LOST-cit
	0		0	Ew/a-LOST-cit	1	Ew/p-LOST-cit
#N/A	0	#N/A	0	#N/A	0	#N/A
#N/A	0	#N/A	0	#N/A	0	#N/A
#N/A	0	#N/A	0	#N/A	0	#N/A
LOST-6_cit	1	LOST>6_cit	1		0	
#N/A	0	#N/A	0	#N/A	0	#N/A
#N/A	0	#N/A	0	#N/A	0	#N/A
#N/A	0	#N/A	0	#N/A	0	#N/A
#N/A	0	#N/A	0	#N/A	0	#N/A
#N/A	0	#N/A	0	#N/A	0	#N/A
#N/A	0	#N/A	0	#N/A	0	#N/A
#N/A	0	#N/A	0	#N/A	0	#N/A
#N/A	0	#N/A	0	#N/A	0	#N/A
#N/A	0	#N/A	0	#N/A	0	#N/A
#N/A	0	#N/A	0	#N/A	0	#N/A
#N/A	0	#N/A	0	#N/A	0	#N/A
#N/A	0	#N/A	0	#N/A	0	#N/A
#N/A	0	#N/A	0	#N/A	0	#N/A
#N/A	0	#N/A	0	#N/A	0	#N/A
#N/A	0	#N/A	0	#N/A	0	#N/A
#N/A	0	#N/A	0	#N/A	0	#N/A
#N/A	0	#N/A	0	#N/A	0	#N/A
#N/A	0	#N/A	0	#N/A	0	#N/A
#N/A	0	#N/A	0	#N/A	0	#N/A
#N/A	0	#N/A	0	#N/A	0	#N/A
#N/A	0	#N/A	0	#N/A	0	#N/A
#N/A	0	#N/A	0	#N/A	0	#N/A
#N/A	0	#N/A	0	#N/A	0	#N/A
#N/A	0	#N/A	0	#N/A	0	#N/A
#N/A	0	#N/A	0	#N/A	0	#N/A
	0	LOST>6_cit	1		0	
#N/A	0	#N/A	0	#N/A	0	#N/A
#N/A	0	#N/A	0	#N/A	0	#N/A
#N/A	0	#N/A	0	#N/A	0	#N/A
#N/A	0	#N/A	0	#N/A	0	#N/A
#N/A	0	#N/A	0	#N/A	0	#N/A
#N/A	0	#N/A	0	#N/A	0	#N/A
#N/A	0	#N/A	0	#N/A	0	#N/A
	0		0		0	Ew/p-LOST-cit

	0		0		0	Ew/p-LOST-cit
#N/A	0	#N/A	0	#N/A	0	#N/A
#N/A	0	#N/A	0	#N/A	0	#N/A
#N/A	0	#N/A	0	#N/A	0	#N/A
#N/A	0	#N/A	0	#N/A	0	#N/A
#N/A	0	#N/A	0	#N/A	0	#N/A
	0		0		0	Ew/p-LOST-cit
	0		0		0	Ew/p-LOST-cit
#N/A	0	#N/A	0	#N/A	0	#N/A
#N/A	0	#N/A	0	#N/A	0	#N/A
#N/A	0	#N/A	0	#N/A	0	#N/A
#N/A	0	#N/A	0	#N/A	0	#N/A
#N/A	0	#N/A	0	#N/A	0	#N/A
#N/A	0	#N/A	0	#N/A	0	#N/A
#N/A	0	#N/A	0	#N/A	0	#N/A
#N/A	0	#N/A	0	#N/A	0	#N/A
#N/A	0	#N/A	0	#N/A	0	#N/A
#N/A	0	#N/A	0	#N/A	0	#N/A
#N/A	0	#N/A	0	#N/A	0	#N/A
#N/A	0	#N/A	0	#N/A	0	#N/A
	0		0	Ew/a-LOST-cit	1	Ew/p-LOST-cit
#N/A	0	#N/A	0	#N/A	0	#N/A
#N/A	0	#N/A	0	#N/A	0	#N/A
#N/A	0	#N/A	0	#N/A	0	#N/A
#N/A	0	#N/A	0	#N/A	0	#N/A
#N/A	0	#N/A	0	#N/A	0	#N/A
#N/A	0	#N/A	0	#N/A	0	#N/A
LOST-6_cit	1	LOST>6_cit	1		0	Ew/p-LOST-cit
LOST-6_cit	1	LOST>6_cit	1		0	Ew/p-LOST-cit
LOST-6_cit	1		0	Ew/a-LOST-cit	1	Ew/p-LOST-cit
LOST-6_cit	1		0	Ew/a-LOST-cit	1	Ew/p-LOST-cit
#N/A	0	#N/A	0	#N/A	0	#N/A
LOST-6_cit	1		0	Ew/a-LOST-cit	1	Ew/p-LOST-cit
LOST-6_cit	1		0	Ew/a-LOST-cit	1	Ew/p-LOST-cit
	0		0	Ew/a-LOST-cit	1	Ew/p-LOST-cit
#N/A	0	#N/A	0	#N/A	0	#N/A
#N/A	0	#N/A	0	#N/A	0	#N/A
#N/A	0	#N/A	0	#N/A	0	#N/A
#N/A	0	#N/A	0	#N/A	0	#N/A
LOST-6_cit	1		0	Ew/a-LOST-cit	1	Ew/p-LOST-cit
#N/A	0	#N/A	0	#N/A	0	#N/A
#N/A	0	#N/A	0	#N/A	0	#N/A
#N/A	0	#N/A	0	#N/A	0	#N/A
#N/A	0	#N/A	0	#N/A	0	#N/A
LOST-6_cit	1	LOST>6_cit	1		0	
LOST-6_cit	1	LOST>6_cit	1		0	
#N/A	0	#N/A	0	#N/A	0	#N/A

#N/A	0	#N/A	0	#N/A	0	#N/A
#N/A	0	#N/A	0	#N/A	0	#N/A
#N/A	0	#N/A	0	#N/A	0	#N/A
#N/A	0	#N/A	0	#N/A	0	#N/A
#N/A	0	#N/A	0	#N/A	0	#N/A
#N/A	0	#N/A	0	#N/A	0	#N/A
#N/A	0	#N/A	0	#N/A	0	#N/A
#N/A	0	#N/A	0	#N/A	0	#N/A
#N/A	0	#N/A	0	#N/A	0	#N/A
#N/A	0	#N/A	0	#N/A	0	#N/A
#N/A	0	#N/A	0	#N/A	0	#N/A
	0		0	Ew/a-LOST-cit	1	Ew/p-LOST-cit
	0		0	Ew/a-LOST-cit	1	Ew/p-LOST-cit
	0		0		0	Ew/p-LOST-cit
#N/A	0	#N/A	0	#N/A	0	#N/A
#N/A	0	#N/A	0	#N/A	0	#N/A
	0		0	Ew/a-LOST-cit	1	Ew/p-LOST-cit
#N/A	0	#N/A	0	#N/A	0	#N/A
#N/A	0	#N/A	0	#N/A	0	#N/A
#N/A	0	#N/A	0	#N/A	0	#N/A
#N/A	0	#N/A	0	#N/A	0	#N/A
#N/A	0	#N/A	0	#N/A	0	#N/A
LOST-6_cit	1		0		0	Ew/p-LOST-cit
#N/A	0	#N/A	0	#N/A	0	#N/A
#N/A	0	#N/A	0	#N/A	0	#N/A
#N/A	0	#N/A	0	#N/A	0	#N/A
#N/A	0	#N/A	0	#N/A	0	#N/A
#N/A	0	#N/A	0	#N/A	0	#N/A
#N/A	0	#N/A	0	#N/A	0	#N/A
	0		0	Ew/a-LOST-cit	1	Ew/p-LOST-cit
#N/A	0	#N/A	0	#N/A	0	#N/A
#N/A	0	#N/A	0	#N/A	0	#N/A
	0		0	Ew/a-LOST-cit	1	Ew/p-LOST-cit
#N/A	0	#N/A	0	#N/A	0	#N/A
#N/A	0	#N/A	0	#N/A	0	#N/A
#N/A	0	#N/A	0	#N/A	0	#N/A
#N/A	0	#N/A	0	#N/A	0	#N/A
#N/A	0	#N/A	0	#N/A	0	#N/A
	0		0	Ew/a-LOST-cit	1	Ew/p-LOST-cit
#N/A	0	#N/A	0	#N/A	0	#N/A
#N/A	0	#N/A	0	#N/A	0	#N/A
#N/A	0	#N/A	0	#N/A	0	#N/A
#N/A	0	#N/A	0	#N/A	0	#N/A
#N/A	0	#N/A	0	#N/A	0	#N/A
#N/A	0	#N/A	0	#N/A	0	#N/A
#N/A	0	#N/A	0	#N/A	0	#N/A
#N/A	0	#N/A	0	#N/A	0	#N/A

#N/A	0	#N/A	0	#N/A	0	#N/A
#N/A	0	#N/A	0	#N/A	0	#N/A
#N/A	0	#N/A	0	#N/A	0	#N/A
#N/A	0	#N/A	0	#N/A	0	#N/A
	0		0	Ew/a-LOST-cit	1	Ew/p-LOST-cit
	0		0	Ew/a-LOST-cit	1	Ew/p-LOST-cit
#N/A	0	#N/A	0	#N/A	0	#N/A
#N/A	0	#N/A	0	#N/A	0	#N/A
#N/A	0	#N/A	0	#N/A	0	#N/A
#N/A	0	#N/A	0	#N/A	0	#N/A
#N/A	0	#N/A	0	#N/A	0	#N/A
#N/A	0	#N/A	0	#N/A	0	#N/A
#N/A	0	#N/A	0	#N/A	0	#N/A
#N/A	0	#N/A	0	#N/A	0	#N/A
#N/A	0	#N/A	0	#N/A	0	#N/A
	0		0		0	
	0		0		0	
#N/A	0	#N/A	0	#N/A	0	#N/A
#N/A	0	#N/A	0	#N/A	0	#N/A
#N/A	0	#N/A	0	#N/A	0	#N/A
#N/A	0	#N/A	0	#N/A	0	#N/A
#N/A	0	#N/A	0	#N/A	0	#N/A
#N/A	0	#N/A	0	#N/A	0	#N/A
#N/A	0	#N/A	0	#N/A	0	#N/A
	0		0	Ew/a-LOST-cit	1	
	0		0	Ew/a-LOST-cit	1	
#N/A	0	#N/A	0	#N/A	0	#N/A
#N/A	0	#N/A	0	#N/A	0	#N/A
#N/A	0	#N/A	0	#N/A	0	#N/A
#N/A	0	#N/A	0	#N/A	0	#N/A
#N/A	0	#N/A	0	#N/A	0	#N/A
#N/A	0	#N/A	0	#N/A	0	#N/A
#N/A	0	#N/A	0	#N/A	0	#N/A
#N/A	0	#N/A	0	#N/A	0	#N/A
LOST-6_cit	1	LOST>6_cit	1		0	
#N/A	0	#N/A	0	#N/A	0	#N/A
#N/A	0	#N/A	0	#N/A	0	#N/A
#N/A	0	#N/A	0	#N/A	0	#N/A
	0		0	Ew/a-LOST-cit	1	Ew/p-LOST-cit
	0		0	Ew/a-LOST-cit	1	Ew/p-LOST-cit
	0		0	Ew/a-LOST-cit	1	Ew/p-LOST-cit
	0		0	Ew/a-LOST-cit	1	Ew/p-LOST-cit
#N/A	0	#N/A	0	#N/A	0	#N/A
	0		0	Ew/a-LOST-cit	1	Ew/p-LOST-cit
#N/A	0	#N/A	0	#N/A	0	#N/A
#N/A	0	#N/A	0	#N/A	0	#N/A
#N/A	0	#N/A	0	#N/A	0	#N/A

#N/A	0	#N/A	0	#N/A	0	#N/A	
#N/A	0	#N/A	0	#N/A	0	#N/A	
#N/A	0	#N/A	0	#N/A	0	#N/A	
#N/A	0	#N/A	0	#N/A	0	#N/A	
#N/A	0	#N/A	0	#N/A	0	#N/A	
#N/A	0	#N/A	0	#N/A	0	#N/A	
#N/A	0	#N/A	0	#N/A	0	#N/A	
#N/A	0	#N/A	0	#N/A	0	#N/A	
#N/A	0	#N/A	0	#N/A	0	#N/A	
#N/A	0	#N/A	0	#N/A	0	#N/A	
#N/A	0	#N/A	0	#N/A	0	#N/A	
#N/A	0	#N/A	0	#N/A	0	#N/A	
#N/A	0	#N/A	0	#N/A	0	#N/A	
#N/A	0	#N/A	0	#N/A	0	#N/A	
#N/A	0	#N/A	0	#N/A	0	#N/A	
#N/A	0	#N/A	0	#N/A	0	#N/A	
#N/A	0	#N/A	0	#N/A	0	#N/A	
#N/A	0	#N/A	0	#N/A	0	#N/A	
#N/A	0	#N/A	0	#N/A	0	#N/A	
#N/A	0	#N/A	0	#N/A	0	#N/A	
#N/A	0	#N/A	0	#N/A	0	#N/A	
	0		0	Ew/a-LOST-cit	1	Ew/p-LOST-cit	
	0		0	Ew/a-LOST-cit	1	Ew/p-LOST-cit	
	0		0	Ew/a-LOST-cit	1	Ew/p-LOST-cit	
#N/A	0	#N/A	0	#N/A	0	#N/A	
#N/A	0	#N/A	0	#N/A	0	#N/A	
#N/A	0	#N/A	0	#N/A	0	#N/A	
#N/A	0	#N/A	0	#N/A	0	#N/A	
#N/A	0	#N/A	0	#N/A	0	#N/A	
#N/A	0	#N/A	0	#N/A	0	#N/A	
LOST-6_cit	1	LOST>6_cit	1	Ew/a-LOST-cit	1	Ew/p-LOST-cit	
LOST-6_cit	1	LOST>6_cit	1	Ew/a-LOST-cit	1	Ew/p-LOST-cit	
LOST-6_cit	1	LOST>6_cit	1	Ew/a-LOST-cit	1	Ew/p-LOST-cit	
#N/A	0	#N/A	0	#N/A	0	#N/A	
	0		0		0		
#N/A	0	#N/A	0	#N/A	0	#N/A	
#N/A	0	#N/A	0	#N/A	0	#N/A	
#N/A	0	#N/A	0	#N/A	0	#N/A	
#N/A	0	#N/A	0	#N/A	0	#N/A	
#N/A	0	#N/A	0	#N/A	0	#N/A	
#N/A	0	#N/A	0	#N/A	0	#N/A	
#N/A	0	#N/A	0	#N/A	0	#N/A	
#N/A	0	#N/A	0	#N/A	0	#N/A	
#N/A	0	#N/A	0	#N/A	0	#N/A	
	0	LOST>6_cit	1		0		
#N/A	0	#N/A	0	#N/A	0	#N/A	
#N/A	0	#N/A	0	#N/A	0	#N/A	

#N/A	0	#N/A	0	#N/A	0	#N/A
#N/A	0	#N/A	0	#N/A	0	#N/A
	0		0	Ew/a-LOST-cit	1	
	0	LOST>6_cit	1		0	
#N/A	0	#N/A	0	#N/A	0	#N/A
#N/A	0	#N/A	0	#N/A	0	#N/A
	0	LOST>6_cit	1	Ew/a-LOST-cit	1	Ew/p-LOST-cit
#N/A	0	#N/A	0	#N/A	0	#N/A
#N/A	0	#N/A	0	#N/A	0	#N/A
#N/A	0	#N/A	0	#N/A	0	#N/A
#N/A	0	#N/A	0	#N/A	0	#N/A
#N/A	0	#N/A	0	#N/A	0	#N/A
#N/A	0	#N/A	0	#N/A	0	#N/A
#N/A	0	#N/A	0	#N/A	0	#N/A
#N/A	0	#N/A	0	#N/A	0	#N/A
#N/A	0	#N/A	0	#N/A	0	#N/A
#N/A	0	#N/A	0	#N/A	0	#N/A
#N/A	0	#N/A	0	#N/A	0	#N/A
#N/A	0	#N/A	0	#N/A	0	#N/A
#N/A	0	#N/A	0	#N/A	0	#N/A
#N/A	0	#N/A	0	#N/A	0	#N/A
#N/A	0	#N/A	0	#N/A	0	#N/A
#N/A	0	#N/A	0	#N/A	0	#N/A
#N/A	0	#N/A	0	#N/A	0	#N/A
#N/A	0	#N/A	0	#N/A	0	#N/A
#N/A	0	#N/A	0	#N/A	0	#N/A
#N/A	0	#N/A	0	#N/A	0	#N/A
#N/A	0	#N/A	0	#N/A	0	#N/A
#N/A	0	#N/A	0	#N/A	0	#N/A
#N/A	0	#N/A	0	#N/A	0	#N/A
#N/A	0	#N/A	0	#N/A	0	#N/A
#N/A	0	#N/A	0	#N/A	0	#N/A
#N/A	0	#N/A	0	#N/A	0	#N/A
#N/A	0	#N/A	0	#N/A	0	#N/A
	0	LOST>6_cit	1		0	
	0	LOST>6_cit	1		0	
#N/A	0	#N/A	0	#N/A	0	#N/A
#N/A	0	#N/A	0	#N/A	0	#N/A
#N/A	0	#N/A	0	#N/A	0	#N/A
#N/A	0	#N/A	0	#N/A	0	#N/A
#N/A	0	#N/A	0	#N/A	0	#N/A
#N/A	0	#N/A	0	#N/A	0	#N/A
#N/A	0	#N/A	0	#N/A	0	#N/A
	0		0		0	
	0		0		0	
#N/A	0	#N/A	0	#N/A	0	#N/A
#N/A	0	#N/A	0	#N/A	0	#N/A

#N/A	0	#N/A	0	#N/A	0	#N/A
#N/A	0	#N/A	0	#N/A	0	#N/A
#N/A	0	#N/A	0	#N/A	0	#N/A
#N/A	0	#N/A	0	#N/A	0	#N/A
#N/A	0	#N/A	0	#N/A	0	#N/A
#N/A	0	#N/A	0	#N/A	0	#N/A
#N/A	0	#N/A	0	#N/A	0	#N/A
#N/A	0	#N/A	0	#N/A	0	#N/A
#N/A	0	#N/A	0	#N/A	0	#N/A
#N/A	0	#N/A	0	#N/A	0	#N/A
#N/A	0	#N/A	0	#N/A	0	#N/A
#N/A	0	#N/A	0	#N/A	0	#N/A
#N/A	0	#N/A	0	#N/A	0	#N/A
	0		0		0	
#N/A	0	#N/A	0	#N/A	0	#N/A
#N/A	0	#N/A	0	#N/A	0	#N/A
#N/A	0	#N/A	0	#N/A	0	#N/A
#N/A	0	#N/A	0	#N/A	0	#N/A
#N/A	0	#N/A	0	#N/A	0	#N/A
#N/A	0	#N/A	0	#N/A	0	#N/A
#N/A	0	#N/A	0	#N/A	0	#N/A
#N/A	0	#N/A	0	#N/A	0	#N/A
#N/A	0	#N/A	0	#N/A	0	#N/A
#N/A	0	#N/A	0	#N/A	0	#N/A
	0		0	Ew/a-LOST-cit	1	Ew/p-LOST-cit
	0		0	Ew/a-LOST-cit	1	Ew/p-LOST-cit
#N/A	0	#N/A	0	#N/A	0	#N/A
#N/A	0	#N/A	0	#N/A	0	#N/A
#N/A	0	#N/A	0	#N/A	0	#N/A
#N/A	0	#N/A	0	#N/A	0	#N/A
#N/A	0	#N/A	0	#N/A	0	#N/A
#N/A	0	#N/A	0	#N/A	0	#N/A
#N/A	0	#N/A	0	#N/A	0	#N/A
#N/A	0	#N/A	0	#N/A	0	#N/A
#N/A	0	#N/A	0	#N/A	0	#N/A
#N/A	0	#N/A	0	#N/A	0	#N/A
#N/A	0	#N/A	0	#N/A	0	#N/A
#N/A	0	#N/A	0	#N/A	0	#N/A
#N/A	0	#N/A	0	#N/A	0	#N/A
#N/A	0	#N/A	0	#N/A	0	#N/A
#N/A	0	#N/A	0	#N/A	0	#N/A
#N/A	0	#N/A	0	#N/A	0	#N/A
#N/A	0	#N/A	0	#N/A	0	#N/A
LOST-6_cit	1		0		0	
	0		0		0	
#N/A	0	#N/A	0	#N/A	0	#N/A
#N/A	0	#N/A	0	#N/A	0	#N/A

#N/A	0	#N/A	0	#N/A	0	#N/A
#N/A	0	#N/A	0	#N/A	0	#N/A
LOST-6_cit	1		0		0	
LOST-6_cit	1		0		0	
#N/A	0	#N/A	0	#N/A	0	#N/A
#N/A	0	#N/A	0	#N/A	0	#N/A
#N/A	0	#N/A	0	#N/A	0	#N/A
	0		0	Ew/a-LOST-cit	1	Ew/p-LOST-cit
	0		0	Ew/a-LOST-cit	1	Ew/p-LOST-cit
#N/A	0	#N/A	0	#N/A	0	#N/A
#N/A	0	#N/A	0	#N/A	0	#N/A
#N/A	0	#N/A	0	#N/A	0	#N/A
#N/A	0	#N/A	0	#N/A	0	#N/A
#N/A	0	#N/A	0	#N/A	0	#N/A
#N/A	0	#N/A	0	#N/A	0	#N/A
#N/A	0	#N/A	0	#N/A	0	#N/A
#N/A	0	#N/A	0	#N/A	0	#N/A
#N/A	0	#N/A	0	#N/A	0	#N/A
#N/A	0	#N/A	0	#N/A	0	#N/A
#N/A	0	#N/A	0	#N/A	0	#N/A
#N/A	0	#N/A	0	#N/A	0	#N/A
#N/A	0	#N/A	0	#N/A	0	#N/A
	0		0	Ew/a-LOST-cit	1	
	0		0	Ew/a-LOST-cit	1	
#N/A	0	#N/A	0	#N/A	0	#N/A
#N/A	0	#N/A	0	#N/A	0	#N/A
#N/A	0	#N/A	0	#N/A	0	#N/A
#N/A	0	#N/A	0	#N/A	0	#N/A
#N/A	0	#N/A	0	#N/A	0	#N/A
#N/A	0	#N/A	0	#N/A	0	#N/A
#N/A	0	#N/A	0	#N/A	0	#N/A
#N/A	0	#N/A	0	#N/A	0	#N/A
#N/A	0	#N/A	0	#N/A	0	#N/A
#N/A	0	#N/A	0	#N/A	0	#N/A
#N/A	0	#N/A	0	#N/A	0	#N/A
#N/A	0	#N/A	0	#N/A	0	#N/A
#N/A	0	#N/A	0	#N/A	0	#N/A
	0	LOST>6_cit	1		0	
#N/A	0	#N/A	0	#N/A	0	#N/A
#N/A	0	#N/A	0	#N/A	0	#N/A
#N/A	0	#N/A	0	#N/A	0	#N/A
	0		0		0	
#N/A	0	#N/A	0	#N/A	0	#N/A
#N/A	0	#N/A	0	#N/A	0	#N/A
#N/A	0	#N/A	0	#N/A	0	#N/A
#N/A	0	#N/A	0	#N/A	0	#N/A
#N/A	0	#N/A	0	#N/A	0	#N/A

	0		0		0	
#N/A	0	#N/A	0	#N/A	0	#N/A
#N/A	0	#N/A	0	#N/A	0	#N/A
#N/A	0	#N/A	0	#N/A	0	#N/A
#N/A	0	#N/A	0	#N/A	0	#N/A
LOST-6_cit	1	LOST>6_cit	1		0	
LOST-6_cit	1	LOST>6_cit	1		0	
#N/A	0	#N/A	0	#N/A	0	#N/A
#N/A	0	#N/A	0	#N/A	0	#N/A
#N/A	0	#N/A	0	#N/A	0	#N/A
#N/A	0	#N/A	0	#N/A	0	#N/A
LOST-6_cit	1		0		0	
LOST-6_cit	1		0		0	
#N/A	0	#N/A	0	#N/A	0	#N/A
LOST-6_cit	1		0		0	
	0		0	Ew/a-LOST-cit	1	
	0		0	Ew/a-LOST-cit	1	
	0		0	Ew/a-LOST-cit	1	
	0		0	Ew/a-LOST-cit	1	
	0		0	Ew/a-LOST-cit	1	
	0		0	Ew/a-LOST-cit	1	
#N/A	0	#N/A	0	#N/A	0	#N/A
#N/A	0	#N/A	0	#N/A	0	#N/A
#N/A	0	#N/A	0	#N/A	0	#N/A
#N/A	0	#N/A	0	#N/A	0	#N/A
#N/A	0	#N/A	0	#N/A	0	#N/A
#N/A	0	#N/A	0	#N/A	0	#N/A
#N/A	0	#N/A	0	#N/A	0	#N/A
#N/A	0	#N/A	0	#N/A	0	#N/A
	0		0	Ew/a-LOST-cit	1	Ew/p-LOST-cit
	0		0	Ew/a-LOST-cit	1	Ew/p-LOST-cit
#N/A	0	#N/A	0	#N/A	0	#N/A
#N/A	0	#N/A	0	#N/A	0	#N/A
#N/A	0	#N/A	0	#N/A	0	#N/A
#N/A	0	#N/A	0	#N/A	0	#N/A
#N/A	0	#N/A	0	#N/A	0	#N/A
#N/A	0	#N/A	0	#N/A	0	#N/A
#N/A	0	#N/A	0	#N/A	0	#N/A
#N/A	0	#N/A	0	#N/A	0	#N/A
#N/A	0	#N/A	0	#N/A	0	#N/A
#N/A	0	#N/A	0	#N/A	0	#N/A
#N/A	0	#N/A	0	#N/A	0	#N/A
#N/A	0	#N/A	0	#N/A	0	#N/A
	0		0	Ew/a-LOST-cit	1	Ew/p-LOST-cit
#N/A	0	#N/A	0	#N/A	0	#N/A
#N/A	0	#N/A	0	#N/A	0	#N/A
#N/A	0	#N/A	0	#N/A	0	#N/A

LOST-6_cit	1		0		0	
LOST-6_cit	1		0		0	
#N/A	0	#N/A	0	#N/A	0	#N/A
#N/A	0	#N/A	0	#N/A	0	#N/A
#N/A	0	#N/A	0	#N/A	0	#N/A
#N/A	0	#N/A	0	#N/A	0	#N/A
#N/A	0	#N/A	0	#N/A	0	#N/A
#N/A	0	#N/A	0	#N/A	0	#N/A
#N/A	0	#N/A	0	#N/A	0	#N/A
#N/A	0	#N/A	0	#N/A	0	#N/A
#N/A	0	#N/A	0	#N/A	0	#N/A
#N/A	0	#N/A	0	#N/A	0	#N/A
#N/A	0	#N/A	0	#N/A	0	#N/A
#N/A	0	#N/A	0	#N/A	0	#N/A
#N/A	0	#N/A	0	#N/A	0	#N/A
#N/A	0	#N/A	0	#N/A	0	#N/A
#N/A	0	#N/A	0	#N/A	0	#N/A
#N/A	0	#N/A	0	#N/A	0	#N/A
#N/A	0	#N/A	0	#N/A	0	#N/A
#N/A	0	#N/A	0	#N/A	0	#N/A
#N/A	0	#N/A	0	#N/A	0	#N/A
#N/A	0	#N/A	0	#N/A	0	#N/A
#N/A	0	#N/A	0	#N/A	0	#N/A
LOST-6_cit	1	LOST>6_cit	1	Ew/a-LOST-cit	1	
LOST-6_cit	1	LOST>6_cit	1	Ew/a-LOST-cit	1	
LOST-6_cit	1	LOST>6_cit	1	Ew/a-LOST-cit	1	
#N/A	0	#N/A	0	#N/A	0	#N/A
#N/A	0	#N/A	0	#N/A	0	#N/A
#N/A	0	#N/A	0	#N/A	0	#N/A
#N/A	0	#N/A	0	#N/A	0	#N/A
#N/A	0	#N/A	0	#N/A	0	#N/A
#N/A	0	#N/A	0	#N/A	0	#N/A
#N/A	0	#N/A	0	#N/A	0	#N/A
#N/A	0	#N/A	0	#N/A	0	#N/A
#N/A	0	#N/A	0	#N/A	0	#N/A
#N/A	0	#N/A	0	#N/A	0	#N/A
	0		0	Ew/a-LOST-cit	1	Ew/p-LOST-cit
	0		0	Ew/a-LOST-cit	1	Ew/p-LOST-cit
#N/A	0	#N/A	0	#N/A	0	#N/A
#N/A	0	#N/A	0	#N/A	0	#N/A
#N/A	0	#N/A	0	#N/A	0	#N/A
#N/A	0	#N/A	0	#N/A	0	#N/A
LOST-6_cit	1		0		0	
#N/A	0	#N/A	0	#N/A	0	#N/A
#N/A	0	#N/A	0	#N/A	0	#N/A
#N/A	0	#N/A	0	#N/A	0	#N/A
#N/A	0	#N/A	0	#N/A	0	#N/A

#N/A	0	#N/A	0	#N/A	0	#N/A
#N/A	0	#N/A	0	#N/A	0	#N/A
LOST-6_cit	1		0		0	
#N/A	0	#N/A	0	#N/A	0	#N/A
#N/A	0	#N/A	0	#N/A	0	#N/A
#N/A	0	#N/A	0	#N/A	0	#N/A
#N/A	0	#N/A	0	#N/A	0	#N/A
#N/A	0	#N/A	0	#N/A	0	#N/A
#N/A	0	#N/A	0	#N/A	0	#N/A
#N/A	0	#N/A	0	#N/A	0	#N/A
LOST-6_cit	1		0		0	Ew/p-LOST-cit
LOST-6_cit	1		0		0	Ew/p-LOST-cit
LOST-6_cit	1		0		0	Ew/p-LOST-cit
#N/A	0	#N/A	0	#N/A	0	#N/A
#N/A	0	#N/A	0	#N/A	0	#N/A
#N/A	0	#N/A	0	#N/A	0	#N/A
#N/A	0	#N/A	0	#N/A	0	#N/A
#N/A	0	#N/A	0	#N/A	0	#N/A
#N/A	0	#N/A	0	#N/A	0	#N/A
#N/A	0	#N/A	0	#N/A	0	#N/A
#N/A	0	#N/A	0	#N/A	0	#N/A
#N/A	0	#N/A	0	#N/A	0	#N/A
#N/A	0	#N/A	0	#N/A	0	#N/A
#N/A	0	#N/A	0	#N/A	0	#N/A
#N/A	0	#N/A	0	#N/A	0	#N/A
#N/A	0	#N/A	0	#N/A	0	#N/A
#N/A	0	#N/A	0	#N/A	0	#N/A
#N/A	0	#N/A	0	#N/A	0	#N/A
#N/A	0	#N/A	0	#N/A	0	#N/A
#N/A	0	#N/A	0	#N/A	0	#N/A
#N/A	0	#N/A	0	#N/A	0	#N/A
#N/A	0	#N/A	0	#N/A	0	#N/A
#N/A	0	#N/A	0	#N/A	0	#N/A
#N/A	0	#N/A	0	#N/A	0	#N/A
#N/A	0	#N/A	0	#N/A	0	#N/A
#N/A	0	#N/A	0	#N/A	0	#N/A
#N/A	0	#N/A	0	#N/A	0	#N/A
#N/A	0	#N/A	0	#N/A	0	#N/A
	0	LOST>6_cit	1		0	
#N/A	0	#N/A	0	#N/A	0	#N/A
#N/A	0	#N/A	0	#N/A	0	#N/A
#N/A	0	#N/A	0	#N/A	0	#N/A
#N/A	0	#N/A	0	#N/A	0	#N/A
#N/A	0	#N/A	0	#N/A	0	#N/A
#N/A	0	#N/A	0	#N/A	0	#N/A
#N/A	0	#N/A	0	#N/A	0	#N/A

#N/A	0	#N/A	0	#N/A	0	#N/A
#N/A	0	#N/A	0	#N/A	0	#N/A
#N/A	0	#N/A	0	#N/A	0	#N/A
#N/A	0	#N/A	0	#N/A	0	#N/A
#N/A	0	#N/A	0	#N/A	0	#N/A
#N/A	0	#N/A	0	#N/A	0	#N/A
#N/A	0	#N/A	0	#N/A	0	#N/A
#N/A	0	#N/A	0	#N/A	0	#N/A
#N/A	0	#N/A	0	#N/A	0	#N/A
#N/A	0	#N/A	0	#N/A	0	#N/A
#N/A	0	#N/A	0	#N/A	0	#N/A
#N/A	0	#N/A	0	#N/A	0	#N/A
#N/A	0	#N/A	0	#N/A	0	#N/A
#N/A	0	#N/A	0	#N/A	0	#N/A
#N/A	0	#N/A	0	#N/A	0	#N/A
#N/A	0	#N/A	0	#N/A	0	#N/A
#N/A	0	#N/A	0	#N/A	0	#N/A
#N/A	0	#N/A	0	#N/A	0	#N/A
#N/A	0	#N/A	0	#N/A	0	#N/A
#N/A	0	#N/A	0	#N/A	0	#N/A
#N/A	0	#N/A	0	#N/A	0	#N/A
#N/A	0	#N/A	0	#N/A	0	#N/A
#N/A	0	#N/A	0	#N/A	0	#N/A
#N/A	0	#N/A	0	#N/A	0	#N/A
#N/A	0	#N/A	0	#N/A	0	#N/A
#N/A	0	#N/A	0	#N/A	0	#N/A
#N/A	0	#N/A	0	#N/A	0	#N/A
#N/A	0	#N/A	0	#N/A	0	#N/A
#N/A	0	#N/A	0	#N/A	0	#N/A
#N/A	0	#N/A	0	#N/A	0	#N/A
#N/A	0	#N/A	0	#N/A	0	#N/A
#N/A	0	#N/A	0	#N/A	0	#N/A
#N/A	0	#N/A	0	#N/A	0	#N/A
#N/A	0	#N/A	0	#N/A	0	#N/A
#N/A	0	#N/A	0	#N/A	0	#N/A
#N/A	0	#N/A	0	#N/A	0	#N/A
#N/A	0	#N/A	0	#N/A	0	#N/A
#N/A	0	#N/A	0	#N/A	0	#N/A
#N/A	0	#N/A	0	#N/A	0	#N/A
#N/A	0	#N/A	0	#N/A	0	#N/A
#N/A	0	#N/A	0	#N/A	0	#N/A
#N/A	0	#N/A	0	#N/A	0	#N/A
LOST-6_cit	1		0		0	
LOST-6_cit	1		0		0	
#N/A	0	#N/A	0	#N/A	0	#N/A
LOST-6_cit	1		0		0	Ew/p-LOST-cit
#N/A	0	#N/A	0	#N/A	0	#N/A

#N/A	0	#N/A	0	#N/A	0	#N/A
#N/A	0	#N/A	0	#N/A	0	#N/A
#N/A	0	#N/A	0	#N/A	0	#N/A
#N/A	0	#N/A	0	#N/A	0	#N/A
#N/A	0	#N/A	0	#N/A	0	#N/A
#N/A	0	#N/A	0	#N/A	0	#N/A
	0		0	Ew/a-LOST-cit	1	Ew/p-LOST-cit
	0		0	Ew/a-LOST-cit	1	Ew/p-LOST-cit
	0		0	Ew/a-LOST-cit	1	Ew/p-LOST-cit
	0		0	Ew/a-LOST-cit	1	Ew/p-LOST-cit
#N/A	0	#N/A	0	#N/A	0	#N/A
#N/A	0	#N/A	0	#N/A	0	#N/A
#N/A	0	#N/A	0	#N/A	0	#N/A
#N/A	0	#N/A	0	#N/A	0	#N/A
#N/A	0	#N/A	0	#N/A	0	#N/A
#N/A	0	#N/A	0	#N/A	0	#N/A
#N/A	0	#N/A	0	#N/A	0	#N/A
LOST-6_cit	1		0		0	Ew/p-LOST-cit
LOST-6_cit	1		0		0	Ew/p-LOST-cit
	0		0		0	
	0		0		0	
#N/A	0	#N/A	0	#N/A	0	#N/A
#N/A	0	#N/A	0	#N/A	0	#N/A
	0	LOST>6_cit	1	Ew/a-LOST-cit	1	Ew/p-LOST-cit
#N/A	0	#N/A	0	#N/A	0	#N/A
#N/A	0	#N/A	0	#N/A	0	#N/A
#N/A	0	#N/A	0	#N/A	0	#N/A
#N/A	0	#N/A	0	#N/A	0	#N/A
#N/A	0	#N/A	0	#N/A	0	#N/A
#N/A	0	#N/A	0	#N/A	0	#N/A
#N/A	0	#N/A	0	#N/A	0	#N/A
	0		0	Ew/a-LOST-cit	1	Ew/p-LOST-cit
	0		0	Ew/a-LOST-cit	1	Ew/p-LOST-cit
#N/A	0	#N/A	0	#N/A	0	#N/A
#N/A	0	#N/A	0	#N/A	0	#N/A
#N/A	0	#N/A	0	#N/A	0	#N/A
LOST-6_cit	1		0	Ew/a-LOST-cit	1	Ew/p-LOST-cit
LOST-6_cit	1		0	Ew/a-LOST-cit	1	Ew/p-LOST-cit
#N/A	0	#N/A	0	#N/A	0	#N/A
#N/A	0	#N/A	0	#N/A	0	#N/A
#N/A	0	#N/A	0	#N/A	0	#N/A
#N/A	0	#N/A	0	#N/A	0	#N/A
#N/A	0	#N/A	0	#N/A	0	#N/A
#N/A	0	#N/A	0	#N/A	0	#N/A
#N/A	0	#N/A	0	#N/A	0	#N/A
#N/A	0	#N/A	0	#N/A	0	#N/A
#N/A	0	#N/A	0	#N/A	0	#N/A

#N/A	0	#N/A	0	#N/A	0	#N/A
#N/A	0	#N/A	0	#N/A	0	#N/A
	0	LOST>6_cit	1		0	
	0	LOST>6_cit	1		0	
#N/A	0	#N/A	0	#N/A	0	#N/A
#N/A	0	#N/A	0	#N/A	0	#N/A
#N/A	0	#N/A	0	#N/A	0	#N/A
#N/A	0	#N/A	0	#N/A	0	#N/A
#N/A	0	#N/A	0	#N/A	0	#N/A
#N/A	0	#N/A	0	#N/A	0	#N/A
#N/A	0	#N/A	0	#N/A	0	#N/A
#N/A	0	#N/A	0	#N/A	0	#N/A
#N/A	0	#N/A	0	#N/A	0	#N/A
#N/A	0	#N/A	0	#N/A	0	#N/A
#N/A	0	#N/A	0	#N/A	0	#N/A
LOST-6_cit	1		0	Ew/a-LOST-cit	1	Ew/p-LOST-cit
	0		0		0	
	0		0		0	
	0	LOST>6_cit	1		0	
	0		0	Ew/a-LOST-cit	1	
	0		0		0	
	0		0		0	
#N/A	0	#N/A	0	#N/A	0	#N/A
#N/A	0	#N/A	0	#N/A	0	#N/A
#N/A	0	#N/A	0	#N/A	0	#N/A
#N/A	0	#N/A	0	#N/A	0	#N/A
#N/A	0	#N/A	0	#N/A	0	#N/A
#N/A	0	#N/A	0	#N/A	0	#N/A
#N/A	0	#N/A	0	#N/A	0	#N/A
#N/A	0	#N/A	0	#N/A	0	#N/A
LOST-6_cit	1	LOST>6_cit	1		0	
LOST-6_cit	1	LOST>6_cit	1		0	
#N/A	0	#N/A	0	#N/A	0	#N/A
LOST-6_cit	1		0	Ew/a-LOST-cit	1	Ew/p-LOST-cit
LOST-6_cit	1		0	Ew/a-LOST-cit	1	Ew/p-LOST-cit
LOST-6_cit	1		0	Ew/a-LOST-cit	1	Ew/p-LOST-cit
LOST-6_cit	1		0	Ew/a-LOST-cit	1	Ew/p-LOST-cit
#N/A	0	#N/A	0	#N/A	0	#N/A
#N/A	0	#N/A	0	#N/A	0	#N/A
	0		0	Ew/a-LOST-cit	1	
#N/A	0	#N/A	0	#N/A	0	#N/A
	0		0		0	
LOST-6_cit	1		0		0	
LOST-6_cit	1		0		0	
#N/A	0	#N/A	0	#N/A	0	#N/A
#N/A	0	#N/A	0	#N/A	0	#N/A
#N/A	0	#N/A	0	#N/A	0	#N/A

#N/A	0	#N/A	0	#N/A	0	#N/A
#N/A	0	#N/A	0	#N/A	0	#N/A
#N/A	0	#N/A	0	#N/A	0	#N/A
#N/A	0	#N/A	0	#N/A	0	#N/A
#N/A	0	#N/A	0	#N/A	0	#N/A
#N/A	0	#N/A	0	#N/A	0	#N/A
#N/A	0	#N/A	0	#N/A	0	#N/A
#N/A	0	#N/A	0	#N/A	0	#N/A
	0		0	Ew/a-LOST-cit	1	Ew/p-LOST-cit
#N/A	0	#N/A	0	#N/A	0	#N/A
#N/A	0	#N/A	0	#N/A	0	#N/A
#N/A	0	#N/A	0	#N/A	0	#N/A
#N/A	0	#N/A	0	#N/A	0	#N/A
#N/A	0	#N/A	0	#N/A	0	#N/A
#N/A	0	#N/A	0	#N/A	0	#N/A
#N/A	0	#N/A	0	#N/A	0	#N/A
#N/A	0	#N/A	0	#N/A	0	#N/A
#N/A	0	#N/A	0	#N/A	0	#N/A
#N/A	0	#N/A	0	#N/A	0	#N/A
#N/A	0	#N/A	0	#N/A	0	#N/A
#N/A	0	#N/A	0	#N/A	0	#N/A
	0		0	Ew/a-LOST-cit	1	Ew/p-LOST-cit
	0		0	Ew/a-LOST-cit	1	Ew/p-LOST-cit
LOST-6_cit	1	LOST>6_cit	1		0	
#N/A	0	#N/A	0	#N/A	0	#N/A
#N/A	0	#N/A	0	#N/A	0	#N/A
#N/A	0	#N/A	0	#N/A	0	#N/A
#N/A	0	#N/A	0	#N/A	0	#N/A
#N/A	0	#N/A	0	#N/A	0	#N/A
	0	LOST>6_cit	1		0	
	0	LOST>6_cit	1		0	
#N/A	0	#N/A	0	#N/A	0	#N/A
#N/A	0	#N/A	0	#N/A	0	#N/A
LOST-6_cit	1		0	Ew/a-LOST-cit	1	Ew/p-LOST-cit
LOST-6_cit	1		0	Ew/a-LOST-cit	1	Ew/p-LOST-cit
#N/A	0	#N/A	0	#N/A	0	#N/A
#N/A	0	#N/A	0	#N/A	0	#N/A
LOST-6_cit	1		0		0	
	0		0	Ew/a-LOST-cit	1	
	0		0	Ew/a-LOST-cit	1	
#N/A	0	#N/A	0	#N/A	0	#N/A
LOST-6_cit	1		0		0	Ew/p-LOST-cit
#N/A	0	#N/A	0	#N/A	0	#N/A
#N/A	0	#N/A	0	#N/A	0	#N/A
#N/A	0	#N/A	0	#N/A	0	#N/A
#N/A	0	#N/A	0	#N/A	0	#N/A
#N/A	0	#N/A	0	#N/A	0	#N/A

LOST-6_cit	1	LOST>6_cit	1		0	
#N/A	0	#N/A	0	#N/A	0	#N/A
#N/A	0	#N/A	0	#N/A	0	#N/A
#N/A	0	#N/A	0	#N/A	0	#N/A
#N/A	0	#N/A	0	#N/A	0	#N/A
#N/A	0	#N/A	0	#N/A	0	#N/A
#N/A	0	#N/A	0	#N/A	0	#N/A
	0		0		0	
	0		0		0	
	0		0		0	Ew/p-LOST-cit
	0		0		0	Ew/p-LOST-cit
#N/A	0	#N/A	0	#N/A	0	#N/A
#N/A	0	#N/A	0	#N/A	0	#N/A
LOST-6_cit	1		0		0	
	0	LOST>6_cit	1		0	
LOST-6_cit	1		0		0	Ew/p-LOST-cit
LOST-6_cit	1		0		0	Ew/p-LOST-cit
#N/A	0	#N/A	0	#N/A	0	#N/A
#N/A	0	#N/A	0	#N/A	0	#N/A
#N/A	0	#N/A	0	#N/A	0	#N/A
#N/A	0	#N/A	0	#N/A	0	#N/A
#N/A	0	#N/A	0	#N/A	0	#N/A
#N/A	0	#N/A	0	#N/A	0	#N/A
#N/A	0	#N/A	0	#N/A	0	#N/A
	0		0		0	
#N/A	0	#N/A	0	#N/A	0	#N/A
#N/A	0	#N/A	0	#N/A	0	#N/A
#N/A	0	#N/A	0	#N/A	0	#N/A
#N/A	0	#N/A	0	#N/A	0	#N/A
#N/A	0	#N/A	0	#N/A	0	#N/A
#N/A	0	#N/A	0	#N/A	0	#N/A
#N/A	0	#N/A	0	#N/A	0	#N/A
#N/A	0	#N/A	0	#N/A	0	#N/A
#N/A	0	#N/A	0	#N/A	0	#N/A
#N/A	0	#N/A	0	#N/A	0	#N/A
#N/A	0	#N/A	0	#N/A	0	#N/A
#N/A	0	#N/A	0	#N/A	0	#N/A
LOST-6_cit	1		0		0	
#N/A	0	#N/A	0	#N/A	0	#N/A
	0	LOST>6_cit	1		0	
	0	LOST>6_cit	1		0	
#N/A	0	#N/A	0	#N/A	0	#N/A
#N/A	0	#N/A	0	#N/A	0	#N/A
#N/A	0	#N/A	0	#N/A	0	#N/A
#N/A	0	#N/A	0	#N/A	0	#N/A
#N/A	0	#N/A	0	#N/A	0	#N/A
#N/A	0	#N/A	0	#N/A	0	#N/A

#N/A	0	#N/A	0	#N/A	0	#N/A
#N/A	0	#N/A	0	#N/A	0	#N/A
#N/A	0	#N/A	0	#N/A	0	#N/A
#N/A	0	#N/A	0	#N/A	0	#N/A
#N/A	0	#N/A	0	#N/A	0	#N/A
	0		0	Ew/a-LOST-cit	1	
	0		0	Ew/a-LOST-cit	1	Ew/p-LOST-cit
	0		0	Ew/a-LOST-cit	1	Ew/p-LOST-cit
#N/A	0	#N/A	0	#N/A	0	#N/A
#N/A	0	#N/A	0	#N/A	0	#N/A
#N/A	0	#N/A	0	#N/A	0	#N/A
#N/A	0	#N/A	0	#N/A	0	#N/A
#N/A	0	#N/A	0	#N/A	0	#N/A
#N/A	0	#N/A	0	#N/A	0	#N/A
#N/A	0	#N/A	0	#N/A	0	#N/A
#N/A	0	#N/A	0	#N/A	0	#N/A
#N/A	0	#N/A	0	#N/A	0	#N/A
#N/A	0	#N/A	0	#N/A	0	#N/A
#N/A	0	#N/A	0	#N/A	0	#N/A
	0		0	Ew/a-LOST-cit	1	Ew/p-LOST-cit
	0		0	Ew/a-LOST-cit	1	Ew/p-LOST-cit
	0		0	Ew/a-LOST-cit	1	Ew/p-LOST-cit
	0		0	Ew/a-LOST-cit	1	Ew/p-LOST-cit
	0		0	Ew/a-LOST-cit	1	Ew/p-LOST-cit
#N/A	0	#N/A	0	#N/A	0	#N/A
#N/A	0	#N/A	0	#N/A	0	#N/A
	0		0	Ew/a-LOST-cit	1	Ew/p-LOST-cit
	0		0	Ew/a-LOST-cit	1	Ew/p-LOST-cit
	0		0	Ew/a-LOST-cit	1	Ew/p-LOST-cit
#N/A	0	#N/A	0	#N/A	0	#N/A
#N/A	0	#N/A	0	#N/A	0	#N/A
	0	LOST>6_cit	1		0	
#N/A	0	#N/A	0	#N/A	0	#N/A
LOST-6_cit	1	LOST>6_cit	1		0	
#N/A	0	#N/A	0	#N/A	0	#N/A
	0		0		0	
	0		0	Ew/a-LOST-cit	1	
	0		0	Ew/a-LOST-cit	1	
#N/A	0	#N/A	0	#N/A	0	#N/A
#N/A	0	#N/A	0	#N/A	0	#N/A
#N/A	0	#N/A	0	#N/A	0	#N/A
#N/A	0	#N/A	0	#N/A	0	#N/A
#N/A	0	#N/A	0	#N/A	0	#N/A
#N/A	0	#N/A	0	#N/A	0	#N/A
#N/A	0	#N/A	0	#N/A	0	#N/A
#N/A	0	#N/A	0	#N/A	0	#N/A
#N/A	0	#N/A	0	#N/A	0	#N/A

#N/A	0	#N/A	0	#N/A	0	#N/A
#N/A	0	#N/A	0	#N/A	0	#N/A
#N/A	0	#N/A	0	#N/A	0	#N/A
LOST-6_cit	1	LOST>6_cit	1	Ew/a-LOST-cit	1	Ew/p-LOST-cit
#N/A	0	#N/A	0	#N/A	0	#N/A
#N/A	0	#N/A	0	#N/A	0	#N/A
#N/A	0	#N/A	0	#N/A	0	#N/A
#N/A	0	#N/A	0	#N/A	0	#N/A
#N/A	0	#N/A	0	#N/A	0	#N/A
#N/A	0	#N/A	0	#N/A	0	#N/A
	0		0		0	
#N/A	0	#N/A	0	#N/A	0	#N/A
#N/A	0	#N/A	0	#N/A	0	#N/A
#N/A	0	#N/A	0	#N/A	0	#N/A
#N/A	0	#N/A	0	#N/A	0	#N/A
#N/A	0	#N/A	0	#N/A	0	#N/A
#N/A	0	#N/A	0	#N/A	0	#N/A
#N/A	0	#N/A	0	#N/A	0	#N/A
#N/A	0	#N/A	0	#N/A	0	#N/A
#N/A	0	#N/A	0	#N/A	0	#N/A
#N/A	0	#N/A	0	#N/A	0	#N/A
#N/A	0	#N/A	0	#N/A	0	#N/A
#N/A	0	#N/A	0	#N/A	0	#N/A
#N/A	0	#N/A	0	#N/A	0	#N/A
LOST-6_cit	1	LOST>6_cit	1		0	
#N/A	0	#N/A	0	#N/A	0	#N/A
#N/A	0	#N/A	0	#N/A	0	#N/A
#N/A	0	#N/A	0	#N/A	0	#N/A
#N/A	0	#N/A	0	#N/A	0	#N/A
#N/A	0	#N/A	0	#N/A	0	#N/A
#N/A	0	#N/A	0	#N/A	0	#N/A
#N/A	0	#N/A	0	#N/A	0	#N/A
#N/A	0	#N/A	0	#N/A	0	#N/A
#N/A	0	#N/A	0	#N/A	0	#N/A
	0		0	Ew/a-LOST-cit	1	Ew/p-LOST-cit
#N/A	0	#N/A	0	#N/A	0	#N/A
#N/A	0	#N/A	0	#N/A	0	#N/A
#N/A	0	#N/A	0	#N/A	0	#N/A
#N/A	0	#N/A	0	#N/A	0	#N/A
#N/A	0	#N/A	0	#N/A	0	#N/A
#N/A	0	#N/A	0	#N/A	0	#N/A
#N/A	0	#N/A	0	#N/A	0	#N/A
#N/A	0	#N/A	0	#N/A	0	#N/A
	0		0		0	
	0		0		0	
#N/A	0	#N/A	0	#N/A	0	#N/A
#N/A	0	#N/A	0	#N/A	0	#N/A

#N/A	0	#N/A	0	#N/A	0	#N/A
#N/A	0	#N/A	0	#N/A	0	#N/A
#N/A	0	#N/A	0	#N/A	0	#N/A
#N/A	0	#N/A	0	#N/A	0	#N/A
#N/A	0	#N/A	0	#N/A	0	#N/A
LOST-6_cit	1	LOST>6_cit	1	Ew/a-LOST-cit	1	
#N/A	0	#N/A	0	#N/A	0	#N/A
#N/A	0	#N/A	0	#N/A	0	#N/A
#N/A	0	#N/A	0	#N/A	0	#N/A
#N/A	0	#N/A	0	#N/A	0	#N/A
#N/A	0	#N/A	0	#N/A	0	#N/A
#N/A	0	#N/A	0	#N/A	0	#N/A
#N/A	0	#N/A	0	#N/A	0	#N/A
#N/A	0	#N/A	0	#N/A	0	#N/A
#N/A	0	#N/A	0	#N/A	0	#N/A
	0		0		0	Ew/p-LOST-cit
#N/A	0	#N/A	0	#N/A	0	#N/A
#N/A	0	#N/A	0	#N/A	0	#N/A
#N/A	0	#N/A	0	#N/A	0	#N/A
#N/A	0	#N/A	0	#N/A	0	#N/A
	0	LOST>6_cit	1		0	
	0	LOST>6_cit	1		0	
#N/A	0	#N/A	0	#N/A	0	#N/A
#N/A	0	#N/A	0	#N/A	0	#N/A
#N/A	0	#N/A	0	#N/A	0	#N/A
#N/A	0	#N/A	0	#N/A	0	#N/A
#N/A	0	#N/A	0	#N/A	0	#N/A
#N/A	0	#N/A	0	#N/A	0	#N/A
#N/A	0	#N/A	0	#N/A	0	#N/A
#N/A	0	#N/A	0	#N/A	0	#N/A
#N/A	0	#N/A	0	#N/A	0	#N/A
#N/A	0	#N/A	0	#N/A	0	#N/A
#N/A	0	#N/A	0	#N/A	0	#N/A
#N/A	0	#N/A	0	#N/A	0	#N/A
#N/A	0	#N/A	0	#N/A	0	#N/A
#N/A	0	#N/A	0	#N/A	0	#N/A
	0		0	Ew/a-LOST-cit	1	
	0		0	Ew/a-LOST-cit	1	
	0		0	Ew/a-LOST-cit	1	Ew/p-LOST-cit
	0		0	Ew/a-LOST-cit	1	Ew/p-LOST-cit
	0	LOST>6_cit	1		0	
#N/A	0	#N/A	0	#N/A	0	#N/A
#N/A	0	#N/A	0	#N/A	0	#N/A
#N/A	0	#N/A	0	#N/A	0	#N/A
#N/A	0	#N/A	0	#N/A	0	#N/A
#N/A	0	#N/A	0	#N/A	0	#N/A
LOST-6_cit	1	LOST>6_cit	1		0	

LOST-6_cit	1	LOST>6_cit	1		0	
#N/A	0	#N/A	0	#N/A	0	#N/A
#N/A	0	#N/A	0	#N/A	0	#N/A
#N/A	0	#N/A	0	#N/A	0	#N/A
#N/A	0	#N/A	0	#N/A	0	#N/A
#N/A	0	#N/A	0	#N/A	0	#N/A
#N/A	0	#N/A	0	#N/A	0	#N/A
#N/A	0	#N/A	0	#N/A	0	#N/A
	0	LOST>6_cit	1		0	
	0	LOST>6_cit	1		0	
	0	LOST>6_cit	1		0	
#N/A	0	#N/A	0	#N/A	0	#N/A
#N/A	0	#N/A	0	#N/A	0	#N/A
#N/A	0	#N/A	0	#N/A	0	#N/A
#N/A	0	#N/A	0	#N/A	0	#N/A
#N/A	0	#N/A	0	#N/A	0	#N/A
#N/A	0	#N/A	0	#N/A	0	#N/A
#N/A	0	#N/A	0	#N/A	0	#N/A
#N/A	0	#N/A	0	#N/A	0	#N/A
#N/A	0	#N/A	0	#N/A	0	#N/A
#N/A	0	#N/A	0	#N/A	0	#N/A
	0		0	Ew/a-LOST-cit	1	
#N/A	0	#N/A	0	#N/A	0	#N/A
#N/A	0	#N/A	0	#N/A	0	#N/A
#N/A	0	#N/A	0	#N/A	0	#N/A
#N/A	0	#N/A	0	#N/A	0	#N/A
#N/A	0	#N/A	0	#N/A	0	#N/A
#N/A	0	#N/A	0	#N/A	0	#N/A
#N/A	0	#N/A	0	#N/A	0	#N/A
#N/A	0	#N/A	0	#N/A	0	#N/A
#N/A	0	#N/A	0	#N/A	0	#N/A
#N/A	0	#N/A	0	#N/A	0	#N/A
#N/A	0	#N/A	0	#N/A	0	#N/A
#N/A	0	#N/A	0	#N/A	0	#N/A
#N/A	0	#N/A	0	#N/A	0	#N/A
#N/A	0	#N/A	0	#N/A	0	#N/A
#N/A	0	#N/A	0	#N/A	0	#N/A
#N/A	0	#N/A	0	#N/A	0	#N/A
#N/A	0	#N/A	0	#N/A	0	#N/A
#N/A	0	#N/A	0	#N/A	0	#N/A
#N/A	0	#N/A	0	#N/A	0	#N/A
#N/A	0	#N/A	0	#N/A	0	#N/A
#N/A	0	#N/A	0	#N/A	0	#N/A
LOST-6_cit	1		0	Ew/a-LOST-cit	1	
LOST-6_cit	1		0	Ew/a-LOST-cit	1	
LOST-6_cit	1		0	Ew/a-LOST-cit	1	
	0		0	Ew/a-LOST-cit	1	

	0		0	Ew/a-LOST-cit	1	
#N/A	0	#N/A	0	#N/A	0	#N/A
#N/A	0	#N/A	0	#N/A	0	#N/A
#N/A	0	#N/A	0	#N/A	0	#N/A
#N/A	0	#N/A	0	#N/A	0	#N/A
#N/A	0	#N/A	0	#N/A	0	#N/A
#N/A	0	#N/A	0	#N/A	0	#N/A
#N/A	0	#N/A	0	#N/A	0	#N/A
#N/A	0	#N/A	0	#N/A	0	#N/A
	0		0		0	
#N/A	0	#N/A	0	#N/A	0	#N/A
	0		0	Ew/a-LOST-cit	1	Ew/p-LOST-cit
	0		0	Ew/a-LOST-cit	1	Ew/p-LOST-cit
	0		0	Ew/a-LOST-cit	1	Ew/p-LOST-cit
#N/A	0	#N/A	0	#N/A	0	#N/A
#N/A	0	#N/A	0	#N/A	0	#N/A
#N/A	0	#N/A	0	#N/A	0	#N/A
#N/A	0	#N/A	0	#N/A	0	#N/A
#N/A	0	#N/A	0	#N/A	0	#N/A
#N/A	0	#N/A	0	#N/A	0	#N/A
#N/A	0	#N/A	0	#N/A	0	#N/A
#N/A	0	#N/A	0	#N/A	0	#N/A
#N/A	0	#N/A	0	#N/A	0	#N/A
#N/A	0	#N/A	0	#N/A	0	#N/A
	0		0	Ew/a-LOST-cit	1	Ew/p-LOST-cit
	0		0	Ew/a-LOST-cit	1	Ew/p-LOST-cit
	0		0	Ew/a-LOST-cit	1	Ew/p-LOST-cit
	0		0	Ew/a-LOST-cit	1	Ew/p-LOST-cit
#N/A	0	#N/A	0	#N/A	0	#N/A
#N/A	0	#N/A	0	#N/A	0	#N/A
#N/A	0	#N/A	0	#N/A	0	#N/A
#N/A	0	#N/A	0	#N/A	0	#N/A
#N/A	0	#N/A	0	#N/A	0	#N/A
#N/A	0	#N/A	0	#N/A	0	#N/A
#N/A	0	#N/A	0	#N/A	0	#N/A
#N/A	0	#N/A	0	#N/A	0	#N/A
#N/A	0	#N/A	0	#N/A	0	#N/A
#N/A	0	#N/A	0	#N/A	0	#N/A
#N/A	0	#N/A	0	#N/A	0	#N/A
#N/A	0	#N/A	0	#N/A	0	#N/A
#N/A	0	#N/A	0	#N/A	0	#N/A
#N/A	0	#N/A	0	#N/A	0	#N/A
#N/A	0	#N/A	0	#N/A	0	#N/A
#N/A	0	#N/A	0	#N/A	0	#N/A
#N/A	0	#N/A	0	#N/A	0	#N/A
#N/A	0	#N/A	0	#N/A	0	#N/A
#N/A	0	#N/A	0	#N/A	0	#N/A

LOST-6_cit	1		0		0	
#N/A	0	#N/A	0	#N/A	0	#N/A
#N/A	0	#N/A	0	#N/A	0	#N/A
#N/A	0	#N/A	0	#N/A	0	#N/A
	0		0	Ew/a-LOST-cit	1	
	0		0	Ew/a-LOST-cit	1	
	0		0	Ew/a-LOST-cit	1	
#N/A	0	#N/A	0	#N/A	0	#N/A
#N/A	0	#N/A	0	#N/A	0	#N/A
	0	LOST>6_cit	1		0	
#N/A	0	#N/A	0	#N/A	0	#N/A
#N/A	0	#N/A	0	#N/A	0	#N/A
#N/A	0	#N/A	0	#N/A	0	#N/A
#N/A	0	#N/A	0	#N/A	0	#N/A
#N/A	0	#N/A	0	#N/A	0	#N/A
#N/A	0	#N/A	0	#N/A	0	#N/A
#N/A	0	#N/A	0	#N/A	0	#N/A
#N/A	0	#N/A	0	#N/A	0	#N/A
#N/A	0	#N/A	0	#N/A	0	#N/A
#N/A	0	#N/A	0	#N/A	0	#N/A
#N/A	0	#N/A	0	#N/A	0	#N/A
	0		0		0	
	0		0		0	
	0		0		0	
#N/A	0	#N/A	0	#N/A	0	#N/A
LOST-6_cit	1		0	Ew/a-LOST-cit	1	
#N/A	0	#N/A	0	#N/A	0	#N/A
#N/A	0	#N/A	0	#N/A	0	#N/A
#N/A	0	#N/A	0	#N/A	0	#N/A
#N/A	0	#N/A	0	#N/A	0	#N/A
#N/A	0	#N/A	0	#N/A	0	#N/A
#N/A	0	#N/A	0	#N/A	0	#N/A
#N/A	0	#N/A	0	#N/A	0	#N/A
#N/A	0	#N/A	0	#N/A	0	#N/A
#N/A	0	#N/A	0	#N/A	0	#N/A
#N/A	0	#N/A	0	#N/A	0	#N/A
#N/A	0	#N/A	0	#N/A	0	#N/A
	0		0	Ew/a-LOST-cit	1	
	0		0	Ew/a-LOST-cit	1	
#N/A	0	#N/A	0	#N/A	0	#N/A
#N/A	0	#N/A	0	#N/A	0	#N/A
#N/A	0	#N/A	0	#N/A	0	#N/A
#N/A	0	#N/A	0	#N/A	0	#N/A
#N/A	0	#N/A	0	#N/A	0	#N/A
#N/A	0	#N/A	0	#N/A	0	#N/A
#N/A	0	#N/A	0	#N/A	0	#N/A
#N/A	0	#N/A	0	#N/A	0	#N/A

#N/A	0	#N/A	0	#N/A	0	#N/A
#N/A	0	#N/A	0	#N/A	0	#N/A
#N/A	0	#N/A	0	#N/A	0	#N/A
#N/A	0	#N/A	0	#N/A	0	#N/A
#N/A	0	#N/A	0	#N/A	0	#N/A
#N/A	0	#N/A	0	#N/A	0	#N/A
#N/A	0	#N/A	0	#N/A	0	#N/A
LOST-6_cit	1	LOST>6_cit	1		0	Ew/p-LOST-cit
LOST-6_cit	1	LOST>6_cit	1		0	Ew/p-LOST-cit
	0		0	Ew/a-LOST-cit	1	Ew/p-LOST-cit
	0		0	Ew/a-LOST-cit	1	Ew/p-LOST-cit
	0		0	Ew/a-LOST-cit	1	Ew/p-LOST-cit
#N/A	0	#N/A	0	#N/A	0	#N/A
#N/A	0	#N/A	0	#N/A	0	#N/A
#N/A	0	#N/A	0	#N/A	0	#N/A
LOST-6_cit	1	LOST>6_cit	1		0	
#N/A	0	#N/A	0	#N/A	0	#N/A
#N/A	0	#N/A	0	#N/A	0	#N/A
	0		0	Ew/a-LOST-cit	1	
	0		0	Ew/a-LOST-cit	1	
#N/A	0	#N/A	0	#N/A	0	#N/A
#N/A	0	#N/A	0	#N/A	0	#N/A
	0		0	Ew/a-LOST-cit	1	Ew/p-LOST-cit
	0		0	Ew/a-LOST-cit	1	Ew/p-LOST-cit
	0		0	Ew/a-LOST-cit	1	Ew/p-LOST-cit
#N/A	0	#N/A	0	#N/A	0	#N/A
	0		0	Ew/a-LOST-cit	1	Ew/p-LOST-cit
#N/A	0	#N/A	0	#N/A	0	#N/A
	0		0	Ew/a-LOST-cit	1	Ew/p-LOST-cit
	0		0	Ew/a-LOST-cit	1	Ew/p-LOST-cit
#N/A	0	#N/A	0	#N/A	0	#N/A
LOST-6_cit	1		0	Ew/a-LOST-cit	1	
LOST-6_cit	1		0	Ew/a-LOST-cit	1	Ew/p-LOST-cit
LOST-6_cit	1		0	Ew/a-LOST-cit	1	Ew/p-LOST-cit
	0	LOST>6_cit	1		0	Ew/p-LOST-cit
#N/A	0	#N/A	0	#N/A	0	#N/A
#N/A	0	#N/A	0	#N/A	0	#N/A
#N/A	0	#N/A	0	#N/A	0	#N/A
#N/A	0	#N/A	0	#N/A	0	#N/A
#N/A	0	#N/A	0	#N/A	0	#N/A
#N/A	0	#N/A	0	#N/A	0	#N/A
#N/A	0	#N/A	0	#N/A	0	#N/A
#N/A	0	#N/A	0	#N/A	0	#N/A
#N/A	0	#N/A	0	#N/A	0	#N/A
	0	LOST>6_cit	1		0	
LOST-6_cit	1		0		0	
#N/A	0	#N/A	0	#N/A	0	#N/A

#N/A	0	#N/A	0	#N/A	0	#N/A
#N/A	0	#N/A	0	#N/A	0	#N/A
#N/A	0	#N/A	0	#N/A	0	#N/A
LOST-6_cit	1		0		0	
#N/A	0	#N/A	0	#N/A	0	#N/A
	0		0	Ew/a-LOST-cit	1	Ew/p-LOST-cit
	0		0	Ew/a-LOST-cit	1	Ew/p-LOST-cit
#N/A	0	#N/A	0	#N/A	0	#N/A
#N/A	0	#N/A	0	#N/A	0	#N/A
LOST-6_cit	1		0		0	
LOST-6_cit	1		0		0	
LOST-6_cit	1		0		0	
#N/A	0	#N/A	0	#N/A	0	#N/A
#N/A	0	#N/A	0	#N/A	0	#N/A
#N/A	0	#N/A	0	#N/A	0	#N/A
#N/A	0	#N/A	0	#N/A	0	#N/A
#N/A	0	#N/A	0	#N/A	0	#N/A
#N/A	0	#N/A	0	#N/A	0	#N/A
#N/A	0	#N/A	0	#N/A	0	#N/A
#N/A	0	#N/A	0	#N/A	0	#N/A
#N/A	0	#N/A	0	#N/A	0	#N/A
#N/A	0	#N/A	0	#N/A	0	#N/A
#N/A	0	#N/A	0	#N/A	0	#N/A
#N/A	0	#N/A	0	#N/A	0	#N/A
#N/A	0	#N/A	0	#N/A	0	#N/A
#N/A	0	#N/A	0	#N/A	0	#N/A
#N/A	0	#N/A	0	#N/A	0	#N/A
#N/A	0	#N/A	0	#N/A	0	#N/A
	0		0	Ew/a-LOST-cit	1	Ew/p-LOST-cit
	0		0	Ew/a-LOST-cit	1	Ew/p-LOST-cit
#N/A	0	#N/A	0	#N/A	0	#N/A
	0		0	Ew/a-LOST-cit	1	
#N/A	0	#N/A	0	#N/A	0	#N/A
#N/A	0	#N/A	0	#N/A	0	#N/A
#N/A	0	#N/A	0	#N/A	0	#N/A
#N/A	0	#N/A	0	#N/A	0	#N/A
#N/A	0	#N/A	0	#N/A	0	#N/A
#N/A	0	#N/A	0	#N/A	0	#N/A
#N/A	0	#N/A	0	#N/A	0	#N/A
	0		0	Ew/a-LOST-cit	1	
	0		0	Ew/a-LOST-cit	1	
#N/A	0	#N/A	0	#N/A	0	#N/A
#N/A	0	#N/A	0	#N/A	0	#N/A
#N/A	0	#N/A	0	#N/A	0	#N/A
#N/A	0	#N/A	0	#N/A	0	#N/A
#N/A	0	#N/A	0	#N/A	0	#N/A
#N/A	0	#N/A	0	#N/A	0	#N/A

#N/A	0	#N/A	0	#N/A	0	#N/A
	0		0		0	
	0		0	Ew/a-LOST-cit	1	Ew/p-LOST-cit
	0		0	Ew/a-LOST-cit	1	Ew/p-LOST-cit
#N/A	0	#N/A	0	#N/A	0	#N/A
#N/A	0	#N/A	0	#N/A	0	#N/A
#N/A	0	#N/A	0	#N/A	0	#N/A
#N/A	0	#N/A	0	#N/A	0	#N/A
#N/A	0	#N/A	0	#N/A	0	#N/A
#N/A	0	#N/A	0	#N/A	0	#N/A
#N/A	0	#N/A	0	#N/A	0	#N/A
#N/A	0	#N/A	0	#N/A	0	#N/A
#N/A	0	#N/A	0	#N/A	0	#N/A
LOST-6_cit	1		0	Ew/a-LOST-cit	1	Ew/p-LOST-cit
LOST-6_cit	1		0	Ew/a-LOST-cit	1	Ew/p-LOST-cit
#N/A	0	#N/A	0	#N/A	0	#N/A
	0		0	Ew/a-LOST-cit	1	Ew/p-LOST-cit
	0		0	Ew/a-LOST-cit	1	Ew/p-LOST-cit
#N/A	0	#N/A	0	#N/A	0	#N/A
LOST-6_cit	1		0		0	
#N/A	0	#N/A	0	#N/A	0	#N/A
#N/A	0	#N/A	0	#N/A	0	#N/A
#N/A	0	#N/A	0	#N/A	0	#N/A
#N/A	0	#N/A	0	#N/A	0	#N/A
#N/A	0	#N/A	0	#N/A	0	#N/A
#N/A	0	#N/A	0	#N/A	0	#N/A
#N/A	0	#N/A	0	#N/A	0	#N/A
#N/A	0	#N/A	0	#N/A	0	#N/A
#N/A	0	#N/A	0	#N/A	0	#N/A
#N/A	0	#N/A	0	#N/A	0	#N/A
LOST-6_cit	1	LOST>6_cit	1		0	
LOST-6_cit	1	LOST>6_cit	1		0	
	0		0	Ew/a-LOST-cit	1	Ew/p-LOST-cit
#N/A	0	#N/A	0	#N/A	0	#N/A
	0		0		0	
	0		0		0	
	0		0		0	
LOST-6_cit	1		0		0	Ew/p-LOST-cit
#N/A	0	#N/A	0	#N/A	0	#N/A
#N/A	0	#N/A	0	#N/A	0	#N/A
LOST-6_cit	1	LOST>6_cit	1	Ew/a-LOST-cit	1	
LOST-6_cit	1	LOST>6_cit	1	Ew/a-LOST-cit	1	
LOST-6_cit	1	LOST>6_cit	1	Ew/a-LOST-cit	1	
LOST-6_cit	1	LOST>6_cit	1	Ew/a-LOST-cit	1	
LOST-6_cit	1		0		0	
	0		0	Ew/a-LOST-cit	1	Ew/p-LOST-cit
	0		0	Ew/a-LOST-cit	1	Ew/p-LOST-cit

#N/A	0	#N/A	0	#N/A	0	#N/A
	0	LOST>6_cit	1		0	
LOST-6_cit	1	LOST>6_cit	1		0	
#N/A	0	#N/A	0	#N/A	0	#N/A
#N/A	0	#N/A	0	#N/A	0	#N/A
#N/A	0	#N/A	0	#N/A	0	#N/A
#N/A	0	#N/A	0	#N/A	0	#N/A
#N/A	0	#N/A	0	#N/A	0	#N/A
#N/A	0	#N/A	0	#N/A	0	#N/A
#N/A	0	#N/A	0	#N/A	0	#N/A
#N/A	0	#N/A	0	#N/A	0	#N/A
#N/A	0	#N/A	0	#N/A	0	#N/A
	0		0	Ew/a-LOST-cit	1	Ew/p-LOST-cit
#N/A	0	#N/A	0	#N/A	0	#N/A
#N/A	0	#N/A	0	#N/A	0	#N/A
#N/A	0	#N/A	0	#N/A	0	#N/A
#N/A	0	#N/A	0	#N/A	0	#N/A
LOST-6_cit	1		0	Ew/a-LOST-cit	1	
	0		0	Ew/a-LOST-cit	1	
	0		0	Ew/a-LOST-cit	1	
#N/A	0	#N/A	0	#N/A	0	#N/A
#N/A	0	#N/A	0	#N/A	0	#N/A
#N/A	0	#N/A	0	#N/A	0	#N/A
#N/A	0	#N/A	0	#N/A	0	#N/A
LOST-6_cit	1	LOST>6_cit	1		0	
#N/A	0	#N/A	0	#N/A	0	#N/A
LOST-6_cit	1		0		0	
#N/A	0	#N/A	0	#N/A	0	#N/A
#N/A	0	#N/A	0	#N/A	0	#N/A
#N/A	0	#N/A	0	#N/A	0	#N/A
#N/A	0	#N/A	0	#N/A	0	#N/A
#N/A	0	#N/A	0	#N/A	0	#N/A
#N/A	0	#N/A	0	#N/A	0	#N/A
#N/A	0	#N/A	0	#N/A	0	#N/A
#N/A	0	#N/A	0	#N/A	0	#N/A
LOST-6_cit	1		0	Ew/a-LOST-cit	1	Ew/p-LOST-cit
#N/A	0	#N/A	0	#N/A	0	#N/A
#N/A	0	#N/A	0	#N/A	0	#N/A
#N/A	0	#N/A	0	#N/A	0	#N/A
LOST-6_cit	1	LOST>6_cit	1		0	
#N/A	0	#N/A	0	#N/A	0	#N/A
	0		0		0	
#N/A	0	#N/A	0	#N/A	0	#N/A
#N/A	0	#N/A	0	#N/A	0	#N/A
#N/A	0	#N/A	0	#N/A	0	#N/A
#N/A	0	#N/A	0	#N/A	0	#N/A
#N/A	0	#N/A	0	#N/A	0	#N/A

LOST-6_cit	1		0		0	
#N/A	0	#N/A	0	#N/A	0	#N/A
	0	LOST>6_cit	1		0	
	0		0	Ew/a-LOST-cit	1	
LOST-6_cit	1		0		0	
LOST-6_cit	1		0		0	
#N/A	0	#N/A	0	#N/A	0	#N/A
#N/A	0	#N/A	0	#N/A	0	#N/A
#N/A	0	#N/A	0	#N/A	0	#N/A
#N/A	0	#N/A	0	#N/A	0	#N/A
#N/A	0	#N/A	0	#N/A	0	#N/A
#N/A	0	#N/A	0	#N/A	0	#N/A
#N/A	0	#N/A	0	#N/A	0	#N/A
LOST-6_cit	1		0		0	
LOST-6_cit	1		0	Ew/a-LOST-cit	1	
#N/A	0	#N/A	0	#N/A	0	#N/A
#N/A	0	#N/A	0	#N/A	0	#N/A
#N/A	0	#N/A	0	#N/A	0	#N/A
#N/A	0	#N/A	0	#N/A	0	#N/A
#N/A	0	#N/A	0	#N/A	0	#N/A
#N/A	0	#N/A	0	#N/A	0	#N/A
#N/A	0	#N/A	0	#N/A	0	#N/A
#N/A	0	#N/A	0	#N/A	0	#N/A
	0		0	Ew/a-LOST-cit	1	
#N/A	0	#N/A	0	#N/A	0	#N/A
#N/A	0	#N/A	0	#N/A	0	#N/A
LOST-6_cit	1	LOST>6_cit	1	Ew/a-LOST-cit	1	
#N/A	0	#N/A	0	#N/A	0	#N/A
#N/A	0	#N/A	0	#N/A	0	#N/A
#N/A	0	#N/A	0	#N/A	0	#N/A
#N/A	0	#N/A	0	#N/A	0	#N/A
#N/A	0	#N/A	0	#N/A	0	#N/A
#N/A	0	#N/A	0	#N/A	0	#N/A
#N/A	0	#N/A	0	#N/A	0	#N/A
#N/A	0	#N/A	0	#N/A	0	#N/A
#N/A	0	#N/A	0	#N/A	0	#N/A
#N/A	0	#N/A	0	#N/A	0	#N/A
#N/A	0	#N/A	0	#N/A	0	#N/A
#N/A	0	#N/A	0	#N/A	0	#N/A
#N/A	0	#N/A	0	#N/A	0	#N/A
#N/A	0	#N/A	0	#N/A	0	#N/A
#N/A	0	#N/A	0	#N/A	0	#N/A
#N/A	0	#N/A	0	#N/A	0	#N/A
#N/A	0	#N/A	0	#N/A	0	#N/A
#N/A	0	#N/A	0	#N/A	0	#N/A
#N/A	0	#N/A	0	#N/A	0	#N/A
	0		0	Ew/a-LOST-cit	1	Ew/p-LOST-cit

#N/A	0	#N/A	0	#N/A	0	#N/A
#N/A	0	#N/A	0	#N/A	0	#N/A
#N/A	0	#N/A	0	#N/A	0	#N/A
#N/A	0	#N/A	0	#N/A	0	#N/A
#N/A	0	#N/A	0	#N/A	0	#N/A
#N/A	0	#N/A	0	#N/A	0	#N/A
#N/A	0	#N/A	0	#N/A	0	#N/A
#N/A	0	#N/A	0	#N/A	0	#N/A
#N/A	0	#N/A	0	#N/A	0	#N/A
#N/A	0	#N/A	0	#N/A	0	#N/A
#N/A	0	#N/A	0	#N/A	0	#N/A
#N/A	0	#N/A	0	#N/A	0	#N/A
#N/A	0	#N/A	0	#N/A	0	#N/A
#N/A	0	#N/A	0	#N/A	0	#N/A
#N/A	0	#N/A	0	#N/A	0	#N/A
#N/A	0	#N/A	0	#N/A	0	#N/A
#N/A	0	#N/A	0	#N/A	0	#N/A
	0		0		0	
#N/A	0	#N/A	0	#N/A	0	#N/A
#N/A	0	#N/A	0	#N/A	0	#N/A
#N/A	0	#N/A	0	#N/A	0	#N/A
#N/A	0	#N/A	0	#N/A	0	#N/A
#N/A	0	#N/A	0	#N/A	0	#N/A
#N/A	0	#N/A	0	#N/A	0	#N/A
#N/A	0	#N/A	0	#N/A	0	#N/A
#N/A	0	#N/A	0	#N/A	0	#N/A
#N/A	0	#N/A	0	#N/A	0	#N/A
#N/A	0	#N/A	0	#N/A	0	#N/A
#N/A	0	#N/A	0	#N/A	0	#N/A
#N/A	0	#N/A	0	#N/A	0	#N/A
#N/A	0	#N/A	0	#N/A	0	#N/A
	0		0	Ew/a-LOST-cit	1	
	0		0	Ew/a-LOST-cit	1	
	0		0	Ew/a-LOST-cit	1	
#N/A	0	#N/A	0	#N/A	0	#N/A
#N/A	0	#N/A	0	#N/A	0	#N/A
#N/A	0	#N/A	0	#N/A	0	#N/A
#N/A	0	#N/A	0	#N/A	0	#N/A
#N/A	0	#N/A	0	#N/A	0	#N/A
#N/A	0	#N/A	0	#N/A	0	#N/A
#N/A	0	#N/A	0	#N/A	0	#N/A
#N/A	0	#N/A	0	#N/A	0	#N/A
#N/A	0	#N/A	0	#N/A	0	#N/A
#N/A	0	#N/A	0	#N/A	0	#N/A
#N/A	0	#N/A	0	#N/A	0	#N/A
	0		0		0	
	0		0	Ew/a-LOST-cit	1	Ew/p-LOST-cit

	0		0	Ew/a-LOST-cit	1	Ew/p-LOST-cit	
	0		0	Ew/a-LOST-cit	1	Ew/p-LOST-cit	
#N/A	0	#N/A	0	#N/A	0	#N/A	
#N/A	0	#N/A	0	#N/A	0	#N/A	
#N/A	0	#N/A	0	#N/A	0	#N/A	
#N/A	0	#N/A	0	#N/A	0	#N/A	
#N/A	0	#N/A	0	#N/A	0	#N/A	
#N/A	0	#N/A	0	#N/A	0	#N/A	
#N/A	0	#N/A	0	#N/A	0	#N/A	
#N/A	0	#N/A	0	#N/A	0	#N/A	
#N/A	0	#N/A	0	#N/A	0	#N/A	
#N/A	0	#N/A	0	#N/A	0	#N/A	
#N/A	0	#N/A	0	#N/A	0	#N/A	
	0	LOST>6_cit	1		0		
#N/A	0	#N/A	0	#N/A	0	#N/A	
#N/A	0	#N/A	0	#N/A	0	#N/A	
#N/A	0	#N/A	0	#N/A	0	#N/A	
#N/A	0	#N/A	0	#N/A	0	#N/A	
#N/A	0	#N/A	0	#N/A	0	#N/A	
#N/A	0	#N/A	0	#N/A	0	#N/A	
#N/A	0	#N/A	0	#N/A	0	#N/A	
	0		0	Ew/a-LOST-cit	1		
	0		0	Ew/a-LOST-cit	1		
#N/A	0	#N/A	0	#N/A	0	#N/A	
#N/A	0	#N/A	0	#N/A	0	#N/A	
#N/A	0	#N/A	0	#N/A	0	#N/A	
LOST-6_cit	1		0	Ew/a-LOST-cit	1	Ew/p-LOST-cit	
LOST-6_cit	1		0	Ew/a-LOST-cit	1	Ew/p-LOST-cit	
#N/A	0	#N/A	0	#N/A	0	#N/A	
#N/A	0	#N/A	0	#N/A	0	#N/A	
#N/A	0	#N/A	0	#N/A	0	#N/A	
#N/A	0	#N/A	0	#N/A	0	#N/A	
	0	LOST>6_cit	1		0		
#N/A	0	#N/A	0	#N/A	0	#N/A	
#N/A	0	#N/A	0	#N/A	0	#N/A	
#N/A	0	#N/A	0	#N/A	0	#N/A	
#N/A	0	#N/A	0	#N/A	0	#N/A	
#N/A	0	#N/A	0	#N/A	0	#N/A	
	0		0		0		
	0		0		0		
	0		0		0		
	0		0		0		
#N/A	0	#N/A	0	#N/A	0	#N/A	
#N/A	0	#N/A	0	#N/A	0	#N/A	
#N/A	0	#N/A	0	#N/A	0	#N/A	
#N/A	0	#N/A	0	#N/A	0	#N/A	
#N/A	0	#N/A	0	#N/A	0	#N/A	

#N/A	0	#N/A	0	#N/A	0	#N/A
#N/A	0	#N/A	0	#N/A	0	#N/A
#N/A	0	#N/A	0	#N/A	0	#N/A
#N/A	0	#N/A	0	#N/A	0	#N/A
#N/A	0	#N/A	0	#N/A	0	#N/A
#N/A	0	#N/A	0	#N/A	0	#N/A
#N/A	0	#N/A	0	#N/A	0	#N/A
#N/A	0	#N/A	0	#N/A	0	#N/A
#N/A	0	#N/A	0	#N/A	0	#N/A
	0		0	Ew/a-LOST-cit	1	
#N/A	0	#N/A	0	#N/A	0	#N/A
#N/A	0	#N/A	0	#N/A	0	#N/A
#N/A	0	#N/A	0	#N/A	0	#N/A
#N/A	0	#N/A	0	#N/A	0	#N/A
#N/A	0	#N/A	0	#N/A	0	#N/A
#N/A	0	#N/A	0	#N/A	0	#N/A
#N/A	0	#N/A	0	#N/A	0	#N/A
LOST-6_cit	1		0		0	
LOST-6_cit	1		0		0	
#N/A	0	#N/A	0	#N/A	0	#N/A
#N/A	0	#N/A	0	#N/A	0	#N/A
#N/A	0	#N/A	0	#N/A	0	#N/A
#N/A	0	#N/A	0	#N/A	0	#N/A
#N/A	0	#N/A	0	#N/A	0	#N/A
#N/A	0	#N/A	0	#N/A	0	#N/A
#N/A	0	#N/A	0	#N/A	0	#N/A
#N/A	0	#N/A	0	#N/A	0	#N/A
#N/A	0	#N/A	0	#N/A	0	#N/A
#N/A	0	#N/A	0	#N/A	0	#N/A
LOST-6_cit	1		0		0	
LOST-6_cit	1		0		0	
#N/A	0	#N/A	0	#N/A	0	#N/A
	0		0		0	
	0		0		0	
#N/A	0	#N/A	0	#N/A	0	#N/A
#N/A	0	#N/A	0	#N/A	0	#N/A
#N/A	0	#N/A	0	#N/A	0	#N/A
#N/A	0	#N/A	0	#N/A	0	#N/A
#N/A	0	#N/A	0	#N/A	0	#N/A
	0		0	Ew/a-LOST-cit	1	
#N/A	0	#N/A	0	#N/A	0	#N/A
#N/A	0	#N/A	0	#N/A	0	#N/A
#N/A	0	#N/A	0	#N/A	0	#N/A
#N/A	0	#N/A	0	#N/A	0	#N/A
#N/A	0	#N/A	0	#N/A	0	#N/A
#N/A	0	#N/A	0	#N/A	0	#N/A
#N/A	0	#N/A	0	#N/A	0	#N/A

#N/A	0	#N/A	0	#N/A	0	#N/A
#N/A	0	#N/A	0	#N/A	0	#N/A
#N/A	0	#N/A	0	#N/A	0	#N/A
#N/A	0	#N/A	0	#N/A	0	#N/A
#N/A	0	#N/A	0	#N/A	0	#N/A
#N/A	0	#N/A	0	#N/A	0	#N/A
	0	LOST>6_cit	1		0	
	0	LOST>6_cit	1		0	
#N/A	0	#N/A	0	#N/A	0	#N/A
#N/A	0	#N/A	0	#N/A	0	#N/A
#N/A	0	#N/A	0	#N/A	0	#N/A
#N/A	0	#N/A	0	#N/A	0	#N/A
#N/A	0	#N/A	0	#N/A	0	#N/A
#N/A	0	#N/A	0	#N/A	0	#N/A
#N/A	0	#N/A	0	#N/A	0	#N/A
#N/A	0	#N/A	0	#N/A	0	#N/A
#N/A	0	#N/A	0	#N/A	0	#N/A
#N/A	0	#N/A	0	#N/A	0	#N/A
#N/A	0	#N/A	0	#N/A	0	#N/A
	0		0		0	
	0		0		0	
	0		0		0	
	0		0		0	
#N/A	0	#N/A	0	#N/A	0	#N/A
#N/A	0	#N/A	0	#N/A	0	#N/A
#N/A	0	#N/A	0	#N/A	0	#N/A
#N/A	0	#N/A	0	#N/A	0	#N/A
#N/A	0	#N/A	0	#N/A	0	#N/A
	0		0	Ew/a-LOST-cit	1	Ew/p-LOST-cit
	0		0	Ew/a-LOST-cit	1	Ew/p-LOST-cit
	0		0	Ew/a-LOST-cit	1	Ew/p-LOST-cit
	0		0	Ew/a-LOST-cit	1	Ew/p-LOST-cit
#N/A	0	#N/A	0	#N/A	0	#N/A
#N/A	0	#N/A	0	#N/A	0	#N/A
#N/A	0	#N/A	0	#N/A	0	#N/A
#N/A	0	#N/A	0	#N/A	0	#N/A
#N/A	0	#N/A	0	#N/A	0	#N/A
LOST-6_cit	1	LOST>6_cit	1		0	
#N/A	0	#N/A	0	#N/A	0	#N/A
#N/A	0	#N/A	0	#N/A	0	#N/A
#N/A	0	#N/A	0	#N/A	0	#N/A
	0		0	Ew/a-LOST-cit	1	Ew/p-LOST-cit
	0		0	Ew/a-LOST-cit	1	Ew/p-LOST-cit
	0		0	Ew/a-LOST-cit	1	Ew/p-LOST-cit
LOST-6_cit	1	LOST>6_cit	1		0	
LOST-6_cit	1	LOST>6_cit	1		0	
#N/A	0	#N/A	0	#N/A	0	#N/A

#N/A	0	#N/A	0	#N/A	0	#N/A
#N/A	0	#N/A	0	#N/A	0	#N/A
#N/A	0	#N/A	0	#N/A	0	#N/A
#N/A	0	#N/A	0	#N/A	0	#N/A
#N/A	0	#N/A	0	#N/A	0	#N/A
#N/A	0	#N/A	0	#N/A	0	#N/A
#N/A	0	#N/A	0	#N/A	0	#N/A
#N/A	0	#N/A	0	#N/A	0	#N/A
#N/A	0	#N/A	0	#N/A	0	#N/A
#N/A	0	#N/A	0	#N/A	0	#N/A
LOST-6_cit	1		0	Ew/a-LOST-cit	1	Ew/p-LOST-cit
LOST-6_cit	1		0	Ew/a-LOST-cit	1	Ew/p-LOST-cit
#N/A	0	#N/A	0	#N/A	0	#N/A
#N/A	0	#N/A	0	#N/A	0	#N/A
#N/A	0	#N/A	0	#N/A	0	#N/A
#N/A	0	#N/A	0	#N/A	0	#N/A
	0		0	Ew/a-LOST-cit	1	Ew/p-LOST-cit
	0		0	Ew/a-LOST-cit	1	Ew/p-LOST-cit
#N/A	0	#N/A	0	#N/A	0	#N/A
#N/A	0	#N/A	0	#N/A	0	#N/A
#N/A	0	#N/A	0	#N/A	0	#N/A
#N/A	0	#N/A	0	#N/A	0	#N/A
LOST-6_cit	1		0	Ew/a-LOST-cit	1	Ew/p-LOST-cit
#N/A	0	#N/A	0	#N/A	0	#N/A
#N/A	0	#N/A	0	#N/A	0	#N/A
#N/A	0	#N/A	0	#N/A	0	#N/A
#N/A	0	#N/A	0	#N/A	0	#N/A
#N/A	0	#N/A	0	#N/A	0	#N/A
#N/A	0	#N/A	0	#N/A	0	#N/A
LOST-6_cit	1		0	Ew/a-LOST-cit	1	
LOST-6_cit	1		0	Ew/a-LOST-cit	1	
#N/A	0	#N/A	0	#N/A	0	#N/A
#N/A	0	#N/A	0	#N/A	0	#N/A
#N/A	0	#N/A	0	#N/A	0	#N/A
#N/A	0	#N/A	0	#N/A	0	#N/A
#N/A	0	#N/A	0	#N/A	0	#N/A
#N/A	0	#N/A	0	#N/A	0	#N/A
#N/A	0	#N/A	0	#N/A	0	#N/A
LOST-6_cit	1		0	Ew/a-LOST-cit	1	Ew/p-LOST-cit
LOST-6_cit	1		0	Ew/a-LOST-cit	1	Ew/p-LOST-cit
#N/A	0	#N/A	0	#N/A	0	#N/A
#N/A	0	#N/A	0	#N/A	0	#N/A
#N/A	0	#N/A	0	#N/A	0	#N/A
#N/A	0	#N/A	0	#N/A	0	#N/A
#N/A	0	#N/A	0	#N/A	0	#N/A
#N/A	0	#N/A	0	#N/A	0	#N/A
#N/A	0	#N/A	0	#N/A	0	#N/A

#N/A	0	#N/A	0	#N/A	0	#N/A
#N/A	0	#N/A	0	#N/A	0	#N/A
#N/A	0	#N/A	0	#N/A	0	#N/A
LOST-6_cit	1	LOST>6_cit	1	Ew/a-LOST-cit	1	Ew/p-LOST-cit
LOST-6_cit	1	LOST>6_cit	1	Ew/a-LOST-cit	1	Ew/p-LOST-cit
LOST-6_cit	1	LOST>6_cit	1	Ew/a-LOST-cit	1	Ew/p-LOST-cit
#N/A	0	#N/A	0	#N/A	0	#N/A
#N/A	0	#N/A	0	#N/A	0	#N/A
#N/A	0	#N/A	0	#N/A	0	#N/A
#N/A	0	#N/A	0	#N/A	0	#N/A
#N/A	0	#N/A	0	#N/A	0	#N/A
	0		0		0	
	0		0		0	
	0		0		0	
#N/A	0	#N/A	0	#N/A	0	#N/A
#N/A	0	#N/A	0	#N/A	0	#N/A
#N/A	0	#N/A	0	#N/A	0	#N/A
#N/A	0	#N/A	0	#N/A	0	#N/A
#N/A	0	#N/A	0	#N/A	0	#N/A
LOST-6_cit	1	LOST>6_cit	1	Ew/a-LOST-cit	1	Ew/p-LOST-cit
#N/A	0	#N/A	0	#N/A	0	#N/A
#N/A	0	#N/A	0	#N/A	0	#N/A
#N/A	0	#N/A	0	#N/A	0	#N/A
#N/A	0	#N/A	0	#N/A	0	#N/A
#N/A	0	#N/A	0	#N/A	0	#N/A
#N/A	0	#N/A	0	#N/A	0	#N/A
#N/A	0	#N/A	0	#N/A	0	#N/A
#N/A	0	#N/A	0	#N/A	0	#N/A
#N/A	0	#N/A	0	#N/A	0	#N/A
#N/A	0	#N/A	0	#N/A	0	#N/A
#N/A	0	#N/A	0	#N/A	0	#N/A
#N/A	0	#N/A	0	#N/A	0	#N/A
#N/A	0	#N/A	0	#N/A	0	#N/A
#N/A	0	#N/A	0	#N/A	0	#N/A
#N/A	0	#N/A	0	#N/A	0	#N/A
#N/A	0	#N/A	0	#N/A	0	#N/A
#N/A	0	#N/A	0	#N/A	0	#N/A
#N/A	0	#N/A	0	#N/A	0	#N/A
#N/A	0	#N/A	0	#N/A	0	#N/A
#N/A	0	#N/A	0	#N/A	0	#N/A
#N/A	0	#N/A	0	#N/A	0	#N/A
#N/A	0	#N/A	0	#N/A	0	#N/A
#N/A	0	#N/A	0	#N/A	0	#N/A
#N/A	0	#N/A	0	#N/A	0	#N/A
#N/A	0	#N/A	0	#N/A	0	#N/A
#N/A	0	#N/A	0	#N/A	0	#N/A
#N/A	0	#N/A	0	#N/A	0	#N/A

	0		0		0	
#N/A	0	#N/A	0	#N/A	0	#N/A
#N/A	0	#N/A	0	#N/A	0	#N/A
LOST-6_cit	1	LOST>6_cit	1		0	
	0		0	Ew/a-LOST-cit	1	Ew/p-LOST-cit
LOST-6_cit	1	LOST>6_cit	1	Ew/a-LOST-cit	1	
LOST-6_cit	1	LOST>6_cit	1	Ew/a-LOST-cit	1	
	0	LOST>6_cit	1		0	
#N/A	0	#N/A	0	#N/A	0	#N/A
#N/A	0	#N/A	0	#N/A	0	#N/A
	0		0		0	
	0		0		0	
#N/A	0	#N/A	0	#N/A	0	#N/A
#N/A	0	#N/A	0	#N/A	0	#N/A
#N/A	0	#N/A	0	#N/A	0	#N/A
#N/A	0	#N/A	0	#N/A	0	#N/A
LOST-6_cit	1	LOST>6_cit	1		0	
#N/A	0	#N/A	0	#N/A	0	#N/A
#N/A	0	#N/A	0	#N/A	0	#N/A
#N/A	0	#N/A	0	#N/A	0	#N/A
#N/A	0	#N/A	0	#N/A	0	#N/A
#N/A	0	#N/A	0	#N/A	0	#N/A
#N/A	0	#N/A	0	#N/A	0	#N/A
#N/A	0	#N/A	0	#N/A	0	#N/A
#N/A	0	#N/A	0	#N/A	0	#N/A
#N/A	0	#N/A	0	#N/A	0	#N/A
#N/A	0	#N/A	0	#N/A	0	#N/A
LOST-6_cit	1		0		0	
#N/A	0	#N/A	0	#N/A	0	#N/A
#N/A	0	#N/A	0	#N/A	0	#N/A
#N/A	0	#N/A	0	#N/A	0	#N/A
#N/A	0	#N/A	0	#N/A	0	#N/A
#N/A	0	#N/A	0	#N/A	0	#N/A
#N/A	0	#N/A	0	#N/A	0	#N/A
LOST-6_cit	1		0		0	Ew/p-LOST-cit
#N/A	0	#N/A	0	#N/A	0	#N/A
#N/A	0	#N/A	0	#N/A	0	#N/A
#N/A	0	#N/A	0	#N/A	0	#N/A
#N/A	0	#N/A	0	#N/A	0	#N/A
#N/A	0	#N/A	0	#N/A	0	#N/A
#N/A	0	#N/A	0	#N/A	0	#N/A
#N/A	0	#N/A	0	#N/A	0	#N/A
#N/A	0	#N/A	0	#N/A	0	#N/A
#N/A	0	#N/A	0	#N/A	0	#N/A
#N/A	0	#N/A	0	#N/A	0	#N/A
#N/A	0	#N/A	0	#N/A	0	#N/A
	0		0		0	

	0		0		0	
#N/A	0	#N/A	0	#N/A	0	#N/A
#N/A	0	#N/A	0	#N/A	0	#N/A
#N/A	0	#N/A	0	#N/A	0	#N/A
#N/A	0	#N/A	0	#N/A	0	#N/A
#N/A	0	#N/A	0	#N/A	0	#N/A
#N/A	0	#N/A	0	#N/A	0	#N/A
	0	LOST>6_cit	1		0	
#N/A	0	#N/A	0	#N/A	0	#N/A
#N/A	0	#N/A	0	#N/A	0	#N/A
#N/A	0	#N/A	0	#N/A	0	#N/A
#N/A	0	#N/A	0	#N/A	0	#N/A
LOST-6_cit	1	LOST>6_cit	1		0	
LOST-6_cit	1	LOST>6_cit	1		0	
LOST-6_cit	1	LOST>6_cit	1		0	
#N/A	0	#N/A	0	#N/A	0	#N/A
	0		0	Ew/a-LOST-cit	1	Ew/p-LOST-cit
	0		0	Ew/a-LOST-cit	1	Ew/p-LOST-cit
	0		0	Ew/a-LOST-cit	1	Ew/p-LOST-cit
#N/A	0	#N/A	0	#N/A	0	#N/A
#N/A	0	#N/A	0	#N/A	0	#N/A
	0		0		0	
#N/A	0	#N/A	0	#N/A	0	#N/A
	0		0		0	
	0		0		0	
#N/A	0	#N/A	0	#N/A	0	#N/A
#N/A	0	#N/A	0	#N/A	0	#N/A
#N/A	0	#N/A	0	#N/A	0	#N/A
#N/A	0	#N/A	0	#N/A	0	#N/A
#N/A	0	#N/A	0	#N/A	0	#N/A
#N/A	0	#N/A	0	#N/A	0	#N/A
	0	LOST>6_cit	1		0	
#N/A	0	#N/A	0	#N/A	0	#N/A
LOST-6_cit	1	LOST>6_cit	1		0	
LOST-6_cit	1	LOST>6_cit	1		0	
LOST-6_cit	1	LOST>6_cit	1		0	
LOST-6_cit	1	LOST>6_cit	1		0	
#N/A	0	#N/A	0	#N/A	0	#N/A
LOST-6_cit	1	LOST>6_cit	1		0	
LOST-6_cit	1	LOST>6_cit	1		0	
LOST-6_cit	1		0	Ew/a-LOST-cit	1	Ew/p-LOST-cit
LOST-6_cit	1		0	Ew/a-LOST-cit	1	Ew/p-LOST-cit
#N/A	0	#N/A	0	#N/A	0	#N/A
	0		0		0	
	0		0		0	
	0	LOST>6_cit	1		0	
	0	LOST>6_cit	1		0	

#N/A	0	#N/A	0	#N/A	0	#N/A
#N/A	0	#N/A	0	#N/A	0	#N/A
#N/A	0	#N/A	0	#N/A	0	#N/A
#N/A	0	#N/A	0	#N/A	0	#N/A
#N/A	0	#N/A	0	#N/A	0	#N/A
#N/A	0	#N/A	0	#N/A	0	#N/A
#N/A	0	#N/A	0	#N/A	0	#N/A
#N/A	0	#N/A	0	#N/A	0	#N/A
#N/A	0	#N/A	0	#N/A	0	#N/A
#N/A	0	#N/A	0	#N/A	0	#N/A
#N/A	0	#N/A	0	#N/A	0	#N/A
#N/A	0	#N/A	0	#N/A	0	#N/A
#N/A	0	#N/A	0	#N/A	0	#N/A
#N/A	0	#N/A	0	#N/A	0	#N/A
#N/A	0	#N/A	0	#N/A	0	#N/A
#N/A	0	#N/A	0	#N/A	0	#N/A
#N/A	0	#N/A	0	#N/A	0	#N/A
	0		0		0	
	0		0		0	
	0		0		0	
	0		0		0	
	0		0		0	
#N/A	0	#N/A	0	#N/A	0	#N/A
#N/A	0	#N/A	0	#N/A	0	#N/A
#N/A	0	#N/A	0	#N/A	0	#N/A
#N/A	0	#N/A	0	#N/A	0	#N/A
	0	LOST>6_cit	1		0	
LOST-6_cit	1	LOST>6_cit	1		0	
#N/A	0	#N/A	0	#N/A	0	#N/A
#N/A	0	#N/A	0	#N/A	0	#N/A
#N/A	0	#N/A	0	#N/A	0	#N/A
#N/A	0	#N/A	0	#N/A	0	#N/A
#N/A	0	#N/A	0	#N/A	0	#N/A
	0	LOST>6_cit	1		0	
	0	LOST>6_cit	1		0	
#N/A	0	#N/A	0	#N/A	0	#N/A
#N/A	0	#N/A	0	#N/A	0	#N/A
#N/A	0	#N/A	0	#N/A	0	#N/A
#N/A	0	#N/A	0	#N/A	0	#N/A
#N/A	0	#N/A	0	#N/A	0	#N/A
#N/A	0	#N/A	0	#N/A	0	#N/A
#N/A	0	#N/A	0	#N/A	0	#N/A
#N/A	0	#N/A	0	#N/A	0	#N/A
#N/A	0	#N/A	0	#N/A	0	#N/A
#N/A	0	#N/A	0	#N/A	0	#N/A
LOST-6_cit	1	LOST>6_cit	1	Ew/a-LOST-cit	1	Ew/p-LOST-cit
LOST-6_cit	1	LOST>6_cit	1	Ew/a-LOST-cit	1	Ew/p-LOST-cit

LOST-6_cit	1	LOST>6_cit	1	Ew/a-LOST-cit	1	Ew/p-LOST-cit
#N/A	0	#N/A	0	#N/A	0	#N/A
#N/A	0	#N/A	0	#N/A	0	#N/A
#N/A	0	#N/A	0	#N/A	0	#N/A
	0		0	Ew/a-LOST-cit	1	Ew/p-LOST-cit
#N/A	0	#N/A	0	#N/A	0	#N/A
#N/A	0	#N/A	0	#N/A	0	#N/A
#N/A	0	#N/A	0	#N/A	0	#N/A
#N/A	0	#N/A	0	#N/A	0	#N/A
#N/A	0	#N/A	0	#N/A	0	#N/A
#N/A	0	#N/A	0	#N/A	0	#N/A
#N/A	0	#N/A	0	#N/A	0	#N/A
#N/A	0	#N/A	0	#N/A	0	#N/A
#N/A	0	#N/A	0	#N/A	0	#N/A
#N/A	0	#N/A	0	#N/A	0	#N/A
	0		0	Ew/a-LOST-cit	1	
	0		0	Ew/a-LOST-cit	1	
#N/A	0	#N/A	0	#N/A	0	#N/A
#N/A	0	#N/A	0	#N/A	0	#N/A
#N/A	0	#N/A	0	#N/A	0	#N/A
#N/A	0	#N/A	0	#N/A	0	#N/A
LOST-6_cit	1		0	Ew/a-LOST-cit	1	Ew/p-LOST-cit
LOST-6_cit	1	LOST>6_cit	1		0	Ew/p-LOST-cit
#N/A	0	#N/A	0	#N/A	0	#N/A
#N/A	0	#N/A	0	#N/A	0	#N/A
	0		0	Ew/a-LOST-cit	1	
	0		0	Ew/a-LOST-cit	1	
	0		0	Ew/a-LOST-cit	1	
	0		0	Ew/a-LOST-cit	1	
#N/A	0	#N/A	0	#N/A	0	#N/A
	0		0		0	
	0		0		0	
	0		0	Ew/a-LOST-cit	1	Ew/p-LOST-cit
#N/A	0	#N/A	0	#N/A	0	#N/A
#N/A	0	#N/A	0	#N/A	0	#N/A
#N/A	0	#N/A	0	#N/A	0	#N/A
#N/A	0	#N/A	0	#N/A	0	#N/A
#N/A	0	#N/A	0	#N/A	0	#N/A
#N/A	0	#N/A	0	#N/A	0	#N/A
LOST-6_cit	1		0		0	
	0	LOST>6_cit	1		0	
#N/A	0	#N/A	0	#N/A	0	#N/A
#N/A	0	#N/A	0	#N/A	0	#N/A
#N/A	0	#N/A	0	#N/A	0	#N/A
#N/A	0	#N/A	0	#N/A	0	#N/A
#N/A	0	#N/A	0	#N/A	0	#N/A
LOST-6_cit	1	LOST>6_cit	1		0	

	0		0	Ew/a-LOST-cit	1	Ew/p-LOST-cit
	0		0	Ew/a-LOST-cit	1	Ew/p-LOST-cit
#N/A	0	#N/A	0	#N/A	0	#N/A
#N/A	0	#N/A	0	#N/A	0	#N/A
#N/A	0	#N/A	0	#N/A	0	#N/A
#N/A	0	#N/A	0	#N/A	0	#N/A
#N/A	0	#N/A	0	#N/A	0	#N/A
#N/A	0	#N/A	0	#N/A	0	#N/A
#N/A	0	#N/A	0	#N/A	0	#N/A
#N/A	0	#N/A	0	#N/A	0	#N/A
#N/A	0	#N/A	0	#N/A	0	#N/A
#N/A	0	#N/A	0	#N/A	0	#N/A
#N/A	0	#N/A	0	#N/A	0	#N/A
#N/A	0	#N/A	0	#N/A	0	#N/A
	0		0	Ew/a-LOST-cit	1	Ew/p-LOST-cit
#N/A	0	#N/A	0	#N/A	0	#N/A
#N/A	0	#N/A	0	#N/A	0	#N/A
#N/A	0	#N/A	0	#N/A	0	#N/A
#N/A	0	#N/A	0	#N/A	0	#N/A
#N/A	0	#N/A	0	#N/A	0	#N/A
#N/A	0	#N/A	0	#N/A	0	#N/A
#N/A	0	#N/A	0	#N/A	0	#N/A
#N/A	0	#N/A	0	#N/A	0	#N/A
#N/A	0	#N/A	0	#N/A	0	#N/A
	0		0	Ew/a-LOST-cit	1	Ew/p-LOST-cit
	0		0	Ew/a-LOST-cit	1	Ew/p-LOST-cit
#N/A	0	#N/A	0	#N/A	0	#N/A
#N/A	0	#N/A	0	#N/A	0	#N/A
#N/A	0	#N/A	0	#N/A	0	#N/A
#N/A	0	#N/A	0	#N/A	0	#N/A
#N/A	0	#N/A	0	#N/A	0	#N/A
#N/A	0	#N/A	0	#N/A	0	#N/A
#N/A	0	#N/A	0	#N/A	0	#N/A
#N/A	0	#N/A	0	#N/A	0	#N/A
#N/A	0	#N/A	0	#N/A	0	#N/A
#N/A	0	#N/A	0	#N/A	0	#N/A
#N/A	0	#N/A	0	#N/A	0	#N/A
#N/A	0	#N/A	0	#N/A	0	#N/A
#N/A	0	#N/A	0	#N/A	0	#N/A
	0	LOST>6_cit	1		0	
#N/A	0	#N/A	0	#N/A	0	#N/A
#N/A	0	#N/A	0	#N/A	0	#N/A
LOST-6_cit	1	LOST>6_cit	1		0	
#N/A	0	#N/A	0	#N/A	0	#N/A
#N/A	0	#N/A	0	#N/A	0	#N/A
#N/A	0	#N/A	0	#N/A	0	#N/A
#N/A	0	#N/A	0	#N/A	0	#N/A

#N/A	0	#N/A	0	#N/A	0	#N/A
#N/A	0	#N/A	0	#N/A	0	#N/A
#N/A	0	#N/A	0	#N/A	0	#N/A
#N/A	0	#N/A	0	#N/A	0	#N/A
#N/A	0	#N/A	0	#N/A	0	#N/A
#N/A	0	#N/A	0	#N/A	0	#N/A
#N/A	0	#N/A	0	#N/A	0	#N/A
#N/A	0	#N/A	0	#N/A	0	#N/A
#N/A	0	#N/A	0	#N/A	0	#N/A
#N/A	0	#N/A	0	#N/A	0	#N/A
	0		0	Ew/a-LOST-cit	1	Ew/p-LOST-cit
	0		0	Ew/a-LOST-cit	1	Ew/p-LOST-cit
#N/A	0	#N/A	0	#N/A	0	#N/A
#N/A	0	#N/A	0	#N/A	0	#N/A
#N/A	0	#N/A	0	#N/A	0	#N/A
#N/A	0	#N/A	0	#N/A	0	#N/A
#N/A	0	#N/A	0	#N/A	0	#N/A
#N/A	0	#N/A	0	#N/A	0	#N/A
#N/A	0	#N/A	0	#N/A	0	#N/A
LOST-6_cit	1		0		0	
LOST-6_cit	1		0		0	
#N/A	0	#N/A	0	#N/A	0	#N/A
LOST-6_cit	1		0		0	
#N/A	0	#N/A	0	#N/A	0	#N/A
#N/A	0	#N/A	0	#N/A	0	#N/A
#N/A	0	#N/A	0	#N/A	0	#N/A
#N/A	0	#N/A	0	#N/A	0	#N/A
#N/A	0	#N/A	0	#N/A	0	#N/A
#N/A	0	#N/A	0	#N/A	0	#N/A
#N/A	0	#N/A	0	#N/A	0	#N/A
#N/A	0	#N/A	0	#N/A	0	#N/A
#N/A	0	#N/A	0	#N/A	0	#N/A
	0		0	Ew/a-LOST-cit	1	Ew/p-LOST-cit
#N/A	0	#N/A	0	#N/A	0	#N/A
	0		0	Ew/a-LOST-cit	1	Ew/p-LOST-cit
#N/A	0	#N/A	0	#N/A	0	#N/A
#N/A	0	#N/A	0	#N/A	0	#N/A
#N/A	0	#N/A	0	#N/A	0	#N/A
#N/A	0	#N/A	0	#N/A	0	#N/A
#N/A	0	#N/A	0	#N/A	0	#N/A
	0		0		0	
	0		0		0	
	0		0		0	
	0		0		0	
	0		0		0	
LOST-6_cit	1		0	Ew/a-LOST-cit	1	Ew/p-LOST-cit
LOST-6_cit	1		0	Ew/a-LOST-cit	1	Ew/p-LOST-cit

LOST-6_cit	1		0	Ew/a-LOST-cit	1	Ew/p-LOST-cit
LOST-6_cit	1		0	Ew/a-LOST-cit	1	Ew/p-LOST-cit
LOST-6_cit	1		0	Ew/a-LOST-cit	1	Ew/p-LOST-cit
#N/A	0	#N/A	0	#N/A	0	#N/A
#N/A	0	#N/A	0	#N/A	0	#N/A
#N/A	0	#N/A	0	#N/A	0	#N/A
#N/A	0	#N/A	0	#N/A	0	#N/A
#N/A	0	#N/A	0	#N/A	0	#N/A
#N/A	0	#N/A	0	#N/A	0	#N/A
#N/A	0	#N/A	0	#N/A	0	#N/A
#N/A	0	#N/A	0	#N/A	0	#N/A
#N/A	0	#N/A	0	#N/A	0	#N/A
#N/A	0	#N/A	0	#N/A	0	#N/A
#N/A	0	#N/A	0	#N/A	0	#N/A
#N/A	0	#N/A	0	#N/A	0	#N/A
#N/A	0	#N/A	0	#N/A	0	#N/A
#N/A	0	#N/A	0	#N/A	0	#N/A
	0		0	Ew/a-LOST-cit	1	Ew/p-LOST-cit
#N/A	0	#N/A	0	#N/A	0	#N/A
#N/A	0	#N/A	0	#N/A	0	#N/A
	0		0		0	
	0		0		0	
#N/A	0	#N/A	0	#N/A	0	#N/A
#N/A	0	#N/A	0	#N/A	0	#N/A
#N/A	0	#N/A	0	#N/A	0	#N/A
#N/A	0	#N/A	0	#N/A	0	#N/A
	0		0	Ew/a-LOST-cit	1	
#N/A	0	#N/A	0	#N/A	0	#N/A
#N/A	0	#N/A	0	#N/A	0	#N/A
#N/A	0	#N/A	0	#N/A	0	#N/A
#N/A	0	#N/A	0	#N/A	0	#N/A
#N/A	0	#N/A	0	#N/A	0	#N/A
#N/A	0	#N/A	0	#N/A	0	#N/A
	0		0	Ew/a-LOST-cit	1	Ew/p-LOST-cit
	0		0	Ew/a-LOST-cit	1	Ew/p-LOST-cit
#N/A	0	#N/A	0	#N/A	0	#N/A
#N/A	0	#N/A	0	#N/A	0	#N/A
	0		0		0	
	0		0		0	
#N/A	0	#N/A	0	#N/A	0	#N/A
#N/A	0	#N/A	0	#N/A	0	#N/A
#N/A	0	#N/A	0	#N/A	0	#N/A
#N/A	0	#N/A	0	#N/A	0	#N/A
#N/A	0	#N/A	0	#N/A	0	#N/A
#N/A	0	#N/A	0	#N/A	0	#N/A
#N/A	0	#N/A	0	#N/A	0	#N/A
#N/A	0	#N/A	0	#N/A	0	#N/A

#N/A	0	#N/A	0	#N/A	0	#N/A	
	0		0		0		
	0		0	Ew/a-LOST-cit	1	Ew/p-LOST-cit	
	0		0	Ew/a-LOST-cit	1	Ew/p-LOST-cit	
	0		0	Ew/a-LOST-cit	1	Ew/p-LOST-cit	
	0		0	Ew/a-LOST-cit	1	Ew/p-LOST-cit	
LOST-6_cit	1	LOST>6_cit	1	Ew/a-LOST-cit	1	Ew/p-LOST-cit	
#N/A	0	#N/A	0	#N/A	0	#N/A	
#N/A	0	#N/A	0	#N/A	0	#N/A	
#N/A	0	#N/A	0	#N/A	0	#N/A	
#N/A	0	#N/A	0	#N/A	0	#N/A	
#N/A	0	#N/A	0	#N/A	0	#N/A	
#N/A	0	#N/A	0	#N/A	0	#N/A	
	0		0	Ew/a-LOST-cit	1	Ew/p-LOST-cit	
	0		0	Ew/a-LOST-cit	1	Ew/p-LOST-cit	
	0		0	Ew/a-LOST-cit	1	Ew/p-LOST-cit	
#N/A	0	#N/A	0	#N/A	0	#N/A	
#N/A	0	#N/A	0	#N/A	0	#N/A	
#N/A	0	#N/A	0	#N/A	0	#N/A	
#N/A	0	#N/A	0	#N/A	0	#N/A	
#N/A	0	#N/A	0	#N/A	0	#N/A	
#N/A	0	#N/A	0	#N/A	0	#N/A	
#N/A	0	#N/A	0	#N/A	0	#N/A	
#N/A	0	#N/A	0	#N/A	0	#N/A	
#N/A	0	#N/A	0	#N/A	0	#N/A	
#N/A	0	#N/A	0	#N/A	0	#N/A	
#N/A	0	#N/A	0	#N/A	0	#N/A	
#N/A	0	#N/A	0	#N/A	0	#N/A	
LOST-6_cit	1		0	Ew/a-LOST-cit	1	Ew/p-LOST-cit	
LOST-6_cit	1		0	Ew/a-LOST-cit	1	Ew/p-LOST-cit	
#N/A	0	#N/A	0	#N/A	0	#N/A	
	0		0	Ew/a-LOST-cit	1	Ew/p-LOST-cit	
#N/A	0	#N/A	0	#N/A	0	#N/A	
#N/A	0	#N/A	0	#N/A	0	#N/A	
#N/A	0	#N/A	0	#N/A	0	#N/A	
#N/A	0	#N/A	0	#N/A	0	#N/A	
#N/A	0	#N/A	0	#N/A	0	#N/A	
#N/A	0	#N/A	0	#N/A	0	#N/A	
#N/A	0	#N/A	0	#N/A	0	#N/A	
#N/A	0	#N/A	0	#N/A	0	#N/A	
#N/A	0	#N/A	0	#N/A	0	#N/A	
#N/A	0	#N/A	0	#N/A	0	#N/A	
#N/A	0	#N/A	0	#N/A	0	#N/A	
#N/A	0	#N/A	0	#N/A	0	#N/A	
#N/A	0	#N/A	0	#N/A	0	#N/A	
#N/A	0	#N/A	0	#N/A	0	#N/A	
#N/A	0	#N/A	0	#N/A	0	#N/A	

#N/A	0	#N/A	0	#N/A	0	#N/A
#N/A	0	#N/A	0	#N/A	0	#N/A
	0	LOST>6_cit	1	Ew/a-LOST-cit	1	Ew/p-LOST-cit
	0	LOST>6_cit	1	Ew/a-LOST-cit	1	Ew/p-LOST-cit
	0	LOST>6_cit	1	Ew/a-LOST-cit	1	Ew/p-LOST-cit
	0		0	Ew/a-LOST-cit	1	Ew/p-LOST-cit
	0		0	Ew/a-LOST-cit	1	Ew/p-LOST-cit
	0		0	Ew/a-LOST-cit	1	Ew/p-LOST-cit
#N/A	0	#N/A	0	#N/A	0	#N/A
#N/A	0	#N/A	0	#N/A	0	#N/A
#N/A	0	#N/A	0	#N/A	0	#N/A
#N/A	0	#N/A	0	#N/A	0	#N/A
#N/A	0	#N/A	0	#N/A	0	#N/A
#N/A	0	#N/A	0	#N/A	0	#N/A
#N/A	0	#N/A	0	#N/A	0	#N/A
#N/A	0	#N/A	0	#N/A	0	#N/A
#N/A	0	#N/A	0	#N/A	0	#N/A
#N/A	0	#N/A	0	#N/A	0	#N/A
#N/A	0	#N/A	0	#N/A	0	#N/A
#N/A	0	#N/A	0	#N/A	0	#N/A
#N/A	0	#N/A	0	#N/A	0	#N/A
#N/A	0	#N/A	0	#N/A	0	#N/A
#N/A	0	#N/A	0	#N/A	0	#N/A
#N/A	0	#N/A	0	#N/A	0	#N/A
#N/A	0	#N/A	0	#N/A	0	#N/A
	0		0	Ew/a-LOST-cit	1	Ew/p-LOST-cit
	0		0	Ew/a-LOST-cit	1	Ew/p-LOST-cit
	0		0	Ew/a-LOST-cit	1	Ew/p-LOST-cit
	0		0	Ew/a-LOST-cit	1	Ew/p-LOST-cit
	0		0	Ew/a-LOST-cit	1	Ew/p-LOST-cit
#N/A	0	#N/A	0	#N/A	0	#N/A
	0		0	Ew/a-LOST-cit	1	Ew/p-LOST-cit
	0		0	Ew/a-LOST-cit	1	Ew/p-LOST-cit
#N/A	0	#N/A	0	#N/A	0	#N/A
#N/A	0	#N/A	0	#N/A	0	#N/A
#N/A	0	#N/A	0	#N/A	0	#N/A
#N/A	0	#N/A	0	#N/A	0	#N/A
#N/A	0	#N/A	0	#N/A	0	#N/A
#N/A	0	#N/A	0	#N/A	0	#N/A
#N/A	0	#N/A	0	#N/A	0	#N/A
	0	LOST>6_cit	1	Ew/a-LOST-cit	1	
#N/A	0	#N/A	0	#N/A	0	#N/A
#N/A	0	#N/A	0	#N/A	0	#N/A
LOST-6_cit	1		0		0	
LOST-6_cit	1		0		0	
#N/A	0	#N/A	0	#N/A	0	#N/A
#N/A	0	#N/A	0	#N/A	0	#N/A

#N/A	0	#N/A	0	#N/A	0	#N/A
#N/A	0	#N/A	0	#N/A	0	#N/A
#N/A	0	#N/A	0	#N/A	0	#N/A
#N/A	0	#N/A	0	#N/A	0	#N/A
#N/A	0	#N/A	0	#N/A	0	#N/A
#N/A	0	#N/A	0	#N/A	0	#N/A
#N/A	0	#N/A	0	#N/A	0	#N/A
LOST-6_cit	1	LOST>6_cit	1		0	
LOST-6_cit	1	LOST>6_cit	1		0	
#N/A	0	#N/A	0	#N/A	0	#N/A
#N/A	0	#N/A	0	#N/A	0	#N/A
#N/A	0	#N/A	0	#N/A	0	#N/A
#N/A	0	#N/A	0	#N/A	0	#N/A
#N/A	0	#N/A	0	#N/A	0	#N/A
#N/A	0	#N/A	0	#N/A	0	#N/A
#N/A	0	#N/A	0	#N/A	0	#N/A
	0		0		0	
#N/A	0	#N/A	0	#N/A	0	#N/A
#N/A	0	#N/A	0	#N/A	0	#N/A
#N/A	0	#N/A	0	#N/A	0	#N/A
#N/A	0	#N/A	0	#N/A	0	#N/A
#N/A	0	#N/A	0	#N/A	0	#N/A
LOST-6_cit	1		0		0	
LOST-6_cit	1		0		0	
#N/A	0	#N/A	0	#N/A	0	#N/A
#N/A	0	#N/A	0	#N/A	0	#N/A
#N/A	0	#N/A	0	#N/A	0	#N/A
#N/A	0	#N/A	0	#N/A	0	#N/A
LOST-6_cit	1	LOST>6_cit	1	Ew/a-LOST-cit	1	
LOST-6_cit	1	LOST>6_cit	1	Ew/a-LOST-cit	1	
	0		0	Ew/a-LOST-cit	1	Ew/p-LOST-cit
	0		0	Ew/a-LOST-cit	1	Ew/p-LOST-cit
#N/A	0	#N/A	0	#N/A	0	#N/A
#N/A	0	#N/A	0	#N/A	0	#N/A
#N/A	0	#N/A	0	#N/A	0	#N/A
	0		0	Ew/a-LOST-cit	1	Ew/p-LOST-cit
	0		0	Ew/a-LOST-cit	1	Ew/p-LOST-cit
#N/A	0	#N/A	0	#N/A	0	#N/A
#N/A	0	#N/A	0	#N/A	0	#N/A
#N/A	0	#N/A	0	#N/A	0	#N/A
#N/A	0	#N/A	0	#N/A	0	#N/A
#N/A	0	#N/A	0	#N/A	0	#N/A
#N/A	0	#N/A	0	#N/A	0	#N/A
#N/A	0	#N/A	0	#N/A	0	#N/A
	0		0	Ew/a-LOST-cit	1	
	0		0	Ew/a-LOST-cit	1	
#N/A	0	#N/A	0	#N/A	0	#N/A

#N/A	0	#N/A	0	#N/A	0	#N/A
#N/A	0	#N/A	0	#N/A	0	#N/A
#N/A	0	#N/A	0	#N/A	0	#N/A
#N/A	0	#N/A	0	#N/A	0	#N/A
#N/A	0	#N/A	0	#N/A	0	#N/A
#N/A	0	#N/A	0	#N/A	0	#N/A
#N/A	0	#N/A	0	#N/A	0	#N/A
#N/A	0	#N/A	0	#N/A	0	#N/A
#N/A	0	#N/A	0	#N/A	0	#N/A
#N/A	0	#N/A	0	#N/A	0	#N/A
LOST-6_cit	1		0	Ew/a-LOST-cit	1	
#N/A	0	#N/A	0	#N/A	0	#N/A
#N/A	0	#N/A	0	#N/A	0	#N/A
#N/A	0	#N/A	0	#N/A	0	#N/A
#N/A	0	#N/A	0	#N/A	0	#N/A
#N/A	0	#N/A	0	#N/A	0	#N/A
#N/A	0	#N/A	0	#N/A	0	#N/A
#N/A	0	#N/A	0	#N/A	0	#N/A
#N/A	0	#N/A	0	#N/A	0	#N/A
#N/A	0	#N/A	0	#N/A	0	#N/A
#N/A	0	#N/A	0	#N/A	0	#N/A
#N/A	0	#N/A	0	#N/A	0	#N/A
#N/A	0	#N/A	0	#N/A	0	#N/A
#N/A	0	#N/A	0	#N/A	0	#N/A
#N/A	0	#N/A	0	#N/A	0	#N/A
#N/A	0	#N/A	0	#N/A	0	#N/A
#N/A	0	#N/A	0	#N/A	0	#N/A
#N/A	0	#N/A	0	#N/A	0	#N/A
#N/A	0	#N/A	0	#N/A	0	#N/A
#N/A	0	#N/A	0	#N/A	0	#N/A
#N/A	0	#N/A	0	#N/A	0	#N/A
#N/A	0	#N/A	0	#N/A	0	#N/A
#N/A	0	#N/A	0	#N/A	0	#N/A
#N/A	0	#N/A	0	#N/A	0	#N/A
	0		0		0	
	0		0		0	
#N/A	0	#N/A	0	#N/A	0	#N/A
#N/A	0	#N/A	0	#N/A	0	#N/A
#N/A	0	#N/A	0	#N/A	0	#N/A
#N/A	0	#N/A	0	#N/A	0	#N/A
	0		0	Ew/a-LOST-cit	1	Ew/p-LOST-cit
	0		0	Ew/a-LOST-cit	1	Ew/p-LOST-cit
	0		0	Ew/a-LOST-cit	1	Ew/p-LOST-cit
#N/A	0	#N/A	0	#N/A	0	#N/A
#N/A	0	#N/A	0	#N/A	0	#N/A
#N/A	0	#N/A	0	#N/A	0	#N/A
#N/A	0	#N/A	0	#N/A	0	#N/A

LOST-6_cit	1		0		0	
LOST-6_cit	1		0		0	
LOST-6_cit	1		0		0	
#N/A	0	#N/A	0	#N/A	0	#N/A
#N/A	0	#N/A	0	#N/A	0	#N/A
#N/A	0	#N/A	0	#N/A	0	#N/A
#N/A	0	#N/A	0	#N/A	0	#N/A
#N/A	0	#N/A	0	#N/A	0	#N/A
#N/A	0	#N/A	0	#N/A	0	#N/A
#N/A	0	#N/A	0	#N/A	0	#N/A
LOST-6_cit	1		0		0	
#N/A	0	#N/A	0	#N/A	0	#N/A
#N/A	0	#N/A	0	#N/A	0	#N/A
#N/A	0	#N/A	0	#N/A	0	#N/A
#N/A	0	#N/A	0	#N/A	0	#N/A
#N/A	0	#N/A	0	#N/A	0	#N/A
	0		0	Ew/a-LOST-cit	1	
#N/A	0	#N/A	0	#N/A	0	#N/A
#N/A	0	#N/A	0	#N/A	0	#N/A
#N/A	0	#N/A	0	#N/A	0	#N/A
#N/A	0	#N/A	0	#N/A	0	#N/A
#N/A	0	#N/A	0	#N/A	0	#N/A
#N/A	0	#N/A	0	#N/A	0	#N/A
#N/A	0	#N/A	0	#N/A	0	#N/A
#N/A	0	#N/A	0	#N/A	0	#N/A
#N/A	0	#N/A	0	#N/A	0	#N/A
	0		0		0	
#N/A	0	#N/A	0	#N/A	0	#N/A
#N/A	0	#N/A	0	#N/A	0	#N/A
	0		0	Ew/a-LOST-cit	1	
	0		0	Ew/a-LOST-cit	1	
#N/A	0	#N/A	0	#N/A	0	#N/A
#N/A	0	#N/A	0	#N/A	0	#N/A
#N/A	0	#N/A	0	#N/A	0	#N/A
#N/A	0	#N/A	0	#N/A	0	#N/A
#N/A	0	#N/A	0	#N/A	0	#N/A
#N/A	0	#N/A	0	#N/A	0	#N/A
#N/A	0	#N/A	0	#N/A	0	#N/A
#N/A	0	#N/A	0	#N/A	0	#N/A
#N/A	0	#N/A	0	#N/A	0	#N/A
#N/A	0	#N/A	0	#N/A	0	#N/A
#N/A	0	#N/A	0	#N/A	0	#N/A
LOST-6_cit	1		0		0	
#N/A	0	#N/A	0	#N/A	0	#N/A
#N/A	0	#N/A	0	#N/A	0	#N/A
#N/A	0	#N/A	0	#N/A	0	#N/A
#N/A	0	#N/A	0	#N/A	0	#N/A

#N/A	0	#N/A	0	#N/A	0	#N/A
#N/A	0	#N/A	0	#N/A	0	#N/A
#N/A	0	#N/A	0	#N/A	0	#N/A
#N/A	0	#N/A	0	#N/A	0	#N/A
#N/A	0	#N/A	0	#N/A	0	#N/A
#N/A	0	#N/A	0	#N/A	0	#N/A
#N/A	0	#N/A	0	#N/A	0	#N/A
#N/A	0	#N/A	0	#N/A	0	#N/A
#N/A	0	#N/A	0	#N/A	0	#N/A
#N/A	0	#N/A	0	#N/A	0	#N/A
#N/A	0	#N/A	0	#N/A	0	#N/A
#N/A	0	#N/A	0	#N/A	0	#N/A
#N/A	0	#N/A	0	#N/A	0	#N/A
#N/A	0	#N/A	0	#N/A	0	#N/A
#N/A	0	#N/A	0	#N/A	0	#N/A
#N/A	0	#N/A	0	#N/A	0	#N/A
#N/A	0	#N/A	0	#N/A	0	#N/A
#N/A	0	#N/A	0	#N/A	0	#N/A
#N/A	0	#N/A	0	#N/A	0	#N/A
#N/A	0	#N/A	0	#N/A	0	#N/A
#N/A	0	#N/A	0	#N/A	0	#N/A
#N/A	0	#N/A	0	#N/A	0	#N/A
#N/A	0	#N/A	0	#N/A	0	#N/A
#N/A	0	#N/A	0	#N/A	0	#N/A
	0		0		0	
#N/A	0	#N/A	0	#N/A	0	#N/A
#N/A	0	#N/A	0	#N/A	0	#N/A
#N/A	0	#N/A	0	#N/A	0	#N/A
LOST-6_cit	1		0		0	
#N/A	0	#N/A	0	#N/A	0	#N/A
LOST-6_cit	1		0		0	
#N/A	0	#N/A	0	#N/A	0	#N/A
LOST-6_cit	1		0		0	
#N/A	0	#N/A	0	#N/A	0	#N/A
#N/A	0	#N/A	0	#N/A	0	#N/A
#N/A	0	#N/A	0	#N/A	0	#N/A
#N/A	0	#N/A	0	#N/A	0	#N/A
#N/A	0	#N/A	0	#N/A	0	#N/A
#N/A	0	#N/A	0	#N/A	0	#N/A
#N/A	0	#N/A	0	#N/A	0	#N/A
#N/A	0	#N/A	0	#N/A	0	#N/A
LOST-6_cit	1		0		0	
LOST-6_cit	1		0		0	
#N/A	0	#N/A	0	#N/A	0	#N/A
#N/A	0	#N/A	0	#N/A	0	#N/A
#N/A	0	#N/A	0	#N/A	0	#N/A
#N/A	0	#N/A	0	#N/A	0	#N/A

#N/A	0	#N/A	0	#N/A	0	#N/A
#N/A	0	#N/A	0	#N/A	0	#N/A
#N/A	0	#N/A	0	#N/A	0	#N/A
#N/A	0	#N/A	0	#N/A	0	#N/A
LOST-6_cit	1		0		0	
LOST-6_cit	1	LOST>6_cit	1	Ew/a-LOST-cit	1	Ew/p-LOST-cit
#N/A	0	#N/A	0	#N/A	0	#N/A
#N/A	0	#N/A	0	#N/A	0	#N/A
#N/A	0	#N/A	0	#N/A	0	#N/A
#N/A	0	#N/A	0	#N/A	0	#N/A
#N/A	0	#N/A	0	#N/A	0	#N/A
#N/A	0	#N/A	0	#N/A	0	#N/A
#N/A	0	#N/A	0	#N/A	0	#N/A
	0		0		0	
	0		0		0	
	0		0		0	
#N/A	0	#N/A	0	#N/A	0	#N/A
#N/A	0	#N/A	0	#N/A	0	#N/A
LOST-6_cit	1		0		0	
#N/A	0	#N/A	0	#N/A	0	#N/A
#N/A	0	#N/A	0	#N/A	0	#N/A
#N/A	0	#N/A	0	#N/A	0	#N/A
#N/A	0	#N/A	0	#N/A	0	#N/A
#N/A	0	#N/A	0	#N/A	0	#N/A
#N/A	0	#N/A	0	#N/A	0	#N/A
#N/A	0	#N/A	0	#N/A	0	#N/A
#N/A	0	#N/A	0	#N/A	0	#N/A
#N/A	0	#N/A	0	#N/A	0	#N/A
#N/A	0	#N/A	0	#N/A	0	#N/A
#N/A	0	#N/A	0	#N/A	0	#N/A
#N/A	0	#N/A	0	#N/A	0	#N/A
#N/A	0	#N/A	0	#N/A	0	#N/A
#N/A	0	#N/A	0	#N/A	0	#N/A
#N/A	0	#N/A	0	#N/A	0	#N/A
#N/A	0	#N/A	0	#N/A	0	#N/A
#N/A	0	#N/A	0	#N/A	0	#N/A
#N/A	0	#N/A	0	#N/A	0	#N/A
#N/A	0	#N/A	0	#N/A	0	#N/A
#N/A	0	#N/A	0	#N/A	0	#N/A
#N/A	0	#N/A	0	#N/A	0	#N/A
#N/A	0	#N/A	0	#N/A	0	#N/A
#N/A	0	#N/A	0	#N/A	0	#N/A
#N/A	0	#N/A	0	#N/A	0	#N/A
#N/A	0	#N/A	0	#N/A	0	#N/A
#N/A	0	#N/A	0	#N/A	0	#N/A
#N/A	0	#N/A	0	#N/A	0	#N/A
#N/A	0	#N/A	0	#N/A	0	#N/A

#N/A	0	#N/A	0	#N/A	0	#N/A
#N/A	0	#N/A	0	#N/A	0	#N/A
#N/A	0	#N/A	0	#N/A	0	#N/A
#N/A	0	#N/A	0	#N/A	0	#N/A
#N/A	0	#N/A	0	#N/A	0	#N/A
#N/A	0	#N/A	0	#N/A	0	#N/A
LOST-6_cit	1		0		0	
LOST-6_cit	1		0		0	
#N/A	0	#N/A	0	#N/A	0	#N/A
#N/A	0	#N/A	0	#N/A	0	#N/A
	0		0	Ew/a-LOST-cit	1	Ew/p-LOST-cit
	0		0	Ew/a-LOST-cit	1	Ew/p-LOST-cit
#N/A	0	#N/A	0	#N/A	0	#N/A
#N/A	0	#N/A	0	#N/A	0	#N/A
#N/A	0	#N/A	0	#N/A	0	#N/A
#N/A	0	#N/A	0	#N/A	0	#N/A
#N/A	0	#N/A	0	#N/A	0	#N/A
#N/A	0	#N/A	0	#N/A	0	#N/A
#N/A	0	#N/A	0	#N/A	0	#N/A
	0		0		0	Ew/p-LOST-cit
	0		0		0	Ew/p-LOST-cit
	0		0		0	Ew/p-LOST-cit
#N/A	0	#N/A	0	#N/A	0	#N/A
#N/A	0	#N/A	0	#N/A	0	#N/A
#N/A	0	#N/A	0	#N/A	0	#N/A
#N/A	0	#N/A	0	#N/A	0	#N/A
#N/A	0	#N/A	0	#N/A	0	#N/A
#N/A	0	#N/A	0	#N/A	0	#N/A
#N/A	0	#N/A	0	#N/A	0	#N/A
#N/A	0	#N/A	0	#N/A	0	#N/A
#N/A	0	#N/A	0	#N/A	0	#N/A
#N/A	0	#N/A	0	#N/A	0	#N/A
#N/A	0	#N/A	0	#N/A	0	#N/A
#N/A	0	#N/A	0	#N/A	0	#N/A
#N/A	0	#N/A	0	#N/A	0	#N/A
#N/A	0	#N/A	0	#N/A	0	#N/A
#N/A	0	#N/A	0	#N/A	0	#N/A
#N/A	0	#N/A	0	#N/A	0	#N/A
#N/A	0	#N/A	0	#N/A	0	#N/A
#N/A	0	#N/A	0	#N/A	0	#N/A
#N/A	0	#N/A	0	#N/A	0	#N/A
#N/A	0	#N/A	0	#N/A	0	#N/A
#N/A	0	#N/A	0	#N/A	0	#N/A
#N/A	0	#N/A	0	#N/A	0	#N/A
LOST-6_cit	1		0		0	
LOST-6_cit	1		0		0	
LOST-6_cit	1		0		0	

#N/A	0	#N/A	0	#N/A	0	#N/A
#N/A	0	#N/A	0	#N/A	0	#N/A
#N/A	0	#N/A	0	#N/A	0	#N/A
#N/A	0	#N/A	0	#N/A	0	#N/A
#N/A	0	#N/A	0	#N/A	0	#N/A
#N/A	0	#N/A	0	#N/A	0	#N/A
#N/A	0	#N/A	0	#N/A	0	#N/A
	0	LOST>6_cit	1		0	
#N/A	0	#N/A	0	#N/A	0	#N/A
#N/A	0	#N/A	0	#N/A	0	#N/A
#N/A	0	#N/A	0	#N/A	0	#N/A
LOST-6_cit	1		0	Ew/a-LOST-cit	1	
LOST-6_cit	1		0	Ew/a-LOST-cit	1	
#N/A	0	#N/A	0	#N/A	0	#N/A
#N/A	0	#N/A	0	#N/A	0	#N/A
#N/A	0	#N/A	0	#N/A	0	#N/A
	0		0	Ew/a-LOST-cit	1	Ew/p-LOST-cit
	0		0	Ew/a-LOST-cit	1	Ew/p-LOST-cit
#N/A	0	#N/A	0	#N/A	0	#N/A
#N/A	0	#N/A	0	#N/A	0	#N/A
LOST-6_cit	1		0		0	
#N/A	0	#N/A	0	#N/A	0	#N/A
#N/A	0	#N/A	0	#N/A	0	#N/A
	0		0	Ew/a-LOST-cit	1	
#N/A	0	#N/A	0	#N/A	0	#N/A
LOST-6_cit	1		0		0	
#N/A	0	#N/A	0	#N/A	0	#N/A
#N/A	0	#N/A	0	#N/A	0	#N/A
#N/A	0	#N/A	0	#N/A	0	#N/A
#N/A	0	#N/A	0	#N/A	0	#N/A
#N/A	0	#N/A	0	#N/A	0	#N/A
#N/A	0	#N/A	0	#N/A	0	#N/A
#N/A	0	#N/A	0	#N/A	0	#N/A
#N/A	0	#N/A	0	#N/A	0	#N/A
LOST-6_cit	1	LOST>6_cit	1	Ew/a-LOST-cit	1	Ew/p-LOST-cit
#N/A	0	#N/A	0	#N/A	0	#N/A
#N/A	0	#N/A	0	#N/A	0	#N/A
#N/A	0	#N/A	0	#N/A	0	#N/A
#N/A	0	#N/A	0	#N/A	0	#N/A
#N/A	0	#N/A	0	#N/A	0	#N/A
#N/A	0	#N/A	0	#N/A	0	#N/A
#N/A	0	#N/A	0	#N/A	0	#N/A
	0		0		0	
#N/A	0	#N/A	0	#N/A	0	#N/A
LOST-6_cit	1	LOST>6_cit	1		0	
#N/A	0	#N/A	0	#N/A	0	#N/A
#N/A	0	#N/A	0	#N/A	0	#N/A

#N/A	0	#N/A	0	#N/A	0	#N/A
#N/A	0	#N/A	0	#N/A	0	#N/A
#N/A	0	#N/A	0	#N/A	0	#N/A
#N/A	0	#N/A	0	#N/A	0	#N/A
#N/A	0	#N/A	0	#N/A	0	#N/A
#N/A	0	#N/A	0	#N/A	0	#N/A
LOST-6_cit	1	LOST>6_cit	1		0	
#N/A	0	#N/A	0	#N/A	0	#N/A
#N/A	0	#N/A	0	#N/A	0	#N/A
#N/A	0	#N/A	0	#N/A	0	#N/A
	0		0	Ew/a-LOST-cit	1	Ew/p-LOST-cit
#N/A	0	#N/A	0	#N/A	0	#N/A
#N/A	0	#N/A	0	#N/A	0	#N/A
#N/A	0	#N/A	0	#N/A	0	#N/A
#N/A	0	#N/A	0	#N/A	0	#N/A
#N/A	0	#N/A	0	#N/A	0	#N/A
#N/A	0	#N/A	0	#N/A	0	#N/A
#N/A	0	#N/A	0	#N/A	0	#N/A
#N/A	0	#N/A	0	#N/A	0	#N/A
#N/A	0	#N/A	0	#N/A	0	#N/A
#N/A	0	#N/A	0	#N/A	0	#N/A
	0		0	Ew/a-LOST-cit	1	
	0		0	Ew/a-LOST-cit	1	
	0		0	Ew/a-LOST-cit	1	
#N/A	0	#N/A	0	#N/A	0	#N/A
#N/A	0	#N/A	0	#N/A	0	#N/A
#N/A	0	#N/A	0	#N/A	0	#N/A
#N/A	0	#N/A	0	#N/A	0	#N/A
#N/A	0	#N/A	0	#N/A	0	#N/A
#N/A	0	#N/A	0	#N/A	0	#N/A
#N/A	0	#N/A	0	#N/A	0	#N/A
#N/A	0	#N/A	0	#N/A	0	#N/A
#N/A	0	#N/A	0	#N/A	0	#N/A
#N/A	0	#N/A	0	#N/A	0	#N/A
#N/A	0	#N/A	0	#N/A	0	#N/A
#N/A	0	#N/A	0	#N/A	0	#N/A
#N/A	0	#N/A	0	#N/A	0	#N/A
#N/A	0	#N/A	0	#N/A	0	#N/A
#N/A	0	#N/A	0	#N/A	0	#N/A
#N/A	0	#N/A	0	#N/A	0	#N/A
	0	LOST>6_cit	1		0	
#N/A	0	#N/A	0	#N/A	0	#N/A
#N/A	0	#N/A	0	#N/A	0	#N/A
LOST-6_cit	1		0	Ew/a-LOST-cit	1	
#N/A	0	#N/A	0	#N/A	0	#N/A
	0	LOST>6_cit	1		0	
#N/A	0	#N/A	0	#N/A	0	#N/A

#N/A	0	#N/A	0	#N/A	0	#N/A
#N/A	0	#N/A	0	#N/A	0	#N/A
#N/A	0	#N/A	0	#N/A	0	#N/A
#N/A	0	#N/A	0	#N/A	0	#N/A
#N/A	0	#N/A	0	#N/A	0	#N/A
#N/A	0	#N/A	0	#N/A	0	#N/A
#N/A	0	#N/A	0	#N/A	0	#N/A
LOST-6_cit	1	LOST>6_cit	1		0	
LOST-6_cit	1	LOST>6_cit	1		0	
LOST-6_cit	1	LOST>6_cit	1		0	
	0		0	Ew/a-LOST-cit	1	Ew/p-LOST-cit
	0		0	Ew/a-LOST-cit	1	Ew/p-LOST-cit
	0		0	Ew/a-LOST-cit	1	Ew/p-LOST-cit
	0		0	Ew/a-LOST-cit	1	Ew/p-LOST-cit
#N/A	0	#N/A	0	#N/A	0	#N/A
#N/A	0	#N/A	0	#N/A	0	#N/A
#N/A	0	#N/A	0	#N/A	0	#N/A
#N/A	0	#N/A	0	#N/A	0	#N/A
#N/A	0	#N/A	0	#N/A	0	#N/A
#N/A	0	#N/A	0	#N/A	0	#N/A
#N/A	0	#N/A	0	#N/A	0	#N/A
#N/A	0	#N/A	0	#N/A	0	#N/A
#N/A	0	#N/A	0	#N/A	0	#N/A
#N/A	0	#N/A	0	#N/A	0	#N/A
#N/A	0	#N/A	0	#N/A	0	#N/A
#N/A	0	#N/A	0	#N/A	0	#N/A
#N/A	0	#N/A	0	#N/A	0	#N/A
#N/A	0	#N/A	0	#N/A	0	#N/A
#N/A	0	#N/A	0	#N/A	0	#N/A
#N/A	0	#N/A	0	#N/A	0	#N/A
#N/A	0	#N/A	0	#N/A	0	#N/A
#N/A	0	#N/A	0	#N/A	0	#N/A
LOST-6_cit	1		0		0	
LOST-6_cit	1		0		0	
#N/A	0	#N/A	0	#N/A	0	#N/A
#N/A	0	#N/A	0	#N/A	0	#N/A
#N/A	0	#N/A	0	#N/A	0	#N/A
	0		0	Ew/a-LOST-cit	1	Ew/p-LOST-cit
	0		0	Ew/a-LOST-cit	1	Ew/p-LOST-cit
#N/A	0	#N/A	0	#N/A	0	#N/A
#N/A	0	#N/A	0	#N/A	0	#N/A
LOST-6_cit	1		0		0	
#N/A	0	#N/A	0	#N/A	0	#N/A
	0		0	Ew/a-LOST-cit	1	Ew/p-LOST-cit
	0		0	Ew/a-LOST-cit	1	Ew/p-LOST-cit
	0		0	Ew/a-LOST-cit	1	Ew/p-LOST-cit
#N/A	0	#N/A	0	#N/A	0	#N/A

#N/A	0	#N/A	0	#N/A	0	#N/A
#N/A	0	#N/A	0	#N/A	0	#N/A
#N/A	0	#N/A	0	#N/A	0	#N/A
#N/A	0	#N/A	0	#N/A	0	#N/A
#N/A	0	#N/A	0	#N/A	0	#N/A
#N/A	0	#N/A	0	#N/A	0	#N/A
#N/A	0	#N/A	0	#N/A	0	#N/A
	0		0	Ew/a-LOST-cit	1	
	0		0	Ew/a-LOST-cit	1	
#N/A	0	#N/A	0	#N/A	0	#N/A
#N/A	0	#N/A	0	#N/A	0	#N/A
#N/A	0	#N/A	0	#N/A	0	#N/A
LOST-6_cit	1	LOST>6_cit	1		0	
#N/A	0	#N/A	0	#N/A	0	#N/A
#N/A	0	#N/A	0	#N/A	0	#N/A
#N/A	0	#N/A	0	#N/A	0	#N/A
#N/A	0	#N/A	0	#N/A	0	#N/A
#N/A	0	#N/A	0	#N/A	0	#N/A
#N/A	0	#N/A	0	#N/A	0	#N/A
#N/A	0	#N/A	0	#N/A	0	#N/A
	0		0		0	
#N/A	0	#N/A	0	#N/A	0	#N/A
	0		0	Ew/a-LOST-cit	1	Ew/p-LOST-cit
	0		0	Ew/a-LOST-cit	1	Ew/p-LOST-cit
LOST-6_cit	1	LOST>6_cit	1		0	
#N/A	0	#N/A	0	#N/A	0	#N/A
#N/A	0	#N/A	0	#N/A	0	#N/A
#N/A	0	#N/A	0	#N/A	0	#N/A
#N/A	0	#N/A	0	#N/A	0	#N/A
LOST-6_cit	1		0		0	
#N/A	0	#N/A	0	#N/A	0	#N/A
#N/A	0	#N/A	0	#N/A	0	#N/A
#N/A	0	#N/A	0	#N/A	0	#N/A
#N/A	0	#N/A	0	#N/A	0	#N/A
#N/A	0	#N/A	0	#N/A	0	#N/A
#N/A	0	#N/A	0	#N/A	0	#N/A
#N/A	0	#N/A	0	#N/A	0	#N/A
#N/A	0	#N/A	0	#N/A	0	#N/A
#N/A	0	#N/A	0	#N/A	0	#N/A
#N/A	0	#N/A	0	#N/A	0	#N/A
#N/A	0	#N/A	0	#N/A	0	#N/A
#N/A	0	#N/A	0	#N/A	0	#N/A
LOST-6_cit	1	LOST>6_cit	1		0	
LOST-6_cit	1		0	Ew/a-LOST-cit	1	Ew/p-LOST-cit
LOST-6_cit	1		0	Ew/a-LOST-cit	1	Ew/p-LOST-cit
LOST-6_cit	1		0	Ew/a-LOST-cit	1	Ew/p-LOST-cit
LOST-6_cit	1		0	Ew/a-LOST-cit	1	Ew/p-LOST-cit

#N/A	0	#N/A	0	#N/A	0	#N/A
#N/A	0	#N/A	0	#N/A	0	#N/A
#N/A	0	#N/A	0	#N/A	0	#N/A
#N/A	0	#N/A	0	#N/A	0	#N/A
#N/A	0	#N/A	0	#N/A	0	#N/A
#N/A	0	#N/A	0	#N/A	0	#N/A
#N/A	0	#N/A	0	#N/A	0	#N/A
#N/A	0	#N/A	0	#N/A	0	#N/A
#N/A	0	#N/A	0	#N/A	0	#N/A
#N/A	0	#N/A	0	#N/A	0	#N/A
#N/A	0	#N/A	0	#N/A	0	#N/A
	0		0		0	
#N/A	0	#N/A	0	#N/A	0	#N/A
#N/A	0	#N/A	0	#N/A	0	#N/A
#N/A	0	#N/A	0	#N/A	0	#N/A
#N/A	0	#N/A	0	#N/A	0	#N/A
#N/A	0	#N/A	0	#N/A	0	#N/A
#N/A	0	#N/A	0	#N/A	0	#N/A
#N/A	0	#N/A	0	#N/A	0	#N/A
#N/A	0	#N/A	0	#N/A	0	#N/A
#N/A	0	#N/A	0	#N/A	0	#N/A
#N/A	0	#N/A	0	#N/A	0	#N/A
#N/A	0	#N/A	0	#N/A	0	#N/A
#N/A	0	#N/A	0	#N/A	0	#N/A
#N/A	0	#N/A	0	#N/A	0	#N/A
#N/A	0	#N/A	0	#N/A	0	#N/A
#N/A	0	#N/A	0	#N/A	0	#N/A
#N/A	0	#N/A	0	#N/A	0	#N/A
#N/A	0	#N/A	0	#N/A	0	#N/A
#N/A	0	#N/A	0	#N/A	0	#N/A
#N/A	0	#N/A	0	#N/A	0	#N/A
#N/A	0	#N/A	0	#N/A	0	#N/A
#N/A	0	#N/A	0	#N/A	0	#N/A
#N/A	0	#N/A	0	#N/A	0	#N/A
	0		0	Ew/a-LOST-cit	1	Ew/p-LOST-cit
	0		0	Ew/a-LOST-cit	1	Ew/p-LOST-cit
#N/A	0	#N/A	0	#N/A	0	#N/A
#N/A	0	#N/A	0	#N/A	0	#N/A
#N/A	0	#N/A	0	#N/A	0	#N/A
	0		0	Ew/a-LOST-cit	1	Ew/p-LOST-cit
	0		0	Ew/a-LOST-cit	1	Ew/p-LOST-cit
	0		0	Ew/a-LOST-cit	1	Ew/p-LOST-cit
	0		0		0	
	0		0		0	
#N/A	0	#N/A	0	#N/A	0	#N/A
#N/A	0	#N/A	0	#N/A	0	#N/A
#N/A	0	#N/A	0	#N/A	0	#N/A

#N/A	0	#N/A	0	#N/A	0	#N/A
#N/A	0	#N/A	0	#N/A	0	#N/A
#N/A	0	#N/A	0	#N/A	0	#N/A
#N/A	0	#N/A	0	#N/A	0	#N/A
LOST-6_cit	1		0		0	
#N/A	0	#N/A	0	#N/A	0	#N/A
#N/A	0	#N/A	0	#N/A	0	#N/A
#N/A	0	#N/A	0	#N/A	0	#N/A
LOST-6_cit	1	LOST>6_cit	1		0	
LOST-6_cit	1	LOST>6_cit	1		0	
LOST-6_cit	1	LOST>6_cit	1		0	
LOST-6_cit	1	LOST>6_cit	1		0	
LOST-6_cit	1	LOST>6_cit	1		0	
	0		0	Ew/a-LOST-cit	1	Ew/p-LOST-cit
#N/A	0	#N/A	0	#N/A	0	#N/A
#N/A	0	#N/A	0	#N/A	0	#N/A
#N/A	0	#N/A	0	#N/A	0	#N/A
#N/A	0	#N/A	0	#N/A	0	#N/A
#N/A	0	#N/A	0	#N/A	0	#N/A
#N/A	0	#N/A	0	#N/A	0	#N/A
#N/A	0	#N/A	0	#N/A	0	#N/A
	0	LOST>6_cit	1	Ew/a-LOST-cit	1	
	0	LOST>6_cit	1	Ew/a-LOST-cit	1	
#N/A	0	#N/A	0	#N/A	0	#N/A
#N/A	0	#N/A	0	#N/A	0	#N/A
#N/A	0	#N/A	0	#N/A	0	#N/A
#N/A	0	#N/A	0	#N/A	0	#N/A
LOST-6_cit	1		0		0	Ew/p-LOST-cit
LOST-6_cit	1		0		0	Ew/p-LOST-cit
#N/A	0	#N/A	0	#N/A	0	#N/A
#N/A	0	#N/A	0	#N/A	0	#N/A
#N/A	0	#N/A	0	#N/A	0	#N/A
#N/A	0	#N/A	0	#N/A	0	#N/A
	0		0		0	Ew/p-LOST-cit
	0		0		0	Ew/p-LOST-cit
#N/A	0	#N/A	0	#N/A	0	#N/A
#N/A	0	#N/A	0	#N/A	0	#N/A
#N/A	0	#N/A	0	#N/A	0	#N/A
	0	LOST>6_cit	1	Ew/a-LOST-cit	1	
LOST-6_cit	1	LOST>6_cit	1		0	
#N/A	0	#N/A	0	#N/A	0	#N/A
#N/A	0	#N/A	0	#N/A	0	#N/A
#N/A	0	#N/A	0	#N/A	0	#N/A
#N/A	0	#N/A	0	#N/A	0	#N/A
#N/A	0	#N/A	0	#N/A	0	#N/A
#N/A	0	#N/A	0	#N/A	0	#N/A
#N/A	0	#N/A	0	#N/A	0	#N/A

#N/A	0	#N/A	0	#N/A	0	#N/A
LOST-6_cit	1		0	Ew/a-LOST-cit	1	Ew/p-LOST-cit
#N/A	0	#N/A	0	#N/A	0	#N/A
#N/A	0	#N/A	0	#N/A	0	#N/A
#N/A	0	#N/A	0	#N/A	0	#N/A
#N/A	0	#N/A	0	#N/A	0	#N/A
#N/A	0	#N/A	0	#N/A	0	#N/A
#N/A	0	#N/A	0	#N/A	0	#N/A
#N/A	0	#N/A	0	#N/A	0	#N/A
#N/A	0	#N/A	0	#N/A	0	#N/A
#N/A	0	#N/A	0	#N/A	0	#N/A
#N/A	0	#N/A	0	#N/A	0	#N/A
#N/A	0	#N/A	0	#N/A	0	#N/A
#N/A	0	#N/A	0	#N/A	0	#N/A
#N/A	0	#N/A	0	#N/A	0	#N/A
#N/A	0	#N/A	0	#N/A	0	#N/A
#N/A	0	#N/A	0	#N/A	0	#N/A
#N/A	0	#N/A	0	#N/A	0	#N/A
#N/A	0	#N/A	0	#N/A	0	#N/A
#N/A	0	#N/A	0	#N/A	0	#N/A
	0		0	Ew/a-LOST-cit	1	Ew/p-LOST-cit
#N/A	0	#N/A	0	#N/A	0	#N/A
	0		0		0	
#N/A	0	#N/A	0	#N/A	0	#N/A
#N/A	0	#N/A	0	#N/A	0	#N/A
	0		0		0	
#N/A	0	#N/A	0	#N/A	0	#N/A
	0		0	Ew/a-LOST-cit	1	Ew/p-LOST-cit
	0		0	Ew/a-LOST-cit	1	Ew/p-LOST-cit
#N/A	0	#N/A	0	#N/A	0	#N/A
#N/A	0	#N/A	0	#N/A	0	#N/A
#N/A	0	#N/A	0	#N/A	0	#N/A
#N/A	0	#N/A	0	#N/A	0	#N/A
#N/A	0	#N/A	0	#N/A	0	#N/A
#N/A	0	#N/A	0	#N/A	0	#N/A
#N/A	0	#N/A	0	#N/A	0	#N/A
#N/A	0	#N/A	0	#N/A	0	#N/A
#N/A	0	#N/A	0	#N/A	0	#N/A
#N/A	0	#N/A	0	#N/A	0	#N/A
#N/A	0	#N/A	0	#N/A	0	#N/A
LOST-6_cit	1		0	Ew/a-LOST-cit	1	Ew/p-LOST-cit
LOST-6_cit	1		0	Ew/a-LOST-cit	1	Ew/p-LOST-cit
LOST-6_cit	1		0	Ew/a-LOST-cit	1	Ew/p-LOST-cit
#N/A	0	#N/A	0	#N/A	0	#N/A
#N/A	0	#N/A	0	#N/A	0	#N/A
#N/A	0	#N/A	0	#N/A	0	#N/A
#N/A	0	#N/A	0	#N/A	0	#N/A

#N/A	0	#N/A	0	#N/A	0	#N/A
#N/A	0	#N/A	0	#N/A	0	#N/A
#N/A	0	#N/A	0	#N/A	0	#N/A
#N/A	0	#N/A	0	#N/A	0	#N/A
LOST-6_cit	1	LOST>6_cit	1		0	
LOST-6_cit	1	LOST>6_cit	1		0	
#N/A	0	#N/A	0	#N/A	0	#N/A
#N/A	0	#N/A	0	#N/A	0	#N/A
#N/A	0	#N/A	0	#N/A	0	#N/A
#N/A	0	#N/A	0	#N/A	0	#N/A
#N/A	0	#N/A	0	#N/A	0	#N/A
#N/A	0	#N/A	0	#N/A	0	#N/A
#N/A	0	#N/A	0	#N/A	0	#N/A
#N/A	0	#N/A	0	#N/A	0	#N/A
#N/A	0	#N/A	0	#N/A	0	#N/A
#N/A	0	#N/A	0	#N/A	0	#N/A
#N/A	0	#N/A	0	#N/A	0	#N/A
#N/A	0	#N/A	0	#N/A	0	#N/A
	0		0		0	Ew/p-LOST-cit
	0		0		0	Ew/p-LOST-cit
	0		0		0	Ew/p-LOST-cit
#N/A	0	#N/A	0	#N/A	0	#N/A
#N/A	0	#N/A	0	#N/A	0	#N/A
#N/A	0	#N/A	0	#N/A	0	#N/A
#N/A	0	#N/A	0	#N/A	0	#N/A
#N/A	0	#N/A	0	#N/A	0	#N/A
#N/A	0	#N/A	0	#N/A	0	#N/A
	0		0		0	
#N/A	0	#N/A	0	#N/A	0	#N/A
#N/A	0	#N/A	0	#N/A	0	#N/A
#N/A	0	#N/A	0	#N/A	0	#N/A
#N/A	0	#N/A	0	#N/A	0	#N/A
#N/A	0	#N/A	0	#N/A	0	#N/A
#N/A	0	#N/A	0	#N/A	0	#N/A
#N/A	0	#N/A	0	#N/A	0	#N/A
	0	LOST>6_cit	1		0	
	0	LOST>6_cit	1		0	
	0		0	Ew/a-LOST-cit	1	
#N/A	0	#N/A	0	#N/A	0	#N/A
#N/A	0	#N/A	0	#N/A	0	#N/A
#N/A	0	#N/A	0	#N/A	0	#N/A
#N/A	0	#N/A	0	#N/A	0	#N/A
#N/A	0	#N/A	0	#N/A	0	#N/A
	0		0		0	
#N/A	0	#N/A	0	#N/A	0	#N/A
#N/A	0	#N/A	0	#N/A	0	#N/A
#N/A	0	#N/A	0	#N/A	0	#N/A

#N/A	0	#N/A	0	#N/A	0	#N/A
#N/A	0	#N/A	0	#N/A	0	#N/A
#N/A	0	#N/A	0	#N/A	0	#N/A
LOST-6_cit	1		0	Ew/a-LOST-cit	1	Ew/p-LOST-cit
#N/A	0	#N/A	0	#N/A	0	#N/A
#N/A	0	#N/A	0	#N/A	0	#N/A
#N/A	0	#N/A	0	#N/A	0	#N/A
#N/A	0	#N/A	0	#N/A	0	#N/A
#N/A	0	#N/A	0	#N/A	0	#N/A
#N/A	0	#N/A	0	#N/A	0	#N/A
#N/A	0	#N/A	0	#N/A	0	#N/A
#N/A	0	#N/A	0	#N/A	0	#N/A
	0		0		0	
	0		0		0	
	0		0		0	
#N/A	0	#N/A	0	#N/A	0	#N/A
#N/A	0	#N/A	0	#N/A	0	#N/A
#N/A	0	#N/A	0	#N/A	0	#N/A
#N/A	0	#N/A	0	#N/A	0	#N/A
#N/A	0	#N/A	0	#N/A	0	#N/A
#N/A	0	#N/A	0	#N/A	0	#N/A
#N/A	0	#N/A	0	#N/A	0	#N/A
#N/A	0	#N/A	0	#N/A	0	#N/A
#N/A	0	#N/A	0	#N/A	0	#N/A
LOST-6_cit	1		0		0	
#N/A	0	#N/A	0	#N/A	0	#N/A
#N/A	0	#N/A	0	#N/A	0	#N/A
#N/A	0	#N/A	0	#N/A	0	#N/A
#N/A	0	#N/A	0	#N/A	0	#N/A
#N/A	0	#N/A	0	#N/A	0	#N/A
#N/A	0	#N/A	0	#N/A	0	#N/A
#N/A	0	#N/A	0	#N/A	0	#N/A
#N/A	0	#N/A	0	#N/A	0	#N/A
#N/A	0	#N/A	0	#N/A	0	#N/A
LOST-6_cit	1		0		0	
LOST-6_cit	1		0		0	
LOST-6_cit	1		0		0	
#N/A	0	#N/A	0	#N/A	0	#N/A
#N/A	0	#N/A	0	#N/A	0	#N/A
#N/A	0	#N/A	0	#N/A	0	#N/A
#N/A	0	#N/A	0	#N/A	0	#N/A
#N/A	0	#N/A	0	#N/A	0	#N/A
#N/A	0	#N/A	0	#N/A	0	#N/A
#N/A	0	#N/A	0	#N/A	0	#N/A
#N/A	0	#N/A	0	#N/A	0	#N/A
#N/A	0	#N/A	0	#N/A	0	#N/A
#N/A	0	#N/A	0	#N/A	0	#N/A

#N/A	0	#N/A	0	#N/A	0	#N/A
#N/A	0	#N/A	0	#N/A	0	#N/A
#N/A	0	#N/A	0	#N/A	0	#N/A
#N/A	0	#N/A	0	#N/A	0	#N/A
#N/A	0	#N/A	0	#N/A	0	#N/A
#N/A	0	#N/A	0	#N/A	0	#N/A
#N/A	0	#N/A	0	#N/A	0	#N/A
LOST-6_cit	1	LOST>6_cit	1		0	
LOST-6_cit	1	LOST>6_cit	1		0	
#N/A	0	#N/A	0	#N/A	0	#N/A
LOST-6_cit	1	LOST>6_cit	1		0	Ew/p-LOST-cit
	0		0	Ew/a-LOST-cit	1	Ew/p-LOST-cit
#N/A	0	#N/A	0	#N/A	0	#N/A
#N/A	0	#N/A	0	#N/A	0	#N/A
#N/A	0	#N/A	0	#N/A	0	#N/A
#N/A	0	#N/A	0	#N/A	0	#N/A
LOST-6_cit	1		0		0	
LOST-6_cit	1		0		0	
	0		0	Ew/a-LOST-cit	1	Ew/p-LOST-cit
	0		0	Ew/a-LOST-cit	1	Ew/p-LOST-cit
	0		0	Ew/a-LOST-cit	1	Ew/p-LOST-cit
	0		0	Ew/a-LOST-cit	1	Ew/p-LOST-cit
#N/A	0	#N/A	0	#N/A	0	#N/A
#N/A	0	#N/A	0	#N/A	0	#N/A
#N/A	0	#N/A	0	#N/A	0	#N/A
#N/A	0	#N/A	0	#N/A	0	#N/A
LOST-6_cit	1		0		0	
LOST-6_cit	1		0		0	
LOST-6_cit	1		0		0	
#N/A	0	#N/A	0	#N/A	0	#N/A
#N/A	0	#N/A	0	#N/A	0	#N/A
#N/A	0	#N/A	0	#N/A	0	#N/A

ewplostcit_b	ewaplostcit	ewaplostcit_b	entryrgglobal	ewargart	ewargart_b	ewargtopart	ewargtopart_
0	#N/A	0			0		0
0	#N/A	0		Ewa-RG-art	1		0
0	#N/A	0			0		0
0	#N/A	0			0		0
0		0		Ewa-RG-art	1		0
0		0		Ewa-RG-art	1		0
0		0		Ewa-RG-art	1		0
0		0		Ewa-RG-art	1		0
0	#N/A	0		Ewa-RG-art	1		0
0	#N/A	0		Ewa-RG-art	1		0
0	#N/A	0		Ewa-RG-art	1		0
0	#N/A	0		Ewa-RG-art	1		0
0	#N/A	0			0		0
0		0			0		0
0	#N/A	0		Ewa-RG-art	1		0
0	#N/A	0		Ewa-RG-art	1		0
0	#N/A	0			0		0
0	#N/A	0		Ewa-RG-art	1		0
0	#N/A	0			0		0
0	#N/A	0			0		0
0	#N/A	0			0		0
0	#N/A	0			0		0
0	#N/A	0		Ewa-RG-art	1		0
0	#N/A	0		Ewa-RG-art	1		0
0	#N/A	0		Ewa-RG-art	1		0
0	#N/A	0			0		0
0	#N/A	0		Ewa-RG-art	1		0
0	#N/A	0		Ewa-RG-art	1		0
0	#N/A	0		Ewa-RG-art	1		0
0	#N/A	0		Ewa-RG-art	1		0
0	#N/A	0		Ewa-RG-art	1		0
0		0			0		0
0	#N/A	0			0		0
0	#N/A	0		Ewa-RG-art	1		0
0	#N/A	0		Ewa-RG-art	1		0
0		0			0		0
0		0			0		0
0	#N/A	0		Ewa-RG-art	1		0
0	#N/A	0			0		0
0	#N/A	0		Ewa-RG-art	1		0
0	#N/A	0		Ewa-RG-art	1		0
0	#N/A	0		Ewa-RG-art	1		0
0	#N/A	0		Ewa-RG-art	1		0
0	#N/A	0		Ewa-RG-art	1		0
0	#N/A	0		Ewa-RG-art	1		0
0	#N/A	0			0		0

0	#N/A	0		0		0
1	Ew/a-p-LOST-	1	Ewa-RG-art	1	Ewa-RG-top-a	1
0	#N/A	0	Ewa-RG-art	1		0
0	#N/A	0	Ewa-RG-art	1		0
0	#N/A	0	Ewa-RG-art	1		0
0	#N/A	0	Ewa-RG-art	1		0
0	#N/A	0	Ewa-RG-art	1		0
1	Ew/a-p-LOST-	1	Ewa-RG-art	1	Ewa-RG-top-a	1
1	Ew/a-p-LOST-	1		0		0
0	#N/A	0		0		0
0	#N/A	0		0		0
0	#N/A	0		0		0
0		0	Ewa-RG-art	1	Ewa-RG-top-a	1
0	#N/A	0	Ewa-RG-art	1		0
0	#N/A	0		0		0
0	#N/A	0		0		0
0	#N/A	0		0		0
0	#N/A	0		0		0
0	#N/A	0		0		0
0	#N/A	0		0		0
0	#N/A	0	Ewa-RG-art	1	Ewa-RG-top-a	1
0	#N/A	0	Ewa-RG-art	1	Ewa-RG-top-a	1
0	#N/A	0	Ewa-RG-art	1		0
0	#N/A	0		0		0
0	#N/A	0	Ewa-RG-art	1	Ewa-RG-top-a	1
0	#N/A	0	Ewa-RG-art	1	Ewa-RG-top-a	1
0	#N/A	0	Ewa-RG-art	1		0
0	#N/A	0	Ewa-RG-art	1		0
0	#N/A	0	Ewa-RG-art	1		0
0	#N/A	0	Ewa-RG-art	1		0
0	#N/A	0	Ewa-RG-art	1		0
0	#N/A	0	Ewa-RG-art	1		0
0	#N/A	0	Ewa-RG-art	1		0
0	#N/A	0	Ewa-RG-art	1		0
0	#N/A	0		0		0
0	#N/A	0		0		0
0	#N/A	0		0		0
0	#N/A	0		0		0
0		0	Ewa-RG-art	1	Ewa-RG-top-a	1
0	#N/A	0	Ewa-RG-art	1		0
0	#N/A	0	Ewa-RG-art	1		0
0	#N/A	0	Ewa-RG-art	1		0
0	#N/A	0	Ewa-RG-art	1		0
0	#N/A	0	Ewa-RG-art	1		0
0	#N/A	0		0		0
0	#N/A	0	Ewa-RG-art	1		0
1	Ew/a-p-LOST-	1	Ewa-RG-art	1		0

1	Ew/a-p-LOST-	1	Ewa-RG-art	1		0
0	#N/A	0		0		0
0	#N/A	0	Ewa-RG-art	1		0
0	#N/A	0	Ewa-RG-art	1		0
0	#N/A	0	Ewa-RG-art	1		0
0	#N/A	0	Ewa-RG-art	1		0
1	Ew/a-p-LOST-	1	Ewa-RG-art	1	Ewa-RG-top-a	1
1	Ew/a-p-LOST-	1	Ewa-RG-art	1	Ewa-RG-top-a	1
0	#N/A	0	Ewa-RG-art	1		0
0	#N/A	0		0		0
0	#N/A	0		0		0
0	#N/A	0	Ewa-RG-art	1		0
0	#N/A	0	Ewa-RG-art	1		0
0	#N/A	0	Ewa-RG-art	1	Ewa-RG-top-a	1
0	#N/A	0		0		0
0	#N/A	0	Ewa-RG-art	1		0
0	#N/A	0	Ewa-RG-art	1		0
0	#N/A	0	Ewa-RG-art	1		0
0	#N/A	0	Ewa-RG-art	1		0
0	#N/A	0		0		0
1	Ew/a-p-LOST-	1	Ewa-RG-art	1		0
0	#N/A	0	Ewa-RG-art	1		0
0	#N/A	0	Ewa-RG-art	1		0
0	#N/A	0		0		0
0	#N/A	0		0		0
0	#N/A	0	Ewa-RG-art	1		0
0	#N/A	0	Ewa-RG-art	1		0
1	Ew/a-p-LOST-	1	Ewa-RG-art	1		0
1	Ew/a-p-LOST-	1	Ewa-RG-art	1		0
1	Ew/a-p-LOST-	1	Ewa-RG-art	1		0
1	Ew/a-p-LOST-	1	Ewa-RG-art	1		0
0	#N/A	0	Ewa-RG-art	1		0
1	Ew/a-p-LOST-	1	Ewa-RG-art	1		0
1	Ew/a-p-LOST-	1	Ewa-RG-art	1		0
1	Ew/a-p-LOST-	1	Ewa-RG-art	1		0
0	#N/A	0	Ewa-RG-art	1		0
0	#N/A	0	Ewa-RG-art	1		0
0	#N/A	0	Ewa-RG-art	1		0
0	#N/A	0	Ewa-RG-art	1		0
1	Ew/a-p-LOST-	1	Ewa-RG-art	1		0
0	#N/A	0	Ewa-RG-art	1		0
0	#N/A	0	Ewa-RG-art	1		0
0	#N/A	0	Ewa-RG-art	1	Ewa-RG-top-a	1
0	#N/A	0		0		0
0		0	Ewa-RG-art	1		0
0		0	Ewa-RG-art	1		0
0	#N/A	0	Ewa-RG-art	1		0

0	#N/A	0	Ewa-RG-art	1		0
0	#N/A	0	Ewa-RG-art	1		0
0	#N/A	0	Ewa-RG-art	1		0
0	#N/A	0	Ewa-RG-art	1		0
0	#N/A	0	Ewa-RG-art	1		0
0	#N/A	0		0		0
0	#N/A	0	Ewa-RG-art	1		0
0	#N/A	0	Ewa-RG-art	1		0
0	#N/A	0		0		0
0	#N/A	0		0		0
0	#N/A	0		0		0
1	Ew/a-p-LOST-	1		0		0
1	Ew/a-p-LOST-	1		0		0
1	Ew/a-p-LOST-	1	Ewa-RG-art	1	Ewa-RG-top-a	1
0	#N/A	0	Ewa-RG-art	1		0
0	#N/A	0	Ewa-RG-art	1		0
1	Ew/a-p-LOST-	1	Ewa-RG-art	1	Ewa-RG-top-a	1
0	#N/A	0	Ewa-RG-art	1		0
0	#N/A	0	Ewa-RG-art	1		0
0	#N/A	0	Ewa-RG-art	1		0
0	#N/A	0		0		0
0	#N/A	0		0		0
1	Ew/a-p-LOST-	1	Ewa-RG-art	1		0
0	#N/A	0		0		0
0	#N/A	0		0		0
0	#N/A	0		0		0
0	#N/A	0	Ewa-RG-art	1		0
0	#N/A	0	Ewa-RG-art	1		0
0	#N/A	0		0		0
1	Ew/a-p-LOST-	1	Ewa-RG-art	1	Ewa-RG-top-a	1
0	#N/A	0	Ewa-RG-art	1		0
0	#N/A	0	Ewa-RG-art	1		0
1	Ew/a-p-LOST-	1	Ewa-RG-art	1		0
0	#N/A	0		0		0
0	#N/A	0	Ewa-RG-art	1		0
0	#N/A	0	Ewa-RG-art	1		0
0	#N/A	0	Ewa-RG-art	1		0
0	#N/A	0	Ewa-RG-art	1		0
1	Ew/a-p-LOST-	1	Ewa-RG-art	1	Ewa-RG-top-a	1
0	#N/A	0		0		0
0	#N/A	0	Ewa-RG-art	1		0
0	#N/A	0	Ewa-RG-art	1		0
0	#N/A	0	Ewa-RG-art	1		0
0	#N/A	0		0		0
0	#N/A	0	Ewa-RG-art	1		0
0	#N/A	0	Ewa-RG-art	1		0
0	#N/A	0	Ewa-RG-art	1		0

0	#N/A	0	Ewa-RG-art	1		0
0	#N/A	0	Ewa-RG-art	1		0
0	#N/A	0	Ewa-RG-art	1		0
0	#N/A	0	Ewa-RG-art	1		0
1	Ew/a-p-LOST-	1	Ewa-RG-art	1		0
1	Ew/a-p-LOST-	1	Ewa-RG-art	1		0
0	#N/A	0	Ewa-RG-art	1		0
0	#N/A	0	Ewa-RG-art	1		0
0	#N/A	0	Ewa-RG-art	1		0
0	#N/A	0	Ewa-RG-art	1		0
0	#N/A	0		0		0
0	#N/A	0		0		0
0	#N/A	0	Ewa-RG-art	1		0
0	#N/A	0	Ewa-RG-art	1		0
0	#N/A	0	Ewa-RG-art	1		0
0		0	Ewa-RG-art	1		0
0		0	Ewa-RG-art	1		0
0	#N/A	0	Ewa-RG-art	1		0
0	#N/A	0		0		0
0	#N/A	0	Ewa-RG-art	1		0
0	#N/A	0	Ewa-RG-art	1		0
0	#N/A	0	Ewa-RG-art	1		0
0	#N/A	0	Ewa-RG-art	1		0
0	#N/A	0	Ewa-RG-art	1		0
0	Ew/a-p-LOST-	1	Ewa-RG-art	1	Ewa-RG-top-a	1
0	Ew/a-p-LOST-	1	Ewa-RG-art	1	Ewa-RG-top-a	1
0	#N/A	0	Ewa-RG-art	1	Ewa-RG-top-a	1
0	#N/A	0	Ewa-RG-art	1	Ewa-RG-top-a	1
0	#N/A	0	Ewa-RG-art	1	Ewa-RG-top-a	1
0	#N/A	0	Ewa-RG-art	1		0
0	#N/A	0	Ewa-RG-art	1		0
0	#N/A	0		0		0
0	#N/A	0		0		0
0	#N/A	0		0		0
0		0		0		0
0	#N/A	0		0		0
0	#N/A	0	Ewa-RG-art	1		0
0	#N/A	0	Ewa-RG-art	1		0
1	Ew/a-p-LOST-	1	Ewa-RG-art	1		0
1	Ew/a-p-LOST-	1	Ewa-RG-art	1		0
1	Ew/a-p-LOST-	1	Ewa-RG-art	1		0
1	Ew/a-p-LOST-	1	Ewa-RG-art	1		0
0	#N/A	0	Ewa-RG-art	1		0
1	Ew/a-p-LOST-	1	Ewa-RG-art	1		0
0	#N/A	0	Ewa-RG-art	1		0
0	#N/A	0	Ewa-RG-art	1		0
0	#N/A	0		0		0

0	#N/A	0	Ewa-RG-art	1		0
0	#N/A	0	Ewa-RG-art	1		0
0	#N/A	0	Ewa-RG-art	1		0
0	#N/A	0	Ewa-RG-art	1		0
0	#N/A	0	Ewa-RG-art	1		0
0	#N/A	0	Ewa-RG-art	1		0
0	#N/A	0	Ewa-RG-art	1		0
0	#N/A	0	Ewa-RG-art	1		0
0	#N/A	0	Ewa-RG-art	1		0
0	#N/A	0	Ewa-RG-art	1	Ewa-RG-top-a	1
0	#N/A	0		0		0
0	#N/A	0		0		0
0	#N/A	0	Ewa-RG-art	1		0
0	#N/A	0	Ewa-RG-art	1		0
0	#N/A	0	Ewa-RG-art	1	Ewa-RG-top-a	1
0	#N/A	0		0		0
0	#N/A	0	Ewa-RG-art	1		0
0	#N/A	0	Ewa-RG-art	1		0
0	#N/A	0	Ewa-RG-art	1		0
0	#N/A	0		0		0
0	#N/A	0		0		0
1	Ew/a-p-LOST-	1	Ewa-RG-art	1		0
1	Ew/a-p-LOST-	1	Ewa-RG-art	1		0
1	Ew/a-p-LOST-	1	Ewa-RG-art	1		0
0	#N/A	0	Ewa-RG-art	1		0
0	#N/A	0		0		0
0	#N/A	0	Ewa-RG-art	1		0
0	#N/A	0	Ewa-RG-art	1		0
0	#N/A	0		0		0
0	#N/A	0		0		0
1	Ew/a-p-LOST-	1	Ewa-RG-art	1		0
1	Ew/a-p-LOST-	1	Ewa-RG-art	1		0
1	Ew/a-p-LOST-	1	Ewa-RG-art	1		0
0	#N/A	0		0		0
0		0		0		0
0	#N/A	0	Ewa-RG-art	1		0
0	#N/A	0	Ewa-RG-art	1		0
0	#N/A	0	Ewa-RG-art	1		0
0	#N/A	0	Ewa-RG-art	1		0
0	#N/A	0	Ewa-RG-art	1		0
0	#N/A	0	Ewa-RG-art	1		0
0	#N/A	0	Ewa-RG-art	1		0
0	#N/A	0	Ewa-RG-art	1		0
0	#N/A	0	Ewa-RG-art	1		0
0		0		0		0
0	#N/A	0		0		0
0	#N/A	0	Ewa-RG-art	1		0

0	#N/A	0	Ewa-RG-art	1	Ewa-RG-top-a	1
0	#N/A	0	Ewa-RG-art	1	Ewa-RG-top-a	1
0	Ew/a-p-LOST-	1	Ewa-RG-art	1		0
0		0		0		0
0	#N/A	0	Ewa-RG-art	1		0
0	#N/A	0	Ewa-RG-art	1		0
1	Ew/a-p-LOST-	1	Ewa-RG-art	1		0
0	#N/A	0		0		0
0	#N/A	0	Ewa-RG-art	1		0
0	#N/A	0		0		0
0	#N/A	0		0		0
0	#N/A	0	Ewa-RG-art	1		0
0	#N/A	0	Ewa-RG-art	1		0
0	#N/A	0	Ewa-RG-art	1		0
0	#N/A	0	Ewa-RG-art	1		0
0	#N/A	0	Ewa-RG-art	1	Ewa-RG-top-a	1
0	#N/A	0	Ewa-RG-art	1	Ewa-RG-top-a	1
0	#N/A	0	Ewa-RG-art	1		0
0	#N/A	0	Ewa-RG-art	1		0
0	#N/A	0	Ewa-RG-art	1		0
0	#N/A	0		0		0
0	#N/A	0		0		0
0	#N/A	0	Ewa-RG-art	1		0
0	#N/A	0	Ewa-RG-art	1		0
0	#N/A	0	Ewa-RG-art	1		0
0	#N/A	0	Ewa-RG-art	1		0
0	#N/A	0	Ewa-RG-art	1		0
0	#N/A	0	Ewa-RG-art	1		0
0	#N/A	0	Ewa-RG-art	1		0
0	#N/A	0	Ewa-RG-art	1		0
0	#N/A	0	Ewa-RG-art	1		0
0	#N/A	0		0		0
0	#N/A	0	Ewa-RG-art	1		0
0	#N/A	0		0		0
0		0		0		0
0		0		0		0
0	#N/A	0	Ewa-RG-art	1		0
0	#N/A	0		0		0
0	#N/A	0	Ewa-RG-art	1		0
0	#N/A	0	Ewa-RG-art	1		0
0	#N/A	0		0		0
0	#N/A	0	Ewa-RG-art	1		0
0	#N/A	0	Ewa-RG-art	1		0
0		0	Ewa-RG-art	1		0
0		0	Ewa-RG-art	1		0
0	#N/A	0		0		0
0	#N/A	0		0		0

0	#N/A	0		0		0
0	#N/A	0	Ewa-RG-art	1		0
0	#N/A	0	Ewa-RG-art	1		0
0	#N/A	0		0		0
0	#N/A	0		0		0
0	#N/A	0	Ewa-RG-art	1		0
0	#N/A	0	Ewa-RG-art	1		0
0	#N/A	0	Ewa-RG-art	1		0
0	#N/A	0	Ewa-RG-art	1		0
0	#N/A	0		0		0
0	#N/A	0		0		0
0	#N/A	0	Ewa-RG-art	1		0
0	#N/A	0	Ewa-RG-art	1		0
0		0	Ewa-RG-art	1		0
0	#N/A	0	Ewa-RG-art	1		0
0	#N/A	0	Ewa-RG-art	1		0
0	#N/A	0		0		0
0	#N/A	0	Ewa-RG-art	1		0
0	#N/A	0	Ewa-RG-art	1		0
0	#N/A	0		0		0
0	#N/A	0	Ewa-RG-art	1	Ewa-RG-top-a	1
0	#N/A	0		0		0
0	#N/A	0	Ewa-RG-art	1		0
0	#N/A	0	Ewa-RG-art	1		0
1	Ew/a-p-LOST-	1	Ewa-RG-art	1		0
1	Ew/a-p-LOST-	1	Ewa-RG-art	1		0
0	#N/A	0	Ewa-RG-art	1		0
0	#N/A	0		0		0
0	#N/A	0		0		0
0	#N/A	0	Ewa-RG-art	1		0
0	#N/A	0		0		0
0	#N/A	0	Ewa-RG-art	1		0
0	#N/A	0	Ewa-RG-art	1		0
0	#N/A	0	Ewa-RG-art	1		0
0	#N/A	0		0		0
0	#N/A	0		0		0
0	#N/A	0		0		0
0	#N/A	0		0		0
0	#N/A	0	Ewa-RG-art	1		0
0	#N/A	0		0		0
0	#N/A	0	Ewa-RG-art	1		0
0	#N/A	0	Ewa-RG-art	1		0
0	#N/A	0	Ewa-RG-art	1		0
0		0		0		0
0		0	Ewa-RG-art	1		0
0	#N/A	0	Ewa-RG-art	1		0
0	#N/A	0	Ewa-RG-art	1		0

0	#N/A	0		0		0
0	#N/A	0		0		0
0		0	Ewa-RG-art	1		0
0		0	Ewa-RG-art	1		0
0	#N/A	0	Ewa-RG-art	1		0
0	#N/A	0	Ewa-RG-art	1		0
0	#N/A	0	Ewa-RG-art	1		0
1	Ew/a-p-LOST-	1	Ewa-RG-art	1		0
1	Ew/a-p-LOST-	1	Ewa-RG-art	1		0
0	#N/A	0	Ewa-RG-art	1		0
0	#N/A	0	Ewa-RG-art	1		0
0	#N/A	0	Ewa-RG-art	1		0
0	#N/A	0	Ewa-RG-art	1		0
0	#N/A	0		0		0
0	#N/A	0		0		0
0	#N/A	0	Ewa-RG-art	1		0
0	#N/A	0	Ewa-RG-art	1		0
0	#N/A	0	Ewa-RG-art	1		0
0	#N/A	0	Ewa-RG-art	1		0
0	#N/A	0		0		0
0	#N/A	0		0		0
0	#N/A	0	Ewa-RG-art	1		0
0	Ew/a-p-LOST-	1		0		0
0	Ew/a-p-LOST-	1		0		0
0	#N/A	0		0		0
0	#N/A	0		0		0
0	#N/A	0	Ewa-RG-art	1		0
0	#N/A	0	Ewa-RG-art	1		0
0	#N/A	0	Ewa-RG-art	1	Ewa-RG-top-a	1
0	#N/A	0	Ewa-RG-art	1	Ewa-RG-top-a	1
0	#N/A	0		0		0
0	#N/A	0	Ewa-RG-art	1		0
0	#N/A	0	Ewa-RG-art	1		0
0	#N/A	0		0		0
0	#N/A	0	Ewa-RG-art	1	Ewa-RG-top-a	1
0	#N/A	0	Ewa-RG-art	1		0
0	#N/A	0	Ewa-RG-art	1		0
0		0	Ewa-RG-art	1	Ewa-RG-top-a	1
0	#N/A	0		0		0
0	#N/A	0	Ewa-RG-art	1		0
0	#N/A	0	Ewa-RG-art	1		0
0		0	Ewa-RG-art	1		0
0	#N/A	0	Ewa-RG-art	1		0
0	#N/A	0	Ewa-RG-art	1		0
0	#N/A	0	Ewa-RG-art	1		0
0	#N/A	0	Ewa-RG-art	1	Ewa-RG-top-a	1
0	#N/A	0	Ewa-RG-art	1	Ewa-RG-top-a	1

0		0		0		0
0	#N/A	0		0		0
0	#N/A	0		0		0
0	#N/A	0	Ewa-RG-art	1	Ewa-RG-top-a	1
0	#N/A	0	Ewa-RG-art	1	Ewa-RG-top-a	1
0		0		0		0
0		0		0		0
0	#N/A	0	Ewa-RG-art	1		0
0	#N/A	0	Ewa-RG-art	1	Ewa-RG-top-a	1
0	#N/A	0	Ewa-RG-art	1		0
0	#N/A	0	Ewa-RG-art	1		0
0		0	Ewa-RG-art	1		0
0		0	Ewa-RG-art	1		0
0	#N/A	0		0		0
0		0	Ewa-RG-art	1		0
0	Ew/a-p-LOST-	1	Ewa-RG-art	1		0
0	Ew/a-p-LOST-	1	Ewa-RG-art	1		0
0	Ew/a-p-LOST-	1	Ewa-RG-art	1		0
0	Ew/a-p-LOST-	1	Ewa-RG-art	1		0
0	Ew/a-p-LOST-	1	Ewa-RG-art	1		0
0	Ew/a-p-LOST-	1	Ewa-RG-art	1		0
0	#N/A	0	Ewa-RG-art	1		0
0	#N/A	0	Ewa-RG-art	1		0
0	#N/A	0	Ewa-RG-art	1		0
0	#N/A	0		0		0
0	#N/A	0		0		0
0	#N/A	0		0		0
0	#N/A	0		0		0
0	#N/A	0		0		0
1	Ew/a-p-LOST-	1	Ewa-RG-art	1		0
1	Ew/a-p-LOST-	1	Ewa-RG-art	1		0
0	#N/A	0		0		0
0	#N/A	0	Ewa-RG-art	1		0
0	#N/A	0	Ewa-RG-art	1		0
0	#N/A	0	Ewa-RG-art	1		0
0	#N/A	0	Ewa-RG-art	1		0
0	#N/A	0		0		0
0	#N/A	0		0		0
0	#N/A	0	Ewa-RG-art	1		0
0	#N/A	0		0		0
0	#N/A	0		0		0
0	#N/A	0	Ewa-RG-art	1		0
0	#N/A	0	Ewa-RG-art	1		0
1	Ew/a-p-LOST-	1	Ewa-RG-art	1		0
0	#N/A	0	Ewa-RG-art	1	Ewa-RG-top-a	1
0	#N/A	0	Ewa-RG-art	1		0
0	#N/A	0	Ewa-RG-art	1		0

0		0	Ewa-RG-art	1		0
0		0	Ewa-RG-art	1		0
0	#N/A	0	Ewa-RG-art	1		0
0	#N/A	0	Ewa-RG-art	1		0
0	#N/A	0	Ewa-RG-art	1		0
0	#N/A	0		0		0
0	#N/A	0	Ewa-RG-art	1		0
0	#N/A	0		0		0
0	#N/A	0		0		0
0	#N/A	0	Ewa-RG-art	1		0
0	#N/A	0	Ewa-RG-art	1		0
0	#N/A	0	Ewa-RG-art	1		0
0	#N/A	0	Ewa-RG-art	1		0
0	#N/A	0	Ewa-RG-art	1		0
0	#N/A	0	Ewa-RG-art	1		0
0	#N/A	0	Ewa-RG-art	1		0
0	#N/A	0	Ewa-RG-art	1		0
0	#N/A	0	Ewa-RG-art	1		0
0	#N/A	0	Ewa-RG-art	1		0
0	#N/A	0	Ewa-RG-art	1		0
0	#N/A	0	Ewa-RG-art	1		0
0	#N/A	0	Ewa-RG-art	1		0
0	#N/A	0	Ewa-RG-art	1		0
0	Ew/a-p-LOST-	1	Ewa-RG-art	1		0
0	Ew/a-p-LOST-	1	Ewa-RG-art	1		0
0	Ew/a-p-LOST-	1	Ewa-RG-art	1		0
0	#N/A	0	Ewa-RG-art	1		0
0	#N/A	0	Ewa-RG-art	1		0
0	#N/A	0	Ewa-RG-art	1		0
0	#N/A	0	Ewa-RG-art	1		0
0	#N/A	0	Ewa-RG-art	1		0
0	#N/A	0	Ewa-RG-art	1		0
0	#N/A	0		0		0
0	#N/A	0		0		0
0	#N/A	0	Ewa-RG-art	1	Ewa-RG-top-a	1
0	#N/A	0		0		0
1	Ew/a-p-LOST-	1	Ewa-RG-art	1		0
1	Ew/a-p-LOST-	1	Ewa-RG-art	1		0
0	#N/A	0		0		0
0	#N/A	0		0		0
0	#N/A	0	Ewa-RG-art	1		0
0	#N/A	0	Ewa-RG-art	1	Ewa-RG-top-a	1
0		0	Ewa-RG-art	1		0
0	#N/A	0	Ewa-RG-art	1		0
0	#N/A	0		0		0
0	#N/A	0	Ewa-RG-art	1		0
0	#N/A	0	Ewa-RG-art	1		0

0	#N/A	0		0		0
0	#N/A	0		0		0
0		0		0		0
0	#N/A	0	Ewa-RG-art	1		0
0	#N/A	0	Ewa-RG-art	1		0
0	#N/A	0		0		0
0	#N/A	0		0		0
0	#N/A	0	Ewa-RG-art	1		0
0	#N/A	0	Ewa-RG-art	1		0
0	#N/A	0	Ewa-RG-art	1		0
1	Ew/a-p-LOST-	1	Ewa-RG-art	1		0
1	Ew/a-p-LOST-	1	Ewa-RG-art	1		0
1	Ew/a-p-LOST-	1	Ewa-RG-art	1		0
0	#N/A	0		0		0
0	#N/A	0	Ewa-RG-art	1		0
0	#N/A	0	Ewa-RG-art	1		0
0	#N/A	0	Ewa-RG-art	1		0
0	#N/A	0	Ewa-RG-art	1		0
0	#N/A	0	Ewa-RG-art	1		0
0	#N/A	0		0		0
0	#N/A	0	Ewa-RG-art	1		0
0	#N/A	0	Ewa-RG-art	1		0
0	#N/A	0	Ewa-RG-art	1		0
0	#N/A	0	Ewa-RG-art	1		0
0	#N/A	0	Ewa-RG-art	1		0
0	#N/A	0	Ewa-RG-art	1		0
0	#N/A	0	Ewa-RG-art	1		0
0	#N/A	0	Ewa-RG-art	1		0
0	#N/A	0	Ewa-RG-art	1		0
0	#N/A	0		0		0
0	#N/A	0	Ewa-RG-art	1		0
0	#N/A	0	Ewa-RG-art	1		0
0	#N/A	0	Ewa-RG-art	1		0
0	#N/A	0	Ewa-RG-art	1		0
0	#N/A	0	Ewa-RG-art	1		0
0	#N/A	0	Ewa-RG-art	1		0
0	#N/A	0	Ewa-RG-art	1	Ewa-RG-top-a	1
0	#N/A	0	Ewa-RG-art	1		0
0	#N/A	0	Ewa-RG-art	1		0
0		0	Ewa-RG-art	1		0
0	#N/A	0	Ewa-RG-art	1		0
0	#N/A	0	Ewa-RG-art	1		0
0	#N/A	0		0		0
0	#N/A	0	Ewa-RG-art	1		0
0	#N/A	0	Ewa-RG-art	1		0
0	#N/A	0	Ewa-RG-art	1		0
0	#N/A	0	Ewa-RG-art	1		0

0	#N/A	0	Ewa-RG-art	1	0
0	#N/A	0	Ewa-RG-art	1	0
0	#N/A	0	Ewa-RG-art	1	0
0	#N/A	0	Ewa-RG-art	1	0
0	#N/A	0	Ewa-RG-art	1	0
0	#N/A	0	Ewa-RG-art	1	0
0	#N/A	0	Ewa-RG-art	1	0
0	#N/A	0	Ewa-RG-art	1	0
0	#N/A	0	Ewa-RG-art	1	0
0	#N/A	0	Ewa-RG-art	1	0
0	#N/A	0	Ewa-RG-art	1	0
0	#N/A	0	Ewa-RG-art	1	0
0	#N/A	0	Ewa-RG-art	1	0
0	#N/A	0	Ewa-RG-art	1	0
0	#N/A	0	Ewa-RG-art	1	0
0	#N/A	0	Ewa-RG-art	1	0
0	#N/A	0	Ewa-RG-art	1	0
0	#N/A	0	Ewa-RG-art	1	0
0	#N/A	0	Ewa-RG-art	1	0
0	#N/A	0	Ewa-RG-art	1	0
0	#N/A	0	Ewa-RG-art	1	0
0	#N/A	0	Ewa-RG-art	1	0
0	#N/A	0	Ewa-RG-art	1	0
0	#N/A	0	Ewa-RG-art	1	0
0	#N/A	0		0	0
0	#N/A	0	Ewa-RG-art	1	0
0	#N/A	0		0	0
0	#N/A	0	Ewa-RG-art	1	0
0	#N/A	0	Ewa-RG-art	1	0
0	#N/A	0	Ewa-RG-art	1	0
0	#N/A	0	Ewa-RG-art	1	0
0	#N/A	0	Ewa-RG-art	1	0
0	#N/A	0	Ewa-RG-art	1	0
0	#N/A	0	Ewa-RG-art	1	0
0	#N/A	0	Ewa-RG-art	1	0
0	#N/A	0	Ewa-RG-art	1	0
0	#N/A	0	Ewa-RG-art	1	0
0	#N/A	0	Ewa-RG-art	1	0
0	#N/A	0	Ewa-RG-art	1	0
0	#N/A	0		0	0
0	#N/A	0		0	0
0	#N/A	0	Ewa-RG-art	1	0
0		0	Ewa-RG-art	1	0
0		0	Ewa-RG-art	1	0
0	#N/A	0		0	0
1	Ew/a-p-LOST-	1	Ewa-RG-art	1	0
0	#N/A	0		0	0

0	#N/A	0	Ewa-RG-art	1		0
0	#N/A	0		0		0
0	#N/A	0	Ewa-RG-art	1	Ewa-RG-top-a	1
0	#N/A	0	Ewa-RG-art	1	Ewa-RG-top-a	1
0	#N/A	0	Ewa-RG-art	1		0
0	#N/A	0	Ewa-RG-art	1		0
1	Ew/a-p-LOST-	1	Ewa-RG-art	1		0
1	Ew/a-p-LOST-	1	Ewa-RG-art	1		0
1	Ew/a-p-LOST-	1	Ewa-RG-art	1		0
1	Ew/a-p-LOST-	1	Ewa-RG-art	1		0
0	#N/A	0		0		0
0	#N/A	0		0		0
0	#N/A	0	Ewa-RG-art	1		0
0	#N/A	0	Ewa-RG-art	1		0
0	#N/A	0		0		0
0	#N/A	0	Ewa-RG-art	1		0
0	#N/A	0	Ewa-RG-art	1		0
1	Ew/a-p-LOST-	1		0		0
1	Ew/a-p-LOST-	1		0		0
0		0	Ewa-RG-art	1		0
0		0	Ewa-RG-art	1		0
0	#N/A	0	Ewa-RG-art	1	Ewa-RG-top-a	1
0	#N/A	0	Ewa-RG-art	1	Ewa-RG-top-a	1
1	Ew/a-p-LOST-	1	Ewa-RG-art	1		0
0	#N/A	0	Ewa-RG-art	1		0
0	#N/A	0	Ewa-RG-art	1		0
0	#N/A	0		0		0
0	#N/A	0		0		0
0	#N/A	0	Ewa-RG-art	1		0
0	#N/A	0		0		0
0	#N/A	0	Ewa-RG-art	1		0
1	Ew/a-p-LOST-	1	Ewa-RG-art	1		0
1	Ew/a-p-LOST-	1	Ewa-RG-art	1		0
0	#N/A	0		0		0
0	#N/A	0		0		0
0	#N/A	0	Ewa-RG-art	1		0
1	Ew/a-p-LOST-	1	Ewa-RG-art	1		0
1	Ew/a-p-LOST-	1	Ewa-RG-art	1		0
0	#N/A	0	Ewa-RG-art	1		0
0	#N/A	0	Ewa-RG-art	1		0
0	#N/A	0	Ewa-RG-art	1		0
0	#N/A	0		0		0
0	#N/A	0	Ewa-RG-art	1		0
0	#N/A	0		0		0
0	#N/A	0		0		0
0	#N/A	0	Ewa-RG-art	1		0
0	#N/A	0		0		0

0	#N/A	0		0		0
0	#N/A	0	Ewa-RG-art	1		0
0		0	Ewa-RG-art	1	Ewa-RG-top-a	1
0		0	Ewa-RG-art	1	Ewa-RG-top-a	1
0	#N/A	0	Ewa-RG-art	1		0
0	#N/A	0		0		0
0	#N/A	0	Ewa-RG-art	1		0
0	#N/A	0	Ewa-RG-art	1		0
0	#N/A	0	Ewa-RG-art	1		0
0	#N/A	0	Ewa-RG-art	1		0
0	#N/A	0	Ewa-RG-art	1		0
0	#N/A	0	Ewa-RG-art	1		0
0	#N/A	0	Ewa-RG-art	1		0
0	#N/A	0	Ewa-RG-art	1		0
0	#N/A	0	Ewa-RG-art	1	Ewa-RG-top-a	1
1	Ew/a-p-LOST-	1		0		0
0		0	Ewa-RG-art	1	Ewa-RG-top-a	1
0		0	Ewa-RG-art	1	Ewa-RG-top-a	1
0		0	Ewa-RG-art	1	Ewa-RG-top-a	1
0	Ew/a-p-LOST-	1	Ewa-RG-art	1		0
0		0	Ewa-RG-art	1		0
0		0	Ewa-RG-art	1		0
0	#N/A	0		0		0
0	#N/A	0		0		0
0	#N/A	0	Ewa-RG-art	1		0
0	#N/A	0	Ewa-RG-art	1		0
0	#N/A	0	Ewa-RG-art	1		0
0	#N/A	0	Ewa-RG-art	1		0
0	#N/A	0	Ewa-RG-art	1		0
0	#N/A	0	Ewa-RG-art	1		0
0		0	Ewa-RG-art	1	Ewa-RG-top-a	1
0		0	Ewa-RG-art	1	Ewa-RG-top-a	1
0	#N/A	0		0		0
1	Ew/a-p-LOST-	1	Ewa-RG-art	1		0
1	Ew/a-p-LOST-	1	Ewa-RG-art	1		0
1	Ew/a-p-LOST-	1	Ewa-RG-art	1		0
1	Ew/a-p-LOST-	1	Ewa-RG-art	1		0
0	#N/A	0	Ewa-RG-art	1		0
0	#N/A	0	Ewa-RG-art	1		0
0	Ew/a-p-LOST-	1	Ewa-RG-art	1		0
0	#N/A	0		0		0
0		0		0		0
0		0		0		0
0		0		0		0
0	#N/A	0	Ewa-RG-art	1		0
0	#N/A	0	Ewa-RG-art	1		0
0	#N/A	0		0		0

0	#N/A	0	Ewa-RG-art	1		0
0	#N/A	0	Ewa-RG-art	1		0
0	#N/A	0	Ewa-RG-art	1		0
0	#N/A	0	Ewa-RG-art	1		0
0	#N/A	0	Ewa-RG-art	1		0
0	#N/A	0	Ewa-RG-art	1		0
0	#N/A	0	Ewa-RG-art	1		0
0	#N/A	0	Ewa-RG-art	1		0
1	Ew/a-p-LOST-	1	Ewa-RG-art	1	Ewa-RG-top-a	1
0	#N/A	0		0		0
0	#N/A	0	Ewa-RG-art	1		0
0	#N/A	0	Ewa-RG-art	1		0
0	#N/A	0	Ewa-RG-art	1		0
0	#N/A	0		0		0
0	#N/A	0		0		0
0	#N/A	0		0		0
0	#N/A	0	Ewa-RG-art	1		0
0	#N/A	0	Ewa-RG-art	1		0
0	#N/A	0	Ewa-RG-art	1		0
0	#N/A	0	Ewa-RG-art	1		0
0	#N/A	0	Ewa-RG-art	1		0
1	Ew/a-p-LOST-	1	Ewa-RG-art	1		0
1	Ew/a-p-LOST-	1	Ewa-RG-art	1		0
0		0		0		0
0	#N/A	0	Ewa-RG-art	1		0
0	#N/A	0	Ewa-RG-art	1		0
0	#N/A	0	Ewa-RG-art	1		0
0	#N/A	0	Ewa-RG-art	1		0
0	#N/A	0	Ewa-RG-art	1	Ewa-RG-top-a	1
0		0	Ewa-RG-art	1	Ewa-RG-top-a	1
0		0	Ewa-RG-art	1	Ewa-RG-top-a	1
0	#N/A	0		0		0
0	#N/A	0		0		0
1	Ew/a-p-LOST-	1	Ewa-RG-art	1		0
1	Ew/a-p-LOST-	1	Ewa-RG-art	1		0
0	#N/A	0		0		0
0	#N/A	0		0		0
0		0	Ewa-RG-art	1		0
0	Ew/a-p-LOST-	1	Ewa-RG-art	1		0
0	Ew/a-p-LOST-	1	Ewa-RG-art	1		0
0	#N/A	0	Ewa-RG-art	1		0
1	Ew/a-p-LOST-	1	Ewa-RG-art	1		0
0	#N/A	0	Ewa-RG-art	1		0
0	#N/A	0	Ewa-RG-art	1		0
0	#N/A	0		0		0
0	#N/A	0	Ewa-RG-art	1		0
0	#N/A	0		0		0

0		0	Ewa-RG-art	1	Ewa-RG-top-a	1
0	#N/A	0	Ewa-RG-art	1	Ewa-RG-top-a	1
0	#N/A	0	Ewa-RG-art	1	Ewa-RG-top-a	1
0	#N/A	0	Ewa-RG-art	1		0
0	#N/A	0	Ewa-RG-art	1		0
0	#N/A	0	Ewa-RG-art	1		0
0	#N/A	0	Ewa-RG-art	1		0
0		0	Ewa-RG-art	1	Ewa-RG-top-a	1
0		0	Ewa-RG-art	1	Ewa-RG-top-a	1
1	Ew/a-p-LOST-	1		0		0
1	Ew/a-p-LOST-	1		0		0
0	#N/A	0	Ewa-RG-art	1		0
0	#N/A	0	Ewa-RG-art	1		0
0		0		0		0
0		0		0		0
1	Ew/a-p-LOST-	1	Ewa-RG-art	1		0
1	Ew/a-p-LOST-	1	Ewa-RG-art	1		0
0	#N/A	0	Ewa-RG-art	1		0
0	#N/A	0	Ewa-RG-art	1		0
0	#N/A	0	Ewa-RG-art	1		0
0	#N/A	0	Ewa-RG-art	1		0
0	#N/A	0	Ewa-RG-art	1		0
0	#N/A	0		0		0
0	#N/A	0		0		0
0		0	Ewa-RG-art	1		0
0	#N/A	0	Ewa-RG-art	1		0
0	#N/A	0	Ewa-RG-art	1		0
0	#N/A	0	Ewa-RG-art	1		0
0	#N/A	0	Ewa-RG-art	1		0
0	#N/A	0	Ewa-RG-art	1		0
0	#N/A	0	Ewa-RG-art	1		0
0	#N/A	0	Ewa-RG-art	1		0
0	#N/A	0	Ewa-RG-art	1		0
0	#N/A	0	Ewa-RG-art	1		0
0	#N/A	0	Ewa-RG-art	1		0
0	#N/A	0	Ewa-RG-art	1		0
0	#N/A	0	Ewa-RG-art	1		0
0		0		0		0
0	#N/A	0	Ewa-RG-art	1		0
0		0		0		0
0		0		0		0
0	#N/A	0	Ewa-RG-art	1		0
0	#N/A	0	Ewa-RG-art	1		0
0	#N/A	0	Ewa-RG-art	1		0
0	#N/A	0		0		0
0	#N/A	0		0		0
0	#N/A	0		0		0

0	#N/A	0		0		0
0	#N/A	0	Ewa-RG-art	1		0
0	#N/A	0	Ewa-RG-art	1		0
0	#N/A	0	Ewa-RG-art	1		0
0	#N/A	0	Ewa-RG-art	1		0
0	Ew/a-p-LOST-	1	Ewa-RG-art	1		0
1	Ew/a-p-LOST-	1	Ewa-RG-art	1		0
1	Ew/a-p-LOST-	1	Ewa-RG-art	1		0
0	#N/A	0	Ewa-RG-art	1		0
0	#N/A	0		0		0
0	#N/A	0	Ewa-RG-art	1		0
0	#N/A	0	Ewa-RG-art	1		0
0	#N/A	0		0		0
0	#N/A	0		0		0
0	#N/A	0	Ewa-RG-art	1		0
0	#N/A	0		0		0
0	#N/A	0		0		0
0	#N/A	0		0		0
0	#N/A	0		0		0
1	Ew/a-p-LOST-	1	Ewa-RG-art	1		0
1	Ew/a-p-LOST-	1	Ewa-RG-art	1		0
1	Ew/a-p-LOST-	1	Ewa-RG-art	1		0
1	Ew/a-p-LOST-	1	Ewa-RG-art	1		0
1	Ew/a-p-LOST-	1	Ewa-RG-art	1		0
0	#N/A	0		0		0
0	#N/A	0		0		0
1	Ew/a-p-LOST-	1	Ewa-RG-art	1		0
1	Ew/a-p-LOST-	1	Ewa-RG-art	1		0
1	Ew/a-p-LOST-	1	Ewa-RG-art	1		0
0	#N/A	0	Ewa-RG-art	1		0
0	#N/A	0		0		0
0		0	Ewa-RG-art	1	Ewa-RG-top-a	1
0	#N/A	0	Ewa-RG-art	1		0
0		0	Ewa-RG-art	1		0
0	#N/A	0	Ewa-RG-art	1		0
0		0	Ewa-RG-art	1		0
0	Ew/a-p-LOST-	1	Ewa-RG-art	1		0
0	Ew/a-p-LOST-	1	Ewa-RG-art	1		0
0	#N/A	0	Ewa-RG-art	1		0
0	#N/A	0	Ewa-RG-art	1		0
0	#N/A	0	Ewa-RG-art	1		0
0	#N/A	0	Ewa-RG-art	1		0
0	#N/A	0	Ewa-RG-art	1		0
0	#N/A	0	Ewa-RG-art	1		0
0	#N/A	0	Ewa-RG-art	1		0
0	#N/A	0	Ewa-RG-art	1		0
0	#N/A	0	Ewa-RG-art	1		0

0	#N/A	0	Ewa-RG-art	1		0
0	#N/A	0	Ewa-RG-art	1		0
0	#N/A	0	Ewa-RG-art	1		0
1	Ew/a-p-LOST-	1	Ewa-RG-art	1		0
0	#N/A	0	Ewa-RG-art	1		0
0	#N/A	0	Ewa-RG-art	1		0
0	#N/A	0		0		0
0	#N/A	0		0		0
0	#N/A	0		0		0
0	#N/A	0		0		0
0		0	Ewa-RG-art	1		0
0	#N/A	0	Ewa-RG-art	1	Ewa-RG-top-a	1
0	#N/A	0	Ewa-RG-art	1	Ewa-RG-top-a	1
0	#N/A	0	Ewa-RG-art	1		0
0	#N/A	0	Ewa-RG-art	1		0
0	#N/A	0		0		0
0	#N/A	0	Ewa-RG-art	1		0
0	#N/A	0	Ewa-RG-art	1		0
0	#N/A	0	Ewa-RG-art	1		0
0	#N/A	0		0		0
0	#N/A	0	Ewa-RG-art	1		0
0	#N/A	0	Ewa-RG-art	1		0
0	#N/A	0	Ewa-RG-art	1		0
0	#N/A	0	Ewa-RG-art	1		0
0		0		0		0
0	#N/A	0	Ewa-RG-art	1		0
0	#N/A	0	Ewa-RG-art	1		0
0	#N/A	0	Ewa-RG-art	1		0
0	#N/A	0	Ewa-RG-art	1		0
0	#N/A	0	Ewa-RG-art	1	Ewa-RG-top-a	1
0	#N/A	0	Ewa-RG-art	1	Ewa-RG-top-a	1
0	#N/A	0	Ewa-RG-art	1		0
0	#N/A	0	Ewa-RG-art	1		0
0	#N/A	0	Ewa-RG-art	1		0
1	Ew/a-p-LOST-	1	Ewa-RG-art	1		0
0	#N/A	0		0		0
0	#N/A	0	Ewa-RG-art	1		0
0	#N/A	0		0		0
0	#N/A	0	Ewa-RG-art	1		0
0	#N/A	0	Ewa-RG-art	1		0
0	#N/A	0	Ewa-RG-art	1		0
0	#N/A	0	Ewa-RG-art	1		0
0	#N/A	0	Ewa-RG-art	1		0
0		0	Ewa-RG-art	1		0
0		0	Ewa-RG-art	1		0
0	#N/A	0		0		0
0	#N/A	0		0		0

0	#N/A	0		0		0
0	#N/A	0		0		0
0	#N/A	0	Ewa-RG-art	1		0
0	#N/A	0		0		0
0	#N/A	0		0		0
0	Ew/a-p-LOST-	1	Ewa-RG-art	1		0
0	#N/A	0		0		0
0	#N/A	0		0		0
0	#N/A	0		0		0
0	#N/A	0	Ewa-RG-art	1		0
0	#N/A	0		0		0
0	#N/A	0	Ewa-RG-art	1	Ewa-RG-top-a	1
0	#N/A	0	Ewa-RG-art	1	Ewa-RG-top-a	1
0	#N/A	0	Ewa-RG-art	1	Ewa-RG-top-a	1
0	#N/A	0		0		0
1	Ew/a-p-LOST-	1	Ewa-RG-art	1		0
0	#N/A	0	Ewa-RG-art	1		0
0	#N/A	0	Ewa-RG-art	1	Ewa-RG-top-a	1
0	#N/A	0	Ewa-RG-art	1		0
0	#N/A	0		0		0
0		0		0		0
0		0		0		0
0	#N/A	0	Ewa-RG-art	1		0
0	#N/A	0	Ewa-RG-art	1		0
0	#N/A	0	Ewa-RG-art	1	Ewa-RG-top-a	1
0	#N/A	0	Ewa-RG-art	1		0
0	#N/A	0	Ewa-RG-art	1		0
0	#N/A	0		0		0
0	#N/A	0	Ewa-RG-art	1		0
0	#N/A	0		0		0
0	#N/A	0		0		0
0	#N/A	0	Ewa-RG-art	1		0
0	#N/A	0	Ewa-RG-art	1		0
0	#N/A	0	Ewa-RG-art	1		0
0	#N/A	0		0		0
0	#N/A	0		0		0
0	Ew/a-p-LOST-	1	Ewa-RG-art	1		0
0	Ew/a-p-LOST-	1	Ewa-RG-art	1		0
1	Ew/a-p-LOST-	1	Ewa-RG-art	1		0
1	Ew/a-p-LOST-	1	Ewa-RG-art	1		0
0		0		0		0
0	#N/A	0	Ewa-RG-art	1		0
0	#N/A	0		0		0
0	#N/A	0		0		0
0	#N/A	0	Ewa-RG-art	1		0
0	#N/A	0	Ewa-RG-art	1		0
0		0	Ewa-RG-art	1		0

0		0	Ewa-RG-art	1		0
0	#N/A	0		0		0
0	#N/A	0		0		0
0	#N/A	0	Ewa-RG-art	1		0
0	#N/A	0	Ewa-RG-art	1		0
0	#N/A	0		0		0
0	#N/A	0	Ewa-RG-art	1		0
0	#N/A	0		0		0
0		0	Ewa-RG-art	1	Ewa-RG-top-a	1
0		0	Ewa-RG-art	1	Ewa-RG-top-a	1
0		0	Ewa-RG-art	1	Ewa-RG-top-a	1
0	#N/A	0	Ewa-RG-art	1		0
0	#N/A	0	Ewa-RG-art	1		0
0	#N/A	0	Ewa-RG-art	1	Ewa-RG-top-a	1
0	#N/A	0	Ewa-RG-art	1	Ewa-RG-top-a	1
0	#N/A	0	Ewa-RG-art	1		0
0	#N/A	0	Ewa-RG-art	1		0
0	#N/A	0		0		0
0	#N/A	0	Ewa-RG-art	1		0
0	#N/A	0	Ewa-RG-art	1		0
0	#N/A	0	Ewa-RG-art	1		0
0	Ew/a-p-LOST-	1	Ewa-RG-art	1		0
0	#N/A	0		0		0
0	#N/A	0		0		0
0	#N/A	0		0		0
0	#N/A	0	Ewa-RG-art	1		0
0	#N/A	0	Ewa-RG-art	1		0
0	#N/A	0	Ewa-RG-art	1		0
0	#N/A	0	Ewa-RG-art	1		0
0	#N/A	0	Ewa-RG-art	1		0
0	#N/A	0	Ewa-RG-art	1		0
0	#N/A	0	Ewa-RG-art	1		0
0	#N/A	0	Ewa-RG-art	1		0
0	#N/A	0		0		0
0	#N/A	0		0		0
0	#N/A	0	Ewa-RG-art	1		0
0	#N/A	0	Ewa-RG-art	1		0
0	#N/A	0		0		0
0	#N/A	0	Ewa-RG-art	1		0
0	#N/A	0		0		0
0	#N/A	0	Ewa-RG-art	1		0
0	#N/A	0	Ewa-RG-art	1		0
0	#N/A	0	Ewa-RG-art	1		0
0	Ew/a-p-LOST-	1	Ewa-RG-art	1		0
0	Ew/a-p-LOST-	1	Ewa-RG-art	1		0
0	Ew/a-p-LOST-	1	Ewa-RG-art	1		0
0	Ew/a-p-LOST-	1	Ewa-RG-art	1		0

0	Ew/a-p-LOST-	1	Ewa-RG-art	1		0
0	#N/A	0	Ewa-RG-art	1		0
0	#N/A	0	Ewa-RG-art	1		0
0	#N/A	0		0		0
0	#N/A	0	Ewa-RG-art	1	Ewa-RG-top-a	1
0	#N/A	0	Ewa-RG-art	1	Ewa-RG-top-a	1
0	#N/A	0	Ewa-RG-art	1	Ewa-RG-top-a	1
0	#N/A	0	Ewa-RG-art	1		0
0	#N/A	0	Ewa-RG-art	1		0
0		0	Ewa-RG-art	1		0
0	#N/A	0	Ewa-RG-art	1		0
1	Ew/a-p-LOST-	1	Ewa-RG-art	1		0
1	Ew/a-p-LOST-	1	Ewa-RG-art	1		0
1	Ew/a-p-LOST-	1	Ewa-RG-art	1		0
0	#N/A	0	Ewa-RG-art	1		0
0	#N/A	0	Ewa-RG-art	1		0
0	#N/A	0	Ewa-RG-art	1		0
0	#N/A	0	Ewa-RG-art	1		0
0	#N/A	0		0		0
0	#N/A	0	Ewa-RG-art	1		0
0	#N/A	0	Ewa-RG-art	1		0
0	#N/A	0	Ewa-RG-art	1		0
0	#N/A	0	Ewa-RG-art	1		0
0	#N/A	0	Ewa-RG-art	1		0
1	Ew/a-p-LOST-	1	Ewa-RG-art	1		0
1	Ew/a-p-LOST-	1	Ewa-RG-art	1		0
1	Ew/a-p-LOST-	1	Ewa-RG-art	1		0
1	Ew/a-p-LOST-	1	Ewa-RG-art	1		0
0	#N/A	0		0		0
0	#N/A	0	Ewa-RG-art	1		0
0	#N/A	0	Ewa-RG-art	1		0
0	#N/A	0	Ewa-RG-art	1	Ewa-RG-top-a	1
0	#N/A	0	Ewa-RG-art	1	Ewa-RG-top-a	1
0	#N/A	0	Ewa-RG-art	1	Ewa-RG-top-a	1
0	#N/A	0	Ewa-RG-art	1		0
0	#N/A	0	Ewa-RG-art	1		0
0	#N/A	0	Ewa-RG-art	1		0
0	#N/A	0		0		0
0	#N/A	0	Ewa-RG-art	1		0
0	#N/A	0	Ewa-RG-art	1		0
0	#N/A	0	Ewa-RG-art	1		0
0	#N/A	0	Ewa-RG-art	1		0
0	#N/A	0	Ewa-RG-art	1		0
0	#N/A	0	Ewa-RG-art	1		0
0	#N/A	0		0		0
0	#N/A	0	Ewa-RG-art	1		0
0	#N/A	0	Ewa-RG-art	1		0

0		0		0		0
0	#N/A	0		0		0
0	#N/A	0		0		0
0	#N/A	0		0		0
0	Ew/a-p-LOST-	1	Ewa-RG-art	1		0
0	Ew/a-p-LOST-	1	Ewa-RG-art	1		0
0	Ew/a-p-LOST-	1	Ewa-RG-art	1		0
0	#N/A	0	Ewa-RG-art	1		0
0	#N/A	0	Ewa-RG-art	1		0
0		0	Ewa-RG-art	1	Ewa-RG-top-a	1
0	#N/A	0	Ewa-RG-art	1		0
0	#N/A	0	Ewa-RG-art	1		0
0	#N/A	0	Ewa-RG-art	1		0
0	#N/A	0	Ewa-RG-art	1		0
0	#N/A	0	Ewa-RG-art	1		0
0	#N/A	0	Ewa-RG-art	1		0
0	#N/A	0	Ewa-RG-art	1		0
0	#N/A	0		0		0
0	#N/A	0	Ewa-RG-art	1	Ewa-RG-top-a	1
0	#N/A	0	Ewa-RG-art	1	Ewa-RG-top-a	1
0	#N/A	0	Ewa-RG-art	1		0
0		0	Ewa-RG-art	1		0
0		0	Ewa-RG-art	1		0
0		0	Ewa-RG-art	1		0
0	#N/A	0		0		0
0	Ew/a-p-LOST-	1	Ewa-RG-art	1		0
0	#N/A	0		0		0
0	#N/A	0	Ewa-RG-art	1		0
0	#N/A	0	Ewa-RG-art	1		0
0	#N/A	0	Ewa-RG-art	1		0
0	#N/A	0	Ewa-RG-art	1		0
0	#N/A	0	Ewa-RG-art	1		0
0	#N/A	0	Ewa-RG-art	1		0
0	#N/A	0	Ewa-RG-art	1		0
0	#N/A	0	Ewa-RG-art	1		0
0	#N/A	0	Ewa-RG-art	1		0
0	#N/A	0		0		0
0	Ew/a-p-LOST-	1	Ewa-RG-art	1		0
0	Ew/a-p-LOST-	1	Ewa-RG-art	1		0
0	#N/A	0		0		0
0	#N/A	0		0		0
0	#N/A	0	Ewa-RG-art	1		0
0	#N/A	0	Ewa-RG-art	1		0
0	#N/A	0		0		0
0	#N/A	0	Ewa-RG-art	1		0
0	#N/A	0	Ewa-RG-art	1		0
0	#N/A	0	Ewa-RG-art	1	Ewa-RG-top-a	1

0	#N/A	0		0		0
0	#N/A	0		0		0
0	#N/A	0		0		0
0	#N/A	0		0		0
0	#N/A	0		0		0
0	#N/A	0	Ewa-RG-art	1		0
0	#N/A	0	Ewa-RG-art	1		0
1	Ew/a-p-LOST-	1	Ewa-RG-art	1	Ewa-RG-top-a	1
1	Ew/a-p-LOST-	1	Ewa-RG-art	1	Ewa-RG-top-a	1
1	Ew/a-p-LOST-	1	Ewa-RG-art	1		0
1	Ew/a-p-LOST-	1	Ewa-RG-art	1		0
1	Ew/a-p-LOST-	1	Ewa-RG-art	1		0
0	#N/A	0		0		0
0	#N/A	0		0		0
0	#N/A	0		0		0
0		0		0		0
0	#N/A	0	Ewa-RG-art	1		0
0	#N/A	0	Ewa-RG-art	1		0
0	Ew/a-p-LOST-	1	Ewa-RG-art	1		0
0	Ew/a-p-LOST-	1	Ewa-RG-art	1		0
0	#N/A	0	Ewa-RG-art	1		0
0	#N/A	0	Ewa-RG-art	1		0
1	Ew/a-p-LOST-	1	Ewa-RG-art	1		0
1	Ew/a-p-LOST-	1	Ewa-RG-art	1		0
1	Ew/a-p-LOST-	1	Ewa-RG-art	1		0
0	#N/A	0	Ewa-RG-art	1		0
1	Ew/a-p-LOST-	1	Ewa-RG-art	1		0
0	#N/A	0		0		0
1	Ew/a-p-LOST-	1	Ewa-RG-art	1		0
1	Ew/a-p-LOST-	1	Ewa-RG-art	1		0
0	#N/A	0		0		0
0	Ew/a-p-LOST-	1	Ewa-RG-art	1		0
1	Ew/a-p-LOST-	1		0		0
1	Ew/a-p-LOST-	1		0		0
1	Ew/a-p-LOST-	1	Ewa-RG-art	1		0
0	#N/A	0	Ewa-RG-art	1		0
0	#N/A	0	Ewa-RG-art	1		0
0	#N/A	0	Ewa-RG-art	1		0
0	#N/A	0	Ewa-RG-art	1		0
0	#N/A	0	Ewa-RG-art	1		0
0	#N/A	0	Ewa-RG-art	1		0
0	#N/A	0		0		0
0	#N/A	0	Ewa-RG-art	1		0
0	#N/A	0		0		0
0		0		0		0
0		0	Ewa-RG-art	1		0
0	#N/A	0	Ewa-RG-art	1		0

0	#N/A	0	Ewa-RG-art	1		0
0	#N/A	0	Ewa-RG-art	1		0
0	#N/A	0	Ewa-RG-art	1		0
0		0		0		0
0	#N/A	0	Ewa-RG-art	1		0
1	Ew/a-p-LOST-	1	Ewa-RG-art	1		0
1	Ew/a-p-LOST-	1	Ewa-RG-art	1		0
0	#N/A	0	Ewa-RG-art	1		0
0	#N/A	0		0		0
0		0	Ewa-RG-art	1		0
0		0	Ewa-RG-art	1		0
0		0	Ewa-RG-art	1		0
0	#N/A	0	Ewa-RG-art	1		0
0	#N/A	0	Ewa-RG-art	1		0
0	#N/A	0	Ewa-RG-art	1		0
0	#N/A	0	Ewa-RG-art	1		0
0	#N/A	0		0		0
0	#N/A	0		0		0
0	#N/A	0	Ewa-RG-art	1		0
0	#N/A	0	Ewa-RG-art	1		0
0	#N/A	0		0		0
0	#N/A	0	Ewa-RG-art	1		0
0	#N/A	0	Ewa-RG-art	1		0
0	#N/A	0	Ewa-RG-art	1		0
0	#N/A	0	Ewa-RG-art	1		0
0	#N/A	0	Ewa-RG-art	1		0
0	#N/A	0		0		0
0	#N/A	0		0		0
1	Ew/a-p-LOST-	1		0		0
1	Ew/a-p-LOST-	1		0		0
0	#N/A	0	Ewa-RG-art	1		0
0	Ew/a-p-LOST-	1	Ewa-RG-art	1		0
0	#N/A	0	Ewa-RG-art	1		0
0	#N/A	0	Ewa-RG-art	1		0
0	#N/A	0		0		0
0	#N/A	0		0		0
0	#N/A	0		0		0
0	#N/A	0		0		0
0	#N/A	0		0		0
0	Ew/a-p-LOST-	1		0		0
0	Ew/a-p-LOST-	1		0		0
0	#N/A	0	Ewa-RG-art	1		0
0	#N/A	0	Ewa-RG-art	1		0
0	#N/A	0	Ewa-RG-art	1		0
0	#N/A	0	Ewa-RG-art	1		0
0	#N/A	0		0		0
0	#N/A	0	Ewa-RG-art	1	Ewa-RG-top-a	1

0	#N/A	0	Ewa-RG-art	1	Ewa-RG-top-a	1
0		0		0		0
1	Ew/a-p-LOST-	1	Ewa-RG-art	1	Ewa-RG-top-a	1
1	Ew/a-p-LOST-	1	Ewa-RG-art	1	Ewa-RG-top-a	1
0	#N/A	0		0		0
0	#N/A	0		0		0
0	#N/A	0	Ewa-RG-art	1		0
0	#N/A	0	Ewa-RG-art	1		0
0	#N/A	0	Ewa-RG-art	1		0
0	#N/A	0	Ewa-RG-art	1		0
0	#N/A	0		0		0
0	#N/A	0		0		0
0	#N/A	0	Ewa-RG-art	1		0
1	Ew/a-p-LOST-	1	Ewa-RG-art	1		0
1	Ew/a-p-LOST-	1	Ewa-RG-art	1		0
0	#N/A	0	Ewa-RG-art	1		0
1	Ew/a-p-LOST-	1	Ewa-RG-art	1		0
1	Ew/a-p-LOST-	1	Ewa-RG-art	1		0
0	#N/A	0	Ewa-RG-art	1		0
0		0	Ewa-RG-art	1		0
0	#N/A	0		0		0
0	#N/A	0		0		0
0	#N/A	0	Ewa-RG-art	1		0
0	#N/A	0	Ewa-RG-art	1		0
0	#N/A	0	Ewa-RG-art	1		0
0	#N/A	0	Ewa-RG-art	1		0
0	#N/A	0		0		0
0	#N/A	0	Ewa-RG-art	1		0
0	#N/A	0	Ewa-RG-art	1		0
0	#N/A	0	Ewa-RG-art	1		0
0		0	Ewa-RG-art	1		0
0		0	Ewa-RG-art	1		0
1	Ew/a-p-LOST-	1		0		0
0	#N/A	0	Ewa-RG-art	1		0
0		0	Ewa-RG-art	1	Ewa-RG-top-a	1
0		0	Ewa-RG-art	1		0
0		0	Ewa-RG-art	1		0
1	Ew/a-p-LOST-	1		0		0
0	#N/A	0		0		0
0	#N/A	0	Ewa-RG-art	1		0
0	Ew/a-p-LOST-	1	Ewa-RG-art	1		0
0	Ew/a-p-LOST-	1	Ewa-RG-art	1		0
0	Ew/a-p-LOST-	1	Ewa-RG-art	1		0
0	Ew/a-p-LOST-	1	Ewa-RG-art	1		0
0		0	Ewa-RG-art	1		0
1	Ew/a-p-LOST-	1		0		0
1	Ew/a-p-LOST-	1		0		0

0	#N/A	0	Ewa-RG-art	1	0
0		0		0	0
0		0	Ewa-RG-art	1	0
0	#N/A	0	Ewa-RG-art	1	0
0	#N/A	0	Ewa-RG-art	1	0
0	#N/A	0	Ewa-RG-art	1	0
0	#N/A	0	Ewa-RG-art	1	0
0	#N/A	0	Ewa-RG-art	1	0
0	#N/A	0	Ewa-RG-art	1	0
0	#N/A	0		0	0
0	#N/A	0	Ewa-RG-art	1	0
0	#N/A	0	Ewa-RG-art	1	0
1	Ew/a-p-LOST-	1		0	0
0	#N/A	0	Ewa-RG-art	1	0
0	#N/A	0	Ewa-RG-art	1	0
0	#N/A	0	Ewa-RG-art	1	0
0	#N/A	0	Ewa-RG-art	1	0
0	Ew/a-p-LOST-	1	Ewa-RG-art	1	0
0	Ew/a-p-LOST-	1	Ewa-RG-art	1	0
0	Ew/a-p-LOST-	1	Ewa-RG-art	1	0
0	#N/A	0		0	0
0	#N/A	0	Ewa-RG-art	1	0
0	#N/A	0		0	0
0	#N/A	0	Ewa-RG-art	1	0
0		0	Ewa-RG-art	1	0
0	#N/A	0		0	0
0		0	Ewa-RG-art	1	0
0	#N/A	0	Ewa-RG-art	1	0
0	#N/A	0	Ewa-RG-art	1	0
0	#N/A	0	Ewa-RG-art	1	0
0	#N/A	0	Ewa-RG-art	1	0
0	#N/A	0	Ewa-RG-art	1	0
0	#N/A	0	Ewa-RG-art	1	0
0	#N/A	0	Ewa-RG-art	1	0
0	#N/A	0	Ewa-RG-art	1	0
1	Ew/a-p-LOST-	1	Ewa-RG-art	1	0
0	#N/A	0		0	0
0	#N/A	0		0	0
0	#N/A	0		0	0
0		0		0	0
0	#N/A	0		0	0
0		0	Ewa-RG-art	1	0
0	#N/A	0	Ewa-RG-art	1	0
0	#N/A	0	Ewa-RG-art	1	0
0	#N/A	0	Ewa-RG-art	1	0
0	#N/A	0	Ewa-RG-art	1	0
0	#N/A	0	Ewa-RG-art	1	0

0		0		0		0
0	#N/A	0		0		0
0		0	Ewa-RG-art	1	Ewa-RG-top-a	1
0	Ew/a-p-LOST-	1	Ewa-RG-art	1		0
0		0	Ewa-RG-art	1		0
0		0	Ewa-RG-art	1		0
0	#N/A	0		0		0
0	#N/A	0	Ewa-RG-art	1		0
0	#N/A	0		0		0
0	#N/A	0	Ewa-RG-art	1		0
0	#N/A	0	Ewa-RG-art	1		0
0	#N/A	0	Ewa-RG-art	1		0
0	#N/A	0	Ewa-RG-art	1		0
0		0	Ewa-RG-art	1		0
0	Ew/a-p-LOST-	1	Ewa-RG-art	1		0
0	#N/A	0	Ewa-RG-art	1		0
0	#N/A	0	Ewa-RG-art	1		0
0	#N/A	0		0		0
0	#N/A	0		0		0
0	#N/A	0	Ewa-RG-art	1		0
0	#N/A	0	Ewa-RG-art	1		0
0	#N/A	0	Ewa-RG-art	1		0
0	#N/A	0	Ewa-RG-art	1		0
0	Ew/a-p-LOST-	1	Ewa-RG-art	1		0
0	#N/A	0	Ewa-RG-art	1		0
0	#N/A	0	Ewa-RG-art	1		0
0	Ew/a-p-LOST-	1	Ewa-RG-art	1	Ewa-RG-top-a	1
0	#N/A	0	Ewa-RG-art	1		0
0	#N/A	0	Ewa-RG-art	1		0
0	#N/A	0	Ewa-RG-art	1		0
0	#N/A	0	Ewa-RG-art	1		0
0	#N/A	0	Ewa-RG-art	1		0
0	#N/A	0	Ewa-RG-art	1		0
0	#N/A	0	Ewa-RG-art	1		0
0	#N/A	0		0		0
0	#N/A	0	Ewa-RG-art	1		0
0	#N/A	0	Ewa-RG-art	1		0
0	#N/A	0	Ewa-RG-art	1		0
0	#N/A	0	Ewa-RG-art	1		0
0	#N/A	0	Ewa-RG-art	1		0
0	#N/A	0	Ewa-RG-art	1		0
0	#N/A	0	Ewa-RG-art	1		0
0	#N/A	0		0		0
0	#N/A	0	Ewa-RG-art	1		0
0	#N/A	0	Ewa-RG-art	1		0
0	#N/A	0	Ewa-RG-art	1		0
1	Ew/a-p-LOST-	1	Ewa-RG-art	1		0

0	#N/A	0		0		0
0	#N/A	0	Ewa-RG-art	1		0
0	#N/A	0	Ewa-RG-art	1		0
0	#N/A	0	Ewa-RG-art	1		0
0	#N/A	0		0		0
0	#N/A	0	Ewa-RG-art	1		0
0	#N/A	0	Ewa-RG-art	1		0
0	#N/A	0	Ewa-RG-art	1		0
0	#N/A	0	Ewa-RG-art	1	Ewa-RG-top-a	1
0	#N/A	0	Ewa-RG-art	1	Ewa-RG-top-a	1
0	#N/A	0	Ewa-RG-art	1		0
0	#N/A	0	Ewa-RG-art	1		0
0	#N/A	0		0		0
0	#N/A	0	Ewa-RG-art	1		0
0	#N/A	0	Ewa-RG-art	1		0
0	#N/A	0	Ewa-RG-art	1		0
0	#N/A	0	Ewa-RG-art	1	Ewa-RG-top-a	1
0		0		0		0
0	#N/A	0	Ewa-RG-art	1		0
0	#N/A	0	Ewa-RG-art	1		0
0	#N/A	0	Ewa-RG-art	1		0
0	#N/A	0	Ewa-RG-art	1		0
0	#N/A	0	Ewa-RG-art	1		0
0	#N/A	0	Ewa-RG-art	1		0
0	#N/A	0	Ewa-RG-art	1		0
0	#N/A	0		0		0
0	#N/A	0	Ewa-RG-art	1		0
0	#N/A	0		0		0
0	#N/A	0	Ewa-RG-art	1	Ewa-RG-top-a	1
0	#N/A	0	Ewa-RG-art	1		0
0	#N/A	0	Ewa-RG-art	1		0
0	Ew/a-p-LOST-	1	Ewa-RG-art	1		0
0	Ew/a-p-LOST-	1	Ewa-RG-art	1		0
0	Ew/a-p-LOST-	1	Ewa-RG-art	1		0
0	#N/A	0		0		0
0	#N/A	0	Ewa-RG-art	1		0
0	#N/A	0	Ewa-RG-art	1		0
0	#N/A	0	Ewa-RG-art	1	Ewa-RG-top-a	1
0	#N/A	0		0		0
0	#N/A	0	Ewa-RG-art	1		0
0	#N/A	0	Ewa-RG-art	1		0
0	#N/A	0	Ewa-RG-art	1		0
0	#N/A	0		0		0
0	#N/A	0		0		0
0	#N/A	0		0		0
0		0	Ewa-RG-art	1		0
1	Ew/a-p-LOST-	1	Ewa-RG-art	1		0

1	Ew/a-p-LOST-	1	Ewa-RG-art	1		0
1	Ew/a-p-LOST-	1	Ewa-RG-art	1		0
0	#N/A	0	Ewa-RG-art	1		0
0	#N/A	0	Ewa-RG-art	1		0
0	#N/A	0	Ewa-RG-art	1		0
0	#N/A	0		0		0
0	#N/A	0		0		0
0	#N/A	0	Ewa-RG-art	1		0
0	#N/A	0	Ewa-RG-art	1		0
0	#N/A	0	Ewa-RG-art	1		0
0	#N/A	0	Ewa-RG-art	1		0
0	#N/A	0	Ewa-RG-art	1		0
0	#N/A	0	Ewa-RG-art	1		0
0		0		0		0
0	#N/A	0	Ewa-RG-art	1		0
0	#N/A	0	Ewa-RG-art	1		0
0	#N/A	0	Ewa-RG-art	1		0
0	#N/A	0	Ewa-RG-art	1		0
0	#N/A	0	Ewa-RG-art	1		0
0	#N/A	0	Ewa-RG-art	1		0
0	#N/A	0	Ewa-RG-art	1		0
0	Ew/a-p-LOST-	1	Ewa-RG-art	1		0
0	Ew/a-p-LOST-	1	Ewa-RG-art	1		0
0	#N/A	0	Ewa-RG-art	1		0
0	#N/A	0		0		0
0	#N/A	0		0		0
1	Ew/a-p-LOST-	1	Ewa-RG-art	1	Ewa-RG-top-a	1
1	Ew/a-p-LOST-	1	Ewa-RG-art	1	Ewa-RG-top-a	1
0	#N/A	0	Ewa-RG-art	1		0
0	#N/A	0	Ewa-RG-art	1		0
0	#N/A	0	Ewa-RG-art	1		0
0	#N/A	0	Ewa-RG-art	1	Ewa-RG-top-a	1
0		0		0		0
0	#N/A	0	Ewa-RG-art	1		0
0	#N/A	0		0		0
0	#N/A	0	Ewa-RG-art	1		0
0	#N/A	0	Ewa-RG-art	1		0
0	#N/A	0	Ewa-RG-art	1		0
0		0	Ewa-RG-art	1		0
0		0	Ewa-RG-art	1		0
0		0	Ewa-RG-art	1		0
0		0	Ewa-RG-art	1		0
0	#N/A	0	Ewa-RG-art	1		0
0	#N/A	0		0		0
0	#N/A	0		0		0
0	#N/A	0		0		0
0	#N/A	0		0		0

0	#N/A	0		0		0
0	#N/A	0	Ewa-RG-art	1		0
0	#N/A	0	Ewa-RG-art	1		0
0	#N/A	0	Ewa-RG-art	1		0
0	#N/A	0		0		0
0	#N/A	0	Ewa-RG-art	1		0
0	#N/A	0		0		0
0	#N/A	0	Ewa-RG-art	1		0
0	#N/A	0	Ewa-RG-art	1		0
0	Ew/a-p-LOST-	1	Ewa-RG-art	1		0
0	#N/A	0		0		0
0	#N/A	0		0		0
0	#N/A	0		0		0
0	#N/A	0	Ewa-RG-art	1		0
0	#N/A	0	Ewa-RG-art	1		0
0	#N/A	0	Ewa-RG-art	1		0
0	#N/A	0	Ewa-RG-art	1		0
0		0	Ewa-RG-art	1		0
0		0	Ewa-RG-art	1		0
0	#N/A	0	Ewa-RG-art	1		0
0	#N/A	0	Ewa-RG-art	1		0
0	#N/A	0	Ewa-RG-art	1		0
0	#N/A	0	Ewa-RG-art	1		0
0	#N/A	0		0		0
0	#N/A	0		0		0
0	#N/A	0	Ewa-RG-art	1		0
0	#N/A	0	Ewa-RG-art	1		0
0	#N/A	0	Ewa-RG-art	1		0
0	#N/A	0	Ewa-RG-art	1		0
0		0	Ewa-RG-art	1	Ewa-RG-top-a	1
0		0	Ewa-RG-art	1	Ewa-RG-top-a	1
0	#N/A	0	Ewa-RG-art	1		0
0		0	Ewa-RG-art	1	Ewa-RG-top-a	1
0		0	Ewa-RG-art	1	Ewa-RG-top-a	1
0	#N/A	0	Ewa-RG-art	1		0
0	#N/A	0	Ewa-RG-art	1		0
0	#N/A	0	Ewa-RG-art	1		0
0	#N/A	0		0		0
0	#N/A	0	Ewa-RG-art	1		0
0	Ew/a-p-LOST-	1	Ewa-RG-art	1		0
0	#N/A	0	Ewa-RG-art	1		0
0	#N/A	0	Ewa-RG-art	1		0
0	#N/A	0		0		0
0	#N/A	0	Ewa-RG-art	1	Ewa-RG-top-a	1
0	#N/A	0	Ewa-RG-art	1	Ewa-RG-top-a	1
0	#N/A	0	Ewa-RG-art	1		0
0	#N/A	0	Ewa-RG-art	1		0

0	#N/A	0	Ewa-RG-art	1	Ewa-RG-top-a	1
0	#N/A	0		0		0
0	#N/A	0		0		0
0	#N/A	0	Ewa-RG-art	1		0
0	#N/A	0		0		0
0	#N/A	0		0		0
0		0		0		0
0		0		0		0
0	#N/A	0	Ewa-RG-art	1		0
0	#N/A	0	Ewa-RG-art	1		0
0	#N/A	0	Ewa-RG-art	1	Ewa-RG-top-a	1
0	#N/A	0	Ewa-RG-art	1	Ewa-RG-top-a	1
0	#N/A	0	Ewa-RG-art	1	Ewa-RG-top-a	1
0	#N/A	0	Ewa-RG-art	1	Ewa-RG-top-a	1
0	#N/A	0		0		0
0	#N/A	0		0		0
0	#N/A	0	Ewa-RG-art	1		0
0	#N/A	0	Ewa-RG-art	1		0
0	#N/A	0	Ewa-RG-art	1		0
0		0	Ewa-RG-art	1		0
0		0	Ewa-RG-art	1		0
0		0	Ewa-RG-art	1		0
0		0	Ewa-RG-art	1		0
0	#N/A	0		0		0
0	#N/A	0	Ewa-RG-art	1		0
0	#N/A	0	Ewa-RG-art	1		0
0	#N/A	0	Ewa-RG-art	1		0
0	#N/A	0		0		0
1	Ew/a-p-LOST-	1	Ewa-RG-art	1		0
1	Ew/a-p-LOST-	1	Ewa-RG-art	1		0
1	Ew/a-p-LOST-	1	Ewa-RG-art	1		0
1	Ew/a-p-LOST-	1	Ewa-RG-art	1		0
0	#N/A	0	Ewa-RG-art	1		0
0	#N/A	0	Ewa-RG-art	1		0
0	#N/A	0	Ewa-RG-art	1		0
0	#N/A	0	Ewa-RG-art	1		0
0	#N/A	0	Ewa-RG-art	1	Ewa-RG-top-a	1
0		0		0		0
0	#N/A	0	Ewa-RG-art	1		0
0	#N/A	0	Ewa-RG-art	1		0
0	#N/A	0	Ewa-RG-art	1		0
1	Ew/a-p-LOST-	1	Ewa-RG-art	1		0
1	Ew/a-p-LOST-	1	Ewa-RG-art	1		0
1	Ew/a-p-LOST-	1	Ewa-RG-art	1		0
0		0		0		0
0		0		0		0
0	#N/A	0		0		0

0	#N/A	0		0		0
0	#N/A	0	Ewa-RG-art	1		0
0	#N/A	0		0		0
0	#N/A	0	Ewa-RG-art	1		0
0	#N/A	0	Ewa-RG-art	1		0
0	#N/A	0		0		0
0	#N/A	0		0		0
0	#N/A	0		0		0
0	#N/A	0	Ewa-RG-art	1		0
0	#N/A	0	Ewa-RG-art	1		0
1	Ew/a-p-LOST-	1	Ewa-RG-art	1	Ewa-RG-top-a	1
1	Ew/a-p-LOST-	1	Ewa-RG-art	1	Ewa-RG-top-a	1
0	#N/A	0	Ewa-RG-art	1		0
0	#N/A	0	Ewa-RG-art	1		0
0	#N/A	0	Ewa-RG-art	1		0
0	#N/A	0	Ewa-RG-art	1		0
1	Ew/a-p-LOST-	1	Ewa-RG-art	1		0
1	Ew/a-p-LOST-	1	Ewa-RG-art	1		0
0	#N/A	0	Ewa-RG-art	1		0
0	#N/A	0	Ewa-RG-art	1		0
0	#N/A	0	Ewa-RG-art	1		0
0	#N/A	0	Ewa-RG-art	1		0
1	Ew/a-p-LOST-	1	Ewa-RG-art	1		0
0	#N/A	0	Ewa-RG-art	1		0
0	#N/A	0	Ewa-RG-art	1		0
0	#N/A	0	Ewa-RG-art	1		0
0	#N/A	0	Ewa-RG-art	1		0
0	#N/A	0	Ewa-RG-art	1		0
0	#N/A	0	Ewa-RG-art	1		0
0	Ew/a-p-LOST-	1	Ewa-RG-art	1		0
0	Ew/a-p-LOST-	1	Ewa-RG-art	1		0
0	#N/A	0	Ewa-RG-art	1		0
0	#N/A	0	Ewa-RG-art	1		0
0	#N/A	0	Ewa-RG-art	1		0
0	#N/A	0	Ewa-RG-art	1		0
0	#N/A	0	Ewa-RG-art	1		0
0	#N/A	0	Ewa-RG-art	1		0
0	#N/A	0	Ewa-RG-art	1		0
1	Ew/a-p-LOST-	1	Ewa-RG-art	1		0
1	Ew/a-p-LOST-	1	Ewa-RG-art	1		0
0	#N/A	0	Ewa-RG-art	1		0
0	#N/A	0		0		0
0	#N/A	0		0		0
0	#N/A	0		0		0
0	#N/A	0		0		0
0	#N/A	0	Ewa-RG-art	1		0
0	#N/A	0		0		0

0	#N/A	0	Ewa-RG-art	1		0
0	#N/A	0	Ewa-RG-art	1		0
0	#N/A	0	Ewa-RG-art	1		0
1	Ew/a-p-LOST-	1	Ewa-RG-art	1		0
1	Ew/a-p-LOST-	1	Ewa-RG-art	1		0
1	Ew/a-p-LOST-	1	Ewa-RG-art	1		0
0	#N/A	0	Ewa-RG-art	1		0
0	#N/A	0	Ewa-RG-art	1		0
0	#N/A	0	Ewa-RG-art	1	Ewa-RG-top-a	1
0	#N/A	0	Ewa-RG-art	1		0
0	#N/A	0	Ewa-RG-art	1		0
0		0	Ewa-RG-art	1		0
0		0	Ewa-RG-art	1		0
0		0	Ewa-RG-art	1		0
0	#N/A	0	Ewa-RG-art	1		0
0	#N/A	0	Ewa-RG-art	1		0
0	#N/A	0	Ewa-RG-art	1		0
0	#N/A	0		0		0
0	#N/A	0	Ewa-RG-art	1		0
1	Ew/a-p-LOST-	1	Ewa-RG-art	1	Ewa-RG-top-a	1
0	#N/A	0		0		0
0	#N/A	0		0		0
0	#N/A	0	Ewa-RG-art	1		0
0	#N/A	0	Ewa-RG-art	1		0
0	#N/A	0		0		0
0	#N/A	0		0		0
0	#N/A	0	Ewa-RG-art	1	Ewa-RG-top-a	1
0	#N/A	0	Ewa-RG-art	1	Ewa-RG-top-a	1
0	#N/A	0		0		0
0	#N/A	0	Ewa-RG-art	1		0
0	#N/A	0	Ewa-RG-art	1		0
0	#N/A	0	Ewa-RG-art	1		0
0	#N/A	0	Ewa-RG-art	1		0
0	#N/A	0	Ewa-RG-art	1	Ewa-RG-top-a	1
0	#N/A	0	Ewa-RG-art	1		0
0	#N/A	0	Ewa-RG-art	1		0
0	#N/A	0	Ewa-RG-art	1		0
0	#N/A	0		0		0
0	#N/A	0		0		0
0	#N/A	0	Ewa-RG-art	1		0
0	#N/A	0	Ewa-RG-art	1		0
0	#N/A	0	Ewa-RG-art	1		0
0	#N/A	0	Ewa-RG-art	1		0
0	#N/A	0	Ewa-RG-art	1		0
0	#N/A	0	Ewa-RG-art	1		0
0	#N/A	0	Ewa-RG-art	1		0
0	#N/A	0	Ewa-RG-art	1		0

0		0		0		0
0	#N/A	0		0		0
0	#N/A	0	Ewa-RG-art	1		0
0		0		0		0
1	Ew/a-p-LOST-	1	Ewa-RG-art	1		0
0	Ew/a-p-LOST-	1	Ewa-RG-art	1	Ewa-RG-top-a	1
0	Ew/a-p-LOST-	1	Ewa-RG-art	1	Ewa-RG-top-a	1
0		0		0		0
0	#N/A	0	Ewa-RG-art	1		0
0	#N/A	0	Ewa-RG-art	1		0
0		0	Ewa-RG-art	1		0
0		0	Ewa-RG-art	1		0
0	#N/A	0		0		0
0	#N/A	0	Ewa-RG-art	1		0
0	#N/A	0	Ewa-RG-art	1		0
0	#N/A	0	Ewa-RG-art	1		0
0		0	Ewa-RG-art	1		0
0	#N/A	0	Ewa-RG-art	1		0
0	#N/A	0	Ewa-RG-art	1		0
0	#N/A	0		0		0
0	#N/A	0		0		0
0	#N/A	0	Ewa-RG-art	1		0
0	#N/A	0		0		0
0	#N/A	0		0		0
0	#N/A	0	Ewa-RG-art	1		0
0	#N/A	0		0		0
0	#N/A	0	Ewa-RG-art	1		0
0		0	Ewa-RG-art	1		0
0	#N/A	0		0		0
0	#N/A	0	Ewa-RG-art	1		0
0	#N/A	0	Ewa-RG-art	1	Ewa-RG-top-a	1
0	#N/A	0	Ewa-RG-art	1	Ewa-RG-top-a	1
0	#N/A	0	Ewa-RG-art	1		0
0	#N/A	0	Ewa-RG-art	1		0
1	Ew/a-p-LOST-	1	Ewa-RG-art	1		0
0	#N/A	0		0		0
0	#N/A	0		0		0
0	#N/A	0	Ewa-RG-art	1		0
0	#N/A	0	Ewa-RG-art	1		0
0	#N/A	0	Ewa-RG-art	1		0
0	#N/A	0	Ewa-RG-art	1		0
0	#N/A	0	Ewa-RG-art	1		0
0	#N/A	0	Ewa-RG-art	1		0
0	#N/A	0	Ewa-RG-art	1		0
0	#N/A	0	Ewa-RG-art	1		0
0	#N/A	0	Ewa-RG-art	1		0
0		0	Ewa-RG-art	1		0

0		0	Ewa-RG-art	1		0
0	#N/A	0		0		0
0	#N/A	0	Ewa-RG-art	1		0
0	#N/A	0		0		0
0	#N/A	0	Ewa-RG-art	1		0
0	#N/A	0	Ewa-RG-art	1		0
0	#N/A	0	Ewa-RG-art	1		0
0		0		0		0
0	#N/A	0	Ewa-RG-art	1		0
0	#N/A	0	Ewa-RG-art	1		0
0	#N/A	0	Ewa-RG-art	1		0
0	#N/A	0	Ewa-RG-art	1		0
0		0	Ewa-RG-art	1		0
0		0	Ewa-RG-art	1		0
0		0	Ewa-RG-art	1		0
0	#N/A	0	Ewa-RG-art	1		0
1	Ew/a-p-LOST-	1	Ewa-RG-art	1		0
1	Ew/a-p-LOST-	1	Ewa-RG-art	1		0
1	Ew/a-p-LOST-	1	Ewa-RG-art	1		0
0	#N/A	0	Ewa-RG-art	1		0
0	#N/A	0	Ewa-RG-art	1		0
0		0		0		0
0	#N/A	0		0		0
0		0	Ewa-RG-art	1		0
0		0	Ewa-RG-art	1		0
0	#N/A	0	Ewa-RG-art	1		0
0	#N/A	0	Ewa-RG-art	1		0
0	#N/A	0	Ewa-RG-art	1		0
0	#N/A	0	Ewa-RG-art	1		0
0	#N/A	0	Ewa-RG-art	1		0
0	#N/A	0	Ewa-RG-art	1		0
0		0		0		0
0	#N/A	0	Ewa-RG-art	1		0
0		0	Ewa-RG-art	1		0
0		0	Ewa-RG-art	1		0
0		0	Ewa-RG-art	1		0
0		0	Ewa-RG-art	1		0
0	#N/A	0	Ewa-RG-art	1		0
0		0	Ewa-RG-art	1	Ewa-RG-top-a	1
0		0	Ewa-RG-art	1	Ewa-RG-top-a	1
1	Ew/a-p-LOST-	1	Ewa-RG-art	1		0
1	Ew/a-p-LOST-	1	Ewa-RG-art	1		0
0	#N/A	0	Ewa-RG-art	1		0
0		0	Ewa-RG-art	1		0
0		0	Ewa-RG-art	1		0
0		0	Ewa-RG-art	1		0
0		0	Ewa-RG-art	1		0

0	#N/A	0	Ewa-RG-art	1		0
0	#N/A	0	Ewa-RG-art	1		0
0	#N/A	0	Ewa-RG-art	1		0
0	#N/A	0	Ewa-RG-art	1		0
0	#N/A	0	Ewa-RG-art	1		0
0	#N/A	0	Ewa-RG-art	1		0
0	#N/A	0	Ewa-RG-art	1		0
0	#N/A	0	Ewa-RG-art	1		0
0	#N/A	0	Ewa-RG-art	1		0
0	#N/A	0	Ewa-RG-art	1		0
0	#N/A	0		0		0
0	#N/A	0	Ewa-RG-art	1	Ewa-RG-top-a	1
0	#N/A	0		0		0
0	#N/A	0		0		0
0	#N/A	0		0		0
0	#N/A	0	Ewa-RG-art	1		0
0	#N/A	0	Ewa-RG-art	1		0
0		0	Ewa-RG-art	1		0
0		0	Ewa-RG-art	1		0
0		0	Ewa-RG-art	1		0
0		0	Ewa-RG-art	1		0
0		0	Ewa-RG-art	1		0
0	#N/A	0		0		0
0	#N/A	0		0		0
0	#N/A	0		0		0
0	#N/A	0	Ewa-RG-art	1		0
0		0		0		0
0		0		0		0
0	#N/A	0	Ewa-RG-art	1		0
0	#N/A	0		0		0
0	#N/A	0		0		0
0	#N/A	0	Ewa-RG-art	1		0
0	#N/A	0	Ewa-RG-art	1		0
0		0	Ewa-RG-art	1		0
0		0	Ewa-RG-art	1		0
0	#N/A	0	Ewa-RG-art	1		0
0	#N/A	0	Ewa-RG-art	1		0
0	#N/A	0		0		0
0	#N/A	0		0		0
0	#N/A	0	Ewa-RG-art	1		0
0	#N/A	0	Ewa-RG-art	1		0
0	#N/A	0	Ewa-RG-art	1		0
0	#N/A	0	Ewa-RG-art	1		0
0	#N/A	0		0		0
0	#N/A	0		0		0
1	Ew/a-p-LOST-	1	Ewa-RG-art	1		0
1	Ew/a-p-LOST-	1	Ewa-RG-art	1		0

1	Ew/a-p-LOST-	1	Ewa-RG-art	1		0
0	#N/A	0	Ewa-RG-art	1		0
0	#N/A	0	Ewa-RG-art	1		0
0	#N/A	0	Ewa-RG-art	1		0
1	Ew/a-p-LOST-	1	Ewa-RG-art	1		0
0	#N/A	0		0		0
0	#N/A	0		0		0
0	#N/A	0	Ewa-RG-art	1		0
0	#N/A	0		0		0
0	#N/A	0	Ewa-RG-art	1		0
0	#N/A	0	Ewa-RG-art	1		0
0	#N/A	0	Ewa-RG-art	1		0
0	#N/A	0		0		0
0	#N/A	0	Ewa-RG-art	1		0
0	#N/A	0	Ewa-RG-art	1		0
0	Ew/a-p-LOST-	1	Ewa-RG-art	1		0
0	Ew/a-p-LOST-	1	Ewa-RG-art	1		0
0	#N/A	0	Ewa-RG-art	1		0
0	#N/A	0	Ewa-RG-art	1		0
0	#N/A	0	Ewa-RG-art	1		0
0	#N/A	0	Ewa-RG-art	1		0
1	Ew/a-p-LOST-	1	Ewa-RG-art	1		0
1	Ew/a-p-LOST-	1	Ewa-RG-art	1		0
0	#N/A	0		0		0
0	#N/A	0	Ewa-RG-art	1		0
0	Ew/a-p-LOST-	1	Ewa-RG-art	1		0
0	Ew/a-p-LOST-	1	Ewa-RG-art	1		0
0	Ew/a-p-LOST-	1	Ewa-RG-art	1		0
0	Ew/a-p-LOST-	1	Ewa-RG-art	1		0
0	#N/A	0		0		0
0		0	Ewa-RG-art	1		0
0		0	Ewa-RG-art	1		0
1	Ew/a-p-LOST-	1	Ewa-RG-art	1	Ewa-RG-top-a	1
0	#N/A	0		0		0
0	#N/A	0	Ewa-RG-art	1		0
0	#N/A	0	Ewa-RG-art	1		0
0	#N/A	0	Ewa-RG-art	1		0
0	#N/A	0		0		0
0	#N/A	0		0		0
0		0	Ewa-RG-art	1	Ewa-RG-top-a	1
0		0	Ewa-RG-art	1		0
0	#N/A	0		0		0
0	#N/A	0	Ewa-RG-art	1		0
0	#N/A	0	Ewa-RG-art	1		0
0	#N/A	0	Ewa-RG-art	1		0
0	#N/A	0		0		0
0		0	Ewa-RG-art	1		0

1	Ew/a-p-LOST-	1	Ewa-RG-art	1	Ewa-RG-top-a	1
1	Ew/a-p-LOST-	1	Ewa-RG-art	1	Ewa-RG-top-a	1
0	#N/A	0	Ewa-RG-art	1		0
0	#N/A	0	Ewa-RG-art	1		0
0	#N/A	0	Ewa-RG-art	1		0
0	#N/A	0	Ewa-RG-art	1		0
0	#N/A	0	Ewa-RG-art	1		0
0	#N/A	0	Ewa-RG-art	1		0
0	#N/A	0	Ewa-RG-art	1		0
0	#N/A	0	Ewa-RG-art	1		0
0	#N/A	0		0		0
0	#N/A	0	Ewa-RG-art	1		0
0	#N/A	0	Ewa-RG-art	1		0
0	#N/A	0		0		0
1	Ew/a-p-LOST-	1	Ewa-RG-art	1		0
0	#N/A	0	Ewa-RG-art	1		0
0	#N/A	0		0		0
0	#N/A	0	Ewa-RG-art	1		0
0	#N/A	0	Ewa-RG-art	1		0
0	#N/A	0	Ewa-RG-art	1		0
0	#N/A	0	Ewa-RG-art	1		0
0	#N/A	0		0		0
0	#N/A	0	Ewa-RG-art	1		0
0	#N/A	0		0		0
1	Ew/a-p-LOST-	1	Ewa-RG-art	1		0
1	Ew/a-p-LOST-	1	Ewa-RG-art	1		0
0	#N/A	0	Ewa-RG-art	1		0
0	#N/A	0	Ewa-RG-art	1		0
0	#N/A	0	Ewa-RG-art	1		0
0	#N/A	0		0		0
0	#N/A	0		0		0
0	#N/A	0	Ewa-RG-art	1		0
0	#N/A	0	Ewa-RG-art	1		0
0	#N/A	0		0		0
0	#N/A	0	Ewa-RG-art	1		0
0	#N/A	0		0		0
0	#N/A	0	Ewa-RG-art	1		0
0	#N/A	0	Ewa-RG-art	1		0
0	#N/A	0	Ewa-RG-art	1		0
0		0	Ewa-RG-art	1		0
0	#N/A	0		0		0
0	#N/A	0	Ewa-RG-art	1		0
0		0	Ewa-RG-art	1	Ewa-RG-top-a	1
0	#N/A	0		0		0
0	#N/A	0	Ewa-RG-art	1		0
0	#N/A	0	Ewa-RG-art	1		0
0	#N/A	0	Ewa-RG-art	1		0

0	#N/A	0	Ewa-RG-art	1	0
0	#N/A	0	Ewa-RG-art	1	0
0	#N/A	0	Ewa-RG-art	1	0
0	#N/A	0	Ewa-RG-art	1	0
0	#N/A	0		0	0
0	#N/A	0	Ewa-RG-art	1	0
0	#N/A	0	Ewa-RG-art	1	0
0	#N/A	0	Ewa-RG-art	1	0
0	#N/A	0		0	0
0	#N/A	0	Ewa-RG-art	1	0
1	Ew/a-p-LOST-	1	Ewa-RG-art	1	0
1	Ew/a-p-LOST-	1	Ewa-RG-art	1	0
0	#N/A	0	Ewa-RG-art	1	0
0	#N/A	0	Ewa-RG-art	1	0
0	#N/A	0	Ewa-RG-art	1	0
0	#N/A	0	Ewa-RG-art	1	0
0	#N/A	0	Ewa-RG-art	1	0
0	#N/A	0	Ewa-RG-art	1	0
0	#N/A	0	Ewa-RG-art	1	0
0		0		0	0
0		0		0	0
0	#N/A	0	Ewa-RG-art	1	0
0		0		0	0
0	#N/A	0	Ewa-RG-art	1	0
0	#N/A	0	Ewa-RG-art	1	0
0	#N/A	0		0	0
0	#N/A	0	Ewa-RG-art	1	0
0	#N/A	0	Ewa-RG-art	1	0
0	#N/A	0		0	0
0	#N/A	0	Ewa-RG-art	1	0
0	#N/A	0	Ewa-RG-art	1	0
0	#N/A	0		0	0
1	Ew/a-p-LOST-	1	Ewa-RG-art	1	0
0	#N/A	0		0	0
1	Ew/a-p-LOST-	1		0	0
0	#N/A	0	Ewa-RG-art	1	0
0	#N/A	0	Ewa-RG-art	1	0
0	#N/A	0	Ewa-RG-art	1	0
0	#N/A	0		0	0
0	#N/A	0		0	0
0		0	Ewa-RG-art	1	0
0		0	Ewa-RG-art	1	0
0		0	Ewa-RG-art	1	0
0		0	Ewa-RG-art	1	0
0		0	Ewa-RG-art	1	0
1	Ew/a-p-LOST-	1	Ewa-RG-art	1	0
1	Ew/a-p-LOST-	1	Ewa-RG-art	1	0

1	Ew/a-p-LOST-	1	Ewa-RG-art	1		0
1	Ew/a-p-LOST-	1	Ewa-RG-art	1		0
1	Ew/a-p-LOST-	1	Ewa-RG-art	1		0
0	#N/A	0	Ewa-RG-art	1		0
0	#N/A	0	Ewa-RG-art	1		0
0	#N/A	0	Ewa-RG-art	1		0
0	#N/A	0	Ewa-RG-art	1		0
0	#N/A	0		0		0
0	#N/A	0		0		0
0	#N/A	0	Ewa-RG-art	1		0
0	#N/A	0	Ewa-RG-art	1		0
0	#N/A	0	Ewa-RG-art	1		0
0	#N/A	0	Ewa-RG-art	1	Ewa-RG-top-a	1
0	#N/A	0		0		0
0	#N/A	0		0		0
0	#N/A	0	Ewa-RG-art	1		0
0	#N/A	0	Ewa-RG-art	1	Ewa-RG-top-a	1
1	Ew/a-p-LOST-	1		0		0
0	#N/A	0	Ewa-RG-art	1		0
0	#N/A	0	Ewa-RG-art	1		0
0		0	Ewa-RG-art	1		0
0		0	Ewa-RG-art	1		0
0	#N/A	0	Ewa-RG-art	1		0
0	#N/A	0		0		0
0	#N/A	0	Ewa-RG-art	1		0
0	#N/A	0	Ewa-RG-art	1		0
0	Ew/a-p-LOST-	1	Ewa-RG-art	1		0
0	#N/A	0	Ewa-RG-art	1	Ewa-RG-top-a	1
0	#N/A	0	Ewa-RG-art	1	Ewa-RG-top-a	1
0	#N/A	0	Ewa-RG-art	1		0
0	#N/A	0	Ewa-RG-art	1		0
0	#N/A	0	Ewa-RG-art	1		0
0	#N/A	0	Ewa-RG-art	1		0
1	Ew/a-p-LOST-	1	Ewa-RG-art	1		0
1	Ew/a-p-LOST-	1	Ewa-RG-art	1		0
0	#N/A	0		0		0
0	#N/A	0		0		0
0		0	Ewa-RG-art	1		0
0		0	Ewa-RG-art	1		0
0	#N/A	0		0		0
0	#N/A	0	Ewa-RG-art	1		0
0	#N/A	0	Ewa-RG-art	1		0
0	#N/A	0	Ewa-RG-art	1		0
0	#N/A	0	Ewa-RG-art	1		0
0	#N/A	0	Ewa-RG-art	1		0
0	#N/A	0	Ewa-RG-art	1		0
0	#N/A	0	Ewa-RG-art	1		0

0	#N/A	0	Ewa-RG-art	1		0
0		0		0		0
1	Ew/a-p-LOST-	1	Ewa-RG-art	1		0
1	Ew/a-p-LOST-	1	Ewa-RG-art	1		0
1	Ew/a-p-LOST-	1	Ewa-RG-art	1		0
1	Ew/a-p-LOST-	1	Ewa-RG-art	1		0
1	Ew/a-p-LOST-	1	Ewa-RG-art	1	Ewa-RG-top-a	1
0	#N/A	0	Ewa-RG-art	1		0
0	#N/A	0	Ewa-RG-art	1		0
0	#N/A	0	Ewa-RG-art	1		0
0	#N/A	0	Ewa-RG-art	1		0
0	#N/A	0	Ewa-RG-art	1	Ewa-RG-top-a	1
0	#N/A	0		0		0
1	Ew/a-p-LOST-	1	Ewa-RG-art	1		0
1	Ew/a-p-LOST-	1	Ewa-RG-art	1		0
1	Ew/a-p-LOST-	1	Ewa-RG-art	1		0
0	#N/A	0	Ewa-RG-art	1		0
0	#N/A	0	Ewa-RG-art	1		0
0	#N/A	0	Ewa-RG-art	1		0
0	#N/A	0	Ewa-RG-art	1		0
0	#N/A	0	Ewa-RG-art	1		0
0	#N/A	0	Ewa-RG-art	1		0
0	#N/A	0	Ewa-RG-art	1		0
0	#N/A	0	Ewa-RG-art	1		0
0	#N/A	0	Ewa-RG-art	1		0
0	#N/A	0	Ewa-RG-art	1		0
0	#N/A	0	Ewa-RG-art	1		0
0	#N/A	0	Ewa-RG-art	1		0
1	Ew/a-p-LOST-	1	Ewa-RG-art	1		0
1	Ew/a-p-LOST-	1	Ewa-RG-art	1		0
0	#N/A	0	Ewa-RG-art	1		0
1	Ew/a-p-LOST-	1	Ewa-RG-art	1		0
0	#N/A	0	Ewa-RG-art	1		0
0	#N/A	0	Ewa-RG-art	1		0
0	#N/A	0	Ewa-RG-art	1		0
0	#N/A	0	Ewa-RG-art	1		0
0	#N/A	0		0		0
0	#N/A	0	Ewa-RG-art	1		0
0	#N/A	0		0		0
0	#N/A	0	Ewa-RG-art	1		0
0	#N/A	0	Ewa-RG-art	1		0
0	#N/A	0	Ewa-RG-art	1		0
0	#N/A	0	Ewa-RG-art	1		0
0	#N/A	0	Ewa-RG-art	1		0
0	#N/A	0	Ewa-RG-art	1		0
0	#N/A	0	Ewa-RG-art	1		0
0	#N/A	0	Ewa-RG-art	1		0

0	#N/A	0	Ewa-RG-art	1		0
0	#N/A	0	Ewa-RG-art	1		0
1	Ew/a-p-LOST-	1	Ewa-RG-art	1	Ewa-RG-top-a	1
1	Ew/a-p-LOST-	1	Ewa-RG-art	1	Ewa-RG-top-a	1
1	Ew/a-p-LOST-	1	Ewa-RG-art	1	Ewa-RG-top-a	1
1	Ew/a-p-LOST-	1	Ewa-RG-art	1		0
1	Ew/a-p-LOST-	1	Ewa-RG-art	1		0
1	Ew/a-p-LOST-	1	Ewa-RG-art	1		0
0	#N/A	0		0		0
0	#N/A	0	Ewa-RG-art	1		0
0	#N/A	0	Ewa-RG-art	1		0
0	#N/A	0		0		0
0	#N/A	0		0		0
0	#N/A	0	Ewa-RG-art	1		0
0	#N/A	0	Ewa-RG-art	1		0
0	#N/A	0	Ewa-RG-art	1		0
0	#N/A	0		0		0
0	#N/A	0		0		0
0	#N/A	0		0		0
0	#N/A	0	Ewa-RG-art	1		0
0	#N/A	0		0		0
0	#N/A	0	Ewa-RG-art	1		0
0	#N/A	0	Ewa-RG-art	1		0
0	#N/A	0	Ewa-RG-art	1		0
0	#N/A	0	Ewa-RG-art	1		0
1	Ew/a-p-LOST-	1	Ewa-RG-art	1		0
1	Ew/a-p-LOST-	1	Ewa-RG-art	1		0
1	Ew/a-p-LOST-	1	Ewa-RG-art	1		0
1	Ew/a-p-LOST-	1	Ewa-RG-art	1		0
1	Ew/a-p-LOST-	1	Ewa-RG-art	1		0
0	#N/A	0	Ewa-RG-art	1		0
1	Ew/a-p-LOST-	1	Ewa-RG-art	1		0
1	Ew/a-p-LOST-	1	Ewa-RG-art	1		0
0	#N/A	0	Ewa-RG-art	1		0
0	#N/A	0	Ewa-RG-art	1		0
0	#N/A	0	Ewa-RG-art	1		0
0	#N/A	0	Ewa-RG-art	1		0
0	#N/A	0	Ewa-RG-art	1		0
0	#N/A	0	Ewa-RG-art	1		0
0	#N/A	0	Ewa-RG-art	1		0
0	Ew/a-p-LOST-	1	Ewa-RG-art	1	Ewa-RG-top-a	1
0	#N/A	0		0		0
0	#N/A	0		0		0
0		0	Ewa-RG-art	1		0
0		0	Ewa-RG-art	1		0
0	#N/A	0	Ewa-RG-art	1		0
0	#N/A	0	Ewa-RG-art	1		0

0	#N/A	0	Ewa-RG-art	1		0
0	#N/A	0	Ewa-RG-art	1	Ewa-RG-top-a	1
0	#N/A	0	Ewa-RG-art	1		0
0	#N/A	0	Ewa-RG-art	1		0
0	#N/A	0	Ewa-RG-art	1		0
0	#N/A	0	Ewa-RG-art	1		0
0	#N/A	0	Ewa-RG-art	1		0
0		0	Ewa-RG-art	1		0
0		0	Ewa-RG-art	1		0
0	#N/A	0	Ewa-RG-art	1		0
0	#N/A	0	Ewa-RG-art	1		0
0	#N/A	0	Ewa-RG-art	1		0
0	#N/A	0	Ewa-RG-art	1		0
0	#N/A	0	Ewa-RG-art	1		0
0	#N/A	0		0		0
0	#N/A	0		0		0
0		0		0		0
0	#N/A	0	Ewa-RG-art	1		0
0	#N/A	0	Ewa-RG-art	1		0
0	#N/A	0	Ewa-RG-art	1		0
0	#N/A	0	Ewa-RG-art	1		0
0	#N/A	0	Ewa-RG-art	1		0
0		0		0		0
0		0		0		0
0	#N/A	0	Ewa-RG-art	1		0
0	#N/A	0	Ewa-RG-art	1		0
0	#N/A	0		0		0
0	#N/A	0	Ewa-RG-art	1		0
0	Ew/a-p-LOST-	1	Ewa-RG-art	1	Ewa-RG-top-a	1
0	Ew/a-p-LOST-	1	Ewa-RG-art	1	Ewa-RG-top-a	1
1	Ew/a-p-LOST-	1	Ewa-RG-art	1		0
1	Ew/a-p-LOST-	1	Ewa-RG-art	1		0
0	#N/A	0	Ewa-RG-art	1		0
0	#N/A	0	Ewa-RG-art	1		0
0	#N/A	0	Ewa-RG-art	1		0
1	Ew/a-p-LOST-	1	Ewa-RG-art	1		0
1	Ew/a-p-LOST-	1	Ewa-RG-art	1		0
0	#N/A	0	Ewa-RG-art	1		0
0	#N/A	0	Ewa-RG-art	1		0
0	#N/A	0		0		0
0	#N/A	0		0		0
0	#N/A	0		0		0
0	#N/A	0	Ewa-RG-art	1		0
0	#N/A	0		0		0
0	Ew/a-p-LOST-	1	Ewa-RG-art	1		0
0	Ew/a-p-LOST-	1	Ewa-RG-art	1		0
0	#N/A	0	Ewa-RG-art	1		0

0	#N/A	0		0		0
0	#N/A	0	Ewa-RG-art	1		0
0	#N/A	0	Ewa-RG-art	1		0
0	#N/A	0		0		0
0	#N/A	0		0		0
0	#N/A	0	Ewa-RG-art	1		0
0	#N/A	0	Ewa-RG-art	1		0
0	#N/A	0	Ewa-RG-art	1		0
0	#N/A	0		0		0
0	#N/A	0	Ewa-RG-art	1		0
0	Ew/a-p-LOST-	1	Ewa-RG-art	1	Ewa-RG-top-a	1
0	#N/A	0	Ewa-RG-art	1	Ewa-RG-top-a	1
0	#N/A	0	Ewa-RG-art	1		0
0	#N/A	0	Ewa-RG-art	1		0
0	#N/A	0	Ewa-RG-art	1		0
0	#N/A	0	Ewa-RG-art	1		0
0	#N/A	0	Ewa-RG-art	1		0
0	#N/A	0	Ewa-RG-art	1		0
0	#N/A	0	Ewa-RG-art	1		0
0	#N/A	0		0		0
0	#N/A	0		0		0
0	#N/A	0	Ewa-RG-art	1		0
0	#N/A	0	Ewa-RG-art	1		0
0	#N/A	0	Ewa-RG-art	1		0
0	#N/A	0		0		0
0	#N/A	0	Ewa-RG-art	1	Ewa-RG-top-a	1
0	#N/A	0	Ewa-RG-art	1	Ewa-RG-top-a	1
0	#N/A	0	Ewa-RG-art	1		0
0	#N/A	0	Ewa-RG-art	1		0
0	#N/A	0	Ewa-RG-art	1		0
0	#N/A	0	Ewa-RG-art	1		0
0	#N/A	0	Ewa-RG-art	1		0
0	#N/A	0	Ewa-RG-art	1		0
0	#N/A	0	Ewa-RG-art	1		0
0		0	Ewa-RG-art	1	Ewa-RG-top-a	1
0		0	Ewa-RG-art	1	Ewa-RG-top-a	1
0	#N/A	0	Ewa-RG-art	1		0
0	#N/A	0	Ewa-RG-art	1	Ewa-RG-top-a	1
0	#N/A	0	Ewa-RG-art	1	Ewa-RG-top-a	1
0	#N/A	0		0		0
1	Ew/a-p-LOST-	1	Ewa-RG-art	1		0
1	Ew/a-p-LOST-	1	Ewa-RG-art	1		0
1	Ew/a-p-LOST-	1	Ewa-RG-art	1		0
0	#N/A	0		0		0
0	#N/A	0	Ewa-RG-art	1		0
0	#N/A	0	Ewa-RG-art	1		0
0	#N/A	0	Ewa-RG-art	1		0

0		0	Ewa-RG-art	1		0
0		0	Ewa-RG-art	1		0
0		0	Ewa-RG-art	1		0
0	#N/A	0	Ewa-RG-art	1		0
0	#N/A	0	Ewa-RG-art	1		0
0	#N/A	0	Ewa-RG-art	1		0
0	#N/A	0		0		0
0	#N/A	0		0		0
0	#N/A	0		0		0
0	#N/A	0	Ewa-RG-art	1		0
0		0		0		0
0	#N/A	0	Ewa-RG-art	1	Ewa-RG-top-a	1
0	#N/A	0	Ewa-RG-art	1		0
0	#N/A	0	Ewa-RG-art	1		0
0	#N/A	0	Ewa-RG-art	1		0
0	#N/A	0		0		0
0	Ew/a-p-LOST-	1	Ewa-RG-art	1		0
0	#N/A	0		0		0
0	#N/A	0	Ewa-RG-art	1		0
0	#N/A	0	Ewa-RG-art	1		0
0	#N/A	0	Ewa-RG-art	1		0
0	#N/A	0	Ewa-RG-art	1		0
0	#N/A	0	Ewa-RG-art	1		0
0	#N/A	0	Ewa-RG-art	1		0
0	#N/A	0	Ewa-RG-art	1		0
0	#N/A	0		0		0
0		0	Ewa-RG-art	1		0
0	#N/A	0	Ewa-RG-art	1		0
0	#N/A	0	Ewa-RG-art	1		0
0	Ew/a-p-LOST-	1	Ewa-RG-art	1		0
0	Ew/a-p-LOST-	1	Ewa-RG-art	1		0
0	#N/A	0	Ewa-RG-art	1		0
0	#N/A	0	Ewa-RG-art	1		0
0	#N/A	0	Ewa-RG-art	1		0
0	#N/A	0	Ewa-RG-art	1		0
0	#N/A	0	Ewa-RG-art	1		0
0	#N/A	0	Ewa-RG-art	1		0
0	#N/A	0	Ewa-RG-art	1		0
0	#N/A	0		0		0
0	#N/A	0	Ewa-RG-art	1		0
0	#N/A	0	Ewa-RG-art	1		0
0	#N/A	0	Ewa-RG-art	1		0
0		0		0		0
0	#N/A	0	Ewa-RG-art	1		0
0	#N/A	0	Ewa-RG-art	1		0
0	#N/A	0	Ewa-RG-art	1		0
0	#N/A	0	Ewa-RG-art	1		0

0	#N/A	0	Ewa-RG-art	1		0
0	#N/A	0		0		0
0	#N/A	0	Ewa-RG-art	1		0
0	#N/A	0	Ewa-RG-art	1		0
0	#N/A	0	Ewa-RG-art	1		0
0	#N/A	0	Ewa-RG-art	1		0
0	#N/A	0		0		0
0	#N/A	0		0		0
0	#N/A	0	Ewa-RG-art	1		0
0	#N/A	0	Ewa-RG-art	1		0
0	#N/A	0		0		0
0	#N/A	0	Ewa-RG-art	1		0
0	#N/A	0	Ewa-RG-art	1		0
0	#N/A	0	Ewa-RG-art	1		0
0	#N/A	0	Ewa-RG-art	1		0
0	#N/A	0	Ewa-RG-art	1		0
0	#N/A	0	Ewa-RG-art	1		0
0	#N/A	0		0		0
0	#N/A	0		0		0
0	#N/A	0	Ewa-RG-art	1		0
0	#N/A	0	Ewa-RG-art	1		0
0	#N/A	0	Ewa-RG-art	1		0
0	#N/A	0	Ewa-RG-art	1		0
0	#N/A	0	Ewa-RG-art	1	Ewa-RG-top-a	1
0		0	Ewa-RG-art	1		0
0	#N/A	0	Ewa-RG-art	1		0
0	#N/A	0		0		0
0	#N/A	0		0		0
0		0		0		0
0	#N/A	0	Ewa-RG-art	1	Ewa-RG-top-a	1
0		0	Ewa-RG-art	1		0
0	#N/A	0	Ewa-RG-art	1		0
0		0		0		0
0	#N/A	0	Ewa-RG-art	1		0
0	#N/A	0	Ewa-RG-art	1		0
0	#N/A	0	Ewa-RG-art	1		0
0	#N/A	0	Ewa-RG-art	1		0
0	#N/A	0	Ewa-RG-art	1	Ewa-RG-top-a	1
0	#N/A	0	Ewa-RG-art	1	Ewa-RG-top-a	1
0	#N/A	0	Ewa-RG-art	1		0
0	#N/A	0	Ewa-RG-art	1		0
0		0	Ewa-RG-art	1		0
0		0	Ewa-RG-art	1		0
0	#N/A	0		0		0
0	#N/A	0		0		0
0	#N/A	0	Ewa-RG-art	1		0
0	#N/A	0	Ewa-RG-art	1		0

0	#N/A	0	Ewa-RG-art	1		0
0	#N/A	0		0		0
0	#N/A	0		0		0
0	#N/A	0		0		0
0		0	Ewa-RG-art	1	Ewa-RG-top-a	1
1	Ew/a-p-LOST-	1		0		0
0	#N/A	0	Ewa-RG-art	1	Ewa-RG-top-a	1
0	#N/A	0	Ewa-RG-art	1		0
0	#N/A	0	Ewa-RG-art	1		0
0	#N/A	0		0		0
0	#N/A	0		0		0
0	#N/A	0	Ewa-RG-art	1		0
0	#N/A	0	Ewa-RG-art	1		0
0		0	Ewa-RG-art	1	Ewa-RG-top-a	1
0		0	Ewa-RG-art	1	Ewa-RG-top-a	1
0		0	Ewa-RG-art	1	Ewa-RG-top-a	1
0	#N/A	0	Ewa-RG-art	1		0
0	#N/A	0	Ewa-RG-art	1		0
0		0		0		0
0	#N/A	0	Ewa-RG-art	1		0
0	#N/A	0	Ewa-RG-art	1		0
0	#N/A	0	Ewa-RG-art	1		0
0	#N/A	0	Ewa-RG-art	1		0
0	#N/A	0		0		0
0	#N/A	0	Ewa-RG-art	1		0
0	#N/A	0	Ewa-RG-art	1		0
0	#N/A	0	Ewa-RG-art	1		0
0	#N/A	0	Ewa-RG-art	1		0
0	#N/A	0	Ewa-RG-art	1		0
0	#N/A	0	Ewa-RG-art	1		0
0	#N/A	0	Ewa-RG-art	1		0
0	#N/A	0		0		0
0	#N/A	0	Ewa-RG-art	1		0
0	#N/A	0	Ewa-RG-art	1		0
0	#N/A	0		0		0
0	#N/A	0	Ewa-RG-art	1		0
0	#N/A	0	Ewa-RG-art	1		0
0	#N/A	0	Ewa-RG-art	1		0
0	#N/A	0	Ewa-RG-art	1		0
0	#N/A	0		0		0
0	#N/A	0		0		0
0	#N/A	0	Ewa-RG-art	1		0
0	#N/A	0		0		0
0	#N/A	0	Ewa-RG-art	1		0
0	#N/A	0	Ewa-RG-art	1		0
0	#N/A	0	Ewa-RG-art	1		0
0	#N/A	0	Ewa-RG-art	1		0

0	#N/A	0	Ewa-RG-art	1		0
0	#N/A	0	Ewa-RG-art	1		0
0	#N/A	0		0		0
0	#N/A	0	Ewa-RG-art	1		0
0	#N/A	0	Ewa-RG-art	1		0
0	#N/A	0		0		0
0		0	Ewa-RG-art	1		0
0		0	Ewa-RG-art	1		0
0	#N/A	0	Ewa-RG-art	1		0
0	#N/A	0	Ewa-RG-art	1		0
1	Ew/a-p-LOST-	1	Ewa-RG-art	1		0
1	Ew/a-p-LOST-	1	Ewa-RG-art	1		0
0	#N/A	0	Ewa-RG-art	1		0
0	#N/A	0	Ewa-RG-art	1		0
0	#N/A	0		0		0
0	#N/A	0	Ewa-RG-art	1		0
0	#N/A	0	Ewa-RG-art	1		0
0	#N/A	0	Ewa-RG-art	1		0
0	#N/A	0	Ewa-RG-art	1		0
1	Ew/a-p-LOST-	1	Ewa-RG-art	1		0
1	Ew/a-p-LOST-	1	Ewa-RG-art	1		0
1	Ew/a-p-LOST-	1	Ewa-RG-art	1		0
0	#N/A	0	Ewa-RG-art	1	Ewa-RG-top-a	1
0	#N/A	0		0		0
0	#N/A	0	Ewa-RG-art	1		0
0	#N/A	0	Ewa-RG-art	1		0
0	#N/A	0	Ewa-RG-art	1		0
0	#N/A	0	Ewa-RG-art	1		0
0	#N/A	0	Ewa-RG-art	1		0
0	#N/A	0	Ewa-RG-art	1	Ewa-RG-top-a	1
0	#N/A	0	Ewa-RG-art	1	Ewa-RG-top-a	1
0	#N/A	0		0		0
0	#N/A	0		0		0
0	#N/A	0	Ewa-RG-art	1		0
0	#N/A	0	Ewa-RG-art	1		0
0	#N/A	0	Ewa-RG-art	1		0
0	#N/A	0	Ewa-RG-art	1		0
0	#N/A	0	Ewa-RG-art	1		0
0	#N/A	0	Ewa-RG-art	1		0
0	#N/A	0	Ewa-RG-art	1		0
0	#N/A	0	Ewa-RG-art	1		0
0	#N/A	0	Ewa-RG-art	1		0
0	#N/A	0	Ewa-RG-art	1		0
0	#N/A	0		0		0
0		0	Ewa-RG-art	1	Ewa-RG-top-a	1
0		0	Ewa-RG-art	1	Ewa-RG-top-a	1
0		0	Ewa-RG-art	1	Ewa-RG-top-a	1

0	#N/A	0	Ewa-RG-art	1		0
0	#N/A	0	Ewa-RG-art	1		0
0	#N/A	0	Ewa-RG-art	1		0
0	#N/A	0	Ewa-RG-art	1		0
0	#N/A	0	Ewa-RG-art	1		0
0	#N/A	0	Ewa-RG-art	1		0
0	#N/A	0	Ewa-RG-art	1	Ewa-RG-top-a	1
0		0	Ewa-RG-art	1		0
0	#N/A	0		0		0
0	#N/A	0	Ewa-RG-art	1		0
0	#N/A	0	Ewa-RG-art	1		0
0	Ew/a-p-LOST-	1	Ewa-RG-art	1		0
0	Ew/a-p-LOST-	1	Ewa-RG-art	1		0
0	#N/A	0	Ewa-RG-art	1		0
0	#N/A	0	Ewa-RG-art	1		0
0	#N/A	0	Ewa-RG-art	1		0
1	Ew/a-p-LOST-	1	Ewa-RG-art	1		0
1	Ew/a-p-LOST-	1	Ewa-RG-art	1		0
0	#N/A	0		0		0
0	#N/A	0		0		0
0		0	Ewa-RG-art	1		0
0	#N/A	0		0		0
0	#N/A	0		0		0
0	Ew/a-p-LOST-	1	Ewa-RG-art	1		0
0	#N/A	0	Ewa-RG-art	1		0
0		0	Ewa-RG-art	1		0
0	#N/A	0	Ewa-RG-art	1		0
0	#N/A	0	Ewa-RG-art	1		0
0	#N/A	0	Ewa-RG-art	1		0
0	#N/A	0		0		0
0	#N/A	0	Ewa-RG-art	1		0
0	#N/A	0	Ewa-RG-art	1		0
0	#N/A	0	Ewa-RG-art	1		0
0	#N/A	0	Ewa-RG-art	1		0
1	Ew/a-p-LOST-	1	Ewa-RG-art	1		0
0	#N/A	0	Ewa-RG-art	1		0
0	#N/A	0	Ewa-RG-art	1		0
0	#N/A	0		0		0
0	#N/A	0		0		0
0	#N/A	0	Ewa-RG-art	1		0
0	#N/A	0	Ewa-RG-art	1		0
0	#N/A	0		0		0
0		0	Ewa-RG-art	1		0
0	#N/A	0	Ewa-RG-art	1		0
0		0		0		0
0	#N/A	0		0		0
0	#N/A	0	Ewa-RG-art	1		0

0	#N/A	0	Ewa-RG-art	1		0
0	#N/A	0	Ewa-RG-art	1		0
0	#N/A	0	Ewa-RG-art	1		0
0	#N/A	0	Ewa-RG-art	1		0
0	#N/A	0	Ewa-RG-art	1		0
0	#N/A	0	Ewa-RG-art	1		0
0		0		0		0
0	#N/A	0	Ewa-RG-art	1		0
0	#N/A	0	Ewa-RG-art	1		0
0	#N/A	0	Ewa-RG-art	1		0
1	Ew/a-p-LOST-	1	Ewa-RG-art	1	Ewa-RG-top-a	1
0	#N/A	0	Ewa-RG-art	1	Ewa-RG-top-a	1
0	#N/A	0	Ewa-RG-art	1	Ewa-RG-top-a	1
0	#N/A	0	Ewa-RG-art	1		0
0	#N/A	0	Ewa-RG-art	1	Ewa-RG-top-a	1
0	#N/A	0	Ewa-RG-art	1		0
0	#N/A	0	Ewa-RG-art	1		0
0	#N/A	0		0		0
0	#N/A	0	Ewa-RG-art	1		0
0	#N/A	0		0		0
0	#N/A	0		0		0
0	Ew/a-p-LOST-	1	Ewa-RG-art	1		0
0	Ew/a-p-LOST-	1	Ewa-RG-art	1		0
0	Ew/a-p-LOST-	1	Ewa-RG-art	1		0
0	#N/A	0		0		0
0	#N/A	0	Ewa-RG-art	1		0
0	#N/A	0	Ewa-RG-art	1		0
0	#N/A	0	Ewa-RG-art	1		0
0	#N/A	0	Ewa-RG-art	1		0
0	#N/A	0	Ewa-RG-art	1		0
0	#N/A	0	Ewa-RG-art	1		0
0	#N/A	0	Ewa-RG-art	1		0
0	#N/A	0	Ewa-RG-art	1		0
0	#N/A	0	Ewa-RG-art	1	Ewa-RG-top-a	1
0	#N/A	0	Ewa-RG-art	1	Ewa-RG-top-a	1
0	#N/A	0	Ewa-RG-art	1		0
0	#N/A	0	Ewa-RG-art	1		0
0	#N/A	0	Ewa-RG-art	1		0
0	#N/A	0	Ewa-RG-art	1		0
0	#N/A	0	Ewa-RG-art	1		0
0		0	Ewa-RG-art	1	Ewa-RG-top-a	1
0	#N/A	0	Ewa-RG-art	1		0
0	#N/A	0	Ewa-RG-art	1		0
0	Ew/a-p-LOST-	1		0		0
0	#N/A	0		0		0
0		0	Ewa-RG-art	1		0
0	#N/A	0	Ewa-RG-art	1	Ewa-RG-top-a	1

0	#N/A	0	Ewa-RG-art	1	Ewa-RG-top-a	1
0	#N/A	0	Ewa-RG-art	1		0
0	#N/A	0	Ewa-RG-art	1		0
0	#N/A	0	Ewa-RG-art	1		0
0	#N/A	0	Ewa-RG-art	1		0
0	#N/A	0	Ewa-RG-art	1		0
0	#N/A	0	Ewa-RG-art	1		0
0		0	Ewa-RG-art	1	Ewa-RG-top-a	1
0		0	Ewa-RG-art	1	Ewa-RG-top-a	1
0		0	Ewa-RG-art	1	Ewa-RG-top-a	1
1	Ew/a-p-LOST-	1	Ewa-RG-art	1		0
1	Ew/a-p-LOST-	1	Ewa-RG-art	1		0
1	Ew/a-p-LOST-	1	Ewa-RG-art	1		0
1	Ew/a-p-LOST-	1	Ewa-RG-art	1		0
0	#N/A	0		0		0
0	#N/A	0	Ewa-RG-art	1		0
0	#N/A	0	Ewa-RG-art	1		0
0	#N/A	0	Ewa-RG-art	1		0
0	#N/A	0	Ewa-RG-art	1		0
0	#N/A	0	Ewa-RG-art	1		0
0	#N/A	0	Ewa-RG-art	1		0
0	#N/A	0		0		0
0	#N/A	0		0		0
0	#N/A	0		0		0
0	#N/A	0	Ewa-RG-art	1		0
0	#N/A	0	Ewa-RG-art	1		0
0	#N/A	0	Ewa-RG-art	1		0
0	#N/A	0	Ewa-RG-art	1		0
0	#N/A	0		0		0
0	#N/A	0	Ewa-RG-art	1		0
0	#N/A	0	Ewa-RG-art	1		0
0	#N/A	0	Ewa-RG-art	1		0
0		0	Ewa-RG-art	1		0
0		0	Ewa-RG-art	1		0
0	#N/A	0	Ewa-RG-art	1		0
0	#N/A	0		0		0
0	#N/A	0	Ewa-RG-art	1		0
1	Ew/a-p-LOST-	1	Ewa-RG-art	1		0
1	Ew/a-p-LOST-	1	Ewa-RG-art	1		0
0	#N/A	0	Ewa-RG-art	1		0
0	#N/A	0	Ewa-RG-art	1		0
0		0	Ewa-RG-art	1		0
0	#N/A	0	Ewa-RG-art	1		0
1	Ew/a-p-LOST-	1	Ewa-RG-art	1		0
1	Ew/a-p-LOST-	1	Ewa-RG-art	1		0
1	Ew/a-p-LOST-	1	Ewa-RG-art	1		0
0	#N/A	0	Ewa-RG-art	1		0

0	#N/A	0	Ewa-RG-art	1		0
0	#N/A	0	Ewa-RG-art	1		0
0	#N/A	0	Ewa-RG-art	1		0
0	#N/A	0	Ewa-RG-art	1		0
0	#N/A	0	Ewa-RG-art	1		0
0	#N/A	0	Ewa-RG-art	1		0
0	#N/A	0	Ewa-RG-art	1		0
0	Ew/a-p-LOST-	1	Ewa-RG-art	1		0
0	Ew/a-p-LOST-	1	Ewa-RG-art	1		0
0	#N/A	0		0		0
0	#N/A	0		0		0
0	#N/A	0	Ewa-RG-art	1		0
0		0		0		0
0	#N/A	0	Ewa-RG-art	1		0
0	#N/A	0	Ewa-RG-art	1		0
0	#N/A	0	Ewa-RG-art	1		0
0	#N/A	0	Ewa-RG-art	1		0
0	#N/A	0	Ewa-RG-art	1		0
0	#N/A	0	Ewa-RG-art	1		0
0	#N/A	0		0		0
0		0		0		0
0	#N/A	0		0		0
1	Ew/a-p-LOST-	1	Ewa-RG-art	1	Ewa-RG-top-a	1
1	Ew/a-p-LOST-	1	Ewa-RG-art	1	Ewa-RG-top-a	1
0		0	Ewa-RG-art	1	Ewa-RG-top-a	1
0	#N/A	0	Ewa-RG-art	1	Ewa-RG-top-a	1
0	#N/A	0		0		0
0	#N/A	0	Ewa-RG-art	1		0
0	#N/A	0		0		0
0		0	Ewa-RG-art	1		0
0	#N/A	0	Ewa-RG-art	1		0
0	#N/A	0	Ewa-RG-art	1		0
0	#N/A	0		0		0
0	#N/A	0	Ewa-RG-art	1		0
0	#N/A	0	Ewa-RG-art	1		0
0	#N/A	0	Ewa-RG-art	1	Ewa-RG-top-a	1
0	#N/A	0	Ewa-RG-art	1		0
0	#N/A	0	Ewa-RG-art	1		0
0	#N/A	0		0		0
0	#N/A	0	Ewa-RG-art	1		0
0	#N/A	0		0		0
0	#N/A	0		0		0
0		0		0		0
1	Ew/a-p-LOST-	1	Ewa-RG-art	1		0
1	Ew/a-p-LOST-	1	Ewa-RG-art	1		0
1	Ew/a-p-LOST-	1	Ewa-RG-art	1		0
1	Ew/a-p-LOST-	1	Ewa-RG-art	1		0

0	#N/A	0	Ewa-RG-art	1		0
0	#N/A	0	Ewa-RG-art	1		0
0	#N/A	0		0		0
0	#N/A	0		0		0
0	#N/A	0	Ewa-RG-art	1		0
0	#N/A	0	Ewa-RG-art	1		0
0	#N/A	0		0		0
0	#N/A	0		0		0
0	#N/A	0	Ewa-RG-art	1		0
0	#N/A	0	Ewa-RG-art	1	Ewa-RG-top-a	1
0	#N/A	0	Ewa-RG-art	1	Ewa-RG-top-a	1
0		0	Ewa-RG-art	1		0
0	#N/A	0	Ewa-RG-art	1		0
0	#N/A	0	Ewa-RG-art	1		0
0	#N/A	0		0		0
0	#N/A	0		0		0
0	#N/A	0		0		0
0	#N/A	0		0		0
0	#N/A	0		0		0
0	#N/A	0	Ewa-RG-art	1	Ewa-RG-top-a	1
0	#N/A	0	Ewa-RG-art	1	Ewa-RG-top-a	1
0	#N/A	0	Ewa-RG-art	1		0
0	#N/A	0	Ewa-RG-art	1		0
0	#N/A	0	Ewa-RG-art	1		0
0	#N/A	0	Ewa-RG-art	1		0
0	#N/A	0	Ewa-RG-art	1		0
0	#N/A	0	Ewa-RG-art	1		0
0	#N/A	0	Ewa-RG-art	1		0
0	#N/A	0	Ewa-RG-art	1		0
0	#N/A	0	Ewa-RG-art	1		0
0	#N/A	0	Ewa-RG-art	1		0
0	#N/A	0	Ewa-RG-art	1		0
0	#N/A	0	Ewa-RG-art	1		0
0	#N/A	0	Ewa-RG-art	1		0
1	Ew/a-p-LOST-	1	Ewa-RG-art	1		0
1	Ew/a-p-LOST-	1	Ewa-RG-art	1		0
0	#N/A	0		0		0
0	#N/A	0	Ewa-RG-art	1		0
0	#N/A	0	Ewa-RG-art	1		0
1	Ew/a-p-LOST-	1	Ewa-RG-art	1		0
1	Ew/a-p-LOST-	1	Ewa-RG-art	1		0
1	Ew/a-p-LOST-	1	Ewa-RG-art	1		0
0		0	Ewa-RG-art	1		0
0		0	Ewa-RG-art	1		0
0	#N/A	0	Ewa-RG-art	1		0
0	#N/A	0	Ewa-RG-art	1		0
0	#N/A	0	Ewa-RG-art	1		0

0	#N/A	0	Ewa-RG-art	1		0
0	#N/A	0		0		0
0	#N/A	0	Ewa-RG-art	1	Ewa-RG-top-a	1
0	#N/A	0	Ewa-RG-art	1	Ewa-RG-top-a	1
0		0		0		0
0	#N/A	0		0		0
0	#N/A	0	Ewa-RG-art	1		0
0	#N/A	0	Ewa-RG-art	1		0
0		0	Ewa-RG-art	1		0
0		0	Ewa-RG-art	1		0
0		0	Ewa-RG-art	1		0
0		0	Ewa-RG-art	1		0
0		0	Ewa-RG-art	1		0
1	Ew/a-p-LOST-	1	Ewa-RG-art	1		0
0	#N/A	0		0		0
0	#N/A	0	Ewa-RG-art	1		0
0	#N/A	0	Ewa-RG-art	1	Ewa-RG-top-a	1
0	#N/A	0	Ewa-RG-art	1		0
0	#N/A	0	Ewa-RG-art	1		0
0	#N/A	0	Ewa-RG-art	1		0
0	#N/A	0	Ewa-RG-art	1		0
0	Ew/a-p-LOST-	1	Ewa-RG-art	1		0
0	Ew/a-p-LOST-	1	Ewa-RG-art	1		0
0	#N/A	0		0		0
0	#N/A	0		0		0
0	#N/A	0		0		0
0	#N/A	0	Ewa-RG-art	1		0
1	Ew/a-p-LOST-	1	Ewa-RG-art	1		0
1	Ew/a-p-LOST-	1	Ewa-RG-art	1		0
0	#N/A	0	Ewa-RG-art	1		0
0	#N/A	0	Ewa-RG-art	1		0
0	#N/A	0	Ewa-RG-art	1		0
0	#N/A	0	Ewa-RG-art	1		0
1	Ew/a-p-LOST-	1	Ewa-RG-art	1		0
1	Ew/a-p-LOST-	1	Ewa-RG-art	1		0
0	#N/A	0	Ewa-RG-art	1		0
0	#N/A	0	Ewa-RG-art	1		0
0	#N/A	0	Ewa-RG-art	1		0
0	Ew/a-p-LOST-	1		0		0
0		0		0		0
0	#N/A	0	Ewa-RG-art	1		0
0	#N/A	0	Ewa-RG-art	1		0
0	#N/A	0	Ewa-RG-art	1		0
0	#N/A	0		0		0
0	#N/A	0		0		0
0	#N/A	0	Ewa-RG-art	1		0
0	#N/A	0	Ewa-RG-art	1		0

0	#N/A	0	Ewa-RG-art	1		0
1	Ew/a-p-LOST-	1	Ewa-RG-art	1	Ewa-RG-top-a	1
0	#N/A	0		0		0
0	#N/A	0	Ewa-RG-art	1		0
0	#N/A	0	Ewa-RG-art	1	Ewa-RG-top-a	1
0	#N/A	0	Ewa-RG-art	1	Ewa-RG-top-a	1
0	#N/A	0		0		0
0	#N/A	0	Ewa-RG-art	1		0
0	#N/A	0	Ewa-RG-art	1		0
0	#N/A	0	Ewa-RG-art	1		0
0	#N/A	0	Ewa-RG-art	1		0
0	#N/A	0		0		0
0	#N/A	0		0		0
0	#N/A	0	Ewa-RG-art	1		0
0	#N/A	0	Ewa-RG-art	1		0
0	#N/A	0	Ewa-RG-art	1		0
0	#N/A	0		0		0
0	#N/A	0	Ewa-RG-art	1		0
0	#N/A	0	Ewa-RG-art	1		0
0	#N/A	0	Ewa-RG-art	1		0
1	Ew/a-p-LOST-	1	Ewa-RG-art	1	Ewa-RG-top-a	1
0	#N/A	0		0		0
0		0	Ewa-RG-art	1		0
0	#N/A	0		0		0
0	#N/A	0	Ewa-RG-art	1	Ewa-RG-top-a	1
0		0	Ewa-RG-art	1		0
0	#N/A	0	Ewa-RG-art	1		0
1	Ew/a-p-LOST-	1		0		0
1	Ew/a-p-LOST-	1		0		0
0	#N/A	0		0		0
0	#N/A	0	Ewa-RG-art	1	Ewa-RG-top-a	1
0	#N/A	0		0		0
0	#N/A	0		0		0
0	#N/A	0	Ewa-RG-art	1		0
0	#N/A	0		0		0
0	#N/A	0	Ewa-RG-art	1		0
0	#N/A	0	Ewa-RG-art	1		0
0	#N/A	0	Ewa-RG-art	1		0
0	#N/A	0	Ewa-RG-art	1		0
0	#N/A	0	Ewa-RG-art	1		0
1	Ew/a-p-LOST-	1	Ewa-RG-art	1	Ewa-RG-top-a	1
1	Ew/a-p-LOST-	1	Ewa-RG-art	1	Ewa-RG-top-a	1
1	Ew/a-p-LOST-	1	Ewa-RG-art	1	Ewa-RG-top-a	1
0	#N/A	0		0		0
0	#N/A	0	Ewa-RG-art	1		0
0	#N/A	0	Ewa-RG-art	1		0
0	#N/A	0	Ewa-RG-art	1		0

0	#N/A	0	Ewa-RG-art	1		0
0	#N/A	0	Ewa-RG-art	1		0
0	#N/A	0	Ewa-RG-art	1		0
0	#N/A	0	Ewa-RG-art	1		0
0		0	Ewa-RG-art	1		0
0		0	Ewa-RG-art	1		0
0	#N/A	0		0		0
0	#N/A	0	Ewa-RG-art	1	Ewa-RG-top-a	1
0	#N/A	0	Ewa-RG-art	1	Ewa-RG-top-a	1
0	#N/A	0	Ewa-RG-art	1		0
0	#N/A	0	Ewa-RG-art	1		0
0	#N/A	0	Ewa-RG-art	1		0
0	#N/A	0	Ewa-RG-art	1		0
0	#N/A	0	Ewa-RG-art	1		0
0	#N/A	0	Ewa-RG-art	1		0
0	#N/A	0	Ewa-RG-art	1		0
0	#N/A	0	Ewa-RG-art	1		0
0	#N/A	0	Ewa-RG-art	1		0
1	Ew/a-p-LOST-	1	Ewa-RG-art	1		0
1	Ew/a-p-LOST-	1	Ewa-RG-art	1		0
1	Ew/a-p-LOST-	1	Ewa-RG-art	1		0
0	#N/A	0	Ewa-RG-art	1		0
0	#N/A	0		0		0
0	#N/A	0	Ewa-RG-art	1		0
0	#N/A	0	Ewa-RG-art	1		0
0	#N/A	0	Ewa-RG-art	1		0
0	#N/A	0	Ewa-RG-art	1		0
0		0	Ewa-RG-art	1		0
0	#N/A	0	Ewa-RG-art	1		0
0	#N/A	0	Ewa-RG-art	1		0
0	#N/A	0	Ewa-RG-art	1		0
0	#N/A	0	Ewa-RG-art	1		0
0	#N/A	0	Ewa-RG-art	1		0
0	#N/A	0	Ewa-RG-art	1		0
0	#N/A	0	Ewa-RG-art	1		0
0		0	Ewa-RG-art	1		0
0		0	Ewa-RG-art	1		0
0	Ew/a-p-LOST-	1	Ewa-RG-art	1		0
0	#N/A	0	Ewa-RG-art	1		0
0	#N/A	0	Ewa-RG-art	1		0
0	#N/A	0		0		0
0	#N/A	0		0		0
0	#N/A	0		0		0
0		0		0		0
0	#N/A	0	Ewa-RG-art	1		0
0	#N/A	0	Ewa-RG-art	1		0
0	#N/A	0	Ewa-RG-art	1		0

0	#N/A	0	Ewa-RG-art	1		0
0	#N/A	0	Ewa-RG-art	1	Ewa-RG-top-a	1
0	#N/A	0	Ewa-RG-art	1		0
1	Ew/a-p-LOST-	1	Ewa-RG-art	1		0
0	#N/A	0	Ewa-RG-art	1		0
0	#N/A	0	Ewa-RG-art	1		0
0	#N/A	0	Ewa-RG-art	1		0
0	#N/A	0		0		0
0	#N/A	0		0		0
0	#N/A	0	Ewa-RG-art	1		0
0	#N/A	0	Ewa-RG-art	1		0
0	#N/A	0	Ewa-RG-art	1		0
0		0	Ewa-RG-art	1		0
0		0	Ewa-RG-art	1		0
0		0	Ewa-RG-art	1		0
0	#N/A	0	Ewa-RG-art	1		0
0	#N/A	0	Ewa-RG-art	1		0
0	#N/A	0	Ewa-RG-art	1		0
0	#N/A	0	Ewa-RG-art	1		0
0	#N/A	0		0		0
0	#N/A	0	Ewa-RG-art	1		0
0	#N/A	0	Ewa-RG-art	1		0
0	#N/A	0	Ewa-RG-art	1		0
0	#N/A	0		0		0
0		0	Ewa-RG-art	1		0
0	#N/A	0	Ewa-RG-art	1		0
0	#N/A	0	Ewa-RG-art	1		0
0	#N/A	0	Ewa-RG-art	1		0
0	#N/A	0	Ewa-RG-art	1		0
0	#N/A	0	Ewa-RG-art	1		0
0	#N/A	0	Ewa-RG-art	1		0
0	#N/A	0	Ewa-RG-art	1		0
0	#N/A	0	Ewa-RG-art	1		0
0	#N/A	0	Ewa-RG-art	1		0
0		0	Ewa-RG-art	1		0
0		0	Ewa-RG-art	1		0
0		0	Ewa-RG-art	1		0
0	#N/A	0		0		0
0	#N/A	0	Ewa-RG-art	1		0
0	#N/A	0	Ewa-RG-art	1		0
0	#N/A	0	Ewa-RG-art	1		0
0	#N/A	0	Ewa-RG-art	1		0
0	#N/A	0	Ewa-RG-art	1		0
0	#N/A	0	Ewa-RG-art	1		0
0	#N/A	0	Ewa-RG-art	1		0
0	#N/A	0	Ewa-RG-art	1		0
0	#N/A	0	Ewa-RG-art	1		0

0	#N/A	0	Ewa-RG-art	1		0
0	#N/A	0		0		0
0	#N/A	0	Ewa-RG-art	1		0
0	#N/A	0	Ewa-RG-art	1		0
0	#N/A	0	Ewa-RG-art	1		0
0	#N/A	0	Ewa-RG-art	1		0
0	#N/A	0	Ewa-RG-art	1		0
0		0	Ewa-RG-art	1	Ewa-RG-top-a	1
0		0	Ewa-RG-art	1	Ewa-RG-top-a	1
0	#N/A	0		0		0
1	Ew/a-p-LOST-	1	Ewa-RG-art	1		0
1	Ew/a-p-LOST-	1	Ewa-RG-art	1		0
0	#N/A	0		0		0
0	#N/A	0	Ewa-RG-art	1		0
0	#N/A	0	Ewa-RG-art	1		0
0	#N/A	0	Ewa-RG-art	1		0
0		0	Ewa-RG-art	1		0
0		0	Ewa-RG-art	1		0
1	Ew/a-p-LOST-	1	Ewa-RG-art	1		0
1	Ew/a-p-LOST-	1	Ewa-RG-art	1		0
1	Ew/a-p-LOST-	1	Ewa-RG-art	1		0
1	Ew/a-p-LOST-	1	Ewa-RG-art	1		0
0	#N/A	0		0		0
0	#N/A	0	Ewa-RG-art	1		0
0	#N/A	0	Ewa-RG-art	1		0
0	#N/A	0	Ewa-RG-art	1		0
0		0	Ewa-RG-art	1		0
0		0	Ewa-RG-art	1		0
0		0	Ewa-RG-art	1		0
0	#N/A	0	Ewa-RG-art	1		0
0	#N/A	0	Ewa-RG-art	1		0
0	#N/A	0		0		0

ewargtoppiar	ewargtoppiar	entrycoauinrg	ewauinrgart	ewauinrgart_	ewauinrg_pi_	ewauinrg_pi_	ewargart_au
	0			0		0	
	0		Ew-AU->in-RG	1	Ew-AU->in-RG	1	Ewa-RG-art_E
	0			0		0	
	0			0		0	
	0			0		0	
	0			0		0	
	0			0		0	
	0			0		0	
	0		Ew-AU->in-RG	1	Ew-AU->in-RG	1	Ewa-RG-art_E
	0		Ew-AU->in-RG	1	Ew-AU->in-RG	1	Ewa-RG-art_E
	0		Ew-AU->in-RG	1	Ew-AU->in-RG	1	Ewa-RG-art_E
	0		Ew-AU->in-RG	1	Ew-AU->in-RG	1	Ewa-RG-art_E
	0		Ew-AU->in-RG	1		0	
	0			0		0	
	0		Ew-AU->in-RG	1	Ew-AU->in-RG	1	Ewa-RG-art_E
	0		Ew-AU->in-RG	1	Ew-AU->in-RG	1	Ewa-RG-art_E
	0			0		0	
	0		Ew-AU->in-RG	1	Ew-AU->in-RG	1	Ewa-RG-art_E
	0			0		0	
	0			0		0	
	0			0		0	
	0			0		0	
	0		Ew-AU->in-RG	1	Ew-AU->in-RG	1	Ewa-RG-art_E
	0		Ew-AU->in-RG	1	Ew-AU->in-RG	1	Ewa-RG-art_E
	0		Ew-AU->in-RG	1	Ew-AU->in-RG	1	Ewa-RG-art_E
	0			0		0	
	0		Ew-AU->in-RG	1	Ew-AU->in-RG	1	Ewa-RG-art_E
	0		Ew-AU->in-RG	1	Ew-AU->in-RG	1	Ewa-RG-art_E
	0		Ew-AU->in-RG	1	Ew-AU->in-RG	1	Ewa-RG-art_E
	0		Ew-AU->in-RG	1	Ew-AU->in-RG	1	Ewa-RG-art_E
	0		Ew-AU->in-RG	1		0	Ewa-RG-art_E
	0			0		0	
	0			0		0	
	0		Ew-AU->in-RG	1	Ew-AU->in-RG	1	Ewa-RG-art_E
	0		Ew-AU->in-RG	1	Ew-AU->in-RG	1	Ewa-RG-art_E
	0			0		0	
	0			0		0	
	0		Ew-AU->in-RG	1	Ew-AU->in-RG	1	Ewa-RG-art_E
	0			0		0	
	0		Ew-AU->in-RG	1	Ew-AU->in-RG	1	Ewa-RG-art_E
	0		Ew-AU->in-RG	1	Ew-AU->in-RG	1	Ewa-RG-art_E
	0		Ew-AU->in-RG	1	Ew-AU->in-RG	1	Ewa-RG-art_E
	0		Ew-AU->in-RG	1	Ew-AU->in-RG	1	Ewa-RG-art_E
	0		Ew-AU->in-RG	1	Ew-AU->in-RG	1	Ewa-RG-art_E
	0		Ew-AU->in-RG	1	Ew-AU->in-RG	1	Ewa-RG-art_E
	0			0		0	

	0		0		0	
Ewa-RG-top-P	1	Ew-AU->in-RG	1	Ew-AU->in-RG	1	Ewa-RG-art_E
	0	Ew-AU->in-RG	1	Ew-AU->in-RG	1	Ewa-RG-art_E
	0	Ew-AU->in-RG	1	Ew-AU->in-RG	1	Ewa-RG-art_E
	0	Ew-AU->in-RG	1	Ew-AU->in-RG	1	Ewa-RG-art_E
	0	Ew-AU->in-RG	1	Ew-AU->in-RG	1	Ewa-RG-art_E
	0	Ew-AU->in-RG	1	Ew-AU->in-RG	1	Ewa-RG-art_E
Ewa-RG-top-P	1	Ew-AU->in-RG	1	Ew-AU->in-RG	1	Ewa-RG-art_E
	0	Ew-AU->in-RG	1		0	
	0		0		0	
	0		0		0	
	0	Ew-AU->in-RG	1		0	
Ewa-RG-top-P	1	Ew-AU->in-RG	1	Ew-AU->in-RG	1	Ewa-RG-art_E
	0	Ew-AU->in-RG	1	Ew-AU->in-RG	1	Ewa-RG-art_E
	0	Ew-AU->in-RG	1		0	
	0	Ew-AU->in-RG	1		0	
	0	Ew-AU->in-RG	1		0	
	0		0		0	
	0		0		0	
	0		0		0	
	0	Ew-AU->in-RG	1	Ew-AU->in-RG	1	Ewa-RG-art_E
	0	Ew-AU->in-RG	1	Ew-AU->in-RG	1	Ewa-RG-art_E
	0	Ew-AU->in-RG	1	Ew-AU->in-RG	1	Ewa-RG-art_E
	0		0		0	
	0	Ew-AU->in-RG	1	Ew-AU->in-RG	1	Ewa-RG-art_E
	0	Ew-AU->in-RG	1	Ew-AU->in-RG	1	Ewa-RG-art_E
	0	Ew-AU->in-RG	1	Ew-AU->in-RG	1	Ewa-RG-art_E
	0	Ew-AU->in-RG	1	Ew-AU->in-RG	1	Ewa-RG-art_E
	0	Ew-AU->in-RG	1	Ew-AU->in-RG	1	Ewa-RG-art_E
	0	Ew-AU->in-RG	1	Ew-AU->in-RG	1	Ewa-RG-art_E
	0	Ew-AU->in-RG	1	Ew-AU->in-RG	1	Ewa-RG-art_E
	0	Ew-AU->in-RG	1	Ew-AU->in-RG	1	Ewa-RG-art_E
	0	Ew-AU->in-RG	1	Ew-AU->in-RG	1	Ewa-RG-art_E
	0	Ew-AU->in-RG	1	Ew-AU->in-RG	1	Ewa-RG-art_E
	0		0		0	
	0		0		0	
	0		0		0	
	0		0		0	
	0		0		0	
	0	Ew-AU->in-RG	1	Ew-AU->in-RG	1	Ewa-RG-art_E
	0	Ew-AU->in-RG	1	Ew-AU->in-RG	1	Ewa-RG-art_E
	0	Ew-AU->in-RG	1	Ew-AU->in-RG	1	Ewa-RG-art_E
	0	Ew-AU->in-RG	1	Ew-AU->in-RG	1	Ewa-RG-art_E
	0	Ew-AU->in-RG	1	Ew-AU->in-RG	1	Ewa-RG-art_E
	0		0		0	
	0	Ew-AU->in-RG	1	Ew-AU->in-RG	1	Ewa-RG-art_E
	0	Ew-AU->in-RG	1	Ew-AU->in-RG	1	Ewa-RG-art_E

	0	Ew-AU->in-RG	1	Ew-AU->in-RG	1	Ewa-RG-art_E
	0		0		0	
	0	Ew-AU->in-RG	1		0	Ewa-RG-art_E
	0	Ew-AU->in-RG	1	Ew-AU->in-RG	1	Ewa-RG-art_E
	0	Ew-AU->in-RG	1	Ew-AU->in-RG	1	Ewa-RG-art_E
	0	Ew-AU->in-RG	1		0	Ewa-RG-art_E
Ewa-RG-top-P	1	Ew-AU->in-RG	1	Ew-AU->in-RG	1	Ewa-RG-art_E
Ewa-RG-top-P	1	Ew-AU->in-RG	1	Ew-AU->in-RG	1	Ewa-RG-art_E
	0	Ew-AU->in-RG	1	Ew-AU->in-RG	1	Ewa-RG-art_E
	0		0		0	
	0		0		0	
	0	Ew-AU->in-RG	1	Ew-AU->in-RG	1	Ewa-RG-art_E
	0	Ew-AU->in-RG	1	Ew-AU->in-RG	1	Ewa-RG-art_E
	0	Ew-AU->in-RG	1	Ew-AU->in-RG	1	Ewa-RG-art_E
	0	Ew-AU->in-RG	1		0	
	0	Ew-AU->in-RG	1	Ew-AU->in-RG	1	Ewa-RG-art_E
	0	Ew-AU->in-RG	1	Ew-AU->in-RG	1	Ewa-RG-art_E
	0	Ew-AU->in-RG	1	Ew-AU->in-RG	1	Ewa-RG-art_E
	0	Ew-AU->in-RG	1	Ew-AU->in-RG	1	Ewa-RG-art_E
	0		0		0	
	0	Ew-AU->in-RG	1	Ew-AU->in-RG	1	Ewa-RG-art_E
	0	Ew-AU->in-RG	1	Ew-AU->in-RG	1	Ewa-RG-art_E
	0	Ew-AU->in-RG	1	Ew-AU->in-RG	1	Ewa-RG-art_E
	0		0		0	
	0	Ew-AU->in-RG	1		0	
	0	Ew-AU->in-RG	1	Ew-AU->in-RG	1	Ewa-RG-art_E
	0	Ew-AU->in-RG	1	Ew-AU->in-RG	1	Ewa-RG-art_E
	0	Ew-AU->in-RG	1		0	Ewa-RG-art_E
	0	Ew-AU->in-RG	1		0	Ewa-RG-art_E
	0	Ew-AU->in-RG	1	Ew-AU->in-RG	1	Ewa-RG-art_E
	0	Ew-AU->in-RG	1	Ew-AU->in-RG	1	Ewa-RG-art_E
	0	Ew-AU->in-RG	1	Ew-AU->in-RG	1	Ewa-RG-art_E
	0	Ew-AU->in-RG	1	Ew-AU->in-RG	1	Ewa-RG-art_E
	0	Ew-AU->in-RG	1	Ew-AU->in-RG	1	Ewa-RG-art_E
	0	Ew-AU->in-RG	1	Ew-AU->in-RG	1	Ewa-RG-art_E
	0	Ew-AU->in-RG	1	Ew-AU->in-RG	1	Ewa-RG-art_E
	0	Ew-AU->in-RG	1	Ew-AU->in-RG	1	Ewa-RG-art_E
	0	Ew-AU->in-RG	1	Ew-AU->in-RG	1	Ewa-RG-art_E
	0	Ew-AU->in-RG	1	Ew-AU->in-RG	1	Ewa-RG-art_E
	0	Ew-AU->in-RG	1	Ew-AU->in-RG	1	Ewa-RG-art_E
	0	Ew-AU->in-RG	1	Ew-AU->in-RG	1	Ewa-RG-art_E
	0	Ew-AU->in-RG	1	Ew-AU->in-RG	1	Ewa-RG-art_E
Ewa-RG-top-P	1	Ew-AU->in-RG	1		0	Ewa-RG-art_E
	0		0		0	
	0	Ew-AU->in-RG	1	Ew-AU->in-RG	1	Ewa-RG-art_E
	0	Ew-AU->in-RG	1	Ew-AU->in-RG	1	Ewa-RG-art_E
	0	Ew-AU->in-RG	1	Ew-AU->in-RG	1	Ewa-RG-art_E

	0	Ew-AU->in-RG	1	Ew-AU->in-RG	1	Ewa-RG-art_E
	0	Ew-AU->in-RG	1	Ew-AU->in-RG	1	Ewa-RG-art_E
	0	Ew-AU->in-RG	1	Ew-AU->in-RG	1	Ewa-RG-art_E
	0	Ew-AU->in-RG	1	Ew-AU->in-RG	1	Ewa-RG-art_E
	0	Ew-AU->in-RG	1	Ew-AU->in-RG	1	Ewa-RG-art_E
	0	Ew-AU->in-RG	1		0	
	0	Ew-AU->in-RG	1	Ew-AU->in-RG	1	Ewa-RG-art_E
	0	Ew-AU->in-RG	1	Ew-AU->in-RG	1	Ewa-RG-art_E
	0		0		0	
	0		0		0	
	0		0		0	
	0	Ew-AU->in-RG	1		0	
	0	Ew-AU->in-RG	1		0	
	0	Ew-AU->in-RG	1	Ew-AU->in-RG	1	Ewa-RG-art_E
	0	Ew-AU->in-RG	1		0	Ewa-RG-art_E
	0	Ew-AU->in-RG	1		0	Ewa-RG-art_E
	0	Ew-AU->in-RG	1		0	Ewa-RG-art_E
	0	Ew-AU->in-RG	1	Ew-AU->in-RG	1	Ewa-RG-art_E
	0	Ew-AU->in-RG	1	Ew-AU->in-RG	1	Ewa-RG-art_E
	0	Ew-AU->in-RG	1	Ew-AU->in-RG	1	Ewa-RG-art_E
	0		0		0	
	0		0		0	
	0	Ew-AU->in-RG	1	Ew-AU->in-RG	1	Ewa-RG-art_E
	0		0		0	
	0		0		0	
	0		0		0	
	0	Ew-AU->in-RG	1	Ew-AU->in-RG	1	Ewa-RG-art_E
	0	Ew-AU->in-RG	1	Ew-AU->in-RG	1	Ewa-RG-art_E
	0		0		0	
Ewa-RG-top-P	1	Ew-AU->in-RG	1	Ew-AU->in-RG	1	Ewa-RG-art_E
	0	Ew-AU->in-RG	1	Ew-AU->in-RG	1	Ewa-RG-art_E
	0	Ew-AU->in-RG	1	Ew-AU->in-RG	1	Ewa-RG-art_E
	0	Ew-AU->in-RG	1	Ew-AU->in-RG	1	Ewa-RG-art_E
	0		0		0	
	0	Ew-AU->in-RG	1		0	Ewa-RG-art_E
	0	Ew-AU->in-RG	1		0	Ewa-RG-art_E
	0	Ew-AU->in-RG	1	Ew-AU->in-RG	1	Ewa-RG-art_E
	0	Ew-AU->in-RG	1	Ew-AU->in-RG	1	Ewa-RG-art_E
Ewa-RG-top-P	1	Ew-AU->in-RG	1	Ew-AU->in-RG	1	Ewa-RG-art_E
	0		0		0	
	0	Ew-AU->in-RG	1	Ew-AU->in-RG	1	Ewa-RG-art_E
	0	Ew-AU->in-RG	1	Ew-AU->in-RG	1	Ewa-RG-art_E
	0	Ew-AU->in-RG	1	Ew-AU->in-RG	1	Ewa-RG-art_E
	0		0		0	
	0	Ew-AU->in-RG	1	Ew-AU->in-RG	1	Ewa-RG-art_E
	0	Ew-AU->in-RG	1	Ew-AU->in-RG	1	Ewa-RG-art_E
	0	Ew-AU->in-RG	1	Ew-AU->in-RG	1	Ewa-RG-art_E

	0	Ew-AU->in-RG	1	Ew-AU->in-RG	1	Ewa-RG-art_E
	0	Ew-AU->in-RG	1	Ew-AU->in-RG	1	Ewa-RG-art_E
	0	Ew-AU->in-RG	1	Ew-AU->in-RG	1	Ewa-RG-art_E
	0	Ew-AU->in-RG	1	Ew-AU->in-RG	1	Ewa-RG-art_E
	0	Ew-AU->in-RG	1	Ew-AU->in-RG	1	Ewa-RG-art_E
	0	Ew-AU->in-RG	1	Ew-AU->in-RG	1	Ewa-RG-art_E
	0	Ew-AU->in-RG	1		0	Ewa-RG-art_E
	0	Ew-AU->in-RG	1		0	Ewa-RG-art_E
	0	Ew-AU->in-RG	1		0	Ewa-RG-art_E
	0	Ew-AU->in-RG	1	Ew-AU->in-RG	1	Ewa-RG-art_E
	0	Ew-AU->in-RG	1		0	
	0	Ew-AU->in-RG	1		0	
	0	Ew-AU->in-RG	1	Ew-AU->in-RG	1	Ewa-RG-art_E
	0	Ew-AU->in-RG	1	Ew-AU->in-RG	1	Ewa-RG-art_E
	0	Ew-AU->in-RG	1	Ew-AU->in-RG	1	Ewa-RG-art_E
	0	Ew-AU->in-RG	1	Ew-AU->in-RG	1	Ewa-RG-art_E
	0	Ew-AU->in-RG	1	Ew-AU->in-RG	1	Ewa-RG-art_E
	0	Ew-AU->in-RG	1	Ew-AU->in-RG	1	Ewa-RG-art_E
	0		0		0	
	0	Ew-AU->in-RG	1	Ew-AU->in-RG	1	Ewa-RG-art_E
	0	Ew-AU->in-RG	1	Ew-AU->in-RG	1	Ewa-RG-art_E
	0	Ew-AU->in-RG	1	Ew-AU->in-RG	1	Ewa-RG-art_E
	0	Ew-AU->in-RG	1	Ew-AU->in-RG	1	Ewa-RG-art_E
	0	Ew-AU->in-RG	1	Ew-AU->in-RG	1	Ewa-RG-art_E
Ewa-RG-top-P	1	Ew-AU->in-RG	1	Ew-AU->in-RG	1	Ewa-RG-art_E
Ewa-RG-top-P	1	Ew-AU->in-RG	1	Ew-AU->in-RG	1	Ewa-RG-art_E
	0	Ew-AU->in-RG	1	Ew-AU->in-RG	1	Ewa-RG-art_E
	0	Ew-AU->in-RG	1	Ew-AU->in-RG	1	Ewa-RG-art_E
	0	Ew-AU->in-RG	1	Ew-AU->in-RG	1	Ewa-RG-art_E
	0	Ew-AU->in-RG	1	Ew-AU->in-RG	1	Ewa-RG-art_E
	0	Ew-AU->in-RG	1	Ew-AU->in-RG	1	Ewa-RG-art_E
	0	Ew-AU->in-RG	1		0	
	0	Ew-AU->in-RG	1		0	
	0	Ew-AU->in-RG	1		0	
	0		0		0	
	0		0		0	
	0	Ew-AU->in-RG	1	Ew-AU->in-RG	1	Ewa-RG-art_E
	0	Ew-AU->in-RG	1	Ew-AU->in-RG	1	Ewa-RG-art_E
	0	Ew-AU->in-RG	1	Ew-AU->in-RG	1	Ewa-RG-art_E
	0	Ew-AU->in-RG	1	Ew-AU->in-RG	1	Ewa-RG-art_E
	0	Ew-AU->in-RG	1	Ew-AU->in-RG	1	Ewa-RG-art_E
	0	Ew-AU->in-RG	1	Ew-AU->in-RG	1	Ewa-RG-art_E
	0	Ew-AU->in-RG	1	Ew-AU->in-RG	1	Ewa-RG-art_E
	0	Ew-AU->in-RG	1	Ew-AU->in-RG	1	Ewa-RG-art_E
	0	Ew-AU->in-RG	1	Ew-AU->in-RG	1	Ewa-RG-art_E
	0	Ew-AU->in-RG	1	Ew-AU->in-RG	1	Ewa-RG-art_E
	0	Ew-AU->in-RG	1		0	

	0	Ew-AU->in-RG	1	Ew-AU->in-RG	1	Ewa-RG-art_E
	0	Ew-AU->in-RG	1	Ew-AU->in-RG	1	Ewa-RG-art_E
	0	Ew-AU->in-RG	1	Ew-AU->in-RG	1	Ewa-RG-art_E
	0	Ew-AU->in-RG	1	Ew-AU->in-RG	1	Ewa-RG-art_E
	0	Ew-AU->in-RG	1	Ew-AU->in-RG	1	Ewa-RG-art_E
	0	Ew-AU->in-RG	1	Ew-AU->in-RG	1	Ewa-RG-art_E
	0	Ew-AU->in-RG	1	Ew-AU->in-RG	1	Ewa-RG-art_E
	0	Ew-AU->in-RG	1	Ew-AU->in-RG	1	Ewa-RG-art_E
	0	Ew-AU->in-RG	1	Ew-AU->in-RG	1	Ewa-RG-art_E
Ewa-RG-top-P	1	Ew-AU->in-RG	1	Ew-AU->in-RG	1	Ewa-RG-art_E
	0		0		0	
	0		0		0	
	0	Ew-AU->in-RG	1	Ew-AU->in-RG	1	Ewa-RG-art_E
	0	Ew-AU->in-RG	1	Ew-AU->in-RG	1	Ewa-RG-art_E
	0		0		0	
	0		0		0	
	0	Ew-AU->in-RG	1	Ew-AU->in-RG	1	Ewa-RG-art_E
	0	Ew-AU->in-RG	1	Ew-AU->in-RG	1	Ewa-RG-art_E
	0	Ew-AU->in-RG	1	Ew-AU->in-RG	1	Ewa-RG-art_E
	0	Ew-AU->in-RG	1	Ew-AU->in-RG	1	
	0	Ew-AU->in-RG	1	Ew-AU->in-RG	1	
	0	Ew-AU->in-RG	1	Ew-AU->in-RG	1	Ewa-RG-art_E
	0	Ew-AU->in-RG	1	Ew-AU->in-RG	1	Ewa-RG-art_E
	0	Ew-AU->in-RG	1	Ew-AU->in-RG	1	Ewa-RG-art_E
	0		0		0	
	0		0		0	
	0	Ew-AU->in-RG	1	Ew-AU->in-RG	1	Ewa-RG-art_E
	0	Ew-AU->in-RG	1	Ew-AU->in-RG	1	Ewa-RG-art_E
	0	Ew-AU->in-RG	1	Ew-AU->in-RG	1	
	0	Ew-AU->in-RG	1	Ew-AU->in-RG	1	
	0	Ew-AU->in-RG	1	Ew-AU->in-RG	1	Ewa-RG-art_E
	0	Ew-AU->in-RG	1	Ew-AU->in-RG	1	Ewa-RG-art_E
	0	Ew-AU->in-RG	1	Ew-AU->in-RG	1	Ewa-RG-art_E
	0		0		0	
	0		0		0	
	0	Ew-AU->in-RG	1	Ew-AU->in-RG	1	Ewa-RG-art_E
	0	Ew-AU->in-RG	1	Ew-AU->in-RG	1	Ewa-RG-art_E
	0	Ew-AU->in-RG	1		0	Ewa-RG-art_E
	0	Ew-AU->in-RG	1	Ew-AU->in-RG	1	Ewa-RG-art_E
	0	Ew-AU->in-RG	1	Ew-AU->in-RG	1	Ewa-RG-art_E
	0	Ew-AU->in-RG	1	Ew-AU->in-RG	1	Ewa-RG-art_E
	0	Ew-AU->in-RG	1	Ew-AU->in-RG	1	Ewa-RG-art_E
	0	Ew-AU->in-RG	1	Ew-AU->in-RG	1	Ewa-RG-art_E
	0	Ew-AU->in-RG	1	Ew-AU->in-RG	1	Ewa-RG-art_E
	0		0		0	
	0	Ew-AU->in-RG	1	Ew-AU->in-RG	1	
	0	Ew-AU->in-RG	1	Ew-AU->in-RG	1	Ewa-RG-art_E

Ewa-RG-top-P	1	Ew-AU->in-RG	1	Ew-AU->in-RG	1	Ewa-RG-art_E
Ewa-RG-top-P	1	Ew-AU->in-RG	1	Ew-AU->in-RG	1	Ewa-RG-art_E
	0	Ew-AU->in-RG	1	Ew-AU->in-RG	1	Ewa-RG-art_E
	0		0		0	
	0	Ew-AU->in-RG	1	Ew-AU->in-RG	1	Ewa-RG-art_E
	0	Ew-AU->in-RG	1	Ew-AU->in-RG	1	Ewa-RG-art_E
	0	Ew-AU->in-RG	1	Ew-AU->in-RG	1	Ewa-RG-art_E
	0		0		0	
	0		0		0	
	0	Ew-AU->in-RG	1		0	
	0	Ew-AU->in-RG	1		0	
	0	Ew-AU->in-RG	1	Ew-AU->in-RG	1	Ewa-RG-art_E
	0	Ew-AU->in-RG	1	Ew-AU->in-RG	1	Ewa-RG-art_E
	0	Ew-AU->in-RG	1	Ew-AU->in-RG	1	Ewa-RG-art_E
	0	Ew-AU->in-RG	1	Ew-AU->in-RG	1	Ewa-RG-art_E
	0		0		0	
	0		0		0	
	0	Ew-AU->in-RG	1	Ew-AU->in-RG	1	Ewa-RG-art_E
	0	Ew-AU->in-RG	1	Ew-AU->in-RG	1	Ewa-RG-art_E
	0	Ew-AU->in-RG	1	Ew-AU->in-RG	1	Ewa-RG-art_E
	0		0		0	
	0		0		0	
	0	Ew-AU->in-RG	1	Ew-AU->in-RG	1	Ewa-RG-art_E
	0	Ew-AU->in-RG	1	Ew-AU->in-RG	1	Ewa-RG-art_E
	0	Ew-AU->in-RG	1	Ew-AU->in-RG	1	Ewa-RG-art_E
	0	Ew-AU->in-RG	1	Ew-AU->in-RG	1	Ewa-RG-art_E
	0	Ew-AU->in-RG	1	Ew-AU->in-RG	1	Ewa-RG-art_E
	0	Ew-AU->in-RG	1	Ew-AU->in-RG	1	Ewa-RG-art_E
	0	Ew-AU->in-RG	1	Ew-AU->in-RG	1	Ewa-RG-art_E
	0	Ew-AU->in-RG	1	Ew-AU->in-RG	1	Ewa-RG-art_E
	0		0		0	
	0		0		0	
	0	Ew-AU->in-RG	1	Ew-AU->in-RG	1	Ewa-RG-art_E
	0		0		0	
	0		0		0	
	0		0		0	
	0	Ew-AU->in-RG	1		0	Ewa-RG-art_E
	0	Ew-AU->in-RG	1		0	
	0	Ew-AU->in-RG	1	Ew-AU->in-RG	1	Ewa-RG-art_E
	0	Ew-AU->in-RG	1	Ew-AU->in-RG	1	Ewa-RG-art_E
	0	Ew-AU->in-RG	1		0	
	0	Ew-AU->in-RG	1		0	Ewa-RG-art_E
	0	Ew-AU->in-RG	1		0	Ewa-RG-art_E
	0	Ew-AU->in-RG	1	Ew-AU->in-RG	1	Ewa-RG-art_E
	0	Ew-AU->in-RG	1	Ew-AU->in-RG	1	Ewa-RG-art_E
	0	Ew-AU->in-RG	1		0	
	0	Ew-AU->in-RG	1		0	

0		0		0	
0	Ew-AU->in-RG	1	Ew-AU->in-RG	1	Ewa-RG-art_E
0	Ew-AU->in-RG	1	Ew-AU->in-RG	1	Ewa-RG-art_E
0	Ew-AU->in-RG	1		0	
0		0		0	
0	Ew-AU->in-RG	1	Ew-AU->in-RG	1	Ewa-RG-art_E
0	Ew-AU->in-RG	1	Ew-AU->in-RG	1	Ewa-RG-art_E
0	Ew-AU->in-RG	1	Ew-AU->in-RG	1	Ewa-RG-art_E
0	Ew-AU->in-RG	1	Ew-AU->in-RG	1	Ewa-RG-art_E
0		0		0	
0		0		0	
0	Ew-AU->in-RG	1	Ew-AU->in-RG	1	Ewa-RG-art_E
0	Ew-AU->in-RG	1	Ew-AU->in-RG	1	Ewa-RG-art_E
0	Ew-AU->in-RG	1	Ew-AU->in-RG	1	Ewa-RG-art_E
0	Ew-AU->in-RG	1	Ew-AU->in-RG	1	Ewa-RG-art_E
0	Ew-AU->in-RG	1	Ew-AU->in-RG	1	Ewa-RG-art_E
0		0		0	
0	Ew-AU->in-RG	1	Ew-AU->in-RG	1	Ewa-RG-art_E
0	Ew-AU->in-RG	1	Ew-AU->in-RG	1	Ewa-RG-art_E
0	Ew-AU->in-RG	1		0	
0	Ew-AU->in-RG	1	Ew-AU->in-RG	1	Ewa-RG-art_E
0		0		0	
0		0		0	
0		0		0	
0	Ew-AU->in-RG	1	Ew-AU->in-RG	1	Ewa-RG-art_E
0	Ew-AU->in-RG	1	Ew-AU->in-RG	1	Ewa-RG-art_E
0	Ew-AU->in-RG	1		0	Ewa-RG-art_E
0	Ew-AU->in-RG	1		0	
0		0		0	
0	Ew-AU->in-RG	1	Ew-AU->in-RG	1	Ewa-RG-art_E
0		0		0	
0	Ew-AU->in-RG	1	Ew-AU->in-RG	1	Ewa-RG-art_E
0	Ew-AU->in-RG	1	Ew-AU->in-RG	1	Ewa-RG-art_E
0	Ew-AU->in-RG	1	Ew-AU->in-RG	1	Ewa-RG-art_E
0		0		0	
0		0		0	
0		0		0	
0		0		0	
0	Ew-AU->in-RG	1	Ew-AU->in-RG	1	Ewa-RG-art_E
0		0		0	
0	Ew-AU->in-RG	1	Ew-AU->in-RG	1	Ewa-RG-art_E
0	Ew-AU->in-RG	1	Ew-AU->in-RG	1	Ewa-RG-art_E
0	Ew-AU->in-RG	1	Ew-AU->in-RG	1	Ewa-RG-art_E
0		0		0	
0		0		0	
0	Ew-AU->in-RG	1	Ew-AU->in-RG	1	Ewa-RG-art_E
0	Ew-AU->in-RG	1	Ew-AU->in-RG	1	Ewa-RG-art_E

	0		0		0	
	0		0		0	
	0	Ew-AU->in-RG	1	Ew-AU->in-RG	1	Ewa-RG-art_E
	0	Ew-AU->in-RG	1	Ew-AU->in-RG	1	Ewa-RG-art_E
	0	Ew-AU->in-RG	1	Ew-AU->in-RG	1	Ewa-RG-art_E
	0	Ew-AU->in-RG	1		0	Ewa-RG-art_E
	0	Ew-AU->in-RG	1		0	Ewa-RG-art_E
	0	Ew-AU->in-RG	1	Ew-AU->in-RG	1	Ewa-RG-art_E
	0	Ew-AU->in-RG	1	Ew-AU->in-RG	1	Ewa-RG-art_E
	0	Ew-AU->in-RG	1	Ew-AU->in-RG	1	Ewa-RG-art_E
	0	Ew-AU->in-RG	1	Ew-AU->in-RG	1	Ewa-RG-art_E
	0	Ew-AU->in-RG	1	Ew-AU->in-RG	1	Ewa-RG-art_E
	0	Ew-AU->in-RG	1	Ew-AU->in-RG	1	Ewa-RG-art_E
	0	Ew-AU->in-RG	1		0	
	0		0		0	
	0	Ew-AU->in-RG	1	Ew-AU->in-RG	1	Ewa-RG-art_E
	0	Ew-AU->in-RG	1	Ew-AU->in-RG	1	Ewa-RG-art_E
	0	Ew-AU->in-RG	1	Ew-AU->in-RG	1	Ewa-RG-art_E
	0	Ew-AU->in-RG	1	Ew-AU->in-RG	1	Ewa-RG-art_E
	0	Ew-AU->in-RG	1		0	
	0	Ew-AU->in-RG	1		0	
	0	Ew-AU->in-RG	1	Ew-AU->in-RG	1	Ewa-RG-art_E
	0		0		0	
	0		0		0	
	0	Ew-AU->in-RG	1	Ew-AU->in-RG	1	
	0	Ew-AU->in-RG	1	Ew-AU->in-RG	1	
	0	Ew-AU->in-RG	1	Ew-AU->in-RG	1	Ewa-RG-art_E
	0	Ew-AU->in-RG	1	Ew-AU->in-RG	1	Ewa-RG-art_E
	0	Ew-AU->in-RG	1	Ew-AU->in-RG	1	Ewa-RG-art_E
	0	Ew-AU->in-RG	1	Ew-AU->in-RG	1	Ewa-RG-art_E
	0		0		0	
	0	Ew-AU->in-RG	1	Ew-AU->in-RG	1	Ewa-RG-art_E
	0	Ew-AU->in-RG	1	Ew-AU->in-RG	1	Ewa-RG-art_E
	0	Ew-AU->in-RG	1		0	
	0	Ew-AU->in-RG	1	Ew-AU->in-RG	1	Ewa-RG-art_E
	0	Ew-AU->in-RG	1	Ew-AU->in-RG	1	Ewa-RG-art_E
	0	Ew-AU->in-RG	1	Ew-AU->in-RG	1	Ewa-RG-art_E
Ewa-RG-top-P	1	Ew-AU->in-RG	1	Ew-AU->in-RG	1	Ewa-RG-art_E
	0		0		0	
	0	Ew-AU->in-RG	1	Ew-AU->in-RG	1	Ewa-RG-art_E
	0	Ew-AU->in-RG	1	Ew-AU->in-RG	1	Ewa-RG-art_E
	0		0		0	
	0	Ew-AU->in-RG	1	Ew-AU->in-RG	1	Ewa-RG-art_E
	0	Ew-AU->in-RG	1	Ew-AU->in-RG	1	Ewa-RG-art_E
	0	Ew-AU->in-RG	1	Ew-AU->in-RG	1	Ewa-RG-art_E
	0		0		0	
	0		0		0	

	0		0		0	
	0	Ew-AU->in-RG	1		0	
	0	Ew-AU->in-RG	1		0	
	0		0		0	
	0		0		0	
	0		0		0	
	0		0		0	
	0	Ew-AU->in-RG	1	Ew-AU->in-RG	1	Ewa-RG-art_E
Ewa-RG-top-P	1	Ew-AU->in-RG	1	Ew-AU->in-RG	1	Ewa-RG-art_E
	0	Ew-AU->in-RG	1	Ew-AU->in-RG	1	Ewa-RG-art_E
	0	Ew-AU->in-RG	1	Ew-AU->in-RG	1	Ewa-RG-art_E
	0		0		0	
	0		0		0	
	0		0		0	
	0		0		0	
	0	Ew-AU->in-RG	1	Ew-AU->in-RG	1	Ewa-RG-art_E
	0	Ew-AU->in-RG	1	Ew-AU->in-RG	1	Ewa-RG-art_E
	0	Ew-AU->in-RG	1	Ew-AU->in-RG	1	Ewa-RG-art_E
	0	Ew-AU->in-RG	1	Ew-AU->in-RG	1	Ewa-RG-art_E
	0	Ew-AU->in-RG	1	Ew-AU->in-RG	1	Ewa-RG-art_E
	0	Ew-AU->in-RG	1	Ew-AU->in-RG	1	Ewa-RG-art_E
	0	Ew-AU->in-RG	1		0	Ewa-RG-art_E
	0	Ew-AU->in-RG	1		0	Ewa-RG-art_E
	0	Ew-AU->in-RG	1		0	Ewa-RG-art_E
	0		0		0	
	0		0		0	
	0		0		0	
	0		0		0	
	0		0		0	
	0	Ew-AU->in-RG	1	Ew-AU->in-RG	1	Ewa-RG-art_E
	0	Ew-AU->in-RG	1	Ew-AU->in-RG	1	Ewa-RG-art_E
	0		0		0	
	0	Ew-AU->in-RG	1	Ew-AU->in-RG	1	Ewa-RG-art_E
	0	Ew-AU->in-RG	1	Ew-AU->in-RG	1	Ewa-RG-art_E
	0	Ew-AU->in-RG	1	Ew-AU->in-RG	1	Ewa-RG-art_E
	0		0		0	
	0	Ew-AU->in-RG	1	Ew-AU->in-RG	1	
	0	Ew-AU->in-RG	1	Ew-AU->in-RG	1	
	0	Ew-AU->in-RG	1	Ew-AU->in-RG	1	Ewa-RG-art_E
	0	Ew-AU->in-RG	1	Ew-AU->in-RG	1	
	0	Ew-AU->in-RG	1	Ew-AU->in-RG	1	
	0	Ew-AU->in-RG	1	Ew-AU->in-RG	1	Ewa-RG-art_E
	0	Ew-AU->in-RG	1	Ew-AU->in-RG	1	Ewa-RG-art_E
	0	Ew-AU->in-RG	1	Ew-AU->in-RG	1	Ewa-RG-art_E
	0	Ew-AU->in-RG	1	Ew-AU->in-RG	1	Ewa-RG-art_E
	0	Ew-AU->in-RG	1	Ew-AU->in-RG	1	Ewa-RG-art_E
	0	Ew-AU->in-RG	1	Ew-AU->in-RG	1	Ewa-RG-art_E

0	Ew-AU->in-RG	1		0	Ewa-RG-art_E
0	Ew-AU->in-RG	1		0	Ewa-RG-art_E
0	Ew-AU->in-RG	1	Ew-AU->in-RG	1	Ewa-RG-art_E
0	Ew-AU->in-RG	1	Ew-AU->in-RG	1	Ewa-RG-art_E
0	Ew-AU->in-RG	1	Ew-AU->in-RG	1	Ewa-RG-art_E
0		0		0	
0		0		0	
0		0		0	
0	Ew-AU->in-RG	1		0	
0	Ew-AU->in-RG	1	Ew-AU->in-RG	1	Ewa-RG-art_E
0	Ew-AU->in-RG	1	Ew-AU->in-RG	1	Ewa-RG-art_E
0	Ew-AU->in-RG	1	Ew-AU->in-RG	1	Ewa-RG-art_E
0	Ew-AU->in-RG	1	Ew-AU->in-RG	1	Ewa-RG-art_E
0	Ew-AU->in-RG	1	Ew-AU->in-RG	1	Ewa-RG-art_E
0	Ew-AU->in-RG	1	Ew-AU->in-RG	1	Ewa-RG-art_E
0	Ew-AU->in-RG	1	Ew-AU->in-RG	1	Ewa-RG-art_E
0	Ew-AU->in-RG	1	Ew-AU->in-RG	1	Ewa-RG-art_E
0	Ew-AU->in-RG	1	Ew-AU->in-RG	1	Ewa-RG-art_E
0	Ew-AU->in-RG	1	Ew-AU->in-RG	1	Ewa-RG-art_E
0	Ew-AU->in-RG	1	Ew-AU->in-RG	1	Ewa-RG-art_E
0	Ew-AU->in-RG	1	Ew-AU->in-RG	1	Ewa-RG-art_E
0	Ew-AU->in-RG	1	Ew-AU->in-RG	1	Ewa-RG-art_E
0		0		0	
0	Ew-AU->in-RG	1		0	Ewa-RG-art_E
0	Ew-AU->in-RG	1		0	Ewa-RG-art_E
0	Ew-AU->in-RG	1		0	Ewa-RG-art_E
0	Ew-AU->in-RG	1		0	Ewa-RG-art_E
0	Ew-AU->in-RG	1		0	Ewa-RG-art_E
0	Ew-AU->in-RG	1	Ew-AU->in-RG	1	Ewa-RG-art_E
0	Ew-AU->in-RG	1	Ew-AU->in-RG	1	Ewa-RG-art_E
0	Ew-AU->in-RG	1	Ew-AU->in-RG	1	Ewa-RG-art_E
0	Ew-AU->in-RG	1	Ew-AU->in-RG	1	Ewa-RG-art_E
0		0		0	
0		0		0	
0	Ew-AU->in-RG	1	Ew-AU->in-RG	1	Ewa-RG-art_E
0		0		0	
0	Ew-AU->in-RG	1	Ew-AU->in-RG	1	Ewa-RG-art_E
0	Ew-AU->in-RG	1	Ew-AU->in-RG	1	Ewa-RG-art_E
0	Ew-AU->in-RG	1		0	
0	Ew-AU->in-RG	1		0	
0	Ew-AU->in-RG	1	Ew-AU->in-RG	1	Ewa-RG-art_E
0	Ew-AU->in-RG	1		0	Ewa-RG-art_E
0	Ew-AU->in-RG	1	Ew-AU->in-RG	1	Ewa-RG-art_E
0	Ew-AU->in-RG	1	Ew-AU->in-RG	1	Ewa-RG-art_E
0		0		0	
0	Ew-AU->in-RG	1	Ew-AU->in-RG	1	Ewa-RG-art_E
0	Ew-AU->in-RG	1	Ew-AU->in-RG	1	Ewa-RG-art_E

0		0		0	
0	Ew-AU->in-RG	1		0	
0		0		0	
0	Ew-AU->in-RG	1	Ew-AU->in-RG	1	Ewa-RG-art_E
0	Ew-AU->in-RG	1	Ew-AU->in-RG	1	Ewa-RG-art_E
0		0		0	
0		0		0	
0	Ew-AU->in-RG	1	Ew-AU->in-RG	1	Ewa-RG-art_E
0	Ew-AU->in-RG	1	Ew-AU->in-RG	1	Ewa-RG-art_E
0	Ew-AU->in-RG	1	Ew-AU->in-RG	1	Ewa-RG-art_E
0	Ew-AU->in-RG	1		0	Ewa-RG-art_E
0	Ew-AU->in-RG	1		0	Ewa-RG-art_E
0	Ew-AU->in-RG	1		0	Ewa-RG-art_E
0		0		0	
0	Ew-AU->in-RG	1	Ew-AU->in-RG	1	Ewa-RG-art_E
0	Ew-AU->in-RG	1	Ew-AU->in-RG	1	Ewa-RG-art_E
0	Ew-AU->in-RG	1	Ew-AU->in-RG	1	Ewa-RG-art_E
0	Ew-AU->in-RG	1	Ew-AU->in-RG	1	Ewa-RG-art_E
0		0		0	
0		0		0	
0	Ew-AU->in-RG	1	Ew-AU->in-RG	1	Ewa-RG-art_E
0	Ew-AU->in-RG	1	Ew-AU->in-RG	1	Ewa-RG-art_E
0	Ew-AU->in-RG	1	Ew-AU->in-RG	1	Ewa-RG-art_E
0	Ew-AU->in-RG	1	Ew-AU->in-RG	1	Ewa-RG-art_E
0	Ew-AU->in-RG	1		0	Ewa-RG-art_E
0	Ew-AU->in-RG	1		0	Ewa-RG-art_E
0	Ew-AU->in-RG	1	Ew-AU->in-RG	1	Ewa-RG-art_E
0	Ew-AU->in-RG	1	Ew-AU->in-RG	1	Ewa-RG-art_E
0	Ew-AU->in-RG	1	Ew-AU->in-RG	1	Ewa-RG-art_E
0		0		0	
0	Ew-AU->in-RG	1	Ew-AU->in-RG	1	Ewa-RG-art_E
0	Ew-AU->in-RG	1	Ew-AU->in-RG	1	Ewa-RG-art_E
0	Ew-AU->in-RG	1	Ew-AU->in-RG	1	Ewa-RG-art_E
0	Ew-AU->in-RG	1	Ew-AU->in-RG	1	Ewa-RG-art_E
0	Ew-AU->in-RG	1	Ew-AU->in-RG	1	Ewa-RG-art_E
0		0		0	
0		0		0	
0	Ew-AU->in-RG	1	Ew-AU->in-RG	1	Ewa-RG-art_E
0	Ew-AU->in-RG	1	Ew-AU->in-RG	1	Ewa-RG-art_E
0	Ew-AU->in-RG	1	Ew-AU->in-RG	1	Ewa-RG-art_E
0	Ew-AU->in-RG	1		0	Ewa-RG-art_E
0	Ew-AU->in-RG	1		0	Ewa-RG-art_E
0	Ew-AU->in-RG	1		0	
0	Ew-AU->in-RG	1	Ew-AU->in-RG	1	Ewa-RG-art_E
0	Ew-AU->in-RG	1	Ew-AU->in-RG	1	Ewa-RG-art_E
0	Ew-AU->in-RG	1	Ew-AU->in-RG	1	Ewa-RG-art_E
0	Ew-AU->in-RG	1		0	Ewa-RG-art_E

0	Ew-AU->in-RG	1	Ew-AU->in-RG	1	Ewa-RG-art_E
0	Ew-AU->in-RG	1	Ew-AU->in-RG	1	Ewa-RG-art_E
0	Ew-AU->in-RG	1	Ew-AU->in-RG	1	Ewa-RG-art_E
0	Ew-AU->in-RG	1	Ew-AU->in-RG	1	Ewa-RG-art_E
0	Ew-AU->in-RG	1	Ew-AU->in-RG	1	Ewa-RG-art_E
0	Ew-AU->in-RG	1	Ew-AU->in-RG	1	Ewa-RG-art_E
0	Ew-AU->in-RG	1	Ew-AU->in-RG	1	Ewa-RG-art_E
0		0		0	
0		0		0	
0		0		0	
0	Ew-AU->in-RG	1	Ew-AU->in-RG	1	Ewa-RG-art_E
0	Ew-AU->in-RG	1	Ew-AU->in-RG	1	Ewa-RG-art_E
0	Ew-AU->in-RG	1	Ew-AU->in-RG	1	Ewa-RG-art_E
0	Ew-AU->in-RG	1	Ew-AU->in-RG	1	Ewa-RG-art_E
0	Ew-AU->in-RG	1	Ew-AU->in-RG	1	Ewa-RG-art_E
0	Ew-AU->in-RG	1	Ew-AU->in-RG	1	Ewa-RG-art_E
0	Ew-AU->in-RG	1	Ew-AU->in-RG	1	Ewa-RG-art_E
0	Ew-AU->in-RG	1	Ew-AU->in-RG	1	Ewa-RG-art_E
0	Ew-AU->in-RG	1	Ew-AU->in-RG	1	Ewa-RG-art_E
0	Ew-AU->in-RG	1	Ew-AU->in-RG	1	Ewa-RG-art_E
0	Ew-AU->in-RG	1	Ew-AU->in-RG	1	Ewa-RG-art_E
0	Ew-AU->in-RG	1	Ew-AU->in-RG	1	Ewa-RG-art_E
0	Ew-AU->in-RG	1	Ew-AU->in-RG	1	Ewa-RG-art_E
0	Ew-AU->in-RG	1	Ew-AU->in-RG	1	Ewa-RG-art_E
0		0		0	
0	Ew-AU->in-RG	1	Ew-AU->in-RG	1	Ewa-RG-art_E
0		0		0	
0	Ew-AU->in-RG	1	Ew-AU->in-RG	1	Ewa-RG-art_E
0	Ew-AU->in-RG	1	Ew-AU->in-RG	1	Ewa-RG-art_E
0	Ew-AU->in-RG	1	Ew-AU->in-RG	1	Ewa-RG-art_E
0	Ew-AU->in-RG	1	Ew-AU->in-RG	1	Ewa-RG-art_E
0	Ew-AU->in-RG	1	Ew-AU->in-RG	1	Ewa-RG-art_E
0	Ew-AU->in-RG	1	Ew-AU->in-RG	1	Ewa-RG-art_E
0	Ew-AU->in-RG	1	Ew-AU->in-RG	1	Ewa-RG-art_E
0	Ew-AU->in-RG	1		0	Ewa-RG-art_E
0	Ew-AU->in-RG	1		0	Ewa-RG-art_E
0	Ew-AU->in-RG	1	Ew-AU->in-RG	1	Ewa-RG-art_E
0	Ew-AU->in-RG	1	Ew-AU->in-RG	1	Ewa-RG-art_E
0	Ew-AU->in-RG	1	Ew-AU->in-RG	1	Ewa-RG-art_E
0		0		0	
0	Ew-AU->in-RG	1		0	
0	Ew-AU->in-RG	1	Ew-AU->in-RG	1	Ewa-RG-art_E
0	Ew-AU->in-RG	1		0	Ewa-RG-art_E
0	Ew-AU->in-RG	1		0	Ewa-RG-art_E
0		0		0	
0	Ew-AU->in-RG	1	Ew-AU->in-RG	1	Ewa-RG-art_E
0		0		0	

0	Ew-AU->in-RG	1	Ew-AU->in-RG	1	Ewa-RG-art_E
0		0		0	
0	Ew-AU->in-RG	1	Ew-AU->in-RG	1	Ewa-RG-art_E
0	Ew-AU->in-RG	1	Ew-AU->in-RG	1	Ewa-RG-art_E
0	Ew-AU->in-RG	1	Ew-AU->in-RG	1	Ewa-RG-art_E
0	Ew-AU->in-RG	1	Ew-AU->in-RG	1	Ewa-RG-art_E
0	Ew-AU->in-RG	1	Ew-AU->in-RG	1	Ewa-RG-art_E
0	Ew-AU->in-RG	1	Ew-AU->in-RG	1	Ewa-RG-art_E
0	Ew-AU->in-RG	1	Ew-AU->in-RG	1	Ewa-RG-art_E
0	Ew-AU->in-RG	1	Ew-AU->in-RG	1	Ewa-RG-art_E
0	Ew-AU->in-RG	1		0	
0	Ew-AU->in-RG	1		0	
0	Ew-AU->in-RG	1	Ew-AU->in-RG	1	Ewa-RG-art_E
0	Ew-AU->in-RG	1	Ew-AU->in-RG	1	Ewa-RG-art_E
0		0		0	
0	Ew-AU->in-RG	1		0	Ewa-RG-art_E
0	Ew-AU->in-RG	1		0	Ewa-RG-art_E
0		0		0	
0		0		0	
0	Ew-AU->in-RG	1		0	Ewa-RG-art_E
0	Ew-AU->in-RG	1		0	Ewa-RG-art_E
0	Ew-AU->in-RG	1	Ew-AU->in-RG	1	Ewa-RG-art_E
0	Ew-AU->in-RG	1	Ew-AU->in-RG	1	Ewa-RG-art_E
0	Ew-AU->in-RG	1	Ew-AU->in-RG	1	Ewa-RG-art_E
0	Ew-AU->in-RG	1	Ew-AU->in-RG	1	Ewa-RG-art_E
0	Ew-AU->in-RG	1	Ew-AU->in-RG	1	Ewa-RG-art_E
0	Ew-AU->in-RG	1		0	
0	Ew-AU->in-RG	1		0	
0	Ew-AU->in-RG	1	Ew-AU->in-RG	1	Ewa-RG-art_E
0		0		0	
0	Ew-AU->in-RG	1	Ew-AU->in-RG	1	Ewa-RG-art_E
0	Ew-AU->in-RG	1	Ew-AU->in-RG	1	Ewa-RG-art_E
0	Ew-AU->in-RG	1	Ew-AU->in-RG	1	Ewa-RG-art_E
0		0		0	
0		0		0	
0	Ew-AU->in-RG	1	Ew-AU->in-RG	1	Ewa-RG-art_E
0	Ew-AU->in-RG	1	Ew-AU->in-RG	1	Ewa-RG-art_E
0	Ew-AU->in-RG	1	Ew-AU->in-RG	1	Ewa-RG-art_E
0	Ew-AU->in-RG	1		0	Ewa-RG-art_E
0	Ew-AU->in-RG	1		0	Ewa-RG-art_E
0	Ew-AU->in-RG	1		0	Ewa-RG-art_E
0		0		0	
0	Ew-AU->in-RG	1	Ew-AU->in-RG	1	Ewa-RG-art_E
0		0		0	
0		0		0	
0	Ew-AU->in-RG	1	Ew-AU->in-RG	1	Ewa-RG-art_E
0		0		0	

	0		0		0	
	0	Ew-AU->in-RG	1	Ew-AU->in-RG	1	Ewa-RG-art_E
	0	Ew-AU->in-RG	1	Ew-AU->in-RG	1	Ewa-RG-art_E
	0	Ew-AU->in-RG	1	Ew-AU->in-RG	1	Ewa-RG-art_E
	0	Ew-AU->in-RG	1	Ew-AU->in-RG	1	Ewa-RG-art_E
	0		0		0	
	0	Ew-AU->in-RG	1	Ew-AU->in-RG	1	Ewa-RG-art_E
	0	Ew-AU->in-RG	1	Ew-AU->in-RG	1	Ewa-RG-art_E
	0	Ew-AU->in-RG	1	Ew-AU->in-RG	1	Ewa-RG-art_E
	0	Ew-AU->in-RG	1	Ew-AU->in-RG	1	Ewa-RG-art_E
	0	Ew-AU->in-RG	1	Ew-AU->in-RG	1	Ewa-RG-art_E
	0	Ew-AU->in-RG	1	Ew-AU->in-RG	1	Ewa-RG-art_E
	0	Ew-AU->in-RG	1	Ew-AU->in-RG	1	Ewa-RG-art_E
	0		0		0	
	0	Ew-AU->in-RG	1	Ew-AU->in-RG	1	Ewa-RG-art_E
	0	Ew-AU->in-RG	1	Ew-AU->in-RG	1	
	0		0		0	
	0		0		0	
	0	Ew-AU->in-RG	1	Ew-AU->in-RG	1	Ewa-RG-art_E
	0	Ew-AU->in-RG	1	Ew-AU->in-RG	1	Ewa-RG-art_E
	0	Ew-AU->in-RG	1	Ew-AU->in-RG	1	Ewa-RG-art_E
	0	Ew-AU->in-RG	1	Ew-AU->in-RG	1	Ewa-RG-art_E
	0		0		0	
	0		0		0	
	0	Ew-AU->in-RG	1	Ew-AU->in-RG	1	Ewa-RG-art_E
	0	Ew-AU->in-RG	1	Ew-AU->in-RG	1	Ewa-RG-art_E
	0	Ew-AU->in-RG	1	Ew-AU->in-RG	1	Ewa-RG-art_E
	0	Ew-AU->in-RG	1	Ew-AU->in-RG	1	Ewa-RG-art_E
	0	Ew-AU->in-RG	1	Ew-AU->in-RG	1	Ewa-RG-art_E
	0	Ew-AU->in-RG	1	Ew-AU->in-RG	1	Ewa-RG-art_E
Ewa-RG-top-F	1	Ew-AU->in-RG	1		0	Ewa-RG-art_E
Ewa-RG-top-F	1	Ew-AU->in-RG	1		0	Ewa-RG-art_E
	0		0		0	
	0	Ew-AU->in-RG	1	Ew-AU->in-RG	1	Ewa-RG-art_E
	0	Ew-AU->in-RG	1	Ew-AU->in-RG	1	Ewa-RG-art_E
	0	Ew-AU->in-RG	1	Ew-AU->in-RG	1	Ewa-RG-art_E
	0	Ew-AU->in-RG	1	Ew-AU->in-RG	1	Ewa-RG-art_E
	0		0		0	
	0		0		0	
	0	Ew-AU->in-RG	1	Ew-AU->in-RG	1	Ewa-RG-art_E
	0		0		0	
	0		0		0	
	0		0		0	
	0		0		0	
	0	Ew-AU->in-RG	1	Ew-AU->in-RG	1	Ewa-RG-art_E
	0	Ew-AU->in-RG	1	Ew-AU->in-RG	1	Ewa-RG-art_E
	0		0		0	

	0	Ew-AU->in-RG	1	Ew-AU->in-RG	1	Ewa-RG-art_E
	0	Ew-AU->in-RG	1	Ew-AU->in-RG	1	Ewa-RG-art_E
	0	Ew-AU->in-RG	1	Ew-AU->in-RG	1	Ewa-RG-art_E
	0	Ew-AU->in-RG	1	Ew-AU->in-RG	1	Ewa-RG-art_E
	0	Ew-AU->in-RG	1	Ew-AU->in-RG	1	Ewa-RG-art_E
	0	Ew-AU->in-RG	1	Ew-AU->in-RG	1	Ewa-RG-art_E
	0	Ew-AU->in-RG	1	Ew-AU->in-RG	1	Ewa-RG-art_E
	0	Ew-AU->in-RG	1	Ew-AU->in-RG	1	Ewa-RG-art_E
	0	Ew-AU->in-RG	1	Ew-AU->in-RG	1	Ewa-RG-art_E
	0		0		0	
	0	Ew-AU->in-RG	1	Ew-AU->in-RG	1	Ewa-RG-art_E
	0	Ew-AU->in-RG	1	Ew-AU->in-RG	1	Ewa-RG-art_E
	0	Ew-AU->in-RG	1	Ew-AU->in-RG	1	Ewa-RG-art_E
	0		0		0	
	0		0		0	
	0	Ew-AU->in-RG	1		0	
	0		0		0	
	0		0		0	
	0	Ew-AU->in-RG	1		0	Ewa-RG-art_E
	0	Ew-AU->in-RG	1	Ew-AU->in-RG	1	Ewa-RG-art_E
	0	Ew-AU->in-RG	1	Ew-AU->in-RG	1	Ewa-RG-art_E
	0	Ew-AU->in-RG	1	Ew-AU->in-RG	1	Ewa-RG-art_E
	0	Ew-AU->in-RG	1	Ew-AU->in-RG	1	Ewa-RG-art_E
	0		0		0	
	0	Ew-AU->in-RG	1	Ew-AU->in-RG	1	Ewa-RG-art_E
	0	Ew-AU->in-RG	1	Ew-AU->in-RG	1	Ewa-RG-art_E
	0	Ew-AU->in-RG	1		0	Ewa-RG-art_E
	0	Ew-AU->in-RG	1		0	Ewa-RG-art_E
	0	Ew-AU->in-RG	1	Ew-AU->in-RG	1	Ewa-RG-art_E
Ewa-RG-top-P	1	Ew-AU->in-RG	1	Ew-AU->in-RG	1	Ewa-RG-art_E
Ewa-RG-top-P	1	Ew-AU->in-RG	1	Ew-AU->in-RG	1	Ewa-RG-art_E
	0	Ew-AU->in-RG	1		0	
	0	Ew-AU->in-RG	1		0	
	0	Ew-AU->in-RG	1		0	Ewa-RG-art_E
	0	Ew-AU->in-RG	1		0	Ewa-RG-art_E
	0		0		0	
	0		0		0	
	0		0		0	
	0	Ew-AU->in-RG	1	Ew-AU->in-RG	1	Ewa-RG-art_E
	0	Ew-AU->in-RG	1	Ew-AU->in-RG	1	Ewa-RG-art_E
	0	Ew-AU->in-RG	1	Ew-AU->in-RG	1	Ewa-RG-art_E
	0	Ew-AU->in-RG	1	Ew-AU->in-RG	1	Ewa-RG-art_E
	0	Ew-AU->in-RG	1	Ew-AU->in-RG	1	Ewa-RG-art_E
	0	Ew-AU->in-RG	1	Ew-AU->in-RG	1	Ewa-RG-art_E
	0		0		0	
	0	Ew-AU->in-RG	1	Ew-AU->in-RG	1	Ewa-RG-art_E
	0		0		0	

Ewa-RG-top-P	1		0		0	
	0		0		0	
	0		0		0	
	0	Ew-AU->in-RG	1	Ew-AU->in-RG	1	Ewa-RG-art_E
	0	Ew-AU->in-RG	1		0	Ewa-RG-art_E
	0	Ew-AU->in-RG	1		0	Ewa-RG-art_E
	0	Ew-AU->in-RG	1		0	Ewa-RG-art_E
Ewa-RG-top-P	1		0		0	
Ewa-RG-top-P	1		0		0	
	0	Ew-AU->in-RG	1	Ew-AU->in-RG	1	
	0	Ew-AU->in-RG	1	Ew-AU->in-RG	1	
	0	Ew-AU->in-RG	1		0	Ewa-RG-art_E
	0	Ew-AU->in-RG	1		0	Ewa-RG-art_E
	0		0		0	
	0		0		0	
	0	Ew-AU->in-RG	1		0	Ewa-RG-art_E
	0	Ew-AU->in-RG	1		0	Ewa-RG-art_E
	0	Ew-AU->in-RG	1	Ew-AU->in-RG	1	Ewa-RG-art_E
	0	Ew-AU->in-RG	1	Ew-AU->in-RG	1	Ewa-RG-art_E
	0	Ew-AU->in-RG	1	Ew-AU->in-RG	1	Ewa-RG-art_E
	0		0		0	
	0		0		0	
	0		0		0	
	0		0		0	
	0	Ew-AU->in-RG	1	Ew-AU->in-RG	1	Ewa-RG-art_E
	0	Ew-AU->in-RG	1		0	Ewa-RG-art_E
	0	Ew-AU->in-RG	1		0	Ewa-RG-art_E
	0	Ew-AU->in-RG	1	Ew-AU->in-RG	1	Ewa-RG-art_E
	0	Ew-AU->in-RG	1	Ew-AU->in-RG	1	Ewa-RG-art_E
	0	Ew-AU->in-RG	1	Ew-AU->in-RG	1	Ewa-RG-art_E
	0	Ew-AU->in-RG	1	Ew-AU->in-RG	1	Ewa-RG-art_E
	0	Ew-AU->in-RG	1	Ew-AU->in-RG	1	Ewa-RG-art_E
	0	Ew-AU->in-RG	1	Ew-AU->in-RG	1	Ewa-RG-art_E
	0	Ew-AU->in-RG	1	Ew-AU->in-RG	1	Ewa-RG-art_E
	0	Ew-AU->in-RG	1		0	Ewa-RG-art_E
	0	Ew-AU->in-RG	1		0	Ewa-RG-art_E
	0	Ew-AU->in-RG	1		0	Ewa-RG-art_E
	0		0		0	
	0	Ew-AU->in-RG	1	Ew-AU->in-RG	1	Ewa-RG-art_E
	0		0		0	
	0		0		0	
	0	Ew-AU->in-RG	1	Ew-AU->in-RG	1	Ewa-RG-art_E
	0	Ew-AU->in-RG	1	Ew-AU->in-RG	1	Ewa-RG-art_E
	0	Ew-AU->in-RG	1	Ew-AU->in-RG	1	Ewa-RG-art_E
	0	Ew-AU->in-RG	1		0	
	0	Ew-AU->in-RG	1		0	
	0		0		0	

	0		0		0	
	0	Ew-AU->in-RG	1	Ew-AU->in-RG	1	Ewa-RG-art_E
	0	Ew-AU->in-RG	1	Ew-AU->in-RG	1	Ewa-RG-art_E
	0	Ew-AU->in-RG	1	Ew-AU->in-RG	1	Ewa-RG-art_E
	0	Ew-AU->in-RG	1	Ew-AU->in-RG	1	Ewa-RG-art_E
	0	Ew-AU->in-RG	1	Ew-AU->in-RG	1	Ewa-RG-art_E
	0	Ew-AU->in-RG	1	Ew-AU->in-RG	1	Ewa-RG-art_E
	0	Ew-AU->in-RG	1	Ew-AU->in-RG	1	Ewa-RG-art_E
	0	Ew-AU->in-RG	1	Ew-AU->in-RG	1	Ewa-RG-art_E
	0		0		0	
	0		0		0	
	0		0		0	
	0	Ew-AU->in-RG	1		0	
	0	Ew-AU->in-RG	1		0	
	0	Ew-AU->in-RG	1	Ew-AU->in-RG	1	Ewa-RG-art_E
	0		0		0	
	0		0		0	
	0		0		0	
	0		0		0	
	0	Ew-AU->in-RG	1	Ew-AU->in-RG	1	Ewa-RG-art_E
	0	Ew-AU->in-RG	1	Ew-AU->in-RG	1	Ewa-RG-art_E
	0	Ew-AU->in-RG	1	Ew-AU->in-RG	1	Ewa-RG-art_E
	0	Ew-AU->in-RG	1	Ew-AU->in-RG	1	Ewa-RG-art_E
	0	Ew-AU->in-RG	1	Ew-AU->in-RG	1	Ewa-RG-art_E
	0		0		0	
	0	Ew-AU->in-RG	1		0	
	0	Ew-AU->in-RG	1	Ew-AU->in-RG	1	Ewa-RG-art_E
	0	Ew-AU->in-RG	1	Ew-AU->in-RG	1	Ewa-RG-art_E
	0	Ew-AU->in-RG	1	Ew-AU->in-RG	1	Ewa-RG-art_E
	0	Ew-AU->in-RG	1	Ew-AU->in-RG	1	Ewa-RG-art_E
	0		0		0	
Ewa-RG-top-P	1	Ew-AU->in-RG	1	Ew-AU->in-RG	1	Ewa-RG-art_E
	0	Ew-AU->in-RG	1	Ew-AU->in-RG	1	Ewa-RG-art_E
	0	Ew-AU->in-RG	1	Ew-AU->in-RG	1	Ewa-RG-art_E
	0	Ew-AU->in-RG	1	Ew-AU->in-RG	1	Ewa-RG-art_E
	0	Ew-AU->in-RG	1	Ew-AU->in-RG	1	Ewa-RG-art_E
	0	Ew-AU->in-RG	1	Ew-AU->in-RG	1	Ewa-RG-art_E
	0	Ew-AU->in-RG	1	Ew-AU->in-RG	1	Ewa-RG-art_E
	0	Ew-AU->in-RG	1	Ew-AU->in-RG	1	Ewa-RG-art_E
	0	Ew-AU->in-RG	1		0	Ewa-RG-art_E
	0	Ew-AU->in-RG	1		0	Ewa-RG-art_E
	0	Ew-AU->in-RG	1	Ew-AU->in-RG	1	Ewa-RG-art_E
	0	Ew-AU->in-RG	1	Ew-AU->in-RG	1	Ewa-RG-art_E
	0	Ew-AU->in-RG	1		0	Ewa-RG-art_E
	0	Ew-AU->in-RG	1		0	Ewa-RG-art_E
	0	Ew-AU->in-RG	1	Ew-AU->in-RG	1	Ewa-RG-art_E
	0	Ew-AU->in-RG	1	Ew-AU->in-RG	1	Ewa-RG-art_E

	0	Ew-AU->in-RG	1	Ew-AU->in-RG	1	Ewa-RG-art_E
	0	Ew-AU->in-RG	1	Ew-AU->in-RG	1	Ewa-RG-art_E
	0	Ew-AU->in-RG	1	Ew-AU->in-RG	1	Ewa-RG-art_E
	0	Ew-AU->in-RG	1	Ew-AU->in-RG	1	Ewa-RG-art_E
	0		0		0	
	0	Ew-AU->in-RG	1	Ew-AU->in-RG	1	Ewa-RG-art_E
	0		0		0	
	0	Ew-AU->in-RG	1		0	
	0		0		0	
	0		0		0	
	0	Ew-AU->in-RG	1	Ew-AU->in-RG	1	Ewa-RG-art_E
	0	Ew-AU->in-RG	1	Ew-AU->in-RG	1	Ewa-RG-art_E
	0	Ew-AU->in-RG	1	Ew-AU->in-RG	1	Ewa-RG-art_E
	0	Ew-AU->in-RG	1	Ew-AU->in-RG	1	Ewa-RG-art_E
	0	Ew-AU->in-RG	1	Ew-AU->in-RG	1	Ewa-RG-art_E
	0		0		0	
	0	Ew-AU->in-RG	1	Ew-AU->in-RG	1	Ewa-RG-art_E
	0	Ew-AU->in-RG	1	Ew-AU->in-RG	1	Ewa-RG-art_E
	0	Ew-AU->in-RG	1	Ew-AU->in-RG	1	Ewa-RG-art_E
	0		0		0	
	0	Ew-AU->in-RG	1	Ew-AU->in-RG	1	Ewa-RG-art_E
	0	Ew-AU->in-RG	1	Ew-AU->in-RG	1	Ewa-RG-art_E
	0	Ew-AU->in-RG	1	Ew-AU->in-RG	1	Ewa-RG-art_E
	0	Ew-AU->in-RG	1	Ew-AU->in-RG	1	Ewa-RG-art_E
	0		0		0	
	0	Ew-AU->in-RG	1	Ew-AU->in-RG	1	Ewa-RG-art_E
	0	Ew-AU->in-RG	1	Ew-AU->in-RG	1	Ewa-RG-art_E
	0		0		0	
	0	Ew-AU->in-RG	1	Ew-AU->in-RG	1	Ewa-RG-art_E
Ewa-RG-top-P	1	Ew-AU->in-RG	1	Ew-AU->in-RG	1	Ewa-RG-art_E
Ewa-RG-top-P	1	Ew-AU->in-RG	1	Ew-AU->in-RG	1	Ewa-RG-art_E
	0		0		0	
	0	Ew-AU->in-RG	1		0	Ewa-RG-art_E
	0	Ew-AU->in-RG	1		0	Ewa-RG-art_E
	0	Ew-AU->in-RG	1	Ew-AU->in-RG	1	Ewa-RG-art_E
	0		0		0	
	0	Ew-AU->in-RG	1	Ew-AU->in-RG	1	Ewa-RG-art_E
	0		0		0	
	0	Ew-AU->in-RG	1	Ew-AU->in-RG	1	Ewa-RG-art_E
	0	Ew-AU->in-RG	1		0	Ewa-RG-art_E
	0	Ew-AU->in-RG	1		0	Ewa-RG-art_E
	0	Ew-AU->in-RG	1	Ew-AU->in-RG	1	Ewa-RG-art_E
	0	Ew-AU->in-RG	1	Ew-AU->in-RG	1	Ewa-RG-art_E
	0		0		0	
	0		0		0	
	0		0		0	
	0		0		0	

	0		0		0	
	0		0		0	
	0	Ew-AU->in-RG	1		0	Ewa-RG-art_E
	0	Ew-AU->in-RG	1		0	
	0	Ew-AU->in-RG	1		0	
	0	Ew-AU->in-RG	1		0	Ewa-RG-art_E
	0		0		0	
	0		0		0	
	0		0		0	
	0	Ew-AU->in-RG	1	Ew-AU->in-RG	1	Ewa-RG-art_E
	0		0		0	
Ewa-RG-top-P	1	Ew-AU->in-RG	1	Ew-AU->in-RG	1	Ewa-RG-art_E
Ewa-RG-top-P	1	Ew-AU->in-RG	1	Ew-AU->in-RG	1	Ewa-RG-art_E
Ewa-RG-top-P	1	Ew-AU->in-RG	1	Ew-AU->in-RG	1	Ewa-RG-art_E
	0		0		0	
	0	Ew-AU->in-RG	1	Ew-AU->in-RG	1	Ewa-RG-art_E
	0	Ew-AU->in-RG	1	Ew-AU->in-RG	1	Ewa-RG-art_E
	0	Ew-AU->in-RG	1	Ew-AU->in-RG	1	Ewa-RG-art_E
	0	Ew-AU->in-RG	1		0	Ewa-RG-art_E
	0	Ew-AU->in-RG	1		0	
	0		0		0	
	0		0		0	
	0	Ew-AU->in-RG	1	Ew-AU->in-RG	1	Ewa-RG-art_E
	0	Ew-AU->in-RG	1	Ew-AU->in-RG	1	Ewa-RG-art_E
	0	Ew-AU->in-RG	1	Ew-AU->in-RG	1	Ewa-RG-art_E
	0	Ew-AU->in-RG	1	Ew-AU->in-RG	1	Ewa-RG-art_E
	0	Ew-AU->in-RG	1	Ew-AU->in-RG	1	Ewa-RG-art_E
	0		0		0	
	0	Ew-AU->in-RG	1	Ew-AU->in-RG	1	Ewa-RG-art_E
	0		0		0	
	0		0		0	
	0	Ew-AU->in-RG	1	Ew-AU->in-RG	1	Ewa-RG-art_E
	0	Ew-AU->in-RG	1	Ew-AU->in-RG	1	Ewa-RG-art_E
	0		0		0	
	0	Ew-AU->in-RG	1		0	
	0	Ew-AU->in-RG	1	Ew-AU->in-RG	1	
	0	Ew-AU->in-RG	1	Ew-AU->in-RG	1	Ewa-RG-art_E
	0	Ew-AU->in-RG	1	Ew-AU->in-RG	1	Ewa-RG-art_E
	0	Ew-AU->in-RG	1	Ew-AU->in-RG	1	Ewa-RG-art_E
	0	Ew-AU->in-RG	1	Ew-AU->in-RG	1	Ewa-RG-art_E
	0		0		0	
	0	Ew-AU->in-RG	1	Ew-AU->in-RG	1	Ewa-RG-art_E
	0	Ew-AU->in-RG	1		0	
	0		0		0	
	0	Ew-AU->in-RG	1		0	Ewa-RG-art_E
	0	Ew-AU->in-RG	1		0	Ewa-RG-art_E
	0	Ew-AU->in-RG	1	Ew-AU->in-RG	1	Ewa-RG-art_E

	0	Ew-AU->in-RG	1	Ew-AU->in-RG	1	Ewa-RG-art_E
	0		0		0	
	0	Ew-AU->in-RG	1		0	
	0	Ew-AU->in-RG	1	Ew-AU->in-RG	1	Ewa-RG-art_E
	0	Ew-AU->in-RG	1	Ew-AU->in-RG	1	Ewa-RG-art_E
	0		0		0	
	0	Ew-AU->in-RG	1	Ew-AU->in-RG	1	Ewa-RG-art_E
	0		0		0	
	0	Ew-AU->in-RG	1	Ew-AU->in-RG	1	Ewa-RG-art_E
	0	Ew-AU->in-RG	1	Ew-AU->in-RG	1	Ewa-RG-art_E
	0	Ew-AU->in-RG	1	Ew-AU->in-RG	1	Ewa-RG-art_E
	0	Ew-AU->in-RG	1	Ew-AU->in-RG	1	Ewa-RG-art_E
	0	Ew-AU->in-RG	1	Ew-AU->in-RG	1	Ewa-RG-art_E
Ewa-RG-top-P	1	Ew-AU->in-RG	1	Ew-AU->in-RG	1	Ewa-RG-art_E
Ewa-RG-top-P	1	Ew-AU->in-RG	1	Ew-AU->in-RG	1	Ewa-RG-art_E
	0	Ew-AU->in-RG	1	Ew-AU->in-RG	1	Ewa-RG-art_E
	0	Ew-AU->in-RG	1		0	Ewa-RG-art_E
	0		0		0	
	0	Ew-AU->in-RG	1		0	Ewa-RG-art_E
	0	Ew-AU->in-RG	1		0	Ewa-RG-art_E
	0	Ew-AU->in-RG	1	Ew-AU->in-RG	1	Ewa-RG-art_E
	0	Ew-AU->in-RG	1	Ew-AU->in-RG	1	Ewa-RG-art_E
	0		0		0	
	0		0		0	
	0		0		0	
	0	Ew-AU->in-RG	1	Ew-AU->in-RG	1	Ewa-RG-art_E
	0	Ew-AU->in-RG	1	Ew-AU->in-RG	1	Ewa-RG-art_E
	0	Ew-AU->in-RG	1	Ew-AU->in-RG	1	Ewa-RG-art_E
	0	Ew-AU->in-RG	1	Ew-AU->in-RG	1	Ewa-RG-art_E
	0	Ew-AU->in-RG	1	Ew-AU->in-RG	1	Ewa-RG-art_E
	0	Ew-AU->in-RG	1	Ew-AU->in-RG	1	Ewa-RG-art_E
	0	Ew-AU->in-RG	1	Ew-AU->in-RG	1	Ewa-RG-art_E
	0	Ew-AU->in-RG	1		0	Ewa-RG-art_E
	0		0		0	
	0		0		0	
	0	Ew-AU->in-RG	1	Ew-AU->in-RG	1	Ewa-RG-art_E
	0	Ew-AU->in-RG	1	Ew-AU->in-RG	1	Ewa-RG-art_E
	0		0		0	
	0	Ew-AU->in-RG	1	Ew-AU->in-RG	1	Ewa-RG-art_E
	0		0		0	
	0	Ew-AU->in-RG	1	Ew-AU->in-RG	1	Ewa-RG-art_E
	0		0		0	
	0		0		0	
	0	Ew-AU->in-RG	1	Ew-AU->in-RG	1	Ewa-RG-art_E
	0	Ew-AU->in-RG	1	Ew-AU->in-RG	1	Ewa-RG-art_E
	0	Ew-AU->in-RG	1	Ew-AU->in-RG	1	Ewa-RG-art_E
	0	Ew-AU->in-RG	1	Ew-AU->in-RG	1	Ewa-RG-art_E

	0	Ew-AU->in-RG	1	Ew-AU->in-RG	1	Ewa-RG-art_E
	0	Ew-AU->in-RG	1	Ew-AU->in-RG	1	Ewa-RG-art_E
	0	Ew-AU->in-RG	1	Ew-AU->in-RG	1	Ewa-RG-art_E
	0		0		0	
Ewa-RG-top-P	1	Ew-AU->in-RG	1	Ew-AU->in-RG	1	Ewa-RG-art_E
Ewa-RG-top-P	1	Ew-AU->in-RG	1	Ew-AU->in-RG	1	Ewa-RG-art_E
Ewa-RG-top-P	1	Ew-AU->in-RG	1	Ew-AU->in-RG	1	Ewa-RG-art_E
	0	Ew-AU->in-RG	1	Ew-AU->in-RG	1	Ewa-RG-art_E
	0	Ew-AU->in-RG	1	Ew-AU->in-RG	1	Ewa-RG-art_E
	0	Ew-AU->in-RG	1	Ew-AU->in-RG	1	Ewa-RG-art_E
	0	Ew-AU->in-RG	1	Ew-AU->in-RG	1	Ewa-RG-art_E
	0	Ew-AU->in-RG	1	Ew-AU->in-RG	1	Ewa-RG-art_E
	0	Ew-AU->in-RG	1	Ew-AU->in-RG	1	Ewa-RG-art_E
	0	Ew-AU->in-RG	1	Ew-AU->in-RG	1	Ewa-RG-art_E
	0	Ew-AU->in-RG	1	Ew-AU->in-RG	1	Ewa-RG-art_E
	0	Ew-AU->in-RG	1	Ew-AU->in-RG	1	Ewa-RG-art_E
	0	Ew-AU->in-RG	1	Ew-AU->in-RG	1	Ewa-RG-art_E
	0	Ew-AU->in-RG	1	Ew-AU->in-RG	1	Ewa-RG-art_E
	0		0		0	
	0	Ew-AU->in-RG	1	Ew-AU->in-RG	1	Ewa-RG-art_E
	0	Ew-AU->in-RG	1	Ew-AU->in-RG	1	Ewa-RG-art_E
	0	Ew-AU->in-RG	1	Ew-AU->in-RG	1	Ewa-RG-art_E
	0	Ew-AU->in-RG	1	Ew-AU->in-RG	1	Ewa-RG-art_E
	0	Ew-AU->in-RG	1	Ew-AU->in-RG	1	Ewa-RG-art_E
	0	Ew-AU->in-RG	1	Ew-AU->in-RG	1	Ewa-RG-art_E
	0	Ew-AU->in-RG	1	Ew-AU->in-RG	1	Ewa-RG-art_E
	0	Ew-AU->in-RG	1	Ew-AU->in-RG	1	Ewa-RG-art_E
	0	Ew-AU->in-RG	1	Ew-AU->in-RG	1	Ewa-RG-art_E
	0		0		0	
	0	Ew-AU->in-RG	1	Ew-AU->in-RG	1	Ewa-RG-art_E
	0	Ew-AU->in-RG	1		0	Ewa-RG-art_E
	0	Ew-AU->in-RG	1		0	Ewa-RG-art_E
	0	Ew-AU->in-RG	1		0	Ewa-RG-art_E
	0	Ew-AU->in-RG	1		0	Ewa-RG-art_E
	0	Ew-AU->in-RG	1	Ew-AU->in-RG	1	Ewa-RG-art_E
	0	Ew-AU->in-RG	1	Ew-AU->in-RG	1	Ewa-RG-art_E
	0	Ew-AU->in-RG	1	Ew-AU->in-RG	1	Ewa-RG-art_E
	0		0		0	
	0	Ew-AU->in-RG	1	Ew-AU->in-RG	1	Ewa-RG-art_E
	0	Ew-AU->in-RG	1	Ew-AU->in-RG	1	Ewa-RG-art_E
	0	Ew-AU->in-RG	1	Ew-AU->in-RG	1	Ewa-RG-art_E
	0	Ew-AU->in-RG	1	Ew-AU->in-RG	1	Ewa-RG-art_E
	0	Ew-AU->in-RG	1	Ew-AU->in-RG	1	Ewa-RG-art_E
	0	Ew-AU->in-RG	1	Ew-AU->in-RG	1	Ewa-RG-art_E
	0		0		0	
	0	Ew-AU->in-RG	1		0	Ewa-RG-art_E
	0	Ew-AU->in-RG	1		0	Ewa-RG-art_E

	0		0		0	
	0		0		0	
	0		0		0	
	0		0		0	
	0	Ew-AU->in-RG	1	Ew-AU->in-RG	1	Ewa-RG-art_E
	0	Ew-AU->in-RG	1	Ew-AU->in-RG	1	Ewa-RG-art_E
	0	Ew-AU->in-RG	1	Ew-AU->in-RG	1	Ewa-RG-art_E
	0	Ew-AU->in-RG	1	Ew-AU->in-RG	1	Ewa-RG-art_E
	0	Ew-AU->in-RG	1	Ew-AU->in-RG	1	Ewa-RG-art_E
Ewa-RG-top-P	1	Ew-AU->in-RG	1	Ew-AU->in-RG	1	Ewa-RG-art_E
	0	Ew-AU->in-RG	1	Ew-AU->in-RG	1	Ewa-RG-art_E
	0	Ew-AU->in-RG	1	Ew-AU->in-RG	1	Ewa-RG-art_E
	0	Ew-AU->in-RG	1	Ew-AU->in-RG	1	Ewa-RG-art_E
	0	Ew-AU->in-RG	1	Ew-AU->in-RG	1	Ewa-RG-art_E
	0	Ew-AU->in-RG	1	Ew-AU->in-RG	1	Ewa-RG-art_E
	0	Ew-AU->in-RG	1	Ew-AU->in-RG	1	Ewa-RG-art_E
	0	Ew-AU->in-RG	1	Ew-AU->in-RG	1	Ewa-RG-art_E
	0		0		0	
	0	Ew-AU->in-RG	1	Ew-AU->in-RG	1	Ewa-RG-art_E
	0	Ew-AU->in-RG	1	Ew-AU->in-RG	1	Ewa-RG-art_E
	0	Ew-AU->in-RG	1	Ew-AU->in-RG	1	Ewa-RG-art_E
	0	Ew-AU->in-RG	1		0	Ewa-RG-art_E
	0	Ew-AU->in-RG	1		0	Ewa-RG-art_E
	0	Ew-AU->in-RG	1		0	Ewa-RG-art_E
	0		0		0	
	0	Ew-AU->in-RG	1	Ew-AU->in-RG	1	Ewa-RG-art_E
	0		0		0	
	0		0		0	
	0		0		0	
	0		0		0	
	0	Ew-AU->in-RG	1	Ew-AU->in-RG	1	Ewa-RG-art_E
	0	Ew-AU->in-RG	1		0	Ewa-RG-art_E
	0	Ew-AU->in-RG	1		0	Ewa-RG-art_E
	0	Ew-AU->in-RG	1		0	Ewa-RG-art_E
	0	Ew-AU->in-RG	1	Ew-AU->in-RG	1	Ewa-RG-art_E
	0	Ew-AU->in-RG	1	Ew-AU->in-RG	1	Ewa-RG-art_E
	0		0		0	
	0	Ew-AU->in-RG	1	Ew-AU->in-RG	1	Ewa-RG-art_E
	0	Ew-AU->in-RG	1	Ew-AU->in-RG	1	Ewa-RG-art_E
	0	Ew-AU->in-RG	1		0	
	0		0		0	
	0	Ew-AU->in-RG	1	Ew-AU->in-RG	1	Ewa-RG-art_E
	0	Ew-AU->in-RG	1	Ew-AU->in-RG	1	Ewa-RG-art_E
	0		0		0	
	0	Ew-AU->in-RG	1	Ew-AU->in-RG	1	Ewa-RG-art_E
	0	Ew-AU->in-RG	1		0	Ewa-RG-art_E
	0	Ew-AU->in-RG	1	Ew-AU->in-RG	1	Ewa-RG-art_E

0		0		0	
0		0		0	
0		0		0	
0		0		0	
0	Ew-AU->in-RG	1		0	
0	Ew-AU->in-RG	1	Ew-AU->in-RG	1	Ewa-RG-art_E
0	Ew-AU->in-RG	1	Ew-AU->in-RG	1	Ewa-RG-art_E
0	Ew-AU->in-RG	1	Ew-AU->in-RG	1	Ewa-RG-art_E
0	Ew-AU->in-RG	1	Ew-AU->in-RG	1	Ewa-RG-art_E
0	Ew-AU->in-RG	1	Ew-AU->in-RG	1	Ewa-RG-art_E
0	Ew-AU->in-RG	1	Ew-AU->in-RG	1	Ewa-RG-art_E
0	Ew-AU->in-RG	1	Ew-AU->in-RG	1	Ewa-RG-art_E
0		0		0	
0	Ew-AU->in-RG	1		0	
0		0		0	
0		0		0	
0	Ew-AU->in-RG	1	Ew-AU->in-RG	1	Ewa-RG-art_E
0	Ew-AU->in-RG	1	Ew-AU->in-RG	1	Ewa-RG-art_E
0	Ew-AU->in-RG	1	Ew-AU->in-RG	1	Ewa-RG-art_E
0	Ew-AU->in-RG	1	Ew-AU->in-RG	1	Ewa-RG-art_E
0	Ew-AU->in-RG	1	Ew-AU->in-RG	1	Ewa-RG-art_E
0	Ew-AU->in-RG	1	Ew-AU->in-RG	1	Ewa-RG-art_E
0	Ew-AU->in-RG	1	Ew-AU->in-RG	1	Ewa-RG-art_E
0	Ew-AU->in-RG	1	Ew-AU->in-RG	1	Ewa-RG-art_E
0	Ew-AU->in-RG	1	Ew-AU->in-RG	1	Ewa-RG-art_E
0	Ew-AU->in-RG	1	Ew-AU->in-RG	1	Ewa-RG-art_E
0	Ew-AU->in-RG	1	Ew-AU->in-RG	1	Ewa-RG-art_E
0		0		0	
0	Ew-AU->in-RG	1	Ew-AU->in-RG	1	Ewa-RG-art_E
0	Ew-AU->in-RG	1	Ew-AU->in-RG	1	Ewa-RG-art_E
0		0		0	
0	Ew-AU->in-RG	1	Ew-AU->in-RG	1	Ewa-RG-art_E
0	Ew-AU->in-RG	1		0	
0	Ew-AU->in-RG	1		0	
0	Ew-AU->in-RG	1	Ew-AU->in-RG	1	Ewa-RG-art_E
0	Ew-AU->in-RG	1	Ew-AU->in-RG	1	Ewa-RG-art_E
0		0		0	
0	Ew-AU->in-RG	1	Ew-AU->in-RG	1	Ewa-RG-art_E
0	Ew-AU->in-RG	1	Ew-AU->in-RG	1	Ewa-RG-art_E
0	Ew-AU->in-RG	1	Ew-AU->in-RG	1	Ewa-RG-art_E
0	Ew-AU->in-RG	1	Ew-AU->in-RG	1	Ewa-RG-art_E
0	Ew-AU->in-RG	1		0	
0	Ew-AU->in-RG	1	Ew-AU->in-RG	1	Ewa-RG-art_E
0		0		0	
0		0		0	
0	Ew-AU->in-RG	1	Ew-AU->in-RG	1	Ewa-RG-art_E
0		0		0	

	0	Ew-AU->in-RG	1	Ew-AU->in-RG	1	Ewa-RG-art_E
	0	Ew-AU->in-RG	1	Ew-AU->in-RG	1	Ewa-RG-art_E
	0	Ew-AU->in-RG	1	Ew-AU->in-RG	1	Ewa-RG-art_E
	0		0		0	
	0	Ew-AU->in-RG	1	Ew-AU->in-RG	1	Ewa-RG-art_E
	0	Ew-AU->in-RG	1	Ew-AU->in-RG	1	Ewa-RG-art_E
	0	Ew-AU->in-RG	1	Ew-AU->in-RG	1	Ewa-RG-art_E
	0	Ew-AU->in-RG	1	Ew-AU->in-RG	1	Ewa-RG-art_E
	0		0		0	
	0	Ew-AU->in-RG	1	Ew-AU->in-RG	1	Ewa-RG-art_E
	0	Ew-AU->in-RG	1	Ew-AU->in-RG	1	Ewa-RG-art_E
	0	Ew-AU->in-RG	1	Ew-AU->in-RG	1	Ewa-RG-art_E
	0		0		0	
	0		0		0	
	0	Ew-AU->in-RG	1		0	Ewa-RG-art_E
	0	Ew-AU->in-RG	1		0	Ewa-RG-art_E
	0		0		0	
	0		0		0	
	0	Ew-AU->in-RG	1	Ew-AU->in-RG	1	Ewa-RG-art_E
	0	Ew-AU->in-RG	1	Ew-AU->in-RG	1	Ewa-RG-art_E
	0		0		0	
	0	Ew-AU->in-RG	1		0	Ewa-RG-art_E
	0	Ew-AU->in-RG	1		0	Ewa-RG-art_E
	0	Ew-AU->in-RG	1		0	Ewa-RG-art_E
	0	Ew-AU->in-RG	1		0	Ewa-RG-art_E
	0	Ew-AU->in-RG	1	Ew-AU->in-RG	1	Ewa-RG-art_E
	0		0		0	
	0		0		0	
	0		0		0	
	0		0		0	
	0	Ew-AU->in-RG	1	Ew-AU->in-RG	1	Ewa-RG-art_E
	0	Ew-AU->in-RG	1	Ew-AU->in-RG	1	Ewa-RG-art_E
	0	Ew-AU->in-RG	1	Ew-AU->in-RG	1	Ewa-RG-art_E
	0	Ew-AU->in-RG	1	Ew-AU->in-RG	1	Ewa-RG-art_E
	0		0		0	
	0		0		0	
	0		0		0	
	0		0		0	
	0		0		0	
	0		0		0	
	0		0		0	
	0	Ew-AU->in-RG	1		0	Ewa-RG-art_E
	0	Ew-AU->in-RG	1		0	Ewa-RG-art_E
	0	Ew-AU->in-RG	1	Ew-AU->in-RG	1	Ewa-RG-art_E
	0	Ew-AU->in-RG	1	Ew-AU->in-RG	1	Ewa-RG-art_E
	0	Ew-AU->in-RG	1		0	
Ewa-RG-top-P	1	Ew-AU->in-RG	1	Ew-AU->in-RG	1	Ewa-RG-art_E

Ewa-RG-top-F	1	Ew-AU->in-RG	1	Ew-AU->in-RG	1	Ewa-RG-art_E
	0		0		0	
	0	Ew-AU->in-RG	1		0	Ewa-RG-art_E
	0	Ew-AU->in-RG	1		0	Ewa-RG-art_E
	0	Ew-AU->in-RG	1		0	
	0	Ew-AU->in-RG	1		0	
	0	Ew-AU->in-RG	1		0	Ewa-RG-art_E
	0	Ew-AU->in-RG	1		0	Ewa-RG-art_E
	0	Ew-AU->in-RG	1	Ew-AU->in-RG	1	Ewa-RG-art_E
	0	Ew-AU->in-RG	1	Ew-AU->in-RG	1	Ewa-RG-art_E
	0		0		0	
	0		0		0	
	0		0		0	
	0	Ew-AU->in-RG	1	Ew-AU->in-RG	1	Ewa-RG-art_E
	0	Ew-AU->in-RG	1	Ew-AU->in-RG	1	Ewa-RG-art_E
	0	Ew-AU->in-RG	1	Ew-AU->in-RG	1	Ewa-RG-art_E
	0	Ew-AU->in-RG	1	Ew-AU->in-RG	1	Ewa-RG-art_E
	0	Ew-AU->in-RG	1	Ew-AU->in-RG	1	Ewa-RG-art_E
	0	Ew-AU->in-RG	1	Ew-AU->in-RG	1	Ewa-RG-art_E
	0	Ew-AU->in-RG	1	Ew-AU->in-RG	1	Ewa-RG-art_E
	0		0		0	
	0		0		0	
	0	Ew-AU->in-RG	1	Ew-AU->in-RG	1	Ewa-RG-art_E
	0	Ew-AU->in-RG	1	Ew-AU->in-RG	1	Ewa-RG-art_E
	0	Ew-AU->in-RG	1	Ew-AU->in-RG	1	Ewa-RG-art_E
	0	Ew-AU->in-RG	1	Ew-AU->in-RG	1	Ewa-RG-art_E
	0		0		0	
	0	Ew-AU->in-RG	1	Ew-AU->in-RG	1	Ewa-RG-art_E
	0	Ew-AU->in-RG	1	Ew-AU->in-RG	1	Ewa-RG-art_E
	0	Ew-AU->in-RG	1	Ew-AU->in-RG	1	Ewa-RG-art_E
	0		0		0	
	0		0		0	
	0	Ew-AU->in-RG	1		0	
	0	Ew-AU->in-RG	1	Ew-AU->in-RG	1	Ewa-RG-art_E
	0		0		0	
	0	Ew-AU->in-RG	1	Ew-AU->in-RG	1	Ewa-RG-art_E
	0	Ew-AU->in-RG	1	Ew-AU->in-RG	1	Ewa-RG-art_E
	0	Ew-AU->in-RG	1		0	
	0		0		0	
	0	Ew-AU->in-RG	1	Ew-AU->in-RG	1	Ewa-RG-art_E
	0	Ew-AU->in-RG	1	Ew-AU->in-RG	1	Ewa-RG-art_E
	0	Ew-AU->in-RG	1	Ew-AU->in-RG	1	Ewa-RG-art_E
	0	Ew-AU->in-RG	1	Ew-AU->in-RG	1	Ewa-RG-art_E
	0	Ew-AU->in-RG	1	Ew-AU->in-RG	1	Ewa-RG-art_E
	0	Ew-AU->in-RG	1	Ew-AU->in-RG	1	Ewa-RG-art_E
	0	Ew-AU->in-RG	1	Ew-AU->in-RG	1	
	0	Ew-AU->in-RG	1	Ew-AU->in-RG	1	

0	Ew-AU->in-RG	1 Ew-AU->in-RG		1 Ewa-RG-art_E
0		0		0
0		0		0
0	Ew-AU->in-RG	1 Ew-AU->in-RG		1 Ewa-RG-art_E
0	Ew-AU->in-RG	1 Ew-AU->in-RG		1 Ewa-RG-art_E
0	Ew-AU->in-RG	1		0 Ewa-RG-art_E
0	Ew-AU->in-RG	1		0 Ewa-RG-art_E
0	Ew-AU->in-RG	1 Ew-AU->in-RG		1 Ewa-RG-art_E
0	Ew-AU->in-RG	1 Ew-AU->in-RG		1 Ewa-RG-art_E
0		0		0
0	Ew-AU->in-RG	1 Ew-AU->in-RG		1 Ewa-RG-art_E
0	Ew-AU->in-RG	1 Ew-AU->in-RG		1 Ewa-RG-art_E
0	Ew-AU->in-RG	1		0
0	Ew-AU->in-RG	1		0 Ewa-RG-art_E
0	Ew-AU->in-RG	1		0 Ewa-RG-art_E
0	Ew-AU->in-RG	1 Ew-AU->in-RG		1 Ewa-RG-art_E
0	Ew-AU->in-RG	1 Ew-AU->in-RG		1 Ewa-RG-art_E
0	Ew-AU->in-RG	1 Ew-AU->in-RG		1 Ewa-RG-art_E
0	Ew-AU->in-RG	1 Ew-AU->in-RG		1 Ewa-RG-art_E
0	Ew-AU->in-RG	1 Ew-AU->in-RG		1 Ewa-RG-art_E
0	Ew-AU->in-RG	1		0
0	Ew-AU->in-RG	1 Ew-AU->in-RG		1 Ewa-RG-art_E
0		0		0
0	Ew-AU->in-RG	1 Ew-AU->in-RG		1 Ewa-RG-art_E
0	Ew-AU->in-RG	1 Ew-AU->in-RG		1 Ewa-RG-art_E
0	Ew-AU->in-RG	1		0
0	Ew-AU->in-RG	1 Ew-AU->in-RG		1 Ewa-RG-art_E
0		0		0
0		0		0
0	Ew-AU->in-RG	1 Ew-AU->in-RG		1 Ewa-RG-art_E
0	Ew-AU->in-RG	1 Ew-AU->in-RG		1 Ewa-RG-art_E
0	Ew-AU->in-RG	1 Ew-AU->in-RG		1 Ewa-RG-art_E
0	Ew-AU->in-RG	1 Ew-AU->in-RG		1 Ewa-RG-art_E
0	Ew-AU->in-RG	1 Ew-AU->in-RG		1 Ewa-RG-art_E
0	Ew-AU->in-RG	1 Ew-AU->in-RG		1 Ewa-RG-art_E
0	Ew-AU->in-RG	1 Ew-AU->in-RG		1 Ewa-RG-art_E
0		0		0
0	Ew-AU->in-RG	1		0
0	Ew-AU->in-RG	1		0
0		0		0
0		0		0
0	Ew-AU->in-RG	1		0 Ewa-RG-art_E
0	Ew-AU->in-RG	1 Ew-AU->in-RG		1 Ewa-RG-art_E
0	Ew-AU->in-RG	1 Ew-AU->in-RG		1 Ewa-RG-art_E
0	Ew-AU->in-RG	1 Ew-AU->in-RG		1 Ewa-RG-art_E
0	Ew-AU->in-RG	1 Ew-AU->in-RG		1 Ewa-RG-art_E
0	Ew-AU->in-RG	1 Ew-AU->in-RG		1 Ewa-RG-art_E

	0	Ew-AU->in-RG	1		0	
	0		0		0	
	0	Ew-AU->in-RG	1	Ew-AU->in-RG	1	Ewa-RG-art_E
	0	Ew-AU->in-RG	1	Ew-AU->in-RG	1	Ewa-RG-art_E
	0		0		0	
	0		0		0	
	0		0		0	
	0	Ew-AU->in-RG	1		0	Ewa-RG-art_E
	0	Ew-AU->in-RG	1		0	
	0	Ew-AU->in-RG	1	Ew-AU->in-RG	1	Ewa-RG-art_E
	0	Ew-AU->in-RG	1	Ew-AU->in-RG	1	Ewa-RG-art_E
	0	Ew-AU->in-RG	1	Ew-AU->in-RG	1	Ewa-RG-art_E
	0	Ew-AU->in-RG	1	Ew-AU->in-RG	1	Ewa-RG-art_E
	0		0		0	
	0	Ew-AU->in-RG	1	Ew-AU->in-RG	1	Ewa-RG-art_E
	0	Ew-AU->in-RG	1	Ew-AU->in-RG	1	Ewa-RG-art_E
	0	Ew-AU->in-RG	1	Ew-AU->in-RG	1	Ewa-RG-art_E
	0	Ew-AU->in-RG	1	Ew-AU->in-RG	1	
	0	Ew-AU->in-RG	1	Ew-AU->in-RG	1	
	0	Ew-AU->in-RG	1	Ew-AU->in-RG	1	Ewa-RG-art_E
	0	Ew-AU->in-RG	1	Ew-AU->in-RG	1	Ewa-RG-art_E
	0	Ew-AU->in-RG	1	Ew-AU->in-RG	1	Ewa-RG-art_E
	0	Ew-AU->in-RG	1	Ew-AU->in-RG	1	Ewa-RG-art_E
	0	Ew-AU->in-RG	1		0	Ewa-RG-art_E
	0	Ew-AU->in-RG	1	Ew-AU->in-RG	1	Ewa-RG-art_E
	0	Ew-AU->in-RG	1	Ew-AU->in-RG	1	Ewa-RG-art_E
Ewa-RG-top-P	1	Ew-AU->in-RG	1	Ew-AU->in-RG	1	Ewa-RG-art_E
	0	Ew-AU->in-RG	1	Ew-AU->in-RG	1	Ewa-RG-art_E
	0	Ew-AU->in-RG	1	Ew-AU->in-RG	1	Ewa-RG-art_E
	0		0		0	
	0	Ew-AU->in-RG	1	Ew-AU->in-RG	1	Ewa-RG-art_E
	0	Ew-AU->in-RG	1	Ew-AU->in-RG	1	Ewa-RG-art_E
	0	Ew-AU->in-RG	1	Ew-AU->in-RG	1	Ewa-RG-art_E
	0	Ew-AU->in-RG	1	Ew-AU->in-RG	1	Ewa-RG-art_E
	0		0		0	
	0		0		0	
	0		0		0	
	0	Ew-AU->in-RG	1	Ew-AU->in-RG	1	Ewa-RG-art_E
	0	Ew-AU->in-RG	1	Ew-AU->in-RG	1	Ewa-RG-art_E
	0	Ew-AU->in-RG	1	Ew-AU->in-RG	1	Ewa-RG-art_E
	0	Ew-AU->in-RG	1		0	Ewa-RG-art_E
	0		0		0	
	0	Ew-AU->in-RG	1		0	
	0	Ew-AU->in-RG	1	Ew-AU->in-RG	1	Ewa-RG-art_E
	0	Ew-AU->in-RG	1	Ew-AU->in-RG	1	Ewa-RG-art_E
	0	Ew-AU->in-RG	1	Ew-AU->in-RG	1	Ewa-RG-art_E
	0	Ew-AU->in-RG	1	Ew-AU->in-RG	1	Ewa-RG-art_E

0		0		0	
0	Ew-AU->in-RG	1	Ew-AU->in-RG	1	Ewa-RG-art_E
0	Ew-AU->in-RG	1	Ew-AU->in-RG	1	Ewa-RG-art_E
0	Ew-AU->in-RG	1	Ew-AU->in-RG	1	Ewa-RG-art_E
0		0		0	
0	Ew-AU->in-RG	1	Ew-AU->in-RG	1	Ewa-RG-art_E
0	Ew-AU->in-RG	1	Ew-AU->in-RG	1	Ewa-RG-art_E
0	Ew-AU->in-RG	1	Ew-AU->in-RG	1	Ewa-RG-art_E
0	Ew-AU->in-RG	1	Ew-AU->in-RG	1	Ewa-RG-art_E
0	Ew-AU->in-RG	1	Ew-AU->in-RG	1	Ewa-RG-art_E
0	Ew-AU->in-RG	1	Ew-AU->in-RG	1	Ewa-RG-art_E
0	Ew-AU->in-RG	1	Ew-AU->in-RG	1	Ewa-RG-art_E
0		0		0	
0		0		0	
0		0		0	
0		0		0	
0	Ew-AU->in-RG	1	Ew-AU->in-RG	1	Ewa-RG-art_E
0		0		0	
0	Ew-AU->in-RG	1	Ew-AU->in-RG	1	Ewa-RG-art_E
0	Ew-AU->in-RG	1	Ew-AU->in-RG	1	Ewa-RG-art_E
0	Ew-AU->in-RG	1	Ew-AU->in-RG	1	Ewa-RG-art_E
0	Ew-AU->in-RG	1	Ew-AU->in-RG	1	Ewa-RG-art_E
0	Ew-AU->in-RG	1	Ew-AU->in-RG	1	Ewa-RG-art_E
0	Ew-AU->in-RG	1	Ew-AU->in-RG	1	Ewa-RG-art_E
0	Ew-AU->in-RG	1	Ew-AU->in-RG	1	Ewa-RG-art_E
0		0		0	
0	Ew-AU->in-RG	1		0	Ewa-RG-art_E
0		0		0	
0	Ew-AU->in-RG	1	Ew-AU->in-RG	1	Ewa-RG-art_E
0	Ew-AU->in-RG	1		0	Ewa-RG-art_E
0	Ew-AU->in-RG	1		0	Ewa-RG-art_E
0	Ew-AU->in-RG	1		0	Ewa-RG-art_E
0	Ew-AU->in-RG	1		0	Ewa-RG-art_E
0	Ew-AU->in-RG	1		0	Ewa-RG-art_E
0		0		0	
0	Ew-AU->in-RG	1	Ew-AU->in-RG	1	Ewa-RG-art_E
0	Ew-AU->in-RG	1	Ew-AU->in-RG	1	Ewa-RG-art_E
0	Ew-AU->in-RG	1	Ew-AU->in-RG	1	Ewa-RG-art_E
0		0		0	
0	Ew-AU->in-RG	1	Ew-AU->in-RG	1	Ewa-RG-art_E
0	Ew-AU->in-RG	1	Ew-AU->in-RG	1	Ewa-RG-art_E
0	Ew-AU->in-RG	1	Ew-AU->in-RG	1	Ewa-RG-art_E
0		0		0	
0		0		0	
0		0		0	
0	Ew-AU->in-RG	1	Ew-AU->in-RG	1	Ewa-RG-art_E
0	Ew-AU->in-RG	1	Ew-AU->in-RG	1	Ewa-RG-art_E

	0	Ew-AU->in-RG	1	Ew-AU->in-RG	1	Ewa-RG-art_E
	0	Ew-AU->in-RG	1	Ew-AU->in-RG	1	Ewa-RG-art_E
	0	Ew-AU->in-RG	1	Ew-AU->in-RG	1	Ewa-RG-art_E
	0	Ew-AU->in-RG	1	Ew-AU->in-RG	1	Ewa-RG-art_E
	0	Ew-AU->in-RG	1	Ew-AU->in-RG	1	Ewa-RG-art_E
	0	Ew-AU->in-RG	1		0	
	0	Ew-AU->in-RG	1		0	
	0	Ew-AU->in-RG	1	Ew-AU->in-RG	1	Ewa-RG-art_E
	0	Ew-AU->in-RG	1		0	Ewa-RG-art_E
	0	Ew-AU->in-RG	1		0	Ewa-RG-art_E
	0	Ew-AU->in-RG	1	Ew-AU->in-RG	1	Ewa-RG-art_E
	0	Ew-AU->in-RG	1	Ew-AU->in-RG	1	Ewa-RG-art_E
	0	Ew-AU->in-RG	1	Ew-AU->in-RG	1	Ewa-RG-art_E
	0		0		0	
	0	Ew-AU->in-RG	1	Ew-AU->in-RG	1	Ewa-RG-art_E
	0		0		0	
	0		0		0	
	0	Ew-AU->in-RG	1	Ew-AU->in-RG	1	Ewa-RG-art_E
	0	Ew-AU->in-RG	1	Ew-AU->in-RG	1	Ewa-RG-art_E
	0	Ew-AU->in-RG	1	Ew-AU->in-RG	1	Ewa-RG-art_E
	0	Ew-AU->in-RG	1	Ew-AU->in-RG	1	Ewa-RG-art_E
	0	Ew-AU->in-RG	1	Ew-AU->in-RG	1	Ewa-RG-art_E
	0	Ew-AU->in-RG	1	Ew-AU->in-RG	1	Ewa-RG-art_E
	0	Ew-AU->in-RG	1	Ew-AU->in-RG	1	Ewa-RG-art_E
	0	Ew-AU->in-RG	1		0	
	0	Ew-AU->in-RG	1		0	
Ewa-RG-top-P	1	Ew-AU->in-RG	1	Ew-AU->in-RG	1	Ewa-RG-art_E
Ewa-RG-top-P	1	Ew-AU->in-RG	1	Ew-AU->in-RG	1	Ewa-RG-art_E
	0	Ew-AU->in-RG	1		0	Ewa-RG-art_E
	0	Ew-AU->in-RG	1		0	Ewa-RG-art_E
	0	Ew-AU->in-RG	1	Ew-AU->in-RG	1	Ewa-RG-art_E
	0	Ew-AU->in-RG	1	Ew-AU->in-RG	1	Ewa-RG-art_E
	0		0		0	
	0	Ew-AU->in-RG	1	Ew-AU->in-RG	1	Ewa-RG-art_E
	0		0		0	
	0	Ew-AU->in-RG	1		0	Ewa-RG-art_E
	0	Ew-AU->in-RG	1	Ew-AU->in-RG	1	Ewa-RG-art_E
	0	Ew-AU->in-RG	1	Ew-AU->in-RG	1	Ewa-RG-art_E
	0	Ew-AU->in-RG	1	Ew-AU->in-RG	1	Ewa-RG-art_E
	0	Ew-AU->in-RG	1	Ew-AU->in-RG	1	Ewa-RG-art_E
	0	Ew-AU->in-RG	1	Ew-AU->in-RG	1	Ewa-RG-art_E
	0	Ew-AU->in-RG	1	Ew-AU->in-RG	1	Ewa-RG-art_E
	0	Ew-AU->in-RG	1	Ew-AU->in-RG	1	Ewa-RG-art_E
	0		0		0	
	0		0		0	
	0		0		0	
	0		0		0	

	0		0		0	
	0	Ew-AU->in-RG	1	Ew-AU->in-RG	1	Ewa-RG-art_E
	0	Ew-AU->in-RG	1	Ew-AU->in-RG	1	Ewa-RG-art_E
	0	Ew-AU->in-RG	1	Ew-AU->in-RG	1	Ewa-RG-art_E
	0		0		0	
	0	Ew-AU->in-RG	1	Ew-AU->in-RG	1	Ewa-RG-art_E
	0	Ew-AU->in-RG	1		0	
	0	Ew-AU->in-RG	1		0	Ewa-RG-art_E
	0	Ew-AU->in-RG	1		0	Ewa-RG-art_E
	0	Ew-AU->in-RG	1	Ew-AU->in-RG	1	Ewa-RG-art_E
	0		0		0	
	0		0		0	
	0		0		0	
	0	Ew-AU->in-RG	1	Ew-AU->in-RG	1	Ewa-RG-art_E
	0	Ew-AU->in-RG	1	Ew-AU->in-RG	1	Ewa-RG-art_E
	0	Ew-AU->in-RG	1	Ew-AU->in-RG	1	Ewa-RG-art_E
	0	Ew-AU->in-RG	1	Ew-AU->in-RG	1	Ewa-RG-art_E
	0	Ew-AU->in-RG	1	Ew-AU->in-RG	1	Ewa-RG-art_E
	0	Ew-AU->in-RG	1	Ew-AU->in-RG	1	Ewa-RG-art_E
	0	Ew-AU->in-RG	1	Ew-AU->in-RG	1	Ewa-RG-art_E
	0	Ew-AU->in-RG	1	Ew-AU->in-RG	1	Ewa-RG-art_E
	0	Ew-AU->in-RG	1	Ew-AU->in-RG	1	Ewa-RG-art_E
	0	Ew-AU->in-RG	1	Ew-AU->in-RG	1	Ewa-RG-art_E
	0		0		0	
	0		0		0	
	0	Ew-AU->in-RG	1	Ew-AU->in-RG	1	Ewa-RG-art_E
	0	Ew-AU->in-RG	1	Ew-AU->in-RG	1	Ewa-RG-art_E
	0	Ew-AU->in-RG	1	Ew-AU->in-RG	1	Ewa-RG-art_E
	0	Ew-AU->in-RG	1	Ew-AU->in-RG	1	Ewa-RG-art_E
Ewa-RG-top-P	1	Ew-AU->in-RG	1	Ew-AU->in-RG	1	Ewa-RG-art_E
Ewa-RG-top-P	1	Ew-AU->in-RG	1	Ew-AU->in-RG	1	Ewa-RG-art_E
	0	Ew-AU->in-RG	1	Ew-AU->in-RG	1	Ewa-RG-art_E
	0		0		0	
	0		0		0	
	0	Ew-AU->in-RG	1	Ew-AU->in-RG	1	Ewa-RG-art_E
	0	Ew-AU->in-RG	1		0	Ewa-RG-art_E
	0	Ew-AU->in-RG	1		0	Ewa-RG-art_E
	0		0		0	
	0	Ew-AU->in-RG	1	Ew-AU->in-RG	1	Ewa-RG-art_E
	0	Ew-AU->in-RG	1	Ew-AU->in-RG	1	Ewa-RG-art_E
	0	Ew-AU->in-RG	1		0	Ewa-RG-art_E
	0	Ew-AU->in-RG	1		0	Ewa-RG-art_E
	0		0		0	
Ewa-RG-top-P	1	Ew-AU->in-RG	1	Ew-AU->in-RG	1	Ewa-RG-art_E
Ewa-RG-top-P	1	Ew-AU->in-RG	1	Ew-AU->in-RG	1	Ewa-RG-art_E
	0	Ew-AU->in-RG	1	Ew-AU->in-RG	1	Ewa-RG-art_E
	0	Ew-AU->in-RG	1	Ew-AU->in-RG	1	Ewa-RG-art_E

Ewa-RG-top-P	1	Ew-AU->in-RG	1	Ew-AU->in-RG	1	Ewa-RG-art_E
	0		0		0	
	0		0		0	
	0	Ew-AU->in-RG	1	Ew-AU->in-RG	1	Ewa-RG-art_E
	0		0		0	
	0	Ew-AU->in-RG	1		0	
	0		0		0	
	0		0		0	
	0	Ew-AU->in-RG	1	Ew-AU->in-RG	1	Ewa-RG-art_E
	0	Ew-AU->in-RG	1	Ew-AU->in-RG	1	Ewa-RG-art_E
Ewa-RG-top-P	1	Ew-AU->in-RG	1		0	Ewa-RG-art_E
Ewa-RG-top-P	1	Ew-AU->in-RG	1		0	Ewa-RG-art_E
Ewa-RG-top-P	1	Ew-AU->in-RG	1		0	Ewa-RG-art_E
Ewa-RG-top-P	1	Ew-AU->in-RG	1		0	Ewa-RG-art_E
	0		0		0	
	0	Ew-AU->in-RG	1	Ew-AU->in-RG	1	
	0	Ew-AU->in-RG	1	Ew-AU->in-RG	1	Ewa-RG-art_E
	0	Ew-AU->in-RG	1	Ew-AU->in-RG	1	Ewa-RG-art_E
	0	Ew-AU->in-RG	1		0	Ewa-RG-art_E
	0	Ew-AU->in-RG	1	Ew-AU->in-RG	1	Ewa-RG-art_E
	0	Ew-AU->in-RG	1	Ew-AU->in-RG	1	Ewa-RG-art_E
	0	Ew-AU->in-RG	1	Ew-AU->in-RG	1	Ewa-RG-art_E
	0	Ew-AU->in-RG	1	Ew-AU->in-RG	1	Ewa-RG-art_E
	0		0		0	
	0	Ew-AU->in-RG	1	Ew-AU->in-RG	1	Ewa-RG-art_E
	0		0		0	
	0		0		0	
	0		0		0	
	0	Ew-AU->in-RG	1	Ew-AU->in-RG	1	Ewa-RG-art_E
	0	Ew-AU->in-RG	1	Ew-AU->in-RG	1	Ewa-RG-art_E
	0	Ew-AU->in-RG	1	Ew-AU->in-RG	1	Ewa-RG-art_E
	0	Ew-AU->in-RG	1	Ew-AU->in-RG	1	Ewa-RG-art_E
	0	Ew-AU->in-RG	1		0	Ewa-RG-art_E
	0	Ew-AU->in-RG	1	Ew-AU->in-RG	1	Ewa-RG-art_E
	0	Ew-AU->in-RG	1	Ew-AU->in-RG	1	Ewa-RG-art_E
	0	Ew-AU->in-RG	1	Ew-AU->in-RG	1	Ewa-RG-art_E
Ewa-RG-top-P	1	Ew-AU->in-RG	1	Ew-AU->in-RG	1	Ewa-RG-art_E
	0		0		0	
	0	Ew-AU->in-RG	1	Ew-AU->in-RG	1	Ewa-RG-art_E
	0	Ew-AU->in-RG	1		0	Ewa-RG-art_E
	0	Ew-AU->in-RG	1		0	Ewa-RG-art_E
	0	Ew-AU->in-RG	1	Ew-AU->in-RG	1	Ewa-RG-art_E
	0	Ew-AU->in-RG	1	Ew-AU->in-RG	1	Ewa-RG-art_E
	0	Ew-AU->in-RG	1	Ew-AU->in-RG	1	Ewa-RG-art_E
	0		0		0	
	0		0		0	
	0	Ew-AU->in-RG	1	Ew-AU->in-RG	1	

0	Ew-AU->in-RG	1	Ew-AU->in-RG	1	
0	Ew-AU->in-RG	1	Ew-AU->in-RG	1	Ewa-RG-art_E
0		0		0	
0	Ew-AU->in-RG	1		0	Ewa-RG-art_E
0	Ew-AU->in-RG	1		0	Ewa-RG-art_E
0		0		0	
0		0		0	
0		0		0	
0	Ew-AU->in-RG	1	Ew-AU->in-RG	1	Ewa-RG-art_E
0	Ew-AU->in-RG	1	Ew-AU->in-RG	1	Ewa-RG-art_E
0	Ew-AU->in-RG	1	Ew-AU->in-RG	1	Ewa-RG-art_E
0	Ew-AU->in-RG	1	Ew-AU->in-RG	1	Ewa-RG-art_E
0	Ew-AU->in-RG	1	Ew-AU->in-RG	1	Ewa-RG-art_E
0	Ew-AU->in-RG	1	Ew-AU->in-RG	1	Ewa-RG-art_E
0	Ew-AU->in-RG	1	Ew-AU->in-RG	1	Ewa-RG-art_E
0	Ew-AU->in-RG	1	Ew-AU->in-RG	1	Ewa-RG-art_E
0	Ew-AU->in-RG	1	Ew-AU->in-RG	1	Ewa-RG-art_E
0	Ew-AU->in-RG	1	Ew-AU->in-RG	1	Ewa-RG-art_E
0	Ew-AU->in-RG	1	Ew-AU->in-RG	1	Ewa-RG-art_E
0	Ew-AU->in-RG	1	Ew-AU->in-RG	1	Ewa-RG-art_E
0	Ew-AU->in-RG	1	Ew-AU->in-RG	1	Ewa-RG-art_E
0	Ew-AU->in-RG	1	Ew-AU->in-RG	1	Ewa-RG-art_E
0	Ew-AU->in-RG	1	Ew-AU->in-RG	1	Ewa-RG-art_E
0		0		0	
0		0		0	
0		0		0	
0	Ew-AU->in-RG	1		0	Ewa-RG-art_E
0	Ew-AU->in-RG	1	Ew-AU->in-RG	1	Ewa-RG-art_E
0	Ew-AU->in-RG	1	Ew-AU->in-RG	1	Ewa-RG-art_E
0	Ew-AU->in-RG	1	Ew-AU->in-RG	1	Ewa-RG-art_E
0	Ew-AU->in-RG	1	Ew-AU->in-RG	1	Ewa-RG-art_E
0		0		0	
0		0		0	
0	Ew-AU->in-RG	1	Ew-AU->in-RG	1	Ewa-RG-art_E
0	Ew-AU->in-RG	1	Ew-AU->in-RG	1	Ewa-RG-art_E
0	Ew-AU->in-RG	1	Ew-AU->in-RG	1	Ewa-RG-art_E
0	Ew-AU->in-RG	1	Ew-AU->in-RG	1	Ewa-RG-art_E
0	Ew-AU->in-RG	1	Ew-AU->in-RG	1	Ewa-RG-art_E
0	Ew-AU->in-RG	1	Ew-AU->in-RG	1	Ewa-RG-art_E
0	Ew-AU->in-RG	1	Ew-AU->in-RG	1	Ewa-RG-art_E
0	Ew-AU->in-RG	1	Ew-AU->in-RG	1	Ewa-RG-art_E
0		0		0	
0		0		0	
0	Ew-AU->in-RG	1		0	
0	Ew-AU->in-RG	1		0	
0	Ew-AU->in-RG	1	Ew-AU->in-RG	1	Ewa-RG-art_E
0		0		0	

	0	Ew-AU->in-RG	1	Ew-AU->in-RG	1	Ewa-RG-art_E
	0	Ew-AU->in-RG	1	Ew-AU->in-RG	1	Ewa-RG-art_E
	0	Ew-AU->in-RG	1	Ew-AU->in-RG	1	Ewa-RG-art_E
	0	Ew-AU->in-RG	1	Ew-AU->in-RG	1	Ewa-RG-art_E
	0	Ew-AU->in-RG	1	Ew-AU->in-RG	1	Ewa-RG-art_E
	0	Ew-AU->in-RG	1	Ew-AU->in-RG	1	Ewa-RG-art_E
	0	Ew-AU->in-RG	1	Ew-AU->in-RG	1	Ewa-RG-art_E
	0	Ew-AU->in-RG	1	Ew-AU->in-RG	1	Ewa-RG-art_E
Ewa-RG-top-P	1	Ew-AU->in-RG	1	Ew-AU->in-RG	1	Ewa-RG-art_E
	0		0		0	
	0		0		0	
	0	Ew-AU->in-RG	1	Ew-AU->in-RG	1	Ewa-RG-art_E
	0	Ew-AU->in-RG	1	Ew-AU->in-RG	1	Ewa-RG-art_E
	0	Ew-AU->in-RG	1	Ew-AU->in-RG	1	Ewa-RG-art_E
	0		0		0	
	0	Ew-AU->in-RG	1	Ew-AU->in-RG	1	Ewa-RG-art_E
	0	Ew-AU->in-RG	1	Ew-AU->in-RG	1	Ewa-RG-art_E
	0		0		0	
	0	Ew-AU->in-RG	1	Ew-AU->in-RG	1	Ewa-RG-art_E
	0	Ew-AU->in-RG	1	Ew-AU->in-RG	1	Ewa-RG-art_E
	0	Ew-AU->in-RG	1		0	
	0		0		0	
	0	Ew-AU->in-RG	1	Ew-AU->in-RG	1	Ewa-RG-art_E
	0	Ew-AU->in-RG	1	Ew-AU->in-RG	1	Ewa-RG-art_E
	0		0		0	
	0	Ew-AU->in-RG	1		0	
	0	Ew-AU->in-RG	1	Ew-AU->in-RG	1	Ewa-RG-art_E
	0	Ew-AU->in-RG	1	Ew-AU->in-RG	1	Ewa-RG-art_E
	0	Ew-AU->in-RG	1		0	
	0	Ew-AU->in-RG	1	Ew-AU->in-RG	1	Ewa-RG-art_E
	0	Ew-AU->in-RG	1	Ew-AU->in-RG	1	Ewa-RG-art_E
	0	Ew-AU->in-RG	1	Ew-AU->in-RG	1	Ewa-RG-art_E
	0	Ew-AU->in-RG	1	Ew-AU->in-RG	1	Ewa-RG-art_E
	0		0		0	
	0	Ew-AU->in-RG	1	Ew-AU->in-RG	1	Ewa-RG-art_E
	0	Ew-AU->in-RG	1	Ew-AU->in-RG	1	Ewa-RG-art_E
	0	Ew-AU->in-RG	1	Ew-AU->in-RG	1	Ewa-RG-art_E
	0		0		0	
	0		0		0	
	0	Ew-AU->in-RG	1	Ew-AU->in-RG	1	Ewa-RG-art_E
	0	Ew-AU->in-RG	1		0	Ewa-RG-art_E
	0	Ew-AU->in-RG	1		0	Ewa-RG-art_E
	0	Ew-AU->in-RG	1	Ew-AU->in-RG	1	Ewa-RG-art_E
	0	Ew-AU->in-RG	1	Ew-AU->in-RG	1	Ewa-RG-art_E
	0	Ew-AU->in-RG	1	Ew-AU->in-RG	1	Ewa-RG-art_E
	0	Ew-AU->in-RG	1	Ew-AU->in-RG	1	Ewa-RG-art_E
	0	Ew-AU->in-RG	1	Ew-AU->in-RG	1	Ewa-RG-art_E

0		0		0	
0		0		0	
0	Ew-AU->in-RG	1	Ew-AU->in-RG	1	Ewa-RG-art_E
0		0		0	
0	Ew-AU->in-RG	1	Ew-AU->in-RG	1	Ewa-RG-art_E
0	Ew-AU->in-RG	1		0	Ewa-RG-art_E
0	Ew-AU->in-RG	1		0	Ewa-RG-art_E
0		0		0	
0	Ew-AU->in-RG	1	Ew-AU->in-RG	1	Ewa-RG-art_E
0	Ew-AU->in-RG	1	Ew-AU->in-RG	1	Ewa-RG-art_E
0	Ew-AU->in-RG	1		0	Ewa-RG-art_E
0	Ew-AU->in-RG	1		0	Ewa-RG-art_E
0		0		0	
0	Ew-AU->in-RG	1	Ew-AU->in-RG	1	Ewa-RG-art_E
0	Ew-AU->in-RG	1	Ew-AU->in-RG	1	Ewa-RG-art_E
0	Ew-AU->in-RG	1	Ew-AU->in-RG	1	Ewa-RG-art_E
0	Ew-AU->in-RG	1	Ew-AU->in-RG	1	Ewa-RG-art_E
0	Ew-AU->in-RG	1	Ew-AU->in-RG	1	Ewa-RG-art_E
0	Ew-AU->in-RG	1	Ew-AU->in-RG	1	Ewa-RG-art_E
0	Ew-AU->in-RG	1		0	
0		0		0	
0	Ew-AU->in-RG	1	Ew-AU->in-RG	1	Ewa-RG-art_E
0		0		0	
0		0		0	
0	Ew-AU->in-RG	1	Ew-AU->in-RG	1	Ewa-RG-art_E
0		0		0	
0	Ew-AU->in-RG	1	Ew-AU->in-RG	1	Ewa-RG-art_E
0	Ew-AU->in-RG	1	Ew-AU->in-RG	1	Ewa-RG-art_E
0		0		0	
0	Ew-AU->in-RG	1	Ew-AU->in-RG	1	Ewa-RG-art_E
0	Ew-AU->in-RG	1	Ew-AU->in-RG	1	Ewa-RG-art_E
0	Ew-AU->in-RG	1	Ew-AU->in-RG	1	Ewa-RG-art_E
0	Ew-AU->in-RG	1	Ew-AU->in-RG	1	Ewa-RG-art_E
0	Ew-AU->in-RG	1	Ew-AU->in-RG	1	Ewa-RG-art_E
0	Ew-AU->in-RG	1	Ew-AU->in-RG	1	Ewa-RG-art_E
0	Ew-AU->in-RG	1		0	
0		0		0	
0	Ew-AU->in-RG	1	Ew-AU->in-RG	1	Ewa-RG-art_E
0	Ew-AU->in-RG	1	Ew-AU->in-RG	1	Ewa-RG-art_E
0	Ew-AU->in-RG	1	Ew-AU->in-RG	1	Ewa-RG-art_E
0	Ew-AU->in-RG	1		0	Ewa-RG-art_E
0	Ew-AU->in-RG	1		0	Ewa-RG-art_E
0	Ew-AU->in-RG	1	Ew-AU->in-RG	1	Ewa-RG-art_E
0	Ew-AU->in-RG	1	Ew-AU->in-RG	1	Ewa-RG-art_E
0	Ew-AU->in-RG	1	Ew-AU->in-RG	1	Ewa-RG-art_E
0	Ew-AU->in-RG	1	Ew-AU->in-RG	1	Ewa-RG-art_E
0	Ew-AU->in-RG	1	Ew-AU->in-RG	1	Ewa-RG-art_E

	0	Ew-AU->in-RG	1	Ew-AU->in-RG	1	Ewa-RG-art_E
	0	Ew-AU->in-RG	1		0	
	0	Ew-AU->in-RG	1	Ew-AU->in-RG	1	Ewa-RG-art_E
	0		0		0	
	0	Ew-AU->in-RG	1	Ew-AU->in-RG	1	Ewa-RG-art_E
	0	Ew-AU->in-RG	1	Ew-AU->in-RG	1	Ewa-RG-art_E
	0	Ew-AU->in-RG	1	Ew-AU->in-RG	1	Ewa-RG-art_E
	0		0		0	
	0	Ew-AU->in-RG	1		0	Ewa-RG-art_E
	0	Ew-AU->in-RG	1		0	Ewa-RG-art_E
	0	Ew-AU->in-RG	1		0	Ewa-RG-art_E
	0	Ew-AU->in-RG	1		0	Ewa-RG-art_E
	0		0		0	
	0		0		0	
	0		0		0	
	0	Ew-AU->in-RG	1	Ew-AU->in-RG	1	Ewa-RG-art_E
	0	Ew-AU->in-RG	1	Ew-AU->in-RG	1	Ewa-RG-art_E
	0	Ew-AU->in-RG	1	Ew-AU->in-RG	1	Ewa-RG-art_E
	0	Ew-AU->in-RG	1	Ew-AU->in-RG	1	Ewa-RG-art_E
	0	Ew-AU->in-RG	1	Ew-AU->in-RG	1	Ewa-RG-art_E
	0	Ew-AU->in-RG	1	Ew-AU->in-RG	1	Ewa-RG-art_E
	0		0		0	
	0		0		0	
	0	Ew-AU->in-RG	1		0	Ewa-RG-art_E
	0	Ew-AU->in-RG	1		0	Ewa-RG-art_E
	0	Ew-AU->in-RG	1	Ew-AU->in-RG	1	Ewa-RG-art_E
	0	Ew-AU->in-RG	1	Ew-AU->in-RG	1	Ewa-RG-art_E
	0	Ew-AU->in-RG	1	Ew-AU->in-RG	1	Ewa-RG-art_E
	0	Ew-AU->in-RG	1	Ew-AU->in-RG	1	Ewa-RG-art_E
	0	Ew-AU->in-RG	1	Ew-AU->in-RG	1	Ewa-RG-art_E
	0	Ew-AU->in-RG	1		0	Ewa-RG-art_E
	0		0		0	
	0	Ew-AU->in-RG	1	Ew-AU->in-RG	1	Ewa-RG-art_E
	0	Ew-AU->in-RG	1	Ew-AU->in-RG	1	Ewa-RG-art_E
	0	Ew-AU->in-RG	1	Ew-AU->in-RG	1	Ewa-RG-art_E
	0	Ew-AU->in-RG	1	Ew-AU->in-RG	1	Ewa-RG-art_E
	0	Ew-AU->in-RG	1	Ew-AU->in-RG	1	Ewa-RG-art_E
	0	Ew-AU->in-RG	1		0	Ewa-RG-art_E
Ewa-RG-top-P	1	Ew-AU->in-RG	1	Ew-AU->in-RG	1	Ewa-RG-art_E
Ewa-RG-top-P	1	Ew-AU->in-RG	1	Ew-AU->in-RG	1	Ewa-RG-art_E
	0	Ew-AU->in-RG	1	Ew-AU->in-RG	1	Ewa-RG-art_E
	0	Ew-AU->in-RG	1	Ew-AU->in-RG	1	Ewa-RG-art_E
	0	Ew-AU->in-RG	1	Ew-AU->in-RG	1	Ewa-RG-art_E
	0		0		0	
	0		0		0	
	0	Ew-AU->in-RG	1	Ew-AU->in-RG	1	Ewa-RG-art_E
	0	Ew-AU->in-RG	1	Ew-AU->in-RG	1	Ewa-RG-art_E

	0	Ew-AU->in-RG	1	Ew-AU->in-RG	1	Ewa-RG-art_E
	0	Ew-AU->in-RG	1	Ew-AU->in-RG	1	Ewa-RG-art_E
	0	Ew-AU->in-RG	1	Ew-AU->in-RG	1	Ewa-RG-art_E
	0	Ew-AU->in-RG	1	Ew-AU->in-RG	1	Ewa-RG-art_E
	0	Ew-AU->in-RG	1	Ew-AU->in-RG	1	Ewa-RG-art_E
	0	Ew-AU->in-RG	1	Ew-AU->in-RG	1	Ewa-RG-art_E
	0	Ew-AU->in-RG	1	Ew-AU->in-RG	1	Ewa-RG-art_E
	0	Ew-AU->in-RG	1	Ew-AU->in-RG	1	Ewa-RG-art_E
	0	Ew-AU->in-RG	1	Ew-AU->in-RG	1	Ewa-RG-art_E
	0	Ew-AU->in-RG	1	Ew-AU->in-RG	1	Ewa-RG-art_E
	0		0		0	
Ewa-RG-top-P	1	Ew-AU->in-RG	1	Ew-AU->in-RG	1	Ewa-RG-art_E
	0		0		0	
	0		0		0	
	0		0		0	
	0	Ew-AU->in-RG	1	Ew-AU->in-RG	1	Ewa-RG-art_E
	0	Ew-AU->in-RG	1	Ew-AU->in-RG	1	Ewa-RG-art_E
	0		0		0	
	0		0		0	
	0		0		0	
	0	Ew-AU->in-RG	1	Ew-AU->in-RG	1	Ewa-RG-art_E
	0	Ew-AU->in-RG	1	Ew-AU->in-RG	1	Ewa-RG-art_E
	0	Ew-AU->in-RG	1		0	
	0	Ew-AU->in-RG	1		0	
	0		0		0	
	0	Ew-AU->in-RG	1	Ew-AU->in-RG	1	Ewa-RG-art_E
	0		0		0	
	0		0		0	
	0	Ew-AU->in-RG	1	Ew-AU->in-RG	1	Ewa-RG-art_E
	0	Ew-AU->in-RG	1	Ew-AU->in-RG	1	
	0	Ew-AU->in-RG	1	Ew-AU->in-RG	1	
	0	Ew-AU->in-RG	1	Ew-AU->in-RG	1	Ewa-RG-art_E
	0	Ew-AU->in-RG	1	Ew-AU->in-RG	1	Ewa-RG-art_E
	0	Ew-AU->in-RG	1	Ew-AU->in-RG	1	Ewa-RG-art_E
	0	Ew-AU->in-RG	1	Ew-AU->in-RG	1	Ewa-RG-art_E
	0	Ew-AU->in-RG	1	Ew-AU->in-RG	1	Ewa-RG-art_E
	0	Ew-AU->in-RG	1	Ew-AU->in-RG	1	Ewa-RG-art_E
	0		0		0	
	0		0		0	
	0	Ew-AU->in-RG	1	Ew-AU->in-RG	1	Ewa-RG-art_E
	0	Ew-AU->in-RG	1	Ew-AU->in-RG	1	Ewa-RG-art_E
	0	Ew-AU->in-RG	1	Ew-AU->in-RG	1	Ewa-RG-art_E
	0	Ew-AU->in-RG	1	Ew-AU->in-RG	1	Ewa-RG-art_E
	0	Ew-AU->in-RG	1		0	
	0		0		0	
	0	Ew-AU->in-RG	1	Ew-AU->in-RG	1	Ewa-RG-art_E
	0	Ew-AU->in-RG	1	Ew-AU->in-RG	1	Ewa-RG-art_E

	0	Ew-AU->in-RG	1	Ew-AU->in-RG	1	Ewa-RG-art_E
	0	Ew-AU->in-RG	1	Ew-AU->in-RG	1	Ewa-RG-art_E
	0	Ew-AU->in-RG	1	Ew-AU->in-RG	1	Ewa-RG-art_E
	0	Ew-AU->in-RG	1	Ew-AU->in-RG	1	Ewa-RG-art_E
	0	Ew-AU->in-RG	1	Ew-AU->in-RG	1	Ewa-RG-art_E
	0	Ew-AU->in-RG	1		0	
	0		0		0	
	0	Ew-AU->in-RG	1	Ew-AU->in-RG	1	Ewa-RG-art_E
	0		0		0	
	0		0		0	
	0		0		0	
	0	Ew-AU->in-RG	1	Ew-AU->in-RG	1	Ewa-RG-art_E
	0		0		0	
	0	Ew-AU->in-RG	1	Ew-AU->in-RG	1	Ewa-RG-art_E
	0	Ew-AU->in-RG	1	Ew-AU->in-RG	1	Ewa-RG-art_E
	0	Ew-AU->in-RG	1	Ew-AU->in-RG	1	Ewa-RG-art_E
	0	Ew-AU->in-RG	1	Ew-AU->in-RG	1	Ewa-RG-art_E
	0	Ew-AU->in-RG	1	Ew-AU->in-RG	1	Ewa-RG-art_E
	0	Ew-AU->in-RG	1	Ew-AU->in-RG	1	Ewa-RG-art_E
	0	Ew-AU->in-RG	1		0	Ewa-RG-art_E
	0	Ew-AU->in-RG	1		0	Ewa-RG-art_E
	0	Ew-AU->in-RG	1	Ew-AU->in-RG	1	Ewa-RG-art_E
	0	Ew-AU->in-RG	1	Ew-AU->in-RG	1	Ewa-RG-art_E
	0		0		0	
	0	Ew-AU->in-RG	1	Ew-AU->in-RG	1	Ewa-RG-art_E
	0	Ew-AU->in-RG	1	Ew-AU->in-RG	1	Ewa-RG-art_E
	0	Ew-AU->in-RG	1	Ew-AU->in-RG	1	Ewa-RG-art_E
	0	Ew-AU->in-RG	1	Ew-AU->in-RG	1	Ewa-RG-art_E
	0	Ew-AU->in-RG	1	Ew-AU->in-RG	1	Ewa-RG-art_E
	0	Ew-AU->in-RG	1		0	
	0		0		0	
	0		0		0	
Ewa-RG-top-P	1	Ew-AU->in-RG	1	Ew-AU->in-RG	1	Ewa-RG-art_E
	0		0		0	
	0	Ew-AU->in-RG	1	Ew-AU->in-RG	1	Ewa-RG-art_E
	0	Ew-AU->in-RG	1	Ew-AU->in-RG	1	Ewa-RG-art_E
	0	Ew-AU->in-RG	1	Ew-AU->in-RG	1	Ewa-RG-art_E
	0		0		0	
	0		0		0	
Ewa-RG-top-P	1	Ew-AU->in-RG	1		0	Ewa-RG-art_E
	0	Ew-AU->in-RG	1		0	Ewa-RG-art_E
	0		0		0	
	0	Ew-AU->in-RG	1	Ew-AU->in-RG	1	Ewa-RG-art_E
	0	Ew-AU->in-RG	1	Ew-AU->in-RG	1	Ewa-RG-art_E
	0	Ew-AU->in-RG	1	Ew-AU->in-RG	1	Ewa-RG-art_E
	0		0		0	
	0	Ew-AU->in-RG	1	Ew-AU->in-RG	1	Ewa-RG-art_E

Ewa-RG-top-F	1	Ew-AU->in-RG	1	Ew-AU->in-RG	1	Ewa-RG-art_E
Ewa-RG-top-F	1	Ew-AU->in-RG	1	Ew-AU->in-RG	1	Ewa-RG-art_E
	0	Ew-AU->in-RG	1	Ew-AU->in-RG	1	Ewa-RG-art_E
	0	Ew-AU->in-RG	1	Ew-AU->in-RG	1	Ewa-RG-art_E
	0	Ew-AU->in-RG	1	Ew-AU->in-RG	1	Ewa-RG-art_E
	0	Ew-AU->in-RG	1	Ew-AU->in-RG	1	Ewa-RG-art_E
	0	Ew-AU->in-RG	1	Ew-AU->in-RG	1	Ewa-RG-art_E
	0		0		0	
	0	Ew-AU->in-RG	1	Ew-AU->in-RG	1	Ewa-RG-art_E
	0	Ew-AU->in-RG	1	Ew-AU->in-RG	1	Ewa-RG-art_E
	0		0		0	
	0	Ew-AU->in-RG	1	Ew-AU->in-RG	1	Ewa-RG-art_E
	0	Ew-AU->in-RG	1	Ew-AU->in-RG	1	Ewa-RG-art_E
	0		0		0	
	0	Ew-AU->in-RG	1	Ew-AU->in-RG	1	Ewa-RG-art_E
	0	Ew-AU->in-RG	1	Ew-AU->in-RG	1	Ewa-RG-art_E
	0	Ew-AU->in-RG	1		0	
	0	Ew-AU->in-RG	1	Ew-AU->in-RG	1	Ewa-RG-art_E
	0	Ew-AU->in-RG	1	Ew-AU->in-RG	1	Ewa-RG-art_E
	0	Ew-AU->in-RG	1	Ew-AU->in-RG	1	Ewa-RG-art_E
	0	Ew-AU->in-RG	1	Ew-AU->in-RG	1	Ewa-RG-art_E
	0		0		0	
	0	Ew-AU->in-RG	1	Ew-AU->in-RG	1	Ewa-RG-art_E
	0		0		0	
	0	Ew-AU->in-RG	1	Ew-AU->in-RG	1	Ewa-RG-art_E
	0	Ew-AU->in-RG	1	Ew-AU->in-RG	1	Ewa-RG-art_E
	0		0		0	
	0		0		0	
	0		0		0	
	0	Ew-AU->in-RG	1		0	
	0		0		0	
	0	Ew-AU->in-RG	1	Ew-AU->in-RG	1	Ewa-RG-art_E
	0	Ew-AU->in-RG	1	Ew-AU->in-RG	1	Ewa-RG-art_E
	0	Ew-AU->in-RG	1		0	
	0	Ew-AU->in-RG	1	Ew-AU->in-RG	1	Ewa-RG-art_E
	0		0		0	
	0	Ew-AU->in-RG	1		0	Ewa-RG-art_E
	0	Ew-AU->in-RG	1		0	Ewa-RG-art_E
	0	Ew-AU->in-RG	1		0	Ewa-RG-art_E
	0	Ew-AU->in-RG	1	Ew-AU->in-RG	1	Ewa-RG-art_E
	0	Ew-AU->in-RG	1		0	
	0	Ew-AU->in-RG	1	Ew-AU->in-RG	1	Ewa-RG-art_E
Ewa-RG-top-F	1	Ew-AU->in-RG	1	Ew-AU->in-RG	1	Ewa-RG-art_E
	0		0		0	
	0	Ew-AU->in-RG	1	Ew-AU->in-RG	1	Ewa-RG-art_E
	0	Ew-AU->in-RG	1	Ew-AU->in-RG	1	Ewa-RG-art_E
	0		0		0	

0	Ew-AU->in-RG	1	Ew-AU->in-RG	1	Ewa-RG-art_E
0		0		0	
0		0		0	
0		0		0	
0		0		0	
0	Ew-AU->in-RG	1	Ew-AU->in-RG	1	Ewa-RG-art_E
0	Ew-AU->in-RG	1	Ew-AU->in-RG	1	Ewa-RG-art_E
0	Ew-AU->in-RG	1	Ew-AU->in-RG	1	Ewa-RG-art_E
0		0		0	
0	Ew-AU->in-RG	1	Ew-AU->in-RG	1	Ewa-RG-art_E
0	Ew-AU->in-RG	1	Ew-AU->in-RG	1	Ewa-RG-art_E
0	Ew-AU->in-RG	1	Ew-AU->in-RG	1	Ewa-RG-art_E
0	Ew-AU->in-RG	1		0	Ewa-RG-art_E
0	Ew-AU->in-RG	1		0	Ewa-RG-art_E
0	Ew-AU->in-RG	1	Ew-AU->in-RG	1	Ewa-RG-art_E
0	Ew-AU->in-RG	1	Ew-AU->in-RG	1	Ewa-RG-art_E
0	Ew-AU->in-RG	1	Ew-AU->in-RG	1	Ewa-RG-art_E
0	Ew-AU->in-RG	1	Ew-AU->in-RG	1	Ewa-RG-art_E
0	Ew-AU->in-RG	1		0	Ewa-RG-art_E
0	Ew-AU->in-RG	1		0	
0	Ew-AU->in-RG	1		0	
0	Ew-AU->in-RG	1	Ew-AU->in-RG	1	Ewa-RG-art_E
0		0		0	
0	Ew-AU->in-RG	1	Ew-AU->in-RG	1	Ewa-RG-art_E
0	Ew-AU->in-RG	1	Ew-AU->in-RG	1	Ewa-RG-art_E
0		0		0	
0	Ew-AU->in-RG	1		0	Ewa-RG-art_E
0	Ew-AU->in-RG	1		0	Ewa-RG-art_E
0		0		0	
0	Ew-AU->in-RG	1	Ew-AU->in-RG	1	Ewa-RG-art_E
0	Ew-AU->in-RG	1	Ew-AU->in-RG	1	Ewa-RG-art_E
0		0		0	
0		0		0	
0		0		0	
0	Ew-AU->in-RG	1		0	
0		0		0	
0		0		0	
0	Ew-AU->in-RG	1		0	Ewa-RG-art_E
0		0		0	
0	Ew-AU->in-RG	1		0	
0	Ew-AU->in-RG	1	Ew-AU->in-RG	1	Ewa-RG-art_E
0	Ew-AU->in-RG	1	Ew-AU->in-RG	1	Ewa-RG-art_E
0	Ew-AU->in-RG	1	Ew-AU->in-RG	1	Ewa-RG-art_E
0	Ew-AU->in-RG	1	Ew-AU->in-RG	1	Ewa-RG-art_E
0	Ew-AU->in-RG	1	Ew-AU->in-RG	1	Ewa-RG-art_E
0	Ew-AU->in-RG	1	Ew-AU->in-RG	1	Ewa-RG-art_E
0	Ew-AU->in-RG	1	Ew-AU->in-RG	1	Ewa-RG-art_E

	0	Ew-AU->in-RG	1	Ew-AU->in-RG	1	Ewa-RG-art_E
	0	Ew-AU->in-RG	1	Ew-AU->in-RG	1	Ewa-RG-art_E
	0	Ew-AU->in-RG	1	Ew-AU->in-RG	1	Ewa-RG-art_E
	0	Ew-AU->in-RG	1		0	Ewa-RG-art_E
	0	Ew-AU->in-RG	1		0	Ewa-RG-art_E
	0	Ew-AU->in-RG	1	Ew-AU->in-RG	1	Ewa-RG-art_E
	0	Ew-AU->in-RG	1	Ew-AU->in-RG	1	Ewa-RG-art_E
	0		0		0	
	0		0		0	
	0		0		0	
	0		0		0	
	0	Ew-AU->in-RG	1	Ew-AU->in-RG	1	Ewa-RG-art_E
	0	Ew-AU->in-RG	1	Ew-AU->in-RG	1	Ewa-RG-art_E
	0		0		0	
	0		0		0	
	0	Ew-AU->in-RG	1	Ew-AU->in-RG	1	Ewa-RG-art_E
	0	Ew-AU->in-RG	1	Ew-AU->in-RG	1	Ewa-RG-art_E
	0	Ew-AU->in-RG	1		0	
	0	Ew-AU->in-RG	1	Ew-AU->in-RG	1	Ewa-RG-art_E
	0	Ew-AU->in-RG	1	Ew-AU->in-RG	1	Ewa-RG-art_E
	0	Ew-AU->in-RG	1	Ew-AU->in-RG	1	Ewa-RG-art_E
	0	Ew-AU->in-RG	1	Ew-AU->in-RG	1	Ewa-RG-art_E
	0	Ew-AU->in-RG	1	Ew-AU->in-RG	1	Ewa-RG-art_E
	0		0		0	
	0	Ew-AU->in-RG	1	Ew-AU->in-RG	1	Ewa-RG-art_E
	0	Ew-AU->in-RG	1	Ew-AU->in-RG	1	Ewa-RG-art_E
	0	Ew-AU->in-RG	1	Ew-AU->in-RG	1	Ewa-RG-art_E
Ewa-RG-top-P	1	Ew-AU->in-RG	1	Ew-AU->in-RG	1	Ewa-RG-art_E
Ewa-RG-top-P	1	Ew-AU->in-RG	1	Ew-AU->in-RG	1	Ewa-RG-art_E
	0	Ew-AU->in-RG	1	Ew-AU->in-RG	1	Ewa-RG-art_E
	0	Ew-AU->in-RG	1	Ew-AU->in-RG	1	Ewa-RG-art_E
	0	Ew-AU->in-RG	1	Ew-AU->in-RG	1	Ewa-RG-art_E
	0	Ew-AU->in-RG	1	Ew-AU->in-RG	1	Ewa-RG-art_E
	0	Ew-AU->in-RG	1	Ew-AU->in-RG	1	Ewa-RG-art_E
	0	Ew-AU->in-RG	1	Ew-AU->in-RG	1	Ewa-RG-art_E
	0		0		0	
	0		0		0	
	0	Ew-AU->in-RG	1	Ew-AU->in-RG	1	Ewa-RG-art_E
	0	Ew-AU->in-RG	1	Ew-AU->in-RG	1	Ewa-RG-art_E
	0		0		0	
	0		0		0	
	0		0		0	
	0	Ew-AU->in-RG	1	Ew-AU->in-RG	1	Ewa-RG-art_E
	0	Ew-AU->in-RG	1	Ew-AU->in-RG	1	Ewa-RG-art_E
	0	Ew-AU->in-RG	1	Ew-AU->in-RG	1	Ewa-RG-art_E
	0		0		0	
	0	Ew-AU->in-RG	1	Ew-AU->in-RG	1	Ewa-RG-art_E

	0	Ew-AU->in-RG	1	Ew-AU->in-RG	1	Ewa-RG-art_E
	0		0		0	
	0	Ew-AU->in-RG	1		0	Ewa-RG-art_E
	0	Ew-AU->in-RG	1		0	Ewa-RG-art_E
	0	Ew-AU->in-RG	1		0	Ewa-RG-art_E
	0	Ew-AU->in-RG	1		0	Ewa-RG-art_E
	0	Ew-AU->in-RG	1	Ew-AU->in-RG	1	Ewa-RG-art_E
	0		0		0	
	0		0		0	
	0		0		0	
	0		0		0	
Ewa-RG-top-P	1	Ew-AU->in-RG	1	Ew-AU->in-RG	1	Ewa-RG-art_E
	0		0		0	
	0	Ew-AU->in-RG	1	Ew-AU->in-RG	1	Ewa-RG-art_E
	0	Ew-AU->in-RG	1	Ew-AU->in-RG	1	Ewa-RG-art_E
	0	Ew-AU->in-RG	1	Ew-AU->in-RG	1	Ewa-RG-art_E
	0	Ew-AU->in-RG	1		0	Ewa-RG-art_E
	0	Ew-AU->in-RG	1		0	Ewa-RG-art_E
	0	Ew-AU->in-RG	1		0	Ewa-RG-art_E
	0	Ew-AU->in-RG	1		0	Ewa-RG-art_E
	0	Ew-AU->in-RG	1	Ew-AU->in-RG	1	Ewa-RG-art_E
	0	Ew-AU->in-RG	1	Ew-AU->in-RG	1	Ewa-RG-art_E
	0	Ew-AU->in-RG	1	Ew-AU->in-RG	1	Ewa-RG-art_E
	0	Ew-AU->in-RG	1	Ew-AU->in-RG	1	Ewa-RG-art_E
	0	Ew-AU->in-RG	1	Ew-AU->in-RG	1	Ewa-RG-art_E
	0	Ew-AU->in-RG	1	Ew-AU->in-RG	1	Ewa-RG-art_E
	0	Ew-AU->in-RG	1	Ew-AU->in-RG	1	Ewa-RG-art_E
	0	Ew-AU->in-RG	1	Ew-AU->in-RG	1	Ewa-RG-art_E
	0	Ew-AU->in-RG	1	Ew-AU->in-RG	1	Ewa-RG-art_E
	0	Ew-AU->in-RG	1	Ew-AU->in-RG	1	Ewa-RG-art_E
	0	Ew-AU->in-RG	1	Ew-AU->in-RG	1	Ewa-RG-art_E
	0	Ew-AU->in-RG	1	Ew-AU->in-RG	1	Ewa-RG-art_E
	0	Ew-AU->in-RG	1	Ew-AU->in-RG	1	Ewa-RG-art_E
	0	Ew-AU->in-RG	1	Ew-AU->in-RG	1	Ewa-RG-art_E
	0	Ew-AU->in-RG	1	Ew-AU->in-RG	1	Ewa-RG-art_E
	0	Ew-AU->in-RG	1	Ew-AU->in-RG	1	Ewa-RG-art_E
	0		0		0	
	0	Ew-AU->in-RG	1	Ew-AU->in-RG	1	Ewa-RG-art_E
	0		0		0	
	0	Ew-AU->in-RG	1	Ew-AU->in-RG	1	Ewa-RG-art_E
	0		0		0	
	0		0		0	
	0	Ew-AU->in-RG	1	Ew-AU->in-RG	1	Ewa-RG-art_E
	0	Ew-AU->in-RG	1	Ew-AU->in-RG	1	Ewa-RG-art_E
	0	Ew-AU->in-RG	1	Ew-AU->in-RG	1	Ewa-RG-art_E
	0	Ew-AU->in-RG	1	Ew-AU->in-RG	1	Ewa-RG-art_E
	0	Ew-AU->in-RG	1	Ew-AU->in-RG	1	Ewa-RG-art_E

	0	Ew-AU->in-RG	1	Ew-AU->in-RG	1	Ewa-RG-art_E
	0	Ew-AU->in-RG	1	Ew-AU->in-RG	1	Ewa-RG-art_E
Ewa-RG-top-P	1	Ew-AU->in-RG	1	Ew-AU->in-RG	1	Ewa-RG-art_E
Ewa-RG-top-P	1	Ew-AU->in-RG	1	Ew-AU->in-RG	1	Ewa-RG-art_E
Ewa-RG-top-P	1	Ew-AU->in-RG	1	Ew-AU->in-RG	1	Ewa-RG-art_E
	0	Ew-AU->in-RG	1	Ew-AU->in-RG	1	Ewa-RG-art_E
	0	Ew-AU->in-RG	1	Ew-AU->in-RG	1	Ewa-RG-art_E
	0	Ew-AU->in-RG	1	Ew-AU->in-RG	1	Ewa-RG-art_E
	0		0		0	
	0	Ew-AU->in-RG	1	Ew-AU->in-RG	1	Ewa-RG-art_E
	0	Ew-AU->in-RG	1	Ew-AU->in-RG	1	Ewa-RG-art_E
	0	Ew-AU->in-RG	1		0	
	0	Ew-AU->in-RG	1		0	
	0	Ew-AU->in-RG	1	Ew-AU->in-RG	1	Ewa-RG-art_E
	0	Ew-AU->in-RG	1	Ew-AU->in-RG	1	Ewa-RG-art_E
	0	Ew-AU->in-RG	1	Ew-AU->in-RG	1	Ewa-RG-art_E
	0		0		0	
	0	Ew-AU->in-RG	1		0	
	0		0		0	
	0	Ew-AU->in-RG	1	Ew-AU->in-RG	1	Ewa-RG-art_E
	0		0		0	
	0	Ew-AU->in-RG	1	Ew-AU->in-RG	1	Ewa-RG-art_E
	0	Ew-AU->in-RG	1	Ew-AU->in-RG	1	Ewa-RG-art_E
	0	Ew-AU->in-RG	1	Ew-AU->in-RG	1	Ewa-RG-art_E
	0	Ew-AU->in-RG	1	Ew-AU->in-RG	1	Ewa-RG-art_E
	0	Ew-AU->in-RG	1	Ew-AU->in-RG	1	Ewa-RG-art_E
	0	Ew-AU->in-RG	1	Ew-AU->in-RG	1	Ewa-RG-art_E
	0	Ew-AU->in-RG	1	Ew-AU->in-RG	1	Ewa-RG-art_E
	0	Ew-AU->in-RG	1	Ew-AU->in-RG	1	Ewa-RG-art_E
	0	Ew-AU->in-RG	1	Ew-AU->in-RG	1	Ewa-RG-art_E
	0	Ew-AU->in-RG	1	Ew-AU->in-RG	1	Ewa-RG-art_E
	0	Ew-AU->in-RG	1	Ew-AU->in-RG	1	Ewa-RG-art_E
	0	Ew-AU->in-RG	1	Ew-AU->in-RG	1	Ewa-RG-art_E
	0	Ew-AU->in-RG	1	Ew-AU->in-RG	1	Ewa-RG-art_E
	0	Ew-AU->in-RG	1	Ew-AU->in-RG	1	Ewa-RG-art_E
	0	Ew-AU->in-RG	1	Ew-AU->in-RG	1	Ewa-RG-art_E
	0	Ew-AU->in-RG	1	Ew-AU->in-RG	1	Ewa-RG-art_E
	0	Ew-AU->in-RG	1		0	Ewa-RG-art_E
	0	Ew-AU->in-RG	1		0	Ewa-RG-art_E
	0	Ew-AU->in-RG	1	Ew-AU->in-RG	1	Ewa-RG-art_E
Ewa-RG-top-P	1	Ew-AU->in-RG	1	Ew-AU->in-RG	1	Ewa-RG-art_E
	0		0		0	
	0		0		0	
	0	Ew-AU->in-RG	1	Ew-AU->in-RG	1	Ewa-RG-art_E
	0	Ew-AU->in-RG	1	Ew-AU->in-RG	1	Ewa-RG-art_E
	0	Ew-AU->in-RG	1	Ew-AU->in-RG	1	Ewa-RG-art_E
	0	Ew-AU->in-RG	1		0	Ewa-RG-art_E

0	Ew-AU->in-RG	1		0	Ewa-RG-art_E
0	Ew-AU->in-RG	1	Ew-AU->in-RG	1	Ewa-RG-art_E
0	Ew-AU->in-RG	1	Ew-AU->in-RG	1	Ewa-RG-art_E
0	Ew-AU->in-RG	1	Ew-AU->in-RG	1	Ewa-RG-art_E
0	Ew-AU->in-RG	1	Ew-AU->in-RG	1	Ewa-RG-art_E
0	Ew-AU->in-RG	1	Ew-AU->in-RG	1	Ewa-RG-art_E
0	Ew-AU->in-RG	1	Ew-AU->in-RG	1	Ewa-RG-art_E
0	Ew-AU->in-RG	1		0	Ewa-RG-art_E
0	Ew-AU->in-RG	1		0	Ewa-RG-art_E
0	Ew-AU->in-RG	1		0	Ewa-RG-art_E
0	Ew-AU->in-RG	1		0	Ewa-RG-art_E
0	Ew-AU->in-RG	1	Ew-AU->in-RG	1	Ewa-RG-art_E
0	Ew-AU->in-RG	1	Ew-AU->in-RG	1	Ewa-RG-art_E
0	Ew-AU->in-RG	1	Ew-AU->in-RG	1	Ewa-RG-art_E
0		0		0	
0		0		0	
0		0		0	
0	Ew-AU->in-RG	1	Ew-AU->in-RG	1	Ewa-RG-art_E
0	Ew-AU->in-RG	1	Ew-AU->in-RG	1	Ewa-RG-art_E
0	Ew-AU->in-RG	1	Ew-AU->in-RG	1	Ewa-RG-art_E
0	Ew-AU->in-RG	1	Ew-AU->in-RG	1	Ewa-RG-art_E
0	Ew-AU->in-RG	1		0	Ewa-RG-art_E
0		0		0	
0		0		0	
0	Ew-AU->in-RG	1	Ew-AU->in-RG	1	Ewa-RG-art_E
0	Ew-AU->in-RG	1	Ew-AU->in-RG	1	Ewa-RG-art_E
0		0		0	
0	Ew-AU->in-RG	1		0	Ewa-RG-art_E
0	Ew-AU->in-RG	1	Ew-AU->in-RG	1	Ewa-RG-art_E
0	Ew-AU->in-RG	1	Ew-AU->in-RG	1	Ewa-RG-art_E
0	Ew-AU->in-RG	1		0	Ewa-RG-art_E
0	Ew-AU->in-RG	1		0	Ewa-RG-art_E
0		0		0	
0	Ew-AU->in-RG	1		0	Ewa-RG-art_E
0	Ew-AU->in-RG	1		0	Ewa-RG-art_E
0	Ew-AU->in-RG	1	Ew-AU->in-RG	1	Ewa-RG-art_E
0	Ew-AU->in-RG	1	Ew-AU->in-RG	1	Ewa-RG-art_E
0	Ew-AU->in-RG	1	Ew-AU->in-RG	1	Ewa-RG-art_E
0	Ew-AU->in-RG	1	Ew-AU->in-RG	1	Ewa-RG-art_E
0		0		0	
0	Ew-AU->in-RG	1		0	
0		0		0	
0	Ew-AU->in-RG	1	Ew-AU->in-RG	1	Ewa-RG-art_E
0	Ew-AU->in-RG	1		0	
0	Ew-AU->in-RG	1	Ew-AU->in-RG	1	Ewa-RG-art_E
0	Ew-AU->in-RG	1	Ew-AU->in-RG	1	Ewa-RG-art_E
0	Ew-AU->in-RG	1	Ew-AU->in-RG	1	Ewa-RG-art_E

	0		0		0	
	0		0		0	
	0		0		0	
	0		0		0	
	0		0		0	
	0	Ew-AU->in-RG	1	Ew-AU->in-RG	1	Ewa-RG-art_E
	0	Ew-AU->in-RG	1	Ew-AU->in-RG	1	Ewa-RG-art_E
	0	Ew-AU->in-RG	1	Ew-AU->in-RG	1	Ewa-RG-art_E
	0		0		0	
	0	Ew-AU->in-RG	1	Ew-AU->in-RG	1	Ewa-RG-art_E
Ewa-RG-top-P	1	Ew-AU->in-RG	1	Ew-AU->in-RG	1	Ewa-RG-art_E
Ewa-RG-top-P	1	Ew-AU->in-RG	1	Ew-AU->in-RG	1	Ewa-RG-art_E
	0		0		0	
	0		0		0	
	0		0		0	
	0	Ew-AU->in-RG	1	Ew-AU->in-RG	1	Ewa-RG-art_E
	0	Ew-AU->in-RG	1	Ew-AU->in-RG	1	Ewa-RG-art_E
	0	Ew-AU->in-RG	1	Ew-AU->in-RG	1	Ewa-RG-art_E
	0	Ew-AU->in-RG	1	Ew-AU->in-RG	1	Ewa-RG-art_E
	0		0		0	
	0		0		0	
	0	Ew-AU->in-RG	1	Ew-AU->in-RG	1	Ewa-RG-art_E
	0	Ew-AU->in-RG	1	Ew-AU->in-RG	1	Ewa-RG-art_E
	0	Ew-AU->in-RG	1	Ew-AU->in-RG	1	Ewa-RG-art_E
	0		0		0	
	0	Ew-AU->in-RG	1		0	Ewa-RG-art_E
	0	Ew-AU->in-RG	1		0	Ewa-RG-art_E
	0	Ew-AU->in-RG	1		0	Ewa-RG-art_E
	0	Ew-AU->in-RG	1		0	Ewa-RG-art_E
	0	Ew-AU->in-RG	1		0	Ewa-RG-art_E
	0	Ew-AU->in-RG	1		0	Ewa-RG-art_E
	0	Ew-AU->in-RG	1	Ew-AU->in-RG	1	Ewa-RG-art_E
	0	Ew-AU->in-RG	1	Ew-AU->in-RG	1	Ewa-RG-art_E
	0	Ew-AU->in-RG	1	Ew-AU->in-RG	1	Ewa-RG-art_E
Ewa-RG-top-P	1		0		0	
Ewa-RG-top-P	1		0		0	
	0	Ew-AU->in-RG	1	Ew-AU->in-RG	1	Ewa-RG-art_E
Ewa-RG-top-P	1	Ew-AU->in-RG	1	Ew-AU->in-RG	1	Ewa-RG-art_E
Ewa-RG-top-P	1	Ew-AU->in-RG	1	Ew-AU->in-RG	1	Ewa-RG-art_E
	0		0		0	
	0	Ew-AU->in-RG	1	Ew-AU->in-RG	1	Ewa-RG-art_E
	0	Ew-AU->in-RG	1	Ew-AU->in-RG	1	Ewa-RG-art_E
	0	Ew-AU->in-RG	1	Ew-AU->in-RG	1	Ewa-RG-art_E
	0		0		0	
	0	Ew-AU->in-RG	1	Ew-AU->in-RG	1	Ewa-RG-art_E
	0	Ew-AU->in-RG	1	Ew-AU->in-RG	1	Ewa-RG-art_E
	0	Ew-AU->in-RG	1	Ew-AU->in-RG	1	Ewa-RG-art_E

0	Ew-AU->in-RG	1	Ew-AU->in-RG	1	Ewa-RG-art_E
0	Ew-AU->in-RG	1	Ew-AU->in-RG	1	Ewa-RG-art_E
0	Ew-AU->in-RG	1	Ew-AU->in-RG	1	Ewa-RG-art_E
0	Ew-AU->in-RG	1		0	Ewa-RG-art_E
0	Ew-AU->in-RG	1		0	Ewa-RG-art_E
0		0		0	
0	Ew-AU->in-RG	1		0	
0	Ew-AU->in-RG	1		0	
0	Ew-AU->in-RG	1		0	
0	Ew-AU->in-RG	1	Ew-AU->in-RG	1	Ewa-RG-art_E
0		0		0	
0	Ew-AU->in-RG	1	Ew-AU->in-RG	1	Ewa-RG-art_E
0	Ew-AU->in-RG	1	Ew-AU->in-RG	1	Ewa-RG-art_E
0	Ew-AU->in-RG	1	Ew-AU->in-RG	1	Ewa-RG-art_E
0		0		0	
0		0		0	
0	Ew-AU->in-RG	1	Ew-AU->in-RG	1	Ewa-RG-art_E
0	Ew-AU->in-RG	1		0	
0	Ew-AU->in-RG	1	Ew-AU->in-RG	1	Ewa-RG-art_E
0	Ew-AU->in-RG	1	Ew-AU->in-RG	1	Ewa-RG-art_E
0	Ew-AU->in-RG	1	Ew-AU->in-RG	1	Ewa-RG-art_E
0	Ew-AU->in-RG	1	Ew-AU->in-RG	1	Ewa-RG-art_E
0	Ew-AU->in-RG	1	Ew-AU->in-RG	1	Ewa-RG-art_E
0		0		0	
0		0		0	
0		0		0	
0	Ew-AU->in-RG	1	Ew-AU->in-RG	1	Ewa-RG-art_E
0	Ew-AU->in-RG	1	Ew-AU->in-RG	1	Ewa-RG-art_E
0	Ew-AU->in-RG	1	Ew-AU->in-RG	1	Ewa-RG-art_E
0	Ew-AU->in-RG	1	Ew-AU->in-RG	1	Ewa-RG-art_E
0	Ew-AU->in-RG	1	Ew-AU->in-RG	1	Ewa-RG-art_E
0	Ew-AU->in-RG	1	Ew-AU->in-RG	1	Ewa-RG-art_E
0	Ew-AU->in-RG	1	Ew-AU->in-RG	1	Ewa-RG-art_E
0	Ew-AU->in-RG	1	Ew-AU->in-RG	1	Ewa-RG-art_E
0	Ew-AU->in-RG	1	Ew-AU->in-RG	1	Ewa-RG-art_E
0	Ew-AU->in-RG	1	Ew-AU->in-RG	1	Ewa-RG-art_E
0	Ew-AU->in-RG	1	Ew-AU->in-RG	1	Ewa-RG-art_E
0	Ew-AU->in-RG	1	Ew-AU->in-RG	1	Ewa-RG-art_E
0		0		0	
0	Ew-AU->in-RG	1	Ew-AU->in-RG	1	Ewa-RG-art_E
0	Ew-AU->in-RG	1	Ew-AU->in-RG	1	Ewa-RG-art_E
0	Ew-AU->in-RG	1	Ew-AU->in-RG	1	Ewa-RG-art_E
0		0		0	
0	Ew-AU->in-RG	1	Ew-AU->in-RG	1	Ewa-RG-art_E
0	Ew-AU->in-RG	1	Ew-AU->in-RG	1	Ewa-RG-art_E
0	Ew-AU->in-RG	1	Ew-AU->in-RG	1	Ewa-RG-art_E
0	Ew-AU->in-RG	1	Ew-AU->in-RG	1	Ewa-RG-art_E

	0	Ew-AU->in-RG	1	Ew-AU->in-RG	1	Ewa-RG-art_E
	0		0		0	
	0	Ew-AU->in-RG	1	Ew-AU->in-RG	1	Ewa-RG-art_E
	0	Ew-AU->in-RG	1	Ew-AU->in-RG	1	Ewa-RG-art_E
	0	Ew-AU->in-RG	1	Ew-AU->in-RG	1	Ewa-RG-art_E
	0	Ew-AU->in-RG	1	Ew-AU->in-RG	1	Ewa-RG-art_E
	0	Ew-AU->in-RG	1		0	
	0		0		0	
	0		0		0	
	0		0		0	
	0		0		0	
	0	Ew-AU->in-RG	1	Ew-AU->in-RG	1	Ewa-RG-art_E
	0	Ew-AU->in-RG	1	Ew-AU->in-RG	1	Ewa-RG-art_E
	0	Ew-AU->in-RG	1	Ew-AU->in-RG	1	Ewa-RG-art_E
	0	Ew-AU->in-RG	1	Ew-AU->in-RG	1	Ewa-RG-art_E
	0	Ew-AU->in-RG	1	Ew-AU->in-RG	1	Ewa-RG-art_E
	0	Ew-AU->in-RG	1	Ew-AU->in-RG	1	Ewa-RG-art_E
	0		0		0	
	0		0		0	
	0	Ew-AU->in-RG	1	Ew-AU->in-RG	1	Ewa-RG-art_E
	0	Ew-AU->in-RG	1		0	Ewa-RG-art_E
	0	Ew-AU->in-RG	1		0	Ewa-RG-art_E
	0	Ew-AU->in-RG	1	Ew-AU->in-RG	1	Ewa-RG-art_E
	0	Ew-AU->in-RG	1	Ew-AU->in-RG	1	Ewa-RG-art_E
	0	Ew-AU->in-RG	1	Ew-AU->in-RG	1	Ewa-RG-art_E
	0	Ew-AU->in-RG	1	Ew-AU->in-RG	1	Ewa-RG-art_E
	0		0		0	
	0		0		0	
	0		0		0	
	0	Ew-AU->in-RG	1	Ew-AU->in-RG	1	Ewa-RG-art_E
	0	Ew-AU->in-RG	1	Ew-AU->in-RG	1	Ewa-RG-art_E
	0	Ew-AU->in-RG	1	Ew-AU->in-RG	1	Ewa-RG-art_E
	0		0		0	
	0	Ew-AU->in-RG	1	Ew-AU->in-RG	1	Ewa-RG-art_E
	0	Ew-AU->in-RG	1	Ew-AU->in-RG	1	Ewa-RG-art_E
	0	Ew-AU->in-RG	1	Ew-AU->in-RG	1	Ewa-RG-art_E
	0	Ew-AU->in-RG	1	Ew-AU->in-RG	1	Ewa-RG-art_E
Ewa-RG-top-P	1	Ew-AU->in-RG	1		0	Ewa-RG-art_E
Ewa-RG-top-P	1	Ew-AU->in-RG	1		0	Ewa-RG-art_E
	0	Ew-AU->in-RG	1	Ew-AU->in-RG	1	Ewa-RG-art_E
	0	Ew-AU->in-RG	1	Ew-AU->in-RG	1	Ewa-RG-art_E
	0	Ew-AU->in-RG	1	Ew-AU->in-RG	1	Ewa-RG-art_E
	0	Ew-AU->in-RG	1	Ew-AU->in-RG	1	Ewa-RG-art_E
	0	Ew-AU->in-RG	1		0	
	0	Ew-AU->in-RG	1		0	
	0	Ew-AU->in-RG	1	Ew-AU->in-RG	1	Ewa-RG-art_E
	0	Ew-AU->in-RG	1	Ew-AU->in-RG	1	Ewa-RG-art_E

0		0		0	
0		0		0	
0		0		0	
0		0		0	
0		0		0	
0	Ew-AU->in-RG	1	Ew-AU->in-RG	1	
0	Ew-AU->in-RG	1	Ew-AU->in-RG	1	Ewa-RG-art_E
0	Ew-AU->in-RG	1	Ew-AU->in-RG	1	Ewa-RG-art_E
0	Ew-AU->in-RG	1	Ew-AU->in-RG	1	Ewa-RG-art_E
0	Ew-AU->in-RG	1		0	
0	Ew-AU->in-RG	1		0	
0	Ew-AU->in-RG	1	Ew-AU->in-RG	1	Ewa-RG-art_E
0	Ew-AU->in-RG	1	Ew-AU->in-RG	1	Ewa-RG-art_E
0	Ew-AU->in-RG	1	Ew-AU->in-RG	1	Ewa-RG-art_E
0	Ew-AU->in-RG	1	Ew-AU->in-RG	1	Ewa-RG-art_E
0	Ew-AU->in-RG	1	Ew-AU->in-RG	1	Ewa-RG-art_E
0	Ew-AU->in-RG	1	Ew-AU->in-RG	1	Ewa-RG-art_E
0	Ew-AU->in-RG	1	Ew-AU->in-RG	1	Ewa-RG-art_E
0		0		0	
0	Ew-AU->in-RG	1	Ew-AU->in-RG	1	Ewa-RG-art_E
0	Ew-AU->in-RG	1	Ew-AU->in-RG	1	Ewa-RG-art_E
0		0		0	
0		0		0	
0		0		0	
0	Ew-AU->in-RG	1	Ew-AU->in-RG	1	Ewa-RG-art_E
0	Ew-AU->in-RG	1	Ew-AU->in-RG	1	Ewa-RG-art_E
0	Ew-AU->in-RG	1	Ew-AU->in-RG	1	Ewa-RG-art_E
0	Ew-AU->in-RG	1	Ew-AU->in-RG	1	Ewa-RG-art_E
0	Ew-AU->in-RG	1	Ew-AU->in-RG	1	Ewa-RG-art_E
0	Ew-AU->in-RG	1	Ew-AU->in-RG	1	Ewa-RG-art_E
0	Ew-AU->in-RG	1	Ew-AU->in-RG	1	Ewa-RG-art_E
0		0		0	
0	Ew-AU->in-RG	1	Ew-AU->in-RG	1	Ewa-RG-art_E
0	Ew-AU->in-RG	1	Ew-AU->in-RG	1	Ewa-RG-art_E
0		0		0	
0	Ew-AU->in-RG	1	Ew-AU->in-RG	1	Ewa-RG-art_E
0	Ew-AU->in-RG	1	Ew-AU->in-RG	1	Ewa-RG-art_E
0	Ew-AU->in-RG	1	Ew-AU->in-RG	1	Ewa-RG-art_E
0	Ew-AU->in-RG	1	Ew-AU->in-RG	1	Ewa-RG-art_E
0		0		0	
0		0		0	
0	Ew-AU->in-RG	1	Ew-AU->in-RG	1	Ewa-RG-art_E
0		0		0	
0	Ew-AU->in-RG	1	Ew-AU->in-RG	1	Ewa-RG-art_E
0	Ew-AU->in-RG	1	Ew-AU->in-RG	1	Ewa-RG-art_E
0	Ew-AU->in-RG	1	Ew-AU->in-RG	1	Ewa-RG-art_E
0	Ew-AU->in-RG	1	Ew-AU->in-RG	1	Ewa-RG-art_E

	0	Ew-AU->in-RG	1	Ew-AU->in-RG	1	Ewa-RG-art_E
	0	Ew-AU->in-RG	1	Ew-AU->in-RG	1	Ewa-RG-art_E
	0		0		0	
	0	Ew-AU->in-RG	1	Ew-AU->in-RG	1	Ewa-RG-art_E
	0	Ew-AU->in-RG	1	Ew-AU->in-RG	1	Ewa-RG-art_E
	0		0		0	
	0	Ew-AU->in-RG	1	Ew-AU->in-RG	1	Ewa-RG-art_E
	0	Ew-AU->in-RG	1	Ew-AU->in-RG	1	Ewa-RG-art_E
	0	Ew-AU->in-RG	1	Ew-AU->in-RG	1	Ewa-RG-art_E
	0	Ew-AU->in-RG	1	Ew-AU->in-RG	1	Ewa-RG-art_E
	0	Ew-AU->in-RG	1	Ew-AU->in-RG	1	Ewa-RG-art_E
	0	Ew-AU->in-RG	1	Ew-AU->in-RG	1	Ewa-RG-art_E
	0		0		0	
	0		0		0	
	0	Ew-AU->in-RG	1		0	
	0	Ew-AU->in-RG	1	Ew-AU->in-RG	1	Ewa-RG-art_E
	0	Ew-AU->in-RG	1	Ew-AU->in-RG	1	Ewa-RG-art_E
	0	Ew-AU->in-RG	1	Ew-AU->in-RG	1	Ewa-RG-art_E
	0	Ew-AU->in-RG	1	Ew-AU->in-RG	1	Ewa-RG-art_E
	0	Ew-AU->in-RG	1	Ew-AU->in-RG	1	Ewa-RG-art_E
	0	Ew-AU->in-RG	1	Ew-AU->in-RG	1	Ewa-RG-art_E
	0	Ew-AU->in-RG	1	Ew-AU->in-RG	1	Ewa-RG-art_E
	0	Ew-AU->in-RG	1	Ew-AU->in-RG	1	Ewa-RG-art_E
	0		0		0	
	0	Ew-AU->in-RG	1	Ew-AU->in-RG	1	Ewa-RG-art_E
	0	Ew-AU->in-RG	1	Ew-AU->in-RG	1	Ewa-RG-art_E
	0	Ew-AU->in-RG	1	Ew-AU->in-RG	1	Ewa-RG-art_E
	0	Ew-AU->in-RG	1	Ew-AU->in-RG	1	Ewa-RG-art_E
	0	Ew-AU->in-RG	1	Ew-AU->in-RG	1	Ewa-RG-art_E
	0		0		0	
	0		0		0	
	0	Ew-AU->in-RG	1		0	
	0	Ew-AU->in-RG	1		0	
	0	Ew-AU->in-RG	1	Ew-AU->in-RG	1	Ewa-RG-art_E
	0	Ew-AU->in-RG	1	Ew-AU->in-RG	1	Ewa-RG-art_E
	0	Ew-AU->in-RG	1	Ew-AU->in-RG	1	Ewa-RG-art_E
	0	Ew-AU->in-RG	1	Ew-AU->in-RG	1	Ewa-RG-art_E
	0	Ew-AU->in-RG	1	Ew-AU->in-RG	1	Ewa-RG-art_E
	0	Ew-AU->in-RG	1	Ew-AU->in-RG	1	Ewa-RG-art_E
	0	Ew-AU->in-RG	1	Ew-AU->in-RG	1	Ewa-RG-art_E
	0	Ew-AU->in-RG	1		0	Ewa-RG-art_E
	0	Ew-AU->in-RG	1		0	Ewa-RG-art_E
	0	Ew-AU->in-RG	1		0	Ewa-RG-art_E
	0		0		0	
Ewa-RG-top-P	1	Ew-AU->in-RG	1		0	Ewa-RG-art_E
Ewa-RG-top-P	1	Ew-AU->in-RG	1		0	Ewa-RG-art_E
Ewa-RG-top-P	1	Ew-AU->in-RG	1		0	Ewa-RG-art_E

0	Ew-AU->in-RG	1	Ew-AU->in-RG	1	Ewa-RG-art_E
0	Ew-AU->in-RG	1	Ew-AU->in-RG	1	Ewa-RG-art_E
0	Ew-AU->in-RG	1	Ew-AU->in-RG	1	Ewa-RG-art_E
0	Ew-AU->in-RG	1	Ew-AU->in-RG	1	Ewa-RG-art_E
0	Ew-AU->in-RG	1	Ew-AU->in-RG	1	Ewa-RG-art_E
0	Ew-AU->in-RG	1	Ew-AU->in-RG	1	Ewa-RG-art_E
0	Ew-AU->in-RG	1	Ew-AU->in-RG	1	Ewa-RG-art_E
0	Ew-AU->in-RG	1	Ew-AU->in-RG	1	Ewa-RG-art_E
0	Ew-AU->in-RG	1		0	
0	Ew-AU->in-RG	1		0	Ewa-RG-art_E
0	Ew-AU->in-RG	1		0	Ewa-RG-art_E
0	Ew-AU->in-RG	1	Ew-AU->in-RG	1	Ewa-RG-art_E
0	Ew-AU->in-RG	1	Ew-AU->in-RG	1	Ewa-RG-art_E
0	Ew-AU->in-RG	1	Ew-AU->in-RG	1	Ewa-RG-art_E
0	Ew-AU->in-RG	1	Ew-AU->in-RG	1	Ewa-RG-art_E
0	Ew-AU->in-RG	1	Ew-AU->in-RG	1	Ewa-RG-art_E
0	Ew-AU->in-RG	1	Ew-AU->in-RG	1	Ewa-RG-art_E
0	Ew-AU->in-RG	1	Ew-AU->in-RG	1	Ewa-RG-art_E
0	Ew-AU->in-RG	1		0	
0	Ew-AU->in-RG	1		0	
0		0		0	
0	Ew-AU->in-RG	1		0	
0	Ew-AU->in-RG	1		0	
0	Ew-AU->in-RG	1	Ew-AU->in-RG	1	Ewa-RG-art_E
0	Ew-AU->in-RG	1	Ew-AU->in-RG	1	Ewa-RG-art_E
0	Ew-AU->in-RG	1	Ew-AU->in-RG	1	Ewa-RG-art_E
0	Ew-AU->in-RG	1	Ew-AU->in-RG	1	Ewa-RG-art_E
0	Ew-AU->in-RG	1	Ew-AU->in-RG	1	Ewa-RG-art_E
0	Ew-AU->in-RG	1	Ew-AU->in-RG	1	Ewa-RG-art_E
0		0		0	
0	Ew-AU->in-RG	1	Ew-AU->in-RG	1	Ewa-RG-art_E
0	Ew-AU->in-RG	1	Ew-AU->in-RG	1	Ewa-RG-art_E
0	Ew-AU->in-RG	1	Ew-AU->in-RG	1	Ewa-RG-art_E
0	Ew-AU->in-RG	1	Ew-AU->in-RG	1	Ewa-RG-art_E
0	Ew-AU->in-RG	1	Ew-AU->in-RG	1	Ewa-RG-art_E
0	Ew-AU->in-RG	1	Ew-AU->in-RG	1	Ewa-RG-art_E
0	Ew-AU->in-RG	1	Ew-AU->in-RG	1	Ewa-RG-art_E
0		0		0	
0		0		0	
0	Ew-AU->in-RG	1	Ew-AU->in-RG	1	Ewa-RG-art_E
0	Ew-AU->in-RG	1	Ew-AU->in-RG	1	Ewa-RG-art_E
0		0		0	
0	Ew-AU->in-RG	1	Ew-AU->in-RG	1	Ewa-RG-art_E
0	Ew-AU->in-RG	1	Ew-AU->in-RG	1	Ewa-RG-art_E
0		0		0	
0		0		0	
0	Ew-AU->in-RG	1		0	Ewa-RG-art_E

	0	Ew-AU->in-RG	1		0	Ewa-RG-art_E
	0	Ew-AU->in-RG	1	Ew-AU->in-RG	1	Ewa-RG-art_E
	0	Ew-AU->in-RG	1	Ew-AU->in-RG	1	Ewa-RG-art_E
	0	Ew-AU->in-RG	1	Ew-AU->in-RG	1	Ewa-RG-art_E
	0	Ew-AU->in-RG	1	Ew-AU->in-RG	1	Ewa-RG-art_E
	0	Ew-AU->in-RG	1	Ew-AU->in-RG	1	Ewa-RG-art_E
	0		0		0	
	0	Ew-AU->in-RG	1	Ew-AU->in-RG	1	Ewa-RG-art_E
	0	Ew-AU->in-RG	1	Ew-AU->in-RG	1	Ewa-RG-art_E
	0	Ew-AU->in-RG	1	Ew-AU->in-RG	1	Ewa-RG-art_E
	0	Ew-AU->in-RG	1	Ew-AU->in-RG	1	Ewa-RG-art_E
Ewa-RG-top-P	1	Ew-AU->in-RG	1	Ew-AU->in-RG	1	Ewa-RG-art_E
Ewa-RG-top-P	1	Ew-AU->in-RG	1	Ew-AU->in-RG	1	Ewa-RG-art_E
	0	Ew-AU->in-RG	1	Ew-AU->in-RG	1	Ewa-RG-art_E
	0	Ew-AU->in-RG	1	Ew-AU->in-RG	1	Ewa-RG-art_E
	0	Ew-AU->in-RG	1	Ew-AU->in-RG	1	Ewa-RG-art_E
	0	Ew-AU->in-RG	1	Ew-AU->in-RG	1	Ewa-RG-art_E
	0		0		0	
	0	Ew-AU->in-RG	1	Ew-AU->in-RG	1	Ewa-RG-art_E
	0	Ew-AU->in-RG	1		0	
	0	Ew-AU->in-RG	1		0	
	0	Ew-AU->in-RG	1	Ew-AU->in-RG	1	Ewa-RG-art_E
	0	Ew-AU->in-RG	1	Ew-AU->in-RG	1	Ewa-RG-art_E
	0	Ew-AU->in-RG	1	Ew-AU->in-RG	1	Ewa-RG-art_E
	0		0		0	
	0	Ew-AU->in-RG	1	Ew-AU->in-RG	1	Ewa-RG-art_E
	0	Ew-AU->in-RG	1		0	Ewa-RG-art_E
	0	Ew-AU->in-RG	1	Ew-AU->in-RG	1	Ewa-RG-art_E
	0	Ew-AU->in-RG	1	Ew-AU->in-RG	1	Ewa-RG-art_E
	0	Ew-AU->in-RG	1	Ew-AU->in-RG	1	Ewa-RG-art_E
	0	Ew-AU->in-RG	1	Ew-AU->in-RG	1	Ewa-RG-art_E
	0	Ew-AU->in-RG	1	Ew-AU->in-RG	1	Ewa-RG-art_E
	0	Ew-AU->in-RG	1	Ew-AU->in-RG	1	Ewa-RG-art_E
Ewa-RG-top-P	1	Ew-AU->in-RG	1	Ew-AU->in-RG	1	Ewa-RG-art_E
Ewa-RG-top-P	1	Ew-AU->in-RG	1	Ew-AU->in-RG	1	Ewa-RG-art_E
	0		0		0	
	0		0		0	
	0	Ew-AU->in-RG	1	Ew-AU->in-RG	1	Ewa-RG-art_E
	0	Ew-AU->in-RG	1	Ew-AU->in-RG	1	Ewa-RG-art_E
	0		0		0	
Ewa-RG-top-P	1	Ew-AU->in-RG	1	Ew-AU->in-RG	1	Ewa-RG-art_E
	0	Ew-AU->in-RG	1	Ew-AU->in-RG	1	Ewa-RG-art_E
	0	Ew-AU->in-RG	1	Ew-AU->in-RG	1	Ewa-RG-art_E
	0	Ew-AU->in-RG	1		0	
	0		0		0	
	0	Ew-AU->in-RG	1	Ew-AU->in-RG	1	Ewa-RG-art_E
Ewa-RG-top-P	1	Ew-AU->in-RG	1	Ew-AU->in-RG	1	Ewa-RG-art_E

Ewa-RG-top-P	1	Ew-AU->in-RG	1	Ew-AU->in-RG	1	Ewa-RG-art_E
	0	Ew-AU->in-RG	1		0	Ewa-RG-art_E
	0	Ew-AU->in-RG	1		0	Ewa-RG-art_E
	0	Ew-AU->in-RG	1	Ew-AU->in-RG	1	Ewa-RG-art_E
	0	Ew-AU->in-RG	1	Ew-AU->in-RG	1	Ewa-RG-art_E
	0	Ew-AU->in-RG	1	Ew-AU->in-RG	1	Ewa-RG-art_E
	0	Ew-AU->in-RG	1	Ew-AU->in-RG	1	Ewa-RG-art_E
Ewa-RG-top-P	1		0		0	
Ewa-RG-top-P	1		0		0	
Ewa-RG-top-P	1		0		0	
	0	Ew-AU->in-RG	1	Ew-AU->in-RG	1	Ewa-RG-art_E
	0	Ew-AU->in-RG	1	Ew-AU->in-RG	1	Ewa-RG-art_E
	0	Ew-AU->in-RG	1	Ew-AU->in-RG	1	Ewa-RG-art_E
	0	Ew-AU->in-RG	1	Ew-AU->in-RG	1	Ewa-RG-art_E
	0		0		0	
	0	Ew-AU->in-RG	1	Ew-AU->in-RG	1	Ewa-RG-art_E
	0	Ew-AU->in-RG	1	Ew-AU->in-RG	1	Ewa-RG-art_E
	0	Ew-AU->in-RG	1	Ew-AU->in-RG	1	Ewa-RG-art_E
	0	Ew-AU->in-RG	1	Ew-AU->in-RG	1	Ewa-RG-art_E
	0	Ew-AU->in-RG	1	Ew-AU->in-RG	1	Ewa-RG-art_E
	0		0		0	
	0		0		0	
	0		0		0	
	0		0		0	
	0	Ew-AU->in-RG	1	Ew-AU->in-RG	1	Ewa-RG-art_E
	0	Ew-AU->in-RG	1		0	Ewa-RG-art_E
	0	Ew-AU->in-RG	1		0	Ewa-RG-art_E
	0		0		0	
	0		0		0	
	0	Ew-AU->in-RG	1		0	Ewa-RG-art_E
	0	Ew-AU->in-RG	1		0	Ewa-RG-art_E
	0	Ew-AU->in-RG	1		0	Ewa-RG-art_E
	0	Ew-AU->in-RG	1	Ew-AU->in-RG	1	Ewa-RG-art_E
	0	Ew-AU->in-RG	1	Ew-AU->in-RG	1	Ewa-RG-art_E
	0	Ew-AU->in-RG	1	Ew-AU->in-RG	1	Ewa-RG-art_E
	0		0		0	
	0	Ew-AU->in-RG	1	Ew-AU->in-RG	1	Ewa-RG-art_E
	0	Ew-AU->in-RG	1	Ew-AU->in-RG	1	Ewa-RG-art_E
	0	Ew-AU->in-RG	1	Ew-AU->in-RG	1	Ewa-RG-art_E
	0	Ew-AU->in-RG	1	Ew-AU->in-RG	1	Ewa-RG-art_E
	0	Ew-AU->in-RG	1	Ew-AU->in-RG	1	Ewa-RG-art_E
	0		0		0	
	0	Ew-AU->in-RG	1	Ew-AU->in-RG	1	Ewa-RG-art_E
	0	Ew-AU->in-RG	1	Ew-AU->in-RG	1	Ewa-RG-art_E
	0	Ew-AU->in-RG	1	Ew-AU->in-RG	1	Ewa-RG-art_E
	0	Ew-AU->in-RG	1	Ew-AU->in-RG	1	Ewa-RG-art_E
	0	Ew-AU->in-RG	1	Ew-AU->in-RG	1	Ewa-RG-art_E

	0	Ew-AU->in-RG	1	Ew-AU->in-RG	1	Ewa-RG-art_E
	0	Ew-AU->in-RG	1	Ew-AU->in-RG	1	Ewa-RG-art_E
	0	Ew-AU->in-RG	1	Ew-AU->in-RG	1	Ewa-RG-art_E
	0	Ew-AU->in-RG	1	Ew-AU->in-RG	1	Ewa-RG-art_E
	0	Ew-AU->in-RG	1	Ew-AU->in-RG	1	Ewa-RG-art_E
	0	Ew-AU->in-RG	1	Ew-AU->in-RG	1	Ewa-RG-art_E
	0	Ew-AU->in-RG	1	Ew-AU->in-RG	1	Ewa-RG-art_E
	0	Ew-AU->in-RG	1	Ew-AU->in-RG	1	Ewa-RG-art_E
	0	Ew-AU->in-RG	1	Ew-AU->in-RG	1	Ewa-RG-art_E
	0	Ew-AU->in-RG	1		0	
	0		0		0	
	0	Ew-AU->in-RG	1		0	Ewa-RG-art_E
	0		0		0	
	0	Ew-AU->in-RG	1	Ew-AU->in-RG	1	Ewa-RG-art_E
	0	Ew-AU->in-RG	1	Ew-AU->in-RG	1	Ewa-RG-art_E
	0	Ew-AU->in-RG	1	Ew-AU->in-RG	1	Ewa-RG-art_E
	0	Ew-AU->in-RG	1	Ew-AU->in-RG	1	Ewa-RG-art_E
	0	Ew-AU->in-RG	1	Ew-AU->in-RG	1	Ewa-RG-art_E
	0	Ew-AU->in-RG	1	Ew-AU->in-RG	1	Ewa-RG-art_E
	0		0		0	
	0		0		0	
	0	Ew-AU->in-RG	1		0	
	0	Ew-AU->in-RG	1	Ew-AU->in-RG	1	Ewa-RG-art_E
	0	Ew-AU->in-RG	1	Ew-AU->in-RG	1	Ewa-RG-art_E
Ewa-RG-top-P	1		0		0	
Ewa-RG-top-P	1	Ew-AU->in-RG	1	Ew-AU->in-RG	1	Ewa-RG-art_E
	0		0		0	
	0	Ew-AU->in-RG	1	Ew-AU->in-RG	1	Ewa-RG-art_E
	0		0		0	
	0	Ew-AU->in-RG	1		0	Ewa-RG-art_E
	0	Ew-AU->in-RG	1	Ew-AU->in-RG	1	Ewa-RG-art_E
	0	Ew-AU->in-RG	1	Ew-AU->in-RG	1	Ewa-RG-art_E
	0		0		0	
	0	Ew-AU->in-RG	1	Ew-AU->in-RG	1	Ewa-RG-art_E
	0	Ew-AU->in-RG	1	Ew-AU->in-RG	1	Ewa-RG-art_E
Ewa-RG-top-P	1	Ew-AU->in-RG	1	Ew-AU->in-RG	1	Ewa-RG-art_E
	0	Ew-AU->in-RG	1	Ew-AU->in-RG	1	Ewa-RG-art_E
	0	Ew-AU->in-RG	1	Ew-AU->in-RG	1	Ewa-RG-art_E
	0		0		0	
	0	Ew-AU->in-RG	1	Ew-AU->in-RG	1	Ewa-RG-art_E
	0		0		0	
	0		0		0	
	0		0		0	
	0	Ew-AU->in-RG	1		0	Ewa-RG-art_E
	0	Ew-AU->in-RG	1		0	Ewa-RG-art_E
	0	Ew-AU->in-RG	1		0	Ewa-RG-art_E
	0	Ew-AU->in-RG	1		0	Ewa-RG-art_E

0	Ew-AU->in-RG	1	Ew-AU->in-RG	1	Ewa-RG-art_E
0	Ew-AU->in-RG	1	Ew-AU->in-RG	1	Ewa-RG-art_E
0	Ew-AU->in-RG	1		0	
0		0		0	
0	Ew-AU->in-RG	1	Ew-AU->in-RG	1	Ewa-RG-art_E
0	Ew-AU->in-RG	1	Ew-AU->in-RG	1	Ewa-RG-art_E
0	Ew-AU->in-RG	1		0	
0		0		0	
0	Ew-AU->in-RG	1	Ew-AU->in-RG	1	Ewa-RG-art_E
0	Ew-AU->in-RG	1	Ew-AU->in-RG	1	Ewa-RG-art_E
0	Ew-AU->in-RG	1	Ew-AU->in-RG	1	Ewa-RG-art_E
0	Ew-AU->in-RG	1	Ew-AU->in-RG	1	Ewa-RG-art_E
0	Ew-AU->in-RG	1	Ew-AU->in-RG	1	Ewa-RG-art_E
0	Ew-AU->in-RG	1	Ew-AU->in-RG	1	Ewa-RG-art_E
0	Ew-AU->in-RG	1	Ew-AU->in-RG	1	
0	Ew-AU->in-RG	1	Ew-AU->in-RG	1	
0	Ew-AU->in-RG	1	Ew-AU->in-RG	1	
0	Ew-AU->in-RG	1	Ew-AU->in-RG	1	
0		0		0	
0	Ew-AU->in-RG	1	Ew-AU->in-RG	1	Ewa-RG-art_E
0	Ew-AU->in-RG	1	Ew-AU->in-RG	1	Ewa-RG-art_E
0	Ew-AU->in-RG	1		0	Ewa-RG-art_E
0	Ew-AU->in-RG	1	Ew-AU->in-RG	1	Ewa-RG-art_E
0	Ew-AU->in-RG	1	Ew-AU->in-RG	1	Ewa-RG-art_E
0	Ew-AU->in-RG	1	Ew-AU->in-RG	1	Ewa-RG-art_E
0	Ew-AU->in-RG	1	Ew-AU->in-RG	1	Ewa-RG-art_E
0	Ew-AU->in-RG	1	Ew-AU->in-RG	1	Ewa-RG-art_E
0	Ew-AU->in-RG	1		0	Ewa-RG-art_E
0	Ew-AU->in-RG	1	Ew-AU->in-RG	1	Ewa-RG-art_E
0	Ew-AU->in-RG	1	Ew-AU->in-RG	1	Ewa-RG-art_E
0	Ew-AU->in-RG	1	Ew-AU->in-RG	1	Ewa-RG-art_E
0	Ew-AU->in-RG	1	Ew-AU->in-RG	1	Ewa-RG-art_E
0	Ew-AU->in-RG	1	Ew-AU->in-RG	1	Ewa-RG-art_E
0	Ew-AU->in-RG	1	Ew-AU->in-RG	1	Ewa-RG-art_E
0	Ew-AU->in-RG	1	Ew-AU->in-RG	1	Ewa-RG-art_E
0	Ew-AU->in-RG	1	Ew-AU->in-RG	1	Ewa-RG-art_E
0		0		0	
0	Ew-AU->in-RG	1	Ew-AU->in-RG	1	Ewa-RG-art_E
0	Ew-AU->in-RG	1	Ew-AU->in-RG	1	Ewa-RG-art_E
0	Ew-AU->in-RG	1	Ew-AU->in-RG	1	Ewa-RG-art_E
0	Ew-AU->in-RG	1	Ew-AU->in-RG	1	Ewa-RG-art_E
0	Ew-AU->in-RG	1	Ew-AU->in-RG	1	Ewa-RG-art_E
0	Ew-AU->in-RG	1	Ew-AU->in-RG	1	Ewa-RG-art_E
0	Ew-AU->in-RG	1	Ew-AU->in-RG	1	Ewa-RG-art_E
0	Ew-AU->in-RG	1	Ew-AU->in-RG	1	Ewa-RG-art_E
0	Ew-AU->in-RG	1	Ew-AU->in-RG	1	Ewa-RG-art_E
0	Ew-AU->in-RG	1	Ew-AU->in-RG	1	Ewa-RG-art_E

	0	Ew-AU->in-RG	1	Ew-AU->in-RG	1	Ewa-RG-art_E
	0		0		0	
Ewa-RG-top-P	1	Ew-AU->in-RG	1	Ew-AU->in-RG	1	Ewa-RG-art_E
Ewa-RG-top-P	1	Ew-AU->in-RG	1	Ew-AU->in-RG	1	Ewa-RG-art_E
	0		0		0	
	0		0		0	
	0		0		0	
	0		0		0	
	0	Ew-AU->in-RG	1		0	Ewa-RG-art_E
	0	Ew-AU->in-RG	1		0	Ewa-RG-art_E
	0	Ew-AU->in-RG	1		0	Ewa-RG-art_E
	0	Ew-AU->in-RG	1		0	Ewa-RG-art_E
	0	Ew-AU->in-RG	1		0	Ewa-RG-art_E
	0	Ew-AU->in-RG	1	Ew-AU->in-RG	1	Ewa-RG-art_E
	0		0		0	
	0	Ew-AU->in-RG	1	Ew-AU->in-RG	1	Ewa-RG-art_E
	0		0		0	
	0	Ew-AU->in-RG	1	Ew-AU->in-RG	1	Ewa-RG-art_E
	0	Ew-AU->in-RG	1	Ew-AU->in-RG	1	Ewa-RG-art_E
	0	Ew-AU->in-RG	1	Ew-AU->in-RG	1	Ewa-RG-art_E
	0	Ew-AU->in-RG	1	Ew-AU->in-RG	1	Ewa-RG-art_E
	0	Ew-AU->in-RG	1	Ew-AU->in-RG	1	Ewa-RG-art_E
	0	Ew-AU->in-RG	1	Ew-AU->in-RG	1	Ewa-RG-art_E
	0		0		0	
	0		0		0	
	0		0		0	
	0	Ew-AU->in-RG	1	Ew-AU->in-RG	1	Ewa-RG-art_E
	0	Ew-AU->in-RG	1	Ew-AU->in-RG	1	Ewa-RG-art_E
	0	Ew-AU->in-RG	1	Ew-AU->in-RG	1	Ewa-RG-art_E
	0	Ew-AU->in-RG	1	Ew-AU->in-RG	1	Ewa-RG-art_E
	0	Ew-AU->in-RG	1	Ew-AU->in-RG	1	Ewa-RG-art_E
	0	Ew-AU->in-RG	1	Ew-AU->in-RG	1	Ewa-RG-art_E
	0	Ew-AU->in-RG	1	Ew-AU->in-RG	1	Ewa-RG-art_E
	0	Ew-AU->in-RG	1	Ew-AU->in-RG	1	Ewa-RG-art_E
	0	Ew-AU->in-RG	1	Ew-AU->in-RG	1	Ewa-RG-art_E
	0	Ew-AU->in-RG	1	Ew-AU->in-RG	1	Ewa-RG-art_E
	0	Ew-AU->in-RG	1	Ew-AU->in-RG	1	Ewa-RG-art_E
	0	Ew-AU->in-RG	1	Ew-AU->in-RG	1	Ewa-RG-art_E
	0		0		0	
	0		0		0	
	0	Ew-AU->in-RG	1	Ew-AU->in-RG	1	Ewa-RG-art_E
	0	Ew-AU->in-RG	1	Ew-AU->in-RG	1	Ewa-RG-art_E
	0	Ew-AU->in-RG	1	Ew-AU->in-RG	1	Ewa-RG-art_E
	0		0		0	
	0		0		0	
	0		0		0	
	0	Ew-AU->in-RG	1		0	Ewa-RG-art_E

	0	Ew-AU->in-RG	1		0	Ewa-RG-art_E
Ewa-RG-top-P	1	Ew-AU->in-RG	1	Ew-AU->in-RG	1	Ewa-RG-art_E
	0	Ew-AU->in-RG	1		0	
	0	Ew-AU->in-RG	1	Ew-AU->in-RG	1	Ewa-RG-art_E
Ewa-RG-top-P	1	Ew-AU->in-RG	1	Ew-AU->in-RG	1	Ewa-RG-art_E
Ewa-RG-top-P	1	Ew-AU->in-RG	1	Ew-AU->in-RG	1	Ewa-RG-art_E
	0		0		0	
	0	Ew-AU->in-RG	1	Ew-AU->in-RG	1	Ewa-RG-art_E
	0	Ew-AU->in-RG	1	Ew-AU->in-RG	1	Ewa-RG-art_E
	0	Ew-AU->in-RG	1	Ew-AU->in-RG	1	Ewa-RG-art_E
	0	Ew-AU->in-RG	1	Ew-AU->in-RG	1	Ewa-RG-art_E
	0		0		0	
	0		0		0	
	0	Ew-AU->in-RG	1		0	Ewa-RG-art_E
	0	Ew-AU->in-RG	1		0	Ewa-RG-art_E
	0	Ew-AU->in-RG	1		0	Ewa-RG-art_E
	0		0		0	
	0	Ew-AU->in-RG	1	Ew-AU->in-RG	1	Ewa-RG-art_E
	0	Ew-AU->in-RG	1	Ew-AU->in-RG	1	Ewa-RG-art_E
	0	Ew-AU->in-RG	1	Ew-AU->in-RG	1	Ewa-RG-art_E
	0	Ew-AU->in-RG	1	Ew-AU->in-RG	1	Ewa-RG-art_E
	0		0		0	
	0	Ew-AU->in-RG	1		0	Ewa-RG-art_E
	0		0		0	
	0	Ew-AU->in-RG	1	Ew-AU->in-RG	1	Ewa-RG-art_E
	0	Ew-AU->in-RG	1		0	Ewa-RG-art_E
	0	Ew-AU->in-RG	1	Ew-AU->in-RG	1	Ewa-RG-art_E
	0		0		0	
	0		0		0	
	0		0		0	
Ewa-RG-top-P	1	Ew-AU->in-RG	1	Ew-AU->in-RG	1	Ewa-RG-art_E
	0		0		0	
	0		0		0	
	0	Ew-AU->in-RG	1	Ew-AU->in-RG	1	Ewa-RG-art_E
	0		0		0	
	0	Ew-AU->in-RG	1	Ew-AU->in-RG	1	Ewa-RG-art_E
	0	Ew-AU->in-RG	1	Ew-AU->in-RG	1	Ewa-RG-art_E
	0	Ew-AU->in-RG	1	Ew-AU->in-RG	1	Ewa-RG-art_E
	0	Ew-AU->in-RG	1	Ew-AU->in-RG	1	Ewa-RG-art_E
	0	Ew-AU->in-RG	1	Ew-AU->in-RG	1	Ewa-RG-art_E
	0	Ew-AU->in-RG	1	Ew-AU->in-RG	1	Ewa-RG-art_E
	0	Ew-AU->in-RG	1	Ew-AU->in-RG	1	Ewa-RG-art_E
	0	Ew-AU->in-RG	1	Ew-AU->in-RG	1	Ewa-RG-art_E
	0	Ew-AU->in-RG	1		0	
	0	Ew-AU->in-RG	1	Ew-AU->in-RG	1	Ewa-RG-art_E
	0	Ew-AU->in-RG	1	Ew-AU->in-RG	1	Ewa-RG-art_E
	0	Ew-AU->in-RG	1	Ew-AU->in-RG	1	Ewa-RG-art_E

0	Ew-AU->in-RG	1	Ew-AU->in-RG	1	Ewa-RG-art_E
0	Ew-AU->in-RG	1		0	Ewa-RG-art_E
0	Ew-AU->in-RG	1		0	Ewa-RG-art_E
0	Ew-AU->in-RG	1		0	Ewa-RG-art_E
0	Ew-AU->in-RG	1		0	Ewa-RG-art_E
0	Ew-AU->in-RG	1		0	Ewa-RG-art_E
0		0		0	
0	Ew-AU->in-RG	1	Ew-AU->in-RG	1	Ewa-RG-art_E
0	Ew-AU->in-RG	1	Ew-AU->in-RG	1	Ewa-RG-art_E
0	Ew-AU->in-RG	1	Ew-AU->in-RG	1	Ewa-RG-art_E
0	Ew-AU->in-RG	1	Ew-AU->in-RG	1	Ewa-RG-art_E
0		0		0	
0	Ew-AU->in-RG	1	Ew-AU->in-RG	1	Ewa-RG-art_E
0	Ew-AU->in-RG	1		0	Ewa-RG-art_E
0	Ew-AU->in-RG	1		0	Ewa-RG-art_E
0	Ew-AU->in-RG	1		0	Ewa-RG-art_E
0	Ew-AU->in-RG	1	Ew-AU->in-RG	1	Ewa-RG-art_E
0	Ew-AU->in-RG	1	Ew-AU->in-RG	1	Ewa-RG-art_E
0	Ew-AU->in-RG	1	Ew-AU->in-RG	1	Ewa-RG-art_E
0	Ew-AU->in-RG	1	Ew-AU->in-RG	1	Ewa-RG-art_E
0	Ew-AU->in-RG	1	Ew-AU->in-RG	1	Ewa-RG-art_E
0	Ew-AU->in-RG	1	Ew-AU->in-RG	1	Ewa-RG-art_E
0		0		0	
0	Ew-AU->in-RG	1	Ew-AU->in-RG	1	Ewa-RG-art_E
0	Ew-AU->in-RG	1	Ew-AU->in-RG	1	Ewa-RG-art_E
0	Ew-AU->in-RG	1	Ew-AU->in-RG	1	Ewa-RG-art_E
0	Ew-AU->in-RG	1	Ew-AU->in-RG	1	Ewa-RG-art_E
0		0		0	
0	Ew-AU->in-RG	1	Ew-AU->in-RG	1	Ewa-RG-art_E
0	Ew-AU->in-RG	1	Ew-AU->in-RG	1	Ewa-RG-art_E
0	Ew-AU->in-RG	1	Ew-AU->in-RG	1	Ewa-RG-art_E
0	Ew-AU->in-RG	1	Ew-AU->in-RG	1	Ewa-RG-art_E
0	Ew-AU->in-RG	1		0	Ewa-RG-art_E
0	Ew-AU->in-RG	1		0	Ewa-RG-art_E
0	Ew-AU->in-RG	1		0	Ewa-RG-art_E
0	Ew-AU->in-RG	1		0	Ewa-RG-art_E
0	Ew-AU->in-RG	1		0	Ewa-RG-art_E
0	Ew-AU->in-RG	1	Ew-AU->in-RG	1	Ewa-RG-art_E
0		0		0	
0		0		0	
0		0		0	
0		0		0	
0		0		0	
0		0		0	
0	Ew-AU->in-RG	1	Ew-AU->in-RG	1	Ewa-RG-art_E
0	Ew-AU->in-RG	1	Ew-AU->in-RG	1	Ewa-RG-art_E
0	Ew-AU->in-RG	1	Ew-AU->in-RG	1	Ewa-RG-art_E

0	Ew-AU->in-RG	1	Ew-AU->in-RG	1	Ewa-RG-art_E
0	Ew-AU->in-RG	1	Ew-AU->in-RG	1	Ewa-RG-art_E
0	Ew-AU->in-RG	1	Ew-AU->in-RG	1	Ewa-RG-art_E
0	Ew-AU->in-RG	1	Ew-AU->in-RG	1	Ewa-RG-art_E
0	Ew-AU->in-RG	1	Ew-AU->in-RG	1	Ewa-RG-art_E
0	Ew-AU->in-RG	1	Ew-AU->in-RG	1	Ewa-RG-art_E
0	Ew-AU->in-RG	1	Ew-AU->in-RG	1	Ewa-RG-art_E
0		0		0	
0		0		0	
0	Ew-AU->in-RG	1	Ew-AU->in-RG	1	Ewa-RG-art_E
0	Ew-AU->in-RG	1	Ew-AU->in-RG	1	Ewa-RG-art_E
0	Ew-AU->in-RG	1	Ew-AU->in-RG	1	Ewa-RG-art_E
0	Ew-AU->in-RG	1		0	Ewa-RG-art_E
0	Ew-AU->in-RG	1		0	Ewa-RG-art_E
0	Ew-AU->in-RG	1		0	Ewa-RG-art_E
0	Ew-AU->in-RG	1	Ew-AU->in-RG	1	Ewa-RG-art_E
0	Ew-AU->in-RG	1	Ew-AU->in-RG	1	Ewa-RG-art_E
0	Ew-AU->in-RG	1	Ew-AU->in-RG	1	Ewa-RG-art_E
0	Ew-AU->in-RG	1	Ew-AU->in-RG	1	Ewa-RG-art_E
0		0		0	
0	Ew-AU->in-RG	1		0	Ewa-RG-art_E
0	Ew-AU->in-RG	1		0	Ewa-RG-art_E
0	Ew-AU->in-RG	1		0	Ewa-RG-art_E
0		0		0	
0		0		0	
0	Ew-AU->in-RG	1	Ew-AU->in-RG	1	Ewa-RG-art_E
0	Ew-AU->in-RG	1	Ew-AU->in-RG	1	Ewa-RG-art_E
0	Ew-AU->in-RG	1	Ew-AU->in-RG	1	Ewa-RG-art_E
0	Ew-AU->in-RG	1	Ew-AU->in-RG	1	Ewa-RG-art_E
0	Ew-AU->in-RG	1	Ew-AU->in-RG	1	Ewa-RG-art_E
0	Ew-AU->in-RG	1	Ew-AU->in-RG	1	Ewa-RG-art_E
0	Ew-AU->in-RG	1	Ew-AU->in-RG	1	Ewa-RG-art_E
0	Ew-AU->in-RG	1	Ew-AU->in-RG	1	Ewa-RG-art_E
0	Ew-AU->in-RG	1	Ew-AU->in-RG	1	Ewa-RG-art_E
0	Ew-AU->in-RG	1	Ew-AU->in-RG	1	Ewa-RG-art_E
0	Ew-AU->in-RG	1	Ew-AU->in-RG	1	Ewa-RG-art_E
0	Ew-AU->in-RG	1	Ew-AU->in-RG	1	Ewa-RG-art_E
0	Ew-AU->in-RG	1		0	
0	Ew-AU->in-RG	1	Ew-AU->in-RG	1	Ewa-RG-art_E
0	Ew-AU->in-RG	1		0	Ewa-RG-art_E
0	Ew-AU->in-RG	1		0	Ewa-RG-art_E
0	Ew-AU->in-RG	1		0	Ewa-RG-art_E
0	Ew-AU->in-RG	1	Ew-AU->in-RG	1	Ewa-RG-art_E
0	Ew-AU->in-RG	1	Ew-AU->in-RG	1	Ewa-RG-art_E
0		0		0	
0		0		0	
0	Ew-AU->in-RG	1	Ew-AU->in-RG	1	Ewa-RG-art_E

	0	Ew-AU->in-RG	1	Ew-AU->in-RG	1	Ewa-RG-art_E
	0		0		0	
	0	Ew-AU->in-RG	1		0	Ewa-RG-art_E
	0	Ew-AU->in-RG	1		0	Ewa-RG-art_E
	0	Ew-AU->in-RG	1	Ew-AU->in-RG	1	Ewa-RG-art_E
	0	Ew-AU->in-RG	1	Ew-AU->in-RG	1	Ewa-RG-art_E
	0	Ew-AU->in-RG	1	Ew-AU->in-RG	1	Ewa-RG-art_E
Ewa-RG-top-P	1	Ew-AU->in-RG	1	Ew-AU->in-RG	1	Ewa-RG-art_E
Ewa-RG-top-P	1	Ew-AU->in-RG	1	Ew-AU->in-RG	1	Ewa-RG-art_E
	0		0		0	
	0	Ew-AU->in-RG	1	Ew-AU->in-RG	1	Ewa-RG-art_E
	0	Ew-AU->in-RG	1	Ew-AU->in-RG	1	Ewa-RG-art_E
	0		0		0	
	0	Ew-AU->in-RG	1	Ew-AU->in-RG	1	Ewa-RG-art_E
	0	Ew-AU->in-RG	1	Ew-AU->in-RG	1	Ewa-RG-art_E
	0	Ew-AU->in-RG	1	Ew-AU->in-RG	1	Ewa-RG-art_E
	0	Ew-AU->in-RG	1	Ew-AU->in-RG	1	Ewa-RG-art_E
	0	Ew-AU->in-RG	1	Ew-AU->in-RG	1	Ewa-RG-art_E
	0	Ew-AU->in-RG	1	Ew-AU->in-RG	1	Ewa-RG-art_E
	0	Ew-AU->in-RG	1	Ew-AU->in-RG	1	Ewa-RG-art_E
	0	Ew-AU->in-RG	1	Ew-AU->in-RG	1	Ewa-RG-art_E
	0	Ew-AU->in-RG	1	Ew-AU->in-RG	1	Ewa-RG-art_E
	0		0		0	
	0	Ew-AU->in-RG	1		0	Ewa-RG-art_E
	0	Ew-AU->in-RG	1		0	Ewa-RG-art_E
	0	Ew-AU->in-RG	1	Ew-AU->in-RG	1	Ewa-RG-art_E
	0	Ew-AU->in-RG	1	Ew-AU->in-RG	1	Ewa-RG-art_E
	0	Ew-AU->in-RG	1	Ew-AU->in-RG	1	Ewa-RG-art_E
	0	Ew-AU->in-RG	1	Ew-AU->in-RG	1	Ewa-RG-art_E
	0	Ew-AU->in-RG	1	Ew-AU->in-RG	1	Ewa-RG-art_E
	0	Ew-AU->in-RG	1	Ew-AU->in-RG	1	Ewa-RG-art_E
	0		0		0	

ewargart_au_	ewargart_au_	ewargart_au_	entrycoauinrg	ewauinrgart_	ewauinrgart_	ewauinrg_pi_	ewauinrg_pi_
0		0			0		0
1	Ewa-RG-art_E	1			0		0
0		0			0		0
0		0			0		0
0		0			0		0
0		0			0		0
0		0			0		0
0		0			0		0
1	Ewa-RG-art_E	1			0		0
1	Ewa-RG-art_E	1			0		0
1	Ewa-RG-art_E	1			0		0
1	Ewa-RG-art_E	1			0		0
0		0			0		0
0		0			0		0
1	Ewa-RG-art_E	1			0		0
1	Ewa-RG-art_E	1			0		0
0		0			0		0
1	Ewa-RG-art_E	1			0		0
0		0			0		0
0		0			0		0
0		0			0		0
0		0			0		0
1	Ewa-RG-art_E	1			0		0
1	Ewa-RG-art_E	1			0		0
1	Ewa-RG-art_E	1			0		0
0		0			0		0
1	Ewa-RG-art_E	1			0		0
1	Ewa-RG-art_E	1			0		0
1	Ewa-RG-art_E	1			0		0
1	Ewa-RG-art_E	1			0		0
1		0			0		0
0		0			0		0
0		0			0		0
1	Ewa-RG-art_E	1			0		0
1	Ewa-RG-art_E	1			0		0
0		0			0		0
0		0			0		0
1	Ewa-RG-art_E	1			0		0
0		0			0		0
1	Ewa-RG-art_E	1			0		0
1	Ewa-RG-art_E	1			0		0
1	Ewa-RG-art_E	1		Ew-AU->in-RG	1		0
1	Ewa-RG-art_E	1			0		0
1	Ewa-RG-art_E	1			0		0
1	Ewa-RG-art_E	1			0		0
0		0			0		0

0		0		0		0
1	Ewa-RG-art_E	1	Ew-AU->in-RG	1	Ew-AU->in-RG	1
1	Ewa-RG-art_E	1		0		0
1	Ewa-RG-art_E	1		0		0
1	Ewa-RG-art_E	1		0		0
1	Ewa-RG-art_E	1		0		0
1	Ewa-RG-art_E	1		0		0
1	Ewa-RG-art_E	1	Ew-AU->in-RG	1	Ew-AU->in-RG	1
0		0	Ew-AU->in-RG	1		0
0		0		0		0
0		0		0		0
0		0		0		0
1	Ewa-RG-art_E	1	Ew-AU->in-RG	1	Ew-AU->in-RG	1
1	Ewa-RG-art_E	1		0		0
0		0	Ew-AU->in-RG	1		0
0		0	Ew-AU->in-RG	1		0
0		0		0		0
0		0		0		0
0		0		0		0
0		0		0		0
1	Ewa-RG-art_E	1	Ew-AU->in-RG	1	Ew-AU->in-RG	1
1	Ewa-RG-art_E	1	Ew-AU->in-RG	1	Ew-AU->in-RG	1
1	Ewa-RG-art_E	1		0		0
0		0		0		0
1	Ewa-RG-art_E	1	Ew-AU->in-RG	1	Ew-AU->in-RG	1
1	Ewa-RG-art_E	1	Ew-AU->in-RG	1	Ew-AU->in-RG	1
1	Ewa-RG-art_E	1		0		0
1	Ewa-RG-art_E	1		0		0
1	Ewa-RG-art_E	1		0		0
1	Ewa-RG-art_E	1		0		0
1	Ewa-RG-art_E	1		0		0
1	Ewa-RG-art_E	1		0		0
1	Ewa-RG-art_E	1		0		0
1	Ewa-RG-art_E	1		0		0
0		0		0		0
0		0		0		0
0		0		0		0
0		0		0		0
0		0		0		0
1	Ewa-RG-art_E	1		0		0
1	Ewa-RG-art_E	1		0		0
1	Ewa-RG-art_E	1		0		0
1	Ewa-RG-art_E	1		0		0
1	Ewa-RG-art_E	1		0		0
0		0		0		0
1	Ewa-RG-art_E	1		0		0
1	Ewa-RG-art_E	1		0		0

1	Ewa-RG-art_E	1		0		0
0		0		0		0
1		0		0		0
1	Ewa-RG-art_E	1		0		0
1	Ewa-RG-art_E	1		0		0
1		0	Ew-AU->in-RG	1		0
1	Ewa-RG-art_E	1	Ew-AU->in-RG	1	Ew-AU->in-RG	1
1	Ewa-RG-art_E	1	Ew-AU->in-RG	1	Ew-AU->in-RG	1
1	Ewa-RG-art_E	1		0		0
0		0		0		0
0		0		0		0
1	Ewa-RG-art_E	1		0		0
1	Ewa-RG-art_E	1		0		0
1	Ewa-RG-art_E	1	Ew-AU->in-RG	1	Ew-AU->in-RG	1
0		0		0		0
1	Ewa-RG-art_E	1		0		0
1	Ewa-RG-art_E	1		0		0
1	Ewa-RG-art_E	1		0		0
1	Ewa-RG-art_E	1		0		0
0		0		0		0
1	Ewa-RG-art_E	1		0		0
1	Ewa-RG-art_E	1		0		0
1	Ewa-RG-art_E	1		0		0
0		0		0		0
0		0	Ew-AU->in-RG	1		0
1	Ewa-RG-art_E	1		0		0
1	Ewa-RG-art_E	1		0		0
1		0	Ew-AU->in-RG	1		0
1		0	Ew-AU->in-RG	1		0
1	Ewa-RG-art_E	1		0		0
1	Ewa-RG-art_E	1		0		0
1	Ewa-RG-art_E	1		0		0
1	Ewa-RG-art_E	1		0		0
1	Ewa-RG-art_E	1		0		0
1	Ewa-RG-art_E	1		0		0
1	Ewa-RG-art_E	1		0		0
1	Ewa-RG-art_E	1		0		0
1	Ewa-RG-art_E	1		0		0
1	Ewa-RG-art_E	1		0		0
1	Ewa-RG-art_E	1		0		0
1	Ewa-RG-art_E	1		0		0
1	Ewa-RG-art_E	1		0		0
1		0	Ew-AU->in-RG	1		0
0		0		0		0
1	Ewa-RG-art_E	1		0		0
1	Ewa-RG-art_E	1		0		0
1	Ewa-RG-art_E	1		0		0

1	Ewa-RG-art_E	1		0		0
1	Ewa-RG-art_E	1		0		0
1	Ewa-RG-art_E	1		0		0
1	Ewa-RG-art_E	1		0		0
1	Ewa-RG-art_E	1		0		0
0		0		0		0
1	Ewa-RG-art_E	1		0		0
1	Ewa-RG-art_E	1		0		0
0		0		0		0
0		0		0		0
0		0		0		0
0		0		0		0
0		0		0		0
1	Ewa-RG-art_E	1	Ew-AU->in-RG	1	Ew-AU->in-RG	1
1		0		0		0
1		0		0		0
1		0	Ew-AU->in-RG	1		0
1	Ewa-RG-art_E	1		0		0
1	Ewa-RG-art_E	1		0		0
1	Ewa-RG-art_E	1		0		0
0		0		0		0
0		0		0		0
1	Ewa-RG-art_E	1		0		0
0		0		0		0
0		0		0		0
0		0		0		0
1	Ewa-RG-art_E	1		0		0
1	Ewa-RG-art_E	1	Ew-AU->in-RG	1		0
0		0		0		0
1	Ewa-RG-art_E	1	Ew-AU->in-RG	1	Ew-AU->in-RG	1
1	Ewa-RG-art_E	1		0		0
1	Ewa-RG-art_E	1		0		0
1	Ewa-RG-art_E	1		0		0
0		0		0		0
1		0		0		0
1		0		0		0
1	Ewa-RG-art_E	1		0		0
1	Ewa-RG-art_E	1		0		0
1	Ewa-RG-art_E	1	Ew-AU->in-RG	1	Ew-AU->in-RG	1
0		0		0		0
1	Ewa-RG-art_E	1		0		0
1	Ewa-RG-art_E	1		0		0
1	Ewa-RG-art_E	1		0		0
0		0		0		0
1	Ewa-RG-art_E	1		0		0
1	Ewa-RG-art_E	1		0		0
1	Ewa-RG-art_E	1		0		0

1	Ewa-RG-art_E	1		0		0
1	Ewa-RG-art_E	1		0		0
1	Ewa-RG-art_E	1		0		0
1	Ewa-RG-art_E	1		0		0
1	Ewa-RG-art_E	1		0		0
1	Ewa-RG-art_E	1		0		0
1		0		0		0
1		0		0		0
1		0		0		0
1	Ewa-RG-art_E	1		0		0
0		0		0		0
0		0		0		0
1	Ewa-RG-art_E	1		0		0
1	Ewa-RG-art_E	1		0		0
1	Ewa-RG-art_E	1		0		0
1	Ewa-RG-art_E	1		0		0
1	Ewa-RG-art_E	1		0		0
1	Ewa-RG-art_E	1		0		0
0		0		0		0
1	Ewa-RG-art_E	1		0		0
1	Ewa-RG-art_E	1		0		0
1	Ewa-RG-art_E	1		0		0
1	Ewa-RG-art_E	1		0		0
1	Ewa-RG-art_E	1		0		0
1	Ewa-RG-art_E	1	Ew-AU->in-RG	1	Ew-AU->in-RG	1
1	Ewa-RG-art_E	1	Ew-AU->in-RG	1	Ew-AU->in-RG	1
1	Ewa-RG-art_E	1	Ew-AU->in-RG	1		0
1	Ewa-RG-art_E	1	Ew-AU->in-RG	1		0
1	Ewa-RG-art_E	1	Ew-AU->in-RG	1		0
1	Ewa-RG-art_E	1		0		0
1	Ewa-RG-art_E	1		0		0
0		0		0		0
0		0		0		0
0		0		0		0
0		0		0		0
0		0		0		0
1	Ewa-RG-art_E	1		0		0
1	Ewa-RG-art_E	1		0		0
1	Ewa-RG-art_E	1		0		0
1	Ewa-RG-art_E	1		0		0
1	Ewa-RG-art_E	1		0		0
1	Ewa-RG-art_E	1		0		0
1	Ewa-RG-art_E	1		0		0
1	Ewa-RG-art_E	1		0		0
1	Ewa-RG-art_E	1		0		0
1	Ewa-RG-art_E	1		0		0
0		0		0		0

1	Ewa-RG-art_E	1		0		0
1	Ewa-RG-art_E	1		0		0
1	Ewa-RG-art_E	1		0		0
1	Ewa-RG-art_E	1		0		0
1	Ewa-RG-art_E	1		0		0
1	Ewa-RG-art_E	1		0		0
1	Ewa-RG-art_E	1		0		0
1	Ewa-RG-art_E	1		0		0
1	Ewa-RG-art_E	1		0		0
1	Ewa-RG-art_E	1	Ew-AU->in-RG	1	Ew-AU->in-RG	1
0		0		0		0
0		0		0		0
1	Ewa-RG-art_E	1		0		0
1	Ewa-RG-art_E	1		0		0
0		0		0		0
0		0		0		0
1	Ewa-RG-art_E	1		0		0
1	Ewa-RG-art_E	1		0		0
1	Ewa-RG-art_E	1		0		0
0		0		0		0
0		0		0		0
1	Ewa-RG-art_E	1		0		0
1	Ewa-RG-art_E	1		0		0
1	Ewa-RG-art_E	1		0		0
0		0		0		0
0		0		0		0
1	Ewa-RG-art_E	1		0		0
1	Ewa-RG-art_E	1		0		0
0		0		0		0
0		0		0		0
1	Ewa-RG-art_E	1	Ew-AU->in-RG	1	Ew-AU->in-RG	1
1	Ewa-RG-art_E	1	Ew-AU->in-RG	1	Ew-AU->in-RG	1
1	Ewa-RG-art_E	1	Ew-AU->in-RG	1	Ew-AU->in-RG	1
0		0		0		0
0		0		0		0
1	Ewa-RG-art_E	1		0		0
1	Ewa-RG-art_E	1		0		0
1		0		0		0
1	Ewa-RG-art_E	1	Ew-AU->in-RG	1		0
1	Ewa-RG-art_E	1	Ew-AU->in-RG	1		0
1	Ewa-RG-art_E	1		0		0
1	Ewa-RG-art_E	1		0		0
1	Ewa-RG-art_E	1		0		0
1	Ewa-RG-art_E	1		0		0
0		0		0		0
0		0	Ew-AU->in-RG	1	Ew-AU->in-RG	1
1	Ewa-RG-art_E	1		0		0

1	Ewa-RG-art_E	1	Ew-AU->in-RG	1	Ew-AU->in-RG	1
1	Ewa-RG-art_E	1	Ew-AU->in-RG	1	Ew-AU->in-RG	1
1	Ewa-RG-art_E	1		0		0
0		0		0		0
1	Ewa-RG-art_E	1		0		0
1	Ewa-RG-art_E	1		0		0
1	Ewa-RG-art_E	1		0		0
0		0		0		0
0		0		0		0
0		0		0		0
0		0		0		0
1	Ewa-RG-art_E	1		0		0
1	Ewa-RG-art_E	1		0		0
1	Ewa-RG-art_E	1		0		0
1	Ewa-RG-art_E	1		0		0
0		0		0		0
0		0		0		0
1	Ewa-RG-art_E	1		0		0
1	Ewa-RG-art_E	1		0		0
1	Ewa-RG-art_E	1		0		0
0		0		0		0
0		0		0		0
1	Ewa-RG-art_E	1		0		0
1	Ewa-RG-art_E	1		0		0
1	Ewa-RG-art_E	1		0		0
1	Ewa-RG-art_E	1		0		0
1	Ewa-RG-art_E	1		0		0
1	Ewa-RG-art_E	1		0		0
1	Ewa-RG-art_E	1		0		0
1	Ewa-RG-art_E	1		0		0
0		0		0		0
0		0		0		0
1	Ewa-RG-art_E	1		0		0
0		0		0		0
0		0		0		0
0		0		0		0
1		0		0		0
0		0		0		0
1	Ewa-RG-art_E	1		0		0
1	Ewa-RG-art_E	1		0		0
0		0		0		0
1		0		0		0
1		0		0		0
1	Ewa-RG-art_E	1		0		0
1	Ewa-RG-art_E	1		0		0
0		0	Ew-AU->in-RG	1		0
0		0	Ew-AU->in-RG	1		0

0		0		0		0
1	Ewa-RG-art_E	1		0		0
1	Ewa-RG-art_E	1		0		0
0		0		0		0
0		0		0		0
1	Ewa-RG-art_E	1		0		0
1	Ewa-RG-art_E	1		0		0
1	Ewa-RG-art_E	1		0		0
1	Ewa-RG-art_E	1		0		0
0		0		0		0
0		0		0		0
1	Ewa-RG-art_E	1		0		0
1	Ewa-RG-art_E	1		0		0
1	Ewa-RG-art_E	1		0		0
1	Ewa-RG-art_E	1		0		0
1	Ewa-RG-art_E	1		0		0
0		0		0		0
1	Ewa-RG-art_E	1		0		0
1	Ewa-RG-art_E	1		0		0
0		0		0		0
1	Ewa-RG-art_E	1	Ew-AU->in-RG	1	Ew-AU->in-RG	1
0		0		0		0
0		0		0		0
0		0		0		0
1	Ewa-RG-art_E	1		0		0
1	Ewa-RG-art_E	1		0		0
1		0		0		0
0		0		0		0
0		0		0		0
1	Ewa-RG-art_E	1		0		0
0		0		0		0
1	Ewa-RG-art_E	1		0		0
1	Ewa-RG-art_E	1		0		0
1	Ewa-RG-art_E	1		0		0
0		0		0		0
0		0		0		0
0		0		0		0
0		0		0		0
1	Ewa-RG-art_E	1		0		0
0		0		0		0
1	Ewa-RG-art_E	1	Ew-AU->in-RG	1		0
1	Ewa-RG-art_E	1		0		0
1	Ewa-RG-art_E	1		0		0
0		0		0		0
0		0		0		0
1	Ewa-RG-art_E	1		0		0
1	Ewa-RG-art_E	1		0		0

0		0		0		0
0		0		0		0
1	Ewa-RG-art_E	1		0		0
1	Ewa-RG-art_E	1		0		0
1	Ewa-RG-art_E	1		0		0
1		0		0		0
1		0		0		0
1	Ewa-RG-art_E	1		0		0
1	Ewa-RG-art_E	1		0		0
1	Ewa-RG-art_E	1		0		0
1	Ewa-RG-art_E	1		0		0
1	Ewa-RG-art_E	1	Ew-AU->in-RG	1		0
1	Ewa-RG-art_E	1	Ew-AU->in-RG	1		0
0		0		0		0
0		0		0		0
1	Ewa-RG-art_E	1		0		0
1	Ewa-RG-art_E	1		0		0
1	Ewa-RG-art_E	1		0		0
1	Ewa-RG-art_E	1		0		0
0		0		0		0
0		0		0		0
1	Ewa-RG-art_E	1		0		0
0		0		0		0
0		0		0		0
0		0		0		0
0		0		0		0
1	Ewa-RG-art_E	1		0		0
1	Ewa-RG-art_E	1		0		0
1	Ewa-RG-art_E	1	Ew-AU->in-RG	1	Ew-AU->in-RG	1
1	Ewa-RG-art_E	1	Ew-AU->in-RG	1	Ew-AU->in-RG	1
0		0		0		0
1	Ewa-RG-art_E	1		0		0
1	Ewa-RG-art_E	1		0		0
0		0		0		0
1	Ewa-RG-art_E	1	Ew-AU->in-RG	1	Ew-AU->in-RG	1
1	Ewa-RG-art_E	1	Ew-AU->in-RG	1		0
1	Ewa-RG-art_E	1		0		0
1	Ewa-RG-art_E	1	Ew-AU->in-RG	1	Ew-AU->in-RG	1
0		0		0		0
1	Ewa-RG-art_E	1		0		0
1	Ewa-RG-art_E	1		0		0
0		0		0		0
1	Ewa-RG-art_E	1		0		0
1	Ewa-RG-art_E	1		0		0
1	Ewa-RG-art_E	1		0		0
0		0		0		0
0		0		0		0

0		0		0		0
0		0	Ew-AU->in-RG	1		0
0		0	Ew-AU->in-RG	1		0
0		0		0		0
0		0		0		0
0		0		0		0
0		0		0		0
1	Ewa-RG-art_E	1		0		0
1	Ewa-RG-art_E	1	Ew-AU->in-RG	1	Ew-AU->in-RG	1
1	Ewa-RG-art_E	1		0		0
1	Ewa-RG-art_E	1		0		0
0		0		0		0
0		0		0		0
0		0		0		0
0		0		0		0
1	Ewa-RG-art_E	1	Ew-AU->in-RG	1		0
1	Ewa-RG-art_E	1	Ew-AU->in-RG	1		0
1	Ewa-RG-art_E	1	Ew-AU->in-RG	1		0
1	Ewa-RG-art_E	1	Ew-AU->in-RG	1		0
1	Ewa-RG-art_E	1		0		0
1	Ewa-RG-art_E	1		0		0
1		0		0		0
1		0		0		0
1		0		0		0
0		0		0		0
0		0		0		0
0		0		0		0
0		0		0		0
0		0		0		0
1	Ewa-RG-art_E	1		0		0
1	Ewa-RG-art_E	1		0		0
0		0		0		0
1	Ewa-RG-art_E	1		0		0
1	Ewa-RG-art_E	1		0		0
1	Ewa-RG-art_E	1		0		0
0		0		0		0
0		0		0		0
0		0		0		0
1	Ewa-RG-art_E	1		0		0
0		0		0		0
0		0		0		0
1	Ewa-RG-art_E	1		0		0
1	Ewa-RG-art_E	1		0		0
1	Ewa-RG-art_E	1		0		0
1	Ewa-RG-art_E	1	Ew-AU->in-RG	1	Ew-AU->in-RG	1
1	Ewa-RG-art_E	1		0		0
1	Ewa-RG-art_E	1		0		0

1		0		0		0
1		0		0		0
1	Ewa-RG-art_E	1		0		0
1	Ewa-RG-art_E	1		0		0
1	Ewa-RG-art_E	1		0		0
0		0		0		0
0		0		0		0
0		0		0		0
0		0		0		0
1	Ewa-RG-art_E	1		0		0
1	Ewa-RG-art_E	1		0		0
1	Ewa-RG-art_E	1		0		0
1	Ewa-RG-art_E	1		0		0
1	Ewa-RG-art_E	1		0		0
1	Ewa-RG-art_E	1		0		0
1	Ewa-RG-art_E	1		0		0
1	Ewa-RG-art_E	1		0		0
1	Ewa-RG-art_E	1		0		0
1	Ewa-RG-art_E	1		0		0
1	Ewa-RG-art_E	1		0		0
1	Ewa-RG-art_E	1		0		0
1	Ewa-RG-art_E	1		0		0
0		0		0		0
1		0		0		0
1		0		0		0
1		0		0		0
1		0		0		0
1		0		0		0
1	Ewa-RG-art_E	1		0		0
1	Ewa-RG-art_E	1		0		0
1	Ewa-RG-art_E	1		0		0
1	Ewa-RG-art_E	1		0		0
0		0		0		0
0		0		0		0
1	Ewa-RG-art_E	1	Ew-AU->in-RG	1	Ew-AU->in-RG	1
0		0		0		0
1	Ewa-RG-art_E	1		0		0
1	Ewa-RG-art_E	1		0		0
0		0	Ew-AU->in-RG	1		0
0		0		0		0
1	Ewa-RG-art_E	1	Ew-AU->in-RG	1		0
1		0	Ew-AU->in-RG	1		0
1	Ewa-RG-art_E	1		0		0
1	Ewa-RG-art_E	1		0		0
0		0		0		0
1	Ewa-RG-art_E	1		0		0
1	Ewa-RG-art_E	1		0		0

0		0		0	0
0		0	Ew-AU->in-R	1	0
0		0		0	0
1	Ewa-RG-art_E	1		0	0
1	Ewa-RG-art_E	1		0	0
0		0		0	0
0		0		0	0
1	Ewa-RG-art_E	1		0	0
1	Ewa-RG-art_E	1		0	0
1	Ewa-RG-art_E	1		0	0
1		0		0	0
1		0		0	0
1		0		0	0
0		0		0	0
1	Ewa-RG-art_E	1		0	0
1	Ewa-RG-art_E	1		0	0
1	Ewa-RG-art_E	1		0	0
1	Ewa-RG-art_E	1		0	0
0		0		0	0
0		0		0	0
1	Ewa-RG-art_E	1		0	0
1	Ewa-RG-art_E	1		0	0
1	Ewa-RG-art_E	1		0	0
1	Ewa-RG-art_E	1		0	0
1		0		0	0
1		0		0	0
1	Ewa-RG-art_E	1		0	0
1	Ewa-RG-art_E	1		0	0
1	Ewa-RG-art_E	1		0	0
0		0		0	0
1	Ewa-RG-art_E	1		0	0
1	Ewa-RG-art_E	1		0	0
1	Ewa-RG-art_E	1		0	0
1	Ewa-RG-art_E	1		0	0
1	Ewa-RG-art_E	1		0	0
0		0		0	0
0		0		0	0
1	Ewa-RG-art_E	1		0	0
1	Ewa-RG-art_E	1		0	0
1	Ewa-RG-art_E	1		0	0
1		0		0	0
1		0		0	0
0		0		0	0
1	Ewa-RG-art_E	1		0	0
1	Ewa-RG-art_E	1		0	0
1	Ewa-RG-art_E	1		0	0
1		0		0	0

1	Ewa-RG-art_E	1		0		0
1	Ewa-RG-art_E	1		0		0
1	Ewa-RG-art_E	1		0		0
1	Ewa-RG-art_E	1		0		0
1	Ewa-RG-art_E	1		0		0
1	Ewa-RG-art_E	1		0		0
1	Ewa-RG-art_E	1		0		0
0		0		0		0
0		0		0		0
0		0		0		0
1	Ewa-RG-art_E	1		0		0
1	Ewa-RG-art_E	1		0		0
1	Ewa-RG-art_E	1	Ew-AU->in-RG	1	Ew-AU->in-RG	1
1	Ewa-RG-art_E	1		0		0
1	Ewa-RG-art_E	1		0		0
1	Ewa-RG-art_E	1		0		0
1	Ewa-RG-art_E	1		0		0
1	Ewa-RG-art_E	1		0		0
1	Ewa-RG-art_E	1		0		0
1	Ewa-RG-art_E	1		0		0
1	Ewa-RG-art_E	1		0		0
1	Ewa-RG-art_E	1		0		0
1	Ewa-RG-art_E	1	Ew-AU->in-RG	1		0
1	Ewa-RG-art_E	1		0		0
0		0		0		0
1	Ewa-RG-art_E	1		0		0
0		0		0		0
1	Ewa-RG-art_E	1		0		0
1	Ewa-RG-art_E	1		0		0
1	Ewa-RG-art_E	1		0		0
1	Ewa-RG-art_E	1		0		0
1	Ewa-RG-art_E	1		0		0
1	Ewa-RG-art_E	1		0		0
1	Ewa-RG-art_E	1		0		0
1		0		0		0
1		0		0		0
1	Ewa-RG-art_E	1		0		0
1	Ewa-RG-art_E	1		0		0
1	Ewa-RG-art_E	1		0		0
0		0		0		0
0		0		0		0
1	Ewa-RG-art_E	1		0		0
1		0		0		0
1		0		0		0
0		0		0		0
1	Ewa-RG-art_E	1		0		0
0		0		0		0

1	Ewa-RG-art_E	1		0		0
0		0		0		0
1	Ewa-RG-art_E	1	Ew-AU->in-RG	1	Ew-AU->in-RG	1
1	Ewa-RG-art_E	1	Ew-AU->in-RG	1	Ew-AU->in-RG	1
1	Ewa-RG-art_E	1		0		0
1	Ewa-RG-art_E	1		0		0
1	Ewa-RG-art_E	1		0		0
1	Ewa-RG-art_E	1		0		0
1	Ewa-RG-art_E	1		0		0
1	Ewa-RG-art_E	1		0		0
0		0		0		0
0		0		0		0
1	Ewa-RG-art_E	1		0		0
1	Ewa-RG-art_E	1		0		0
0		0		0		0
1		0		0		0
1		0		0		0
0		0		0		0
0		0		0		0
1		0		0		0
1		0		0		0
1	Ewa-RG-art_E	1	Ew-AU->in-RG	1	Ew-AU->in-RG	1
1	Ewa-RG-art_E	1	Ew-AU->in-RG	1	Ew-AU->in-RG	1
1	Ewa-RG-art_E	1		0		0
1	Ewa-RG-art_E	1		0		0
1	Ewa-RG-art_E	1		0		0
0		0		0		0
0		0		0		0
1	Ewa-RG-art_E	1		0		0
0		0		0		0
1	Ewa-RG-art_E	1		0		0
1	Ewa-RG-art_E	1		0		0
1	Ewa-RG-art_E	1		0		0
0		0		0		0
0		0		0		0
1	Ewa-RG-art_E	1		0		0
1	Ewa-RG-art_E	1		0		0
1	Ewa-RG-art_E	1		0		0
1		0		0		0
1		0		0		0
1		0		0		0
0		0		0		0
1	Ewa-RG-art_E	1		0		0
0		0		0		0
0		0		0		0
1	Ewa-RG-art_E	1		0		0
0		0		0		0

0		0		0		0
1	Ewa-RG-art_E	1	Ew-AU->in-RG	1		0
1	Ewa-RG-art_E	1	Ew-AU->in-RG	1	Ew-AU->in-RG	1
1	Ewa-RG-art_E	1	Ew-AU->in-RG	1	Ew-AU->in-RG	1
1	Ewa-RG-art_E	1		0		0
0		0		0		0
1	Ewa-RG-art_E	1		0		0
1	Ewa-RG-art_E	1		0		0
1	Ewa-RG-art_E	1		0		0
1	Ewa-RG-art_E	1		0		0
1	Ewa-RG-art_E	1		0		0
1	Ewa-RG-art_E	1		0		0
1	Ewa-RG-art_E	1		0		0
0		0		0		0
1	Ewa-RG-art_E	1	Ew-AU->in-RG	1	Ew-AU->in-RG	1
0		0		0		0
0		0		0		0
0		0		0		0
1	Ewa-RG-art_E	1	Ew-AU->in-RG	1	Ew-AU->in-RG	1
1	Ewa-RG-art_E	1		0		0
1	Ewa-RG-art_E	1		0		0
1	Ewa-RG-art_E	1		0		0
0		0		0		0
0		0		0		0
1	Ewa-RG-art_E	1		0		0
1	Ewa-RG-art_E	1		0		0
1	Ewa-RG-art_E	1		0		0
1	Ewa-RG-art_E	1		0		0
1	Ewa-RG-art_E	1		0		0
1	Ewa-RG-art_E	1		0		0
1		0	Ew-AU->in-RG	1		0
1		0	Ew-AU->in-RG	1		0
0		0		0		0
1	Ewa-RG-art_E	1		0		0
1	Ewa-RG-art_E	1		0		0
1	Ewa-RG-art_E	1		0		0
1	Ewa-RG-art_E	1		0		0
0		0		0		0
0		0		0		0
1	Ewa-RG-art_E	1		0		0
0		0		0		0
0		0		0		0
0		0		0		0
0		0		0		0
1	Ewa-RG-art_E	1		0		0
1	Ewa-RG-art_E	1		0		0
0		0		0		0

1	Ewa-RG-art_E	1		0		0
1	Ewa-RG-art_E	1		0		0
1	Ewa-RG-art_E	1		0		0
1	Ewa-RG-art_E	1		0		0
1	Ewa-RG-art_E	1		0		0
1	Ewa-RG-art_E	1		0		0
1	Ewa-RG-art_E	1		0		0
1	Ewa-RG-art_E	1		0		0
1	Ewa-RG-art_E	1	Ew-AU->in-RG	1	Ew-AU->in-RG	1
0		0		0		0
1	Ewa-RG-art_E	1		0		0
1	Ewa-RG-art_E	1		0		0
1	Ewa-RG-art_E	1		0		0
0		0		0		0
0		0		0		0
0		0		0		0
0		0		0		0
0		0		0		0
1		0		0		0
1	Ewa-RG-art_E	1		0		0
1	Ewa-RG-art_E	1		0		0
1	Ewa-RG-art_E	1		0		0
1	Ewa-RG-art_E	1		0		0
0		0		0		0
1	Ewa-RG-art_E	1		0		0
1	Ewa-RG-art_E	1		0		0
1		0		0		0
1		0		0		0
1	Ewa-RG-art_E	1	Ew-AU->in-RG	1	Ew-AU->in-RG	1
1	Ewa-RG-art_E	1	Ew-AU->in-RG	1	Ew-AU->in-RG	1
1	Ewa-RG-art_E	1	Ew-AU->in-RG	1	Ew-AU->in-RG	1
0		0		0		0
0		0		0		0
1		0		0		0
1		0		0		0
0		0		0		0
0		0		0		0
0		0		0		0
1	Ewa-RG-art_E	1		0		0
1	Ewa-RG-art_E	1		0		0
1	Ewa-RG-art_E	1		0		0
1	Ewa-RG-art_E	1		0		0
1	Ewa-RG-art_E	1		0		0
1	Ewa-RG-art_E	1		0		0
0		0		0		0
1	Ewa-RG-art_E	1		0		0
0		0		0		0

0		0		0	0
0		0		0	0
0		0		0	0
1	Ewa-RG-art_E	1		0	0
1		0		0	0
1		0		0	0
1		0		0	0
0		0		0	0
0		0		0	0
0		0		0	0
0		0		0	0
1		0		0	0
1		0		0	0
0		0		0	0
0		0		0	0
1		0		0	0
1		0		0	0
1	Ewa-RG-art_E	1		0	0
1	Ewa-RG-art_E	1		0	0
1	Ewa-RG-art_E	1		0	0
0		0		0	0
0		0		0	0
0		0		0	0
0		0		0	0
1	Ewa-RG-art_E	1		0	0
1		0		0	0
1		0		0	0
1	Ewa-RG-art_E	1		0	0
1	Ewa-RG-art_E	1		0	0
1	Ewa-RG-art_E	1		0	0
1	Ewa-RG-art_E	1		0	0
1	Ewa-RG-art_E	1		0	0
1	Ewa-RG-art_E	1		0	0
1	Ewa-RG-art_E	1		0	0
1		0		0	0
1		0		0	0
1		0		0	0
0		0		0	0
1	Ewa-RG-art_E	1		0	0
0		0		0	0
0		0		0	0
1	Ewa-RG-art_E	1		0	0
1	Ewa-RG-art_E	1		0	0
1	Ewa-RG-art_E	1		0	0
0		0		0	0
0		0	Ew-AU->in-RG	1	0
0		0		0	0

0		0		0		0
1	Ewa-RG-art_E	1		0		0
1	Ewa-RG-art_E	1		0		0
1	Ewa-RG-art_E	1		0		0
1	Ewa-RG-art_E	1		0		0
1	Ewa-RG-art_E	1		0		0
1	Ewa-RG-art_E	1	Ew-AU->in-RG	1		0
1	Ewa-RG-art_E	1	Ew-AU->in-RG	1		0
1	Ewa-RG-art_E	1		0		0
0		0		0		0
0		0		0		0
0		0		0		0
0		0		0		0
0		0		0		0
1	Ewa-RG-art_E	1		0		0
0		0		0		0
0		0		0		0
0		0		0		0
0		0		0		0
1	Ewa-RG-art_E	1		0		0
1	Ewa-RG-art_E	1		0		0
1	Ewa-RG-art_E	1		0		0
1	Ewa-RG-art_E	1		0		0
1	Ewa-RG-art_E	1		0		0
0		0		0		0
0		0		0		0
1	Ewa-RG-art_E	1		0		0
1	Ewa-RG-art_E	1		0		0
1	Ewa-RG-art_E	1		0		0
1	Ewa-RG-art_E	1		0		0
0		0		0		0
1	Ewa-RG-art_E	1	Ew-AU->in-RG	1	Ew-AU->in-RG	1
1	Ewa-RG-art_E	1		0		0
1	Ewa-RG-art_E	1		0		0
1	Ewa-RG-art_E	1		0		0
1	Ewa-RG-art_E	1		0		0
1	Ewa-RG-art_E	1		0		0
1	Ewa-RG-art_E	1		0		0
1	Ewa-RG-art_E	1		0		0
1		0	Ew-AU->in-RG	1		0
1		0	Ew-AU->in-RG	1		0
1	Ewa-RG-art_E	1		0		0
1	Ewa-RG-art_E	1		0		0
1		0		0		0
1		0		0		0
1	Ewa-RG-art_E	1		0		0
1	Ewa-RG-art_E	1		0		0

1	Ewa-RG-art_E	1		0		0
1	Ewa-RG-art_E	1		0		0
1	Ewa-RG-art_E	1		0		0
1	Ewa-RG-art_E	1		0		0
0		0		0		0
1	Ewa-RG-art_E	1		0		0
0		0		0		0
0		0	Ew-AU->in-RG	1		0
0		0		0		0
0		0		0		0
1	Ewa-RG-art_E	1		0		0
1	Ewa-RG-art_E	1	Ew-AU->in-RG	1	Ew-AU->in-RG	1
1	Ewa-RG-art_E	1	Ew-AU->in-RG	1	Ew-AU->in-RG	1
1	Ewa-RG-art_E	1		0		0
1	Ewa-RG-art_E	1		0		0
0		0		0		0
1	Ewa-RG-art_E	1		0		0
1	Ewa-RG-art_E	1		0		0
1	Ewa-RG-art_E	1		0		0
0		0		0		0
1	Ewa-RG-art_E	1		0		0
1	Ewa-RG-art_E	1		0		0
1	Ewa-RG-art_E	1		0		0
1	Ewa-RG-art_E	1		0		0
0		0		0		0
1	Ewa-RG-art_E	1		0		0
1	Ewa-RG-art_E	1		0		0
0		0		0		0
1	Ewa-RG-art_E	1		0		0
1	Ewa-RG-art_E	1	Ew-AU->in-RG	1	Ew-AU->in-RG	1
1	Ewa-RG-art_E	1	Ew-AU->in-RG	1	Ew-AU->in-RG	1
0		0		0		0
1		0		0		0
1		0		0		0
1	Ewa-RG-art_E	1		0		0
0		0		0		0
1	Ewa-RG-art_E	1		0		0
0		0		0		0
1	Ewa-RG-art_E	1		0		0
1		0		0		0
1		0		0		0
1	Ewa-RG-art_E	1		0		0
1	Ewa-RG-art_E	1		0		0
0		0		0		0
0		0		0		0
0		0		0		0
0		0		0		0

0		0		0		0
0		0		0		0
1		0		0		0
0		0	Ew-AU->in-RG	1		0
0		0	Ew-AU->in-RG	1		0
1		0		0		0
0		0		0		0
0		0		0		0
0		0		0		0
1	Ewa-RG-art_E	1		0		0
0		0		0		0
1	Ewa-RG-art_E	1	Ew-AU->in-RG	1	Ew-AU->in-RG	1
1	Ewa-RG-art_E	1	Ew-AU->in-RG	1	Ew-AU->in-RG	1
1	Ewa-RG-art_E	1	Ew-AU->in-RG	1	Ew-AU->in-RG	1
0		0		0		0
1	Ewa-RG-art_E	1		0		0
1	Ewa-RG-art_E	1		0		0
1	Ewa-RG-art_E	1	Ew-AU->in-RG	1	Ew-AU->in-RG	1
1		0		0		0
0		0		0		0
0		0		0		0
0		0		0		0
1	Ewa-RG-art_E	1		0		0
1	Ewa-RG-art_E	1		0		0
1	Ewa-RG-art_E	1	Ew-AU->in-RG	1	Ew-AU->in-RG	1
1	Ewa-RG-art_E	1		0		0
1	Ewa-RG-art_E	1		0		0
0		0		0		0
1	Ewa-RG-art_E	1		0		0
0		0		0		0
0		0		0		0
1	Ewa-RG-art_E	1		0		0
1	Ewa-RG-art_E	1		0		0
0		0		0		0
0		0		0		0
0		0	Ew-AU->in-RG	1	Ew-AU->in-RG	1
1	Ewa-RG-art_E	1		0		0
1	Ewa-RG-art_E	1		0		0
1	Ewa-RG-art_E	1		0		0
1	Ewa-RG-art_E	1		0		0
0		0		0		0
1	Ewa-RG-art_E	1		0		0
0		0		0		0
0		0		0		0
1		0		0		0
1		0		0		0
1	Ewa-RG-art_E	1		0		0

1	Ewa-RG-art_E	1		0		0
0		0		0		0
0		0	Ew-AU->in-RG	1		0
1	Ewa-RG-art_E	1		0		0
1	Ewa-RG-art_E	1		0		0
0		0		0		0
1	Ewa-RG-art_E	1		0		0
0		0		0		0
1	Ewa-RG-art_E	1	Ew-AU->in-RG	1	Ew-AU->in-RG	1
1	Ewa-RG-art_E	1	Ew-AU->in-RG	1	Ew-AU->in-RG	1
1	Ewa-RG-art_E	1	Ew-AU->in-RG	1	Ew-AU->in-RG	1
1	Ewa-RG-art_E	1		0		0
1	Ewa-RG-art_E	1		0		0
1	Ewa-RG-art_E	1	Ew-AU->in-RG	1	Ew-AU->in-RG	1
1	Ewa-RG-art_E	1	Ew-AU->in-RG	1	Ew-AU->in-RG	1
1	Ewa-RG-art_E	1		0		0
1		0		0		0
0		0		0		0
1		0		0		0
1		0		0		0
1	Ewa-RG-art_E	1		0		0
1	Ewa-RG-art_E	1		0		0
0		0		0		0
0		0		0		0
0		0		0		0
1	Ewa-RG-art_E	1		0		0
1	Ewa-RG-art_E	1		0		0
1	Ewa-RG-art_E	1		0		0
1	Ewa-RG-art_E	1		0		0
1	Ewa-RG-art_E	1		0		0
1	Ewa-RG-art_E	1		0		0
1	Ewa-RG-art_E	1		0		0
1		0		0		0
0		0		0		0
0		0		0		0
1	Ewa-RG-art_E	1		0		0
1	Ewa-RG-art_E	1		0		0
0		0		0		0
1	Ewa-RG-art_E	1		0		0
0		0		0		0
1	Ewa-RG-art_E	1		0		0
0		0		0		0
0		0		0		0
1	Ewa-RG-art_E	1		0		0
1	Ewa-RG-art_E	1		0		0
1	Ewa-RG-art_E	1		0		0
1	Ewa-RG-art_E	1		0		0

1	Ewa-RG-art_E	1		0		0
1	Ewa-RG-art_E	1		0		0
1	Ewa-RG-art_E	1		0		0
0		0		0		0
1	Ewa-RG-art_E	1	Ew-AU->in-RG	1	Ew-AU->in-RG	1
1	Ewa-RG-art_E	1	Ew-AU->in-RG	1	Ew-AU->in-RG	1
1	Ewa-RG-art_E	1	Ew-AU->in-RG	1	Ew-AU->in-RG	1
1	Ewa-RG-art_E	1		0		0
1	Ewa-RG-art_E	1		0		0
1	Ewa-RG-art_E	1		0		0
1	Ewa-RG-art_E	1		0		0
1	Ewa-RG-art_E	1		0		0
1	Ewa-RG-art_E	1		0		0
1	Ewa-RG-art_E	1		0		0
1	Ewa-RG-art_E	1		0		0
1	Ewa-RG-art_E	1		0		0
1	Ewa-RG-art_E	1		0		0
1	Ewa-RG-art_E	1		0		0
0		0		0		0
1	Ewa-RG-art_E	1		0		0
1	Ewa-RG-art_E	1		0		0
1	Ewa-RG-art_E	1		0		0
1	Ewa-RG-art_E	1		0		0
1	Ewa-RG-art_E	1		0		0
1	Ewa-RG-art_E	1		0		0
1	Ewa-RG-art_E	1		0		0
1	Ewa-RG-art_E	1		0		0
1	Ewa-RG-art_E	1		0		0
0		0		0		0
1	Ewa-RG-art_E	1		0		0
1		0		0		0
1		0		0		0
1		0		0		0
1		0		0		0
1	Ewa-RG-art_E	1		0		0
1	Ewa-RG-art_E	1		0		0
1	Ewa-RG-art_E	1		0		0
0		0		0		0
1	Ewa-RG-art_E	1		0		0
1	Ewa-RG-art_E	1		0		0
1	Ewa-RG-art_E	1		0		0
1	Ewa-RG-art_E	1		0		0
1	Ewa-RG-art_E	1		0		0
1	Ewa-RG-art_E	1		0		0
0		0		0		0
1		0		0		0
1		0		0		0

0		0		0		0
0		0		0		0
0		0		0		0
0		0		0		0
1	Ewa-RG-art_E	1		0		0
1	Ewa-RG-art_E	1		0		0
1	Ewa-RG-art_E	1		0		0
1	Ewa-RG-art_E	1		0		0
1	Ewa-RG-art_E	1		0		0
1	Ewa-RG-art_E	1	Ew-AU->in-RG	1	Ew-AU->in-RG	1
1	Ewa-RG-art_E	1		0		0
1	Ewa-RG-art_E	1		0		0
1	Ewa-RG-art_E	1		0		0
1	Ewa-RG-art_E	1		0		0
1	Ewa-RG-art_E	1		0		0
1	Ewa-RG-art_E	1		0		0
1	Ewa-RG-art_E	1		0		0
0		0		0		0
1	Ewa-RG-art_E	1	Ew-AU->in-RG	1	Ew-AU->in-RG	1
1	Ewa-RG-art_E	1	Ew-AU->in-RG	1	Ew-AU->in-RG	1
1	Ewa-RG-art_E	1		0		0
1		0		0		0
1		0		0		0
1		0		0		0
0		0		0		0
1	Ewa-RG-art_E	1		0		0
0		0		0		0
0		0		0		0
0		0		0		0
0		0		0		0
1	Ewa-RG-art_E	1		0		0
1		0		0		0
1		0		0		0
1		0		0		0
1	Ewa-RG-art_E	1		0		0
1	Ewa-RG-art_E	1		0		0
0		0		0		0
1	Ewa-RG-art_E	1		0		0
1	Ewa-RG-art_E	1		0		0
0		0		0		0
0		0		0		0
1	Ewa-RG-art_E	1		0		0
1	Ewa-RG-art_E	1		0		0
0		0		0		0
1	Ewa-RG-art_E	1		0		0
1		0		0		0
1	Ewa-RG-art_E	1	Ew-AU->in-RG	1	Ew-AU->in-RG	1

0		0		0		0
0		0		0		0
0		0		0		0
0		0		0		0
0		0		0		0
1	Ewa-RG-art_E	1		0		0
1	Ewa-RG-art_E	1		0		0
1	Ewa-RG-art_E	1	Ew-AU->in-RG	1	Ew-AU->in-RG	1
1	Ewa-RG-art_E	1	Ew-AU->in-RG	1	Ew-AU->in-RG	1
1	Ewa-RG-art_E	1		0		0
1	Ewa-RG-art_E	1		0		0
1	Ewa-RG-art_E	1		0		0
0		0		0		0
0		0	Ew-AU->in-RG	1		0
0		0		0		0
0		0		0		0
1	Ewa-RG-art_E	1		0		0
1	Ewa-RG-art_E	1		0		0
1	Ewa-RG-art_E	1		0		0
1	Ewa-RG-art_E	1		0		0
1	Ewa-RG-art_E	1		0		0
1	Ewa-RG-art_E	1		0		0
1	Ewa-RG-art_E	1		0		0
1	Ewa-RG-art_E	1		0		0
1	Ewa-RG-art_E	1		0		0
1	Ewa-RG-art_E	1		0		0
1	Ewa-RG-art_E	1		0		0
0		0		0		0
1	Ewa-RG-art_E	1		0		0
1	Ewa-RG-art_E	1		0		0
0		0		0		0
1	Ewa-RG-art_E	1		0		0
0		0		0		0
0		0		0		0
1	Ewa-RG-art_E	1		0		0
1	Ewa-RG-art_E	1		0		0
0		0		0		0
1	Ewa-RG-art_E	1		0		0
1	Ewa-RG-art_E	1		0		0
1	Ewa-RG-art_E	1		0		0
1	Ewa-RG-art_E	1		0		0
0		0		0		0
1	Ewa-RG-art_E	1		0		0
0		0		0		0
0		0		0		0
1	Ewa-RG-art_E	1		0		0
0		0		0		0

1	Ewa-RG-art_E	1		0		0
1	Ewa-RG-art_E	1		0		0
1	Ewa-RG-art_E	1		0		0
0		0		0		0
1	Ewa-RG-art_E	1		0		0
1	Ewa-RG-art_E	1		0		0
1	Ewa-RG-art_E	1		0		0
1	Ewa-RG-art_E	1		0		0
0		0		0		0
1	Ewa-RG-art_E	1		0		0
1	Ewa-RG-art_E	1		0		0
1	Ewa-RG-art_E	1		0		0
0		0		0		0
0		0		0		0
1		0		0		0
1		0		0		0
0		0		0		0
0		0		0		0
1	Ewa-RG-art_E	1		0		0
1	Ewa-RG-art_E	1		0		0
0		0		0		0
1		0		0		0
1		0		0		0
1		0		0		0
1		0		0		0
1	Ewa-RG-art_E	1		0		0
0		0		0		0
0		0		0		0
0		0		0		0
0		0		0		0
1	Ewa-RG-art_E	1		0		0
1	Ewa-RG-art_E	1		0		0
1	Ewa-RG-art_E	1		0		0
1	Ewa-RG-art_E	1		0		0
0		0		0		0
0		0		0		0
0		0		0		0
0		0		0		0
0		0		0		0
0		0		0		0
0		0		0		0
1		0		0		0
1		0		0		0
1	Ewa-RG-art_E	1		0		0
1	Ewa-RG-art_E	1		0		0
0		0		0		0
1	Ewa-RG-art_E	1	Ew-AU->in-RG	1	Ew-AU->in-RG	1

1	Ewa-RG-art_E	1	Ew-AU->in-RG	1	Ew-AU->in-RG	1
0		0		0		0
1		0	Ew-AU->in-RG	1		0
1		0	Ew-AU->in-RG	1		0
0		0		0		0
0		0		0		0
1		0		0		0
1		0		0		0
1	Ewa-RG-art_E	1		0		0
1	Ewa-RG-art_E	1		0		0
0		0		0		0
0		0		0		0
0		0		0		0
1	Ewa-RG-art_E	1		0		0
1	Ewa-RG-art_E	1		0		0
1	Ewa-RG-art_E	1		0		0
1	Ewa-RG-art_E	1		0		0
1	Ewa-RG-art_E	1		0		0
1	Ewa-RG-art_E	1		0		0
1	Ewa-RG-art_E	1		0		0
0		0		0		0
0		0		0		0
1	Ewa-RG-art_E	1		0		0
1	Ewa-RG-art_E	1		0		0
1	Ewa-RG-art_E	1		0		0
1	Ewa-RG-art_E	1		0		0
0		0		0		0
1	Ewa-RG-art_E	1		0		0
1	Ewa-RG-art_E	1		0		0
1	Ewa-RG-art_E	1		0		0
0		0		0		0
0		0		0		0
0		0	Ew-AU->in-RG	1		0
1	Ewa-RG-art_E	1		0		0
0		0		0		0
1	Ewa-RG-art_E	1		0		0
1	Ewa-RG-art_E	1		0		0
0		0		0		0
0		0		0		0
1	Ewa-RG-art_E	1		0		0
1	Ewa-RG-art_E	1		0		0
1	Ewa-RG-art_E	1		0		0
1	Ewa-RG-art_E	1		0		0
1	Ewa-RG-art_E	1		0		0
1	Ewa-RG-art_E	1		0		0
0		0		0		0
0		0		0		0

1	Ewa-RG-art_E	1		0	0
0		0		0	0
0		0		0	0
1	Ewa-RG-art_E	1		0	0
1	Ewa-RG-art_E	1		0	0
1		0		0	0
1		0		0	0
1	Ewa-RG-art_E	1		0	0
1	Ewa-RG-art_E	1		0	0
0		0		0	0
1	Ewa-RG-art_E	1		0	0
1	Ewa-RG-art_E	1		0	0
0		0	Ew-AU->in-RG	1	0
1		0		0	0
1		0		0	0
1	Ewa-RG-art_E	1		0	0
1	Ewa-RG-art_E	1		0	0
1	Ewa-RG-art_E	1		0	0
1	Ewa-RG-art_E	1		0	0
1	Ewa-RG-art_E	1		0	0
0		0		0	0
1	Ewa-RG-art_E	1		0	0
0		0		0	0
1	Ewa-RG-art_E	1		0	0
1	Ewa-RG-art_E	1		0	0
0		0	Ew-AU->in-RG	1	0
1	Ewa-RG-art_E	1		0	0
0		0		0	0
0		0		0	0
1	Ewa-RG-art_E	1		0	0
1	Ewa-RG-art_E	1		0	0
1	Ewa-RG-art_E	1		0	0
1	Ewa-RG-art_E	1		0	0
1	Ewa-RG-art_E	1		0	0
1	Ewa-RG-art_E	1		0	0
1	Ewa-RG-art_E	1		0	0
0		0		0	0
0		0	Ew-AU->in-RG	1	0
0		0	Ew-AU->in-RG	1	0
0		0		0	0
0		0		0	0
1		0		0	0
1	Ewa-RG-art_E	1		0	0
1	Ewa-RG-art_E	1		0	0
1	Ewa-RG-art_E	1		0	0
1	Ewa-RG-art_E	1		0	0
1	Ewa-RG-art_E	1		0	0

0		0	Ew-AU->in-RG	1		0
0		0		0		0
1	Ewa-RG-art_E	1	Ew-AU->in-RG	1	Ew-AU->in-RG	1
1	Ewa-RG-art_E	1		0		0
0		0		0		0
0		0		0		0
0		0		0		0
1		0		0		0
0		0		0		0
1	Ewa-RG-art_E	1		0		0
1	Ewa-RG-art_E	1		0		0
1	Ewa-RG-art_E	1		0		0
1	Ewa-RG-art_E	1		0		0
0		0		0		0
1	Ewa-RG-art_E	1		0		0
1	Ewa-RG-art_E	1		0		0
1	Ewa-RG-art_E	1		0		0
0		0		0		0
0		0		0		0
1	Ewa-RG-art_E	1		0		0
1	Ewa-RG-art_E	1		0		0
1	Ewa-RG-art_E	1		0		0
1	Ewa-RG-art_E	1		0		0
1		0		0		0
1	Ewa-RG-art_E	1		0		0
1	Ewa-RG-art_E	1		0		0
1	Ewa-RG-art_E	1	Ew-AU->in-RG	1	Ew-AU->in-RG	1
1	Ewa-RG-art_E	1		0		0
1	Ewa-RG-art_E	1		0		0
0		0		0		0
1	Ewa-RG-art_E	1		0		0
1	Ewa-RG-art_E	1		0		0
1	Ewa-RG-art_E	1		0		0
1	Ewa-RG-art_E	1		0		0
0		0		0		0
0		0		0		0
0		0		0		0
1	Ewa-RG-art_E	1		0		0
1	Ewa-RG-art_E	1		0		0
1	Ewa-RG-art_E	1		0		0
1		0	Ew-AU->in-RG	1		0
0		0		0		0
0		0	Ew-AU->in-RG	1		0
1	Ewa-RG-art_E	1		0		0
1	Ewa-RG-art_E	1		0		0
1	Ewa-RG-art_E	1		0		0
1	Ewa-RG-art_E	1		0		0

0		0		0		0
1	Ewa-RG-art_E	1		0		0
1	Ewa-RG-art_E	1		0		0
1	Ewa-RG-art_E	1		0		0
0		0		0		0
1	Ewa-RG-art_E	1		0		0
1	Ewa-RG-art_E	1		0		0
1	Ewa-RG-art_E	1		0		0
1	Ewa-RG-art_E	1	Ew-AU->in-RG	1	Ew-AU->in-RG	1
1	Ewa-RG-art_E	1	Ew-AU->in-RG	1	Ew-AU->in-RG	1
1	Ewa-RG-art_E	1		0		0
1	Ewa-RG-art_E	1		0		0
0		0		0		0
0		0		0		0
0		0		0		0
0		0		0		0
1	Ewa-RG-art_E	1	Ew-AU->in-RG	1	Ew-AU->in-RG	1
0		0		0		0
1	Ewa-RG-art_E	1		0		0
1	Ewa-RG-art_E	1		0		0
1	Ewa-RG-art_E	1		0		0
1	Ewa-RG-art_E	1		0		0
1	Ewa-RG-art_E	1		0		0
1	Ewa-RG-art_E	1		0		0
1	Ewa-RG-art_E	1		0		0
0		0		0		0
1		0		0		0
0		0		0		0
1	Ewa-RG-art_E	1	Ew-AU->in-RG	1	Ew-AU->in-RG	1
1		0		0		0
1		0		0		0
1		0		0		0
1		0		0		0
1		0		0		0
0		0		0		0
1	Ewa-RG-art_E	1		0		0
1	Ewa-RG-art_E	1		0		0
1	Ewa-RG-art_E	1	Ew-AU->in-RG	1	Ew-AU->in-RG	1
0		0		0		0
1	Ewa-RG-art_E	1		0		0
1	Ewa-RG-art_E	1		0		0
1	Ewa-RG-art_E	1		0		0
0		0		0		0
0		0		0		0
0		0		0		0
1	Ewa-RG-art_E	1		0		0
1	Ewa-RG-art_E	1		0		0

1	Ewa-RG-art_E	1		0		0
1	Ewa-RG-art_E	1		0		0
1	Ewa-RG-art_E	1		0		0
1	Ewa-RG-art_E	1		0		0
1	Ewa-RG-art_E	1		0		0
0		0		0		0
0		0		0		0
1	Ewa-RG-art_E	1		0		0
1		0		0		0
1		0		0		0
1	Ewa-RG-art_E	1		0		0
1	Ewa-RG-art_E	1		0		0
1	Ewa-RG-art_E	1		0		0
0		0		0		0
1	Ewa-RG-art_E	1		0		0
0		0		0		0
0		0		0		0
1	Ewa-RG-art_E	1		0		0
1	Ewa-RG-art_E	1		0		0
1	Ewa-RG-art_E	1		0		0
1	Ewa-RG-art_E	1		0		0
1	Ewa-RG-art_E	1		0		0
1	Ewa-RG-art_E	1		0		0
1	Ewa-RG-art_E	1		0		0
0		0		0		0
0		0		0		0
1	Ewa-RG-art_E	1	Ew-AU->in-RG	1	Ew-AU->in-RG	1
1	Ewa-RG-art_E	1	Ew-AU->in-RG	1	Ew-AU->in-RG	1
1		0	Ew-AU->in-RG	1		0
1		0	Ew-AU->in-RG	1		0
1	Ewa-RG-art_E	1		0		0
1	Ewa-RG-art_E	1	Ew-AU->in-RG	1	Ew-AU->in-RG	1
0		0		0		0
1	Ewa-RG-art_E	1	Ew-AU->in-RG	1		0
0		0		0		0
1		0		0		0
1	Ewa-RG-art_E	1		0		0
1	Ewa-RG-art_E	1		0		0
1	Ewa-RG-art_E	1		0		0
1	Ewa-RG-art_E	1		0		0
1	Ewa-RG-art_E	1		0		0
1	Ewa-RG-art_E	1		0		0
1	Ewa-RG-art_E	1		0		0
0		0		0		0
0		0		0		0
0		0		0		0
0		0		0		0

0		0		0		0
1	Ewa-RG-art_E	1		0		0
1	Ewa-RG-art_E	1		0		0
1	Ewa-RG-art_E	1		0		0
0		0		0		0
1	Ewa-RG-art_E	1		0		0
0		0		0		0
1		0		0		0
1		0		0		0
1	Ewa-RG-art_E	1		0		0
0		0		0		0
0		0		0		0
0		0		0		0
1	Ewa-RG-art_E	1		0		0
1	Ewa-RG-art_E	1		0		0
1	Ewa-RG-art_E	1		0		0
1	Ewa-RG-art_E	1		0		0
1	Ewa-RG-art_E	1		0		0
1	Ewa-RG-art_E	1		0		0
1	Ewa-RG-art_E	1		0		0
1	Ewa-RG-art_E	1		0		0
1	Ewa-RG-art_E	1		0		0
1	Ewa-RG-art_E	1		0		0
0		0		0		0
0		0		0		0
1	Ewa-RG-art_E	1		0		0
1	Ewa-RG-art_E	1		0		0
1	Ewa-RG-art_E	1		0		0
1	Ewa-RG-art_E	1		0		0
1	Ewa-RG-art_E	1	Ew-AU->in-RG	1	Ew-AU->in-RG	1
1	Ewa-RG-art_E	1	Ew-AU->in-RG	1	Ew-AU->in-RG	1
1	Ewa-RG-art_E	1		0		0
0		0		0		0
0		0		0		0
1	Ewa-RG-art_E	1		0		0
1		0		0		0
1		0		0		0
0		0		0		0
1	Ewa-RG-art_E	1		0		0
1	Ewa-RG-art_E	1		0		0
1		0		0		0
1		0		0		0
0		0		0		0
1	Ewa-RG-art_E	1	Ew-AU->in-RG	1	Ew-AU->in-RG	1
1	Ewa-RG-art_E	1	Ew-AU->in-RG	1	Ew-AU->in-RG	1
1	Ewa-RG-art_E	1		0		0
1	Ewa-RG-art_E	1		0		0

1	Ewa-RG-art_E	1	Ew-AU->in-RG	1	Ew-AU->in-RG	1
0		0		0		0
0		0		0		0
1	Ewa-RG-art_E	1		0		0
0		0		0		0
0		0		0		0
0		0		0		0
0		0		0		0
1	Ewa-RG-art_E	1		0		0
1	Ewa-RG-art_E	1		0		0
1		0	Ew-AU->in-RG	1		0
1		0	Ew-AU->in-RG	1		0
1		0	Ew-AU->in-RG	1		0
1		0	Ew-AU->in-RG	1		0
0		0		0		0
0		0		0		0
1	Ewa-RG-art_E	1		0		0
1	Ewa-RG-art_E	1		0		0
1		0		0		0
1	Ewa-RG-art_E	1		0		0
1	Ewa-RG-art_E	1		0		0
1	Ewa-RG-art_E	1		0		0
1	Ewa-RG-art_E	1		0		0
0		0		0		0
1	Ewa-RG-art_E	1		0		0
0		0		0		0
0		0		0		0
0		0		0		0
1	Ewa-RG-art_E	1		0		0
1	Ewa-RG-art_E	1		0		0
1	Ewa-RG-art_E	1		0		0
1	Ewa-RG-art_E	1		0		0
1		0		0		0
1	Ewa-RG-art_E	1		0		0
1	Ewa-RG-art_E	1		0		0
1	Ewa-RG-art_E	1		0		0
1	Ewa-RG-art_E	1	Ew-AU->in-RG	1	Ew-AU->in-RG	1
0		0		0		0
1	Ewa-RG-art_E	1		0		0
1		0		0		0
1		0		0		0
1	Ewa-RG-art_E	1		0		0
1	Ewa-RG-art_E	1		0		0
1	Ewa-RG-art_E	1		0		0
0		0		0		0
0		0		0		0
0		0		0		0

0		0		0		0
1	Ewa-RG-art_E	1		0		0
0		0		0		0
1		0		0		0
1		0		0		0
0		0		0		0
0		0		0		0
0		0		0		0
1	Ewa-RG-art_E	1		0		0
1	Ewa-RG-art_E	1		0		0
1	Ewa-RG-art_E	1	Ew-AU->in-RG	1	Ew-AU->in-RG	1
1	Ewa-RG-art_E	1	Ew-AU->in-RG	1	Ew-AU->in-RG	1
1	Ewa-RG-art_E	1		0		0
1	Ewa-RG-art_E	1		0		0
1	Ewa-RG-art_E	1		0		0
1	Ewa-RG-art_E	1		0		0
1	Ewa-RG-art_E	1		0		0
1	Ewa-RG-art_E	1		0		0
1	Ewa-RG-art_E	1		0		0
1	Ewa-RG-art_E	1		0		0
1	Ewa-RG-art_E	1		0		0
1	Ewa-RG-art_E	1		0		0
1	Ewa-RG-art_E	1		0		0
0		0		0		0
0		0		0		0
0		0		0		0
1		0	Ew-AU->in-RG	1		0
1	Ewa-RG-art_E	1		0		0
1	Ewa-RG-art_E	1		0		0
1	Ewa-RG-art_E	1		0		0
1	Ewa-RG-art_E	1		0		0
0		0		0		0
0		0		0		0
1	Ewa-RG-art_E	1		0		0
1	Ewa-RG-art_E	1		0		0
1	Ewa-RG-art_E	1		0		0
1	Ewa-RG-art_E	1		0		0
1	Ewa-RG-art_E	1		0		0
1	Ewa-RG-art_E	1		0		0
1	Ewa-RG-art_E	1		0		0
1	Ewa-RG-art_E	1		0		0
0		0		0		0
0		0		0		0
0		0		0		0
0		0		0		0
1	Ewa-RG-art_E	1		0		0
0		0		0		0

1	Ewa-RG-art_E	1		0		0
1	Ewa-RG-art_E	1		0		0
1	Ewa-RG-art_E	1		0		0
1	Ewa-RG-art_E	1		0		0
1	Ewa-RG-art_E	1		0		0
1	Ewa-RG-art_E	1		0		0
1	Ewa-RG-art_E	1		0		0
1	Ewa-RG-art_E	1		0		0
1	Ewa-RG-art_E	1	Ew-AU->in-RG	1	Ew-AU->in-RG	1
0		0		0		0
0		0		0		0
1	Ewa-RG-art_E	1		0		0
1	Ewa-RG-art_E	1		0		0
1	Ewa-RG-art_E	1		0		0
0		0		0		0
1	Ewa-RG-art_E	1		0		0
1	Ewa-RG-art_E	1		0		0
0		0		0		0
1	Ewa-RG-art_E	1		0		0
1	Ewa-RG-art_E	1	Ew-AU->in-RG	1	Ew-AU->in-RG	1
0		0	Ew-AU->in-RG	1		0
0		0		0		0
1	Ewa-RG-art_E	1		0		0
1	Ewa-RG-art_E	1		0		0
0		0		0		0
0		0		0		0
1	Ewa-RG-art_E	1	Ew-AU->in-RG	1	Ew-AU->in-RG	1
1	Ewa-RG-art_E	1	Ew-AU->in-RG	1	Ew-AU->in-RG	1
0		0		0		0
1	Ewa-RG-art_E	1		0		0
1	Ewa-RG-art_E	1		0		0
1	Ewa-RG-art_E	1		0		0
1	Ewa-RG-art_E	1		0		0
0		0		0		0
1	Ewa-RG-art_E	1		0		0
1	Ewa-RG-art_E	1		0		0
1	Ewa-RG-art_E	1		0		0
0		0		0		0
0		0		0		0
1	Ewa-RG-art_E	1		0		0
1		0		0		0
1		0		0		0
1	Ewa-RG-art_E	1		0		0
1	Ewa-RG-art_E	1		0		0
1	Ewa-RG-art_E	1		0		0
1	Ewa-RG-art_E	1		0		0
1	Ewa-RG-art_E	1		0		0

0		0		0	0
0		0		0	0
1	Ewa-RG-art_E	1		0	0
0		0		0	0
1	Ewa-RG-art_E	1		0	0
1		0	Ew-AU->in-RG	1	0
1		0	Ew-AU->in-RG	1	0
0		0		0	0
1	Ewa-RG-art_E	1		0	0
1	Ewa-RG-art_E	1		0	0
1		0		0	0
1		0		0	0
0		0		0	0
1	Ewa-RG-art_E	1		0	0
1	Ewa-RG-art_E	1		0	0
1	Ewa-RG-art_E	1		0	0
1	Ewa-RG-art_E	1		0	0
1	Ewa-RG-art_E	1		0	0
1	Ewa-RG-art_E	1		0	0
0		0		0	0
0		0		0	0
1	Ewa-RG-art_E	1		0	0
0		0		0	0
0		0		0	0
1	Ewa-RG-art_E	1		0	0
0		0		0	0
1	Ewa-RG-art_E	1		0	0
1	Ewa-RG-art_E	1		0	0
0		0		0	0
1	Ewa-RG-art_E	1		0	0
1	Ewa-RG-art_E	1		0	0
1	Ewa-RG-art_E	1		0	0
1	Ewa-RG-art_E	1		0	0
1	Ewa-RG-art_E	1		0	0
1	Ewa-RG-art_E	1		0	0
0		0		0	0
0		0		0	0
1	Ewa-RG-art_E	1		0	0
1	Ewa-RG-art_E	1		0	0
1	Ewa-RG-art_E	1		0	0
1		0		0	0
1		0		0	0
1	Ewa-RG-art_E	1		0	0
1	Ewa-RG-art_E	1		0	0
1	Ewa-RG-art_E	1		0	0
1	Ewa-RG-art_E	1		0	0
1	Ewa-RG-art_E	1		0	0

1	Ewa-RG-art_E	1		0		0
0		0	Ew-AU->in-RG	1		0
1	Ewa-RG-art_E	1		0		0
0		0		0		0
1	Ewa-RG-art_E	1		0		0
1	Ewa-RG-art_E	1		0		0
1	Ewa-RG-art_E	1		0		0
0		0		0		0
1		0		0		0
1		0		0		0
1		0		0		0
1		0		0		0
0		0		0		0
0		0		0		0
0		0		0		0
1	Ewa-RG-art_E	1		0		0
1	Ewa-RG-art_E	1		0		0
1	Ewa-RG-art_E	1		0		0
1	Ewa-RG-art_E	1		0		0
1	Ewa-RG-art_E	1		0		0
1	Ewa-RG-art_E	1		0		0
0		0		0		0
0		0		0		0
1		0		0		0
1		0		0		0
1	Ewa-RG-art_E	1		0		0
1	Ewa-RG-art_E	1		0		0
1	Ewa-RG-art_E	1		0		0
1	Ewa-RG-art_E	1		0		0
1	Ewa-RG-art_E	1		0		0
1		0		0		0
0		0		0		0
1	Ewa-RG-art_E	1		0		0
1	Ewa-RG-art_E	1		0		0
1	Ewa-RG-art_E	1		0		0
1	Ewa-RG-art_E	1		0		0
1	Ewa-RG-art_E	1		0		0
1		0		0		0
1	Ewa-RG-art_E	1	Ew-AU->in-RG	1	Ew-AU->in-RG	1
1	Ewa-RG-art_E	1	Ew-AU->in-RG	1	Ew-AU->in-RG	1
1	Ewa-RG-art_E	1		0		0
1	Ewa-RG-art_E	1		0		0
1	Ewa-RG-art_E	1		0		0
0		0		0		0
0		0		0		0
1	Ewa-RG-art_E	1		0		0
1	Ewa-RG-art_E	1		0		0

1	Ewa-RG-art_E	1		0		0
1	Ewa-RG-art_E	1		0		0
1	Ewa-RG-art_E	1		0		0
1	Ewa-RG-art_E	1		0		0
1	Ewa-RG-art_E	1		0		0
1	Ewa-RG-art_E	1		0		0
1	Ewa-RG-art_E	1		0		0
1	Ewa-RG-art_E	1		0		0
1	Ewa-RG-art_E	1		0		0
1	Ewa-RG-art_E	1		0		0
0		0		0		0
1	Ewa-RG-art_E	1	Ew-AU->in-RG	1	Ew-AU->in-RG	1
0		0		0		0
0		0		0		0
0		0		0		0
1	Ewa-RG-art_E	1		0		0
1	Ewa-RG-art_E	1		0		0
0		0		0		0
0		0		0		0
0		0		0		0
1	Ewa-RG-art_E	1		0		0
1	Ewa-RG-art_E	1		0		0
0		0	Ew-AU->in-RG	1		0
0		0	Ew-AU->in-RG	1		0
0		0		0		0
1	Ewa-RG-art_E	1		0		0
0		0		0		0
0		0		0		0
1	Ewa-RG-art_E	1		0		0
0		0		0		0
0		0		0		0
1	Ewa-RG-art_E	1		0		0
1	Ewa-RG-art_E	1		0		0
1	Ewa-RG-art_E	1		0		0
1	Ewa-RG-art_E	1		0		0
1	Ewa-RG-art_E	1		0		0
1	Ewa-RG-art_E	1		0		0
0		0		0		0
0		0		0		0
1	Ewa-RG-art_E	1		0		0
1	Ewa-RG-art_E	1		0		0
1	Ewa-RG-art_E	1		0		0
1	Ewa-RG-art_E	1		0		0
0		0		0		0
0		0		0		0
1	Ewa-RG-art_E	1		0		0
1	Ewa-RG-art_E	1		0		0

1	Ewa-RG-art_E	1		0		0
1	Ewa-RG-art_E	1		0		0
1	Ewa-RG-art_E	1		0		0
1	Ewa-RG-art_E	1		0		0
1	Ewa-RG-art_E	1		0		0
0		0		0		0
0		0		0		0
1	Ewa-RG-art_E	1		0		0
0		0		0		0
0		0		0		0
0		0		0		0
1	Ewa-RG-art_E	1		0		0
0		0		0		0
1	Ewa-RG-art_E	1		0		0
1	Ewa-RG-art_E	1		0		0
1	Ewa-RG-art_E	1		0		0
1	Ewa-RG-art_E	1		0		0
1	Ewa-RG-art_E	1		0		0
1	Ewa-RG-art_E	1		0		0
1		0		0		0
1		0		0		0
1	Ewa-RG-art_E	1		0		0
1	Ewa-RG-art_E	1		0		0
0		0		0		0
1	Ewa-RG-art_E	1		0		0
1	Ewa-RG-art_E	1		0		0
1	Ewa-RG-art_E	1		0		0
1	Ewa-RG-art_E	1		0		0
1	Ewa-RG-art_E	1		0		0
0		0		0		0
0		0		0		0
0		0		0		0
1	Ewa-RG-art_E	1	Ew-AU->in-RG	1	Ew-AU->in-RG	1
0		0		0		0
1	Ewa-RG-art_E	1		0		0
1	Ewa-RG-art_E	1		0		0
1	Ewa-RG-art_E	1		0		0
0		0		0		0
0		0		0		0
1		0		0		0
1		0		0		0
0		0		0		0
1	Ewa-RG-art_E	1		0		0
1	Ewa-RG-art_E	1		0		0
1	Ewa-RG-art_E	1	Ew-AU->in-RG	1		0
0		0		0		0
1	Ewa-RG-art_E	1		0		0

1	Ewa-RG-art_E	1	Ew-AU->in-RG	1	Ew-AU->in-RG	1
1	Ewa-RG-art_E	1	Ew-AU->in-RG	1	Ew-AU->in-RG	1
1	Ewa-RG-art_E	1		0		0
1	Ewa-RG-art_E	1		0		0
1	Ewa-RG-art_E	1		0		0
1	Ewa-RG-art_E	1		0		0
1	Ewa-RG-art_E	1		0		0
0		0		0		0
1	Ewa-RG-art_E	1		0		0
1	Ewa-RG-art_E	1		0		0
0		0		0		0
1	Ewa-RG-art_E	1		0		0
1	Ewa-RG-art_E	1		0		0
0		0		0		0
1	Ewa-RG-art_E	1		0		0
1	Ewa-RG-art_E	1		0		0
0		0		0		0
1	Ewa-RG-art_E	1		0		0
1	Ewa-RG-art_E	1		0		0
1	Ewa-RG-art_E	1		0		0
1	Ewa-RG-art_E	1		0		0
0		0		0		0
1	Ewa-RG-art_E	1		0		0
0		0		0		0
1	Ewa-RG-art_E	1		0		0
1	Ewa-RG-art_E	1		0		0
0		0		0		0
0		0		0		0
0		0		0		0
0		0		0		0
0		0		0		0
1	Ewa-RG-art_E	1		0		0
1	Ewa-RG-art_E	1		0		0
0		0		0		0
1	Ewa-RG-art_E	1		0		0
0		0		0		0
1		0		0		0
1		0		0		0
1		0		0		0
1	Ewa-RG-art_E	1		0		0
0		0		0		0
1	Ewa-RG-art_E	1		0		0
1	Ewa-RG-art_E	1	Ew-AU->in-RG	1	Ew-AU->in-RG	1
0		0		0		0
1	Ewa-RG-art_E	1		0		0
1	Ewa-RG-art_E	1		0		0
0		0		0		0

1	Ewa-RG-art_E	1		0	0
0		0		0	0
0		0		0	0
0		0		0	0
0		0		0	0
1	Ewa-RG-art_E	1		0	0
1	Ewa-RG-art_E	1		0	0
1	Ewa-RG-art_E	1		0	0
0		0		0	0
1	Ewa-RG-art_E	1		0	0
1	Ewa-RG-art_E	1	Ew-AU->in-RG	1	0
1	Ewa-RG-art_E	1	Ew-AU->in-RG	1	0
1		0		0	0
1		0		0	0
1	Ewa-RG-art_E	1		0	0
1	Ewa-RG-art_E	1		0	0
1	Ewa-RG-art_E	1		0	0
1	Ewa-RG-art_E	1		0	0
1		0		0	0
0		0		0	0
0		0		0	0
1	Ewa-RG-art_E	1		0	0
0		0		0	0
1	Ewa-RG-art_E	1		0	0
1	Ewa-RG-art_E	1		0	0
0		0		0	0
1		0		0	0
1		0		0	0
0		0		0	0
1	Ewa-RG-art_E	1		0	0
1	Ewa-RG-art_E	1		0	0
0		0		0	0
0		0		0	0
0		0		0	0
0		0		0	0
0		0		0	0
0		0		0	0
1		0		0	0
0		0		0	0
0		0	Ew-AU->in-RG	1	0
1	Ewa-RG-art_E	1		0	0
1	Ewa-RG-art_E	1		0	0
1	Ewa-RG-art_E	1		0	0
1	Ewa-RG-art_E	1		0	0
1	Ewa-RG-art_E	1		0	0
1	Ewa-RG-art_E	1		0	0
1	Ewa-RG-art_E	1		0	0

1	Ewa-RG-art_E	1		0		0
1	Ewa-RG-art_E	1		0		0
1	Ewa-RG-art_E	1		0		0
1		0		0		0
1		0		0		0
1	Ewa-RG-art_E	1		0		0
1	Ewa-RG-art_E	1		0		0
0		0		0		0
0		0		0		0
0		0		0		0
0		0		0		0
1	Ewa-RG-art_E	1		0		0
1	Ewa-RG-art_E	1	Ew-AU->in-RG	1	Ew-AU->in-RG	1
0		0		0		0
0		0		0		0
1	Ewa-RG-art_E	1		0		0
1	Ewa-RG-art_E	1	Ew-AU->in-RG	1	Ew-AU->in-RG	1
0		0		0		0
1	Ewa-RG-art_E	1		0		0
1	Ewa-RG-art_E	1		0		0
1	Ewa-RG-art_E	1		0		0
1	Ewa-RG-art_E	1		0		0
1	Ewa-RG-art_E	1		0		0
0		0		0		0
1	Ewa-RG-art_E	1		0		0
1	Ewa-RG-art_E	1		0		0
1	Ewa-RG-art_E	1		0		0
1	Ewa-RG-art_E	1	Ew-AU->in-RG	1	Ew-AU->in-RG	1
1	Ewa-RG-art_E	1	Ew-AU->in-RG	1	Ew-AU->in-RG	1
1	Ewa-RG-art_E	1		0		0
1	Ewa-RG-art_E	1		0		0
1	Ewa-RG-art_E	1	Ew-AU->in-RG	1		0
1	Ewa-RG-art_E	1	Ew-AU->in-RG	1		0
1	Ewa-RG-art_E	1		0		0
1	Ewa-RG-art_E	1		0		0
0		0		0		0
0		0		0		0
1	Ewa-RG-art_E	1		0		0
1	Ewa-RG-art_E	1		0		0
0		0		0		0
0		0		0		0
0		0		0		0
1	Ewa-RG-art_E	1		0		0
1	Ewa-RG-art_E	1		0		0
1	Ewa-RG-art_E	1		0		0
0		0		0		0
1	Ewa-RG-art_E	1		0		0

1	Ewa-RG-art_E	1		0		0
0		0		0		0
1		0		0		0
1		0		0		0
1		0		0		0
1		0		0		0
1	Ewa-RG-art_E	1	Ew-AU->in-RG	1	Ew-AU->in-RG	1
0		0		0		0
0		0		0		0
0		0		0		0
0		0		0		0
1	Ewa-RG-art_E	1	Ew-AU->in-RG	1	Ew-AU->in-RG	1
0		0		0		0
1	Ewa-RG-art_E	1		0		0
1	Ewa-RG-art_E	1		0		0
1	Ewa-RG-art_E	1		0		0
1		0		0		0
1		0		0		0
1		0		0		0
1		0		0		0
1	Ewa-RG-art_E	1		0		0
1	Ewa-RG-art_E	1		0		0
1	Ewa-RG-art_E	1		0		0
1	Ewa-RG-art_E	1		0		0
1	Ewa-RG-art_E	1		0		0
1	Ewa-RG-art_E	1		0		0
1	Ewa-RG-art_E	1		0		0
1	Ewa-RG-art_E	1		0		0
1	Ewa-RG-art_E	1		0		0
1	Ewa-RG-art_E	1		0		0
1	Ewa-RG-art_E	1		0		0
1	Ewa-RG-art_E	1		0		0
1	Ewa-RG-art_E	1		0		0
1	Ewa-RG-art_E	1		0		0
1	Ewa-RG-art_E	1		0		0
1	Ewa-RG-art_E	1		0		0
0		0		0		0
1	Ewa-RG-art_E	1		0		0
0		0		0		0
1	Ewa-RG-art_E	1		0		0
0		0		0		0
0		0		0		0
1	Ewa-RG-art_E	1		0		0
1	Ewa-RG-art_E	1		0		0
1	Ewa-RG-art_E	1		0		0
1	Ewa-RG-art_E	1		0		0
1	Ewa-RG-art_E	1		0		0

1	Ewa-RG-art_E	1		0		0
1	Ewa-RG-art_E	1		0		0
1	Ewa-RG-art_E	1	Ew-AU->in-RG	1	Ew-AU->in-RG	1
1	Ewa-RG-art_E	1	Ew-AU->in-RG	1	Ew-AU->in-RG	1
1	Ewa-RG-art_E	1	Ew-AU->in-RG	1	Ew-AU->in-RG	1
1	Ewa-RG-art_E	1		0		0
1	Ewa-RG-art_E	1		0		0
1	Ewa-RG-art_E	1		0		0
0		0		0		0
1	Ewa-RG-art_E	1		0		0
1	Ewa-RG-art_E	1		0		0
0		0		0		0
0		0		0		0
1	Ewa-RG-art_E	1		0		0
1	Ewa-RG-art_E	1		0		0
1	Ewa-RG-art_E	1		0		0
0		0		0		0
0		0		0		0
0		0		0		0
1	Ewa-RG-art_E	1		0		0
0		0		0		0
1	Ewa-RG-art_E	1		0		0
1	Ewa-RG-art_E	1		0		0
1	Ewa-RG-art_E	1		0		0
1	Ewa-RG-art_E	1		0		0
1	Ewa-RG-art_E	1		0		0
1	Ewa-RG-art_E	1		0		0
1	Ewa-RG-art_E	1		0		0
1	Ewa-RG-art_E	1		0		0
1	Ewa-RG-art_E	1		0		0
1	Ewa-RG-art_E	1		0		0
1	Ewa-RG-art_E	1		0		0
1	Ewa-RG-art_E	1		0		0
1	Ewa-RG-art_E	1		0		0
1	Ewa-RG-art_E	1		0		0
1	Ewa-RG-art_E	1		0		0
1	Ewa-RG-art_E	1		0		0
1		0		0		0
1		0		0		0
1	Ewa-RG-art_E	1		0		0
1	Ewa-RG-art_E	1	Ew-AU->in-RG	1	Ew-AU->in-RG	1
0		0		0		0
0		0		0		0
1	Ewa-RG-art_E	1		0		0
1	Ewa-RG-art_E	1		0		0
1	Ewa-RG-art_E	1		0		0
1		0		0		0

1		0		0		0
1	Ewa-RG-art_E	1	Ew-AU->in-RG	1	Ew-AU->in-RG	1
1	Ewa-RG-art_E	1		0		0
1	Ewa-RG-art_E	1		0		0
1	Ewa-RG-art_E	1		0		0
1	Ewa-RG-art_E	1		0		0
1	Ewa-RG-art_E	1		0		0
1		0		0		0
1		0		0		0
1		0		0		0
1		0		0		0
1	Ewa-RG-art_E	1		0		0
1	Ewa-RG-art_E	1		0		0
1	Ewa-RG-art_E	1		0		0
0		0		0		0
0		0		0		0
0		0		0		0
1	Ewa-RG-art_E	1		0		0
1	Ewa-RG-art_E	1		0		0
1	Ewa-RG-art_E	1		0		0
1	Ewa-RG-art_E	1		0		0
1		0		0		0
0		0		0		0
0		0		0		0
1	Ewa-RG-art_E	1		0		0
1	Ewa-RG-art_E	1		0		0
0		0		0		0
1		0		0		0
1	Ewa-RG-art_E	1	Ew-AU->in-RG	1	Ew-AU->in-RG	1
1	Ewa-RG-art_E	1	Ew-AU->in-RG	1	Ew-AU->in-RG	1
1		0		0		0
1		0		0		0
0		0		0		0
1		0		0		0
1		0		0		0
1	Ewa-RG-art_E	1		0		0
1	Ewa-RG-art_E	1		0		0
1	Ewa-RG-art_E	1		0		0
1	Ewa-RG-art_E	1		0		0
0		0		0		0
0		0		0		0
0		0		0		0
1	Ewa-RG-art_E	1		0		0
0		0	Ew-AU->in-RG	1		0
1	Ewa-RG-art_E	1		0		0
1	Ewa-RG-art_E	1		0		0
1	Ewa-RG-art_E	1		0		0

0		0		0		0
0		0		0		0
0		0		0		0
0		0		0		0
0		0		0		0
1	Ewa-RG-art_E	1		0		0
1	Ewa-RG-art_E	1		0		0
1	Ewa-RG-art_E	1		0		0
0		0		0		0
1	Ewa-RG-art_E	1		0		0
1	Ewa-RG-art_E	1	Ew-AU->in-RG	1	Ew-AU->in-RG	1
1	Ewa-RG-art_E	1	Ew-AU->in-RG	1	Ew-AU->in-RG	1
0		0		0		0
0		0		0		0
0		0		0		0
1	Ewa-RG-art_E	1		0		0
1	Ewa-RG-art_E	1		0		0
1	Ewa-RG-art_E	1		0		0
1	Ewa-RG-art_E	1		0		0
0		0		0		0
0		0		0		0
1	Ewa-RG-art_E	1		0		0
1	Ewa-RG-art_E	1		0		0
1	Ewa-RG-art_E	1		0		0
0		0		0		0
1		0	Ew-AU->in-RG	1		0
1		0	Ew-AU->in-RG	1		0
1		0		0		0
1		0		0		0
1		0		0		0
1		0		0		0
1	Ewa-RG-art_E	1		0		0
1	Ewa-RG-art_E	1		0		0
1	Ewa-RG-art_E	1		0		0
0		0		0		0
0		0		0		0
1	Ewa-RG-art_E	1		0		0
1	Ewa-RG-art_E	1	Ew-AU->in-RG	1	Ew-AU->in-RG	1
1	Ewa-RG-art_E	1	Ew-AU->in-RG	1	Ew-AU->in-RG	1
0		0		0		0
1	Ewa-RG-art_E	1		0		0
1	Ewa-RG-art_E	1		0		0
1	Ewa-RG-art_E	1		0		0
0		0		0		0
1	Ewa-RG-art_E	1		0		0
1	Ewa-RG-art_E	1		0		0
1	Ewa-RG-art_E	1		0		0

1	Ewa-RG-art_E	1		0		0
1	Ewa-RG-art_E	1		0		0
1	Ewa-RG-art_E	1		0		0
1		0		0		0
1		0		0		0
0		0		0		0
0		0		0		0
0		0		0		0
0		0		0		0
1	Ewa-RG-art_E	1		0		0
0		0		0		0
1	Ewa-RG-art_E	1	Ew-AU->in-RG	1	Ew-AU->in-RG	1
1	Ewa-RG-art_E	1		0		0
1	Ewa-RG-art_E	1		0		0
0		0		0		0
0		0		0		0
1	Ewa-RG-art_E	1		0		0
0		0		0		0
1	Ewa-RG-art_E	1		0		0
1	Ewa-RG-art_E	1		0		0
1	Ewa-RG-art_E	1		0		0
1	Ewa-RG-art_E	1		0		0
1	Ewa-RG-art_E	1		0		0
0		0		0		0
0		0		0		0
0		0		0		0
1	Ewa-RG-art_E	1		0		0
1	Ewa-RG-art_E	1		0		0
1	Ewa-RG-art_E	1		0		0
1	Ewa-RG-art_E	1		0		0
1	Ewa-RG-art_E	1		0		0
1	Ewa-RG-art_E	1		0		0
1	Ewa-RG-art_E	1		0		0
1	Ewa-RG-art_E	1		0		0
1	Ewa-RG-art_E	1		0		0
1	Ewa-RG-art_E	1		0		0
1	Ewa-RG-art_E	1		0		0
1	Ewa-RG-art_E	1		0		0
0		0		0		0
1	Ewa-RG-art_E	1		0		0
1	Ewa-RG-art_E	1		0		0
1	Ewa-RG-art_E	1		0		0
0		0		0		0
1	Ewa-RG-art_E	1		0		0
1	Ewa-RG-art_E	1		0		0
1	Ewa-RG-art_E	1		0		0
1	Ewa-RG-art_E	1		0		0

1	Ewa-RG-art_E	1		0		0
0		0		0		0
1	Ewa-RG-art_E	1		0		0
1	Ewa-RG-art_E	1		0		0
1	Ewa-RG-art_E	1		0		0
1	Ewa-RG-art_E	1		0		0
0		0		0		0
0		0		0		0
0		0		0		0
0		0		0		0
0		0		0		0
1	Ewa-RG-art_E	1		0		0
1	Ewa-RG-art_E	1		0		0
1	Ewa-RG-art_E	1		0		0
1	Ewa-RG-art_E	1		0		0
1	Ewa-RG-art_E	1		0		0
1	Ewa-RG-art_E	1		0		0
0		0		0		0
0		0		0		0
1	Ewa-RG-art_E	1		0		0
1		0		0		0
1		0		0		0
1	Ewa-RG-art_E	1		0		0
1	Ewa-RG-art_E	1	Ew-AU->in-RG	1	Ew-AU->in-RG	1
1	Ewa-RG-art_E	1		0		0
1	Ewa-RG-art_E	1		0		0
0		0		0		0
0		0		0		0
0		0		0		0
1	Ewa-RG-art_E	1	Ew-AU->in-RG	1	Ew-AU->in-RG	1
1	Ewa-RG-art_E	1		0		0
1	Ewa-RG-art_E	1		0		0
0		0		0		0
1	Ewa-RG-art_E	1	Ew-AU->in-RG	1		0
1	Ewa-RG-art_E	1	Ew-AU->in-RG	1		0
1	Ewa-RG-art_E	1		0		0
1	Ewa-RG-art_E	1		0		0
1		0	Ew-AU->in-RG	1		0
1		0	Ew-AU->in-RG	1		0
1	Ewa-RG-art_E	1		0		0
1	Ewa-RG-art_E	1		0		0
1	Ewa-RG-art_E	1		0		0
1	Ewa-RG-art_E	1		0		0
0		0		0		0
0		0		0		0
1	Ewa-RG-art_E	1		0		0
1	Ewa-RG-art_E	1		0		0

0		0		0		0
0		0		0		0
0		0		0		0
0		0		0		0
0		0		0		0
0		0		0		0
1	Ewa-RG-art_E	1	Ew-AU->in-RG	1	Ew-AU->in-RG	1
1	Ewa-RG-art_E	1		0		0
1	Ewa-RG-art_E	1		0		0
0		0		0		0
0		0		0		0
1	Ewa-RG-art_E	1		0		0
1	Ewa-RG-art_E	1		0		0
1	Ewa-RG-art_E	1		0		0
1	Ewa-RG-art_E	1		0		0
1	Ewa-RG-art_E	1		0		0
1	Ewa-RG-art_E	1		0		0
1	Ewa-RG-art_E	1		0		0
0		0		0		0
1	Ewa-RG-art_E	1		0		0
1	Ewa-RG-art_E	1		0		0
0		0		0		0
0		0		0		0
0		0		0		0
1	Ewa-RG-art_E	1		0		0
1	Ewa-RG-art_E	1		0		0
1	Ewa-RG-art_E	1		0		0
1	Ewa-RG-art_E	1		0		0
1	Ewa-RG-art_E	1		0		0
1	Ewa-RG-art_E	1		0		0
1	Ewa-RG-art_E	1		0		0
0		0		0		0
1	Ewa-RG-art_E	1		0		0
1	Ewa-RG-art_E	1		0		0
0		0		0		0
1	Ewa-RG-art_E	1		0		0
1	Ewa-RG-art_E	1		0		0
1	Ewa-RG-art_E	1		0		0
1	Ewa-RG-art_E	1		0		0
0		0		0		0
0		0		0		0
1	Ewa-RG-art_E	1		0		0
0		0		0		0
1	Ewa-RG-art_E	1		0		0
1	Ewa-RG-art_E	1		0		0
1	Ewa-RG-art_E	1		0		0
1	Ewa-RG-art_E	1		0		0

1	Ewa-RG-art_E	1		0		0
1	Ewa-RG-art_E	1		0		0
0		0		0		0
1	Ewa-RG-art_E	1		0		0
1	Ewa-RG-art_E	1		0		0
0		0		0		0
1	Ewa-RG-art_E	1		0		0
1	Ewa-RG-art_E	1		0		0
1	Ewa-RG-art_E	1		0		0
1	Ewa-RG-art_E	1		0		0
1	Ewa-RG-art_E	1		0		0
1	Ewa-RG-art_E	1		0		0
0		0		0		0
0		0		0		0
0		0	Ew-AU->in-RG	1		0
1	Ewa-RG-art_E	1		0		0
1	Ewa-RG-art_E	1		0		0
1	Ewa-RG-art_E	1		0		0
1	Ewa-RG-art_E	1		0		0
1	Ewa-RG-art_E	1		0		0
1	Ewa-RG-art_E	1		0		0
1	Ewa-RG-art_E	1		0		0
1	Ewa-RG-art_E	1	Ew-AU->in-RG	1	Ew-AU->in-RG	1
0		0		0		0
1	Ewa-RG-art_E	1		0		0
1	Ewa-RG-art_E	1		0		0
1	Ewa-RG-art_E	1		0		0
1	Ewa-RG-art_E	1	Ew-AU->in-RG	1		0
1	Ewa-RG-art_E	1		0		0
0		0		0		0
0		0		0		0
0		0	Ew-AU->in-RG	1		0
0		0	Ew-AU->in-RG	1		0
1	Ewa-RG-art_E	1		0		0
1	Ewa-RG-art_E	1		0		0
1	Ewa-RG-art_E	1		0		0
1	Ewa-RG-art_E	1	Ew-AU->in-RG	1		0
1	Ewa-RG-art_E	1	Ew-AU->in-RG	1		0
1	Ewa-RG-art_E	1	Ew-AU->in-RG	1		0
1	Ewa-RG-art_E	1		0		0
1		0		0		0
1		0		0		0
1		0		0		0
0		0		0		0
1		0	Ew-AU->in-RG	1		0
1		0	Ew-AU->in-RG	1		0
1		0	Ew-AU->in-RG	1		0

1	Ewa-RG-art_E	1		0		0
1	Ewa-RG-art_E	1		0		0
1	Ewa-RG-art_E	1		0		0
1	Ewa-RG-art_E	1		0		0
1	Ewa-RG-art_E	1		0		0
1	Ewa-RG-art_E	1		0		0
1	Ewa-RG-art_E	1	Ew-AU->in-RG	1	Ew-AU->in-RG	1
1	Ewa-RG-art_E	1		0		0
0		0		0		0
1		0		0		0
1		0		0		0
1	Ewa-RG-art_E	1		0		0
1	Ewa-RG-art_E	1		0		0
1	Ewa-RG-art_E	1		0		0
1	Ewa-RG-art_E	1		0		0
1	Ewa-RG-art_E	1		0		0
1	Ewa-RG-art_E	1		0		0
1	Ewa-RG-art_E	1		0		0
0		0		0		0
0		0		0		0
0		0		0		0
0		0		0		0
0		0		0		0
1	Ewa-RG-art_E	1		0		0
1	Ewa-RG-art_E	1		0		0
1	Ewa-RG-art_E	1		0		0
1	Ewa-RG-art_E	1		0		0
1	Ewa-RG-art_E	1		0		0
1	Ewa-RG-art_E	1		0		0
0		0		0		0
1	Ewa-RG-art_E	1		0		0
1	Ewa-RG-art_E	1		0		0
1	Ewa-RG-art_E	1		0		0
1	Ewa-RG-art_E	1		0		0
1	Ewa-RG-art_E	1		0		0
1	Ewa-RG-art_E	1		0		0
1	Ewa-RG-art_E	1		0		0
0		0		0		0
0		0		0		0
1	Ewa-RG-art_E	1		0		0
1	Ewa-RG-art_E	1		0		0
0		0		0		0
1	Ewa-RG-art_E	1		0		0
1	Ewa-RG-art_E	1	Ew-AU->in-RG	1		0
0		0		0		0
0		0		0		0
1		0		0		0

1		0		0		0
1	Ewa-RG-art_E	1		0		0
1	Ewa-RG-art_E	1		0		0
1	Ewa-RG-art_E	1		0		0
1	Ewa-RG-art_E	1		0		0
1	Ewa-RG-art_E	1		0		0
0		0		0		0
1	Ewa-RG-art_E	1		0		0
1	Ewa-RG-art_E	1		0		0
1	Ewa-RG-art_E	1		0		0
1	Ewa-RG-art_E	1	Ew-AU->in-RG	1	Ew-AU->in-RG	1
1	Ewa-RG-art_E	1	Ew-AU->in-RG	1	Ew-AU->in-RG	1
1	Ewa-RG-art_E	1	Ew-AU->in-RG	1	Ew-AU->in-RG	1
1	Ewa-RG-art_E	1		0		0
1	Ewa-RG-art_E	1	Ew-AU->in-RG	1	Ew-AU->in-RG	1
1	Ewa-RG-art_E	1	Ew-AU->in-RG	1		0
1	Ewa-RG-art_E	1	Ew-AU->in-RG	1		0
0		0		0		0
1	Ewa-RG-art_E	1		0		0
0		0		0		0
0		0		0		0
1	Ewa-RG-art_E	1		0		0
1	Ewa-RG-art_E	1		0		0
1	Ewa-RG-art_E	1		0		0
0		0		0		0
1	Ewa-RG-art_E	1		0		0
1		0		0		0
1	Ewa-RG-art_E	1		0		0
1	Ewa-RG-art_E	1		0		0
1	Ewa-RG-art_E	1		0		0
1	Ewa-RG-art_E	1		0		0
1	Ewa-RG-art_E	1		0		0
1	Ewa-RG-art_E	1		0		0
1	Ewa-RG-art_E	1	Ew-AU->in-RG	1	Ew-AU->in-RG	1
1	Ewa-RG-art_E	1	Ew-AU->in-RG	1	Ew-AU->in-RG	1
0		0		0		0
0		0		0		0
1	Ewa-RG-art_E	1		0		0
1	Ewa-RG-art_E	1		0		0
0		0		0		0
1	Ewa-RG-art_E	1	Ew-AU->in-RG	1	Ew-AU->in-RG	1
1	Ewa-RG-art_E	1		0		0
1	Ewa-RG-art_E	1		0		0
0		0		0		0
0		0		0		0
1	Ewa-RG-art_E	1		0		0
1	Ewa-RG-art_E	1	Ew-AU->in-RG	1	Ew-AU->in-RG	1

1	Ewa-RG-art_E	1	Ew-AU->in-RG	1	Ew-AU->in-RG	1
1		0		0		0
1		0		0		0
1	Ewa-RG-art_E	1		0		0
1	Ewa-RG-art_E	1		0		0
1	Ewa-RG-art_E	1		0		0
1	Ewa-RG-art_E	1		0		0
0		0		0		0
0		0		0		0
0		0		0		0
1	Ewa-RG-art_E	1		0		0
1	Ewa-RG-art_E	1		0		0
1	Ewa-RG-art_E	1		0		0
1	Ewa-RG-art_E	1		0		0
0		0		0		0
1	Ewa-RG-art_E	1		0		0
1	Ewa-RG-art_E	1		0		0
1	Ewa-RG-art_E	1		0		0
1	Ewa-RG-art_E	1		0		0
1	Ewa-RG-art_E	1		0		0
0		0		0		0
0		0		0		0
0		0		0		0
0		0		0		0
1	Ewa-RG-art_E	1		0		0
1		0		0		0
1		0		0		0
0		0		0		0
0		0		0		0
1		0		0		0
1		0		0		0
1		0		0		0
1	Ewa-RG-art_E	1		0		0
1	Ewa-RG-art_E	1		0		0
1	Ewa-RG-art_E	1		0		0
0		0		0		0
1	Ewa-RG-art_E	1		0		0
1	Ewa-RG-art_E	1		0		0
1	Ewa-RG-art_E	1		0		0
1	Ewa-RG-art_E	1		0		0
1	Ewa-RG-art_E	1		0		0
0		0		0		0
1	Ewa-RG-art_E	1		0		0
1	Ewa-RG-art_E	1		0		0
1	Ewa-RG-art_E	1		0		0
1	Ewa-RG-art_E	1		0		0
1	Ewa-RG-art_E	1		0		0

1	Ewa-RG-art_E	1		0		0
1	Ewa-RG-art_E	1		0		0
1	Ewa-RG-art_E	1		0		0
1	Ewa-RG-art_E	1		0		0
1	Ewa-RG-art_E	1		0		0
1	Ewa-RG-art_E	1		0		0
1	Ewa-RG-art_E	1		0		0
1	Ewa-RG-art_E	1		0		0
1	Ewa-RG-art_E	1		0		0
0		0		0		0
0		0		0		0
1		0		0		0
0		0		0		0
1	Ewa-RG-art_E	1		0		0
1	Ewa-RG-art_E	1		0		0
1	Ewa-RG-art_E	1		0		0
1	Ewa-RG-art_E	1		0		0
1	Ewa-RG-art_E	1		0		0
1	Ewa-RG-art_E	1		0		0
0		0		0		0
0		0		0		0
0		0		0		0
1	Ewa-RG-art_E	1	Ew-AU->in-RG	1	Ew-AU->in-RG	1
1	Ewa-RG-art_E	1	Ew-AU->in-RG	1	Ew-AU->in-RG	1
0		0		0		0
1	Ewa-RG-art_E	1	Ew-AU->in-RG	1	Ew-AU->in-RG	1
0		0		0		0
1	Ewa-RG-art_E	1		0		0
0		0		0		0
1		0		0		0
1	Ewa-RG-art_E	1		0		0
1	Ewa-RG-art_E	1		0		0
0		0		0		0
1	Ewa-RG-art_E	1		0		0
1	Ewa-RG-art_E	1		0		0
1	Ewa-RG-art_E	1	Ew-AU->in-RG	1	Ew-AU->in-RG	1
1	Ewa-RG-art_E	1		0		0
1	Ewa-RG-art_E	1		0		0
0		0		0		0
1	Ewa-RG-art_E	1		0		0
0		0		0		0
0		0		0		0
0		0		0		0
1		0		0		0
1		0		0		0
1		0		0		0
1		0		0		0

1	Ewa-RG-art_E	1		0		0
1	Ewa-RG-art_E	1		0		0
0		0		0		0
0		0		0		0
1	Ewa-RG-art_E	1		0		0
1	Ewa-RG-art_E	1		0		0
0		0	Ew-AU->in-RG	1		0
0		0		0		0
1	Ewa-RG-art_E	1		0		0
1	Ewa-RG-art_E	1	Ew-AU->in-RG	1	Ew-AU->in-RG	1
1	Ewa-RG-art_E	1	Ew-AU->in-RG	1	Ew-AU->in-RG	1
1	Ewa-RG-art_E	1		0		0
1	Ewa-RG-art_E	1		0		0
1	Ewa-RG-art_E	1		0		0
0		0	Ew-AU->in-RG	1		0
0		0	Ew-AU->in-RG	1		0
0		0	Ew-AU->in-RG	1		0
0		0	Ew-AU->in-RG	1		0
0		0		0		0
1	Ewa-RG-art_E	1	Ew-AU->in-RG	1	Ew-AU->in-RG	1
1	Ewa-RG-art_E	1	Ew-AU->in-RG	1	Ew-AU->in-RG	1
1		0		0		0
1	Ewa-RG-art_E	1		0		0
1	Ewa-RG-art_E	1		0		0
1	Ewa-RG-art_E	1		0		0
1	Ewa-RG-art_E	1		0		0
1	Ewa-RG-art_E	1		0		0
1		0	Ew-AU->in-RG	1		0
1	Ewa-RG-art_E	1		0		0
1	Ewa-RG-art_E	1		0		0
1	Ewa-RG-art_E	1		0		0
1	Ewa-RG-art_E	1		0		0
1	Ewa-RG-art_E	1	Ew-AU->in-RG	1		0
1	Ewa-RG-art_E	1	Ew-AU->in-RG	1		0
1	Ewa-RG-art_E	1		0		0
1	Ewa-RG-art_E	1		0		0
0		0		0		0
1	Ewa-RG-art_E	1		0		0
1	Ewa-RG-art_E	1		0		0
1	Ewa-RG-art_E	1		0		0
1	Ewa-RG-art_E	1		0		0
1	Ewa-RG-art_E	1		0		0
1	Ewa-RG-art_E	1		0		0
1	Ewa-RG-art_E	1		0		0
1	Ewa-RG-art_E	1		0		0
1	Ewa-RG-art_E	1		0		0
1	Ewa-RG-art_E	1		0		0

1	Ewa-RG-art_E	1		0		0
0		0		0		0
1	Ewa-RG-art_E	1	Ew-AU->in-RG	1	Ew-AU->in-RG	1
1	Ewa-RG-art_E	1	Ew-AU->in-RG	1	Ew-AU->in-RG	1
0		0		0		0
0		0		0		0
0		0		0		0
0		0		0		0
1		0		0		0
1		0		0		0
1		0		0		0
1		0		0		0
1		0		0		0
1	Ewa-RG-art_E	1		0		0
0		0		0		0
1	Ewa-RG-art_E	1		0		0
0		0		0		0
1	Ewa-RG-art_E	1		0		0
1	Ewa-RG-art_E	1		0		0
1	Ewa-RG-art_E	1		0		0
1	Ewa-RG-art_E	1		0		0
1	Ewa-RG-art_E	1		0		0
1	Ewa-RG-art_E	1		0		0
0		0		0		0
0		0		0		0
0		0		0		0
1	Ewa-RG-art_E	1		0		0
1	Ewa-RG-art_E	1		0		0
1	Ewa-RG-art_E	1		0		0
1	Ewa-RG-art_E	1		0		0
1	Ewa-RG-art_E	1		0		0
1	Ewa-RG-art_E	1		0		0
1	Ewa-RG-art_E	1		0		0
1	Ewa-RG-art_E	1		0		0
1	Ewa-RG-art_E	1		0		0
1	Ewa-RG-art_E	1		0		0
1	Ewa-RG-art_E	1		0		0
1	Ewa-RG-art_E	1		0		0
0		0		0		0
0		0		0		0
1	Ewa-RG-art_E	1		0		0
1	Ewa-RG-art_E	1		0		0
1	Ewa-RG-art_E	1		0		0
0		0		0		0
0		0		0		0
0		0		0		0
1		0		0		0

1		0		0		0
1	Ewa-RG-art_E	1	Ew-AU->in-RG	1	Ew-AU->in-RG	1
0		0	Ew-AU->in-RG	1		0
1	Ewa-RG-art_E	1		0		0
1	Ewa-RG-art_E	1	Ew-AU->in-RG	1	Ew-AU->in-RG	1
1	Ewa-RG-art_E	1	Ew-AU->in-RG	1	Ew-AU->in-RG	1
0		0		0		0
1	Ewa-RG-art_E	1		0		0
1	Ewa-RG-art_E	1		0		0
1	Ewa-RG-art_E	1		0		0
1	Ewa-RG-art_E	1		0		0
0		0		0		0
0		0		0		0
1		0		0		0
1		0		0		0
1		0		0		0
0		0		0		0
1	Ewa-RG-art_E	1	Ew-AU->in-RG	1		0
1	Ewa-RG-art_E	1		0		0
1	Ewa-RG-art_E	1		0		0
1	Ewa-RG-art_E	1	Ew-AU->in-RG	1	Ew-AU->in-RG	1
0		0		0		0
1		0		0		0
0		0		0		0
1	Ewa-RG-art_E	1	Ew-AU->in-RG	1	Ew-AU->in-RG	1
1		0		0		0
1	Ewa-RG-art_E	1		0		0
0		0		0		0
0		0		0		0
0		0		0		0
1	Ewa-RG-art_E	1	Ew-AU->in-RG	1	Ew-AU->in-RG	1
0		0		0		0
0		0		0		0
1	Ewa-RG-art_E	1		0		0
0		0		0		0
1	Ewa-RG-art_E	1		0		0
1	Ewa-RG-art_E	1		0		0
1	Ewa-RG-art_E	1		0		0
1	Ewa-RG-art_E	1		0		0
1	Ewa-RG-art_E	1		0		0
1	Ewa-RG-art_E	1	Ew-AU->in-RG	1	Ew-AU->in-RG	1
1	Ewa-RG-art_E	1	Ew-AU->in-RG	1	Ew-AU->in-RG	1
1	Ewa-RG-art_E	1	Ew-AU->in-RG	1	Ew-AU->in-RG	1
0		0	Ew-AU->in-RG	1		0
1	Ewa-RG-art_E	1		0		0
1	Ewa-RG-art_E	1		0		0
1	Ewa-RG-art_E	1		0		0

1	Ewa-RG-art_E	1		0		0
1		0		0		0
1		0		0		0
1		0		0		0
1		0		0		0
1		0		0		0
0		0		0		0
1	Ewa-RG-art_E	1	Ew-AU->in-RG	1	Ew-AU->in-RG	1
1	Ewa-RG-art_E	1	Ew-AU->in-RG	1	Ew-AU->in-RG	1
1	Ewa-RG-art_E	1		0		0
1	Ewa-RG-art_E	1		0		0
0		0		0		0
1	Ewa-RG-art_E	1		0		0
1		0		0		0
1		0		0		0
1		0		0		0
1	Ewa-RG-art_E	1		0		0
1	Ewa-RG-art_E	1		0		0
1	Ewa-RG-art_E	1		0		0
1	Ewa-RG-art_E	1		0		0
1	Ewa-RG-art_E	1		0		0
1	Ewa-RG-art_E	1		0		0
0		0		0		0
1	Ewa-RG-art_E	1		0		0
1	Ewa-RG-art_E	1		0		0
1	Ewa-RG-art_E	1	Ew-AU->in-RG	1		0
1	Ewa-RG-art_E	1	Ew-AU->in-RG	1		0
0		0		0		0
1	Ewa-RG-art_E	1		0		0
1	Ewa-RG-art_E	1		0		0
1	Ewa-RG-art_E	1		0		0
1	Ewa-RG-art_E	1		0		0
1		0		0		0
1		0		0		0
1		0		0		0
1		0		0		0
1		0		0		0
1	Ewa-RG-art_E	1		0		0
0		0		0		0
0		0		0		0
0		0		0		0
0		0		0		0
0		0		0		0
0		0		0		0
1	Ewa-RG-art_E	1		0		0
1	Ewa-RG-art_E	1		0		0
1	Ewa-RG-art_E	1		0		0

1	Ewa-RG-art_E	1		0		0
1	Ewa-RG-art_E	1	Ew-AU->in-RG	1	Ew-AU->in-RG	1
1	Ewa-RG-art_E	1		0		0
1	Ewa-RG-art_E	1		0		0
1	Ewa-RG-art_E	1		0		0
1	Ewa-RG-art_E	1		0		0
1	Ewa-RG-art_E	1		0		0
0		0		0		0
0		0		0		0
1	Ewa-RG-art_E	1		0		0
1	Ewa-RG-art_E	1		0		0
1	Ewa-RG-art_E	1		0		0
1		0		0		0
1		0		0		0
1		0		0		0
1	Ewa-RG-art_E	1		0		0
1	Ewa-RG-art_E	1		0		0
1	Ewa-RG-art_E	1		0		0
1	Ewa-RG-art_E	1		0		0
0		0		0		0
1		0		0		0
1		0		0		0
1		0		0		0
0		0		0		0
0		0		0		0
1	Ewa-RG-art_E	1		0		0
1	Ewa-RG-art_E	1		0		0
1	Ewa-RG-art_E	1		0		0
1	Ewa-RG-art_E	1		0		0
1	Ewa-RG-art_E	1		0		0
1	Ewa-RG-art_E	1		0		0
1	Ewa-RG-art_E	1		0		0
1	Ewa-RG-art_E	1		0		0
1	Ewa-RG-art_E	1		0		0
1	Ewa-RG-art_E	1		0		0
1	Ewa-RG-art_E	1		0		0
1	Ewa-RG-art_E	1		0		0
0		0		0		0
1	Ewa-RG-art_E	1		0		0
1		0		0		0
1		0		0		0
1		0		0		0
1	Ewa-RG-art_E	1		0		0
1	Ewa-RG-art_E	1		0		0
0		0		0		0
0		0		0		0
1	Ewa-RG-art_E	1		0		0

1	Ewa-RG-art_E	1		0		0
0		0		0		0
1		0		0		0
1		0		0		0
1	Ewa-RG-art_E	1		0		0
1	Ewa-RG-art_E	1		0		0
1	Ewa-RG-art_E	1		0		0
1	Ewa-RG-art_E	1	Ew-AU->in-RG	1	Ew-AU->in-RG	1
1	Ewa-RG-art_E	1	Ew-AU->in-RG	1	Ew-AU->in-RG	1
0		0		0		0
1	Ewa-RG-art_E	1		0		0
1	Ewa-RG-art_E	1	Ew-AU->in-RG	1		0
0		0		0		0
1	Ewa-RG-art_E	1		0		0
1	Ewa-RG-art_E	1		0		0
1	Ewa-RG-art_E	1		0		0
1	Ewa-RG-art_E	1		0		0
1	Ewa-RG-art_E	1		0		0
1	Ewa-RG-art_E	1		0		0
1	Ewa-RG-art_E	1		0		0
1	Ewa-RG-art_E	1		0		0
1	Ewa-RG-art_E	1		0		0
0		0		0		0
1		0		0		0
1		0		0		0
1	Ewa-RG-art_E	1		0		0
1	Ewa-RG-art_E	1		0		0
1	Ewa-RG-art_E	1		0		0
1	Ewa-RG-art_E	1		0		0
1	Ewa-RG-art_E	1		0		0
1	Ewa-RG-art_E	1		0		0
0		0		0		0

ewauinrgart_	ewauinrgart_	ewauinrg_pi_	ewauinrg_pi_	ewargart_au_	ewargart_au_	ewargart_au_	ewargart_au_
	0		0		0		0
	0		0		0		0
	0		0		0		0
	0		0		0		0
	0		0		0		0
	0		0		0		0
	0		0		0		0
	0		0		0		0
	0		0		0		0
	0		0		0		0
	0		0		0		0
	0		0		0		0
	0		0		0		0
	0		0		0		0
	0		0		0		0
	0		0		0		0
	0		0		0		0
	0		0		0		0
	0		0		0		0
	0		0		0		0
	0		0		0		0
	0		0		0		0
	0		0		0		0
	0		0		0		0
	0		0		0		0
	0		0		0		0
	0		0		0		0
	0		0		0		0
	0		0		0		0
	0		0		0		0
	0		0		0		0
	0		0		0		0
	0		0		0		0
	0		0		0		0
	0		0		0		0
	0		0		0		0
	0		0		0		0
	0		0		0		0
	0		0		0		0
	0		0		0		0
	0		0		0		0
	0		0		0		0
	0		0		0		0
	0		0		0		0
	0		0		0		0
	0		0		0		0

	0		0		0		0
	0		0	Ewa-RG-top-a	1	Ewa-RG-top-a	1
	0		0		0		0
	0		0		0		0
	0		0		0		0
	0		0		0		0
	0		0		0		0
	0		0	Ewa-RG-top-a	1	Ewa-RG-top-a	1
	0		0		0		0
	0		0		0		0
	0		0		0		0
	0		0		0		0
Ew-AU->in-RG	1	Ew-AU->in-RG	1	Ewa-RG-top-a	1	Ewa-RG-top-a	1
	0		0		0		0
	0		0		0		0
	0		0		0		0
	0		0		0		0
	0		0		0		0
	0		0		0		0
	0		0		0		0
	0		0	Ewa-RG-top-a	1	Ewa-RG-top-a	1
	0		0	Ewa-RG-top-a	1	Ewa-RG-top-a	1
	0		0		0		0
	0		0		0		0
	0		0	Ewa-RG-top-a	1	Ewa-RG-top-a	1
	0		0	Ewa-RG-top-a	1	Ewa-RG-top-a	1
	0		0		0		0
	0		0		0		0
	0		0		0		0
	0		0		0		0
	0		0		0		0
	0		0		0		0
	0		0		0		0
	0		0		0		0
	0		0		0		0
	0		0		0		0
	0		0		0		0
	0		0		0		0
	0		0		0		0
	0		0		0		0
	0		0		0		0
	0		0		0		0
	0		0		0		0
	0		0		0		0
	0		0		0		0
	0		0		0		0
	0		0		0		0

	0	0		0		0
	0	0		0		0
	0	0		0		0
	0	0		0		0
	0	0		0		0
	0	0		0		0
	0	0	Ewa-RG-top-a	1	Ewa-RG-top-a	1
	0	0	Ewa-RG-top-a	1	Ewa-RG-top-a	1
	0	0		0		0
	0	0		0		0
	0	0		0		0
	0	0		0		0
	0	0		0		0
	0	0	Ewa-RG-top-a	1	Ewa-RG-top-a	1
	0	0		0		0
	0	0		0		0
	0	0		0		0
	0	0		0		0
	0	0		0		0
	0	0		0		0
	0	0		0		0
	0	0		0		0
	0	0		0		0
	0	0		0		0
	0	0		0		0
	0	0		0		0
	0	0		0		0
	0	0		0		0
	0	0		0		0
	0	0		0		0
	0	0		0		0
	0	0		0		0
	0	0		0		0
	0	0		0		0
	0	0		0		0
	0	0		0		0
	0	0		0		0
	0	0		0		0
	0	0		0		0
	0	0		0		0
	0	0		0		0
	0	0		0		0
Ew-AU->in-R	1	0	Ewa-RG-top-a	1		0
	0	0		0		0
	0	0		0		0
	0	0		0		0
	0	0		0		0

	0	0		0		0
	0	0		0		0
	0	0		0		0
	0	0		0		0
	0	0		0		0
	0	0		0		0
	0	0		0		0
	0	0		0		0
	0	0		0		0
	0	0		0		0
	0	0		0		0
	0	0		0		0
	0	0		0		0
	0	0	Ewa-RG-top-a	1	Ewa-RG-top-a	1
	0	0		0		0
	0	0		0		0
Ew-AU->in-RG	1	0	Ewa-RG-top-a	1		0
	0	0		0		0
	0	0		0		0
	0	0		0		0
	0	0		0		0
	0	0		0		0
	0	0		0		0
	0	0		0		0
	0	0		0		0
	0	0		0		0
	0	0		0		0
Ew-AU->in-RG	1	0		0		0
	0	0		0		0
	0	0	Ewa-RG-top-a	1	Ewa-RG-top-a	1
	0	0		0		0
	0	0		0		0
	0	0		0		0
	0	0		0		0
	0	0		0		0
	0	0		0		0
	0	0		0		0
	0	0		0		0
	0	0	Ewa-RG-top-a	1	Ewa-RG-top-a	1
	0	0		0		0
	0	0		0		0
	0	0		0		0
	0	0		0		0
	0	0		0		0
	0	0		0		0
	0	0		0		0
	0	0		0		0

0	0		0		0
0	0		0		0
0	0		0		0
0	0		0		0
0	0		0		0
0	0		0		0
0	0		0		0
0	0		0		0
0	0		0		0
0	0		0		0
0	0		0		0
0	0		0		0
0	0		0		0
0	0		0		0
0	0		0		0
0	0		0		0
0	0		0		0
0	0		0		0
0	0		0		0
0	0		0		0
0	0		0		0
0	0		0		0
0	0		0		0
0	0		0		0
0	0	Ewa-RG-top-a	1	Ewa-RG-top-a	1
0	0	Ewa-RG-top-a	1	Ewa-RG-top-a	1
0	0	Ewa-RG-top-a	1		0
0	0	Ewa-RG-top-a	1		0
0	0	Ewa-RG-top-a	1		0
0	0		0		0
0	0		0		0
0	0		0		0
0	0		0		0
0	0		0		0
0	0		0		0
0	0		0		0
0	0		0		0
0	0		0		0
0	0		0		0
0	0		0		0
0	0		0		0
0	0		0		0
0	0		0		0
0	0		0		0
0	0		0		0
0	0		0		0
0	0		0		0

	0		0		0		0
	0		0		0		0
	0		0		0		0
	0		0		0		0
	0		0		0		0
	0		0		0		0
	0		0		0		0
	0		0		0		0
	0		0		0		0
	0		0	Ewa-RG-top-a	1	Ewa-RG-top-a	1
	0		0		0		0
	0		0		0		0
	0		0		0		0
	0		0		0		0
	0		0		0		0
	0		0		0		0
	0		0		0		0
	0		0		0		0
	0		0		0		0
	0		0		0		0
	0		0		0		0
	0		0		0		0
	0		0		0		0
	0		0		0		0
	0		0		0		0
	0		0		0		0
	0		0		0		0
	0		0		0		0
	0		0		0		0
	0		0		0		0
Ew-AU->in-RG	1	Ew-AU->in-RG	1		0		0
Ew-AU->in-RG	1	Ew-AU->in-RG	1		0		0
Ew-AU->in-RG	1	Ew-AU->in-RG	1		0		0
	0		0		0		0
	0		0		0		0
	0		0		0		0
	0		0		0		0
	0		0		0		0
	0		0		0		0
	0		0		0		0
	0		0		0		0
	0		0		0		0
	0		0		0		0
	0		0		0		0
	0		0		0		0
	0		0		0		0
	0		0		0		0

0	0	Ewa-RG-top-a	1	Ewa-RG-top-a	1
0	0	Ewa-RG-top-a	1	Ewa-RG-top-a	1
0	0		0		0
0	0		0		0
0	0		0		0
0	0		0		0
0	0		0		0
0	0		0		0
0	0		0		0
0	0		0		0
0	0		0		0
0	0		0		0
0	0		0		0
0	0		0		0
0	0		0		0
0	0		0		0
0	0		0		0
0	0		0		0
0	0		0		0
0	0		0		0
0	0		0		0
0	0		0		0
0	0		0		0
0	0		0		0
0	0		0		0
0	0		0		0
0	0		0		0
0	0		0		0
0	0		0		0
0	0		0		0
0	0		0		0
0	0		0		0
0	0		0		0
0	0		0		0
0	0		0		0
0	0		0		0
0	0		0		0
0	0		0		0
0	0		0		0
0	0		0		0
0	0		0		0
0	0		0		0
0	0		0		0
0	0		0		0
0	0		0		0
0	0		0		0
0	0		0		0

0	0		0		0
0	0		0		0
0	0		0		0
0	0		0		0
0	0		0		0
0	0		0		0
0	0		0		0
0	0		0		0
0	0		0		0
0	0		0		0
0	0		0		0
0	0		0		0
0	0		0		0
0	0		0		0
0	0		0		0
0	0		0		0
0	0		0		0
0	0		0		0
0	0		0		0
0	0		0		0
0	0	Ewa-RG-top-a	1	Ewa-RG-top-a	1
0	0		0		0
0	0		0		0
0	0		0		0
0	0		0		0
0	0		0		0
0	0		0		0
0	0		0		0
0	0		0		0
0	0		0		0
0	0		0		0
0	0		0		0
0	0		0		0
0	0		0		0
0	0		0		0
0	0		0		0
0	0		0		0
0	0		0		0
0	0		0		0
0	0		0		0
0	0		0		0
0	0		0		0
0	0		0		0
0	0		0		0
0	0		0		0
0	0		0		0
0	0		0		0

	0		0		0		0
	0		0		0		0
	0		0		0		0
	0		0		0		0
	0		0		0		0
	0		0		0		0
	0		0		0		0
	0		0		0		0
	0		0		0		0
	0		0		0		0
	0		0		0		0
Ew-AU->in-RG	1		0		0		0
Ew-AU->in-RG	1		0		0		0
	0		0		0		0
	0		0		0		0
	0		0		0		0
	0		0		0		0
	0		0		0		0
	0		0		0		0
	0		0		0		0
	0		0		0		0
	0		0		0		0
	0		0		0		0
	0		0		0		0
	0		0		0		0
	0		0		0		0
	0		0		0		0
	0		0		0		0
Ew-AU->in-RG	1	Ew-AU->in-RG	1	Ewa-RG-top-a	1	Ewa-RG-top-a	1
Ew-AU->in-RG	1	Ew-AU->in-RG	1	Ewa-RG-top-a	1	Ewa-RG-top-a	1
	0		0		0		0
	0		0		0		0
	0		0		0		0
	0		0		0		0
Ew-AU->in-RG	1	Ew-AU->in-RG	1	Ewa-RG-top-a	1	Ewa-RG-top-a	1
	0		0		0		0
	0		0		0		0
	0		0	Ewa-RG-top-a	1	Ewa-RG-top-a	1
	0		0		0		0
	0		0		0		0
	0		0		0		0
	0		0		0		0
	0		0		0		0
	0		0		0		0
	0		0		0		0
	0		0		0		0
	0		0		0		0

	0		0		0		0
	0		0		0		0
	0		0		0		0
	0		0		0		0
	0		0		0		0
	0		0		0		0
	0		0		0		0
	0		0		0		0
	0		0	Ewa-RG-top-a	1	Ewa-RG-top-a	1
	0		0		0		0
	0		0		0		0
	0		0		0		0
	0		0		0		0
	0		0		0		0
	0		0		0		0
	0		0		0		0
	0		0		0		0
	0		0		0		0
	0		0		0		0
	0		0		0		0
	0		0		0		0
	0		0		0		0
	0		0		0		0
	0		0		0		0
	0		0		0		0
	0		0		0		0
	0		0		0		0
	0		0		0		0
	0		0		0		0
	0		0		0		0
	0		0		0		0
	0		0		0		0
	0		0		0		0
	0		0		0		0
	0		0		0		0
	0		0		0		0
	0		0		0		0
	0		0		0		0
	0		0		0		0
	0		0		0		0
	0		0		0		0
	0		0		0		0
	0		0		0		0
Ew-AU->in-RG	1	Ew-AU->in-RG	1	Ewa-RG-top-a	1	Ewa-RG-top-a	1
	0		0		0		0
	0		0		0		0

0	0		0		0
0	0		0		0
0	0		0		0
0	0		0		0
0	0		0		0
0	0		0		0
0	0		0		0
0	0		0		0
0	0		0		0
0	0		0		0
0	0		0		0
0	0		0		0
0	0		0		0
0	0		0		0
0	0		0		0
0	0		0		0
0	0		0		0
0	0		0		0
0	0		0		0
0	0		0		0
0	0		0		0
0	0		0		0
0	0		0		0
0	0		0		0
0	0		0		0
0	0		0		0
0	0		0		0
0	0		0		0
0	0		0		0
0	0		0		0
0	0		0		0
0	0		0		0
0	0		0		0
0	0		0		0
0	0	Ewa-RG-top-a	1	Ewa-RG-top-a	1
0	0		0		0
0	0		0		0
0	0		0		0
0	0		0		0
0	0		0		0
0	0		0		0
0	0	Ewa-RG-top-a	1		0
0	0		0		0
0	0		0		0
0	0		0		0
0	0		0		0
0	0		0		0

0	0	0	0
0	0	0	0
0	0	0	0
0	0	0	0
0	0	0	0
0	0	0	0
0	0	0	0
0	0	0	0
0	0	0	0
0	0	0	0
0	0	0	0
0	0	0	0
0	0	0	0
0	0	0	0
0	0	0	0
0	0	0	0
0	0	0	0
0	0	0	0
0	0	0	0
0	0	0	0
0	0	0	0
0	0	0	0
0	0	0	0
0	0	0	0
0	0	0	0
0	0	0	0
0	0	0	0
0	0	0	0
0	0	0	0
0	0	0	0
0	0	0	0
0	0	0	0
0	0	0	0
0	0	0	0
0	0	0	0
0	0	0	0
0	0	0	0
0	0	0	0
0	0	0	0
0	0	0	0
0	0	0	0
0	0	0	0
0	0	0	0
0	0	0	0
0	0	0	0
0	0	0	0
0	0	0	0

0	0	0	0
0	0	0	0
0	0	0	0
0	0	0	0
0	0	0	0
0	0	0	0
0	0	0	0
0	0	0	0
0	0	0	0
0	0	0	0
0	0	0	0
0	0	0	0
0	0	0	0
0	0	0	0
0	0	0	0
0	0	0	0
0	0	0	0
0	0	0	0
0	0	0	0
0	0	0	0
0	0	0	0
0	0	0	0
0	0	0	0
0	0	0	0
0	0	0	0
0	0	0	0
0	0	0	0
0	0	0	0
0	0	0	0
0	0	0	0
0	0	0	0
0	0	0	0
0	0	0	0
0	0	0	0
0	0	0	0
0	0	0	0
0	0	0	0
0	0	0	0
0	0	0	0
0	0	0	0
0	0	0	0
0	0	0	0
0	0	0	0
0	0	0	0
0	0	0	0
0	0	0	0
0	0	0	0

	0		0		0		0
	0		0		0		0
	0		0	Ewa-RG-top-a	1	Ewa-RG-top-a	1
	0		0	Ewa-RG-top-a	1	Ewa-RG-top-a	1
	0		0		0		0
	0		0		0		0
	0		0		0		0
	0		0		0		0
	0		0		0		0
	0		0		0		0
	0		0		0		0
	0		0		0		0
	0		0		0		0
	0		0		0		0
	0		0		0		0
	0		0		0		0
	0		0		0		0
	0		0		0		0
	0		0		0		0
	0		0		0		0
	0		0		0		0
Ew-AU->in-RG	1	Ew-AU->in-RG	1	Ewa-RG-top-a	1	Ewa-RG-top-a	1
Ew-AU->in-RG	1	Ew-AU->in-RG	1	Ewa-RG-top-a	1	Ewa-RG-top-a	1
	0		0		0		0
	0		0		0		0
	0		0		0		0
	0		0		0		0
	0		0		0		0
	0		0		0		0
	0		0		0		0
	0		0		0		0
	0		0		0		0
	0		0		0		0
	0		0		0		0
	0		0		0		0
	0		0		0		0
	0		0		0		0
	0		0		0		0
	0		0		0		0
	0		0		0		0
	0		0		0		0
	0		0		0		0
	0		0		0		0
	0		0		0		0
	0		0		0		0
	0		0		0		0

	0		0		0		0
	0		0		0		0
	0		0	Ewa-RG-top-a	1	Ewa-RG-top-a	1
	0		0	Ewa-RG-top-a	1	Ewa-RG-top-a	1
	0		0		0		0
	0		0		0		0
	0		0		0		0
	0		0		0		0
	0		0		0		0
	0		0		0		0
	0		0		0		0
	0		0		0		0
	0		0		0		0
	0		0		0		0
	0		0	Ewa-RG-top-a	1	Ewa-RG-top-a	1
	0		0		0		0
	0		0		0		0
	0		0		0		0
Ew-AU->in-RG	1	Ew-AU->in-RG	1	Ewa-RG-top-a	1	Ewa-RG-top-a	1
	0		0		0		0
	0		0		0		0
	0		0		0		0
	0		0		0		0
	0		0		0		0
	0		0		0		0
	0		0		0		0
	0		0		0		0
	0		0		0		0
	0		0		0		0
	0		0		0		0
Ew-AU->in-RG	1		0	Ewa-RG-top-a	1		0
Ew-AU->in-RG	1		0	Ewa-RG-top-a	1		0
	0		0		0		0
	0		0		0		0
	0		0		0		0
	0		0		0		0
	0		0		0		0
	0		0		0		0
	0		0		0		0
	0		0		0		0
	0		0		0		0
	0		0		0		0
	0		0		0		0
	0		0		0		0
	0		0		0		0
	0		0		0		0
	0		0		0		0

	0		0		0		0
	0		0		0		0
	0		0		0		0
	0		0		0		0
	0		0		0		0
	0		0		0		0
	0		0		0		0
	0		0		0		0
	0		0	Ewa-RG-top-a	1	Ewa-RG-top-a	1
	0		0		0		0
	0		0		0		0
	0		0		0		0
	0		0		0		0
	0		0		0		0
	0		0		0		0
	0		0		0		0
	0		0		0		0
	0		0		0		0
	0		0		0		0
	0		0		0		0
	0		0		0		0
	0		0		0		0
	0		0		0		0
	0		0		0		0
	0		0		0		0
	0		0		0		0
	0		0		0		0
	0		0		0		0
	0		0	Ewa-RG-top-a	1	Ewa-RG-top-a	1
Ew-AU->in-RG	1	Ew-AU->in-RG	1	Ewa-RG-top-a	1	Ewa-RG-top-a	1
Ew-AU->in-RG	1	Ew-AU->in-RG	1	Ewa-RG-top-a	1	Ewa-RG-top-a	1
	0		0		0		0
	0		0		0		0
	0		0		0		0
	0		0		0		0
	0		0		0		0
	0		0		0		0
	0		0		0		0
	0		0		0		0
	0		0		0		0
	0		0		0		0
	0		0		0		0
	0		0		0		0
	0		0		0		0
	0		0		0		0
	0		0		0		0
	0		0		0		0

	0	0	0	0
	0	0	0	0
	0	0	0	0
	0	0	0	0
	0	0	0	0
	0	0	0	0
	0	0	0	0
	0	0	0	0
	0	0	0	0
	0	0	0	0
	0	0	0	0
	0	0	0	0
	0	0	0	0
	0	0	0	0
	0	0	0	0
	0	0	0	0
	0	0	0	0
	0	0	0	0
	0	0	0	0
	0	0	0	0
	0	0	0	0
	0	0	0	0
	0	0	0	0
	0	0	0	0
	0	0	0	0
	0	0	0	0
	0	0	0	0
	0	0	0	0
	0	0	0	0
	0	0	0	0
	0	0	0	0
	0	0	0	0
	0	0	0	0
	0	0	0	0
	0	0	0	0
	0	0	0	0
	0	0	0	0
	0	0	0	0
	0	0	0	0
	0	0	0	0
	0	0	0	0
	0	0	0	0
	0	0	0	0
	0	0	0	0
	0	0	0	0
Ew-AU->in-RG	1	0	0	0
	0	0	0	0

	0	0		0		0
	0	0		0		0
	0	0		0		0
	0	0		0		0
	0	0		0		0
	0	0		0		0
Ew-AU->in-RG	1	0		0		0
Ew-AU->in-RG	1	0		0		0
	0	0		0		0
	0	0		0		0
	0	0		0		0
	0	0		0		0
	0	0		0		0
	0	0		0		0
	0	0		0		0
	0	0		0		0
	0	0		0		0
	0	0		0		0
	0	0		0		0
	0	0		0		0
	0	0		0		0
	0	0		0		0
	0	0		0		0
	0	0		0		0
	0	0		0		0
	0	0		0		0
	0	0		0		0
	0	0		0		0
	0	0		0		0
	0	0		0		0
	0	0		0		0
	0	0	Ewa-RG-top-a	1	Ewa-RG-top-a	1
	0	0		0		0
	0	0		0		0
	0	0		0		0
	0	0		0		0
	0	0		0		0
	0	0		0		0
	0	0		0		0
Ew-AU->in-RG	1	0		0		0
Ew-AU->in-RG	1	0		0		0
	0	0		0		0
	0	0		0		0
	0	0		0		0
	0	0		0		0
	0	0		0		0
	0	0		0		0

	0		0		0		0
	0		0		0		0
	0		0		0		0
	0		0		0		0
	0		0		0		0
	0		0		0		0
	0		0		0		0
	0		0		0		0
	0		0		0		0
	0		0		0		0
	0		0		0		0
Ew-AU->in-RG	1	Ew-AU->in-RG	1	Ewa-RG-top-a	1	Ewa-RG-top-a	1
Ew-AU->in-RG	1	Ew-AU->in-RG	1	Ewa-RG-top-a	1	Ewa-RG-top-a	1
	0		0		0		0
	0		0		0		0
	0		0		0		0
	0		0		0		0
	0		0		0		0
	0		0		0		0
	0		0		0		0
	0		0		0		0
	0		0		0		0
	0		0		0		0
	0		0		0		0
	0		0		0		0
	0		0		0		0
	0		0		0		0
	0		0		0		0
	0		0		0		0
	0		0	Ewa-RG-top-a	1	Ewa-RG-top-a	1
	0		0	Ewa-RG-top-a	1	Ewa-RG-top-a	1
	0		0		0		0
	0		0		0		0
	0		0		0		0
	0		0		0		0
	0		0		0		0
	0		0		0		0
	0		0		0		0
	0		0		0		0
	0		0		0		0
	0		0		0		0
	0		0		0		0
	0		0		0		0
	0		0		0		0
	0		0		0		0
	0		0		0		0
	0		0		0		0

	0		0		0		0
	0		0		0		0
	0		0		0		0
Ew-AU->in-RG	1		0		0		0
Ew-AU->in-RG	1		0		0		0
	0		0		0		0
	0		0		0		0
	0		0		0		0
	0		0		0		0
	0		0		0		0
	0		0		0		0
	0		0	Ewa-RG-top-a	1	Ewa-RG-top-a	1
Ew-AU->in-RG	1	Ew-AU->in-RG	1	Ewa-RG-top-a	1	Ewa-RG-top-a	1
Ew-AU->in-RG	1	Ew-AU->in-RG	1	Ewa-RG-top-a	1	Ewa-RG-top-a	1
	0		0		0		0
	0		0		0		0
	0		0		0		0
	0		0	Ewa-RG-top-a	1	Ewa-RG-top-a	1
	0		0		0		0
	0		0		0		0
	0		0		0		0
	0		0		0		0
	0		0		0		0
	0		0		0		0
	0		0	Ewa-RG-top-a	1	Ewa-RG-top-a	1
	0		0		0		0
	0		0		0		0
	0		0		0		0
	0		0		0		0
	0		0		0		0
	0		0		0		0
	0		0		0		0
	0		0		0		0
	0		0		0		0
	0		0		0		0
	0		0		0		0
	0		0		0		0
	0		0		0		0
	0		0		0		0
	0		0		0		0
	0		0		0		0
	0		0		0		0
	0		0		0		0
	0		0		0		0
	0		0		0		0
	0		0		0		0
	0		0		0		0

0	0		0		0
0	0		0		0
0	0		0		0
0	0		0		0
0	0		0		0
0	0		0		0
0	0		0		0
0	0		0		0
0	0	Ewa-RG-top-a	1	Ewa-RG-top-a	1
0	0	Ewa-RG-top-a	1	Ewa-RG-top-a	1
0	0	Ewa-RG-top-a	1	Ewa-RG-top-a	1
0	0		0		0
0	0		0		0
0	0	Ewa-RG-top-a	1	Ewa-RG-top-a	1
0	0	Ewa-RG-top-a	1	Ewa-RG-top-a	1
0	0		0		0
0	0		0		0
0	0		0		0
0	0		0		0
0	0		0		0
0	0		0		0
0	0		0		0
0	0		0		0
0	0		0		0
0	0		0		0
0	0		0		0
0	0		0		0
0	0		0		0
0	0		0		0
0	0		0		0
0	0		0		0
0	0		0		0
0	0		0		0
0	0		0		0
0	0		0		0
0	0		0		0
0	0		0		0
0	0		0		0
0	0		0		0
0	0		0		0
0	0		0		0
0	0		0		0
0	0		0		0
0	0		0		0
0	0		0		0
0	0		0		0
0	0		0		0

0	0		0		0
0	0		0		0
0	0		0		0
0	0		0		0
0	0	Ewa-RG-top-a	1	Ewa-RG-top-a	1
0	0	Ewa-RG-top-a	1	Ewa-RG-top-a	1
0	0	Ewa-RG-top-a	1	Ewa-RG-top-a	1
0	0		0		0
0	0		0		0
0	0		0		0
0	0		0		0
0	0		0		0
0	0		0		0
0	0		0		0
0	0		0		0
0	0		0		0
0	0		0		0
0	0		0		0
0	0		0		0
0	0		0		0
0	0		0		0
0	0		0		0
0	0		0		0
0	0		0		0
0	0		0		0
0	0		0		0
0	0		0		0
0	0		0		0
0	0		0		0
0	0		0		0
0	0		0		0
0	0		0		0
0	0		0		0
0	0		0		0
0	0		0		0
0	0		0		0
0	0		0		0
0	0		0		0
0	0		0		0
0	0		0		0
0	0		0		0
0	0		0		0
0	0		0		0
0	0		0		0
0	0		0		0
0	0		0		0
0	0		0		0
0	0		0		0
0	0		0		0
0	0		0		0
0	0		0		0
0	0		0		0
0	0		0		0

	0		0		0		0
	0		0		0		0
	0		0		0		0
	0		0		0		0
	0		0		0		0
	0		0		0		0
	0		0		0		0
	0		0		0		0
	0		0		0		0
	0		0	Ewa-RG-top-a	1	Ewa-RG-top-a	1
	0		0		0		0
	0		0		0		0
	0		0		0		0
	0		0		0		0
	0		0		0		0
	0		0		0		0
	0		0		0		0
	0		0		0		0
Ew-AU->in-RG	1	Ew-AU->in-RG	1	Ewa-RG-top-a	1	Ewa-RG-top-a	1
Ew-AU->in-RG	1	Ew-AU->in-RG	1	Ewa-RG-top-a	1	Ewa-RG-top-a	1
	0		0		0		0
	0		0		0		0
	0		0		0		0
	0		0		0		0
	0		0		0		0
	0		0		0		0
	0		0		0		0
	0		0		0		0
	0		0		0		0
	0		0		0		0
	0		0		0		0
	0		0		0		0
	0		0		0		0
	0		0		0		0
	0		0		0		0
	0		0		0		0
	0		0		0		0
	0		0		0		0
	0		0		0		0
	0		0		0		0
	0		0		0		0
	0		0		0		0
	0		0		0		0
	0		0		0		0
	0		0		0		0
	0		0		0		0
	0		0	Ewa-RG-top-a	1	Ewa-RG-top-a	1

	0		0		0		0
	0		0		0		0
	0		0		0		0
	0		0		0		0
	0		0		0		0
	0		0		0		0
	0		0		0		0
Ew-AU->in-RG	1	Ew-AU->in-RG	1	Ewa-RG-top-a	1	Ewa-RG-top-a	1
Ew-AU->in-RG	1	Ew-AU->in-RG	1	Ewa-RG-top-a	1	Ewa-RG-top-a	1
	0		0		0		0
	0		0		0		0
	0		0		0		0
	0		0		0		0
Ew-AU->in-RG	1		0		0		0
	0		0		0		0
	0		0		0		0
	0		0		0		0
	0		0		0		0
	0		0		0		0
	0		0		0		0
	0		0		0		0
	0		0		0		0
	0		0		0		0
	0		0		0		0
	0		0		0		0
	0		0		0		0
	0		0		0		0
	0		0		0		0
	0		0		0		0
	0		0		0		0
	0		0		0		0
	0		0		0		0
	0		0		0		0
	0		0		0		0
	0		0		0		0
	0		0		0		0
	0		0		0		0
	0		0		0		0
	0		0		0		0
	0		0		0		0
	0		0		0		0
	0		0		0		0
	0		0		0		0
	0		0		0		0
	0		0		0		0
	0		0		0		0
	0		0		0		0

0	0		0		0
0	0		0		0
0	0		0		0
0	0		0		0
0	0		0		0
0	0		0		0
0	0		0		0
0	0		0		0
0	0		0		0
0	0		0		0
0	0		0		0
0	0		0		0
0	0		0		0
0	0		0		0
0	0		0		0
0	0		0		0
0	0		0		0
0	0		0		0
0	0		0		0
0	0		0		0
0	0		0		0
0	0		0		0
0	0		0		0
0	0		0		0
0	0		0		0
0	0		0		0
0	0		0		0
0	0		0		0
0	0		0		0
0	0		0		0
0	0		0		0
0	0		0		0
0	0		0		0
0	0		0		0
0	0		0		0
0	0		0		0
0	0		0		0
0	0		0		0
0	0		0		0
0	0		0		0
0	0		0		0
0	0		0		0
0	0		0		0
0	0		0		0
0	0		0		0
0	0		0		0
0	0	Ewa-RG-top-a	1	Ewa-RG-top-a	1

0	0	Ewa-RG-top-a	1	Ewa-RG-top-a	1
0	0		0		0
0	0	Ewa-RG-top-a	1		0
0	0	Ewa-RG-top-a	1		0
0	0		0		0
0	0		0		0
0	0		0		0
0	0		0		0
0	0		0		0
0	0		0		0
0	0		0		0
0	0		0		0
0	0		0		0
0	0		0		0
0	0		0		0
0	0		0		0
0	0		0		0
0	0		0		0
0	0		0		0
0	0		0		0
0	0		0		0
0	0		0		0
0	0		0		0
0	0		0		0
0	0		0		0
0	0		0		0
0	0		0		0
0	0		0		0
0	0		0		0
0	0		0		0
0	0		0		0
0	0		0		0
0	0		0		0
0	0		0		0
0	0		0		0
0	0		0		0
0	0		0		0
0	0		0		0
0	0		0		0
0	0		0		0
0	0		0		0
0	0		0		0
0	0		0		0
0	0		0		0
0	0		0		0
0	0		0		0
0	0		0		0

	0	0	0	0
	0	0	0	0
	0	0	0	0
	0	0	0	0
	0	0	0	0
	0	0	0	0
	0	0	0	0
	0	0	0	0
	0	0	0	0
	0	0	0	0
	0	0	0	0
	0	0	0	0
	0	0	0	0
	0	0	0	0
	0	0	0	0
	0	0	0	0
	0	0	0	0
	0	0	0	0
	0	0	0	0
	0	0	0	0
	0	0	0	0
	0	0	0	0
	0	0	0	0
	0	0	0	0
	0	0	0	0
	0	0	0	0
	0	0	0	0
	0	0	0	0
	0	0	0	0
	0	0	0	0
	0	0	0	0
	0	0	0	0
	0	0	0	0
	0	0	0	0
	0	0	0	0
	0	0	0	0
	0	0	0	0
Ew-AU->in-R	1	0	0	0
Ew-AU->in-R	1	0	0	0
	0	0	0	0
	0	0	0	0
	0	0	0	0
	0	0	0	0
	0	0	0	0
	0	0	0	0
	0	0	0	0
	0	0	0	0

Ew-AU->in-RG	1		0		0		0
	0		0		0		0
	0		0	Ewa-RG-top-a	1	Ewa-RG-top-a	1
	0		0		0		0
	0		0		0		0
	0		0		0		0
	0		0		0		0
	0		0		0		0
	0		0		0		0
	0		0		0		0
	0		0		0		0
	0		0		0		0
	0		0		0		0
	0		0		0		0
	0		0		0		0
	0		0		0		0
	0		0		0		0
	0		0		0		0
	0		0		0		0
	0		0		0		0
	0		0		0		0
	0		0		0		0
	0		0		0		0
	0		0		0		0
	0		0		0		0
	0		0		0		0
Ew-AU->in-RG	1	Ew-AU->in-RG	1	Ewa-RG-top-a	1	Ewa-RG-top-a	1
	0		0		0		0
	0		0		0		0
	0		0		0		0
	0		0		0		0
	0		0		0		0
	0		0		0		0
	0		0		0		0
	0		0		0		0
	0		0		0		0
	0		0		0		0
	0		0		0		0
	0		0		0		0
	0		0		0		0
	0		0		0		0
	0		0		0		0
	0		0		0		0
	0		0		0		0
	0		0		0		0
	0		0		0		0
	0		0		0		0

	0		0		0		0
	0		0		0		0
	0		0		0		0
	0		0		0		0
	0		0		0		0
	0		0		0		0
	0		0		0		0
	0		0		0		0
	0		0	Ewa-RG-top-a	1	Ewa-RG-top-a	1
	0		0	Ewa-RG-top-a	1	Ewa-RG-top-a	1
	0		0		0		0
	0		0		0		0
	0		0		0		0
	0		0		0		0
	0		0		0		0
	0		0		0		0
Ew-AU->in-RG	1	Ew-AU->in-RG	1	Ewa-RG-top-a	1	Ewa-RG-top-a	1
	0		0		0		0
	0		0		0		0
	0		0		0		0
	0		0		0		0
	0		0		0		0
	0		0		0		0
	0		0		0		0
	0		0		0		0
	0		0		0		0
	0		0		0		0
	0		0		0		0
	0		0	Ewa-RG-top-a	1	Ewa-RG-top-a	1
	0		0		0		0
	0		0		0		0
	0		0		0		0
	0		0		0		0
	0		0		0		0
	0		0		0		0
	0		0		0		0
	0		0		0		0
	0		0	Ewa-RG-top-a	1	Ewa-RG-top-a	1
	0		0		0		0
	0		0		0		0
	0		0		0		0
	0		0		0		0
	0		0		0		0
	0		0		0		0
	0		0		0		0
	0		0		0		0
	0		0		0		0

	0		0		0		0
	0		0		0		0
	0		0		0		0
	0		0		0		0
	0		0		0		0
	0		0		0		0
	0		0		0		0
	0		0		0		0
	0		0		0		0
	0		0		0		0
	0		0		0		0
	0		0		0		0
	0		0		0		0
	0		0		0		0
	0		0		0		0
	0		0		0		0
	0		0		0		0
	0		0		0		0
	0		0		0		0
	0		0		0		0
	0		0		0		0
	0		0		0		0
	0		0		0		0
	0		0		0		0
	0		0		0		0
	0		0		0		0
	0		0	Ewa-RG-top-a	1	Ewa-RG-top-a	1
	0		0	Ewa-RG-top-a	1	Ewa-RG-top-a	1
	0		0		0		0
	0		0		0		0
	0		0		0		0
Ew-AU->in-R	1	Ew-AU->in-R	1	Ewa-RG-top-a	1	Ewa-RG-top-a	1
	0		0		0		0
	0		0		0		0
	0		0		0		0
	0		0		0		0
	0		0		0		0
	0		0		0		0
	0		0		0		0
	0		0		0		0
	0		0		0		0
	0		0		0		0
	0		0		0		0
	0		0		0		0
	0		0		0		0
	0		0		0		0
	0		0		0		0

	0		0		0		0
	0		0		0		0
	0		0		0		0
	0		0		0		0
	0		0		0		0
	0		0		0		0
	0		0		0		0
	0		0		0		0
	0		0		0		0
	0		0		0		0
	0		0		0		0
	0		0		0		0
	0		0		0		0
	0		0		0		0
	0		0		0		0
	0		0		0		0
	0		0		0		0
	0		0		0		0
	0		0		0		0
	0		0		0		0
	0		0		0		0
	0		0		0		0
	0		0		0		0
	0		0		0		0
	0		0		0		0
	0		0		0		0
	0		0		0		0
	0		0		0		0
	0		0		0		0
	0		0	Ewa-RG-top-a	1	Ewa-RG-top-a	1
	0		0	Ewa-RG-top-a	1	Ewa-RG-top-a	1
	0		0		0		0
	0		0		0		0
	0		0		0		0
	0		0		0		0
	0		0		0		0
	0		0		0		0
	0		0		0		0
	0		0		0		0
	0		0		0		0
	0		0		0		0
	0		0		0		0
	0		0		0		0
Ew-AU->in-RG	1	Ew-AU->in-RG	1	Ewa-RG-top-a	1	Ewa-RG-top-a	1
Ew-AU->in-RG	1	Ew-AU->in-RG	1	Ewa-RG-top-a	1	Ewa-RG-top-a	1
	0		0		0		0
	0		0		0		0

0	0	Ewa-RG-top-a	1	Ewa-RG-top-a	1
0	0		0		0
0	0		0		0
0	0		0		0
0	0		0		0
0	0		0		0
0	0		0		0
0	0		0		0
0	0		0		0
0	0		0		0
0	0	Ewa-RG-top-a	1		0
0	0	Ewa-RG-top-a	1		0
0	0	Ewa-RG-top-a	1		0
0	0	Ewa-RG-top-a	1		0
0	0		0		0
0	0		0		0
0	0		0		0
0	0		0		0
0	0		0		0
0	0		0		0
0	0		0		0
0	0		0		0
0	0		0		0
0	0		0		0
0	0		0		0
0	0		0		0
0	0		0		0
0	0		0		0
0	0		0		0
0	0		0		0
0	0		0		0
0	0		0		0
0	0		0		0
0	0		0		0
0	0		0		0
0	0		0		0
0	0	Ewa-RG-top-a	1	Ewa-RG-top-a	1
0	0		0		0
0	0		0		0
0	0		0		0
0	0		0		0
0	0		0		0
0	0		0		0
0	0		0		0
0	0		0		0
0	0		0		0
0	0		0		0

	0		0		0		0
	0		0		0		0
	0		0		0		0
	0		0		0		0
	0		0		0		0
	0		0		0		0
	0		0		0		0
	0		0		0		0
	0		0		0		0
	0		0		0		0
Ew-AU->in-RG	1	Ew-AU->in-RG	1	Ewa-RG-top-a	1	Ewa-RG-top-a	1
Ew-AU->in-RG	1	Ew-AU->in-RG	1	Ewa-RG-top-a	1	Ewa-RG-top-a	1
	0		0		0		0
	0		0		0		0
	0		0		0		0
	0		0		0		0
	0		0		0		0
	0		0		0		0
	0		0		0		0
	0		0		0		0
	0		0		0		0
	0		0		0		0
	0		0		0		0
	0		0		0		0
	0		0		0		0
	0		0		0		0
Ew-AU->in-RG	1		0		0		0
	0		0		0		0
	0		0		0		0
	0		0		0		0
	0		0		0		0
	0		0		0		0
	0		0		0		0
	0		0		0		0
	0		0		0		0
	0		0		0		0
	0		0		0		0
	0		0		0		0
	0		0		0		0
	0		0		0		0
	0		0		0		0
	0		0		0		0
	0		0		0		0
	0		0		0		0
	0		0		0		0
	0		0		0		0
	0		0		0		0

	0		0		0		0
	0		0		0		0
	0		0		0		0
	0		0		0		0
	0		0		0		0
	0		0		0		0
	0		0		0		0
	0		0		0		0
	0		0	Ewa-RG-top-a	1	Ewa-RG-top-a	1
	0		0		0		0
	0		0		0		0
	0		0		0		0
	0		0		0		0
	0		0		0		0
	0		0		0		0
	0		0		0		0
	0		0		0		0
	0		0		0		0
	0		0		0		0
	0		0	Ewa-RG-top-a	1	Ewa-RG-top-a	1
Ew-AU->in-R	1		0		0		0
	0		0		0		0
	0		0		0		0
	0		0		0		0
	0		0		0		0
	0		0		0		0
	0		0	Ewa-RG-top-a	1	Ewa-RG-top-a	1
	0		0	Ewa-RG-top-a	1	Ewa-RG-top-a	1
	0		0		0		0
	0		0		0		0
	0		0		0		0
	0		0		0		0
	0		0		0		0
	0		0		0		0
	0		0		0		0
	0		0		0		0
	0		0		0		0
	0		0		0		0
	0		0		0		0
	0		0		0		0
	0		0		0		0
	0		0		0		0
	0		0		0		0
	0		0		0		0
	0		0		0		0
	0		0		0		0
	0		0		0		0

	0	0		0	0
	0	0		0	0
	0	0		0	0
	0	0		0	0
	0	0		0	0
Ew-AU->in-RG	1	0	Ewa-RG-top-a	1	0
Ew-AU->in-RG	1	0	Ewa-RG-top-a	1	0
	0	0		0	0
	0	0		0	0
	0	0		0	0
	0	0		0	0
	0	0		0	0
	0	0		0	0
	0	0		0	0
	0	0		0	0
	0	0		0	0
	0	0		0	0
	0	0		0	0
	0	0		0	0
	0	0		0	0
	0	0		0	0
	0	0		0	0
	0	0		0	0
	0	0		0	0
	0	0		0	0
	0	0		0	0
	0	0		0	0
	0	0		0	0
	0	0		0	0
	0	0		0	0
	0	0		0	0
	0	0		0	0
	0	0		0	0
	0	0		0	0
	0	0		0	0
	0	0		0	0
	0	0		0	0
	0	0		0	0
	0	0		0	0
	0	0		0	0
	0	0		0	0
	0	0		0	0
	0	0		0	0
	0	0		0	0
	0	0		0	0
	0	0		0	0
	0	0		0	0

0	0		0		0
0	0		0		0
0	0		0		0
0	0		0		0
0	0		0		0
0	0		0		0
0	0		0		0
0	0		0		0
0	0		0		0
0	0		0		0
0	0		0		0
0	0		0		0
0	0		0		0
0	0		0		0
0	0		0		0
0	0		0		0
0	0		0		0
0	0		0		0
0	0		0		0
0	0		0		0
0	0		0		0
0	0		0		0
0	0		0		0
0	0		0		0
0	0		0		0
0	0		0		0
0	0		0		0
0	0		0		0
0	0		0		0
0	0		0		0
0	0		0		0
0	0		0		0
0	0		0		0
0	0		0		0
0	0		0		0
0	0		0		0
0	0		0		0
0	0		0		0
0	0	Ewa-RG-top-a	1	Ewa-RG-top-a	1
0	0	Ewa-RG-top-a	1	Ewa-RG-top-a	1
0	0		0		0
0	0		0		0
0	0		0		0
0	0		0		0
0	0		0		0
0	0		0		0
0	0		0		0

0	0		0		0
0	0		0		0
0	0		0		0
0	0		0		0
0	0		0		0
0	0		0		0
0	0		0		0
0	0		0		0
0	0		0		0
0	0		0		0
0	0		0		0
0	0	Ewa-RG-top-a	1	Ewa-RG-top-a	1
0	0		0		0
0	0		0		0
0	0		0		0
0	0		0		0
0	0		0		0
0	0		0		0
0	0		0		0
0	0		0		0
0	0		0		0
0	0		0		0
0	0		0		0
0	0		0		0
0	0		0		0
0	0		0		0
0	0		0		0
0	0		0		0
0	0		0		0
0	0		0		0
0	0		0		0
0	0		0		0
0	0		0		0
0	0		0		0
0	0		0		0
0	0		0		0
0	0		0		0
0	0		0		0
0	0		0		0
0	0		0		0
0	0		0		0
0	0		0		0
0	0		0		0
0	0		0		0
0	0		0		0
0	0		0		0
0	0		0		0

0	0		0		0
0	0		0		0
0	0		0		0
0	0		0		0
0	0		0		0
0	0		0		0
0	0		0		0
0	0		0		0
0	0		0		0
0	0		0		0
0	0		0		0
0	0		0		0
0	0		0		0
0	0		0		0
0	0		0		0
0	0		0		0
0	0		0		0
0	0		0		0
0	0		0		0
0	0		0		0
0	0		0		0
0	0		0		0
0	0		0		0
0	0		0		0
0	0		0		0
0	0		0		0
0	0		0		0
0	0		0		0
0	0		0		0
0	0		0		0
0	0		0		0
0	0		0		0
0	0	Ewa-RG-top-a	1	Ewa-RG-top-a	1
0	0		0		0
0	0		0		0
0	0		0		0
0	0		0		0
0	0		0		0
0	0		0		0
0	0		0		0
0	0		0		0
0	0		0		0
0	0		0		0
0	0		0		0
0	0		0		0
0	0		0		0
0	0		0		0

	0		0	Ewa-RG-top-a	1	Ewa-RG-top-a	1
	0		0	Ewa-RG-top-a	1	Ewa-RG-top-a	1
	0		0		0		0
	0		0		0		0
	0		0		0		0
	0		0		0		0
	0		0		0		0
	0		0		0		0
	0		0		0		0
	0		0		0		0
	0		0		0		0
	0		0		0		0
	0		0		0		0
	0		0		0		0
	0		0		0		0
	0		0		0		0
	0		0		0		0
	0		0		0		0
	0		0		0		0
	0		0		0		0
	0		0		0		0
	0		0		0		0
	0		0		0		0
	0		0		0		0
	0		0		0		0
	0		0		0		0
	0		0		0		0
	0		0		0		0
	0		0		0		0
	0		0		0		0
	0		0		0		0
	0		0		0		0
	0		0		0		0
	0		0		0		0
	0		0		0		0
	0		0		0		0
	0		0		0		0
	0		0		0		0
	0		0		0		0
	0		0		0		0
	0		0		0		0
	0		0		0		0
Ew-AU->in-RG	1	Ew-AU->in-RG	1	Ewa-RG-top-a	1	Ewa-RG-top-a	1
	0		0		0		0
	0		0		0		0
	0		0		0		0
	0		0		0		0

	0	0	0	0
	0	0	0	0
	0	0	0	0
	0	0	0	0
	0	0	0	0
	0	0	0	0
	0	0	0	0
	0	0	0	0
	0	0	0	0
	0	0	0	0
Ew-AU->in-RG	1	0	0	0
Ew-AU->in-RG	1	0	0	0
	0	0	0	0
	0	0	0	0
	0	0	0	0
	0	0	0	0
	0	0	0	0
	0	0	0	0
	0	0	0	0
	0	0	0	0
	0	0	0	0
	0	0	0	0
	0	0	0	0
	0	0	0	0
	0	0	0	0
	0	0	0	0
	0	0	0	0
	0	0	0	0
	0	0	0	0
	0	0	0	0
	0	0	0	0
	0	0	0	0
	0	0	0	0
	0	0	0	0
	0	0	0	0
	0	0	0	0
	0	0	0	0
	0	0	0	0
	0	0	0	0
	0	0	0	0
	0	0	0	0
	0	0	0	0
	0	0	0	0
	0	0	0	0
	0	0	0	0
	0	0	0	0
	0	0	0	0

0	0		0		0
0	0		0		0
0	0		0		0
0	0		0		0
0	0		0		0
0	0		0		0
0	0		0		0
0	0		0		0
0	0		0		0
0	0		0		0
0	0		0		0
0	0		0		0
0	0	Ewa-RG-top-a	1	Ewa-RG-top-a	1
0	0		0		0
0	0		0		0
0	0		0		0
0	0	Ewa-RG-top-a	1	Ewa-RG-top-a	1
0	0		0		0
0	0		0		0
0	0		0		0
0	0		0		0
0	0		0		0
0	0		0		0
0	0		0		0
0	0		0		0
0	0		0		0
0	0		0		0
0	0	Ewa-RG-top-a	1	Ewa-RG-top-a	1
0	0	Ewa-RG-top-a	1	Ewa-RG-top-a	1
0	0		0		0
0	0		0		0
0	0		0		0
0	0		0		0
0	0		0		0
0	0		0		0
0	0		0		0
0	0		0		0
0	0		0		0
0	0		0		0
0	0		0		0
0	0		0		0
0	0		0		0
0	0		0		0
0	0		0		0
0	0		0		0
0	0		0		0
0	0		0		0

	0		0		0		0
	0		0		0		0
	0		0		0		0
	0		0		0		0
	0		0		0		0
	0		0		0		0
Ew-AU->in-RG	1	Ew-AU->in-RG	1	Ewa-RG-top-a	1	Ewa-RG-top-a	1
	0		0		0		0
	0		0		0		0
	0		0		0		0
	0		0		0		0
Ew-AU->in-RG	1	Ew-AU->in-RG	1	Ewa-RG-top-a	1	Ewa-RG-top-a	1
	0		0		0		0
	0		0		0		0
	0		0		0		0
	0		0		0		0
	0		0		0		0
	0		0		0		0
	0		0		0		0
	0		0		0		0
	0		0		0		0
	0		0		0		0
	0		0		0		0
	0		0		0		0
	0		0		0		0
	0		0		0		0
	0		0		0		0
	0		0		0		0
	0		0		0		0
	0		0		0		0
	0		0		0		0
	0		0		0		0
	0		0		0		0
	0		0		0		0
	0		0		0		0
	0		0		0		0
	0		0		0		0
	0		0		0		0
	0		0		0		0
	0		0		0		0
	0		0		0		0
	0		0		0		0
	0		0		0		0
	0		0		0		0
	0		0		0		0
	0		0		0		0
	0		0		0		0

0	0		0		0
0	0		0		0
0	0	Ewa-RG-top-a	1	Ewa-RG-top-a	1
0	0	Ewa-RG-top-a	1	Ewa-RG-top-a	1
0	0	Ewa-RG-top-a	1	Ewa-RG-top-a	1
0	0		0		0
0	0		0		0
0	0		0		0
0	0		0		0
0	0		0		0
0	0		0		0
0	0		0		0
0	0		0		0
0	0		0		0
0	0		0		0
0	0		0		0
0	0		0		0
0	0		0		0
0	0		0		0
0	0		0		0
0	0		0		0
0	0		0		0
0	0		0		0
0	0		0		0
0	0		0		0
0	0		0		0
0	0		0		0
0	0		0		0
0	0		0		0
0	0		0		0
0	0		0		0
0	0		0		0
0	0		0		0
0	0		0		0
0	0		0		0
0	0		0		0
0	0		0		0
0	0		0		0
0	0		0		0
0	0		0		0
0	0	Ewa-RG-top-a	1	Ewa-RG-top-a	1
0	0		0		0
0	0		0		0
0	0		0		0
0	0		0		0
0	0		0		0
0	0		0		0

	0		0		0		0
Ew-AU->in-RG	1	Ew-AU->in-RG	1	Ewa-RG-top-a	1	Ewa-RG-top-a	1
	0		0		0		0
	0		0		0		0
	0		0		0		0
	0		0		0		0
	0		0		0		0
	0		0		0		0
	0		0		0		0
	0		0		0		0
	0		0		0		0
	0		0		0		0
	0		0		0		0
	0		0		0		0
	0		0		0		0
	0		0		0		0
	0		0		0		0
	0		0		0		0
	0		0		0		0
	0		0		0		0
	0		0		0		0
	0		0		0		0
	0		0		0		0
	0		0		0		0
	0		0		0		0
	0		0		0		0
	0		0		0		0
	0		0		0		0
Ew-AU->in-RG	1	Ew-AU->in-RG	1	Ewa-RG-top-a	1	Ewa-RG-top-a	1
Ew-AU->in-RG	1	Ew-AU->in-RG	1	Ewa-RG-top-a	1	Ewa-RG-top-a	1
	0		0		0		0
	0		0		0		0
	0		0		0		0
	0		0		0		0
	0		0		0		0
	0		0		0		0
	0		0		0		0
	0		0		0		0
	0		0		0		0
	0		0		0		0
	0		0		0		0
	0		0		0		0
	0		0		0		0
	0		0		0		0
	0		0		0		0
	0		0		0		0
	0		0		0		0

	0		0		0		0
	0		0		0		0
	0		0		0		0
	0		0		0		0
	0		0		0		0
	0		0		0		0
	0		0		0		0
	0		0		0		0
	0		0		0		0
	0		0		0		0
Ew-AU->in-RG	1	Ew-AU->in-RG	1	Ewa-RG-top-a	1	Ewa-RG-top-a	1
Ew-AU->in-RG	1	Ew-AU->in-RG	1	Ewa-RG-top-a	1	Ewa-RG-top-a	1
	0		0		0		0
	0		0		0		0
	0		0		0		0
	0		0		0		0
	0		0		0		0
	0		0		0		0
	0		0		0		0
	0		0		0		0
	0		0		0		0
	0		0		0		0
	0		0		0		0
	0		0		0		0
	0		0		0		0
Ew-AU->in-RG	1		0	Ewa-RG-top-a	1		0
Ew-AU->in-RG	1		0	Ewa-RG-top-a	1		0
	0		0		0		0
	0		0		0		0
	0		0		0		0
	0		0		0		0
	0		0		0		0
	0		0		0		0
	0		0		0		0
	0		0		0		0
	0		0		0		0
	0		0		0		0
	0		0	Ewa-RG-top-a	1	Ewa-RG-top-a	1
	0		0	Ewa-RG-top-a	1	Ewa-RG-top-a	1
	0		0		0		0
	0		0		0		0
	0		0		0		0
	0		0		0		0
	0		0		0		0
	0		0		0		0
	0		0		0		0
	0		0		0		0

0	0		0		0
0	0		0		0
0	0		0		0
0	0		0		0
0	0		0		0
0	0		0		0
0	0		0		0
0	0		0		0
0	0		0		0
0	0		0		0
0	0		0		0
0	0	Ewa-RG-top-a	1	Ewa-RG-top-a	1
0	0		0		0
0	0		0		0
0	0		0		0
0	0		0		0
0	0		0		0
0	0		0		0
0	0		0		0
0	0		0		0
0	0		0		0
0	0		0		0
0	0		0		0
0	0		0		0
0	0		0		0
0	0		0		0
0	0		0		0
0	0		0		0
0	0		0		0
0	0		0		0
0	0		0		0
0	0		0		0
0	0		0		0
0	0		0		0
0	0		0		0
0	0		0		0
0	0		0		0
0	0		0		0
0	0		0		0
0	0		0		0
0	0		0		0
0	0		0		0
0	0		0		0
0	0		0		0
0	0		0		0
0	0		0		0
0	0		0		0

	0		0		0		0
	0		0		0		0
	0		0		0		0
	0		0		0		0
	0		0		0		0
	0		0		0		0
	0		0		0		0
	0		0		0		0
	0		0		0		0
	0		0		0		0
	0		0		0		0
	0		0		0		0
	0		0		0		0
	0		0		0		0
	0		0		0		0
	0		0		0		0
	0		0		0		0
	0		0		0		0
	0		0		0		0
	0		0		0		0
	0		0		0		0
	0		0		0		0
	0		0		0		0
Ew-AU->in-RG	1	Ew-AU->in-RG	1	Ewa-RG-top-a	1	Ewa-RG-top-a	1
	0		0		0		0
	0		0		0		0
	0		0		0		0
	0		0		0		0
	0		0		0		0
	0		0	Ewa-RG-top-a	1	Ewa-RG-top-a	1
	0		0		0		0
	0		0		0		0
	0		0		0		0
Ew-AU->in-RG	1		0		0		0
Ew-AU->in-RG	1		0		0		0
	0		0		0		0
	0		0		0		0
	0		0	Ewa-RG-top-a	1		0
	0		0	Ewa-RG-top-a	1		0
	0		0		0		0
	0		0		0		0
	0		0		0		0
	0		0		0		0
	0		0		0		0
	0		0		0		0
	0		0		0		0
	0		0		0		0

	0		0		0		0
	0		0		0		0
	0		0		0		0
	0		0		0		0
	0		0		0		0
	0		0		0		0
Ew-AU->in-RG	1	Ew-AU->in-RG	1	Ewa-RG-top-a	1	Ewa-RG-top-a	1
	0		0		0		0
	0		0		0		0
	0		0		0		0
	0		0		0		0
	0		0		0		0
	0		0		0		0
	0		0		0		0
	0		0		0		0
	0		0		0		0
	0		0		0		0
	0		0		0		0
	0		0		0		0
	0		0		0		0
	0		0		0		0
	0		0		0		0
	0		0		0		0
	0		0		0		0
	0		0		0		0
	0		0		0		0
	0		0		0		0
	0		0		0		0
	0		0		0		0
	0		0		0		0
	0		0		0		0
	0		0		0		0
	0		0		0		0
	0		0		0		0
	0		0		0		0
	0		0		0		0
	0		0		0		0
	0		0		0		0
	0		0		0		0
	0		0		0		0
	0		0		0		0
	0		0		0		0
	0		0		0		0
	0		0		0		0
	0		0		0		0
	0		0		0		0
	0		0		0		0

	0		0		0		0
	0		0		0		0
	0		0		0		0
	0		0		0		0
	0		0		0		0
	0		0		0		0
	0		0		0		0
	0		0		0		0
	0		0		0		0
	0		0		0		0
	0		0		0		0
	0		0		0		0
	0		0		0		0
	0		0		0		0
Ew-AU->in-RG	1		0		0		0
	0		0		0		0
	0		0		0		0
	0		0		0		0
	0		0		0		0
	0		0		0		0
	0		0		0		0
	0		0		0		0
Ew-AU->in-RG	1	Ew-AU->in-RG	1	Ewa-RG-top-a	1	Ewa-RG-top-a	1
	0		0		0		0
	0		0		0		0
	0		0		0		0
	0		0		0		0
Ew-AU->in-RG	1		0		0		0
	0		0		0		0
	0		0		0		0
	0		0		0		0
	0		0		0		0
	0		0		0		0
	0		0		0		0
	0		0		0		0
	0		0		0		0
Ew-AU->in-RG	1		0		0		0
Ew-AU->in-RG	1		0		0		0
Ew-AU->in-RG	1		0		0		0
	0		0		0		0
	0		0		0		0
	0		0		0		0
	0		0		0		0
	0		0		0		0
	0		0	Ewa-RG-top-a	1		0
	0		0	Ewa-RG-top-a	1		0
	0		0	Ewa-RG-top-a	1		0

0	0		0		0
0	0		0		0
0	0		0		0
0	0		0		0
0	0		0		0
0	0		0		0
0	0	Ewa-RG-top-a	1	Ewa-RG-top-a	1
0	0		0		0
0	0		0		0
0	0		0		0
0	0		0		0
0	0		0		0
0	0		0		0
0	0		0		0
0	0		0		0
0	0		0		0
0	0		0		0
0	0		0		0
0	0		0		0
0	0		0		0
0	0		0		0
0	0		0		0
0	0		0		0
0	0		0		0
0	0		0		0
0	0		0		0
0	0		0		0
0	0		0		0
0	0		0		0
0	0		0		0
0	0		0		0
0	0		0		0
0	0		0		0
0	0		0		0
0	0		0		0
0	0		0		0
0	0		0		0
0	0		0		0
0	0		0		0
0	0		0		0
0	0		0		0
0	0		0		0
0	0		0		0
0	0		0		0
0	0		0		0
0	0		0		0
0	0		0		0

	0		0		0		0
	0		0		0		0
	0		0		0		0
	0		0		0		0
	0		0		0		0
	0		0		0		0
	0		0		0		0
	0		0		0		0
	0		0		0		0
	0		0		0		0
Ew-AU->in-RG	1	Ew-AU->in-RG	1	Ewa-RG-top-a	1	Ewa-RG-top-a	1
	0		0	Ewa-RG-top-a	1	Ewa-RG-top-a	1
	0		0	Ewa-RG-top-a	1	Ewa-RG-top-a	1
	0		0		0		0
	0		0	Ewa-RG-top-a	1	Ewa-RG-top-a	1
	0		0		0		0
	0		0		0		0
	0		0		0		0
	0		0		0		0
	0		0		0		0
	0		0		0		0
	0		0		0		0
	0		0		0		0
	0		0		0		0
	0		0		0		0
	0		0		0		0
	0		0		0		0
	0		0		0		0
	0		0		0		0
	0		0		0		0
	0		0		0		0
	0		0		0		0
	0		0		0		0
Ew-AU->in-RG	1	Ew-AU->in-RG	1	Ewa-RG-top-a	1	Ewa-RG-top-a	1
Ew-AU->in-RG	1	Ew-AU->in-RG	1	Ewa-RG-top-a	1	Ewa-RG-top-a	1
	0		0		0		0
	0		0		0		0
	0		0		0		0
	0		0		0		0
	0		0		0		0
Ew-AU->in-RG	1	Ew-AU->in-RG	1	Ewa-RG-top-a	1	Ewa-RG-top-a	1
	0		0		0		0
	0		0		0		0
	0		0		0		0
	0		0		0		0
	0		0		0		0
	0		0	Ewa-RG-top-a	1	Ewa-RG-top-a	1

0	0	Ewa-RG-top-a	1	Ewa-RG-top-a	1
0	0		0		0
0	0		0		0
0	0		0		0
0	0		0		0
0	0		0		0
0	0		0		0
0	0		0		0
0	0		0		0
0	0		0		0
0	0		0		0
0	0		0		0
0	0		0		0
0	0		0		0
0	0		0		0
0	0		0		0
0	0		0		0
0	0		0		0
0	0		0		0
0	0		0		0
0	0		0		0
0	0		0		0
0	0		0		0
0	0		0		0
0	0		0		0
0	0		0		0
0	0		0		0
0	0		0		0
0	0		0		0
0	0		0		0
0	0		0		0
0	0		0		0
0	0		0		0
0	0		0		0
0	0		0		0
0	0		0		0
0	0		0		0
0	0		0		0
0	0		0		0
0	0		0		0
0	0		0		0
0	0		0		0
0	0		0		0
0	0		0		0
0	0		0		0
0	0		0		0
0	0		0		0

	0		0		0		0
	0		0		0		0
	0		0		0		0
	0		0		0		0
	0		0		0		0
	0		0		0		0
	0		0		0		0
	0		0		0		0
	0		0		0		0
	0		0		0		0
	0		0		0		0
	0		0		0		0
	0		0		0		0
	0		0		0		0
	0		0		0		0
	0		0		0		0
	0		0		0		0
	0		0		0		0
	0		0		0		0
	0		0		0		0
	0		0		0		0
	0		0		0		0
	0		0	Ewa-RG-top-a	1	Ewa-RG-top-a	1
	0		0	Ewa-RG-top-a	1	Ewa-RG-top-a	1
	0		0		0		0
	0		0	Ewa-RG-top-a	1	Ewa-RG-top-a	1
	0		0		0		0
	0		0		0		0
	0		0		0		0
	0		0		0		0
	0		0		0		0
	0		0		0		0
	0		0		0		0
	0		0		0		0
	0		0		0		0
Ew-AU->in-RG	1	Ew-AU->in-RG	1	Ewa-RG-top-a	1	Ewa-RG-top-a	1
	0		0		0		0
	0		0		0		0
	0		0		0		0
	0		0		0		0
	0		0		0		0
	0		0		0		0
	0		0		0		0
	0		0		0		0
	0		0		0		0
	0		0		0		0
	0		0		0		0

0	0		0		0
0	0		0		0
0	0		0		0
0	0		0		0
0	0		0		0
0	0		0		0
0	0		0		0
0	0		0		0
0	0		0		0
0	0	Ewa-RG-top-a	1	Ewa-RG-top-a	1
0	0	Ewa-RG-top-a	1	Ewa-RG-top-a	1
0	0		0		0
0	0		0		0
0	0		0		0
0	0		0		0
0	0		0		0
0	0		0		0
0	0		0		0
0	0		0		0
0	0	Ewa-RG-top-a	1	Ewa-RG-top-a	1
0	0	Ewa-RG-top-a	1	Ewa-RG-top-a	1
0	0		0		0
0	0		0		0
0	0		0		0
0	0		0		0
0	0		0		0
0	0		0		0
0	0		0		0
0	0		0		0
0	0		0		0
0	0		0		0
0	0		0		0
0	0		0		0
0	0		0		0
0	0		0		0
0	0		0		0
0	0		0		0
0	0		0		0
0	0		0		0
0	0		0		0
0	0		0		0
0	0		0		0
0	0		0		0
0	0		0		0
0	0		0		0
0	0		0		0
0	0		0		0

0	0		0		0
0	0		0		0
0	0	Ewa-RG-top-a	1	Ewa-RG-top-a	1
0	0	Ewa-RG-top-a	1	Ewa-RG-top-a	1
0	0		0		0
0	0		0		0
0	0		0		0
0	0		0		0
0	0		0		0
0	0		0		0
0	0		0		0
0	0		0		0
0	0		0		0
0	0		0		0
0	0		0		0
0	0		0		0
0	0		0		0
0	0		0		0
0	0		0		0
0	0		0		0
0	0		0		0
0	0		0		0
0	0		0		0
0	0		0		0
0	0		0		0
0	0		0		0
0	0		0		0
0	0		0		0
0	0		0		0
0	0		0		0
0	0		0		0
0	0		0		0
0	0		0		0
0	0		0		0
0	0		0		0
0	0		0		0
0	0		0		0
0	0		0		0
0	0		0		0
0	0		0		0
0	0		0		0
0	0		0		0
0	0		0		0
0	0		0		0
0	0		0		0
0	0		0		0
0	0		0		0

	0	0		0		0
	0	0	Ewa-RG-top-a	1	Ewa-RG-top-a	1
	0	0		0		0
	0	0		0		0
Ew-AU->in-RG	1	0	Ewa-RG-top-a	1	Ewa-RG-top-a	1
Ew-AU->in-RG	1	0	Ewa-RG-top-a	1	Ewa-RG-top-a	1
	0	0		0		0
	0	0		0		0
	0	0		0		0
	0	0		0		0
	0	0		0		0
	0	0		0		0
	0	0		0		0
	0	0		0		0
	0	0		0		0
	0	0		0		0
	0	0		0		0
	0	0		0		0
	0	0		0		0
	0	0		0		0
	0	0	Ewa-RG-top-a	1	Ewa-RG-top-a	1
	0	0		0		0
	0	0		0		0
	0	0		0		0
	0	0	Ewa-RG-top-a	1	Ewa-RG-top-a	1
	0	0		0		0
	0	0		0		0
	0	0		0		0
	0	0		0		0
	0	0		0		0
	0	0	Ewa-RG-top-a	1	Ewa-RG-top-a	1
	0	0		0		0
	0	0		0		0
	0	0		0		0
	0	0		0		0
	0	0		0		0
	0	0		0		0
	0	0		0		0
	0	0		0		0
	0	0		0		0
	0	0	Ewa-RG-top-a	1	Ewa-RG-top-a	1
	0	0	Ewa-RG-top-a	1	Ewa-RG-top-a	1
	0	0	Ewa-RG-top-a	1	Ewa-RG-top-a	1
	0	0		0		0
	0	0		0		0
	0	0		0		0
	0	0		0		0

	0		0		0		0
	0		0		0		0
	0		0		0		0
	0		0		0		0
	0		0		0		0
	0		0		0		0
	0		0		0		0
Ew-AU->in-RG	1	Ew-AU->in-RG	1	Ewa-RG-top-a	1	Ewa-RG-top-a	1
Ew-AU->in-RG	1	Ew-AU->in-RG	1	Ewa-RG-top-a	1	Ewa-RG-top-a	1
	0		0		0		0
	0		0		0		0
	0		0		0		0
	0		0		0		0
	0		0		0		0
	0		0		0		0
	0		0		0		0
	0		0		0		0
	0		0		0		0
	0		0		0		0
	0		0		0		0
	0		0		0		0
	0		0		0		0
	0		0		0		0
	0		0		0		0
	0		0		0		0
	0		0		0		0
	0		0		0		0
	0		0		0		0
	0		0		0		0
	0		0		0		0
	0		0		0		0
	0		0		0		0
	0		0		0		0
	0		0		0		0
	0		0		0		0
	0		0		0		0
	0		0		0		0
	0		0		0		0
	0		0		0		0
	0		0		0		0
	0		0		0		0
	0		0		0		0
	0		0		0		0
	0		0		0		0
	0		0		0		0
	0		0		0		0
	0		0		0		0

0	0		0		0
0	0	Ewa-RG-top-a	1	Ewa-RG-top-a	1
0	0		0		0
0	0		0		0
0	0		0		0
0	0		0		0
0	0		0		0
0	0		0		0
0	0		0		0
0	0		0		0
0	0		0		0
0	0		0		0
0	0		0		0
0	0		0		0
0	0		0		0
0	0		0		0
0	0		0		0
0	0		0		0
0	0		0		0
0	0		0		0
0	0		0		0
0	0		0		0
0	0		0		0
0	0		0		0
0	0		0		0
0	0		0		0
0	0		0		0
0	0		0		0
0	0		0		0
0	0		0		0
0	0		0		0
0	0		0		0
0	0		0		0
0	0		0		0
0	0		0		0
0	0		0		0
0	0		0		0
0	0		0		0
0	0		0		0
0	0		0		0
0	0		0		0
0	0		0		0
0	0		0		0
0	0		0		0
0	0		0		0
0	0		0		0
0	0		0		0

	0	0		0		0
	0	0		0		0
	0	0		0		0
	0	0		0		0
	0	0		0		0
	0	0		0		0
	0	0		0		0
	0	0	Ewa-RG-top-a	1	Ewa-RG-top-a	1
	0	0	Ewa-RG-top-a	1	Ewa-RG-top-a	1
	0	0		0		0
	0	0		0		0
Ew-AU->in-RG	1	0		0		0
	0	0		0		0
	0	0		0		0
	0	0		0		0
	0	0		0		0
	0	0		0		0
	0	0		0		0
	0	0		0		0
	0	0		0		0
	0	0		0		0
	0	0		0		0
	0	0		0		0
	0	0		0		0
	0	0		0		0
	0	0		0		0
	0	0		0		0
	0	0		0		0
	0	0		0		0
	0	0		0		0
	0	0		0		0
	0	0		0		0
	0	0				
	0	0				
	0	0				
	0	0				
	0	0				
	0	0				
	0	0				
	0	0				
	0	0				
	0	0				
	0	0				
	0	0				
	0	0				
	0	0				
	0	0				

0	0
0	0
0	0
0	0
0	0
0	0
0	0
0	0
0	0
0	0
0	0
0	0
0	0
0	0
0	0
0	0
0	0
0	0
0	0
0	0
0	0
0	0
0	0
0	0
0	0
0	0
0	0
0	0
0	0
0	0
0	0
0	0
0	0
0	0
0	0
0	0
0	0
0	0
0	0
0	0
0	0
0	0
0	0
0	0
0	0
0	0
0	0

0	0
0	0
0	0
0	0
0	0
0	0
0	0
0	0
0	0
0	0
0	0
0	0
0	0
0	0
0	0
0	0
0	0
0	0
0	0
0	0
0	0
0	0
0	0
0	0
0	0
0	0
0	0
0	0
0	0
0	0
0	0
0	0
0	0
0	0
0	0
0	0
0	0
0	0
0	0
0	0
0	0
0	0
0	0
0	0
0	0
0	0
0	0

0	0
0	0
0	0
0	0
0	0
0	0
0	0
0	0
0	0
0	0
0	0
0	0
0	0
0	0
0	0
0	0
0	0
0	0
0	0
0	0
0	0
0	0
0	0
0	0
0	0
0	0
0	0
0	0
0	0
0	0
0	0
0	0
0	0
0	0
0	0
0	0
0	0
0	0
0	0
0	0
0	0
0	0
0	0
0	0
0	0
0	0
0	0

0	0
0	0
0	0
0	0
0	0
0	0
0	0
0	0
0	0
0	0
0	0
0	0
0	0
0	0
0	0
0	0
0	0
0	0
0	0
0	0
0	0
0	0
0	0
0	0
0	0
0	0
0	0
0	0
0	0
0	0
0	0
0	0
0	0
0	0
0	0
0	0
0	0
0	0
0	0
0	0
0	0
0	0
0	0
0	0
0	0
0	0
0	0

0	0
0	0
0	0
0	0
0	0
0	0
0	0
0	0
0	0
0	0
0	0
0	0
0	0
0	0
0	0
0	0
0	0
0	0
0	0
0	0
0	0
0	0
0	0
0	0
0	0
0	0
0	0
0	0
0	0
0	0
0	0
0	0
0	0
0	0
0	0
0	0
0	0
0	0
0	0
0	0
0	0
0	0
0	0
0	0
0	0
0	0
0	0

0	0
0	0
0	0
0	0
0	0
0	0
0	0
0	0
0	0
0	0
0	0
0	0
0	0
0	0
0	0
0	0
0	0
0	0
0	0
0	0
0	0
0	0
0	0
0	0
0	0
0	0
0	0
0	0
0	0
0	0
0	0
0	0
0	0
0	0
0	0
0	0
0	0
0	0
0	0
0	0
0	0
0	0
0	0
0	0
0	0
0	0
0	0

0	0
0	0
0	0
0	0
0	0
0	0
0	0
0	0
0	0
0	0
0	0
0	0
0	0
0	0
0	0
0	0
0	0
0	0
0	0
0	0
0	0
0	0
0	0
0	0
0	0
0	0
0	0
0	0
0	0
0	0
0	0
0	0
0	0
0	0
0	0
0	0
0	0
0	0
0	0
0	0
0	0
0	0
0	0
0	0
0	0
0	0
0	0

0	0
0	0
0	0
0	0
0	0
0	0
0	0
0	0
0	0
0	0
0	0
0	0
0	0
0	0
0	0
0	0
0	0
0	0
0	0
0	0
0	0
0	0
0	0
0	0
0	0
0	0
0	0
0	0
0	0
0	0
0	0
0	0
0	0
0	0
0	0
0	0
0	0
0	0
0	0
0	0
0	0
0	0
0	0
0	0
0	0
0	0
0	0

0	0
0	0
0	0
0	0
0	0
0	0
0	0
0	0
0	0
0	0
0	0
0	0
0	0
0	0
0	0
0	0
0	0
0	0
0	0
0	0
0	0
0	0
0	0
0	0
0	0
0	0
0	0
0	0
0	0
0	0
0	0
0	0
0	0
0	0
0	0
0	0
0	0
0	0
0	0
0	0
0	0
0	0
0	0
0	0
0	0
0	0
0	0

0	0
0	0
0	0
0	0
0	0
0	0
0	0
0	0
0	0
0	0
0	0
0	0
0	0
0	0
0	0
0	0
0	0
0	0
0	0
0	0
0	0
0	0
0	0
0	0
0	0
0	0
0	0
0	0
0	0
0	0
0	0
0	0
0	0
0	0
0	0
0	0
0	0
0	0
0	0
0	0
0	0
0	0
0	0
0	0
0	0
0	0
0	0

0	0
0	0
0	0
0	0
0	0
0	0
0	0
0	0
0	0
0	0
0	0
0	0
0	0
0	0
0	0
0	0
0	0
0	0
0	0
0	0
0	0
0	0
0	0
0	0
0	0
0	0
0	0
0	0
0	0
0	0
0	0
0	0
0	0
0	0
0	0
0	0
0	0
0	0
0	0
0	0
0	0
0	0
0	0
0	0
0	0
0	0
0	0

0	0
0	0
0	0
0	0
0	0
0	0
0	0
0	0
0	0
0	0
0	0
0	0
0	0
0	0
0	0
0	0
0	0
0	0
0	0
0	0
0	0
0	0
0	0
0	0
0	0
0	0
0	0
0	0
0	0
0	0
0	0
0	0
0	0
0	0
0	0
0	0
0	0
0	0
0	0
0	0
0	0
0	0
0	0
0	0
0	0
0	0
0	0

0	0
0	0
0	0
0	0
0	0
0	0
0	0
0	0
0	0
0	0
0	0
0	0
0	0
0	0
0	0
0	0
0	0
0	0
0	0
0	0
0	0
0	0
0	0
0	0
0	0
0	0
0	0
0	0
0	0
0	0
0	0
0	0
0	0
0	0
0	0
0	0
0	0
0	0
0	0
0	0
0	0
0	0
0	0
0	0
0	0
0	0
0	0

0	0
0	0
0	0
0	0
0	0
0	0
0	0
0	0
0	0
0	0
0	0
0	0
0	0
0	0
0	0
0	0
0	0
0	0
0	0
0	0
0	0
0	0
0	0
0	0
0	0
0	0
0	0
0	0
0	0
0	0
0	0
0	0
0	0
0	0
0	0
0	0
0	0
0	0
0	0
0	0
0	0
0	0
0	0
0	0
0	0
0	0
0	0

0	0
0	0
0	0
0	0
0	0
0	0
0	0
0	0
0	0
0	0
0	0
0	0
0	0
0	0
0	0
0	0
0	0
0	0
0	0
0	0
0	0
0	0
0	0
0	0
0	0
0	0
0	0
0	0
0	0
0	0
0	0
0	0
0	0
0	0
0	0
0	0
0	0
0	0
0	0
0	0
0	0
0	0
0	0
0	0
0	0
0	0
0	0

0	0
0	0
0	0
0	0
0	0
0	0
0	0
0	0
0	0
0	0
0	0
0	0
0	0
0	0
0	0
0	0
0	0
0	0
0	0
0	0
0	0
0	0
0	0
0	0
0	0
0	0
0	0
0	0
0	0
0	0
0	0
0	0
0	0
0	0
0	0
0	0
0	0
0	0
0	0
0	0
0	0
0	0
0	0
0	0
0	0
0	0
0	0

0	0
0	0
0	0
0	0
0	0
0	0
0	0
0	0
0	0
0	0
0	0
0	0
0	0
0	0
0	0
0	0
0	0
0	0
0	0
0	0
0	0
0	0
0	0
0	0
0	0
0	0
0	0
0	0
0	0
0	0
0	0
0	0
0	0
0	0
0	0
0	0
0	0
0	0
0	0
0	0
0	0
0	0
0	0
0	0
0	0
0	0
0	0

0	0
0	0
0	0
0	0
0	0
0	0
0	0
0	0
0	0
0	0
0	0
0	0
0	0
0	0
0	0
0	0
0	0
0	0
0	0
0	0
0	0
0	0
0	0
0	0
0	0
0	0
0	0
0	0
0	0
0	0
0	0
0	0
0	0
0	0
0	0
0	0
0	0
0	0
0	0
0	0
0	0
0	0
0	0
0	0
0	0
0	0
0	0

0	0
0	0
0	0
0	0
0	0
0	0
0	0
0	0
0	0
0	0
0	0
0	0
0	0
0	0
0	0
0	0
0	0
0	0
0	0
0	0
0	0
0	0
0	0
0	0
0	0
0	0
0	0
0	0
0	0
0	0
0	0
0	0
0	0
0	0
0	0
0	0
0	0
0	0
0	0
0	0
0	0
0	0
0	0
0	0
0	0
0	0
0	0

0	0
0	0
0	0
0	0
0	0
0	0
0	0
0	0
0	0
0	0
0	0
0	0
0	0
0	0
0	0
0	0
0	0
0	0
0	0
0	0
0	0
0	0
0	0
0	0
0	0
0	0
0	0
0	0
0	0
0	0
0	0
0	0
0	0
0	0
0	0
0	0
0	0
0	0
0	0
0	0
0	0
0	0
0	0
0	0
0	0
0	0
0	0

0	0
0	0
0	0
0	0
0	0
0	0
0	0
0	0
0	0
0	0
0	0
0	0
0	0
0	0
0	0
0	0
0	0
0	0
0	0
0	0
0	0
0	0
0	0
0	0
0	0
0	0
0	0
0	0
0	0
0	0
0	0
0	0
0	0
0	0
0	0
0	0
0	0
0	0
0	0
0	0
0	0
0	0
0	0
0	0
0	0
0	0
0	0

0	0
0	0
0	0
0	0
0	0
0	0
0	0
0	0
0	0
0	0
0	0
0	0
0	0
0	0
0	0
0	0
0	0
0	0
0	0
0	0
0	0
0	0
0	0
0	0
0	0
0	0
0	0
0	0
0	0
0	0
0	0
0	0
0	0
0	0
0	0
0	0
0	0
0	0
0	0
0	0
0	0
0	0
0	0
0	0
0	0
0	0
0	0

0	0
0	0
0	0
0	0
0	0
0	0
0	0
0	0
0	0
0	0
0	0
0	0
0	0
0	0
0	0
0	0
0	0
0	0
0	0
0	0
0	0
0	0
0	0
0	0
0	0
0	0
0	0
0	0
0	0
0	0
0	0
0	0
0	0
0	0
0	0
0	0
0	0
0	0
0	0
0	0
0	0
0	0
0	0
0	0
0	0
0	0
0	0

0	0
0	0
0	0
0	0
0	0
0	0
0	0
0	0
0	0
0	0
0	0
0	0
0	0
0	0
0	0
0	0
0	0
0	0
0	0
0	0
0	0
0	0
0	0
0	0
0	0
0	0
0	0
0	0
0	0
0	0
0	0
0	0
0	0
0	0
0	0
0	0
0	0
0	0
0	0
0	0
0	0
0	0
0	0
0	0
0	0
0	0
0	0

0	0
0	0
0	0
0	0
0	0
0	0
0	0
0	0
0	0
0	0
0	0
0	0
0	0
0	0
0	0
0	0
0	0
0	0
0	0
0	0
0	0
0	0
0	0
0	0
0	0
0	0
0	0
0	0
0	0
0	0
0	0
0	0
0	0
0	0
0	0
0	0
0	0
0	0
0	0
0	0
0	0
0	0
0	0
0	0
0	0
0	0
0	0

0	0
0	0
0	0
0	0
0	0
0	0
0	0
0	0
0	0
0	0
0	0
0	0
0	0
0	0
0	0
0	0
0	0
0	0
0	0
0	0
0	0
0	0
0	0
0	0
0	0
0	0
0	0
0	0
0	0
0	0
0	0
0	0
0	0
0	0
0	0
0	0
0	0
0	0
0	0
0	0
0	0
0	0
0	0
0	0
0	0
0	0
0	0

0	0
0	0
0	0
0	0
0	0
0	0
0	0
0	0
0	0
0	0
0	0
0	0
0	0
0	0
0	0
0	0
0	0
0	0
0	0
0	0
0	0
0	0
0	0
0	0
0	0
0	0
0	0
0	0
0	0
0	0
0	0
0	0
0	0
0	0
0	0
0	0
0	0
0	0
0	0
0	0
0	0
0	0
0	0
0	0
0	0
0	0
0	0

0	0
0	0
0	0
0	0
0	0
0	0
0	0
0	0
0	0
0	0
0	0
0	0
0	0
0	0
0	0
0	0
0	0
0	0
0	0
0	0
0	0
0	0
0	0
0	0
0	0
0	0
0	0
0	0
0	0
0	0
0	0
0	0
0	0
0	0
0	0
0	0
0	0
0	0
0	0
0	0
0	0
0	0
0	0
0	0
0	0
0	0
0	0

0	0
0	0
0	0
0	0
0	0
0	0
0	0
0	0
0	0
0	0
0	0
0	0
0	0
0	0
0	0
0	0
0	0
0	0
0	0
0	0
0	0
0	0
0	0
0	0
0	0
0	0
0	0
0	0
0	0
0	0
0	0
0	0
0	0
0	0
0	0
0	0
0	0
0	0
0	0
0	0
0	0
0	0
0	0
0	0
0	0
0	0
0	0

0	0
0	0
0	0
0	0
0	0
0	0
0	0
0	0
0	0
0	0
0	0
0	0
0	0
0	0
0	0
0	0
0	0
0	0
0	0
0	0
0	0
0	0
0	0
0	0
0	0
0	0
0	0
0	0
0	0
0	0
0	0
0	0
0	0
0	0
0	0
0	0
0	0
0	0
0	0
0	0
0	0
0	0
0	0
0	0
0	0
0	0
0	0

0	0
0	0
0	0
0	0
0	0
0	0
0	0
0	0
0	0
0	0
0	0
0	0
0	0
0	0
0	0
0	0
0	0
0	0
0	0
0	0
0	0
0	0
0	0
0	0
0	0
0	0
0	0
0	0
0	0
0	0
0	0
0	0
0	0
0	0
0	0
0	0
0	0
0	0
0	0
0	0
0	0
0	0
0	0
0	0
0	0
0	0
0	0

0	0
0	0
0	0
0	0
0	0
0	0
0	0
0	0
0	0
0	0
0	0
0	0
0	0
0	0
0	0
0	0
0	0
0	0
0	0
0	0
0	0
0	0
0	0
0	0
0	0
0	0
0	0
0	0
0	0
0	0
0	0
0	0
0	0
0	0
0	0
0	0
0	0
0	0
0	0
0	0
0	0
0	0
0	0
0	0
0	0
0	0
0	0

0	0
0	0
0	0
0	0
0	0
0	0
0	0
0	0
0	0
0	0
0	0
0	0
0	0
0	0
0	0
0	0
0	0
0	0
0	0
0	0
0	0
0	0
0	0
0	0
0	0
0	0
0	0
0	0
0	0
0	0
0	0
0	0
0	0
0	0
0	0
0	0
0	0
0	0
0	0
0	0
0	0
0	0
0	0
0	0
0	0
0	0
0	0

0	0
0	0
0	0
0	0
0	0
0	0
0	0
0	0
0	0
0	0
0	0
0	0
0	0
0	0
0	0
0	0
0	0
0	0
0	0
0	0
0	0
0	0
0	0
0	0
0	0
0	0
0	0
0	0
0	0
0	0
0	0
0	0
0	0
0	0
0	0
0	0
0	0
0	0
0	0
0	0
0	0
0	0
0	0
0	0
0	0
0	0
0	0

0	0
0	0
0	0
0	0
0	0
0	0
0	0
0	0
0	0
0	0
0	0
0	0
0	0
0	0
0	0
0	0
0	0
0	0
0	0
0	0
0	0
0	0
0	0
0	0
0	0
0	0
0	0
0	0
0	0
0	0
0	0
0	0
0	0
0	0
0	0
0	0
0	0
0	0
0	0
0	0
0	0
0	0
0	0
0	0
0	0
0	0
0	0

0	0
0	0
0	0
0	0
0	0
0	0
0	0
0	0
0	0
0	0
0	0
0	0
0	0
0	0
0	0
0	0
0	0
0	0
0	0
0	0
0	0
0	0
0	0
0	0
0	0
0	0
0	0
0	0
0	0
0	0
0	0
0	0
0	0
0	0
0	0
0	0
0	0
0	0
0	0
0	0
0	0
0	0
0	0
0	0
0	0
0	0
0	0

0	0
0	0
0	0
0	0
0	0
0	0
0	0
0	0
0	0
0	0
0	0
0	0
0	0
0	0
0	0
0	0
0	0
0	0
0	0
0	0
0	0
0	0
0	0
0	0
0	0
0	0
0	0
0	0
0	0
0	0
0	0
0	0
0	0
0	0
0	0
0	0
0	0
0	0
0	0
0	0
0	0
0	0
0	0
0	0
0	0
0	0
0	0

0	0
0	0
0	0
0	0
0	0
0	0
0	0
0	0
0	0
0	0
0	0
0	0
0	0
0	0
0	0
0	0
0	0
0	0
0	0
0	0
0	0
0	0
0	0
0	0
0	0
0	0
0	0
0	0
0	0
0	0
0	0
0	0
0	0
0	0
0	0
0	0
0	0
0	0
0	0
0	0
0	0
0	0
0	0
0	0
0	0
0	0
0	0

0	0
0	0
0	0
0	0
0	0
0	0
0	0
0	0
0	0
0	0
0	0
0	0
0	0
0	0
0	0
0	0
0	0
0	0
0	0
0	0
0	0
0	0
0	0
0	0
0	0
0	0
0	0
0	0
0	0
0	0
0	0
0	0
0	0
0	0
0	0
0	0
0	0
0	0
0	0
0	0
0	0
0	0
0	0
0	0
0	0
0	0
0	0

0	0
0	0
0	0
0	0
0	0
0	0
0	0
0	0
0	0
0	0
0	0
0	0
0	0
0	0
0	0
0	0
0	0
0	0
0	0
0	0
0	0
0	0
0	0
0	0
0	0
0	0
0	0
0	0
0	0
0	0
0	0
0	0
0	0
0	0
0	0
0	0
0	0
0	0
0	0
0	0
0	0
0	0
0	0
0	0
0	0
0	0
0	0

0	0
0	0
0	0
0	0
0	0
0	0
0	0
0	0
0	0
0	0
0	0
0	0
0	0
0	0
0	0
0	0
0	0
0	0
0	0
0	0
0	0
0	0
0	0
0	0
0	0
0	0
0	0
0	0
0	0
0	0
0	0
0	0
0	0
0	0
0	0
0	0
0	0
0	0
0	0
0	0
0	0
0	0
0	0
0	0
0	0
0	0
0	0

0	0
0	0
0	0
0	0
0	0
0	0
0	0
0	0
0	0
0	0
0	0
0	0
0	0
0	0
0	0
0	0
0	0
0	0
0	0
0	0
0	0
0	0
0	0
0	0
0	0
0	0
0	0
0	0
0	0
0	0
0	0
0	0
0	0
0	0
0	0
0	0
0	0
0	0
0	0
0	0
0	0
0	0
0	0
0	0
0	0
0	0
0	0

0	0
0	0
0	0
0	0
0	0
0	0
0	0
0	0
0	0
0	0
0	0
0	0
0	0
0	0
0	0
0	0
0	0
0	0
0	0
0	0
0	0
0	0
0	0
0	0
0	0
0	0
0	0
0	0
0	0
0	0
0	0
0	0
0	0
0	0
0	0
0	0
0	0
0	0
0	0
0	0
0	0
0	0
0	0
0	0
0	0
0	0
0	0

0	0
0	0
0	0
0	0
0	0
0	0
0	0
0	0
0	0
0	0
0	0
0	0
0	0
0	0
0	0
0	0
0	0
0	0
0	0
0	0
0	0
0	0
0	0
0	0
0	0
0	0
0	0
0	0
0	0
0	0
0	0
0	0
0	0
0	0
0	0
0	0
0	0
0	0
0	0
0	0
0	0
0	0
0	0
0	0
0	0
0	0
0	0

0	0
0	0
0	0
0	0
0	0
0	0
0	0
0	0
0	0
0	0
0	0
0	0
0	0
0	0
0	0
0	0
0	0
0	0
0	0
0	0
0	0
0	0
0	0
0	0
0	0
0	0
0	0
0	0
0	0
0	0
0	0
0	0
0	0
0	0
0	0
0	0
0	0
0	0
0	0
0	0
0	0
0	0
0	0
0	0
0	0
0	0
0	0

0	0
0	0
0	0
0	0
0	0
0	0
0	0
0	0
0	0
0	0
0	0
0	0
0	0
0	0
0	0
0	0
0	0
0	0
0	0
0	0
0	0
0	0
0	0
0	0
0	0
0	0
0	0
0	0
0	0
0	0
0	0
0	0
0	0
0	0
0	0
0	0
0	0
0	0
0	0
0	0
0	0
0	0
0	0
0	0
0	0
0	0
0	0

0	0
0	0
0	0
0	0
0	0
0	0
0	0
0	0
0	0
0	0
0	0
0	0
0	0
0	0
0	0
0	0
0	0
0	0
0	0
0	0
0	0
0	0
0	0
0	0
0	0
0	0
0	0
0	0
0	0
0	0
0	0
0	0
0	0
0	0
0	0
0	0
0	0
0	0
0	0
0	0
0	0
0	0
0	0
0	0
0	0
0	0
0	0

0	0
0	0
0	0
0	0
0	0
0	0
0	0
0	0
0	0
0	0
0	0
0	0
0	0
0	0
0	0
0	0
0	0
0	0
0	0
0	0
0	0
0	0
0	0
0	0
0	0
0	0
0	0
0	0
0	0
0	0
0	0
0	0
0	0
0	0
0	0
0	0
0	0
0	0
0	0
0	0
0	0
0	0
0	0
0	0
0	0
0	0
0	0

0	0
0	0
0	0
0	0
0	0
0	0
0	0
0	0
0	0
0	0
0	0
0	0
0	0
0	0
0	0
0	0
0	0
0	0
0	0
0	0
0	0
0	0
0	0
0	0
0	0
0	0
0	0
0	0
0	0
0	0
0	0
0	0
0	0
0	0
0	0
0	0
0	0
0	0
0	0
0	0
0	0
0	0
0	0
0	0
0	0
0	0
0	0

0	0
0	0
0	0
0	0
0	0
0	0
0	0
0	0
0	0
0	0
0	0
0	0
0	0
0	0
0	0
0	0
0	0
0	0
0	0
0	0
0	0
0	0
0	0
0	0
0	0
0	0
0	0
0	0
0	0
0	0
0	0
0	0
0	0
0	0
0	0
0	0
0	0
0	0
0	0
0	0
0	0
0	0
0	0
0	0
0	0
0	0
0	0

0	0
0	0
0	0
0	0
0	0
0	0
0	0
0	0
0	0
0	0
0	0
0	0
0	0
0	0
0	0
0	0
0	0
0	0
0	0
0	0
0	0
0	0
0	0
0	0
0	0
0	0
0	0
0	0
0	0
0	0
0	0
0	0
0	0
0	0
0	0
0	0
0	0
0	0
0	0
0	0
0	0
0	0
0	0
0	0
0	0
0	0
0	0

0	0
0	0
0	0
0	0
0	0
0	0
0	0
0	0
0	0
0	0
0	0
0	0
0	0
0	0
0	0
0	0
0	0
0	0
0	0
0	0
0	0
0	0
0	0
0	0
0	0
0	0
0	0
0	0
0	0
0	0
0	0

entrywithaau	ewauinrgartg	ewauinrgartg	ewauinrg_pi_	ewauinrg_pi_	ewargart_au_	ewargart_au_	ewargart_au_
		0		0		0	
		0		0		0	
		0		0		0	
		0		0		0	
		0		0		0	
		0		0		0	
		0		0		0	
		0		0		0	
		0		0		0	
		0		0		0	
	Ew-AU->in-RG	1	Ew-AU->in-RG	1	Ewa-RG-art_E	1	Ewa-RG-art_E
		0		0		0	
		0		0		0	
		0		0		0	
		0		0		0	
		0		0		0	
		0		0		0	
		0		0		0	
		0		0		0	
		0		0		0	
		0		0		0	
		0		0		0	
	Ew-AU->in-RG	1	Ew-AU->in-RG	1	Ewa-RG-art_E	1	Ewa-RG-art_E
	Ew-AU->in-RG	1	Ew-AU->in-RG	1	Ewa-RG-art_E	1	Ewa-RG-art_E
	Ew-AU->in-RG	1	Ew-AU->in-RG	1	Ewa-RG-art_E	1	Ewa-RG-art_E
		0		0		0	
		0		0		0	
		0		0		0	
		0		0		0	
	Ew-AU->in-RG	1	Ew-AU->in-RG	1	Ewa-RG-art_E	1	Ewa-RG-art_E
		0		0		0	
		0		0		0	
		0		0		0	
		0		0		0	
		0		0		0	
		0		0		0	
		0		0		0	
		0		0		0	
		0		0		0	
		0		0		0	
		0		0		0	
		0		0		0	
	Ew-AU->in-RG	1	Ew-AU->in-RG	1	Ewa-RG-art_E	1	Ewa-RG-art_E
	Ew-AU->in-RG	1	Ew-AU->in-RG	1	Ewa-RG-art_E	1	Ewa-RG-art_E
	Ew-AU->in-RG	1	Ew-AU->in-RG	1	Ewa-RG-art_E	1	Ewa-RG-art_E
		0		0		0	

	0		0		0	
	0		0		0	
Ew-AU->in-RG	1	Ew-AU->in-RG	1	Ewa-RG-art_E	1	Ewa-RG-art_E
	0		0		0	
Ew-AU->in-RG	1	Ew-AU->in-RG	1	Ewa-RG-art_E	1	Ewa-RG-art_E
	0		0		0	
	0		0		0	
Ew-AU->in-RG	1	Ew-AU->in-RG	1	Ewa-RG-art_E	1	Ewa-RG-art_E
Ew-AU->in-RG	1		0		0	
	0		0		0	
	0		0		0	
	0		0		0	
	0		0		0	
Ew-AU->in-RG	1	Ew-AU->in-RG	1	Ewa-RG-art_E	1	Ewa-RG-art_E
Ew-AU->in-RG	1		0		0	
Ew-AU->in-RG	1		0		0	
	0		0		0	
	0		0		0	
	0		0		0	
	0		0		0	
	0		0		0	
	0		0		0	
	0		0		0	
	0		0		0	
Ew-AU->in-RG	1	Ew-AU->in-RG	1	Ewa-RG-art_E	1	Ewa-RG-art_E
Ew-AU->in-RG	1	Ew-AU->in-RG	1	Ewa-RG-art_E	1	Ewa-RG-art_E
	0		0		0	
	0		0		0	
	0		0		0	
Ew-AU->in-RG	1	Ew-AU->in-RG	1	Ewa-RG-art_E	1	Ewa-RG-art_E
Ew-AU->in-RG	1	Ew-AU->in-RG	1	Ewa-RG-art_E	1	Ewa-RG-art_E
Ew-AU->in-RG	1	Ew-AU->in-RG	1	Ewa-RG-art_E	1	Ewa-RG-art_E
Ew-AU->in-RG	1	Ew-AU->in-RG	1	Ewa-RG-art_E	1	Ewa-RG-art_E
Ew-AU->in-RG	1	Ew-AU->in-RG	1	Ewa-RG-art_E	1	Ewa-RG-art_E
	0		0		0	
	0		0		0	
	0		0		0	
	0		0		0	
	0		0		0	
	0		0		0	
	0		0		0	
	0		0		0	
	0		0		0	
	0		0		0	
	0		0		0	
Ew-AU->in-RG	1	Ew-AU->in-RG	1	Ewa-RG-art_E	1	Ewa-RG-art_E
	0		0		0	

	0		0		0	
	0		0		0	
	0		0		0	
Ew-AU->in-RG	1	Ew-AU->in-RG	1	Ewa-RG-art_E	1	Ewa-RG-art_E
	0		0		0	
	0		0		0	
	0		0		0	
	0		0		0	
Ew-AU->in-RG	1	Ew-AU->in-RG	1	Ewa-RG-art_E	1	Ewa-RG-art_E
	0		0		0	
	0		0		0	
	0		0		0	
	0		0		0	
Ew-AU->in-RG	1	Ew-AU->in-RG	1	Ewa-RG-art_E	1	Ewa-RG-art_E
	0		0		0	
	0		0		0	
	0		0		0	
	0		0		0	
	0		0		0	
	0		0		0	
Ew-AU->in-RG	1	Ew-AU->in-RG	1	Ewa-RG-art_E	1	Ewa-RG-art_E
	0		0		0	
	0		0		0	
	0		0		0	
Ew-AU->in-RG	1		0		0	
Ew-AU->in-RG	1	Ew-AU->in-RG	1	Ewa-RG-art_E	1	Ewa-RG-art_E
Ew-AU->in-RG	1	Ew-AU->in-RG	1	Ewa-RG-art_E	1	Ewa-RG-art_E
	0		0		0	
	0		0		0	
Ew-AU->in-RG	1	Ew-AU->in-RG	1	Ewa-RG-art_E	1	Ewa-RG-art_E
Ew-AU->in-RG	1	Ew-AU->in-RG	1	Ewa-RG-art_E	1	Ewa-RG-art_E
	0		0		0	
	0		0		0	
	0		0		0	
Ew-AU->in-RG	1	Ew-AU->in-RG	1	Ewa-RG-art_E	1	Ewa-RG-art_E
Ew-AU->in-RG	1	Ew-AU->in-RG	1	Ewa-RG-art_E	1	Ewa-RG-art_E
Ew-AU->in-RG	1	Ew-AU->in-RG	1	Ewa-RG-art_E	1	Ewa-RG-art_E
Ew-AU->in-RG	1	Ew-AU->in-RG	1	Ewa-RG-art_E	1	Ewa-RG-art_E
Ew-AU->in-RG	1	Ew-AU->in-RG	1	Ewa-RG-art_E	1	Ewa-RG-art_E
Ew-AU->in-RG	1	Ew-AU->in-RG	1	Ewa-RG-art_E	1	Ewa-RG-art_E
Ew-AU->in-RG	1		0	Ewa-RG-art_E	1	
Ew-AU->in-RG	1		0	Ewa-RG-art_E	1	
	0		0		0	
	0		0		0	
	0		0		0	
	0		0		0	
	0		0		0	

	0		0		0	
	0		0		0	
	0		0		0	
	0		0		0	
	0		0		0	
	0		0		0	
Ew-AU->in-RG	1	Ew-AU->in-RG	1	Ewa-RG-art_E	1	Ewa-RG-art_E
Ew-AU->in-RG	1	Ew-AU->in-RG	1	Ewa-RG-art_E	1	Ewa-RG-art_E
	0		0		0	
	0		0		0	
	0		0		0	
	0		0		0	
	0		0		0	
	0		0		0	
	0		0		0	
	0		0		0	
Ew-AU->in-RG	1		0	Ewa-RG-art_E	1	
	0		0		0	
	0		0		0	
	0		0		0	
	0		0		0	
	0		0		0	
Ew-AU->in-RG	1	Ew-AU->in-RG	1	Ewa-RG-art_E	1	Ewa-RG-art_E
	0		0		0	
	0		0		0	
	0		0		0	
	0		0		0	
Ew-AU->in-RG	1	Ew-AU->in-RG	1	Ewa-RG-art_E	1	Ewa-RG-art_E
	0		0		0	
Ew-AU->in-RG	1	Ew-AU->in-RG	1	Ewa-RG-art_E	1	Ewa-RG-art_E
	0		0		0	
	0		0		0	
	0		0		0	
	0		0		0	
Ew-AU->in-RG	1		0	Ewa-RG-art_E	1	
Ew-AU->in-RG	1		0	Ewa-RG-art_E	1	
	0		0		0	
Ew-AU->in-RG	1		0	Ewa-RG-art_E	1	
Ew-AU->in-RG	1	Ew-AU->in-RG	1	Ewa-RG-art_E	1	Ewa-RG-art_E
	0		0		0	
Ew-AU->in-RG	1		0	Ewa-RG-art_E	1	
Ew-AU->in-RG	1	Ew-AU->in-RG	1	Ewa-RG-art_E	1	Ewa-RG-art_E
Ew-AU->in-RG	1	Ew-AU->in-RG	1	Ewa-RG-art_E	1	Ewa-RG-art_E
	0		0		0	
	0		0		0	
	0		0		0	
	0		0		0	

	0		0		0	
	0		0		0	
Ew-AU->in-RG	1	Ew-AU->in-RG	1	Ewa-RG-art_E	1	Ewa-RG-art_E
	0		0		0	
	0		0		0	
	0		0		0	
	0		0		0	
	0		0		0	
	0		0		0	
Ew-AU->in-RG	1	Ew-AU->in-RG	1	Ewa-RG-art_E	1	Ewa-RG-art_E
Ew-AU->in-RG	1		0		0	
Ew-AU->in-RG	1		0		0	
Ew-AU->in-RG	1	Ew-AU->in-RG	1	Ewa-RG-art_E	1	Ewa-RG-art_E
Ew-AU->in-RG	1	Ew-AU->in-RG	1	Ewa-RG-art_E	1	Ewa-RG-art_E
	0		0		0	
Ew-AU->in-RG	1	Ew-AU->in-RG	1	Ewa-RG-art_E	1	Ewa-RG-art_E
Ew-AU->in-RG	1	Ew-AU->in-RG	1	Ewa-RG-art_E	1	Ewa-RG-art_E
	0		0		0	
	0		0		0	
	0		0		0	
	0		0		0	
	0		0		0	
Ew-AU->in-RG	1	Ew-AU->in-RG	1	Ewa-RG-art_E	1	Ewa-RG-art_E
Ew-AU->in-RG	1	Ew-AU->in-RG	1	Ewa-RG-art_E	1	Ewa-RG-art_E
Ew-AU->in-RG	1	Ew-AU->in-RG	1	Ewa-RG-art_E	1	Ewa-RG-art_E
Ew-AU->in-RG	1	Ew-AU->in-RG	1	Ewa-RG-art_E	1	Ewa-RG-art_E
Ew-AU->in-RG	1		0	Ewa-RG-art_E	1	
Ew-AU->in-RG	1		0	Ewa-RG-art_E	1	
Ew-AU->in-RG	1		0	Ewa-RG-art_E	1	
	0		0		0	
	0		0		0	
	0		0		0	
	0		0		0	
	0		0		0	
	0		0		0	
	0		0		0	
Ew-AU->in-RG	1	Ew-AU->in-RG	1	Ewa-RG-art_E	1	Ewa-RG-art_E
Ew-AU->in-RG	1	Ew-AU->in-RG	1	Ewa-RG-art_E	1	Ewa-RG-art_E
Ew-AU->in-RG	1	Ew-AU->in-RG	1	Ewa-RG-art_E	1	Ewa-RG-art_E
Ew-AU->in-RG	1	Ew-AU->in-RG	1	Ewa-RG-art_E	1	Ewa-RG-art_E
Ew-AU->in-RG	1	Ew-AU->in-RG	1	Ewa-RG-art_E	1	Ewa-RG-art_E
Ew-AU->in-RG	1	Ew-AU->in-RG	1	Ewa-RG-art_E	1	Ewa-RG-art_E
	0		0		0	
Ew-AU->in-RG	1	Ew-AU->in-RG	1	Ewa-RG-art_E	1	Ewa-RG-art_E
	0		0		0	
	0		0		0	
	0		0		0	

Ew-AU->in-RG	1	Ew-AU->in-RG	1	Ewa-RG-art_E	1	Ewa-RG-art_E
Ew-AU->in-RG	1	Ew-AU->in-RG	1	Ewa-RG-art_E	1	Ewa-RG-art_E
	0		0		0	
Ew-AU->in-RG	1	Ew-AU->in-RG	1	Ewa-RG-art_E	1	Ewa-RG-art_E
Ew-AU->in-RG	1	Ew-AU->in-RG	1	Ewa-RG-art_E	1	Ewa-RG-art_E
	0		0		0	
Ew-AU->in-RG	1	Ew-AU->in-RG	1	Ewa-RG-art_E	1	Ewa-RG-art_E
Ew-AU->in-RG	1	Ew-AU->in-RG	1	Ewa-RG-art_E	1	Ewa-RG-art_E
Ew-AU->in-RG	1	Ew-AU->in-RG	1	Ewa-RG-art_E	1	Ewa-RG-art_E
Ew-AU->in-RG	1	Ew-AU->in-RG	1	Ewa-RG-art_E	1	Ewa-RG-art_E
	0		0		0	
	0		0		0	
	0		0		0	
	0		0		0	
	0		0		0	
	0		0		0	
Ew-AU->in-RG	1	Ew-AU->in-RG	1	Ewa-RG-art_E	1	Ewa-RG-art_E
Ew-AU->in-RG	1	Ew-AU->in-RG	1	Ewa-RG-art_E	1	Ewa-RG-art_E
Ew-AU->in-RG	1	Ew-AU->in-RG	1	Ewa-RG-art_E	1	Ewa-RG-art_E
	0		0		0	
	0		0		0	
Ew-AU->in-RG	1	Ew-AU->in-RG	1	Ewa-RG-art_E	1	Ewa-RG-art_E
Ew-AU->in-RG	1	Ew-AU->in-RG	1	Ewa-RG-art_E	1	Ewa-RG-art_E
Ew-AU->in-RG	1	Ew-AU->in-RG	1	Ewa-RG-art_E	1	Ewa-RG-art_E
	0		0		0	
	0		0		0	
Ew-AU->in-RG	1	Ew-AU->in-RG	1	Ewa-RG-art_E	1	Ewa-RG-art_E
Ew-AU->in-RG	1	Ew-AU->in-RG	1	Ewa-RG-art_E	1	Ewa-RG-art_E
	0		0		0	
	0		0		0	
Ew-AU->in-RG	1	Ew-AU->in-RG	1	Ewa-RG-art_E	1	Ewa-RG-art_E
Ew-AU->in-RG	1	Ew-AU->in-RG	1	Ewa-RG-art_E	1	Ewa-RG-art_E
Ew-AU->in-RG	1	Ew-AU->in-RG	1	Ewa-RG-art_E	1	Ewa-RG-art_E
	0		0		0	
	0		0		0	
	0		0		0	
	0		0		0	
	0		0		0	
Ew-AU->in-RG	1	Ew-AU->in-RG	1	Ewa-RG-art_E	1	Ewa-RG-art_E
Ew-AU->in-RG	1	Ew-AU->in-RG	1	Ewa-RG-art_E	1	Ewa-RG-art_E
	0		0		0	
	0		0		0	
	0		0		0	
	0		0		0	
	0		0		0	
Ew-AU->in-RG	1	Ew-AU->in-RG	1		0	
	0		0		0	

Ew-AU->in-RG	1	Ew-AU->in-RG	1	Ewa-RG-art_E	1	Ewa-RG-art_E
Ew-AU->in-RG	1	Ew-AU->in-RG	1	Ewa-RG-art_E	1	Ewa-RG-art_E
	0		0		0	
	0		0		0	
Ew-AU->in-RG	1	Ew-AU->in-RG	1	Ewa-RG-art_E	1	Ewa-RG-art_E
Ew-AU->in-RG	1	Ew-AU->in-RG	1	Ewa-RG-art_E	1	Ewa-RG-art_E
Ew-AU->in-RG	1	Ew-AU->in-RG	1	Ewa-RG-art_E	1	Ewa-RG-art_E
	0		0		0	
	0		0		0	
Ew-AU->in-RG	1		0		0	
Ew-AU->in-RG	1		0		0	
	0		0		0	
	0		0		0	
	0		0		0	
	0		0		0	
	0		0		0	
	0		0		0	
	0		0		0	
	0		0		0	
	0		0		0	
	0		0		0	
	0		0		0	
	0		0		0	
Ew-AU->in-RG	1		0	Ewa-RG-art_E	1	
Ew-AU->in-RG	1		0	Ewa-RG-art_E	1	
Ew-AU->in-RG	1	Ew-AU->in-RG	1	Ewa-RG-art_E	1	Ewa-RG-art_E
Ew-AU->in-RG	1	Ew-AU->in-RG	1	Ewa-RG-art_E	1	Ewa-RG-art_E
Ew-AU->in-RG	1	Ew-AU->in-RG	1	Ewa-RG-art_E	1	Ewa-RG-art_E
Ew-AU->in-RG	1	Ew-AU->in-RG	1	Ewa-RG-art_E	1	Ewa-RG-art_E
Ew-AU->in-RG	1	Ew-AU->in-RG	1	Ewa-RG-art_E	1	Ewa-RG-art_E
	0		0		0	
	0		0		0	
	0		0		0	
	0		0		0	
	0		0		0	
	0		0		0	
	0		0		0	
	0		0		0	
	0		0		0	
	0		0		0	
	0		0		0	
	0		0		0	
	0		0		0	
	0		0		0	
	0		0		0	
	0		0		0	
	0		0		0	

	0		0		0	
	0		0		0	
	0		0		0	
	0		0		0	
	0		0		0	
Ew-AU->in-RG	1	Ew-AU->in-RG	1	Ewa-RG-art_E	1	Ewa-RG-art_E
Ew-AU->in-RG	1	Ew-AU->in-RG	1	Ewa-RG-art_E	1	Ewa-RG-art_E
	0		0		0	
	0		0		0	
	0		0		0	
	0		0		0	
	0		0		0	
	0		0		0	
Ew-AU->in-RG	1		0	Ewa-RG-art_E	1	
Ew-AU->in-RG	1	Ew-AU->in-RG	1	Ewa-RG-art_E	1	Ewa-RG-art_E
Ew-AU->in-RG	1	Ew-AU->in-RG	1	Ewa-RG-art_E	1	Ewa-RG-art_E
	0		0		0	
Ew-AU->in-RG	1	Ew-AU->in-RG	1	Ewa-RG-art_E	1	Ewa-RG-art_E
	0		0		0	
Ew-AU->in-RG	1		0		0	
	0		0		0	
	0		0		0	
	0		0		0	
	0		0		0	
Ew-AU->in-RG	1	Ew-AU->in-RG	1	Ewa-RG-art_E	1	Ewa-RG-art_E
Ew-AU->in-RG	1	Ew-AU->in-RG	1	Ewa-RG-art_E	1	Ewa-RG-art_E
	0		0		0	
	0		0		0	
	0		0		0	
	0		0		0	
	0		0		0	
	0		0		0	
	0		0		0	
	0		0		0	
	0		0		0	
	0		0		0	
	0		0		0	
	0		0		0	
	0		0		0	
	0		0		0	
Ew-AU->in-RG	1		0	Ewa-RG-art_E	1	
	0		0		0	
	0		0		0	
	0		0		0	
	0		0		0	
Ew-AU->in-RG	1	Ew-AU->in-RG	1	Ewa-RG-art_E	1	Ewa-RG-art_E
Ew-AU->in-RG	1	Ew-AU->in-RG	1	Ewa-RG-art_E	1	Ewa-RG-art_E

	0		0		0	
	0		0		0	
	0		0		0	
	0		0		0	
	0		0		0	
	0		0		0	
	0		0		0	
Ew-AU->in-RG	1	Ew-AU->in-RG	1	Ewa-RG-art_E	1	Ewa-RG-art_E
Ew-AU->in-RG	1	Ew-AU->in-RG	1	Ewa-RG-art_E	1	Ewa-RG-art_E
	0		0		0	
	0		0		0	
Ew-AU->in-RG	1	Ew-AU->in-RG	1	Ewa-RG-art_E	1	Ewa-RG-art_E
Ew-AU->in-RG	1	Ew-AU->in-RG	1	Ewa-RG-art_E	1	Ewa-RG-art_E
Ew-AU->in-RG	1		0		0	
	0		0		0	
	0		0		0	
	0		0		0	
	0		0		0	
	0		0		0	
	0		0		0	
	0		0		0	
Ew-AU->in-RG	1	Ew-AU->in-RG	1	Ewa-RG-art_E	1	Ewa-RG-art_E
	0		0		0	
	0		0		0	
	0		0		0	
	0		0		0	
	0		0		0	
	0		0		0	
Ew-AU->in-RG	1	Ew-AU->in-RG	1	Ewa-RG-art_E	1	Ewa-RG-art_E
Ew-AU->in-RG	1	Ew-AU->in-RG	1	Ewa-RG-art_E	1	Ewa-RG-art_E
	0		0		0	
Ew-AU->in-RG	1	Ew-AU->in-RG	1	Ewa-RG-art_E	1	Ewa-RG-art_E
Ew-AU->in-RG	1	Ew-AU->in-RG	1	Ewa-RG-art_E	1	Ewa-RG-art_E
	0		0		0	
Ew-AU->in-RG	1	Ew-AU->in-RG	1	Ewa-RG-art_E	1	Ewa-RG-art_E
	0		0		0	
	0		0		0	
	0		0		0	
	0		0		0	
	0		0		0	
	0		0		0	
	0		0		0	
	0		0		0	
	0		0		0	
	0		0		0	
	0		0		0	
	0		0		0	

	0		0		0	
Ew-AU->in-RG	1		0		0	
Ew-AU->in-RG	1		0		0	
	0		0		0	
	0		0		0	
	0		0		0	
	0		0		0	
	0		0		0	
Ew-AU->in-RG	1		0	Ewa-RG-art_E	1	
	0		0		0	
	0		0		0	
	0		0		0	
	0		0		0	
	0		0		0	
	0		0		0	
	0		0		0	
	0		0		0	
	0		0		0	
	0		0		0	
	0		0		0	
	0		0		0	
	0		0		0	
	0		0		0	
Ew-AU->in-RG	1		0	Ewa-RG-art_E	1	
	0		0		0	
	0		0		0	
	0		0		0	
	0		0		0	
	0		0		0	
	0		0		0	
	0		0		0	
	0		0		0	
	0		0		0	
	0		0		0	
	0		0		0	
	0		0		0	
	0		0		0	
	0		0		0	
	0		0		0	
	0		0		0	
	0		0		0	
	0		0		0	
	0		0		0	
	0		0		0	
Ew-AU->in-RG	1	Ew-AU->in-RG	1	Ewa-RG-art_E	1	Ewa-RG-art_E
Ew-AU->in-RG	1	Ew-AU->in-RG	1	Ewa-RG-art_E	1	Ewa-RG-art_E
	0		0		0	

	0		0		0	
	0		0		0	
	0		0		0	
	0		0		0	
	0		0		0	
	0		0		0	
	0		0		0	
	0		0		0	
	0		0		0	
	0		0		0	
Ew-AU->in-RG	1	Ew-AU->in-RG	1	Ewa-RG-art_E	1	Ewa-RG-art_E
Ew-AU->in-RG	1	Ew-AU->in-RG	1	Ewa-RG-art_E	1	Ewa-RG-art_E
Ew-AU->in-RG	1	Ew-AU->in-RG	1	Ewa-RG-art_E	1	Ewa-RG-art_E
	0		0		0	
	0		0		0	
	0		0		0	
	0		0		0	
	0		0		0	
	0		0		0	
	0		0		0	
Ew-AU->in-RG	1	Ew-AU->in-RG	1	Ewa-RG-art_E	1	Ewa-RG-art_E
Ew-AU->in-RG	1	Ew-AU->in-RG	1	Ewa-RG-art_E	1	Ewa-RG-art_E
	0		0		0	
	0		0		0	
	0		0		0	
	0		0		0	
	0		0		0	
	0		0		0	
	0		0		0	
	0		0		0	
	0		0		0	
	0		0		0	
	0		0		0	
	0		0		0	
Ew-AU->in-RG	1	Ew-AU->in-RG	1	Ewa-RG-art_E	1	Ewa-RG-art_E
	0		0		0	
	0		0		0	
	0		0		0	
	0		0		0	
	0		0		0	
Ew-AU->in-RG	1		0	Ewa-RG-art_E	1	
	0		0		0	
	0		0		0	
Ew-AU->in-RG	1	Ew-AU->in-RG	1	Ewa-RG-art_E	1	Ewa-RG-art_E
	0		0		0	
Ew-AU->in-RG	1		0	Ewa-RG-art_E	1	
Ew-AU->in-RG	1		0	Ewa-RG-art_E	1	

	0		0		0	
	0		0		0	
	0		0		0	
	0		0		0	
	0		0		0	
	0		0		0	
	0		0		0	
Ew-AU->in-RG	1	Ew-AU->in-RG	1	Ewa-RG-art_E	1	Ewa-RG-art_E
Ew-AU->in-RG	1	Ew-AU->in-RG	1	Ewa-RG-art_E	1	Ewa-RG-art_E
	0		0		0	
	0		0		0	
	0		0		0	
	0		0		0	
	0		0		0	
	0		0		0	
	0		0		0	
	0		0		0	
	0		0		0	
	0		0		0	
	0		0		0	
Ew-AU->in-RG	1	Ew-AU->in-RG	1	Ewa-RG-art_E	1	Ewa-RG-art_E
Ew-AU->in-RG	1	Ew-AU->in-RG	1	Ewa-RG-art_E	1	Ewa-RG-art_E
Ew-AU->in-RG	1	Ew-AU->in-RG	1	Ewa-RG-art_E	1	Ewa-RG-art_E
Ew-AU->in-RG	1	Ew-AU->in-RG	1	Ewa-RG-art_E	1	Ewa-RG-art_E
	0		0		0	
	0		0		0	
Ew-AU->in-RG	1		0	Ewa-RG-art_E	1	
	0		0		0	
	0		0		0	
	0		0		0	
Ew-AU->in-RG	1	Ew-AU->in-RG	1	Ewa-RG-art_E	1	Ewa-RG-art_E
Ew-AU->in-RG	1	Ew-AU->in-RG	1	Ewa-RG-art_E	1	Ewa-RG-art_E
Ew-AU->in-RG	1	Ew-AU->in-RG	1	Ewa-RG-art_E	1	Ewa-RG-art_E
Ew-AU->in-RG	1	Ew-AU->in-RG	1	Ewa-RG-art_E	1	Ewa-RG-art_E
	0		0		0	
	0		0		0	
	0		0		0	
Ew-AU->in-RG	1	Ew-AU->in-RG	1	Ewa-RG-art_E	1	Ewa-RG-art_E
Ew-AU->in-RG	1	Ew-AU->in-RG	1	Ewa-RG-art_E	1	Ewa-RG-art_E
	0		0		0	
	0		0		0	
	0		0		0	
Ew-AU->in-RG	1		0		0	
	0		0		0	
Ew-AU->in-RG	1	Ew-AU->in-RG	1	Ewa-RG-art_E	1	Ewa-RG-art_E
Ew-AU->in-RG	1	Ew-AU->in-RG	1	Ewa-RG-art_E	1	Ewa-RG-art_E
	0		0		0	

Ew-AU->in-RG	1	Ew-AU->in-RG	1	Ewa-RG-art_E	1	Ewa-RG-art_E
Ew-AU->in-RG	1	Ew-AU->in-RG	1	Ewa-RG-art_E	1	Ewa-RG-art_E
	0		0		0	
	0		0		0	
Ew-AU->in-RG	1	Ew-AU->in-RG	1	Ewa-RG-art_E	1	Ewa-RG-art_E
Ew-AU->in-RG	1	Ew-AU->in-RG	1	Ewa-RG-art_E	1	Ewa-RG-art_E
Ew-AU->in-RG	1	Ew-AU->in-RG	1	Ewa-RG-art_E	1	Ewa-RG-art_E
	0		0		0	
	0		0		0	
	0		0		0	
	0		0		0	
	0		0		0	
Ew-AU->in-RG	1		0	Ewa-RG-art_E	1	
	0		0		0	
	0		0		0	
Ew-AU->in-RG	1	Ew-AU->in-RG	1	Ewa-RG-art_E	1	Ewa-RG-art_E
Ew-AU->in-RG	1	Ew-AU->in-RG	1	Ewa-RG-art_E	1	Ewa-RG-art_E
	0		0		0	
	0		0		0	
	0		0		0	
	0		0		0	
	0		0		0	
	0		0		0	
	0		0		0	
	0		0		0	
Ew-AU->in-RG	1		0	Ewa-RG-art_E	1	
	0		0		0	
	0		0		0	
	0		0		0	
Ew-AU->in-RG	1	Ew-AU->in-RG	1	Ewa-RG-art_E	1	Ewa-RG-art_E
	0		0		0	
	0		0		0	
	0		0		0	
	0		0		0	
	0		0		0	
	0		0		0	
	0		0		0	
Ew-AU->in-RG	1	Ew-AU->in-RG	1	Ewa-RG-art_E	1	Ewa-RG-art_E
Ew-AU->in-RG	1	Ew-AU->in-RG	1	Ewa-RG-art_E	1	Ewa-RG-art_E
	0		0		0	
Ew-AU->in-RG	1		0		0	
	0		0		0	
	0		0		0	
	0		0		0	
	0		0		0	
	0		0		0	
	0		0		0	

	0		0		0	
	0		0		0	
Ew-AU->in-RG	1	Ew-AU->in-RG	1	Ewa-RG-art_E	1	Ewa-RG-art_E
Ew-AU->in-RG	1	Ew-AU->in-RG	1	Ewa-RG-art_E	1	Ewa-RG-art_E
	0		0		0	
	0		0		0	
Ew-AU->in-RG	1	Ew-AU->in-RG	1	Ewa-RG-art_E	1	Ewa-RG-art_E
Ew-AU->in-RG	1	Ew-AU->in-RG	1	Ewa-RG-art_E	1	Ewa-RG-art_E
Ew-AU->in-RG	1	Ew-AU->in-RG	1	Ewa-RG-art_E	1	Ewa-RG-art_E
Ew-AU->in-RG	1	Ew-AU->in-RG	1	Ewa-RG-art_E	1	Ewa-RG-art_E
	0		0		0	
	0		0		0	
	0		0		0	
	0		0		0	
	0		0		0	
	0		0		0	
	0		0		0	
	0		0		0	
	0		0		0	
	0		0		0	
	0		0		0	
Ew-AU->in-RG	1	Ew-AU->in-RG	1	Ewa-RG-art_E	1	Ewa-RG-art_E
Ew-AU->in-RG	1	Ew-AU->in-RG	1	Ewa-RG-art_E	1	Ewa-RG-art_E
Ew-AU->in-RG	1	Ew-AU->in-RG	1	Ewa-RG-art_E	1	Ewa-RG-art_E
Ew-AU->in-RG	1	Ew-AU->in-RG	1	Ewa-RG-art_E	1	Ewa-RG-art_E
Ew-AU->in-RG	1	Ew-AU->in-RG	1	Ewa-RG-art_E	1	Ewa-RG-art_E
	0		0		0	
	0		0		0	
	0		0		0	
	0		0		0	
	0		0		0	
Ew-AU->in-RG	1	Ew-AU->in-RG	1	Ewa-RG-art_E	1	Ewa-RG-art_E
Ew-AU->in-RG	1	Ew-AU->in-RG	1	Ewa-RG-art_E	1	Ewa-RG-art_E
	0		0		0	
	0		0		0	
	0		0		0	
	0		0		0	
	0		0		0	
Ew-AU->in-RG	1		0	Ewa-RG-art_E	1	
Ew-AU->in-RG	1		0	Ewa-RG-art_E	1	
Ew-AU->in-RG	1		0	Ewa-RG-art_E	1	
	0		0		0	
Ew-AU->in-RG	1	Ew-AU->in-RG	1	Ewa-RG-art_E	1	Ewa-RG-art_E
	0		0		0	
	0		0		0	
	0		0		0	
	0		0		0	

	0		0		0	
	0		0		0	
	0		0		0	
	0		0		0	
	0		0		0	
	0		0		0	
	0		0		0	
Ew-AU->in-RG	1	Ew-AU->in-RG	1	Ewa-RG-art_E	1	Ewa-RG-art_E
	0		0		0	
	0		0		0	
	0		0		0	
	0		0		0	
	0		0		0	
	0		0		0	
Ew-AU->in-RG	1	Ew-AU->in-RG	1	Ewa-RG-art_E	1	Ewa-RG-art_E
	0		0		0	
	0		0		0	
	0		0		0	
	0		0		0	
Ew-AU->in-RG	1		0	Ewa-RG-art_E	1	
	0		0		0	
	0		0		0	
	0		0		0	
	0		0		0	
	0		0		0	
	0		0		0	
	0		0		0	
Ew-AU->in-RG	1	Ew-AU->in-RG	1	Ewa-RG-art_E	1	Ewa-RG-art_E
	0		0		0	
	0		0		0	
	0		0		0	
	0		0		0	
	0		0		0	
Ew-AU->in-RG	1	Ew-AU->in-RG	1	Ewa-RG-art_E	1	Ewa-RG-art_E
Ew-AU->in-RG	1	Ew-AU->in-RG	1	Ewa-RG-art_E	1	Ewa-RG-art_E
Ew-AU->in-RG	1	Ew-AU->in-RG	1	Ewa-RG-art_E	1	Ewa-RG-art_E
Ew-AU->in-RG	1	Ew-AU->in-RG	1	Ewa-RG-art_E	1	Ewa-RG-art_E
	0		0		0	
	0		0		0	
	0		0		0	
	0		0		0	
	0		0		0	
	0		0		0	
	0		0		0	
	0		0		0	
	0		0		0	
	0		0		0	

	0		0		0	
	0		0		0	
	0		0		0	
	0		0		0	
Ew-AU->in-RG	1	Ew-AU->in-RG	1	Ewa-RG-art_E	1	Ewa-RG-art_E
	0		0		0	
	0		0		0	
	0		0		0	
Ew-AU->in-RG	1	Ew-AU->in-RG	1	Ewa-RG-art_E	1	Ewa-RG-art_E
	0		0		0	
Ew-AU->in-RG	1	Ew-AU->in-RG	1	Ewa-RG-art_E	1	Ewa-RG-art_E
Ew-AU->in-RG	1	Ew-AU->in-RG	1	Ewa-RG-art_E	1	Ewa-RG-art_E
Ew-AU->in-RG	1	Ew-AU->in-RG	1	Ewa-RG-art_E	1	Ewa-RG-art_E
	0		0		0	
	0		0		0	
Ew-AU->in-RG	1		0		0	
	0		0		0	
	0		0		0	
	0		0		0	
Ew-AU->in-RG	1	Ew-AU->in-RG	1	Ewa-RG-art_E	1	Ewa-RG-art_E
Ew-AU->in-RG	1	Ew-AU->in-RG	1	Ewa-RG-art_E	1	Ewa-RG-art_E
Ew-AU->in-RG	1		0	Ewa-RG-art_E	1	
Ew-AU->in-RG	1		0	Ewa-RG-art_E	1	
	0		0		0	
Ew-AU->in-RG	1	Ew-AU->in-RG	1	Ewa-RG-art_E	1	Ewa-RG-art_E
	0		0		0	
	0		0		0	
	0		0		0	
Ew-AU->in-RG	1	Ew-AU->in-RG	1	Ewa-RG-art_E	1	Ewa-RG-art_E
Ew-AU->in-RG	1	Ew-AU->in-RG	1	Ewa-RG-art_E	1	Ewa-RG-art_E
Ew-AU->in-RG	1	Ew-AU->in-RG	1	Ewa-RG-art_E	1	Ewa-RG-art_E
Ew-AU->in-RG	1		0		0	
Ew-AU->in-RG	1		0		0	
	0		0		0	
	0		0		0	
	0		0		0	
	0		0		0	
	0		0		0	
	0		0		0	
	0		0		0	
	0		0		0	
Ew-AU->in-RG	1	Ew-AU->in-RG	1	Ewa-RG-art_E	1	Ewa-RG-art_E
	0		0		0	
	0		0		0	
	0		0		0	
	0		0		0	
	0		0		0	

	0		0		0	
	0		0		0	
	0		0		0	
	0		0		0	
	0		0		0	
	0		0		0	
	0		0		0	
	0		0		0	
	0		0		0	
	0		0		0	
	0		0		0	
	0		0		0	
	0		0		0	
	0		0		0	
	0		0		0	
	0		0		0	
	0		0		0	
Ew-AU->in-RG	1	Ew-AU->in-RG	1	Ewa-RG-art_E	1	Ewa-RG-art_E
Ew-AU->in-RG	1	Ew-AU->in-RG	1	Ewa-RG-art_E	1	Ewa-RG-art_E
	0		0		0	
	0		0		0	
	0		0		0	
	0		0		0	
	0		0		0	
	0		0		0	
	0		0		0	
	0		0		0	
	0		0		0	
	0		0		0	
	0		0		0	
	0		0		0	
	0		0		0	
	0		0		0	
	0		0		0	
	0		0		0	
	0		0		0	
	0		0		0	
	0		0		0	
	0		0		0	
	0		0		0	
	0		0		0	
	0		0		0	
	0		0		0	
	0		0		0	
	0		0		0	
Ew-AU->in-RG	1		0		0	
	0		0		0	

	0		0		0	
	0		0		0	
	0		0		0	
Ew-AU->in-RG	1	Ew-AU->in-RG	1	Ewa-RG-art_E	1	Ewa-RG-art_E
	0		0		0	
	0		0		0	
Ew-AU->in-RG	1	Ew-AU->in-RG	1	Ewa-RG-art_E	1	Ewa-RG-art_E
Ew-AU->in-RG	1	Ew-AU->in-RG	1	Ewa-RG-art_E	1	Ewa-RG-art_E
	0		0		0	
	0		0		0	
	0		0		0	
	0		0		0	
	0		0		0	
	0		0		0	
Ew-AU->in-RG	1	Ew-AU->in-RG	1	Ewa-RG-art_E	1	Ewa-RG-art_E
	0		0		0	
	0		0		0	
	0		0		0	
	0		0		0	
Ew-AU->in-RG	1	Ew-AU->in-RG	1	Ewa-RG-art_E	1	Ewa-RG-art_E
Ew-AU->in-RG	1	Ew-AU->in-RG	1	Ewa-RG-art_E	1	Ewa-RG-art_E
Ew-AU->in-RG	1	Ew-AU->in-RG	1	Ewa-RG-art_E	1	Ewa-RG-art_E
Ew-AU->in-RG	1	Ew-AU->in-RG	1	Ewa-RG-art_E	1	Ewa-RG-art_E
Ew-AU->in-RG	1	Ew-AU->in-RG	1	Ewa-RG-art_E	1	Ewa-RG-art_E
	0		0		0	
Ew-AU->in-RG	1		0		0	
Ew-AU->in-RG	1	Ew-AU->in-RG	1	Ewa-RG-art_E	1	Ewa-RG-art_E
Ew-AU->in-RG	1	Ew-AU->in-RG	1	Ewa-RG-art_E	1	Ewa-RG-art_E
Ew-AU->in-RG	1	Ew-AU->in-RG	1	Ewa-RG-art_E	1	Ewa-RG-art_E
	0		0		0	
	0		0		0	
	0		0		0	
Ew-AU->in-RG	1	Ew-AU->in-RG	1	Ewa-RG-art_E	1	Ewa-RG-art_E
	0		0		0	
	0		0		0	
	0		0		0	
Ew-AU->in-RG	1	Ew-AU->in-RG	1	Ewa-RG-art_E	1	Ewa-RG-art_E
Ew-AU->in-RG	1	Ew-AU->in-RG	1	Ewa-RG-art_E	1	Ewa-RG-art_E
Ew-AU->in-RG	1	Ew-AU->in-RG	1	Ewa-RG-art_E	1	Ewa-RG-art_E
Ew-AU->in-RG	1		0	Ewa-RG-art_E	1	
Ew-AU->in-RG	1		0	Ewa-RG-art_E	1	
Ew-AU->in-RG	1	Ew-AU->in-RG	1	Ewa-RG-art_E	1	Ewa-RG-art_E
Ew-AU->in-RG	1	Ew-AU->in-RG	1	Ewa-RG-art_E	1	Ewa-RG-art_E
	0		0		0	
	0		0		0	
	0		0		0	
	0		0		0	

Ew-AU->in-RG	1	Ew-AU->in-RG	1	Ewa-RG-art_E	1	Ewa-RG-art_E
Ew-AU->in-RG	1		0	Ewa-RG-art_E	1	
	0		0		0	
	0		0		0	
	0		0		0	
	0		0		0	
	0		0		0	
	0		0		0	
	0		0		0	
	0		0		0	
	0		0		0	
Ew-AU->in-RG	1	Ew-AU->in-RG	1	Ewa-RG-art_E	1	Ewa-RG-art_E
Ew-AU->in-RG	1	Ew-AU->in-RG	1	Ewa-RG-art_E	1	Ewa-RG-art_E
Ew-AU->in-RG	1	Ew-AU->in-RG	1	Ewa-RG-art_E	1	Ewa-RG-art_E
	0		0		0	
	0		0		0	
	0		0		0	
	0		0		0	
	0		0		0	
	0		0		0	
Ew-AU->in-RG	1	Ew-AU->in-RG	1	Ewa-RG-art_E	1	Ewa-RG-art_E
Ew-AU->in-RG	1	Ew-AU->in-RG	1	Ewa-RG-art_E	1	Ewa-RG-art_E
Ew-AU->in-RG	1	Ew-AU->in-RG	1	Ewa-RG-art_E	1	Ewa-RG-art_E
Ew-AU->in-RG	1	Ew-AU->in-RG	1	Ewa-RG-art_E	1	Ewa-RG-art_E
	0		0		0	
	0		0		0	
	0		0		0	
	0		0		0	
	0		0		0	
	0		0		0	
	0		0		0	
	0		0		0	
Ew-AU->in-RG	1		0	Ewa-RG-art_E	1	
Ew-AU->in-RG	1		0	Ewa-RG-art_E	1	
Ew-AU->in-RG	1	Ew-AU->in-RG	1	Ewa-RG-art_E	1	Ewa-RG-art_E
	0		0		0	
	0		0		0	
	0		0		0	
	0		0		0	
Ew-AU->in-RG	1		0	Ewa-RG-art_E	1	
Ew-AU->in-RG	1		0	Ewa-RG-art_E	1	
	0		0		0	
	0		0		0	
	0		0		0	
	0		0		0	
	0		0		0	
	0		0		0	

	0		0		0	
	0		0		0	
	0		0		0	
	0		0		0	
	0		0		0	
Ew-AU->in-RG	1		0	Ewa-RG-art_E	1	
	0		0		0	
	0		0		0	
	0		0		0	
	0		0		0	
	0		0		0	
Ew-AU->in-RG	1	Ew-AU->in-RG	1	Ewa-RG-art_E	1	Ewa-RG-art_E
Ew-AU->in-RG	1	Ew-AU->in-RG	1	Ewa-RG-art_E	1	Ewa-RG-art_E
Ew-AU->in-RG	1	Ew-AU->in-RG	1	Ewa-RG-art_E	1	Ewa-RG-art_E
	0		0		0	
	0		0		0	
	0		0		0	
Ew-AU->in-RG	1		0	Ewa-RG-art_E	1	
	0		0		0	
	0		0		0	
	0		0		0	
	0		0		0	
Ew-AU->in-RG	1	Ew-AU->in-RG	1	Ewa-RG-art_E	1	Ewa-RG-art_E
Ew-AU->in-RG	1	Ew-AU->in-RG	1	Ewa-RG-art_E	1	Ewa-RG-art_E
Ew-AU->in-RG	1	Ew-AU->in-RG	1	Ewa-RG-art_E	1	Ewa-RG-art_E
Ew-AU->in-RG	1	Ew-AU->in-RG	1	Ewa-RG-art_E	1	Ewa-RG-art_E
Ew-AU->in-RG	1	Ew-AU->in-RG	1	Ewa-RG-art_E	1	Ewa-RG-art_E
	0		0		0	
	0		0		0	
	0		0		0	
	0		0		0	
	0		0		0	
	0		0		0	
	0		0		0	
	0		0		0	
Ew-AU->in-RG	1	Ew-AU->in-RG	1		0	
	0		0		0	
	0		0		0	
Ew-AU->in-RG	1	Ew-AU->in-RG	1	Ewa-RG-art_E	1	Ewa-RG-art_E
Ew-AU->in-RG	1	Ew-AU->in-RG	1	Ewa-RG-art_E	1	Ewa-RG-art_E
	0		0		0	
	0		0		0	
Ew-AU->in-RG	1		0		0	
	0		0		0	
	0		0		0	
	0		0		0	
	0		0		0	

	0		0		0	
	0		0		0	
	0		0		0	
Ew-AU->in-RG	1	Ew-AU->in-RG	1	Ewa-RG-art_E	1	Ewa-RG-art_E
Ew-AU->in-RG	1	Ew-AU->in-RG	1	Ewa-RG-art_E	1	Ewa-RG-art_E
	0		0		0	
	0		0		0	
	0		0		0	
Ew-AU->in-RG	1	Ew-AU->in-RG	1	Ewa-RG-art_E	1	Ewa-RG-art_E
Ew-AU->in-RG	1	Ew-AU->in-RG	1	Ewa-RG-art_E	1	Ewa-RG-art_E
Ew-AU->in-RG	1	Ew-AU->in-RG	1	Ewa-RG-art_E	1	Ewa-RG-art_E
Ew-AU->in-RG	1	Ew-AU->in-RG	1	Ewa-RG-art_E	1	Ewa-RG-art_E
Ew-AU->in-RG	1	Ew-AU->in-RG	1	Ewa-RG-art_E	1	Ewa-RG-art_E
Ew-AU->in-RG	1	Ew-AU->in-RG	1	Ewa-RG-art_E	1	Ewa-RG-art_E
Ew-AU->in-RG	1	Ew-AU->in-RG	1	Ewa-RG-art_E	1	Ewa-RG-art_E
	0		0		0	
	0		0		0	
	0		0		0	
	0		0		0	
	0		0		0	
	0		0		0	
Ew-AU->in-RG	1	Ew-AU->in-RG	1	Ewa-RG-art_E	1	Ewa-RG-art_E
	0		0		0	
	0		0		0	
	0		0		0	
Ew-AU->in-RG	1	Ew-AU->in-RG	1	Ewa-RG-art_E	1	Ewa-RG-art_E
Ew-AU->in-RG	1	Ew-AU->in-RG	1	Ewa-RG-art_E	1	Ewa-RG-art_E
Ew-AU->in-RG	1	Ew-AU->in-RG	1	Ewa-RG-art_E	1	Ewa-RG-art_E
Ew-AU->in-RG	1	Ew-AU->in-RG	1	Ewa-RG-art_E	1	Ewa-RG-art_E
	0		0		0	
	0		0		0	
	0		0		0	
	0		0		0	
	0		0		0	
	0		0		0	
	0		0		0	
	0		0		0	
	0		0		0	
	0		0		0	
	0		0		0	
	0		0		0	
	0		0		0	
Ew-AU->in-RG	1	Ew-AU->in-RG	1	Ewa-RG-art_E	1	Ewa-RG-art_E
Ew-AU->in-RG	1	Ew-AU->in-RG	1	Ewa-RG-art_E	1	Ewa-RG-art_E
Ew-AU->in-RG	1	Ew-AU->in-RG	1	Ewa-RG-art_E	1	Ewa-RG-art_E
	0		0		0	

	0		0		0	
	0		0		0	
	0		0		0	
	0		0		0	
	0		0		0	
	0		0		0	
	0		0		0	
Ew-AU->in-RG	1	Ew-AU->in-RG	1	Ewa-RG-art_E	1	Ewa-RG-art_E
Ew-AU->in-RG	1	Ew-AU->in-RG	1	Ewa-RG-art_E	1	Ewa-RG-art_E
	0		0		0	
	0		0		0	
Ew-AU->in-RG	1	Ew-AU->in-RG	1	Ewa-RG-art_E	1	Ewa-RG-art_E
Ew-AU->in-RG	1	Ew-AU->in-RG	1	Ewa-RG-art_E	1	Ewa-RG-art_E
Ew-AU->in-RG	1	Ew-AU->in-RG	1	Ewa-RG-art_E	1	Ewa-RG-art_E
	0		0		0	
	0		0		0	
	0		0		0	
	0		0		0	
	0		0		0	
Ew-AU->in-RG	1	Ew-AU->in-RG	1	Ewa-RG-art_E	1	Ewa-RG-art_E
Ew-AU->in-RG	1	Ew-AU->in-RG	1	Ewa-RG-art_E	1	Ewa-RG-art_E
Ew-AU->in-RG	1	Ew-AU->in-RG	1	Ewa-RG-art_E	1	Ewa-RG-art_E
	0		0		0	
	0		0		0	
	0		0		0	
	0		0		0	
	0		0		0	
	0		0		0	
	0		0		0	
Ew-AU->in-RG	1	Ew-AU->in-RG	1	Ewa-RG-art_E	1	Ewa-RG-art_E
	0		0		0	
	0		0		0	
	0		0		0	
	0		0		0	
	0		0		0	
	0		0		0	
	0		0		0	
	0		0		0	
	0		0		0	
	0		0		0	
	0		0		0	
	0		0		0	
	0		0		0	
	0		0		0	
	0		0		0	
Ew-AU->in-RG	1		0	Ewa-RG-art_E	1	
Ew-AU->in-RG	1		0	Ewa-RG-art_E	1	

	0		0		0	
	0		0		0	
	0		0		0	
	0		0		0	
	0		0		0	
	0		0		0	
	0		0		0	
Ew-AU->in-RG	1	Ew-AU->in-RG	1	Ewa-RG-art_E	1	Ewa-RG-art_E
Ew-AU->in-RG	1	Ew-AU->in-RG	1	Ewa-RG-art_E	1	Ewa-RG-art_E
	0		0		0	
	0		0		0	
	0		0		0	
Ew-AU->in-RG	1	Ew-AU->in-RG	1	Ewa-RG-art_E	1	Ewa-RG-art_E
Ew-AU->in-RG	1	Ew-AU->in-RG	1	Ewa-RG-art_E	1	Ewa-RG-art_E
Ew-AU->in-RG	1	Ew-AU->in-RG	1	Ewa-RG-art_E	1	Ewa-RG-art_E
	0		0		0	
	0		0		0	
	0		0		0	
Ew-AU->in-RG	1	Ew-AU->in-RG	1	Ewa-RG-art_E	1	Ewa-RG-art_E
Ew-AU->in-RG	1	Ew-AU->in-RG	1	Ewa-RG-art_E	1	Ewa-RG-art_E
Ew-AU->in-RG	1	Ew-AU->in-RG	1	Ewa-RG-art_E	1	Ewa-RG-art_E
	0		0		0	
	0		0		0	
	0		0		0	
	0		0		0	
Ew-AU->in-RG	1	Ew-AU->in-RG	1	Ewa-RG-art_E	1	Ewa-RG-art_E
	0		0		0	
	0		0		0	
	0		0		0	
	0		0		0	
Ew-AU->in-RG	1	Ew-AU->in-RG	1	Ewa-RG-art_E	1	Ewa-RG-art_E
	0		0		0	
	0		0		0	
	0		0		0	
Ew-AU->in-RG	1	Ew-AU->in-RG	1	Ewa-RG-art_E	1	Ewa-RG-art_E
Ew-AU->in-RG	1	Ew-AU->in-RG	1	Ewa-RG-art_E	1	Ewa-RG-art_E
	0		0		0	
	0		0		0	
	0		0		0	
Ew-AU->in-RG	1		0		0	
	0		0		0	
	0		0		0	
	0		0		0	
	0		0		0	
	0		0		0	
	0		0		0	
Ew-AU->in-RG	1	Ew-AU->in-RG	1	Ewa-RG-art_E	1	Ewa-RG-art_E

	0		0		0	
	0		0		0	
	0		0		0	
	0		0		0	
Ew-AU->in-RG	1		0		0	
	0		0		0	
	0		0		0	
Ew-AU->in-RG	1	Ew-AU->in-RG	1	Ewa-RG-art_E	1	Ewa-RG-art_E
Ew-AU->in-RG	1	Ew-AU->in-RG	1	Ewa-RG-art_E	1	Ewa-RG-art_E
	0		0		0	
	0		0		0	
	0		0		0	
	0		0		0	
	0		0		0	
	0		0		0	
	0		0		0	
	0		0		0	
	0		0		0	
	0		0		0	
	0		0		0	
	0		0		0	
Ew-AU->in-RG	1	Ew-AU->in-RG	1	Ewa-RG-art_E	1	Ewa-RG-art_E
Ew-AU->in-RG	1	Ew-AU->in-RG	1	Ewa-RG-art_E	1	Ewa-RG-art_E
Ew-AU->in-RG	1	Ew-AU->in-RG	1	Ewa-RG-art_E	1	Ewa-RG-art_E
Ew-AU->in-RG	1	Ew-AU->in-RG	1	Ewa-RG-art_E	1	Ewa-RG-art_E
	0		0		0	
Ew-AU->in-RG	1	Ew-AU->in-RG	1	Ewa-RG-art_E	1	Ewa-RG-art_E
	0		0		0	
	0		0		0	
	0		0		0	
	0		0		0	
	0		0		0	
Ew-AU->in-RG	1		0		0	
Ew-AU->in-RG	1		0		0	
Ew-AU->in-RG	1	Ew-AU->in-RG	1	Ewa-RG-art_E	1	Ewa-RG-art_E
Ew-AU->in-RG	1	Ew-AU->in-RG	1	Ewa-RG-art_E	1	Ewa-RG-art_E
	0		0		0	
	0		0		0	
	0		0		0	
Ew-AU->in-RG	1	Ew-AU->in-RG	1	Ewa-RG-art_E	1	Ewa-RG-art_E
Ew-AU->in-RG	1	Ew-AU->in-RG	1	Ewa-RG-art_E	1	Ewa-RG-art_E
Ew-AU->in-RG	1		0		0	
Ew-AU->in-RG	1	Ew-AU->in-RG	1	Ewa-RG-art_E	1	Ewa-RG-art_E
	0		0		0	
	0		0		0	
	0		0		0	
	0		0		0	

Ew-AU->in-RG	1	Ew-AU->in-RG	1	Ewa-RG-art_E	1	Ewa-RG-art_E
Ew-AU->in-RG	1	Ew-AU->in-RG	1	Ewa-RG-art_E	1	Ewa-RG-art_E
Ew-AU->in-RG	1	Ew-AU->in-RG	1	Ewa-RG-art_E	1	Ewa-RG-art_E
	0		0		0	
	0		0		0	
	0		0		0	
	0		0		0	
	0		0		0	
	0		0		0	
	0		0		0	
	0		0		0	
	0		0		0	
	0		0		0	
	0		0		0	
	0		0		0	
	0		0		0	
	0		0		0	
	0		0		0	
	0		0		0	
	0		0		0	
	0		0		0	
	0		0		0	
	0		0		0	
	0		0		0	
	0		0		0	
Ew-AU->in-RG	1	Ew-AU->in-RG	1	Ewa-RG-art_E	1	Ewa-RG-art_E
	0		0		0	
	0		0		0	
	0		0		0	
	0		0		0	
	0		0		0	
Ew-AU->in-RG	1	Ew-AU->in-RG	1	Ewa-RG-art_E	1	Ewa-RG-art_E
	0		0		0	
	0		0		0	
	0		0		0	
	0		0		0	
	0		0		0	
	0		0		0	
	0		0		0	
	0		0		0	
	0		0		0	
	0		0		0	
	0		0		0	
	0		0		0	
	0		0		0	
	0		0		0	
	0		0		0	

	0		0		0	
	0		0		0	
	0		0		0	
	0		0		0	
	0		0		0	
	0		0		0	
	0		0		0	
	0		0		0	
	0		0		0	
	0		0		0	
	0		0		0	
	0		0		0	
	0		0		0	
	0		0		0	
	0		0		0	
	0		0		0	
Ew-AU->in-RG	1	Ew-AU->in-RG	1	Ewa-RG-art_E	1	Ewa-RG-art_E
Ew-AU->in-RG	1	Ew-AU->in-RG	1	Ewa-RG-art_E	1	Ewa-RG-art_E
	0		0		0	
	0		0		0	
	0		0		0	
	0		0		0	
	0		0		0	
	0		0		0	
	0		0		0	
	0		0		0	
	0		0		0	
	0		0		0	
	0		0		0	
	0		0		0	
	0		0		0	
	0		0		0	
Ew-AU->in-RG	1		0		0	
	0		0		0	
	0		0		0	
	0		0		0	
	0		0		0	
	0		0		0	
	0		0		0	
	0		0		0	
	0		0		0	
	0		0		0	
	0		0		0	
	0		0		0	
	0		0		0	
	0		0		0	
	0		0		0	

Ew-AU->in-RG	1	Ew-AU->in-RG	1	Ewa-RG-art_E	1	Ewa-RG-art_E
	0		0		0	
	0		0		0	
	0		0		0	
	0		0		0	
	0		0		0	
	0		0		0	
Ew-AU->in-RG	1	Ew-AU->in-RG	1	Ewa-RG-art_E	1	Ewa-RG-art_E
Ew-AU->in-RG	1	Ew-AU->in-RG	1	Ewa-RG-art_E	1	Ewa-RG-art_E
	0		0		0	
Ew-AU->in-RG	1	Ew-AU->in-RG	1	Ewa-RG-art_E	1	Ewa-RG-art_E
Ew-AU->in-RG	1	Ew-AU->in-RG	1	Ewa-RG-art_E	1	Ewa-RG-art_E
Ew-AU->in-RG	1		0		0	
	0		0		0	
	0		0		0	
Ew-AU->in-RG	1	Ew-AU->in-RG	1	Ewa-RG-art_E	1	Ewa-RG-art_E
Ew-AU->in-RG	1	Ew-AU->in-RG	1	Ewa-RG-art_E	1	Ewa-RG-art_E
Ew-AU->in-RG	1	Ew-AU->in-RG	1	Ewa-RG-art_E	1	Ewa-RG-art_E
Ew-AU->in-RG	1		0	Ewa-RG-art_E	1	
Ew-AU->in-RG	1		0	Ewa-RG-art_E	1	
	0		0		0	
	0		0		0	
	0		0		0	
	0		0		0	
	0		0		0	
Ew-AU->in-RG	1		0		0	
	0		0		0	
	0		0		0	
	0		0		0	
	0		0		0	
	0		0		0	
	0		0		0	
	0		0		0	
Ew-AU->in-RG	1	Ew-AU->in-RG	1	Ewa-RG-art_E	1	Ewa-RG-art_E
Ew-AU->in-RG	1	Ew-AU->in-RG	1	Ewa-RG-art_E	1	Ewa-RG-art_E
Ew-AU->in-RG	1	Ew-AU->in-RG	1	Ewa-RG-art_E	1	Ewa-RG-art_E
	0		0		0	
Ew-AU->in-RG	1		0		0	
Ew-AU->in-RG	1		0		0	
	0		0		0	
	0		0		0	
	0		0		0	
	0		0		0	
	0		0		0	
	0		0		0	
	0		0		0	
	0		0		0	

Ew-AU->in-RG	1		0		0	
	0		0		0	
Ew-AU->in-RG	1	Ew-AU->in-RG	1	Ewa-RG-art_E	1	Ewa-RG-art_E
	0		0		0	
	0		0		0	
	0		0		0	
	0		0		0	
	0		0		0	
	0		0		0	
	0		0		0	
Ew-AU->in-RG	1	Ew-AU->in-RG	1	Ewa-RG-art_E	1	Ewa-RG-art_E
Ew-AU->in-RG	1	Ew-AU->in-RG	1	Ewa-RG-art_E	1	Ewa-RG-art_E
	0		0		0	
	0		0		0	
	0		0		0	
Ew-AU->in-RG	1	Ew-AU->in-RG	1	Ewa-RG-art_E	1	Ewa-RG-art_E
Ew-AU->in-RG	1	Ew-AU->in-RG	1	Ewa-RG-art_E	1	Ewa-RG-art_E
	0		0		0	
	0		0		0	
Ew-AU->in-RG	1	Ew-AU->in-RG	1	Ewa-RG-art_E	1	Ewa-RG-art_E
Ew-AU->in-RG	1	Ew-AU->in-RG	1	Ewa-RG-art_E	1	Ewa-RG-art_E
Ew-AU->in-RG	1	Ew-AU->in-RG	1	Ewa-RG-art_E	1	Ewa-RG-art_E
Ew-AU->in-RG	1	Ew-AU->in-RG	1	Ewa-RG-art_E	1	Ewa-RG-art_E
Ew-AU->in-RG	1		0	Ewa-RG-art_E	1	
	0		0		0	
Ew-AU->in-RG	1	Ew-AU->in-RG	1	Ewa-RG-art_E	1	Ewa-RG-art_E
	0		0		0	
	0		0		0	
	0		0		0	
	0		0		0	
	0		0		0	
	0		0		0	
	0		0		0	
	0		0		0	
	0		0		0	
	0		0		0	
	0		0		0	
	0		0		0	
	0		0		0	
Ew-AU->in-RG	1	Ew-AU->in-RG	1	Ewa-RG-art_E	1	Ewa-RG-art_E
	0		0		0	
	0		0		0	
Ew-AU->in-RG	1		0		0	
Ew-AU->in-RG	1	Ew-AU->in-RG	1	Ewa-RG-art_E	1	Ewa-RG-art_E
	0		0		0	
	0		0		0	
	0		0		0	

	0		0		0	
	0		0		0	
	0		0		0	
	0		0		0	
	0		0		0	
	0		0		0	
	0		0		0	
	0		0		0	
Ew-AU->in-RG	1	Ew-AU->in-RG	1	Ewa-RG-art_E	1	Ewa-RG-art_E
Ew-AU->in-RG	1	Ew-AU->in-RG	1	Ewa-RG-art_E	1	Ewa-RG-art_E
	0		0		0	
	0		0		0	
	0		0		0	
	0		0		0	
	0		0		0	
	0		0		0	
Ew-AU->in-RG	1	Ew-AU->in-RG	1	Ewa-RG-art_E	1	Ewa-RG-art_E
	0		0		0	
	0		0		0	
	0		0		0	
Ew-AU->in-RG	1	Ew-AU->in-RG	1	Ewa-RG-art_E	1	Ewa-RG-art_E
Ew-AU->in-RG	1	Ew-AU->in-RG	1	Ewa-RG-art_E	1	Ewa-RG-art_E
	0		0		0	
	0		0		0	
	0		0		0	
	0		0		0	
Ew-AU->in-RG	1		0	Ewa-RG-art_E	1	
	0		0		0	
	0		0		0	
	0		0		0	
	0		0		0	
	0		0		0	
	0		0		0	
	0		0		0	
	0		0		0	
Ew-AU->in-RG	1	Ew-AU->in-RG	1	Ewa-RG-art_E	1	Ewa-RG-art_E
	0		0		0	
Ew-AU->in-RG	1		0	Ewa-RG-art_E	1	
	0		0		0	
	0		0		0	
Ew-AU->in-RG	1	Ew-AU->in-RG	1	Ewa-RG-art_E	1	Ewa-RG-art_E
Ew-AU->in-RG	1	Ew-AU->in-RG	1	Ewa-RG-art_E	1	Ewa-RG-art_E
	0		0		0	
	0		0		0	
	0		0		0	
	0		0		0	
Ew-AU->in-RG	1	Ew-AU->in-RG	1	Ewa-RG-art_E	1	Ewa-RG-art_E

Ew-AU->in-RG	1	Ew-AU->in-RG	1	Ewa-RG-art_E	1	Ewa-RG-art_E
Ew-AU->in-RG	1	Ew-AU->in-RG	1	Ewa-RG-art_E	1	Ewa-RG-art_E
Ew-AU->in-RG	1		0	Ewa-RG-art_E	1	
Ew-AU->in-RG	1		0	Ewa-RG-art_E	1	
Ew-AU->in-RG	1		0	Ewa-RG-art_E	1	
Ew-AU->in-RG	1		0		0	
Ew-AU->in-RG	1		0		0	
Ew-AU->in-RG	1	Ew-AU->in-RG	1	Ewa-RG-art_E	1	Ewa-RG-art_E
	0		0		0	
	0		0		0	
Ew-AU->in-RG	1	Ew-AU->in-RG	1	Ewa-RG-art_E	1	Ewa-RG-art_E
Ew-AU->in-RG	1	Ew-AU->in-RG	1	Ewa-RG-art_E	1	Ewa-RG-art_E
Ew-AU->in-RG	1	Ew-AU->in-RG	1	Ewa-RG-art_E	1	Ewa-RG-art_E
	0		0		0	
	0		0		0	
	0		0		0	
	0		0		0	
	0		0		0	
	0		0		0	
	0		0		0	
	0		0		0	
Ew-AU->in-RG	1	Ew-AU->in-RG	1	Ewa-RG-art_E	1	Ewa-RG-art_E
Ew-AU->in-RG	1	Ew-AU->in-RG	1	Ewa-RG-art_E	1	Ewa-RG-art_E
	0		0		0	
Ew-AU->in-RG	1		0		0	
Ew-AU->in-RG	1		0		0	
Ew-AU->in-RG	1	Ew-AU->in-RG	1	Ewa-RG-art_E	1	Ewa-RG-art_E
Ew-AU->in-RG	1	Ew-AU->in-RG	1	Ewa-RG-art_E	1	Ewa-RG-art_E
Ew-AU->in-RG	1		0	Ewa-RG-art_E	1	
Ew-AU->in-RG	1		0	Ewa-RG-art_E	1	
Ew-AU->in-RG	1	Ew-AU->in-RG	1	Ewa-RG-art_E	1	Ewa-RG-art_E
Ew-AU->in-RG	1	Ew-AU->in-RG	1	Ewa-RG-art_E	1	Ewa-RG-art_E
	0		0		0	
Ew-AU->in-RG	1		0	Ewa-RG-art_E	1	
	0		0		0	
	0		0		0	
Ew-AU->in-RG	1	Ew-AU->in-RG	1	Ewa-RG-art_E	1	Ewa-RG-art_E
Ew-AU->in-RG	1	Ew-AU->in-RG	1	Ewa-RG-art_E	1	Ewa-RG-art_E
Ew-AU->in-RG	1		0	Ewa-RG-art_E	1	
Ew-AU->in-RG	1		0	Ewa-RG-art_E	1	
Ew-AU->in-RG	1		0	Ewa-RG-art_E	1	
Ew-AU->in-RG	1		0	Ewa-RG-art_E	1	
	0		0		0	
	0		0		0	
	0		0		0	
	0		0		0	
	0		0		0	

	0		0		0	
Ew-AU->in-RG	1	Ew-AU->in-RG	1	Ewa-RG-art_E	1	Ewa-RG-art_E
Ew-AU->in-RG	1	Ew-AU->in-RG	1	Ewa-RG-art_E	1	Ewa-RG-art_E
Ew-AU->in-RG	1	Ew-AU->in-RG	1	Ewa-RG-art_E	1	Ewa-RG-art_E
	0		0		0	
Ew-AU->in-RG	1	Ew-AU->in-RG	1	Ewa-RG-art_E	1	Ewa-RG-art_E
	0		0		0	
Ew-AU->in-RG	1		0	Ewa-RG-art_E	1	
Ew-AU->in-RG	1		0	Ewa-RG-art_E	1	
Ew-AU->in-RG	1	Ew-AU->in-RG	1	Ewa-RG-art_E	1	Ewa-RG-art_E
	0		0		0	
	0		0		0	
	0		0		0	
Ew-AU->in-RG	1	Ew-AU->in-RG	1	Ewa-RG-art_E	1	Ewa-RG-art_E
Ew-AU->in-RG	1	Ew-AU->in-RG	1	Ewa-RG-art_E	1	Ewa-RG-art_E
	0		0		0	
	0		0		0	
	0		0		0	
	0		0		0	
Ew-AU->in-RG	1	Ew-AU->in-RG	1	Ewa-RG-art_E	1	Ewa-RG-art_E
Ew-AU->in-RG	1	Ew-AU->in-RG	1	Ewa-RG-art_E	1	Ewa-RG-art_E
Ew-AU->in-RG	1	Ew-AU->in-RG	1	Ewa-RG-art_E	1	Ewa-RG-art_E
Ew-AU->in-RG	1	Ew-AU->in-RG	1	Ewa-RG-art_E	1	Ewa-RG-art_E
	0		0		0	
	0		0		0	
	0		0		0	
	0		0		0	
	0		0		0	
	0		0		0	
Ew-AU->in-RG	1	Ew-AU->in-RG	1	Ewa-RG-art_E	1	Ewa-RG-art_E
Ew-AU->in-RG	1	Ew-AU->in-RG	1	Ewa-RG-art_E	1	Ewa-RG-art_E
	0		0		0	
	0		0		0	
	0		0		0	
Ew-AU->in-RG	1	Ew-AU->in-RG	1	Ewa-RG-art_E	1	Ewa-RG-art_E
	0		0		0	
	0		0		0	
	0		0		0	
	0		0		0	
	0		0		0	
	0		0		0	
	0		0		0	
	0		0		0	
	0		0		0	
	0		0		0	
	0		0		0	
	0		0		0	

	0		0		0	
	0		0		0	
	0		0		0	
	0		0		0	
	0		0		0	
	0		0		0	
	0		0		0	
	0		0		0	
	0		0		0	
	0		0		0	
Ew-AU->in-RG	1		0	Ewa-RG-art_E	1	
Ew-AU->in-RG	1		0	Ewa-RG-art_E	1	
Ew-AU->in-RG	1		0	Ewa-RG-art_E	1	
Ew-AU->in-RG	1		0	Ewa-RG-art_E	1	
	0		0		0	
	0		0		0	
Ew-AU->in-RG	1	Ew-AU->in-RG	1	Ewa-RG-art_E	1	Ewa-RG-art_E
Ew-AU->in-RG	1	Ew-AU->in-RG	1	Ewa-RG-art_E	1	Ewa-RG-art_E
	0		0		0	
	0		0		0	
	0		0		0	
	0		0		0	
	0		0		0	
	0		0		0	
	0		0		0	
	0		0		0	
	0		0		0	
	0		0		0	
	0		0		0	
	0		0		0	
	0		0		0	
	0		0		0	
	0		0		0	
	0		0		0	
	0		0		0	
Ew-AU->in-RG	1	Ew-AU->in-RG	1	Ewa-RG-art_E	1	Ewa-RG-art_E
Ew-AU->in-RG	1	Ew-AU->in-RG	1	Ewa-RG-art_E	1	Ewa-RG-art_E
	0		0		0	
	0		0		0	
	0		0		0	
	0		0		0	
Ew-AU->in-RG	1	Ew-AU->in-RG	1	Ewa-RG-art_E	1	Ewa-RG-art_E
Ew-AU->in-RG	1	Ew-AU->in-RG	1	Ewa-RG-art_E	1	Ewa-RG-art_E
Ew-AU->in-RG	1	Ew-AU->in-RG	1	Ewa-RG-art_E	1	Ewa-RG-art_E
	0		0		0	
	0		0		0	
	0		0		0	

	0		0		0	
Ew-AU->in-RG	1	Ew-AU->in-RG	1	Ewa-RG-art_E	1	Ewa-RG-art_E
	0		0		0	
	0		0		0	
	0		0		0	
	0		0		0	
	0		0		0	
	0		0		0	
	0		0		0	
Ew-AU->in-RG	1	Ew-AU->in-RG	1	Ewa-RG-art_E	1	Ewa-RG-art_E
Ew-AU->in-RG	1	Ew-AU->in-RG	1	Ewa-RG-art_E	1	Ewa-RG-art_E
Ew-AU->in-RG	1	Ew-AU->in-RG	1	Ewa-RG-art_E	1	Ewa-RG-art_E
	0		0		0	
	0		0		0	
	0		0		0	
	0		0		0	
Ew-AU->in-RG	1	Ew-AU->in-RG	1	Ewa-RG-art_E	1	Ewa-RG-art_E
Ew-AU->in-RG	1	Ew-AU->in-RG	1	Ewa-RG-art_E	1	Ewa-RG-art_E
	0		0		0	
	0		0		0	
Ew-AU->in-RG	1		0	Ewa-RG-art_E	1	
Ew-AU->in-RG	1		0	Ewa-RG-art_E	1	
Ew-AU->in-RG	1	Ew-AU->in-RG	1	Ewa-RG-art_E	1	Ewa-RG-art_E
	0		0		0	
	0		0		0	
	0		0		0	
	0		0		0	
	0		0		0	
	0		0		0	
Ew-AU->in-RG	1	Ew-AU->in-RG	1	Ewa-RG-art_E	1	Ewa-RG-art_E
Ew-AU->in-RG	1	Ew-AU->in-RG	1	Ewa-RG-art_E	1	Ewa-RG-art_E
	0		0		0	
	0		0		0	
	0		0		0	
	0		0		0	
	0		0		0	
Ew-AU->in-RG	1	Ew-AU->in-RG	1	Ewa-RG-art_E	1	Ewa-RG-art_E
	0		0		0	
	0		0		0	
	0		0		0	
	0		0		0	
	0		0		0	
	0		0		0	
	0		0		0	
	0		0		0	
	0		0		0	
	0		0		0	

	0		0		0	
	0		0		0	
Ew-AU->in-RG	1	Ew-AU->in-RG	1	Ewa-RG-art_E	1	Ewa-RG-art_E
	0		0		0	
	0		0		0	
	0		0		0	
	0		0		0	
	0		0		0	
Ew-AU->in-RG	1	Ew-AU->in-RG	1	Ewa-RG-art_E	1	Ewa-RG-art_E
	0		0		0	
	0		0		0	
	0		0		0	
	0		0		0	
	0		0		0	
	0		0		0	
	0		0		0	
	0		0		0	
	0		0		0	
	0		0		0	
Ew-AU->in-RG	1	Ew-AU->in-RG	1	Ewa-RG-art_E	1	Ewa-RG-art_E
	0		0		0	
	0		0		0	
	0		0		0	
	0		0		0	
	0		0		0	
	0		0		0	
Ew-AU->in-RG	1		0	Ewa-RG-art_E	1	
Ew-AU->in-RG	1		0	Ewa-RG-art_E	1	
Ew-AU->in-RG	1		0		0	
Ew-AU->in-RG	1	Ew-AU->in-RG	1	Ewa-RG-art_E	1	Ewa-RG-art_E
Ew-AU->in-RG	1	Ew-AU->in-RG	1	Ewa-RG-art_E	1	Ewa-RG-art_E
Ew-AU->in-RG	1	Ew-AU->in-RG	1	Ewa-RG-art_E	1	Ewa-RG-art_E
Ew-AU->in-RG	1	Ew-AU->in-RG	1	Ewa-RG-art_E	1	Ewa-RG-art_E
	0		0		0	
Ew-AU->in-RG	1	Ew-AU->in-RG	1	Ewa-RG-art_E	1	Ewa-RG-art_E
Ew-AU->in-RG	1	Ew-AU->in-RG	1	Ewa-RG-art_E	1	Ewa-RG-art_E
Ew-AU->in-RG	1	Ew-AU->in-RG	1	Ewa-RG-art_E	1	Ewa-RG-art_E
	0		0		0	
	0		0		0	
Ew-AU->in-RG	1	Ew-AU->in-RG	1	Ewa-RG-art_E	1	Ewa-RG-art_E
	0		0		0	
	0		0		0	
Ew-AU->in-RG	1	Ew-AU->in-RG	1	Ewa-RG-art_E	1	Ewa-RG-art_E
Ew-AU->in-RG	1	Ew-AU->in-RG	1	Ewa-RG-art_E	1	Ewa-RG-art_E
Ew-AU->in-RG	1	Ew-AU->in-RG	1	Ewa-RG-art_E	1	Ewa-RG-art_E
	0		0		0	
	0		0		0	

	0		0		0	
	0		0		0	
Ew-AU->in-RG	1	Ew-AU->in-RG	1	Ewa-RG-art_E	1	Ewa-RG-art_E
	0		0		0	
	0		0		0	
Ew-AU->in-RG	1		0	Ewa-RG-art_E	1	
Ew-AU->in-RG	1		0	Ewa-RG-art_E	1	
	0		0		0	
	0		0		0	
	0		0		0	
	0		0		0	
	0		0		0	
	0		0		0	
	0		0		0	
	0		0		0	
	0		0		0	
	0		0		0	
	0		0		0	
Ew-AU->in-RG	1	Ew-AU->in-RG	1	Ewa-RG-art_E	1	Ewa-RG-art_E
Ew-AU->in-RG	1		0		0	
	0		0		0	
	0		0		0	
	0		0		0	
	0		0		0	
Ew-AU->in-RG	1	Ew-AU->in-RG	1	Ewa-RG-art_E	1	Ewa-RG-art_E
	0		0		0	
	0		0		0	
	0		0		0	
	0		0		0	
Ew-AU->in-RG	1	Ew-AU->in-RG	1	Ewa-RG-art_E	1	Ewa-RG-art_E
	0		0		0	
	0		0		0	
	0		0		0	
Ew-AU->in-RG	1	Ew-AU->in-RG	1	Ewa-RG-art_E	1	Ewa-RG-art_E
	0		0		0	
	0		0		0	
	0		0		0	
Ew-AU->in-RG	1	Ew-AU->in-RG	1	Ewa-RG-art_E	1	Ewa-RG-art_E
Ew-AU->in-RG	1	Ew-AU->in-RG	1	Ewa-RG-art_E	1	Ewa-RG-art_E
Ew-AU->in-RG	1	Ew-AU->in-RG	1	Ewa-RG-art_E	1	Ewa-RG-art_E
	0		0		0	
	0		0		0	
	0		0		0	
Ew-AU->in-RG	1	Ew-AU->in-RG	1	Ewa-RG-art_E	1	Ewa-RG-art_E
Ew-AU->in-RG	1	Ew-AU->in-RG	1	Ewa-RG-art_E	1	Ewa-RG-art_E
Ew-AU->in-RG	1	Ew-AU->in-RG	1	Ewa-RG-art_E	1	Ewa-RG-art_E
	0		0		0	

	0		0		0	
Ew-AU->in-RG	1		0		0	
	0		0		0	
	0		0		0	
Ew-AU->in-RG	1	Ew-AU->in-RG	1	Ewa-RG-art_E	1	Ewa-RG-art_E
Ew-AU->in-RG	1	Ew-AU->in-RG	1	Ewa-RG-art_E	1	Ewa-RG-art_E
	0		0		0	
	0		0		0	
Ew-AU->in-RG	1		0	Ewa-RG-art_E	1	
Ew-AU->in-RG	1		0	Ewa-RG-art_E	1	
Ew-AU->in-RG	1		0	Ewa-RG-art_E	1	
Ew-AU->in-RG	1		0	Ewa-RG-art_E	1	
	0		0		0	
	0		0		0	
	0		0		0	
	0		0		0	
Ew-AU->in-RG	1	Ew-AU->in-RG	1	Ewa-RG-art_E	1	Ewa-RG-art_E
Ew-AU->in-RG	1	Ew-AU->in-RG	1	Ewa-RG-art_E	1	Ewa-RG-art_E
Ew-AU->in-RG	1	Ew-AU->in-RG	1	Ewa-RG-art_E	1	Ewa-RG-art_E
	0		0		0	
	0		0		0	
	0		0		0	
	0		0		0	
	0		0		0	
	0		0		0	
	0		0		0	
	0		0		0	
	0		0		0	
	0		0		0	
	0		0		0	
Ew-AU->in-RG	1		0	Ewa-RG-art_E	1	
	0		0		0	
	0		0		0	
	0		0		0	
	0		0		0	
	0		0		0	
	0		0		0	
	0		0		0	
	0		0		0	
	0		0		0	
	0		0		0	
	0		0		0	
Ew-AU->in-RG	1	Ew-AU->in-RG	1	Ewa-RG-art_E	1	Ewa-RG-art_E
	0		0		0	
	0		0		0	
	0		0		0	
	0		0		0	

	0		0		0	
	0		0		0	
	0		0		0	
Ew-AU->in-RG	1	Ew-AU->in-RG	1	Ewa-RG-art_E	1	Ewa-RG-art_E
Ew-AU->in-RG	1	Ew-AU->in-RG	1	Ewa-RG-art_E	1	Ewa-RG-art_E
Ew-AU->in-RG	1	Ew-AU->in-RG	1	Ewa-RG-art_E	1	Ewa-RG-art_E
Ew-AU->in-RG	1	Ew-AU->in-RG	1	Ewa-RG-art_E	1	Ewa-RG-art_E
Ew-AU->in-RG	1	Ew-AU->in-RG	1	Ewa-RG-art_E	1	Ewa-RG-art_E
	0		0		0	
	0		0		0	
	0		0		0	
Ew-AU->in-RG	1	Ew-AU->in-RG	1	Ewa-RG-art_E	1	Ewa-RG-art_E
	0		0		0	
	0		0		0	
	0		0		0	
	0		0		0	
	0		0		0	
	0		0		0	
	0		0		0	
	0		0		0	
	0		0		0	
	0		0		0	
Ew-AU->in-RG	1		0		0	
Ew-AU->in-RG	1		0		0	
	0		0		0	
	0		0		0	
	0		0		0	
	0		0		0	
	0		0		0	
	0		0		0	
	0		0		0	
	0		0		0	
	0		0		0	
	0		0		0	
	0		0		0	
	0		0		0	
	0		0		0	
	0		0		0	
	0		0		0	
Ew-AU->in-RG	1	Ew-AU->in-RG	1	Ewa-RG-art_E	1	Ewa-RG-art_E
Ew-AU->in-RG	1	Ew-AU->in-RG	1	Ewa-RG-art_E	1	Ewa-RG-art_E
	0		0		0	
	0		0		0	
	0		0		0	
	0		0		0	
	0		0		0	
	0		0		0	

	0		0		0	
	0		0		0	
	0		0		0	
	0		0		0	
	0		0		0	
	0		0		0	
	0		0		0	
	0		0		0	
	0		0		0	
	0		0		0	
	0		0		0	
	0		0		0	
	0		0		0	
Ew-AU->in-RG	1	Ew-AU->in-RG	1	Ewa-RG-art_E	1	Ewa-RG-art_E
Ew-AU->in-RG	1	Ew-AU->in-RG	1	Ewa-RG-art_E	1	Ewa-RG-art_E
	0		0		0	
	0		0		0	
	0		0		0	
	0		0		0	
Ew-AU->in-RG	1		0	Ewa-RG-art_E	1	
Ew-AU->in-RG	1		0	Ewa-RG-art_E	1	
	0		0		0	
	0		0		0	
	0		0		0	
	0		0		0	
Ew-AU->in-RG	1		0	Ewa-RG-art_E	1	
Ew-AU->in-RG	1		0	Ewa-RG-art_E	1	
	0		0		0	
	0		0		0	
Ew-AU->in-RG	1		0		0	
	0		0		0	
	0		0		0	
Ew-AU->in-RG	1	Ew-AU->in-RG	1	Ewa-RG-art_E	1	Ewa-RG-art_E
	0		0		0	
	0		0		0	
Ew-AU->in-RG	1	Ew-AU->in-RG	1	Ewa-RG-art_E	1	Ewa-RG-art_E
Ew-AU->in-RG	1	Ew-AU->in-RG	1	Ewa-RG-art_E	1	Ewa-RG-art_E
	0		0		0	
	0		0		0	
Ew-AU->in-RG	1		0	Ewa-RG-art_E	1	
	0		0		0	
	0		0		0	
	0		0		0	
	0		0		0	
Ew-AU->in-RG	1		0	Ewa-RG-art_E	1	
	0		0		0	
	0		0		0	

Ew-AU->in-RG	1	Ew-AU->in-RG	1	Ewa-RG-art_E	1	Ewa-RG-art_E
Ew-AU->in-RG	1	Ew-AU->in-RG	1	Ewa-RG-art_E	1	Ewa-RG-art_E
	0		0		0	
	0		0		0	
	0		0		0	
	0		0		0	
	0		0		0	
	0		0		0	
Ew-AU->in-RG	1		0	Ewa-RG-art_E	1	
Ew-AU->in-RG	1		0	Ewa-RG-art_E	1	
	0		0		0	
	0		0		0	
	0		0		0	
	0		0		0	
Ew-AU->in-RG	1	Ew-AU->in-RG	1	Ewa-RG-art_E	1	Ewa-RG-art_E
	0		0		0	
	0		0		0	
Ew-AU->in-RG	1	Ew-AU->in-RG	1	Ewa-RG-art_E	1	Ewa-RG-art_E
Ew-AU->in-RG	1	Ew-AU->in-RG	1	Ewa-RG-art_E	1	Ewa-RG-art_E
Ew-AU->in-RG	1		0	Ewa-RG-art_E	1	
Ew-AU->in-RG	1		0	Ewa-RG-art_E	1	
	0		0		0	
Ew-AU->in-RG	1	Ew-AU->in-RG	1	Ewa-RG-art_E	1	Ewa-RG-art_E
	0		0		0	
	0		0		0	
	0		0		0	
	0		0		0	
	0		0		0	
	0		0		0	
Ew-AU->in-RG	1		0		0	
	0		0		0	
	0		0		0	
	0		0		0	
Ew-AU->in-RG	1		0		0	
	0		0		0	
	0		0		0	
Ew-AU->in-RG	1		0	Ewa-RG-art_E	1	
Ew-AU->in-RG	1		0	Ewa-RG-art_E	1	
Ew-AU->in-RG	1		0	Ewa-RG-art_E	1	
	0		0		0	
	0		0		0	
	0		0		0	
Ew-AU->in-RG	1	Ew-AU->in-RG	1	Ewa-RG-art_E	1	Ewa-RG-art_E
	0		0		0	
	0		0		0	
	0		0		0	
	0		0		0	

	0		0		0	
	0		0		0	
	0		0		0	
	0		0		0	
	0		0		0	
Ew-AU->in-RG	1	Ew-AU->in-RG	1	Ewa-RG-art_E	1	Ewa-RG-art_E
Ew-AU->in-RG	1	Ew-AU->in-RG	1	Ewa-RG-art_E	1	Ewa-RG-art_E
Ew-AU->in-RG	1	Ew-AU->in-RG	1	Ewa-RG-art_E	1	Ewa-RG-art_E
	0		0		0	
	0		0		0	
Ew-AU->in-RG	1	Ew-AU->in-RG	1	Ewa-RG-art_E	1	Ewa-RG-art_E
Ew-AU->in-RG	1	Ew-AU->in-RG	1	Ewa-RG-art_E	1	Ewa-RG-art_E
	0		0		0	
	0		0		0	
	0		0		0	
	0		0		0	
Ew-AU->in-RG	1	Ew-AU->in-RG	1	Ewa-RG-art_E	1	Ewa-RG-art_E
Ew-AU->in-RG	1	Ew-AU->in-RG	1	Ewa-RG-art_E	1	Ewa-RG-art_E
	0		0		0	
	0		0		0	
	0		0		0	
	0		0		0	
	0		0		0	
	0		0		0	
	0		0		0	
	0		0		0	
	0		0		0	
	0		0		0	
	0		0		0	
	0		0		0	
	0		0		0	
	0		0		0	
	0		0		0	
	0		0		0	
Ew-AU->in-RG	1		0		0	
	0		0		0	
	0		0		0	
	0		0		0	
	0		0		0	
	0		0		0	
	0		0		0	
	0		0		0	
Ew-AU->in-RG	1	Ew-AU->in-RG	1	Ewa-RG-art_E	1	Ewa-RG-art_E
Ew-AU->in-RG	1	Ew-AU->in-RG	1	Ewa-RG-art_E	1	Ewa-RG-art_E
Ew-AU->in-RG	1	Ew-AU->in-RG	1	Ewa-RG-art_E	1	Ewa-RG-art_E
Ew-AU->in-RG	1	Ew-AU->in-RG	1	Ewa-RG-art_E	1	Ewa-RG-art_E
Ew-AU->in-RG	1	Ew-AU->in-RG	1	Ewa-RG-art_E	1	Ewa-RG-art_E

Ew-AU->in-RG	1	Ew-AU->in-RG	1	Ewa-RG-art_E	1	Ewa-RG-art_E
Ew-AU->in-RG	1	Ew-AU->in-RG	1	Ewa-RG-art_E	1	Ewa-RG-art_E
Ew-AU->in-RG	1	Ew-AU->in-RG	1	Ewa-RG-art_E	1	Ewa-RG-art_E
	0		0		0	
	0		0		0	
Ew-AU->in-RG	1	Ew-AU->in-RG	1	Ewa-RG-art_E	1	Ewa-RG-art_E
	0		0		0	
	0		0		0	
	0		0		0	
	0		0		0	
	0		0		0	
	0		0		0	
Ew-AU->in-RG	1	Ew-AU->in-RG	1	Ewa-RG-art_E	1	Ewa-RG-art_E
	0		0		0	
	0		0		0	
	0		0		0	
Ew-AU->in-RG	1	Ew-AU->in-RG	1	Ewa-RG-art_E	1	Ewa-RG-art_E
	0		0		0	
	0		0		0	
	0		0		0	
Ew-AU->in-RG	1	Ew-AU->in-RG	1	Ewa-RG-art_E	1	Ewa-RG-art_E
Ew-AU->in-RG	1	Ew-AU->in-RG	1	Ewa-RG-art_E	1	Ewa-RG-art_E
	0		0		0	
	0		0		0	
Ew-AU->in-RG	1	Ew-AU->in-RG	1	Ewa-RG-art_E	1	Ewa-RG-art_E
Ew-AU->in-RG	1	Ew-AU->in-RG	1	Ewa-RG-art_E	1	Ewa-RG-art_E
	0		0		0	
Ew-AU->in-RG	1	Ew-AU->in-RG	1	Ewa-RG-art_E	1	Ewa-RG-art_E
Ew-AU->in-RG	1	Ew-AU->in-RG	1	Ewa-RG-art_E	1	Ewa-RG-art_E
Ew-AU->in-RG	1	Ew-AU->in-RG	1	Ewa-RG-art_E	1	Ewa-RG-art_E
	0		0		0	
Ew-AU->in-RG	1	Ew-AU->in-RG	1	Ewa-RG-art_E	1	Ewa-RG-art_E
Ew-AU->in-RG	1	Ew-AU->in-RG	1	Ewa-RG-art_E	1	Ewa-RG-art_E
Ew-AU->in-RG	1	Ew-AU->in-RG	1	Ewa-RG-art_E	1	Ewa-RG-art_E
Ew-AU->in-RG	1	Ew-AU->in-RG	1	Ewa-RG-art_E	1	Ewa-RG-art_E
	0		0		0	
	0		0		0	
	0		0		0	
	0		0		0	
	0		0		0	
	0		0		0	
	0		0		0	
	0		0		0	
	0		0		0	
	0		0		0	
	0		0		0	
	0		0		0	

	0		0		0	
	0		0		0	
Ew-AU->in-RG	1		0	Ewa-RG-art_E	1	
Ew-AU->in-RG	1		0	Ewa-RG-art_E	1	
Ew-AU->in-RG	1		0	Ewa-RG-art_E	1	
Ew-AU->in-RG	1		0	Ewa-RG-art_E	1	
Ew-AU->in-RG	1	Ew-AU->in-RG	1	Ewa-RG-art_E	1	Ewa-RG-art_E
	0		0		0	
	0		0		0	
	0		0		0	
	0		0		0	
Ew-AU->in-RG	1	Ew-AU->in-RG	1	Ewa-RG-art_E	1	Ewa-RG-art_E
	0		0		0	
	0		0		0	
	0		0		0	
	0		0		0	
Ew-AU->in-RG	1		0	Ewa-RG-art_E	1	
Ew-AU->in-RG	1		0	Ewa-RG-art_E	1	
Ew-AU->in-RG	1		0	Ewa-RG-art_E	1	
Ew-AU->in-RG	1		0	Ewa-RG-art_E	1	
	0		0		0	
	0		0		0	
	0		0		0	
	0		0		0	
	0		0		0	
	0		0		0	
	0		0		0	
	0		0		0	
Ew-AU->in-RG	1	Ew-AU->in-RG	1	Ewa-RG-art_E	1	Ewa-RG-art_E
Ew-AU->in-RG	1	Ew-AU->in-RG	1	Ewa-RG-art_E	1	Ewa-RG-art_E
	0		0		0	
Ew-AU->in-RG	1	Ew-AU->in-RG	1	Ewa-RG-art_E	1	Ewa-RG-art_E
Ew-AU->in-RG	1	Ew-AU->in-RG	1	Ewa-RG-art_E	1	Ewa-RG-art_E
Ew-AU->in-RG	1	Ew-AU->in-RG	1	Ewa-RG-art_E	1	Ewa-RG-art_E
Ew-AU->in-RG	1	Ew-AU->in-RG	1	Ewa-RG-art_E	1	Ewa-RG-art_E
	0		0		0	
	0		0		0	
	0		0		0	
	0		0		0	
Ew-AU->in-RG	1	Ew-AU->in-RG	1	Ewa-RG-art_E	1	Ewa-RG-art_E
	0		0		0	
	0		0		0	
Ew-AU->in-RG	1	Ew-AU->in-RG	1	Ewa-RG-art_E	1	Ewa-RG-art_E
Ew-AU->in-RG	1	Ew-AU->in-RG	1	Ewa-RG-art_E	1	Ewa-RG-art_E
	0		0		0	
	0		0		0	
	0		0		0	

	0		0		0	
	0		0		0	
Ew-AU->in-RG	1	Ew-AU->in-RG	1	Ewa-RG-art_E	1	Ewa-RG-art_E
Ew-AU->in-RG	1	Ew-AU->in-RG	1	Ewa-RG-art_E	1	Ewa-RG-art_E
Ew-AU->in-RG	1	Ew-AU->in-RG	1	Ewa-RG-art_E	1	Ewa-RG-art_E
Ew-AU->in-RG	1		0	Ewa-RG-art_E	1	
Ew-AU->in-RG	1		0	Ewa-RG-art_E	1	
Ew-AU->in-RG	1		0	Ewa-RG-art_E	1	
	0		0		0	
	0		0		0	
Ew-AU->in-RG	1	Ew-AU->in-RG	1	Ewa-RG-art_E	1	Ewa-RG-art_E
	0		0		0	
	0		0		0	
	0		0		0	
Ew-AU->in-RG	1	Ew-AU->in-RG	1	Ewa-RG-art_E	1	Ewa-RG-art_E
Ew-AU->in-RG	1	Ew-AU->in-RG	1	Ewa-RG-art_E	1	Ewa-RG-art_E
	0		0		0	
Ew-AU->in-RG	1		0		0	
	0		0		0	
	0		0		0	
	0		0		0	
	0		0		0	
	0		0		0	
	0		0		0	
	0		0		0	
Ew-AU->in-RG	1	Ew-AU->in-RG	1	Ewa-RG-art_E	1	Ewa-RG-art_E
Ew-AU->in-RG	1	Ew-AU->in-RG	1	Ewa-RG-art_E	1	Ewa-RG-art_E
Ew-AU->in-RG	1	Ew-AU->in-RG	1	Ewa-RG-art_E	1	Ewa-RG-art_E
Ew-AU->in-RG	1	Ew-AU->in-RG	1	Ewa-RG-art_E	1	Ewa-RG-art_E
Ew-AU->in-RG	1	Ew-AU->in-RG	1	Ewa-RG-art_E	1	Ewa-RG-art_E
	0		0		0	
	0		0		0	
	0		0		0	
Ew-AU->in-RG	1	Ew-AU->in-RG	1	Ewa-RG-art_E	1	Ewa-RG-art_E
	0		0		0	
	0		0		0	
	0		0		0	
Ew-AU->in-RG	1		0	Ewa-RG-art_E	1	
Ew-AU->in-RG	1		0	Ewa-RG-art_E	1	
	0		0		0	
Ew-AU->in-RG	1	Ew-AU->in-RG	1	Ewa-RG-art_E	1	Ewa-RG-art_E
	0		0		0	
	0		0		0	
Ew-AU->in-RG	1	Ew-AU->in-RG	1	Ewa-RG-art_E	1	Ewa-RG-art_E
Ew-AU->in-RG	1	Ew-AU->in-RG	1	Ewa-RG-art_E	1	Ewa-RG-art_E
Ew-AU->in-RG	1	Ew-AU->in-RG	1	Ewa-RG-art_E	1	Ewa-RG-art_E
	0		0		0	

	0		0		0	
Ew-AU->in-RG	1	Ew-AU->in-RG	1	Ewa-RG-art_E	1	Ewa-RG-art_E
	0		0		0	
	0		0		0	
	0		0		0	
	0		0		0	
	0		0		0	
	0		0		0	
	0		0		0	
	0		0		0	
	0		0		0	
Ew-AU->in-RG	1	Ew-AU->in-RG	1	Ewa-RG-art_E	1	Ewa-RG-art_E
Ew-AU->in-RG	1		0	Ewa-RG-art_E	1	
Ew-AU->in-RG	1		0	Ewa-RG-art_E	1	
	0		0		0	
	0		0		0	
	0		0		0	
Ew-AU->in-RG	1		0	Ewa-RG-art_E	1	
Ew-AU->in-RG	1		0	Ewa-RG-art_E	1	
Ew-AU->in-RG	1	Ew-AU->in-RG	1	Ewa-RG-art_E	1	Ewa-RG-art_E
Ew-AU->in-RG	1	Ew-AU->in-RG	1	Ewa-RG-art_E	1	Ewa-RG-art_E
	0		0		0	
	0		0		0	
	0		0		0	
	0		0		0	
Ew-AU->in-RG	1	Ew-AU->in-RG	1	Ewa-RG-art_E	1	Ewa-RG-art_E
	0		0		0	
	0		0		0	
	0		0		0	
	0		0		0	
Ew-AU->in-RG	1		0	Ewa-RG-art_E	1	
Ew-AU->in-RG	1		0	Ewa-RG-art_E	1	
	0		0		0	
	0		0		0	
	0		0		0	
	0		0		0	
	0		0		0	
	0		0		0	
	0		0		0	
	0		0		0	
	0		0		0	
	0		0		0	
Ew-AU->in-RG	1		0		0	
Ew-AU->in-RG	1	Ew-AU->in-RG	1	Ewa-RG-art_E	1	Ewa-RG-art_E
Ew-AU->in-RG	1	Ew-AU->in-RG	1	Ewa-RG-art_E	1	Ewa-RG-art_E
	0		0		0	

	0		0		0	
	0		0		0	
	0		0		0	
	0		0		0	
	0		0		0	
	0		0		0	
Ew-AU->in-RG	1	Ew-AU->in-RG	1	Ewa-RG-art_E	1	Ewa-RG-art_E
Ew-AU->in-RG	1	Ew-AU->in-RG	1	Ewa-RG-art_E	1	Ewa-RG-art_E
	0		0		0	
	0		0		0	
Ew-AU->in-RG	1	Ew-AU->in-RG	1	Ewa-RG-art_E	1	Ewa-RG-art_E
Ew-AU->in-RG	1	Ew-AU->in-RG	1	Ewa-RG-art_E	1	Ewa-RG-art_E
	0		0		0	
	0		0		0	
	0		0		0	
Ew-AU->in-RG	1	Ew-AU->in-RG	1	Ewa-RG-art_E	1	Ewa-RG-art_E
Ew-AU->in-RG	1	Ew-AU->in-RG	1	Ewa-RG-art_E	1	Ewa-RG-art_E
	0		0		0	
	0		0		0	
	0		0		0	
	0		0		0	
Ew-AU->in-RG	1	Ew-AU->in-RG	1	Ewa-RG-art_E	1	Ewa-RG-art_E
Ew-AU->in-RG	1	Ew-AU->in-RG	1	Ewa-RG-art_E	1	Ewa-RG-art_E
Ew-AU->in-RG	1	Ew-AU->in-RG	1	Ewa-RG-art_E	1	Ewa-RG-art_E
	0		0		0	
Ew-AU->in-RG	1		0	Ewa-RG-art_E	1	
Ew-AU->in-RG	1		0	Ewa-RG-art_E	1	
	0		0		0	
	0		0		0	
	0		0		0	
	0		0		0	
	0		0		0	
Ew-AU->in-RG	1	Ew-AU->in-RG	1	Ewa-RG-art_E	1	Ewa-RG-art_E
Ew-AU->in-RG	1	Ew-AU->in-RG	1	Ewa-RG-art_E	1	Ewa-RG-art_E
	0		0		0	
	0		0		0	
	0		0		0	
Ew-AU->in-RG	1	Ew-AU->in-RG	1	Ewa-RG-art_E	1	Ewa-RG-art_E
Ew-AU->in-RG	1	Ew-AU->in-RG	1	Ewa-RG-art_E	1	Ewa-RG-art_E
	0		0		0	
	0		0		0	
	0		0		0	
	0		0		0	
	0		0		0	
Ew-AU->in-RG	1		0	Ewa-RG-art_E	1	
Ew-AU->in-RG	1		0	Ewa-RG-art_E	1	
	0		0		0	

Ew-AU->in-RG	1		0	Ewa-RG-art_E	1	
Ew-AU->in-RG	1		0	Ewa-RG-art_E	1	
Ew-AU->in-RG	1		0	Ewa-RG-art_E	1	
	0		0		0	
	0		0		0	
	0		0		0	
Ew-AU->in-RG	1		0		0	
Ew-AU->in-RG	1		0		0	
Ew-AU->in-RG	1		0		0	
Ew-AU->in-RG	1		0	Ewa-RG-art_E	1	
	0		0		0	
Ew-AU->in-RG	1	Ew-AU->in-RG	1	Ewa-RG-art_E	1	Ewa-RG-art_E
	0		0		0	
	0		0		0	
	0		0		0	
	0		0		0	
Ew-AU->in-RG	1	Ew-AU->in-RG	1	Ewa-RG-art_E	1	Ewa-RG-art_E
	0		0		0	
	0		0		0	
	0		0		0	
	0		0		0	
	0		0		0	
	0		0		0	
	0		0		0	
	0		0		0	
	0		0		0	
	0		0		0	
	0		0		0	
	0		0		0	
Ew-AU->in-RG	1	Ew-AU->in-RG	1	Ewa-RG-art_E	1	Ewa-RG-art_E
Ew-AU->in-RG	1	Ew-AU->in-RG	1	Ewa-RG-art_E	1	Ewa-RG-art_E
	0		0		0	
	0		0		0	
Ew-AU->in-RG	1	Ew-AU->in-RG	1	Ewa-RG-art_E	1	Ewa-RG-art_E
Ew-AU->in-RG	1	Ew-AU->in-RG	1	Ewa-RG-art_E	1	Ewa-RG-art_E
Ew-AU->in-RG	1	Ew-AU->in-RG	1	Ewa-RG-art_E	1	Ewa-RG-art_E
Ew-AU->in-RG	1	Ew-AU->in-RG	1	Ewa-RG-art_E	1	Ewa-RG-art_E
Ew-AU->in-RG	1	Ew-AU->in-RG	1	Ewa-RG-art_E	1	Ewa-RG-art_E
	0		0		0	
Ew-AU->in-RG	1	Ew-AU->in-RG	1	Ewa-RG-art_E	1	Ewa-RG-art_E
Ew-AU->in-RG	1	Ew-AU->in-RG	1	Ewa-RG-art_E	1	Ewa-RG-art_E
Ew-AU->in-RG	1	Ew-AU->in-RG	1	Ewa-RG-art_E	1	Ewa-RG-art_E
	0		0		0	
Ew-AU->in-RG	1	Ew-AU->in-RG	1	Ewa-RG-art_E	1	Ewa-RG-art_E
Ew-AU->in-RG	1	Ew-AU->in-RG	1	Ewa-RG-art_E	1	Ewa-RG-art_E
Ew-AU->in-RG	1	Ew-AU->in-RG	1	Ewa-RG-art_E	1	Ewa-RG-art_E
	0		0		0	

Ew-AU->in-RG	1	Ew-AU->in-RG	1	Ewa-RG-art_E	1	Ewa-RG-art_E
	0		0		0	
	0		0		0	
	0		0		0	
	0		0		0	
	0		0		0	
	0		0		0	
	0		0		0	
	0		0		0	
	0		0		0	
	0		0		0	
	0		0		0	
	0		0		0	
	0		0		0	
Ew-AU->in-RG	1	Ew-AU->in-RG	1	Ewa-RG-art_E	1	Ewa-RG-art_E
Ew-AU->in-RG	1	Ew-AU->in-RG	1	Ewa-RG-art_E	1	Ewa-RG-art_E
Ew-AU->in-RG	1	Ew-AU->in-RG	1	Ewa-RG-art_E	1	Ewa-RG-art_E
	0		0		0	
	0		0		0	
	0		0		0	
	0		0		0	
	0		0		0	
	0		0		0	
Ew-AU->in-RG	1	Ew-AU->in-RG	1	Ewa-RG-art_E	1	Ewa-RG-art_E
	0		0		0	
	0		0		0	
	0		0		0	
	0		0		0	
	0		0		0	
	0		0		0	
	0		0		0	
	0		0		0	
	0		0		0	
Ew-AU->in-RG	1		0	Ewa-RG-art_E	1	
Ew-AU->in-RG	1		0	Ewa-RG-art_E	1	
	0		0		0	
	0		0		0	
Ew-AU->in-RG	1		0	Ewa-RG-art_E	1	
Ew-AU->in-RG	1		0	Ewa-RG-art_E	1	
Ew-AU->in-RG	1	Ew-AU->in-RG	1	Ewa-RG-art_E	1	Ewa-RG-art_E
Ew-AU->in-RG	1	Ew-AU->in-RG	1	Ewa-RG-art_E	1	Ewa-RG-art_E
	0		0		0	
	0		0		0	
	0		0		0	
	0		0		0	
	0		0		0	
Ew-AU->in-RG	1	Ew-AU->in-RG	1	Ewa-RG-art_E	1	Ewa-RG-art_E

	0		0		0	
	0		0		0	
	0		0		0	
	0		0		0	
	0		0		0	
	0		0		0	
Ew-AU->in-RG	1	Ew-AU->in-RG	1	Ewa-RG-art_E	1	Ewa-RG-art_E
	0		0		0	
	0		0		0	
Ew-AU->in-RG	1		0		0	
Ew-AU->in-RG	1		0		0	
	0		0		0	
	0		0		0	
	0		0		0	
	0		0		0	
	0		0		0	
Ew-AU->in-RG	1	Ew-AU->in-RG	1	Ewa-RG-art_E	1	Ewa-RG-art_E
Ew-AU->in-RG	1	Ew-AU->in-RG	1	Ewa-RG-art_E	1	Ewa-RG-art_E
	0		0		0	
	0		0		0	
	0		0		0	
	0		0		0	
	0		0		0	
	0		0		0	
	0		0		0	
Ew-AU->in-RG	1	Ew-AU->in-RG	1	Ewa-RG-art_E	1	Ewa-RG-art_E
Ew-AU->in-RG	1	Ew-AU->in-RG	1	Ewa-RG-art_E	1	Ewa-RG-art_E
Ew-AU->in-RG	1	Ew-AU->in-RG	1	Ewa-RG-art_E	1	Ewa-RG-art_E
Ew-AU->in-RG	1	Ew-AU->in-RG	1	Ewa-RG-art_E	1	Ewa-RG-art_E
	0		0		0	
	0		0		0	
	0		0		0	
	0		0		0	
	0		0		0	
	0		0		0	
	0		0		0	
	0		0		0	
	0		0		0	
	0		0		0	
	0		0		0	
	0		0		0	
Ew-AU->in-RG	1	Ew-AU->in-RG	1	Ewa-RG-art_E	1	Ewa-RG-art_E
	0		0		0	
Ew-AU->in-RG	1	Ew-AU->in-RG	1	Ewa-RG-art_E	1	Ewa-RG-art_E
Ew-AU->in-RG	1	Ew-AU->in-RG	1	Ewa-RG-art_E	1	Ewa-RG-art_E
Ew-AU->in-RG	1	Ew-AU->in-RG	1	Ewa-RG-art_E	1	Ewa-RG-art_E
	0		0		0	

	0		0		0	
	0		0		0	
	0		0		0	
	0		0		0	
	0		0		0	
	0		0		0	
	0		0		0	
	0		0		0	
	0		0		0	
	0		0		0	
Ew-AU->in-RG	1	Ew-AU->in-RG	1	Ewa-RG-art_E	1	Ewa-RG-art_E
Ew-AU->in-RG	1	Ew-AU->in-RG	1	Ewa-RG-art_E	1	Ewa-RG-art_E
	0		0		0	
	0		0		0	
	0		0		0	
Ew-AU->in-RG	1	Ew-AU->in-RG	1	Ewa-RG-art_E	1	Ewa-RG-art_E
Ew-AU->in-RG	1	Ew-AU->in-RG	1	Ewa-RG-art_E	1	Ewa-RG-art_E
Ew-AU->in-RG	1	Ew-AU->in-RG	1	Ewa-RG-art_E	1	Ewa-RG-art_E
Ew-AU->in-RG	1	Ew-AU->in-RG	1	Ewa-RG-art_E	1	Ewa-RG-art_E
Ew-AU->in-RG	1	Ew-AU->in-RG	1	Ewa-RG-art_E	1	Ewa-RG-art_E
Ew-AU->in-RG	1	Ew-AU->in-RG	1	Ewa-RG-art_E	1	Ewa-RG-art_E
Ew-AU->in-RG	1	Ew-AU->in-RG	1	Ewa-RG-art_E	1	Ewa-RG-art_E
Ew-AU->in-RG	1	Ew-AU->in-RG	1	Ewa-RG-art_E	1	Ewa-RG-art_E
	0		0		0	
Ew-AU->in-RG	1	Ew-AU->in-RG	1	Ewa-RG-art_E	1	Ewa-RG-art_E
Ew-AU->in-RG	1	Ew-AU->in-RG	1	Ewa-RG-art_E	1	Ewa-RG-art_E
Ew-AU->in-RG	1	Ew-AU->in-RG	1	Ewa-RG-art_E	1	Ewa-RG-art_E
Ew-AU->in-RG	1	Ew-AU->in-RG	1	Ewa-RG-art_E	1	Ewa-RG-art_E
	0		0		0	
	0		0		0	
	0		0		0	
	0		0		0	
	0		0		0	
Ew-AU->in-RG	1	Ew-AU->in-RG	1	Ewa-RG-art_E	1	Ewa-RG-art_E
	0		0		0	
	0		0		0	
Ew-AU->in-RG	1		0	Ewa-RG-art_E	1	
Ew-AU->in-RG	1		0	Ewa-RG-art_E	1	
Ew-AU->in-RG	1		0	Ewa-RG-art_E	1	
Ew-AU->in-RG	1	Ew-AU->in-RG	1	Ewa-RG-art_E	1	Ewa-RG-art_E
	0		0		0	
	0		0		0	
	0		0		0	
	0		0		0	
Ew-AU->in-RG	1		0	Ewa-RG-art_E	1	
Ew-AU->in-RG	1		0	Ewa-RG-art_E	1	
Ew-AU->in-RG	1		0	Ewa-RG-art_E	1	

Ew-AU->in-RG	1	Ew-AU->in-RG	1	Ewa-RG-art_E	1	Ewa-RG-art_E
Ew-AU->in-RG	1	Ew-AU->in-RG	1	Ewa-RG-art_E	1	Ewa-RG-art_E
	0		0		0	
	0		0		0	
	0		0		0	
	0		0		0	
Ew-AU->in-RG	1	Ew-AU->in-RG	1	Ewa-RG-art_E	1	Ewa-RG-art_E
	0		0		0	
	0		0		0	
	0		0		0	
	0		0		0	
	0		0		0	
	0		0		0	
	0		0		0	
	0		0		0	
	0		0		0	
	0		0		0	
	0		0		0	
	0		0		0	
	0		0		0	
	0		0		0	
Ew-AU->in-RG	1		0		0	
Ew-AU->in-RG	1		0		0	
	0		0		0	
	0		0		0	
	0		0		0	
	0		0		0	
	0		0		0	
	0		0		0	
	0		0		0	
	0		0		0	
	0		0		0	
	0		0		0	
	0		0		0	
	0		0		0	
	0		0		0	
	0		0		0	
	0		0		0	
	0		0		0	
Ew-AU->in-RG	1	Ew-AU->in-RG	1	Ewa-RG-art_E	1	Ewa-RG-art_E
Ew-AU->in-RG	1	Ew-AU->in-RG	1	Ewa-RG-art_E	1	Ewa-RG-art_E
	0		0		0	
	0		0		0	
	0		0		0	
	0		0		0	
	0		0		0	
	0		0		0	

	0		0		0	
	0		0		0	
	0		0		0	
	0		0		0	
Ew-AU->in-RG	1	Ew-AU->in-RG	1	Ewa-RG-art_E	1	Ewa-RG-art_E
Ew-AU->in-RG	1	Ew-AU->in-RG	1	Ewa-RG-art_E	1	Ewa-RG-art_E
	0		0		0	
	0		0		0	
	0		0		0	
	0		0		0	
Ew-AU->in-RG	1	Ew-AU->in-RG	1	Ewa-RG-art_E	1	Ewa-RG-art_E
Ew-AU->in-RG	1	Ew-AU->in-RG	1	Ewa-RG-art_E	1	Ewa-RG-art_E
Ew-AU->in-RG	1	Ew-AU->in-RG	1	Ewa-RG-art_E	1	Ewa-RG-art_E
Ew-AU->in-RG	1	Ew-AU->in-RG	1	Ewa-RG-art_E	1	Ewa-RG-art_E
Ew-AU->in-RG	1	Ew-AU->in-RG	1	Ewa-RG-art_E	1	Ewa-RG-art_E
Ew-AU->in-RG	1		0	Ewa-RG-art_E	1	
Ew-AU->in-RG	1		0	Ewa-RG-art_E	1	
	0		0		0	
	0		0		0	
	0		0		0	
	0		0		0	
	0		0		0	
	0		0		0	
	0		0		0	
	0		0		0	
	0		0		0	
	0		0		0	
	0		0		0	
	0		0		0	
	0		0		0	
Ew-AU->in-RG	1	Ew-AU->in-RG	1	Ewa-RG-art_E	1	Ewa-RG-art_E
Ew-AU->in-RG	1	Ew-AU->in-RG	1	Ewa-RG-art_E	1	Ewa-RG-art_E
Ew-AU->in-RG	1	Ew-AU->in-RG	1	Ewa-RG-art_E	1	Ewa-RG-art_E
Ew-AU->in-RG	1	Ew-AU->in-RG	1	Ewa-RG-art_E	1	Ewa-RG-art_E
Ew-AU->in-RG	1	Ew-AU->in-RG	1	Ewa-RG-art_E	1	Ewa-RG-art_E
	0		0		0	
	0		0		0	
	0		0		0	
	0		0		0	
	0		0		0	
	0		0		0	
	0		0		0	
	0		0		0	
	0		0		0	
	0		0		0	
	0		0		0	
Ew-AU->in-RG	1	Ew-AU->in-RG	1	Ewa-RG-art_E	1	Ewa-RG-art_E

Ew-AU->in-RG	1	Ew-AU->in-RG	1	Ewa-RG-art_E	1	Ewa-RG-art_E
	0		0		0	
	0		0		0	
	0		0		0	
	0		0		0	
Ew-AU->in-RG	1		0	Ewa-RG-art_E	1	
Ew-AU->in-RG	1		0	Ewa-RG-art_E	1	
	0		0		0	
	0		0		0	
	0		0		0	
Ew-AU->in-RG	1	Ew-AU->in-RG	1	Ewa-RG-art_E	1	Ewa-RG-art_E
Ew-AU->in-RG	1	Ew-AU->in-RG	1	Ewa-RG-art_E	1	Ewa-RG-art_E
Ew-AU->in-RG	1	Ew-AU->in-RG	1	Ewa-RG-art_E	1	Ewa-RG-art_E
Ew-AU->in-RG	1	Ew-AU->in-RG	1	Ewa-RG-art_E	1	Ewa-RG-art_E
	0		0		0	
	0		0		0	
	0		0		0	
	0		0		0	
Ew-AU->in-RG	1	Ew-AU->in-RG	1	Ewa-RG-art_E	1	Ewa-RG-art_E
Ew-AU->in-RG	1	Ew-AU->in-RG	1	Ewa-RG-art_E	1	Ewa-RG-art_E
	0		0		0	
	0		0		0	
	0		0		0	
	0		0		0	
	0		0		0	
	0		0		0	
	0		0		0	
	0		0		0	
	0		0		0	
Ew-AU->in-RG	1		0	Ewa-RG-art_E	1	
Ew-AU->in-RG	1		0	Ewa-RG-art_E	1	
Ew-AU->in-RG	1		0	Ewa-RG-art_E	1	
	0		0		0	
	0		0		0	
	0		0		0	
	0		0		0	
	0		0		0	
Ew-AU->in-RG	1	Ew-AU->in-RG	1	Ewa-RG-art_E	1	Ewa-RG-art_E
Ew-AU->in-RG	1	Ew-AU->in-RG	1	Ewa-RG-art_E	1	Ewa-RG-art_E
	0		0		0	
	0		0		0	
	0		0		0	
	0		0		0	
Ew-AU->in-RG	1	Ew-AU->in-RG	1	Ewa-RG-art_E	1	Ewa-RG-art_E
Ew-AU->in-RG	1	Ew-AU->in-RG	1	Ewa-RG-art_E	1	Ewa-RG-art_E
Ew-AU->in-RG	1	Ew-AU->in-RG	1	Ewa-RG-art_E	1	Ewa-RG-art_E
	0		0		0	

	0		0		0	
	0		0		0	
Ew-AU->in-RG	1	Ew-AU->in-RG	1	Ewa-RG-art_E	1	Ewa-RG-art_E
Ew-AU->in-RG	1	Ew-AU->in-RG	1	Ewa-RG-art_E	1	Ewa-RG-art_E
	0		0		0	
Ew-AU->in-RG	1	Ew-AU->in-RG	1	Ewa-RG-art_E	1	Ewa-RG-art_E
Ew-AU->in-RG	1	Ew-AU->in-RG	1	Ewa-RG-art_E	1	Ewa-RG-art_E
	0		0		0	
	0		0		0	
	0		0		0	
	0		0		0	
	0		0		0	
	0		0		0	
	0		0		0	
Ew-AU->in-RG	1	Ew-AU->in-RG	1	Ewa-RG-art_E	1	Ewa-RG-art_E
Ew-AU->in-RG	1	Ew-AU->in-RG	1	Ewa-RG-art_E	1	Ewa-RG-art_E
	0		0		0	
	0		0		0	
Ew-AU->in-RG	1	Ew-AU->in-RG	1	Ewa-RG-art_E	1	Ewa-RG-art_E
	0		0		0	
	0		0		0	
Ew-AU->in-RG	1		0		0	
Ew-AU->in-RG	1	Ew-AU->in-RG	1	Ewa-RG-art_E	1	Ewa-RG-art_E
Ew-AU->in-RG	1	Ew-AU->in-RG	1	Ewa-RG-art_E	1	Ewa-RG-art_E
	0		0		0	
	0		0		0	
	0		0		0	
	0		0		0	
	0		0		0	
	0		0		0	
Ew-AU->in-RG	1	Ew-AU->in-RG	1	Ewa-RG-art_E	1	Ewa-RG-art_E
	0		0		0	
	0		0		0	
	0		0		0	
	0		0		0	
Ew-AU->in-RG	1	Ew-AU->in-RG	1	Ewa-RG-art_E	1	Ewa-RG-art_E
	0		0		0	
	0		0		0	
	0		0		0	
	0		0		0	
	0		0		0	
	0		0		0	
	0		0		0	
Ew-AU->in-RG	1		0	Ewa-RG-art_E	1	
Ew-AU->in-RG	1		0	Ewa-RG-art_E	1	
Ew-AU->in-RG	1		0	Ewa-RG-art_E	1	
Ew-AU->in-RG	1		0	Ewa-RG-art_E	1	

Ew-AU->in-RG	1		0	Ewa-RG-art_E	1	
Ew-AU->in-RG	1		0	Ewa-RG-art_E	1	
	0		0		0	
	0		0		0	
Ew-AU->in-RG	1		0	Ewa-RG-art_E	1	
Ew-AU->in-RG	1		0	Ewa-RG-art_E	1	
Ew-AU->in-RG	1		0		0	
	0		0		0	
	0		0		0	
Ew-AU->in-RG	1	Ew-AU->in-RG	1	Ewa-RG-art_E	1	Ewa-RG-art_E
Ew-AU->in-RG	1	Ew-AU->in-RG	1	Ewa-RG-art_E	1	Ewa-RG-art_E
	0		0		0	
Ew-AU->in-RG	1	Ew-AU->in-RG	1	Ewa-RG-art_E	1	Ewa-RG-art_E
Ew-AU->in-RG	1	Ew-AU->in-RG	1	Ewa-RG-art_E	1	Ewa-RG-art_E
Ew-AU->in-RG	1		0		0	
Ew-AU->in-RG	1		0		0	
Ew-AU->in-RG	1		0		0	
Ew-AU->in-RG	1		0		0	
	0		0		0	
	0		0		0	
	0		0		0	
	0		0		0	
	0		0		0	
	0		0		0	
	0		0		0	
	0		0		0	
	0		0		0	
	0		0		0	
Ew-AU->in-RG	1	Ew-AU->in-RG	1	Ewa-RG-art_E	1	Ewa-RG-art_E
	0		0		0	
	0		0		0	
	0		0		0	
Ew-AU->in-RG	1		0	Ewa-RG-art_E	1	
Ew-AU->in-RG	1		0	Ewa-RG-art_E	1	
Ew-AU->in-RG	1	Ew-AU->in-RG	1	Ewa-RG-art_E	1	Ewa-RG-art_E
Ew-AU->in-RG	1	Ew-AU->in-RG	1	Ewa-RG-art_E	1	Ewa-RG-art_E
	0		0		0	
	0		0		0	
	0		0		0	
Ew-AU->in-RG	1	Ew-AU->in-RG	1	Ewa-RG-art_E	1	Ewa-RG-art_E
Ew-AU->in-RG	1	Ew-AU->in-RG	1	Ewa-RG-art_E	1	Ewa-RG-art_E
Ew-AU->in-RG	1	Ew-AU->in-RG	1	Ewa-RG-art_E	1	Ewa-RG-art_E
	0		0		0	
	0		0		0	
	0		0		0	
	0		0		0	
	0		0		0	

	0		0		0	
	0		0		0	
Ew-AU->in-RG	1	Ew-AU->in-RG	1	Ewa-RG-art_E	1	Ewa-RG-art_E
Ew-AU->in-RG	1	Ew-AU->in-RG	1	Ewa-RG-art_E	1	Ewa-RG-art_E
	0		0		0	
	0		0		0	
	0		0		0	
	0		0		0	
	0		0		0	
	0		0		0	
	0		0		0	
	0		0		0	
	0		0		0	
	0		0		0	
	0		0		0	
	0		0		0	
	0		0		0	
	0		0		0	
	0		0		0	
	0		0		0	
	0		0		0	
	0		0		0	
	0		0		0	
	0		0		0	
	0		0		0	
	0		0		0	
	0		0		0	
	0		0		0	
	0		0		0	
	0		0		0	
	0		0		0	
	0		0		0	
	0		0		0	
	0		0		0	
	0		0		0	
	0		0		0	
	0		0		0	
	0		0		0	
	0		0		0	
	0		0		0	
Ew-AU->in-RG	1	Ew-AU->in-RG	1	Ewa-RG-art_E	1	Ewa-RG-art_E
Ew-AU->in-RG	1	Ew-AU->in-RG	1	Ewa-RG-art_E	1	Ewa-RG-art_E
Ew-AU->in-RG	1	Ew-AU->in-RG	1	Ewa-RG-art_E	1	Ewa-RG-art_E
	0		0		0	
	0		0		0	
	0		0		0	
	0		0		0	

	0		0		0	
Ew-AU->in-RG	1	Ew-AU->in-RG	1	Ewa-RG-art_E	1	Ewa-RG-art_E
	0		0		0	
	0		0		0	
Ew-AU->in-RG	1	Ew-AU->in-RG	1	Ewa-RG-art_E	1	Ewa-RG-art_E
Ew-AU->in-RG	1	Ew-AU->in-RG	1	Ewa-RG-art_E	1	Ewa-RG-art_E
	0		0		0	
Ew-AU->in-RG	1		0	Ewa-RG-art_E	1	
Ew-AU->in-RG	1		0	Ewa-RG-art_E	1	
	0		0		0	
	0		0		0	
	0		0		0	
	0		0		0	
Ew-AU->in-RG	1		0	Ewa-RG-art_E	1	
	0		0		0	
	0		0		0	
	0		0		0	
	0		0		0	
Ew-AU->in-RG	1	Ew-AU->in-RG	1	Ewa-RG-art_E	1	Ewa-RG-art_E
Ew-AU->in-RG	1	Ew-AU->in-RG	1	Ewa-RG-art_E	1	Ewa-RG-art_E
Ew-AU->in-RG	1	Ew-AU->in-RG	1	Ewa-RG-art_E	1	Ewa-RG-art_E
	0		0		0	
	0		0		0	
	0		0		0	
Ew-AU->in-RG	1	Ew-AU->in-RG	1	Ewa-RG-art_E	1	Ewa-RG-art_E
	0		0		0	
	0		0		0	
	0		0		0	
	0		0		0	
	0		0		0	
	0		0		0	
	0		0		0	
	0		0		0	
Ew-AU->in-RG	1	Ew-AU->in-RG	1	Ewa-RG-art_E	1	Ewa-RG-art_E
	0		0		0	
	0		0		0	
	0		0		0	
	0		0		0	
	0		0		0	
	0		0		0	
Ew-AU->in-RG	1	Ew-AU->in-RG	1	Ewa-RG-art_E	1	Ewa-RG-art_E
Ew-AU->in-RG	1	Ew-AU->in-RG	1	Ewa-RG-art_E	1	Ewa-RG-art_E
Ew-AU->in-RG	1	Ew-AU->in-RG	1	Ewa-RG-art_E	1	Ewa-RG-art_E
Ew-AU->in-RG	1		0		0	
Ew-AU->in-RG	1	Ew-AU->in-RG	1	Ewa-RG-art_E	1	Ewa-RG-art_E
Ew-AU->in-RG	1	Ew-AU->in-RG	1	Ewa-RG-art_E	1	Ewa-RG-art_E
	0		0		0	

	0		0		0	
Ew-AU->in-RG	1		0	Ewa-RG-art_E	1	
Ew-AU->in-RG	1		0	Ewa-RG-art_E	1	
Ew-AU->in-RG	1		0	Ewa-RG-art_E	1	
	0		0		0	
	0		0		0	
	0		0		0	
	0		0		0	
	0		0		0	
	0		0		0	
	0		0		0	
	0		0		0	
	0		0		0	
	0		0		0	
	0		0		0	
	0		0		0	
Ew-AU->in-RG	1	Ew-AU->in-RG	1	Ewa-RG-art_E	1	Ewa-RG-art_E
Ew-AU->in-RG	1	Ew-AU->in-RG	1	Ewa-RG-art_E	1	Ewa-RG-art_E
	0		0		0	
	0		0		0	
	0		0		0	
	0		0		0	
	0		0		0	
	0		0		0	
	0		0		0	
	0		0		0	
	0		0		0	
	0		0		0	
	0		0		0	
	0		0		0	
Ew-AU->in-RG	1	Ew-AU->in-RG	1	Ewa-RG-art_E	1	Ewa-RG-art_E
Ew-AU->in-RG	1	Ew-AU->in-RG	1	Ewa-RG-art_E	1	Ewa-RG-art_E
Ew-AU->in-RG	1		0	Ewa-RG-art_E	1	
Ew-AU->in-RG	1		0	Ewa-RG-art_E	1	
Ew-AU->in-RG	1		0	Ewa-RG-art_E	1	
	0		0		0	
	0		0		0	
	0		0		0	
	0		0		0	
	0		0		0	
	0		0		0	
	0		0		0	
	0		0		0	
	0		0		0	
Ew-AU->in-RG	1		0	Ewa-RG-art_E	1	
Ew-AU->in-RG	1		0	Ewa-RG-art_E	1	
Ew-AU->in-RG	1		0	Ewa-RG-art_E	1	

	0		0		0	
	0		0		0	
	0		0		0	
	0		0		0	
	0		0		0	
	0		0		0	
	0		0		0	
	0		0		0	
	0		0		0	
	0		0		0	
	0		0		0	
	0		0		0	
	0		0		0	
	0		0		0	
	0		0		0	
Ew-AU->in-RG	1	Ew-AU->in-RG	1	Ewa-RG-art_E	1	Ewa-RG-art_E
Ew-AU->in-RG	1	Ew-AU->in-RG	1	Ewa-RG-art_E	1	Ewa-RG-art_E
Ew-AU->in-RG	1	Ew-AU->in-RG	1	Ewa-RG-art_E	1	Ewa-RG-art_E
	0		0		0	
	0		0		0	
Ew-AU->in-RG	1		0	Ewa-RG-art_E	1	
Ew-AU->in-RG	1		0	Ewa-RG-art_E	1	
Ew-AU->in-RG	1		0	Ewa-RG-art_E	1	
	0		0		0	
	0		0		0	
	0		0		0	
	0		0		0	
	0		0		0	
	0		0		0	
	0		0		0	
	0		0		0	
	0		0		0	
	0		0		0	
	0		0		0	
	0		0		0	
	0		0		0	
	0		0		0	
	0		0		0	
	0		0		0	
	0		0		0	
	0		0		0	
	0		0		0	
	0		0		0	
	0		0		0	
	0		0		0	
	0		0		0	
	0		0		0	

	0		0		0	
	0		0		0	
	0		0		0	
	0		0		0	
	0		0		0	
	0		0		0	
	0		0		0	
	0		0		0	
	0		0		0	
	0		0		0	
	0		0		0	
Ew-AU->in-RG	1	Ew-AU->in-RG	1	Ewa-RG-art_E	1	Ewa-RG-art_E
	0		0		0	
Ew-AU->in-RG	1		0	Ewa-RG-art_E	1	
Ew-AU->in-RG	1		0	Ewa-RG-art_E	1	
Ew-AU->in-RG	1		0	Ewa-RG-art_E	1	
	0		0		0	
	0		0		0	
Ew-AU->in-RG	1	Ew-AU->in-RG	1	Ewa-RG-art_E	1	Ewa-RG-art_E
Ew-AU->in-RG	1	Ew-AU->in-RG	1	Ewa-RG-art_E	1	Ewa-RG-art_E
Ew-AU->in-RG	1	Ew-AU->in-RG	1	Ewa-RG-art_E	1	Ewa-RG-art_E
Ew-AU->in-RG	1	Ew-AU->in-RG	1	Ewa-RG-art_E	1	Ewa-RG-art_E
	0		0		0	
	0		0		0	
	0		0		0	
	0		0		0	
	0		0		0	
	0		0		0	
	0		0		0	
	0		0		0	
	0		0		0	
	0		0		0	

ewargart_au_	entrywithaau	ewauinrgart_	ewauinrgart_	ewauinrg_pi_	ewauinrg_pi_	ewargart_au_	ewargart_au_
0			0		0		0
0			0		0		0
0			0		0		0
0			0		0		0
0			0		0		0
0			0		0		0
0			0		0		0
0			0		0		0
0			0		0		0
0			0		0		0
1			0		0		0
0			0		0		0
0			0		0		0
0			0		0		0
0			0		0		0
0			0		0		0
0			0		0		0
0			0		0		0
0			0		0		0
0			0		0		0
0			0		0		0
0			0		0		0
1			0		0		0
1			0		0		0
1			0		0		0
0			0		0		0
0			0		0		0
0			0		0		0
0			0		0		0
1			0		0		0
0			0		0		0
0			0		0		0
0			0		0		0
0			0		0		0
0			0		0		0
0			0		0		0
0			0		0		0
0			0		0		0
0			0		0		0
0			0		0		0
0			0		0		0
0			0		0		0
1			0		0		0
1			0		0		0
1			0		0		0
0			0		0		0

0		0		0		0
0		0		0		0
1		0		0		0
0		0		0		0
1		0		0		0
0		0		0		0
0		0		0		0
1	Ew-AU->in-RG	1	Ew-AU->in-RG	1	Ewa-RG-top-a	1
0	Ew-AU->in-RG	1		0		0
0		0		0		0
0		0		0		0
0		0		0		0
0		0		0		0
1		0		0		0
0		0		0		0
0		0		0		0
0		0		0		0
0		0		0		0
0		0		0		0
0		0		0		0
0		0		0		0
0		0		0		0
0		0		0		0
0		0		0		0
1	Ew-AU->in-RG	1	Ew-AU->in-RG	1	Ewa-RG-top-a	1
1	Ew-AU->in-RG	1	Ew-AU->in-RG	1	Ewa-RG-top-a	1
0		0		0		0
0		0		0		0
0		0		0		0
1		0		0		0
1		0		0		0
1		0		0		0
1		0		0		0
1		0		0		0
0		0		0		0
0		0		0		0
0		0		0		0
0		0		0		0
0		0		0		0
0		0		0		0
0		0		0		0
0		0		0		0
0		0		0		0
0		0		0		0
0		0		0		0
1		0		0		0
0		0		0		0

0		0		0		0
0		0		0		0
0		0		0		0
1		0		0		0
0		0		0		0
0		0		0		0
0		0		0		0
0		0		0		0
1		0		0		0
0		0		0		0
0		0		0		0
0		0		0		0
0		0		0		0
1	Ew-AU->in-RG	1	Ew-AU->in-RG	1	Ewa-RG-top-a	1
0		0		0		0
0		0		0		0
0		0		0		0
0		0		0		0
0		0		0		0
0		0		0		0
1		0		0		0
0		0		0		0
0		0		0		0
0		0		0		0
0	Ew-AU->in-RG	1		0		0
1		0		0		0
1		0		0		0
0		0		0		0
0		0		0		0
1		0		0		0
1		0		0		0
0		0		0		0
0		0		0		0
0		0		0		0
1		0		0		0
1		0		0		0
1		0		0		0
1		0		0		0
1		0		0		0
1		0		0		0
0		0		0		0
0		0		0		0
0		0		0		0
0		0		0		0
0		0		0		0
0		0		0		0
0		0		0		0

0		0		0		0
0		0		0		0
0		0		0		0
0		0		0		0
0		0		0		0
0		0		0		0
1		0		0		0
1		0		0		0
0		0		0		0
0		0		0		0
0		0		0		0
0		0		0		0
0		0		0		0
0		0		0		0
0		0		0		0
0		0		0		0
0	Ew-AU->in-RG	1		0	Ewa-RG-top-a	1
0		0		0		0
0		0		0		0
0		0		0		0
0		0		0		0
0		0		0		0
1		0		0		0
0		0		0		0
0		0		0		0
0		0		0		0
0		0		0		0
1	Ew-AU->in-RG	1		0		0
0		0		0		0
1	Ew-AU->in-RG	1	Ew-AU->in-RG	1	Ewa-RG-top-a	1
0		0		0		0
0		0		0		0
0		0		0		0
0		0		0		0
0		0		0		0
0		0		0		0
0		0		0		0
0		0		0		0
1	Ew-AU->in-RG	1	Ew-AU->in-RG	1	Ewa-RG-top-a	1
0		0		0		0
0		0		0		0
1		0		0		0
1		0		0		0
0		0		0		0
0		0		0		0
0		0		0		0
0		0		0		0

0		0		0		0
0		0		0		0
1		0		0		0
0		0		0		0
0		0		0		0
0		0		0		0
0		0		0		0
0		0		0		0
0		0		0		0
1		0		0		0
0		0		0		0
0		0		0		0
1		0		0		0
1		0		0		0
0		0		0		0
1		0		0		0
1		0		0		0
0		0		0		0
0		0		0		0
0		0		0		0
0		0		0		0
0		0		0		0
1		0		0		0
1		0		0		0
1	Ew-AU->in-RG	1	Ew-AU->in-RG	1	Ewa-RG-top-a	1
1	Ew-AU->in-RG	1	Ew-AU->in-RG	1	Ewa-RG-top-a	1
0	Ew-AU->in-RG	1		0	Ewa-RG-top-a	1
0	Ew-AU->in-RG	1		0	Ewa-RG-top-a	1
0	Ew-AU->in-RG	1		0	Ewa-RG-top-a	1
0		0		0		0
0		0		0		0
0		0		0		0
0		0		0		0
0		0		0		0
0		0		0		0
0		0		0		0
1		0		0		0
1		0		0		0
1		0		0		0
1		0		0		0
1		0		0		0
1		0		0		0
0		0		0		0
1		0		0		0
0		0		0		0
0		0		0		0
0		0		0		0

1		0		0		0
1		0		0		0
0		0		0		0
1		0		0		0
1		0		0		0
0		0		0		0
1		0		0		0
1		0		0		0
1		0		0		0
1	Ew-AU->in-RG	1	Ew-AU->in-RG	1	Ewa-RG-top-a	1
0		0		0		0
0		0		0		0
0		0		0		0
0		0		0		0
0		0		0		0
0		0		0		0
1		0		0		0
1		0		0		0
1		0		0		0
0		0		0		0
0		0		0		0
1		0		0		0
1		0		0		0
1		0		0		0
0		0		0		0
0		0		0		0
1		0		0		0
1		0		0		0
0		0		0		0
0		0		0		0
1		0		0		0
1		0		0		0
1		0		0		0
0		0		0		0
0		0		0		0
0		0		0		0
0		0		0		0
0		0		0		0
1	Ew-AU->in-RG	1		0		0
1	Ew-AU->in-RG	1		0		0
0		0		0		0
0		0		0		0
0		0		0		0
0		0		0		0
0		0		0		0
0	Ew-AU->in-RG	1	Ew-AU->in-RG	1		0
0		0		0		0

1	Ew-AU->in-RG	1	Ew-AU->in-RG	1	Ewa-RG-top-a	1
1	Ew-AU->in-RG	1	Ew-AU->in-RG	1	Ewa-RG-top-a	1
0		0		0		0
0		0		0		0
1		0		0		0
1		0		0		0
1		0		0		0
0		0		0		0
0		0		0		0
0		0		0		0
0		0		0		0
0		0		0		0
0		0		0		0
0		0		0		0
0		0		0		0
0		0		0		0
0		0		0		0
0		0		0		0
0		0		0		0
0		0		0		0
0		0		0		0
0		0		0		0
0		0		0		0
0		0		0		0
0		0		0		0
1		0		0		0
1		0		0		0
1		0		0		0
1		0		0		0
1		0		0		0
0		0		0		0
0		0		0		0
0		0		0		0
0		0		0		0
0		0		0		0
0		0		0		0
0		0		0		0
0		0		0		0
0		0		0		0
0		0		0		0
0		0		0		0
0		0		0		0
0		0		0		0
0		0		0		0
0		0		0		0
0		0		0		0
0		0		0		0

0		0	0	0
0		0	0	0
0		0	0	0
0		0	0	0
0		0	0	0
1		0	0	0
1		0	0	0
0		0	0	0
0		0	0	0
0		0	0	0
0		0	0	0
0		0	0	0
0		0	0	0
0		0	0	0
1		0	0	0
1		0	0	0
0		0	0	0
1		0	0	0
0		0	0	0
0		0	0	0
0		0	0	0
0		0	0	0
0		0	0	0
0		0	0	0
1		0	0	0
1		0	0	0
0		0	0	0
0		0	0	0
0		0	0	0
0		0	0	0
0		0	0	0
0		0	0	0
0		0	0	0
0		0	0	0
0		0	0	0
0		0	0	0
0		0	0	0
0		0	0	0
0		0	0	0
0		0	0	0
0	Ew-AU->in-RG	1	0	0
0		0	0	0
0		0	0	0
0		0	0	0
0		0	0	0
1		0	0	0
1		0	0	0

0		0		0		0
0		0		0		0
0		0		0		0
0		0		0		0
0		0		0		0
0		0		0		0
0		0		0		0
1		0		0		0
1		0		0		0
0		0		0		0
0		0		0		0
1		0		0		0
1		0		0		0
0		0		0		0
0		0		0		0
0		0		0		0
0		0		0		0
0		0		0		0
0		0		0		0
0		0		0		0
0		0		0		0
1		0		0		0
0		0		0		0
0		0		0		0
0		0		0		0
0		0		0		0
0		0		0		0
0		0		0		0
1	Ew-AU->in-RG	1	Ew-AU->in-RG	1	Ewa-RG-top-a	1
1	Ew-AU->in-RG	1	Ew-AU->in-RG	1	Ewa-RG-top-a	1
0		0		0		0
1		0		0		0
1		0		0		0
0		0		0		0
1	Ew-AU->in-RG	1	Ew-AU->in-RG	1	Ewa-RG-top-a	1
0		0		0		0
0		0		0		0
0		0		0		0
0		0		0		0
0		0		0		0
0		0		0		0
0		0		0		0
0		0		0		0
0		0		0		0
0		0		0		0
0		0		0		0
0		0		0		0

0		0		0		0
0	Ew-AU->in-RG	1		0		0
0	Ew-AU->in-RG	1		0		0
0		0		0		0
0		0		0		0
0		0		0		0
0		0		0		0
0		0		0		0
0		0		0		0
0		0		0		0
0		0		0		0
0		0		0		0
0		0		0		0
0		0		0		0
0		0		0		0
0		0		0		0
0		0		0		0
0		0		0		0
0		0		0		0
0		0		0		0
0		0		0		0
0		0		0		0
0		0		0		0
0		0		0		0
0		0		0		0
0		0		0		0
0		0		0		0
0		0		0		0
0		0		0		0
0		0		0		0
0		0		0		0
0		0		0		0
0		0		0		0
0		0		0		0
0		0		0		0
0		0		0		0
0		0		0		0
0		0		0		0
0		0		0		0
0		0		0		0
0		0		0		0
0		0		0		0
0		0		0		0
0		0		0		0
1	Ew-AU->in-RG	1	Ew-AU->in-RG	1	Ewa-RG-top-a	1
1		0		0		0
0		0		0		0

0		0		0		0
0		0		0		0
0		0		0		0
0		0		0		0
0		0		0		0
0		0		0		0
0		0		0		0
0		0		0		0
0		0		0		0
0		0		0		0
1		0		0		0
1		0		0		0
1		0		0		0
0		0		0		0
0		0		0		0
0		0		0		0
0		0		0		0
0		0		0		0
0		0		0		0
0		0		0		0
1		0		0		0
1		0		0		0
0		0		0		0
0		0		0		0
0		0		0		0
0		0		0		0
0		0		0		0
0		0		0		0
0		0		0		0
0		0		0		0
0		0		0		0
0		0		0		0
0		0		0		0
0		0		0		0
1	Ew-AU->in-RG	1	Ew-AU->in-RG	1	Ewa-RG-top-a	1
0		0		0		0
0		0		0		0
0		0		0		0
0		0		0		0
0		0		0		0
0	Ew-AU->in-RG	1		0		0
0		0		0		0
0		0		0		0
1		0		0		0
0		0		0		0
0		0		0		0
0		0		0		0

0	0	0	0
0	0	0	0
0	0	0	0
0	0	0	0
0	0	0	0
0	0	0	0
0	0	0	0
1	0	0	0
1	0	0	0
0	0	0	0
0	0	0	0
0	0	0	0
0	0	0	0
0	0	0	0
0	0	0	0
0	0	0	0
0	0	0	0
0	0	0	0
0	0	0	0
0	0	0	0
1	0	0	0
1	0	0	0
1	0	0	0
1	0	0	0
0	0	0	0
0	0	0	0
0	0	0	0
0	0	0	0
0	0	0	0
0	0	0	0
1	0	0	0
1	0	0	0
1	0	0	0
1	0	0	0
0	0	0	0
0	0	0	0
0	0	0	0
1	0	0	0
1	0	0	0
0	0	0	0
0	0	0	0
0	0	0	0
0	0	0	0
0	0	0	0
1	0	0	0
1	0	0	0
0	0	0	0

1		0	0	0
1		0	0	0
0		0	0	0
0		0	0	0
1		0	0	0
1		0	0	0
1		0	0	0
0		0	0	0
0		0	0	0
0		0	0	0
0		0	0	0
0		0	0	0
0	Ew-AU->in-RG	1	0	0
0		0	0	0
0		0	0	0
1		0	0	0
1		0	0	0
0		0	0	0
0		0	0	0
0		0	0	0
0		0	0	0
0		0	0	0
0		0	0	0
0		0	0	0
0		0	0	0
0		0	0	0
0		0	0	0
0		0	0	0
0		0	0	0
1		0	0	0
0		0	0	0
0		0	0	0
0		0	0	0
0		0	0	0
0		0	0	0
0		0	0	0
0		0	0	0
1		0	0	0
1		0	0	0
0		0	0	0
0		0	0	0
0		0	0	0
0		0	0	0
0		0	0	0
0		0	0	0
0		0	0	0
0		0	0	0

0		0		0		0
0		0		0		0
1	Ew-AU->in-RG	1	Ew-AU->in-RG	1	Ewa-RG-top-a	1
1	Ew-AU->in-RG	1	Ew-AU->in-RG	1	Ewa-RG-top-a	1
0		0		0		0
0		0		0		0
1		0		0		0
1		0		0		0
1		0		0		0
1		0		0		0
0		0		0		0
0		0		0		0
0		0		0		0
0		0		0		0
0		0		0		0
0		0		0		0
0		0		0		0
0		0		0		0
0		0		0		0
0		0		0		0
0		0		0		0
1	Ew-AU->in-RG	1	Ew-AU->in-RG	1	Ewa-RG-top-a	1
1	Ew-AU->in-RG	1	Ew-AU->in-RG	1	Ewa-RG-top-a	1
1		0		0		0
1		0		0		0
1		0		0		0
0		0		0		0
0		0		0		0
0		0		0		0
0		0		0		0
0		0		0		0
1		0		0		0
1		0		0		0
0		0		0		0
0		0		0		0
0		0		0		0
0		0		0		0
0		0		0		0
0		0		0		0
0		0		0		0
0		0		0		0
0		0		0		0
1		0		0		0
0		0		0		0
0		0		0		0
0		0		0		0
0		0		0		0

0		0		0		0
0		0		0		0
0		0		0		0
0		0		0		0
0		0		0		0
0		0		0		0
0		0		0		0
1		0		0		0
0		0		0		0
0		0		0		0
0		0		0		0
0		0		0		0
0		0		0		0
0		0		0		0
1	Ew-AU->in-R	1	Ew-AU->in-R	1	Ewa-RG-top-a	1
0		0		0		0
0		0		0		0
0		0		0		0
0		0		0		0
0		0		0		0
0		0		0		0
0		0		0		0
0		0		0		0
0		0		0		0
0		0		0		0
0		0		0		0
0		0		0		0
1		0		0		0
0		0		0		0
0		0		0		0
0		0		0		0
0		0		0		0
0		0		0		0
1		0		0		0
1		0		0		0
1		0		0		0
1		0		0		0
0		0		0		0
0		0		0		0
0		0		0		0
0		0		0		0
0		0		0		0
0		0		0		0
0		0		0		0
0		0		0		0
0		0		0		0
0		0		0		0

0		0		0		0
0		0		0		0
0		0		0		0
0		0		0		0
1		0		0		0
0		0		0		0
0		0		0		0
0		0		0		0
1	Ew-AU->in-RG	1	Ew-AU->in-RG	1	Ewa-RG-top-a	1
0		0		0		0
1		0		0		0
1		0		0		0
1		0		0		0
0		0		0		0
0		0		0		0
0		0		0		0
0		0		0		0
0		0		0		0
0		0		0		0
1		0		0		0
1		0		0		0
0		0		0		0
0		0		0		0
0		0		0		0
1		0		0		0
0		0		0		0
0		0		0		0
0		0		0		0
1	Ew-AU->in-RG	1	Ew-AU->in-RG	1	Ewa-RG-top-a	1
1	Ew-AU->in-RG	1	Ew-AU->in-RG	1	Ewa-RG-top-a	1
1	Ew-AU->in-RG	1	Ew-AU->in-RG	1	Ewa-RG-top-a	1
0		0		0		0
0		0		0		0
0		0		0		0
0		0		0		0
0		0		0		0
0		0		0		0
0		0		0		0
0		0		0		0
0		0		0		0
0		0		0		0
1		0		0		0
0		0		0		0
0		0		0		0
0		0		0		0
0		0		0		0
0		0		0		0

0		0	0	0
0		0	0	0
0		0	0	0
0		0	0	0
0		0	0	0
0		0	0	0
0		0	0	0
0		0	0	0
0		0	0	0
0		0	0	0
0		0	0	0
0		0	0	0
0		0	0	0
0		0	0	0
0		0	0	0
0		0	0	0
0		0	0	0
1		0	0	0
1		0	0	0
0		0	0	0
0		0	0	0
0		0	0	0
0		0	0	0
0		0	0	0
0		0	0	0
0		0	0	0
0		0	0	0
0		0	0	0
0		0	0	0
0		0	0	0
0		0	0	0
0		0	0	0
0		0	0	0
0		0	0	0
0		0	0	0
0		0	0	0
0		0	0	0
0		0	0	0
0		0	0	0
0		0	0	0
0		0	0	0
0		0	0	0
0		0	0	0
0		0	0	0
0		0	0	0
0	Ew-AU->in-RG	1	0	0
0		0	0	0

0		0	0	0
0		0	0	0
0		0	0	0
1		0	0	0
0		0	0	0
0		0	0	0
1	Ew-AU->in-RG	1	0	0
1	Ew-AU->in-RG	1	0	0
0		0	0	0
0		0	0	0
0		0	0	0
0		0	0	0
0		0	0	0
0		0	0	0
1		0	0	0
0		0	0	0
0		0	0	0
0		0	0	0
0		0	0	0
1		0	0	0
1		0	0	0
1		0	0	0
1		0	0	0
1		0	0	0
0		0	0	0
0		0	0	0
1		0	0	0
1		0	0	0
1		0	0	0
0		0	0	0
0		0	0	0
0		0	0	0
1		0	0	0
0		0	0	0
0		0	0	0
0		0	0	0
1		0	0	0
1		0	0	0
1		0	0	0
0	Ew-AU->in-RG	1	0	0
0	Ew-AU->in-RG	1	0	0
1		0	0	0
1		0	0	0
0		0	0	0
0		0	0	0
0		0	0	0
0		0	0	0

1		0		0		0
0		0		0		0
0		0		0		0
0		0		0		0
0		0		0		0
0		0		0		0
0		0		0		0
0		0		0		0
0		0		0		0
0		0		0		0
0		0		0		0
1	Ew-AU->in-RG	1	Ew-AU->in-RG	1	Ewa-RG-top-a	1
1	Ew-AU->in-RG	1	Ew-AU->in-RG	1	Ewa-RG-top-a	1
1		0		0		0
0		0		0		0
0		0		0		0
0		0		0		0
0		0		0		0
0		0		0		0
0		0		0		0
1		0		0		0
1		0		0		0
1		0		0		0
1		0		0		0
0		0		0		0
0		0		0		0
0		0		0		0
0		0		0		0
0		0		0		0
0		0		0		0
0		0		0		0
0		0		0		0
0		0		0		0
0		0		0		0
1		0		0		0
0		0		0		0
0		0		0		0
0		0		0		0
0		0		0		0
0		0		0		0
0		0		0		0
0		0		0		0
0		0		0		0
0		0		0		0
0		0		0		0
0		0		0		0
0		0		0		0

0		0		0		0
0		0		0		0
0		0		0		0
0		0		0		0
0		0		0		0
[truncated: 410,889 more chars]
